# Correlation of pathological complete response with survival after neoadjuvant chemotherapy in gastric or gastroesophageal junction cancer treated with radical surgery: A meta-analysis

**DOI:** 10.1371/journal.pone.0189294

**Published:** 2018-01-25

**Authors:** Ziyu Li, Fei Shan, Yinkui Wang, Yan Zhang, Lianhai Zhang, Shuangxi Li, Yongning Jia, Kan Xue, Rulin Miao, Zhemin Li, Jiafu Ji

**Affiliations:** Key laboratory of Carcinogenesis and Translational Research (Ministry of Education), Department of Gastrointestinal Surgery, Peking University Cancer Hospital & Institute, Beijing, China; Taipei Medical University College of Medicine, TAIWAN

## Abstract

**Background:**

Neoadjuvant chemotherapy before radical gastrectomy is preferred for locally advanced gastric cancer. To avoid the problematic use of pTNM for patients after neoadjuvant chemotherapy, the Union for International Cancer Control (UICC) and the American Joint Committee on Cancer (AJCC) gastric cancer TNM staging system (8th edition) added ypTNM for the first time. But patients achieving pathological complete response were not covered by the new ypTNM staging system. To investigate whether pathological complete response is associated with better outcome in gastric cancer, as was reported in rectal, breast and bladder cancer.

**Methods:**

We systematically searched the databases of PubMed, EMBASE, Web of Science and Cochrane Collaboration’s Central register of controlled trials from January 1988 to April 2015 for publications which reported outcomes of patients with and without pathological complete response (pCR) (pT0N0M0) to investigate whether pCR after neoadjuvant chemotherapy in gastric or gastroesophageal junction (GEJ) treated with radical surgery is associated with better survival. The primary outcome was overall survival (OS). The secondary outcome was disease-free survival (DFS). Both were measured with a relative risk (RR). A meta-analysis was performed using the fixed effects model. Forest plots and the Q test was used to evaluate overall heterogeneity for OS and DFS.

**Results:**

A total of seven trials, 1143 patients were included and analyzed after neoadjuvant chemotherapy and radical surgery with no other preoperative treatment. The average rate of pCR was 6.74% (range: 3%-15%). The RR of patients who achieved pCR in the primary tumor and lymph nodes is 0.5 (95% confidence interval [CI], 0.25–0.98; p = 0.04), 0.34 (95% CI, 0.21–0.55; p<0.0001) and 0.44 (95% CI, 0.30–0.63; p<0.0001) for one-year-OS, three-year-OS and five-year-OS, respectively. The summary RR for three-year-DFS was 0.43 (95% CI, 0.25–0.72; p = 0.002).

**Conclusion:**

Patients with resectable gastric or GEJ cancer who achieved pCR after neoadjuvant chemotherapy can gain a better outcome than patients without pCR.

## Introduction

Neoadjuvant chemotherapy followed by radical surgery is recommended by NCCN as an initial therapy for locally advanced gastric or GEJ cancer[1]. A few multicenter phase III trials have proved that neoadjuvant chemotherapy can improve the overall survival and the disease-free survival of locally advanced gastric or GEJ cancer.[2, 3] To avoid the problematic use of pTNM for patients after neoadjuvant chemotherapy, the TNM staging system of Union for International Cancer Control (UICC) and American Joint Committee on Cancer (AJCC), the most important staging system to evaluate the prognosis of gastric cancer patients, added ypTNM to the 8th edition for the first time. However, patients achieving pCR were not covered by the new ypTNM staging system.

With breast cancer, rectal cancer and bladder cancer, achieving pCR after neoadjuvant chemotherapy is associated with a better overall survival and disease-free survival.[4–7] A meta-analysis of 12 randomized controlled trials with 11955 patients by the FDA indicated that pCR was associated with a better event-free-survival and overall survival in different subtypes of breast cancer, and the association was strongest in patients with triple-negative breast cancer.[8] Therefore, the FDA uses pCR as an endpoint in neoadjuvant treatment of high-risk early-stage breast cancer to support accelerated approval.[9]

However, with gastric or GEJ cancer, there is still no agreement about whether pCR is related to survival after neoadjuvant chemotherapy. Only a few single center retrospective studies proved that pCR impacted the disease-free survival and overall survival.[10] Some studies held the opposite opinion. A retrospective study of 168 patients after neoadjuvant chemotherapy was reported by Brenner B et al.[11] They found that multivariable analysis reflected that only lymph node status, nerve invasion and vascular invasion were independent factors for disease-free-survival. Andrew M. Lowy et al[12, 13] achieved similar results. They supported that the tumor regression grade was not an independent factor of overall survival. Owing to the limited sample size, high-level evidence is still needed.

The pCR rate of gastric or GEJ cancer after neoadjuvant chemotherapy is relatively low; therefore, it is difficult for a single center to provide enough data about the survival of patients who achieved pCR after neoadjuvant chemotherapy. Moreover, currently, most studies investigating the correlation between the pathologic response with survival after neoadjuvant chemotherapy in gastric or gastroesophageal junction cancer are focused on the comparison between good-response and no-response.[14–16] Few data have been reported regarding the difference between the survival of patients who achieved pCR with neoadjuvant chemotherapy. Therefore, a meta-analysis is a good choice to explore the correlation between pCR and survival after neoadjuvant chemotherapy in gastric or GEJ cancer. A systematic review of the literature regarding survival outcomes of advanced gastric cancer patients who achieved pCR with neoadjuvant chemotherapy was reported by Haruhiko Cho et al[17] in 2015. They reached the conclusion that pCR was related to a better overall survival and recurrence-free survival rates. However, one shortcoming of this study is that the conclusion was based on published case reports. As we all know, a case report may not be suitable to everyone. To date, there is no meta-analysis regarding the correlation between pCR and survival in patients who was treated with neoadjuvant chemotherapy for gastric or GEJ cancer. Therefore, we conducted this study to investigate whether achieving pCR after neoadjuvant chemotherapy would result in a better overall and disease-free survival than not achieving pCR in patients with gastric or GEJ cancer.

## 1. Methods

### 1.1 Literature search

To identify useful studies and published abstracts, we systematically searched electronic databases, including PubMed, EMBASE, Web of Science and Cochrane Collaboration’s Central register of controlled trials. The search included literature published from January 1988 to April 2015, was restricted to articles published in English and used the following search keywords: (gastric cancer OR stomach cancer OR gastric carcinoma OR stomach carcinoma OR gastric adenocarcinoma OR gastric tumor OR stomach tumor OR adenocarcinoma of the stomach OR cancers of the stomach OR gastroesophageal junction tumors OR gastroesophageal junction adenocarcinoma OR gastroesophageal junction cancer OR adenocarcinoma of the gastroesophageal junction OR gastroesophageal junction tumors OR cancers of the gastroesophageal junction) AND (preoperative chemotherapy OR neoadjuvant chemotherapy OR induction chemotherapy) AND (pCR OR pT0 OR P0 OR pathologic response OR pathological complete response OR pathological complete remission OR down-staging) AND (OS OR DFS OR survival OR prognosis OR outcome OR disease- free survival OR recurrence-free survival). We also reviewed the referenced literature of all included studies. Two investigators conducted the search independently. Then we combined their results.

### 1.2 Study selection criteria

Two authors (Zhemin Li and Rulin Miao) reviewed the studies independently. The titles and abstracts were in agreement with the articles to be retrieved. To identify studies for the analysis, the inclusion criteria were designed as follows: (1) used a retrospective or prospective study design; (2) evaluated the association between pCR and survival result; (3) presented odds ratio (OR), hazard ratio (HR) or relative risk (RR) estimates with 95% confidence intervals (CIs), standard errors (SEs) or outcome parameters, such as OS or DFS, in addition to p values; and (4) included > 10 gastric or GEJ cancer patients treated with neoadjuvant chemotherapy and radical surgery (D1 or D2 lymph node dissection). A study would be excluded if it contained neoadjuvant radiotherapy, perioperative targeted therapy or immunotherapy; therefore, other adjuvant treatment factors did not complicate results. A study would also be excluded if distant metastases were included in it to reduce the effect of stage IV patients. If multiple publications from the same study or institution were available, we chose the one with the largest cases and most applicable information. When two investigators could not reach an agreement whether an article met the inclusion criterion, then the decision was made by the third investigator (Kan Xue). All analyses were based on previous published studies, thus no ethical approval and patient consent are required.

### 1.3 Data extraction

The quality of included studies were evaluated by the Newcastle-Ottawa Scale. The following information was extracted from each included publication: the first authors’ name, year of publication, duration of follow up, total number of patients, median age, percentage of patients achieving pCR, neoadjuvant chemotherapy regimen and adjuvant chemotherapy, clinical TNM stage and survival data (number of events). Once the correlation between tumor down-staging and outcome was identified by the authors, it would be recorded. The analysis of study quality and data extraction was conducted by Shuangxi Li and Yongning Jia.

### 1.4 Statistical analysis

The primary outcome was OS, and the secondary end point was DFS. If possible, RR and the corresponding standard errors were obtained directly from the article; otherwise, they were calculated using the methods of Parmar[18], Tierney[19] and Williamson.[20] Confidence intervals, log-rank, p-values, number of events, and Kaplan–Meier survival curves were used to estimate the HR and standard errors.

Summary statistics of patients achieving pCR after neoadjuvant chemotherapy with RRs and 95% CI were calculated using the fixed effects model when there was minimal heterogeneity in the variables among studies and the random effects model when there was significant heterogeneity. Possible bias was investigated using subgroup analysis or sensitivity analysis when evidence of heterogeneity presented. The x^2^ and I^2^ test were used for the between-study heterogeneity of the RRs. Criteria for statistically significant differences included <0.1 for the x^2^ p value and >50% for the I^2^ test.

Forest plots were generated by standard techniques to summarize the included studies, with horizontal lines representing 95% CI, the area of each square representing the weighting and the positions of each square demonstrating the RR point estimate. The vertical line was at the position RR = 1.

The Egger linear regression, the Begg rank correlation and funnel plots were used to evaluate the publication bias for OS and DFS analysis.[21, 22] A p value <0.05 for the Egger or Begg test indicated significant statistical publication bias.

The RR associated with pCR in each study was displayed by the funnel plots. The X axis represented the RR value, and the Y axis represented the standard error. The vertical line indicated the pooled estimate of the overall RR with the sloping lines representing the expected 95% CI for a given SE.

All statistical analyses were performed with Review Manager v. 5.3 (The Nordic Cochrane Centre, The Cochrane Collaboration) and Stata 12.1(Stata Corp., College Station, TX).

## 2. Results

### 2.1 Data synthesis

**Fig 1:**
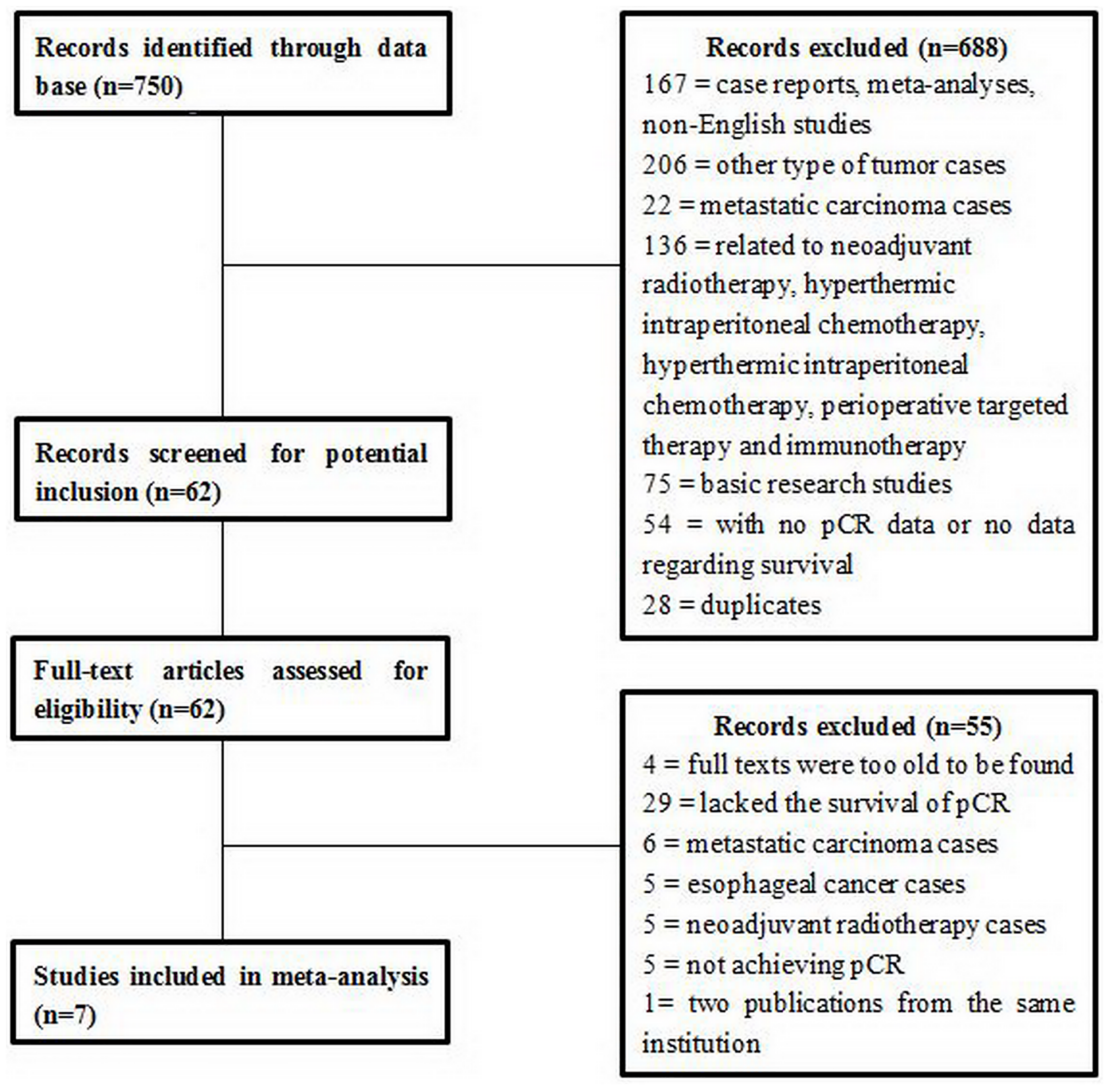
PRISMA diagram. The figure displays the information retrieval process for valuable articles and exclusion process of irrelative articles for this research.

The results of the literature search are displayed in a Preferred Reporting Items for Systematic Reviews and Meta-Analyses (PRISMA) diagram (Fig 1). A total of 750 studies were retrieved in the database, including PubMed 384, Web of science 295, EMBASE for publications 51 and Cochrane Collaboration`s Central register of controlled trials 20. Among them we found 90 case reports, 53 meta-analyses, 24 non-English studies, 206 other type of tumor cases, 22 metastatic carcinoma cases, 136 cases related to neoadjuvant radiotherapy, hyperthermic intraperitoneal chemotherapy, hyperthermic intraperitoneal chemotherapy, perioperative targeted therapy and immunotherapy, 75 basic research studies, 32 cases with no pCR data and 22 cases with no data regarding survival. All of them were excluded. For the other 90 papers, 28 studies were iterative, and four full texts were too old to be found. By reading through the full context, 29 cases lacked the survival of pCR, and there were six metastatic carcinoma cases, five esophageal cancer cases, five neoadjuvant radiotherapy cases and five cases not achieving pCR. All of them were excluded as well. For the rest eight papers, two of them from the same institution, we just used the one with larger number of cases and more applicable information. Finally, seven studies met our inclusion criteria, which examined a total of 1143 patients. All of the studies were retrospective or prospective case series. All the seven studies were in high quality assessed by the Newcastle-Ottawa Scale.(S1 Table) The median age of patients ranged from 53 to 62 years, and the proportion of females was 24.5%. The pCR rates ranged from 3% to 15% in the included studies. Median follow-up ranged from 17 mon to 41 mon. The main features of the trials included in the meta-analysis are shown in Table 1.

**Table 1:** Characteristics of included studies.

Study	Study type	Patients,no.	Median age, yr	Median follow-up,mon	Clinical stage	Neoadjuvant treatment	Cycles, no.	Resection rate,%	R0 rate,%	pCR rate,%	Adjuvant Treatment	Cycles, no.	Survival data available	
													OS	DFS
Lorenzen S 2013[10]	Retrospective	120	59.5	41.1	T2N+,T3/4	TPLF/DCX/ FLOT	1–8	100	93	15	CTX	>1	√	√
Lowy AM 1999[12]	Retrospective	83	54.8	26	>T2	EFP/EAP/FIP	2–5	75.9	96.8	4	EFP/EAP	0–3	√	
Heger U 2014[23]	Retrospective	723	NA	36.7	T3/4	Variable	NA	96.8	74.9	4.6	Variable	Variable	√	
Peixoto RD 2014[24]	Retrospective	83	62	NA	NA	ECF/ECX	1–3	93.9	71	7.2	ECF/ECX	0–3	√	
Koh YW 2013[25]	Retrospective	143	53	35	NA	DFP/FP/EFP	>1	100	100	11.2	NA	NA	√	√
Leichman L 1992[26]	Prospective	38	55	17	NA	CLF	2	92	82.8	3	IP	2	√	
Persiani R 2005[27]	Prospective	34	56.2	28	T3/4,T<2 N+	EEP/ECF	1–3	97.1	81.8	3	Variable	0–3	√	

NA-not available, CLF- cisplatin+leucovorin+5FU, IP-intraperitoneal therapy, EEP- etoposide +epirubicin +ciplastin, ECF- epirubicin+cisplatin +5FU, ECX- epirubicin+ cisplatin+ capecitabine, TPLF- docetaxel+ cisplatin +leucovorin +5-FU, DCX- docetaxel+ cisplatin +capecitabine, FLOT- oxaliplatin+ docetaxel+ 5-FU, DFP-docetaxel+ fluoropyrimidine+platinum, FP-fluoropyrimidine+ platinum, EFP-epirubicin+fluoropyrimidine+platinum, EAP- etoposide+ doxorubicin +cisplatin, FIP- 5-FU+ a-interferon + cisplatin.

### 2.2 Primary end point: Overall survival

**Fig 2:**
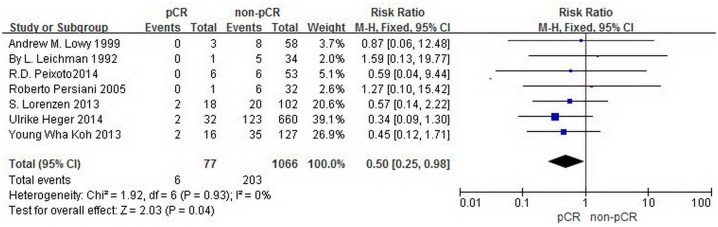
Forest plot of pooled relative risk for one-year OS from eligible studies. The area of each square represents the weighting, and the positions of each square demonstrate the risk ratio point estimate. Horizontal lines represent 95% confidence interval (CI). M-H = Mantel-Haenszel. Events = patients died from any cause within one year.

**Fig 3:**
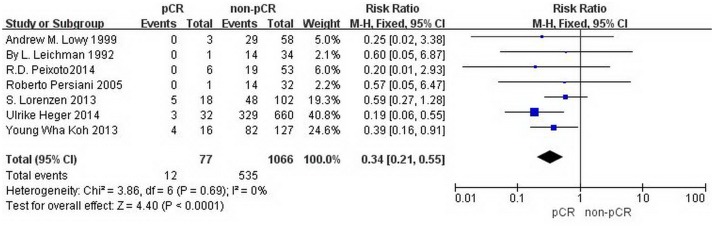
Forest plot of pooled relative risk for three-year OS from eligible studies. The area of each square represents the weighting, and the positions of each square demonstrate the risk ratio point estimate. Horizontal lines represent 95% confidence interval (CI). M-H = Mantel-Haenszel. Events = patients died from any cause within three years.

**Fig 4:**
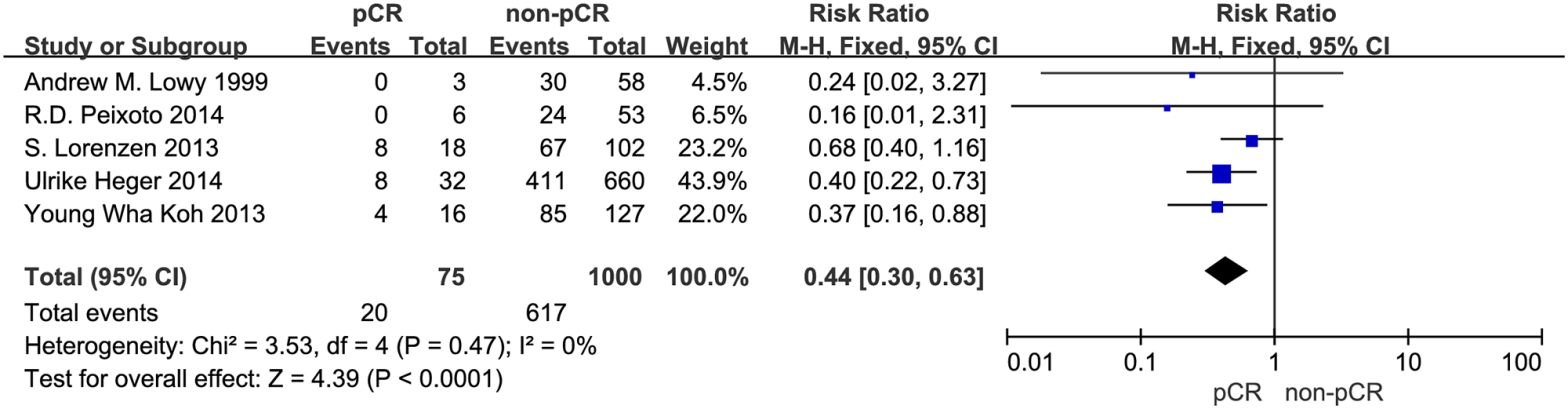
Forest plot of pooled relative risk for five-year OS from eligible studies. The area of each square represents the weighting, and the positions of each square demonstrate the risk ratio point estimate. Horizontal lines represent 95% confidence interval (CI). M-H = Mantel-Haenszel. Events = patients died from any cause within five years.

This meta-analysis discusses the difference between pCR and non-pCR in one-year, three-year and five-year overall survival, respectively. The data regarding the one-year and three-year overall survival were provided by all seven studies with 1143 patients. The RR and 95% CI for one-year overall survival in each study and the summary RR are shown in Fig 2. The summary estimate RR was 0.50 (95% CI, 0.25–0.98; p = 0.04). Heterogeneity testing revealed I^2^ = 0%, and the p for heterogeneity was 0.93 (fixed effects model). The RR and 95% CI for three-year overall survival in each study and the summary RR are shown in Fig 3. The summary estimate RR was 0.34 (95% CI, 0.21–0.55; p<0.0001 =. Heterogeneity testing revealed I^2^ = 0%, and the p for heterogeneity was 0.69 (fixed effects model). The data regarding the five-year overall survival were provided by five studies with 1075 patients. The RR and 95% CI for five-year overall survival in each study and the summary RR are shown in Fig 4. The summary estimate RR was 0.44 (95% CI, 0.3–0.63; p<0.0001 =. Heterogeneity testing revealed I^2^ = 0%, and the p for heterogeneity was 0.47 (fixed effects model).

### 2.3 Secondary end points

**Fig 5:**
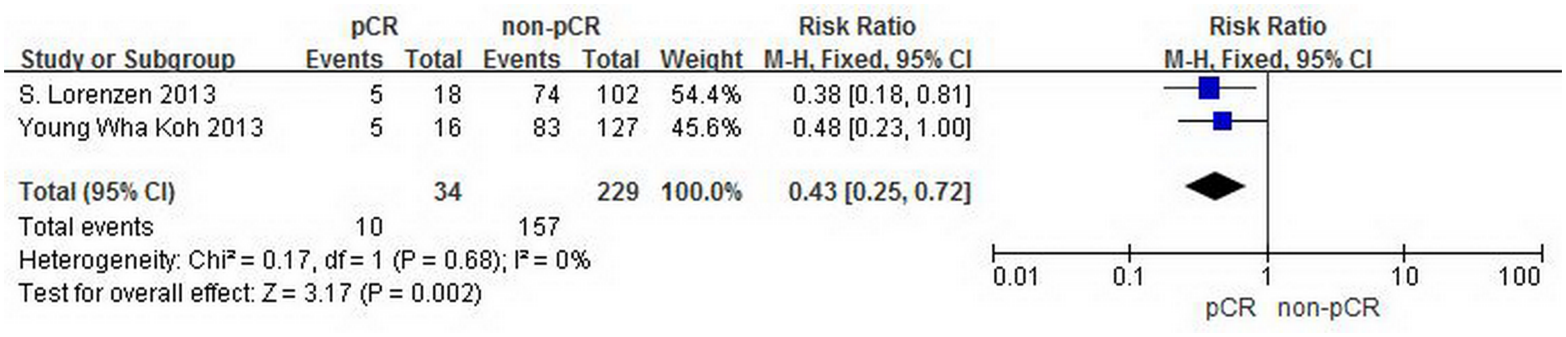
Forest plot of pooled relative risk for three-year DFS from eligible studies. The area of each square represents the weighting, and the positions of each square demonstrate the risk ratio point estimate. Horizontal lines represent 95% confidence interval (CI). M-H = Mantel-Haenszel. Events = recurrence or metastasis happened within three years.

The data for three-year disease-free survival were provided by two studies with 263 patients. The RR and 95% CI for three-year disease-free survival in each study and the summary RR are shown in Fig 5. The summary estimate RR was 0.43 (95% CI, 0.25–0.72; p = 0.002). Heterogeneity testing revealed I^2^ = 0%, and the p for heterogeneity was 0.17 (fixed effects model).

### 2.4 Subgroup analysis

Considering that D1 gastrectomy may affect the result of pCR, because of the lymph node dissection degree, further analysis was undertaken to investigate the correlation of pCR and survival in patients after D2 gastrectomy. Five studies with 1103 patients were included in the study. The summary estimate RR for one-year overall survival was 0.45 (95% CI, 0.22–0.94; p = 0.03). Heterogeneity testing revealed I^2^ = 0%, and the p for heterogeneity was 0.97 (fixed effects model). The summary estimate RR for three-year overall survival was 0.34 (95% CI, 0.20–0.56; p< 0.0001). Heterogeneity testing revealed I^2^ = 0%, and the p for heterogeneity was 0.49 (fixed effects model). And the summary estimate RR for five-year overall survival was 0.46 (95% CI, 0.31–0.66; p< 0.0001). Heterogeneity testing revealed I^2^ = 0%, and the p for heterogeneity was 0.44 (fixed effects model). All were similar to the previous results.(S2 File). The summary RR of three-year disease-free survival was the same as the previous one.

### 2.5 Publication bias

**Fig 6:**
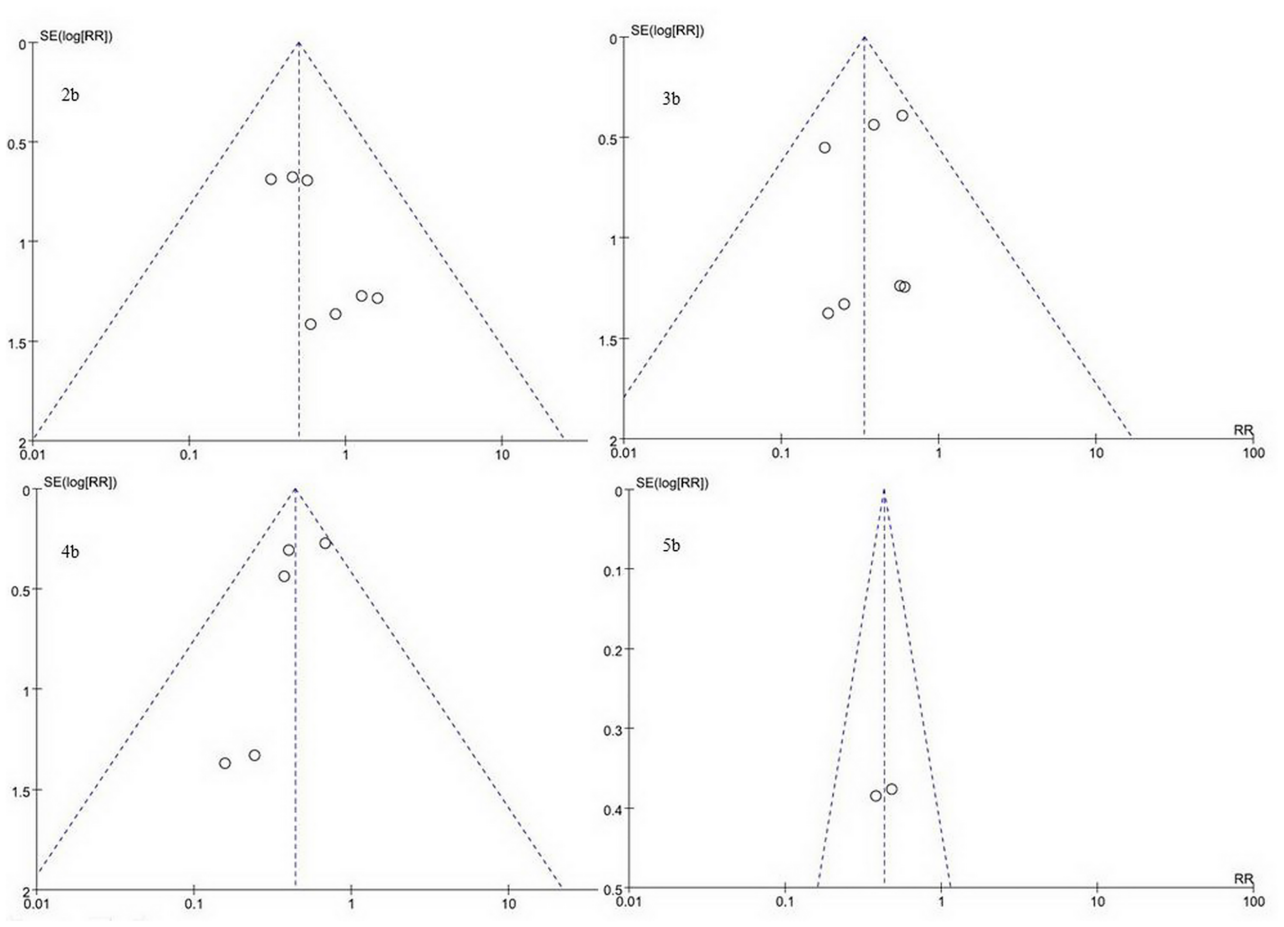
Funnel plots for publication bias of one-year OS (a), three-year OS (b), five-year OS (c), and three-year DFS (d). The vertical line indicates the pooled estimate of the overall risk ratio, with the sloping lines representing the expected 95% CI for a given standard error.

The Begg funnel plot (Fig 6) indicates the absence of remarkable asymmetry. Both the Begg test and the Egger test p values for one-year overall survival, three-year overall survival, five-year overall survival and three-year disease-free survival were not significant, respectively.

## 3. Discussion

As far as we are concerned, this is the first meta-analysis investigating the correlation between pCR and survival after neoadjuvant chemotherapy in gastric or gastroesophageal junction cancer. It proved that pCR was related to overall survival and disease-free survival, which was similar to the results of breast cancer, rectal cancer and bladder cancer.[4–9]

In this meta-analysis, the non-pCR patients were defined as patients after neoadjuvant chemotherapy and radical surgery without pCR, no matter whether they received R0 resection. The reason we included the R1 patients in our study was that R1 resection was related to a bad response to neoadjuvant chemotherapy. In fact, in Ulrike Heger`s 2014[23] study, the survival data of non-pCR patients did not include eight patients who did not receive pathological evaluation after surgery. From the survival curve, we found that the OS and DFS of the eight patients were worse than that of the other non-pCR patients. Furthermore, the survival data of non-pCR patients in the studies by Andrew M. Lowy 1999, Young Wha Koh 2013 and R.D. Peixoto 2014[12, 24, 25] did not include the patients who received R1 resection. Still, we can make the conclusion that pCR after neoadjuvant chemotherapy in gastric or gastroesophageal junction cancer was related to survival.

A few reasons accounted for the opposite conclusion reached by Brenner B, Andrew M and Lowy et al[11–13]. Firstly, all three of these studies were single-center retrospective studies with limited sample sizes. So, the insufficient number may lead to the bias. Additionally, in these studies, patients were divided into two groups, good pathologic response (>50%, >60% or >90% pathologic response) and the non-response group. Then, the long-term outcome was compared between groups. The long-term outcome of the good pathologic response group was significantly different from that of the pCR patients[28]. In addition, the median follow-up duration was relative shorter. Perhaps it was not long enough to show the difference between the two groups.

Compared with neoadjuvant chemotherapy, neoadjuvant chemoradiotherapy usually achieves a better pCR rate[29]. However, it is difficult to determine whether the long-term outcome of patients after neoadjuvant chemoradiotherapy is better than that of neoadjuvant chemotherapy. In other words, even if we have shown a correlation between pCR after neoadjuvant chemotherapy and survival, we cannot conclude that pCR after neoadjuvant chemoradiotherapy is related to survival. Although, a retrospective study from RC Fields[30] reported that recurrence at five years was significantly lower for patients with pCR after preoperative chemotherapy for gastric or preoperative chemoradiation for GEJ adenocarcinoma vs non-pCR patients. Additionally, a meta-analysis[6] reported that rectal cancer patients with pCR after chemoradiation have better a long-term outcome than those without pCR. We cannot agree that pCR after neoadjuvant chemoradiotherapy in gastric or gastroesophageal junction cancer is related to long-term outcome. In our opinion, the mechanism of chemotherapy and radiotherapy is quite different from each other. Chemotherapy is a systemic treatment, but radiotherapy is a local regional therapy. The theoretical foundation of the correlation between pCR and survival is that the systemic response of the tumor is similar to that of the local regional response. Therefore, the significance of pCR after neoadjuvant chemotherapy is quite different from that of neoadjuvant chemoradiotherapy. As for the result of RC Fields, on the one hand, it is a single-center retrospective study. On the other hand, some of the patients in the study received neoadjuvant chemotherapy instead of chemoradiotherapy, which would help the author conclude that pCR was related to long-term outcome. As for the meta-analysis of rectal cancer, the main reason underlying their conclusion is that local recurrence is the factor most important for the survival of rectal cancer patients. For the T3/T4 or N_+_ rectal cancer patients, the local recurrence rate is approximately 50% after surgery without neoadjuvant or adjuvant therapy.[31] So, rectal cancer patients can benefit from the therapy and have a better survival if local recurrence is well controlled. However, for gastric cancer, the main recurrent pattern is peritoneal implantation metastasis instead of local recurrence.[32, 33] Therefore, more evidence is needed to conclude whether pCR after neoadjuvant chemoradiotherapy is related to long-term outcome.

The pCR rate of gastric or gastroesophageal junction cancer patients after neoadjuvant chemotherapy is likely to be affected by the location of the tumor, the degree of differentiation, the Lauren classification and chemotherapy regimens and cycles.[14, 34, 35] The pCR rates of the studies in our meta-analysis are different from each other. This is due to the differences between pathological types and chemotherapy regimens and cycles. Because pCR rate after neoadjuvant chemotherapy is an important indicator of prognosis, can we improve the survival of patients by enhancing the chemotherapy regimens and cycles? There is still no conclusive data.

Our meta-analysis, which focused on the outcome based on pathologic stage of disease after preoperative chemotherapy, shows that pCR is clearly associated with a more than 50% lower risk of death and recurrence compared with patients with any residual disease. Probably, with the assistance of pCR, the extent of surgery does not necessary result in a compromised survival; however, narrowing the scope of the operation is not possible because of the limited cases available. Moreover, patients with gastric or GEJ adenocarcinoma who achieve pCR following preoperative therapy still have a significant risk of recurrence and cancer-specific death following resection. One third of the recurrences in the pCR group were symptomatic central nervous system (CNS) recurrences.[30] There is still no evidence whether patients who achieved pCR would benefit from the adjuvant chemotherapy after surgery.

This meta-analysis is of great clinical significance. Firstly, this article proved that patients who achieved pCR after neoadjuvant chemotherapy were more likely to be cured. It will also help to standardize adjuvant chemotherapy according to the downstaged grades of patients. Furthermore, molecular biological and genetics studies on the patients who achieved pCR will help to find sensitive targets for chemotherapy, as well as prognostic factors of gastric cancer. Finally, the pCR rate can be the surrogate endpoint of research about chemotherapy to reduce the length of the study period.

Our analysis, advantageous as it is, has limitations. In regard to the limitations of our published case-control-level meta-analysis, the correlation between pCR and survival cannot be adjusted with other prognostic factors using multivariate analysis. The recurrent pCR cases cannot be analyzed to evaluate further risk factors for the recurrence. There is no answer regarding whether patients who achieved pCR with different clinical stages would have a similar overall survival and disease-free survival. Moreover, some articles lack detailed descriptions of adjuvant chemotherapy after surgery, and we cannot exclude the differences in adjuvant chemotherapy between the pCR groups and non-pCR groups. Furthermore, this study is not as large as that for other disease settings, such as breast cancer or rectal cancer, which is likely due to the relatively lower incidence of gastric cancer and low pCR rate.[36] Besides, three included studies contained about 248 (less than 26.8%) gastroesophageal junction cancer patients in Seiwert I, which may interfer the final result. Considering that the proportion of Seiwert I was small and the subgroup analysis of the rest four studies achieved the same result (S3 File), the conclusion of this study was still credible. Finally, the trials included are heterogeneous in design (both prospective[26, 27] and retrospective, without randomized controlled trial), but our article is focused on the difference of survival between pCR and non-pCR patients. We cannot randomize the patients before surgery. Moreover, there is no statistical heterogeneity even in the presence of different types of studies.

## 4. Conclusion

Patients with resectable gastric or gastroesophageal junction cancer who achieved pCR after neoadjuvant chemotherapy have a better outcome compared with the patients without pCR.

## Supporting information

S1 Table The Newcastle-Ottawa Scale of case-control study. (DOC)

S1 File PRISMA checklist. (DOC)

S2 File Forest plot of pooled relative risk for one-year, three-year and five-year OS from studies undertaken D2 gastrectomy. (PDF)

S3 File Forest plot of pooled relative risk for one-year, three-year, five-year OS and three-year DFS from studies without gastroesophageal junction cancer. (PDF)

S4 File Abstracts from Pubmed. (PDF)

S5 File Abstracts from Embase. (PDF)

S6 File Abstracts from Web of science. (PDF)
