# Supplementary material for: Correlation of pathological complete response with survival after neoadjuvant chemotherapy in gastric or gastroesophageal junction cancer treated with radical surgery: A meta-analysis
Source: PLoS One. 2018 Jan 25;13(1):e0189294. doi: 10.1371/journal.pone.0189294 (PMC5784899; doi:10.1371/journal.pone.0189294)
Supplement: S1 Table — (DOC) [file pone.0189294.s001.doc]

**S1 Table. The Newcastle-Ottawa Scale of case-control study**

| Study | Selection | Comparability | Exposure |
| --- | --- | --- | --- |
|
| Leichman L/ 38 **^4^** |  |  |  |
| Persiani R/ 34 **^5^** |  |  |  |
| Heger U/ 723 **^6^** |  |  |  |
| Peixoto RD/ 83 **^7^** |  |  |  |
| Lorenzen S/ 120 **^8^** |  |  |  |
| Koh YW/ 143 **^9^** |  |  |  |
| Lowy AM/ 83**^10^** |  |  |  |
