# Supplementary figures and images for: Correlation of pathological complete response with survival after neoadjuvant chemotherapy in gastric or gastroesophageal junction cancer treated with radical surgery: A meta-analysis
Source: PLoS One. 2018 Jan 25;13(1):e0189294. doi: 10.1371/journal.pone.0189294 (PMC5784899; doi:10.1371/journal.pone.0189294)

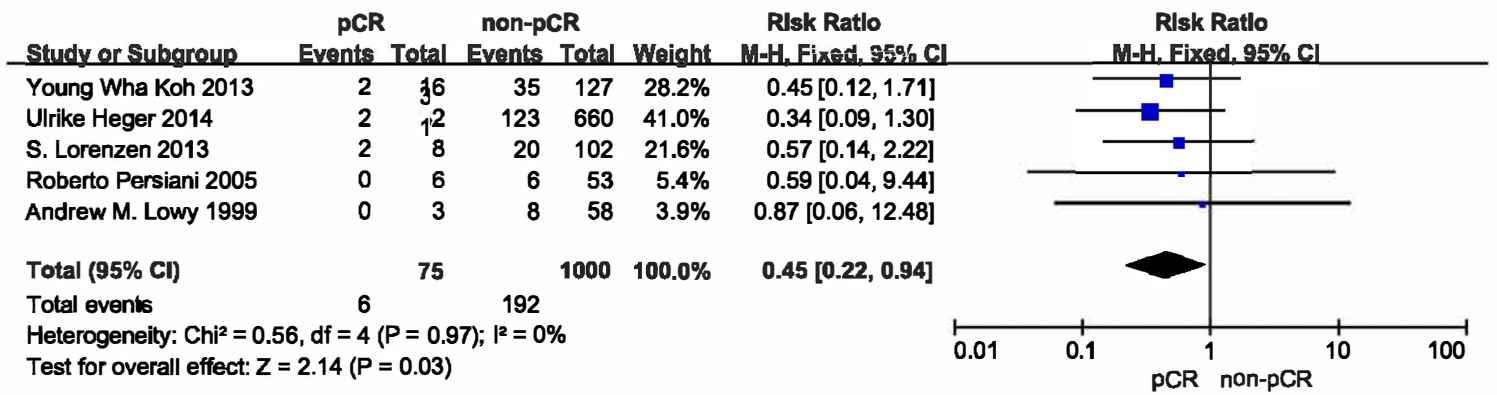

One year overall survival

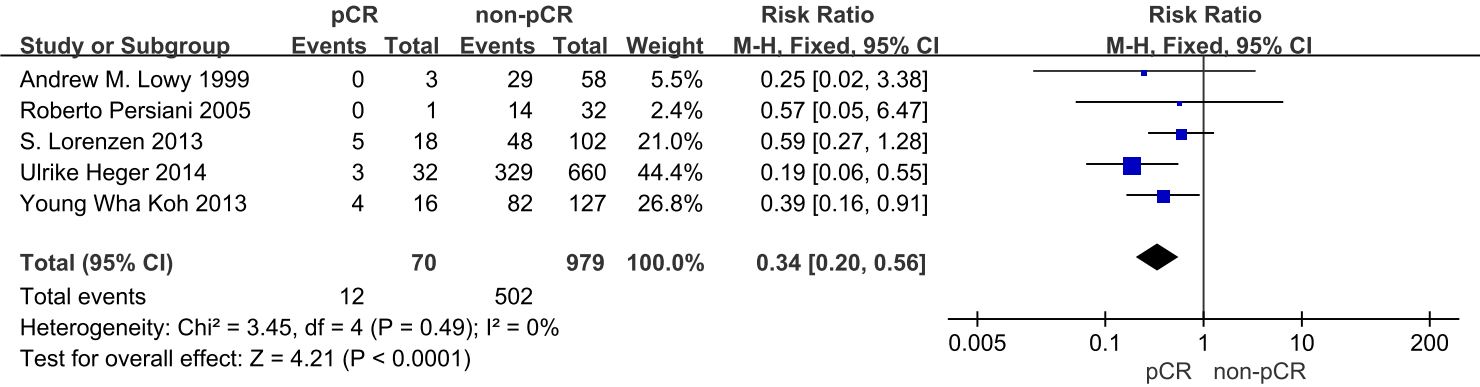

Three year overall survival

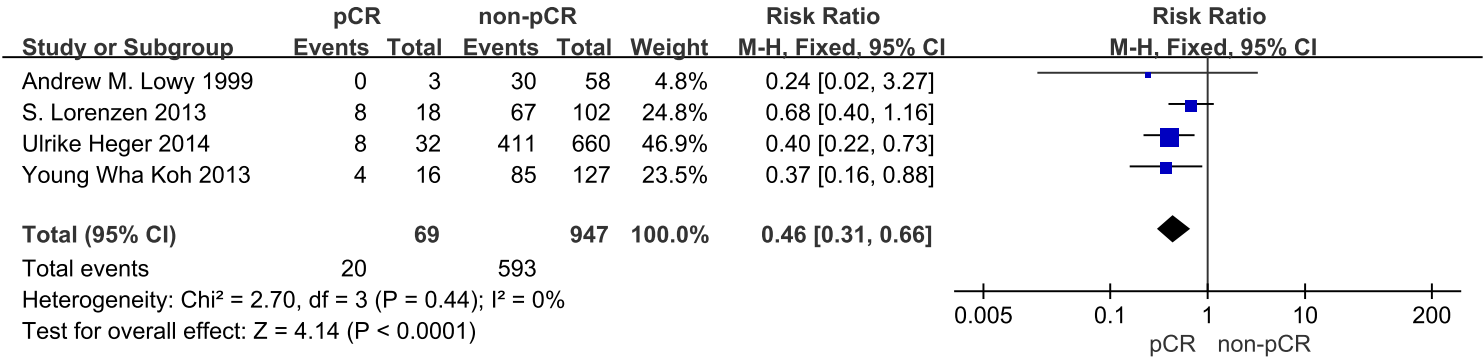

Five year overall survival

Supplement: S2 File — (PDF) [file pone.0189294.s003.pdf]
