# Supplementary material for: Correlation of pathological complete response with survival after neoadjuvant chemotherapy in gastric or gastroesophageal junction cancer treated with radical surgery: A meta-analysis
Source: PLoS One. 2018 Jan 25;13(1):e0189294. doi: 10.1371/journal.pone.0189294 (PMC5784899; doi:10.1371/journal.pone.0189294)
Supplement: S3 File — (PDF) [file pone.0189294.s004.pdf]

Study or Subgroup	pCR Events	pCR Total	non-pCR Events	non-pCR Total	Weight	Risk Ratio M-H, Fixed, 95% CI
Andrew M. Lowy 1999	0	3	8	58	10.5%	0.87 [0.06, 12.48]
By L. Leichman 1992	0	1	5	34	5.8%	1.59 [0.13, 19.77]
Roberto Persiani 2005	0	1	6	32	7.2%	1.27 [0.10, 15.42]
Young Wha Koh 2013	2	16	35	127	76.4%	0.45 [0.12, 1.71]
**Total (95% CI)**		**21**		**251**	**100.0%**	**0.62 [0.23, 1.68]**
Total events	2		54			

Heterogeneity: Chi² = 1.12, df = 3 (P = 0.77); I² = 0%

Test for overall effect: Z = 0.94 (P = 0.35)

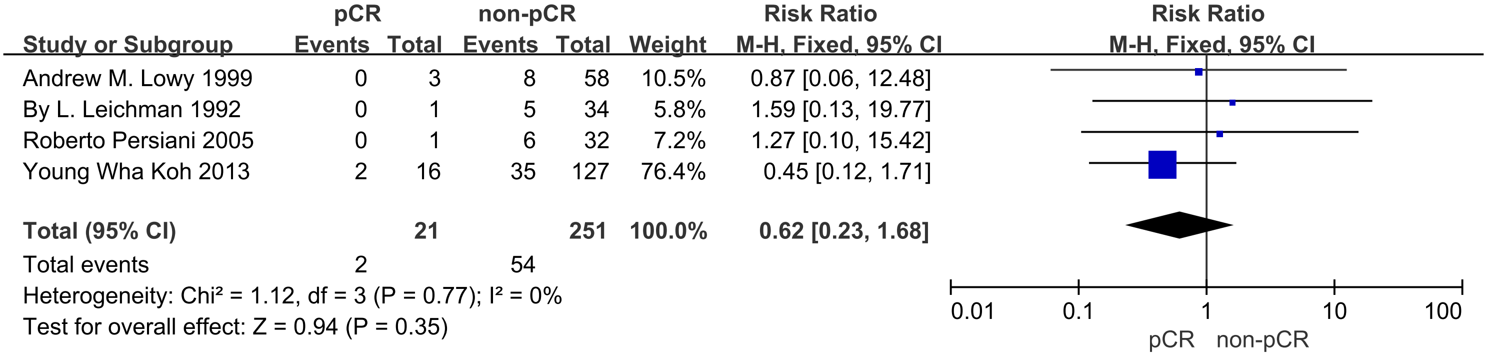

One year overall survival

	pCR		non-pCR			Risk Ratio
**Study or Subgroup**	**Events**	**Total**	**Events**	**Total**	**Weight**	**M-H, Fixed, 95% CI**
Andrew M. Lowy 1999	0	3	29	58	16.0%	0.25 [0.02, 3.38]
By L. Leichman 1992	0	1	5	34	2.5%	1.59 [0.13, 19.77]
Roberto Persiani 2005	0	1	6	32	3.2%	1.27 [0.10, 15.42]
Young Wha Koh 2013	4	16	82	127	78.3%	0.39 [0.16, 0.91]
**Total (95% CI)**		**21**		**251**	**100.0%**	**0.42 [0.20, 0.89]**
Total events	4		122			

Heterogeneity: Chi² = 2.00, df = 3 (P = 0.57); I² = 0%

Test for overall effect: Z = 2.27 (P = 0.02)

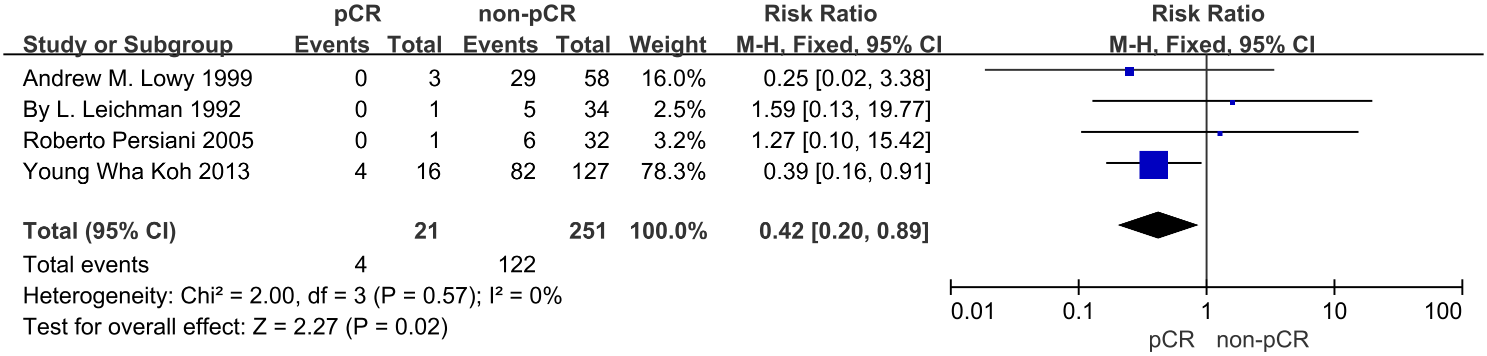

Three year overall survival

Study or Subgroup	pCR Events	pCR Total	non-pCR Events	non-pCR Total	Weight	Risk Ratio M-H, Fixed, 95% CI
Young Wha Koh 2013	4	16	85	127	83.1%	0.37 [0.16, 0.88]
Andrew M. Lowy 1999	0	3	30	58	16.9%	0.24 [0.02, 3.27]
**Total (95% CI)**		**19**		**185**	**100.0%**	**0.35 [0.15, 0.80]**
Total events	4		115			

Heterogeneity: Chi² = 0.10, df = 1 (P = 0.75); I² = 0%

Test for overall effect: Z = 2.50 (P = 0.01)

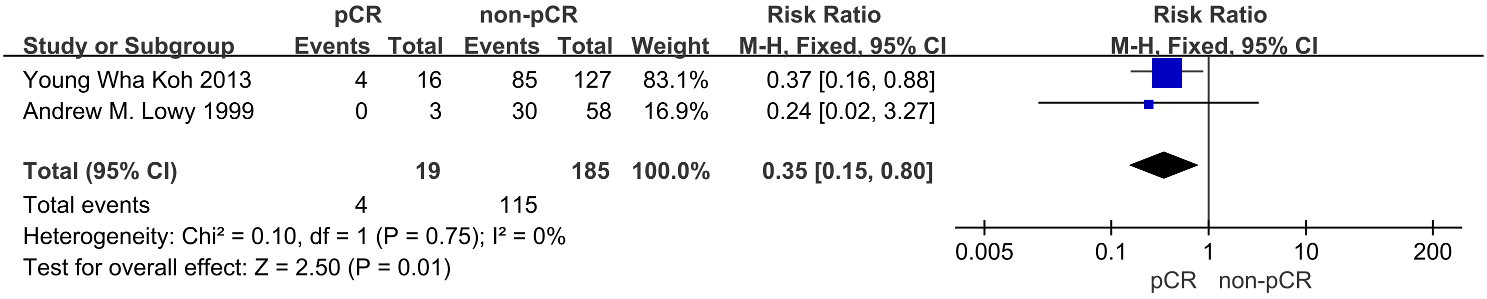

Five year overall survival

Study or Subgroup	pCR Events	pCR Total	non-pCR Events	non-pCR Total	Weight	Risk Ratio M-H, Fixed, 95% CI
Young Wha Koh 2013	5	16	83	127	100.0%	0.48 [0.23, 1.00]
**Total (95% CI)**		**16**		**127**	**100.0%**	**0.48 [0.23, 1.00]**
Total events	5		83			

Heterogeneity: Not applicable
Test for overall effect: Z = 1.96 (P = 0.05)

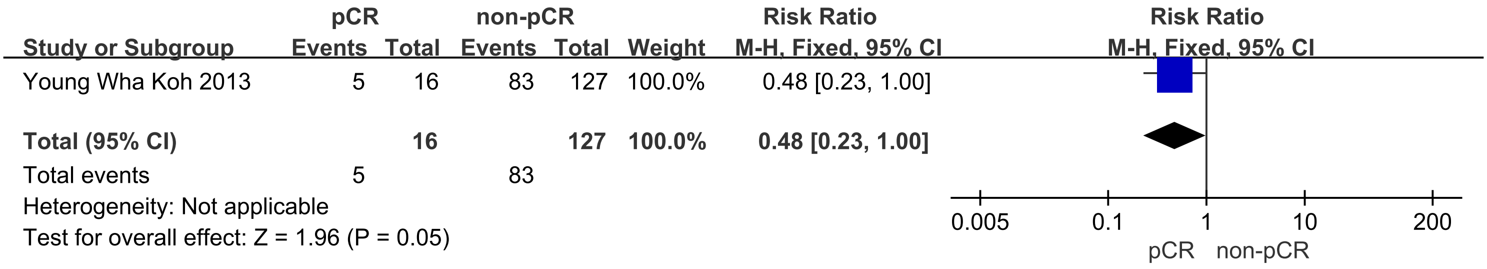

Three year disease free survival
