# Supplementary material for: Correlation of pathological complete response with survival after neoadjuvant chemotherapy in gastric or gastroesophageal junction cancer treated with radical surgery: A meta-analysis
Source: PLoS One. 2018 Jan 25;13(1):e0189294. doi: 10.1371/journal.pone.0189294 (PMC5784899; doi:10.1371/journal.pone.0189294)
Supplement: S4 File — (PDF) [file pone.0189294.s005.pdf]

Items 1 -100 of 100 (Display the 100 citations in PubMed)

1. Gan To Kagaku Ryoho. 2014 Dec;41(13):2611-4.

[A case of early gastric cancer with multiple synchronous bone metastases treated complete response with S-1+CDDP].

[Article in Japanese]

Takishita C(1), Yajima K, Iwasaki Y, Ohashi M, Iwanaga T, Oohinata R.

Author information:
(1)Dept. of Surgery, Tokyo Metropolitan Cancer and Infectious Diseases Center Komagome Hospital.

We report a case of complete response (CR) following induction chemotherapy using S-1 for a patient with early gastric cancer accompanied by multiple synchronous bone metastases. An asymptomatic 70-year-old woman was diagnosed with early gastric cancer by upper gastrointestinal endoscopy during a periodic medical examination. An abdomino-pelvic computed tomography (CT) scan revealed no primary tumor in the stomach and the absence of lymph node or liver metastases. However, osteoplastic changes were detected in the lumbar vertebrae and the ilium. Multiple synchronous bone metastases from early gastric cancer were detected on magnetic resonance imaging, bone scintigraphy, and positron emission tomography-CT. After a regimen consisting of 15 courses of S-1 plus cisplatin (CDDP), and an additional 5 courses of S-1 were administered, clinical CR was confirmed for the bone metastases. Laparoscopic distal gastrectomy with D1 lymphadenectomy was performed for treating the primary gastric cancer 33 months after the initiation of chemotherapy. Pathological CR was also achieved for the primary gastric cancer. Imaging analysis did not show disease progression 48 months after the initiation of chemotherapy. Synchronous bone metastases from early gastric cancer are extremely rare, and a good outcome was achieved in the present case through induction chemotherapy.

PMID: 25596058 [PubMed - indexed for MEDLINE]

2. Ann Surg Oncol. 2015 Jan 7. [Epub ahead of print]

Safety, Efficacy, and Long-Term Follow-Up Evaluation of Perioperative Epirubicin, Cisplatin, and Capecitabine Chemotherapy in Esophageal Resection for Adenocarcinoma.

van der Sluis PC(1), Ubink I, van der Horst S, Boonstra JJ, Voest EE, Ruurda JP, Borel Rinkes IH, Wiezer MJ, Schipper ME, Siersema PD, Los M, Lolkema MP, van Hillegersberg R.

Author information:
(1)Department of Surgery, G04.228, University Medical Center Utrecht, Utrecht, The Netherlands, P.C.vanderSluis-2@umcutrecht.nl.

BACKGROUND: Perioperative epirubicin, cisplatin, and capecitabine (ECC) chemotherapy was evaluated in patients who underwent esophageal resection for adenocarcinoma of the esophagus or gastroesophageal junction (GEJ).
METHODS: A cohort of 93 consecutive patients was analyzed. The median follow-up period was 60 months. Source data verification of adverse events was performed by two independent observers.
RESULTS: All three planned preoperative chemotherapy cycles were administered to 65 patients (69.9 %). Only 27 % of the patients completed both pre- and postoperative chemotherapy. The reasons for not receiving postoperative adjuvant chemotherapy could be separated in two main problems: toxicity of the preoperative chemotherapy and postoperative problems involving difficulty in recovery and postoperative complications. Finally, 25 patients (27 %), completed three preoperative and three postoperative cycles. Grades 3 and 4 nonhematologic adverse events of preoperative chemotherapy mainly consisted of thromboembolic events (16.2 %) and cardiac complications (7.5 %). A history of cardiac and vascular disease was independently associated with discontinuation of preoperative chemotherapy and the occurrence of grade 3 or higher adverse events. Surgery was performed for 94 % of all the patients who started with ECC chemotherapy. A radical resection (R0) was achieved in 93 % of the patients. A complete pathologic response was observed in 8 % of the patients. During a median follow-up period of 60 months, the median disease-free survival time was 28 months, and the median overall survival time was 36 months. The 3-year overall survival rate was 50 %, and the 5-year overall survival rate was 42 %.
CONCLUSION: For patients with adenocarcinoma of the esophagus or GEJ, six cycles of ECC-based perioperative chemotherapy is associated with a relatively high number of adverse events. Although this toxicity did not affect the esophageal resectability rate, this regimen should be used with caution in this patient

population.

PMID: 25564156 [PubMed - as supplied by publisher]

3. World J Surg Oncol. 2014 Dec 29;12:397. doi: 10.1186/1477-7819-12-397.

Clinical evaluation of CEA, CA19-9, CA72-4 and CA125 in gastric cancer patients with neoadjuvant chemotherapy.

Sun Z, Zhang N(1).

Author information:
(1)Beijing Shijitan Hospital, Capital Medical University, Room 334, Administrative Building, Beijing 100038, China. zhangnw1@sohu.com.

BACKGROUND: In the clinical practice of neoadjuvant chemotherapy, response markers are very important. We aimed o investigate whether tumor markers CEA(carcino-embryonic antigen), CA19-9(carbohydrate antigen 19-9), CA72-4(carbohydrate antigen 72-4), and CA125(carbohydrate antigen 125) can be used to evaluate the response to neoadjuvant chemotherapy, and to evaluate the diagnosis and prognosis value of four tumor markers in the patients of gastric cancer.
METHODS: A retrospective review was performed of 184 gastric cancer patients who underwent a 5-Fu, leucovorin, and oxaliplatin (FOLFOX) neoadjuvant chemotherapy regimen, followed by surgical treatment. Blood samples for CEA, CA19-9, CA72-4, and CA125 levels were taken from patients upon admission to the hospital and after neoadjuvant chemotherapy. Statistical analysis was performed to identify the clinical value of these tumor markers in predicting the survival and the response to neoadjuvant chemotherapy.
RESULTS: Median overall survival times of pretreatment CA19-9-positive and CA72-4-positive patients (14.0 +/-2.8 months and 14.8 +/-4.0 months, respectively) were significantly less than negative patients (32.5 +/-8.9 months and 34.0 +/-10.1 months, respectively) (P = 0.000 and P = 0.002, respectively). Pretreatment status of CA19-9 and CA72-4 were independent prognostic factors in gastric cancer patients (P = 0.029 and P = 0.008, respectively). Pretreatment CEA >50 ng/ml had a positive prediction value for clinical disease progression after neoadjuvant chemotherapy according to the ROC curve (AUC: 0.694, 95% CI: 0.517 to 0.871, P = 0.017). The decrease of tumor markers CEA, CA72-4, and CA125 was

significant after neoadjuvant chemotherapy (P = 0.030, P = 0.010, and P = 0.009, respectively), especially in patients with disease control (including complete, partial clinical response, and stable disease) (P = 0.012, P = 0.020, and P = 0.025, respectively). A decrease in CA72-4 by more than 70% had a positive prediction value for pathologic response to neoadjuvant chemotherapy according to the ROC curve (AUC: 0.764, 95% CI: 0.584 to 0.945, P = 0.020).
CONCLUSIONS: Our results suggest that high preoperative serum levels of CA72-4 and CA19-9 are associated with higher risk of death, high pretreatment CEA levels (>50 ng/ml) may predict clinical disease progression after neoadjuvant chemotherapy, and a decrease (>70%) of CA72-4 may predict pathologic response to neoadjuvant chemotherapy.

PMCID: PMC4320462
PMID: 25543664 [PubMed - in process]

4. Int J Cancer. 2014 Dec 22. doi: 10.1002/ijc.29403. [Epub ahead of print]

NeoFLOT: Multicenter phase II study of perioperative chemotherapy in resectable adenocarcinoma of the gastroesophageal junction or gastric adenocarcinoma-Very good response predominantly in patients with intestinal type tumors.

Schulz C(1), Kullmann F, Kunzmann V, Fuchs M, Geissler M, Vehling-Kaiser U, Stauder H, Wein A, Al-Batran SE, Kubin T, Schäfer C, Stintzing S, Giessen C, Modest DP, Ridwelski K, Heinemann V.

Author information:
(1)Department of Medical Oncology, Klinikum Grosshadern and Comprehensive Cancer Center, University of Munich, Munich, Germany.

Perioperative treatment is a standard of care in locally advanced gastroesophageal cancer (GEC) (gastric adenocarcinoma and gastroesophageal junction (GEJ) adenocarcinoma). While preoperative treatment can be applied to the majority of patients, postoperative chemotherapy can be given only to a fraction. The NeoFLOT-study therefore investigates the application of prolonged neoadjuvant chemotherapy (NACT). Patients with T3, T4, and/or node-positive adenocarcinoma (GEC) were eligible for this multicenter phase II trial. NACT consisted of 6 cycles of oxaliplatin 85 mg/m(2) , leucovorin 200 mg/m(2) , 5-fluorouracil 2600 mg/m(2) and docetaxel 50 mg/m(2) (FLOT) applied q 2 wks.

Application of adjuvant chemotherapy was explicitly not part of the protocol. R0-resection rate was evaluated as a primary endpoint. Of 59 enrolled patients, 50 patients underwent surgery and were assessable for the primary endpoint. R0-resection rate was 86.0% (43/50). Pathologic complete response (pCR) was 20.0% (10/50) and a further 20% (10/50) of patients achieved near complete histological remission (<10% residual tumor). Among these very good responders, 85% (17/20) had intestinal type tumors, 10% (2/20) had diffuse and 5% (1/20) had mixed type tumors. After 3 cycles of NACT, 6.9% (4/58) of patients developed progressive disease. Median disease-free survival was 32.9 months. The 1-year survival-rate was 79.3%. Grade 3-4 toxicities included neutropenia 29.3%, febrile neutropenia 1.7%, diarrhea 12.1% and mucositis 6.9%. This study indicates that intensified NACT with 6 cycles of FLOT is highly effective and tolerable in resectable GEC. Very good response (pCR and <10% residual tumor) was predominantly observed in patients with intestinal type tumors.

© 2014 UICC.

PMID: 25530271 [PubMed - as supplied by publisher]

5. Zhonghua Wei Chang Wai Ke Za Zhi. 2014 Nov;17(11):1062-7.

[Application of molecular targeted agents in comprehensive treatment of gastrointestinal cancer].

[Article in Chinese]

Li J(1), Shen L.

Author information:
(1)Key Laboratory of Carcinogenesis and Translational Research (Ministry of Education), Department of Gastrointestinal Oncology, Peking University Cancer Hospital and Institute, Beijing 100142, China. linshenpku@163.com.

Targeted agents increase response rates and improved overall survival in treatment of metastatic gastrointestinal cancer. Therefore, physicians pay more attention to the role of targeted agents in treatment of local advanced gastrointestinal cancer. The clinical trials are ongoing to evaluate the efficacy of Trastuzumab in neoadjuvant treatment of local advanced gastric cancer with

HER-2 gene over expression. Many studies reported Cetuximab plus chemotherapy as a conversion treatment improve R0 resection rates and prolonged overall survival of the patients with potentially resectable colorectal cancer liver metastasis with wild type KRAS gene status. A phase III(clinical trial is assessing the conversion efficacy of Bevacizumab in unresectable disease with KRAS gene mutation. Current evidence showed that neoadjuvant therapy of targeted agents did not prolong survival of patients with resectable liver metastasis. However, this is controversial. In neoadjuvant therapy of local advanced rectal cancer, Cetuximab did not improve the rates of pathological complete response in most of the phase II(trials. Furthermore, there are no phase III(trials to assess the role of Bevacizumab. Compared to chemotherapy alone for metastatic cancer, it is more important to evaluate the interaction and synergistic action of targeted agents, cytotoxic drugs, surgery and radiation, to make a scientific multidisciplinary model in comprehensive treatment of local advanced cancer.

PMID: 25421761 [PubMed - in process]

6. Case Rep Oncol Med. 2014;2014:532924. doi: 10.1155/2014/532924. Epub 2014 Sep 14.

Pathological complete response and long-term survival in a very elderly patient after neoadjuvant chemotherapy for locally advanced, unresectable gastric cancer.

Izuishi K(1), Kobayashi M(2), Sano T(1), Mori H(1), Ebara K(1).

Author information:
(1)Department of Gastroenterological Surgery, Federation of Public Services and Affiliated Personnel Aid Associations, Takamatsu Hospital, 4-18 Tenjinmae, Takamatsu, Kagawa 760-0018, Japan. (2)Department of Internal Medicine of Gastroenterology, Federation of Public Services and Affiliated Personnel Aid Associations, Takamatsu Hospital, 4-18 Tenjinmae, Takamatsu, Kagawa 760-0018, Japan.

We address the pathological complete response and long-term survival of elderly patients after neoadjuvant chemotherapy in locally advanced, unresectable gastric cancer. An 83-year-old man was hospitalized for upper abdominal pain. Gastrointestinal endoscopy showed a large tumor spanning from the gastric angle to the antrum, and extending to the duodenum. Histological analysis of the biopsy

specimen revealed a poorly differentiated adenocarcinoma. Computed tomography images showed thickening of the gastric wall and invasion of the body and head of the pancreas, but did not show distant metastases. The patient was diagnosed with unresectable gastric cancer, and was treated with neoadjuvant chemotherapy using S-1 (80 mg/m(2)) and paclitaxel (60 mg/m(2)). After the third course of chemotherapy, gastrointestinal endoscopy and abdominal computed tomography revealed a remarkable reduction in tumor size. This reduction allowed distal gastrectomy to be conducted. Histological examination of the specimen revealed no cancer cells in the primary lesion or lymph nodes. The patient was treated with adjuvant chemotherapy of oral tegafur-uracil (300 mg/day) for one year after surgery. He lived for five years after surgery without recurrence. Neoadjuvant chemotherapy using S-1 and paclitaxel is a potent strategy for improving survival in very elderly patients with unresectable gastric cancer.

PMCID: PMC4179970
PMID: 25298899 [PubMed]

7. Asian Pac J Cancer Prev. 2014;15(18):7737-40.

The neutrophil to lymphocyte ratio has a high negative predictive value for pathologic complete response in locally advanced breast cancer patients receiving neoadjuvant chemotherapy.

Eryilmaz MK(1), Mutlu H, Salim DK, Musri FY, Tural D, Coskun HS.

Author information:
(1)Department of Medical Oncology, Akdeniz University School of Medicine, Antalya, TurkeyE-mail : drangelkarakurt@hotmail.com.

BACKGROUND: The neutrophil-to-lymphocyte ratio (NLR) is a strong predictor of mortality in patients with pancreatic, colorectal, lung, gastric cancer and renal cell carcinoma. The aim of this study was to determine the relationship between pathological complete response (pCR) and pretreatment NLR values in locally advanced breast cancer (BC) patients receiving neoadjuvant chemotherapy (NACT).
MATERIALS AND METHODS: Datawere collected retrospectively from the Akdeniz University School of Medicine Database for locally advanced BC patients treated with NACT between January 2000- December 2013.
RESULTS: A total of 78 patients were analyzed. Sixteen (20%) patients achieved

pCR. Estrogen receptor (ER) positivity was lower in pCR+ than pCR- cases (p=0.011). The median NLR values were similar in both arms. The optimum NLR cut-off point for BC patients with PCR+ was 2.33 (AUC:0.544, 95%CI [0.401-0.688], p=0.586) with sensitivity, specificity, positive predictive value and negative predictive value (NPV) of 50%, 51,6%, 21,1%, and 80%, respectively.
CONCLUSIONS: This study showed no relationship between the pCR and pretreatment NLR values. Because of a considerable high NPV, in the patients with higher NLR who had luminal type BC in which pCR is lower after NACT, such treatment may not be recommended.

PMID: 25292055 [PubMed - in process]

8. Eur J Surg Oncol. 2014 Oct;40(10):1321-30. doi: 10.1016/j.ejso.2014.01.006. Epub 2014 Feb 25.

Clinical effectiveness of neoadjuvant chemotherapy in advanced gastric cancer: an updated meta-analysis of randomized controlled trials.

Xiong B(1), Ma L(2), Cheng Y(3), Zhang C(4).

Author information:
(1)Department of General Surgery, The First Affiliated Hospital of Chongqing Medical University, No. 1, Youyi Road, Yuanjiagang, Yuzhong District, Chongqing 400016, PR China. Electronic address: xbh791220@163.com. (2)Department of Internal Medicine, Chongqing Huaxi Hospital, Banan District, Chongqing 400054, PR China. (3)Department of General Surgery, The First Affiliated Hospital of Chongqing Medical University, No. 1, Youyi Road, Yuanjiagang, Yuzhong District, Chongqing 400016, PR China. Electronic address: 351893768@qq.com. (4)Department of General Surgery, The First Affiliated Hospital of Chongqing Medical University, No. 1, Youyi Road, Yuanjiagang, Yuzhong District, Chongqing 400016, PR China. Electronic address: 280343115@qq.com.

AIMS: To assess the efficacy and safety of neoadjuvant chemotherapy (NAC) for advanced gastric cancer (AGC).
METHODS: By searching electronic databases (PubMed, Embase, Cochrane Library) and ASCO proceedings from 1990 to 2012, all randomized controlled trials (RCTs) which compared the effect of NAC-combined surgery versus surgery alone in AGC were included. All calculations and statistical tests were performed using RevMan 5.0

software.
RESULTS: 12 RCTs with a total of 1820 patients were included. All patients had locally advanced but resectable gastric cancer and received NAC. NAC can slightly improve the survival rate (OR = 1.32, 95% confidence interval (CI): 1.07-1.64, P = 0.01), with little or no significant benefits in subgroup analyses between either different population or regimens. NAC can significantly improve the 3-year progression-free survival (PFS) (OR: 1.85, 95% CI: 1.39-2.46, p < 0.0001), tumor down-staging rate (OR: 1.71, 95% CI: 1.26, 2.33, p = 0.0006) and R0 resection rate (OR: 1.38, 95% CI: 1.08-1.78, P = 0.01) of patients with AGC. There was no difference between the two arms, in terms of relapse rates (OR: 1.03, 95% CI: 0.60-1.78, p = 0.92), operative complications (OR: 1.20, 95% CI: 0.90-1.58, p = 0.21), perioperative mortality (OR: 1.14, 95% CI: 0.64-2.05, p = 0.65) and grade 3/4 adverse effects: gastrointestinal problem (OR: 0.57, 95% CI: 0.25-1.30, p = 0.18), leukopenia (OR: 0.88, 95% CI: 0.41-1.91, p = 0.75), thrombocytopenia (OR: 1.27, 95% CI: 0.27-5.93, p = 0.76).
CONCLUSION: NAC is effective and safe. However, further prospective multi-national and multi-center RCTs are still needed in order to investigate the long-term oncological and functional outcomes to define the clinical benefits of NAC and the most effective strategies for AGC.

Copyright © 2014 Elsevier Ltd. All rights reserved.

PMID: 25239442 [PubMed - indexed for MEDLINE]

9. Cancer Chemother Pharmacol. 2014 Dec;74(6):1139-47. doi: 10.1007/s00280-014-2586-6. Epub 2014 Sep 19.

Docetaxel, cisplatin, and fluorouracil combination in neoadjuvant setting in the treatment of locally advanced gastric adenocarcinoma: Phase II NEOTAX study.

Ozdemir N(1), Abali H, Vural M, Yalcin S, Oksuzoglu B, Civelek B, Oguz D, Bostanci B, Yalcin B, Zengin N.

Author information:
(1)Department of Medical Oncology, Ankara Numune Training and Research Hospital, Ankara, Turkey.

PURPOSE: This phase II trial aimed to evaluate the efficacy and safety of

docetaxel, cisplatin, and fluorouracil (DCF) combination in neoadjuvant setting in patients with locally advanced gastric adenocarcinoma.
METHODS: Fifty-nine patients with resectable or unresectable locally advanced gastric and gastroesophageal cancer were recruited in this multicenter, single-arm, open-label, local clinical phase II study conducted at three centers from Turkey between June 2006 and March 2012. Patients had T3-4 or lymph node-positive disease. After staging with imaging and laparotomy or laparoscopy, they received three cycles of DCF with lenograstim. Imaging studies were repeated after the last two cycles. Patients who underwent surgery were followed up for at least 1 year after the surgery. Toxicity and response were evaluated in accordance with NCI-CTC version3.0 and RECIST 1.0.
RESULTS: At baseline, 66.1 % of patients were considered resectable. In 47 patients evaluable, partial response in 16 (34.0 %), stable disease in 27 (57.5 %), and progressive disease in four (8.5 %) were observed. Forty-six patients underwent surgery. In 38 (64.4 %; 95 % confidence interval (CI) 52.2-76.6 %) out of 59 patients, complete resection (R0) was achieved. Median overall and disease-free survival were 19.1 months (95 % CI 13.5-24.7) and 11.6 months (95 % CI 5.9-17.4), respectively. The most frequent grade 3-4 adverse events were neutropenia (52.5 %), febrile neutropenia (11.9 %), leukopenia (39.0 %), and diarrhea (10.5 %). One patient died from an unknown cause.
CONCLUSIONS: Classical DCF triplet with lenograstim showed a good clinical response with acceptable safety profile in the treatment of locally advanced gastric and gastroesophageal cancer with a significant R0 rate and manageable toxicity.

PMID: 25234436 [PubMed - indexed for MEDLINE]

10. Oncology. 2014;87(6):371-80. doi: 10.1159/000366425. Epub 2014 Sep 13.

O-MAX chemotherapy: high activity in metastatic esophagogastric adenocarcinoma and possible relation to subclinical hemolysis.

Ahlgren J(1), Patel N, Simmens S, Akin E, Bishop C, Kirkel D, Siegel P, Schuck S, Guebre-Xabiher H, Siegel R.

Author information:
(1)Division of Hematology and Oncology, The George Washington University Medical Center, Washington, D.C., USA.

OBJECTIVES: Our objectives were to confirm the activity of O-MAX chemotherapy in adenocarcinoma of the stomach and esophagus, particularly the high rate of complete remission (CR) and the relation of subclinical hemolysis to CR.
PATIENTS AND METHODS: Twenty-five patients with metastatic esophagogastric adenocarcinoma were treated with O-MAX. Two developed cancer-related hemolytic-uremic syndrome (C-HUS); both achieved CR. Subsequent patients were monitored for serum haptoglobin for subclinical hemolysis.
RESULTS: Median survival was 16.5 months. The objective response rate was 90%, with 38% CR. Three patients achieving CR relapsed in the central nervous system and died (2 without systemic disease). Four patients have remained alive, off therapy, the longest for 20 years. Two patients developed clinical C-HUS and 5 of 8 monitored patients developed subclinical hemolysis based on abnormal serum haptoglobin. Four of the patients with subclinical hemolysis achieved CR. Of the 7 patients developing clinical C-HUS or subclinical hemolysis, 6 (86%) achieved CR.
CONCLUSIONS: O-MAX appears highly active in esophagogastric adenocarcinoma. A few long-term survivors of metastatic disease are being seen. CR and long-term survival appear to correlate with the development of hemolysis. Although highly promising, these results should be considered only as hypothesis-generating and require confirmation in a prospective trial.

© 2014 S. Karger AG, Basel.

PMID: 25227924 [PubMed - indexed for MEDLINE]

11. Ann Surg Oncol. 2015 Mar;22(3):787-92. doi: 10.1245/s10434-014-4084-9. Epub 2014
Sep 16.

Long-term Survival Outcomes of Advanced Gastric Cancer Patients Who Achieved a Pathological Complete Response with Neoadjuvant Chemotherapy: A Systematic Review of the Literature.

Cho H(1), Nakamura J, Asaumi Y, Yabusaki H, Sakon M, Takasu N, Kobayashi T, Aoki T, Shiraishi O, Kishimoto H, Nunobe S, Yanagisawa S, Suda T, Ueshima S, Matono S, Maruyama H, Tatsumi M, Seya T, Tanizawa Y, Yoshikawa T.

Author information:
(1)Department of Gastrointestinal Surgery, Kanagawa Cancer Center, Yokohama, Japan.

BACKGROUND: A pathologic complete response (pCR) can sometimes be induced by intensive or long-term neoadjuvant chemotherapy (NAC). This prognostic research study based on a systematic review of the literature evaluated the impact of a pCR on the long-term survival of gastric cancer (GC) patients.
METHODS: Articles were extracted from PubMed and the Japanese medical search engine "Ichu-shi," using the terms "GC," "NAC," and "pCR." Articles were selected based on the following criteria: (1) full-text case report, (2) R0 resection following NAC for locally advanced GC, and (3) pathological complete response in both the primary stomach and in the lymph nodes. A questionnaire regarding the patients' prognoses was sent to the corresponding authors of the articles selected in July 2013.
RESULTS: Twenty-four articles met the criteria. Twenty authors responded to the questionnaire. Finally, 22 patients from 20 articles were entered into the present study. The median follow-up time (range) of the survivors was 76 (range 13-161) months. Tumors that were stage III/IV (86 %: 19/22) and of an undifferentiated histology (61.9 %: 13/21) were dominant. An S1-based regimen was frequently selected for the NAC. All patients underwent R0 resection and D2/D3 lymphadenectomy. The overall survival and recurrence-free survival rates at 3 and 5 years were 96 % and 85 % and 91 % and 75 %, respectively.
CONCLUSIONS: Although a pCR was a relatively rare event, a high pCR rate would be helpful to select the regimen and courses of NAC, especially when the pathological response rates are similar.

PMID: 25223927 [PubMed - in process]

12. Gan To Kagaku Ryoho. 2014 Aug;41(8):1009-12.

[A case of advanced gastric cancer with multiple liver metastases treated with preoperative TS-1/CDDP chemotherapy and resection, with a complete response and survival for 7 years].

[Article in Japanese]

Mitan M(1), Miyamoto M, Tsuda H, Nishiwaki K, Kagawa I, Matsumoto Y, Kishi S,

Yokohira M, Imaida K, Saoo K.

Author information:
(1)Dept. of Surgery, Asada General Hospital.

CASE: An 82-year-old man died because of squamous cell carcinoma of the right lung with metastasis to the left femoral bone. At the age of 75 years, he was admitted to our hospital because of hematemesis. Widespread type 3 gastric cancer was detected in the lesser curvature. Computed tomography(CT)showed multiple liver metastases. Preoperative chemotherapy with TS-1/cisplatin(CDDP)was administered. TS-1 was orally administered at 80mg/body/day and CDDP was administered by intravenous infusion at 20mg/body/day every week for 3 weeks and this was followed by a drug-free 2-week period as the first course. After the fourth course, gastrectomy was performed for the primary lesion and radiofrequency ablation(RFA)was performed for the liver metastases. The patient survived for more than 7 years with a complete response (CR)and died thereafter because of squamous cell carcinoma of the lung.

PMID: 25132035 [PubMed - indexed for MEDLINE]

13. J Clin Oncol. 2014 Sep 20;32(27):2983-90.

Tumor stage after neoadjuvant chemotherapy determines survival after surgery for adenocarcinoma of the esophagus and esophagogastric junction.

Davies AR, Gossage JA, Zylstra J, Mattsson F, Lagergren J, Maisey N, Smyth EC, Cunningham D, Allum WH, Mason RC.

PURPOSE: Neoadjuvant chemotherapy is established in the management of most resectable esophageal and esophagogastric junction adenocarcinomas. However, assessing the downstaging effects of chemotherapy and predicting response to treatment remain challenging, and the relative importance of tumor stage before and after chemotherapy is debatable.
METHODS: We analyzed consecutive resections for esophageal or esophagogastric junction adenocarcinomas performed at two high-volume cancer centers in London between 2000 and 2010. After standard investigations and multidisciplinary team consensus, all patients were allocated a clinical tumor stage before treatment, which was compared with pathologic stage after surgical resection. Survival

analysis was conducted using Kaplan-Meier analysis and Cox regression analysis.
RESULTS: Among 584 included patients, 400 patients (68%) received neoadjuvant chemotherapy. Patients with downstaged tumors after neoadjuvant chemotherapy experienced improved survival compared with patients without response ($P < .001$), and such downstaging (hazard ratio, 0.43; 95% CI, 0.31 to 0.59) was the strongest independent predictor of survival after adjusting for patient age, tumor grade, clinical tumor stage, lymphovascular invasion, resection margin status, and surgical resection type. Patients downstaged by chemotherapy, compared with patients with no response, experienced lower rates of local recurrence (6% v. 13%, respectively; $P = .030$) and systemic recurrence (19% v. 29%, respectively; $P = .027$) and improved Mandard tumor regression scores ($P = .001$). Survival was strongly dictated by stage after neoadjuvant chemotherapy, rather than clinical stage at presentation.
CONCLUSION: The stage of esophageal or esophagogastric junction adenocarcinoma after neoadjuvant chemotherapy determines prognosis rather than the clinical stage before neoadjuvant chemotherapy, indicating the importance of focusing on postchemotherapy staging to more accurately predict outcome and eligibility for surgery. Patients who are downstaged by neoadjuvant chemotherapy benefit from reduced rates of local and systemic recurrence.

PMID: 25071104 [PubMed - indexed for MEDLINE]

14. Curr Oncol. 2014 Jun;21(3):125-33. doi: 10.3747/co.21.1570.

Effect of preoperative chemoradiotherapy on outcome of patients with locally advanced esophagogastric junction adenocarcinoma-a pilot study.

Orditura M(1), Galizia G(2), Di Martino N(2), Ancona E(3), Castoro C(3), Pacelli R(4), Morgillo F(1), Rossetti S(1), Gambardella V(1), Farella A(4), Laterza MM(1), Ruol A(3), Fabozzi A(1), Napolitano V(2), Iovino F(2), Lieto E(2), Fei L(2), Conzo G(2), Ciardiello F(1), De Vita F(1).

Author information:
(1)Division of Medical Oncology, Department of Clinical and Experimental Medicine and Surgery, Second University of Naples School of Medicine, Naples, Italy.
(2)Divisions of Surgical Oncology, Department of Anesthesiologic, Surgical, and Emergency Sciences, Second University of Naples School of Medicine, Naples, Italy. (3)Division of General Surgery 1, University of Padua, School of Medicine,

Padua, Italy. (4)Division of Radiotherapy, Federico ii University of Naples School of Medicine, Naples, Italy.

BACKGROUND: To date, few studies of preoperative chemotherapy or chemoradiotherapy (crt) in gastroesophageal junction (gej) cancer have been statistically powered; indeed, gej tumours have thus far been grouped with esophageal or gastric cancer in phase iii trials, thereby generating conflicting results.
METHODS: We studied 41 patients affected by locally advanced Siewert type i and ii gej adenocarcinoma who were treated with a neoadjuvant crt regimen [folfox4 (leucovorin-5-fluorouracil-oxaliplatin) for 4 cycles, and concurrent computed tomography-based three-dimensional conformal radiotherapy delivered using 5 daily fractions of 1.8 Gy per week for a total dose of 45 Gy], followed by surgery. Completeness of tumour resection (performed approximately 6 weeks after completion of crt), clinical and pathologic response rates, and safety and outcome of the treatment were the main endpoints of the study.
RESULTS: All 41 patients completed preoperative treatment. Combined therapy was well tolerated, with no treatment-related deaths. Dose reduction was necessary in 8 patients (19.5%). After crt, 78% of the patients showed a partial clinical response, 17% were stable, and 5% experienced disease progression. Pathology examination of surgical specimens demonstrated a 10% complete response rate. The median and mean survival times were 26 and 36 months respectively (95% confidence interval: 14 to 37 months and 30 to 41 months respectively). On multivariate analysis, TNM staging and clinical response were demonstrated to be the only independent variables related to long-term survival.
CONCLUSIONS: In our experience, preoperative chemoradiotherapy with folfox4 is feasible in locally advanced gej adenocarcinoma, but shows mild efficacy, as suggested by the low rate of pathologic complete response.

PMCID: PMC4059797
PMID: 24940093 [PubMed]

15. Gan To Kagaku Ryoho. 2014 May;41(5):641-3.

[A case of advanced gastric cancer with para-aortic lymph node metastases responding to S-1/CDDP chemotherapy that leads to a pathological complete response].

[Article in Japanese]

Nakano A(1), Endo M, Hirayama N, Akai T, Matsubara H.

Author information:
(1)Dept. of Surgery, Kumagaya General Hospital.

A 66-year-old man complaining of epigastralgia was referred to our hospital. We examined the patient and diagnosed advanced gastric cancer (ML, type 3, por, cT3, cN3, cH0, cP0, cM1[LYM], cStage IV). A poor outcome was predicted, so we attempted induction chemotherapy and expected tumor downstaging. We chose S-1/CDDP therapy. S-1 was administered orally for 21 days, followed by CDDP div on day 8. Total gastrectomy and lymph node dissection (D2+No. 12a, No. 13,16) was performed using Roux-en-Y reconstruction. Histological examination of the resected stomach and lymph nodes revealed no residual cancer cells, suggesting complete histological remission (grade 3) according to the Japanese classification of gastric carcinoma. The patient has been in good health without recurrence for 12 months after surgery.

PMID: 24917013 [PubMed - indexed for MEDLINE]

16. World J Surg Oncol. 2014 May 19;12:150. doi: 10.1186/1477-7819-12-150.

Histological complete response to a combined docetaxel/cisplatin/fluorouracil neoadjuvant chemotherapy for T4 stage gastric adenocarcinoma.

Guo Mg(1), Zheng Q, zhong Di J, Yang Z.

Author information:
(1)Department of Surgery, the Six People's Hospital of Shanghai, Shanghai Jiaotong University, Shanghai 200233, China. guominggao203@hotmail.com.

Local advanced gastric carcinoma has a very poor prognosis. When a T4 gastric carcinoma has invaded the surrounding tissues and organs, curative resection is unlikely. We present here a case of a 63-year-old woman with a T4 unresectable gastric adenocarcinoma. She underwent two 3-week cycles of docetaxel/cisplatin/fluorouracil chemotherapy, followed by radical gastric resection. Each cycle consisted of 75 mg/m2 docetaxel and 75 mg/m2 cisplatin on

day 1, and 200 mg/m2 leucovorin and 500 mg/m2 fluorouracil on days 1 through 5. The patient exhibited a complete histologic response. Our results indicate that docetaxel/cisplatin/fluorouracil neoadjuvant chemotherapy is a promising method of treatment for advanced gastric cancer.

PMCID: PMC4032865
PMID: 24885421 [PubMed - indexed for MEDLINE]

17. Radiother Oncol. 2014 Aug;112(2):284-8. doi: 10.1016/j.radonc.2014.05.003. Epub
2014 May 22.

Preoperative chemoradiotherapy in locally advanced gastric cancer, a phase I/II feasibility and efficacy study.

Trip AK(1), Poppema BJ(2), van Berge Henegouwen MI(3), Siemerink E(4), Beukema JC(5), Verheij M(1), Plukker JT(6), Richel DJ(7), Hulshof MC(8), van Sandick JW(9), Cats A(10), Jansen EP(1), Hospers GA(11).

Author information:
(1)Department of Radiation Oncology, Netherlands Cancer Institute - Antoni van Leeuwenhoek Hospital, Amsterdam, The Netherlands. (2)Department of Medical Oncology, University Medical Centre Groningen, The Netherlands. (3)Department of Surgical Oncology, Academic Medical Centre - University of Amsterdam, The Netherlands. (4)Department of Internal Medicine, Ziekenhuisgroep Twente, Hengelo, The Netherlands. (5)Department of Radiation Oncology, University Medical Centre Groningen, The Netherlands. (6)Department of Surgical Oncology, University Medical Centre Groningen, The Netherlands. (7)Department of Medical Oncology, Academic Medical Centre - University of Amsterdam, The Netherlands. (8)Department of Radiation Oncology, Academic Medical Centre - University of Amsterdam, The Netherlands. (9)Department of Surgical Oncology, Netherlands Cancer Institute - Antoni van Leeuwenhoek Hospital, Amsterdam, The Netherlands. (10)Department of Gastroenterology and Hepatology, Netherlands Cancer Institute - Antoni van Leeuwenhoek Hospital, Amsterdam, The Netherlands. (11)Department of Medical Oncology, University Medical Centre Groningen, The Netherlands. Electronic address: g.a.p.hospers@umcg.nl.

OBJECTIVES: This study was initiated to investigate the feasibility and efficacy

of preoperative radiotherapy with weekly paclitaxel and carboplatin in locally advanced gastric cancer.
METHODS: In a prospective study, patients with locally advanced gastric cancer stage IB-IV(M0) were treated with chemoradiotherapy followed by surgery 4-6 weeks after the last irradiation. Chemoradiotherapy consisted of radiation to a total dose of 45 Gy given in 25 fractions of 1.8 Gy, combined with concurrent weekly carboplatin and paclitaxel.
RESULTS: Between December 2007 and January 2012, 25 patients with cT3 (64%) or cT4 (36%) gastric cancer were included. One patient discontinued concurrent chemotherapy in the 4th week due to toxicity, but completed radiotherapy. Another patient discontinued chemoradiotherapy after the 3rd week due to progressive disease. Grade III adverse events of chemoradiotherapy were: gastrointestinal 12%, haematological 12% and other 8%. All patients, except one who developed progressive disease, were operated. Surgical complications were: general/infectious 48%, anastomotic leakage 12%, and bowel perforation 8%. Postoperative mortality was 4%. Microscopically radical resection rate was 72%. Pathological complete response rate was 16% and near complete response rate 24%.
CONCLUSIONS: In this study, preoperative chemoradiotherapy for patients with locally advanced gastric cancer was associated with manageable toxicity and encouraging pathological response rates.

Copyright © 2014 Elsevier Ireland Ltd. All rights reserved.

PMID: 24856116 [PubMed - in process]

18. Cancer Chemother Pharmacol. 2014 Jul;74(1):141-50. doi:
10.1007/s00280-014-2482-0. Epub 2014 May 14.

Phase II multicentre study of efficacy and feasibility of dose-intensified preoperative weekly cisplatin, epirubicin, and paclitaxel (PET) in resectable gastroesophageal cancer.

Jary M(1), Ghiringhelli F, Jacquin M, Fein F, Nguyen T, Cleau D, Nerich V, El Gani M, Mathieu P, Valmary-Degano S, Arnould L, Lassabe C, Lamfichekh N, Fratté S, Paget-Bailly S, Bonnetain F, Borg C, Kim S.

Author information:
(1)Department of Medical Oncology, University Hospital of Besançon, Besançon,

France.

BACKGROUND: Perioperative chemotherapy improves the overall survival of resectable gastroesophageal adenocarcinoma (GEA) patients. However, more than 40 % of the patients are not healthy enough to complete their post-operative chemotherapy, and the progression-free survival rate is lower than 35 % at 5 years. In order to optimise neoadjuvant chemotherapy regimen, a pilot study of weekly dose-intensified cisplatin, epirubicin, and paclitaxel (PET) was conducted. The primary objective was a complete resection (R0) rate. Then, a R0 rate $\leq$80 % was considered as uninteresting, with an expected R0 rate of 92 %. Secondary objectives were the feasibility, safety, histological response rate (Becker score), and survival (Trial registration: NCT01830270).
METHODS: Patients with >T1N0M0 GEA were included. Treatment consisted of eight preoperative cycles of weekly PET regimen at 30/50/80 mg/m^2 of cisplatin, epirubicin, and paclitaxel, respectively. Primary prophylaxis by granulocyte colony-stimulating factor was administered. Surgery was performed 4-6 weeks following the last cycle of chemotherapy. Using Fleming two-step design with a unilateral alpha type one error of 5 % and a statistical power of 80 %, it would be required to include 68 patients. At planned interim analysis for futility, it was required to observe at least 25 of 29 patients with R0 resection to pursue inclusion. At the second step, it was required to observe at least 61 of 68 patients with R0 resection to conclude for promising activity of the dose-intensified chemotherapy.
RESULTS: Between May 2011 and January 2013, 29 patients were enrolled. Median age was 62 years (range 39-83 years), and seven (24 %) patients presented signet-ring cell histology. Twenty-seven (93 %) patients underwent surgery. Pathological complete responses (Becker score 1a) were observed in four patients, and nearly complete responses (Becker score 1b) for additional three patients. A R0 rate was achieved for 24 of 29 (82.7 %; 95 % CI 64-94 %) patients. No Becker score 1a/1b response was observed among patients with signet-ring cell GEA. Twenty-one (72 %) patients completed all eight cycles, and 86 % received seven or more cycles. Sixteen (56 %) patients experienced grade 3-4 neutropenia, and five patients had febrile neutropenia. Among non-haematological toxicities, mucositis and fatigue were the most frequent ones. The median-delivered relative dose intensity (DI) was 80 % for cisplatin, 75 % for epirubicin, and 79 % for paclitaxel. However, only 45 % of the patients received at least 80 % of the planned median DI for all three drugs.
CONCLUSIONS: Despite high R0 and pathological response rates, neoadjuvant PET chemotherapy did not meet the primary end-point and failed to show an acceptable

relative DI. PET chemotherapy is not recommended in resectable GEA patients.

PMID: 24824852 [PubMed - indexed for MEDLINE]

19. Cancer Invest. 2014 Jul;32(6):272-84. doi: 10.3109/07357907.2014.911877. Epub 2014 May 6.

An updated meta-analysis of randomized controlled trial assessing the effect of neoadjuvant chemotherapy in advanced gastric cancer.

Xiong BH(1), Cheng Y, Ma L, Zhang CQ.

Author information:
(1)1Department of General Surgery, the First Affiliated Hospital of Chongqing Medical University, Chongqing, P. R. China;

Patients with locally advanced gastric cancer (AGC) have a poor outcome. We performed an updated meta-analysis to assess the effect of neoadjuvant chemotherapy (NAC). By searching electronic databases (PubMed, Embase, Cochrane Library) and ASCO proceedings from 1990 to 2012, all randomized controlled trials (RCTs) which compared the effect of NAC combined surgery versus surgery alone in advanced gastric and gastroesophageal cancer would be included. All calculations and statistical tests were performed. Twelve RCTs with a total of 1,820 patients were included. All patients had resectable gastric or gastroesophageal cancer and received NAC. NAC can slightly improve the survival rate [OR = 1.32, 95% confidence interval (CI): 1.07-1.64, P = 0.01], little, or no significant benefits were suggested in subgroup analyses between different population and regimens either. It can significantly improved the 3-year progression-free survival (PFS) [OR: 1.85 (1.39, 2.46), p < .0001], tumor down-staging rate [OR: 1.71 (1.26, 2.33), p = .0006] and R0 resection rate [OR: 1.38 (1.08, 1.78) p = .01] of patients with AGC. There were no difference between the two arms, in terms of relapse rates [OR: 1.03 (0.60, 1.78), p = 0.92], operative complications [OR: 1.20 (0.90, 1.58), p = 0.21], perioperative mortality [OR: 1.14 (0.64, 2.05), p = 0.65], and grade 3/4 adverse effects. NAC can significantly down-stage the tumor and improve R0 resection rate of patients with gastric and gastroesophageal cancer. It is safe and feasible, and can be tolerated. NAC can slightly improve the survival rate. It needs further prospective multinational multicenter RCTs to define the clinical benefits of NAC and the most effective

strategies for gastric and gastroesophageal cancer.

PMID: 24800782 [PubMed - indexed for MEDLINE]

20. Cancer Chemother Pharmacol. 2014 Jun;73(6):1155-61. doi:
10.1007/s00280-014-2449-1. Epub 2014 Apr 21.

A phase II trial of Xeloda and oxaliplatin (XELOX) neo-adjuvant chemotherapy followed by surgery for advanced gastric cancer patients with para-aortic lymph node metastasis.

Wang Y(1), Yu YY, Li W, Feng Y, Hou J, Ji Y, Sun YH, Shen KT, Shen ZB, Qin XY, Liu TS.

Author information:
(1)Department of Medical Oncology, Fudan University Zhongshan Hospital, 180 Fenglin Road, Shanghai, 200032, People's Republic of China.

Comment in
    Cancer Chemother Pharmacol. 2014 Aug;74(2):435-6.
    Cancer Chemother Pharmacol. 2014 Aug;74(2):433-4.

PURPOSE: Gastric cancer with para-aortic lymph node (PAN) involvement is regarded as advanced disease, and only chemotherapy is recommended from the guidelines. In unresectable cases, neoadjuvant chemotherapy could prolong survival if conversion to resectability could be achieved.
METHODS: The study was a single-arm phase II trial. Patients who were diagnosed with gastric cancer and PAN involvement (Stations No. 16a2/16b1) were treated with capecitabine and oxaliplatin combination chemotherapy every 3 weeks for a maximum of six cycles. After every two cycles, abdominal computed tomographic scans were repeated to evaluate the response, and surgery was performed at the physician(')s discretion in patients with sufficient tumor response, followed by chemotherapy with the same regimen to complete a total of six cycles. The primary end point was the response rate of the preoperative chemotherapy. The secondary end points were R0 resection rate, progression-free survival (PFS), overall survival (OS), and adverse events.
RESULTS: A total of 48 patients were enrolled. The response rate of the first-line chemotherapy was 49.0 %, and the clinical benefit response was 85.1 %.

After a median of four cycles of chemotherapy, 28 patients received surgery (58.3 %). The median PFS and OS of all patients were 10.0 and 29.8 months, respectively. Patients in the surgery group had much longer PFS (18.1 vs. 5.6 mo, P = 0.001) and OS (not reached vs. 12.5 mo, P = 0.016) compared with those in the non-surgery group.
CONCLUSIONS: For gastric cancer patients with PAN involvement, neoadjuvant chemotherapy with XELOX demonstrated a good response rate, and a sufficient R0 resection rate, with acceptable toxicities. Further study is needed to confirm the effectiveness of this regimen.

PMCID: PMC4032640
PMID: 24748418 [PubMed - indexed for MEDLINE]

21. Gan To Kagaku Ryoho. 2014 Feb;41(2):245-8.

[A case of complete response(CR)to S-1 and paclitaxel(PTX)combination therapy in a patient with unresectable gastric cancer].

[Article in Japanese]

Hori Y(1), Itagaki R, Iwata H, Maekawa S.

Author information:
(1)Dept. of Surgery, Shinkatsushika Hospital.

We report a case of a patient with unresectable gastric cancer who showed complete response(CR)to S-1 and paclitaxel (PTX)combination therapy. The patient(a 67-year-old woman)was diagnosed with unresectable advanced gastric cancer with metastases in the Virchow's lymph nodes and para-aortic lymph nodes. Systemic chemotherapy with 70mg/m2 S-1 (days 1-14)and 70mg/m2 PTX(day 1)was administered every 3 weeks. At the end of 7 courses of chemotherapy, the primary lesion and swollen lymph nodes became markedly smaller. After 7 courses, an additional 39 courses were administered over 2.5 years. No notable adverse events were seen, and the patient's performance status(PS)was 0. CR was monitored by imaging studies. No cancer cells were detected on cytological examination of the primary lesion. Monotherapy with 70mg/m2 S-1(days 1-28, 2-week drug holiday)has been administered for the past 3 years. The patient is currently treated as an outpatient and maintains CR and a PS of 0.

PMID: 24743207 [PubMed - indexed for MEDLINE]

22. Zhonghua Yi Xue Za Zhi. 2014 Jan 14;94(2):127-30.

[Correlated analysis of 5 fluorouracil metabolic enzymes with tumor response after SOX regimen neoadjuvant chemotherapy in advanced gastric cancer].

[Article in Chinese]

Li T(1), Liang M, Yuan J, Guo X, Feng D, Li T, Teng D, Peng Z, Wu X, Li Z, Wang H, Chen L(2).

Author information:
(1)Department of General Surgery, Chinese PLA General Hospital, Beijing 100853, China. (2)Email: chenlinbj@vip.sina.com.

OBJECTIVE: To analyze the impact of mRNA expression of oral fluoropyrimidine (S-1) metabolism on treatment outcomes in locally advanced gastric cancer patients on preoperative S-1 oxaliplatin-based chemotherapy.
METHODS: Between June 2012 and March 2013, 32 patients with preoperative AJCC stage II-III gastric cancer patients were enrolled. They received S-1 (80 mg·m^{-2} × d^{-1}, days 1-14) and oxaliplatin (130 mg/m^2, day 1) every 3 weeks and subsequently underwent gastrectomy with D2 lymphadenectomy. Paired tumor and normal fresh frozen tissues were collected to evaluate the mRNA levels of thymidylate synthase (TS), thymidine phosphorylase (TP), dihydropyrimidine dehydrogenase (DPD) and OPRT with quantitative reverse transcription(RT) -PCR.
RESULTS: Among them, 21 (65.6%) patients had clinical tumor response and histological response occurred in 10 (31.3%) patients. Quantitative RT-PCR results showed that OPRT mRNA expression was significantly higher in clinical tumor responders than non-responders (3.95 ± 0.81 vs 1.79 ± 0.64, P = 0.005). Diffuse-type gastric cancer patients (n = 22) demonstrated higher OPRT expression levels than intestinal-type(n = 10) ones (2.54 ± 0.75 vs 1.49 ± 0.56, P = 0.014). The mRNA expressions of TS and TP in gastric cancer tissues with lymph node (LN) metastasis (n = 13) were significantly higher than those in gastric cancer tissues without LN metastasis (n = 19, both P < 0.05) .Similar results were not found for comparing dihydropyrimidine dehydrogenase expression levels (all P >

0.05).
CONCLUSION: OPRT, TS and TP may become potential predictive biomarkers in advanced gastric cancer patients on oral fluoropyrimidine (S-1)-based chemotherapy.

PMID: 24721353 [PubMed - indexed for MEDLINE]

23. Anticancer Res. 2014 Apr;34(4):1483-91.

Pertuzumab: development beyond breast cancer.

Barthélémy P(1), Leblanc J, Goldbarg V, Wendling F, Kurtz JE.

Author information:
(1)Department of Oncology and Hematology, Hôpitaux Universitaires de Strasbourg, 1 Place de l'hôpital, 67091 Strasbourg, France.
j-emmanuel.kurtz@chru-strasbourg.fr.

Pertuzumab (Perjeta®) represents the first monoclonal antibody in a new class of agents known as dimerization inhibitors. Pertuzumab was recently approved for the treatment of Human Epidermal Receptor 2 (HER2)-positive breast cancer in the metastatic and neo-adjuvant setting. This approval for first-line therapy for metastatic breast cancer was based on the results of a large randomized multicenter phase III trial showing a significant improvement in overall survival when pertuzumab was combined with trastuzumab and docetaxel in HER2-positive metastatic breast cancer. In the neoadjuvant setting, dual HER2 blockade by trastuzumab and pertuzumab improved the complete pathological response rate. However, pertuzumab development was not confined to breast cancer and in the present article, we focus on pertuzumab data for solid tumors other than breast cancer, and review the biological rationale for its use, the published pre-clinical and clinical evidence, as well ongoing trials.

PMID: 24692675 [PubMed - indexed for MEDLINE]

24. Br J Surg. 2014 May;101(6):653-60. doi: 10.1002/bjs.9484. Epub 2014 Mar 25.

Neoadjuvant chemotherapy with S-1 and cisplatin followed by D2 gastrectomy with

para-aortic lymph node dissection for gastric cancer with extensive lymph node metastasis.

Tsuburaya A(1), Mizusawa J, Tanaka Y, Fukushima N, Nashimoto A, Sasako M; Stomach Cancer Study Group of the Japan Clinical Oncology Group.

Collaborators: Sano T, Iwasaki Y, Katayama H, Nakamura K, Kaba H, Katsuki H, Fukuda H.

Author information:
(1)Shonan Kamakura General Hospital, Kamakura, Tokyo, Japan.

BACKGROUND: Locally advanced gastric cancer with extensive regional and/or para-aortic lymph node (PAN) metastases is typically unresectable and associated with poor outcomes. This study investigated the safety and efficacy of S-1 plus cisplatin followed by extended surgery with PAN dissection for gastric cancer with extensive lymph node metastasis.
METHODS: Patients with gastric cancer with bulky lymph node metastasis along the coeliac artery and its branches and/or PAN metastasis received two or three 28-day cycles of S-1 plus cisplatin, followed by gastrectomy with D2 plus PAN dissection. The primary endpoint was the percentage of complete resections with clear margins in the primary tumour (R0 resection). A target sample size of 50 with one-sided α of 0.105 and β of approximately 0.2 corresponded to an expected R0 rate of 65 per cent and a threshold of 50 per cent.
RESULTS: Between February 2005 and June 2007, 53 patients were enrolled, of whom 51 were eligible. The R0 resection rate was 82 per cent. Clinical and pathological response rates were 65 and 51 per cent respectively. The 3- and 5-year overall survival rates were 59 and 53 per cent respectively. During chemotherapy, grade 3/4 neutropenia occurred in 19 per cent and grade 3/4 non-haematological adverse events in 15.4 per cent. The incidence of grade 3/4 adverse events related to surgery was 12 per cent. There were no reoperations or treatment-related deaths.
CONCLUSION: For locally advanced gastric cancer with extensive lymph node metastasis, 4-weekly S-1 plus cisplatin followed by surgery including PAN dissection was safe and effective for some patients. Further investigation of this treatment strategy is warranted.

© 2014 BJS Society Ltd. Published by John Wiley & Sons Ltd.

PMID: 24668391 [PubMed - indexed for MEDLINE]

25. Target Oncol. 2014 Mar;9(1):85-94. doi: 10.1007/s11523-014-0313-1. Epub 2014 Mar
26.

Subcutaneous trastuzumab: a review of its use in HER2-positive breast cancer.

Sanford M(1).

Author information:
(1)Adis, Springer Healthcare, 41 Centorian Drive, Private Bag 65901, Mairangi Bay, North Shore, 0754, Auckland, New Zealand, demail@springer.com.

Trastuzumab (Herceptin®) is a humanized IgG1 monoclonal antibody that is an efficacious treatment for HER2-positive breast and gastric cancers. Subcutaneous trastuzumab is a new formulation approved in the European Union for use in patients with early or metastatic breast cancer. In the randomized, open-label, multinational HannaH (enHANced treatment with NeoAdjuvant Herceptin) study of neoadjuvant/adjuvant trastuzumab in patients with early HER2-positive breast cancer, the pharmacokinetics of neoadjuvant subcutaneous trastuzumab were similar to those after intravenous administration, meeting the noninferiority criterion for mean predose trough concentrations, as assessed prior to surgery (primary pharmacokinetic endpoint). Trastuzumab blood concentrations throughout the dosing interval remained above those considered necessary for anticancer activity. In this study, the pathologic complete response rates (primary efficacy endpoint) were 45.4 and 40.7 % in the subcutaneous and intravenous administration groups, respectively, meeting a study noninferiority criterion. In the randomized, open-label, crossover, multinational PrefHer study of neoadjuvant/adjuvant or adjuvant trastuzumab in early HER2-positive breast cancer, subcutaneous administration of trastuzumab was preferred over intravenous administration by >85 % of patients, most commonly because it was time saving and induced less pain and discomfort. In the HannaH study, the tolerability profile of subcutaneous trastuzumab was similar to that of intravenous trastuzumab, except that the rate of serious adverse events was 21 % (vs. 12 % with intravenous administration), partly because of more infections with subcutaneous administration. Whether this finding is of any clinical significance should emerge from ongoing studies. On the evidence, subcutaneous trastuzumab is an effective and generally

well-tolerated treatment option that is preferred by patients over intravenous administration.

PMID: 24664187 [PubMed - indexed for MEDLINE]

26. PLoS One. 2014 Mar 12;9(3):e90526. doi: 10.1371/journal.pone.0090526. eCollection
2014.

Increased microRNA-630 expression in gastric cancer is associated with poor overall survival.

Chu D(1), Zhao Z(2), Li Y(3), Li J(4), Zheng J(4), Wang W(4), Zhao Q(4), Ji G(4).

Author information:
(1)State Key Laboratory of Cancer Biology and Xijing Hospital of Digestive Diseases, Xijing Hospital, Fourth Military Medical University, Xi'an, Shaanxi, China; State Key Laboratory of Cancer Biology and Department of Biochemistry and Molecular Biology, Fourth Military Medical University, Xi'an, Shaanxi, China. (2)Department of General Surgery, Tangdu Hospital, Fourth Military Medical University, Xi'an, China. (3)Department of Medical Affair, General Hospital of Chengdu Military Region, Chengdu, Sichuan, China. (4)State Key Laboratory of Cancer Biology and Xijing Hospital of Digestive Diseases, Xijing Hospital, Fourth Military Medical University, Xi'an, Shaanxi, China.

MicroRNAs are noncoding RNAs that regulate multiple cellular processes during cancer progression. Among various microRNAs, MiR-630 has recently been identified to be implicated in many critical processes in human malignancies. We aimed to investigate the significance and prognostic value of miR-630 in human gastric cancer. Gastric cancer and adjacent normal specimens from 236 patients from who had not received neoadjuvant chemotherapy were collected. The expression of miR-630 was investigated by quantitative real-time PCR assay and its association with overall survival of patients was analyzed by statistical analysis. MiR-630 expression level was significantly elevated in gastric cancer in comparison to adjacent normal specimens. It is also proved that miR-630 expression was to be associated with gastric cancer invasion, lymph node metastasis, distant metastasis and TNM stage. In addition, survival analysis proved that elevated miR-630 expression was associated with poor overall survival of patients.

Multivariate survival analysis also proved that miR-630 was an independent prognostic marker after adjusted for known prognostic factors. The present study proved the over-expression of miR-630 and its association with tumor progression in human gastric cancer. It also provided the first evidence that miR-630 expression was an independent prognostic factor for patients with gastric cancer, which might be a potential valuable biomarker for gastric cancer.

PMCID: PMC3951214
PMID: 24621930 [PubMed - in process]

27. Ann Surg Oncol. 2014 Jun;21 Suppl 3:S385-9. doi: 10.1245/s10434-014-3615-8. Epub 2014 Mar 5.

Accuracy of CT staging of locally advanced gastric cancer after neoadjuvant chemotherapy: cohort evaluation within a randomized phase II study.

Yoshikawa T(1), Tanabe K, Nishikawa K, Ito Y, Matsui T, Kimura Y, Hasegawa S, Aoyama T, Hayashi T, Morita S, Miyashita Y, Tsuburaya A, Sakamoto J.

Author information:
(1)Department of Gastrointestinal Surgery, Kanagawa Cancer Center, Yokohama, Japan, yoshikawat@kcch.jp.

BACKGROUND: Accuracy of the radiologic diagnosis of gastric cancer staging after neoadjuvant chemotherapy remains unclear.
METHODS: Patients enrolled in the COMPASS trial, a randomized phase II study comparing two and four courses of S-1 plus cisplatin and paclitaxel and cisplatin followed by gastrectomy, were examined. The radiologic stage was determined by using thin-slice computed tomography (CT) or multidetector low CT by following Habermann's method.
RESULTS: A total of 75 patients registered in the COMPASS study who underwent surgical resection were examined in this study. The radiologic T and pathologic T stages were not significantly correlated ($p = 0.221$). The radiologic accuracy and rates of underdiagnosis and overdiagnosis were 42.7, 10.7, and 46.7%, respectively. When patients were stratified according to the pathologic response of the primary tumor, the correlation was not significant in either the responders ($n = 32$, $p = 0.410$) or the nonresponders ($n = 43$, $p = 0.742$). The radiologic accuracy was 37.5% in the responders and 42.7% in the nonresponders.

The radiologic N and pathologic N stages were significantly correlated ($p = 0.000$). The radiologic accuracy and rates of underdiagnosis and overdiagnosis were 44, 29.3, and 26.7%, respectively. When stratifying the patients with measurable lymph nodes according only to the radiologic response, the correlation was significant in the nonresponders ($n = 23$, $p = 0.035$) but not in the responders ($n = 28$, $p = 0.634$). The radiologic accuracy was 39.3% in the responders and 52.1% in the nonresponders.
CONCLUSIONS: Restaging using CT after neoadjuvant chemotherapy for gastric cancer is considered to be inaccurate and unreliable. In particular, the radiologic T-staging determined after neoadjuvant chemotherapy should not be considered in clinical decision-making.

PMID: 24595801 [PubMed - indexed for MEDLINE]

28. Ann Oncol. 2014 May;25(5):1039-44. doi: 10.1093/annonc/mdu091. Epub 2014 Feb 20.

Phase II study of neoadjuvant therapy with docetaxel, cisplatin, panitumumab, and radiation therapy followed by surgery in patients with locally advanced adenocarcinoma of the distal esophagus (ACOSOG Z4051).

Lockhart AC(1), Reed CE, Decker PA, Meyers BF, Ferguson MK, Oeltjen AR, Putnam JB, Cassivi SD, Montero AJ, Schefter TE; American College of Surgeons Oncology Group.

Author information:
(1)Division of Oncology, Department of Medicine, Washington University School of Medicine, St Louis.

BACKGROUND: Preoperative chemoradiotherapy (CRT) improves outcomes in patients with locally advanced but resectable adenocarcinoma of the esophagus. ACOSOG Z4051 evaluated CRT with docetaxel, cisplatin, and panitumumab (DCP) in this patient group with a primary end point of a pathologic complete response (pCR) ≥35%.
PATIENTS AND METHODS: From 15 January 2009 to 22 July 2011, 70 patients with locally advanced but resectable distal esophageal adenocarcinoma were enrolled. Patients received docetaxel (40 mg/m(2)), cisplatin (40 mg/m(2)), and panitumumab (6 mg/kg) on weeks 1, 3, 5, 7, and 9 with RT (5040 cGy, 180 cGy/day × 28 days)

beginning week 5. Resection was planned after completing CRT. PCR was defined as no viable residual tumor cells. Secondary objectives included near-pCR (≤10% viable cancer cells), toxicity, and overall and disease-free survival. Adverse events were graded using the CTCAE Version 3.0.
RESULTS: Five of 70 patients were ineligible. Of 65 eligible patients (59 M; median age 61), 11 did not undergo surgery, leaving 54 assessable. PCR rate was 33.3% and near-pCR was 20.4%. Secenty-three percent of patients completed DCP (n = 70) and 92% completed RT. 48.5% had toxicity ≥grade 4. Lymphopenia (43%) was most common. Operative mortality was 3.7%. Adult respiratory distress syndrome was encountered in two patients (3.7%). At median follow-up of 26.3 months, median overall survival was 19.4 months and 3-year overall survival was 38.6% (95% confidence interval 24.5% to 60.8%).
CONCLUSIONS: Neoadjuvant CRT with DCP is active (pCR + near-pCR = 53.7%) but toxicity is significant. Further evaluation of this regimen in an unselected population is not recommended.
CLINICALTRIALSGOV IDENTIFIER: NCT00757172.

PMID: 24562448 [PubMed - indexed for MEDLINE]

29. PLoS One. 2014 Jan 30;9(1):e86941. doi: 10.1371/journal.pone.0086941. eCollection
2014.

Neoadjuvant chemotherapy followed by surgery versus surgery alone for gastric carcinoma: systematic review and meta-analysis of randomized controlled trials.

Xu AM(1), Huang L(1), Liu W(2), Gao S(3), Han WX(1), Wei ZJ(1).

Author information:
(1)Anhui Medical University, Hefei, China ; Department of General Surgery, the First Affiliated Hospital of Anhui Medical University, Hefei, China. (2)Guangdong Provincial Key Laboratory of Liver Disease Research, The Third Affiliated Hospital of Sun Yat-sen University, Guangzhou, China. (3)Anhui Medical University, Hefei, China ; Department of Medical Oncology, the First Affiliated Hospital of Anhui Medical University, Hefei, China.

BACKGROUND: The effect of neoadjuvant chemotherapy (NAC) on Gastric carcinoma (GC) has been extensively studied, while its survival and surgical benefits

remain controversial. This study aims to perform a meta-analysis of high-quality randomized controlled trials (RCTs), comparing efficacy, safety and other outcomes of NAC followed by surgery with surgery alone (SA) for GC.
METHODS: We systematically searched databases of MEDLINE, EMBASE, The Cochrane Library and Springer for RCTs comparing NAC with SA when treating GC. Reference lists of relevant articles and reviews, conference proceedings and ongoing trial databases were also searched. Primary outcomes were 3-year and 5-year survival rates, survival time, and total and perioperative mortalities. Secondary outcomes included down-staging effects, R0 resection rate, and postoperative complications. Meta-analysis was conducted where possible comparing items using relative risks (RRs) and weighted mean differences (WMDs) according to type of data. NAC-related objective response, safety and toxicity were also specifically analyzed.
RESULTS: A total of 9 RCTs comparing NAC (n = 511) with SA (n = 545) published from 1995 to 2010 were identified. SA tended to be accompanied with higher overall mortality rate than NAC (46.03% vs 40.61%, RR: 0.83, 95% CI: 0.65-1.06, P = 0.14). Significantly, higher incidence of cases without regional lymph node metastasis observed upon resection were achieved among patients receiving NAC than those undergoing SA (25.68% vs 16.95%, RR: 1.92, 95% CI: 1.20-3.06, P = 0.006). All other parameters were comparable. Of the evaluable patients, 43.0% demonstrated either complete or partial response. The comprehensive NAC-related side-effect rate was 18.2% among patients available for safety assessment.
CONCLUSIONS: NAC contributes to lowering nodal stages, and potentially reduces overall mortality. Response rate may be an important influential factor impacting advantages, with chemotherapy-related adverse effects as a drawback. This level 1a evidence doesn't support NAC to outweigh SA in terms of survival and surgical benefits when dealing with GC.

PMCID: PMC3907439
PMID: 24497999 [PubMed - indexed for MEDLINE]

30. Aliment Pharmacol Ther. 2014 Mar;39(6):619-28. doi: 10.1111/apt.12635. Epub 2014
Jan 27.

Rituximab, alkylating agents or combination therapy for gastric mucosa-associated lymphoid tissue lymphoma: a monocentric non-randomised observational study.

Amiot A(1), Lévy M, Copie-Bergman C, Dupuis J, Szablewski V, Le Baleur Y, Baia M, Belhadj K, Sobhani I, Leroy K, Haioun C, Delchier JC.

Author information:
(1)Department of Gastroenterology, Albert Chenevier-Henri Mondor Hospital, Créteil, France; Department of Pathology, Albert Chenevier-Henri Mondor Hospital, Créteil, France; Lymphoid Malignancies Unit, Albert Chenevier-Henri Mondor Hospital, Créteil, France.

BACKGROUND: There is no consensus on the standard treatment of gastric mucosa-associated lymphoid tissue (MALT) lymphoma for Helicobacter pylori-negative patients and for patients with persistent disease despite H. pylori eradication.
AIM: To evaluate the comparative efficacy and safety of alkylating agents and rituximab alone or in combination.
METHODS: In this monocentric retrospective study, which included 106 patients who had not been previously treated with anti-cancer agents, we evaluated the efficacy and safety of oral alkylating agents monotherapy (n = 48), rituximab monotherapy (n = 28) and the therapy combining both drugs (n = 30). Evaluations were performed at weeks 6 (W6), 25 (W25), and 52 (W52) and after 2 years (W104).
RESULTS: After a median follow-up period of 4.9 years (range 0.4-17.2 years), complete remission and overall response were significantly higher in patients in the combination therapy group at W104 (92% and 100% respectively) compared with patients treated with alkylating agents alone (66% and 68%) and rituximab alone (64% and 73%). The 5-year progression-free survival probabilities were 68%, 70% and 89% in patients treated with alkylating agents alone, rituximab alone and combination therapy respectively. Haematological adverse events were reported in 32 (30%) patients (mostly grade 1) and were more frequent in the two groups receiving alkylating agents ($P = 0.05$ and $P < 0.001$). No toxicity-related death was reported.
CONCLUSIONS: The use of anti-cancer systemic therapy is safe and efficient in gastric MALT lymphoma. In this retrospective study, the combination of rituximab plus chlorambucil seems more efficient than rituximab or alkylating agents alone. Rituximab has a better safety profile than regimens containing alkylating agents.

© 2014 John Wiley & Sons Ltd.

PMID: 24467480 [PubMed - indexed for MEDLINE]

31. BMC Surg. 2014 Jan 24;14:5. doi: 10.1186/1471-2482-14-5.

Impact of neoadjuvant chemotherapy with PELF-protocoll versus surgery alone in the treatment of advanced gastric carcinoma.

Ruf C, Thomusch O, Goos M, Makowiec F, Illerhaus G, Ruf G(1).

Author information:
(1)Department of Surgery, University of Freiburg, Universitätsklinikum, Hugstetterstr, 55, D-79106 Freiburg, Germany. guenther.ruf@uniklinik-freiburg.de.

BACKGROUND: In a retrospective study we analyzed the impact of neoadjuvant chemotherapy (CTx) with the PELF - protocol (Cisplatin, Epirubicin, Leukovorin, 5-Fluoruracil) on mortality, recurrence and prognosis of patients with advanced gastric carcinoma, UICC stages Ib-III.
METHODS: 64 patients were included. 26 patients received neoadjuvant CTx followed by surgical resection, 38 received surgical resection only. Tumor staging was performed by endoscopy, endosonography, computed tomography and laparoscopy. Patients staged Ib - III received two cycles of CTx according to the PELF-protocol. Adjuvant chemotherapy was not performed at all.
RESULTS: Complete (CR) or partial response (PR) was seen in 20 patients (77%), 19% showing CR and 58% PR. No benefit was observed in 6 patients (23%). Two of these 6 patients displayed tumor progression during CTx. Major toxicity was defined as grade 3 to 4 neutropenia or gastrointestinal side effects. One patient died under CTx because of neutropenia and was excluded from the overall patient collective. The curative resection rate was 77% after CTx and 74% after surgery only. The perioperative morbidity rate after CTx was 39% versus 66% after resection only. Recurrence rate after CTx was 38% and 61% after surgery alone; we detected an effective reduction of locoregional recurrence (12% vs. 26%). The overall survival was 38% after CTx and 42% after resection only. The 5-year survival rates were 45% in responders, 20% in non - responders and 42% in only resected patients. A subgroup analysis indicates that responders with stage III tumors may benefit with respect to their 5-year survival in comparable patients without neoadjuvant CTx. As to be expected, non-responders with stage III tumors did not benefit with respect to their survival. The 5-year-survival was approximated using a Kaplan-Meier curve and compared using a log-rank test.
CONCLUSION: In patients with advanced gastric carcinoma, neoadjuvant CTx with the

PELF- protocol significantly reduces the recurrence rate, especially locoregionally, compared to surgery alone. In our study, there was no overall survival benefit after a 5-year follow-up period. Alone a subgroup of patients with stage III tumors appear to benefit significantly in the long term from neoadjuvant CTx.

PMCID: PMC3909936
PMID: 24461063 [PubMed - indexed for MEDLINE]

32. Z Gastroenterol. 2014 Jan;52(1):50-4. doi: 10.1055/s-0033-1356371. Epub 2014 Jan
13.

[Gastrointestinal stromal tumours bigger than 20 cm: experience with imatinib chemotherapy in neoadjuvant intention].

[Article in German]

Girotti P(1), Rolinger J(1), Kopp HG(2), Königsrainer A(1), Ladurner R(1).

Author information:
(1)Klinik für Allgemeine, Viszeral- und Transplantationschirurgie, Universitätsklinikum Tübingen. (2)Medizinische Klinik II, Hämatoonkologie, Universitätsklinikum Tübingen.

The size of the primary tumour is considered the most important risk factor for the development of metastasis or local recurrence in case of gastrointestinal stromal tumour (GIST). Until now no prospective data are available in the literature about the role of neadjuvant therapy with Imatinib. Between 2009 and 2012 seven patients with a giant GIST > 20 cm underwent a neadjuvant treatment with Imatinib, a radical operation, followed by an adjuvant therapy. These patients were controlled with regard to peri- and postoperative morbidity and disease-free survival. Two patients were considered not resectable and one patient showed liver metastasis at the time of diagnosis. RECIST responses to the neoadjuvant Imatinib were: 2/7 patients with stable disease, 3/7 partial response, 2/7 partial response with down-staging (resectable disease). Because of the following tumour localisations (6 gastric and 1 rectal), six gastrectomies (one en-bloc with left pancreas) and one Holm operation were performed. The

patient with simultaneous liver metastasis developed a tumour progression during the follow-up but the others are still tumour free after 2 years. We detected a significant tumour volume regression due to the neadjuvant chemotherapy in cases of GIST > 20 cm (30 %). Our series showed good results for a neadjuvant therapy in cases of giant GIST with the achievement of 100 % R0 resection without a high morbidity rate (in the literature a tumor size > 10 cm and poor localisation is associated to a high risk of R1 - 2 and high morbidity). Peri- and postoperative morbidity are acceptable and the tumour free survival at 2 years is 85 %.

© Georg Thieme Verlag KG Stuttgart • New York.

PMID: 24420799 [PubMed - indexed for MEDLINE]

33. Ann Surg Oncol. 2014 May;21(5):1739-48. doi: 10.1245/s10434-013-3462-z. Epub 2014
Jan 14.

Is preoperative chemotherapy followed by surgery the appropriate treatment for signet ring cell containing adenocarcinomas of the esophagogastric junction and stomach?

Heger U(1), Blank S, Wiecha C, Langer R, Weichert W, Lordick F, Bruckner T, Dobritz M, Burian M, Springfeld C, Grenacher L, Siewert JR, Büchler M, Ott K.

Author information:
(1)Department of Surgery, Heidelberg University Hospital, University of Heidelberg, Heidelberg, Germany, ulrike.heger@med.uni-heidelberg.de.

BACKGROUND: Recent data suggest primary resection as the preferable approach in patients with signet ring cell gastric cancer (SRC). The aim of our retrospective exploratory study was to evaluate the influence of SRC on prognosis and response in esophagogastric adenocarcinoma treated with neoadjuvant chemotherapy.
METHODS: A total of 723 locally advanced esophagogastric adenocarcinomas (cT3/4 N any) documented in a prospective database from two academic centers were classified according to the WHO definition for SRC (more than 50 % SRC) and analyzed for their association with response and prognosis after neoadjuvant treatment.
RESULTS: A total of 235 tumors (32.5 %) contained SRC. Median survival of SRC was

26.3 compared with 46.6 months (p < 0.001) for non-SRC. SRC were significantly associated with female gender, gastric localization, advanced ypT and R1/2 categories, and lower risk of surgical complications and anastomotic leakage (each p < 0.001). Clinical (21.1 vs. 33.7 %, p = 0.001) and histopathological response (less than 10 % residual tumor: 16.3 vs. 28.9 %, p < 0.001) were significantly less frequent in SRC. Clinical response (p = 0.003) and complete histopathological response (pCR) (3.4 %) (p = 0.003) were associated with improved prognosis in SRC. Clinical response, surgical complications, ypTN categories, but not SRC were independent prognostic factors in forward Cox regression analysis in R0 resected patients. Risk of peritoneal carcinomatosis was increased (p < 0.001), while local (p = 0.015) and distant metastases (p = 0.02) were less frequent than in non-SRC.
CONCLUSIONS: Prognosis of SRC is unfavorable. Although response to neoadjuvant chemotherapy is rare in SRC, it is associated with improved outcome. Thus, chemotherapy might not generally be abandoned in SRC. A stratification based on SRC should be included in clinical trials.

PMID: 24419755 [PubMed - indexed for MEDLINE]

34. Anticancer Res. 2014 Jan;34(1):301-6.

Successful use of Trastuzumab with anthracycline-based chemotherapy followed by trastuzumab maintenance in patients with advanced HER2-positive gastric cancer.

Palacio S(1), Loaiza-Bonilla A, Kittaneh M, Kyriakopoulos C, Ochoa RE, Escobar M, Arango B, Restrepo MH, Merchan JR, Rocha Lima CM, Hosein PJ.

Author information:
(1)1475 NW 12th Avenue. Suite 3400, Miami, 33136, FL, U.S.A. phosein@med.miami.edu.

BACKGROUND: There is no standard chemotherapy regimen that is universally accepted for the treatment of advanced gastric cancer. Trastuzumab added to chemotherapy improves survival in patients with metastatic human epidermal growth factor receptor-2 (Her2/neu)-overexpressing gastric cancer. Data are lacking for the combination of trastuzumab with other chemotherapy regimens, apart from the cisplatin/fluorouracil backbone used in the pivotal TOGA trial.
PATIENTS AND METHODS: In this retrospective analysis, we included patients with

gastric cancer with HER2 overexpression who received trastuzumab in addition to their first-line chemotherapy, with or without trastuzumab maintenance therapy. The end-points were response and tolerance to treatment.
RESULTS: We identified seven patients who met the search criteria; six had metastatic disease and one had locally advanced unresectable disease. Four patients received epirubicin/oxaliplatin/capecitabine/trastuzumab, and the others had non-anthracycline-based chemotherapy with trastuzumab. All patients had radiological responses to treatment - one had a complete response and six had partial responses. Among the four patients who received anthracycline-based chemotherapy with trastuzumab, there was a transient decline in cardiac ejection fraction in three, but all resolved without sequelae. All patients received a period of chemotherapy induction followed by trastuzumab monotherapy for maintenance. The median progression-free survival was 14.6 months and median overall survival was 16.4 months.
CONCLUSION: Trastuzumab is an important agent for the treatment of HER2-overexpressing gastric cancer. We recorded an acceptable safety and efficacy profile in this small cohort treated with anthracycline-based chemotherapy with trastuzumab followed by trastuzumab maintenance.

PMID: 24403478 [PubMed - indexed for MEDLINE]

35. J Laparoendosc Adv Surg Tech A. 2014 Feb;24(2):89-94. doi:
10.1089/lap.2013.0444.
Epub 2014 Jan 8.

Outcomes of robotic-assisted transhiatal esophagectomy for esophageal cancer after neoadjuvant chemoradiation.

Coker AM(1), Barajas-Gamboa JS, Cheverie J, Jacobsen GR, Sandler BJ, Talamini MA, Bouvet M, Horgan S.

Author information:
(1)Department of Surgery, University of California San Diego , La Jolla, California.

BACKGROUND: We previously reported our experience performing robotic-assisted transhiatal esophagectomy (RATE) in patients with early-stage esophageal cancer who had had no preoperative treatment. The purpose of this report was to

determine if RATE could be performed safely with good outcomes for esophageal cancer in a more recent series of patients, the majority of whom were treated with neoadjuvant chemoradiation.
SUBJECTS AND METHODS: This was a retrospective review of patients with adenocarcinoma of the distal esophagus or gastroesophageal junction who underwent RATE between November 2006 and November 2012 at a single tertiary-care hospital. Main outcome measures included operative and oncologic parameters, morbidity, and mortality.
RESULTS: In total, 23 patients underwent RATE, consisting of 20 men and 3 women with a median age of 64 years (range, 40-81 years). The majority of patients (19/23 [83%]) underwent neoadjuvant chemoradiation, although 1 patient had preoperative chemotherapy only, and 3 patients went straight to surgery. Median operative time was 231 minutes (range, 179-319 minutes), and median estimated blood loss was 100 mL (range, 25-400 mL). There were no conversions to open surgery. Complications included seven strictures, two anastomotic leaks, and two pericardial/pleural effusions requiring drainage. One patient required pyloroplasty 3 months after esophagectomy. One patient died from pulmonary failure 21 days after surgery (30-day mortality rate of 4%). The median length of stay was 9 days (range, 7-37 days). Seven of the 19 patients who underwent preoperative chemoradiation had a complete response on final pathology. The mean lymph node yield was 15 (range, 5-29), and surgical margins were negative for cancer in 21 cases.
CONCLUSIONS: RATE can be performed safely with good oncologic outcomes following neoadjuvant chemoradiation in patients with esophageal cancer. This technique has become our choice of operation for most patients with esophageal cancer.

PMID: 24401141 [PubMed - indexed for MEDLINE]

36. Radiat Oncol. 2014 Jan 8;9:9. doi: 10.1186/1748-717X-9-9.

Phase I study of neoadjuvant chemoradiotherapy with S-1 plus biweekly cisplatin for advanced gastric cancer patients with lymph node metastasis: -KOGC04-.

Matsuda S, Takahashi T(1), Fukada J, Fukuda K, Kawakubo H, Saikawa Y, Kawaguchi O, Takeuchi H, Shigematsu N, Kitagawa Y.

Author information:
(1)Department of Surgery, Keio University School of Medicine, 35 Shinanomachi,

Shinjuku-ku, Tokyo 160-8582, Japan. surgeontsune@yahoo.co.jp.

Erratum in
Radiat Oncol. 2014;9:140.

BACKGROUND: In patients with highly advanced gastric cancer, the recurrence rate remains high and the prognosis disappointing. We previously reported a phase I study of a neoadjuvant chemoradiotherapy of S-1 plus weekly cisplatin. Although adequate safety and efficacy were reported, myelosuppression was frequently observed, leading to treatment delay in several cases. To decrease toxicity and improve efficacy, we planned a phase I study with a modified chemotherapy regimen with biweekly cisplatin.
METHODS: Patients with advanced gastric cancer and lymph node metastasis who were treated by our institution between 2011 and 2012 were eligible for inclusion. The initial chemoradiotherapy schedule consisted of 6 weeks of S-1 orally administered on days 1-15 with an escalating dose of cisplatin administered on days 1 and 15. The starting dose (level 1) of cisplatin was 15 mg/m(2), the second dose (level 2) was 20 mg/m(2), and the third dose (level 3) was 25 mg/m(2). Radiation of 40 Gy was administered in 20 fractions. After initial chemoradiotherapy, one cycle of combination chemotherapy with S-1 plus cisplatin was delivered. The second cycle was 42 days in duration and included S-1 administered on days 1-29 plus biweekly cisplatin administered on days 1, 15, and 29. After neoadjuvant treatment, a curative gastrectomy with extended (D2) lymph node dissection was planned.
RESULTS: Nine patients were enrolled. At level 3, one patient had dose-limiting grade 3 diarrhea. Another patient experienced grade 3 nausea and intended to discontinue the treatment. Overall, because 2 of 3 patients experienced dose-limiting toxicity at level 3, we confirmed level 3 (Cisplatin 25 mg/m(2)) as the maximum tolerated dose and level 2 (Cisplatin 20 mg/m(2)) as the recommended dose (RD). The response rate was 78%, and 8 patients underwent curative gastrectomy. Resected specimens showed a histological response in 6 patients (75%), including one with a pathological complete response.
CONCLUSIONS: In this phase I trial, RD of cisplatin was identified as 20 mg/m(2). Generally, S-1 plus biweekly cisplatin can be given safely with concurrent radiation. We have initiated a multicenter phase II trial to further confirm the efficacy and safety of this approach.
TRIAL REGISTRATION: UMIN000008941.

PMCID: PMC3904203

PMID: 24398302 [PubMed - indexed for MEDLINE]

37. World J Gastrointest Oncol. 2013 Dec 15;5(12):222-9. doi:
10.4251/wjgo.v5.i12.222.

Pneumo-CT assessing response to neoadjuvant therapy in esophageal cancer:
Imaging-pathological correlation.

Ulla M(1), Gentile E(1), Yeyati EL(1), Diez ML(1), Cavadas D(1), Garcia-Monaco RD(1), Ros PR(1).

Author information:
(1)Marina Ulla, Ernestina Gentile, Ezequiel Levy Yeyati, Maria L Diez, Demetrio Cavadas, Ricardo D Garcia-Monaco, Pablo R Ros, Department of Radiology, Hospital Italiano de Buenos Aires, Gascón 450, Argentina.

Pneumo-computed tomography (PnCT) is a technique primarily developed and used to study stenotic lesions of the esophagus, gastroesophageal junction and stomach for pre-surgical planning. It helps to define both upper and lower borders of neoplasms located in the aforementioned areas. It achieves maximum lumen distension with CO2 highlighting thickened areas of the esophageal wall, thus allowing an accurate quantification of their extents. Although there are other alternatives for distension (oral contrast agents, water and effervescent granules), they may be suboptimal. Patients with locally advanced esophageal cancer have a dismal prognosis despite surgical resection. Therefore, neoadjuvant treatment strategies using radiation therapy and chemotherapy were developed to improve survival. Neoadjuvant therapy improves esophageal tumor prognosis in a substantial proportion of patients, and the use of imaging techniques is mandatory to detect their response. PnCT combined with virtual endoscopy and multiplanar reconstruction enhances morphologic details in esophageal cancer, and thus would allow an improved assessment of response to neoadjuvant treatment. Therefore, more information could be provided to assess the efficacy of pre-surgical treatment. We describe the potential use of PnCT to assess the response to neoadjuvant therapy in esophageal cancer with an imaging pathologic correlation.

PMCID: PMC3868717
PMID: 24363830 [PubMed]

38. Ann Surg Oncol. 2014 Apr;21(4):1147-52. doi: 10.1245/s10434-013-3443-2. Epub 2013
Dec 20.

Outcome data of patients with peritoneal carcinomatosis from gastric origin treated by a strategy of bidirectional chemotherapy prior to cytoreductive surgery and hyperthermic intraperitoneal chemotherapy in a single specialized center in Japan.

Canbay E(1), Mizumoto A, Ichinose M, Ishibashi H, Sako S, Hirano M, Takao N, Yonemura Y.

Author information:
(1)NPO to Support Peritoneal Dissemination Treatment, Department of General Surgery, Tokushu-Kai Hospital, Kishiwada, Osaka, Japan, drecanbay@gmail.com.

BACKGROUND: Management of peritoneal disseminated gastric cancer (GC) remains a challenging problem. The purpose of our study was to evaluate the outcome of bidirectional induction chemotherapy [bidirectional intraperitoneal and systemic induction chemotherapy (BIPSC)] in patients with peritoneal carcinomatosis (PC) arising from GC who underwent cytoreductive surgery (CRS) and hyperthermic intraperitoneal chemotherapy (HIPEC).
PATIENTS AND METHODS: Overall, 194 patients with PC arising from GC were treated with BIPSC comprising intraperitoneal docetaxel at a dose of 20 mg/m(2) and cisplatin at a dose of 30 mg/m(2) followed by four cycles of oral S-1 at a dose of 60 mg/m(2). CRS and HIPEC were performed in responders to BIPSC.
RESULTS: Of these 194 patients, 152 (78.3 %) underwent CRS and HIPEC between January 2005 and December 2012. Treatment-related mortality was 3.9 %, and major complications occurred in 23.6 % of patients. The median survival rate was 15.8 months, with 1-, 2-, and 5-year survival rates of 66, 32 and 10.7 %, respectively, in the patients treated with combined treatment. Multivariate analysis identified pathologic response to BIPSC ($p = 0.001$), low tumor burden [peritoneal cancer index (PCI) ≤ 6] ($p = 0.001$), and completeness of CRS (CC-0, CC-1) ($p = 0.001$) as independent predictors for a better prognosis.
CONCLUSION: As a viable option, BIPSC with CRS and HIPEC for patients with PC arising from GC may be performed safely, with acceptable morbidity and mortality, in a specialized unit. Response to BIPSC, optimal CRS and limited peritoneal

dissemination seem to be essential to achieve the best outcomes in these patients.

PMID: 24356799 [PubMed - indexed for MEDLINE]

39. Br J Cancer. 2014 Jan 21;110(2):421-9. doi: 10.1038/bjc.2013.712. Epub 2013 Dec 3.

Death-associated protein-3, DAP-3, correlates with preoperative chemotherapy effectiveness and prognosis of gastric cancer patients following perioperative chemotherapy and radical gastrectomy.

Jia Y(1), Ye L(2), Ji K(2), Zhang L(3), Hargest R(2), Ji J(3), Jiang WG(2).

Author information:
(1)1] Cardiff University-Peking University School of Oncology Joint Institute, Cardiff University School of Medicine, Heath Park, Cardiff CF14 4XN, UK [2] Metastasis and Angiogenesis Research Group, Cardiff University School of Medicine, Heath Park, Cardiff CF14 4XN, UK [3] Key Laboratory of Carcinogenesis and Translational Research (Ministry of Education), Department of Gastrointestinal Surgery, Peking University Cancer Hospital and Institute, No. 52, Haidian District, Beijing 100142, China. (2)1] Cardiff University-Peking University School of Oncology Joint Institute, Cardiff University School of Medicine, Heath Park, Cardiff CF14 4XN, UK [2] Metastasis and Angiogenesis Research Group, Cardiff University School of Medicine, Heath Park, Cardiff CF14 4XN, UK. (3)1] Cardiff University-Peking University School of Oncology Joint Institute, Cardiff University School of Medicine, Heath Park, Cardiff CF14 4XN, UK [2] Key Laboratory of Carcinogenesis and Translational Research (Ministry of Education), Department of Gastrointestinal Surgery, Peking University Cancer Hospital and Institute, No. 52, Haidian District, Beijing 100142, China.

BACKGROUND: DAP3 is a member of the death-associated protein (DAP) family and is characterised by proapoptotic function. It is involved in both exogenous and endogenous apoptotic pathways. In our previous studies, apoptotic level was found to be correlated with the effectiveness of preoperative chemotherapy. The effectiveness of preoperative chemotherapy was also associated with the overall effectiveness of the combined therapy and prognosis. The present study aimed to investigate the role of DAP3 in the evaluation of preoperative chemotherapy

effectiveness and its ability to predict prognosis in gastric cancer.
METHODS: Quantitative PCR and immunohistochemistry staining were performed in 87 patients who received combined therapy. Knockdown of DAP3 was conducted in gastric cancer cell lines to investigate its impact on cell growth, migration, adhesion and invasion. Tolerance to chemotherapy agents was determined by assessing apoptosis and caspase-3.
RESULTS: Higher DAP3 expression in gastric tumours was correlated with better prognosis. Knockdown of DAP3 expression promoted cell migration and enhanced resistance to chemotherapy by inhibiting apoptosis.
CONCLUSION: DAP3 is a potential molecular marker for response to preoperative chemotherapy and for predicting prognosis in gastric cancer patients treated with neoadjuvant chemotherapy and gastrectomy.

PMCID: PMC3899757
PMID: 24300973 [PubMed - indexed for MEDLINE]

40. Mol Oncol. 2014 Feb;8(1):142-9. doi: 10.1016/j.molonc.2013.10.007. Epub 2013 Oct
28.

ALDH-1 expression levels predict response or resistance to preoperative chemoradiation in resectable esophageal cancer patients.

Ajani JA(1), Wang X(2), Song S(3), Suzuki A(3), Taketa T(3), Sudo K(3), Wadhwa R(3), Hofstetter WL(4), Komaki R(5), Maru DM(6), Lee JH(7), Bhutani MS(7), Weston B(7), Baladandayuthapani V(2), Yao Y(3), Honjo S(3), Scott AW(3), Skinner HD(5), Johnson RL(8), Berry D(2).

Author information:
(1)Department of Gastrointestinal Medical Oncology, University of Texas M. D. Anderson Cancer Center, 1515 Holcombe Blvd, Houston 77030, USA. Electronic address: jajani@mdanderson.org. (2)Department of Biostatistics, University of Texas M. D. Anderson Cancer Center, 1515 Holcombe Blvd, Houston 77030, USA. (3)Department of Gastrointestinal Medical Oncology, University of Texas M. D. Anderson Cancer Center, 1515 Holcombe Blvd, Houston 77030, USA. (4)Department of Cardiac and Thoracic Surgery, University of Texas M. D. Anderson Cancer Center, 1515 Holcombe Blvd, Houston 77030, USA. (5)Department of Radiation Oncology, University of Texas M. D. Anderson Cancer Center, 1515 Holcombe Blvd, Houston

77030, USA. (6)Department of Pathology, University of Texas M. D. Anderson Cancer Center, 1515 Holcombe Blvd, Houston 77030, USA. (7)Department of Gastroenterology, Hepatology, and Nutrition, University of Texas M. D. Anderson Cancer Center, 1515 Holcombe Blvd, Houston 77030, USA. (8)Department of Genetics, University of Texas M. D. Anderson Cancer Center, 1515 Holcombe Blvd, Houston 77030, USA.

PURPOSE: Operable thoracic esophageal/gastroesophageal junction carcinoma (EC) is often treated with chemoradiation and surgery but tumor responses are unpredictable and heterogeneous. We hypothesized that aldehyde dehydrogenase-1 (ALDH-1) could be associated with response.
METHODS: The labeling indices (LIs) of ALDH-1 by immunohistochemistry in untreated tumor specimens were established in EC patients who had chemoradiation and surgery. Univariate logistic regression and 3-fold cross validation were carried out for the training (67% of patients) and validation (33%) sets. Non-clinical experiments in EC cells were performed to generate complimentary data.
RESULTS: Of 167 EC patients analyzed, 40 (24%) had a pathologic complete response (pathCR) and 27 (16%) had an extremely resistant (exCRTR) cancer. The median ALDH-1 LI was 0.2 (range, 0.01-0.85). There was a significant association between pathCR and low ALDH-1 LI ($p \leqslant 0.001$; odds-ratio [OR] = 0.432). The 3-fold cross validation led to a concordance index (C-index) of 0.798 for the fitted model. There was a significant association between exCRTR and high ALDH-1 LI ($p \leqslant 0.001$; OR = 3.782). The 3-fold cross validation led to the C-index of 0.960 for the fitted model. In several cell lines, higher ALDH-1 LIs correlated with resistant/aggressive phenotype. Cells with induced chemotherapy resistance upregulated ALDH-1 and resistance conferring genes (SOX9 and YAP1). Sorted ALDH-1+ cells were more resistant and had an aggressive phenotype in tumor spheres than ALDH-1- cells.
CONCLUSIONS: Our clinical and non-clinical data demonstrate that ALDH-1 LIs are predictive of response to therapy and further research could lead to individualized therapeutic strategies and novel therapeutic targets for EC patients.

Copyright © 2013 Federation of European Biochemical Societies. Published by Elsevier B.V. All rights reserved.

PMCID: PMC3946849
PMID: 24210755 [PubMed - indexed for MEDLINE]

41. Thorac Surg Clin. 2013 Nov;23(4):509-23. doi: 10.1016/j.thorsurg.2013.07.005. Epub 2013 Oct 13.

Neoadjuvant chemotherapy or chemoradiotherapy for locally advanced esophageal cancer.

Smithers BM(1), Thomson I.

Author information:
(1)Upper GI, Soft Tissue Unit, Discipline of Surgery, Princess Alexandra Hospital, The University of Queensland, Ipswich Road, Buranda, Brisbane 4102, Australia. Electronic address: m.smithers@uq.edu.au.

In patients with operable esophageal cancer, there is evidence supporting the use of preoperative chemotherapy or preoperative chemoradiation. The addition of radiotherapy to chemotherapy seems more relevant for the more locally advanced cancers. There is a need to examine in trials more modern chemotherapy combinations with and without concurrent radiation and for research into assessing methods for predicting outcomes from neoadjuvant therapy as part of the paradigm of therapy for this disease.

Copyright © 2013 Elsevier Inc. All rights reserved.

PMID: 24199701 [PubMed - indexed for MEDLINE]

42. J Nippon Med Sch. 2013;80(5):378-83.

Adjuvant chemotherapy with S-1 followed by docetaxel for gastric cancer and CY1P0 peritoneal metastasis after relatively curative surgery.

Kanazawa Y(1), Kato S, Fujita I, Onodera H, Uchida E.

Author information:
(1)Department of Surgery, Nippon Medical School.

OBJECTIVE: The aim of this study was to assess the feasibility and safety of

adjuvant chemotherapy with S-1 followed by docetaxel.
PATIENTS AND METHOD: Twenty-eight patients with advanced gastric cancer underwent gastrectomy without preoperative chemotherapy. These patients were divided into 3 groups on the basis of cytologic results of peritoneal lavage (CY) and the presence of local peritoneal metastatic nodules (P): CY1-P0, CY0-P1, and CY1-P1. Oral S-1 (80 mg/m(2)/day) was administered for 3 consecutive weeks, followed by intravenous docetaxel (35 mg/m(2)) on days 29 and 43 (1 cycle). This cycle was repeated every 8 weeks. The primary endpoint was the ability to complete 6 cycles of S-1 followed by docetaxel. The secondary endpoints were safety, progression-free survival, mean survival time (MST), and overall survival (OS).
RESULTS: The subjects were 18 men and 10 women (39 to 78 years old, median age, 64 years). The extent of peritoneal metastasis was CY1-P0 in 8 patients, CY0-P1 in 14 patients, and CY1-P1 in 6 patients. Both hematologic and nonhematologic toxicities were generally mild. The completion rate of the planned 6 cycles of the protocol was 71.4% (20 of 28 patients). Median progression-free survival was 22.9 months, and the 2-year survival rate was 78.6%. The overall MST was 34.3 months, and the MST by group was 34.5 for CY1-P0, 34.3 for CY0-P1, and 19.3 months for CY1-P1. The OS in the CY1-P0 and CY0-P1 groups was significantly longer than that in the CY1-P1 group (P<0.05).
CONCLUSION: Adjuvant chemotherapy with S-1 followed by docetaxel is safe and well tolerated and has the potential to improve OS in patients with a status of CY1P0 following relatively curative resection.

PMID: 24189356 [PubMed - indexed for MEDLINE]

43. Ann Ital Chir. 2013 Sep-Oct;84(5):551-6.

Preliminary results of prophylactic HIPEC in patients with locally advanced gastric cancer.

Graziosi L, Cantarella F, Mingrone E, Gunnellini M, Cavazzoni E, Liberati M, Donini A.

BACKGROUND: The prognosis of locally advanced Gastric Cancer following surgical therapy alone is poor. Peritoneum represents a preferential site of dissemination in such neoplasm. Hyperthermic intraperitoneal chemotherapy (HIPEC) has been used in association with cytoreductive surgery (CRS) in the treatment of GC peritoneal carcinomatosis (PC). Aim of our preliminary experience is reporting our data on

prophylactic HIPEC (P-HIPEC) in patients with GC at high risk of developing PC.
METHODS: Eleven patients underwent P-HIPEC at our General and Emergency Surgery Department. All the patients were affected of high risk GC: serosa invasive tumors (T4), conventional cytology-positive or quantitative PCR detection of CEA mRNA on peritoneal lavage. Seven subtotal and four total gastrectomies with D2 or D2+ were performed. All the anastomoses were made before HIPEC. The procedure was carried out for 60 minutes with Mytomicin C and Cisplatin in all patients. Post-operative monitoring in Intensive Care Unit least for 24-48 hours. Oral nutrition was started precociously (day 5) also according with bowel movements and stool/gas passage. Follow-up took place in all patients at 1 month from surgery then every 6 months for 2 years and every 12 months for the following years.
RESULTS: In four patients a neoadjuvant treatment was scheduled due to T or N stage at pre-operative evaluation. Gastric resection was guided on tumor location while the choice of performing a D2 or D2 + lymphadenectomy was up to preoperative imaging and intra-operative nodal status. No intra-operative complications were recorded. Median operation time was 398 minutes. In our series we recorded 20 adverse events. Median number for each patient was 1 adverse effect (range 0-2). Eight patients experienced a surgical adverse effect (G2-G3) that did not require any surgical treatment. Only one patient with duodenal stump dehiscence and intra-abdominal sepsis (G4-G5) underwent re-operation and died for severe hemorrhagic pancreatitis. Another patient died for ARDS. Per-operative mortality was 18%. Both patients were older then 70 years old. Median hospital stay was 14 days. Median follow-up was 15.9 months. Median survival was 29.6 months and median DFS was 20 months. Only one patient developed a peritoneal recurrence at 12 months and died for disease progression. Seven patients are still alive and disease free at last follow-up. One patient affected of variable immunodeficiency died at 9 months for pulmonary sepsis without any sign of local recurrence.
CONCLUSIONS: Peritoneal dissemination appears to be a strong determinant in defining GC patients prognosis. Even after curative resection, peritoneal recurrence develops in about 60% of the patients with T3 and T4 tumors, and up to 40% of resected gastric cancer patients die as a direct result of peritoneal dissemination. Clinical trials showed that surgery plus HIPEC was associated with a significant improvement in survival compared to surgery alone in patients affected of GC with resectable PC. At present day there are not studies evaluating the role of P-HIPEC in patients at high risk of developing PC. The rationale of P-HIPEC is based on the concept that positive peritoneal lavage is considered an M1 (stage IV) similarly to macroscopic PC by the 7th TNM

classification. Also analogous is the median survival of this 2 groups of patients. Detection of peritoneal micrometastases with cytologic examination has been considered a major method to predict peritoneal recurrences; the sensitivity of this assay is low. Recently, molecular approaches using real-time reverse-transcriptase polymerase chain reaction (RT-PCR) technique has made possible the increase in the sensitivity. We can conclude, although the preliminary experience, that prophylactic HIPEC in locally advanced gastric cancer is feasible, increasing median survival compared to surgery alone. For sure this procedure need to be performed in the highly specialized centres strongly respecting the eligibility criteria.

PMID: 24140896 [PubMed - in process]

44. World J Gastroenterol. 2013 Sep 28;19(36):6062-8. doi:
10.3748/wjg.v19.i36.6062.

Clinical outcomes of radiation therapy for early-stage gastric mucosa-associated lymphoid tissue lymphoma.

Kim SW(1), Lim do H, Ahn YC, Kim WS, Kim SJ, Ko YH, Kim KM.

Author information:
(1)Sang-Won Kim, Do Hoon Lim, Yong Chan Ahn, Department of Radiation Oncology, Samsung Medical Center, Sungkyunkwan University School of Medicine, Seoul 135-710, South Korea.

AIM: To evaluate the clinical outcomes of radiation therapy (RT) for early-stage gastric mucosa-associated lymphoid tissue lymphoma (MALToma).
METHODS: The records of 64 patients treated between 1998 and 2011 were analyzed retrospectively. For Helicobacter pylori (H. pylori)-positive patients (n = 31), chemotherapy or H. pylori eradication therapy was the initial treatment. In patients with failure after H. pylori eradication, RT was performed. For H. pylori-negative patients (n = 33), chemotherapy or RT was the first-line treatment. The median RT dose was 36 Gy. The target volume included the entire stomach and the perigastric lymph node area.
RESULTS: All of the patients completed RT without interruption and showed complete remission on endoscopic biopsy after treatment. Over a median follow-up period of 39 mo, the 5-year local control rate was 89%. Salvage therapy was

successful in all relapsed patients. Secondary malignancies developed in three patients. The 5-year overall survival rate was 94%. No patient presented symptoms of moderate-to-severe treatment-related toxicities during or after RT.
CONCLUSION: Radiotherapy results in favorable clinical outcomes in patients with early-stage gastric MALToma who experience failure of H. pylori eradication therapy and those who are H. pylori negative.

PMCID: PMC3785628
PMID: 24106407 [PubMed - indexed for MEDLINE]

45. Gan To Kagaku Ryoho. 2013 Oct;40(10):1331-5.

[Neoadjuvant chemotherapy with combined S-1 plus weekly low-dose cisplatin followed by surgical resection for advanced gastric cancer].

[Article in Japanese]

Isobe T(1), Hashimoto K, Kizaki J, Miyagi M, Aoyagi K, Koufuji K, Shirouzu K.

Author information:
(1)Dept. of Surgery, Kurume University School of Medicine.

We retrospectively examined patients with advanced gastric cancer who underwent gastrectomy following neoadjuvant chemotherapy (NAC) with S-1 plus weekly low-dose cisplatin (CDDP). Between 2007 and 2009, 27 patients with advanced gastric cancer not amenable to curative surgery were enrolled. One course of NAC comprised S-1 (80 mg/m2/day) for 21 consecutive days and CDDP (20 mg/m2) on days 1, 8, and 15; this was followed by a 2-week rest after the end of S-1 administration. Grade 3 side effects were observed in 5 patients: 3 experienced neutropenia and 2 experienced digestive symptoms. The outpatient completion rate was 81.5% (22/27); there was no incidence of renal dysfunction. During pretherapy diagnosis, depth of invasion was classified as T4 in all cases. Postoperative pathologic results showed that the depth of invasion was T3 or lower in 4 patients. In addition, the number of patients with N0 and M0 classification increased and downstaging was observed in 12 patients (44.4%). A comprehensive assessment revealed that a partial response (PR) was observed in 13 patients and stable disease (SD) was observed in 12 patients, resulting in a response rate of 48.1%. The median survival time was 580 days, and the 1-year survival rate was

72%. NAC with S-1 plus weekly low-dose CDDP can also be administered on an outpatient basis, and it is a potential regimen for the treatment of advanced gastric cancer associated with a poor prognosis.

PMID: 24105055 [PubMed - indexed for MEDLINE]

46. J Thorac Oncol. 2013 Nov;8(11):1425-9. doi: 10.1097/JTO.0b013e3182a46c3b.

A phase II study with cetuximab and radiation therapy for patients with surgically resectable esophageal and GE junction carcinomas: Hoosier Oncology Group G05-92.

Becerra CR(1), Hanna N, McCollum AD, Becharm N, Timmerman RD, DiMaio M, Kesler KA, Yu M, Yan T, Choy H.

Author information:
(1)*US Oncology Research, McKesson Specialty Health, The Woodlands, Texas, and Texas Oncology-Baylor Sammons Cancer Center, Dallas, Texas; †Indiana University Melvin and Bren Simon Cancer Center, Indianapolis, Indiana; ‡Community Oncology Center, Kokomo, Indiana; §University of Texas Southwestern Medical Center at Dallas, Dallas, Texas; and ‖Indiana University School of Medicine, Indianapolis, Indiana.

INTRODUCTION: On the basis of the promising activity of cetuximab and radiation therapy for head and neck cancers, we evaluated the efficacy of this regimen followed by surgery in patients with resectable esophageal cancer. This was a phase II, open-label, single-arm, multicenter study of patients with potentially resectable esophageal cancer.
METHODS: Patients received two weekly doses of cetuximab followed by weekly cetuximab combined with radiation therapy for 6 weeks. After a 6- to 8-week rest, patients' primary tumor was resected. The main objective was to evaluate pathologic complete response (pCR) rate in the primary tumor after cetuximab and radiation therapy.
RESULTS: Thirty-nine patients completed the study. Most patients were men (93%), median age was 64 years, performance status was 0 to 1 (95%), patients had a histology of adenocarcinoma (78%), and tumors were located in the esophagus (63%). Grade 3 toxicities in more than 5% of patients included dysphagia (17%), anorexia and dehydration (7%), and dyspnea, fatigue, hypernatremia (5%). Grade 5

aspiration occurred in 2% (1 patient). Four patients died, two from disease progression, one from aspiration pneumonia postsurgery, and one from septic shock. Thirty-one patients (76%) underwent esophagectomy. The pCR rate was 36.6% by intention-to-treat and 48% for patients who underwent esophagectomy. The pCR by histology was 6 of 9 (67%) for squamous cell carcinomas and 9 of 32 (28%) for adenocarcinoma. Earlier-stage disease was associated with increased pCR (IIA 70%, IIB 29%, III 28%).
CONCLUSIONS: Cetuximab and radiation therapy results in a pCR rate that seems at least comparable with that of chemotherapy and radiation therapy. This regimen may be better tolerated than preoperative chemotherapy and radiation therapy in patients with resectable esophageal cancers.

PMID: 24084441 [PubMed - indexed for MEDLINE]

47. Ann Oncol. 2013 Nov;24(11):2854-9. doi: 10.1093/annonc/mdt340. Epub 2013 Aug 29.

Results of the baseline positron emission tomography can customize therapy of localized esophageal adenocarcinoma patients who achieve a clinical complete response after chemoradiation.

Suzuki A(1), Xiao L, Taketa T, Sudo K, Wadhwa R, Blum MA, Skinner H, Komaki R, Weston B, Lee JH, Bhutani MS, Rice DC, Maru DM, Erasmus J, Swisher SG, Hofstetter WL, Ajani JA.

Author information:
(1)Department of Gastrointestinal Medical Oncology.

BACKGROUND: Patients with localized esophageal adenocarcinoma (EAC) who achieve a clinical complete response (clinCR) after preoperative chemoradiation (trimodality therapy; TMT) or definitive chemoradiation (bimodality therapy; BMT) live longer than those who achieve a <clinCR (Suzuki A, Xiao LC, Hayashi Y et al. Prognostic significance of baseline positron emission tomography and importance of clinical complete response in patients with esophageal or gastroesophageal junction cancer treated with definitive chemoradiotherapy. Cancer 2011; 117: 4823-4833; Cheedella NK, Suzuki A, Xiao L et al. Association between clinical complete response and pathological complete response after preoperative chemoradiation in patients with gastroesophageal cancer: analysis in a large

cohort. Ann Oncol 2013; 24: 1262-1266; Ajani JA, Correa AM, Hofstetter WL et al. Clinical parameters model for predicting pathologic complete response following preoperative chemoradiation in patients with esophageal cancer. Ann Oncol 2012; 23: 2638-2642). We hypothesized that the initial standardized uptake value (iSUV) of positron emission tomography will define novel subsets of clinCR patients.
METHODS: We analyzed 323 EAC patients, from our prospective database, who achieved a clinCR. Various statistical methods were used to assess the influence of iSUV on patient outcome.
RESULTS: The median follow-up of 323 patients was 40.8 months [95% confidence interval (CI) 35.6-47.3 months]. Two hundred six (63.8%) patients had TMT and 117 (36.2%) had BMT. If iSUV was ≥6, TMT patients had a longer median OS (94.8 months; 95% CI 66.07-NA) than BMT patients (31.4 months; 95% CI 21.7-42.1; P ≤ 0.001). However, if iSUV was <6, the median OS of TMT and BMT patients was similar (P = 0.62). iSVU did not influence the pathologic complete response rate in TMT patients (P = 0.85).
CONCLUSION: clinCR patients with iSUV of <6 are identified as a new subset that fared equally well when treated with TMT or BMT. Future esophageal preservation strategy may be best suited for this newly identified subset of EAC patients.

PMID: 23994746 [PubMed - indexed for MEDLINE]

48. Gan To Kagaku Ryoho. 2013 Jun;40(6):793-5.

[A case of advanced gastric cancer with bulky lymph node metastases responding to S-1/CDDP neoadjuvant chemotherapy and leading to less invasive surgery and a pathologically complete response].

[Article in Japanese]

Eshita Y(1), Ogawa F.

Author information:
(1)Dept. of Surgery, Takashimadaira Central General Hospital, Japan.

A 53-year-old man was diagnosed with advanced gastric cancer(cT4aN2M0, StageIII B), with regional bulky lymph node metastases invading the splenic artery. S-1 plus cisplatin treatment(S-1/CDDP)was administered as neoadjuvant chemo-therapy(NAC). S-1(80mg/m2)was administered orally for 21 days, followed by 14

drug-free days as a course. CDDP(60 mg/m2)was administered by intravenous drip on day 8. After two courses, significant tumor reduction was obtained, and the patient then had total gastrectomy and splenectomy performed with a D2 dissection. Distal pancreatectomy was avoided. Macroscopically, the stomach seemed to be penetrated by the tumor into the serosa, yet the histological diagnosis revealed complete disappearance of cancer cells in all of the lymph nodes, and very few residual tumor cells were noted, only on the gastric mucosa(pT1aN0M0, Stage I A). Therefore, downstaging was confirmed. S-1/CDDP as a NAC regimen for advanced gastric cancer appears to be an effective treatment.

PMID: 23863661 [PubMed - indexed for MEDLINE]

49. Gan To Kagaku Ryoho. 2013 May;40(5):643-6.

[Pathological complete response in a case of advanced esophageal cancer invading aorta treated by preoperative chemotherapy with docetaxel and cisplatin plus 5-FU].

[Article in Japanese]

Nishino T(1), Yamamoto Y, Ikeda M, Morimoto M, Furukawa T, Goto M, Furukita Y, Takechi H, Seike J, Tangoku A, Fujiwara H.

Author information:
(1)Dept. of Thoracic Endocrine Surgery and Oncology, Tokushima University Hospital, Japan.

The patient was a 64-year-old man, diagnosed as cStage IVa esophageal cancer invading the aorta with lymph node metastasis. He received combination chemotherapy with docetaxel/cisplatin/5-FU(DFP therapy). After one course, CT and endoscopic examination showed remarkable reduction of the primary lesion and lymph node metastasis. We performed subtotal esophagectomy and gastric tube reconstruction by the retroposterior mediastinum route. The pathological specimen evidenced fibrosis and infiltration of inflammatory cells on almost all layers, but showed no viable malignant cells in the middle thoracic esophagus. Therefore, the pathological effect was judged as Grade 3(pCR). This case suggested that DFP combination chemotherapy may prove to be a useful treatment for advanced esophageal cancer with invasion to other organs.

PMID: 23863591 [PubMed - indexed for MEDLINE]

50. Ann Surg Oncol. 2014 Jan;21(1):213-9. doi: 10.1245/s10434-013-3055-x. Epub 2013 Jul 10.

Induction of a pathological complete response by four courses of neoadjuvant chemotherapy for gastric cancer: early results of the randomized phase II COMPASS trial.

Yoshikawa T(1), Tanabe K, Nishikawa K, Ito Y, Matsui T, Kimura Y, Hirabayashi N, Mikata S, Iwahashi M, Fukushima R, Takiguchi N, Miyashiro I, Morita S, Miyashita Y, Tsuburaya A, Sakamoto J.

Author information:
(1)Department of Gastrointestinal Surgery, Kanagawa Cancer Center, Yokohama, Japan, yoshikawat@kcch.jp.

Comment in
    Ann Surg Oncol. 2014 Jan;21(1):13-5.

BACKGROUND: The prognosis for stage 3 gastric cancer is not satisfactory, even with S-1 adjuvant chemotherapy. A randomized phase II trial was conducted to compare two and four courses of neoadjuvant S-1/cisplatin (SC) and paclitaxel/cisplatin (PC) using a two-by-two factorial design for locally advanced gastric cancer. The primary endpoint was overall survival. We clarified the impact of these regimens on the secondary endpoints, including the clinical and pathological responses, chemotherapy-related toxicities, and surgical results.
METHODS: Patients received S-1 (80 mg/m(2) for 21 days with 1 week's rest)/cisplatin (60 mg/m(2) at day 8) or paclitaxel/cisplatin (80 and 25 mg/m(2), respectively, on days 1, 8, and 15 with 1 week's rest) as neoadjuvant chemotherapy.
RESULTS: Eighty-three patients were assigned to arm A (two courses of SC, n = 21), arm B (four courses of SC, n = 20), arm C (two courses of PC, n = 21), and arm D (four courses of PC, n = 21). Pathological response rate was 43 % in arm A, 40 % in arm B, 29 % in arm C, and 38 % in arm D. Pathological complete response was only observed in arms B (10 %) and D (10 %). Most bone marrow toxicities,

nausea, vomiting, alopecia, and fatigue were slightly higher but acceptable in arms B and D. Grade 3/4 surgical morbidities were not commonly observed in all four arms.
CONCLUSIONS: Pathological complete response could be induced by four courses of neoadjuvant chemotherapy without a marked increase of toxicities, regardless of a SC or PC regimen.

PMID: 23838904 [PubMed - indexed for MEDLINE]

51. Eur J Surg Oncol. 2013 Aug;39(8):814-22. doi: 10.1016/j.ejso.2013.05.003. Epub 2013 Jun 5.

Perioperative chemotherapy for resectable gastroesophageal cancer: a single-center experience.

Molina R(1), Lamarca A, Martínez-Amores B, Gutiérrez A, Blázquez A, López A, Granell J, Álvarez-Mon M.

Author information:
(1)Department of Oncology, Principe de Asturias Universitary Hospital, Alcalá University, Alcalá de Henares, Madrid, Spain.

BACKGROUNDS: Multimodal treatment for locally advanced gastric cancer has been reported to improve disease-free survival when compared to surgery alone. We aimed to clarify the efficacy and safety of perioperative chemotherapy for locally advanced gastric cancer patients treated in daily clinical practice.
METHODS: Patients diagnosed with locally advanced gastric cancer were treated with perioperative chemotherapy and surgery. The primary end point was the complete resection (R0) rate. Secondary end points were disease-free survival (DFS), overall survival (OS), toxicity, radiological response rate, pathological response rate and downstaging rate. We also looked for prognostic and predictive factors for DFS, OS, pathological complete response and the R0 rate.
RESULTS: Forty patients were found eligible for this retrospective analysis. At diagnosis, 52.5% of patients were classified as stage II and 47.5% were stage III. Forty percent of patients completed three preoperative cycles and three postoperative cycles. A tolerable toxicity related to chemotherapy was found. Thirty-nine patients underwent surgery: 80% reached a complete resection (R0), down-staging was detected in 57.5% and 17.5% had a pathologically complete

response. The median time of disease-free survival was 34.05 months (95%CI 25.6-42.4), and the median time of overall survival was 39.01 months (95%CI 30.8-47.1). We found that the presence of comorbidities were independent predictive factors for the pathologic response, while the chemotherapy schedule and the clinical response could independently predict a complete resection.
CONCLUSIONS: Our results support that perioperative chemotherapy for locally advanced gastric cancer can be safely delivered in daily clinical practice, obtaining an improvement of the pathologic response and the complete resection of gastric cancer.

Copyright © 2013 Elsevier Ltd. All rights reserved.

PMID: 23755989 [PubMed - indexed for MEDLINE]

52. BMC Cancer. 2013 Jun 7;13:279. doi: 10.1186/1471-2407-13-279.

Short-course radiotherapy followed by neo-adjuvant chemotherapy in locally advanced rectal cancer--the RAPIDO trial.

Nilsson PJ(1), van Etten B, Hospers GA, Påhlman L, van de Velde CJ, Beets-Tan RG, Blomqvist L, Beukema JC, Kapiteijn E, Marijnen CA, Nagtegaal ID, Wiggers T, Glimelius B.

Author information:
(1)Department of Molecular Medicine and Surgery, Karolinska Institutet and Center for Surgical Gastroenterology, Karolinska University Hospital, Solna P9:03, SE 171 76 Stockholm, Sweden. per.nilsson@karolinska.se

BACKGROUND: Current standard for most of the locally advanced rectal cancers is preoperative chemoradiotherapy, and, variably per institution, postoperative adjuvant chemotherapy. Short-course preoperative radiation with delayed surgery has been shown to induce tumour down-staging in both randomized and observational studies. The concept of neo-adjuvant chemotherapy has been proven successful in gastric cancer, hepatic metastases from colorectal cancer and is currently tested in primary colon cancer.
METHODS AND DESIGN: Patients with rectal cancer with high risk features for local or systemic failure on magnetic resonance imaging are randomized to either a standard arm or an experimental arm. The standard arm consists of chemoradiation

(1.8 Gy x 25 or 2 Gy x 25 with capecitabine) preoperatively, followed by selective postoperative adjuvant chemotherapy. Postoperative chemotherapy is optional and may be omitted by participating institutions. The experimental arm includes short-course radiotherapy (5 Gy x 5) followed by full-dose chemotherapy (capecitabine and oxaliplatin) in 6 cycles before surgery. In the experimental arm, no postoperative chemotherapy is prescribed. Surgery is performed according to TME principles in both study arms. The hypothesis is that short-course radiotherapy with neo-adjuvant chemotherapy increases disease-free and overall survival without compromising local control. Primary end-point is disease-free survival at 3 years. Secondary endpoints include overall survival, local control, toxicity profile, and treatment completion rate, rate of pathological complete response and microscopically radical resection, and quality of life.
DISCUSSION: Following the advances in rectal cancer management, increased focus on survival rather than only on local control is now justified. In an experimental arm, short-course radiotherapy is combined with full-dose chemotherapy preoperatively, an alternative that offers advantages compared to concomitant chemoradiotherapy with or without postoperative chemotherapy. In a multi-centre setting this regimen is compared to current standard with the aim of improving survival for patients with locally advanced rectal cancer.
TRIAL REGISTRATION: ClinicalTrials.gov NCT01558921.

PMCID: PMC3680047
PMID: 23742033 [PubMed - indexed for MEDLINE]

53. Am J Surg Pathol. 2013 Jul;37(7):1022-9. doi: 10.1097/PAS.0b013e31828778fd.

Postoperative nodal status and diffuse-type histology are independent prognostic factors in resectable advanced gastric carcinomas after preoperative chemotherapy.

Koh YW(1), Park YS, Ryu MH, Ryoo BY, Park HJ, Yook JH, Kim BS, Kang YK.

Author information:
(1)Department of Pathology, Asan Medical Center, University of Ulsan College of Medicine, Seoul, Republic of Korea.

Surgical resection of primary gastric lesions after neoadjuvant or palliative chemotherapy is performed for curative or palliative purpose in locally advanced

(LA) or initially metastatic (IM) gastric cancer. We investigated which histomorphologic features were associated with patient prognosis. We examined 143 patients (57 LA and 86 IM) who underwent gastrectomy after chemotherapy between 2000 and 2009. The tumor regression grade (TRG)-determined by examining the residual neoplastic cells and background stromal changes-was evaluated. Progression-free (PFS) and overall survival (OS) were evaluated according to pretherapeutic and posttherapeutic clinicopathologic factors using univariate and multivariate analyses. Because both the LA and the IM groups showed similar trends of PFS and OS according to TRG, the 2 groups were analyzed together. Patients with TRG1 (no residual primary tumor) showed a superior PFS and OS than the remaining TRGs. We defined pathologic complete regression (pCR) as TRG1 with negative lymph nodes (LN) and the others as non-pCR. Sixteen patients (11.1%) had pCR with better PFS (P=0.007) and OS (P=0.006). Initial disease status (LA or IM) remained as independent prognostic factors for PFS (P=0.021) but not for OS (P=0.109). The postoperative negative LN status correlated with good outcome and postoperative diffuse-type histology correlated with poor outcome after multivariate analysis. This study showed that pCR, but not partial regression, provides meaningful prognostic information in gastrectomy after chemotherapy. In addition, postoperative LN positivity and diffuse-type histology were independent poor prognostic factors for PFS and OS.

PMID: 23715160 [PubMed - indexed for MEDLINE]

54. Yonsei Med J. 2013 Jul;54(4):888-94. doi: 10.3349/ymj.2013.54.4.888.

Pathologic and oncologic outcomes in locally advanced gastric cancer with neoadjuvant chemotherapy or chemoradiotherapy.

An JY(1), Kim HI, Cheong JH, Hyung WJ, Kim CB, Noh SH.

Author information:
(1)Department of Surgery, Yonsei University College of Medicine, 50 Yonsei-ro, Seodaemun-gu, Seoul 120-752, Korea. sunghoonn@yuhs.ac.

PURPOSE: Although neoadjuvant therapy has been accepted as a treatment option in locally-advanced gastric cancer, its prognostic value has been difficult to evaluate.
MATERIALS AND METHODS: Seventy-four gastric cancer patients who underwent

gastrectomy after neoadjuvant treatment were divided into two groups according to the pathologic response: favorable (ypT0) and others (ypT1-4). The clinicopathologic characteristics, predictive factors for pathologic response, and oncologic outcome were evaluated.
RESULTS: Eleven patients (14.8%) demonstrated ypT0 and the remaining 63 patients (85.2%) were ypT1-4. Chemoradiotherapy (CCRTx) rather than chemotherapy (CTx) was the only predictive factor for a favorable pathologic response. Chemotherapeutic factors and tumor marker levels did not predict pathologic response. The 1-, 2-, and 3-year disease-free survivals were 83.4%, 70%, and 52.2%. The 1-, 3-, 5-year overall survivals were 88.5%, 67.5%, and 51.2%, respectively. Although a complete pathologic response (ypT0N0M0) was achieved in 7 patients, 28.6% of them demonstrated recurrence of the tumor within 6 months after curative surgery.
CONCLUSION: CCRTx rather than CTx appears to be more effective for achieving good pathologic response. Although favorable pathologic response has been achieved after neoadjuvant treatment, the survival benefit remains controversial.

PMCID: PMC3663211
PMID: 23709422 [PubMed - indexed for MEDLINE]

55. Ann Oncol. 2013 Aug;24(8):2068-73. doi: 10.1093/annonc/mdt141. Epub 2013 Apr 16.

Impact of pathologic complete response on disease-free survival in patients with esophagogastric adenocarcinoma receiving preoperative docetaxel-based chemotherapy.

Lorenzen S(1), Thuss-Patience P, Al-Batran SE, Lordick F, Haller B, Schuster T, Pauligk C, Luley K, Bichev D, Schumacher G, Homann N.

Author information:
(1)3rd Department of Internal Medicine, Hematology/Medical Oncology, Klinikum rechts der Isar, Technische Universität München, Munich, Germany.
sylvie.lorenzen@mri.tum.de

BACKGROUND: The aim of this study was to evaluate the impact of pathologic complete response (pCR) on outcome in patients with gastric or esophagogastric junction (EGJ) adenocarcinoma after neoadjuvant docetaxel/platin/fluoropyrimidine-based chemotherapy.

PATIENTS AND METHODS: Patients received at least one cycle of chemotherapy for potentially operable disease. Pretreatment clinicopathologic factors and pCR were investigated. Disease-free survival (DFS), overall survival (OS) and tumor-related death were correlated with pCR.
RESULTS: One hundred twenty patients were included in this analysis. Eighteen patients (15%) achieved a pCR. Tumor localization in the EGJ was identified as the only significant predictor of pCR (P = 0.019). Median follow-up was 41.1 months. Median DFS and OS for all patients were 24.1 and 48.6 months, respectively. Median DFS for patients with a pCR was not reached versus 22.1 months non-pCR patients (hazard ratio, HR 0.38; 3-year DFS: 71.8% and 37.7%, respectively, P = 0.018). While OS was not significantly different, the risk for tumor-related death was significantly lower for pCR patients compared with non-pCR patients (3-year cumulative incidences of 6.4% and 45.4%, respectively, P = 0.009).
CONCLUSION: A pCR following preoperative docetaxel/platin/fluoropyrimidine indicates favorable outcome in patients with gastric or EGJ adenocarcinoma. Tumor location in the EGJ is associated with a higher pCR rate.

PMID: 23592699 [PubMed - indexed for MEDLINE]

56. Zhonghua Wei Chang Wai Ke Za Zhi. 2013 Feb;16(2):166-9.

[Efficacy of neoadjuvant chemotherpy in patients with locally advanced gastric cancer].

[Article in Chinese]

Wang Y(1), Liu TS, Zhuang RY, Cui YH, Wang ZM, Yu YY, Hou J, Sun YH, Shen KT, Shen ZB.

Author information:
(1)Department of Medical Oncology, Zhongshan Hospital, Fudan University, Shanghai 200032, China.

OBJECTIVE: To evaluate the efficacy and safety of neoadjuvant chemotherapy in patients with locally advanced gastric cancer, and to analyze the relevant factors of recurrent death of gastric cancer after adjuvant chemotherapy.
METHODS: Clinical data of 49 patients who underwent neoadjuvant chemotherapy for

locally advanced gastric cancer between July 2007 and June 2011 were reviewed. Preoperative staging was determined by endoscopic ultrasonography and abdominal computer tomography (CT) or magnetic resonance imaging (MRI). Chemotherapy was administered for regimen of two or three drugs. Prognostic factors were analyzed by univariate and multivariate analysis with Cox proportional hazard model.
RESULTS: The response rate was 33.3% (16/48) and disease control rate was 93.8% (45/48). Forty-four (89.8%, 44/49) patients received curative resection after neoadjuvant chemotherapy, among whom 90.9% (40/44) underwent D2 lymphadenctomy. Thirty-two cases had pathological response and 2 patients had pathological complete response. The average hospital stay was 11.6 days and 2 patients had longer hospitalization because of postoperative pancreatic complications. The toxicities were most in grade 1-2. All the patients were followed up postoperatively and the median follow-up was 21.6 months. Median progression-free survival was 29.6 (95%CI:24.0-35.2) months and median overall survival was 34.6 months (95%CI:29.8-39.4). Imaging response (P=0.038, RR=0.168, 95%CI:0.031-0.904) and pathological response (P=0.007, RR=0.203, 95%CI:0.064-0.642) were identified as independent prognostic factors with COX multivariate analysis.
CONCLUSIONS: Neoadjuvant chemotherapy has quite high disease control rate and R0 resecting rate for patients with locally advanced gastric cancer. Imaging response and pathological response are most important prognostic factors in those patients.

PMID: 23446480 [PubMed - indexed for MEDLINE]

57. Oncologist. 2013;18(3):281-7. doi: 10.1634/theoncologist.2012-0208. Epub 2013 Feb
21.

Preoperative cetuximab, irinotecan, cisplatin, and radiation therapy for patients with locally advanced esophageal cancer.

Lee MS(1), Mamon HJ, Hong TS, Choi NC, Fidias PM, Kwak EL, Meyerhardt JA, Ryan DP, Bueno R, Donahue DM, Jaklitsch MT, Lanuti M, Rattner DW, Fuchs CS, Enzinger PC.

Author information:
(1)Department of Medicine, Brigham and Women’s Hospital, Boston, Massachusetts 02215, USA.

PURPOSE: To determine the efficacy and toxicity of weekly neoadjuvant cetuximab combined with irinotecan, cisplatin, and radiation therapy in patients with locally advanced esophageal or gastroesophageal junction cancer.
METHODS AND MATERIALS: Patients with stage IIA-IVA esophageal or gastroesophageal junction cancer were enrolled in a Simon's two-stage phase II study. Patients received weekly cetuximab on weeks 0-8 and irinotecan and cisplatin on weeks 1, 2, 4, and 5, with concurrent radiotherapy (50.4 Gy on weeks 1-6), followed by surgical resection.
RESULTS: In the first stage, 17 patients were enrolled, 16 of whom had adenocarcinoma. Because of a low pathologic complete response (pCR) rate in this cohort, the trial was discontinued for patients with adenocarcinoma but squamous cell carcinoma patients continued to be enrolled; two additional patients were enrolled before the study was closed as a result of poor accrual. Of the 19 patients enrolled, 18 patients proceeded to surgery, and 16 patients underwent an R0 resection. Three patients (16%) had a pCR. The median progression-free survival interval was 10 months, and the median overall survival duration was 31 months. Severe neutropenia occurred in 47% of patients, and severe diarrhea occurred in 47% of patients. One patient died preoperatively from sepsis, and one patient died prior to hospital discharge following surgical resection.
CONCLUSIONS: This schedule of cetuximab in combination with irinotecan, cisplatin, and radiation therapy was toxic and did not achieve a sufficient pCR rate in patients with localized esophageal adenocarcinoma to undergo further evaluation.

PMCID: PMC3607524
PMID: 23429739 [PubMed - indexed for MEDLINE]

58. Eur J Surg Oncol. 2013 Aug;39(8):823-30. doi: 10.1016/j.ejso.2013.01.005. Epub 2013 Jan 31.

A reliable risk score for stage IV esophagogastric cancer.

Blank S(1), Lordick F, Dobritz M, Grenacher L, Burian M, Langer R, Roth W, Schaible A, Becker K, Bläker H, Sisic L, Stange A, Compani P, Schulze-Bergkamen H, Jäger D, Büchler M, Siewert JR, Ott K.

Author information:

(1)Department of Surgery, University of Heidelberg, Im Neuenheimer Feld 110, 69120 Heidelberg, Germany.

BACKGROUND: The role of surgery for patients with metastatic esophagogastric adenocarcinoma (EGC) is not defined. The purpose of this study was to define selection criteria for patients who may benefit from resection following systemic chemotherapy.
METHODS: From 1987 to 2007, 160 patients presenting with synchronous metastatic EGC (cT3/4 cNany cM0/1 finally pM1) were treated with chemotherapy followed by resection of the primary tumor and metastases. Clinical and histopathological data, site and number of metastases were analyzed. A prognostic score was established and validated in a second cohort from another academic center (n = 32).
RESULTS: The median survival (MS) in cohort 1 was 13.6 months. Significant prognostic factors were grading (p = 0.046), ypT- (p = 0.001), ypN- (p = 0.011) and R-category (p = 0.015), lymphangiosis (p = 0.021), clinical (p = 0.004) and histopathological response (p = 0.006), but not localization or number of metastases. The addition of grading (G1/2:0 points; G3/4:1 points), clinical response (responder: 0; nonresponder: 1) and R-category (complete:0; R1:1; R2:2) defines two groups of patients with significantly different survival (p = 0.001) [low risk group (Score 0/1), n = 22: MS 35.3 months, 3-year-survival 47.6%); high risk group (Score 2/3/4) n = 126: MS 12.0 months, 3-year-survival 14.2%]. The score showed a strong trend in the validation cohort (p = 0.063) [low risk group (MS not reached, 3-year-survival 57.1%); high risk group (MS 19.9 months, 3-year-survival 6.7%)].
CONCLUSION: We observed long-term survival after resection of metastatic EGC. A simple clinical score may help to identify a subgroup of patients with a high chance of benefit from resection. However, the accurate estimation of achieving a complete resection, which is an integral element of the score, remains challenging.

Copyright © 2013 Elsevier Ltd. All rights reserved.

PMID: 23375470 [PubMed - indexed for MEDLINE]

59. Cancer Chemother Pharmacol. 2013 Apr;71(4):921-8. doi:
10.1007/s00280-013-2083-3.
Epub 2013 Jan 25.

Correlation between expressions of ERCC1/TS mRNA and effects of gastric cancer to chemotherapy in the short term.

Chen L(1), Li G, Li J, Fan C, Xu J, Wu B, Liu K, Zhang C.

Author information:
(1)Department of Surgery, Jinling Hospital, Medical School of Nanjing University, Nanjing, 210002 Jiangsu, China.

PURPOSE: To study the correlation between expression levels of ERCC1/TS mRNA and the susceptibility of preoperative chemotherapy for patients with gastric cancer.
METHODS: A total of forty cases with advanced gastric cancer of T3-4N1-2M0 were treated with preoperative chemotherapy according to FLEEOX regimen based on endarterial-intravenous coadministration. Sufficient, fresh gastric tissue specimens were obtained with the help of gastroscope, and the expression levels of ERCC1/TS mRNA were detected by qRT-PCR before chemotherapy. The chemotherapeutic response was evaluated with Choi Criteria after chemotherapy, and pathologic remission extent was observed after surgery. The correlation between the expression levels of ERCC1/TS mRNA before chemotherapy and the chemotherapeutic effect based on imageology and pathology was analyzed.
RESULTS: The response rate of Chemotherapy in this cohort was 80.0 % based on imageology and 51.43 % based on pathology. The expression levels of ERCC1/TS mRNA were significantly associated with imageology remission extent (P = 0.033, P = 0.025) and pathologic remission extent (P = 0.044, P = 0.016), respectively. The chemotherapeutic effect on patients with low-expression levels of ERCC1/TS mRNA was better.
CONCLUSIONS: From the perspective of pathology and imageology evaluating the preoperative chemotherapeutic response for patients with gastric cancer, ERCC1 and TS were used as the molecular predictors and provided prognostic information in this study.

PMID: 23355039 [PubMed - indexed for MEDLINE]

60. Hepatogastroenterology. 2013 May;60(123):638-40.

Histological complete response after neoadjuvant XELOX in advanced gastric carcinoma.

Zhao Q(1), Li Y, Tian Y, Chen YN, Tan BB, Zhao XF, Jiao ZK, Zhang ZD, Chang SL.

Author information:
(1)Department of Surgery, Fourth Hospital of Hebei Medical University, Shijiazhuang, China.

We report on a case of a 65-year-old Chinese male with locally advanced gastric adenocarcinoma achieving pathological complete response after neoadjuvant chemotherapy with capecitabine and oxaliplatin (XELOX) regimen. He underwent esophagogastroduodenoscopy, which revealed a 6x5cm gastric ulcer. Biopsy of gastric ulcer revealed adenocarcinoma. Further workups with abdominal enhancement computed tomography (CT) staged his cancer as T4N2M0. He received 2 cycles of neoadjuvant chemotherapy with XELOX without severe toxicity. Afterwards, he underwent curative surgery consisting of total gastrectomy with extended D2 lymph node dissections and a Roux-en-Y esophagojejunostomy. On microscopic examination, no tumor cells were detected in the ulcer scar of the resected stomach and in the regional lymph nodes. The benefit of XELOX regimen as neoadjuvant chemotherapy in gastric cancer is worth further investigation.

PMID: 23340232 [PubMed - indexed for MEDLINE]

61. World J Gastroenterol. 2012 Dec 28;18(48):7384-93. doi: 10.3748/wjg.v18.i48.7384.

Effectiveness of 5-flurouracil-based neoadjuvant chemotherapy in locally-advanced gastric/gastroesophageal cancer: a meta-analysis.

Ge L(1), Wang HJ, Yin D, Lei C, Zhu JF, Cai XH, Zhang GQ.

Author information:
(1)Department of Gastrointestinal Surgery, Tumor Hospital, Xinjiang Medical University, Urumqi 830011, Xinjiang Uyghur Autonomous Region, China.

AIM: To investigate the effectiveness of 5-flurouracil-based neoadjuvant

chemotherapy (NAC) for gastroesophageal and gastric cancer by meta-analysis.
METHODS: MEDLINE and manual searches were performed to identify all published randomized controlled trials (RCTs) investigating the efficacy of the flurouracil-based NAC for gastroesophageal and gastric cancer, and RCTs of NAC for advanced gastroesophageal and gastric cancer vs no therapy before surgery. Studies that included patients with metastases at enrollment were excluded. Primary endpoint was the odds ratio (OR) for improving overall survival rate of patients with gastroesophageal and gastric cancer. Secondary endpoints were the OR of efficiency for down-staging tumor and increasing R0 resection in patients with gastroesophageal and gastric cancer. Safety analyses were also performed. The OR was the principal measurement of effect, which was calculated as the treatment group (NAC plus surgery) vs control group (surgery alone) and was presented as a point estimate with 95% confidence intervals (CI). All calculations and statistical tests were performed using RevMan 5.1 software.
RESULTS: Seven RCTs were included for the analysis. A total of 1249 patients with advanced gastroesophageal and gastric cancer enrolled in the seven trials were divided into treatment group (n = 620) and control group (n = 629). The quality scores of the RCTs were assessed according to the method of Jadad. The RCT quality scores ranged from 2 to 7 (5-point scale), with a mean of 3.75. The median follow-up time in these studies was over 3 years. The meta-analysis showed that NAC improved the overall survival rate (OR 1.40, 95%CI 1.11-1.76; P = 0.005), which was statistically significant. The 3-year progression-free survival rate was significantly higher in treatment group than in control group (37.7% vs 27.3%) (OR 1.62, 95%CI 1.21-2.15; P = 0.001). The tumor down-stage rate was higher in treatment group than in control group (55.76% vs 41.38%) (OR 1.77, 95%CI 1.27-2.49; P = 0.0009) and the R0 resection rate of the gastroesophageal and gastric cancer was higher in treatment group than in control group (75.11% vs 68.56%) (OR 1.38, 95%CI 1.03-1.85; P = 0.03), with significant differences. No obvious safety concerns about mortality and complications were raised in these trials. There were no statistically significant differences in perioperative mortality (5.08% vs 4.86%) (OR 1.05, 95%CI 0.57-1.94; P = 0.87 fixed-effect model) and in the complication rate between the two groups (13.25% vs 9.66%) (OR 1.40, 95%CI 0.91-2.14; P = 0.12 fixed-effect model). Trials showed that patients from Western countries favored NAC compared with those from Asian countries (OR 1.40, 95%CI 1.07-1.83). Monotherapy was inferior to multiple chemotherapy (OR 1.40, 95%CI 1.07-1.83). Intravenous administration of NAC was more advantageous than oral route (OR 1.41, 95%CI 1.09-1.81).
CONCLUSION: Flurouracil-based NAC can safely improve overall survival rate of patients with gastroesophageal/gastric cancer. Additionally, NAC can down the

tumor stage and improve R0 resection.

PMCID: PMC3544046
PMID: 23326149 [PubMed - indexed for MEDLINE]

62. Gastrointest Cancer Res. 2012 Nov;5(6):191-7.

Phase II Trial of Paclitaxel/Cisplatin Followed by Surgery and Adjuvant Radiation Therapy and 5-Fluorouracil/Leucovorin for Gastric Cancer (ECOG E7296).

Chakravarthy AB(1), Catalano PJ, Mondschein JK, Rosenthal DI, Haller DG, Whittington R, Spitz FR, Wagner H, Sigurdson ER, Tschetter LK, Bayer GK, Mulcahy MF, Benson AB.

Author information:
(1)Vanderbilt-Ingram Cancer Center Nashville, TN.

BACKGROUND: Randomized trials have shown an increase in survival with perioperative chemotherapy as well as with postoperative chemoradiation. It was hypothesized that combining induction chemotherapy with postoperative chemoradiation would be well tolerated and improve pathologic complete response.
METHODS: Patients with resectable cancers of the stomach/gastroesophageal junction were eligible. Neoadjuvant chemotherapy consisted of 3 cycles of paclitaxel and cisplatin. Adjuvant therapy consisted of 1 cycle of 5-fluorouracil (FU) and leucovorin (LV) followed by chemoradiation (45 Gy with concurrent 5-FU/LV). Chemoradiation was followed by 2 additional cycles of 5-FU/LV. Response to neoadjuvant therapy was based on pathology.
RESULTS: From 1999 to 2002, 38 eligible patients were enrolled; 35 completed induction chemotherapy, and 29 went on to surgery. Sixteen patients did not develop metastatic progression, 10 developed metastatic disease, and 12 were unevaluable. There were no pathologic complete responses after induction therapy. Twenty-five of 38 patients suffered grade 3-4 toxicities during induction paclitaxel/cisplatin. Six of the 7 patients who received postoperative therapy suffered grade 3-4 toxicities. Only 3 of 38 (7.9%) eligible patients completed all assigned treatment. The median overall survival was 1.6 years, and the 2-year survival was 40%.
CONCLUSIONS: This regimen of neoadjuvant paclitaxel/cisplatin followed by postoperative 5-FU/LV-based chemoradiation did not have a high enough response

rate and proved to be too toxic for further development.

PMCID: PMC3533847
PMID: 23293700 [PubMed]

63. Radiat Oncol. 2013 Jan 4;8:6. doi: 10.1186/1748-717X-8-6.

Preoperative chemoradiotherapy for locally advanced gastric cancer.

Pepek JM(1), Chino JP, Willett CG, Palta M, Blazer Iii DG, Tyler DS, Uronis HE, Czito BG.

Author information:
(1)Department of Radiation Oncology, Duke University School of Medicine, Box 3085, Durham, NC 27710, USA.

BACKGROUND: To examine toxicity and outcomes for patients treated with preoperative chemoradiotherapy (CRT) for gastric cancer.
METHODS: Patients with gastroesophageal (GE) junction (Siewert type II and III) or gastric adenocarcinoma who underwent neoadjuvant CRT followed by planned surgical resection at Duke University between 1987 and 2009 were reviewed. Overall survival (OS), local control (LC) and disease-free survival (DFS) were estimated using the Kaplan-Meier method. Toxicity was graded according to the Common Toxicity Criteria for Adverse Events version 4.0.
RESULTS: Forty-eight patients were included. Most (73%) had proximal (GE junction, cardia and fundus) tumors. Median radiation therapy dose was 45 Gy. All patients received concurrent chemotherapy. Thirty-six patients (75%) underwent surgery. Pathologic complete response and R0 resection rates were 19% and 86%, respectively. Thirty-day surgical mortality was 6%. At 42 months median follow-up, 3-year actuarial OS was 40%. For patients undergoing surgery, 3-year OS, LC and DFS were 50%, 73% and 41%, respectively.
CONCLUSIONS: Preoperative CRT for gastric cancer is well tolerated with acceptable rates of perioperative morbidity and mortality. In this patient cohort with primarily advanced disease, OS, LC and DFS rates in resected patients are comparable to similarly staged, adjuvantly treated patients in randomized trials. Further study comparing neoadjuvant CRT to standard treatment approaches for gastric cancer is indicated.

PMCID: PMC3552754
PMID: 23286735 [PubMed - indexed for MEDLINE]

64. Med Oncol. 2013 Mar;30(1):377. doi: 10.1007/s12032-012-0377-7. Epub 2012 Dec 29.

Perioperative DCF chemotherapy protocol for patients with gastroesophageal adenocarcinoma: correlation between response to treatment and outcome.

Alcindor T(1), Ferri LE, Marcus V, Andalib A, Hickeson M, Artho G, Chasen M, Thirlwell MP, Ades S.

Author information:
(1)Division of Medical Oncology, McGill University Health Centre, 1650 Cedar Avenue, suite A7-130, Montreal H3G 1A4, QC, Canada. thierry.alcindor@mcgill.ca

To determine whether metabolic or pathological response to preoperative chemotherapy can predict the relapse-free survival of gastroesophageal adenocarcinoma patients treated on a perioperative chemotherapy protocol. The prospectively collected data of a recently reported phase II trial of perioperative DCF chemotherapy (docetaxel/cisplatin/5-fluorouracil) were analyzed. Median relapse-free survival (RFS) was compared with the Wilcoxon rank-sum test between responders and non-responders according to defined metabolic (reduction in maximum standard uptake value of at least 35 %) and pathological (greater than 50 % tumor regression or ypN(0) status) criteria. A double-sided p value equal or inferior to 0.05 was considered significant. Patients were followed for a median of 807 days (95 % CI: 607-896). RFS was 576 days in metabolic non-responders versus not reached in metabolic responders (p 0.009) and 562 days in ypN+ versus not reached in ypN(0) patients (p 0.045). No statistically significant RFS difference was seen between low and high pathologic responders classified according to tumor regression criteria, although a trend was observed in favor of high pathologic responders. Simple metabolic and pathologic criteria used for the assessment of response to the preoperative part of perioperative chemotherapy can help to estimate the outcome of gastroesophageal adenocarcinoma patients.

PMID: 23275118 [PubMed - indexed for MEDLINE]

65. Langenbecks Arch Surg. 2013 Feb;398(2):239-49. doi: 10.1007/s00423-012-1039-0. Epub 2012 Dec 27.

Factors predicting prognosis and recurrence in patients with esophago-gastric adenocarcinoma and histopathological response with less than 10 % residual tumor.

Ott K(1), Blank S, Becker K, Langer R, Weichert W, Roth W, Sisic L, Stange A, Jäger D, Büchler M, Siewert JR, Lordick F.

Author information:
(1)Department of Surgery, University Hospital of Heidelberg, Heidelberg, Germany. katja.ott@med.uni-heidelberg.de

PURPOSE: Neoadjuvant treatment is an accepted standard approach for treating locally advanced esophago-gastric adenocarcinomas. Despite a response of the primary tumor, a significant percentage dies from tumor recurrence. The aim of this retrospective exploratory study from two academic centers was to identify predictors of survival and recurrence in histopathologically responding patients.
METHODS: Two hundred thirty one patients with adenocarcinomas (esophagus: n = 185, stomach: n = 46, cT3/4, cN0/+, cM0) treated with preoperative chemotherapy (n = 212) or chemoradiotherapy (n = 19) followed by resection achieved a histopathological response (regression 1a: no residual tumor (n = 58), and regression 1b < 10 % residual tumor (n = 173)).
RESULTS: The estimated median overall survival was 92.4 months (5-year survival, 56.6 %) for all patients. For patients with regression 1a, median survival is not reached (5-year survival, 71.6 %) compared to patients with regression 1b with 75.3 months median (5-year survival, 52.2 %) (p = 0.031). Patients with a regression 1a had lymph node metastases in 19.0 versus 33.7 % in regression 1b. The ypT-category (p < 0.001), the M-category (p = 0.005), and the type of treatment (p = 0.04) were found to be independent prognostic factors in R0-resected patients. The recurrence rate was 31.7 % (n = 66) (local, 39.4 %; peritoneal carcinomatosis, 25.7 %; distant metastases, 50 %). Recurrence was predicted by female gender (p = 0.013), ypT-category (p = 0.007), and M-category (p = 0.003) in multivariate analysis.
CONCLUSION: Response of the primary tumor does not guarantee recurrence-free long-term survival, but histopathological complete responders have better prognosis compared to partial responders. Established prognostic factors strongly influence the outcome, which could, in the future, be used for stratification of

adjuvant treatment approaches. Increasing the rate of histopathological complete responders is a valid endpoint for future clinical trials investigating new drugs.

PMID: 23269519 [PubMed - indexed for MEDLINE]

66. Ann Oncol. 2013 May;24(5):1262-6. doi: 10.1093/annonc/mds617. Epub 2012 Dec 17.

Association between clinical complete response and pathological complete response after preoperative chemoradiation in patients with gastroesophageal cancer: analysis in a large cohort.

Cheedella NK(1), Suzuki A, Xiao L, Hofstetter WL, Maru DM, Taketa T, Sudo K, Blum MA, Lin SH, Welch J, Lee JH, Bhutani MS, Rice DC, Vaporciyan AA, Swisher SG, Ajani JA.

Author information:
(1)Department of Gastrointestinal Medical Oncology, Unit 426, The University of Texas MD Anderson Cancer Center, 1515 Holcombe Boulevard, Houston, TX 77030, USA.

BACKGROUND: Chemoradiation followed by surgery is the preferred treatment of localized gastroesophageal cancer (GEC). Surgery causes considerable life-altering consequences and achievement of clinical complete response (clinCR; defined as postchemoradiation [but presurgery] endoscopic biopsy negative for cancer and positron emission tomographic (PET) scan showing physiologic uptake) is an enticement to avoid/delay surgery. We examined the association between clinCR and pathologic complete response (pathCR).
PATIENTS AND METHODS: Two hundred eighty-four patients with GEC underwent chemoradiation and esophagectomy. The chi-square test, Fisher exact test, t-test, Kaplan-Meier method, and log-rank test were used.
RESULTS: Of 284 patients, 218 (77%) achieved clinCR. However, only 67 (31%) of the 218 achieved pathCR. The sensitivity of clinCR for pathCR was 97.1% (67/69), but the specificity was low (29.8%; 64/215). Of the 66 patients who had less than a clinCR, only 2 (3%) had a pathCR. Thus, the rate of pathCR was significantly different in patients with clinCR than in those with less than a clinCR ($P < 0.001$).
CONCLUSIONS: clinCR is not highly associated with pathCR; the specificity of clinCR for pathCR is too low to be used for clinical decision making on

delaying/avoiding surgery. Surgery-eligible GEC patients should be encouraged to undergo surgery following chemoradiation despite achieving a clinCR.

PMCID: PMC3629896
PMID: 23247658 [PubMed - indexed for MEDLINE]

67. Recent Results Cancer Res. 2012;196:187-99. doi: 10.1007/978-3-642-31629-6_13.

Radiotherapy of gastroesophageal junction cancer.

Sterzing F(1), Grenacher L, Debus J.

Author information:
(1)Department of Radiation Oncology, INF 400, 69120, Heidelberg, Germany.
florian.sterzing@med.uni-heidelberg.de

Adenocarcinomas of the gastroesophageal junction (GEJ) require multimodal treatment approaches to accomplish good local control and overall survival. While early T1/2 N0 tumors are treated with surgery alone, they are only found in a small subset of patients due to the lack of symptoms at this stage. Most of the tumors are detected in locally advanced stage where surgery alone results in disappointing outcome. Chemotherapy and/or chemoirradiation in the neoadjuvant setting are used to improve conditions for oncological surgery. They aim to achieve a downsizing with a pathological complete remission in the optimal case, improve R0 rates, and upfront treat microscopic metastatic tumor cells. The optimal neoadjuvant treatment approach-chemotherapy, chemoirradiation, or a multiphase approach of both-is yet unclear. Chemoirradiation can improve local control after incomplete surgery and is an important option for patients unfit for surgery. In addition, it enables symptom relief in a palliative setting, namely dysphagia, pain, or bleeding. While target volumes are very much standardized, new technologies as image-guided intensity-modulated radiotherapy (IG-IMRT) and particle therapy have the potential to improve the therapeutic window by minimizing toxicity. Challenges of the present and the future will be the combination of radiotherapy with other cytostatic drugs and modern targeted therapies. This should ideally be integrated into a multimodal setting that is able to identify risk groups according to predictive markers and tumor response, altogether leading to a personalized oncological approach.

PMID: 23129375 [PubMed - indexed for MEDLINE]

68. World J Surg Oncol. 2012 Oct 29;10:225. doi: 10.1186/1477-7819-10-225.

Decreased expression of microRNA let-7i and its association with chemotherapeutic response in human gastric cancer.

Liu K(1), Qian T, Tang L, Wang J, Yang H, Ren J.

Author information:
(1)Department of General Surgery, Changzhou No, 2 Hospital, Nanjing Medical University, Nanjing, China.

BACKGROUND: MicroRNA let-7i has been proven to be down-regulated in many human malignancies and correlated with tumor progression and anticancer drug resistance. Our study aims to characterize the contribution of miRNA let-7i to the initiation and malignant progression of locally advanced gastric cancer (LAGC), and evaluate its possible value in neoadjuvant chemotherapeutic efficacy prediction.
METHODS: Eighty-six previously untreated LAGC patients who underwent preoperative chemotherapy and radical resection were included in our study. Let-7i expression was examined for pairs of cancer tissues and corresponding normal adjacent tissues (NATs), using quantitative RT-PCR. The relationship of let-7i level to clinicopathological characteristics, pathologic tumor regression grades after chemotherapy, and overall survival (OS) was also investigated.
RESULTS: Let-7i was significantly down-regulated in most tumor tissues (78/86: 91%) compared with paired NATs ($P < 0.001$), and low levels of let-7i were significantly correlated with local invasion, lymphatic metastasis, and poor pathologic tumor response. Multivariate Cox regression analysis revealed that low let-7i expression was an unfavorable prognostic factor of OS (hazard ratio (HR) = 2.316, $P = 0.024$) independently of other clinicopathological factors, including tumor node metastasis (TNM) stage (HR = 3.226, $P = 0.013$), depth of infiltration (HR = 4.167, $P < 0.001$), and lymph node status (HR = 2.245, $P = 0.037$).
CONCLUSIONS: These findings indicate that let-7i may be a good candidate for use a therapeutic target and a potential tissue marker for the prediction of chemotherapeutic sensitivity and prognosis in LAGC patients.

PMCID: PMC3500711

PMID: 23107361 [PubMed - indexed for MEDLINE]

69. J Gastrointest Cancer. 2013 Jun;44(2):162-9. doi: 10.1007/s12029-012-9449-3.

Docetaxel-based preoperative chemoradiation in localized gastric cancer: impact of pathological complete response on patient outcome.

Rostom Y(1), Zaghloul H, Khedr G, El-Shazly W, Abd-Allah D.

Author information:
(1)Clinical Oncology Department, Faculty of Medicine, Alexandria University, Champlion Street, Alazarita, Alexandria 21131, Egypt.

PURPOSE: This study was conducted to evaluate the feasibility, efficacy, and toxicities of docetaxel-based induction chemotherapy and chemoradiotherapy in patients with localized gastric or gastroesophageal adenocarcinoma.
METHODS: Patients with localized, operable gastric or gastroesophageal adenocarcinoma received two cycles of induction chemotherapy of fluorouracil, docetaxel, and cisplatin (TPF) followed by 45 Gy of radiation and concurrent fluorouracil plus docetaxel then surgery for nonmetastatic patients.
RESULTS: Forty-one patients were included. Pretreatment T3 was encountered in 56 % of patients while 61 % had N1 disease. A pathologic complete response (CR) was noted in 24 % of patients. Pathologic response was significantly associated with baseline T stage (P < 0.001) and N stage (P = 0.002). The 3-year overall survival (OS) and disease-free survival were 47.3 and 42.1 %, respectively. OS was significantly correlated with R0 resection (P = 0.027), pathological response (P = 0.01), dissected pathologically positive lymph node (P = 0.037), and postsurgery (T) stage (P = 0.02). Toxicities were manageable and there were no treatment-related deaths.
CONCLUSION: Docetaxel-based chemoradiotherapy in localized gastric adenocarcinoma patients resulted in 24 % path CR and was not associated with a higher percentage of postoperative complications. A well-designed randomized controlled trial is mandatory to further endorse this evolving approach.

PMID: 23104208 [PubMed - indexed for MEDLINE]

70. J Chemother. 2012 Aug;24(4):217-20. doi: 10.1179/1973947812Y.0000000020.

The efficacy and toxicity of irinotecan with leucovorin and bolus and continuous infusional 5-fluorouracil (FOLFIRI) as salvage therapy for patients with advanced gastric cancer previously treated with platinum and taxane-based chemotherapy regimens.

Kaya AO(1), Coskun U, Gumus M, Dane F, Ozkan M, Isıkdogan A, Alkis N, Buyukberber S, Yumuk F, Budakoglu B, Demirci U, Berk V, Bilici A, Inal A, Arpacı E, Benekli M; Anatolian Society of Medical Oncology (ASMO).

Author information:
(1)Bakırkoy Dr Sadl Konuk Training and Research Hospital, Department of Medical Oncology, Istanbul, Turkey. aosmankaya@gmail.com

There is no established standard salvage chemotherapy in the second-line setting for patients with advanced gastric cancer (AGC) pre-treated with platinum and taxane-based chemotherapy. Our study aims to evaluate the safety and efficacy of FOLFIRI regimen (irinotecan with leucovorin and bolus and continuous infusion with 5-fluorouracil) as a salvage chemotherapy regimen in patients with AGC. Medical records of 97 patients with AGC who received second-line FOLFIRI regimen between March 2006 and February 2011 were examined. Complete and partial responses were observed in 3 (3.1%) and 23 (23.7%) patients, respectively. The median time to progression (TTP) was 3.5 months (95% CI: 2.4-4.6) and the median overall survival (OS) was 10.5 months (95% CI: 8.8-12.2). The most common observed grade 3/4 toxicities were neutropenia (23.7%), diarrhea (6.2%), and stomatitis (5.2%). FOLFIRI regimen is safe and effective in the second-line treatment of AGC patients pre-treated with cisplatin and taxanes.

PMID: 23040686 [PubMed - indexed for MEDLINE]

71. PLoS One. 2012;7(9):e44566. doi: 10.1371/journal.pone.0044566. Epub 2012 Sep 10.

Expression profiling of stem cell-related genes in neoadjuvant-treated gastric cancer: a NOTCH2, GSK3B and β-catenin gene signature predicts survival.

Bauer L(1), Langer R, Becker K, Hapfelmeier A, Ott K, Novotny A, Höfler H, Keller G.

Author information:
(1)Institute of Pathology, Technische Universität München, München, Germany.

Cancer stem cell (CSC) based gene expression signatures are associated with prognosis in various tumour types and CSCs are suggested to be particularly drug resistant. The aim of our study was first, to determine the prognostic significance of CSC-related gene expression in residual tumour cells of neoadjuvant-treated gastric cancer (GC) patients. Second, we wished to examine, whether expression alterations between pre- and post-therapeutic tumour samples exist, consistent with an enrichment of drug resistant tumour cells. The expression of 44 genes was analysed in 63 formalin-fixed, paraffin embedded tumour specimens with partial tumour regression (10-50% residual tumour) after neoadjuvant chemotherapy by quantitative real time PCR low-density arrays. A signature of combined GSK3B(high), β-catenin (CTNNB1)(high) and NOTCH2(low) expression was strongly correlated with better patient survival ($p<0.001$). A prognostic relevance of these genes was also found analysing publically available gene expression data. The expression of 9 genes was compared between pre-therapeutic biopsies and post-therapeutic resected specimens. A significant post-therapeutic increase in NOTCH2, LGR5 and POU5F1 expression was found in tumours with different tumour regression grades. No significant alterations were observed for GSK3B and CTNNB1. Immunohistochemical analysis demonstrated a chemotherapy-associated increase in the intensity of NOTCH2 staining, but not in the percentage of NOTCH2. Taken together, the GSK3B, CTNNB1 and NOTCH2 expression signature is a novel, promising prognostic parameter for GC. The results of the differential expression analysis indicate a prominent role for NOTCH2 and chemotherapy resistance in GC, which seems to be related to an effect of the drugs on NOTCH2 expression rather than to an enrichment of NOTCH2 expressing tumour cells.

PMCID: PMC3438181
PMID: 22970250 [PubMed - indexed for MEDLINE]

72. Oncology. 2012;83(5):300-4. doi: 10.1159/000341353. Epub 2012 Sep 4.

Outcome of trimodality-eligible esophagogastric cancer patients who declined surgery after preoperative chemoradiation.

Taketa T(1), Correa AM, Suzuki A, Blum MA, Chien P, Lee JH, Welsh J, Lin SH, Maru DM, Erasmus JJ, Bhutani MS, Weston B, Rice DC, Vaporciyan AA, Hofstetter WL, Swisher SG, Ajani JA.

Author information:
(1)Department of Gastrointestinal Medical Oncology, The University of Texas MD Anderson Cancer Center, Houston, Texas 77030, USA.

BACKGROUND: For patients with localized esophageal cancer (EC) who can withstand surgery, the preferred therapy is chemoradiation followed by surgery (trimodality). However, after achieving a clinical complete response [clinCR; defined as both post-chemoradiation endoscopic biopsy showing no cancer and physiologic uptake by positron emission tomography (PET)], some patients decline surgery. The literature on the outcome of such patients is sparse.
METHOD: Between 2002 and 2011, we identified 622 trimodality-eligible EC patients in our prospectively maintained databases. All patients had to be trimodality eligible and must have completed preoperative staging after chemoradiation that included repeat endoscopic biopsy and PET among other routine tests.
RESULTS: Out of 622 trimodality-eligible patients identified, 61 patients (9.8%) declined surgery. All 61 patients had a clinCR. The median age was 69 years (range 47-85). Males (85.2%) and Caucasians (88.5%) were dominant. Baseline stage was II (44.2%) or III (52.5%), and histology was adenocarcinoma (65.6%) or squamous cell carcinoma (29.5%). Forty-two patients are alive at a median follow-up of 50.9 months (95% CI 39.5-62.3). The 5-year overall and relapse-free survival rates were 58.1 ± 8.4 and 35.3 ± 7.6%, respectively. Of 13 patients with local recurrence during surveillance, 12 had successful salvage resection.
CONCLUSION: Although the outcome of 61 EC patients with clinCR who declined surgery appears reasonable, in the absence of a validated prediction/prognosis model, surgery must be encouraged for all trimodality-eligible patients.

Copyright © 2012 S. Karger AG, Basel.

PMCID: PMC3832345
PMID: 22964903 [PubMed - indexed for MEDLINE]

73. Zhonghua Yi Xue Za Zhi. 2012 Jun 26;92(24):1677-80.

[Phase II clinical trial of docetaxel, platinum and S-1 for advanced gastric

cancer].

[Article in Chinese]

Zhou AP(1), Li YS, Yang L, Song Y, Sun YK, Zhang W, Cui CX, Chi Y, Yuan XH, Lü N, Wang JW.

Author information:
(1)Cancer Hospital, Peking Union Medical College & Chinese Academy of Medical Sciences, Beijing 100021, China.

OBJECTIVE: To evaluate the efficacy and safety profile and to explore the role of docetaxel, S-1 plus cisplatin (DCS) or oxaliplatin (DOS) in the treatment of advanced gastric cancer.
METHODS: A total of 45 patients with advanced gastric cancer were recruited. They received DCS or DOS at the discretion of investigators. Docetaxel was given intravenously at the dose of 60 mg/m^2 at d1, S-1 60 $mg \times m^{-2} \times d^{-1}$ or 80 - 120 mg/d according to individual patient's area of body surface orally from d1 to d14 and cisplatin 30 mg/m^2 at d1, d2 or oxaliplatin 111 - 127 (median: 117) mg/m^2 at d2. Each cycle was for 21 days.
RESULTS: Forty-three patients received ≥ 1 complete cycle of DCS/DOS with a median cycle number of 5(range: 1 - 8). Among 42 patients evaluated for efficacy, the outcomes were partial response (n = 28), stable disease (n = 9) and progression (n = 5). The response rate was 66.7%. Progression-free survival (PFS) of 32 patients on chemotherapy alone was 7.1 months and the median overall survival (OS) was not reached. The most common grade 3/4 adverse effects included neutropenia (46.5%), thrombocytopenia (9.3%), vomiting (9.3%), nausea (7.0%) and diarrhea (4.7%). Ten of fourteen patients with advanced unresectable gastric cancer without clinically detectable distant metastases underwent surgical resection after a median of 4 (2-6) cycles of DCS or DOS and 9 (64.3%) had R0 resection.
CONCLUSIONS: DCS/DOS is effective for advanced gastric cancer and in the setting of neoadjuvant chemotherapy. And the toxicities of DCS/DOS are manageable.

PMID: 22944157 [PubMed - indexed for MEDLINE]

74. Int J Surg Oncol. 2012;2012:148420. doi: 10.1155/2012/148420. Epub 2012 Jul 31.

Effects of neoadjuvant intraperitoneal/systemic chemotherapy (bidirectional chemotherapy) for the treatment of patients with peritoneal metastasis from gastric cancer.

Yonemura Y(1), Elnemr A, Endou Y, Ishibashi H, Mizumoto A, Miura M, Li Y.

Author information:
(1)NPO Organization to Support Peritoneal Surface Malignancy Treatment, Osaka, Kishiwada 596-0032, Japan.

Novel multidisciplinary treatment combined with neoadjuvant intraperitoneal-systemic chemotherapy protocol (NIPS) and peritonectomy was developed. Ninety-six patients were enrolled. Peritoneal wash cytology was performed before and after NIPS through a port system. Patients were treated with 60 mg/m(2) of oral S-1 for 21 days, followed by a 1-week rest. On days 1, 8, and 15, 30 mg/m(2) of Taxotere and 30 mg/m(2) of cisplatin with 500 mL of saline were introduced through the port. NIPS is done 2 cycles before surgery. Three weeks after NIPS, 82 patients were eligible to intend cytoreductive surgery (CRS) by gastrectomy + D2 dissection + periotnectomy to achieve complete cytoreduction. Sixty-eight patients showed positice cytology before NIPS, and the positive cytology results became negative in 47 (69%) patients after NIPS. Complete pathologic response on PC after NIPS was experienced in 30 (36.8%) patients. Stage migration was experienced in 12 patients (14.6%). Complete cytoreduction was achieved in 58 patients (70.7%). By the multivariate analysis, complete cytoreduction and pathologic response became a significantly good survival. However the high morbidity and mortality, stringent patient selection is important. The best indications of the therapy are patients with good pathologic response and PCI ≤ 6, which are supposed to be removed completely by peritonectomy.

PMCID: PMC3415092
PMID: 22900159 [PubMed]

75. Clin Adv Hematol Oncol. 2012 Jul;10(7):430-7.

A phase II trial of preoperative concurrent chemotherapy/radiation therapy plus bevacizumab/erlotinib in the treatment of localized esophageal cancer.

Bendell JC(1), Meluch A, Peyton J, Rubin M, Waterhouse D, Webb C, Burris HA 3rd, Hainsworth JD.

Author information:
(1)Sarah Cannon Research Institute, Nashville, Tennessee 37203, USA.
jbendell@tnonc.com

PURPOSE: To evaluate the efficacy of bevacizumab (Avastin, Genentech) and erlotinib (Tarceva, Genentech/Roche) when added to preoperative chemoradiation therapy with paclitaxel, carboplatin, and infusional 5-fluorouracil (5-FU) in the treatment of localized cancers of the esophagus or gastroesophageal (GE) junction. The primary endpoint was the pathologic complete response (pCR) rate.
METHODS: Eligible patients had previously untreated localized squamous cell, adenocarcinoma, or adenosquamous carcinoma of the esophagus or GE junction, and were considered surgical candidates at enrollment. Daily erlotinib (100 mg orally) was administered on days 1-42 of preoperative treatment. Patients received paclitaxel (200 mg/m2 intravenously [IV]), carboplatin (area under the curve [AUC] 5.0 IV), and bevacizumab (15 mg/kg IV) on days 1 and 22, and 5-FU by continuous infusion (225 mg/m2/day IV) on days 1-35, with radiation therapy in 1.8-Gy single fractions, Monday-Friday (to a total of 45 Gy). Those who were deemed surgical candidates proceeded to resection during weeks 12-14.
RESULTS: Between February 2007 and September 2009, 62 patients (median age, 64 years; 92% male; 94% adenocarcinoma) were enrolled; 44 patients (71%) completed neoadjuvant treatment and proceeded to surgery. Eighteen patients (29%) achieved pCR, with partial pathologic remission in an additional 22 patients (35%). Common grade 3/4 toxicities included leukopenia (64%), neutropenia (44%), mucositis/stomatitis (42%), diarrhea (27%), and esophagitis (27%). There were 40 instances of treatment-related hospitalization, and 2 postoperative deaths.
CONCLUSIONS: The addition of bevacizumab and erlotinib to neoadjuvant chemoradiation did not demonstrate survival benefit or improved pCR rate over similar regimens. While the overall rates of toxicity were not increased, targeted agent-specific toxicity was evident. Further study of this specific regimen is not warranted.

PMID: 22895283 [PubMed - indexed for MEDLINE]

76. Chin Med J (Engl). 2012 Jun;125(12):2144-50.

Efficacy and safety of neoadjuvant chemotherapy with modified FOLFOX7 regimen on the treatment of advanced gastric cancer.

Zhang J(1), Chen RX, Zhang J, Cai J, Meng H, Wu GC, Zhang ZT, Wang Y, Wang KL.

Author information:
(1)Department of General Surgery, Beijing Friendship Hospital, Capital Medical University, Beijing 100050, China.

BACKGROUND: Gastric cancer is one of the most common types of malignant tumors in China and East Asia and has the highest mortality rate of the malignant gastrointestinal tumors. Neoadjuvant chemotherapy is a systemic or local chemotherapy that is given prior to the local treatment of malignant tumors. Neoadjuvant therapy is currently showing some positive prospects; however, its clinical effects remain controversial. In this study, we used the modified FOLFOX7 (mFOLFOX7) regimen as a neoadjuvant chemotherapy regimen. Perioperative clinical and pathological efficacy, toxicity, effects of surgery, postoperative observation, and prognosis were studied to investigate its clinical efficacy and safety.
METHODS: Eighty patients with advanced gastric cancer were treated in our surgery department from 2005 to 2009; 38 of these patients received mFOLFOX7 neoadjuvant chemotherapy, the other 42 patients assigned to the control group. The perioperative effects of mFOLFOX7 chemotherapy, including clinical effects and toxicity, were observed in each patient.
RESULTS: After mFOLFOX7 chemotherapy, clinical and pathologic stages decreased in 21.1% and 36.8% of the patients, respectively, but the results were not statistically significant (P = 0.129). The clinical response rate was 50% (19/38). Toxicity was mild; most adverse events were grade I or II and involved no severe infections or deaths. Compared with the control group, the radical resection rate increased (92.1% vs. 85.7%; P = 0.437); surgical effects were completed without an increased incidence of perioperative complications. The 1-, 2-, and 3-year survival rates were 78.70%, 57.40%, and 51.66%, respectively, in the neoadjuvant chemotherapy group and 78.57%, 56.87%, and 43.16%, respectively, in the control group.
CONCLUSIONS: The mFOLFOX7 regimen was very effective and well-tolerated as a neoadjuvant chemotherapy for advanced gastric cancer. However, the 1-, 2-, and 3-year survival rates in the mFOLFOX7 group were not significantly different from the control group.

PMID: 22884144 [PubMed - indexed for MEDLINE]

77. BMJ Case Rep. 2012 Jul 25;2012. pii: bcr2012006361. doi: 10.1136/bcr-2012-006361.

Small cell carcinoma of gastro-oesophageal junction with remarkable response to chemo-radiotherapy.

Matsunaga M(1), Miwa K, Noguchi T, Sasaki Y.

Author information:
(1)Department of Medical Oncology, International Medical Center, Comprehensive Cancer Center, Saitama Medical University, Hidaka, Japan.
mmandmacgyver@yahoo.co.jp

Small cell carcinoma of the gastrointestinal tract is rare, and no effective strategy has yet been established. On the basis of regimens reportedly effective for small cell lung cancer, we performed chemotherapy with cisplatin plus etoposide in combination with radiotherapy to relieve obstruction, in a patient with small cell carcinoma of the gastro-oesophageal junction. Chemotherapy was switched to carboplatin plus etoposide due to renal toxicity. No distant metastases were detected and lesion spread was limited. A complete response, with no evidence of recurrence to date, was achieved. Curative resection was suggested but refused by the patient. He has been closely followed up in our outpatient clinic for more than a year and has shown no evidence of recurrence since the completion of treatment. Although cisplatin plus etoposide is a standard chemotherapy regimen for small cell carcinoma, carboplatin plus etoposide may be effective in cases in which cisplatin is contraindicated due to renal toxicity.

PMID: 22843758 [PubMed - indexed for MEDLINE]

78. World J Surg Oncol. 2012 Jul 3;10:133. doi: 10.1186/1477-7819-10-133.

A complete response to S-1 plus cis-diamminedichloroplatinum in advanced-stage esophageal and gastric adenocarcinoma: a case report.

Matsuno Y(1), Kochi M, Fujii M, Kanamori N, Kaiga T, Mihara Y, Funada T, Miyazaki

T, Takayama T.

Author information:
(1)Department of Digestive Surgery, Nihon University School of Medicine, Tokyo, Japan. kochi.mitsugu@nihon-u.ac.jp.

BACKGROUND: Complete remission from advanced-stage synchronous double primary (SDP) esophageal and gastric adenocarcinoma by chemotherapy alone is rare. We report a case of advanced-stage SDP esophageal and gastric adenocarcinoma in which a complete response to treatment was obtained with S-1 and cis-diamminedichloroplatinum (CDDP).
CASE PRESENTATION: The patient was a 74-year-old man referred to our hospital complaining of dysphagia. Gastrointestinal endoscopy was performed and advanced-stage SDP esophageal and gastric adenocarcinoma diagnosed. Computed tomography revealed multiple regional lymph node metastases in the mediastinum. Neoadjuvant chemotherapy with S-1 and CDDP for advanced esophageal and gastric cancer was planned. An endoscopy following two courses of chemotherapy revealed that the esophageal cancer had been replaced with a normal mucosal lesion and the gastric tumor with a scar lesion; the results of biopsies of both were negative for cancer. Computed tomography revealed that the multiple lymph node metastases had disappeared. We diagnosed a complete response to S-1 and CDDP in advanced-stage SDP esophageal and gastric cancer. The patient is still alive with no signs of recurrence 22 months after the disappearance of the original tumor and metastatic lesions without surgical treatment.
CONCLUSION: These results suggest that complete remission from advanced-stage esophageal and gastric cancer can be obtained with chemotherapy with S-1 plus CDDP.

PMCID: PMC3438035
PMID: 22759597 [PubMed - indexed for MEDLINE]

79. Zhonghua Wei Chang Wai Ke Za Zhi. 2012 Jun;15(6):546-8.

[Related problems of perioperative radiotherapy for gastric cancer].

[Article in Chinese]

Zhang Z(1).

Author information:
(1)Department of Radiology, Fudan University, Shanghai, China.
zhenzhang6@gmail.com

Radiotherapy has been an important component in the multidisciplinary treatment strategy of gastric cancer. INT0116 has showed the improvement of survival with adjuvant chemoradiation in resected gastric cancer. The benefit of adjuvant chemoradiation has been confirmed further in gastric cancer population. Recent reported ARTIST trial which all patients got D2 dissection has showed the benefit of adjuvant chemoradiation only seen in patients with lymph node positive. As the same observed in other two trail, ACT-GC and CLASSIC, extremely low local recurrence were reported in these trial. We should be very cautious when we interpret these results and treatment into our clinical practice due to the difference of local recurrence between trials and daily practice. Neoadjuvant radiation has been reported its effectiveness of cardiac gastric cancer in earlier randomized trial. Phase II(trials have shown the high pCR rate with neoadjuvant chemoradiation. However, concurrent chemoradiation was with more toxicity and limited it development. Even though, further investigation in neoadjuvant setting is worthy but with reduce of toxicity. New progress in high technique of radiation will help the application of radiotherapy in gastric cancer. The future of radiation in gastric cancer treatment will focus on the selection of patients which are of most benefit, detection of radiosensitivity and how to optimize combination of radiation with surgery and novel chemotherapy and target therapy.

PMID: 22736118 [PubMed - indexed for MEDLINE]

80. N Engl J Med. 2012 May 31;366(22):2074-84. doi: 10.1056/NEJMoa1112088.

Preoperative chemoradiotherapy for esophageal or junctional cancer.

van Hagen P(1), Hulshof MC, van Lanschot JJ, Steyerberg EW, van Berge Henegouwen MI, Wijnhoven BP, Richel DJ, Nieuwenhuijzen GA, Hospers GA, Bonenkamp JJ, Cuesta MA, Blaisse RJ, Busch OR, ten Kate FJ, Creemers GJ, Punt CJ, Plukker JT, Verheul HM, Spillenaar Bilgen EJ, van Dekken H, van der Sangen MJ, Rozema T, Biermann K, Beukema JC, Piet AH, van Rij CM, Reinders JG, Tilanus HW, van der Gaast A; CROSS Group.

Collaborators: Bergman JJ, Bartelsman JF, Bissumbar A, Blom RL, Geijsen ED, van Heijl M, Obertop H, Koning CC, Offerhaus GJ, Omloo JM, Wilmink H, Aparicio Pages MN, van den Nieuwenhof-Biesheuvel L, Eijkenboom WM, Koppert LB, Meijer DA, Siersema PD, Spaander MC, Verheij C, Vollebregt C, van Krieken JH, van Mansum W, van Dam G, van Dullemen HM, Eerens A, van der Jagt E, Karnebeld A, Kluin P, Mul VE, Pruim J, Siemerink E, Weersma RK, Fraikin T, Peters CW.

Author information:
(1)Department of Surgery, Erasmus University Medical Center, Rotterdam, The Netherlands.

Comment in
Strahlenther Onkol. 2013 Feb;189(2):161-2.
N Engl J Med. 2012 Aug 30;367(9):873; author reply 873-4.
N Engl J Med. 2012 Aug 30;367(9):873; author reply 873-4.
N Engl J Med. 2012 Aug 30;367(9):872-3; author reply 873-4.

BACKGROUND: The role of neoadjuvant chemoradiotherapy in the treatment of patients with esophageal or esophagogastric-junction cancer is not well established. We compared chemoradiotherapy followed by surgery with surgery alone in this patient population.
METHODS: We randomly assigned patients with resectable tumors to receive surgery alone or weekly administration of carboplatin (doses titrated to achieve an area under the curve of 2 mg per milliliter per minute) and paclitaxel (50 mg per square meter of body-surface area) for 5 weeks and concurrent radiotherapy (41.4 Gy in 23 fractions, 5 days per week), followed by surgery.
RESULTS: From March 2004 through December 2008, we enrolled 368 patients, 366 of whom were included in the analysis: 275 (75%) had adenocarcinoma, 84 (23%) had squamous-cell carcinoma, and 7 (2%) had large-cell undifferentiated carcinoma. Of the 366 patients, 178 were randomly assigned to chemoradiotherapy followed by surgery, and 188 to surgery alone. The most common major hematologic toxic effects in the chemoradiotherapy-surgery group were leukopenia (6%) and neutropenia (2%); the most common major nonhematologic toxic effects were anorexia (5%) and fatigue (3%). Complete resection with no tumor within 1 mm of the resection margins (R0) was achieved in 92% of patients in the chemoradiotherapy-surgery group versus 69% in the surgery group ($P<0.001$). A pathological complete response was achieved in 47 of 161 patients (29%) who underwent resection after chemoradiotherapy. Postoperative complications were

similar in the two treatment groups, and in-hospital mortality was 4% in both. Median overall survival was 49.4 months in the chemoradiotherapy-surgery group versus 24.0 months in the surgery group. Overall survival was significantly better in the chemoradiotherapy-surgery group (hazard ratio, 0.657; 95% confidence interval, 0.495 to 0.871; P=0.003).
CONCLUSIONS: Preoperative chemoradiotherapy improved survival among patients with potentially curable esophageal or esophagogastric-junction cancer. The regimen was associated with acceptable adverse-event rates. (Funded by the Dutch Cancer Foundation [KWF Kankerbestrijding]; Netherlands Trial Register number, NTR487.).

PMID: 22646630 [PubMed - indexed for MEDLINE]

81. Gan To Kagaku Ryoho. 2012 May;39(5):825-7.

[A case of advanced gastric cancer found to be remaining by gastrectomy after a clinically complete response to chemotherapy].

[Article in Japanese]

Sakaki M(1), Taniguchi H, Ohgaki M, Kobayashi T, Haradai K, Okajima W, Ishii W, Yamada K, Morimura R, Kakihara N, Matsumura H, Fujii N, Iizuka R, Ikawa O, Fujii K, Izumi H, Takenaka A, Nakase K.

Author information:
(1)Department of Surgery, Kyoto Second Red Cross Hospital.

The patient was a 72-year-old woman diagnosed with advanced gastric cancer, hepatic portal lymph node and para-aortic lymph node metastases. After five courses of S-1/CDDP combination therapy, both the primary tumor and lymph node metastases disappeared clinically. She wished to continue chemotherapy instead of having a resection. After three more courses of S-1/CDDP therapy, gastric cancer and lymph node metastases were still completely regressed, but complications of carcinoma of the gallbladder were suspected. Gastrectomy was performed with cholecystectomy, and a histopathological examination revealed cancer cells remaining in the gastric submucosa and xanthogranulomatous cholecystitis. We consider surgical therapy for clinically completely disappearing advanced gastric cancer by chemotherapy, in addition to case report.

PMID: 22584341 [PubMed - indexed for MEDLINE]

82. Curr Oncol Rep. 2012 Aug;14(4):342-9. doi: 10.1007/s11912-012-0239-7.

Current developments in the management of locally advanced esophageal cancer.

McNamara MJ(1), Adelstein DJ.

Author information:
(1)Cleveland Clinic Foundation, Taussig Cancer Institute, 9500 Euclid Avenue R35, Cleveland, OH 44195, USA. mcnamam@ccf.org

Loco-regionally advanced esophageal cancer is a lethal disease with poor outcomes despite aggressive multimodality therapy. The appropriate management of these patients is contentious and no single standard of care has been defined. Literature suggests that preoperative chemoradiotherapy may be superior to preoperative chemotherapy. Recently, several developments have impacted the care of these patients. The 2010 AJCC TNM staging system now recognizes the biologic heterogeneity of the disease and stages adenocarcinoma and squamous cell carcinoma separately. Studies suggest potentially less toxic chemotherapeutic agents including oxaliplatin may be useful in the management of this disease. FDG-PET imaging appears to have prognostic value and may predict for pathologic response. In addition, several trials have explored inhibition of the ErbB1 (EGFR) and ErbB2 (Her2) receptors. The monoclonal antibody trastuzumab appears to extend survival for patients with metastatic gastric and gastroesophageal junction adenocarcinoma and is under investigation for use in patients with loco-regionally advanced disease.

PMID: 22544559 [PubMed - indexed for MEDLINE]

83. Jpn J Clin Oncol. 2012 Jun;42(6):556-9. doi: 10.1093/jjco/hys054. Epub 2012 Apr 23.

A Phase II study of systemic chemotherapy with docetaxel, cisplatin, and S-1 (DCS) followed by surgery in gastric cancer patients with extensive lymph node metastasis: Japan Clinical Oncology Group study JCOG1002.

Katayama H(1), Ito S, Sano T, Takahari D, Mizusawa J, Boku N, Tsuburaya A, Terashima M, Sasako M; Stomach Cancer Study Group of the Japan Clinical Oncology Group.

Author information:
(1)Department of Gastroenterological Surgery, Aichi Cancer Center Hospital, 1-1 Kanokoden, Chikusa-ku, Nagoya, Japan.

A Phase II trial was initiated in Japan to evaluate the efficacy and safety of preoperative chemotherapy with docetaxel, cisplatin and S-1 for gastric cancer with extensive lymph node metastasis. Patients are eligible to participate in the study if they have para-aortic lymph node metastases (stations no. 16a2/16b1) and/or a bulky lymph node (≥3 cm × 1 or ≥1.5 cm × 2) along the celiac, splenic, common or proper hepatic arteries or the superior mesenteric vein, while patients with other distant metastases are ineligible. A total of 50 patients will be enrolled over 2.5 years. The primary endpoint is the response rate of the preoperative chemotherapy, which will be assessed based on the Response Evaluation Criteria in Solid Tumors ver. 1.0. The secondary endpoints are %3-year survival, %5-year survival, proportion of patients with R0 resection, proportion of patients who complete the preoperative chemotherapy and surgery, proportion of patients who complete the protocol treatment, pathological response rate and adverse events. This trial was registered at the UMIN Clinical Trials Registry (www.umin.ac.jp/ctr/) as UMIN000006069.

PMID: 22525210 [PubMed - indexed for MEDLINE]

84. Gan To Kagaku Ryoho. 2012 Apr;39(4):657-61.

[Two cases of stage IV gastric cancer undergoing adjuvant surgery after down staging by chemotherapy].

[Article in Japanese]

Mori Y(1), Ohtani H, Tamamori Y, Inoue T, Azuma T.

Author information:
(1)Dept. of Surgery, Osaka City Sumiyoshi Hospital.

CASE 1: A 72-year-old man with epigastralgia was diagnosed with gastric cancer and referred to our hospital. An abdominal CT scan revealed liver metastasis and para-aortic lymph node metastasis. He was treated with S-1+CDDP. After 4 courses of this treatment, the liver metastasis and para-aortic lymph node metastasis disappeared, and adjuvant surgery was performed. There has been no recurrence for 16 months postoperatively. CASE 2: A 66-year-old man with anorexia was diagnosed with gastric cancer and referred to our hospital. An abdominal CT scan revealed para-aortic lymph node metastasis. He was treated with S-1+CDDP. After 9 courses of this treatment, para-aortic lymph node metastasis disappeared, and adjuvant surgery was performed. Eight months after the operation, lymph node metastases were confirmed by abdominal CT scan, and he was treated with chemotherapy as an outpatient as of 13 months after the operation. We experienced two cases of Stage IV gastric cancer undergoing adjuvant surgery after down staging by chemotherapy. It was suggested that adjuvant surgery to highly advanced gastric cancer could improve the prognosis of patients.

PMID: 22504697 [PubMed - indexed for MEDLINE]

85. Gan To Kagaku Ryoho. 2012 Apr;39(4):645-8.

[A successful resected case of advanced esophageal cancer with early gastric cancer responding to neoadjuvant chemotherapy of docetaxel, CDDP and 5-FU].

[Article in Japanese]

Matsutani T(1), Yoshida H, Sasajima K, Maruyama H, Yokoyama T, Matsushita A, Hirakata A, Takao Y, Umakoshi M, Hayakawa T, Katayama H, Hosone M, Uchida E.

Author information:
(1)Dept. of Surgery,Nippon Medical School Tama-Nagayama Hospital.

A 72-year-old male with a chief complaint of dysphagia was admitted to our hospital. Upper gastrointestinal endoscopic examination showed double cancers with thoracic esophageal cancer in the middle esophagus and gastric cancer in the antrum. Pathological examinations of the double cancer revealed the first one to be moderately-differentiated squamous cell carcinoma and the second to be well-differentiated adenocarcinoma. Computed tomography (CT) of the chest and abdomen showed no distant or lymph node metastases. Clinical stagings of the

double cancer were stage II (T2N0M0)in esophageal cancer and stage I A (T1N0M0) in gastric cancer. The patient received neoadjuvant chemotherapy using docetaxel, CDDP and 5-FU. After 2 courses of chemotherapy, the adverse event was grade 2 in leucopenia and grade 2 in alopecia. Repeated macroscopic and histological examinations after chemotherapy revealed that the esophageal cancer had significant reductions in the size of tumors, leading to a partial response, and the gastric cancer had disappeared, leading to a complete response. He underwent thoracoscopy-assisted esophagectomy in the prone position, and laparoscopy-assisted gastric tube reconstruction. This neoadjuvant chemotherapy of docetaxel, CDDP and 5-FU might be effective and tolerable as with patients with double cancer of esophageal and gastric cancers.

PMID: 22504694 [PubMed - indexed for MEDLINE]

86. Chin Med J (Engl). 2012 Mar;125(5):780-5.

Retrospective analysis of 56 patients with advanced gastric cancer treated with combination of intravenous and intra-arterial intensified neoadjuvant chemotherapy.

Li GL(1), Liu K, Bao Y, Cao JM, Xu J, Wang XL, Wu B, Li JS.

Author information:
(1)Research Institute of General Surgery, Department of Medical Imaging, Jinling Hospital, Nanjing University School of Medicine, Nanjing, Jiangsu 210002, China. liguoli201107@163.com

BACKGROUND: Pre-operative chemotherapy has gained widespread interest while treating advanced gastric cancer in eastern countries. However, there is currently no established standard regimen for gastric cancer. The aim of this research was to explore the value of preoperative chemotherapy with a combination of intravenous and intra-arterial intensified chemotherapy in advanced gastric cancer.
METHODS: A total of 56 histologically proven gastric cancer patients, who were considered to be stage II or higher with metastatic lymph nodes and with or without distant metastasis (T2-4, N1-3, and M0-1), were treated with a neoadjuvant chemotherapy. Patients received a combination of intravenous 5-Fu (370 mg/m2) and leucovorin (200 mg/m2) on days 1-5, and intra-arterial etoposide

(80 mg/m2) and cisplatin (80 mg/m2) on days 6 and 20. After two cycles of preoperative chemotherapy, patients with resectable tumors underwent laparotomy.
RESULTS: All patients finished two cycles of chemotherapy. The overall response rate was 78.57% (44 cases), of which 7.14% (four cases) clinical complete response. Forty-six patients underwent resection, including 21 initially unresectable diseases. R0 resection rate for prechemotherapy resectable and unresectable diseases was 96.15% (25/26 cases) and 66.67% (20/30 cases), respectively. Pathological complete response was observed in 8.70% of patients. Toxicity was moderate and there were no chemotherapy-related deaths. With a median follow-up of 31 months (range 6-76 months), the 5-year survival rate for the whole group and patients with initially resectable tumors were 21.8% and 42.3%, respectively. The median survival for initially resectable and unresectable patients were 41 months (95%CI, 31.006-50.994) and 18 months (95%CI, 13.399-22.601; P<0.01), respectively.
CONCLUSION: Preliminary results proved that the combined intensive chemotherapy was a safe and promising regimen for pre-operative treatment of advanced gastric cancer.

PMID: 22490574 [PubMed - indexed for MEDLINE]

87. Ann Surg Oncol. 2012 Sep;19(9):2937-45. doi: 10.1245/s10434-012-2332-4. Epub 2012
Mar 31.

Pilot feasibility study of neoadjuvant chemoradiotherapy with S-1 in patients with locally advanced gastric cancer featuring adjacent tissue invasion or JGCA bulky N2 lymph node metastases.

Inoue T(1), Yachida S, Usuki H, Kimura T, Hagiike M, Okano K, Suzuki Y.

Author information:
(1)Department of Gastroenterological Surgery, Faculty of Medicine, Kagawa University, Kagawa, Japan. GZT04437@nifty.ne.jp

BACKGROUND: To improve the prognosis of locally advanced gastric cancer, clinical trials of neoadjuvant chemotherapy (NAC) are being performed. Although neoadjuvant chemoradiotherapy (NACRT) generally achieves superior local tumor control to NAC, its efficacy for locally advanced gastric cancers remains

unclear. Therefore, a prospective trial was conducted to explore the feasibility and safety of NACRT with oral S-1 in a series of cases.
METHODS: Patients who had Japanese Gastric Cancer Association (JGCA) cStage IIIB gastric cancer were enrolled onto this study and received oral S-1 (65 mg/m(2)/day) administration and 50-Gy radiotherapy followed by radical surgery. The primary end points were completion of therapy and safety.
RESULTS: Between October 2005 and September 2008, 12 eligible patients were enrolled. Two could not complete the chemotherapy because of grade 3 toxicity. R0 resections were performed in 11 patients (91.7 %) (95 % confidence interval 61.5-99.8). Although operative morbidity was observed in two cases, there were no postoperative deaths. A pathologic response was observed in 10 patients (83.3 %). In five (62.5 %) of eight gastric cancers with invasion to adjacent structures, microscopic tumor deposits were not found in the affected organs. The 3-year survival rate was 58.3 % during a median follow-up period of 36 months.
CONCLUSIONS: Although this study is preliminary, the present regimen seems to be feasible and safe as a treatment for locally advanced gastric cancers featuring adjacent tissue invasion or JGCA bulky N2 disease. This treatment approach should now be tested using the new tumor, node, metastasis staging system in a large clinical trial.

PMID: 22466666 [PubMed - indexed for MEDLINE]

88. Blood. 2012 May 24;119(21):4838-44; quiz 5057. doi:
10.1182/blood-2012-01-404194.
Epub 2012 Mar 7.

Helicobacter pylori eradication therapy is effective in the treatment of early-stage H pylori-positive gastric diffuse large B-cell lymphomas.

Kuo SH(1), Yeh KH, Wu MS, Lin CW, Hsu PN, Wang HP, Chen LT, Cheng AL.

Author information:
(1)Department of Oncology, National Taiwan University Hospital and National Taiwan University College of Medicine, Taipei, Taiwan.

Comment in
    Blood. 2012 May 24;119(21):4818-9.

An explorative study evaluates the efficacy of Helicobacter pylori (HP) eradication (HPE) therapy on early-stage gastric diffuse large B-cell lymphomas (DLBCLs) without features of mucosa-associated lymphoid tissue (MALT), the pure (de novo) DLBCLs, in comparison with its efficacy on high-grade transformed gastric MALT lymphomas, the DLBCL(MALT). In total, 50 patients of stage IE/IIE1 HP-positive gastric DLBCLs with frontline HPE treatment were included. HP infection was successfully eradicated in 100% (16/16) of the pure (de novo) DLBCL patients and 94.1% (32/34) of the DLBCL(MALT) patients. In total, 68.8% (11/16) of pure (de novo) DLBCL patients and 56.3% (18/32) of DLBCL(MALT) patients achieved complete pathologic remission (pCR) after HPE therapy. The median time to pCR was 2.1 months (95% confidence interval, 0.6%-3.7%) for pure (de novo) DLBCLs and 5.0 months (95% confidence interval, 2.8%-7.5%; P = .024) for DLBCL(MALT). At a median follow-up of 7.7 years, all patients with pCR after HPE therapy were alive and free of lymphomas, except for one patient with pure (de novo) DLBCL who died of lung cancer. Similar to DLBCL(MALT), a substantial portion of early-stage HP-positive gastric pure (de novo) DLBCLs remains HP-dependent and responds to antibiotic treatment. Prospective studies to validate the findings are warranted.

PMID: 22403257 [PubMed - indexed for MEDLINE]

89. Ann Surg Oncol. 2012 Jul;19(7):2119-27. doi: 10.1245/s10434-012-2254-1. Epub 2012
Mar 7.

Prediction of response and prognosis by a score including only pretherapeutic parameters in 410 neoadjuvant treated gastric cancer patients.

Lorenzen S(1), Blank S, Lordick F, Siewert JR, Ott K.

Author information:
(1)National Center of Tumor Diseases, University of Heidelberg, and Department of Surgery, University Hospital of Heidelberg, Heidelberg, Germany.
sylvielorenzen@gmx.de

BACKGROUND: Response to neoadjuvant chemotherapy is an independent prognostic factor in locally advanced gastric cancer. However, no prospectively tested pretherapeutic parameters predicting response and/or survival in gastric cancer

are available in clinical routine.
METHODS: We evaluated the prognostic significance of various clinical pathologic parameters in 410 patients who were treated with neoadjuvant chemotherapy followed by gastrectomy. Clinical and histopathologic response evaluation was performed by using standardized criteria. A prognostic score was created on the basis of the variables identified in the multivariate analysis.
RESULTS: Three pretherapeutic parameters were identified as positive predictive factors for response and prognosis: tumor localization in the middle third of the stomach (P=0.001), well-differentiated tumors (P=0.001), and intestinal tumor type according to Laurén classification (P=0.03). A prognostic index was constructed, dividing the patients into three risk groups: low (n=73), intermediate (n=274), and high (n=63). The three groups had significantly different clinical (P=0.007) and histopathologic response rates (P=0.001) and survival times, with a median survival time that was not reached in the low-risk group, 39.2 months in the intermediate-risk group, and 20.5 months in the high-risk group. The corresponding 5-year survival rates were 65.3, 41.2, and 21.2% (P<0.001), respectively.
CONCLUSIONS: A simple scoring system based on three clinicopathologic parameters accurately predicts response and prognosis in neoadjuvant treated gastric cancer. This system provides additional useful information that could be applied to select gastric cancer patients pretherapeutically for different treatment approaches. Prospective testing of the score in an independent patient cohort is warranted.

PMID: 22395980 [PubMed - indexed for MEDLINE]

90. Cancer Chemother Pharmacol. 2012 May;69(5):1333-8. doi: 10.1007/s00280-012-1836-8. Epub 2012 Feb 5.

Phase I study of neoadjuvant chemoradiotherapy with S-1 and oxaliplatin in patients with locally advanced gastric cancer.

Lee DJ(1), Sohn TS, Lim do H, Ahn HK, Park SH, Lee J, Park JO, Park YS, Lim HY, Choi DI, Kim KM, Choi MG, Noh JH, Bae JM, Kim S, Min BH, Kang WK.

Author information:
(1)Division of Hematology-Oncology, Department of Medicine, Samsung Medical Center, Sungkyunkwan University School of Medicine, 50 Irwon-dong Kangnam-gu,

Seoul 135-710, Korea.

PURPOSE: The aim of this phase I study was to investigate the optimal dose of S-1 and oxaliplatin with concurrent radiotherapy in a preoperative setting for locally advanced gastric cancer.
PATIENTS AND METHODS: Twelve patients with histologically confirmed clinical stage T2N+ or T3-T4 gastric adenocarcinoma received dose level -1 (oral S-1 at 60 mg/m(2)/day + oxaliplatin 40 mg/m(2) intravenously on days 1, 8, 15 and 22) or dose level 1 (S-1 80 mg/m(2)/day + oxaliplatin 40 mg/m(2)), with concurrent radiotherapy at daily fractions of 1.8 Gy 5 days per week, to a total dose of 41.4 Gy. Surgical resection, including D2 dissection, was performed within 4 weeks after the last day of chemotherapy.
RESULTS: Chemoradiotherapy was generally well tolerated, with the most common dose-related grade 1 or 2 adverse events being anemia, nausea, vomiting, anorexia and abdominal pain. Two DLTs (prolonged thrombocytopenia and stomach perforation) were observed at dose level 1 (n = 6) and resulted in dose de-escalation to level -1. The recommended dose for future study is dose level -1, at which 1 of 6 patients developed grade 3 vomiting and anorexia. R0 resection was possible in 11 patients. Pathologic down-staging was observed in 6 patients, including one complete response. No clinically relevant postoperative complications occurred.
CONCLUSIONS: The activity of preoperative concurrent chemoradiotherapy with S-1 (60 mg/m(2)/day for 28 consecutive days) and oxaliplatin (40 mg/m(2) on days 1, 8, 15 and 22) will be explored more extensively in a phase II study in patients with locally advanced GC.

PMID: 22311161 [PubMed - indexed for MEDLINE]

91. J Int Med Res. 2011;39(6):2096-102.

Treatment of Borrmann type IV gastric cancer with a neoadjuvant chemotherapy combination of docetaxel, cisplatin and 5-fluorouracil/leucovorin.

Sun XC(1), Lin J, Ju AH.

Author information:
(1)Department of Oncology Surgery, Yantai Yuhuangding Hospital, Yantai, China.

This study evaluated the efficacy and safety of docetaxel, cisplatin and

5-fluorouracil/leucovorin as neoadjuvant chemotherapy before surgery (NCT group; n = 29) compared with postoperative chemotherapy alone (non-NCT group; n = 26) in the treatment of Borrmann type IV gastric carcinoma. Primary tumour response rate, surgical parameters, incised-edge residue rate, lymphatic metastasis status and side-effects were evaluated. The overall response rate was 58.6% in the NCT group, which included three (10.3%) patients in complete remission and 14 (48.3%) patients in partial remission. The postoperative pathological complete response rate was 6.9% (two patients) in the NCT group. NCT was associated with a significant increase in the radical resection rate and a significant decrease in the rate of incised-edge residues, compared with postoperative chemotherapy alone. Side-effects due to NCT were minimal and resolved with appropriate treatment. There were no chemotherapy-related deaths in either group. In conclusion, docetaxel, cisplatin and 5-fluorouracil/leucovorin was an effective and well-tolerated NCT regimen for Borrmann type IV gastric cancer.

PMID: 22289524 [PubMed - indexed for MEDLINE]

92. Acta Oncol. 2012 May;51(5):636-44. doi: 10.3109/0284186X.2011.643822. Epub 2012 Jan 2.

Locally advanced esophageal adenocarcinoma: response to neoadjuvant chemotherapy and survival predicted by ([18F])FDG-PET/CT.

Kauppi JT(1), Oksala N, Salo JA, Helin H, Karhumäki L, Kemppainen J, Sihvo EI, Räsänen JV.

Author information:
(1)Helsinki University Central Hospital, Division of General Thoracic and Esophageal Surgery, Department of Cardiothoracic Surgery, Helsinki University Central Hospital, Haartmaninkatu 4, Helsinki, Finland.

BACKGROUND: ([18F])fluorodeoxyglycose-Positron Emission Tomography/Computer Tomography (([18F])FDG-PET/CT) is commonly used in staging of locally advanced esophageal cancer. Its predictive value for response to neoadjuvant therapy and survival after multimodality therapy is controversial.
METHODS: Sixty-six consecutive patients with locally advanced adenocarcinoma of the esophagus or esophagogastric junction underwent surgery after neoadjuvant chemotherapy. Staging was done prospectively with ([18F])FDG-PET/CT, before and

after completion of neoadjuvant therapy. Pre- and post-therapy maximal standardized uptake values for the primary tumor (SUV1 and SUV2) were determined, and their relative change (SUVΔ%) calculated. Percentage change in SUV1 was compared with histopathologic response (HPR, complete or subtotal histologic remission), disease-free- (DFS) and overall survival (OS).
RESULTS: Resection with negative margins was achieved in 60 patients. HPR rate was 14 of 66 (21.2%). Median follow-up was 16 months (range 4-72). For all patients, OS probability at three years was 59% and DFS 50%. In receiver operating characteristics (ROC) analysis, HPR was optimally predicted by a > 67% change in baseline maximal SUV (sensitivity 79% and specificity 75%). In univariate survival analysis (Cox regression proportional hazards), HPR associated with improved DFS (HR 0.208, p = 0.033) but not OS (HR 0.030, p = 0.101), SUV % > 67% associated with improved OS (HR 0.249, p = 0.027) and DFS (HR 0.383, p = 0.040). In a multivariate model (adjusted by age, sex, and ASA score), neither HPR nor SUVΔ% > 67% was predictive of improved OS and DFS. However, SUV
Δ%
as a continuous variable was an independent predictor of OS (HR 0.966, p < 0.0001) or DFS (HR 0.973, p < 0.0001).
CONCLUSION: Our results support previous results showing that ([18F])FDG-PET/CT can distinguish a group of patients with worse prognosis after neoadjuvant chemotherapy in adenocarcinoma of the esophagus or esophagogastric junction. This information could offer a new independent preoperative marker of prognosis.

PMID: 22208782 [PubMed - indexed for MEDLINE]

93. Gan To Kagaku Ryoho. 2011 Nov;38(12):2334-5.

[A case of locally advanced gastric cancer responding to preoperative S-1/CDDP combination chemotherapy].

[Article in Japanese]

Katsuta E(1), Kaneko J, Iwata N, Aoyagi H, Yoshida T, Takahata T, Hasegawa K, Maejima S.

Author information:
(1)Dept. of Surgery, Hasuda Hospital.

A 59-year-old man visited our department with the complaint of an abdominal mass. After detailed examination, he was diagnosed with cT3 (SE) N2M0, cStage III C gastric cancer and underwent a 3 course preoperative S-1/CDDP combination chemotherapy. After tumor down-staging was achieved after the 3 course combination chemotherapy, a surgery was scheduled. The patient underwent distal gastrectomy with D2 lymph node dissection. Histopathological diagnosis was M, type 1, 75 × 35 mm, papillary>moderately-differentiated tubular adenocarcinoma [pT2 (MP), ly3, v0, pN2, Stage IIB]. The patient is now treated with oral S-1 as postoperative adjuvant chemotherapy on an outpatient basis, and there are no signs of recurrence as of 1 year after the surgery. Preoperative chemotherapy appears to be a promising treatment option for gastric cancer with extensive lymph node metastasis.

PMID: 22202373 [PubMed - indexed for MEDLINE]

94. Gan To Kagaku Ryoho. 2011 Nov;38(12):2100-2.

[Prolonged complete response obtained by radiation and chemotherapy with paclitaxel in a case of recurrent gastric cancer in the rectovesical pouch].

[Article in Japanese]

Kanaya N(1), Kagawa S, Uno F, Kunitomi A, Ogawa N, Fujiwara T.

Author information:
(1)Dept. of Gastroenterological Surgery, Okayama University Graduate School of Medicine, Dentistry and Pharmaceutical Sciences.

The case is a 75-year-old man, who underwent curative resection for the upper part of stomach cancer (Stage II), and was without a recurrence for two years. The patient had been well until September 2006, when CT examination revealed a 5 cm tumor in the rectovesical pouch. The tumor was histologically diagnosed as a peritoneal metastasis of gastric cancer through CT-guided percutaneous needle biopsy. Chemotherapy with S-1 was initiated, but it resulted in progressive disease. To locally control the growing tumor and avoid rectal stenosis, irradiation was applied for a total of 56 Gy in December 2007 and then followed by chemotherapy with paclitaxel. He obtained a complete response 7-month later, and remains CR for 3 years by receiving paclitaxel chemotherapy. Although

chemotherapy is of choice for gastric cancer recurrence, in some cases radiotherapy can play an important role in local control even in peritoneal metastasis.

PMID: 22202296 [PubMed - indexed for MEDLINE]

95. Gan To Kagaku Ryoho. 2011 Nov;38(12):1954-6.

[A case of advanced gastric cancer with splenic vein thrombus successfully treated with S-1 plus CPT-11 neoadjuvant chemotherapy].

[Article in Japanese]

Taniguchi H(1), Kimura Y, Kim C, Danno K, Kagara N, Kanoh T, Ohnishi T, Tohno T, Nakano Y, Monden T, Imaoka S.

Author information:
(1)Dept. of Surgery, NTT West Osaka Hospital.

A 63-year-old male admitted for hematemesis was diagnosed with type 3 advanced gastric cancer located in the upper and middle body of the stomach in an endoscopic examination. Abdominal computed tomography demonstrated lymph nodes metastasis and a splenic vein thrombus. Since curative resection was not deemed possible, we performed neoadjuvant chemotherapy using S-1 (120 mg, day 1-21) plus CPT-11 (135 mg, day 1 and 15) except for down-staging. After 4 courses of chemotherapy, gastric tumor and metastatic lymph nodes were reduced in size and the splenic vein thrombus was disappeared, and then total gastrectomy was performed (tub2, T2 (MP) N0 H0 M0 P0 CY0, Stage IB). S-1 medication was applied as adjuvant chemotherapy. Forty months passed from the operation, the patient remains alive with no signs of relapse.

PMID: 22202250 [PubMed - indexed for MEDLINE]

96. Gan To Kagaku Ryoho. 2011 Nov;38(12):1951-3.

[A case of S-1/CDDP chemotherapy for inoperable advanced gastric cancer which led to gastrectomy with histological complete response].

[Article in Japanese]

Kobayashi K(1), Tanizaki K, Aoki T, Takachi K, Nishioka K, Matsumoto T, Komori T, Chono T, Kato A, Hyuga S, Watanabe R, Uemura Y.

Author information:
(1)Dept. of Surgery, Kinki Central Hospital.

As the treatment for inoperable advanced gastric cancer, S-1/CDDP combination therapy (SP chemotherapy) has become a standard treatment. In our hospital, a second course of chemotherapy was performed on an outpatient basis in order to improve a traditional QOL. In this case, it showed remarkable effects in 15 months after starting chemotherapy. Then gastrectomy was performed. Histological findings of the resected specimens confirmed pCR in all tumors. We report on progress of this case and explain about the ingenuity of SP chemotherapy.

PMID: 22202249 [PubMed - indexed for MEDLINE]

97. Anticancer Res. 2011 Dec;31(12):4625-30.

Weekly paclitaxel in combination with doxifluridine for peritoneally disseminated gastric cancer with malignant ascites.

Takeyoshi I(1), Makita F, Iwazaki S, Ishikawa H, Kakinuma S, Sato Y, Ohya T, Nakagami K, Tomizawa N, Izumi M, Kobayashi I, Tanahashi Y, Kobayashi J, Kamoshita N, Kawate S, Sunose Y, Sakamoto I, Yoshinari D, Yamada T, Okabe T.

Author information:
(1)Department of Thoracic and Visceral Organ Surgery, Gunma University, Faculty of Medicine, 3-39-15 Showa-machi, Maebashi, Gunma 371-8511, Japan.
takeyosi@showa.gunma-u.ac.jp

BACKGROUND: The efficacy of systemic chemotherapy for peritoneal dissemination of gastric cancer remains unclear. The efficacy of weekly paclitaxel in combination with doxifluridine (5'-DFUR) in gastric cancer patients with malignant ascites was evaluated.
PATIENTS AND METHODS: Patients with histologically confirmed gastric cancer with

ascites were eligible. The treatment consisted of paclitaxel intravenously (i.v.) administered at 80 mg/m(2) on days 1, 8 and 15 every 4 weeks, and doxifluridine administered orally at 533 mg/m(2) on days 1-5 every week. The response rate for patients with ascites was determined based on the Japanese Classification of Gastric Carcinoma. Also, the concentration of paclitaxel in the ascites was measured.
RESULTS: Twenty-four patients were investigated. The response rate (RR) was 41.7%, including complete remission (CR) and partial remission (PR) in 4 and 6 patients, respectively. The concentration of paclitaxel in the ascites was maintained between 0.01 μM and 0.05 μM until 72 hours. The median overall survival (OS) was 215 days, and 1-year survival rate was 29.2%. No severe toxicity was noted.
CONCLUSION: Weekly paclitaxel in combination with doxifluridine is effective for gastric cancer patients with malignant ascites with an acceptable toxicity profile.

PMID: 22199340 [PubMed - indexed for MEDLINE]

98. J Surg Oncol. 2012 Jun 15;105(8):793-9. doi: 10.1002/jso.23009. Epub 2011 Dec 20.

Neoadjuvant chemotherapy with FOLFOX: improved outcomes in Chinese patients with locally advanced gastric cancer.

Li ZY(1), Koh CE, Bu ZD, Wu AW, Zhang LH, Wu XJ, Wu Q, Zong XL, Ren H, Tang L, Zhang XP, Li JY, Hu Y, Shen L, Ji JF.

Author information:
(1)Department of Gastrointestinal Surgery, Beijing Cancer Hospital and Institute, Peking University School of Oncology, Key Laboratory of Carcinogenesis and Translational Research (Ministry of Education), Beijing, China.

BACKGROUND: Although the role of peri-operative chemotherapy is established in the treatment of locally advanced gastric cancer, the optimal regime remains to be determined. FOLFOX has been used in palliative setting with good response rates but its role in a neoadjuvant setting is not well established.
METHODS: This is a prospective non-randomized study comparing peri-operative FOLFOX versus adjuvant FOLFOX in patients with resectable locally advanced

gastric cancer. Response to chemotherapy was assessed according to WHO criteria and pathological changes. Kaplan-Meier log rank test was used to calculate and compare survival differences.
RESULTS: There were 73 patients (neoadjuvant = 36). Complete and partial response was observed in 2 (6%) and 21 (64%) patients, respectively. Four-year overall survival (OS) in the neoadjuvant arm was 78% versus 51% in the adjuvant arm (P = 0.031). Subgroup analysis found R0 resection (86% vs. 55%, P = 0.011) and patients with proximal cancers (87% vs. 14%, P < 0.001) to have improved OS. The most common side effect was grade 1-2 leukopenia. There were no grade 3 neuropathies, grade 4 cytopaenias, or treatment related deaths.
CONCLUSION: Peri-operative treatment with FOLFOX shows promise in patients with resectable locally advanced gastric cancer. It warrants further evaluation and should be considered an alternative to peri-operative ECF.

Copyright © 2011 Wiley Periodicals, Inc.

PMID: 22189752 [PubMed - indexed for MEDLINE]

99. Ann Surg Oncol. 2012 Jun;19(6):1936-43. doi: 10.1245/s10434-011-2165-6. Epub 2011
Dec 21.

Posttherapy nodal status, not graded histologic response, predicts survival after neoadjuvant chemotherapy for advanced gastric cancer.

Fujitani K(1), Mano M, Hirao M, Kodama Y, Tsujinaka T.

Author information:
(1)Department of Surgery, Osaka National Hospital, Osaka, Japan.
fujitani@onh.go.jp

BACKGROUND: Neoadjuvant chemotherapy (NAC) has been attempted as a means of improving survival of potentially resectable advanced gastric cancer (AGC). In the course of exploring the most promising NAC regimen, a superior surrogate marker reflecting overall survival (OS) is necessary. We investigated prognostic factors in AGC patients who underwent NAC followed by gastric resection and evaluated whether histologic response to NAC was predictive of survival.
METHODS: Seventy consecutive patients with gastric cancer treated with NAC

followed by surgical resection between Jan 1, 2000, and Dec 31, 2009, at Osaka National Hospital were identified from a prospective database. Prognostic factors for OS were investigated by univariate and multivariate analyses.
RESULTS: Median survival time for all patients was 668 days after surgical resection. Age less than 65 years (hazard ratio 0.463, 95% confidence interval 0.244-0.879) and pathologic nodal stage of N0-1 (hazard ratio 0.318, 95% confidence interval 0.160-0.635) were identified as significant independent prognostic factors for longer OS, whereas graded histologic response of primary tumor to NAC was statistically significant on univariate analysis, but not on multivariate analysis, as a prognostic factor.
CONCLUSIONS: Posttherapy nodal status, not graded histologic response, predicts survival after NAC for AGC and could serve as a reliable surrogate marker for OS in the course of exploring the most promising regimen for NAC.

PMID: 22187120 [PubMed - indexed for MEDLINE]

100. Int J Radiat Oncol Biol Phys. 2012 Jun 1;83(2):581-6. doi: 10.1016/j.ijrobp.2011.07.035. Epub 2011 Dec 2.

Intensity-modulated radiation therapy with concurrent chemotherapy as preoperative treatment for localized gastric adenocarcinoma.

Chakravarty T(1), Crane CH, Ajani JA, Mansfield PF, Briere TM, Beddar AS, Mok H, Reed VK, Krishnan S, Delclos ME, Das P.

Author information:
(1)Department of Radiation Oncology, The University of Texas MD Anderson Cancer Center, Houston, TX 77030, USA.

PURPOSE: The goal of this study was to evaluate dosimetric parameters, acute toxicity, pathologic response, and local control in patients treated with preoperative intensity-modulated radiation therapy (IMRT) and concurrent chemotherapy for localized gastric adenocarcinoma.
METHODS: Between November 2007 and April 2010, 25 patients with localized gastric adenocarcinoma were treated with induction chemotherapy, followed by preoperative IMRT and concurrent chemotherapy and, finally, surgical resection. The median radiation therapy dose was 45 Gy. Concurrent chemotherapy was 5-fluorouracil and oxaliplatin in 18 patients, capecitabine in 3, and other regimens in 4.

Subsequently, resection was performed with total gastrectomy in 13 patients, subtotal gastrectomy in 7, and other surgeries in 5.
RESULTS: Target coverage, expressed as the ratio of the minimum dose received by 99% of the planning target volume to the prescribed dose, was a median of 0.97 (range, 0.92-1.01). The median V(30) (percentage of volume receiving at least 30 Gy) for the liver was 26%; the median V(20) (percentage of volume receiving at least 20 Gy) for the right and left kidneys was 14% and 24%, respectively; and the median V(40) (percentage of volume receiving at least 40 Gy) for the heart was 18%. Grade 3 acute toxicity developed in 14 patients (56%), including dehydration in 10, nausea in 8, and anorexia in 5. Grade 4 acute toxicity did not develop in any patient. There were no significant differences in the rates of acute toxicity, hospitalization, or feeding tube use in comparison to those in a group of 50 patients treated with preoperative three-dimensional conformal radiation therapy with concurrent chemotherapy. R0 resection was obtained in 20 patients (80%), and pathologic complete response occurred in 5 (20%).
CONCLUSIONS: Preoperative IMRT for gastric adenocarcinoma was well tolerated, accomplished excellent target coverage and normal structure sparing, and led to appropriate pathologic outcomes.

Copyright © 2012 Elsevier Inc. All rights reserved.

PMID: 22137021 [PubMed - indexed for MEDLINE]

Items 1 -284 of 284   (Display the 284 citations in PubMed)

1. Ann Oncol. 2012 Jun;23(6):1512-7. doi: 10.1093/annonc/mdr465. Epub 2011 Oct 29.

Perioperative docetaxel, cisplatin, and 5-fluorouracil (DCF) for locally advanced esophageal and gastric adenocarcinoma: a multicenter phase II trial.

Ferri LE(1), Ades S, Alcindor T, Chasen M, Marcus V, Hickeson M, Artho G, Thirlwell MP.

Author information:
(1)Department of Surgery, University of Vermont, Burlington, VT, USA.
lorenzo.ferri@mcgill.ca

BACKGROUND: Although perioperative chemotherapy for esophagogastric

adenocarcinoma (ADC) improves survival, the overall poor prognosis suggests that further refinement of treatment is required. Docetaxel, cisplatin, and 5-fluorouracil (5-FU) (DCF) is effective for metastatic ADC of the upper gastrointestinal (GI) tract; we thus sought to investigate the efficacy of this regimen in patients with resectable disease.
PATIENTS AND METHODS: Patients with resectable ADC of the upper GI tract received DCF [docetaxel (Taxotere) 75 mg/m(2) I.V. day 1, cisplatin 75 mg/m(2) I.V. day 1, 5-FU 750 mg/m(2) continuous infusion for 120 h, every 3 weeks] for three cycles before and after resection. Primary end point was complete resection; secondary end points were response, toxicity, surgical morbidity, and overall survival.
RESULTS: Forty-three patients with ADC of the esophagus (11), gastroesophageal junction (25), or stomach (7) started treatment and 86% completed all preoperative cycles with grade 3-4 toxicity arising in 47%. Metabolic response to chemotherapy (reduction in maximal standard uptake value >35%) was achieved in 25/33 (76%) patients. Surgery was carried out in 41/43 and complete resection was achieved in all 41 patients with pathologic complete response in 4/41. Postoperative chemotherapy was started in 29 patients and completed in 24. Three-year overall survival was 60%.
CONCLUSION: Perioperative DCF is a tolerable and highly effective regimen for the treatment of esophagogastric ADC.

PMID: 22039085 [PubMed - indexed for MEDLINE]

2. Med Oncol. 2012 Sep;29(3):1707-10. doi: 10.1007/s12032-011-0093-8. Epub 2011 Oct 28.

Preoperative docetaxel/cisplatin/5-fluorouracil chemotherapy in patients with locally advanced gastro-esophageal adenocarcinoma.

Bayraktar UD(1), Bayraktar S, Hosein P, Chen E, Koniaris LG, Rocha-Lima CM, Montero AJ.

Author information:
(1)Division of Hematology/Oncology, Department of Medicine, Sylvester Comprehensive Cancer Center, University of Miami, 1475 NW 12th Ave St, Miami, FL 3300, USA. darda.bayraktar@gmail.com

Perioperative chemotherapy plus surgery improves survival compared to surgery

alone in GE junctional (GEJ) and gastric adenocarcinomas. The docetaxel/cisplatin/5-fluorouracil (DCF) combination is superior to CF in patients with metastatic gastric cancer. We retrospectively evaluated the safety and efficacy of preoperative DCF chemotherapy in patients with locally advanced gastric and GEJ cancer. Twenty-one gastric and 10 gastroesophageal junctional (GEJ) cancer patients received 2-3 cycles of preoperative docetaxel 75 mg/m(2) and cisplatin 75 mg/m(2) on day 1, 5-FU 750 mg/m(2) (continuous infusion) on days 1-5 every 3 weeks. Clinical response was evaluated by comparing pre- and postchemotherapy CT scans. Overall survival (OS) and progression-free survival (PFS) were calculated from the initiation of chemotherapy. None of the patients achieved complete clinical remission while 11 (35%) patients achieved partial clinical remission. Ten patients with GEJ cancer (100%) and 13 with gastric cancer (62%) underwent curative surgery (P = 0.023). Seventeen (55%) patients experienced grade 3-4 chemotherapy-related adverse events. The most common adverse events were anemia, nausea/vomiting, diarrhea, and febrile neutropenia. At a median follow-up of 17.0 months, median OS and PFS were 26.1 months (95% CI: 22.7-29.5) and 18.8 months (95% CI: 9.9-27.7), respectively. The DCF regimen is active in patients with gastric and GEJ adenocarcinoma in the preoperative setting.

PMID: 22033912 [PubMed - indexed for MEDLINE]

3. Int J Radiat Oncol Biol Phys. 2012 Mar 1;82(3):e513-9. doi: 10.1016/j.ijrobp.2011.05.071. Epub 2011 Oct 17.

Anastomotic complications after Ivor Lewis esophagectomy in patients treated with neoadjuvant chemoradiation are related to radiation dose to the gastric fundus.

Vande Walle C(1), Ceelen WP, Boterberg T, Vande Putte D, Van Nieuwenhove Y, Varin O, Pattyn P.

Author information:
(1)Department of Gastrointestinal Surgery, University Hospital, Ghent, Belgium.

PURPOSE: Neoadjuvant chemoradiation (CRT) is increasingly used in locally advanced esophageal cancer. Some studies have suggested that CRT results in increased surgical morbidity. We assessed the influence of CRT on anastomotic complications in a cohort of patients who underwent CRT followed by Ivor Lewis

esophagectomy.
PATIENTS AND METHODS: Clinical and pathologic data were collected from all patients treated with neoadjuvant CRT (36 Gy combined with 5-fluorouracil and cisplatin) followed by Ivor Lewis esophagectomy. On the radiotherapy (RT) planning computed tomography scans, normal tissue volumes were drawn encompassing the proximal esophageal region and the gastric fundus. Within these volumes, dose-volume histograms were analyzed to generate the total dose to 50% of the volume (D(50)). We studied the ability of the D(50) to predict anastomotic complications (leakage, ischemia, or stenosis). Dose limits were derived using receiver operating characteristics analysis.
RESULTS: Fifty-four patients were available for analysis. RT resulted in either T or N downstaging in 51% of patients; complete pathologic response was achieved in 11%. In-hospital mortality was 5.4%, and major morbidity occurred in 36% of patients. Anastomotic complications (AC) developed in 7 patients (13%). No significant influence of the D(50) on the proximal esophagus was noted on the anastomotic complication rate. The median D(50) on the gastric fundus, however, was 33 Gy in patients with AC and 18 Gy in patients without AC (p = 0.024). Using receiver operating characteristics analysis, the D(50) limit on the gastric fundus was defined as 29 Gy.
CONCLUSIONS: In patients undergoing neoadjuvant CRT followed by Ivor Lewis esophagectomy, the incidence of AC is related to the RT dose on the gastric fundus but not to the dose received by the proximal esophagus. When planning preoperative RT, efforts should be made to limit the median dose on the gastric fundus to 29 Gy with a V(30) below 40%.

Copyright © 2012 Elsevier Inc. All rights reserved.

PMID: 22014951 [PubMed - indexed for MEDLINE]

4. Cancer. 2012 Jun 1;118(11):2820-7. doi: 10.1002/cncr.26591. Epub 2011 Oct 11.

Phase 2 trial of induction and concurrent chemoradiotherapy with weekly irinotecan and cisplatin followed by surgery for esophageal cancer.

Ilson DH(1), Minsky BD, Ku GY, Rusch V, Rizk N, Shah M, Kelsen DP, Capanu M, Tang L, Campbell J, Bains M.

Author information:

(1)Gastrointestinal Oncology Service, Department of Medicine, Memorial Sloan-Kettering Cancer Center, New York, New York 10065, USA. ilsond@mskcc.org

BACKGROUND: Preoperative chemoradiation improves survival in esophageal and gastroesophageal junction (GEJ) cancer. We evaluated irinotecan and cisplatin as induction chemotherapy followed by concurrent chemoradiation in esophageal cancer.
METHODS: Patients with uT1N1M0 or uT2-4NanyM0 resectable squamous cancer or adenocarcinoma of the esophagus or GEJ received irinotecan 65 mg/m(2) and cisplatin 30 mg/m(2) for 4 treatments in weeks 1 through 5, followed by 4 treatments in weeks 7 through 11 with 50.4 Gy in daily fractions, followed by surgery. The primary endpoint was pathologic complete response (pCR). Positron emission tomography (PET) scan was performed prior to chemotherapy and as restaging prior to radiotherapy.
RESULTS: Fifty-five patients were evaluable, 75% of whom had adenocarcinoma and 65% of whom had uT3N1 disease. Thirty-eight patients underwent R0 resection (69%). The incidence of pCR was 16% (95% confidence interval, 8%-29%). Median overall survival was 31.7 months. An exploratory analysis of PET response to induction chemotherapy indicated a correlation with pCR (32% vs 4%), R0 resection (84% vs 57%), progression-free survival (24.1 vs 7.7 months), and overall survival (40.2 vs 25.5 months).
CONCLUSIONS: Weekly treatment with irinotecan, cisplatin, and radiation achieved results no better and potentially inferior to other phase 2 chemoradiotherapy trials with a low rate of pCR. The use of PET scan after induction chemotherapy to direct chemotherapy during subsequent radiotherapy merits further study.

Copyright © 2011 American Cancer Society.

PMID: 21990000 [PubMed - indexed for MEDLINE]

5. World J Surg Oncol. 2011 Sep 28;9:112. doi: 10.1186/1477-7819-9-112.

Is there any advantage to combined trastuzumab and chemotherapy in perioperative setting her 2neu positive localized gastric adenocarcinoma?

Sbitti Y(1), Essaidi I, Debbagh A, Kadiri H, Oukabli M, Moussaid Y, Slimani K, Fetohi M, Elkaoui H, Albouzidi A, Mahi M, Ali AA, Ichou M, Errihani H.

Author information:
(1)Department of Medical Oncology, University Military Hospital; Rabat, 10000, Morocco. sbittiyassir@yahoo.fr

We report here a 44-year-old Moroccan man with resectable gastric adenocarcinoma with overexpression of human epidermal growth factor receptor 2 (HER2) by immunohistochemistry who was treated with trastuzumab in combination with chemotherapy in perioperative setting. He received 3 cycles of neoadjuvant chemotherapy consisting of trastuzumab, oxaliplatin, and capecitabine. Afterwards, he received total gastrectomy with extended D2 lymphadenectomy without spleno-pancreatectomy. A pathologic complete response was obtained with a combination of trastuzumab and oxaliplatin and capecitabine. He received 3 more cycles of trastuzumab containing regimen postoperatively.We conclude that resectable gastric carcinoma with overexpression of the c-erbB-2 protein should ideally be managed with perioperative combination of trastuzumab with chemotherapy. Further research to evaluate trastuzumab in combination with chemotherapy regimens in the perioperative and adjuvant setting is urgently needed.

PMCID: PMC3204255
PMID: 21955806 [PubMed - indexed for MEDLINE]

6. J Surg Oncol. 2012 Mar;105(3):293-6. doi: 10.1002/jso.22085. Epub 2011 Aug 31.

Clinicopathologic variables predicting tumor response to neoadjuvant chemotherapy in patients with locally advanced gastric cancer.

Wang LB(1), Teng RY, Jiang ZN, Hu WX, Dong MJ, Yuan XM, Chen WJ, Jin M, Shen JG.

Author information:
(1)Department of Surgical Oncology, Sir Run Run Shaw Hospital, Zhejiang University College of Medicine, Hangzhou, China.

BACKGROUND AND OBJECTIVES: To identify clinicopathologic variables that could predict pathologic tumor response to neoadjuvant chemotherapy for patients with locally advanced gastric cancer.
METHODS: The study enrolled 108 patients who underwent neoadjuvant chemotherapy followed by surgery between July 2004 and December 2010. Tumor responses to

neoadjuvant chemotherapy were assessed in terms of tumor regression. Statistical analyses were performed to identify factors associated with pathologic tumor response.
RESULTS: Tumor regression was found in 22.2% (24/108) patients, patients with tumor regression observed better overall survival as compared to that of patients without tumor regression. Univariate and multivariate analyses observed that both tumor differentiation and tumor size were independent predictors of tumor regression.
CONCLUSIONS: This study suggests that both tumor differentiation and tumor size is the most important clinical predicator of pathologic tumor response, it may be of benefit in the selection of treatment options in locally advanced gastric cancer.

Copyright © 2011 Wiley Periodicals, Inc.

PMID: 21882201 [PubMed - indexed for MEDLINE]

7. Gan To Kagaku Ryoho. 2011 Aug;38(8):1349-52.

[A case of long-term survival after resection of aortic arch for locally advanced non-small cell lung cancer with induction chemotherapy].

[Article in Japanese]

Higuchi M(1), Suzuki H, Shio Y, Satoh Y, Kitamura M, Gotoh M.

Author information:
(1)Dept. of Regenerative Surgery, Fukushima Medical University School of Medicine.

Our patient was a 57-year-old male with a history of esophageal cancer. He was referred to our hospital for squamous cell lung carcinoma(SCC). Chest computed tomography identified a mass in the left lung field, which was suspected to be invading the reconstructed gastric tube, left subclavian artery, common carotid artery, and distal aortic arch. He was diagnosed as primary pulmonary squamous cell carcinoma(SCC)because six years had already passed since a previous surgery for early esophageal cancer. He received three courses of induction chemotherapy including S-1/CDDP. We evaluated the therapy as a partial response. He underwent

an extended resection of distal aortic arch and left subclavian artery with left upper lobectomy, and those vessels were reconstructed using prosthetic grafts. Pathological findings showed the tumor as a well differentiated SCC of pT4N0M0 at stage III A, with a residual tumor on the reconstructed gastric tube, even though the effect of induction chemotherapy was Ef2. He received three courses of S-1/CDDP after surgery. The patient has been well without recurrence for 31 months after surgery.

PMID: 21829079 [PubMed - indexed for MEDLINE]

8. Gan To Kagaku Ryoho. 2011 Aug;38(8):1246-51.

[Treatment strategy for marginally resectable gastric cancer].

[Article in Japanese]

Nashimoto A(1), Yabusaki H, Nakagawa S, Matsuki A.

Author information:
(1)Division of Surgery, Niigata Cancer Center Hospital, kawagishi-cho, chuo-ku, Niigata, Japan.

It has been postulated that preoperative chemotherapy might promote tumor regression, eradicate nodal metastases, and improve resectability in patients with marginally resectable gastric cancer.For a marginally resectable tumor of gastric cancer, we selected the advanced gastric cancer patients with metastases and recurrences to the abdominal para-aortic lymph node (PAN), liver and invasion to the pancreas head and/or the duodenum.Patients with positive peritoneal cytology(P0, CY1)or localized peritoneal metastasis(P1), and Stage IV gastric cancer patients, were also considered candidates in this category. The strategy and results of surgical treatment for marginally resectable gastric cancer were explained as the dissection of PAN, hepatic resection, pancreaticoduodenectomy, perioperative chemotherapy for P0CY1 or P1, and neoadjuvant chemotherapy for Stage IV gastric cancer, which was still considered an experimental approach, although its use may be justified in unresectable or marginally resectable GC.The result of the resection of a marginally resectable gastric cancer is poor, but when there are no other non-curative factors, extended surgical resection should be performed because complete response is difficult at present with chemotherapy

alone.In conclusion, there was no evidence suggesting that extended surgical procedures are effective, but a strategy of multidisciplinary treatment including extended surgical approach should be verified based on randomized controlled trials.

PMID: 21829060 [PubMed - indexed for MEDLINE]

9. Int J Hematol. 2011 Aug;94(2):209-12. doi: 10.1007/s12185-011-0897-5. Epub 2011 Jul 29.

Sister Mary Joseph's nodule as a rare sign of lymphoma.

Cetin B(1), Benekli M, Bulutay P, Akyurek N, Cura E, Coskun U, Buyukberber S.

Author information:
(1)Division of Medical Oncology, Department of Internal Medicine, Gazi University Faculty of Medicine, Besevler, Ankara, 06500, Turkey. caretta06@hotmail.com

Sister Mary Joseph's periumbilical metastatic nodule is an ominous harbinger of an internal malignancy usually originating from gastrointestinal or genital system primary cancers. At the time of diagnosis, they are inoperable and therefore deemed incurable, suggesting an invariably dismal prognosis. Periumbilical neoplastic deposits from primary non-Hodgkin's lymphoma are extremely rare. A 72-year-old white male with a history of gastric cancer was referred with a painful lesion in the umbilicus. A delayed biopsy of the nodule showed diffuse large B-cell lymphoma. After a work up, he was successfully treated with standard combination chemotherapy and achieved complete remission. However, the patient developed central nervous system relapse and died a few months later. Our patient illustrates the importance of obtaining a tissue diagnosis before diagnosing an internal carcinoma as the underlying cause in patients with Sister Mary Joseph's nodule. We should keep in mind that not all periumbilical tumoral deposits are the same.

PMID: 21800076 [PubMed - indexed for MEDLINE]

10. Gan To Kagaku Ryoho. 2011 Jul;38(7):1183-6.

[Pathological complete response in advanced gastric carcinoma by S-1/CDDP combination chemotherapy].

[Article in Japanese]

Toriumi F(1), Ei S, Takahashi H, Okada R, Masai K, Mito M, Ishimaru N, Mizuno K, Handa K, Yamanashi T, Koshida Y, Akamatsu H, Motegi K, Tajima T, Mukai K, Shimoyama Y.

Author information:
(1)Division of Surgery, Saiseikai Central Hospital, Japan.

A59 -year-old woman was referred to our hospital for a close examination and treatment of an advanced gastric carcinoma. A physical examination and CT scan showed that the right cervical and axillar lymph nodes were swelling, and a histopathological examination of the axillar lymph node revealed metastatic growth of the gastric carcinoma (Stage IV). Then, we started S-1/CDDP combination chemotherapy. S-1 (80 mg/m2/day)was orally administered for 3 weeks followed by 2 weeks of rest, and CDDP (60 mg/m2) was administered by drip on day 8. Since the distant metastases were greatly reduced after 6 courses of combination therapy, a distal gastrectomy with lymph nodes dissection (D2) was performed. Histopathological examination of the resected tissues revealed no residual cancer cells, suggesting a pathologically complete response. The clinical course after the operation went well without any complications, and the patient is alive with no evidence of recurrence 1 year after surgery. S-1/CDDP combination chemotherapy appears to be one of the effective treatments for advanced gastric carcinoma.

PMID: 21772108 [PubMed - indexed for MEDLINE]

11. Gan To Kagaku Ryoho. 2011 Jul;38(7):1175-8.

[A case of advanced gastric cancer with tumor embolus in the portal vein successfully treated with S-1 and CDDP therapy].

[Article in Japanese]

Asaumi Y(1), Miyanaga T, Ito H, Shin H, Fujita M, Miyazaki M, Yagi D, Kitamura H, Hirano M, Hayashida Y, Maeda K, Ohta K, Hayashi H, Doden K, Hattori M, Hashizume

Y, Kaizaki Y.

Author information:
(1)Dept. of Surgery, Fukui Prefectural Hospital, Japan.

A 50-year-old man with advanced gastric cancer and a tumor embolus in the portal vein was referred to our hospital. We diagnosed the tumor as cStage III B (cT3, cN2, cH0, P0, M0) gastric cancer, and selected neoadjuvant S-1 (80 mg/m2) and CDDP (60 mg/m2) therapy for him. After 2 courses of chemotherapy, the embolus in the portal vein disappeared. After additional chemotherapy, the primary tumor and regional lymph node revealed a partial response (PR), and judging from the results from the barium meal study, upper GI endoscopic findings and CT scan, a total gastrectomy with lymph node dissection was performed.

PMID: 21772106 [PubMed - indexed for MEDLINE]

12. Clin Oncol (R Coll Radiol). 2011 Dec;23(10):696-705. doi:
10.1016/j.clon.2011.05.005. Epub 2011 Jun 17.

Neoadjuvant chemoradiotherapy for resectable oesophageal and gastro-oesophageal junction cancer--do we need another randomised trial?

Hingorani M(1), Crosby T, Maraveyas A, Dixit S, Bateman A, Roy R.

Author information:
(1)Queen's Centre for Oncology and Haematology, Castle Hill Hospital, Cottingham, UK. mohan.hingorani@hey.nhs.uk

AIMS: The optimal neoadjuvant therapy option for locally advanced oesophageal cancer remains elusive. Neoadjuvant chemoradiotherapy (CRT) is the preferred modality of choice in the USA. In contrast, neoadjuvant chemotherapy is commonly used in the UK. We provide a comprehensive overview of the available evidence for defining the ideal neoadjuvant treatment algorithm.
MATERIALS AND METHODS: The PubMed database combined with American Society of Clinical Oncology and American Society for Therapeutic Radiology and Oncology websites were searched online to identify randomised studies and published meta-analyses that have compared these modalities compared with surgery alone. In particular, we searched for randomised trials that may have directly compared

outcomes after neoadjuvant CRT or chemotherapy.
RESULTS: We identified 17 published randomised studies of neoadjuvant CRT (n = 9) and chemotherapy (n = 8) compared with surgery alone and one prospective series that compared the above modalities against each other. Studies evaluating CRT have reported pathological complete response rates of 15-40% and no increase in postoperative mortality was observed, except in one study that used a hypofractionated radiation schedule. Two randomised studies showed significant survival benefit and the remaining (n = 7) were negative, but showed a trend towards improved survival. Furthermore, at least four meta-analyses have shown improved survival in favour of CRT extending up to an absolute benefit of 13% at 2 years. In comparison, five studies of neoadjuvant chemotherapy showed no survival difference and two of the remaining studies that showed significant benefit included gastric adenocarcinomas and used peri-operative chemotherapy. All the above studies have shown uniformly poor pathological complete response rates of less than 10 percent. Moreover, three meta-analyses were negative, but two showed up to 7% absolute survival benefit at 2 years in favour of chemotherapy. The trial comparing the above modalities showed a trend towards improved survival in favour of CRT, but closed early due to poor recruitment.
CONCLUSION: Data from the above studies are potentially conflicting and inconclusive for defining the optimal neoadjuvant treatment schedule. In our opinion, the above question can only be answered within the context of a randomised control trial. We have included a proposal for a trial design for direct comparison of these modalities.

Copyright Â© 2011 The Royal College of Radiologists. Published by Elsevier Ltd. All rights reserved.

PMID: 21684129 [PubMed - indexed for MEDLINE]

13. Ann Oncol. 2012 Mar;23(3):664-70. doi: 10.1093/annonc/mdr291. Epub 2011 Jun 7.

Phase II trial of preoperative chemoradiotherapy with oxaliplatin, cisplatin, and 5-FU in locally advanced esophageal and gastric cancer.

Pera M(1), Gallego R, Montagut C, Martín-Richard M, Iglesias M, Conill C, Reig A, Balagué C, Pétriz L, Momblan D, Bellmunt J, Maurel J.

Author information:

(1)Section of Gastrointestinal Surgery, Hospital Universitario del Mar and Institut de Recerca Hospital del Mar, Universitat Autónoma de Barcelona, Spain. pera@parcdesalutmar.cat

BACKGROUND: Based on a phase I study showing the feasibility of combining of oxaliplatin, cisplatin, and 5-fluorouracil (5-FU) (OCF) with radiation therapy (RT) in esophageal cancer, the efficacy of this regimen in esophageal, gastroesophageal (GE), and gastric (G) cancer was assessed in this phase II multicenter study.
PATIENTS AND METHODS: Patients with resectable tumors were eligible. Treatment included two cycles of oxaliplatin 85 mg/m(2), cisplatin 55 mg/m(2), and continuously infused 5-FU 3 g/m(2) in 96 h and concurrent RT (45 Gy), followed by surgery after 6-8 weeks. Primary end point was complete pathologic response (pCR).
RESULTS: Forty-one patients were enrolled. Tumor location was esophagus 39% (squamous 10/adenocarcinoma 6), GE junction 32%, and stomach 29%. G3-G4 adverse events included asthenia (27%) and neutropenia (14%). One toxic death occurred. Thirty-one patients (75.6%) underwent surgery (R0 in 94%). Pathologic response was achieved in 58% of patients, with pCR in 50% and 16% of esophageal and GE/G cancer, respectively. pCR was achieved in 67% of squamous cell carcinoma. Survival: median follow-up, 50.4 months; median progression-free survival and overall survival were 23.2 and 28.4 months, respectively.
CONCLUSION: Preoperative OCF plus RT showed an acceptable toxicity and promising activity especially in squamous cell esophageal cancer.

PMID: 21652581 [PubMed - indexed for MEDLINE]

14. Oncologist. 2011;16(6):844-58. doi: 10.1634/theoncologist.2010-0387. Epub 2011 May 31.

Targeting angiogenesis in esophagogastric adenocarcinoma.

Okines AF(1), Reynolds AR, Cunningham D.

Author information:
(1)The Royal Marsden Hospital NHS Foundation Trust London & Surrey, Surrey, UK.

The possibility of targeting tumor angiogenesis was postulated almost 40 years

ago. The vascular endothelial growth factor (VEGF) family and its receptors have since been characterized and extensively studied. VEGF overexpression is a common finding in solid tumors, including esophagogastric cancer, and frequently correlates with poor prognosis. Monoclonal antibodies, soluble receptors, and small-molecule tyrosine kinase inhibitors have been developed to inhibit tumor angiogenesis, and antiangiogenic therapy is now a component of standard treatment for advanced renal cell, hepatocellular, colorectal, breast, and non-small cell lung carcinomas. The small-molecule tyrosine kinase inhibitors sunitinib and sorafenib have been evaluated in phase II studies in esophagogastric cancer but appear to have only modest activity. Similarly, despite promising efficacy signals from phase II studies, the addition of the anti-VEGF-A monoclonal antibody bevacizumab to cisplatin plus capecitabine failed to result in a longer overall survival duration than with the chemotherapy doublet plus placebo. The response rate and progression-free survival interval were significantly greater with bevacizumab, confirming some efficacy in advanced gastric cancer, but with inadequate benefit to justify the high cost of treatment. Evaluation of bevacizumab in the neoadjuvant and perioperative settings continues, hypothesizing that a higher response rate will translate into longer survival in patients with operable disease. Despite extensive research, the discovery of a reliable predictive biomarker for antiangiogenic therapy continues to elude the scientific and oncology communities, and mechanisms of primary and acquired resistance are incompletely understood. We are therefore currently unable to personalize antiangiogenic therapy for established indications, or use molecular selection for clinical trials evaluating novel indications.

PMCID: PMC3228215
PMID: 21632459 [PubMed - indexed for MEDLINE]

15. Int J Cancer. 2012 Apr 1;130(7):1706-13. doi: 10.1002/ijc.26180. Epub 2011 Aug 5.

Pathological complete remission in patients with oesophagogastric cancer receiving preoperative 5-fluorouracil, oxaliplatin and docetaxel.

Homann N(1), Pauligk C, Luley K, Werner Kraus T, Bruch HP, Atmaca A, Noack F, Altmannsberger HM, Jäger E, Al-Batran SE.

Author information:

(1)Department of Medicine II, Klinikum Wolfsburg, Wolfsburg, Germany.

The aim of this study was to determine the pathological complete remission (pCR) rate, and its relationship to clinical outcome, in patients with adenocarcinoma of the stomach or oesophagogastric junction receiving preoperative 5-fluorouracil, leucovorin, oxaliplatin and docetaxel (FLOT) every 2 weeks. Data from these patients who received at least one cycle of preoperative FLOT followed by surgery were prospectively collected in three German centres. Outcome analyses were conducted and tumour samples were evaluated for pathological remission by a central pathologist. A total of 46 patients were included in this analysis. All patients had clinical T3- and/or N+-stages and 11 (23.9%) had distant metastases (M1). After a median of 4 (range 2-8) preoperative cycles, 8 of 46 patients (17.4%) achieved a pCR. The pCR rate was highest in tumours of intestinal type histology (30.8%) and in those located in the oesophagogastric junction (30.4%) and lowest in patients with diffuse/mixed type tumours (0%) or tumours located in the stomach (4.3%; $p < 0.05$ for both comparisons). Patients with pCR had 100% probability of overall and disease-free survival (DFS) during the observation period, which was significantly higher ($p = 0.037$ and $p = 0.009$, respectively) than the survival probability in patients without pCR. In conclusion, treatment intensification using FLOT was associated with significant pCR rates in patients with oesophagogastric cancer. The distribution of pCR appeared to be significantly different according to histological type and location of the tumours.

Copyright © 2011 UICC.

PMID: 21618509 [PubMed - indexed for MEDLINE]

16. Br J Cancer. 2011 Jun 7;104(12):1840-7. doi: 10.1038/bjc.2011.175. Epub 2011 May
24.

Recurrence and survival after pathologic complete response to preoperative therapy followed by surgery for gastric or gastrooesophageal adenocarcinoma.

Fields RC(1), Strong VE, Gönen M, Goodman KA, Rizk NP, Kelsen DP, Ilson DH, Tang LH, Brennan MF, Coit DG, Shah MA.

Author information:
(1)Gastric and Mixed Tumor Service, Department of Surgery, Memorial
Sloan-Kettering Cancer Center, New York, NY 10065, USA.

BACKGROUND: To characterise recurrence patterns and survival following pathologic complete response (pCR) in patients who received preoperative therapy for localised gastric or gastrooesophageal junction (GEJ) adenocarcinoma.
METHODS: A retrospective review of a prospective database identified patients with pCR after preoperative chemotherapy for gastric or preoperative chemoradiation for GEJ (Siewert II/III) adenocarcinoma. Recurrence patterns, overall survival, recurrence-free survival, and disease-specific survival were analysed.
RESULTS: From 1985 to 2009, 714 patients received preoperative therapy for localised gastric/GEJ adenocarcinoma, and 609 (85%) underwent a subsequent R0 resection. There were 60 patients (8.4%) with a pCR. Median follow-up was 46 months. Recurrence at 5 years was significantly lower for pCR vs non-pCR patients (27% and 51%, respectively, P=0.01). The probability of recurrence for patients with pCR was similar to non-pCR patients with pathologic stage I or II disease. Although the overall pattern of local/regional (LR) vs distant recurrence was comparable (43% LR vs 57% distant) between pCR and non-pCR groups, there was a significantly higher incidence of central nervous system (CNS) first recurrences in pCR patients (36 vs 4%, P=0.01).
CONCLUSION: Patients with gastric or GEJ adenocarcinoma who achieve a pCR following preoperative therapy still have a significant risk of recurrence and cancer-specific death following resection. One third of the recurrences in the pCR group were symptomatic CNS recurrences. Increased awareness of the risk of CNS metastases and selective brain imaging in patients who achieve a pCR following preoperative therapy for gastric/GEJ adenocarcinoma is warranted.

PMCID: PMC3111205
PMID: 21610705 [PubMed - indexed for MEDLINE]

17. Ann Surg Oncol. 2011 Dec;18(13):3726-31. doi: 10.1245/s10434-011-1770-8. Epub 2011 May 17.

Neoadjuvant intraperitoneal and systemic chemotherapy for gastric cancer patients with peritoneal dissemination.

Fujiwara Y(1), Takiguchi S, Nakajima K, Miyata H, Yamasaki M, Kurokawa Y, Okada K, Mori M, Doki Y.

Author information:
(1)Department of Gastroenterological Surgery, Graduate School of Medicine, Osaka University, Suita, Osaka, Japan. yfujiwara@gesurg.med.osaka-u.ac.jp

Comment in
Ann Surg Oncol. 2011 Dec;18 Suppl 3:S189; author reply S187-8.

BACKGROUND: The present study was designed to assess the feasibility and efficiency of intraperitoneal and intravenous neoadjuvant chemotherapy in gastric cancer patients with peritoneal dissemination.
METHODS: The study subjects were 25 treatment-naïve patients with gastric cancer. Patients with positive cytology or with peritoneal carcinomatosis received neoadjuvant intraperitoneal and systemic chemotherapy (NIPS), comprising intraperitoneal (i.p.) mitomycin C (MMC) and cisplatin (CDDP), followed by two cycles of intravenous triplet chemotherapy of docetaxel, 5-fluorouracil (5-FU), and CDDP. Gastrectomy with lymph node dissection was performed after NIPS in patients free of peritoneal deposits, confirmed by staging laparoscopy.
RESULTS: Seventeen patients had measurable lymph node metastases by the RECIST criteria. CT examination showed response to the treatment in ten (59%, 0 complete response, 10 partial response). Of the 25 patients, 14 (56%) showed negative results on peritoneal cytology with no macroscopic peritoneal metastasis, whereas the remaining 11 were cancer cell-positive on peritoneal cytology or macroscopic peritoneal metastasis even after NIPS. The median survival time for all 25 patients was 16.7 months. Prognosis was better in patients who showed negative cytology and disappearance of peritoneal cancer metastases after NIPS than in those with positive cytology or existing peritoneal deposits ($P < 0.0001$). The predominant toxicity was myelosuppression and grade 3-4 leukopenia and neutropenia occurred in 20 (80%) patients, which were manageable. No treatment-related mortality was observed during and after NIPS and surgery.
CONCLUSIONS: The results of this prospective phase II study indicated that the newly designed NIPS was highly effective and well tolerated in patients with advanced gastric cancer and peritoneal dissemination.

PMID: 21584835 [PubMed - indexed for MEDLINE]

18. Zhonghua Zhong Liu Za Zhi. 2011 Apr;33(4):295-8.

[Comparison between the effects of irinotecan or oxaliplatin combined with capecitabine in the treatment of advanced gastric cancer].

[Article in Chinese]

Zhao WY(1), Chen DY, Qi Q.

Author information:
(1)Department of Medical Oncology, Yijishan Hospital, Wannan Medical College, Anhui Wuhu 241000, China. zhaowy98@yahoo.com.cn

OBJECTIVE: To observe and compare the response rate and toxicity of irinotecan or oxaliplatin combined with capecitabine in the treatment of advanced gastric cancer.
METHODS: Sixty-three patients with advanced gastric cancer were randomly divided into two groups. The CPT-11 + CAP group consisted of 32 patients who received irinotecan plus capecitabine: CPT-11 100 mg/m(2) was injected in 90 minutes on d 1, 8;capecitabine 2000 mg/m(2), bid, with the first dose in the evening of day 1 and last dose the morning of day 15, repeated for every 21 days. The L-OHP + CAP group consisted of 31 patients who received oxaliplatin plus capecitabine: oxaliplatin 100 mg/m(2) on day 1, capecitabine 2000 mg/m(2), bid, with the first dose in the evening of day 1 and last dose the morning of day 15, repeated for every 21 days. Two or more cycle chemotherapy was completed in each group.
RESULTS: In the CPT-11 + CAP group, no patient achieved complete response and 13 patients achieved partial response. The overall response rate was 40.6% (13/32), and the median progression-free survival time was 6.3 months. In the L-OHP + CAP group, no patient achieved complete response and 12 patients achieved partial response. The overall response rate was 38.7% (12/31), and the median progression-free survival time was 6.1 months. There was no significant difference between them ($P > 0.05$). The most common toxicities were gastrointestinal reaction, peripheral neuropathy and myelosuppression in the two groups. Patients in CPT-11 + CAP group experienced more III/IV diarrhea (28.1%/3.2%, $P = 0.018$). On the contrary, the rate of III/IV neurotoxicity in the group B was higher (25.8%/3.1%, $P = 0.027$). No chemotherapy-related death occurred.
CONCLUSION: The therapeutic effects of irinotecan or oxaliplatin combined with capecitabine in the treatment of advanced gastric cancer are good and comparable,

and their toxicities are tolerable.

PMID: 21575503 [PubMed - indexed for MEDLINE]

19. Gan To Kagaku Ryoho. 2011 May;38(5):823-6.

[A case of stage III B advanced gastric cancer with psoriasis vulgaris responding to S-1/CDDP neoadjuvant chemotherapy leading to a pathologically complete response].

[Article in Japanese]

Onozawa H(1), Ishii Y, Yamada M, Okada R, Takenoshita S.

Author information:
(1)Dept. of Surgery, Ota Nishinouchi Hospital.

A 39-year-old man with psoriasis vulgaris who complained of severe anemia was examined and diagnosed with advanced gastric cancer (UM, Type 3, cT3 cN2 cH0 cP0 cM0, cStage III B). He was treated with S-1/CDDP as neoadjuvant chemotherapy. S-1 (120mg/day) was administered orally for 14 days, followed by 7 drug-free days as a course, and CDDP (100mg/ body) was administered by intravenous drip on day 8. After the third course, a significant tumor reduction was obtained. Total gastrectomy and lymph node dissection (D2) were performed. The histological diagnosis revealed a complete disappearance of cancer cells in the stomach and all of the lymph nodes. He has been doing well without any recurrence for 9 months since the start of treatment.

PMID: 21566446 [PubMed - indexed for MEDLINE]

20. J Thorac Oncol. 2011 Jun;6(6):1121-7. doi: 10.1097/JTO.0b013e31821529a9.

Preoperative taxane-based chemotherapy and celecoxib for carcinoma of the esophagus and gastroesophageal junction: results of a phase 2 trial.

Altorki NK(1), Christos P, Port JL, Lee PC, Mirza F, Spinelli C, Keresztes R, Beneck D, Paul S, Stiles BM, Zhang Y, Schrump DS.

Author information:
(1)Thoracic Surgery Division, Department of Cardiothoracic Surgery, New York Presbyterian Hospital, Weill Cornell Medical College, New York City, New York 10021, USA. nkaltork@med.cornell.edu

PURPOSE: The primary objective of this study was to determine the rate of pathological response after preoperative celecoxib and concurrent taxane-based chemotherapy in patients with cancer of the esophagus and gastroesophageal junction.
METHODS: Thirty-nine patients were enrolled in this single-arm, phase II clinical trial. Patients were administered daily celecoxib in combination with two to three cycles of carboplatin and paclitaxel with preoperative intent. Levels of cyclooxygenase (COX)-2 expression in resected tumors were analyzed by immunohistochemistry and correlated with clinical outcome measures. Postoperatively, patients were administered daily celecoxib for 1 year or until documented tumor recurrence.
RESULTS: All patients received two to three cycles of chemotherapy plus celecoxib 800 mg/d. Toxicities were as expected. A major clinical response (complete response + partial response) was noted in 22 patients (56%); six patients (15%) had a complete clinical response. Thirty-seven patients underwent esophagectomy. Five patients had a major pathological response (12.8%). Four-year overall and disease-free survivals were 40.9% and 30.3%, respectively. Patients with tumors expressing COX-2 demonstrated a higher likelihood of a major clinical response response (62% versus 50%) and an improved overall survival, compared with patients with COX-2-negative tumors.
CONCLUSIONS: Preoperative celecoxib with concurrent chemotherapy demonstrated sufficient effect on pathologic response to warrant further study. Patients with tumors expressing COX-2 demonstrated trends toward improved response to preoperative therapy and improved overall survival compared with nonexpressors.

PMID: 21532508 [PubMed - indexed for MEDLINE]

21. Gan To Kagaku Ryoho. 2011 Apr;38(4):655-7.

[A case of effective weekly paclitaxel administration for advanced gastric cancer].

[Article in Japanese]

Hasegawa Y(1), Kuroda F, Doi T, Takemura S, Nakamura H.

Author information:
(1)Dept. of Surgery, Shirakawa Kosei General Hospital, Japan.

A 74-year-old man was admitted to our hospital with severe anemia. Endoscopic examination revealed a type 3 advanced gastric cancer. Abdominal computed tomography revealed massive lymph node metastasis(N3). We performed distal gastrectomy(noncurative resection)because anemia had progressed. After operation, we administered S-1/CDDP combination chemotherapy. Although he received two courses of S-1/CDDP, renal dysfunction was found. Afterwards a rise in tumor marker(CEA)occurred, so we changed to chemotherapy with weekly paclitaxel. Abdominal computed tomography then revealed a complete response(CR)after the chemotherapy was completed. At present, 7 years after the operation, the patient remains free of a rise in tumor markers and presents no evidence of a recurrence.

PMID: 21498999 [PubMed - indexed for MEDLINE]

22. Gan To Kagaku Ryoho. 2011 Apr;38(4):647-9.

[Weekly paclitaxel therapy leading to complete disappearance of multiple liver metastases and subsequent surgical resection for advanced gastric cancer].

[Article in Japanese]

Jin Y(1), Hasuo K, Tsuchida K, Sawazaki S, Hayashi S, Rino Y, Masuda M.

Author information:
(1)Dept. of Surgery, Hadano Red Cross Hospital, Japan.

A 59-year-old man visited our hospital. After examination he was diagnosed with advanced gastric cancer with multiple liver metastases. At first, chemotherapy of S-1/CDDP was administered. After two weeks, he had severe diarrhea and anorexia, so the therapy was discontinued. Weekly paclitaxel was selected as the next therapy. Depending on the leukocytopenia, doses and intervals were controlled. After 4 courses, CT and MRI revealed that the liver metastases had disappeared,

while the primary lesion remained. Seven months after beginning treatment, distal gastrectomy was performed. After the operation, 6 courses of paclitaxel therapy were given at the same doses and the same intervals as before the operation. Two years have passed since the operation, and no recurrence was seen on CT and MRI. Because of the relatively low frequency of adverse events in the digestive system, continued treatment with paclitaxel is possible. These findings show that paclitaxel is an effective drug for advanced gastric cancer with liver metastasis.

PMID: 21498997 [PubMed - indexed for MEDLINE]

23. Anticancer Res. 2011 Mar;31(3):1019-22.

A case of complete response to S-1 plus CDDP in early-stage mucosal esophageal cancer.

Takayama Y(1), Kochi M, Fujii M, Kanamori N, Kaiga T, Mihara Y, Miyazaki T, Tamegai H, Watanabe M, Takayama T.

Author information:
(1)Department of Digestive Surgery, Nihon University School of Medicine, 30-1 Ohyaguchi Kamimachi, Itabashi-ku, Tokyo 173-8610, Japan
takayuri@med.nihon-u.ac.jp

We report a case of early-stage mucosal esophageal cancer, showing a complete response to S-1 and cis-diamminedichloplatinum (CDDP). The patient was a 67-year-old man with synchronous double primary early-stage mucosal esophageal and advanced gastric cancer. We planned neoadjuvant chemotherapy with S-1 and CDDP for the advanced gastric cancer and endoscopic mucosal resection for the early-stage esophageal cancer. After the first course of chemotherapy, the endoscopy revealed that the esophageal cancer had become a normal mucosal lesion, and the biopsy was negative for cancer. We diagnosed a complete response to S-1 and CDDP in early-stage esophageal cancer. After two courses of chemotherapy, distal gastrectomy was performed. The patient is still alive with no sign of recurrence at 16 months after the disappearance of the original tumor. These results suggest that chemotherapy with S-1 plus CDDP may be effective in early-stage esophageal cancer.

PMID: 21498731 [PubMed - indexed for MEDLINE]

24. Zentralbl Chir. 2013 Dec;138 Suppl 2:e99-100. doi: 10.1055/s-0031-1271363. Epub 2011 Apr 6.

[Palliative abdominothoracal resection for stent-induced perforation of the oesophagus in a patient with recurrent metastasised gastric cancer with complete remission--a case report].

[Article in German]

Dittmar Y(1), Rauchfuss F(1), Schmidt C(2), Settmacher U(1).

Author information:
(1)Universitätsklinikum Jena, Klinik für Allgemein-, Viszeral- und Gefäßchirurgie, Jena, Deutschland. (2)Universitätsklinikum Jena, Klinik für Innere Medizin II, Abtlg. für Gastroenterologie, Hepatologie und Infektiologie, Jena, Deutschland.

PMID: 21472670 [PubMed - indexed for MEDLINE]

25. Hepatogastroenterology. 2010 Nov-Dec;57(104):1650-4.

Neoadjuvant chemotherapy combining docetaxel, cisplatin and S-1 in gastric cancer with para-aortic lymph node metastases: report of five cases.

Fushida S(1), Fujimura T, Oyama K, Kinoshita J, Fujita H, Ninomiya I, Ohta T.

Author information:
(1)Department of Gastroenterological Surgery, Kanazawa University Hospital, 13-1 Takara-machi, Kanazawa 920-8641, Japan. fushida@staff.kanazawa-u.ac.jp

We report on five patients with a median age of 56 years (range, 53-68 years) who were diagnosed as advanced gastric cancer with para-aortic lymph node metastases, determined by gastrofiberscope and abdominal spiral computed tomography. These patients received intravenous docetaxel (30 mg/m2) and cisplatin (30 mg/m2) on day 1, 15 and oral S-1 (40 mg/m2 bid) on days 1-14 every 4 weeks. After two

cycles of neoadjuvant chemotherapy, all patients received total gastrectomy with D2 lymphadenectomy plus para-aortic lymph nodes dissection. No patient revealed hematological or non-hematological toxicities associated with the chemotherapy (more than grade 2). The postoperative courses were uneventful without major surgery-related complications. Pathological complete response was confirmed one patient in primary lesion and three patients in metastatic lymph nodes including para-aortic lymph nodes. All of five patients are alive without recurrence (median: 36 months, 21-46 months). Thus, neoadjuvant chemotherapy described here may be rather promising regimen for advanced gastric cancer with para-aortic lymph node metastases, considering its low grade toxicities and pathological responses.

PMID: 21443137 [PubMed - indexed for MEDLINE]

26. Cancer Epidemiol Biomarkers Prev. 2011 May;20(5):1021-7. doi: 10.1158/1055-9965.EPI-10-1080. Epub 2011 Mar 10.

MET expression and amplification in patients with localized gastric cancer.

Janjigian YY(1), Tang LH, Coit DG, Kelsen DP, Francone TD, Weiser MR, Jhanwar SC, Shah MA.

Author information:
(1)Gastrointestinal Oncology Service, Department of Medicine, Memorial Sloan-Kettering Cancer Center, Weill Medical College of Cornell University, New York, NY 10065, USA. janjigiy@mskcc.org

BACKGROUND: MET, the receptor for hepatocyte growth factor, has been proposed as a therapeutic target in gastric cancer. This study assessed the incidence of MET expression and gene amplification in tumors of Western patients with gastric cancer.
METHODS: Tumor specimens from patients enrolled on a preoperative chemotherapy study (NCI 5700) were examined for the presence of MET gene amplification by FISH, MET mRNA expression by quantitative PCR, MET overexpression by immunohistochemistry (IHC), and for evidence of MET pathway activation by phospho-MET (p-MET) IHC.
RESULTS: Although high levels of MET protein and mRNA were commonly encountered (in 63% and 50% of resected tumor specimens, respectively), none of these tumors

had MET gene amplification by FISH, and only 6.6% had evidence of MET tyrosine kinase activity by p-MET IHC.
CONCLUSIONS: In this cohort of patients with localized gastric cancer, the presence of high MET protein and RNA expression does not correlate with MET gene amplification or pathway activation, as evidenced by the absence of amplification by FISH and negative p-MET IHC analysis.
IMPACT: This article shows a lack of MET amplification and pathway activation in a cohort of 38 patients with localized gastric cancer, suggesting that MET-driven gastric cancers are relatively rare in Western patients.

©2011 AACR.

PMCID: PMC3690490
PMID: 21393565 [PubMed - indexed for MEDLINE]

27. Gan To Kagaku Ryoho. 2011 Jan;38(1):101-4.

[Histological complete response in a case of advanced gastric cancer treated by neoadjuvant TS-1 combined with CDDP therapy].

[Article in Japanese]

Tanaka K(1), Kobayashi S, Fujita Y, Nakamura J, Kaneko K, Nakagawa K, Miyazaki M.

Author information:
(1)Dept. of Surgery, Saitama Red Cross Hospital, Japan.

A 63-year-old woman with advanced gastric cancer was referred to our hospital.Upper gastrointestinal endoscopy revealed a type 2 tumor in the lesser curvature of the angle of stomach. Moderately-differentiated adenocarcinoma was found in the biopsy specimens. Lymph node metastases of No.3 and No.7 were suspected by abdominal CT. We diagnosed the tumor as cStage III A (cT2N2H0P0M0) gastric cancer.For better curability, we selected neoadjuvant chemotherapy with TS-1/ CDDP. Two courses were completed without serious side effects. Upper gastrointestinal endoscopy and abdominal CT revealed that the primary tumor and metastatic lymph nodes had become smaller, suggesting that a partial response had been achieved. The patient underwent curative surgery, including distal gastrectomy and D2 lymph node dissection.No cancer cells were found by

pathological evaluation of the resected stomach and all the regional lymph nodes, confirming a pathological complete response. It is suggested that the neoadjuvant chemotherapy is a useful therapeutic strategy for advanced gastric cancer.

PMID: 21368467 [PubMed - indexed for MEDLINE]

28. Am J Clin Oncol. 2012 Feb;35(1):64-7. doi: 10.1097/COC.0b013e318201a126.

Neoadjuvant paclitaxel poliglumex, cisplatin, and radiation for esophageal cancer: a phase 2 trial.

Dipetrillo T(1), Suntharalingam M, Ng T, Fontaine J, Horiba N, Oldenburg N, Perez K, Birnbaum A, Battafarano R, Burrows W, Safran H.

Author information:
(1)The Brown University Oncology Group, Providence, Rhode Island, USA.

PURPOSE: To evaluate the pathologic complete response (CR) rate and safety of paclitaxel poliglumex (PPX), cisplatin, and concurrent radiation for patients with esophageal cancer.
PATIENTS AND METHODS: Patients with adenocarcinoma or squamous cell carcinoma of the esophagus or gastroesophageal junction with no evidence of distant metastasis received PPX (50 mg/m(2)/wk) and cisplatin (25 mg/m(2)/wk) for 6 weeks with 50.4 Gy concurrent radiation. Six to eight weeks after completion of chemoradiotherapy, patients underwent surgical resection.
RESULTS: Forty patients were enrolled, 37 patients with adenocarcinoma and 3 patients with squamous cell cancer. The treatment-related grade 3 nonhematologic toxicities included esophagitis (7%), nausea (7%), and fatigue (5%). Three patients with clinical endoscopic CR (2 with squamous cell cancer) refused surgery. Twelve of the remaining 37 patients (32%) had a pathologic CR. The 12 patients with pathologic CR all had adenocarcinoma.
CONCLUSION: PPX, cisplatin, and concurrent radiation are well tolerated, easily administered regimen for esophageal cancer with a low incidence of significant esophagitis and a high pathologic CR rate consistent with the preclinical data of PPX and radiation.

PMID: 21297434 [PubMed - indexed for MEDLINE]

29. Gan To Kagaku Ryoho. 2010 Nov;37(12):2430-2.

[A case of AGC with pCR after preoperative chemotherapy including S-1 plus cisplatin].

[Article in Japanese]

Funaki H(1), Ohnishi T, Ohno Y, Tomita Y, Hosokawa K, Yokoi M, Yoshitani S, Kinami S, Omote K, Ueda N, Nakano Y, Kosaka T.

Author information:
(1)Dept. of Surgical Oncology, Kanazawa Medical University.

A 79-year-old man complaining of epigastralgia was examined and diagnosed with advanced gastric cancer (UML, Type 5, Ant-Less-Gre, cT4a, cN1, cH0, cP1, cStage IV). A poor prognosis was predicted, but we tried preoperative chemotherapy hoping for a down-staging of the tumor. We chose a regimen of S-1 plus cisplatin as follows: S-1 (60 mg/m2) was administered orally for 3 weeks followed 2 weeks of rest, and cisplatin (50 mg/m2) was administered by intravenous drip on day 8. After three cycles of treatment, diagnostic laparoscopic examination revealed a suspected serosal invasion of the main tumor, but peritoneal dissemination was not seen, and abdominal washing cytology was negative. After the fourth cycle of treatment, total gastrectomy with lymph node dissection (D1+No. 7, 8a, 9, R0) was performed. Histological examination of the resected specimens revealed no residual cancer cells in the primary lesion or regional lymph nodes, resulting in a diagnosis of complete response to chemotherapy according to the Japanese Classification of Gastric Carcinoma. The postoperative course was uneventful, and he has been fine as an outpatient.

PMID: 21224596 [PubMed - indexed for MEDLINE]

30. Gan To Kagaku Ryoho. 2010 Nov;37(12):2334-6.

[Long-term survival of patient with gastric cancer treated by S-1 + paclitaxel combination chemotherapy against multiple liver metastases after gastrectomy and adrenalectomy].

[Article in Japanese]

Shimizu H(1), Itokawa Y, Shioaki Y, Sonoda H, Ikeda J, Koide K, Taniguchi F, Ueshima Y, Takashina K, Otsuji E.

Author information:
(1)Division of Digestive Surgery, Dept. of Surgery, Kyoto Prefectural University of Medicine.

The patient was a 74-year-old man whose chief complaint was epigastralgia. A detailed examination revealed a gastric cancer located from antrum to duodenal bulb with multiple liver metastases. Because of a difficulty with oral intake, we performed a distal gastrectomy at first. After the operation, a combination chemotherapy with S-1 and weekly paclitaxel was performed, and liver metastases were successfully disappeared after 4 courses of the regimen. A subsequent CT evaluation after 6 courses of the regimen revealed that liver metastases maintained the clinical complete response (cCR), but a right adrenal tumor was detected. We performed a right adrenalectomy after 13 months from gastrectomy, and a histopathological examination revealed that the adrenal tumor was a recurrent gastric cancer. After the second operation, only one course treatment of S-1 alone was performed because the patient rejected the chemotherapy. The patient is alive without a chemotherapy and maintained cCR for 75 months after the second operation.

PMID: 21224564 [PubMed - indexed for MEDLINE]

31. Virchows Arch. 2011 Mar;458(3):363-9. doi: 10.1007/s00428-010-1034-1. Epub 2010 Dec 30.

An unusual and potentially misleading phenotypic change in a primary gastrointestinal stromal tumour (GIST) under imatinib mesylate therapy.

Vassos N(1), Agaimy A, Schlabrakowski A, Hohenberger W, Schneider-Stock R, Croner RS.

Author information:
(1)Department of Surgery, University Hospital, Erlangen, Germany.

We present a unique case of a 62-year-old female who was diagnosed with a huge gastric gastrointestinal stromal tumour (GIST). Core needle biopsy revealed a cellular spindle cell GIST with diffuse expression of CD117 and CD34. Four mitotic figures were counted in ten available HPFs, indicating a high-risk tumour. Computed tomography scan, performed after 8 months of neoadjuvant imatinib mesylate treatment (Glivec, 400 mg/day), revealed a partial response with reduction of tumour size from 20 × 15 × 15 cm to 13.3 × 8 × 7.6 cm. The patient underwent complete tumour resection. The tumour revealed extensive cystic changes and hyalinisation in 90% of the tumour mass. Multiple viable tumour clones, measuring up to 1 cm, showed highly anaplastic, large epithelioid cells with vesicular nuclei and prominent, centrally located nucleoli, strikingly mimicking the appearance of proximal-type epithelioid sarcoma, anaplastic carcinoma, melanoma or epithelioid angiosarcoma. These anaplastic tumour cells expressed pankeratin (KL-1) and vimentin, but they were completely negative for CD117, DOG-1, CD34, S100, desmin, α-smooth muscle actin, HMB45, CD30, CD45, CK7, CK20 and 34βE-12. Sufficient tissue for molecular analysis was available from the resected tumour. No mutations were detected in KIT exons 9, 11, 13, 17, PDGFRA exons 12, 14, 18, KRAS and BRAF. The patient was alive with no evidence of recurrence 28 months later. To our knowledge, this represents the first report on this unusual type of trans-differentiation in GIST under imatinib therapy. Awareness of this phenomenon would help to avoid diagnostic confusion when evaluating post-treatment resections from GISTs.

PMID: 21191613 [PubMed - indexed for MEDLINE]

32. Leuk Lymphoma. 2011 Jan;52(1):42-5. doi: 10.3109/10428194.2010.534519. Epub 2010
Dec 6.

90Y-ibritumomab tiuxetan (Zevalin) in heavily pretreated patients with mucosa associated lymphoid tissue lymphoma.

Hoffmann M(1), Troch M, Eidherr H, Traub-Weidinger T, Jonak C, Muellauer L, Raderer M.

Author information:
(1)Department of Nuclear Medicine, Medical University Vienna, Vienna, Austria.

Comment in
    Leuk Lymphoma. 2011 Feb;52(2):159-60.

Radioimmunotherapy using (90)Y-ibritumomab tiuxetan has predominantly been used in patients with follicular lymphoma, but little is known about its activity in patients with extranodal marginal zone lymphoma of the mucosa associated lymphoid tissue (MALT). A total of six patients progressing/relapsing following conventional therapy for MALT lymphoma were treated with (90)Y-ibritumomab tiuxetan at our institution. Two patients had gastric MALT lymphoma, one suffered from orbital MALT lymphoma, and two had cutaneous MALT lymphoma, while one patient had a widely disseminated lymphoma involving the stomach, lungs, lymph nodes, and salivary glands. All patients were at least in third relapse following various forms of therapy including Helicobacter pylori-eradication, radiation, chemotherapy, and application of rituximab. Following two doses of rituximab at 250 mg/m(2) at an interval of 1 week, (90)Y-ibritumomab tiuxetan was given immediately at a dose of 0.4 mCi/kg body weight. Treatment was well tolerated apart from one episode of pneumonia requiring hospitalization. Four patients developed a complete remission (ongoing now for 4, 16, 23, and 24 months), one patient had a partial response lasting for 5 months, and one patient had stable disease for 13 months. After a follow-up of 9-29 months, all patients are alive. Application of (90)Y-ibritumomab tiuxetan is active and safe in heavily pretreated patients with MALT lymphoma.

PMID: 21133720 [PubMed - indexed for MEDLINE]

33. Tumour Biol. 2011 Apr;32(2):335-46. doi: 10.1007/s13277-010-0126-5. Epub 2010 Nov
27.

The G1 phase arrest and apoptosis by intrinsic pathway induced by valproic acid inhibit proliferation of BGC-823 gastric carcinoma cells.

Zhao X(1), Yang W, Shi C, Ma W, Liu J, Wang Y, Jiang G.

Author information:
(1)The Central Laboratory, Qianfoshan Hospital of Shandong Province, Jinan 250014, China.

Recent studies have demonstrated that the histone deacetylation level was closely related to the genesis and development of tumors. Thus, activating histone acetyltransferases and/or suppressing histone deacetylases (HDACs) can become an approach for tumor chemotherapy. The histone acetylation regulation often results in the inhibition of cell proliferation, induction of cell apoptosis or differentiation, and cell cycle arrest in G1 phase. It has been demonstrated recently that the traditional anticonvulsant valproic acid was an efficient class I HDAC inhibitor (HDACI); however, its antitumor effect and mechanisms on gastric cancers so far has not been elucidated clearly. In the present study, gastric carcinoma cell lines BGC-823, HGC-27, and SGC-7901 were cultured with valproic acid (VPA) in vitro. The cell morphology was observed by invert microscope, the proliferation was detected by MTT assay, the apoptosis and cell cycle were analyzed by flow cytometry assay with Annexin V/PI and PI, the activities and protein expressions of Caspase 3, Caspase 8, Caspase 9 of BGC-823 cells were detected by spectrophotometry and indirect immunofluorescence technique, respectively. The protein expressions of Cyclin A, Cyclin D1, Cyclin E, P21(Waf/cip1) of BGC-823 cells were analyzed by indirect immunofluorescence assay, and messenger ribonucleic acid (mRNA) expressions were detected by RT-PCR assay. The results showed that the proliferation of three kinds of gastric carcinoma cells could be inhibited obviously by VPA, which was related to the apoptosis induction and cell cycle arrest in G1 phase. The intrinsic pathway (cytochrome C pathway) was chiefly involved in the mechanism of apoptosis, which was indicated by activation of Caspase 9 and Caspase 3. The extrinsic pathway was partially involved, with slight activation of Caspase 8. The mechanism underlying its effect on cell cycle arrest in G1 phase induction was due to the upregulation of P21(Waf/cip1), Mad1 expression and downregulation of Cyclin A, c-Myc expression.

PMID: 21113745 [PubMed - indexed for MEDLINE]

34. World J Gastroenterol. 2010 Nov 28;16(44):5621-8.

Neoadjuvant chemotherapy for advanced gastric cancer: a meta-analysis.

Li W(1), Qin J, Sun YH, Liu TS.

Author information:
(1)Department of Medical Oncology, Shanghai Zhongshan Hospital, Fudan University,

180 Feng Lin Road, Shanghai 200032, China.

Comment in
    World J Gastroenterol. 2011 Oct 28;17(40):4542-4.

AIM: To study the value of neoadjuvant chemotherapy (NAC) for advanced gastric cancer by performing a meta-analysis of the published studies.
METHODS: All published controlled trials of NAC for advanced gastric cancer vs no therapy before surgery were searched. Studies that included patients with metastases at enrollment were excluded. Databases included Cochrane Library of Clinical Comparative Trials, MEDLINE, Embase, and American Society of Clinical Oncology meeting abstracts from 1978 to 2010. The censor date was up to April 2010. Primary outcome was the odds ratio (OR) for improving overall survival rate of patients with advanced gastric cancer. Secondary outcome was the OR for down-staging tumor and increasing R0 resection in patients with advanced gastric cancer. Safety analyses were also performed. All calculations and statistical tests were performed using RevMan 5.0 software.
RESULTS: A total of 2271 patients with advanced gastric cancer enrolled in 14 trials were divided into NAC group (n = 1054) and control group (n = 1217). The patients were followed up for a median time of 54 mo. NAC significantly improved the survival rate [OR = 1.27, 95% confidence interval (CI): 1.04-1.55], tumor stage (OR = 1.71, 95% CI: 1.26-2.33) and R0 resection rate (OR = 1.51, 95% CI: 1.19-1.91) of patients with advanced gastric cancer. No obvious safety concerns were raised in these trials.
CONCLUSION: NAC can improve tumor stage and survival rate of patients with advanced gastric cancer with a rather good safety.

PMCID: PMC2992682
PMID: 21105197 [PubMed - indexed for MEDLINE]

35. Ann Thorac Surg. 2010 Dec;90(6):1805-11; discussion 1811. doi: 10.1016/j.athoracsur.2010.06.085.

Predictors of cervical and recurrent laryngeal lymph node metastases from esophageal cancer.

Stiles BM(1), Mirza F, Port JL, Lee PC, Paul S, Christos P, Altorki NK.

Author information:
(1)Division of Thoracic Surgery, New York Presbyterian Hospital, Weill Cornell Medical College, New York, New York 10021, USA.

BACKGROUND: Although patients with esophageal cancer (EC) often develop lymph node metastases in the cervical and recurrent laryngeal (CRL) distribution, lymphadenectomy in this field is rarely performed. The purpose of this study was to determine factors associated with CRL node positivity and to determine the appropriate indications to perform a "three field" lymphadenectomy.
METHODS: In a retrospective review, EC patients who underwent three-field lymphadenectomy were analyzed. Predictors of positive CRL nodes were examined univariately, then selected for inclusion in a multivariate logistic regression model.
RESULTS: From 1994 to 2009, 185 patients had a three-field lymphadenectomy, of whom 46 patients (24.9%) had positive CRL nodes. Final pathology stages (seventh edition) were I in 24 patients, II in 43, III in 109, and IV in 1 patient. Eight patients had a major pathologic response after induction therapy. On univariate analysis, variables significantly associated with positive CRL nodes included squamous cell histology, proximal location, advanced clinical presentation, the presence of clinical nodal disease, higher pT classification, and higher pN classification. There was no reduction in the rate of positive CRL nodes after induction chemotherapy. On multivariate analysis, higher pN classification (adjusted odds ratio 16.25, 95% confidence interval: 5.40 to 48.87; $p < 0.0001$) and squamous histology (adjusted odds ratio 6.04, 95% confidence interval: 2.21 to 16.56; $p < 0.0001$) predicted positive CRL nodes.
CONCLUSIONS: Complete lymphadenectomy is necessary in esophageal cancer to appropriately stage patients. Low rates of positive CRL nodes are present with early clinical stage, with pT0-2 tumors, and with pN0 classification, particularly in patients with adenocarcinoma and gastroesophageal junction tumors. Dissection of the CRL field should be considered with advanced disease for adenocarcinoma and in all patients with squamous cell cancer.

Copyright © 2010 The Society of Thoracic Surgeons. Published by Elsevier Inc. All rights reserved.

PMID: 21095315 [PubMed - indexed for MEDLINE]

36. J Thorac Oncol. 2010 Dec;5(12):1994-8. doi: 10.1097/JTO.0b013e3181fb5c3e.

Phase II study of preoperative pemetrexed, carboplatin, and radiation followed by surgery for locally advanced esophageal cancer and gastroesophageal junction tumors.

Jatoi A(1), Soori G, Foster NR, Hiatt BK, Knost JA, Fitch TR, Callister MD, Nichols FC 3rd, Husted TM, Alberts SR.

Author information:
(1)Department of Oncology, Mayo Clinic Rochester, Rochester, Minnesota 55905, USA. Jatoi.aminah@mayo.edu

INTRODUCTION: Based on favorable preliminary clinical data and the need to identify effective, well-tolerated neoadjuvant regimens for patients with locally advanced esophageal cancer, this clinical trial was undertaken.
METHODS: This phase II study tested 500 mg/m neoadjuvant pemetrexed intravenously and carboplatin with an area under the curve of 6 intravenously on days 1 and 22 in conjunction with concomitant radiation of 5040 centigray, which was given in 28 daily fractions of 180 centigray. The primary endpoint was the rate of pathologic complete response.
RESULTS: This trial closed early because, during an interim analysis, the primary endpoint fell short. However, 26 eligible patients were accrued. Twenty (74%) were men. Performance scores of 0, 1, and 2 were seen in 16 (59%), 9 (33%), and 2 (7%), respectively. Among eligible patients, 6 of 26 (23%; 95% confidence interval 9-44%) demonstrated a pathologic complete response. Twenty-two underwent a complete cancer resection. The median survival was 17.8 months (95% confidence interval: 12.2-30.7 months). In the neoadjuvant setting, 22 patients had at least one grade 3 or worse adverse event, and 8 patients had at least one grade 4 event. Postoperatively (within 30 days of surgery), there were three deaths, one grade 4 event (thrombosis), and three grade 3 events.
CONCLUSIONS: The neoadjuvant regimen tested within this phase II trial demonstrated antineoplastic activity but fell short of yielding a complete pathologic response rate that merits further testing.

PMID: 20975604 [PubMed - indexed for MEDLINE]

37. Gan To Kagaku Ryoho. 2010 Oct;37(10):1965-9.

[A case of locally advanced gastric cancer responding to pathological CR treated with S-1/CDDP neoadjuvant chemotherapy].

[Article in Japanese]

Kobayashi N(1), Mizuta M, Otani H, Kubo M, Udaka T, Shirakawa K.

Author information:
(1)Department of Surgery, Mitoyo General Hospital.

A 75-year-old woman was referred to our hospital because of locally advanced gastric cancer. Gastrointestinal fiberscopy revealed type 3 advanced gastric cancer in the posterior wall of the gastric cardia extending to the middle body. Abdominal CT scan revealed direct invasion of pancreas and regional lymph node metastases, indicating clinical stage IV (cT4N2H0P0M0). After two courses of S-1/CDDP, neoadjuvant chemotherapy was administered, and total gastrectomy with D2 lymphadectomy was performed. Histological examination revealed no residual cancer cells in the surgically obtained stomach and lymph nodes, suggesting a complete pathological response (Grade 3). She was treated with S-1 for one year after operation and presently, 16 months after operation, she is in good health without recurrence.

PMID: 20948265 [PubMed - indexed for MEDLINE]

38. Cancer Chemother Pharmacol. 2011 Jun;67(6):1435-43. doi:
10.1007/s00280-010-1444-4. Epub 2010 Sep 2.

Phase I/II study of a combination of docetaxel, capecitabine, and cisplatin (DXP) as first-line chemotherapy in patients with advanced gastric cancer.

Kang YK(1), Ryu MH, Yoo C, Chang HM, Yook JH, Oh ST, Kim BS, Kim TW.

Author information:
(1)Department of Oncology, Asan Medical Center, 86 Asanbyeongwon-gil, Songpa-gu, Seoul 138-736, South Korea. ykkang@amc.seoul.kr

BACKGROUND: This study was conducted to determine the optimal dosage of the docetaxel-capecitabine-cisplatin (DXP) regimen and to evaluate its efficacy and

safety in patients with advanced gastric cancer.
METHODS: Patients with advanced gastric or esophagogastric junctional adenocarcinoma received capecitabine (days 1-14) and intravenous docetaxel and cisplatin (day 1) every 3 weeks.
RESULTS: In the phase I study, 15 patients were treated with 4 different dose levels. Asthenia and neutropenic fever were the dose-limiting toxicities. For the phase II study, 1,125 mg/m(2) of capecitabine was initially recommended with 60 mg/m(2) docetaxel and 60 mg/m(2) cisplatin. However, frequent dose modifications at this dose level resulted in a final optimal dose of 937.5 mg/m(2) capecitabine. Among the 40 patients enrolled in the phase II study, 4 complete and 23 partial responses were observed, presenting objective response rate of 68%. Ten patients achieving good response with complete disappearance of distant metastases underwent surgery, and 4 pathologic complete responses were identified. After the median follow-up of 83.7 months (range, 20.2-86.5) in surviving patients, the median overall survival was 14.4 months and median progression-free survival was 7.6 months. The most frequent grade 3/4 adverse events were neutropenia (62.5%) and asthenia (37.5%). Ten per cent of the patients experienced neutropenic fever, with one case of sepsis-induced death.
CONCLUSION: DXP displays considerable antitumor activity, and may thus present effective first-line treatment for advanced gastric cancer. Further investigation of the efficacy and safety of this regimen in both first-line and neoadjuvant settings is warranted.

PMID: 20811894 [PubMed - indexed for MEDLINE]

39. Med Oncol. 2011 Dec;28 Suppl 1:S152-61. doi: 10.1007/s12032-010-9658-1. Epub 2010
Aug 21.

Long-term outcome of a phase II study of docetaxel-based multimodality chemoradiotherapy for locally advanced carcinoma of the esophagus or gastroesophageal junction.

Choong NW(1), Mauer AM, Haraf DC, Ferguson MK, Sandler AB, Kesler KA, Fishkin PA, Ansari RH, Wade J 3rd, Krauss SA, Sciortino DF, Posner MC, Kocherginsky M, Hoffman PC, Szeto L, Vokes EE.

Author information:

(1)Section of Hematology-Oncology, University of Chicago Medical Center, MC 2115, 5841, S Maryland Avenue, Chicago, IL 60637, USA. nchoong@mcw.edu

We performed a phase II trial to evaluate a docetaxel-based regimen in locoregionally advanced esophageal cancer. Untreated stage II-IVa esophageal cancer patients with performance status 0-2 were included. Tumor resectability was determined prior to initiation of study. Induction docetaxel (75 mg/m(2)) and cisplatin (75 mg/m(2)) day 1 with prophylactic filgrastim was delivered every 21 days for 3 cycles. Subsequent concomitant chemoradiotherapy (CRT) utilized weekly docetaxel (20 mg/m(2)) and concurrent radiotherapy (2 Gy/day) in resectable/resected patients (50 Gy) and in unresectable patients (66 Gy). A total of 78 patients (15 squamous cell carcinoma, 60 adenocarcinoma, 3 mixed/undifferentiated; 68 men, 10 women; median age 61 years) were accrued. The regimen was administered to 59 (76%) potentially resectable patients and 13 (17%) unresectable patients; 6 patients (8%) received the regimen post-operatively. Response rate in 66 evaluable patients following induction chemotherapy was 30%. Sixty-nine patients underwent CRT. Ten patients had disease progression during CRT. Forty-five out of 59 potentially resectable patients underwent esophagectomy after CRT, and 42 patients had complete tumor resection with negative margins. Eighteen out of 59 patients who were potentially resectable patients had pathologic complete response (pCR-31%). Grade 3/4 toxicity during induction chemotherapy included leucopenia, neutropenia, vomiting, and neuropathy. Esophagitis was the predominant toxicity during CRT. Median overall survival was 11.4 months for unresectable patients, 14.3 months for resectable patients and 10.4 months for patients who received the regimen post-operatively (log-rank P = 0.2492). Docetaxel-based CRT regimen is active and tolerable in esophageal cancer. The observed pCR in the potentially resectable group indicates good local control.

PMID: 20730572 [PubMed - indexed for MEDLINE]

40. Recent Results Cancer Res. 2010;182:179-91. doi: 10.1007/978-3-540-70579-6_15.

Molecular response prediction in multimodality treatment for adenocarcinoma of the esophagus and esophagogastric junction.

Lurje G(1), Lenz HJ.

Author information:
(1)Department of Visceral- and Transplantation Surgery, University Hospital of Zurich, Raemistrasse 100, 8091, Zurich, Switzerland. georg.lurje@usz.ch

Cancers arising from the esophagus are becoming more common in the United States and Europe. In 2009, an estimate of 14,530 new cases will be diagnosed and more than 90% will die of their disease. Esophageal cancer is currently the most rapidly increasing cancer in the western world and is coinciding with a shift in histological type and primary tumor location. Despite recent improvements in the detection, surgical resection, and (radio-) chemotherapy, the overall survival (OS) of esophageal cancer remains relatively poor. It is becoming increasingly apparent that neoadjuvant chemoradiation followed by surgery may be beneficial in terms of increasing resectability and OS compared to surgery alone. Results from clinical trials are encouraging; however, they also demonstrated that only patients with major histopathological response (pCR) will benefit from neoadjuvant therapy. In addition, these therapies are expensive and the prognoses of patients who do not respond to trimodality treatment strategies appear to be inferior to that of patients who had surgery alone. Accordingly, the development of validated predictive molecular markers may not only be helpful in identifying EA patients who are more likely to respond, but they will also be critical in selecting more efficient treatment strategies with the means of a tailored, targeted, and effective therapy to the molecular profile of both the patient and their disease while minimizing and avoiding life-threatening toxicities.

PMID: 20676881 [PubMed - indexed for MEDLINE]

41. Int J Radiat Oncol Biol Phys. 2011 Jul 1;80(3):698-704. doi: 10.1016/j.ijrobp.2010.02.054. Epub 2010 Jul 23.

Patterns of response after preoperative treatment in gastric cancer.

Díaz-González JA(1), Rodríguez J, Hernández-Lizoain JL, Ciérvide R, Gaztañaga M, San Miguel I, Arbea L, Aristu JJ, Chopitea A, Martínez-Regueira F, Valentí V, García-Foncillas J, Martínez-Monge R, Sola JJ.

Author information:
(1)Division of Radiation Oncology, Clínica Universidad de Navarra, Pamplona, Spain. jadiaz@unav.es

PURPOSE: To analyze the rate of pathologic response in patients with locally advanced gastric cancer treated with preoperative chemotherapy with and without chemoradiation at our institution.
METHODS AND MATERIALS: From 2000 to 2007 patients were retrospectively identified who received preoperative treatment for gastric cancer (cT3-4/ N+) with induction chemotherapy (Ch) or with Ch followed by concurrent chemoradiotherapy (45 Gy in 5 weeks) (ChRT). Surgery was planned 4-6 weeks after the completion of neoadjuvant treatment. Pathologic assessment was used to investigate the patterns of pathologic response after neoadjuvant treatment.
RESULTS: Sixty-one patients were analyzed. Of 61 patients, 58 (95%) underwent surgery. The R0 resection rate was 87%. Pathologic complete response was achieved in 12% of the patients. A major pathologic response (<10% of residual tumor) was observed in 53% of patients, and T downstaging was observed in 75%. Median follow-up was 38.7 months. Median disease-free survival (DFS) was 36.5 months. The only patient-, tumor-, and treatment-related factor associated with pathologic response was the use of preoperative ChRT. Patients achieving major pathologic response had a 3-year actuarial DFS rate of 63%.
CONCLUSIONS: The patterns of pathologic response after preoperative ChRT suggest encouraging intervals of DFS. Such a strategy may be of interest to be explored in gastric cancer.

Copyright © 2011 Elsevier Inc. All rights reserved.

PMID: 20656414 [PubMed - indexed for MEDLINE]

42. Eur J Surg Oncol. 2010 Jul;36(7):610-6. doi: 10.1016/j.ejso.2010.05.006.

Introducing national guidelines on perioperative chemotherapy for gastric cancer in Norway: a retrospective audit.

Hølmebakk T(1), Frykholm G, Viste A; Norwegian Gastrointestinal Cancer Group (NGICG).

Author information:
(1)Department of Surgical Gastroenterology, Oslo University Hospital, Aker, 0514 Oslo, Norway. toto.holmebakk@oslo-universitetssykehus.no

BACKGROUND: In 2006, perioperative chemotherapy with epirubicin, cisplatin/oxaliplatin, and capecitabine was recommended in the National Guidelines for patients with resectable gastric cancer in Norway. We conducted a national audit related to clinical aspects, local organisation and the implementation of this multimodal treatment.
PATIENTS AND METHODS: All Norwegian departments of oncology were asked to submit aggregated data on gastric cancer patients who had started perioperative chemotherapy for cure; departments of surgery were asked to report on patients undergoing resection after preoperative chemotherapy. Data were retrospectively collected.
RESULTS: All 20 departments of oncology and 20 of 21 departments of surgery responded. Of 336 patients operated on for gastric cancer and reported by surgeons, 144 (43%) received preoperative chemotherapy. 169 patients were reported by departments of oncology. 152 (90%) completed the preoperative cycles; 92 (54%) started the postoperative cycles; and 68 (40%) completed all cycles. Toxicity grade >or= 3, overall and haematological, increased during postoperative compared to preoperative cycles, 50 vs. 34% (P = 0.012) and 35 vs. 20% (P = 0.012), respectively. Surgical morbidity and mortality were 26 and <2%, respectively. R0 resection was achieved in 86% of surgically treated patients. Five per cent had a complete pathological response (ypT0) and 48% were node negative (ypN0). Within the first year, the National Guidelines were implemented in 19 of 25 hospitals (76%).
CONCLUSIONS: In this population-based series, the tolerability of perioperative chemotherapy reported in the MAGIC trial was reproduced. Toxicity grade >or= 3 was considerable and significantly increased related to postoperative cycles. The National Guidelines were rapidly adopted.

PMID: 20627647 [PubMed - indexed for MEDLINE]

43. Gan To Kagaku Ryoho. 2010 Jun;37(6):1125-9.

[A case of long-term survival after undergoing S-1 treatment and splenectomy for liver, lung and splenic metastases following curative distal gastrectomy for gastric cancer].

[Article in Japanese]

Ogura M(1), Tanaka N, Furuya T, Nomura Y, Nagai M, Takahashi M, Takayama T, Hirao

H, Nakamura H, Suzuki Y.

Author information:
(1)Department of Surgery, Asahi General Hospital.

A 7 0-year-old female underwent distal gastrectomy for gastric cancer in November 2001. She did not wish to receive postoperative adjuvant chemotherapy. In May 2002, her serum carcinoembryonic antigen(CEA)level rose. CT demonstrated liver(S5/6)and lung(S9)metastases in August 2002. We started to treat her with S-1(100mg/day day 1-14 orally), and restaging CT showed complete regression of liver and lung metastases in August 2003. In spite her complete response(CR), we continued S-1 treatment for the successive two years. No adverse reaction to chemotherapy occurred. Although CR was maintained for about 4 years, she was found to have a 9-mm solitary lesion in the upper pole of the spleen in June 2007. After 6 months, this tumor increased to 15mm in size, and we considered it as a solitary metastasis to the spleen from gastric cancer. S-1 chemotherapy was restarted, but tumor size gradually increased. Tumor size finally reached 25mm in December 2008. She underwent splenectomy in January 2009. From then until now, she has not received any chemotherapy, and has been followed well without any recurrence.

PMID: 20567121 [PubMed - indexed for MEDLINE]

44. Cancer. 2010 Sep 1;116(17):4023-32. doi: 10.1002/cncr.25349.

Phase 2 trial of preoperative irinotecan plus cisplatin and conformal radiotherapy, followed by surgery for esophageal cancer.

Knox JJ(1), Wong R, Visbal AL, Horgan AM, Guindi M, Hornby J, Xu W, Ringash J, Keshavjee S, Chen E, Haider M, Darling G.

Author information:
(1)Department of Medical Oncology, Princess Margaret and Toronto General Hospitals of the University Health Network, Toronto, Ontario, Canada.
jennfer.knox@uhn.on.ca

BACKGROUND: Esophagectomy for locally advanced esophageal cancer (LAEC) is associated with limited survival. Trimodality therapy yields a small survival

advantage, with cisplatin and 5-fluorouracil regimens most frequently studied. Newer regimens may impact these poor outcomes. This phase 2 trial assessed the feasibility and efficacy of induction chemoradiotherapy with cisplatin and irinotecan followed by esophagectomy.
METHODS: Patients with LAEC of the thoracic esophagus or gastroesophageal junction underwent chemotherapy with preoperative irinotecan (65 mg/m(2)) plus cisplatin (30 mg/m(2)) on Weeks 1, 2, 4, 5, 7, and 8 with concurrent conformal radiotherapy (40 grays [Gy]/20 fractions during Weeks 4-7) and external beam boost (10 Gy/5 fractions at Week 8). Esophagectomy was performed between Weeks 12 and 16. Pathologic response was the primary endpoint with follow-up data on progression, survival, and toxicity as secondary endpoints.
RESULTS: Fifty-two patients were enrolled from November 2002 to October 2005. Nineteen patients had American Joint Committee on Cancer stage II, 22 had stage III, and 11 had stage IVA disease. Grade 3 to 4 toxicity (graded according to the National Cancer Institute Common Toxicity Criteria 2.0) during induction included neutropenia (36%), febrile neutropenia (8%), diarrhea (10%), and esophagitis (4%). Three patients withdrew from treatment due to toxicity. There was 1 treatment-related death. Clinical responses included complete response in 2%, partial response in 30%, stable disease in 62%, and progressive disease in 6% of patients. Dysphagia improved/resolved in 72% of patients during induction. Forty-three patients underwent esophagectomy and 7 (16%) achieved pathologic complete responses. Median and 3-year overall survival for patients receiving trimodality therapy was 36 months and 51%, respectively.
CONCLUSIONS: In LAEC, concurrent irinotecan/cisplatin and radiotherapy followed by esophagectomy is reported to be associated with dysphagia improvement in 72% of patients, a significant but manageable toxicity profile, and encouraging survival compared with historic controls.

Cancer 2010. (c) 2010 American Cancer Society.

PMID: 20533506 [PubMed - indexed for MEDLINE]

45. Mol Imaging Biol. 2011 Feb;13(1):178-86. doi: 10.1007/s11307-010-0330-0.

Association of the VEGF 936C>T polymorphism with FDG uptake, clinical, histopathological, and metabolic response in patients with adenocarcinomas of the esophagogastric junction.

Lorenzen S(1), Panzram B, Keller G, Lordick F, Herrmann K, Becker K, Langer R, Schwaiger M, Siewert JR, Ott K.

Author information:
(1)National Center of Tumor Diseases, University of Heidelberg, Heidelberg, Germany.

PURPOSE: The MUNICON trial confirmed prospectively the usefulness of early response evaluation by 2-deoxy-2-[F-18]fluoro-D-glucose-positron emission tomography (FDG-PET) . Metabolic responders (R) showed initially a higher FDG uptake compared with nonresponders ($p = 0.018$). An association of the vascular endothelial growth factor (VEGF) 936C>T polymorphism and FDG uptake was reported for breast cancer. Therefore, we investigated the VEGF 936C>T polymorphism for an association with response and survival.
PROCEDURES: The study was based on 110 patients included in the MUNCON trial (103 male, seven female; 75 AEG I, 35 AEG II, event-free survival (EFS) median 21.1 ± 4.6 months). Response was significantly associated with EFS. The VEGF 936C>T polymorphism was determined by PCR and restriction fragment length polymorphism analysis. For analysis, the T-variants were combined.
RESULTS: One hundred two patients were evaluable. Seventy-two patients showed the CC, 24 the CT, and six the TT genotype. Median EFS was 29.3 months for CC and 11.7 months for CT/TT ($p = 0.04$). No association of the genotypes (CC or CT/TT) with the SUV or response was found. Multivariate analysis revealed histopathological regression ($p = 0.003$) and genotype ($p = 0.04$) as independent prognostic factors. A combination of genotype and PET response (Gen-PET) defines three prognostic groups early in the course of treatment ($p = 0.002$). Cox regression analysis including clinical and histopathological response and Gen-PET reveals Gen-PET as independent prognostic factor ($p = 0.003$).
CONCLUSION: The VEGF 936C>T polymorphism is a prognostic factor in patients undergoing neoadjuvant chemotherapy, although it is not associated with FDG uptake and response. The combination of metabolic response and VEGF 936C>T polymorphism defines three different prognostic groups. These findings need to be confirmed prospectively. This study has been registered in the European Clinical Trials Database as trial 2007-003356-11.

PMID: 20449668 [PubMed - indexed for MEDLINE]

46. G Chir. 2010 Apr;31(4):147-50.

[Preliminary experience in treatment of locally advanced gastric adenocarcinoma with peri-operative chemotherapy].

[Article in Italian]

Graziosi L(1), Bugiantella W, Gunnellini M, Qweider NA, Cavazzoni E, Donini A.

Author information:
(1)Università degli Studi di Perugia, "Ospedale Santa Maria della Misericordia", Perugia.

AIM: Gastric Cancer (GC) is a poor prognosis neoplasm and it is often diagnosed at advanced stage. Neoadjuvant Chemotherapy (NAC) may increase the possibility of complete surgical resection and improve Overall Survival (OS).
PATIENTS AND METHODS: Since November 2006, six patients with gastric adenocarcinoma, diagnosed by endoscopy, endoscopic ultrasonography and total body 18FDG-PET-CT, were enrolled in a program of peri-operative chemotherapy, according to the following inclusion criteria: cT2N+M0 or cT3-4NxM0, age < 75 years, Karnofsky Performance Status > 60%, absence of hepatic, renal and bone marrow failures. The patients undergo three cycles of pre-operative and post-operative chemotherapy based on Epirubicine, Cisplatin, and 5-Fluorouracil (ECF) as MAGIC Trial proposed. Fifteen days after the end of pre-operative chemotherapy the patient undergoes endoscopic ultrasonography and total body 18FDG-PET-CT to evaluate the tumor response to treatment, and then he is addressed to surgery. Thirty days after surgery he starts the post-operative chemotherapy.
RESULTS: All patients completed the pre-operative chemotherapy. Five patients underwent D2 subtotal gastrectomy and only one patient D1 total gastrectomy. No peri- and post-operative mortality and morbidity were observed. One month after surgery all patients started chemotherapy. During post-operative chemotherapy two patients developed subclavian vein thrombosis, one patient developed renal failure.
CONCLUSIONS: Although surgical resection remains the key component in the treatment of GC, it is clear that improved outcome will depend on a multidisciplinary treatment. NAC is associated with appreciable toxicity and it may improve the OS, allowing the downstaging of the primary tumor and increasing its resecability, as shown by MAGIC trial.

PMID: 20444330 [PubMed - indexed for MEDLINE]

47. Gan To Kagaku Ryoho. 2010 Apr;37(4):703-6.

[Successful management with S-1 of recurrent gastric cancer after adjuvant chemotherapy with paclitaxel/UFT].

[Article in Japanese]

Tokunaga Y(1), Sasaki H, Saito T.

Author information:
(1)Department of Surgery, Osaka North Japan Post Hospital.

We report here two cases of recurrent gastric cancer after post operative adjuvant chemotherapy, in which S-1 has been effective to control the recurrence and provided long-term survival. Case 1: A 75-year-old male presented with malaise. Endoscopy showed an advanced gastric cancer. He underwent total gastrectomy with lymph adenectomy and received adjuvant chemotherapy with 3 courses of weekly paclitaxel and 6 months of UFT. An abdominal tumor developed with elevation of tumor markers 1 year and 2 months after surgery. After 5 courses of S-1(100mg/day), the tumor resolved and a complete response(CR)was obtained with decline of the markers for 2 years. Case 2: A 62-year-old male presented with abdominal pain. Endoscopy showed an advanced gastric cancer. He underwent distal gastrectomy with lymph adenectomy. Peritonitis carcinomatosa developed with ascites though adjuvant chemotherapy with UFT had been continued for 6 months after paclitaxel. After 10 courses of S-1(100 mg/day), ascites disappeared with decline of the markers. He has been well without any sign of recurrence or elevation of tumor markers for 2 years. Differences in the 5-fluorouracil concentration of UFT and that of S-1 may explain the effectiveness of S-1 for recurrence of gastric cancer after adjuvant chemotherapy with UFT.

PMID: 20414030 [PubMed - indexed for MEDLINE]

48. Gan To Kagaku Ryoho. 2010 Apr;37(4):697-701.

[Histological complete response in a case of advanced gastric cancer treated by

neo-adjuvant chemotherapy with S-1/CDDP].

[Article in Japanese]

Oshima N(1), Tanizawa Y, Bando E, Kawamura T, Tokunaga M, Sugisawa N, Taki Y, Motegi Y, Boku N, Sasaki K, Terashima M.

Author information:
(1)Division of Gastric Surgery, Shizuoka Cancer Center.

A 59-year-old male was found to have advanced gastric cancer with multiple lymph node metastasis including para-aortic lymph nodes(cT3, cN3, cM0, cH0, cP0, cStage IV). Since curative surgery was deemed not feasible, we tried neoadjuvant chemotherapy expecting downstaging of the tumor. S-1(120 mg/body)was orally administered for three weeks followed by one week rest, and CDDP(60 mg/m2)was administered on day 8. Three courses of treatment resulted in a marked shrinkage of the primary lesion and a reduction of lymph nodes. He was judged as clinical PR by RECIST, and a radical resection was considered possible. Laparotomy revealed neither ascites nor peritoneal dissemination, and he underwent total gastrectomy, splenectomy and D2+para-aortic lymph node dissection with curative intent. The histological diagnosis revealed complete disappearance of cancer cells in the primary lesion of the stomach and lymph nodes, confirming a pathological complete response. The patient has been alive for six months without recurrence.

PMID: 20414029 [PubMed - indexed for MEDLINE]

49. World J Gastroenterol. 2010 Apr 21;16(15):1901-7.

Chemotherapy with laparoscope-assisted continuous circulatory hyperthermic intraperitoneal perfusion for malignant ascites.

Ba MC(1), Cui SZ, Lin SQ, Tang YQ, Wu YB, Wang B, Zhang XL.

Author information:
(1)Department of Abdominal Surgery (Section 2), Cancer Hospital of Guangzhou Medical College, Guangzhou 510095, Guangdong Province, China.

AIM: To investigate the procedure, feasibility and effects of laparoscope-assisted continuous circulatory hyperthermic intraperitoneal perfusion chemotherapy (CHIPC) in treatment of malignant ascites induced by peritoneal carcinomatosis from gastric cancers.
METHODS: From August 2006 to March 2008, the laparoscopic approach was used to perform CHIPC on 16 patients with malignant ascites induced by gastric cancer or postoperative intraperitoneal seeding. Each patient underwent CHIPC three times after laparoscope-assisted perfusion catheters placing. The first session was completed in operative room under general anesthesia, 5% glucose solution was selected as perfusion liquid, and 1500 mg 5-fluorouracil (5-FU) and 200 mg oxaliplatin were added in the perfusion solution. The second and third sessions were performed in intensive care unit, 0.9% sodium chloride solution was selected as perfusion liquid, and 1500 mg 5-FU was added in the perfusion solution alone. CHIPC was performed for 90 min at a velocity of 450-600 mL/min and an inflow temperature of 43 +/- 0.2 degrees C.
RESULTS: The intraoperative course was uneventful in all cases, and the mean operative period for laparoscope-assisted perfusion catheters placing was 80 min for each case. No postoperative deaths or complications related to laparoscope-assisted CHIPC occurred in this study. Clinically complete remission of ascites and related symptoms were achieved in 14 patients, and partial remission was achieved in 2 patients. During the follow-up, 13 patients died 2-9 mo after CHIPC, with a median survival time of 5 mo. Two patients with partial remission suffered from port site seeding and tumor metastasis,and died 2 and 3 mo after treatment. Three patients who are still alive today survived 4, 6 and 7 mo, respectively. The Karnofsky marks of patients (50-90) increased significantly ($P < 0.01$) and the general status improved after CHIPC. Thus satisfactory clinical efficacy has been achieved in these patients treated by laparoscopic CHIPC.
CONCLUSION: Laparoscope-assisted CHIPC is a safe, feasible and effective procedure in the treatment of debilitating malignant ascites induced by unresectable gastric cancers.

PMCID: PMC2856833
PMID: 20397270 [PubMed - indexed for MEDLINE]

50. Bull Cancer. 2010 Apr;97(4):435-43. doi: 10.1684/bdc.2010.1085.

[Primary digestive tract lymphoma in central region of Tunisia: anatomoclinical

study and therapeutic results about 153 cases].

[Article in French]

Landolsi A(1), Chabchoub I, Limem S, Gharbi O, Chaafai R, Hochlef M, Fatma LB, Trimech M, Krifa A, Ajmi S, Mokni M, Hadj Hmida MB, Ahmed SB.

Author information:
(1)Service de médecine carcinologique, CHU Farhat-Hached, Sousse, Tunisie.
amel.landolsi@rns.tn

Primary gastro-intestinal lymphoma (PGIL) is the most common type of extra-nodal non Hodgkin's lymphoma. Their clinical and histological presentations are heterogeneous depending on the site of the lesion. There is no consensus regarding the role of surgery and chemotherapy in the therapeutic approach. In our country epidemiology of the disease is unknown with IPSID being the most frequent type. We report anatomo-clinical features and prognostic factors of PGIL and compare intestinal to gastric forms in our region. This is a retrospective study of 153 cases of PGIL in adults diagnosed and treated in the department of medical oncology in Farhat Hached Hospital between 1994 and 2006. The median age was 52 years and the sex-ratio 2.1. Tumor sites were gastric (67%), intestinal (26%) and gastrointestinal (7%). Abdominal pain (87%) followed by vomiting and diarrhoea (37 and 15%) were the most common symptoms. Performance status (PS) < 2 was seen in 80% of patients, high grade lymphoma in 70.5% of cases and B phenotype was noted in 85%. MALT lymphoma accounts for 50% of cases, and IPSID for only 5% of PGIL. About 47.5% of cases were stage IE, 138 patients had chemotherapy with an objective response rate of 77%. Only 46% of patients had surgery (14 for surgical complication, 6 for residual tumor after chemotherapy and 22 to have histological diagnosis). The five-year overall survival (OS) was 62%. In high grade lymphoma patients favorable prognostic factors for OS included young age < or = 60 years, PS < 2, normal serum LDH, hemoglobin > 12 g/dL, B phenotype, localised stage (IE-IIE1), anthracycline-based chemotherapy regimen, achieving complete or partial response to induction chemotherapy and no relapse. In multivariate study only relapse and PS were significant prognostic factors for OS. In low-grade lymphoma patients, none of these factors had a significant correlation with OS: age < or = 60 years, PS < 2, stage (IE-IIE1), response to induction chemotherapy, relapse. Compared to gastric lymphomas, intestinal cases occurred at a younger age, frequently with diarrhoea, weight loss, and occlusion. They are more often high-grade, T phenotype and have locally advanced stage

(IIE); surgery is more common in this group. We conclude that stomach is the main site of PGIL in our region, intestinal lymphoma is less frequent and IPSID has become rare. Recent progress in chemotherapy has allowed good therapeutic results with a conservative approach. Surgery may be performed in case of emergency or for residual lesions after medical treatment.

PMID: 20395189 [PubMed - indexed for MEDLINE]

51. J Clin Oncol. 2010 May 1;28(13):2213-9. doi: 10.1200/JCO.2009.24.8773. Epub 2010 Mar 29.

Phase I/II trial of preoperative oxaliplatin, docetaxel, and capecitabine with concurrent radiation therapy in localized carcinoma of the esophagus or gastroesophageal junction.

Spigel DR(1), Greco FA, Meluch AA, Lane CM, Farley C, Gray JR, Clark BL, Burris HA 3rd, Hainsworth JD.

Author information:
(1)Sarah Cannon Research Institute, 3322 West End Ave, Suite 201, Nashville, TN 37203, USA. dspigel@tnonc.com

PURPOSE: Preoperative chemoradiotherapy is a primary treatment option for patients with resectable esophageal cancer. Combination regimens using newer agents may improve patient outcomes. This multicenter community-based phase I/II trial examined a modern triplet regimen comprised of oxaliplatin, docetaxel, and capecitabine (ODC) combined with radiation therapy (RT).
PATIENTS AND METHODS: The primary end point was the pathologic complete response (pCR) rate. Eligibility criteria included resectable stage I to III cancer of the mid-/distal-esophagus or gastroesophageal junction, measurable disease, and Eastern Cooperative Oncology Group performance status (ECOG PS) of 0 or 1. Treatment included oxaliplatin 40 mg/m(2), docetaxel 20 mg/m(2) (intravenous, weekly x 5); capecitabine 1,000 mg/m(2) orally twice daily on days 1 to 7, 15 to 21, and 29 to 35; and concurrent RT (45 Gy). Resection was performed during weeks 9 to 12. ODC and RT safety was determined in a phase I portion (n = 10) preceding phase II.
RESULTS: Fifty-nine patients were enrolled (September 2005 to February 2008; phase I/cohort 1, 10 patients; phase I/cohort 2/phase II, 49 patients). Baseline

characteristics included median age of 63 years; 84% male; ECOG PS 0 and 1, 51% and 49%, respectively; adenocarcinoma and squamous cell, 69% and 18%, respectively; stage I, II, and III, 12%, 41%, and 45%, respectively. Phase I revealed no dose-limiting toxicity. Responses: pCR rate, 49%; objective response rate, 61% (24 complete and six partial responses); stable disease, 6%; and progressive disease, 2%. Sixty-nine percent of patients underwent surgery. Survival: median follow-up, 116 weeks; median disease-free survival (DFS) and overall survival (OS) were 16.3 and 24.1 months, respectively. Two-year DFS and OS were 45.1% and 52.2%, respectively. Most common (>or= 5%) grade 3 to 4 nonhematologic toxicities were anorexia (20%), dehydration (16%), diarrhea (8%), dysphagia (10%), esophagitis (20%), fatigue (12%), hyperglycemia (6%), nausea (16%), pulmonary symptoms (14%), sepsis (6%), and vomiting (16%). All other grade 3 to 4 hematologic and nonhematologic toxicities were uncommon (< 5%). CONCLUSION: Preoperative ODC plus RT is active and relatively safe in patients with locoregional esophageal cancer. Importantly, this therapy can be administered within 8 weeks. This regimen warrants additional study in this setting and in combination with newer biologic agents.

PMID: 20351330 [PubMed - indexed for MEDLINE]

52. J Hematol Oncol. 2010 Mar 23;3:11. doi: 10.1186/1756-8722-3-11.

Recent advances in gastrointestinal oncology--updates and insights from the 2009 annual meeting of the American society of clinical oncology.

Javle M(1), Hsueh CT.

Author information:
(1)Division of Medical Oncology and Hematology, Loma Linda University, Loma Linda, CA 92354, USA.

We have reviewed the pivotal presentations related to gastrointestinal malignancies from 2009 annual meeting of the American Society of Clinical Oncology with the theme of "personalizing cancer care". We have discussed the scientific findings and the impact on practice guidelines and ongoing clinical trials. Adding trastuzumab to chemotherapy improved the survival of patients with advanced gastric cancer overexpressing human epidermal growth factor receptor 2. Gemcitabine plus cisplatin has become a new standard for first-line treatment of

advanced biliary cancer. Octreotide LAR significantly lengthened median time to tumor progression compared with placebo in patients with metastatic neuroendocrine tumors of the midgut. Addition of oxaliplatin to fluoropyrimidines for preoperative chemoradiotherapy in patients with stage II or III rectal cancer did not improve local tumor response but increased toxicities. Bevacizumab did not provide additional benefit to chemotherapy in adjuvant chemotherapy for stage II or III colon cancer. In patients with resected stage II colon cancer, recurrence score estimated by multigene RT-PCR assay has been shown to provide additional risk stratification. In stage IV colorectal cancer, data have supported the routine use of prophylactic skin treatment in patients receiving antibody against epidermal growth factor receptor, and the use of upfront chemotherapy as initial management in patients with synchronous metastasis without obstruction or bleeding from the primary site.

PMCID: PMC2856525
PMID: 20331897 [PubMed - indexed for MEDLINE]

53. Chin J Cancer. 2010 Mar;29(3):321-4.

A phase II trial of docetaxel plus nedaplatin and 5-fluorouracil in treating advanced esophageal carcinoma.

Guo JF(1), Zhang B, Wu F, Wang B, Xing H, Zhu GY, Nie XY, Peng J.

Author information:
(1)Hexian Memorial Hospital, Guangzhou, Guangdong 511400, P.R. China.

BACKGROUND AND OBJECTIVE: Accumulating data indicate that docetaxel plus cisplatin and 5-fluorouracil has certain effect on advanced gastric or gastro-oesophageal junction adenocarcinoma. This study was to evaluate the efficacy and toxicity of docetaxel plus nedaplatin and 5-fluorouracil (DNF regimen) in treating advanced esophageal carcinoma.
METHODS: Forty-three patients with pathologically confirmed advanced esophageal carcinoma treated by DNF regimen: intravenous infusion of docetaxel (75 mg/m(2)) over 1 h, intravenous infusion of nedaplatin (100 mg/m(2)) over 3 h, intravenous infusion of leucovorin (CF, 200 mg/m(2)) over 2 h, intravenous injection of 5-fluorouracil (375 mg/m(2)) over 10 min, followed by a 46-hour infusion of 5-fluorouracil (2.6 g/m(2)). The cycle was repeated every three weeks. Treatment

efficacy was evaluated every two weeks according to the WHO standards. All patients received at least two cycles of chemotherapy.
RESULTS: Patients received a total of 144 cycles of treatment, and all were evaluable for efficacy and toxicity. Of the 43 patients, 2 (4.65%) achieved complete response (CR), 25 (58.14%) achieved partial response (PR), 9 (20.93%) had stable disease (SD), and 7 (16.28%) had progressive disease (PD). The overall response rate was 62.8%. The median time-to-progression (TTP) was 201 days and the median survival time (MST) was 310 days. Grade III/IV adverse events mainly included neutropenia (20.93%), febrile neutropenia (4.65%), thrombocytopenia (6.98%) and vomiting (9.30%). One patient died of grade IV thrombocytopenia.
CONCLUSION: DNF regimen is effective for and well tolerated by patients with advanced esophageal carcinoma.

PMID: 20193118 [PubMed - indexed for MEDLINE]

54. J Surg Oncol. 2010 Mar 15;101(4):305-14. doi: 10.1002/jso.21483.

Neoadjuvant therapy of locally advanced gastric cancer.

Mezhir JJ(1), Tang LH, Coit DG.

Author information:
(1)Department of Surgery, Memorial Sloan-Kettering Cancer Center, New York, New York 10065, USA. mezhirj@mskcc.org

Treatment of gastric cancer has evolved with the advent of randomized trials demonstrating chemotherapeutic agents with efficacy in advanced disease. Level I evidence supports delivering chemotherapy in the neoadjuvant setting; the data shows improvement in progression-free and overall survival. A clinical response to therapy is associated with improved R0 resection rates, pathologic response, and outcome in patients with locally advanced disease. Early assessment of metabolic response to therapy can potentially be utilized to tailor treatment.

PMID: 20187070 [PubMed - indexed for MEDLINE]

55. Gan To Kagaku Ryoho. 2010 Feb;37(2):315-8.

[A case of advanced gastric cancer responding to S-1 chemotherapy for three weeks].

[Article in Japanese]

Sakurai K(1), Yamashita Y, Shimizu S, Yoshida K, Fukuoka T, Xiang Z, Yamamoto S, Yamamoto A, Kanazawa A, Takemura M, Tsukamoto T, Nishiguchi Y, Ikehara T.

Author information:
(1)Dept. of Digestive Surgery, Osaka City General Hospital.

We reported a patient with advanced gastric cancer successfully treated with S-1 chemotherapy for three weeks. The patient was a 67-year-old man who had gastric cancer clinically diagnosed as cT3N1H0P0M0, stage IIIA. His treatment was supposed to be daily oral administration of 120 mg S-1 for 28 days. At 21 days, this treatment was stopped due to severe appetite loss. The histological diagnosis of the resected stomach revealed complete disappearance of cancer cells in the stomach and the regional lymph nodes. Our report suggested that S-1 may have a potent therapeutic effect in neoadjuvant chemotherapy for advanced gastric cancer.

PMID: 20154493 [PubMed - indexed for MEDLINE]

56. World J Gastroenterol. 2010 Feb 21;16(7):868-74.

Surgical outcome after docetaxel-based neoadjuvant chemotherapy in locally-advanced gastric cancer.

Biffi R(1), Fazio N, Luca F, Chiappa A, Andreoni B, Zampino MG, Roth A, Schuller JC, Fiori G, Orsi F, Bonomo G, Crosta C, Huber O.

Author information:
(1)Division of Abdomino-pelvic Surgery, European Institute of Oncology, Via Ripamonti 435, I-20141 Milan, Italy. roberto.biffi@ieo.it

AIM: To investigate feasibility, morbidity and surgical mortality of a docetaxel-based chemotherapy regimen randomly administered before or after gastrectomy in patients suffering from locally-advanced resectable gastric

cancer.
METHODS: Patients suffering from locally-advanced (T3-4 any N M0 or any T N1-3 M0) gastric carcinoma, staged with endoscopic ultrasound, bone scan, computed tomography, and laparoscopy, were assigned to receive four 21 d/cycles of TCF (docetaxel 75 mg/m(2) day 1, cisplatin 75 mg/m(2) day 1, and fluorouracil 300 mg/m(2) per day for days 1-14), either before (Arm A) or after (Arm B) gastrectomy. Operative morbidity, overall mortality, and severe adverse events were compared by intention-to-treat analysis.
RESULTS: From November 1999 to November 2005, 70 patients were treated. After preoperative TCF (Arm A), thirty-two (94%) resections were performed, 85% of which were R0. Pathological response was complete in 4 patients (11.7%), and partial in 18 (55%). No surgical mortality and 28.5% morbidity rate were observed, similar to those of immediate surgery arm (P = 0.86). Serious chemotherapy adverse events tended to be more frequent in arm B (23% vs 11%, P = 0.07), with a single death per arm.
CONCLUSION: Surgery following docetaxel-based chemotherapy was safe and with similar morbidity to immediate surgery in patients with locally-advanced resectable gastric carcinoma.

PMCID: PMC2825334
PMID: 20143466 [PubMed - indexed for MEDLINE]

57. Cancer. 2010 Apr 1;116(7):1656-63. doi: 10.1002/cncr.24935.

Trimodality therapy without a platinum compound for localized carcinoma of the esophagus and gastroesophageal junction.

Ajani JA(1), Correa AM, Walsh GL, Komaki R, Lee JH, Vaporciyan AA, Rice DC, Yao JC, Maru DM, Hofstetter WL, Phan AT, Swisher SG.

Author information:
(1)Department of Gastrointestinal Medical Oncology, The University of Texas M. D. Anderson Cancer Center, Houston, Texas, USA. jajani@mdanderson.org

BACKGROUND: : The use of platinum-based chemoradiation for esophageal cancer is routine, but it is unclear which class of cytotoxic are optimum. It was hypothesized that chemoradiotherapy with fluoropyrimidine, taxane, and camptothecin would have preserved or improved efficacy with no compromise in

safety.
METHODS: : Patients with histologically confirmed, resectable esophageal carcinoma were eligible. In addition to other tests, a baseline endoscopic ultrasonography (EUS) was obtained. Patients were medically fit and had near-normal organ functions. Patients received docetaxel and irinotecan, plus 5-fluorouracil as induction therapy and then the same cytotoxics with 50.4 grays of radiotherapy followed by an attempted surgery. Pathologic complete response (pathCR) at a rate of > or =20% was the primary endpoint. The pathCR and R0 resection were correlated with overall survival (OS). Safety was documented.
RESULTS: : Fifty-five patients were enrolled. Seven were women, and the median age was 56 years. Fifty-three (96%) patients had EUST3, and 41 (75%) had EUSN1 disease. Forty-three (78%) patients underwent surgery, 20% achieved a pathCR, and 76.4% underwent an R0 resection. The median survival (n = 55 patients) was 43.3 months (range, 19-75 months). Baseline clinical parameters were not found to be predictive of OS; however, patients with a pathCR (P = .005) and who underwent R0 resection (P < or = .0001) had an improved OS. There was 1 treatment-related postsurgical death reported. Grade 3 or 4 toxicity (graded according to the National Cancer Institute Common Toxicity Criteria [version 2.0]) was observed in 62% of patients.
CONCLUSIONS: : The results of the current study documented that this 3-drug, noncisplatin-based chemoradiotherapy was feasible, safe, and active but not better than the published cisplatin-based chemoradiotherapy. A fluoropyrimidine and another cytotoxic (from any class) may be adequate to establish a baseline chemoradiotherapy regimen to combine biologics. Cancer 2010. (c) 2010 American Cancer Society.

PMID: 20143431 [PubMed - indexed for MEDLINE]

58. Cancer Treat Rev. 2010 Aug;36(5):400-9. doi: 10.1016/j.ctrv.2010.01.001. Epub 2010 Feb 1.

Localized adenocarcinoma of the esophagogastric junction--is there a standard of care?

Power DG(1), Reynolds JV.

Author information:
(1)Department of Medical Oncology, St. James's Hospital, Dublin, Ireland.

Adenocarcinoma of the esophagogastric junction (AEG) is the most rapidly increasing tumour in the Western world. Most patients present with locally advanced resectable disease and treatment can be curative. However, no accepted standard treatment exists. Cancer specialists frequently differ on optimum treatment strategies. Areas of debate include the aetiology of AEG, TNM staging, type and extent of resection, relative benefits of preoperative chemotherapy versus preoperative chemoradiation (CRT) versus post-operative CRT, use of early PET scan, and integration of targeted therapy. Randomized trials are weakened by underpowered numbers for AEG tumours, and by methodologic flaws. R0 resection and pathologic complete responses (pCR) predict long-term survival, and most treatment strategies target this as a proxy measure of improved outcome. Some preoperative chemotherapy trials show a benefit but the numbers of true AEG tumours in these studies is unclear. The MAGIC study was powered for gastric cancer only, with just 27% of patients having AEG. Compared with chemotherapy alone, preoperative CRT trials show higher rates of pCR. A large randomized study, with significant toxicity, has shown long-term benefit with adjuvant CRT after resection of gastric cancer (20% AEG). An international consensus on the true definition and optimum management of AEG is required. Molecular and imaging biomarkers will play a vital role in future trials. Trimodality therapy is likely to be optimum with surgery shifted to later in the treatment pathway. Rectal cancer provides an analogous paradigm in this regard. As systemic disease is the primary cause of mortality chemosensitivity should be determined early.

Copyright 2010 Elsevier Ltd. All rights reserved.

PMID: 20117883 [PubMed - indexed for MEDLINE]

59. Int J Cancer. 2010 Oct 15;127(8):1984-90. doi: 10.1002/ijc.25200.

Polaprezinc prevents oral mucositis associated with radiochemotherapy in patients with head and neck cancer.

Watanabe T(1), Ishihara M, Matsuura K, Mizuta K, Itoh Y.

Author information:
(1)Department of Pharmacy, Gifu University Hospital, 1-1 Yanagido, Gifu, Japan.

Oral mucositis is frequent but serious adverse event associated with radiotherapy or radiochemotherapy in head and neck cancer severely impairs health-related quality of life, leading to poor prognosis due to discontinuation of the therapy. Although a number of compounds have been tested for prophylaxis of oral mucositis, few of them are satisfactory. We investigated the effect of polaprezinc (zinc L-carnosine), a gastric mucosal protective drug, on radiochemotherapy-induced oral mucositis, pain, xerostomia and taste disturbance in patients with head and neck cancer. Patients were randomly assigned to receive polaprezinc (n = 16) or azulene oral rinse as the control (n = 15). The incidence rates of mucositis, pain, xerostomia and taste disturbance were all markedly lower in polaprezinc group than in control. Moreover, the use of analgesics was significantly (p = 0.003) less frequent and the amount of food intake was significantly (p = 0.002) higher in polaprezinc group than in control. On the other hand, tumor response rate in patients with neoadjuvant radiochemotherapy was not significantly affected by polaprezinc, in which the response rate (complete plus partial response) was 88% for polaprezinc and 92% for control (p = 1.000). Therefore, it is highly assumable that polaprezinc is potentially useful for prevention of oral mucositis and improvement of quality of life without reducing the tumor response.

PMID: 20104529 [PubMed - indexed for MEDLINE]

60. Zhonghua Wai Ke Za Zhi. 2009 Sep 1;47(17):1305-8.

[A prospective study of FOLFOX7 scheme as neoadjuvant chemotherapy for stage III gastric adenocarcinoma].

[Article in Chinese]

Liu YH(1).

Author information:
(1)Department of General Surgery, First Hospital of Peking University, Beijing 100034, China. liuyinhua@medmail.com.cn

OBJECTIVE: To evaluate the efficacy and safety of FOLFOX7 scheme as neoadjuvant chemotherapy in patients with stage III gastric adenocarcinoma.
METHODS: From May 2005 to May 2007, 27 patients with stage III gastric

adenocarcinoma were given neoadjuvant chemotherapy with FOLFOX7 scheme. Gastroscopy, endoscopic ultrasonography, abdominal B ultrasonography and abdominal CT was taken before chemotherapy and after 2 - 4 cycles of neoadjuvant chemotherapy to evaluate the objective response rate of the tumor. Then operations were carried out and the pathological responses was evaluated in those cases. The safety, objective response rate and pathological rate of neoadjuvant chemotherapy was assessed according to NCI-CTC v3.0, RECIST 2000, and the criteria established by Japanese Research Society for Gastric Cancer, respectively. R0 resection rate and surgery-related complications was also assessed in this group.
RESULTS: The treatment was well tolerated, no grade 3 - 5 toxicity was observed. Complete response was obtained in 1 case, and partial response in 18 patients, overall response rate was 70.4% (19/27). Twenty-six patients received operation and R0 resection rate was 88.4% (23/26); no patient died in the perioperative period. The pathological response rate of patients had R0 excision was 60.9% (14/23).
CONCLUSION: FOLFOX7 scheme as neoadjuvant chemotherapy for selected patients with stage III gastric adenocarcinoma can be well tolerated, it could induce tumor down-staging and improve R0 resection rate, although the long term efficacy remains to be evaluated.

PMID: 20092724 [PubMed - indexed for MEDLINE]

61. Gan To Kagaku Ryoho. 2009 Nov;36(12):2436-8.

[A case of stage IV advanced esophageal cancer with a long term survival by radiation therapy combined with nedaplatin and 5-FU chemotherapy].

[Article in Japanese]

Morimoto J(1), Oohira M, Kubo N, Tanaka H, Dan N, Muguruma K, Yashiro M, Sawada T, Yamashita Y, Nishiguchi Y, Hirakawa K.

Author information:
(1)Department of Surgical Oncology, Osaka City University Graduate School Division of Medicine.

The patient was a 73-year-old man who complained of dysphagea. Various

examinations revealed an esophageal cancer with direct invasion to the left main bronchus (cT4, N2 (104R, 106recR), M0, Stage IVa) and gastric cancer (cT2, N0, M0, Stage IB). The patient was given preoperative chemoradiotherapy (40 Gy/20 fr with CDGP 10 mg/body day 1-5, 8- 12, 15-19 and 5-FU 250 mg/body day 1-5, 8-12, 15-19). After the chemoradiotherapy, we estimated that the esophageal cancer was down stage (cT4-->T3), and that a curative operation was possible. Therefore, subtotal esophagectomy and partial gastrectomy were performed without a complication. Pathological therapeutic evaluation of the esophageal cancer was complete response (CR) and the gastric cancer was T2, N0. Adjuvant chemotherapy was undergone with S-1. However, two years after the first operation, we found a recurrence of gastric duct. Therefore a surgical resection for recurrence of gastric duct was performed. The patient is still alive without recurrence 5 years and 2 months after the first treatment. Radiation therapy combined with nedaplatin and 5-FU is a safe and effective method for treating cT4 advanced esophageal cancer.

PMID: 20037448 [PubMed - indexed for MEDLINE]

62. Zhonghua Wai Ke Za Zhi. 2009 Aug 1;47(15):1171-4.

[Neoadjuvant chemotherapy of FLEEOX regimen for unresectable gastric cancer resulting from advanced abdominal lymph nodes metastases].

[Article in Chinese]

Li GL(1), Fan CG, Bao Y, Jiang J, Li N, Li JS.

Author information:
(1)Research Institute of General Surgery, Nanjing General Hospital of Nanjing Military Command, Nanjing 210002, China. drguoli@yahoo.com.cn

OBJECTIVE: To observe the therapeutic response of advanced gastric cancer with severe lymph nodes metastasis to FLEEOX regimen neoadjuvant chemotherapy that combined arterial and venous administration.
METHODS: Neoadjuvant chemotherapy was administered to 32 cases of gastric cancer with advanced abdominal lymph nodes metastases from January 2007 to October 2008. Of the 32 patients, 28 had severe local lymph nodes metastasis, such as No.3, 7, 9, 12 lymph nodes metastasis, one patient had No.16 lymph nodes metastasis, and

the other 3 patients had both regional and No.16 lymph nodes metastasis under CT scan. Neoadjuvant chemotherapy was administered as follows: 5-Fu 370 mg/m(2), intravenous drip, day 1 - 5; Leukovorin 120 mg, intravenous drip, day 1 - 5; oxaliplatin 150 mg/m(2), epirubicin 30 mg/m(2) and epotoside 70 mg/m(2), intravascular infusion through arteria gastrica sinistra, day 6 and 20. The protocol was repeated every five weeks for two or three courses. After 2 or 3 cycles of chemotherapy, abdominal CT was taken to evaluate the radiological therapeutic response and calculate the reductive rate of the tumor.
RESULTS: The general conditions and symptoms was improved significantly in all the patients. Four cases got complete response (CR), 24 got partial response (PR) and 4 got no change under CT scan. For the 32 case, the radiological response rate (CR + PR) was 87.5%(28/32). Thirty patients underwent subtotal or total gastrectomy, even combined organ resection, with D2 or D2 + alpha lymphadenectomy.
CONCLUSIONS: The FLEEOX regimen which combines arterial and venous administration carries a satisfactory therapeutic effect in advanced gastric cancer with severe lymph nodes metastasis. The combined routes of drug administration may improve the effects of neoadjuvant chemotherapy in stomach cancer.

PMID: 20021910 [PubMed - indexed for MEDLINE]

63. Nihon Hinyokika Gakkai Zasshi. 2009 Nov;100(7):698-702.

[Case of malignant lymphoma of the prostate complicated with prostate adenocarcinoma].

[Article in Japanese]

Chin K(1), Fujimura M, Sekit N, Mikami K, Kamijima S, Suzuki H, Ichikawa T.

Author information:
(1)The Department of Urology, Chibaken Saiseikai Narashino Hospital.

A 68-year-old man was referred to our hospital with complaints of palpation, hematemesis and melena. Esophagogastroduodenoscopy revealed a huge ulcer in the stomach, and based on biopsy findings, he was pathologically diagnosed as having diffuse large B-cell type malignant lymphoma. A computed tomographic scan demonstrated prostatic enlargement and swelling of the left external iliac lymph

nodes. Since his serum PSA level was 13.0 ng/ml, prostatic needle biopsy was performed. Histological findings revealed diffuse large B-cell type malignant lymphoma and moderately differentiated adenocarcinoma of the prostate. The patient achieved complete response after eight cycles of combination chemotherapy with rituximab cyclophosphamide, adriamycin, vincristine and predonisolone. At the same time of chemotherapy, androgen deprivation therapy was initiated. The current his PSA level is 0.2 ng/ml or less.

PMID: 19999135 [PubMed - indexed for MEDLINE]

64. World J Gastroenterol. 2009 Dec 7;15(45):5746-50.

Complete remission of gastric Burkitt's lymphoma after eradication of Helicobacter pylori.

Baumgaertner I(1), Copie-Bergman C, Levy M, Haioun C, Charachon A, Baia M, Sobhani I, Delchier JC.

Author information:
(1)Department of Gastroenterology, AP-HP, Henri Mondor Hospital, Paris 12 University, Créteil, France. jean-charles.delchier@hmn.aphp.fr

Burkitt's lymphoma is a highly aggressive non-Hodgkin lymphoma, often presenting in extra-nodal sites. It generally has a poor spontaneous outcome and needs aggressive treatment with systemic and intrathecal chemotherapy. Occurrence at the gastric site is rare. We report the case of a 39-year old woman who presented with a prominent ulcerated lesion of the antrum corresponding histologically to a Burkitt's lymphoma associated with Helicobacter pylori (H pylori) infection. Interphase fluorescence in situ hybridization (FISH) demonstrated c-MYC gene rearrangement in tumour cells without BCL2 or BCL6 gene translocations. Ulcer healing and tumour regression with a complete histological response were obtained 8 wk after H pylori eradication. In spite of this complete remission, taking into account the high risk of recurrence, the patient received systemic and intrathecal chemotherapy. Two years later, the patient remained in complete remission. This is the first report of a gastric Burkitt's lymphoma responding to H pylori eradication. These findings raise the question of the potential role of H pylori in the pathogenesis of some gastric Burkitt's lymphomas, and show the importance of searching for and eradicating the bacteria in combination with

conventional chemotherapy regimens.

PMCID: PMC2789231
PMID: 19960575 [PubMed - indexed for MEDLINE]

65. Med Oncol. 2010 Dec;27(4):1089-95. doi: 10.1007/s12032-009-9340-7. Epub 2009 Nov
3.

Neoadjuvant chemotherapy with a combination of docetaxel, cisplatin, fluorouracil, and leucovorin in nonresectable advanced gastric cancer: a short communication.

Ma Y(1), Qin H, Zheng Q, Wang Y, Wang Z, Yang Z.

Author information:
(1)Department of Surgery, The Sixth People's Hospital Affiliated to Shanghai Jiao Tong University, 600 Yishan Road, Shanghai, 200233, People's Republic of China.

The aim of this study was to evaluate the efficacy and toxicity of docetaxel (TAX), cisplatin (CDDP), and fluorouracil (5-FU) plus leucovorin (CF) as the neoadjuvant chemotherapy (NACT) regimens in the treatment of nonresectable advanced gastric cancer. Twelve patients with nonresectable advanced gastric cancer were treated with NACT regimens consisted of docetaxel, cisplatin, fluorouracil, plus leucovorin before operation. Nine of the 12 patients were downstaged and 8 were radically operated after the end of the NACT. The overall response rate was 75% with 8.3% complete response and 66.7% partial response, and the ascites disappeared in 63.6%. The most common toxicities were bone marrow suppression, nausea, vomiting, alopecia, and heptoses. The toxicities were recoverable after symptomatic treatment. The results confirmed that the combination of docetaxel, cisplatin, fluorouracil plus leucovorin (CF) is a very effective and well-tolerated regimen as NACT for the patients with nonresectable advanced gastric cancer.

PMID: 19885749 [PubMed - indexed for MEDLINE]

66. Cancer Sci. 2010 Jan;101(1):94-102. doi: 10.1111/j.1349-7006.2009.01364.x. Epub
2009 Sep 14.

Silencing of galectin-3 changes the gene expression and augments the sensitivity of gastric cancer cells to chemotherapeutic agents.

Cheong TC(1), Shin JY, Chun KH.

Author information:
(1)Gastric Cancer Branch, Division of Translational and Clinical Research I, National Cancer Center Research Institute and Hospital, Madu1-dong, Ilsandong-gu, Goyang-si, Gyeonggi-do, Korea.

Galectin-3 is known to modulate cell proliferation and apoptosis and is highly expressed in human cancers, but its function in gastric cancer is still controversial. Here, we examined the role of galectin-3 in gastric cancer cells by silencing it with synthetic double-stranded siRNA. After silencing of galectin-3, cell numbers decreased and cell shape changed. Galectin-3 siRNA treatment also induced G(1) arrest. DNA microarray analysis was used to assess changes in gene expression following galectin-3 silencing. We found that silencing of galectin-3 caused changes in gene expression. RT-PCR and real-time PCR were utilized for validation of the changes found in microarray studies. Western blot analysis confirmed changes in the expression of proteins of interest: cyclin D1, survivin, XIAP, XAF, PUMA, and GADD45alpha. Generally, it tended to increase the expression of several pro-apoptotic genes, and to decrease the expression of cell cycle progressive genes. We also confirmed that changes in the expression of these genes were caused by galectin-3 overexpression. Finally, we demonstrated that silencing of galectin-3 enhanced apoptosis induction with chemotherapeutic agents by further reducing the expression of anti-apoptotic and/or cell survival molecules such as survivin, cyclin D1, and XIAP, and increasing the expression of pro-apoptotic XAF-1. We conclude that galectin-3 is involved in cancer progression and malignancy by modulating the expression of several relevant genes, and inhibition of galectin-3 may be an approach to improve chemotherapy of gastric cancers.

PMID: 19843071 [PubMed - indexed for MEDLINE]

67. Hepatogastroenterology. 2009 Jul-Aug;56(93):1213-8.

Expression of p53, p21 and p16 does not correlate with response to preoperative chemoradiation in gastric carcinoma.

Sirak I(1), Petera J, Hatlova J, Vosmik M, Melichar B, Dvorak J, Tycova V, Zoul Z, Lesko M.

Author information:
(1)Department of Oncology and Radiotherapy, Charles University Medical School and University Hospital, Hradec Kralove, Czech Republic. sirak@fnhk.cz

BACKGROUND/AIMS: A retrospective evaluation was oerformed to determine whether the intensity of p53, p21 or p16 expression predicts response to preoperative chemoradiotherapy in locally advanced gastric carcinoma.
METHODOLOGY: Thirty-six patients (cT2-4 or N+) were studied. Preoperative treatment consisted of 30-45Gy of radiation with continuous 5-fluorouracil and weekly cisplatin. Expression of p53, p21 and p16 in pretreatment biopsies was assessed by immunohistochemistry. Level of expression was determined from the intensity and extent of staining. Tumor downstaging was defined as a reduction of at least one T-stage level and/or finding of intense tumor regression in histopathologic examination.
RESULTS: Seventeen patients responded to chemoradiation: 8 patients had pathologic complete response, 9 patients were downstaged. The multivariate analysis showed no significant influence of p53 ($p = 0.76$), p21 ($p = 0.10$) nor p16 ($p = 0.70$) expression upon tumor response. Response was found in 52% of patients with low, and in 40% of patients with high p53 staining ($p = 0.21$); in 54% of patients with low, and in 30% of patients with high p21 staining ($p = 0.14$); and in 48% of patients with low, and in 43% of patients with high p16 staining ($p = 0.32$).
CONCLUSIONS: We found no significant influence of p53, p21 nor p16 expression upon response to preoperative chemoradiotherapy in gastric carcinoma.

PMID: 19760973 [PubMed - indexed for MEDLINE]

68. Gan To Kagaku Ryoho. 2009 Sep;36(9):1481-4.

[Analysis of patients with locally advanced gastric cancer undergoing gastrectomy

after pre-operative chemotherapy including S-1].

[Article in Japanese]

Kinouchi M(1), Shibata C, Miura K, Okabe M, Andou T, Kakyou M, Saijou F, Haneda S, Ogawa H, Unno M, Sasaki I.

Author information:
(1)Division of Biological-regulation and Oncology, Graduate School of Medicine, Tohoku University.

To improve the prognosis of advanced gastric cancer, various adjuvant therapies were tried. We retrospectively examined 7 cases of gastrectomy after pre-operative chemotherapy using S-1+CDDP, and reported its effectiveness and the result. An adverse event of more than Grade 3 showed neutropenia in two cases. Anti-tumor effect on the imaging was found in 6 cases of PR and one case of SD. We performed gastrectomy within 4 weeks after completion of chemotherapy in each case. The histological antitumor effect was more than Grade 2 in four cases, but there was no complete response. We compared the overall survival of a patient of more than Grade 2 by histological antitumor effect under grade 1b. In the former, MST was 982 days and the two-year survival rate was 50.0%; in the latter, MST was 443 days and the two-year survival rate was 33.3%. We can perform pre-operative chemotherapy using S-1+CDDP and expect prognostic improvement for a histologically effective case of chemotherapy. If the utility of preoperative chemotherapy mainly with S-1 is proved in a phase III trial, this would appear to be a therapy of choice for advanced gastric cancer in future.

PMID: 19755816 [PubMed - indexed for MEDLINE]

69. Orv Hetil. 2009 Aug 30;150(35):1649-53. doi: 10.1556/OH.2009.28692.

[Primary gastrointestinal non-Hodgkin's lymphoma in two Hungarian regions].

[Article in Hungarian]

Kollár B(1), Rajnics P, Hunyady B, Zeleznik E, Jakucs J, Egyed M.

Author information:

(1)Kaposi Mór Oktató Kórház Belgyógyászati Osztály Kaposvár. kollar.b@freemail.hu

Over the past few decades, the occurrence of adult onset non-Hodgkin's lymphoma has significantly increased. The patient population involved is very heterogeneous, with different clinical and morphological manifestations. In addition to the most typical nodal involvement, extra-nodal manifestations are also frequent, affecting, most often, the gastrointestinal tract, the central nervous system and the skin. The treatment strategy for non-Hodgkin's lymphoma has changed over the past decade: chemo-immunotherapy has largely taken over surgical intervention, the dominant treatment option of the past.METHODS: The authors present their experience with 48 patients with non-Hodgkin's lymphoma, affecting the gastrointestinal tract, treated in Kaposvár, in the Kaposi Mór Teaching Hospital and in Gyula, in the Pándy Kálmán County Hospital. Demography: 27 female, 21 male; mean age: 67.8 years. Localization, pathological classification and the international prognostic index (IPI) have been analysed and correlated with the clinical response to different therapeutic strategies.
RESULTS: The most frequently involved GI organ was the stomach (n = 26), with the dominant histological type of diffuse large B-cell lymphoma. Fourty-six patients received chemo-immunotherapy, 6 received radiotherapy, 3 patients were primarily treated with Helicobacter pylori eradication therapy, and 4 patients were referred for stomach resection. A complete remission was achieved in 68% of the patients, a partial remission in 13%, while 19% did not show clinical response. Based on the international prognostic index, the majority of the patients fulfilled criteria of low or high intermediate risk categories, with an IPI average of 2.68. Patients with upper gastrointestinal tract involvement carried the best prognosis (IPI: 2.0); at the same time, patients with stomach lymphoma achieved the highest rate of remission (73%).
CONCLUSIONS: With chemo-immunotherapy the chances of a complete remission have significantly improved over the past decade, thus a significant portion of non-Hodgkin's lymphomas involving the gastrointestinal tract can be cured. IPI index represents the most recognised indicator for assessing the prognosis of non-Hodgkin's lymphoma. Patients who achieved complete remission had the lowest prognostic index in this cohort; nevertheless, numerous data indicate that factors other than the IPI can also have an impact on patients' response to treatment.

PMID: 19692309 [PubMed - indexed for MEDLINE]

70. Ai Zheng. 2009 Jul;28(7):734-9.

[Clinical features and prognosis of mucosa-associated lymphoid tissue lymphoma: a report of 90 cases].

[Article in Chinese]

Zhai LZ(1), Xiao J, Fu XH, Ye S, Guo CC, Huang JJ, Tian Y, Lin HL, Lin TY.

Author information:
(1)State Key Laboratory of Oncology in South China, Guangzhou, Guangdong, 510060, People's Republic of China.

BACKGROUND AND OBJECTIVE: Mucosa-associated lymphoid tissue lymphoma is a histological type of marginal zone non-Hodgkin's lymphoma (NHL). Its clinical features and prognosis have seldom been reported because of its indolent clinical course. This study was to explore the clinical features and prognosis of this disease.
METHODS: Clinical data of 90 pathologically confirmed mucosa-associated lymphoid tissue lymphoma patients, treated from December 1997 to February 2007, were analyzed.
RESULTS: Of the 90 patients, 23 (25.6%) had gastric lymphoma and 67 (74.4%) had non-gastric lymphoma, with a median age of 52 (range, 13-77); 75 (83.3%) had stage I-II disease and 15 (16.7%) had stage III-IV disease; 31 (34.4%) had multiple organ involvement and 40 (44.4%) had nodal involvement. The percentage of nodal involvement was significantly higher in non-gastric group than in gastric group (P=0.040). The complete remission (CR) rate after treatment was 72.1%. The patients were followed up for a median of 31.4 months. The 5-year overall survival rates of patients with and without nodal involvement were 58.7% and 88.4%, respectively (P=0.012). The median time to progression was significantly longer in patients with IPI score of 0-2 than in those with IPI score of > 2 (61.9 months vs. 5.2 months, P=0.005), and was significantly longer in patients who got CR after initial treatment than in those without CR (not reached vs. 15.0 months, P=0.030). In non-gastric lymphoma group, IPI score was an independent prognostic variable of overall survival (P=0.023).
CONCLUSIONS: Mucosa-associated lymphoid tissue lymphoma should be considered as a kind of disseminated indolent lymphoma. The patients with non-gastric lymphoma are likely to have nodal involvement. Patients with poor prognostic factors should be treated more aggressively.

PMID: 19624901 [PubMed - indexed for MEDLINE]

71. Ai Zheng. 2009 Apr;28(4):412-5.

[Oxaliplatin combined with capecitabine as first-line chemotherapy for patients with advanced gastric cancer].

[Article in Chinese]

Dong NN(1), Wang MY, Zhang Q, Liu ZF.

Author information:
(1)Department of Internal Medicine, Shandong Tumor Hospital and Institute, Jinan, Shandong, 250117, PR China.

BACKGROUND AND OBJECTIVE: Combination therapy of oxaliplatin and capecitabine has certain effects on advanced gastric cancer (AGC). This study was to investigate the efficacy and safety of oxaliplatin in combination with capecitabine as first-line chemotherapy for AGC patients.
METHODS: Thirty-three chemotherapy-naive patients with AGC were entered into this study. They received 2 h intravenous infusion of oxaliplatin 130 mg/m2 on day 1 and oral administration of capecitabine 2000 mg/m2, given in two daily doses, on days 1-14 (XELOX regimen). The regimen was repeated every 21 days. A maximum of eight cycles were given.
RESULTS: Thirty-three patients completed 159 cycles of chemotherapy with a median number of five cycles. Thirty-one patients were evaluable for efficacy. The response rate was 54.8% [95% confidence interval (CI): 37.3%-72.3%], with one complete response (3.2%), 16 partial responses (51.6%), eight stable diseases (25.8%), and six progressions (19.4%). At a mean follow-up of 10.5 months, the median time to progression and overall survival were 5.9 (95% CI: 4.7-7.1) and 10.4 months (95% CI: 7.9-12.9), respectively. The most common adverse events were myelosuppression, peripheral neuropathy, diarrhea, nausea/vomiting, and hand-foot syndrome.
CONCLUSION: XELOX is an effective and well-tolerated first-line chemotherapy regimen for patients with AGC.

PMID: 19622303 [PubMed - indexed for MEDLINE]

72. Anticancer Drugs. 2009 Sep;20(8):752-6. doi: 10.1097/CAD.0b013e32832ec02b.

Feasibility and efficacy of preoperative chemotherapy with docetaxel, cisplatin and S-1 in gastric cancer patients with para-aortic lymph node metastases.

Fushida S(1), Fujimura T, Oyama K, Yagi Y, Kinoshita J, Ohta T.

Author information:
(1)Department of Gastroenterological Surgery, Kanazawa University Hospital, 13-1 Takara-machi, Kanazawa 920-8641, Japan. fushida@surg2.m.kanazawa-u.ac.jp

We performed preoperative chemotherapy with combined docetaxel, cisplatin and S-1 (DCS therapy) for treatment of advanced gastric cancer with para-aortic lymph node metastases. The aim of this study was to determine the maximum tolerated dose (MTD) and the dose-limiting toxicities. Furthermore, we evaluated the feasibility of DCS therapy in a preoperative setting, and also examined the pathological response. Fifteen patients received intravenous docetaxel and cisplatin (30, 35 or 40 mg/m2, each dose escalation was reciprocal) on days 1 and 15 and oral S-1 (40 mg/m2 twice daily) on days 1-14 every 4 weeks. After one cycle of chemotherapy, toxicities were evaluated and after two cycles of chemotherapy, patients who were judged to be candidates for curative resection underwent gastrectomy with D2 lymphadenectomy plus para-aortic lymph node dissection. The MTD of this combination was presumed to be at dose level 3 (docetaxel 40 mg/m2 and cisplatin 35 mg/m2). The dose-limiting toxicities were grade 4 neutropenia in one patient, grade 3 febrile neutropenia in two patients and grade 3 diarrhoea in two patients. Thirteen of the 15 patients received complete resection and there was no operation-related death. Good pathological responses were observed in 12 cases with lesions in the lymph nodes (complete response, n = 4; partial response, n = 8) and 11 patients with primary stomach lesions (complete response, n = 2; partial response, n = 9). This preoperative DCS therapy was considered feasible and provided a high pathological response rate in gastric cancer patients with para-aortic lymph node metastases.

PMID: 19543076 [PubMed - indexed for MEDLINE]

73. Int J Radiat Oncol Biol Phys. 2009 Dec 1;75(5):1430-6. doi:

10.1016/j.ijrobp.2008.12.087. Epub 2009 Jun 18.

Phase II trial of preoperative irinotecan-cisplatin followed by concurrent irinotecan-cisplatin and radiotherapy for resectable locally advanced gastric and esophagogastric junction adenocarcinoma.

Rivera F(1), Galán M, Tabernero J, Cervantes A, Vega-Villegas ME, Gallego J, Laquente B, Rodríguez E, Carrato A, Escudero P, Massutí B, Alonso-Orduña V, Cardenal A, Sáenz A, Giralt J, Yuste AL, Antón A, Aranda E; Spanish Cooperative Group for Digestive Tumor Therapy.

Author information:
(1)Department of Medical Oncology, Hospital Universitario Marqués de Valdecilla, Santander, Spain. oncrhf@humv.es

PURPOSE: To determine in a Phase II trial whether preoperative irinotecan-cisplatin (IC) followed by concurrent IC therapy and radiotherapy (IC/RT) improved outcome in patients with resectable, locally advanced gastric adenocarcinoma (GC) or esophagogastric junction cancer (EGJC).
PATIENTS AND METHODS: Patients with resectable Stage II-IV, M0 GC or EGJC made up the study population. The primary endpoint was pathologic complete response (pCR). Two courses of IC (irinotecan, 65 mg/m(2); cisplatin, 30 mg/m(2) on Days 1 and 8 every 21 days) were given. Patients without progression then received IC/RT, consisting of daily radiotherapy (45Gy) with concurrent IC (irinotecan, 65 mg/m(2); cisplatin, 30 mg/m(2) on Days 1, 8, 15, and 22). Surgical resection was performed, if feasible, 5-8 weeks after the end of radiotherapy.
RESULTS: Twenty-three patients were included in the study: 10 with EGJC and 13 with GC. Two patients (9%) achieved pCR. The incidences of Grade 3-4 toxicities were as follows: IC: neutropenia 35% (febrile 13%), anemia 22%, diarrhea 22%, emesis 8%; IC/RT: neutropenia 52% (febrile 5%), asthenia 19%, anemia 9%, emesis 9%, diarrhea 5%, cardiotoxicity 5%. No patients died during IC or IC/RT. R0 resection was achieved in 15 patients (65%). Median survival was 14.5 months, and the actuarial 2-year survival rate was 35%.
CONCLUSIONS: Preoperative IC followed by IC/RT resulted in moderate response and resection rates with mild toxicity in patients with GC and EGJC.

PMID: 19540072 [PubMed - indexed for MEDLINE]

74. Zhonghua Zhong Liu Za Zhi. 2009 Jan;31(1):75-8.

[Oxaliplatin combined with ELF regimen in the treatment of patients with advanced gastric cancer].

[Article in Chinese]

Lou F(1), Zhu YH, Pan HM.

Author information:
(1)Department of Oncology Center, Sir Run Run Shaw Hospital, Medical College of Zhejiang University, SIR Run Run ShawInstitute of Clinical Medicine of Zhejiang University, Hangzhou 310016, China.

OBJECTIVE: To evaluate the efficacy and safety of the combination of oxaliplatin and ELF (VP16/CF/5-Fu) regimen in the treatment of patients with advanced gastric cancer.
METHODS: Oxaliplatin was given at a dose of 100 mg/m(2) i.v. 2 hours D1, calcium folinate (CF) 200 mg/m(2) i.v. 1/2 hour D1 approximately D3, 5-fluorouracil (5-Fu) 500 mg/m(2) i.v. 2 hours D1 approximately D3 and etoposide 100 mg/m(2) i.v. 3 hours D1 approximately D3. Cycles were repeated every 21 days. Efficacy and safety were evaluated every 2 cycles.
RESULTS: Sixty-nine patients were enrolled into the study. All cases were pathologically confirmed as gastric cancer (adenocarcinoma in 57 cases and signet ring cell carcinoma in 12 cases). 42 patients had newly diagnosed disease, and 27 patients had received previous chemotherapy. 62 patients were analyzed for response (7 complete responses and 25 partial responses) with total response rate 51.61%. The median time to progression was 5.7 months and the median overall survival was 9.2 months. The most common hematologic toxicities were anemia (29.0%), leucopenia (51.2%) and thrombocytopenia (21.2%). No grade 4 and grade 5 hematologic toxicities were observed. The most common non-hematologic toxicities were nausea (46.5%), vomiting (41.1%), peripheral sensory neuropathy (47.1%), and grade 2 alopecia (27.3%).
CONCLUSION: This oxaliplatin combined with ELF regimen shows good efficacy and acceptable safety in advanced gastric cancer patients. It is worthy to be proved as a suitable alternative regimen in this indication.

PMID: 19538878 [PubMed - indexed for MEDLINE]

75. J Thorac Oncol. 2009 Jul;4(7):875-9. doi: 10.1097/JTO.0b013e3181a8cebf.

Predictive value of initial PET-SUVmax in patients with locally advanced esophageal and gastroesophageal junction adenocarcinoma.

Rizk NP(1), Tang L, Adusumilli PS, Bains MS, Akhurst TJ, Ilson D, Goodman K, Rusch VW.

Author information:
(1)Memorial Sloan-Kettering Cancer Center, New York City, New York 10021, USA. rizkn@mskcc.org

INTRODUCTION: We have previously shown that in early clinical stage esophageal adenocarcinoma, a positron emission tomography standardized uptake values (PET SUVmax) of <4.5 is associated with earlier pathologic stage and predicts better survival. In this study, we analyze the impact of the pretreatment PET SUVmax in patients with locally advanced esophageal adenocarcinoma who undergo preoperative chemoradiotherapy.
METHODS: We performed a retrospective analysis, selecting patients with adenocarcinoma of the esophagus who had a pretreatment PET scan and who received chemoradiotherapy before esophagectomy. Data recorded included demographics, PET SUVmax, treatment details, pathologic details, and survival data. Comparison of categorical variables was done by chi analysis, continuous variables by t test, survival analysis by the Kaplan-Meier method, and comparisons of survival using the log-rank test.
RESULTS: Between January 1996 and September 2007, 189 patients were appropriate for this analysis. The initial PET SUVmax was <4.5 in 28 patients and >or=4.5 in 161 patients. The two groups were similar with regards to demographics and treatment details. Patients in the low SUV group were less likely to show evidence of treatment response after chemoradiotherapy, including a higher likelihood of residual nodal disease and a lower likelihood of a pathologic complete response and estimated treatment response. However, both groups had similar survival.
CONCLUSIONS: Although the initial PET SUVmax does not predict survival in patients with locally advanced esophageal adenocarcinoma who receive preoperative chemoradiotherapy, patients with a high initial SUVmax respond better to preoperative therapy. These results can be used to better select esophageal cancer patients for combined modality treatment.

PMID: 19487968 [PubMed - indexed for MEDLINE]

76. Leuk Lymphoma. 2009 Jun;50(6):925-31. doi: 10.1080/10428190902912478.

Early stage gastric diffuse large B-cell lymphomas: results of a randomized trial comparing chemotherapy alone versus chemotherapy + involved field radiotherapy. (IELSG 4). [corrected].

Martinelli G(1), Gigli F, Calabrese L, Ferrucci PF, Zucca E, Crosta C, Pruneri G, Preda L, Piperno G, Gospodarowicz M, Cavalli F, Moreno Gomez H.

Author information:
(1)Division of Haematoncology, European Institute of Oncology, Milan, Italy. giovanni.martinelli@ieo.it

Erratum in
Leuk Lymphoma. 2010 Apr;51(4):733.
Leuk Lymphoma. 2009 Nov;50(11):1904.

Here, we present the results of a randomised clinical trial carried out between 1998 and 2004, evaluating the possible role of radiotherapy (RT) as consolidation treatment after induction chemotherapy (CT) in diffuse large B-cell (DLBC) gastric lymphoma. Fifty-four patients were enrolled and all received anthracycline containing regimens as induction CT. Patients were evaluated after four to six cycles and those in complete remission (CR) were randomised to receive gastric involved field (IF) RT or two addition cycles of the same CT. Forty-five patients (83%) were randomised after the induction CT. Clinical results of patients allocated to the RT arm showed a significant reduction in incidence of local relapse versus patients who received CT alone. However, overall survival was not different between the two arms. Our results confirm that CT could be considered as first line therapy for newly diagnosed gastric DLBC lymphoma; IF RT delivered in those patients achieving CR after induction CT is able to prevent local relapse.

PMID: 19479614 [PubMed - indexed for MEDLINE]

77. Gan To Kagaku Ryoho. 2009 May;36(5):847-9.

[A case of complete response (CR) persisting for 3 years and 3 months from combination therapy of S-1 and CDDP in a patient with advanced gastric cancer with multiple liver metastases].

[Article in Japanese]

Sekino Y(1), Okada M, Hamada J, Tagami S, Sakon M, Okita K, Takata M, Seki H, Hayashi K, Munakata Y.

Author information:
(1)Dept. of Surgery, Nagano Municipal Hospital.

The case was a 70-year-old man with type-2 gastric cancer in the lesser curvature accompanied by multiple liver metastases. He received combination chemotherapy of S-1 and CDDP. S-1 was administered at 100 mg/body/day for 21 days followed by withdrawal for 14 days, and CDDP was prescribed at 80 mg/body/day div on day 8. After 3 courses of treatment, the multiple liver metastases disappeared. The primary gastric lesion had changed to a scar and endoscopic biopsy revealed no cancer cell. After the 4th course, we changed the therapy to S-1 alone and after that to UFT alone. Now, 3 years and 3 months after inducing CR, the patient continues to receive UFT with no regrowth of the tumor.

PMID: 19461192 [PubMed - indexed for MEDLINE]

78. Br J Cancer. 2009 Jun 2;100(11):1725-30. doi: 10.1038/sj.bjc.6605070. Epub 2009 May 12.

A phase II trial of preoperative chemotherapy with epirubicin, cisplatin and capecitabine for patients with localised gastro-oesophageal junctional adenocarcinoma.

Starling N(1), Okines A, Cunningham D, Allum W, Wotherspoon A, Benson M, Thompson J, Thomas J, Brown G, Riddell A, Stavridi F, Ashley S, Oates J, Chau I.

Author information:
(1)Department of Medicine, Royal Marsden Hospital NHS Foundation Trust, Sutton,

Surrey, UK.

Preoperative cisplatin/fluorouracil is used for the treatment of localised oesophageal carcinoma. This phase II study aimed to assess the efficacy and safety of administering preoperative epirubicin/cisplatin/capecitabine (ECX). Patients with stage II or III oesophageal/gastro-oesophageal junctional adenocarcinoma from one institution received 4 cycles of ECX (epirubicin 50 mg m(-2) day 1, cisplatin 60 mg m(-2) day 1, capecitabine 625 mg m(-2) b.i.d. daily) followed by surgery. The primary end point was the pathological complete response (pCR) rate based on a Simon two-stage design. Secondary end points included overall and progression-free survival (OS/PFS). Thirty-four patients were recruited: median age 60 years (range 41-81), 91% male, 97% PS 0/1, 80% T3, 68% N1. Thirty-one patients completed four ECX cycles. Grade 3/4 toxicities >or=5% included neutropenia (62%), hand-foot syndrome (15%) and nausea/vomiting (9%). Thirteen out of 28 (46%) evaluable patients responded to chemotherapy by EUS (>or=30% reduction in maximal tumour thickness). Twenty-six out of 34 (76%) patients underwent resection (R0=73%, R1=27%). Post-operatively, two patients died within 60 days of surgery. The pCR rate was 5.9% (95% CI 0-14%) in the intent-to-treat population. According to the statistical design, this prompted early study termination. However, with a median follow-up of 34 months the median OS and 1- and 2-year survival rates were 17 months, 67 and 39% respectively. Median PFS was 13 months. Of the 14 relapsed patients, 10 presented with distant metastases. Preoperative ECX is feasible and well tolerated. Although associated with a low pCR rate, survival with ECX was comparable with published studies suggesting that pCR may not correlate with satisfactory outcome from preoperative chemotherapy for localised oesophageal adenocarcinoma.

PMCID: PMC2695693
PMID: 19436301 [PubMed - indexed for MEDLINE]

79. Anticancer Drugs. 2009 Mar;20(3):191-6. doi: 10.1097/CAD.0b013e328325a9ec.

Phase II study of capecitabine plus cisplatin in patients with gastric cancer.

Salah-Eldin MA(1), Ebrahim MA, AL-Ashry MS.

Author information:
(1)Department of Medical Oncology, Mansoura University, Mansoura, Egypt.

manasalah@mans.edu.eg

A phase II study was conducted to assess the efficacy and toxicity of combination therapy with capecitabine and cisplatin in patients with de-novo advanced gastric cancer, and in patients with refractory/recurrent gastric cancer after previous nonplatinum-based therapy. Sixty-four patients were enrolled in the study. Of these, 50 patients had untreated gastric cancer, and 14 had received previous therapy with nonplatinum-based therapy. All patients received oral capecitabine 1250 mg/m2 twice daily, days 1-14, and intravenous cisplatin 60 mg/m2 on day 1. This cycle was repeated every 3 weeks. Among the 50 previously untreated patients, three achieved complete response, and 19 had partial response, giving a response rate of 44% in the intention-to-treat population. The median time to progression and median overall survival were 6 months [95% confidence interval (CI): 1.4-10.6] and 9 months (95% CI: 5.7-12.3), respectively. In patients who had received previous therapy, clinical usefulness was evaluated resulting in response rate of 14%, disease control rate of 28.5%, and median overall survival of 4 months (95% CI: 3.1-4.9). The principal grade 3/4 adverse events were neutropenia (20%), anemia (14%). No neutropenic fever or treatment-related deaths. Capecitabine in combination with cisplatin is effective and well tolerated as first-line treatment in patients with advanced gastric cancer. Unfortunately, we could not positively suggest the usefulness of the same combination regimen as salvage therapy in patients with progressive or recurrent disease after nonplatinum-based therapy.

PMID: 19396018 [PubMed - indexed for MEDLINE]

80. Am J Clin Oncol. 2009 Aug;32(4):356-62. doi: 10.1097/COC.0b013e31818c08e8.

Perineural invasion after preoperative chemotherapy predicts poor survival in patients with locally advanced gastric cancer: gene expression analysis with pathologic validation.

Jhawer M(1), Coit D, Brennan M, Qin LX, Gonen M, Klimstra D, Tang L, Kelsen DP, Shah MA.

Author information:
(1)Department of Medicine, Gastrointestinal Oncology Service, Memorial Sloan-Kettering Cancer Center and Weill School of Medicine, Cornell University of

New York, NY 10021, USA.

BACKGROUND: We examined gene expression profiles and clinicopathologic features (tumor location, stage, graded pathologic response, perineural invasion (PNI), Lauren's classification, and survival) of patients with gastric cancer who received preoperative chemotherapy to identify prognostic markers.
METHODS: Thirty-eight patients with locally advanced gastric cancer received preoperative chemotherapy on a phase II trial. Twelve fresh-frozen tumor samples were available for RNA expression analysis. Differential gene expression between tumors with and without PNI was identified and correlated with clinicopathologic features.
RESULTS: Preliminary hierarchical clustering suggested a separation between long- and short-term survivors. The close association between PNI and overall survival was identified and validated immunohistochemically in 31 completely resected gastric tumors. Five-year survival for patients with PNI and without PNI was 5% and 65%, respectively ($P < 0.01$). PNI added significant prognostic value to posttreatment pathologic stage, ($P < 0.01$). Differential gene expression profile for PNI and non-PNI tumors identified 111 potentially relevant genes.
CONCLUSIONS: Our results demonstrate that the presence of PNI after preoperative chemotherapy is associated with poor survival. These results need to be validated in prospective studies, to help establish whether patients with evidence of PNI would be candidates for more aggressive therapy or enrollment into clinical trials. The presence of PNI provides additional prognostic importance to posttreatment pathologic stage and may indicate treatment resistance. Understanding the molecular events associated with PNI, may provide insight into new therapeutic agents for this subset of patients with resistant tumors.

PMID: 19381079 [PubMed - indexed for MEDLINE]

81. Drugs Aging. 2009;26(3):185-94. doi: 10.2165/00002512-200926030-00001.

Stage-specific therapy for cancer of the oesophagus: a new 'cancer of the elderly'.

Krasna MJ(1).

Author information:
(1)Saint Joseph Cancer Institute, St Joseph Medical Center, Towson, Maryland

21204, USA. markkrasna@catholichealth.net

Oesophageal and gastric cancers are amongst the most frequent and lethal of cancers worldwide. In the US alone, some 13 000 individuals are affected each year, and mortality is particularly high in elderly patients with advanced stage disease and multiple co-morbidities. Patients usually do not present until later in the disease when symptoms occur, once the tumour is sufficiently large to cause obstruction or invasion of adjacent structures. Oesophageal cancer can metastasize to almost any organ, and widespread distant metastases are almost always present at the time of death. Overall mortality from this cancer is around 80-90%. Curative treatment of oesophageal cancer must achieve local control of the primary lesion as well as control and/or prevention of metastases. These are important contributors to overall results when therapy is undertaken in elderly patients, as are the significant risks of adverse effects such as morbidity from chemoradiation and the morbidity and mortality of oesophagectomy. Surgical resection affords the best chance for local control and the best means of palliation of dysphagia for most patients with localized disease, although both local and systemic recurrence of disease are common when surgery is used alone. Because of the low cure rates associated with the use of surgery alone, other modalities have been added to the treatment regimen. Elderly patients with significant cardiac and pulmonary co-morbidity are candidates for nonoperative therapy, even at an early disease stage. There are few data to support a survival advantage from adjuvant radiotherapy or chemotherapy following complete resection, in the absence of documented metastatic disease. Chemotherapy and radiotherapy have both been reported to improve survival when administered preoperatively in patients with oesophageal cancer, while current data using trimodal therapy show a trend towards increased treatment-related mortality with only a slight increase in overall survival. There is currently no completely reliable preoperative method for restaging patients following neoadjuvant chemoradiation in order to assess pathological complete response. Novel restaging techniques are therefore required, in addition to further study of the risks and benefits of neoadjuvant chemoradiotherapy for this disease.

PMID: 19358615 [PubMed - indexed for MEDLINE]

82. Gastroenterol Clin North Am. 2009 Mar;38(1):135-52, ix. doi: 10.1016/j.gtc.2009.01.012.

Preoperative therapy for esophageal cancer.

Ku GY(1), Ilson DH.

Author information:
(1)Ludwig Center for Cancer Immunotherapy, Memorial Sloan-Kettering Cancer Center, 1275 York Avenue, New York, NY 10065, USA.

This article examines the role of combined-modality therapy for treating locally advanced esophageal cancer. Although surgery remains a cornerstone of treatment, recent studies have demonstrated that pre- or perioperative chemotherapy is associated with improved survival for patients who have adenocarcinoma histology. Primary chemoradiotherapy is the accepted standard of care for medically inoperable patients. Recent studies also suggest that definitive chemoradiotherapy is acceptable for patients who have squamous histology, while subsequent surgery improves local control without conferring a clear survival benefit. Neoadjuvant chemoradiotherapy continues to be investigated but is associated with several advantages over neoadjuvant chemotherapy alone, including an improvement in the pathologic complete response rate and resectability. Patients who achieve a pathologic complete response also appear to have improved survival. Adjuvant chemoradiotherapy may be considered for patients who undergo primary resection of lower esophageal/gastroesophageal junction adenocarcinoma.

PMID: 19327572 [PubMed - indexed for MEDLINE]

83. Zhonghua Wei Chang Wai Ke Za Zhi. 2009 Mar;12(2):126-9.

[Application of neoadjuvant chemotherapy in laparoscopic gastrectomy for advanced gastric cancer].

[Article in Chinese]

Zhou DL(1), Zheng CZ, Li JH, Yin K, Ke CW, Liu HT, Hu XG, Chen DL, Zhang DY.

Author information:
(1)Department of General Surgery, Changhai Hospital, The Second Military Medical University, Shanghai 200433, China.

OBJECTIVE: To explore the benefit of neoadjuvant chemotherapy in advanced gastric cancer patients treated by laparoscopy.
METHODS: Fifteen patients with histologically proved gastric adenocarcinomas (stages II(, III(, IIII(M(0)) were treated with FOLFOX7 neoadjuvant chemotherapy followed by laparoscopy between June 2005 and March 2007(trial group). Thirty patients were assigned to the control group with only laparoscopic treatment in the same period. The clinicopathological data were compared between two groups.
RESULTS: All the patients in trial group accepted four cycles of preoperative chemotherapy and the toxicity was less than grade 3. Two of them achieved complete response, 10 achieved partial response and 3 kept stable disease. Ten patients of trial group underwent laparoscopic-assisted radical gastrectomy. The rates of R(0)-resection(80.0%) and pN(0) (60.0%) in trial group were significantly higher than those in control group(46.7% and 20.0%), while the rate of positive lymph node 11.0%(34/309) was significantly lower than that of control group 23.8%(142/596). The operation time and postoperative complication were similar in two groups.
CONCLUSIONS: Advanced gastric cancer after neoadjuvant chemotherapy can be down-regulated in the stage, increase the rate of R(0)-resection, diminish the infiltration extent of tumor, decrease the metastasis of lymph node, and increase the possibility of laparoscopic radical gastrectomy.

PMID: 19296244 [PubMed - indexed for MEDLINE]

84. Gan To Kagaku Ryoho. 2009 Feb;36(2):313-5.

[A case of recurrence after curative total gastrectomy for gastric cancer successfully treated by combination chemotherapy with CPT-11 and CDDP].

[Article in Japanese]

Fujimoto H(1), Urayama M, Kawaguchi K, Ohta K, Seo N.

Author information:
(1)Dept. of Surgery, Yamagata Saisei Hospital.

A male in his sixties underwent total gastrectomy for gastric cancer in August, 2002. After surgery, he underwent 4 courses of adjuvant chemotherapy with S-1. About 2 years after the surgery, PET-CT detected liver metastasis and lymph node

metastasis, so treatment with CPT-11+CDDP was started with the following regimen: 60 mg/m2 CPT-11 and 30 mg/m2 CDDP biweekly. Five months later, PET-CT revealed that the liver metastasis and lymph node metastasis had disappeared. After judgment of complete response(CR), we continued that treatment every four weeks for one year and every eight weeks for another one year. The patient was followed without any recurrence in January, 2007. For this case, PET-CT was effective for planning the treatment and assessing its response.

PMID: 19223754 [PubMed - indexed for MEDLINE]

85. Gan To Kagaku Ryoho. 2009 Jan;36(1):115-8.

[A case of complete response for advanced gastric cancer with liver metastasis treated with combination chemotherapy of weekly paclitaxel and doxifluridine].

[Article in Japanese]

Okabe T(1), Ohya T, Matsumoto H, Tago K, Totsuka O, Numaga Y, Higuchi T, Iesato H, Yokomori T, Kawate S, Takeyoshi I.

Author information:
(1)Dept. of Surgery, Ojiya General Hospital, Japan.

A 68-year-old man underwent total gastrectomy for Type 3 gastric cancer with liver metastasis. The final finding was T3(SE), N1, H1, P0, CY0(class IV), Stage IV, Cur C. After surgery, he was treated with combination chemotherapy of weekly paclitaxel(PTX)/doxifluridine(5'-DFUR). Paclitaxel was administered at a dose of 80 mg/m(2) on day 1, 8 and 15, and doxifluridine was orally administered at a dose of 533 mg/m(2) day for five days followed by withdrawal for two days. This regimen was repeated every four weeks. After 2 courses, the tumor marker level normalized, and the size of the liver metastasis was remarkably decreased. After 5 courses, a CT scan revealed the liver metastasis had disappeared, and he has now survived without recurrence after the disappearance of the liver metastasis. No severe adverse reactions were observed, and the man can be treated as an outpatient. This therapy may thus be effective in the treatment of advanced gastric cancer following non-curative operation.

PMID: 19151575 [PubMed - indexed for MEDLINE]

86. Gan To Kagaku Ryoho. 2009 Jan;36(1):111-3.

[Advanced gastric cancer showing complete response to neoadjuvant chemotherapy with CPT-11 and S-1-a case report].

[Article in Japanese]

Takasu N(1), Nomura T, Fukumoto T, Shibata K, Kamio Y, Hachiya O, Kimura W.

Author information:
(1)Dept. of Gastroenterological and General Surgery, Yamagata University Faculty of Medicine, Japan.

We report a case of advanced gastric cancer that showed a complete histological response to neoadjuvant chemotherapy. The patient, a 56-year-old man, was diagnosed as having advanced gastric cancer with lymph node metastases(cT3 cN1 cH0 cP0 cM0, cStageIIIA). He was initially treated with combined neoadjuvant chemotherapy comprising CPT-11+S-1. S-1(120 mg/day)was administered orally for 21 days, followed by CPT-11(130 mg/body)divon days 1 and 15. The primary lesion and lymph node metastases were diminished by 2 courses of chemotherapy, and no serious toxicities were observed. Distal gastrectomy and lymph node dissection(D2)were performed. Only a small ulcer was observed on the resected stomach. Histological examination of the resected stomach and lymph nodes revealed no remaining viable cancer cells. The patient has been doing well without any recurrence for 1 year since the start of treatment.

PMID: 19151574 [PubMed - indexed for MEDLINE]

87. J Clin Oncol. 2009 Feb 20;27(6):851-6. doi: 10.1200/JCO.2008.17.0506. Epub 2009 Jan 12.

Phase III comparison of preoperative chemotherapy compared with chemoradiotherapy in patients with locally advanced adenocarcinoma of the esophagogastric junction.

Stahl M(1), Walz MK, Stuschke M, Lehmann N, Meyer HJ, Riera-Knorrenschild J, Langer P, Engenhart-Cabillic R, Bitzer M, Königsrainer A, Budach W, Wilke H.

Author information:
(1)Department of Medical Oncology and Hematology, Kliniken Essen-Mitte, Henricistr. 92, D-45136 Essen, Germany. m.stahl@kliniken-essen-mitte.de

Comment in
J Clin Oncol. 2009 Feb 20;27(6):836-7.

PURPOSE: Preoperative chemotherapy is an accepted standard in the treatment of localized esophagogastric adenocarcinoma. Adding radiation therapy to preoperative chemotherapy appears promising, but its definitive value remains unknown.
PATIENTS AND METHODS: Patients with locally advanced (uT3-4NXM0) adenocarcinoma of the lower esophagus or gastric cardia were randomly allocated to one of two treatment groups: induction chemotherapy (15 weeks) followed by surgery (arm A); or chemotherapy (12 weeks) followed by chemoradiotherapy (3 weeks) followed by surgery (arm B). Primary outcome was overall survival time. A total of 354 patients were needed to detect a 10% increase in 3-year survival from 25% to 35% by addition of radiation therapy. The study was prematurely closed due to low accrual.
RESULTS: The median observation time was 46 months. A total of 126 patients were randomly assigned and 119 eligible patients were evaluated. The number of patients undergoing complete tumor resection was not different between treatment groups (69.5% v 71.5%). Patients in arm B had a significant higher probability of showing pathologic complete response (15.6% v 2.0%) or tumor-free lymph nodes (64.4% v 37.7%) at resection. Preoperative radiation therapy improved 3-year survival rate from 27.7% to 47.4% (log-rank P = .07, hazard ratio adjusted for randomization strata variables 0.67, 95% CI, 0.41 to 1.07). Postoperative mortality was nonsignificantly increased in the chemoradiotherapy group (10.2% v 3.8%; P = .26).
CONCLUSION: Although the study was closed early and statistical significance was not achieved, results point to a survival advantage for preoperative chemoradiotherapy compared with preoperative chemotherapy in adenocarcinomas of the esophagogastric junction.

PMID: 19139439 [PubMed - indexed for MEDLINE]

88. Eur J Surg Oncol. 2009 Jul;35(7):739-45. doi: 10.1016/j.ejso.2008.11.005. Epub

2008 Dec 24.

Preoperative imatinib mesylate for unresectable or locally advanced primary gastrointestinal stromal tumors (GIST).

Fiore M(1), Palassini E, Fumagalli E, Pilotti S, Tamborini E, Stacchiotti S, Pennacchioli E, Casali PG, Gronchi A.

Author information:
(1)Department of Surgery, Istituto Nazionale Tumori, via Venezian 1, 20133 Milan, Italy.

AIM: To explore the effect of preoperative imatinib mesylate (IM) in patients with unresectable or locally advanced primary gastrointestinal stromal tumor (GIST).
METHODS: From January 2003 to January 2008, all patients affected by bulky localized GIST who presented at our institution were considered for preoperative IM with cytoreductive intent. Clinical, pathological and molecular characteristics were assessed and the rate of response recorded. Progression-free survival (PFS) was calculated according to Kaplan-Meier analysis.
RESULTS: Fifteen patients (1 esophageal, 7 gastric, 3 duodenal, 4 rectal GISTs) received preoperative IM for a median of 9 months. All patients had tumor shrinkage, with a median size reduction of 34%. One patient had radiological complete response. In all cases an improvement of the originally planned surgical procedure was obtained: 3 patients initially considered unresectable underwent complete surgery; 7 patients with initial indication for extensive surgery were more conservatively operated on; 4 patients initially deemed at high perioperative risk underwent safe surgery. Due to the small sample size, no association between tumor shrinkage and tumor site, size, IM duration, mutational status and pathological response could be formally explored. PFS at 3 years from IM onset was 77%.
CONCLUSIONS: In unresectable or locally advanced GISTs, preoperative IM is a useful tool both to improve resectability and reduce surgical morbidity. It should be therefore always be considered before embarking on a major surgical procedure. The long-term impact of IM on PFS and survival is presently under investigation in multicenter prospective randomized trials.

PMID: 19110398 [PubMed - indexed for MEDLINE]

89. Gan To Kagaku Ryoho. 2008 Nov;35(12):2045-7.

[A case of advanced gastric cancer responding to S-1/paclitaxel was pathologically complete response].

[Article in Japanese]

Aoki T(1), Tanida T, Tsukao Y, Igarashi Y, Komori T, Matsumoto T, Takachi K, Nishioka K, Uemura Y, Kobayashi K.

Author information:
(1)Dept. of Surgery, Kinki Central Hospital.

A 63-year-old man was found to have advanced gastric cancer and staging laparoscopy revealed positivity of peritoneal washing cytology. Since curative surgery was deemed not possible, we started chemotherapy using S-1 (80 mg/m2) orally administered for 2 weeks and paclitaxel (50 mg/m2) administered intravenously on days 1 and 8. After 3 courses of chemotherapy, the primary lesion and regional metastatic lymph node were diminished by CT. We confirmed a peritoneal washing cytology negative by laparoscopic examination. We performed a total gastrectomy and dissection of regional lymph node (D2). Pathologically, cancer cells disappeared both in the main tumor and lymph nodes, and histological efficacy was evaluated as grade 3. Adjuvant chemotherapy was done with same regimen, and he has had no recurrence as of 15 months after surgery.

PMID: 19106518 [PubMed - indexed for MEDLINE]

90. Zhonghua Zhong Liu Za Zhi. 2008 Aug;30(8):573-7.

[Differential gene expression of the inhibitor of apoptosis proteins in docetaxel-resistant gastric cancer cells].

[Article in Chinese]

Wang TT(1), Wei J, Qian XP, Yu LX, Liu BR.

Author information:

(1)Department of Oncology, Drum Tower Hospital Affiliated to Medical School of Nanjing University and Clinical Cancer Institute of Nanjing University, Nanjing 210008, China.

OBJECTIVE: Resistance to chemotherapy may indicate an unfavorable outcome for patients with gastric cancer. The purpose of this study was to examine whether docetaxel-resistance could be due in part to the expression of the inhibitor of apoptosis proteins (IAP).
METHODS: Docetaxel-resistant cells, BGC-823/R1, BGC-823/R2 and BGC-823/R3, were established from parent BGC-823 cells by stepwise increasing concentration of docetaxel. To characterize these cells, we examined the effects of docetaxel on cell growth and apoptosis by MTT assay and double staining with both annexin-V-FITC and PI, and analyzed the cross-resistance to various anticancer drugs. Expression of IAP compared with that in parental cells was evaluated by real-time quantitative PCR.
RESULTS: The BGC-823 resistant cells, BGC-823/R1, R2 and R3 cells, were 10.2-, 24.5-, 56.3-fold more resistant to docetaxel than parental cells, respectively, and this resistance was paralleled with reduced induction of apoptosis. BGC-823/R3 cells showed cross-resistance to paclitaxel, whereas exhibited weak or no cross-resistance against 5-fluorouracil, cisplatin and oxaliplatin. The expressions of survivin and XIAP were gradually increased with the extent of docetaxel resistance ($r = 0.909$, $P < 0.001$ and $r = 0.892$, $P < 0.001$, respectively).
CONCLUSION: IAP may make an important contribution to the resistance to the apoptotic effect of docetaxel in gastric cancer, and could be used as a potential therapeutic target.

PMID: 19102932 [PubMed - indexed for MEDLINE]

91. Hepatogastroenterology. 2008 Sep-Oct;55(86-87):1895-8.

Neoadjuvant chemotherapy versus surgery alone for locally advanced gastric cancer: a retrospective comparative study.

Wang LB(1), Shen JG, Xu CY, Chen WJ, Song XY, Yuan XM.

Author information:
(1)Department of Surgical Oncology, Sir Run Run Shaw Hospital, Zhejiang

University College of Medicine, Hangzhou 310016, China.

BACKGROUND/AIMS: Preoperative chemotherapy is considered an effective treatment option for patients with gastric cancer. We retrospectively evaluated neoadjuvant chemotherapy with oxaliplatin, leucovorin and 5-flurouracil (OLF) in patients with locally advanced gastric cancer to determine its feasibility, as well as impact on the curative resection rate and patients' survival.
METHODOLOGY: A total of 87 patients with locally advanced gastric cancer that underwent preoperative chemotherapy combined with surgery or surgery alone were randomly matched according to the clinical TNM stage. The clinical responses to chemotherapy were assessed. The curative rate, postoperative complications and patients' survival between both groups were compared.
RESULTS: The two groups were well matched. Complete or partial response was observed in 51.7% (15/29) of patients in the OLF group, and three (10.3%) of them had complete pathologic response. The curative resection rates were 89.7% in the OLF group and 77.6% in the surgery alone group. The postoperative complications were equal for both groups. The mean survival is 20.6 months in the OLF group vs. 19.9 months in the surgery alone group (p=0.02).
CONCLUSIONS: Neoadjuvant chemotherapy using OLF combination is active in gastric cancer and the toxicity level is acceptable. This treatment improves the curative resection rate and patients' survival in locally advanced gastric cancer.

PMID: 19102417 [PubMed - indexed for MEDLINE]

92. Expert Rev Anticancer Ther. 2008 Dec;8(12):1953-64. doi: 10.1586/14737140.8.12.1953.

Multimodality therapy for the curative treatment of cancer of the esophagus and gastroesophageal junction.

Ku GY(1), Ilson DH.

Author information:
(1)Immune Monitoring Core Facility, Ludwig Center for Cancer Immunotherapy, Memorial Sloan-Kettering Cancer Center, 1275 York Avenue, NY 10065, USA. kug@mskcc.org

This review examines the role of combined-modality therapy in the treatment of

locally advanced esophageal cancer. While surgery remains a cornerstone of treatment, recent studies have demonstrated that pre- or perioperative chemotherapy is associated with improved survival. Primary chemoradiotherapy is the accepted standard of care for medically inoperable patients. Neoadjuvant chemoradiotherapy continues to be investigated and is associated with several advantages over neoadjuvant chemotherapy alone, including an improvement in the pathologic complete response rate and resectability; patients who achieve a pathologic complete response also appear to have improved survival. Adjuvant chemoradiotherapy may be considered for patients who undergo primary resection of lower esophageal/gastroesophageal junction adenocarcinoma. Future directions include the investigation of novel chemotherapy regimens, the addition of targeted therapies and the use of PET to provide an early assessment of response.

PMID: 19046115 [PubMed - indexed for MEDLINE]

93. Strahlenther Onkol. 2008 Nov;184(11):592-7. doi: 10.1007/s00066-008-1880-9. Epub
2008 Nov 19.

Epidermal growth factor receptor as a predictor of tumor response to preoperative chemoradiation in locally advanced gastric carcinoma.

Sirak I(1), Petera J, Hatlova J, Vosmik M, Melichar B, Dvorak J, Zoul Z, Tycova V, Lesko M, Hajduch M.

Author information:
(1)Department of Oncology & Radiotherapy, Charles University Medical School & University Hospital, Hradec Kralove, Czech Republic. sirak@fnhk.cz

PURPOSE: The purpose of our study was a retrospective evaluation whether the intensity of epidermal growth factor receptor (EGFR) expression predicts tumor response to preoperative chemoradiotherapy in patients with locally advanced gastric carcinoma.
PATIENTS AND METHODS: Thirty-six patients with gastric adenocarcinoma (cT2-4 or N+) were studied. Preoperative treatment consisted of 30-45 Gy of gastric irradiation with continuous 5-fluorouracil and weekly cisplatin. Surgical resection was performed 4-6 weeks later. EGFR expression in pretreatment tumor biopsies was assessed by immunohistochemistry. Level of EGFR expression was

determined from the intensity and extent of staining. Tumor response was defined as a reduction of at least one T-stage level and/or finding of intense tumor regression in histopathologic examination.
RESULTS: Seventeen patients responded to preoperative chemoradiation -- 8 patients (22%) had pathologic complete response, 9 patients (25%) were downstaged. Positive EGFR expression was found in 8 tumors (22%), and represented a significant predictive marker of poor tumor response in multivariate logistic regression analysis (p = 0.015). Response to chemoradiotherapy was found in 60% (16/28) of EGFR negative patients and in 13% (1/8) of EGFR positive patients (p = 0.044). None of the eight EGFR positive patients achieved pathologic complete response in comparison with 8/28 (29%) of patients with EGFR negative staining (p = 0.16).
CONCLUSION: EGFR may represent a molecular marker predictive for poor response to preoperative chemoradiotherapy in locally advanced gastric carcinoma.

PMID: 19016018 [PubMed - indexed for MEDLINE]

94. Zhonghua Zhong Liu Za Zhi. 2008 May;30(5):389-91.

[Preliminary study of biweekly regimen of docetaxel, oxaliplatin, 5-fluorouracil and leucovorin for advanced gastric cancer].

[Article in Chinese]

Wang ZH(1), Guo J, Chen Z, Li CZ, Sheng LJ, Zhou DG, Liu B, Liu J, Wang QC, Zhang EN.

Author information:
(1)Department of Medical Oncology, Shandong Cancer Hospital, Jinan 250117, China. wzhai8778@sina.com

OBJECTIVE: To evaluate the efficacy and toxicity of a biweekly DOF regimen consisting of docetaxel, oxaliplatin, 5-fluorouracil and leucovorin for advanced gastric cancer.
METHODS: The biweekly DOF regimen was administered in 37 advanced gastric cancer patients. Docetaxel, oxaliplatin and leucovorin were given intravenously at a dose of 35 mg/m2, 85 mg/m2 and 200 mg/m2 for 1 h, 2 h and 2 h on D1, respectively, and 5-Fu was administered as continuous intravenous infusion for 48

h at a dose of 1500 mg/m2 on D1 and D2. This regimen was repeated every 2 weeks. The efficacy and toxicity were evaluated after completion of 3 cycles at least.
RESULTS: The overall response rate (RR) of this series was 67.6%, complete response rate and partial response rate were 27.0% and 40.5%, respectively. The time to progression (TTP) was 9.2 months, and median survival time (MST) was 13.7 months. The RRs of 11 chemotherpy-naïve patients and 26 patients pre-treated with chemotherapy were 81.8% and 61.5%, respectively.
CONCLUSION: Our preliminary results showed that this biweekly combination regimen of docetaxel, oxaliplatin, 5-fluorouracil and leucovorin is effective and tolerable for advanced gastric cancer. However, further investigation of this regimen is mandatory.

PMID: 18953843 [PubMed - indexed for MEDLINE]

95. Gan To Kagaku Ryoho. 2008 Oct;35(10):1745-8.

[A case of long survival in Stage IV gastric carcinoma responding to combination treatment with paclitaxel and 5- fluorouracil followed by surgical resection].

[Article in Japanese]

Masumura K(1), Ninomiya M, Nishizaki M, Harano M, Ohno S, Takakura N, Takata S.

Author information:
(1)Department of Surgery, Hiroshima City Hospital.

A 62-year-old female with epigastric pain was diagnosed with Type 4 gastric cancer upon detailed examination. Abdominal computed tomography(CT)revealed metastasis to the paraaortic lymph node and ascites at pelvis, and aspiration cytology of the ascites through vagina was positive(CY1). The clinical stage was determined as T4(panc) N1H0P0CY1M1(LYM), cStageIV. Three courses of neoadjuvant chemotherapy combined with paclitaxel and 5-fluorouracil(FT therapy)were performed. FT therapy showed a substantial reduction of the size of metastatic lymph nodes by sequentialCT examination, which was evaluated as partial response. Surgical resection consisted of total gastrectomy, and D2 lymph node dissection was performed. Operative cytology of ascites proved negative. The pathologic effect on primary lesion and metastatic lymph nodes was diagnosed as Grade 2. Although the prognosis of gastric cancer with carcinomatous peritonitis is poor,

we here reported a patient with StageIV gastric cancer who markedly responded to FT therapy, which made surgical resection possible with the anticipation of extended survival. FT therapy may be a useful method for a patient with StageIVgastric cancer.

PMID: 18931580 [PubMed - indexed for MEDLINE]

96. Gan To Kagaku Ryoho. 2008 Sep;35(9):1573-5.

[A case of advanced gastric cancer with giant lymph node metastasis responding to S-1/CDDP neoadjuvant chemotherapy].

[Article in Japanese]

Matono S(1), Horiuchi H, Kishimoto Y, Sasahara H, Ureshino M, Kashihara M, Sueyoshi S, Shirouzu K.

Author information:
(1)Dept. of Surgery, Omuta City General Hospital.

The patient was a 66-year-old man with advanced gastric cancer and bulky lymph node metastases. Since a radical resection appeared impossible, we tried neoadjuvant chemotherapy (NAC) with S-1/CDDP, expecting down staging of the tumor. S-1 (120 mg/body) was orally administered for 21 days and CDDP (40 mg/body) was administered by intravenous drip on day 8, 15 and 22. Partial response (PR) was obtained after 2 courses, and distal gastrectomy with D2 lymph node dissection was performed. The histological diagnosis revealed complete disappearance of cancer cells in stomach and a few regional lymph node metastasis (3/30). The patient was administered S-1 for one year after operation, and he is well without recurrence at one year and four months postoperatively. We considered the S-1 and CDDP in combination useful as preoperative chemotherapy for advanced gastric cancer.

PMID: 18799914 [PubMed - indexed for MEDLINE]

97. Leuk Lymphoma. 2008 Aug;49(8):1516-22. doi: 10.1080/10428190802210692.

Persistent monoclonality after histological remission in gastric mucosa-associated lymphoid tissue lymphoma treated with chemotherapy and/or surgery: influence of t(11;18)(q21;q21).

Santón A(1), García-Cosio M, Bellosillo B, Rodríguez P, Cristóbal E, Serrano S, Besses C, Abraira V, Salar A, Montalbán C.

Author information:
(1)Department of Pathology, Hospital Ramón y Cajal, Madrid, Spain.

The purpose of this work was to study retrospectively the molecular response and outcome of 19 gastric mucosa associated lymphoid tissue (MALT) lymphoma patients achieving histological remission after chemotherapy or surgery. Immunoglobulin heavy chain variable (IgV(H)) gene rearrangements were studied by PCR in biopsies obtained at diagnosis and follow-up. Presence of t(11;18)(q21;q21) was studied by FISH or RT-PCR. Sequencing analysis of three t(11;18)(q21;q21) positive and two negative lymphomas with persistent monoclonal IgV(H) rearrangements was also performed. Long-term IgV(H) monoclonality was demonstrated in 11/19 patients (58%); in five of them monoclonal rearrangements were present in all samples throughout the follow-up. Persistent IgV(H) monoclonality was detected a median of 49 months after the achievement of histological response and did not condition histological relapse in most cases. All three t(11;18)(q21;q21) positive patients had maintained IgV(H) monoclonality and sequencing analyses revealed the same mutated IgV(H) alleles in the diagnostic and the follow-up samples. Over half of the patients with gastric MALT lymphoma with histological response after chemotherapy and/or surgery have long-term persistent monoclonality. The presence of t(11;18)(q21;q21) seems to condition long-term persistence of the initial lymphoma clone.trade mark.

PMID: 18766964 [PubMed - indexed for MEDLINE]

98. Gan To Kagaku Ryoho. 2008 Aug;35(8):1383-6.

[A case of advanced gastric cancer responding to paclitaxel/CDDP neoadjuvant chemotherapy leading to pathologically complete response].

[Article in Japanese]

Sakon M(1), Sekino Y, Okita K, Kusama K, Seki H, Munakata Y, Hosaka N, Mikami K.

Author information:
(1)Dept. of Surgery, Nagano Municipal Hospital.

A 74-year-old male with advanced gastric cancer(cT3N1M0H0P0CY0, cStage III A)was treated with paclitaxel/ CDDP as neoadjuvant chemotherapy. Paclitaxel (80 mg/m(2)) and CDDP (25 mg/m(2)) were administered on days 1, 8 and 15 as one cycle. After the second course, a significant tumor reduction was obtained. Total gastrectomy, splenectomy, and D2 type nodal dissection were performed. The histological diagnosis revealed complete disappearance of cancer cells in the stomach and all of the lymph nodes, a so-called pathologically complete response. The patient has now been in good health without any recurrence for 9 months after surgery. This case suggests that neoadjuvant chemotherapy with paclitaxel/CDDP is a potential regimen for advanced gastric cancer.

PMID: 18701854 [PubMed - indexed for MEDLINE]

99. Oncol Rep. 2008 Aug;20(2):259-64.

Pathological complete response induced by the combination therapy of S-1 and 24-h infusion of cisplatin in two cases initially diagnosed as inoperable advanced gastric cancer.

Ina K(1), Kataoka T, Takeuchi Y, Fukuoka T, Miwa T, Nishio T, Furuta R, Masaki A, Mori F, Kayukawa S, Nagao S, Ando T, Goto H.

Author information:
(1)Department of Medical Oncology, Nagoya Memorial Hospital, Nagoya 468-8520, Japan. kina@hospy.or.jp

We report on two patients, successfully treated by the combination therapy of S-1 and 24-h infusion of cisplatin (CDDP), who were initially diagnosed with unresectable stage 4 advanced gastric cancer. Each patient had a very good clinical response and underwent curative gastrectomy after completion of 14 and 10 courses of S-1/CDDP chemotherapy, respectively. A microscopically detailed examination of surgically obtained specimens showed the complete disappearance of malignant cells in the two cases. S-1/CDDP combination therapy can, therefore, be

highly active in incurable advanced gastric carcinoma.

PMID: 18636184 [PubMed - indexed for MEDLINE]

100. Chang Gung Med J. 2008 Mar-Apr;31(2):159-66.

Primary gastric diffuse large B-cell lymphoma.

Hung YS(1), Lin TL, Kuo MC, Tang TC, Dunn P, Wang PN, Wu JH, Chang H, Kuo TT, Shih LY.

Author information:
(1)Division of Hematology-Oncology, Department of Internal Medicine, Chang Gung Memorial Hospital, Taipei, Taiwan, R.O.C.

BACKGROUND: The optimal treatment of primary gastric large-cell non-Hodgkin's lymphoma (PGL) has not been defined. Recent studies have suggested that organ-preserving treatment produces the same results as surgical treatment.
METHODS: We retrospectively reviewed the data of 88 patients diagnosed with PGL between 1995 and 2003 at Chang Gung Memorial Hospital. Sixty-two patients received chemotherapy (CT), three received CT followed by radiotherapy (CT+RT), three received surgery (ST), 14 received surgery followed by CT (ST+ adjuvant CT), one patient received ST followed by radiotherapy (ST+RT), one patient received radiotherapy (RT) alone, one received eradication therapy for Helicobacter pylori only and 3 patients received no further therapy after diagnosis.
RESULTS: Of the 81 patients who received endoscopic biopsy of gastric lesions, the diagnosis of PGL could be made in all but one. Seven patients were diagnosed by pathology after ST without preoperative pathologic diagnosis. The complete remission rate was 77.3%. The 5-year overall survival (OS) and disease-free survival (DFS) were 50.0% and 81.6%, respectively. There was no difference in OS ($p = 0.4051$) and DFS ($p = 0.8519$) between patients receiving mainly CT (CT or CT+RT) and those receiving primary surgery (ST, ST+ adjuvant CT or ST+RT). We found that poor performance status ($p < 0.0001$), elevated beta2-microglobulin level ($p = 0.0082$) and no CT ($p = 0.0002$) had adverse effects on OS.
CONCLUSION: The present data show that CT should be the primary treatment for patients with PGL if the diagnosis can be made with endoscopic biopsy.

PMID: 18567416 [PubMed - indexed for MEDLINE]

101. Abdom Imaging. 2009 Jul;34(4):430-40. doi: 10.1007/s00261-008-9420-8.

Usefulness of CT volumetry for primary gastric lesions in predicting pathologic response to neoadjuvant chemotherapy in advanced gastric cancer.

Lee SM(1), Kim SH, Lee JM, Im SA, Bang YJ, Kim WH, Kim MA, Yang HK, Lee HJ, Kang WJ, Han JK, Choi BI.

Author information:
(1)Department of Radiology, Seoul National University College of Medicine, Seoul, Korea.

BACKGROUND: To investigate the utility of CT volumetry for primary gastric lesions in the prediction of pathologic response to neoadjuvant chemotherapy in patients with resectable advanced gastric cancer (AGC).
MATERIALS AND METHODS: Thirty-three consecutive patients with resectable AGC stage >or=T2 and N1), who had been treated with neoadjuvant chemotherapy and radical gastric resection, were prospectively enrolled in this study. There were 30 men and 3 women with a mean age of 53.8 years. Contrast-enhanced CT was obtained after gastric distention with air before and after chemotherapy using a MDCT scanner. Pre- and post-chemotherapy thickness or short diameter and volume of the primary gastric tumor and largest lymph node (LN), were measured using a dedicated 3D software by two radiologists in consensus. PET/CT was also performed and the peak standardized uptake value (SUV) of primary gastric tumor and largest LN before and after chemotherapy was measured. The percentage diameter, volume, and SUV reduction rates for both the primary gastric tumor and the LN, were calculated and correlated with the histopathologic grades of regression using the Spearman correlation test. Differentiation between pathologic responders and nonresponders was assessed using receiver operating characteristic (ROC) analysis.
RESULTS: Among the three CT parameters which showed significant correlation with the histopathologic grades of regression, the correlation factor was highest in the percentage volume reduction rate of primary gastric tumor (rho = 0.484, P = 0.004) followed by percentage volume reduction of the index node (rho = 0.397, P = 0.022), and percentage diameter reduction of the index node (rho = 0.359, P = 0.04). However, the percentage thickness decrease rate (P = 0.208) and the

percentage SUV reduction rate (P = 0.619) of primary gastric tumor were not significantly correlated with the histopathologic grades of regression. When the optimal cutoff value of the percentage volume reduction rate of primary gastric tumor was determined to be 35.6%, a sensitivity of 100% (16/16) and a specificity of 58.8% (10/17) were achieved.
CONCLUSION: CT volumetry for primary gastric tumor may be the most accurate tool in the prediction of pathologic response following neoadjuvant chemotherapy in patients with resectable AGC.

PMID: 18546037 [PubMed - indexed for MEDLINE]

102. Clin Adv Hematol Oncol. 2008 May;6(5):371-9.

Preoperative therapy in esophageal cancer.

Ku GY(1), Ilson DH.

Author information:
(1)Gastrointestinal Oncology Service, Department of Medicine, Memorial Sloan-Kettering Cancer Center, New York, NY 10065, USA.

Progress has been made in the treatment of locally advanced esophageal cancer. Preoperative and postoperative chemotherapy also appears to improve survival in gastroesophageal junction adenocarcinoma compared to surgery alone. Adding radiotherapy to preoperative chemotherapy enhances rates of curative resection, achieves measurable rates of pathologic complete response, and recent trials indicate a survival benefit for preoperative chemoradiotherapy compared to surgery alone in esophageal cancer. Given the achievement of pathologic complete responses with combined chemoradiotherapy in esophageal cancer, recent trials have evaluated the contribution of surgery after chemoradiotherapy. With currently available systemic therapy for squamous cancers of the esophagus that respond to combined chemoradiotherapy, there is no clear survival benefit for the addition of surgery after chemoradiotherapy despite improvements in local tumor control with the addition of surgery. Surgery may salvage nonresponding patients with biopsy-positive residual disease. For adenocarcinoma of the esophagus, a histology with consistently lower rates of pathologic complete response than squamous cell cancer, surgery appears to play a greater role. Trials are now evaluating the use of newer chemotherapy agents combined with radiotherapy,

including taxanes, irinotecan, and oxaliplatin. Response on postiron emission tomography early on during induction chemotherapy may be a strong prognostic measure of outcome. Targeted agents, including monoclonal antibodies that target the epidermal and vascular endothelial growth factor receptors, are in active development in phase II and III trials.

PMID: 18516027 [PubMed - indexed for MEDLINE]

103. Surg Oncol Clin N Am. 2008 Jul;17(3):485-502, vii-viii. doi: 10.1016/j.soc.2008.02.007.

Multidisciplinary treatment of advanced cancer of the esophagus and gastroesophageal junction: a European center's approach.

Lerut T(1), Moons J, Coosemans W, Decaluwé H, Decker G, De Leyn P, Nafteux P, Van Raemdonck D.

Author information:
(1)Department of Thoracic Surgery, University Hospitals Leuven, Herestraat 49, 3000 Leuven, Belgium. toni.lerut@uzleuven.be

Tremendous progress has been made in surgery for cancer of the esophagus and gastroesophageal junction. After primary surgery, overall 5-year survival rates of 35% or more are obtained in high-volume units, and for advanced stage III cancer, 5-year survival reaches 25%. Multimodality therapy, in particular induction chemotherapy with or without radiotherapy, results in a complete response rate in up to 25% of the patients. Approximately 50% of the patients receiving such treatment do not respond, however, and their outcome is dismal. Therefore, further efforts are needed to elaborate more precise algorithms for selecting candidates for induction therapy versus primary surgery.

PMID: 18486879 [PubMed - indexed for MEDLINE]

104. Zhonghua Zhong Liu Za Zhi. 2007 Nov;29(11):867-70.

[Evaluation of efficacy and influence factors of transarterial interventional therapy in patients with liver metastasis from malignancy of alimentary tract].

[Article in Chinese]

Yan D(1), Li H, Wei WQ, Liu DZ, Zeng HY, Yang LX.

Author information:
(1)Department of Diagnostic Radiology, Cancer Hospital (Institute), Chinese Academy of Medical Sciences, Beijing 100021, China.

OBJECTIVE: To evaluate the efficacy and prognostic factors of transarterial interventional therapy (TAIT) in patient with liver metastasis from malignancy of the alimentary tract.
METHODS: 266 patients with unresectable liver metastases from malignancy of the alimentary tract received totally 754 sessions of transarterial interventional therapy. Cox regression was used in the proportional hazard analysis.
RESULTS: The overall response rate of TAIT was 45.4%, The median survival time (MS) was 14.3 months in this series. The 0.5-, 1-, 2-, 3-, 5-year cumulative survival rate (CSR) was 83.1%, 56.8%, 17.7%, 9.3% and 1.5% , respectively. No severe adverse reaction was observed except nausea, vomiting and mild fever as well as pain in the hepatic area. It was found that portal vein tumor thrombosis (PVTT), the blood supply of tumor, metastasis from esophageal carcinoma, the number of metastasis, multi-lobe involvement, resection nature of primary tumor were independent factors affecting survival.
CONCLUSION: Transarterial interventional therapy is effective for treatment of liver metastasis from malignancy of the alimentary tract. Portal vein tumor thrombosis, metastasis from esophageal carcinoma, multiple metastatic lesions, multi-lobe involvement are poor prognostic factors, while complete resection of the primary tumor and rich blood supply of metastatic lesion are good independent prognostic factors.

PMID: 18396650 [PubMed - indexed for MEDLINE]

105. Gan To Kagaku Ryoho. 2008 Mar;35(3):499-501.

[A case of advanced gastric cancer responding to neoadjuvant S-1/CDDP therapy].

[Article in Japanese]

Matsui K(1), Nashimoto A, Nakagawa S, Nomura T, Yabusaki H, Takii Y, Tsuchiya Y, Tanaka O, Ohta T.

Author information:
(1)Dept. of Surgery, Niigata Cancer Center Hospital, Japan.

A 77-year-old male had complaints of epigastralgia. Gastrointestinal endoscopic examination revealed type 2 advanced gastric cancer. Computed tomography revealed metastatic Bulkey group 2 lymph nodes. The diagnosis was sStage IIIB gastric cancer (sT3 sN2sH0 sP0 CY0) at staging laparoscopy. S-1 (100 mg/body/day) was orally administered for 3 weeks followed by a drug-free 2 weeks, and CDDP (74 mg/body/day) was given intravenously on day 8. After 3 courses of chemotherapy, the primary lesion and the regional lymph nodes were significantly reduced in size. He was judged as clinical PR, followed by total gastrectomy, splenectomy and lymph node dissection. The pathological findings showed that there were very few cancer cells in the primary lesion, and lymph nodes had become scarred and fibrous. The final diagnosis was T2N0 H0 P0, fStageIB and curability A.

PMID: 18347404 [PubMed - indexed for MEDLINE]

106. Cancer Chemother Pharmacol. 2008 Dec;63(1):1-8. doi:
10.1007/s00280-008-0701-2.
Epub 2008 Feb 21.

A phase II study of irinotecan and docetaxel combination chemotherapy for patients with previously treated metastatic or recurrent advanced gastric cancer.

Sym SJ(1), Chang HM, Kang HJ, Lee SS, Ryu MH, Lee JL, Kim TW, Yook JH, Oh ST, Kim BS, Kang YK.

Author information:
(1)Department of Medicine, Division of Oncology, Asan Medical Center, University of Ulsan College of Medicine, Songpa Gu, Seoul, South Korea.

PURPOSE: Irinotecan (I) and docetaxel (D), each of which has a unique mechanism of action, were recently introduced in the treatment of patients with advanced gastric cancer (AGC). We have evaluated the efficacy and safety of the ID combination for AGC patients after failure of fluoropyrimidine- or platinum-based

chemotherapy.
MATERIALS AND METHODS: Patients with relapsed or progressive AGC after prior fluoropyrimidine- or platinum-based chemotherapy were treated with I (160 mg/m(2), 90 min) followed by D (65 mg/m(2), 1 h) every 3 weeks. Because of the unacceptable toxicity among the first ten patients, the doses were reduced for I (120 mg/m(2)) and D (50 mg/m(2)) every 3 weeks.
RESULTS: Forty-nine patients, of median age 53 years (range, 27-68 years), were treated with 170 cycles of chemotherapy (median, 2 cycles; range, 1-12 cycles). Three patients achieved complete response and seven achieved partial response, resulting in an overall response rate (ORR) of 20.4% [95% confidence interval (CI), 9.1-31.7%], with a median duration of 7.1 months (range: 2.1-69.1 months). ORR was 60% (95% CI, 29.6-90.3%) for the higher dose and 10.3% (95% CI, 0.7-19.8%) for the lower dose. Median time to progression for all patients was 2.7 months (95% CI, 1.7-3.8 months) and the median overall survival was 8.9 months (95% CI, 6.6-11.3 months). Grade 3/4 toxicities included neutropenia (90%), febrile neutropenia (50%), asthenia (40%), and diarrhea (10%) with the higher dose and neutropenia (71%), febrile neutropenia (11%), diarrhea (24%), and asthenia (24%) with the lower dose. There were two possible treatment-related deaths.
CONCLUSION: The combination of irinotecan and docetaxel, once every three weeks shows anti-tumor activity but is not feasible as a second-line treatment for AGC patients after failure of fluoropyrimidine- or platinum-based chemotherapy due to the high rate of toxicities.

PMID: 18288477 [PubMed - indexed for MEDLINE]

107. Br J Cancer. 2008 Jan 29;98(2):316-22. doi: 10.1038/sj.bjc.6604186. Epub 2008 Jan
22.

A phase II study of paclitaxel and capecitabine as a first-line combination chemotherapy for advanced gastric cancer.

Kang HJ(1), Chang HM, Kim TW, Ryu MH, Sohn HJ, Yook JH, Oh ST, Kim BS, Lee JS, Kang YK.

Author information:
(1)Division of Oncology, Department of Medicine, University of Ulsan College of

Medicine, Asan Medical Center, Seoul, Korea.

Paclitaxel and capecitabine, which have distinct mechanisms of action and toxicity profiles, have each shown high activity as single agents in gastric cancer. Synergistic interaction between these two drugs was suggested by taxane-induced upregulation of thymidine phosphorylase. We, therefore, evaluated the antitumour activity and toxicities of paclitaxel and capecitabine as first-line therapy in patients with advanced gastric cancer (AGC). Patients with histologically confirmed unresectable or metastatic AGC were treated with capecitabine 825 mg m(-2) p.o. twice daily on days 1-14 and paclitaxel 175 mg m(-2) i.v. on day 1 every 3 weeks until disease progression or unacceptable toxicities. Between June 2002 and May 2004, 45 patients, of median age 57 years (range=38-73 years), were treated with the combination of capecitabine and paclitaxel. After a median 6 cycles (range=1-9 cycles) of chemotherapy, 43 were evaluable for toxicity and response. A total of 2 patients showed complete response and 20 showed partial response making the overall response rate 48.9% (95% CI=30.3-63.5%). After a median follow-up of 42.2 months (range=31.2-54.3 months), median time to progression was 5.6 months (95% CI=3.9-7.2 months) and median overall survival was 11.3 months (95% CI=8.1-14.4 months). Grade 3 or 4 adverse events include neutropaenia (46.5% of patients), hand-foot syndrome (9.3%), arthralgia (9.3%), and asthenia (4.7%). There was no neutropaenic fever or treatment-related deaths. Paclitaxel and capecitabine combination chemotherapy was active and highly tolerable as a first-line therapy for AGC.

PMCID: PMC2361449
PMID: 18219288 [PubMed - indexed for MEDLINE]

108. Int J Radiat Oncol Biol Phys. 2008 Jul 1;71(3):741-7. doi: 10.1016/j.ijrobp.2007.10.030. Epub 2007 Dec 31.

Incidence, natural history, and patterns of locoregional recurrence in gastric cancer patients treated with preoperative chemoradiotherapy.

Reed VK(1), Krishnan S, Mansfield PF, Bhosale PR, Kim M, Das P, Janjan NA, Delclos ME, Lowy AM, Feig BW, Pisters PW, Ajani JA, Crane CH.

Author information:
(1)Department of Radiation Oncology, The University of Texas MD Anderson Cancer

Center, Houston, TX 77030, USA.

PURPOSE: To retrospectively determine the incidence and patterns (in-field, marginal, or out-of-field) of locoregional gastric cancer recurrence in patients who received preoperative chemoradiotherapy and to determine the outcome in these patients.
METHODS AND MATERIALS: Between 1994 and 2004, 149 patients with gastric carcinoma were treated according to institutional protocols with preoperative chemoradiotherapy. Ultimately, 105 patients had an R0 resection. Of these 105 patients, 65 received preoperative chemotherapy followed by chemoradiotherapy and 40 received preoperative chemoradiotherapy. Most (96%) of these patients received 5-fluorouracil-based chemotherapy during radiotherapy, and the median radiation dose was 45 Gy. We retrospectively identified and classified the patterns of locoregional recurrence.
RESULTS: The 3-year actuarial incidence of locoregional recurrence was 13%, with locoregional disease recurring as any part of the failure pattern in 14 patients. Most (64%) of the evaluable locoregional recurrences were in-field. Of the 4 patients with a marginal recurrence, 2 had had inadequate coverage of the regional nodal volumes on their oblique fields. The pathologic complete response rate was 23%. A pathologic complete response was the only statistically significant predictor of locoregional control.
CONCLUSION: Patients with gastric cancer who received preoperative chemoradiotherapy had low rates of locoregional recurrence. This strategy merits prospective multi-institutional and randomized evaluation.

PMID: 18164837 [PubMed - indexed for MEDLINE]

109. Gan To Kagaku Ryoho. 2007 Dec;34(13):2297-300.

[Complete histological response in gastric cancer stage IV after neoadjuvant chemotherapy including S-1 combined with CDDP--report of a case].

[Article in Japanese]

Fujisawa T(1), Sano W, Ouchi S, Ueyama S, Mori T, Tsuchihashi D, Uchikoga O, Koyanagi M, Yoshimura H, Tachibana S, Hirano H.

Author information:

(1)Dept. of Internal Medicine, Nippon Steel Hirohata Hospital.

A 60-year-old man complaining of black stool, body weight loss, and anemia, was examined and diagnosed with advanced gastric cancer (M, type 3, por 2, cT3, cN3, cH0, cP0, cM0, cStage IV). A poor prognosis was predicted, yet we tried neoadjuvant chemotherapy (NAC) expecting downstaging of the tumor. Considering the efficacy and safety, we chose S-1+CDDP as the NAC regimen. S-1 (120 mg/day) was administered orally for 21 days, followed by CDDP (75 mg/body) div on day 8. Distal partial gastrectomy and lymph node dissection (D2) were performed, with Billroth I reconstruction. Histological examination of the resected stomach and lymph nodes revealed no residual cancer cells, suggesting complete histological remission (grade 3) according to the Japanese classification of gastric carcinoma.

PMID: 18079634 [PubMed - indexed for MEDLINE]

110. Int J Clin Oncol. 2007 Dec;12(6):472-7. Epub 2007 Dec 21.

Complete response of a patient with advanced gastric cancer, showing Epstein-Barr virus infection, to preoperative chemotherapy with S-1 and cisplatin.

Seya T(1), Tanaka N, Yokoi K, Ishikawa N, Horiba K, Kanazawa Y, Yamada T, Koizumi M, Shinji S, Okazaki H, Ohaki Y, Ishiwata T, Naito Z, Tajiri T.

Author information:
(1)Department of Surgery, Chiba-Hokusoh Hospital, Nippon Medical School, Inbamura, Chiba, Japan. seya@nms.ac.jp

Here we report the case of a patient with advanced gastric cancer with esophageal invasion who was treated with chemotherapy using S-1 and cisplatin (CDDP) preoperatively. The patient was a 72-year-old woman who was diagnosed with advanced gastric cancer (T3N2M0) with esophageal invasion. S-1 was orally administered at 80 mg/day (60 mg/m(2) per day) on days 1-14 and CDDP was infused at 80 mg/day (60 mg/m(2) per day) on day 8, followed by a 1-week rest. Marked reductions in the sizes of the primary tumor and metastatic lymph nodes around the stomach were observed after two cycles of the therapy. Adverse reactions occurring during the therapy were only grade 2 gastrointestinal disorder and grade 1 leukocytopenia. Radiological and endoscopic examinations before surgery

showed that a partial response (PR) had been achieved. The patient underwent curative surgery consisting of total gastrectomy, D2 lymph node dissection, and splenectomy. Her postoperative course was uneventful, without surgical complications. No gastric cancer cells were detected in the primary lesion or lymph nodes by immunohistochemical staining with cytokeratin, confirming a histological complete response (CR). As Epstein-Barr virus-encoded small RNA (EBER) had been detected by in-situ hybridization in the gastric cancer cells of a biopsy specimen, this tumor was diagnosed as an Epstein-Barr virus (EBV)-associated gastric carcinoma (EBVaGC), which was effectively treated with S-1 and cisplatin chemotherapy.

PMID: 18071868 [PubMed - indexed for MEDLINE]

111. Gan To Kagaku Ryoho. 2007 Nov;34(11):1853-6.

[A case of advanced gastric cancer with peritoneal dissemination responding to S-1/CDDP neoadjuvant chemotherapy].

[Article in Japanese]

Mizutani S(1), Oyama T, Uchikoshi F, Yoshidome K, Tori M, Ueshima S, Hiraoka K, Yamagami Y, Takahashi H, Nakahara M.

Author information:
(1)Dept. of Surgery, Osaka Police Hospital.

The patient was a 72-year-old male diagnosed with type III poorly-differentiated adenocarcinoma in the lesser curvature by gastric fiberscopy. An abdominal computed tomography (CT) scan showed the thickness of the gastric wall and the enlarged lymph node around the stomach and laparoscopic examination revealed peritoneal dissemination. The patient received neoadjuvant combined chemotherapy with S-1 and CDDP. S-1 (100 mg/day) was orally administered for 3 weeks followed by 2 drug-free weeks as a course, and CDDP (100 mg/body) was administered by intravenous drip on day 8. After the third course, significant tumor reduction was obtained. Total gastrectomy, splenectomy and D2 nodal dissection were performed. Peritoneal dissemination disappeared, and the histological diagnosis revealed complete disappearance of cancer cells in the ascites and no metastasis in all lymph nodes. The patient has now been in good health with no recurrence

for 22 months after surgery. The combined neoadjuvant chemotherapy with S-1 and CDDP can be an effective treatment of choice for advanced gastric carcinoma with peritoneal dissemination.

PMID: 18030023 [PubMed - indexed for MEDLINE]

112. Int J Radiat Oncol Biol Phys. 2008 May 1;71(1):173-9. Epub 2007 Nov 8.

Phase II study of chemoradiotherapy with S-1 and low-dose cisplatin for inoperable advanced gastric cancer.

Saikawa Y(1), Kubota T, Kumagai K, Nakamura R, Kumai K, Shigematsu N, Kubo A, Kitajima M, Kitagawa Y.

Author information:
(1)Department of Surgery, Keio University School of Medicine, Shinjuku-ku, Tokyo, Japan. saiky@sc.itc.keio.ac.jp

PURPOSE: The results of a pilot study using S-1/low-dose cisplatin/radiotherapy led us to hypothesize that the initial chemoradiotherapy regimen would induce a 70% efficacy rate with a 10% pathologic complete response rate.
PATIENTS AND METHODS: Only patients with unresectable or incurable advanced gastric cancer were eligible. The patients received induction S-1 and cisplatin therapy with radiotherapy followed by chemotherapy alone.
RESULTS: Of the 30 patients recruited and assessed, 29 were eligible for clinical evaluation of measurable lesions. The response rate was 65.5%, with 19 with a partial response, 8 with no change, and 2 with progressive disease of 29 patients. Of the 30 patients recruited, 10 (33.3%) underwent stomach resection and D2 LN dissections. The pathologic complete response rate was 13.3% (4 patients), and the R0 resection rate was 100% (10 patients). The survival analysis showed a median survival time of 25 months. Grade 3 toxicity occurred in 66.7% for leukocytopenia, 33.3% for thrombocytopenia, 23.3% for nausea and appetite loss, and 6.7% for anemia, diarrhea, and renal dysfunction. Although all the patients had been hospitalized with a poor performance status with a giant tumor, 97% (29 of 30) could be discharged after the first cycle, resulting in an improvement in quality of life.
CONCLUSION: Chemoradiotherapy could be a powerful regimen for controlling tumor progression in advanced gastric cancer, improving patients' quality of life with

tolerable toxicity. A complete histologic response rate of >10% would be expected, even for large tumors with metastatic lesions.

PMID: 17996385 [PubMed - indexed for MEDLINE]

113. Intern Med. 2007;46(21):1783-7. Epub 2007 Nov 1.

Long-term remission of primary gastric T cell lymphoma associated with human T lymphotropic virus type 1: a report of two cases and review of the literature.

Tanaka K(1), Nakamura S, Matsumoto T, Hirakawa K, Yanaru-Fujisawa R, Onoyama K, Sakata H, Ohshima K, Yao T, Iida M.

Author information:
(1)Department of Medicine and Clinical Science, Graduate School of Medical Sciences, Kyushu University, Fukuoka, Japan. k-ichi@intmed2.med.kyushu-u.ac.jp

Two cases of primary gastric T-cell lymphoma associated with human T lymphotropic virus type 1 (HTLV-1) are presented. Case 1 was a 54-year-old man who had multiple ulcerating tumors in the lower corpus and gastric antrum. Case 2, a 60-year-old man, showed a large ulcerating tumor in the upper corpus. Both patients were positive for serum anti-HTLV-1 antibody and for the monoclonal integration of HTLV-1 proviral DNA in the tumor cells by Southern blot analysis. The patients were thus diagnosed as having primary gastric T-cell lymphoma associated with HTLV-1 of stage II(1). Case 1 underwent total gastrectomy followed by chemotherapy, while Case 2 was treated by chemotherapy and radiotherapy. Both patients have been in complete remission for more than 4 years (96 months in Case 1 and 50 months in Case 2) after the treatments. Although primary gastric T-cell lymphomas associated with HTLV-1 is characterized by an extremely poor prognosis, the present cases suggest that in the early stage, long-term survival can possibly be achieved with appropriate treatments.

PMID: 17978535 [PubMed - indexed for MEDLINE]

114. Ann Surg Oncol. 2007 Dec;14(12):3412-8. Epub 2007 Oct 2.

Does graded histologic response after neoadjuvant chemotherapy predict survival

for completely resected gastric cancer?

Mansour JC(1), Tang L, Shah M, Bentrem D, Klimstra DS, Gonen M, Kelsen DP, Brennan MF, Coit DG.

Author information:
(1)Memorial Sloan-Kettering Cancer Center, 1275 York Avenue, New York, NY 10021, USA.

Comment in
Ann Surg Oncol. 2008 Jun;15(6):1795-7; author reply 1798-9.
Ann Surg Oncol. 2007 Dec;14(12):3290-2.

BACKGROUND: After publication of the MAGIC trial results, preoperative chemotherapy is increasingly used to treat advanced gastric cancer before resection. Tools for measuring response must be assessed.
METHODS: We identified all patients with gastric cancer treated with neoadjuvant chemotherapy and R0 resection between 1991 and 2005 from a prospective database. Patients receiving preoperative radiation were excluded. Histologic response to treatment was graded from 0% to 100% by a single pathologist. Kaplan-Meier survival analysis was performed to identify the relationship between response and outcome and to identify factors predictive of disease-specific survival (DSS). Multivariate analysis was performed to identify independent predictors.
RESULTS: A total of 168 patients underwent R0 resection after receiving neoadjuvant chemotherapy. Thirty-three percent of tumors were at the gastroesophageal junction. Cisplatin-based therapy was used for 68% of patients. Twenty-two percent of patients had a >50% pathologic response to treatment. Median follow-up after resection for all patients was 25 months. Median DSS for all patients was 33 months. Three-year DSS improved from 44% to 69% with at least a 50% histologic response (P = .01). Factors associated with decreased DSS included positive nodes at resection, pT3 tumor or greater, high grade, perineural or vascular invasion, and <50% response. Multivariate analysis identified nodal status and perineural or vascular invasion as independent predictors of survival.
CONCLUSIONS: Posttreatment nodal status and perineural or vascular invasion at resection, but not graded histologic response, independently predict DSS after neoadjuvant chemotherapy and surgical resection of gastric cancer.

PMID: 17909917 [PubMed - indexed for MEDLINE]

115. Int J Radiat Oncol Biol Phys. 2007 Nov 1;69(3):770-6.

Paclitaxel-based chemoradiotherapy in the treatment of patients with operable esophageal cancer.

Kelsey CR(1), Chino JP, Willett CG, Clough RW, Hurwitz HI, Morse MA, Bendell JC, D'Amico TA, Czito BG.

Author information:
(1)Department of Radiation Oncology, Duke University Medical Center, Durham, NC 27710, USA. kelse003@mc.duke.edu

PURPOSE: To compare a neoadjuvant regimen of cisplatin/5-fluorouracil (5-FU) and concurrent radiation therapy (RT) with paclitaxel-based regimens and RT in the management of operable esophageal (EC)/gastroesophageal junction (GEJ) cancer.
METHODS AND MATERIALS: All patients receiving neoadjuvant chemotherapy (CT) and RT for EC/GEJ cancer at Duke University between January 1995 and December 2004 were included. Clinical end points were compared for patients receiving paclitaxel-based regimens (TAX) vs. alternative regimens (non-TAX). Local control (LC), disease-free survival (DFS), and overall survival (OS) were estimated using the Kaplan-Meier method. Chi-square analysis was performed to test the effect of TAX on pathologic complete response (pCR) rates and toxicity.
RESULTS: A total of 109 patients received CT-RT followed by esophagectomy (95 M; 14 F). Median RT dose was 45 Gy (range, 36-66 Gy). The TAX and non-TAX groups comprised 47% and 53% of patients, respectively. Most (83%) TAX patients received three drug regimens including platinum and a fluoropyrimidine. In the non-TAX group, 89% of the patients received cisplatin and 5-FU. The remainder received 5-FU or capecitabine alone. Grade 3-4 toxicity occurred in 41% of patients receiving TAX vs. 24% of those receiving non-TAX ($p = 0.19$). Overall pCR rate was 39% (39% with TAX vs. 40% with non-TAX, $p = 0.9$). Overall LC, DFS, and OS at 3 years were 80%, 34%, and 37%, respectively. At 3 years, there were no differences in LC (75% vs. 85%, $p = 0.33$) or OS (37% vs. 37%, $p = 0.32$) between TAX and non-TAX groups.
CONCLUSIONS: In this large experience, paclitaxel-containing regimens did not improve pCR rates or clinical end points compared to non-paclitaxel-containing regimens.

PMID: 17889266 [PubMed - indexed for MEDLINE]

116. Cancer J. 2007 May-Jun;13(3):162-7.

Esophageal cancer: adjuvant therapy.

Ku GY(1), Ilson DH.

Author information:
(1)Department of Medicine, Memorial Sloan-Kettering Cancer Center and Weill-Cornell University Medical College, New York, NY 10021, USA.

In the United States, esophageal cancer is an uncommon but aggressive malignancy. Prior research has focused on the incorporation of chemotherapy and radiotherapy in both the pre- and postoperative setting. Both squamous cell and adenocarcinoma histologies have been treated in trials, with adenocarcinoma now the predominant histology seen in the United States. Although preoperative chemotherapy improves survival compared with surgery alone, the addition of concurrent radiotherapy to preoperative chemotherapy improves rates of curative resection, reduces local tumor recurrence, and achieves a significant rate of pathologic complete response. Combined preoperative chemoradiotherapy is the preferred preoperative strategy for locally advanced esophageal cancers in the United States. Definitive chemoradiotherapy alone appears to be equivalent in terms of overall survival compared with chemoradiotherapy followed by surgery in squamous cancers, although the addition of surgery after chemoradiotherapy may afford superior local control of disease. Postoperatively, survival is improved with postoperative chemotherapy and radiotherapy in adenocarcinoma of the gastroesophageal junction, if none has been delivered preoperatively. Ongoing research involves evaluating regimens with newer chemotherapeutic drugs, such as paclitaxel or irinotecan, as well as the incorporation of targeted molecular therapies.

PMID: 17620765 [PubMed - indexed for MEDLINE]

117. Clin Transl Oncol. 2007 May;9(5):335-8.

Pathological complete response following docetaxel-based neoadjuvant chemotherapy for locally advanced gastric adenocarcinoma.

Bueno Muiño C(1), Puente Vázquez J, Sastre Valera J, García-Sáenz JA, Martín M, García Miralles N, Sánchez-Pernaute A, Díaz-Rubio E.

Author information:
(1)Servicio de Oncología Médica, Hospital Clínico San Carlos, Madrid, Spain. corabumu@hotmail.com

Locally advanced gastric adenocarcinoma has a poor outcome. Neoadjuvant treatment is being tested in locally advanced non-resectable tumours and in those resectable tumours with a high risk of recurrence. Efforts to identify prognostic factors and more active and less toxic preoperative regimens are being searched for. We report the case of a patient achieving a complete histopathological complete response following docetaxel- based neoadjuvant chemotherapy.

PMID: 17525046 [PubMed - indexed for MEDLINE]

118. Am J Clin Oncol. 2007 Apr;30(2):172-80.

A phase II trial of accelerated multimodality therapy for locoregionally advanced cancer of the esophagus and gastroesophageal junction: the impact of clinical heterogeneity.

Adelstein DJ(1), Rice TW, Rybicki LA, Saxton JP, Videtic GM, Murthy SC, Zuccaro G, Vargo JJ, Dumot JA, Carroll MA.

Author information:
(1)The Taussig Cancer Center and the Department of Solid Tumor Oncology, Cleveland Clinic, Cleveland, Ohio 44195, USA. adelstd@ccf.org

OBJECTIVES: This is a report of mature results from a phase II trial of an accelerated multimodality treatment program for locoregionally advanced cancer of the esophagus and gastroesophageal junction with a focus on the impact of clinical heterogeneity on outcomes. A split course of pre- and postoperative hyperfractionated radiation therapy and concurrent chemotherapy was used in an effort to limit perioperative mortality.
METHODS: Eligibility required a diagnosis of esophageal or gastroesophageal junction cancer and an esophageal ultrasound stage of at least T3, N1, or M1A.

Patients received a 12-day induction course of radiation (1.5 Gy twice a dose to a dose of 30 Gy) concurrent with 4-day continuous intravenous infusions of cisplatin (20 mg/m2 per day) and 5-fluorouracil (1000 mg/m2 per day) beginning on day 1. Surgery followed in 4 to 6 weeks followed 6 to 10 weeks later by a second, identical course of chemoradiotherapy.
RESULTS: From October 1999 through March 2003, 93 patients were enrolled; 96% were white, 86% male, and 83% had adenocarcinoma. Resection was possible in 83 patients (89%) with 4 (5%) perioperative deaths. With a median follow up of 50 months (range, 34-72 months), the 3-year projected overall survival rate is 27.9%, freedom from recurrence 30.5%, and distant metastatic control 32.4%. Locoregional control in resected patients is 86%. Freedom from recurrence and distant control were significantly better in patients with 1) earlier pretreatment clinical stage, 2) earlier postinduction pathologic stage, 3) squamous cell cancer, and 4) a pathologic response.
CONCLUSIONS: This accelerated multimodality treatment program is feasible and perioperative mortality proved acceptable. Despite excellent locoregional control, freedom from recurrence, and overall survival proved disappointing reflecting the frequency of distant metastases. Heterogeneity in patient populations makes comparisons with similar nonrandomized experiences problematic.

PMID: 17414467 [PubMed - indexed for MEDLINE]

119. Gan To Kagaku Ryoho. 2007 Jan;34(1):93-5.

[A case of stage IV advanced gastric cancer responding to TS-1/CDDP neoadjuvant chemotherapy which leads to a pathological complete response].

[Article in Japanese]

Hiraoka K(1), Mizutani S, Oyama T, Uchikoshi F, Yoshidome K, Tori M, Ueshima S, Yamagami Y, Takahashi H, Nakahara M, Tsujimoto M.

Author information:
(1)Dept. of Surgery, Osaka Police Hospital.

A 72-year-old male with advanced gastric cancer (cT3N2M0H0P0CY1, cStage IV) was treated with TS-1/CDDP as neoadjuvant chemotherapy. TS-1 (60 mg/m(2)/day) was orally administered for 3 weeks followed by 2 drug free weeks as a course, and

CDDP (60 mg/m(2)) was administered by intravenous drip on day 8. After the fourth course,a significant tumor reduction was obtained. Total gastrectomy, splenectomy, and D 2 type nodal dissection were performed. The histological diagnosis revealed complete disappearance of cancer cells in the stomach and all of the lymph nodes, which is a so-called pathological complete response. The patient has now been in good health without a recurrence for 24 months after surgery. This case suggests that neoadjuvant chemotherapy with TS-1/CDDP is a potential regimen for advanced gastric cancer.

PMID: 17220679 [PubMed - indexed for MEDLINE]

120. J Egypt Natl Canc Inst. 2005 Dec;17(4):251-9.

Clinico-pathological features and outcome of management of pediatric gastrointestinal lymphoma.

Morsi A(1), Abd El-Ghani Ael-G, El-Shafiey M, Fawzy M, Ismail H, Monir M.

Author information:
(1)The Department of Surgical Oncology, NCI, Cairo University.

PURPOSE: The purpose of this study is to evaluate pediatric GIT lymphomas as regards clinico-pathological features, controversies in surgical treatment, role of chemotherapy and the prognostic features.
PATIENTS AND METHODS: This study included forty three patients with pediatric GIT Non-Hodgkin's lymphoma collected over 7 years at the NCI Cairo University between January 1997 and December 2003. The data of every patient included: Age, sex, presenting symptoms and signs, preoperative investigations, extent of the disease at diagnosis and the type of resection performed, histopathological examination, details of chemotherapy and state at follow up. Overall and disease free survival were calculated and correlated with all parameters.
RESULTS: The study included 30 boys and 13 girls with median age 5.00 years (range: 0.4:17). The lesions were located in the small intestine (n=15), the large intestine (n=14), the ileocecal region (n=10), stomach (n=2), and multifocally (n=2). Burkitt's lymphoma was the commonest histological type (n=24). The majority were stage IIE and IIIE (22 and 17 respectively). Exploration was done in thirty nine patients (complete resections were done in 23 cases, incomplete resections (debulking) were done in 14 cases and in 2 cases

only lymph node biopsies were done). All patients received a sort of systemic chemotherapy. The median follow up duration was 44 months (range 4- 116 months). The only parameters that had significantly affected the overall survival were localized disease, complete resection, earlier stage and response to chemotherapy with p values, (0.005, 0.001, 0.005 and <0.001 respectively). As regards the disease free survival the only significant factor was localized disease (p=0.035).
CONCLUSION: The extent of disease at presentation is the most important prognostic factor in pediatric GIT lymphoma. Surgery still plays an important role such as complete resection in localized disease, management of complicated disease and diagnostic biopsy. There is no value of debulking and surgery is not advised for gastric lesions. Chemotherapy represents a cornerstone in the treatment and offers an excellent chance for long term, disease free survival.

PMID: 17102814 [PubMed - indexed for MEDLINE]

121. J Clin Oncol. 2006 Sep 10;24(26):4347-55.

Prognostic factors in adenocarcinoma of the esophagus or gastroesophageal junction.

Lagarde SM(1), ten Kate FJ, Reitsma JB, Busch OR, van Lanschot JJ.

Author information:
(1)Department of Surgery, Academic Medical Center, Amsterdam, The Netherlands.
S.M.Lagarde@amc.uva.nl

Comment in
    J Clin Oncol. 2007 Mar 1;25(7):907-8; author reply 908-9.
    J Clin Oncol. 2007 Mar 1;25(7):906-7; author reply 908-9.

The incidence of adenocarcinoma of the esophagus is rising rapidly in Western Europe and North America. It is an aggressive disease with early lymphatic and hematogenous dissemination. TNM cancer staging systems predict survival on the basis of the anatomic extent of the tumor. However, the adequacy of the current TNM staging system for adenocarcinoma of the esophagus or gastroesophageal junction (GEJ) is questioned repeatedly. Numerous prognostic factors have been described, but are not included in the TNM system. This review describes clinical

parameters, aspects of operative technique, response to preoperative chemoradiotherapy therapy, complications and established pathologic determinants found in the resection specimen that have a prognostic impact. Furthermore, their potential application in the clinical setting in patients with adenocarcinoma of the esophagus or GEJ is discussed. Future directions to improve staging systems are given.

PMID: 16963732 [PubMed - indexed for MEDLINE]

122. Cancer. 2006 Oct 1;107(7):1475-82.

Surgical pathology stage by American Joint Commission on Cancer criteria predicts patient survival after preoperative chemoradiation for localized gastric carcinoma.

Rohatgi PR(1), Mansfield PF, Crane CH, Wu TT, Sunder PK, Ross WA, Morris JS, Pisters PW, Feig BW, Gunderson LL, Ajani JA.

Author information:
(1)Department of Gastrointestinal Medical Oncology, the University of Texas M. D. Anderson Cancer Center, Houston, Texas, USA.

BACKGROUND: Preoperative chemoradiation for localized gastric cancer can modify baseline stage, as determined by surgical pathology stage. Therefore, the authors hypothesized that surgical pathology stage would be a better prognosticator of overall survival (OS) than baseline stage.
METHODS: Patient populations were combined from 2 prospectively conducted, preoperative chemoradiation trials that used the same therapeutic strategy. Patients must have had localized gastric adenocarcinoma and were staged extensively, including endoscopic ultrasonography and laparoscopy. Patients had to be fit for surgery medically with a technically resectable cancer. All patients provided written informed consent. Patients first received induction chemotherapy for up to 2 months followed by chemoradiation (45 grays) and an attempted surgery. OS was correlated with pretreatment and posttreatment parameters, including surgical pathology stage according to American Joint Commission on Cancer criteria.
RESULTS: Of 74 patients who were registered, 69 patients (93%) had undergone surgery. Nineteen patients (26%) had a pathologic complete response (pathCR), and

55 patients (81%) had a curative (R0) resection. None of the pretreatment parameters correlated with OS; however, longer OS correlated with lower pathologic stage ($P < .0001$), R0 resection ($P < .001$), clinical response noted prior to surgery ($P = .002$), pathCR ($P = .004$), lower pathologic lymph node classification ($P = .006$), and lower pathologic tumor classification ($P = .03$). Pathologic stage and R0 resection were independent prognostic factors for OS (multivariate Cox model; both $P = .05$).
CONCLUSIONS: When preoperative chemoradiation strategy was employed for gastric cancer, the surgical pathology stage, a reflection of cancer's biologic heterogeneity, was a better prognosticator of OS than the baseline clinical stage. Surgical pathology stage, in this setting, may serve as an intermediate endpoint for Phase II/III trials.

(c) 2006 American Cancer Society.

PMID: 16944539 [PubMed - indexed for MEDLINE]

123. Eur J Surg Oncol. 2006 Dec;32(10):1105-9. Epub 2006 Aug 22.

Response to neoadjuvant chemotherapy and effects of tumor regression in gastric cancer.

D'Ugo D(1), Persiani R, Rausei S, Biondi A, Vigorita V, Boccia S, Ricci R.

Author information:
(1)Division of Surgical Oncology, Hi-Tech Center for Education and Research in Biomedical Sciences, Catholic University - Località Tappino, 86100 Campobasso, Italy.

AIMS: Aim of this study is the evaluation of the effects induced by neoadjuvant chemotherapy (NACT) and its impact on survival on a series of locally advanced gastric carcinomas.
METHODS: Downstaging was assessed comparing pre-treatment clinical and laparoscopic staging with post-operative pathologic staging on 30 consecutive patients who completed a 3-year follow-up. Tumor downstaging and the grade of pathologic response were included in a statistical correlation between tumor regression induced by NACT and 3-year survival.
RESULTS: In this series tumor downstaging was obtained in 13 out of 30 patients.

After the completion of 3-year follow-up, overall survival was >37.5months with an overall survival rate of 56.7%; this figure reached up to 70.8% in those cases who benefited from a R0-resection (24/30 patients: R0-resection rate 80.0%).
CONCLUSIONS: In this study the 3-year survival for locally advanced gastric cancer treated by NACT prior to "D2" surgical resection, compares favourably with historical series treated by surgery alone. Patients who obtained T-downstaging and subsequently benefited from a R0-resection had a definitely better chance of cure, according to a complete 3-year follow-up.

PMID: 16930932 [PubMed - indexed for MEDLINE]

124. J Clin Oncol. 2006 Aug 20;24(24):3953-8.

Phase II trial of preoperative chemoradiation in patients with localized gastric adenocarcinoma (RTOG 9904): quality of combined modality therapy and pathologic response.

Ajani JA(1), Winter K, Okawara GS, Donohue JH, Pisters PW, Crane CH, Greskovich JF, Anne PR, Bradley JD, Willett C, Rich TA.

Author information:
(1)Department of Gastrointestinal Medical Oncology, The University of Texas M.D. Anderson Cancer Center, Houston, TX 77030-4009, USA. Jajani@mdanderson.org

PURPOSE: Preoperative therapy for localized gastric cancer has considerable appeal. We hypothesized that, in a cooperative group setting, preoperative chemoradiotherapy would induce a 20% pathologic complete response (pathCR) rate. Combined-modality therapy quality, survival, and safety were secondary end points.
PATIENTS AND METHODS: Patients with localized gastric adenocarcinoma were eligible. A negative laparoscopic evaluation was required. Patients received two cycles of induction fluorouracil, leucovorin, and cisplatin followed by concurrent radiation and chemotherapy (infusional fluorouracil and weekly paclitaxel). Resection was attempted 5 to 6 weeks after chemoradiotherapy was completed. Quality of therapy was assessed with other end points.
RESULTS: Twenty institutions participated. Forty-nine patients were entered and 43 were assessable (12% stage IB; 37% stage II; and 52% stage III). The pathCR and R0 resection rates were 26% and 77%, respectively. At 1 year, more patients

with pathCR (82%) are living than those with less than pathCR (69%). Grade 4 toxicity occurred in 21% of patients. Chemotherapy, radiotherapy, and surgery per protocol (including acceptable variations) occurred in 98%, 44%, and 63% of patients, respectively. A D2 dissection was performed in 50% of patients. Of 18 major radiotherapy variations, 17 were due to the lack of inclusion of the L3-4 vertebral interphase as prespecified.
CONCLUSION: For localized gastric cancer, preoperative chemoradiotherapy strategy achieved a pathCR rate of more than 20% in a cooperative group setting. The quality of surgery improved (50% with D2 dissection) possibly because surgery was part of this trial. With some refinements, this preoperative chemoradiotherapy strategy is poised for a randomized comparison with postoperative adjuvant chemoradiotherapy in patients with gastric cancer.

PMID: 16921048 [PubMed - indexed for MEDLINE]

125. Curr Treat Options Oncol. 2006 Sep;7(5):410-23.

Cancer of the gastroesophageal junction: Current therapy options.

Ilson DH(1).

Author information:
(1)Gastrointestinal Oncology Service, Department of Medicine, Memorial Sloan-Kettering Cancer Center and Weill-Cornell University Medical College, 1275 York Avenue, New York, NY 10021, USA. ilsond@mskcc.org

Active chemotherapy agents in metastatic adenocarcinoma of the esophagus include taxanes (docetaxel or paclitaxel), 5-fluorouracil, irinotecan, platinum drugs (including cisplatin, oxaliplatin, and carboplatin), and anthracyclines. Conventional chemotherapy combines infusional 5-fluorouracil with cisplatin. The addition of a third drug to this backbone results in greater toxicity and only marginal improvements in outcome. Alternative and potentially better-tolerated chemotherapy involves two-drug regimens, combining 5-fluorouracil with a taxane or irinotecan, or combining a platinum drug with irinotecan or a taxane. Although preoperative chemotherapy improves survival compared with surgery alone, the addition of radiation therapy to chemotherapy preoperatively improves rates of curative resection, reduces local tumor recurrence, and achieves a significant rate of pathologic complete response. Combined preoperative chemotherapy and

concurrent radiotherapy is the preferred preoperative strategy for locally advanced adenocarcinoma of the esophagus. Survival is improved with postoperative chemotherapy and radiotherapy if none has been delivered preoperatively.

PMID: 16904058 [PubMed - indexed for MEDLINE]

126. Ann Oncol. 2006 Oct;17(10):1546-52. Epub 2006 Aug 3.

Sequential high dose chemotherapy as initial treatment for aggressive sub-types of non-Hodgkin lymphoma: results of the international randomized phase III trial (MISTRAL).

Betticher DC(1), Martinelli G, Radford JA, Kaufmann M, Dyer MJ, Kaiser U, Aulitzky WE, Beck J, von Rohr A, Kovascovics T, Cogliatti SB, Cina S, Maibach R, Cerny T, Linch DC.

Author information:
(1)SAKK (Swiss Group for Clinical Cancer Research), Bern, Switzerland.
betticherd@hopcantfr.ch

INTRODUCTION: Sequential high dose (SHiDo) chemotherapy with stem cell support has been shown to prolong the event-free survival in patients with diffuse large B-cell lymphoma.
METHODS: To confirm this result in a multicenter trial, we randomized patients with aggressive NHL, to receive either eight cycles of CHOP or SHiDo. The primary endpoint was overall survival.
RESULTS: 129 evaluable patients were randomized to receive either CHOP or SHiDo: median age, 48 years; 62% male; stage III+IV: 73%; age adjusted International Prognostic Index 1/2/3: 21%/52%/27%. Toxicity grades 3+4 were more pronounced in the SHiDo-arm with 13% versus 3% of patients with fever; 34% versus 13% with infections; 13% versus 2% with esophagitis/dysphagia/gastric ulcer. The remission rates were similar in SHiDo and CHOP arms with 34%/37% complete remissions and 31%/31% partial remissions, respectively. After a median observation time of 48 months, there was no difference in overall survival at 3 years, with 46% for SHiDo and 53% for CHOP (P = 0.48).
CONCLUSION: In this multicenter trial, early intensification with SHiDo did not confer any survival benefit in previously untreated patients with aggressive NHL and was associated with a higher incidence of grades 3/4 toxicity.

PMID: 16888080 [PubMed - indexed for MEDLINE]

127. Nucl Med Rev Cent East Eur. 2006;9(1):37-40.

Diagnostic role of 18F-FDG PET in gastric MALT lymphoma.

Ambrosini V(1), Rubello D, Castellucci P, Nanni C, Farsad M, Zinzani P, Alavi A, Tehranipour N, Al-Nahhas A, Fanti S.

Author information:
(1)Department of Nuclear Medicine - PET Unit, Policlinico S. Orsola-Malpighi Hospital, Bologna, Italy.

BACKGROUND: The aim of the study was to evaluate the usefulness of 18F-FDG-PET in patients with gastric lymphoma, in particular those affected by mucosa-associated lymphoid tissue (MALT) type and aggressive gastric non-Hodgkin's lymphoma (NHL).
MATERIAL AND METHODS: The study group consists of 15 patients with a previous diagnosis of gastric NHL referred to our PET centres in Bologna Hospital and Rovigo Hospital, Italy, in the period 2003-2004. In 9/15 patients the subsequent histological evaluation was consistent with a gastric MALT lymphoma, while aggressive gastric NHL was diagnosed in the other 6/15. PET scan was carried out in patients with known active disease in order to stage or re-stage disease prior to treatment or in patients in complete clinical remission to monitor disease during follow up. Patients were considered in complete clinical remission if free from disease for at least 8 months after chemotherapy or surgery.18F-FDG PET was performed following standard procedures.
RESULTS: Overall 18F-FDG-PET was true positive in all cases of gastric MALT and non-MALT aggressive NHL with known active disease, while no pathological 18F-FDG uptake was evident in the subjects who were in complete clinical remission. The degree of 18F-FDG uptake (mean SUVmax values) in MALT lymphoma was much less intense in comparison to aggressive gastric NHL, suggesting a prognostic role of SUV calculation in gastric lymphomas.
CONCLUSION: Our data demonstrate the significant accuracy of 18F-FDG-PET in detecting active disease in gastric lymphoma of both MALT and non-MALT NHL type. A higher SUV value appears to be related to a more aggressive disease.

PMID: 16791802 [PubMed - indexed for MEDLINE]

128. Ann Oncol. 2006 Sep;17(9):1404-11. Epub 2006 Jun 20.

A phase II trial of neoadjuvant cisplatin-fluorouracil followed by postoperative intraperitoneal floxuridine-leucovorin in patients with locally advanced gastric cancer.

Brenner B(1), Shah MA, Karpeh MS, Gonen M, Brennan MF, Coit DG, Klimstra DS, Tang LH, Kelsen DP.

Author information:
(1)Gastrointestinal Oncology Service, Department of Medicine, Memorial Sloan-Kettering Cancer Center, Cornell University of New York, New York 10021, USA.

BACKGROUND: The aim of the study was to evaluate the efficacy and toxicity of neoadjuvant chemotherapy with intravenous (i.v.) cisplatin and fluorouracil (5-FU), surgery and postoperative intraperitoneal (i.p.) floxuridine (FUdR) and leucovorin (LV) in patients with locally advanced gastric cancer.
PATIENTS AND METHODS: Preoperative staging was confirmed by laparoscopy (LAP). Two cycles of i.v. cisplatin (20 mg/m(2)/day, rapid infusion) and 5-FU (1000 mg/m(2), continuous 24-h infusion), given on days 1-5 and 29-34, were followed by a radical gastrectomy and a D2 lymphadenectomy. Patients having R0 resections were to receive three cycles of i.p. FUdR (1000 mg/m(2)) and LV (240 mg/m(2)), given on days 1-3, 15-17 and 29-31. Intraperitoneal chemotherapy was begun 5-10 days from surgery.
RESULTS: Thirty-eight patients were treated. Both preoperative and postoperative chemotherapy were well tolerated. T stage downstaging (pretreatment LAP versus surgical pathological stage) was seen in 23% of patients. The R0 resection rate was 84%. Neither an increase in postoperative morbidity nor operative mortality was noted. With a median follow-up of 43.0 months, 15 patients (39.5%) are still alive (median survival 30.3 months). Good pathologic response, seen in five patients (15%), was associated with better survival (P = 0.053). Peritoneal and hepatic failures were found in 22% and 9% of patients, respectively. Quality of life seemed to be preserved.
CONCLUSIONS: Neoadjuvant cisplatin/5-FU followed by postoperative i.p. FUdR/LV can be safely delivered to patients undergoing radical gastrectomy and D2 lymphadenectomy. The R0 resection and the survival rates are encouraging. An

association between pathologic response and patient outcome was suggested.

PMID: 16788003 [PubMed - indexed for MEDLINE]

129. J Clin Oncol. 2006 Aug 10;24(23):3789-98. Epub 2006 Jun 19.

Genetic variations in radiation and chemotherapy drug action pathways predict clinical outcomes in esophageal cancer.

Wu X(1), Gu J, Wu TT, Swisher SG, Liao Z, Correa AM, Liu J, Etzel CJ, Amos CI, Huang M, Chiang SS, Milas L, Hittelman WN, Ajani JA.

Author information:
(1)Department of Epidemiology, Experimental Therapeutics, GI Medical Oncology, The University of Texas M.D. Anderson Cancer Center, Houston, TX 77030, USA. xwu@mdanderson.org

PURPOSE: Understanding how specific genetic variants modify drug action pathways may provide informative blueprints for individualized chemotherapy.
METHODS: We applied a pathway-based approach to examine the impact of a comprehensive panel of genetic polymorphisms on clinical outcomes in 210 esophageal cancer patients.
RESULTS: In the Cox proportional hazards model, MTHFR Glu429Ala variant genotypes were associated with significantly improved survival (hazard ratio [HR] = 0.56; 95% CI, 0.35 to 0.89) in patients treated with fluorouracil (FU). The 3-year survival rates for patients with the variant genotypes and the wild genotypes were 65.26% and 46.43%, respectively. Joint analysis of five polymorphisms in three FU pathway genes showed a significant trend for reduced recurrence risk and longer recurrence-free survival as the number of adverse alleles decreased (P = .004). For patients receiving platinum drugs, the MDR1 C3435T variant allele was associated with significantly reduced recurrence risk (HR = 0.25; 95% CI, 0.10 to 0.64) and improved survival (HR = 0.44; 95% CI, 0.23 to 0.85). In nucleotide excision repair genes, there was a significant trend for a decreasing risk of death with a decreasing number of high-risk alleles (P for trend = .0008). In base excision repair genes, the variant alleles of XRCC1 Arg399Gln were significantly associated with the absence of pathologic complete response (odds ratio = 2.75; 95% CI, 1.14 to 6.12) and poor survival (HR = 1.92; 95% CI, 1.00 to 3.72).

CONCLUSION: Several biologically plausible associations between individual single nucleotide polymorphisms and clinical outcomes were found. Our data also strongly suggest that combined pathway-based analysis may provide valuable prognostic markers of clinical outcomes.

PMID: 16785472 [PubMed - indexed for MEDLINE]

130. Gastric Cancer. 2006;9(2):136-9.

Histological complete response in advanced gastric cancer after 2 weeks of S-1 administration as neoadjuvant chemotherapy.

Mori S(1), Kishimoto H, Tauchi K, Higuchi K.

Author information:
(1)Department of Surgery, Aizawa Hospital, 2-5-1 Honjou, Matsumoto, Nagano 390-8510, Japan.

Single-agent or combined chemotherapy with the novel oral fluoropyrimidine anticancer drug, S-1 (TS-1), has been reported to be useful for the treatment of advanced gastric cancer. Here, we report a patient with advanced gastric cancer achieving a complete response (CR) after 2 weeks of administration of S-1 as neoadjuvant chemotherapy. A 78-year-old woman with epigastric pain was diagnosed as having advanced gastric cancer. S-1 was administered orally, at a dose of 50 mg twice a day every day for 2 weeks, followed by a 2-week drug-free period. No obvious adverse reactions occurred. Subsequently, the patient underwent distal partial gastrectomy with D2 lymph node dissection. Pathological examination indicated no remnant signet-ring cells in the excised specimen, no lymph node metastasis, and unnatural fibrosis in one of the No. 3 lymph nodes. The neoadjuvant chemotherapy induced a CR according to the Japanese classification of gastric carcinoma.

PMID: 16767370 [PubMed - indexed for MEDLINE]

131. Gastric Cancer. 2006;9(2):114-9.

A feasibility study of sequential paclitaxel and S-1 (PTX/S-1) chemotherapy as

postoperative adjuvant chemotherapy for advanced gastric cancer.

Kobayashi M(1), Tsuburaya A, Nagata N, Miyashita Y, Oba K, Sakamoto J.

Author information:
(1)Department of Tumor Surgery, Kochi Medical School, Kohasu, Japan.

BACKGROUND: The most frequent recurrence pattern of advanced gastric cancer is peritoneal dissemination. We investigated the safety of and compliance with sequential chemotherapy consisting of paclitaxel and S-1, both of which are effective in the treatment of peritoneal dissemination.
METHODS: The patients in the study all had histologically proven gastric cancer, classified according to the TNM and the Japanese criteria for gastric cancer as T3-4, N0-2, P0, H0 M0, and CY0-1. In all patients, standard gastrectomy of more than a D2 dissection was performed. A dose of 80 mg/m2 of paclitaxel was administered for three courses. One course comprised weekly administration for 3 weeks, followed by a 1-week rest, except for the first course (following S-1 administration at 80 mg/m2 body surface area), in which paclitaxel was administered for only 2 weeks, followed by a 1-week rest. S-1 was administered from day 78 for four courses, with one course comprising 2 weeks' administration followed by a 1-week rest. Fifty patients received paclitaxel chemotherapy. The median age was 62.5 years overall; among the 34 male patients it was 65.5 years, and among the female patients it was 48.0 years.
RESULTS: The patient compliance rate was 84%. There were no cases of grade 4 hematological toxicity during either paclitaxel or S-1 treatment. With respect to nonhematological toxicities, there was one case of grade 3 neuropathy during the course of paclitaxel treatment and one case of grade 3 diarrhea during the course of S-1 treatment. These patients recovered and completed the scheduled treatment regimen.
CONCLUSION: Sequential chemotherapy of paclitaxel and S-1 as postoperative adjuvant chemotherapy for advanced gastric cancer is feasible.

PMID: 16767367 [PubMed - indexed for MEDLINE]

132. Clin Oncol (R Coll Radiol). 2006 May;18(4):345-50.

Symptomatic responses to neoadjuvant chemotherapy for carcinoma of the oesophagus and oesophagogastric junction: are they worth measuring?

Forshaw MJ(1), Gossage JA, Chrystal K, Cheong K, Harper PG, Mason RC.

Author information:
(1)Department of General Surgery, Guy's and St Thomas' NHS Trust, St Thomas' Hospital, London, UK.

AIMS: Neoadjuvant chemotherapy is used to downstage locally advanced oesophagogastric cancer. This study assessed whether changes in dysphagia and weight correlated with radiological and pathological assessment of response and surgical decision-making.
MATERIALS AND METHODS: All patients with locally advanced carcinoma of the lower oesophagus or oesophagogastric junction treated with neoadjuvant ECF (epirubicin, cisplatin, and 5-fluorouracil) chemotherapy from January 2000 to January 2003 were included in this study. Patients were considered to be operable depending upon their chemotherapy response. Weight and swallowing were assessed before and after chemotherapy. Statistical analysis was carried out using ANOVA, unpaired t test and Fisher's exact test.
RESULTS: Seventy-eight patients (male-female ratio: 6.8: 1; median age: 62.2 years; range: 44.1-78.0 years) underwent a median of three cycles (range: 1-7) of neoadjuvant ECF chemotherapy. Forty patients (51%) gained weight, and swallowing improved in 53 patients (68%). Radiological changes (based on computed tomography) were assessed according to WHO criteria: complete response (5%), partial response (27%), stable disease (46%) and progressive disease (15%). Patients whose swallowing improved gained significantly more weight ($P < 0.0001$). Swallowing ($P = 0.0009$) was significantly improved in radiological responders but not weight ($P = 0.06$); when radiological non-responders were separated into stable and progressive disease, patients with progressive disease were identified as failing to gain weight ($P = 0.005$). Both swallowing ($P < 0.0001$) and weight gain ($P < 0.0001$) were better in patients undergoing surgery. The use of changes of weight ($P = 0.42$) and swallowing ($P = 0.61$) failed to separate pathological responders from nonresponders in the subset of patients undergoing surgery.
CONCLUSIONS: Weight gain and improved swallowing are good but not absolute indicators of radiological response to chemotherapy and patient selection for surgery. However, changes in these variables are not sufficiently sensitive to identify pathological responders from non-responders.

PMID: 16703754 [PubMed - indexed for MEDLINE]

133. Gan To Kagaku Ryoho. 2006 May;33(5):625-9.

[Thymidylate synthase and dihydropyrimidine dehydrogenase expression and histological effects of preoperative UFT in gastric cancer patients].

[Article in Japanese]

Uyama R(1), Yoshizawa Y, Tajiri T, Endo Y, Mitsuya T, Sanada Y.

Author information:
(1)Dept. of Surgery, Showa University Fujigaoka Hospital.

During DNA synthesis in tumors, fluoropyrimidine anticancer agents target thymidylate synthase (TS) that catalyze the synthesis of dTMP from dUMP and are metabolized by dihydropyrimidine dehydrogenase (DPD). We administered UFT to patients with gastric cancer preoperatively to prevent cancers from advancing while they await surgery or down staging.PATIENTS AND METHODS: We administered UFT to 24 gastric cancer patients at 360 mg/m(2)/day for longer than 3 weeks as a preoperative chemotherapy. TS and DPD expression in the tumor were measured by immunohistochemistry staining before and after (during surgery) chemotherapy and compared with the results of histological assessment.
RESULTS: TS and DPD expression decreased significantly after UFT administration (p<0.05). Histological assessment showed Grade 1 b or 2 in 11 of 24 patients (46%). Eight of 15 patients with high DPD (53.3%) exhibited Grade 1 b or 2.
CONCLUSIONS: Histological assessment revealed the efficacy of UFT, through a DPD-inhibitory fluoropyrimidine (DIF) effect, in patients with high DPD. This suggests that preoperative administration of UFT can be a useful clinical measure.

PMID: 16685161 [PubMed - indexed for MEDLINE]

134. Eur J Surg Oncol. 2006 Aug;32(6):661-5. Epub 2006 Apr 18.

Neoadjuvant treatment of gastric cancer with peritoneal dissemination.

Yonemura Y(1), Bandou E, Sawa T, Yoshimitsu Y, Endou Y, Sasaki T, Sugarbaker PH.

Author information:
(1)Peritoneal Dissemination Program, Shizuoka Cancer Center, 1007 Shimo-nagakubo, Suntou-gun, Shizuoka, 411-8777, Japan. y.yonemura@scchr.jp

AIMS: To report our experience of neoadjuvant intraperitoneal and systemic chemotherapy (NIPS) for patients having a complete resection of the primary gastric cancer and peritoneal carcinomatosis (PC).
PATIENTS AND METHODS: Patients with advanced peritoneal dissemination of primary gastric cancer had the placement of a peritoneal port system. For intraperitoneal chemotherapy, 40 mg of docetaxel and 150 mg of carboplatin were introduced in 1000 ml of saline on a weekly basis. Simultaneously, 100 mg/m2 of methotrexate and 600 mg/m2 of 5-fluorouracil were infused via a peripheral vein. A minimum of two cycles and up to six cycles of NIPS were used prior to cancer resection. At surgery a complete removal of the primary gastric cancer and the peritoneal implants by peritonectomy was attempted.
RESULTS: Sixty-one patients were enrolled in the study. Thirty-nine had positive intraperitoneal cytology which reverted to negative cytology after treatment in 22. Thirty-eight showed a partial response. Thirty patients came to resection and 14 patients could be made disease-free. Median survival time of all patients was 14.4 months. Patients who received a complete resection had a median survival time of 20.4 months. Grade III/IV toxicities were not found after two courses of NIPS, but did develop in seven patients after more than three courses of NIPS.
CONCLUSION: NIPS can downstage large volume peritoneal dissemination of gastric cancer. When combined with gastrectomy including peritonectomy a complete surgical resection was possible in one-quarter of the patients and resulted in a prolonged survival. This combined intraperitoneal and systemic chemotherapy for PC from gastric cancer is worthy of consideration for phase III clinical investigations.

PMID: 16621433 [PubMed - indexed for MEDLINE]

135. World J Gastroenterol. 2006 Apr 7;12(13):2000-5.

Chemotherapy as a component of multimodal therapy for gastric carcinoma.

Kodera Y(1), Fujiwara M, Koike M, Nakao A.

Author information:

(1)Department of Surgery II, Nagoya University Graduate School of Medicine, Nagoya, Aichi 466-8550, Japan. ykodera@med.nagoya-u.ac.jp

Prognosis of locally advanced gastric cancer remains poor, and several multimodality strategies involving surgery, chemotherapy, and radiation have been tested in clinical trials. Phase III trial testing the benefit of postoperative adjuvant chemotherapy over treatment with surgery alone have revealed little impact on survival, with the exception of some small trials in Western nations. A large trial from the United States exploring postoperative chemoradiation was the first major success in this category. Results from Japanese trials suggest that moderate chemotherapy with oral fluoropyrimidines may be effective against less-advanced (T2-stage) cancer, although another confirmative trial is needed to prove this point. Investigators have recently turned to neoadjuvant chemotherapy, and some promising results have been reported from phase II trials using active drug combinations. In 2005, a large phase III trial testing pre- and postoperative chemotherapy has proven its survival benefit for resectable gastric cancer. Since the rate of pathologic complete response is considered to affect treatment results of this strategy, neoadjuvant chemoradiation that further increases the incidence of pathologic complete response could be a breakthrough, and phase III studies testing this strategy may be warranted in the near future.

PMCID: PMC4087675
PMID: 16610047 [PubMed - indexed for MEDLINE]

136. Ann Hematol. 2006 May;85(5):285-90. Epub 2006 Mar 4.

Gastric Burkitt lymphoma is a distinct subtype that has superior outcomes to other types of Burkitt lymphoma/leukemia.

Park YH(1), Kim WS, Kang HJ, Na II, Ryoo BY, Yang SH, Lee SS, Uhm JE, Kim K, Jung CW, Park K, Ko YH.

Author information:
(1)Division of Hematology-Oncology, Department of Medicine, Korea Cancer Center Hospital, Korea Institute of Radiological & Medical Sciences, 215-4, Gongneung-Dong, Nowon-Gu, 139-706, Seoul, South Korea.

Burkitt lymphoma/leukemia (BL) is a highly aggressive non-Hodgkin’s lymphoma

(NHL) often presenting in extranodal sites or as an acute leukemia. Because of the shared molecular and genetic features, the World Health Organization classification of lymphoid diseases recognizes the lymphomatous and leukemic phases of BL as a single entity: a mature B cell neoplasm, subtype Burkitt lymphoma/Burkitt cell leukemia. Because BL frequently appears in the stomach, we investigated the clinical presentation and outcome of gastric BL. We discovered 21 patients with gastric BL through a survey of our NHL registry, and retrospectively analyzed the distinct features of BL, including the St Jude/Murphy staging, other extranodal involvement, morphology and immunophenotype, response to treatment, and clinical outcome. The patients' median age was 48 years (range, 7-75), and the male-to-female ratio was 2.5. Stage 1 was found in five patients, stage 2 in five patients, and stage 4 in 11 patients. The stomach body and antrum were most frequently involved. All 21 patients were treated with systemic intensive chemotherapy, producing a 71% (15/21) rate of complete response (CR) to chemotherapy. Both the 2-year disease-free and overall survival rates were 55%. All ten patients in BL stages 1 and 2 showed outstanding outcomes, and nine of the ten patients exhibited CR and long-term survival. These data show that a high proportion of patients with gastric BL have a localized disease that is limited to stage 1 and 2, and that these localized BLs have outstanding outcomes. These findings suggest that these BLs could represent a distinct subtype that might have a different biology, which needs to be defined.

PMID: 16518604 [PubMed - indexed for MEDLINE]

137. Ann Surg Oncol. 2006 Mar;13(3):340-6. Epub 2006 Jan 30.

Pathologic nodal status predicts disease-free survival after neoadjuvant chemoradiation for gastroesophageal junction carcinoma.

Gaca JG(1), Petersen RP, Peterson BL, Harpole DH Jr, D'Amico TA, Pappas TN, Seigler HF, Wolfe WG, Tyler DS.

Author information:
(1)Department of Surgery, Duke University Medical Center, Box 3118, Durham, North Carolina 27710, USA.

Comment in

Ann Surg Oncol. 2006 Mar;13(3):285-7.

BACKGROUND: The incidence of carcinoma of the gastroesophageal junction (GEJ) is rapidly increasing, and the prognosis remains poor. We examined outcomes in patients who received neoadjuvant chemoradiation for GEJ tumors to identify factors that predict disease-free (DFS) and overall (OS) survival.
METHODS: A retrospective analysis was performed of 101 consecutive patients who received chemoradiation and surgery for GEJ carcinoma between 1992 and 2001.
RESULTS: The median DFS and OS of all patients were 16 and 25 months, respectively. Twenty-eight patients with a complete histological response (T0N0) experienced greater DFS compared with all others (P = .02). Node-negative patients, regardless of T stage, experienced improved median DFS (24 months) compared with N1 patients (9 months; P = .01). Preoperative stage, age, tumor location, or Barrett's esophagus did not independently predict OS by univariate analysis. Multivariate analysis demonstrated that only posttreatment nodal status (P = .03)-not the degree of primary tumor response-predicted DFS.
CONCLUSIONS: The nodal status of patients with GEJ tumors after neoadjuvant therapy is predictive of DFS after resection. The poor outcome in node-positive patients supports postneoadjuvant therapy nodal staging, because surgical aggressiveness should be tempered by the realization that cure is unlikely and median survival is short.

PMID: 16485154 [PubMed - indexed for MEDLINE]

138. Cancer. 2006 Mar 1;106(5):1017-25.

The number of lymph nodes with metastasis predicts survival in patients with esophageal or esophagogastric junction adenocarcinoma who receive preoperative chemoradiation.

Gu Y(1), Swisher SG, Ajani JA, Correa AM, Hofstetter WL, Liao Z, Komaki RR, Rashid A, Hamilton SR, Wu TT.

Author information:
(1)Department of Pathology, The University of Texas M. D. Anderson Cancer Center, Houston, Texas 77030, USA.

BACKGROUND: The survival of patients with locoregional adenocarcinoma of the

esophagus or the esophagogastric junction (EGJ) who receive preoperative chemoradiation is reported to be better among patients who achieve a pathologic complete response than among patients who have residual tumor, including lymph node (LN) metastasis. However, the prognostic significance of the number of LNs with residual metastasis remains unclear.
METHODS: The authors studied 187 consecutive patients who received chemoradiation followed by an esophagectomy. The number of positive LNs and the size of metastatic tumor in each positive LN were examined with regard to overall survival (OS) and recurrence-free survival (RFS).
RESULTS: A pathologic complete response was achieved by 29% of patients. No LN metastasis (posttherapy pathologic negative LN status [ypN0]) was present in 49% of patients who had residual carcinoma, and LN metastasis (ypN1) was present in 51% of patients. The 5-year OS and 2-year RFS rates achieved by patients who had 1 positive LN (34% and 45%, respectively) were similar to the rates achieved by patients in the ypN0 group (38% [P = 0.84] and 50% [P = 0.77], respectively) but were significantly better than the rates achieved by patients who had > or = 2 positive LNs (6% [P = 0.02] and 18% [P = 0.01], respectively). The size of metastatic tumor in LNs among patients who had 1 positive LN was a prognostic factor (> or = 4 mm vs. < 4 mm; P = 0.04). In multivariate analysis, OS was better in patients who had 1 LN metastasis among patients in the ypN1 group (P = 0.02) independent of their posttherapy pathologic tumor status.
CONCLUSIONS: The current results suggested that the number of LNs with metastasis is an independent prognostic factor in patients with residual adenocarcinoma of the esophagus or the EGJ after preoperative chemoradiation. The authors suggest modification of the tumor-lymph node-metastasis (TNM) staging classification (ypTNM) to include the number of positive LNs in the ypN1 category.

PMID: 16456809 [PubMed - indexed for MEDLINE]

139. Cancer Sci. 2006 Feb;97(2):163-6.

Epstein-Barr virus involvement is a predictive factor for the resistance to chemoradiotherapy of gastric diffuse large B-cell lymphoma.

Yoshino T(1), Nakamura S, Matsuno Y, Ochiai A, Yokoi T, Kitadai Y, Suzumiya J, Tobinai K, Kobayashi Y, Oda I, Mera K, Ohtsu A, Ishikura S.

Author information:

(1)Department of Pathology, Okayama University Graduate School of Medicine, Dentistry and Pharmaceutical Science, 2-5-1, Shikata-cho, Okayama 700-8558, Japan. yoshino@md.okayama-u.ac.jp

Primary gastric diffuse large B-cell lymphomas are generally well controlled by non-surgical treatment with combination chemotherapy followed by radiotherapy. We have previously reported that over 90% of patients achieved complete response (CR) with this therapeutic strategy: three cycles of cyclophosphamide, adriamycin, vincristine and prednisone followed by radiotherapy (40.5 Gy). Although the CR rate was very high, some patients still showed resistance to this combination therapy. In order to clarify the factors related to therapy resistance, we examined the relationship between Epstein-Barr virus (EBV), which was examined using an in situ hybridization technique, and the patients' clinical courses. Out of the 50 patients, four were EBV positive; over half of lymphoma cells were positive for EBV by in situ hybridization. Of the three EBV-positive patients, two showed progressive disease and one achieved partial response (PR). Two of the patients died of disease progression. The other patient achieved CR, but the lymphoma recurred with distant metastasis in the cerebellum 3 months after remission. In the present study, eight patients did not achieve CR or they relapsed, four patients showed progressive disease, one patient achieved PR, and three patients achieved CR with recurrence. Therefore, half of these unfavorable patients were EBV positive. This finding strongly indicated that EBV-associated gastric diffuse large B-cell lymphomas frequently show resistance to standard chemoradiotherapy, although some other adverse factors remain unclear.

PMID: 16441428 [PubMed - indexed for MEDLINE]

140. Gan To Kagaku Ryoho. 2005 Dec;32(13):2079-85.

[Neoadjuvant chemotherapy using TS-1 and CDDP against large type 3/Type 4/Bulky N 2 advanced gastric cancer].

[Article in Japanese]

Tanemura H(1), Oshita H, Kanno A, Kusakabe M, Hatoh T, Yamada M, Adachi T, Nishio K, Saito S, Tomita E, Sugiyama A, Yamada T.

Author information:

(1)Dept. of Surgery, Gifu Municipal Hospital.

This study was conducted to assess therapeutic results following neoadjuvant chemotherapy (NAC) for large type 3/type 4/Bulky N 2 advanced gastric cancer having a poor prognosis following resection. The subjects consisted of cases (< or = 75 y.o.) having large type 3 (diameter > or = 8 cm), type 4 or Bulky N 2 gastric cancer curable by resection based on preoperative imaging diagnostics. The NAC regimen consisted of TS-1 at 80-120 mg/body on days 1-21 p. o. and CDDP at 60 mg/m2 on day 8 divided. Upon completion of two courses of 4 weeks per course, gastrectomy with > or = D2 lymph node dissection was carried out on days 21-34. The average age of the subjects was 60.7 years, and the therapy completion rate was 80% (8/10 cases). Five of ten cases were responders diagnosed as grade 2 by histopathological examination of excised specimens (response rate 50%). Two of five responders were histopathologically evaluated as down-staging as a result of NAC (Stage III A--> f Stage I A, Stage IV--> f Stage I A). Three of the five non-responders have relapsed, and the relapse-free interval was an average 238 days. In the five responders,one has relapsed at 331 days,while the other 4 responders have shown no relapse yet. Although NAC consisting of TS-1 and CDDP is considered to be effective against advanced gastric cancer, a phase III study with surgical treatment only will be necessary to confirm its true value.

PMID: 16352932 [PubMed - indexed for MEDLINE]

141. Expert Rev Anticancer Ther. 2005 Aug;5(4):719-25.

Preoperative and adjuvant therapies for upper gastrointestinal cancers.

Varadhachary G(1), Ajani JA.

Author information:
(1)MD Anderson Cancer Center, GI Medical Oncology Department, University of Texas, 1515 Holcombe Boulevard, Mailbox 426, Houston, TX 77030, USA.

Survival of esophageal, gastrointestinal junction and gastric cancers is poor given that they frequently present with locally advanced or metastatic disease. The incidence of gastrointestinal junction adenocarcinoma is increasing whereas that of squamous cell carcinoma of the esophagus is decreasing. The accuracy of staging has improved with newer diagnostic techniques, including positron

emission tomography, endoscopic ultrasound and laparoscopy, and this should be integrated in prospective Phase III clinical trials evaluating neoadjuvant and adjuvant therapies for some esophageal and all gastric carcinomas. For esophageal cancer (except for one trial by Walsh and colleagues), four randomized Phase III trials comparing preoperative chemoradiation followed by surgery versus surgery alone have not shown a survival benefit. Neither have the trials, where preoperative chemoradiation followed by surgery, is compared with definitive chemoradiation. Nevertheless, it is commonly practiced in the USA and has become a preferred combined modality approach. Postoperative chemoradiation is favored in the USA for good performance status patients with resected, high-risk gastric or gastroesophageal junction carcinoma (more than Stage IA). The UK-MAGIC trial results, showing survival benefit with perioperative chemotherapy in operable gastric and lower esophageal cancers, probably has an impact on the treatment practice of these cancers in Europe and Asia. Promising results from trials involving preoperative chemoradiation followed by surgery in gastric cancer (pathologic complete response of 20-30%) need to be further evaluated in a Phase III setting and compared with postoperative chemoradiation. Active ongoing research will help us clarify the role of preoperative and adjuvant therapies in esophageal and gastric cancers. The role of molecular profiling is evolving and will help us differentiate the responders from the nonresponders.

PMID: 16111471 [PubMed - indexed for MEDLINE]

142. J Gastroenterol Hepatol. 2005 Sep;20(9):1470-1.

Long-term complete remission of metastatic gastric cancer after weekly docetaxel, 24 h infusion of high-dose 5-FU/leucovorin and cisplatin.

Chan CH, Liu TW, Chen LT, Chang JY, Whang-Peng J.

PMID: 16105146 [PubMed - indexed for MEDLINE]

143. Asian Pac J Cancer Prev. 2005 Apr-Jun;6(2):195-6.

Gastric carcinoma: 5 year experience of a single institute.

Sadighi S(1), Raafat J, Mohagheghi M, Meemary F.

Author information:
(1)Cancer Institute, Tehran University of Medical Sciences, Iran.

PURPOSE: Gastric cancer (GC) is the most common cause of cancer death registered in cancer institute. Background clinical information is important for cancer prevention and therefore we here present characteristics and outcome of GC patients, more than half coming from northern parts of Iran.
MATERIALS AND METHODS: we retrospectively studied records patients with pathologic diagnosis of GC referred to the Medical Oncology Department of the Cancer Institute from 1998 to 2003.
RESULTS: Four hundred and thirteen patients were registered with GC with the average age of 58 and a male to female ratio of 3/1. Tumor stage based on AJCC was stage 2(12.5%), stage 3(22%), stage 4(63%) and 2% unknown. Most common site of involvement was cardia (43%). Median survival time of all patients (with or without treatment) was 10 months overall. Gastrectomy was performed for 214 patients(39% with positive surgical margins), and 175 of the gastrectomised patients received chemotherapy. Median survival with surgery only was 7 months but 20 months with both surgery and chemotherapy. Only 21 patients received neoadjuvant chemotherapy. Median survival of patients who had response to preoperative chemotherapy was 30 months. By multivariate analysis lower extent of disease (p=0.0024), free surgical margin (p=0.0017), and chemotherapy (p=0.001) were associated with better prognosis.
CONCLUSIONS: Only curative resection with free margins was associated with a survival benefit in this study. More than 80% of patients were diagnosed in locally advanced or metastatic stage of disease and even with neoadjuvant chemotherapy and salvage surgery the outcome was poor. Clearly more efforts need to be given to early detection of lesions to allow a better cure rate.

PMID: 16101332 [PubMed - indexed for MEDLINE]

144. Int J Clin Oncol. 2005 Jun;10(3):204-7.

Amelanotic malignant melanoma of the esophagus: report of a patient with recurrence successfully treated with chemoendocrine therapy.

Suzuki Y(1), Aoyama N, Minamide J, Takata K, Ogata T.

Author information:
(1)Department of Digestive Surgery, Kanagawa Cancer Center, Yokohama, Japan.

We report a case of primary amelanotic malignant melanoma of the esophagus, an extremely rare disease. A 58-year-old man was diagnosed as having middle esophageal cancer with lymph node metastasis, which was classified as esophageal cancer, Stage III:T3N1M0, by International Union Against Cancer (UICC) criteria. Preoperative chemotherapy was performed, but the response assessment was no change (NC). The patient underwent a subtotal esophagectomy via right thoracotomy and laparotomy. Reconstruction was performed by pulling up the stomach via the retrosternal route; the site of anastomosis was the neck. Adjuvant chemotherapy consisted of five courses of dacarbazine (DITC), nimustine (ACNU), vincristine (VCR), and interferon-beta. Eleven months after the surgery, computed tomography (CT) demonstrated recurrence in the upper mediastinum. The patient received chemoendocrine therapy, consisting of the first planned course of DITC, ACNU, and cisplatin (CDDP), given intravenously; and tamoxifen (TAM), given orally. Subsequently with a modified regimen of this therapy he attained a complete response (CR). In general, the prognosis of esophageal malignant melanoma is very poor. Although our patient had a recurrence, he is alive 4 years and 5 months after the surgery and 3 years and 6 months after the recurrence. The chemoendocrine therapy probably contributed to this outcome.

PMID: 15990972 [PubMed - indexed for MEDLINE]

145. Clin Cancer Res. 2005 Apr 15;11(8):3025-31.

Combined GADD45A and thymidine phosphorylase expression levels predict response and survival of neoadjuvant-treated gastric cancer patients.

Napieralski R(1), Ott K, Kremer M, Specht K, Vogelsang H, Becker K, Müller M, Lordick F, Fink U, Rüdiger Siewert J, Höfler H, Keller G.

Author information:
(1)Department of Pathology, Technische Universität München, Munich, Germany.

PURPOSE: We evaluated the expression of seven therapy-related genes to predict the clinical outcome of advanced gastric cancer patients treated with a neoadjuvant chemotherapeutic protocol.

EXPERIMENTAL DESIGN: Pretherapeutic, formalin-fixed, and paraffin-embedded biopsies of 61 patients, who received a 5-fluorouracil (5-FU)- and cisplatin-based chemotherapy were studied. The expressions of the 5-FU-related genes TS, DPD, and TP and of the cisplatin-related genes ERCC1, ERCC4, KU80, and GADD45A were analyzed by quantitative real-time PCR. The expression levels of single genes and of various combinations were tested for an association with response and overall survival.
RESULTS: High DPD levels were more frequently found in nonresponding patients and were associated with worse survival. GADD45A and TP levels showed weak associations with response, but GADD45A expression correlated with survival. There was no association with response for TS expression, but tumors with a high TS level were associated with worse survival. The combination of GADD45A and TP revealed the strongest predictive effect. High expression values of TP and/or GADD45A were exclusively found in nonresponding patients ($P = 0.002$) and were associated with a significantly poorer survival ($P = 0.04$).
CONCLUSIONS: Combined gene expression levels of TP and GADD45A represent a new variable to predict the clinical outcome after neoadjuvant chemotherapy in gastric cancer. The association of DPD expression with response and survival underlines a predominant role of DPD to predict 5-FU sensitivity. The association of TS expression levels with survival but not with response suggests an importance of this gene for tumor progression.

PMID: 15837757 [PubMed - indexed for MEDLINE]

146. Chir Ital. 2005 Jan-Feb;57(1):9-14.

[Preliminary results of neoadjuvant treatment of adenocarcinoma of the gastro-esophageal junction].

[Article in Italian]

Pedrazzani C(1), Pasini F, Giacopuzzi S, Bernini M, Gabbani M, Grandinetti A, Tomezzoli A, Ruzzenente A, Guglielmi A, de Manzoni G.

Author information:
(1)Prima Divisione Clinicizzata di Chirurgia Generale, Università di Verona.

The prognosis of adenocarcinoma of the gastro-oesophageal junction is poor and

only surgery yields long-term survival in no more than 30% of patients. We tested a new neoadjuvant chemo-radiotherapy regimen based on the administration of weekly docetaxel and cisplatin and continuous infusion of 5-FU with concurrent radiotherapy in order to evaluate its feasibility and efficacy. Thirty-three patients enrolled in a dose-finding study and observed at the 1st Division of General Surgery of the University of Verona between January 2000 and October 2003 underwent neoadjuvant chemo-radiotherapy for gastro-oesophageal junction adenocarcinoma (Siewert type I and II). The induction treatment was completed in 97.0% of cases with no treatment-related mortality. After completion of chemo-radiation 30 patients underwent surgery (90.9%) while three patients did not (progression in 2 cases and chemotherapy toxicity in one). Two operated patients did not undergo resection because of liver metastasis at laparotomy (respectability: 84.8%) and 3 more cases had incomplete tumour resection (R0-resectability: 75.8%). No postoperative in-hospital mortality was observed. A complete response (pT0N0) was achieved in 7 cases (23.3%) while minimal residual disease without evidence of lymph node involvement was found in a further 5 cases (16.7%). Worthy of note is the high rate of positive histopathological responses in the later period (6 out of 8) with 4 cases presenting complete responses. This protocol regimen proved to be feasible and well tolerated. Surgery-related deaths and morbidity were not increased. A high rate of positive pathological responses was obtained particularly in the later period of the study with the increased dosage of the protocol regimen.

PMID: 15832733 [PubMed - indexed for MEDLINE]

147. Clin Cancer Res. 2005 Mar 15;11(6):2229-36.

Signet-ring cell or mucinous histology after preoperative chemoradiation and survival in patients with esophageal or esophagogastric junction adenocarcinoma.

Chirieac LR(1), Swisher SG, Correa AM, Ajani JA, Komaki RR, Rashid A, Hamilton SR, Wu TT.

Author information:
(1)Department of Pathology, University of Texas M.D. Anderson Cancer Center, 1515 Holcombe Boulevard, Houston, TX 77030, USA.

PURPOSE: The survival of patients with local-regional adenocarcinoma of the

esophagus or esophagogastric junction (EGJ) treated with preoperative chemoradiation is much better in patients with pathologic complete response than those with residual tumor. Some adenocarcinomas have mixed patterns, including signet-ring cell and mucinous histology, but the clinical significance of these subtypes is unknown.
EXPERIMENTAL DESIGN: We studied 412 consecutive patients with esophageal or EGJ adenocarcinoma treated with chemoradiation followed by esophagectomy (193 patients) or surgery alone (219 patients). We evaluated signet-ring cell and mucinous histology in the resection and pretherapy biopsy specimens and compared clinicopathologic features with overall survival.
RESULTS: The fraction of signet-ring cell and mucinous histology was similar in evaluated specimens of patients treated with preoperative chemoradiation or surgery alone (17% and 18%, respectively). The overall survival rate at 5 years of patients treated with preoperative chemoradiation was significantly better if residual signet-ring cell or mucinous histology was present in the esophagectomy specimen (63% versus 28%; P = 0.02). All 13 patients with acellular mucin pools and no residual carcinoma are still alive after an average follow-up time of 36 months. By contrast, in patients treated with surgery alone, overall survival rate was significantly worse if signet-ring cell or mucinous histology was present (14% versus 30%; P = 0.05). In multivariate analysis, overall survival was independently predicted by presence of signet-ring cell or mucinous histology (P = 0.04).
CONCLUSIONS: Our study showed that patients with esophageal or EGJ adenocarcinoma who have signet-ring cell or mucinous histology benefited substantially from preoperative chemoradiation and esophagectomy.

PMID: 15788671 [PubMed - indexed for MEDLINE]

148. Gastric Cancer. 2005;8(1):50-4.

Neoadjuvant chemotherapy with S-1 and surgical resection for a mucinous gastric cancer with peritoneal dissemination.

Hamada M(1), Tsuji A, Iwata J, Nishioka Y, Ozaki K, Shima Y, Horimi T.

Author information:
(1)Department of Surgery, Kochi Municipal Central Hospital, Kochi 780-0821, Japan.

We herein report the case of a patient with mucinous gastric carcinoma with peritoneal dissemination that disappeared after neoadjuvant chemotherapy with S-1 alone. The patient has survived for over 23 months after surgery, without recurrence. A 60-year old man was referred to our hospital because of an advanced gastric cancer, detected by upper gastrointestinal endoscopy at another hospital. Staging laparoscopy was performed on October 25, 2002, and revealed massive peritoneal dissemination. Two courses of neoadjuvant chemotherapy with S-1 were administered, at 120 mg/day for 28 days, as one course. Total gastrectomy, with D2 lymph node dissection, was performed on January 24, 2003. The peritoneal dissemination had macroscopically disappeared and the cytology of the peritoneal lavage fluid was class III. His final diagnosis was gastric carcinoma, MLU, type 3, T2(SS), P0, H0, M0, N3, CY0, stage IV.

PMID: 15747176 [PubMed - indexed for MEDLINE]

149. Zhonghua Zhong Liu Za Zhi. 2004 Dec;26(12):749-52.

[Short-term effects of chemotherapy with combination of hydroxycamptothecine and oxaliplatin in the treatment of advanced digestive tract cancers].

[Article in Chinese]

Sun YJ(1), Zhao H, Guo YW, Lin F, Cai X, Tang XC, Yao Y.

Author information:
(1)Department of Medical Oncology, Sixth People's Hospital, Shanghai University of Commanications, Shanghai 200233, China.

OBJECTIVE: To evaluate the short-term therapeutic effects and side effects of combined hydroxycamptothecine and oxaliplatin in the treatment of advanced digestive tract cancers.
METHODS: Thirty patients suffering from advanced digestive tract tumors including gastric cancer 8, colorectal cancer 20, cholecystic cancer 1 and malignant fibroadenoma 1 were studied. They were treated with hydroxycamptothecine plus oxaliplatin for 2 cycles with interval of 21 days.
RESULTS: The complete response, partial response, stable disease and progressive disease rates were 3.3% (1/30), 36.7% (11/30), 53.3% (15/30) and 6.7% (3/30)

respectively with an overall response rate (CR + PR) of 40.0% (12/30). In the whole 77 cycles, leukocytopenia was observed in 34 cycles (44.2%) and 19 cycles (55.9%) at grades III and IV. Diarrhea developed in 42 cycles (54.5%) and 20 cycles (47.6%) grades III and IV. The other side effects were fever, alopecia, nausea and vomiting, constipation, hepatic and renal function abnormity and neuritis.
CONCLUSION: Satisfactory response rate is obtainable in advanced colorectal cancer as treated by hydroxycamptothecine plus oxaliplatin. The toxicity consists of severe leukocytopenia and diarrhea.

PMID: 15733397 [PubMed - indexed for MEDLINE]

150. J Surg Oncol. 2005 Mar 15;89(4):227-36; discussion 237-8.

Prognostic indicators in locally advanced gastric cancer (LAGC) treated with preoperative chemotherapy and D2-gastrectomy.

Persiani R(1), D'Ugo D, Rausei S, Sermoneta D, Barone C, Pozzo C, Ricci R, La Torre G, Picciocchi A.

Author information:
(1)Department of Surgery, Catholic University, Rome, Italy.
rpersiani@rm.unicatt.it <rpersiani@rm.unicatt.it>

BACKGROUND AND OBJECTIVES: Neoadjuvant chemotherapy is increasingly considered an effective treatment option for patients with gastric carcinoma. Aim of the study is to evaluate the prognostic significance of the pathological response and of known prognostic factors in a group of accurately staged locally advanced gastric cancer (LAGC) patients.
METHODS: Thirty-three patients with LAGC, staged by laparoscopy, underwent D2-gastrectomy after preoperative chemotherapy. Survival was calculated by Kaplan-Meier method and differences were assessed by the Log-rank and Breslow test. Multivariate analysis was performed using the Cox proportional hazard model in backward stepwise regression.
RESULTS: Curative resection (R0) was achieved in 81.8% of patients. A complete or subtotal pathological response was documented in 3 and 6%, respectively. Nineteen out of thirty-three (57.6%) patients were alive and 16 of them were free of relapse at last follow-up. Survival rates were 81, 67, and 59% at 12, 24, and 36

months, respectively. At univariate and multivariate analysis, only R0 resection was found to be an independent prognostic factor.
CONCLUSIONS: In the current study, R0 resection is the most important prognostic factor for resectable LAGC; according to our results we feel encouraged to consider neoadjuvant chemotherapy a promising modality for increasing the R0-percentage of gastric carcinoma patients who could benefit from a curative surgery.

PMID: 15726615 [PubMed - indexed for MEDLINE]

151. J Clin Oncol. 2005 Feb 20;23(6):1237-44.

Paclitaxel-based chemoradiotherapy in localized gastric carcinoma: degree of pathologic response and not clinical parameters dictated patient outcome.

Ajani JA(1), Mansfield PF, Crane CH, Wu TT, Lunagomez S, Lynch PM, Janjan N, Feig B, Faust J, Yao JC, Nivers R, Morris J, Pisters PW.

Author information:
(1)Department of Gastrointestinal Medical Oncology, Unit 426, The University of Texas M.D. Anderson Cancer Center, 1515 Holcombe Blvd, Houston, TX 77030-4009, USA. jajani@mdanderson.org

PURPOSE: Preoperative chemoradiotherapy may increase the R0 (curative) resection rate, overall survival (OS) duration, and disease-free survival (DFS) duration. We evaluated paclitaxel-based induction chemotherapy and chemoradiotherapy in patients with localized gastric or gastroesophageal adenocarcinoma to determine its feasibility, impact on the R0 resection rate, type of pathologic response, OS, and DFS.
PATIENTS AND METHODS: Patients with operable, localized gastric, or gastroesophageal adenocarcinoma were eligible. Staging included endoscopic ultrasonography (EUS) and laparoscopy. Patients received two 28-day cycles of induction chemotherapy of fluorouracil, paclitaxel, and cisplatin followed by 45 Gy of radiation and concurrent fluorouracil plus paclitaxel. The cancer was restaged and surgery was attempted. Postsurgery pathologic findings and R0 resection were correlated with OS and DFS.
RESULTS: Forty-one patients were enrolled. Most carcinomas were proximal (83%) and pretreatment stage EUST3 (85%). Forty patients (98%) underwent surgery, and

78% had an R0 resection. We observed a pathologic complete response (pathCR) rate of 20% and a pathologic partial response (pathPR) rate of 15% (< 10% residual cancer cells in the resected specimen). No pretreatment parameter (sex, cancer location, baseline T stage, or baseline N stage) predicted the type of postsurgery pathologic response, OS, or DFS. However, pathCR (P = .02), pathCR + pathPR (P = .006), R0 resection (P < .001), and postsurgery T and N stages (P = .01 and P < .001, respectively) were associated with OS. Same parameters were significantly correlated with DFS. Toxicity was manageable.
CONCLUSION: The type of pathologic response but not pretreatment parameters was associated with OS and DFS. Efforts to increase the rate of pathologic response and better systemic cancer control are warranted.

PMID: 15718321 [PubMed - indexed for MEDLINE]

152. Gastroenterol Clin Biol. 2004 Aug-Sep;28(8-9):651-7.

Locally advanced adenocarcinomas of the gastric cardia: results of pre-operative chemoradiotherapy.

Balandraud P(1), Moutardier V, Giovannini M, Giovannini MH, Lelong B, Guiramand J, Magnin V, Houvenaeghel G, Delpero JR.

Author information:
(1)Service de Chirurgie Digestive, Institut Paoli-Calmette, 232 Boulevard Sainte-Marguerite, 13009 Marseille. balandraudp@yahoo.fr

Overall prognosis of adenocarcinomas of the gastro-esophageal junction remains poor as most patients present with advanced disease.AIM: To examine the effects of preoperative chemoradiotherapy in locally advanced adenocarcinomas of the gastro-esophageal junction.
METHODS: Forty-two consecutive patients received a course of radiotherapy (45 Gy, administred in 25 fractions) with concurrent infusion of 5-Fluorouracil and cisplatin, followed by surgery. Endoscopic ultrasonography was used to assess response to chemoradiotherapy. A transhiatal or a transthoracic approach was used for surgical resection. Tumor size, node invasion and margins of resection were analyzed.
RESULTS: Thirty-eight patients underwent subsequent surgery and complete resection (R0) was achieved in 34. Operative mortality was 13.2% (5/38). A

histological complete response was observed in 6 patients. Median survival was 23 months (range: 15-31) and median disease-free survival was 19 months (range: 15-23). At one and two years, 70.7 and 45.6% of the patients were alive, respectively. The pTNM status, node involvement and tumor size were predictors of survival.
CONCLUSIONS: Pre-operative chemoradiotherapy is effective in patients with locally advanced carcinoma of the gastro-esophageal junction, resulting in high resection rates. However it seems to increase operative morbidity and mortality. Certain prognostic factors such as resection margins, need to be examined in further detail.

PMID: 15646531 [PubMed - indexed for MEDLINE]

153. Anticancer Drugs. 2005 Jan;16(1):87-91.

In vitro chemosensitivity to gemcitabine, oxaliplatin and zoledronic acid predicts treatment response in metastatic gastric cancer.

Trojan J(1), Kim SZ, Engels K, Kriener S, Mitrou PS, Chow KU.

Author information:
(1)Division of Gastroenterology, Department of Internal Medicine, Johann Wolfgang Goethe University Medical Center, Frankfurt, Germany. trojan@em.uni-frankfurt.de

Individual response of disseminated cancer to chemotherapy is unpredictable. In vitro chemotherapy-induced apoptosis can be measured and might be a method to evaluate in vivo activity of tested drugs. In this report, tumor cells of a patient with signet cell carcinoma of the stomach and diffuse bone marrow infiltration were cultured and tested for in vitro chemosensitivity. The drugs gemcitabine, oxaliplatin and zoledronic acid were found to induce in vitro tumor cell apoptosis synergistically, and subsequently were used as combination chemotherapy regimen. An initially existing disseminated intravascular coagulopathy quickly resolved and after 6 months of treatment on ongoing complete response was induced, thus confirming the results of in vitro chemosensitivity testing.

PMID: 15613910 [PubMed - indexed for MEDLINE]

154. Ai Zheng. 2004 Dec;23(12):1704-6.

[Biweekly regimen of high dose of leucovorin, 5-fluorouracil, and paclitaxel for patients with advanced gastric cancer].

[Article in Chinese]

Feng JF(1), Lu JW, Sun XF.

Author information:
(1)Department of Internal Medicine, Jiangsu Provincial Tumor Hospital, Nanjing, Jiangsu 210009, P.R. China. fjif@vip.sina.com

BACKGROUND & OBJECTIVE: Clinical study showed that paclitaxel (PTX) may be used to treat gastric cancer. The combination of PTX and 5-fluorouracil (5-FU) is effective, and safe in treating advanced gastric cancer. This study was to investigate efficacy of biweekly regimen of high dose of leucovorin (CF), 5-FU, and PTX on advanced gastric cancer, and its toxicities.
METHODS: Twenty patients with advanced gastric cancer received biweekly regimen of CF/5-FU/PTX (200 mg/m(2) of CF, intravenous infusion for 2 h, day 1; 500 mg/m(2) of 5-FU, intravenous injection, day 1; 1 500 mg/m(2) of 5-FU, intravenous infusion for 46 h, day 1, 2; 90 mg/m(2) of PTX, intravenous infusion for 3 h,day 1). Efficacy, and toxicities were evaluated after 4 cycles.
RESULTS: Total response rate was 65.0% (13/20) with 2 (10.0%) cases of complete remission (CR), and 11 (55.0%) cases of partial remission (PR). No treatment-related death occurred. Stomatitis, hand-foot syndrome, and loss of hair were main toxicities.
CONCLUSION: Biweekly regimen of high dose of CF, 5-FU, and PTX may achieve a high response rate with tolerable toxicities in patients with advanced gastric cancer.

PMID: 15601565 [PubMed - indexed for MEDLINE]

155. Ai Zheng. 2004 Nov;23(11 Suppl):1520-2.

[Palliative surgery combined with oxaliplatin-based chemotherapy in treatment of patients with advanced gastric cancer].

[Article in Chinese]

Chen YB(1), Guan YX, Zhan YQ, Li W, Sun XW, Li YF, Xu DZ.

Author information:
(1)Department of Abdominal Surgery, Cancer Center, Sun Yat-sen University, Guangzhou,Guangdong,510060, P.R.China. c.yingbo@medmail.com.cn

BACKGROUND & OBJECTIVE: Gastric cancer has the highest incidence rate and mortality rate among gastrointestinal malignancies. Twenty percent of the patients with dissectable gastric cancer cannot be cured simply by surgery due to local infiltration and distant metastasis. To evaluate the therapeutic effectiveness and safety of oxaliplatin combined with 5-fluorouracil and leucovorin on the patients with gastric carcinoma after palliative gastric resection, we analyzed all of the cases of gastric adenocarcinoma undergone palliative gastric resection in our Cancer Center in recent years.
METHODS: A total of 41 patients who underwent palliative gastric resection from Jan. 2000 to May 2004 in our Cancer Center were evaluated. Chemotherapy was given 8-18 days post-surgically with oxaliplatin (130 mg/m(2), intravenus infusion) on day 1, leukovorin (200 mg/m(2)) on day 1, 5-FU (500 mg/m(2), infusion) on day 1 followed by (5-FU 2600 mg/m(2), continuous infusion) for 48 h, the cycle was repeated every 4 weeks. Primary evaluation was performed after 3 cycles of chemotherapy. The chemotherapy was terminated in the patients without response to the treatment. In the patients with response to the treatment, the chemotherapy continued until 6-8 cycles for further analyses.
RESULTS: None of the patients died from surgery or chemotherapy. Complete response occurred in 2 cases, partial response occurred in 19 cases, stable disease in 8 cases, and progressive disease in 12 cases. The total response rate was 52.5%. The 1-year, 2-year, and 3-year survival rates were 71%, 43%, and 32%, respectively. The side effects included neuropathy, nausea, vomiting, and myelosuppression. No grade 3 or 4 myelosuppression was observed.
CONCLUSION: Palliative surgery in late stage gastric cancer followed by combination chemotherapy of oxaliplatin, 5-fluorouracil and leucovorin is a safe therapeutic modality with promising short-term effectiveness and mild side effects.

PMID: 15566671 [PubMed - indexed for MEDLINE]

156. Zhonghua Zhong Liu Za Zhi. 2004 Sep;26(9):562-4.

[Taxol based chemotherapy in the treatment of advanced gastric cancer].

[Article in Chinese]

Li Y(1), Wu XQ, Cui HJ, Tan HY.

Author information:
(1)Department of Oncology, China-Japan Friendship Hospital, Beijing 100029, China. liyuantt@yahoo.com.cn

OBJECTIVE: To evaluate the efficacy and toxicity of taxol-based chemotherapy in the treatment of advanced gastric cancer (AGC).
METHODS: Twenty-nine patients with AGC treated with taxol-based protocols: taxol plus 5-fluouracil 17 patients, taxol plus cisplatin 10 patients, taxol plus epirubicin 2 patients.
RESULTS: Twenty-six patients were evaluated for clinical response. There was no complete response, PR 10 (34.5%), SD 12 (41.4%), PD 4 (13.8%). The total response rate was 34.5%. The clinical beneficial response rate was 72.4% (21/29). Median time to progression (mTTP) was 5.8 months and median overall survival time was 9.3 months. The main side effects were: suppression of bone marrow in 26 patients (89.7%), alopecia in 25 (86.2%), myodynia and arthrodynia in 23 (79.3%). There was no death during the treatment.
CONCLUSION: Taxol-based chemotherapy is an effective and well tolerated regimen in the treatment of AUC, which can relieve suffering and improve quality of life of the patients. It can be used as the second-line therapy for relapsed advanced gastric cancer.

PMID: 15555290 [PubMed - indexed for MEDLINE]

157. Gan To Kagaku Ryoho. 2004 Oct;31(11):1723-6.

[Treatment results of peritoneal dissemination from gastric cancer by neoadjuvant intraperitoneal-systemic chemotherapy].

[Article in Japanese]

Yonemura Y(1), Kawamura T, Bando E, Takahashi S, Sawa T, Yoshimitsu Y, Obata T, Endo Y, Sasaki T, Sugarbaker PH.

Author information:
(1)Peritoneal Dissemination Program, Shizuoka Cancer Center.

No standard treatment exists for peritoneal dissemination from gastric cancer. We reviewed our experience using a novel treatment consisting of peritonectomy and intraoperative chemo-hyperthermic peritoneal perfusion (CHPP). Records of all patients who underwent CHPP and cytoreductive surgery from 1992 to 2001 were reviewed.RESULTS: Data from 107 patients (average age, 52 years) were available. P3 dissemination was found in 72 patients, and 8 and 27 patients showed P1 or P2 dissemination, respectively. Peritoneal metastasis was synchronous in 75 and metachronous in 32 patients. All patients received CHPP after cytoreductive surgery. Peritonectomy was performed in 42 patients. Complete cytoreduction (CC-0) was achieved in 47 patients (44%). Peritonectomy, resulted in CC-0 in 69% (29/42), but CC-0 was achieved in 18 of 65 (28%) patients by ordinary surgical techniques. There were 23 postoperative complications (21%) after operation. The overall operative mortality was 2.8% (3/107). Median follow-up for the entire study group was 46 months. Seventeen patients (15%) were disease-free, and 90 patients were dead at the time of analysis. Eighty-seven deaths were related to progression of disease. The median survival of all patients was 16.2 months, with an actual 5-year survival of 6%. Median survival of CHPP plus ordinary cyoreduction was 12.0 months and that after CHPP and peritonectomy was 22.8 months. Completeness of cytoreduction and peritonectomy were significant prognostic factors on univariate analysis and 5-year survival rate was 27%. Lymph node status, grade of peritoneal dissemination (P1-2 vs P3), age (>60 years vs <60 years), tumor volume of dissemination (>2.5 cm vs <2.5 cm in diameter), and histologic type (differentiated vs. poorly differentiated type) did not affect survival. The cox proportional model demonstrated that completeness of cytoreduction was the strongest prognostic factor. Patients who had an incomplete resection had 2.8-fold higher risk of dying from disease than patients who underwent complete cytoreduction. The 5-year survival after complete cytoreduction was 12%, compared with 2% for incomplete resection. Four patients lived more than 5 years. Cytoreduction was incomplete in one 5-year survivor who showed complete response to CHPP.
CONCLUSION: Complete cytoreduction using peritonectomy and CHPP may improve survival of patients with peritoneal dissemination from gastric cancer. This procedure is most appropriate for highly motivated patients who are committed to

survive as long as possible.

PMID: 15553695 [PubMed - indexed for MEDLINE]

158. Oncology. 2004;67(1):48-53.

Long-term follow-up of a pilot phase II study with neoadjuvant epidoxorubicin, etoposide and cisplatin in gastric cancer.

Barone C(1), Cassano A, Pozzo C, D'Ugo D, Schinzari G, Persiani R, Basso M, Brunetti IM, Longo R, Picciocchi A.

Author information:
(1)Unit of Medical Oncology, Department of Internal Medicine, Università Cattolica del S. Cuore, Roma, Italy. carlobarone@rm.unicatt.it

OBJECTIVE: The prognosis in T3-T4 or N+ gastric cancer is dismal, and the role of adjuvant therapy remains uncertain. Neoadjuvant chemotherapy could improve both resectability and survival. Here, we report the results of the long-term follow-up of a pilot study aimed at evaluating a neoadjuvant treatment in a group of patients carefully staged by computed tomography (CT), endoscopic ultrasound and laparoscopy.
METHODS: Twenty-five stage II-III patients with histologically proven gastric adenocarcinoma were enrolled in the study. All patients gave informed consent and were thoroughly staged. Patients were treated with epidoxorubicin (40 mg/m2 i.v.) on days 1 and 4, etoposide (VP-16; 100 mg/m2) on days 1, 3 and 4 and cisplatinum (80 mg/m2) on day 2, every 21-28 days for 3 pre-operative cycles before CT clinical restaging followed by laparotomy and D2 gastrectomy. Three further cycles of chemotherapy were planned after radical surgery.
RESULTS: Twenty-four patients received the planned pre-operative chemotherapy and underwent surgical resection; total (13 patients) or subtotal (7 patients) R0 D2 gastrectomy was possible in 20 patients. One patient died as a result of gastric bleeding. Perioperative complications occurred in 5 patients (failure of anastomosis in 1 patient and wound infection in the other 4). The pathologic response rate included 7 partial responses (29.1%) and 10 patients with stable disease (41.7%). The main toxicity was grade 3/4 neutropenia (68%), which occurred more frequently during the postoperative chemotherapy, and fatigue (68%). Fever or infection, however, were never observed. The median disease-free

survival was 37 months, and median survival has not been reached after 40 months of median follow-up. One-, 2- and 3-year survival rates were 80, 64 and 60%, respectively.
CONCLUSION: The notable long-term survival in the present study suggests a comparison between the neoadjuvant approach, including new drug combinations, and adjuvant chemo- or chemoradio-therapy in locally advanced gastric cancer.

Copyright 2004 S. Karger AG, Basel

PMID: 15459495 [PubMed - indexed for MEDLINE]

159. Int J Radiat Oncol Biol Phys. 2004 Oct 1;60(2):427-36.

Induction chemotherapy improved outcomes of patients with resectable esophageal cancer who received chemoradiotherapy followed by surgery.

Jin J(1), Liao Z, Zhang Z, Ajani J, Swisher S, Chang JY, Jeter M, Guerrero T, Stevens CW, Vaporciyan A, Putnam J Jr, Walsh G, Smythe R, Roth J, Yao J, Allen P, Cox JD, Komaki R.

Author information:
(1)Department of Radiation Oncology, Cancer Hospital, Peking Union Medical College and Chinese Academy of Medical Sciences, Beijing, People's Republic of China.

PURPOSE: To investigate the effect of induction chemotherapy (CHT) before trimodality therapy on the outcome of patients with resectable cancer of the esophagus.
METHODS AND MATERIALS: This retrospective study included 81 consecutive patients with resectable cancer of the esophagus who received neoadjuvant chemoradiotherapy followed by esophagectomy between January 1990 and December 1998 (inclusive). Thirty-nine patients underwent chemoradiotherapy followed by esophagectomy (CHT/RT+S), 42 received additional induction CHT followed by CHT/RT+S (CHT+CHT/RT+S). Of the 81 patients, 47 were entered in institutional or national prospective trials (6 in the CHT/RT+S and 41 in the CHT+CHT/RT+S group). Induction CHT consisted of three courses of 5-fluorouracil (5-FU), cisplatin, and paclitaxel given in 28-day cycles in 37 patients (88.1%). Concurrent CHT was 5-FU and platinum based. The median radiation dose for patients treated with CHT/RT+S

was 30 Gy (range, 30-50.4 Gy) delivered in a median of 10 fractions (range, 10-28 fractions) and 45 Gy (range, 30-45 Gy) in a median of 25 fractions (range, 10-25 fractions) for patients treated with CHT+CHT/RT+S. Esophagectomy was performed 6-8 weeks after completion of concurrent chemoradiotherapy. Most patients underwent transthoracic esophagectomy (n = 66, 82.5%).
RESULTS: The pretreatment characteristics were well balanced between the two groups except for age. The median follow-up time was 29 months (22 months for the CHT/RT+S group and 38.5 months for the CHT+CHT/RT+S group) for all patients and 49 months for living patients. The actuarial overall survival (OS), disease-free survival (DFS), locoregional control (LRC), and distant metastasis-free survival (DMFS) rate at 5 years for the entire group was 46%, 36.6%, 70.7%, and 53.2%, respectively. Statistically significant differences in the OS, DFS, and LRC rates between the two groups were detected. Specifically, the 5-year OS rate was 22.8% and 71.1% in the CHT/RT+S and CHT+CHT/RT+S group ($p = 0.0001$), respectively. The 5-year DFS rate was 27.6% and 56.6% in the CHT/RT+S and CHT+CHT/RT+S group ($p = 0.003$), respectively. The 5-year LRC rate was 64.2% and 85.6% in the CHT/RT+S and CHT+CHT/RT+S group ($p = 0.007$), respectively. The difference in the DMFS rate between the two groups was statistically significant, with a 2- and 5-year actuarial rate of 63.9% and 51.9%, respectively, in the CHT/RT+S group and 76.9% and 74.1%, respectively, in the CHT+CHT/RT+S group ($p = 0.04$). The statistically significant differences persisted when patients who received >/=45 Gy in each group were compared. Among those patients, the 5-year OS, DFS, LRC, and DMFS rates were 23.1%, 15.4%, 58.6%, and 39.2%, respectively, for those receiving CHT/RT+S, and 71.4% ($p = 0.001$), 55.8% ($p = 0.0008$), 84.6% ($p = 0.005$), and 77.3% ($p = 0.009$), respectively, for those receiving CHT+CHT/RT+S. The pathologic complete response (pCR) rate was greater in the CHT+CHT/RT+S group compared with in the CHT/RT+S group ($p = 0.008$). In univariate analysis, young age, good Karnofsky performance status, Stage II disease, total radiation dose, multiple drug regimen for concurrent CHT, pCR, R0 resection, distant disease progression, and CHT+CHT/RT+S treatment proved to be prognostic factors for OS. Lower esophageal/gastroesophageal junction tumor location, pCR, R0 resection, and CHT+CHT/RT+S treatment were favorable prognostic factors for LRC. Neither the total radiation dose nor multiple drugs for concurrent CHT were negative prognostic factors for LRC. In multivariate analysis, pCR, R0 resection, and treatment with CHT+CHT/RT+S were independent positive predictive factors for OS, and distant recurrences were negative predictive factors for OS. R0 resection, CHT+CHT/RT+S treatment, and lower esophageal/gastroesophageal junction tumor location were positive predictive factors for LRC. The radiation dose was not identified as an independent prognostic factor for either OS or LRC in the

multivariate analysis. Meaningful multivariate analysis could not be performed when the multiple drug vperformed when the multiple drug variable was included in the model because of the small number of patients.
CONCLUSION: Significantly greater LRC, DFS, OS, and DMFS were found in patients treated with CHT+CHT/RT+S compared with those treated with CHT/RT+S. The pCR rate was significantly higher in the CHT+CHT/RT+S group. Induction CHT was an independent favorable prognostic factor for both LRC and OS for the population included in this study. Our data suggest that a randomized trial comparing CHT+CHT/RT+S and CHT/RT+S is warranted to assess further the merits of this treatment in patients with this currently very lethal cancer.

PMID: 15380576 [PubMed - indexed for MEDLINE]

160. Hepatogastroenterology. 2004 Sep-Oct;51(59):1567-70.

Long-term survivor of gastric small cell carcinoma.

Hosokawa A(1), Shimada Y, Shirao K, Matsumura Y, Yamada Y, Muro K, Hamaguchi T, Gotoh M, Shimoda T.

Author information:
(1)Medical Oncology Division, National Cancer Center Hospital, Tokyo, Japan.

We describe the long-term survival of a patient following the diagnosis of primary gastric small cell carcinoma. In January 2000, a 73-year-old male was found to have advanced gastric small cell carcinoma directly invading his liver. He received combination chemotherapy with cisplatin and irinotecan as first-line chemotherapy, then cisplatin and etoposide as second-line chemotherapy. He had a complete response after four cycles of second-line chemotherapy. In March 2001, the tumor recurred in the stomach and the patient underwent a total gastrectomy. He has survived free of disease for more than 2 years after the first diagnosis.

PMID: 15362803 [PubMed - indexed for MEDLINE]

161. World J Gastroenterol. 2004 Oct 1;10(19):2911-5.

Clinical and experimental study of oxaliplatin in treating human gastric

carcinoma.

Lin WL(1), Li DG, Chen Q, Lu HM.

Author information:
(1)Department of Gastroenterology, Affiliated Xinhua Hospital, Shanghai Second Medical University, Shanghai 200092, China. linwanlong155@sohu.com

AIM: To evaluate the therapeutic effectiveness of oxaliplatin on human gastric carcinoma and to explore its mechanisms.
METHODS: Twenty-two cases of stage IV gastric carcinoma received 4-6 (mean 4.6) cycles of first line combined chemotherapy with oxaliplatin (oxaliplatin 85 mg/m(2), iv, gtt, 1 h, d 1; leukovorin 200 mg/m(2), iv, gtt, 1 h, d 1 and d 2; 5-FU 300 mg/m(2),iv, d 1 and d 2, 5-FU, continuous iv, gtt, 48 h; 1 cycle/2 wk). Response rate, progression-free survival (PFS), total survival time, toxic side effects were evaluated. The inhibitory effect of oxaliplatin on human gastric cell line SGC-7901 was detected and IC(50) was calculated by MTT. Transmission electron microscopy, flow cytometry and TUNEL were performed to evaluate the apoptosis of cell line induced by the drug. The expression of Caspase-3 m-RNA was detected by RT-PCR. AC-DEVD-CHO, a Caspase-3 specific inhibitor, was used to elucidate the role of activated Caspase-3 in the process of apoptosis induced by oxaliplatin.
RESULTS: Total response (complete and partial) occurred in 9 (40.9%) patients. Mean PFS was 4.2 mo and mean total survival time was 7.2 mo. Cumulative neurotoxicity (all grade I-II), vomiting and diarrhea, myelosuppression appeared in 93.5%, 20%, 32.9% patients, respectively. IC(50) was calculated to be 0.71 mg/L by MTT assay. A maximal inhibitory rate reached 85.3%. Apoptosis index was elevated after incubated with 1 mmol/L oxaliplatin for 30 min, but without statistic significance (P>0.05). However it could be detected at a much higher degree both by flowcytometry and by TUNEL with a statistical significance (68.47+/-7.92% and 8.23+/-2.67%, respectively, P<0.05) after incubated with 1 mmol/L oxaliplatin for 2 d. By means of RT-PCR, we detected an enhancement of Caspase-3 m-RNA expression induced by oxaliplatin which was also in positive correlation with the apoptotic level. AC-DEVD-CHO, a Caspase-3 specific inhibitor, could significantly inhibit and delay apoptosis induced by oxaliplatin.
CONCLUSION: Oxaliplatin is effective and well-tolerated in patients with advanced gastric carcinoma. Oxaliplatin could significantly inhibit the growth of human gastric cell line SGC-7901. The induction of Caspase-3 m-RNA expression,

activation of Caspase-3 and promotion of apoptosis may be some of the therapeutic mechanisms of oxaliplatin on gastric carcinoma. Annexin-V-fluorescein labeling flow cytometry is much more sensitive than TUNEL in detecting early stage apoptosis.

PMID: 15334700 [PubMed - indexed for MEDLINE]

162. Eur J Surg Oncol. 2004 Aug;30(6):643-9.

Neo-adjuvant chemotherapy for operable gastric cancer: long term results of the Dutch randomised FAMTX trial.

Hartgrink HH(1), van de Velde CJ, Putter H, Songun I, Tesselaar ME, Kranenbarg EK, de Vries JE, Wils JA, van der Bijl J, van Krieken JH; Cooperating Investigators of The Dutch Gastric Cancer Group.

Author information:
(1)Department of Surgery, Leiden University Medical Centre, P.O. Box 9600, 2300 RC Leiden, The Netherlands. h.h.hartgrink@lumc.nl

AIMS: Gastric cancer in Western countries is often diagnosed in an advanced stage and prognosis is poor. We performed a randomised trial with pre-operative FAMTX vs. surgery alone in order to evaluate the effect of pre-operative chemotherapy on resectability and survival.
METHODS: Patients with proven adenocarcinoma of the stomach were randomised to receive four courses of chemotherapy using 5-Fluorouracil, doxorubicin and methotrexate (FAMTX) prior to surgery or to undergo surgery alone.
RESULTS: Fifty-nine patients were randomised; 29 patients were allocated to the FAMTX regimen prior to surgery and 30 patients had surgery alone. Resectability rates were equal for both groups. Complete or partial response was registered in 32% of the FAMTX group. With a median follow-up of 83 months the median survival since randomisation is 18 months in the FAMTX group vs. 30 months in the surgery alone group (p=0.17).
CONCLUSIONS: This trial could not show a beneficial effect of pre-operative FAMTX. Until large randomised studies prove otherwise, adequate surgery without delay is the best treatment for operable gastric cancer.

PMID: 15256239 [PubMed - indexed for MEDLINE]

163. J Clin Oncol. 2004 Jul 15;22(14):2774-80.

Multi-institutional trial of preoperative chemoradiotherapy in patients with potentially resectable gastric carcinoma.

Ajani JA(1), Mansfield PF, Janjan N, Morris J, Pisters PW, Lynch PM, Feig B, Myerson R, Nivers R, Cohen DS, Gunderson LL.

Author information:
(1)Department of Gastrointestinal Medical Oncology, Stop 426, The University of Texas M.D. Anderson Cancer Center, 1515 Holcombe Blvd, Houston, TX 77030, USA. jajani@mdanderson.org

Erratum in
J Clin Oncol. 2004 Nov 1;22(21):4435.
J Clin Oncol. 2005 Jan 20;23(3):656.

Comment in
J Clin Oncol. 2005 Jun 1;23(16):3870; author reply 3870-1.

PURPOSE: In the West, curative (R0) resection is achieved in approximately 50% of patients with localized gastric carcinoma, and more than 60% die of cancer following an R0 resection. A multi-institutional study of preoperative chemoradiotherapy was done to assess the R0 resection rate, pathologic complete response (pathCR) rate, safety, and survival in patients with resectable gastric carcinoma.
PATIENTS AND METHODS: Operable patients with localized gastric adenocarcinoma were eligible. Staging also included a laparoscopy and endoscopic ultrasonography (EUS). Patients received up to two 28-day cycles of induction chemotherapy of fluorouracil, leucovorin, and cisplatin, followed by 45 Gy of radiation plus concurrent fluorouracil. Patients were then staged and surgery was attempted.
RESULTS: Thirty-four patients were registered at three institutions. One ineligible patient was excluded. Most patients had a promixal cancer and EUST3N1 designation. Twenty-eight (85%) of 33 patients underwent surgery. The R0 resection rate was 70% and pathCR rate was 30%. A pathologic partial response (< 10% residual carcinoma in the primary) occurred in eight patients (24%). EUS T plus N and postsurgery T plus N correlation showed significant downstaging (P =

<.01). The median survival time for 33 patients was 33.7 months. Patients achieving a pathCR or pathPR had a significantly longer median survival time (63.9 months) than those achieving less than pathPR (12.6 months; P =.03). There were two treatment-related deaths.
CONCLUSION: Our data suggest that the three-step strategy of preoperative induction chemotherapy followed by chemoradiotherapy resulted in substantial pathologic response that resulted in durable survival time. This strategy is worthy of a direct comparison with postoperative adjuvant chemoradiotherapy.

PMID: 15254045 [PubMed - indexed for MEDLINE]

164. Jpn J Clin Oncol. 2004 May;34(5):255-61.

A preliminary study of preoperative chemotherapy combining irinotecan and cisplatin in patients with gastric cancer with unresectable para-aortic lymph node metastases.

Yamao T(1), Ohta K, Ohyama S, Ishihara S, Chin K, Maruyama M, Takahashi T, Nakajima T.

Author information:
(1)Department of Internal Medicine, Cancer Institute Hospital, Tokyo, Japan. tyamao-gi@umin.ac.jp

BACKGROUND: A high response rate has been reported for chemotherapy combining irinotecan (CPT-11) and cisplatin (CDDP) against advanced gastric cancer. The strong anti-tumor activity of this regimen makes it very attractive as a preoperative chemotherapy. We conducted a preliminary study on preoperative chemotherapy with this regimen in patients with unresectable gastric cancer with para-aortic lymph node metastases to evaluate the feasibility of it as a treatment strategy.
METHODS: Patients with unresectable para-aortic lymph node metastasis without distant hematogenous metastasis (H0, M0 and M1 LYM) and peritoneal dissemination (P0) were eligible for entry. The preoperative chemotherapy consisted of at least three cycles of CPT-11 (70 mg/m(2)) on days 1 and 15 and CDDP (80 mg/m(2)) on day 15, repeated every 4-6 weeks. Chemotherapy was followed by surgery with extended lymph node dissection in patients who achieved complete or partial responses and whose cancers were judged to be resectable.

RESULTS: Six patients were entered into the study. In total, 18 cycles of chemotherapy were performed and five patients received at least three cycles. Objective partial responses were achieved in four patients. The major toxicities in the chemotherapy were neutropenia and diarrhea, but these were clinically acceptable. Four patients underwent surgery after the chemotherapy, and macroscopically complete resections with extended lymph node dissection were achieved in two patients. There were no therapy-related deaths. We found no pathological complete responses, but observed a definite histopathological effect caused by the chemotherapy in surgical specimens. The median survival time of all patients was 12 months. The longest survival without relapse is >6 years from the start of therapy.
CONCLUSIONS: We conclude that preoperative chemotherapy with CPT-11/CDDP therapy is feasible in patients with advanced gastric cancer and that the regimen is safe when followed by surgery. Further clinical studies with larger numbers of patients are warranted to evaluate the efficacy of this strategy.

PMID: 15231860 [PubMed - indexed for MEDLINE]

165. Dis Esophagus. 2004;17(1):38-43.

Significance of post-chemoradiation biopsy in predicting residual esophageal carcinoma in the surgical specimen.

Yang Q(1), Cleary KR, Yao JC, Swisher SG, Roth JA, Lynch PM, Komaki R, Ajani JA, Rashid A, Hamilton SR, Wu TT.

Author information:
(1)Department of Pathology, The University of Texas M.D., Anderson Cancer Center, Houston, Texas, USA.

Pathologic complete response in the resected esophagus can be achieved in approximately 30% of patients with locally advanced esophageal or gastroesophageal junction carcinoma after preoperative chemoradiation therapy. These patients tend to have a longer survival than those who have less than pathologic complete response. Post-chemoradiation esophageal biopsy (PCEB) is used to check for the presence of residual tumor before a definitive resection is performed, but the clinical significance of PCEB findings is not clear due to the possibility of sampling bias and the superficial nature of the specimen obtained.

We evaluated the use of PCEB (defined as biopsy taken within 30 days before esophagectomy) in predicting residual cancer in post-treatment esophagectomy specimens. PCEB was performed in 65 of 183 (36%) patients with locally advanced esophageal or gastroesophageal junction carcinoma, who received preoperative chemoradiation therapy. The cancer status in PCEB was correlated with the residual cancer in the esophagectomy specimens. PCEB had no cancer in 80% (52 of 65) of patients (Bx-negative) and cancer in 20% (13 of 65) of patients (Bx-positive). There was no difference in the presence of residual cancer (either in esophagus or lymph node) in esophagectomy specimens between Bx-negative patients (77%, 40 of 52) or Bx-positive patients (92%, 12 of 13), P = 0.44. The positive predictive value of biopsy was 92% (12 of 13), negative predictive value 23% (12 of 52), sensitivity 23% (12 of 52) and specificity 92% (12 of 13). There was no difference in the residual cancer staging in the esophagectomy specimen between Bx-positive and Bx-negative patients. In contrast, residual metastatic carcinoma in lymph nodes was more frequent in Bx-positive patients (69.2%, 9 of 13) than in Bx-negative patients (28.8%, 15 of 52), P = 0.01. Our data suggest that PCEB is a specific but not a sensitive predictor of residual cancer following esophagectomy. Bx-positive patients tend to have more frequent residual tumor in lymph nodes. The utility of PCEB in predicting residual cancer in the lymph nodes needs to be explored further along with molecular predictors of response to preoperative therapy.

Copyright 2004 ISDE

PMID: 15209739 [PubMed - indexed for MEDLINE]

166. Cancer. 2004 Jun 1;100(11):2347-54.

Preoperative induction of CPT-11 and cisplatin chemotherapy followed by chemoradiotherapy in patients with locoregional carcinoma of the esophagus or gastroesophageal junction.

Ajani JA(1), Walsh G, Komaki R, Morris J, Swisher SG, Putnam JB Jr, Lynch PM, Wu TT, Smythe R, Vaporciyan A, Faust J, Cohen DS, Nivers R, Roth JA.

Author information:
(1)Department of Gastrointestinal Medical Oncology, The University of Texas M. D. Anderson Cancer Center, Houston, Texas 77030-4009, USA. jajani@mdanderson.org

BACKGROUND: Patients with localized esophageal carcinoma often develop locoregional and distant disease recurrence. The current study investigated the outcome of a new chemotherapy combination as induction therapy before chemoradiotherapy.
METHODS: Forty-three patients with resectable carcinoma of the esophagus or gastroesophageal junction were enrolled. Most of the tumors were endoscopic ultrasonography (EUS) (EUS)T3 (84%) and (EUS)N1 (63%). The patients received < or = 2 6-week cycles of CPT-11 and cisplatin followed by chemoradiotherapy (45 grays with 5-fluorouracil and paclitaxel). Five to six weeks after chemoradiotherapy, the patients underwent staging and surgery. The feasibility, curative resection rates, overall and disease-free survival rates, rate of significant pathologic response, and patterns of disease recurrence were assessed.
RESULTS: Of the 43 patients, 39 (91%) underwent an R0 resection. Two patients (5%) died after surgery. A pathologic complete response (pathCR) was observed in 11 (28%) of the 39 patients (or 26% of the 43 patients). In addition, 16 patients (41% of 39 patients or 37% of 43 patients) had < 10% viable tumor in the surgical specimen (pathPR). A comparison of endoscopic ultrasonograpy T and N classifications with surgical T and N classifications demonstrated significant down-staging (P < 0.01). The median survival period of all 43 patients was 22.1 months. Patients who had achieved a pathCR or pathPR had a longer median survival (25.6 months) than those who achieved less than a pathPR (18.5 months; P = 0.52). None of the clinical parameters examined were found to correlate with survival or pathologic response.
CONCLUSIONS: CPT-11-based induction chemotherapy resulted in substantial pathCR and pathPR rates, both of which lead to a favorable survival outcome. The three-step strategy needs to be developed further, with the investigation of targeted therapies with chemotherapy and radiotherapy.

Copyright 2004 American Cancer Society.

PMID: 15160337 [PubMed - indexed for MEDLINE]

167. Gan To Kagaku Ryoho. 2004 Apr;31(4):579-83.

[A case report of advanced cardiac cancer showing a complete response to TS-1 as neoadjuvant chemotherapy].

[Article in Japanese]

Yamamoto Y(1), Kino Y, Ohara K, Onuma S, Asai K, Kobayashi T, Kono T, Kasai S.

Author information:
(1)Dept. of Surgery, Kobayashi Hospital.

A 69-year-old female had complaints of vomiting, appetite loss and feeling of pharyngeal obstruction. She was diagnosed with a 3'-shaped advanced cardiac cancer with esophageal invasion. A biopsy revealed poorly differentiated adenocarcinoma. The tumor was T3 (SE) N2, Stage IIIB indicating a poor prognosis. After informed consent, TS-1 was administrated as preoperative chemotherapy. Chemotherapy with TS-1 was very effective, and the tumor noticeably decreased. Next, total gastrectomy was performed. Histopathological findings revealed that the primary tumor and lymph node had become scarred and fibrous, indicating a complete response (Grade 3). In the future, TS-1 can be expected to display efficacy in neoadjuvant chemotherapy for patients with advanced gastric cancer who have poor prognoses.

PMID: 15114703 [PubMed - indexed for MEDLINE]

168. Br J Cancer. 2004 Apr 19;90(8):1521-5.

High curative resection rate with weekly cisplatin, 5-fluorouracil, epidoxorubicin, 6S-leucovorin, glutathione, and filgastrim in patients with locally advanced, unresectable gastric cancer: a report from the Italian Group for the Study of Digestive Tract Cancer (GISCAD).

Cascinu S(1), Scartozzi M, Labianca R, Catalano V, Silva RR, Barni S, Zaniboni A, D'Angelo A, Salvagni S, Martignoni G, Beretta GD, Graziano F, Berardi R, Franciosi V; Italian Group for the Study of Digestive Tract Cancer (GISCAD).

Author information:
(1)Clinica di Oncologia, Università Politecnica delle Marche-Azienda Ospedaliera Umberto I, via Conca, Ancona 60020, Italy. cascinu@yahoo.com

The aim of the present study was to evaluate the role of a weekly preoperative chemotherapy in locally advanced, unresectable gastric cancer. In all, 82

patients with an Eastern Oncology Cooperative Group PS <or=2 and normal cardiac function were enrolled onto the study. Surgical unresectability was confirmed in 52 patients (63%) at laparotomy, and in 30 (27%) cases by CT scan of the abdomen and endoscopic ultrasonography. Chemotherapy treatment was: cisplatin 40 mg x m(-2); 5-fluorouracil 500 mg x m(-2); epidoxorubicin 35 mg x m(-2); 6S-leucovorin 250 mg x m(-2) and glutathione 1.5 g x m(-2) (PELF). One cycle consisted of 8 weekly treatments. Response to chemotherapy was observed in 40 of 82 patients (49%): six (7%) complete and 34 (41%) partial responses, and in four (5%) cases a complete pathological response was confirmed. Of the 40 responding patients, 37 (45%) had potentially curative surgery. Grade 3/4 leucopenia and thrombocytopenia occurred in three and two patients. At a median follow-up of 48 months, 25 of the 37 resected patients (68%) were alive and 24 (65%) were disease free. The median and 4-year survival for the whole group was 17 months and 31%, respectively. The median survival was 12 months for inoperable patients and it was not reached in resected patients.

PMCID: PMC2409717
PMID: 15083179 [PubMed - indexed for MEDLINE]

169. Magy Seb. 2003 Oct;56(5):177-84.

[Neoadjuvant chemotherapy for locally advanced gastric cancer].

[Article in Hungarian]

Kalmár K(1), Cseke L, Káposztás Z, Horváth G, Varga E.

Author information:
(1)PTE AOK Sebészeti Klinika, 7643 Pécs Ifjúság u. 13. kkalmar@aok.pte.hu

Only surgical resection offers cure in gastric cancer. Curative resection unfortunately is possible only in about every second patient diagnosed with gastric cancer. Neoadjuvant chemotherapy may help to reach R0 resection in patients with locally advanced gastric cancer.AIM: To evaluate the ability of ECF neoadjuvant chemotherapy in locally advanced gastric cancer.
PATIENTS AND METHODS: Between December 1998 and April 2003 twenty-four patients with locally advanced gastric cancer were given neoadjuvant chemotherapy according to the ECF scheme. Before treatment, staging examinations were carried

out: endoscopy, barium swallow, chest X-ray, computer-tomography and laparotomy or diagnostic laparoscopy. We treated patients with four three-weeks-long courses of chemotherapy with bolus injection of epirubicin and cisplatin on day 1, and continuous infusion of 5-fluorouracil on day 1 to 21. Twelve weeks long treatment was followed by 4 weeks free of treatment, than staging was repeated to determine response.
RESULTS: 45.83% response rate was achieved. One complete response was observed. Ten patients showed partial response, 3 stable diseases and 10 progressive disease were detected. Twenty-one patients were operated on. Sixteen resections were carried out, ten with curative intent. Overall survival and disease free survival in the whole group were 12.19 and 8.66 months, while in patients with R0 resection 20.66 and 18.33 months.
CONCLUSION: Neoadjuvant chemotherapy provides a hope for cure for patient with locally advanced gastric cancer.

PMID: 15022621 [PubMed - indexed for MEDLINE]

170. Ann Surg Oncol. 2004 Jan;11(1):14-20.

Application of molecular diagnosis for detection of peritoneal micrometastasis and evaluation of preoperative chemotherapy in advanced gastric carcinoma.

Mori T(1), Fujiwara Y, Sugita Y, Azama T, Ishii T, Taniguchi K, Yamazaki K, Takiguchi S, Yasuda T, Yano M, Monden M.

Author information:
(1)Department of Surgery and Clinical Oncology, Graduate School of Medicine, Osaka University, Japan.

Comment in
    Ann Surg Oncol. 2004 Jan;11(1):2-3.

BACKGROUND: In advanced gastric cancer, peritoneal recurrence is the main cause of death after curative surgical resection. The aim of this report was to describe a novel approach for quantitative genetic diagnosis using peritoneal lavage for the identification of patients at high risk for peritoneal recurrence and for evaluation of the clinical response to intraperitoneal chemotherapy in advanced gastric cancer.

METHODS: Nineteen patients with advanced gastric cancer who underwent staging laparoscopy and intraperitoneal chemotherapy before surgical resection or systemic chemotherapy between June 1999 and September 2001 were enrolled in this study. All peritoneal lavage specimens, collected at both staging laparoscopy and gastrectomy, were subjected to real-time quantitative genetic diagnosis.
RESULTS: The reverse transcriptase polymerase chain reaction (RT-PCR) values decreased in 8 cases, stabilized as negative in 5, and increased in 6 during therapy. Patients whose RT-PCR values diminished and were ultimately negative survived except for one, and all but one patient whose values increased during treatment died of recurrence.
CONCLUSIONS: Quantitative evaluation of genetic changes can provide accurate, useful information on the effects of preoperative intra-abdominal chemotherapy and overall prognosis for patients with advanced gastric cancer.

PMID: 14699028 [PubMed - indexed for MEDLINE]

171. Oncology (Williston Park). 2003 Sep;17(9 Suppl 8):32-6.

Irinotecan in esophageal cancer.

Ilson DH(1), Minsky B.

Author information:
(1)Gastrointestinal Oncology Division, Solid Tumor Service, Department of Medicine, Memorial Sloan-Kettering Cancer Center, New York, New York, USA. ilsond@mskcc.org

The limited effectiveness of chemotherapy in esophageal cancer used to palliate metastatic disease or to combine with radiotherapy in locally advanced disease has prompted the evaluation of new systemic agents. Irinotecan (CPT-11, Camptosar) has shown promising activity in a number of gastrointestinal cancers, including esophageal cancer. The phase II evaluation of the combination of weekly irinotecan and cisplatin has shown encouraging response rates exceeding 30% to 50% in esophageal and gastric cancer. Novel regimens include the combination of irinotecan with mitomycin (Mutamycin), the taxanes docetaxel (Taxotere) and paclitaxel, and continuous infusion fluorouracil (5-FU). Irinotecan is an active radiosensitizer, and trials have evaluated the combination of irinotecan with concurrent radiotherapy. We completed a phase I trial combining weekly

irinotecan, cisplatin, and concurrent radiotherapy in locally advanced esophageal cancer. Minimal toxicity has been observed, with no grade 3/4 esophagitis or diarrhea, and hematologic toxicity was also surprisingly minimal. Full doses of weekly irinotecan (65 mg/m2) and cisplatin (30 mg/m2) could be combined safely with concurrent radiotherapy, with a significant rate of pathologic complete response. Phase II evaluation of this chemoradiotherapy regimen as preoperative therapy is planned at single institutions and at the cooperative group level in the United States. Further phase I and II investigation of combined irinotecan, cisplatin, and concurrent radiation is ongoing with the addition of targeted agents, including celecoxib (Celebrex), cetuximab (Erbitux), and bevacizumab (Avastin). Alternative combinations of irinotecan with radiotherapy, including the addition of docetaxel and continuous infusion 5-FU, are also undergoing phase I and II evaluation.

PMID: 14569846 [PubMed - indexed for MEDLINE]

172. Gastric Cancer. 2003;6(3):185-90.

Complete response of a highly advanced gastric carcinoma to preoperative chemoradiotherapy with S-1 and low-dose cisplatin.

Yoshimizu N(1), Saikawa Y, Kubota T, Akiba Y, Yoshida M, Otani Y, Kumai K, Hibi T, Kitajima M.

Author information:
(1)Department of Surgery, School of Medicine, Keio University, 35 Shinanomachi, Shinjuku-ku, Tokyo 160-8582, Japan.

S-1 has been developed as a new oral anticancer drug, based on the biological modulation of 5-fluorouracil. We report a patient with highly advanced gastric carcinoma who was treated successfully with a new combination chemoradiotherapy using S-1 and cisplatin (CDDP). The patient was a 37-year-old man who was diagnosed with advanced gastric carcinoma (T4N3M0) that had invaded the diaphragm and the paraaortic tissues. Remarkable tumor reduction was observed in the primary tumor and metastatic lymph nodes around the stomach after three cycles of the therapy. Radiological examination before surgery determined that a partial response (PR) had been achieved by the initial therapy. Adverse effects included only a gastrointestinal disorder that was limited to grade 2 when low-dose CDDP

was utilized in the regimen, while an initial high dose of CDDP resulted in grade 3 toxicity, due to myelosuppression. The patient underwent curative surgery, including total gastrectomy, D2 lymph node dissection, and splenectomy, after completion of the radiochemotherapy regimen. No surgical complication was observed. No tumor cells were detected by pathological evaluation of the resected stomach and all the regional lymph nodes, confirming a pathological complete response (CR; grade 3). This regimen is a potent treatment for advanced gastric carcinoma, especially when used as preoperative chemotherapy to control cancer cells.

PMID: 14520533 [PubMed - indexed for MEDLINE]

173. Gastric Cancer. 2003;6(3):159-67.

Neoadjuvant chemotherapy with cisplatin, 5-FU, and leucovorin (PLF) in locally advanced gastric cancer: a prospective phase II study.

Ott K(1), Sendler A, Becker K, Dittler HJ, Helmberger H, Busch R, Kollmannsberger C, Siewert JR, Fink U.

Author information:
(1)Department of Surgery, Klinikum rechts der Isar der Technischen Universität München, Ismaninger Str. 22, 81675 Munich, Germany.

Comment in
    Gastric Cancer. 2003;6(3):131-3.

BACKGROUND: Patients with locally advanced gastric cancer (cT3, cT4, N+, M0) have a dismal prognosis, despite complete resection. The objective of this study was to evaluate the toxicity and efficacy of neoadjuvant chemotherapy using the PLF (cisplatin/leucovorin [folinic acid]/5-fluorouracil [FU]) regimen in these patients. Primary endpoints of the study were the toxicity and the response to chemotherapy. Secondary endpoints were the rate of complete resection, survival, and first site of failure.
METHODS: Forty-nine patients with adenocarcinoma of the stomach were enrolled. Staging was based on abdominal computed tomography (CT) scans, endosonography, and laparoscopy. The intention was to administer two cycles (each containing six courses) of preoperative chemotherapy, consisting of cisplatin 50 mg/m(2),

high-dose folinic acid (HD-FA) 500 mg/m(2), and HD 5-FU (HD-5-FU) 2000 mg/m(2) (PLF). Following chemotherapy all patients were referred to surgery. To be evaluable for response, survival, and first site of failure, the patient had to receive at least one cycle of chemotherapy.
RESULTS: Toxicity observed was low, with grade 3 toxicity in fewer than 5% of the patients and two events of grade 4 toxicity (diarrhea and pulmonary embolism). Forty-two of the patients (86%) received at least one cycle of chemotherapy. The clinical response rate in these patients was 26% (11/42 patients). In 76% of the patients (32/42), a complete resection was possible. The median duration of follow-up for the surviving patients was 58 months (range, 38 to 80+ months). The median survival time for the 42 patients assessable for response was 25.4 months (range, 6 to 80+ months). After complete resection, median survival time was 32 months (range, 7.6 to 80+ months). The median survival time for clinically responding patients has not yet been determined, but 5-year survival is 90%. Twenty of the 32 completely resected patients (62.5%) had recurrences. First site of failure was peritoneal dissemination in 10 patients; locoregional and distant recurrences were rare.
CONCLUSION: Neoadjuvant chemotherapy with PLF in patients with locally advanced gastric cancer has low toxicity and reasonable efficacy, allowing administration on an outpatient basis. Clinically responding patients have an excellent outcome after complete resection. The development of peritoneal dissemination even after neoadjuvant chemotherapy and complete resection remains an unsolved problem in patients with nonintestinal type tumors.

PMID: 14520529 [PubMed - indexed for MEDLINE]

174. Gan To Kagaku Ryoho. 2003 Sep;30(9):1351-6.

[A case of advanced gastric cancer attaining histological CR of paraaortic lymph node after TS-1/CDDP neoadjuvant chemotherapy].

[Article in Japanese]

Koizumi Y(1), Hara A, Tomita M, Sakamoto K, Obata H, Shiomi T, Nakajo S, Hashimoto K.

Author information:
(1)Dept. of Medicine, Saiseikai Suita Hospital.

A 60-year-old male complaining of anemic symptoms went through examinations and was diagnosed with gastric cancer (cardia, type 3', cT2, cN3, cH0, cP0, cM0, cStage IV). Further inspection showed multiple lymph node metastases, including, No. 1, 3, 7, 11, and 16 (paraaortic LNs). Poor prognosis was predicted, yet we tried neoadjuvant chemotherapy (NAC) expecting down staging of the tumor. With the efficacy and safety previously proven, we chose TS-1 + CDDP as NAC regimen. TS-1 (tegafur gimestat otastat potassium, = 80 mg/m2) was administered orally for 21 days, followed by CDDP (cisplatin, = 60 mg/m2) i.v. on day 9. One course was completed without any significant adverse effects. The tumor itself showed PR-MR to the chemotherapy, but all the lymph nodes were expected to attain PR from CT findings. Total gastrectomy, lymph node dissection (D3) with Roux-en-Y reconstruction was performed, and histological re-evaluation was made. Macroscopically, the stomach seemed to be penetrated into serosa by the tumor, i.e., se invasion was suggested, yet histologically no cancerous cells were detected within mp and ss layer. Many of the lymph nodes were replaced with fibrosis, some with normal lymph node structure remained. Definitely no malignant cells were detected throughout all the lymph node specimens (Grade 3). Because pathological CR of paraaortic lymph nodes has never been reported previously, this case shows TS-1 + CDDP as a promising NAC regimen for advanced gastric cancer, in a sense that tumors once diagnosed as inoperable would still have the possibility of CR.

PMID: 14518420 [PubMed - indexed for MEDLINE]

175. Am Surg. 2003 Aug;69(8):693-700; discussion 700-2.

Multimodality treatment for esophageal cancer: the role of surgery and neoadjuvant therapy.

Makary MA(1), Kiernan PD, Sheridan MJ, Tonnesen G, Hetrick V, Vaughan B, Graling P, Elster E.

Author information:
(1)Department of Surgery, Georgetown University Hospital, Washington, DC, USA.

Treatment of esophageal cancer has traditionally included surgery as the initial modality. Neoadjuvant chemoradiation therapy has been introduced with the goal of

downstaging tumors before surgical resection; however, its role in esophageal cancer remains controversial. We report 116 patients who underwent esophagogastrectomy with reconstruction for carcinoma of the esophagus or esophagogastric junction over a 10-year period (January 1, 1990 to June 1, 2001). Forty patients underwent neoadjuvant radiation and chemotherapy followed by surgery. Hospital mortality in this group was 7.5 per cent, complete pathologic response (CPR) was 37.5 per cent, and overall 3- and 5-year survival rates were 47 and 38 per cent. Five-year survival in the 15 patients with CPR was 85 per cent. Five patients underwent neoadjuvant single-agent therapy (four chemotherapy and one radiation) followed by surgery, and none survived to 3 years. Seventy-one patients underwent surgery without neoadjuvant therapy. Hospital mortality in this group was 1.4 per cent, with 3- and 5-year survival of 21 and 17 per cent--a decreased long-term survival compared with the neoadjuvant therapy group despite the observation that patients who underwent neoadjuvant therapy had a larger tumor size on presentation (5.5 +/- 0.4 cm vs 3.8 +/- 0.2 cm; P = 0.002). Squamous cell carcinomas seemed to be more responsive to neoadjuvant radiation and chemotherapy followed by surgery than were adenocarcinomas, with a CPR of 44.4 versus 35.5 per cent; however, 5-year survival rates in these complete responders were not significantly different (100% and 78%, respectively; P = 0.97). We report that esophagogastrectomy in conjunction with neoadjuvant therapy results in increased survival compared with surgery without neoadjuvant therapy (P < 0.01), although there may be an increased perioperative mortality associated with neoadjuvant therapy. Further studies are needed to evaluate the role of preoperative chemoradiation and to better identify the pretreatment characteristics of patients with a complete pathological response.

PMID: 12953828 [PubMed - indexed for MEDLINE]

176. Tumori. 2003 Jul-Aug;89(4 Suppl):112-4.

[Combined treatment of inoperable liver metastases from colorectal cancer].

[Article in Italian]

Milandri C(1), Calzolari F, Giampalma E, Vagliasindi A, Bertagni A, Ridolfi L, Passardi A, Ridolfi R, Golfieri R, Verdecchia GM.

Author information:

(1)Dipartimento di Oncologia Medica, Generale II, Unità di Chirurgia Oncologica Avanzata.

AIMS: Liver resection improves survival in selected patients with colorectal liver metastases. However, the majority of patients with colorectal liver metastases have an inoperable oncological disease. The aim is to investigate whether intra-arterial infusion of chemotherapy, improves response to treatment and may convert a selected group of patients with irresectable liver metastases into an operable state.
MATERIALS AND METHODS: Thirty-sex patients (pts) with inoperable hepatic metastases from colorectal cancer were treated with intra-arterial chemotherapy, by angiographic technique. All patients underwent a short 5-FU-based locoregional infusion and the 13 non pretreated patients also received systemic therapy. Evaluation of response was made by CT scan.
RESULTS: Total cycles administered angiographically: 132 (range, 1-11). There were no complications associated with the angiographic procedure and no cases of > grade 2 toxicity. One heavily pretreated pt experienced moderate cholangitis and superficial gastric erosion. Thirty-one pts were assessable (20 pretreated and 11 not); there was 1 complete response (CR), 3 partial remissions (PR), 2 stabilizations (SD) among non-pretreated pts (6/11; CR + PR + SD = 55%) and 1 PR and 8 SD among pretreated pts (9/20; PR + SD = 45%). The remaining 16 pts progressed. Four pts became eligible for radical hepatic resection (1 refused surgery and 3 patients were operated on). There was no peri-operative deaths. Median survival of these 3 pts was 24, 28 and 39+ months.
CONCLUSIONS: Our data, even if based on a relatively small case series, appear to confirm effective local disease control in this clinical setting. Regional chemotherapy used singly or in combination with systemic chemotherapy may convert a selected group of patients with irresectable liver metastases to an oncological disease that can benefit from surgical treatment.

PMID: 12903565 [PubMed - indexed for MEDLINE]

177. Gastric Cancer. 2002;5 Suppl 1:35-40.

Advances in the therapy of gastric cancer.

Macdonald JS(1).

Author information:
(1)Saint Vincents Comprehensive Cancer Center, 325 West 15th Street, New York, New York 10011, USA.

Many issues remain unclear in the management of gastric cancer. Randomized trials have failed to show the superiority of D2 over D1 dissection, and comparisons between countries showing higher survival rates following more extensive surgery may be influenced at least in part by the fact that D1 dissection underestimates disease stage in many patients. No studies have yet shown a benefit from adjuvant chemotherapy. However, the Southwest Oncology Group (SWOG) 9008 trial provides convincing evidence that a regimen of postoperative 5-fluorouracil (5-FU)-based chemoradiotherapy improves disease-free and overall survival when compared with observation alone. In the chemotherapy of advanced disease, use of the epirubicin plus cisplatin plus 5-FU (ECF) regimen leads to significantly longer median survival than 5-FU plus adriamycin plus methotrexate-C (FAMTX), but the rate of complete response remains low and the effect on longterm survival minimal. There are indications that neoadjuvant chemotherapy may increase the resectability of tumors and reduce risk of postoperative recurrence. Substantial improvements in outcome are likely to depend on the integration into multimodality strategies of novel, molecularly targeted agents in all stages of gastric cancer treatment.

PMID: 12772886 [PubMed - indexed for MEDLINE]

178. Scand J Gastroenterol. 2003 Mar;38(3):294-7.

Early cancer of the stomach arising after successful treatment of gastric MALT lymphoma in patients with autoimmune disease.

Raderer M(1), Püspök A, Stummvoll G, Längle F, Chott A.

Author information:
(1)Dept. of Internal Medicine I, Division of Oncology, University of Vienna, Austria. markus.raderer@akh-wien.ac.at

BACKGROUND: Extranodal marginal zone B-cell lymphoma of the mucosa associated lymphoid tissue (MALT lymphoma) arises in lymphoid tissue acquired through chronic antigenic stimulation as exemplified by Helicobacter pylori. Secondary development of gastric cancer, however, is thought to be a rare event. The

detection of a signet ring cell carcinoma during follow-up endoscopy after successful therapy of MALT lymphoma in a patient with Sjögren's syndrome prompted us to analyse the frequency of subsequent gastric cancer in patients with underlying autoimmune disease (AD).
METHODS: Patients with early stage MALT lymphoma and an underlying AD were evaluated for the occurrence of a secondary gastric cancer during the course of follow-up. Data analysed included the type of AD, stage of MALT lymphoma, H. pylori status, treatment for MALT lymphoma and response, follow-up, the presence of a secondary cancer, and time to development of cancer. In all patients, histologic samples were reassessed for the extent of gastritis, presence of intestinal metaplasia or focal atrophy at the time of lymphoma diagnosis.
RESULTS: A total of eight patients with overt AD at the time of diagnosis of MALT lymphoma were identified. All patients were women aged between 56 and 77 years; 5 had Sjögren's syndrome, 2 had autoimmune thyroiditis (1 along with psoriasis) and 1 suffered from polymyalgia rheumatica. All patients had early stage MALT lymphoma restricted to the mucosa and submucosa at the time of diagnosis, and the presence of H. pylori was found in all cases. Two of these patients achieved complete remission (CR) of the lymphoma following H. pylori eradication, while six were judged unresponsive and underwent chemotherapy, resulting in CR in all cases. One patient died from stroke while being in CR for 2 months following chemotherapy. Two patients (25%) developed early cancer limited to the gastric mucosa while being in CR from lymphoma for 9 and 27 months, respectively, and underwent partial gastrectomy. Final staging of gastric cancer revealed pT1pN0M0 in both cases. Of the remaining 5 cases, 1 patient had a local lymphoma relapse 18 months after CR and was salvaged with radiotherapy. In the remaining 4 patients, no evidence of lymphoma recurrence or a second malignancy has been found so far by regular follow-up every 3 months for a time-span between 52 and 63 months after initial diagnosis.
CONCLUSION: Patients with concurrent MALT lymphoma and an underlying autoimmune condition show not only an impaired response to H. pylori eradication but might also be at increased risk for the development of gastric cancer. In view of this, such patients should be followed closely by regular endoscopies after remission of MALT lymphoma.

PMID: 12737445 [PubMed - indexed for MEDLINE]

179. Gastroenterol Clin Biol. 2002 Dec;26(12):1168-71.

[Squamous cell carcinoma of the celiac area. Report of a case and review of the literature].

[Article in French]

Michalet V(1), Gaudin JL, Bancel B, El Khaddari S, Baulieux J, Rode A, Souquet JC.

Author information:
(1)Service d'Hépato-Gastroentérologie, Hôpital de la Croix-Rousse, Lyon, France.

Primary squamous cell carcinoma of the pancreas or of the stomach is rare and represents a controversial entity. The unusual case of a 50-year-old woman with a large squamous cell carcinoma located in the celiac area and involving liver, stomach and pancreas, is reported here. The patient underwent complete surgical resection. The microscopic diagnosis was well-differentiated squamous cell carcinoma without glandular structure. Following the procedure, search for another possible primary lesion (esophagus, anus, colon, lung, head and neck, pelvic floor) was performed. This search was negative. In this context, final diagnosis was primary gastric or pancreatic squamous cell carcinoma. Local recurrence located in the eso-jejunal anastomosis was discovered three years later. Subsequent radiation combined with chemotherapy was instituted, allowing complete remission. During the subsequent 27-month follow-up, no local or systemic recurrence was observed. Pathogenesis of gastric as well as pancreatic primary squamous cell carcinoma remains obscure and controversial. These tumors usually have a very poor prognosis with rapid vascular and lymphatic involvement. Nevertheless, favorable outcome seems possible, as exhibited in our patient.

PMID: 12520205 [PubMed - indexed for MEDLINE]

180. Magy Seb. 2002 Oct;55(5):321-4.

[Neoadjuvant chemotherapy in advanced stomach cancer--a case of complete response].

[Article in Hungarian]

Káposztás Z(1), Cseke L, Kalmár K, Horváth OP.

Author information:
(1)PTE AOK Sebészeti Klinika, 7623 Pécs, Ifjúság u. 13.
zsolt.kaposztas@aok.pte.hu

In the treatment of gastric cancer R0 surgical resection is the only hope for cure. Unfortunately most patients are first seen when they are in an advanced stage, when the possibility of R0 resection is very poor. In these cases administration of other therapies is justified. In the last decade neoadjuvant combination chemotherapy had been introduced with promising results. ECF neoadjuvant chemotherapy has 60% response rate in irresectable cases. We report about a patient with locally advanced gastric cancer treated with neoadjuvant ECF chemotherapy. The patient reacted with complete response, so following chemotherapy R0 resection could be carried out. We describe different therapies used for patients with locally advanced gastric cancer. Neoadjuvant chemotherapy is a recommended therapeutic modality in locally advanced gastric cancer, because it may have the effect of irresectable disease becoming resectable.

PMID: 12474518 [PubMed - indexed for MEDLINE]

181. J Clin Oncol. 2002 Sep 15;20(18):3872-7.

Treatment of extranodal marginal zone B-cell lymphoma of mucosa-associated lymphoid tissue type with cladribine: a phase II study.

Jäger G(1), Neumeister P, Brezinschek R, Hinterleitner T, Fiebiger W, Penz M, Neumann HJ, Mlineritsch B, DeSantis M, Quehenberger F, Chott A, Beham-Schmid C, Höfler G, Linkesch W, Raderer M.

Author information:
(1)Division of Hematology, Division of Gastroenterology, Institute of Medical Informatics, Statistics and Documentation, Karl-Franzens University of Graz, Graz, Austria. gerald.jaeger@kfunigraz.ac.at

PURPOSE: As chemotherapy has not been extensively studied in patients with lymphoma of the mucosa-associated lymphoid tissue (MALT), we initiated a prospective study to evaluate the activity of the nucleoside analog cladribine (2-chlorodeoxyadenosine [2-CdA]) in this disease.

PATIENTS AND METHODS: Patients with histologically verified MALT-type lymphoma were enrolled. 2-CdA was administered at a dose of 0.12 mg/kg body weight on 5 consecutive days, as a 2-hour infusion. Cycles were repeated every 4 weeks for a maximum of six cycles.
RESULTS: Nineteen patients with gastric and seven patients with extragastric MALT lymphoma were enrolled. All patients were chemotherapy-naive, and two had been locally irradiated before systemic relapse of the lymphoma. A total of 102 cycles was administered to our patients (median number of cycles per patient, four). All 25 assessable patients responded to treatment: 21 patients (84%) achieved complete remission (CR) and four patients achieved partial remission. All patients (100%) with gastric presentation, but only three patients (43%) with extragastric presentation, achieved CR. Toxicities were moderate and mainly hematologic and required dose reduction and/or premature discontinuation of therapy in only three cases. Two patients died from vascular events, one shortly after the first cycle because of myocardial infarction and the other from stroke 3 months after the second course. Three patients relapsed after 13, 18, and 22 months and one patient showed progressive disease after 15 months. At present, 24 patients are alive at a median follow-up time of 32 months.
CONCLUSION: Our data demonstrate that 2-CdA is highly effective in inducing CR in 84% of patients with MALT-type lymphoma.

PMID: 12228207 [PubMed - indexed for MEDLINE]

182. Gan To Kagaku Ryoho. 2002 Aug;29(8):1455-9.

[Two cases of stage IV type 4 gastric cancer with good response to TS-1].

[Article in Japanese]

Shibata N(1), Watari J, Satoh T, Tanabe H, Fujiya M, Yasuda A, Saitoh Y, Yokota K, Kohgo Y, Kino Y, Matsuda M, Kasai S.

Author information:
(1)3rd Dept. of Internal Medicine, Asahikawa Medical College.

We report two patients with Type 4 gastric cancers having multiple lymph node metastasis and carcinomatosa which responded well to TS-1. After administration of TS-1 orally for two courses, both patients showed improved extension of the

gastric wall and almost complete reduction of metastatic lymph nodes. In case 2, colonic stenosis due to peritonum carcinomatosa disappeared after chemotherapy with TS-1. Total gastrectomy was performed in both patients in accordance with their wishes. It was confirmed histopathologically that TS-1 was effective against the primary sites and lymph node metastasis. Both patients are well without recurrence and continue taking TS-1.

PMID: 12214477 [PubMed - indexed for MEDLINE]

183. World J Surg. 2002 Sep;26(9):1155-9. Epub 2002 Jun 24.

Neoadjuvant chemotherapy followed by salvage surgery: effect on survival of patients with primary noncurative gastric cancer.

Yano M(1), Shiozaki H, Inoue M, Tamura S, Doki Y, Yasuda T, Fujiwara Y, Tsujinaka T, Monden M.

Author information:
(1)Department of Surgery and Clinical Oncology, Osaka University Graduate School of Medicine, 2-2 Yamadaoka, Suita, Osaka 565-0871, Japan.
myano@surg2.med.osaka-u.ac.jp

The prognosis for gastric cancer patients who undergo noncurative resection is extremely poor. This study evaluated the effects of neoadjuvant chemotherapy for primary noncurative gastric cancer. Thirty-four patients with biopsy-proven noncurative gastric cancer were treated with either of two neoadjuvant chemotherapies: FEMTXP (5-fluorouracil, epirubicin, methotrexate, cisplatin) or THP-FLPM (pirarubicin, 5-fluorouracil, leucovorin, cisplatin, mitomycin C). Noncurability was determined by conventional staging procedures, staging laparoscopy, and exploratory laparotomy. After chemotherapy the resectability of the tumors was reassessed. Patients who were judged to be candidates for curative resection underwent salvage surgery. Of the final 33 patients, 8 (24.2%) showed a major response [0 complete response (CR), 8 partial response (PR)]. In three patients the second laparoscopy revealed disappearance of the peritoneal metastasis. Of the 33 patients, 14 (42.4%) underwent salvage surgery, including 8 curative resections (2 curability A, 6 curability B). Pathologic examinations revealed a grade 2 response in eight patients but no grade 3 response. Univariate analysis showed the following to be significant prognostic factors: histology

type (differentiated type vs. undifferentiated type; p = 0.035), T4 as a noncurative factor (T4 vs. T3 or less; p = 0.025), clinical response (PR + no change vs. progressive disease; p = 0.002), and salvage surgery (resected vs. unresected; p = 0.001). Among these factors, salvage surgery was found to be the only independent prognostic factor by multivariate analysis, with a relative risk of 0.253 and a 95% confidence interval of 0.066 to 0.974. The treatment was well tolerated. Major toxicities of WHO grade 3 or more were leukopenia in 20 (60.6%), gastrointestinal toxicities in 5 (15.2%), renal toxicities in 2 (6.1%), and alopecia in 1 (3.0%). In conclusion, neoadjuvant chemotherapy is effective for primary noncurative gastric cancer when salvage surgery can be performed. A chemotherapy regimen with a higher complete response rate would improve the prognosis of this dismal disease even more.

PMID: 12209246 [PubMed - indexed for MEDLINE]

184. Oncology (Williston Park). 2002 Aug;16(8 Suppl 7):21-5.

Mitomycin as a modulator of irinotecan anticancer activity.

Villalona-Calero MA(1), Kolesar JM.

Author information:
(1)Department of Medicine and Comprehensive Cancer Center, The Ohio State University, Columbus, USA. villalona-1@medctr.osu.edu

Irinotecan and mitomycin (Mutamycin) possess significant single-agent activity against several tumor types, and mitomycin activates topoisomerase I, the cellular target of irinotecan. We conducted a phase I dose-escalation study of irinotecan and mitomycin in 37 evaluable patients with solid tumors. Antitumor responses included 2 complete responses, 5 partial responses, 10 minor responses, and a CA 19-9 tumor marker response. Responders included 14 patients previously treated with chemotherapy for metastatic disease. No pharmacokinetic interaction between mitomycin and irinotecan was apparent when these agents were given 24 hours apart. Responders (complete and partial responses) demonstrated the largest topoisomerase I induction 24 hours following mitomycin infusion. In addition, since maximum topoisomerase I up-regulation was reached 24 hours after administration of mitomycin, a delay in the administration of irinotecan after mitomycin appeared justified. Based on these encouraging phase I data, phase II

clinical trials in breast and esophageal/gastroesophageal junction adenocarcinomas at the recommended doses and schedule are under way.

PMID: 12199629 [PubMed - indexed for MEDLINE]

185. Oncology (Williston Park). 2002 May;16(5 Suppl 5):11-5.

Irinotecan, cisplatin, and radiation in esophageal cancer.

Ilson DH(1), Minsky B, Kelsen D.

Author information:
(1)Department of Medicine, Memorial Sloan-Kettering Cancer Center, New York, New York 10021, USA.

The limited effectiveness of currently available chemotherapy in the treatment of advanced esophageal cancer, and the poor survival achieved in locally advanced disease with combined chemoradiotherapy with or without surgery, have prompted the evaluation of new agents. Irinotecan (CPT-11, Camptosar) has promising single-agent activity in gastrointestinal cancers. In phase II evaluation of weekly irinotecan plus cisplatin, response rates have exceeded 30% in esophageal and gastric cancers. Irinotecan is an active radiosensitizer in preclinical studies and clinical trials in lung cancer. We performed a phase I trial of weekly irinotecan, cisplatin, and concurrent radiotherapy in locally advanced esophageal cancer. Induction chemotherapy with irinotecan and cisplatin was given prior to radiotherapy, over 6 weeks, cycled on a 2-week-on, 1-week-off schedule to relieve dysphagia. Radiotherapy was given subsequently in 180-cGy daily fractions to a total dose of 5,040 cGy. Doses of chemotherapy, when given with concurrent radiotherapy, were cisplatin at 30 mg/m2 followed by irinotecan at escalated doses (40, 50, 65, and 80 mg/m2), on days 1, 8, 22, and 29. Among 18 patients entered in the trial, minimal toxicity has been observed, with no grade 3/4 esophagitis or diarrhea. Hematologic toxicity has been minimal. Dose-limiting toxicity (ie, requiring more than a 2-week delay in radiotherapy) has been seen in one of three patients at the 80-mg/M2 irinotecan dose level, and accrual continues at this dose level. Among 13 evaluable patients, five complete responses have been seen (38%), including three pathologic complete responses in 10 patients undergoing surgery (30%). Asymptomatic pulmonary emboli were noted on the posttreatment computed tomography scan in 3 of 15 patients, prompting the

addition of warfarin sodium (Coumadin) prophylaxis on protocol. Full doses of weekly irinotecan (65 mg/ m2) and cisplatin (30 mg/m2) can be combined safely with concurrent radiotherapy in patients with locally advanced esophageal cancer.

PMID: 12109799 [PubMed - indexed for MEDLINE]

186. Chirurg. 2002 Apr;73(4):312-5.

[Is neoadjuvant therapy for locally advanced stomach carcinoma standard?].

[Article in German]

Ajani JA(1).

Author information:
(1)Department of GI Oncology, Box 426, University of Texas M.D. Anderson Cancer Center, 1515 Holcombe Blvd, Houston, TX 77030-4009, USA. Jajani@mdanderson.org

Despite surgical efforts and encouraging data of a few postoperative therapy trials, locally advanced gastric cancer is in need of the development of effective multimodal therapeutic concepts. Regarding preoperative therapy the goal is to raise the number of complete tumor resections (R0-resections) leading to an improved prognosis of the disease. Neoadjuvant therapy has the theoretical advantage of early destruction of distant micrometastasis with a consecutive reduction of tumor relapse outside the resection margins. The likelihood of R0-resections should be increased with the response of the primary tumor to neoadjuvant therapy. Neoadjuvant chemotherapy using platinum based regimens in gastric cancer has shown its activity in a number of phase II studies. Especially after response to chemotherapy the survival was significantly better after complete surgical tumor resection. The neoadjuvant use of a sequence of chemotherapy followed by radiotherapy before gastrectomy did result in a complete histopathological response in 20-25% of gastric cancer patients. This regimen seems to be promising, but there are still no long term results available. Parallel to the expected data from the first phase III studies the main impact of research in this field has to be focused on to the development of new and effective therapeutic agents and with accompanying identification of factors which are able to predict the response to neoadjuvant treatment.

PMID: 12063914 [PubMed - indexed for MEDLINE]

187. Gastric Cancer. 2001;4(4):192-7.

Phase II study of sequential high-dose methotrexate and fluorouracil combined with doxorubicin as a neoadjuvant chemotherapy for scirrhous gastric cancer.

Takahashi S(1), Kinoshita T, Konishi M, Nakagouri T, Inoue K, Ono M, Sugitou M, Ohtsu A, Boku N, Yoshida S.

Author information:
(1)Department of Surgery, National Cancer Center Hospital East, Kashiwa, Chiba, Japan.

BACKGROUND: The prognosis of scirrhous gastric cancer remains poor when it is treated with surgical resection alone or chemotherapy alone. A phase II study of sequential high-dose methotrexate and fluorouracil, combined with doxorubicin, as a neoadjuvant chemotherapy was conducted in an attempt to evaluate the efficacy of this regimen in improving the survival of patients with scirrhous gastric cancer.
METHODS: Patients were eligible if they had potentially resectable scirrhous gastric cancer with adequate organ functions and no prior treatment. The treatment schedule consisted of methotrexate (1 g/m2, day 1) fluorouracil (1.5 g/m2, day 1), leucovorin (15 mg/m2, days 2-4), and doxorubicin (30 mg/m2, day 15), repeated at a 28-day interval, and followed by radical surgery.
RESULTS: A total of 20 eligible patients were registered. Objective responses in the neoadjuvant chemotherapy segment were observed in 3 of the 20 (15%) patients. No complete remission was observed. The neoadjuvant chemotherapy was associated with grade 3 or 4 neutropenia in 14 of the 20 (70%) patients. The median time from the initial therapy to the operative day was 82 days. Thirteen of the 20 (65%) patients underwent curative resection. No treatment-related deaths occurred. However, the 2-year survival rate in this treatment program (25%) did not show any superiority over that in historical controls.
CONCLUSIONS: Sequential high-dose methotrexate and fluorouracil, combined, with doxorubicin, as a neoadjuvant chemotherapy for scirrhous gastric cancer did not improve the survival rate in spite of improving the curative resection rate.

PMID: 11846062 [PubMed - indexed for MEDLINE]

188. Am J Clin Oncol. 2002 Feb;25(1):71-5.

A phase II study of etoposide, doxorubicin, and carboplatin in the treatment of advanced gastric cancer.

Wang X(1), Pang L, Feng J.

Author information:
(1)Department of Medical Oncology, Jiangsu Cancer Hospital, Jiangsu Cancer Research Institute, Baiziting 42, Nanjing 210009, People's Republic of China.

Many phase II studies have reported improved response rates with severe toxicity of etoposide, doxorubicin (Adriamycin), and cisplatin in advanced gastric cancer. In an attempt to obtain a better regimen with high efficacy and less toxicity, a combination regimen of etoposide, doxorubicin, and carboplatin (EAC) had been developed and evaluated in this phase II study. Forty-six patients with advanced gastric cancer were enrolled in the study. The treatment consisted of doxorubicin 20 mg/m2 given intravenously on days 1 and 7, etoposide 70 mg/m2 intravenously on days 4, 5, and 6, and carboplatin 200 mg/m2 intravenously on days 2 and 8. Therapy was repeated every 4 weeks. Patients who had stable disease or who responded, received an additional two to six cycles of therapy. Among 45 patients evaluable for response and toxicity, there was a 49% objective response rate, including 7% complete remission and 42% partial response. There was 11% stable disease and 27% progressive disease. Among 11 patients with lymph node metastasis only after a curative gastrectomy, there was an 82% objective response rates with 27% having complete remission and 55% having partial response. The median follow-up was 16 months. The median survival duration of all 45 patients was 11 months. The median time to progression was 5 months. The main toxicity was myelosuppression, with a high incidence of 82% leukopenia but only 9% of grades III to IV. Gastrointestinal toxicity was mild, with a low incidence of 42% nausea and vomiting and only 2% of grades III to IV. There were no chemotherapy-related deaths. With mild and tolerable toxicity, the EAC regimen in our study has active antitumor activity in advanced gastric cancer, which may have a positive influence on long-term survival time. It has a high efficacy, especially in patients with lymph node metastasis only after a curative gastrectomy. This regimen deserves further clinical studies for testing activity and toxicity in advanced gastric cancer.

PMID: 11823701 [PubMed - indexed for MEDLINE]

189. World J Gastroenterol. 1999 Feb;5(1):53-56.

Congenital expression of mdr-1 gene in tissues of carcinoma and its relation with pathomorphology and prognosis.

Zhang LJ, Chen KN, Xu GW, Xing HP, Shi XT.

AIM:To detect the congenital expression patterns of mdr-1 gene in commonly encountered malignant tumors in clinic, and the relationship between the expression of mdr-1 gene and the prognostic morphology in esophageal carcinomas.METHODS:A total of 151 resected samples of malignant tumors without preoperative treatment were taken from Anyang City Tumor Hospital.The congenital expression of their mdr-1 gene was detected with reverse transcription polymerase chain reaction (RT-PCR) and was compared with each other.The positive incidence of mdr-1 gene in 46 samples of esophageal carcinoma was compared with their differentiated grades, TNM stages and macroscopic types, and the precautions and advantages of RT-PCR were evaluated.RESULTS:All the 151 samples were confirmed to be malignant histopathologically, including cancers of stomach and gastric cardia (n = 51), esophagus (n = 46), colorectum (n = 16),breast (n = 15), thyroid (n = 10), lung (n = 9) and uterine cervix (n = 24). The positive expression rate of their mdr-1 gene was 33.3%, 37%, 31.3%, 13.2%, 40%, 55%, and 0% respectively. All the 46 samples of esophageal carcinoma were pathologically confirmed to be squamous cell carcinoma. The total expression rate of their mdr-1 gene was 37% (17/46), 35% (6/17), 40% (8/20), and 33% (3/9) for differentiation grade I, II and III respectively. The expression rate of TNM classification was 33% (6/18), 40% (5/12) and 37% (6/16) in stage IIa, IIb andIII. The expression rate was 33% (3/9) in ulcerous type, 37% (3/8) in constrictive types, 33% (5/15) in fungoid types, and 40% (6/14) in medullary types.No statistically significant difference was found.CONCLUSION:Compared with other methods, RT-PCR is more simple, reliable and accurate in detecting mdr-1 gene expression in tissues of tumor. The overexpression of mdr-1 gene in these neoplasms suggested that cases should be handled differently for chemotherapy with rational use of drugs. Excision is the chief treatment for carcinoma of esophagus. The expression of mdr-1 gene in tissues of esophageal cancer is correlated with the parameters of tumor molecular biology which are independent of histopathological morphology.

PMID: 11819387 [PubMed - as supplied by publisher]

190. Gan To Kagaku Ryoho. 2002 Jan;29(1):119-23.

[A complete response after neoadjuvant chemotherapy for advanced gastric cancer with esophageal invasion].

[Article in Japanese]

Yabusaki H(1), Nashimoto A, Tanaka O.

Author information:
(1)Division of Surgery, Niigata Cancer Center Hospital.

The patient was a 65-year-old woman with type 3 gastric cancer (por) in the upper third of the stomach invading esophagus. Because of No. 16 lymph node swelling on abdominal CT examination, she was treated with FLP (5-fluorouracil + Leucovorin + cisplatin) as a neoadjuvant chemotherapy (NAC). The activities of thymidylate synthase (TS) and dihydropyrimidine dehydrogenase (DPD) in the primary tumors upon endoscopic examination were 2.72 pmol/g tissue and 129.1 pmol/mg/min, respectively. After the second course, we carried out lower esophagectomy and spleno-total gastrectomy with D3 including the No. 16 lymph nodes. Histopathological examination of resected specimens showed dense fibrosis and xanthogranulomatous inflammation with foamy cells and giant cells. No residual carcinoma was seen (complete response). The patient is still alive with no sign of recurrence 1 year after surgery. NAC by combination of FLP is thought to be effective for the treatment of highly advanced gastric cancer, especially in cases with locally advanced disease and lymph node metastasis such as the present. Although no relations were seen between NAC effects and TS, DPD activities and TSIR in primary tumors in 12 gastric cancer patients, the survival rate of a low DPD activity group was significantly better than a high group in 106 cases undergoing adjuvant chemotherapy including 5-FU after surgery.

PMID: 11816467 [PubMed - indexed for MEDLINE]

191. Hepatogastroenterology. 2001 Nov-Dec;48(42):1652-5.

Therapeutic strategy for postoperative liver metastasis from esophageal squamous cell carcinoma; clinical efficacy of and problem with hepatic arterial infusion chemotherapy.

Nakajima Y(1), Nagai K, Kawano T, Inoue H, Nara S, Kumagai Y, Iwai T.

Author information:
(1)First Department of Surgery, Tokyo Medical and Dental University School of Medicine, Yushima 1-5-45, Bunkyo-ku, Tokyo 113-8519, Japan.
yasu.nakajima.srg1@tmd.ac.jp

BACKGROUND/AIMS: Despite recent advances in diagnosis and treatment, the prognosis for esophageal squamous cell carcinoma is unsatisfactory. Liver recurrence is frequent in postoperative esophageal squamous cell carcinoma patients, and the prognosis for patients with liver metastasis is poor. This report concerns the therapeutic strategy, especially the efficacy of and the problem with hepatic arterial infusion chemotherapy for liver metastasis from esophageal squamous cell carcinoma.
METHODOLOGY: We performed a retrospective analysis of 8 patients who underwent hepatic arterial infusion between 1993 and 1998. All patients underwent esophagectomy and reconstruction with stomach roll without preoperative chemotherapy and/or radiotherapy. For 6 patients, preceding systemic chemotherapy was performed before hepatic arterial infusion.
RESULTS: The overall response rate of hepatic arterial infusion was 50%, and for the responders, hepatic arterial infusion provided a good quality of life. Hepatic arterial infusion was effective for responders to preceding systemic chemotherapy, but ineffective for non-responders. A complete response was seen in 2 patients, and the liver tumors showed no re-growth after the completion of hepatic arterial infusion. Two patients developed stomach roll ulcers and one experienced the catheter thrombosis, but there were no instances of severe toxicity or complications.
CONCLUSIONS: For postoperative liver recurrence of esophageal squamous cell carcinoma, hepatic arterial infusion is the favorable therapy in terms of efficacy and low-grade toxicity, but has a risk of causing severe complications. We consider it suitable that when preceding systemic chemotherapy is performed before hepatic arterial infusion, hepatic arterial infusion is performed in responders to preceding systemic chemotherapy, and that hepatic arterial infusion is continued as long as possible.

PMID: 11813593 [PubMed - indexed for MEDLINE]

192. Ann Hematol. 2001;80 Suppl 3:B106-7.

Helicobacter pylori eradication therapy in gastric high grade non Hodgkin's lymphoma (NHL).

Alpen B(1), Röbbecke J, Wündisch T, Stolte M, Neubauer A.

Author information:
(1)Department of Hematology, Oncology, Immunology, Hospital of the Philipps-University Marburg, Germany.

BACKGROUND: Primary gastric low-grade lymphoma of the mucosa associated lymphoid tissue (MALT) develops on the background of a chronic Helicobacter pylori (H. pylori) infection. Stable remissions can be induced by H. pylori eradication therapy as shown in clinical trials. In 8 cases of high-grade gastric lymphomas remissions after H. pylori eradication were observed retrospectively.
AIM: We started a pilot-trial to investigate the value of H. pylori eradication therapy in early gastric high-grade B-cell lymphoma prospectively.
PATIENTS AND METHODS: So far, two H. pylori positive patients with high-grade B-cell lymphoma of the stomach stage Ann Arbor I E are included. They received a triple eradication-therapy (Clarithromycin 500 mg/d, Metronidazol 800 mg/d and Omeprazol 40 mg/d) for 7 days. Endoscopic controls are preformed every 4 weeks.
RESULTS: Both patients became H. pylori negative after eradication therapy. One patient achieved complete remission (CR) 38 days after eradication. The continuous complete remission lasts now for 170 days. The second patient received only a partial remission (PR) 4 weeks after eradication and showed a slight progress 4 weeks later. He presently receives chemotherapy (CHOP).
CONCLUSIONS: Patients with early high-grade gastric B-cell lymphomas should receive H. pylori eradication only within clinical trials. It seems to be possible to induce remissions of early high-grade gastric B-cell lymphomas with exclusive H. pylori eradication therapy. The stability of remission remains to be unclear and should be evaluated by following up the patients closely.

PMID: 11757689 [PubMed - indexed for MEDLINE]

193. Leuk Lymphoma. 2001 Aug;42(4):639-47.

Changes in pattern of immunoglobulin heavy chain gene rearrangement and MIB-1 staining before and after eradication of Helicobacter pylori in gastric mucosa-associated lymphoid tissue (MALT) lymphoma.

Kanda M(1), Suzumiya J, Ohshima K, Okada M, Tamura K, Kikuchi M.

Author information:
(1)First Department of Pathology, School of Medicine, Fukuoka University, School of Medicine, Japan.

Gastric low-grade mucosa-associated lymphoid tissue (MALT) lymphomas are strongly associated with infection by Helicobacter pylori (H. pylori). Antibiotic treatment for H. pylori induces a sustained remission in a significant number of patients. We report here the outcome in 13 patients with gastric low-grade MALT lymphomas or suspected gastric lesions, treated for eradication of H. pylori. Patients were followed closely with sequential histological studies, polymerase chain reaction (PCR) amplification of the immunoglobulin heavy chain (IgH) gene and immunohistochemistry for MIB-1. Antibiotic therapy resulted in eradication of H. pylori in all but one case, as assessed histologically. In 12 cases with successful eradication, complete regression was observed histologically in 9 cases (75%) and no regression in 3 (25%). In 7 of 9 (78%) patients who had a complete remission, clonal bands of IgH gene detected on PCR before therapy disappeared after therapy. All 9 patients with complete regression showed a reduced number of MIB-1 positive cells, while 4 cases with no change or disease progression showed no change or increased number of MIB-1 positive cells. There was a strong relationship between density of MIB-1-positive cells and histological score. These results indicate that combination analysis of PCR of IgH and MIB-1 seems to represent a very good current approach for the diagnosis of gastric low-grade MALT lymphoma and to assess the effects of chemotherapy, especially in problematic cases.

PMID: 11697492 [PubMed - indexed for MEDLINE]

194. Int J Radiat Oncol Biol Phys. 2001 Aug 1;50(5):1258-64.

Stage I and II MALT lymphoma: results of treatment with radiotherapy.

Tsang RW(1), Gospodarowicz MK, Pintilie M, Bezjak A, Wells W, Hodgson DC, Crump M.

Author information:
(1)Department of Radiation Oncology, Princess Margaret Hospital, University Health Network, University of Toronto, Toronto, Canada.
richard.tsang@rmp.uhn.on.ca

PURPOSE: Mucosa-associated lymphoid tissue (MALT) lymphoma is a distinct disease with specific clinical and pathologic features that may affect diverse organs. We analyzed our recent experience with Stage I/II MALT lymphoma presenting in the stomach and other organs to assess the outcome following involved field radiation therapy (RT).
PATIENTS AND METHODS: Seventy patients with Stage IE (62) and IIE (8) disease were treated between 1989 and 1998. Patients with transformed MALT were excluded. The median age was 62 years (range, 24--83 years), M:F ratio 1:2.2. Presenting sites included stomach, 15; orbital adnexa, 19; salivary glands, 15; thyroid, 8; lung, 5; upper airways, 3 (nasopharynx, 2; larynx, 1); urinary bladder, 3; breast, 1; and rectum, 1. Staging included site-specific imaging, CT abdomen in 66 patients (94%) and bone marrow biopsy in 54 (77%). Sixty-two patients received radiation therapy: 52 received RT alone, 7 received chemotherapy and RT, and 3 received antibiotics followed by RT. Median RT dose was 30 Gy (range, 17.5--35 Gy). Most frequently used RT prescriptions were 25 Gy (26 patients-18 orbit, 6 stomach, and 2 salivary glands), 30 Gy (23 patients), and 35 Gy (8 patients). Five patients had complete surgical excision of lymphoma and no other treatment (stomach 1, salivary 2, lung 2), whereas 2 patients with gastric lymphoma received antibiotics only. One patient refused treatment and was excluded from the analysis of treatment outcome, leaving 69 patients with a median follow-up of 4.2 years (range, 0.3-11.4 years).
RESULTS: A complete response was achieved in 66/69 patients, and 3 patients had partial response (2 lung, 1 orbit). The 5-year disease-free survival (DFS) was 76%, and the overall survival was 96%. No relapses were observed in patients with stomach and thyroid lymphoma. The 5-year DFS for these patients was 93%, in contrast to 69% for patients presenting in other sites ($p = 0.006$). Among the 5 patients treated with surgery only, 2 relapsed locally (lung, and minor salivary gland). Among 62 patients who received RT, 8 relapsed (2 salivary, 3 orbit, 1 nasopharynx, 1 larynx, 1 breast). Three patients relapsed in the nonirradiated

contralateral paired organ, 4 in distant sites, and 1 in both local and distant sites. The overall local control rate with radiation was 97% (60/62 patients). CONCLUSION: Localized MALT lymphomas have excellent prognosis following moderate-dose RT. Gastric and thyroid MALT lymphomas have better early outcome, as compared to the other sites where distant failure is more common. Relapses were observed in nonirradiated paired organs or distant sites. Further follow-up is required to assess the impact of failure on survival.

PMID: 11483337 [PubMed - indexed for MEDLINE]

195. Oncology. 2001;61(1):16-22.

Complete response of highly advanced gastric cancer with peritoneal dissemination after new combined chemotherapy of S-1 and low-dose cisplatin: report of a case.

Iwahashi M(1), Nakamori M, Tani M, Yamaue H, Sakaguchi S, Nakamura M, Ueda K, Ichiro M, Nishino E, Tanimura H.

Author information:
(1)Second Department of Surgery, Wakayama Medical University, School of Medicine, Wakayama, Japan. makoto@wakayama-med.ac.jp

TS-1(S-1) has been developed as a new oral anticancer drug based on the biological modulation of 5-fluorouracil. We treated a patient with highly advanced gastric carcinoma with a new combination chemotherapy of S-1 and low-dose cisplatin. Remarkable tumor reduction was observed after two cycles of this therapy in the primary tumor and metastatic lymph nodes, and the ascites disappeared. This was concluded to be a partial response. The only adverse effect was skin pigmentation of the fingers (grade 1), leading to early timing of operation after chemotherapy. The gastric tumor showed evident invasion to the serosa. Lymph nodes around the stomach were swollen. Peritoneal dissemination was also recognized in the omentum and mesocolon. Total gastrectomy with regional lymph node dissection was performed. Disseminated tumors were all resected. Histological examination showed that no tumor cells were detected in the gastric primary lesion, metastatic lymph nodes or disseminated peritoneal tumors, suggesting pathological complete remission. It was suggested that this regimen could be a potent combined therapy for the treatment of patients with highly advanced gastric carcinoma, and it could be useful as neoadjuvant chemotherapy.

Further studies are necessary to evaluate the efficacy of this therapy.

Copyright 2001 S. Karger AG, Basel

PMID: 11474243 [PubMed - indexed for MEDLINE]

196. Cancer. 2001 Jul 15;92(2):279-86.

A three-step strategy of induction chemotherapy then chemoradiation followed by surgery in patients with potentially resectable carcinoma of the esophagus or gastroesophageal junction.

Ajani JA(1), Komaki R, Putnam JB, Walsh G, Nesbitt J, Pisters PW, Lynch PM, Vaporciyan A, Smythe R, Lahoti S, Raijman I, Swisher S, Martin FD, Roth JA.

Author information:
(1)Department of Gastrointestinal Medical Oncology, The University of Texas M. D. Anderson Cancer Center, Houston, Texas 77030, USA. jajani@mdanderson.org

BACKGROUND: Patients with locoregional carcinoma of the esophagus or gastroesophageal junction have a poor survival rate after surgery. Preoperative chemotherapy or chemoradiotherapy has not improved the outcome for these patients. Our study was designed to assess the feasibility of preoperative induction combination chemotherapy in addition to chemoradiotherapy to improve the curative resection rate, local control, and survival. PATIENTS AND METHODS Patients having histologic proof of localized carcinoma (either squamous cell carcinoma or adenocarcinoma) of the esophagus or gastroesophageal junction underwent full classification including endoscopic ultrasonography (EUS). Patients first received up to two courses of induction chemotherapy consisting of 5-fluorouracil at 750 mg/m(2)/day as continuous infusion on Days 1--5, cisplatin at 15 mg/m(2)/day as an intravenous bolus on Days 1--5, and paclitaxel at 200 mg/m(2) as a 24-hour intravenous infusion on Day 1. The second course was repeated on Day 29. This was followed by radiotherapy (45 grays in 25 fractions) and concurrent admission of 5-fluorouracil (300 mg/m(2)/day as a continuous infusion 5 days/week) and cisplatin (20 mg/m(2) on Days 1--5 of radiotherapy). After chemoradiotherapy, patients underwent surgery. The feasibility of this approach, curative resection rates, patient survival, and patterns of failure were assessed.

RESULTS: Thirty-seven of 38 patients enrolled were evaluable for toxicity and survival. Adenocarcinoma and distal esophageal location of carcinoma were observed frequently. Thirty-five (95%) of the 37 patients underwent surgery, all of whom had an R0 (curative) resection. A pathologic complete response was noted in 11 (30%) of the 37 total patients. In addition, 5 patients (14%) had only microscopic carcinoma. According to EUS classification, 31 (89%) of the 35 patients who underwent surgery had a T3 carcinoma whereas according to pathologic classification only 3 (9%) had a T3 carcinoma (P </= 0.01). Similarly, according to EUS classification, 23 patients (66%) had an N1 carcinoma, whereas according to pathologic classification only 7 patients (20%) had an N1 carcinoma (P < or = 0.01). At a median follow-up of 20 months (minimum follow-up, 13+ months; maximum follow-up, 36+ months), the median survival duration for the 37 patients had not yet been reached. In addition, there were two deaths related to surgery.
CONCLUSIONS: These data show that the three-step strategy of preoperative paclitaxel-based induction chemotherapy then chemoradiotherapy followed by surgery is feasible and appears quite active in patients having locoregional carcinoma of the esophagus or gastroesophageal junction. Future investigations should focus on substituting cisplatin with less toxic agents and including more systemic therapy with newer classes of agents.

Copyright 2001 American Cancer Society.

PMID: 11466680 [PubMed - indexed for MEDLINE]

197. Ann Ital Chir. 2001 Jan-Feb;72(1):47-53.

[Neoadjuvant chemotherapy in gastric carcinoma].

[Article in Italian]

D'Ugo D(1), Persiani R, Pende V, Picciocchi A.

Author information:
(1)Istituto di Patologia Speciale Chirurgica Policlinico Agostino Gemelli, Università Cattolica del Sacro Cuore Roma. ddugo@tin.it

A complete surgical resection currently represents the only curative treatment option for gastric carcinoma, but as regards locally advanced cancer the

possibility of local or distant recurrence remains extremely high even following a R0 resection. As far as T3-4/N+ tumors are concerned, unsatisfying results of surgery alone have stressed the need for multimodal treatments: in the recent past adjuvant chemotherapy has represented a common complementary treatment for locally advanced gastric cancer, but conclusive results of most randomized trials did not show a significant impact on long term survival. Literature review shows a growing trend throughout the 90's towards the adoption of a preoperative chemotherapy, initially evaluated as a form of "salvage" palliative treatment for unresectable patients. To date a number of phase II study suggests the efficacy of neo-adjuvant treatment administered to resectable patients with the purpose of inducing tumor downstaging, increasing the rate of R0 resections and controlling recurrencies. From March 1996 the Authors have started a controlled study on neo-adjuvant therapy for locally advanced gastric cancer. Accurate staging and patients selection were based upon immediately preoperative laparoscopy. In this ongoing study, patients are administered two preoperative cycles of EEP chemotherapy (Etoposide, Epirubicin, cis-Platin). Preliminary data have been evaluated on the first 15 cases. Grade I myelosuppression has been observed in 12/15 cases and grade II/III in 3/15 cases; 1 patient died by septic complications. Restaging has not shown progression of the disease in 13/14 cases; a macroscopic response was evidenced in 7/14 patients; 14/14 patients could undergo a successful D2 surgical resection following neo-adjuvant therapy. Pathological staging confirmed tumor downstaging in 7 out of 14 cases; 12/14 patients in this group (85.7%) could benefit a R0 resection. These preliminary data encourage us to proceed in our prospective investigation.

PMID: 11464495 [PubMed - indexed for MEDLINE]

198. Ann Surg Oncol. 2001 Jul;8(6):519-24.

A pilot study of preoperative chemoradiotherapy for resectable gastric cancer.

Lowy AM(1), Feig BW, Janjan N, Rich TA, Pisters PW, Ajani JA, Mansfield PF.

Author information:
(1)Department of Surgical Oncology, University of Cincinnati, Ohio, USA.

Comment in
Ann Surg Oncol. 2001 Jul;8(6):482-3.

BACKGROUND: The goals of this study were to assess the feasibility and toxicity of a regimen of preoperative chemoradiotherapy, surgery, and intraoperative radiotherapy in the treatment of patients with potentially resectable gastric cancer. A secondary objective was to assess pathologic response to chemoradiotherapy in the treated tumors.
METHODS: Twenty-four patients were entered in the protocol. Treatment regimen consisted of 45 Gy of external beam radiotherapy with concurrent 5-FU given as a continuous infusion at a dose of 300 mg/m2. Patients were restaged 4-6 weeks after chemoradiotherapy and then underwent surgical resection and intraoperative radiotherapy to a dose of 10 Gy.
RESULTS: Twenty-three patients (96%) completed chemoradiotherapy in accordance with the study protocol. Nineteen (83%) of 23 patients who completed chemoradiotherapy underwent surgical resection with D2 lymphadenectomy. Four patients (17%) had progressive disease and were not resected. The morbidity and mortality rates were 32% and 5%, respectively. Of the resected patients, two (11%) had complete pathologic responses while 12 (63%) had pathologic evidence of significant treatment effect.
CONCLUSIONS: Preoperative chemoradiotherapy for gastric cancer can be delivered safely and is well tolerated. The rate of surgical complications is consistent with that of other recently reported prospective trials of gastrectomy alone. Preoperative chemoradiotherapy resulted in significant pathologic responses in the majority of treated tumors, and complete pathologic responses were achieved in some patients.

PMID: 11456051 [PubMed - indexed for MEDLINE]

199. Onkologie. 2001 Jun;24(3):278-82.

Neoadjuvant radio-chemotherapy of adenocarcinoma of the oesophagogastric junction.

[Article in English, German]

Samel S(1), Hofheinz R, Hundt A, Sturm J, Knoll MR, Wenz F, Queisser W, Post S.

Author information:
(1)Chirurgische Klinik, Universitätsklinik Mannheim, Universität Heidelberg,

Theodor-Kutzer-Strasse 1-3, D-68157 Mannheim, Germany.
stephan.samel@chir.ma.uni-heidelberg.de

BACKGROUND: Recently, neoadjuvant radio-chemotherapy has been demonstrated to induce tumour remission and to prolong survival of patients with locally advanced adenocarcinoma of the oesophagogastric junction.The present study was performed to re-evaluate these data.
PATIENTS AND METHOD: A non-randomised trial of multimodal treatment was conducted in order to investigate histopathologic response and survival of patients with adenocarcinoma of the oesophagogastric junction. Treatment consisted of 2 courses of combined chemotherapy with 15 mg/kg 5-fluorouracil on days 1-5 and 75 mg/m(2) cisplatin on day 8 and simultaneous radiation (40 Gy), and a second course starting on day 36, followed by surgery. Abdomino-thoracic oesophagectomy and systematic 2-field lymphadenectomy were performed in patients with Barrett's carcinoma. D2-gastrectomy was performed in patients with type 2 or 3 cancer of the oesophagogastric junction according to the Siewert classification. Probability of survival was estimated using the Kaplan-Meier method.
RESULTS: 16 patients with a mean age of 57 years were enrolled in this study. Surgery was performed in 14 of these patients. Response to treatment was evident in 10 patients, but none of these patients had complete histopathologic response. Toxicity related to radiochemotherapy was mild to moderate (37.5%). Perioperative complications, both medical and surgical, occurred in 71.4% of patients. 2 patients had fatal complications. 30-day mortality was 25.4%. The probability of survival at 2 years after surgery was 61.2%.
CONCLUSION: Neoadjuvant radio-chemotherapy followed by surgery for cancer of the oesophagogastric junction is associated with a considerable rate of complications. Histopathologic response to radio-chemotherapy is poor. In consequence of these preliminary results, the present study was terminated and the protocol of a future study was modified.

Copyright 2001 S. Karger GmbH, Freiburg

PMID: 11455222 [PubMed - indexed for MEDLINE]

200. Int J Oncol. 2001 Aug;19(2):341-6.

Changes in intratumoral thymidylate synthase (TS) and dihydropyrimidine dehydrogenase (DPD) mRNA expression in colorectal and gastric cancer during

continuous tegafur infusion.

Uchida K(1), Hayashi K, Kuramochi H, Takasaki K.

Author information:
(1)Department of Surgery, Institute of Gastroenterology, Tokyo Women's Medical University, 8-1 Kawadachou, Shinjuku-ku, Tokyo 162-8666, Japan.

Thymidylate synthase (TS) is the target enzyme of 5-fluorouracil (5-FU), and dihydropyrimidine dehydrogenase (DPD) is the key enzyme in the 5-FU catabolic pathway. We wanted to determine whether the TS and DPD mRNA expression levels of gastric and colorectal cancer patients would be affected by tegafur (futrafur:FT)-based chemotherapy and whether changes in their expression might be responsible for patient outcome. Thirty-five patients with resectable advanced primary gastric cancer and 36 patients with resectable advanced primary colorectal cancer were the subjects of this study. They all underwent neoadjuvant chemotherapy with protracted infusion of FT alone or FT plus low doses of cisplatin. The TS and DPD mRNA expression levels of endoscopic biopsy specimens before chemotherapy and surgical specimens after chemotherapy were measured by TaqMan reverse transcription-PCR assay using glyceraldehyde-3-phosphate dehydrogenase (GAPDH) as the internal standard. There was a significant difference in the DPD mRNA levels during chemotherapy in the colorectal cancers. Although the TS and DPD levels were unrelated to any conventional histopathological grade factors, colorectal cancer patients whose surgical specimens contained lower TS and DPD mRNA levels had longer disease-free intervals. The results of this study suggest that FT may affect DPD mRNA expression in colorectal cancer patients, that TS/DPD expression can be regarded as an independent prognostic factor, and that colorectal cancer patients with low TS and low DPD mRNA are candidates for FT-based adjuvant chemotherapy. In addition, quantitative analysis of the change in TS/DPD mRNA in surgical specimens during FT-based chemotherapy might be a more accurate means of predicting the post-operative disease-free interval of colorectal cancer patients than analysis of endoscopic specimens before chemotherapy. There also seems to be a relation between regulation of TS and DPD during FT chemotherapy. Elucidation of the mechanisms regulating TS and DPD mRNA expression might make it possible to predict sensitivity and/or toxicity to FT.

PMID: 11445849 [PubMed - indexed for MEDLINE]

201. Clin Cancer Res. 2001 Jun;7(6):1647-53.

Prediction of peritoneal micrometastasis by peritoneal lavaged cytology and reverse transcriptase-polymerase chain reaction for matrix metalloproteinase-7 mRNA.

Yonemura Y(1), Fujimura T, Ninomiya I, Kim BS, Bandou E, Sawa T, Kinoshita K, Endo Y, Sugiyama K, Sasaki T.

Author information:
(1)Second Department of Surgery, School of Medicine, Kanazawa University, Kanazawa 920-8640, Japan. yonemu@med.kanazawa-u.ac.jp

PURPOSE: Peritoneal dissemination is the most common cause of death associated with gastric cancer. In this study, we report the significance of molecular diagnosis of peritoneal dissemination by means of matrix metalloproteinase-7 (MMP-7) reverse transcriptase-PCR (RT-PCR) assay using preoperative peritoneal wash fluid.
EXPERIMENTAL DESIGN: Preoperative peritoneal lavage by paracentesis was performed on 152 patients with gastric cancer. The peritoneal lavaged fluid was subjected to RT-PCR analysis with primers specific for MMP-7 and conventional cytological Papanicolaou examination.
RESULTS: The MMP-7 RT-PCR assay was able to detect cancer cells at densities even lower than 10 cells/sample. There was no signal of MMP-7 mRNA from mesothelial cells, fibroblasts, peripheral blood, and lavaged fluid from patients with benign disease. Cytological examination and MMP-7 RT-PCR assay results were positive for 27 (18%) and 28 (18%) samples, respectively. The sensitivity for the prediction of peritoneal dissemination by cytology and MMP-7 RT-PCR assay were 46% and 33%, but the combination analysis using both parameters improved the sensitivity rate with 62%. Logistic regression analysis revealed that the cytological examination and MMP-7 RT-PCR assay are independent predictors of peritoneal dissemination.
CONCLUSION: The combination of cytological examination and RT-PCR assay of preoperative peritoneal lavaged fluid is a highly efficient and reliable method for the selection of patients for adjuvant i.p. chemotherapy.

PMID: 11410502 [PubMed - indexed for MEDLINE]

202. J Clin Oncol. 2001 Jun 15;19(12):3058-65.

Prediction of response to preoperative chemotherapy in adenocarcinomas of the esophagogastric junction by metabolic imaging.

Weber WA(1), Ott K, Becker K, Dittler HJ, Helmberger H, Avril NE, Meisetschläger G, Busch R, Siewert JR, Schwaiger M, Fink U.

Author information:
(1)Departments of Nuclear Medicine, Surgery, Pathology, Radiology, and Medical Statistics, Technische Universität München, München, Germany. w.weber@lrz.tum.de

PURPOSE: Preoperative chemotherapy in patients with gastroesophageal cancer is hampered by the lack of reliable predictors of tumor response. This study evaluates whether positron emission tomography (PET) using fluorine-18 fluorodeoxyglucose (FDG) may predict response early in the course of therapy.
PATIENTS AND METHODS: Forty consecutive patients with locally advanced adenocarcinomas of the esophagogastric junction were studied by FDG-PET at baseline and 14 days after initiation of cisplatin-based polychemotherapy. Clinical response (reduction of tumor length and wall thickness by > 50%) was evaluated after 3 months of therapy using endoscopy and standard imaging techniques. Patients with potentially resectable tumors underwent surgery, and tumor regression was assessed histopathologically.
RESULTS: The reduction of tumor FDG uptake (mean +/- 1 SD) after 14 days of therapy was significantly different between responding (-54% +/- 17%) and nonresponding tumors (-15% +/- 21%). Optimal differentiation was achieved by a cutoff value of 35% reduction of initial FDG uptake. Applying this cutoff value as a criterion for a metabolic response predicted clinical response with a sensitivity and specificity of 93% (14 of 15 patients) and 95% (21 of 22), respectively. Histopathologically complete or subtotal tumor regression was achieved in 53% (eight of 15) of the patients with a metabolic response but only in 5% (one of 22) of the patients without a metabolic response. Patients without a metabolic response were also characterized by significantly shorter time to progression/recurrence (P =.01) and shorter overall survival (P =.04).
CONCLUSION: PET imaging may differentiate responding and nonresponding tumors early in the course of therapy. By avoiding ineffective and potentially harmful treatment, this may markedly facilitate the use of preoperative therapy, especially in patients with potentially resectable tumors.

PMID: 11408502 [PubMed - indexed for MEDLINE]

203. Rev Med Interne. 2001 May;22(5):465-8.

[Gastroduodenal metastases: an unusual manifestation of lung cancer. Study of two cases and review ot the literature].

[Article in French]

Metges JP(1), Labat JP, Giroux MA, Simon H, Lucas B, Malhaire JP, Gouerou H.

Author information:
(1)Fédération de cancérologie, hôpital Morvan, CHU, 5, avenue Foch 29200 Brest, France. Jean-Philippe.Metges@univ-brest.fr

INTRODUCTION: Diagnosis of gastroduodenal metastases is rare. Primary tumors are essentially melanomas and breast cancer, and exceptionally lung cancer.
EXEGESIS: We report two patients who have a diagnosis of gastroduodenal metastases as initial manifestation of lung cancer. In one case, the patient died 3 weeks after the diagnosis. In the other case, chemotherapy was performed and complete response was obtained for the gastric metastasis. After a few months, node recurrence was diagnosed and the patient died 8 months after the diagnosis.
CONCLUSION: We review the endoscopic and non-endoscopic literature and discuss the different histological types and therapeutic strategies concerning these unusual manifestations of lung cancer.

PMID: 11402518 [PubMed - indexed for MEDLINE]

204. Cancer. 2001 May 15;91(10):1882-8.

MAGE, BAGE, and GAGE gene expression in patients with esophageal squamous cell carcinoma and adenocarcinoma of the gastric cardia.

Zambon A(1), Mandruzzato S, Parenti A, Macino B, Dalerba P, Ruol A, Merigliano S, Zaninotto G, Zanovello P.

Author information:

(1)Department of Oncology and Surgical Sciences, Oncology Section, University of Padova, Italy.

BACKGROUND: The MAGE, BAGE, and GAGE gene families code for distinct, tumor specific antigens that are recognized by cytotoxic T lymphocytes in the context of HLA molecules. The purpose of this study was to analyze MAGE, BAGE, and GAGE gene expression in the two major histologic types of esophageal carcinoma, squamous carcinoma (ESCc) and adenocarcinoma (CAc), and to correlate their expression patterns with the principal prognostic parameters and long term survival.
METHODS: Gene expression was analyzed in surgical samples from 24 patients with ESCc and 24 patients with CAc by reverse transcriptase-polymerase chain reaction amplification (RT-PCR). None of the patients had received preoperative chemotherapy or radiotherapy, and all were followed until death or for a minimum of 4 years.
RESULTS: Sixteen ESCc samples (67%) and 9 CAc samples (37.5%) expressed at least one of the genes under study. The expression of each MAGE gene in the two histologic types was not significantly different, with the exception of MAGE-4, which was expressed more in ESCc samples than in CAc samples. BAGE and GAGE expression was rather low and, in every case, was associated with the expression of at least one MAGE gene.
CONCLUSIONS: In the group as a whole, and in both ESCc and CAc subgroups, no significant correlation emerged between the expression of any gene and prognostic parameters, such as pathologic tumor, lymph node, or disease stage. Nevertheless, BAGE or GAGE expression was related significantly to a poor prognosis, whereas the expression of MAGE genes (in the absence of BAGE and GAGE expression) was related significantly to a good prognosis.

Copyright 2001 American Cancer Society.

PMID: 11346870 [PubMed - indexed for MEDLINE]

205. Yale J Biol Med. 2001 Jan-Feb;74(1):13-20.

Intra-abdominal desmoplastic small round cell tumor.

Nathan JD(1), Gingalewski C, Salem RR.

Author information:
(1)Department of Surgery, Yale University School of Medicine, New Haven, Connecticut, USA. natha002@mc.duke.edu

BACKGROUND: Intra-abdominal desmoplastic small round cell tumor is a rare malignancy with a predilection for young males. Unique histological and immunocytochemical features distinguish the tumor from other members of the family of small round cell tumors of infancy and childhood. The aggressive nature of tumor spread, relative insensitivity to chemotherapy, and generally incomplete resectability result in a very poor prognosis. The authors report a case of a 39-year-old man with diffuse abdominal and pelvic involvement of intra-abdominal desmoplastic small round cell tumor treated with aggressive chemotherapy and surgery.
METHODS: Computed tomography (CT)-guided biopsy of an omental mass was performed. Histologically, discrete nests of uniform closely packed malignant cells were distributed in a background of focally desmoplastic stroma. Immunocytochemistry demonstrated positivity for epithelial, mesenchymal, and neural markers. On the basis of these unique histological and immunohistochemical characteristics, the diagnosis of desmoplastic small round cell tumor was made. The patient was treated with aggressive neoadjuvant chemotherapy consisting of a high-dose alkylator -based combination regimen, followed by surgery.
RESULTS: The patient had a 10 to 15 percent regression in tumor mass in response to chemotherapy. Laparotomy revealed two large omental masses, another large mass adherent to the left colon and pelvic sidewall, and diaphragmatic, peritoneal and mesenteric studding with small nodules. Complete surgical resection was not possible.
CONCLUSIONS: Intra-abdominal desmoplastic small round cell tumor remains an aggressive malignancy with an extremely poor prognosis. Although some response to chemotherapy may be possible, complete resection is rare, and surgical efforts are generally palliative.

PMCID: PMC2588677
PMID: 11249235 [PubMed - indexed for MEDLINE]

206. Surg Today. 2001;31(1):62-7.

Complete cure of malignant lymphoma of the stomach with a huge adrenal lesion achieved by preoperative chemotherapy and surgery: report of a case.

Matsuda T(1), Okihama Y, Egami K, Wada M, Yoshioka M, Maeda S, Onda M.

Author information:
(1)Department of Gastroenterology, Nippon Medical School Tama-Nagayama Hospital, Tama-shi, Tokyo, Japan.

We report herein the case of a 53-year-old woman with malignant lymphoma of the stomach who was successfully treated by preoperative chemotherapy and surgery. The patient consulted our hospital with the chief complaint of upper abdominal pain. Endoscopy demonstrated a protruding lesion at the antral posterior wall of the stomach, and a post-biopsy pathological diagnosis of diffuse large cell type B-cell lymphoma was established. Moreover, abdominal ultrasonography, computed tomography, and magnetic resonance imaging demonstrated a tumor measuring approximately 10cm in diameter in the left adrenal gland. A total of three courses of chemotherapy using the CHOP regimen were given preoperatively. The CHOP regimen consisted of 100mg of prednisolone administered for 8 days together with 1.9mg of vincristine, 1,000mg of cyclophosphamide, and 60mg of epirubicin administered intravenously on the first day. This resulted in tumor shrinkage, and a distal gastrectomy, lymph node dissection, and left adrenalectomy were subsequently performed. Since the pathological findings of the resected tissue specimen demonstrated complete elimination of the malignant lymphoma, this combination of procedures was defined as having resulted in a complete response. The postoperative course of this patient was uneventful. She is still alive without any sign of tumor recurrence 6 years after her operation, and is being followed up at the outpatient clinic.

PMID: 11213047 [PubMed - indexed for MEDLINE]

207. Chir Ital. 2000 Jul-Aug;52(4):385-91.

[Systemic neoadjuvant chemotherapy in locally advanced gastric carcinoma: phase II study with 5-fluorouracil, epirubicin and etoposide].

[Article in Italian]

Catania G(1), Puleo C, Catalano F, Altadonna V, Scilletta S, Migliore M, Iuppa A, Cardì F.

Author information:
(1)Dipartimento di Chirurgia, Sezione di Chirurgia Generale ed Oncologica, Cattedra di Chirurgia Generale, Università degli Studi di Catania.

Locally advanced gastric adenocarcinomas have a poor prognosis, particularly when the tumours are bulky, located in the cardia or when they present local/regional lymph node involvement. Neoadjuvant chemotherapy for locally advanced gastric cancer is an experimental treatment strategy that may increase resectability and improve survival in patients suffering from an almost uniformly fatal neoplasm. At our institution 11 patients younger than 70 years of age in good physical and mental condition with non-resectable adenocarcinomas of the stomach as determined by endoscopy, computed tomography scans and pathology examinations, were treated with combination chemotherapy [5-fluorouracil (375 mg/m2 i.v. for 5 days, epirubicin (60 mg/m2 i.v. on day 1), etoposide 80 mg/m2 on days 1, 2 and 3, leucovorin 100 mg/m2 for 5 days] every 4 weeks as neoadjuvant chemotherapy. The response to chemotherapy was evaluated after three courses. After three courses, we had one complete response, 8 partial responses or stable disease, and no response in two cases. One patient was still alive 36 months postoperatively. These preliminary results suggest that this protocol is an effective form of neoadjuvant chemotherapy for locally advanced gastric carcinoma.

PMID: 11190529 [PubMed - indexed for MEDLINE]

208. Br J Cancer. 2001 Feb;84(4):470-4.

A phase II study of sequential chemotherapy with docetaxel after the weekly PELF regimen in advanced gastric cancer. A report from the Italian group for the study of digestive tract cancer.

Cascinu S(1), Graziano F, Barni S, Labianca R, Comella G, Casaretti R, Frontini L, Catalano V, Baldelli AM, Catalano G.

Author information:
(1)Division of Medical Oncology, Azienda Ospedale di Parma, Italy.

In advanced gastric cancer, we investigated feasibility and activity of sequential chemotherapy with docetaxel after an intensive weekly regimen

consisting of cisplatin, epidoxorubicin, fluorouracil, leucovorin (PELF) plus filgrastim. Chemotherapy-naive patients with relapsed or metastatic gastric cancer received 8 weekly administrations of chemotherapy with cisplatin 40 mg/m(2), fluorouracil 500 mg/m(2), epidoxorubicin 35 mg/m(2), 6S-stereoisomer of leucovorin 250 mg/m(2) and glutathione 1.5 g/m(2). On the other days filgrastim 5 microg kg(-1) was administered by subcutaneous injection. Subsequently, patients with partial response or stable disease received 3 cycles of docetaxel 100 mg/m(2) every 3 weeks. 40 patients have been enrolled and they are evaluable for response and toxicity. After the PELF regimen, 3 patients achieved complete response, 13 patients showed partial response, 21 patients had stable disease and 3 patients progressed (40% response rate; 95% CI 25% to 55%). After docetaxel, 9 out 34 patients improved the outcome (26.5%); 7 patients with stable disease achieved partial response and 2 patients with partial response achieved complete response. The overall response rate in the 40 patients was 57.5% (95% CI, 42.5% to 72.5%). The PELF regimen did not cause any grade IV toxicity, the most frequent grade III acute side-effects were thrombocytopenia and vomiting which occurred in the 10% of 320 PELF cycles. Docetaxel caused grade III-IV neutropenia and thrombocytopenia in the 10% and the 19% of cycles respectively. Fatigue was a frequent side-effect during both PELF and docetaxel chemotherapy. The sequential application of docetaxel after PELF chemotherapy gained major objective responses with manageable toxicity. This strategy is worth of further investigation in the setting of palliative or neoadjuvant chemotherapy.

Copyright 2001 Cancer Research Campaign.

PMCID: PMC2363773
PMID: 11207039 [PubMed - indexed for MEDLINE]

209. Gan To Kagaku Ryoho. 2000 Nov;27(13):2028-32.

[Significance of neoadjuvant chemotherapy for gastric cancer].

[Article in Japanese]

Fujii M(1), Kochi M, Mochizuki F.

Author information:
(1)Dept. of Surgery III, Nihon University School of Medicine, Tokyo, Japan.

Neoadjuvant chemotherapy for high-risk patients with advanced gastric cancer is important to increase the chance for curative resection and make unresectable gastric cancer tumors resectable by down-staging of the tumor. Tumors with H0, P0, T3, T4, or N3 are the best candidates for this therapy. Randomized controlled phase III studies are needed in conjunction with accurate staging of the disease by laparoscopy. The results of histopathologic evaluation of resected materials following preoperative chemotherapy using oral fluoropyrimidine are thought to be useful as an indicator of chemosensitivity for postoperative adjuvant setting.

PMID: 11103233 [PubMed - indexed for MEDLINE]

210. World J Surg. 2000 Sep;24(9):1130-5; discussion 1135-6.

Appraisal of treatment strategy by staging laparoscopy for locally advanced gastric cancer.

Yano M(1), Tsujinaka T, Shiozaki H, Inoue M, Sekimoto M, Doki Y, Takiguchi S, Imamura H, Taniguchi M, Monden M.

Author information:
(1)Department of Surgery II, Osaka University Medical School, Japan.

More accurate preoperative staging is necessary to determine the treatment strategy for locally advanced gastric cancer. Thirty-two patients with T3 or T4 gastric cancer expected to undergo curative resection based on conventional examinations underwent staging laparoscopy. The disease stages determined were compared with those obtained by conventional methods. The discrepancy rate of disease staging was 16 of 32 (50.0%), with down-staging in 5 of 32 (15.6%) and up-staging in 11 of 32 (34.4%). Of the 32 patients, 13 (40.6%) were found to have unsuspected peritoneal dissemination. The positive predictive value for peritoneal metastasis by staging laparoscopy was 100%, whereas the negative predictive value was 89% (17/19). The accuracy rate was 94%. After laparoscopy, 15 of the 32 (46.9%) were diagnosed as candidates for curative resection. Of these 15 patients who underwent surgery, 13 (86.7%) underwent curative resection (1 R0 and 12 R1); the remaining two underwent R2 resection because of peritoneal metastasis that was undetected by staging laparoscopy. Patients with tumors judged noncurable by laparoscopy (n = 11) received neoadjuvant chemotherapy. In 7

of the 11 cases, salvage surgery was done (one R0, three R1, three R2 resections). A second staging laparoscopy was performed in four cases to determine the indication for salvage surgery. Three of the four were judged to be curable and underwent curative resection. Staging laparoscopy is an effective tool for detecting unsuspected peritoneal metastasis, and it can increase the curative resection rate and decrease unnecessary laparotomy for advanced gastric cancer. Second-look laparoscopy enables accurate assessment of the chemotherapeutic response, which can help in decisions about salvage surgery.

PMID: 11036293 [PubMed - indexed for MEDLINE]

211. Br J Cancer. 2000 Aug;83(4):458-62.

A phase II study of paclitaxel, weekly, 24-hour continous infusion 5-fluorouracil, folinic acid and cisplatin in patients with advanced gastric cancer.

Kollmannsberger C(1), Quietzsch D, Haag C, Lingenfelser T, Schroeder M, Hartmann JT, Baronius W, Hempel V, Clemens M, Kanz L, Bokemeyer C.

Author information:
(1)Department of Hematology/Oncology, University of Tuebingen Medical Center, Tuebingen, Germany.

To evaluate the toxicity and efficacy of combination chemotherapy with paclitaxel, cisplatin and 24 h continuous infusion of 5-FU/folinic acid in patients (pts) with unresectable, locally advanced or metastatic gastric adenocarcinoma. Forty-five chemotherapy-naive pts (28 male and 17 female) with a median age of 60 years (range 35-74) were enrolled. 5-FU 2 g/m2 was given weekly over 24 h i.v. preceded by folinic acid 500 mg/m2 as a 2 h infusion. Paclitaxel 175 mg/m2 was administered as a 3 h-infusion on days 1 and 22 and cisplatin 50 mg/m2 as 1 h infusion on days 8 and 29. Six weeks of therapy (days 1, 8, 15, 22, 29, 36) followed by 2 weeks rest were considered one cycle. A median of 3 cycles (range 1-4) were administered to 45 pts assessable for response, survival and toxicity. Five pts (11%) obtained a CR and 18 pts (40%) a PR (ORR 51%; 95% CI: 35.8-66.3%). Responses were achieved in the liver, lymph nodes, lungs and at the site of the primary tumour. Nine pts (20%) had stable disease. Thirteen pts (29%) were considered to have failed treatment, 8 pts (18%) due to progressive disease

and 5 pts (11%) who did not receive one complete cycle of therapy due to acute non-haematologic toxicity. The median progression-free and overall survival times were 9 months (range 1-36+) and 14 months (range 2-36+), respectively. Neutropenia WHO III(o)/IV(o) occurred in 7 pts (15%) with only 1 pt having grade IV. Additional non-haematologic WHO III(o)/IV(o) toxicities included nausea/vomiting in 5 (11%), alopecia in 22 (49%), and diarrhoea in 1 patient each (2%). Dose reductions or treatment delays were necessary in 8 pts (17%), mainly due to neutropenia. All pts were treated on an outpatient basis. The combination of paclitaxel, cisplatin and continuously infused 5-FU/folinic acid appears to be a highly active regimen for the treatment of pts with advanced gastric cancer. While the overall acceptable toxicity allows its use in the palliative setting, it may also be an attractive option to be tested for neoadjuvant or adjuvant treatment.

PMCID: PMC2374647
PMID: 10945491 [PubMed - indexed for MEDLINE]

212. Clin Oncol (R Coll Radiol). 2000;12(3):182-7.

Preoperative ECF chemotherapy in gastro-oesophageal adenocarcinoma.

Geh JI(1), Glynne-Jones R, Kwok QS, Banerji U, Livingstone JI, Townsend ER, Harrison RA, Mitchell IC.

Author information:
(1)Mount Vernon Hospital, Northwood, UK.

Epirubicin, cisplatin and continuous 5-fluorouracil (5-FU) infusion (ECF) has been reported to result in high clinical response rates in advanced gastro-oesophageal adenocarcinoma and is currently the 'gold standard' chemotherapy regimen for this tumour site. Despite this, its role as preoperative (neoadjuvant) treatment is unproven and therefore remains under investigation. We report our experience using ECF (intravenous epirubicin 50 mg/m2 and cisplatin 60 mg/m2 every 3 weeks, with continuous infusion of 5-FU 200 mg/m2 per day) as preoperative treatment in locally advanced adenocarcinoma of the lower oesophagus, gastro-oesophageal junction and stomach. Of the 23 patients treated (median age 54 years), 19 had potentially resectable disease, four were unresectable and seven had radiological evidence of lymph node involvement. A

median of four cycles of ECF was delivered (range 1-6). Ten of 12 patients (83%) with dysphagia reported improvement of symptoms. Clinical disease progression occurred in six patients (26%) during chemotherapy. WHO grade 3 or 4 toxicity occurred in six patients (26%): four haematological, one mucositis, one vomiting. Seventeen patients (74%) proceeded to surgery; 14 (61%) were resected and three were unresectable. There were two (12%) postoperative deaths from respiratory failure. Major pathological response was seen in three patients (13%): one pathological complete response, two microscopic residual disease. Two patients had Stage II (T2N(0-1)) disease and nine were Stage III (T(3-4)N(0-1)). None of the patients with initially unresectable disease was rendered resectable. After a median follow-up interval of 33 months (range 26-53), the overall median survival was 12 months and 2-year survival was 30%. All patients who were initially unresectable or had radiological evidence of lymph node involvement have died. Therefore, despite good symptomatic response rates, ECF chemotherapy given in the preoperative setting did not appear to improve the outcome of patients with unresectable or radiologically lymph node-positive gastro-oesophageal adenocarcinoma. The role of ECF chemotherapy in resectable tumours is unclear and is currently under investigation in the randomized MRC Adjuvant Gastric Infusional Chemotherapy (MAGIC) study.

PMID: 10942336 [PubMed - indexed for MEDLINE]

213. Surg Clin North Am. 2000 Apr;80(2):659-82; discussions 683-6.

Multidisciplinary approach to esophageal and gastric cancer.

Stein HJ(1), Sendler A, Fink U, Siewert JR.

Author information:
(1)Chirurgische Klinik und Poliklinik, Klinikum rechts der Isar of the Technische Universität München, Germany. stein@ntl.chir.med.tu-muenchen.de

Despite marked advances in surgical therapy for patients with esophageal, esophagogastric, and gastric cancers, the overall prognosis of these patients has not markedly improved during the past decades. Multidisciplinary approaches using adjuvant postoperative and neoadjuvant preoperative therapeutic principles have received increasing attention with regard to the management of these patients. A series of randomized, prospective trials has demonstrated that adjuvant

postoperative radiation or chemotherapy does not result in a convincing survival advantage after complete tumor resection in esophageal, esophagogastric junction, or gastric cancer. The available data on the role of neoadjuvant preoperative therapy are not yet conclusive. Although neoadjuvant therapy may reduce the tumor mass in many patients, several randomized, controlled trials have shown that, compared with primary resection, a multimodal approach does not result in a survival benefit in patients with locoregional, that is, potentially resectable, tumors. In contrast, in patients with locally advanced tumors, that is, patients in whom complete tumor removal with primary surgery seems unlikely, neoadjuvant therapy increases the likelihood of complete tumor resection on subsequent surgery, but only patients with objective histopathologic response to preoperative therapy seem to benefit from this approach. Consequently, in the future, improvements in the overall survival of patients with esophageal, esophagogastric junction, or gastric cancer most likely will be achieved only by tailored therapeutic strategies that are based on the individual tumor location, tumor stage, and consideration of established prognostic factors. A clear classification of the underlying tumor entity, a profound knowledge of the prognostic factors applicable, a thorough preoperative staging, and identification of parameters that allow for the prediction of response to preoperative therapy will become essential for the selection of the optimal therapeutic modality for individual patients.

PMID: 10836011 [PubMed - indexed for MEDLINE]

214. Zentralbl Chir. 2000;125(4):341-7.

[Utilization of multimodal therapy concepts in stomach carcinoma in Germany].

[Article in German]

Bösing NM(1), Heise JW, Röher HD.

Author information:
(1)Klinik für Allgemeine und Unfallchirurgie, Heinrich-Heine-Universität Düsseldorf.

INTRODUCTION: In view of disappointing results after surgery alone multimodal therapeutic regimes are used to improve long-term prognosis in locally advanced

gastric carcinomas. In presence of many reports about encouraging results ("down staging", improved R0-resection rates) but simultaneously missing evidence of efficiency of neoadjuvant therapies in respect to long-term survival (large randomized multicenter trials do not exist until today) and the herewith related uncertainties, we started an inquiry among many surgical units with the intention to evaluate the clinical practice of multimodal treatment for gastric cancer patients in Germany today.

METHODS: In a questionnaire (3/99) we asked among 97 surgical units (41 university hospitals, 56 big community hospitals) in Germany for the management of gastric cancer patients with special interest to practice and state of adjuvant and neoadjuvant therapeutic strategies. Further we analyzed all resected gastric cancer patients (1986-1995) without neoadjuvant treatment in advanced stage of disease (pT3/4NxMx; stage III/IV (UICC'92) in respect to R0-resection rate and long-term prognosis (Kaplan-Meier).

RESULTS: Overall feedback amounted to 78% (76/97) and was higher in university hospitals (90%) than in big community hospitals (70%). Today, neoadjuvant therapies are of more interest than adjuvant therapeutic regimes. But also neoadjuvant therapy is only used in 32% as a rule (in 16% with, in 16% without study conditions). 25% of all surgical units do not employ any neoadjuvant therapy in locally advanced gastric cancer until today. In all other surgical units neoadjuvant treatment is performed more individually and sporadically (43%) only in some patients. Neoadjuvant therapies are practiced by haematooncologists in 50%, gastroenterologists in 32% and surgeons in 27%. The predominant neoadjuvant therapeutic strategy is chemotherapy alone (84%). Many surgical units in Germany are interested to participate in a multicenter trial with more interest in neoadjuvant than adjuvant therapy. 185 of 309 resected gastric cancer patients (60%) were classified as stage IIIa, stage IIIb or stage IV patients. R0-resection rate of these advanced gastric cancer patients amounted to 37%; only 24% of them survived 5 years or more.

CONCLUSIONS: Considering the missing evidence that multimodal therapies are able to prolong long-term survival in advanced gastric cancer patients, its use without study conditions is questionable. Conclusions, taken from data of clinical trials regarding carcinomas of the esophagus and esophagealgastric junction, are inconsistent in respect to long-term prognosis and results are not transferable to gastric carcinomas. A prospective randomized multicenter trial in advanced gastric cancer patients is of great importance. Following our data, in Germany a high readiness to participate in the forthcoming EORTC-study is present.

PMID: 10829314 [PubMed - indexed for MEDLINE]

215. Zentralbl Chir. 2000;125(4):333-40.

[Preoperative downstaging in advanced stomach carcinoma. Wishful thinking or reality].

[Article in German]

Schuhmacher C(1), Fink U, Siewert JR.

Author information:
(1)Chirurgische Klinik und Poliklinik, TU München, Klinikum rechts der Isar.

By the time it is diagnosed, gastric carcinoma is usually already advanced and, as a result, has a poor prognosis. Surgery, with complete (R0) resection of the tumor, is the only chance of cure for this disease. However, in locally advanced gastric carcinoma this is only possible in approximately half of all cases. In order to help improve the prognosis of patients with advanced stage carcinomas, the concept of multimodal therapy is presently being evaluated. The results of studies of postoperative adjuvant therapy have been contradictory, with the result that no indication for such treatment outside of study protocols presently exists. Recently, preoperative application of chemotherapy, the so-called "neoadjuvant" therapy concept, has become increasingly important, since it has been demonstrated that, in individual cases, tumors thought to be primarily unresectable have been able to be completely resected after chemotherapy. Based on the available studies, one can assume that, in a subgroup of patients with not yet identified favorable tumor biologic characteristics, a true down staging of the tumor occurs. To what extent a preoperative "over-staging" may be a factor can only be estimated statistically, since the presently available methods for clinical estimation of tumor stage are never as accurate as the final histopathologic evaluation. Since the recently started, randomized multicenter study under the auspices of the EORTC compares surgery alone with a combination of surgery and preoperative chemotherapy in locally advanced gastric carcinoma, information will soon be available which will help clarify the effectiveness of this therapy concept.

PMID: 10829313 [PubMed - indexed for MEDLINE]

216. Am J Surg. 2000 Mar;179(3):216-22.

Management and long-term results of surgery for localized gastric lymphomas.

Vaillant JC(1), Ruskoné-Fourmestraux A, Aegerter P, Gayet B, Rambaud JC, Valleur P, Parc R.

Author information:
(1)Centre de Chirurgie Digestive, Hôpital Saint Antoine, Paris, France.

BACKGROUND: High- and low-grade gastric lymphomas (GL) differ in their behavior and chemosensitivity. Surgery has to be reevaluated according to the histologic grade of malignancy. We aimed to assess the place of surgery in the management of GL and its results after long-term follow-up.
METHODS: Among 54 patients with primary GL prospectively enrolled from 1984 to 1990, 45 with localized disease were studied. Primary resection was done whenever safe. All patients received chemotherapy adapted to the grade of malignancy and/or to the completeness of the resection.
RESULTS: Among 18 low- and 27 high-grade GL, 35 patients had primary resections; of those, 23 were complete. The complete response rate for all patients with low- and high-grade GL was 67% and 89%, respectively. After a median follow-up of 8 years, the disease-free survival rates for low-grade GL and high-grade GL were 94% and 89%, respectively. It was better after complete resection.
CONCLUSION: Complete resection is a major determinant of prolonged complete remission.

PMID: 10827324 [PubMed - indexed for MEDLINE]

217. Cancer. 2000 May 1;88(9):1979-85.

Chemotherapy for the treatment of patients with primary high grade gastric B-cell lymphoma of modified Ann Arbor Stages IE and IIE.

Raderer M(1), Valencak J, Osterreicher C, Drach J, Hejna M, Kornek G, Scheithauer W, Brodowicz T, Chott A, Dragosics B.

Author information:
(1)Department of Internal Medicine I, University of Vienna, Austria.

BACKGROUND: Surgical intervention and combined modality treatment including radiation and chemotherapy have been studied widely in patients with high grade gastric B-cell lymphoma, whereas to the authors' knowledge the role of chemotherapy alone in patients with localized disease has not been investigated extensively.
METHODS: Twenty-five consecutive patients with primary high grade gastric B-cell lymphoma of localized modified Ann Arbor Stages IE and IIE were studied prospectively at the study institution. Patients age < 75 years (n = 17; age range, 41-75 years) were given a standard regimen comprised of doxorubicin, cyclophosphamide, vincristine, and prednisone (CHOP), whereas patients age > 75 years (n = 8; age range, 82-93 years) were treated at a reduced dose. Restaging was performed after 3 and 6 cycles, followed by every 3 months for the first 2 years, and every 6 months thereafter.
RESULTS: A total of 123 cycles were administered to the study patients, with the median number of 6 cycles per patient (range, 1-9 cycles). At a median follow-up of 24 months (range, 1.5-87+ months), 22 patients were alive without evidence of disease and 3 patients had died (1 patient death was treatment-related). Twenty-four patients who were considered evaluable achieved a complete remission, 21 patients after 3 cycles and the remaining 3 patients after 6 cycles of treatment. Side effects generally were manageable, with only one patient requiring premature discontinuation of treatment due to protracted thrombocytopenia after three courses of therapy, and tolerance was not different between the two age groups. No recurrences were observed at last follow-up.
CONCLUSIONS: The authors believe that chemotherapy using the CHOP regimen is highly effective in the treatment of patients with localized primary high grade gastric lymphoma.

PMID: 10813708 [PubMed - indexed for MEDLINE]

218. J Cancer Res Clin Oncol. 2000 Apr;126(4):233-7.

Gastric cancer in very young adults: apropos four patients and a review of the literature.

Kath R(1), Fiehler J, Schneider CP, Höffken K.

Author information:
(1)Klinik und Poliklinik für Innere Medizin II, Friedrich-Schiller Universität Jena, Germany.

Whether gastric cancer in young adults differs from gastric cancer in older patients has been a controversial issue. It has long been suspected that young patients with gastric cancer have different biological features with a more aggressive course of disease and a poorer prognosis than older patients. This, however, has not been firmly substantiated. We report on the clinical course of four patients (three female and one male) with locally advanced (n = 1) or metastasized (n = 3) non-resectable gastric cancer diagnosed under the age of 29 years (23, 25, 27, 28 years). Prior to diagnosis, all three women had recently been pregnant (1-22 months). Diagnosis was endoscopically biopsy-proven and staging work-up was performed by primary explorative surgery (n = 1), laparoscopy and explorative surgery (n = 1) or CAT scan and ultrasound (n = 2). The delay between initial symptoms and diagnosis was 8-22 weeks (median, 10 weeks). The histology was signet-ring cell (n = 2) or undifferentiated (n = 2) gastric cancer. All patients had the diffuse type of gastric cancer according to Lauren. Patients were treated with the FLAP polychemotherapy regimen consisting of leucovorin, 5-fluorouracil, doxorubicin and cisplatinum, as previously reported. The best response after chemotherapy was partial in two patients. Two patients showed progressive disease. Secondary surgery was performed in three responding patients (one of them responded only locally). One patient achieved no evidence of disease after complete tumor resection (R0). In two patients surgery was palliative (R2/exploration). Three patients died 6, 4 and 8 months after diagnosis. One patient is still alive. In our series, very young adults with gastric cancer had adverse clinical and pathological features. In accordance with other reports, we observed a predominance of female patients and a possible association with recent pregnancies. Though the delay between the first symptoms and diagnosis in our patients was no different from that reported for older patients, special emphasis should be given to prompt referral and diagnostic investigations, ensuring the diagnosis of gastric cancer early in the course of disease.

PMID: 10782897 [PubMed - indexed for MEDLINE]

219. Gan To Kagaku Ryoho. 2000 Mar;27(3):459-63.

[Seven patients with stage IVb advanced gastric cancer who were treated with preoperative chemotherapy].

[Article in Japanese]

Yamanaka N(1), Morisaki T, Nakamura K, Ogawa T, Uchiyama A, Noshiro H, Kuroiwa T, Chijiiwa K, Tanaka M.

Author information:
(1)Dept. of Surgery I, Kyushu University School of Medicine.

We examined the usefulness of preoperative chemotherapy using 5-FU and low-dose CDDP in patients with stage IVb gastric cancer. Between 1996 and 1998, seven patients with stage IVb gastric cancer who received preoperative chemotherapy achieved complete or partial response. One course of the chemotherapy was as follows: arterial or venous infusion of 5-FU (500 or 250 mg/day on day 1-5) and low-dose CDDP (5-10 mg/day on day 1-5) for three weeks. In addition to preoperative chemotherapy, biological response modifiers such as OK-432 and lentinan were used. We evaluated the response with abdominal or chest CT and tumor markers. Although preoperative chemotherapy did not improve the survival rate significantly, the prognosis of these patients seemed to be relatively good. Only slight side effects were found. These results suggest that preoperative chemotherapy using 5-FU and low-dose CDDP may be useful for patients with stage IVb gastric cancer.

PMID: 10740641 [PubMed - indexed for MEDLINE]

220. Ann Surg Oncol. 2000 Jan-Feb;7(1):45-50.

Neoadjuvant chemotherapy with P-ELF (cisplatin, etoposide, leucovorin, 5-fluorouracil) followed by radical resection in patients with initially unresectable gastric adenocarcinoma: a phase II study.

Gallardo-Rincón D(1), Oñate-Ocaña LF, Calderillo-Ruiz G.

Author information:
(1)Medical Oncology Department, Instituto Nacional de Cancerología, Tlalpan,

Mexico DF, Mexico.

BACKGROUND: Gastric cancer is the most frequent gastrointestinal cancer in Mexico. Only 33% of cases are resectable. Our aim was to determine the activity and toxicity of the cisplatin, etoposide, leucovorin, and 5-fluorouracil combination in initially unresectable tumors and to determine its ability to permit resection.
METHODS: Sixty patients with unresectable gastric adenocarcinoma were treated with cisplatin 80 mg/m2, etoposide 80 mg/m2, leucovorin 25 mg/m2, and 5-fluorouracil 800 mg/m2 by central intravenous catheter for 4 consecutive days. Two courses of this combination were followed by surgical resection.
RESULTS: The overall response rate was 36.8% (20 partial responses and one complete response). By using logistic regression analysis, the tumor, node, and metastasis stage (risk ratio, 2.04; 95% confidence interval, 1.03-4.02; P = .039) was identified as the response determinant to chemotherapy. Major toxicity was grade 3 or 4 neutropenia in 67% of patients. Ten resections were performed (17.5%); five were curative and five palliative. Operative morbidity and mortality rates were 40% and 10%, respectively. The median length of survival was 7.46 and 13.3 months for nonresponders and responders, respectively (P = .011).
CONCLUSIONS: The cisplatin, etoposide, leucovorin, and 5-fluorouracil combination is active in advanced gastric cancer and the toxicity level is acceptable. This treatment permits a 17.5% resection rate in previously unresectable tumors. A randomized trial of surgery vs. neoadjuvant chemotherapy plus surgery is warranted.

PMID: 10674448 [PubMed - indexed for MEDLINE]

221. Arch Surg. 2000 Jan;135(1):81-7; discussion 88.

Preoperative chemotherapy, radiotherapy, and surgical resection of locally advanced pancreatic cancer.

Wanebo HJ(1), Glicksman AS, Vezeridis MP, Clark J, Tibbetts L, Koness RJ, Levy A.

Author information:
(1)Department of Surgery, Boston University School of Medicine and Roger Williams Medical Center, Providence, RI 02908, USA.

HYPOTHESIS: Neoadjuvant therapy has the potential to induce regression of high-risk, locally advanced cancers and render them resectable. Preoperative chemoradiotherapy is proposed as a testable treatment concept for locally advanced pancreatic cancer.
DESIGN: Fourteen patients (8 men, 6 women) with locally advanced pancreatic cancer were surgically explored to exclude distant spread of disease, to perform bypass of biliary and/or gastric obstruction, and to provide a jejunostomy feeding tube for long-term nutritional support. A course of chemotherapy with fluorouracil and cisplatin plus radiotherapy was then initiated. Reexploration and resection were planned subsequent to neoadjuvant therapy.
MAIN OUTCOME MEASURES: Tumor regression and survival.
INTERVENTIONS: Surgically staged patients with locally advanced pancreatic cancer were treated by preoperative chemotherapy with bolus fluorouracil, 400 mg/m2, on days 1 through 3 and 28 through 30 accompanied by a 3-day infusion of cisplatin, 25 mg m2, on days 1 through 3 and 28 through 30 and concurrent radiotherapy, 45 Gy. Enteral nutritional support was maintained via jejunostomy tube.
RESULTS: Of 14 patients who enrolled in the protocol and were initially surgically explored, 3 refused the second operation and 11 were reexplored; 2 showed progressive disease and were unresectable and 9 (81%) had definitive resection. Surgical pathologic stages of the resected patients were: Ib (2 patients), II (2 patients), and III (5 patients). Pancreatic resection included standard Whipple resection in 1 patient, resection of body and neck in 1 patient, and extended resection in 6 patients (portal vein resection in 6, arterial resection in 4). One patient who was considered too frail for resection had core biopsies of the pancreatic head, node dissection, and an interstitial implant of the tumorous head. Pathologic response: 2 patients had apparent complete pathologic response; 1 patient had no residual cancer in the pancreatectomy specimen, the other patient who had an iridium 192 interstitial implant had normal core biopsies of the pancreatic head. Five patients had minimal residual cancer in the resected pancreas or microscopic foci only with extensive fibrosis, and 2 patients had fully viable residual cancer. Lymph node downstaging occurred in 2 of 4 patients who had positive peripancreatic nodes at the initial surgical staging. There was 1 postoperative death at 10 days. Sepsis, prolonged ileus, and failure to thrive were major complications. In the definitive surgery group the median survival was 19 months after beginning chemoradiotherapy and 16 months after definitive surgery. The absolute 5-year survival was 11% of 9 patients, 1 is surviving 96 months (with no evidence of disease) after chemoradiotherapy and extended pancreatic resection including resection of the superior mesenteric artery and the portal vein for stage III cancer. In the nonresected group the

mean survival was 9 months (survival range, 7-12 months) after initiation of chemoradiotherapy.
CONCLUSION: A pilot study of preoperative chemoradiotherapy with infusional cisplatin and radiation induced a high rate of clinical pathologic response in patients with locally advanced pancreatic cancer and merits further study in these high-risk patients.

PMID: 10636353 [PubMed - indexed for MEDLINE]

222. Chest. 1999 Dec;116(6 Suppl):463S-465S.

Treatment of esophageal carcinoma.

Lerut T(1), Coosemans W, De Leyn P, Van Raemdonck D, Deneffe G, Decker G.

Author information:
(1)Department of Thoracic Surgery, University Hospital Gasthuisberg, Leuven, Belgium.

Cancer of the esophagus and gastroesophageal junction remains a virulent malignancy with an overall poor prognosis. Especially in the Western hemisphere, the incidence of adenocarcinoma is sharply rising. Over the last two decades, surgery has become the mainstay of treatment. Decreased surgical mortality and standardization of oncologic principles focusing on the completeness of resection are believed to be responsible for the improved 5-year survival rates, which are reaching > or = 30%. Until now, there has been no proven benefit from combined neoadjuvant treatment modalities using chemotherapy or chemoradiotherapy except for the subset of patients showing a complete response at pathologic examination. Further research should focus on new chemotherapeutic agents and the development of molecular markers that allow better identification of candidates for multimodality regimens.

PMID: 10619509 [PubMed - indexed for MEDLINE]

223. Am J Clin Oncol. 1999 Dec;22(6):587-92.

Clinicopathologic features and prognostic factors of primary extranodal

non-Hodgkin's lymphomas in Turkey.

Arican A(1), Dinçol D, Akbulut H, Onur H, Demirkazik A, Cay F, Içli F.

Author information:
(1)Department of Medical Oncology, Baŝkent University, Faculty of Medicine, Ankara, Turkey.

Clinical, histopathologic, and prognostic features of 114 patients with primary extranodal non-Hodgkin's lymphoma were evaluated. Median age of the patients was 48 (range, 15-76) and the ratio of male/female was 55/59. Thirty-seven patients had stage 1, 55 patients stage II, 6 patients stage III, and 16 patients stage IV. The most common sites of primary extranodal non-Hodgkin's lymphoma were the gastrointestinal (GI) tract and head-neck region. Stomach (66%) and tonsils (33%) were the most frequently involved organ in GI tract and head-neck region, respectively. Eighty percent of patients had intermediate or high-grade lymphomas, 20% had low-grade subtypes. Complete remission was achieved in 83% of all patients with chemotherapy +/- radiotherapy +/- surgery. Overall and disease-free survival at 5 years were 63% and 59%, respectively. In conclusion, clinical and histopathologic characteristics and prognosis of our cases with primary extranodal non-Hodgkin's lymphoma were usually similar to those of the cases in Western countries with some differences in the incidence of some specific primary extranodal non-Hodgkin's lymphomas and in the histopathologic subtypes.

PMID: 10597743 [PubMed - indexed for MEDLINE]

224. Am J Clin Oncol. 1999 Dec;22(6):580-6.

Phase II trial of the combination of paclitaxel and 5-fluorouracil in the treatment of advanced gastric cancer: a novel, safe, and effective regimen.

Murad AM(1), Petroianu A, Guimaraes RC, Aragao BC, Cabral LO, Scalabrini-Neto AO.

Author information:
(1)Division of Oncology, Hospital das Clínicas, Federal University of Minas Gerais, Belo Horizonte, MG-Brazil.

This prospective phase II clinical trial was performed to explore the activity and efficacy of the combination of paclitaxel and 5-fluorouracil in the treatment of advanced gastric adenocarcinoma. Thirty-one patients ages 18 to 70 years, with Karnofsky performance status (KPS) >50, adequate cardiac, renal, and hepatic functions, measurable metastatic or locally unresectable disease, life expectancy > or =3 months, signed written informed consent, and without any previous chemotherapy were assigned to receive on an outpatient basis: paclitaxel--175 mg/m2, in a 3-hour infusion on day 1 and 5-fluorouracil--1.5 g/m2, also in a 3-hour infusion on day 2 every 21 days, for a maximum of seven cycles. A system to assess clinical benefit based on KPS, analgesic consumption, and weight gain was also used in this trial. Median age was 61 years (range, 31-70 years). The 29 patients eligible for response and toxicity evaluation underwent 147 cycles of chemotherapy. There were 19 (65.5%) objective responses (95% confidence interval: 48%-83%), including 7 (24.1%) complete responses and 12 (41.4%) partial responses. Three patients had the complete response pathologically confirmed. In three of six patients who went to second-look laparotomy, a potentially curative esophagogastrectomy was possible. The toxicity of this combination was considered low, predictable, and manageable and was characterized mainly by reversible alopecia, peripheral neuropathy, myalgia, and mild neutropenia. Fifteen (51.7%) patients attained a clinical benefit response. The median overall survival was 12 months (range, 2-30+ months) and the 30-month overall survival was 20%. This novel regimen appears to be very effective in advanced gastric cancer. The projected 2-year survival of 20% is higher than that achieved with other first-line regimens. These encouraging results indicate the need for further studies to confirm the merit of this regimen.

PMID: 10597742 [PubMed - indexed for MEDLINE]

225. Anticancer Drugs. 1999 Sep;10(8):729-33.

Mitomycin C continuous infusion as salvage chemotherapy in pretreated patients with advanced gastric cancer.

Hartmann JT(1), Quietzsch D, Daikeler T, Kollmannsberger C, Mayer F, Kanz L, Bokemeyer C.

Author information:
(1)Department of Hematology/Oncology/Immunology, Eberhard-Karls-University

Medical Center II, Tübingen, Germany. joerg.hartmann@med.uni-tuebingen.de

Our purpose was to evaluate the safety and therapeutic activity of continuously infused mitomycin C in patients with recurring or progressive metastatic gastric cancer following first-line chemotherapy. Patients were treated with mitomycin C 20 mg/m2 i.v. over a time period of 120 h followed by a 3-week rest period. All patients received prednisone 50 mg p.o. prophylactically for 5 days to prevent hemolytic uremic syndrome and pulmonary side effects. Twenty-two consecutively enrolled patients were assessable for toxicity and 20 for response evaluation completing at least one full course of chemotherapy (two patients evaluable but not measurable).PATIENT CHARACTERISTICS: median age: 63 years (39-76); Sex (M/F): 13/9; median Karnofsky status: 70% (50-100%); resection of primary tumor n = 12 (55%); sites of metastases: liver n = 17 (77%), locally advanced n = 10 (45%), peritoneum n = 13 (59%), lungs n=5 (23%), bone n=3 (14%) and lymph nodes n=14 (64%). Previous chemotherapy regimens: bolus 5-FU/folinic acid n=6 (27%), ELF n=4 (18%), EAP n=3 (14%) and continuous 5-FU/folinic acid/cisplatin/paclitaxel n=9 (41%). In 20 evaluable patients one complete and five partial remissions were observed; overall response rate 30.0% [95% confidence interval (CI): 9.1-50.9%] with a median response duration of 2.1 months (range: 2-6). The median survival was 3.6 months (95% CI: 2.1-6.0) resulting in a 6-month survival rate of 30% since start of mitomycin C. WHO grade III/IV mucositis, diarrhea and fever/infection occurred in 9% of patients each. Cumulative thrombo- and leukocytopenia (WHO grade II/IV) were observed in four and two patients, respectively. Treatment had to be stopped early in two patients. No severe renal dysfunction, pulmonary toxicity or evidence of hemolytic uremic syndrome was observed. Fatigue during the 120 h infusion of mitomycin C was common (11 of 22 patients). We conclude that continuous infusion of mitomycin C is feasible on an outpatient basis, revealing an acceptable toxicity. Mitomycin C demonstrates single-agent activity in pretreated gastric cancer, but has only limited efficacy following cisplatin/paclitaxel-based first-line chemotherapy.

PMID: 10573205 [PubMed - indexed for MEDLINE]

226. Br J Cancer. 1999 Apr;80(1-2):269-72.

Long-term survival after epirubicin, cisplatin and fluorouracil for gastric cancer: results of a randomized trial.

Waters JS(1), Norman A, Cunningham D, Scarffe JH, Webb A, Harper P, Joffe JK, Mackean M, Mansi J, Leahy M, Hill A, Oates J, Rao S, Nicolson M, Hickish T.

Author information:
(1)Cancer Research Campaign, Section of Medicine and Gastrointestinal Unit, Royal Marsden Hospital and Institute of Cancer Research, Sutton, Surrey, UK.

We report the final results of a prospectively randomized study that compared the combination of epirubicin, cisplatin and protracted venous infusion fluorouracil (5-FU) (ECF regimen) with the standard combination of 5-FU, doxorubicin and methotrexate (FAMTX) in previously untreated patients with advanced oesophagogastric cancer. Between 1992 and 1995, 274 patients with adenocarcinoma or undifferentiated carcinoma were randomized from eight oncology centres in the UK and analysed for response and survival. The overall response rate was 46% (95% confidence interval (CI), 37-55%) with ECF, and 21% (95% CI, 13-28%) with FAMTX (P = 0.00003). The median survival was 8.7 months with ECF and 6.1 months with FAMTX (P = 0.0005). The 2-year survival rates were 14% (95% CI, 8-20%) for the ECF arm, and 5% (95% CI, 2-10%) for the FAMTX arm (P = 0.03). Histologically complete surgical resection following chemotherapy was achieved in ten patients in the ECF arm (three pathological complete responses to chemotherapy) and three patients in the FAMTX arm (no pathological complete responses). The ECF regimen resulted in a response and survival advantage compared with FAMTX chemotherapy. The probability of long-term survival following surgical resection of residual disease is increased by this treatment. The high response rates seen with ECF support its use in the neoadjuvant setting.

PMCID: PMC2363002
PMID: 10390007 [PubMed - indexed for MEDLINE]

227. Ann Surg. 1999 Mar;229(3):303-8.

Response to neoadjuvant chemotherapy best predicts survival after curative resection of gastric cancer.

Lowy AM(1), Mansfield PF, Leach SD, Pazdur R, Dumas P, Ajani JA.

Author information:
(1)Department of Surgical Oncology, The University of Texas M. D. Anderson Cancer

Center, Houston, USA.

Comment in
    Ann Surg. 1999 Mar;229(3):309-12.

OBJECTIVE: In Western populations, long-term survival rates after curative resection of gastric cancer remain extremely poor. The lack of effective adjuvant therapy has prompted the evaluation of neoadjuvant approaches. Since 1988, we have conducted three separate phase II trials using neoadjuvant chemotherapy to treat patients with potentially resectable gastric cancer. The present study was conducted to evaluate whether response to neoadjuvant chemotherapy is predictive of survival in patients with resectable gastric cancer.
METHODS: Eighty-three patients with pathologically confirmed gastric adenocarcinoma were treated with neoadjuvant chemotherapy before planned surgical resection. Response was assessed by upper gastrointestinal series, endoscopy, computed tomography scan, and pathologic examination.
RESULTS: For the three phase II trials, clinical response rates ranged from 24% to 38%. Three patients (4%) had a complete pathologic response. Sixty-one patients (73%) underwent a curative resection. Median follow-up was 26 months. Univariate analysis revealed T stage, number of positive nodes, and response to chemotherapy to be significant predictors of overall survival. However, on multivariate analysis, response to chemotherapy was found to be the only independent prognostic factor.
CONCLUSIONS: Response to neoadjuvant chemotherapy is the single most important predictor of overall survival after neoadjuvant chemotherapy for gastric cancer. These findings support further evaluation of neoadjuvant approaches in the treatment of this disease.

PMCID: PMC1191693
PMID: 10077040 [PubMed - indexed for MEDLINE]

228. Cancer. 1999 Jan 15;85(2):295-301.

Paclitaxel, 5-fluorouracil, and cisplatin combination chemotherapy for the treatment of advanced gastric carcinoma.

Kim YH(1), Shin SW, Kim BS, Kim JH, Kim JG, Mok YJ, Kim CS, Rhyu HS, Hyun JH, Kim JS.

Author information:
(1)Department of Hematooncology, Korea University, College of Medicine, Seoul.

BACKGROUND: Although the clinical efficacy of paclitaxel in the treatment of gastric carcinoma has not been clearly defined, recent reports have suggested a possible role in the treatment of upper gastrointestinal carcinomas in vitro and in vivo. In this study, the authors evaluated the efficacy and toxicity of a combination chemotherapy that included paclitaxel, 5-fluorouracil (5-FU), and cisplatin in the treatment of patients with advanced gastric carcinoma.
METHODS: Forty-one gastric carcinoma patients with metastatic disease, unresectable advanced disease, or relapsed disease were treated with the following regimen, administered every 28 days: paclitaxel 175 mg/m2 by 3-hour intravenous (i.v.) infusion on Day 1, 5-FU 750 mg/m2 by 24-hour continuous i.v. infusion on Days 1-5, and cisplatin 20 mg/m2 by 2-hour i.v. infusion on Days 1-5. Twenty-six patients had measurable disease, and 15 had evaluable disease. All patients were assessable for toxicity.
RESULTS: Twenty-one of the 41 patients (51%; 95% confidence interval [CI], 36.5-65.7%) demonstrated an objective response, including 4 complete responses (10%; 95% CI, 3.9-22.5%). Sixty-five percent of the patients with measurable disease (17 of 26; 95% CI, 58-92.5%) and 27% of the patients with evaluable disease (4 of 15: 95% CI, 11.1-52.3%) achieved a complete response or a partial response. The median response duration was 17 weeks (range, 4-90 weeks), and the median survival duration for all patients was 26 weeks (range, 8 to 118+ weeks). The major toxicity of this treatment was myelosuppression with neutropenia of World Health Organization Grade 3 and 4 in 24% and 10% of the patients, respectively. Nonhematologic toxicity included mucositis, nausea/vomiting, diarrhea, neurotoxicity, and alopecia. Fluid retention occurred in two patients, and one patient had an anaphylatic reaction. Dose reduction was necessary for one patient, because Grade 4 neutropenia and mucositis occurred.
CONCLUSIONS: Paclitaxel, 5-FU, and cisplatin was an active combination regimen in the treatment of advanced gastric carcinoma. The toxicity of this regimen was tolerable. Based on these findings, this combination regimen could be an attractive treatment in the preoperative setting.

PMID: 10023695 [PubMed - indexed for MEDLINE]

229. Langenbecks Arch Chir Suppl Kongressbd. 1998;115:312-7.

[Hypothetical and reliable aspects of pre-, intra- and postoperative adjuvant therapy of stomach carcinoma].

[Article in German]

Meyer HJ(1), Opitz GJ, Jähne J, Wilke H.

Author information:
(1)Klinik für Allgemein- und Viszeralchirurgie, Städtisches Klinikum, Solingen.

The results of surgical therapy of gastric cancer could not be improved in recent years. Therefore, different perioperative modalities were investigated for this tumor. A series of studies could not show any survival benefit using postoperative adjuvant radio- or chemotherapy after complete resection of the tumor. Data available about preoperative chemotherapy in locally advanced tumor stages may demonstrate an increased RO-resection rate after objective remission resulting in a prolonged survival compared to surgery alone. Furthermore, in others trials intraoperative radiation or intraperitoneal chemotherapy could decrease the incidence of locoregional recurrence or peritoneal carcinomatosis and improve the overall survival rate. These treatment modalities, above all preoperative chemotherapy, must be proven in precisely defined patient populations within prospective trials to achieve clearcut data in the future.

PMID: 9931631 [PubMed - indexed for MEDLINE]

230. Cancer. 1998 Nov 1;83(9):1908-16.

High dose chemoradiotherapy followed by esophagectomy for adenocarcinoma of the esophagus and gastroesophageal junction: results of a phase II study of the Eastern Cooperative Oncology Group.

Keller SM(1), Ryan LM, Coia LR, Dang P, Vaught DJ, Diggs C, Weiner LM, Benson AB.

Author information:
(1)Department of Surgery, The Beth Israel Medical Center, New York, New York 10003, USA.

BACKGROUND: To assess the toxicity, local response, and survival associated with multimodality therapy in a cooperative group setting, patients with biopsy-proven clinical Stage I or II adenocarcinoma of the esophagus (staged according to 1983 American Joint Committee on Cancer criteria) or gastroesophageal junction were treated with concomitant radiation and chemotherapy followed by esophagectomy.
METHODS: Radiotherapy was administered in daily 2-gray (Gy) fractions 5 days a week until a total of 60 Gy was reached. 5-fluorouracil (5-FU) was infused continuously at a dose of 1000 mg/m2/day for 96 hours on Days 2-5 and 28-31. On Day 2, a 10 mg/m2 bolus of mitomycin was injected intravenously. Esophagectomy was performed 4-8 weeks following completion of the radiotherapy.
RESULTS: During the 18-month study period (August 1991 through January 1993), 46 eligible patients were accrued from 21 institutions. Eight patients were Stage I and 38 Stage II. Eighty-seven percent of patients (40 of 46) received 6000 centigray (cGy), and all received >5000 cGy. Seventy-eight percent of patients (36 of 46) received >90% of the planned 5-FU dose. Follow-up ranged from 11 to 36 months (median, 22 months). There were eight treatment-related deaths; two were preoperative (from adult respiratory distress syndrome) and six were postoperative. Complete or partial response prior to esophagectomy was observed in 63% of cases, stable disease in 15%, and progression in 20%. Thirty-three patients underwent esophagectomy (transhiatal, n=14; Ivor Lewis, n=16; other, n=3). No tumor was found in the specimens resected from 8 of these 33 patients; this represented a pathologic complete response rate of 17% overall and 24% for those who underwent esophagectomy. Overall median survival was 16.6 months, 1-year survival 57%, and 2-year survival 27%. Survival was significantly worse for patients with circumferential cancers (median, 18.1 months vs. 8.3 months; P <0.05).
CONCLUSION: High dose radiation therapy with concurrent 5-FU and mitomycin may be administered to patients with esophageal adenocarcinoma with acceptable morbidity. However, in a cooperative group setting, esophagogastrectomy following intensive chemoradiotherapy is associated with excessive morbidity and mortality. Circumferential tumor growth is a significant adverse prognostic factor.

PMID: 9806648 [PubMed - indexed for MEDLINE]

231. Eur J Cancer. 1998 Apr;34(5):668-73.

Pre-operative sequential chemo- and radiochemotherapy in locally advanced carcinomas of the lower oesophagus and gastro-oesophageal junction.

Stahl M(1), Vanhoefer U, Stuschke M, Walz MK, Müller C, Wilke H.

Author information:
(1)Department of Internal Medicine (Cancer Research), Essen University Medical School, Germany.

The purpose of this trial was to examine the feasibility of intensive, sequential chemo- and radiochemotherapy followed by surgery in patients with locally advanced carcinomas of the lower oesophagus and the gastro-oesophageal junction (GO junction). The chemotherapy consisted of two courses of 6 weekly administrations of 5-fluorouracil (5-FU) (2.0 g/m2, 24 h infusion) and folinic acid (FA) (500 mg/m2, 2 h infusion) combined with twice weekly cisplatin (50 mg/m2, 1 h infusion). Irradiation of 30 Gy was given concurrently with one course of cisplatin and etoposide. 25 patients with locally advanced (T3-T4 NX M0) squamous cell or adenocarcinoma of the lower oesophagus and GO junction were included and evaluated. Toxicity was usually mild to moderate (WHO grade 1 and 2) with mucositis as the most important side-effect of the pre-operative treatment. Of the patients, 94 and 88% completed the chemo- and radiochemotherapy according to the protocol, respectively. A major response (= partial remission with subjective improvement) to chemotherapy was achieved in 6/10 patients with squamous cell carcinoma and 10/15 with adenocarcinoma. 19 patients had subsequent surgery and complete resection was achieved in 16 (3 patients had intra-abdominal metastases observed at laparotomy). The operative mortality rate was 16% (3/19). 10 of the 16 patients with tumour resection had a pathological complete response. 15 patients (43%) remain alive at a median follow-up of 20 months and the median survival exceeds 16+ months. Our data suggest that this intensive pre-operative chemoradiotherapy programme is feasible and remarkably effective in patients with locally advanced carcinomas of the lower oesophagus or GO junction.

PMID: 9713272 [PubMed - indexed for MEDLINE]

232. Br J Cancer. 1998 Aug;78(3):390-3.

Intensive weekly chemotherapy for locally advanced gastric cancer using 5-fluorouracil, cisplatin, epidoxorubicin, 6S-leucovorin, glutathione and filgrastim: a report from the Italian Group for the Study of Digestive Tract Cancer (GISCAD).

Cascinu S(1), Labianca R, Graziano F, Pancera G, Barni S, Frontini L, Luporini G, Cellerino R, Catalano G.

Author information:
(1)Division of Medical Oncology, S. Salvatore Hospital, Pesaro, Italy.

Local extension prevents curative resection in more than two-thirds of gastric cancer patients. Unfortunately, resectability is one of the main prognostic factors in these patients, and survival is longer when tumours are completely removed. Preoperative chemotherapy is an attractive concept for obtaining curative resection. Thirty-two locally advanced unresectable gastric cancer patients were enrolled in five Italian Group for the Study of Digestive Tract Cancer (GISCAD) centres. For 16 patients, surgical unresectability was based on computerized tomography scan evaluation of tumour size (four patients) and invasion of adjacent structures (12 patients), whereas in another 16 patients locally advanced disease was confirmed by laparotomy. They received weekly administration of cisplatin 40 mg m(-2), 5-fluorouracil 500 mg m(-2), epidoxorubicin 35 mg m(-2), 6S-stereoisomer of leucovorin 250 mg m(-2) and glutathione 1.5 g m(-2). From the day after to the day before each chemotherapy administration, filgrastim was administered by subcutaneous injection at a dose of 5 microg kg(-1). One cycle of therapy consisted of eight weekly treatments. Fifteen of 32 patients (47%) responded to chemotherapy, whereas 13 (41 %) had stable disease and four (12%) progressed on therapy. Of the 15 responding patients, 13 were completely resected after chemotherapy and two of them had a complete pathological response. Two clinically responding patients were found unresectable at operation because of peritoneal seeding. At a median follow-up from the start of treatment of 24 months (range 11-39 months), 10 of 13 resected patients are alive and eight are relapse free. Three patients died after 11, 12, and 14 months respectively. Toxicity was acceptable: side-effects consisted mainly of grade II National Cancer Institute common toxicity criteria (NCICTC) leucopenia and thrombocytopenia in ten patients. Neither treatment-related death nor surgical complications in patients undergoing surgery were observed. This weekly intensive regimen enabled resection in half of previously inoperable tumours with a moderate toxicity. It can be offered to patients with locally advanced unresectable gastric cancer to obtain curative resection.

PMCID: PMC2063041
PMID: 9703289 [PubMed - indexed for MEDLINE]

233. Cancer J Sci Am. 1998 Jul-Aug;4(4):237-46.

Preoperative chemoradiotherapy for carcinoma of the esophagus and gastroesophageal junction.

Posner MC(1), Gooding WE, Landreneau RJ, Rosenstein MM, Clarke MR, Peterson MS, Lembersky BC.

Author information:
(1)Department of Surgery, University of Pittsburgh, Pennsylvania, USA.

Comment in
Cancer J Sci Am. 1998 Jul-Aug;4(4):226-9.

PURPOSE: To determine whether combination 5-fluorouracil, cisplatin, and interferon alfa, an active regimen in advanced esophageal cancer, is efficacious as induction therapy before esophagectomy.
MATERIALS AND METHODS: Forty-four patients with potentially resectable esophageal/gastroesophageal junction adenocarcinoma or squamous cell carcinoma were entered into a phase I/II study of this chemotherapeutic regimen and concurrent external-beam radiotherapy before resection. The initial 16 patients were treated with prolonged-infusion 5-fluorouracil (300 mg/m2 on days 1 to 28), cisplatin (20 mg/m2 on days 1 to 5 and 24 to 28), interferon alfa (3 x 10(6) U/m2 intravenously on days 1 to 5 and 24 to 28; subcutaneous injection every other day on days 6 to 23), and radiation (4000 cGy). The subsequent 28 patients were treated over 21 days with two modifications: dose escalation of 5-fluorouracil (250 to 350 mg/m2) and double-fractionated radiotherapy to a total dose of 4500 cGy.
RESULTS: Forty-one patients completed chemoradiotherapy and were evaluable for toxicity. Adverse events were substantial but tolerable, and most toxic episodes were hematologic and gastrointestinal. Three patients died, and one patient had progressive disease before resection. Of the 37 patients eligible for curative resection, 36 had all gross tumor removed. Thirty-three (80%) patients had a major pathologic response: 10 (24%) with no residual tumor and 23 with only microscopic residual tumor. Median survival for all patients was 27 months and for responders was 36 months.
CONCLUSIONS: This combination regimen is active but yields results similar to

those of other chemoradiotherapy phase II trials; therefore, the contribution of interferon alfa to treatment efficacy remains uncertain. The true worth of preoperative chemoradiotherapy is unknown pending results of phase III trials.

PMID: 9689982 [PubMed - indexed for MEDLINE]

234. Pathol Oncol Res. 1998;4(2):87-95.

Chemotherapy of oesophago-gastric cancer.

Ross PJ(1), Rao S, Cunningham D.

Author information:
(1)The Royal Marsden Hospital, Department of Medicine and the Gastrointestinal Unit, London, England.

Oesophageal and gastric cancers are common tumors that represent a number of challenges for oncologists, gastroenterologists and surgeons. The prognosis remains poor with the majority of patients presenting with advanced disease. Combined chemotherapy and radiotherapy has demonstrated a survival benefit in patients with loco-regional oesophageal cancer compared to radiotherapy alone. In an interim analysis we have observed a 62% response rate using a chemoradiation regimen based on protracted venous infusion of 5-fluorouracil and cisplatin combined with radiotherapy in patients with inoperable oesophageal cancer. Improved outcomes with loco-regional disease has rekindled interest in preoperative therapy. In a trial comparing preoperative chemoradiation to surgery alone in patients with operable oesophageal adenocarcinoma, survival was improved with multimodality treatment. In addition, a study including both adeno- and squamous carcinomas demonstrated a trend towards improved survival. A complete pathological response to chemoradiation was associated with significantly improved survival. Gastric cancer is one of the most chemosensitive solid tumors of the gastrointestinal tract with the majority of patients being suitable for palliative chemotherapy. The ECF (epirubicin, cisplatin, protracted venous infusion 5-fluorouracil) regimen was developed in the Gastrointestinal unit of the Royal Marsden Hospital and first reported in 1991. In a prospective randomised trial including 274 patients ECF has been compared with the standard combination of 5-fluorouracil, adriamycin and methotrexate (FAMTX) in patients with previously untreated gastric cancer. Overall response rate, failure-free and

overall survival were significantly improved with ECE, ECF also demonstrated improved quality of life and cost effectiveness when compared to the FAMTX regimen. ECF should now be regarded as the standard treatment for advanced oesophago-gastric cancer against which new therapies should be compared. In addition the Medical Research Council are conducting a trial randomising patients between surgery alone and perioperative chemotherapy using the ECF regimen in operable gastric cancer.

PMID: 9654592 [PubMed - indexed for MEDLINE]

235. Clin Radiol. 1998 Jun;53(6):422-7.

Correlation of CT with histopathological findings in patients with gastric and gastro-oesophageal carcinomas following neoadjuvant chemotherapy.

Ng CS(1), Husband JE, MacVicar AD, Ross P, Cunningham DC.

Author information:
(1)Department of Diagnostic Radiology, The Royal Marsden Hospital, Sutton, Surrey, UK.

Gastric carcinoma is the fourth commonest cause of death from malignant disease in United Kingdom. In the Western hemisphere, it usually presents with advanced disease, which contributes to its very poor prognosis. Pre-operative (neoadjuvant) chemotherapy offers the possibility of down-staging such tumours and the potential to render tumours operable. Computed tomography (CT) plays a central role in the assessment of patients presenting with the disease, and in those who undergo chemotherapy, in evaluating their response.OBJECTIVE: This study was undertaken to evaluate the role of CT in predicting loco-regional spread of tumour following neoadjuvant chemotherapy in non-metastatic gastric and gastro-oesophageal cancers.
METHODS AND MATERIALS: We correlated CT evidence of loco-regional spread with pathological findings following surgery in 21 patients who received pre-operative chemotherapy.
RESULTS: Residual masses were seen on CT in 19 patients, and 15 contained active tumour, although in four patients no viable tumour was demonstrated at histopathology. The overall accuracy of CT in assessing loco-regional disease was disappointing with sensitivities, specificities, positive and negative predictive

values of 57%, 43%, 75% and 33%, respectively.
CONCLUSIONS: We conclude that CT is not accurate in identifying residual loco-regional spread and therefore should not preclude surgery in those patients who have received neoadjuvant chemotherapy.

PMID: 9651057 [PubMed - indexed for MEDLINE]

236. Chirurg. 1998 Apr;69(4):349-59.

[Multimodal therapy of tumors of the upper gastrointestinal tract].

[Article in German]

Fink U(1), Stein HJ, Siewert JR.

Author information:
(1)Chirurgische Klinik und Poliklinik, Klinikum rechts der Isar, Technischen Universität München.

Adjuvant and neoadjuvant therapeutic principles have in recent years received increasing attention in the management of patients with carcinoma of the upper gastrointestinal tract. A series of randomized prospective trials has demonstrated that adjuvant postoperative radiation or chemotherapy does not result in a convincing survival advantage after complete tumor resection in gastric or esophageal cancer. The available data on the role of neoadjuvant preoperative therapy in these patients as yet permit no conclusion. While neoadjuvant therapy may reduce the tumor mass in a substantial portion of patients, a series of randomized controlled trials has shown that, compared to primary resection, a multimodal approach does not result in a survival benefit in patients with loco-regional, i.e. potentially resectable, tumors. In contrast, in patients with locally advanced tumors, i.e. tumors for which complete removal with primary surgery appears unlikely, neoadjuvant therapy increases the chance for complete tumor resection on subsequent surgery. However, only patients with objective histopathologic response to preoperative therapy appear to benefit from this approach. Compared to preoperative chemotherapy alone, combined radio-chemotherapy increases the rate of response, particularly in squamous cell esophageal cancer, but may also increase postoperative morbidity and mortality. Neoadjuvant therapy should therefore currently only be performed in experienced

centers within the context of prospective clinical trials. The identification of factors that would allow prediction of response to neoadjuvant or adjuvant therapy is the focus of ongoing studies.

PMID: 9612616 [PubMed - indexed for MEDLINE]

237. Med Clin (Barc). 1998 Jan 24;110(2):45-50.

[Primary gastrointestinal lymphomas: response to eradicative therapy and prognostic factors in 52 patients].

[Article in Spanish]

Hernández JA(1), Ribera JM, Oriol A, Batlle M, Vaquero M, Flores A, Fernández-Llamazares J, Piñol M, Millá F, Feliu E.

Author information:
(1)Servicio de Hematología-Hemoterapia, Hospital Universitari Germans Trias i Pujol, Badalona.

Comment in
    Med Clin (Barc). 1998 Jan 24;110(2):56-61.

BACKGROUND: To analyze the response to eradicative therapy and prognostic factors in 52 patients with primary gastrointestinal lymphoma (PGIL) diagnosed at a single institution in a 13 year period.
PATIENTS AND METHODS: The main clinical, biological and evolutive data were recorded. Pathologic diagnosis of PGIL was made according to the Working Formulation. Clinical stage was determined by the Ann Arbor system modified by Mushoff. The results of therapy as well as the influence of such characteristics on complete remission (CR), disease-free survival (DFS) and overall survival (OS) were studied.
RESULTS: Mean age of the series was 53 years (SD 15). Thirty patients were males. HIV infection preceded PGIL diagnosis in 10 cases. Seventeen had bad performance status (ECOG 2-4) and 30 B symptoms. The PGIL localization was gastric in 31 cases and 29 had a low grade malignant lymphoma. B phenotype was demonstrated in 98% and 22 patients presented advanced stages (IIE2-IV). Treatment was radical surgery followed by intensive chemotherapy in 32 cases, intensive chemotherapy

alone in 17, and surgical resection in 3. CR was obtained in 34 patients and 6 of them relapsed. The projected DFS from CR at 9 years was 72% and OS was 26%. CR and survival were not influenced by PGIL localization and treatment type. The main unfavourable prognostic factors were advanced stage (CR and OS), B symptoms (DFS and OS) and advanced ECOG score (CR, DFS and OS). Previous HIV infection had an independent prognostic influence on both CR and OS.
CONCLUSIONS: In patients with PGIL, the achievement of CR, DFS and survival have been independent of the type of eradicative treatment used. Performance status, B symptoms and clinical stage have been the main prognostic factors. HIV infection carried an independent prognostic significance.

PMID: 9580161 [PubMed - indexed for MEDLINE]

238. Anticancer Res. 1998 Jan-Feb;18(1B):523-30.

Neoadjuvant chemotherapy of gastric cancer with oral UFT (a mixture of uracil and fturafur) during the waiting period for surgery.

Nio Y(1), Sato Y, Nagami H, Teramoto M, Inoue Y, Yano S, Sumi S, Tamura K, Fukumoto M.

Author information:
(1)First Department of Surgery, Shimane Medical University, Shimane, Japan.

Our previous experience has demonstrated that growth of gastric cancer during the waiting period for surgery cannot be neglected, and some patients hope to receive prophylactic treatment to inhibit the growth of tumor until surgery. The present study was designed to assess the clinical benefits of preoperative chemotherapy with oral UFT for gastric cancer during the waiting period for surgery. Fifty patients with gastric cancer (24 early, 25 advanced and 1 recurrent cancers) were treated with oral UFT at 300-600 mg/day for 7-36 days before surgery and the objective responses and the postsurgical survivals were evaluated. In 42 of 50 patients objective responses of primary lesions were assessed by endoscopy or upper gastrointestinal series examination, and 2 CRs, 15 PRs and 25 NCs were seen (40% response). The histological effect was evaluated in 50 patients and the following classifications were made: grade 3 (complete disappearance or necrosis of tumor cells), 2; grade 2 (necrotic changes > 2/3 area), 4; grade 1b (> 1/3 area), 7; grade 1a (< 1/3 area), 15; and grade 0 (no histological changes), 22. A

longer period of UFT administration was associated with CR or PR. All the patients underwent gastrectomy (38 curative and 12 palliative gastrectomies): all patients with Stage I-III primary gastric cancer are alive after surgery, and the 50% survival period of the patients with Stage IV cancer was 20 months. The side effects were not serious, including slight myelotoxicity, liver dysfunction and anorexia. It is concluded that preoperative chemotherapy for gastric cancer with oral UFT on outpatient basis may result in down-staging as well as the prevention of tumor growth during the waiting period for surgery without serious side effects.

PMID: 9568172 [PubMed - indexed for MEDLINE]

239. Am J Kidney Dis. 1998 Apr;31(4):713-8.

Successful treatment of IgA nephropathy in association with low-grade B-cell lymphoma of the mucosa-associated lymphoid tissue type.

Mak SK(1), Wong PN, Lo KY, Wong AK.

Author information:
(1)Department of Medicine, Kwong Wah Hospital, Kowloon, Hong Kong.
skmak@medi.net.hk

Kidney and the urogenital tract are among the various mucosal sites involved in mucosa-associated lymphoid tissue (MALT) lymphoma. We report a case with simultaneous onset of crescentic immunoglobulin (Ig) A nephropathy and gastrointestinal low-grade B-cell lymphoma of the MALT type with kidney infiltration. M-component of IgM lambda was detected in the serum, and the renal biopsy specimen showed monotypic lambda light chain staining in the lymphoma cells but not the glomeruli. The heavy proteinuria and impaired creatinine clearance returned to normal, and microscopic hematuria disappeared 20 months after treatment with chlorambucil as single-agent chemotherapy. This coincided with a complete resolution of the gastric and renal lymphoma infiltration. The close association of both the onset and successful outcome of the two entities thus support their possible causal relationship, and we discuss the possibility of an association of the disturbance of the MALT by the lymphoma cells with the pathogenesis of IgA nephropathy.

PMID: 9531192 [PubMed - indexed for MEDLINE]

240. J Clin Oncol. 1998 Jan;16(1):309-16.

ERCC1 mRNA levels complement thymidylate synthase mRNA levels in predicting response and survival for gastric cancer patients receiving combination cisplatin and fluorouracil chemotherapy.

Metzger R(1), Leichman CG, Danenberg KD, Danenberg PV, Lenz HJ, Hayashi K, Groshen S, Salonga D, Cohen H, Laine L, Crookes P, Silberman H, Baranda J, Konda B, Leichman L.

Author information:
(1)University of Southern California/Norris Comprehensive Cancer Center, Los Angeles, CA 90033, USA.

PURPOSE: We have previously shown that relative thymidylate synthase (TS) mRNA levels in primary gastric adenocarcinomas treated with fluorouracil (5-FU) and cisplatin are inversely associated with response and survival. This is a presumed function of TS as a target for 5-FU activity. We now test the hypotheses that the relative mRNA level of the excision repair cross-complementing (ERCC1) gene is inversely associated with response and survival as an independent function of cisplatin efficacy.
PATIENTS AND METHODS: Patients had intact, untreated, primary gastric adenocarcinoma cancer and were evaluated for eligibility on a preoperative cisplatin infusion-5-FU protocol. cDNA, derived from primary gastric tumors before chemotherapy, was used to determine ERCC1 mRNA levels, expressed as the ratio of polymerase chain reaction (PCR) product of the ERCC1 gene and the beta-actin gene.
RESULTS: The median ERCC1 mRNA level from 38 primary gastric cancers (33 assessable for response) was 5.8 x 10(-3) (range, 1.8 x 10(-3) to 19.5 x 10(-3)). Of 17 responding patients, 13 (76%) were less than or equal to 5.8 x 10(-3) and four were greater than 5.8 x 10(-3) (P = .003). The median survival for patients with ERCC1 mRNA levels less than or equal to 5.8 x 10(-3) has not been reached, whereas for those greater than 5.8 x 10(-3) it was 5.4 months (P = .034). The median TS mRNA level, 3.7 x 10(-3) (range, 0.9 to 18.9) also segregated responsive versus resistant tumors (P = .024). With both ERCC1 and TS mRNA levels below their medians, 11 of 13 patients (85%) responded; with both ERCC1 and TS

mRNA levels above their medians, two of 10 patients (20%) responded (P = .003). CONCLUSION: Considered separately, either ERCC1 or TS mRNA levels in a primary gastric adenocarcinoma has a statistically significant relationship to response. ERCC1 mRNA levels have a statistically significant association with survival; in this cohort TS mRNA levels did not reach statistically significant association with survival as in our previous publication. Whether these molecular parameters are independent of each other as predictors of outcome remains to be determined.

PMID: 9440758 [PubMed - indexed for MEDLINE]

241. Chest. 1998 Jan;113(1 Suppl):112S-119S.

Current status of new drugs and multidisciplinary approaches in patients with carcinoma of the esophagus.

Ajani JA(1).

Author information:
(1)Department of Gastrointestinal Medical Oncology and Digestive Diseases, University of Texas, M.D. Anderson Cancer Center, Houston 77030, USA.

The incidence of distal esophageal adenocarcinoma and primary proximal gastric carcinoma has increased substantially in the past 15 years, particularly in North America and in some European countries. Patients with curatively resected cancer consistently have a 10 to 20% 5-year survival rate. Radiation therapy alone should not be recommended. Based on the Radiation Therapy Oncology Group/Eastern Cooperative Oncology Group (ECOG) trial in patients with predominantly squamous cell carcinoma, chemoradiotherapy (fluorouracil [5-FU]/cisplatin + 50 Gy of radiotherapy) has been shown to be superior in this setting. The most active single agents against squamous cell carcinoma are cisplatin, 5-FU, bleomycin, paclitaxel, mitomycin, mitoguazone, vinorelbine, and methotrexate. The most active agents against adenocarcinoma include paclitaxel and probably mitomycin, mitoguazone, and cisplatin. To my knowledge, there is currently no effective postoperative adjuvant therapy (chemotherapy, radiation therapy, or both). Evidence that preoperative therapy can prolong survival of patients with potentially resectable carcinoma of the esophagus is lacking. Preoperative chemoradiotherapy can result in an approximately 25% complete pathologic response of the primary tumor. Preoperative chemoradiotherapy, however, results in

substantial morbidity and even mortality. A recent single-institution, randomized study comparing surgery alone with preoperative 5-FU/cisplatin/vinblastine and concurrent radiotherapy demonstrated no difference in median survival (18 months). Nevertheless, combined-modality therapy holds promise. Multiple combined-modality strategies have been formulated and will be investigated in the next few years.

PMID: 9438700 [PubMed - indexed for MEDLINE]

242. Haematologica. 1997 May-Jun;82(3):305-8.

Primary intestinal lymphoma: clinical and therapeutic features of 32 patients.

Zinzani PL(1), Magagnoli M, Pagliani G, Bendandi M, Gherlinzoni F, Merla E, Salvucci M, Tura S.

Author information:
(1)Institute of Hematology and Oncology Seràgnoli, University of Bologna, Italy.

BACKGROUND AND OBJECTIVE: Lymphomas of the gastrointestinal tract are the most common type of primary extranodal lymphomas, accounting for 5 to 10% of all non-Hodgkin's lymphomas. In particular, primary intestinal lymphomas represent about 15-20% of gastrointestinal lymphomas. New multimodal therapeutic approaches have improved the prognosis of this once deadly disease: we report a retrospective analysis of our experience with 32 cases of primary western intestinal lymphomas, presenting clinical, therapeutical and prognostic data.
PATIENTS AND METHODS: From March 1989 to November 1995, 32 patients with untreated primary western intestinal lymphomas were submitted to radical surgery plus polychemotherapy (early stages, I and II; 22 patients), or polychemotherapy alone (advanced stage, III and IV; 10 patients). The most frequent symptoms were abdominal pain, nausea, vomiting and weight loss. The tumor was located in the jejunum in 2 cases (6.2%), in the proximal small bowel in 15 cases (46.9%), in the distal and terminal ileum in 8 cases (25%), in the colon and rectum in 4 cases (12.5%), and multiple sites were found in 3 cases (9.4%). According to histology, 26 patients had high-grade and 6 low-grade non-Hodgkin's lymphoma.
RESULTS: Stage I-II patients underwent radical resection of the tumor and chemotherapy; advanced (III-IV) stage patients were treated with chemotherapy alone as first-line approach. Of the 32 patients, 24 (75%) achieved a complete

response (CR); according to stage, all stage I-II patients had CR, while only 2 of the 10 stage III-IV patients reached CR. The risk of a lower response rate was significantly correlated with the presence of advanced stage (III-IV) (p = 0.000001). The overall 5-year survival rate was 59%, with a relapse-free survival rate of 72% among the 24 complete responders.
INTERPRETATION AND CONCLUSIONS: Intestinal lymphomas differ significantly from their gastric counterpart, not only in pathology, but also with regard to clinical features, management and prognosis. Our experience confirm the efficacy of the surgery-chemotherapy combination in obtaining a good remission rate for localized early primary intestinal lymphoma and indicates that this combination represents the only means for managing complications.

PMID: 9234576 [PubMed - indexed for MEDLINE]

243. Eur J Cardiothorac Surg. 1997 May;11(5):828-37.

Induction therapy for clinical T4 oesophageal carcinoma; a plea for continued surgical exploration.

Van Raemdonck D(1), Van Cutsem E, Menten J, Ectors N, Coosemans W, De Leyn P, Lerut T.

Author information:
(1)Department of Thoracic Surgery, University Hospitals, UZ Gasthuisberg, Leuven, Belgium. Dirk.VanRaemdonck@uz.kuleuven.ac.be

OBJECTIVE: Complete resection of a locally advanced oesophageal carcinoma is not always feasible when invading mediastinal structures. The use of induction therapy prior to surgical exploration in patients with these clinical T4 tumours is anticipated to improve the resectability rate.
METHODS: Patients, 18, who presented with a carcinoma of the thoracic oesophagus with clinical invasion into the carina (n = 6), trachea (n = 5), aorta (n = 4), lung (n = 2) and diaphragm (n = 1) were treated with concurrent chemotherapy and radiotherapy followed by surgical exploration. Follow-up was complete (mean of 17 +/- 3 months in all patients and 27 +/- 2 months in surviving patients).
RESULTS: All patients completed the induction therapy with acceptable toxicity and no mortality. Subjective improvement in dysphagia was substantial in 11 patients (in 8/11 patients (73%) however, there was still viable tumour in the

resected specimen), it was minimal in six patients and absent in one patient. Objective response on imaging was complete in one patient, partial in eight patients and minimal in nine patients [in two of these nine patients (22%) nevertheless, the primary tumour had disappeared completely in the resected specimen (pT0)]. Resection was complete (R0) in 14 patients (78%) and incomplete (R1) in one patient (5%). Resection of the primary tumour was impossible (R2) in three patients (17%) because of macroscopic airway (n = 2) and hilar (n = 1) invasion on exploration. In these three patients the tumour was bypassed using a retrosternal split stomach. One patient was proven at the time of surgery to have a previously unidentified lung metastasis. In three patients (17%), no residual tumour cells were found in the resected oesophagus nor in the lymph nodes (pT0N0M0). There have been no in-hospital deaths. Actuarial 3 year survival was 43% in all patients, 55% in completely resected patients and 100% in sterilized patients (pT0N0M0). Median survival was 18 months in all patients.
CONCLUSIONS: Chemo/radiotherapy followed by surgery in patients with a clinical T4 oesophageal carcinoma is feasible with acceptable toxicity and no treatment-related mortality. Operability and resectability rate were high (100 and 83%, respectively) compared with historical controls. The primary tumour disappeared completely (pT0N0-1M0-1) in 28%. Tumour sterilization rate was 17%. Survival looks promising compared with historical controls. Subjective neither objective response following induction therapy clearly correlated with the final pTNM staging. This indicates that, in the absence of tumour progression, neither the patient nor the treating physician should jeopardize the chance for ultimate cure by denying surgical exploration following induction therapy.

PMID: 9196296 [PubMed - indexed for MEDLINE]

244. Int J Oncol. 1996 Oct;9(4):613-7.

Phase II pilot trial of preoperative high-dose chemotherapy in patients with malignant tumors of the upper gastrointestinal tract.

Berdel W(1), Heldmann T, Germer C, Wiedenmann B, Rosewicz S, Boeselandgraf J, Karavias T, Kreuser E, Buhr H, Thiel E.

Author information:
(1)FREE UNIV BERLIN,UNIV KLINIKUM BENJAMIN FRANKLIN,ABT HAMATOL & ONKOL,D-12200 BERLIN,GERMANY. FREE UNIV BERLIN,UNIV KLINIKUM BENJAMIN FRANKLIN,CHIRURG

ABT,D-12200 BERLIN,GERMANY. FREE UNIV BERLIN,UNIV KLINIKUM BENJAMIN FRANKLIN,GASTROENTEROL ABT,D-12200 BERLIN,GERMANY.

The purpose of this trial was to test feasibility and tolerability of a multimodality treatment approach for patients with tumors in the upper gastrointestinal tract (EC, esophageal cancer; JC, cancer of the gastro-esophageal junction; GC, gastric cancer) including preoperative chemotherapy with the EAP-protocol as induction and a consecutive high-dose-chemotherapy for responding patients. Sixteen patients with locally advanced tumors of the esophagus, the gastro-esophageal junction or the stomach were treated with two cycles of EAP-chemotherapy (etoposide, 3x120 mg/m(2); adriamycin, 2x20 mg/m(2); cisplatin, 2x40 mg/m(2)). Responding (cPR, cCR) patients were included into a high-dose MCVB-chemotherapy protocol (mitomycin, 10 mg/m(2); cisplatin, 4x40 mg/m(2); vepeside, 5x200 mg/m(2); BCNU 300 mg/m(2)) and subsequent rescue with peripheral blood stem cells (PBSC). After a second restaging, surgery was performed in patients with no change or further response. Postoperative chemotherapy was given with either two cycles of EAP or FAMTX (methotrexate, 1,500 mg/m(2) + folinic acid rescue; 5-flourouracil, 1,500 mg/m(2); adriamycin, 30 mg/m(2)) according to pathological staging results. A total of 16 patients (EC, 7; JC, 6; GC 3) were treated within the protocol. Six patients achieved a major response upon EAP and 5/6 were included in the high-dose MCVB-protocol with stem cell rescue. All 5 could be yielded R(0) by definitive surgery and 2/5 had a pCR upon surgery. MCVB toxicity was predominantly hematologic (grade 4 in all 5 patients) with non-hematological toxicity not exceeding grade 2 (predominantly mucositis). Median survival time is 12 months for the non-responding patients and has not been reached for the MCVB patients. In conclusion, multimodality therapy including high-dose chemotherapy and stem cell rescue is feasible with tolerable toxicity in patients with locally advanced tumors of the upper gastrointestinal tract and should be further studied in phase II and III trials.

PMID: 21541559 [PubMed]

245. Gan To Kagaku Ryoho. 1996 Sep;23(10):1299-303.

[Neoadjuvant chemotherapy in high-grade advanced gastric cancer with protracted infusional 5-fluorouracil and consecutive low-dose cisplatin].

[Article in Japanese]

Kondo K(1), Murase M, Yokoyama Y, Kato J, Hibi K, Kasai Y, Akiyama S, Ito K, Takagi H.

Author information:
(1)Dept. of Surgery II, Nagoya University School of Medicine.

Twenty-one evaluable patients with primary gastric cancer/local invasion, liver metastasis and peritoneal metastasis were entered in a pilot study of neoadjuvant chemotherapy that used continuous 24-hour infusion 5-FU, 330 mg/m2/day plus low dose CDDP, 6 mg/m2 daily by bolus infusion d1-5. This regimen was repeated for 4 weeks. The overall response rate was 52%, including one complete and ten partial responses. The response rate of differentiated adenocarcinomas was significantly higher than that of poorly differentiated adenocarcinomas. In 15 patients (71%), gastrectomy and lymphadenectomy could be done after this regimen. chemotherapy-induced downstaging from the initial clinical stage was pathologically found in 5 patients who underwent gastrectomy. Toxicity was primarily hematologic. Leukopenia and thrombocytopenia of grade 3 or 4 occurred in 19% and 14% of patients, respectively. The patients were able to take meals during therapy and preserved good quality of life. Median survival time was 11 months for the cancers with liver metastasis and five of the 8 locally advanced cancers are alive 11 months after the therapy. This therapy was effective for patients with high-grade advanced gastric cancer.

PMID: 8831742 [PubMed - indexed for MEDLINE]

246. Semin Oncol. 1996 Jun;23(3):369-78.

Adjuvant therapy of gastric cancer: the Japanese experience.

Fukushima M(1).

Author information:
(1)Department of Internal Medicine, Aichi Cancer Center, Chikusa-ku, Japan.

The 5-year survival rate of patients with advanced gastric cancer who undergo curative resection is gradually increasing and currently ranges between 67.1% to

76.4% at the five major cancer centers in Japan. A belief that minimal residual disease has a high probability of being cured with adjuvant therapy prompted Japanese investigators to develop the D2 dissection with extended lymphadenectomy, more detailed pathologic staging, perioperative chemotherapy, and chemoimmunotherapy. This review focuses on comparative trials performed in Japan studying the use of adjuvant therapy with either chemotherapy alone or chemotherapy plus immunotherapy in the treatment of patients with curatively resected gastric carcinoma. Preoperative and intraperitoneal therapy also has been evaluated. At present, however, no chemotherapy has been shown to impact favorably on the survival of gastric cancer patients, whereas the biological response modifiers, PSK or OK-432, seem to add some benefit to chemotherapy in the adjuvant setting. Carefully designed randomized controlled trials with sufficient size which include a surgery-alone control arm are the only way to establish the efficacy of adjuvant therapy.

PMID: 8658221 [PubMed - indexed for MEDLINE]

247. Cancer. 1996 May 15;77(10):1998-2004.

Characteristics of Epstein-Barr virus-associated gastric carcinoma with lymphoid stroma in Japan.

Matsunou H(1), Konishi F, Hori H, Ikeda T, Sasaki K, Hirose Y, Yamamichi N.

Author information:
(1)Department of Pathology, Kanazawa Medical University, Ishikawa-ken, Japan.

BACKGROUND: Gastric carcinoma with lymphoid stroma (GCLS), known to have a more favorable prognosis than ordinary gastric carcinoma, has been suggested to be closely associated with the Epstein-Barr virus (EBV). However, there are many clinicopathologic problems that remain unsolved.
METHODS: In 21 patients, 26 GCLS lesions and 4 non-GCLS intramucosal adenocarcinomas that developed synchronously or metachronously with GCLS were examined for EBV involvement by in situ hybridization (ISH) and were analyzed clinicopathologically. In addition, nine patients who had advanced gastric carcinoma with massive liver metastases, who showed good response to chemotherapy and had prolonged survival, were examined for the presence or absence of EBV-associated GCLS.

RESULTS: On ISH with EBV-encoded small RNAs, diffuse hybridization signals were noted in 22 (84.6%) of 26 GCLS. Hybridization signals were also noted in all four non-GCLS adenocarcinomas accompanying GCLS. As a result, hybridization signals were noted in nine of ten cancerous lesions in four cases of synchronous multiple cancers and in all five cancerous lesions in two cases of metachronous multiple cancers. Long term survivors with liver metastases included two patients with EBV-associated GCLS.
CONCLUSION: Approximately 84.6% of GCLS were related to EBV. EBV-associated GCLS constitutes one-half of the EBV-infected stomach cancers in our institution. The complete response and long term survival after conventional chemotherapy of two patients with Stage IV GCLS suggests that this form of gastric carcinoma may be especially sensitive to this treatment. The identification of EBV-associated synchronous multicentric cancers of both GCLS and non-GCLS type suggests that EBV infection may be an early event in the induction process of these tumors.

PMID: 8640662 [PubMed - indexed for MEDLINE]

248. Sangre (Barc). 1996 Apr;41(2):109-14.

[Diagnosis and therapeutic management of gastric lymphomas].

[Article in Spanish]

Grau E(1), Perella M, Gómez A, Pastor E, Mirabet MD, García F, Ibáñez JL, Aguiló J.

Author information:
(1)Servicio de Hematología y Hemoterapia, Hospital Lluís Alcanyís, Xàtiva, Valencia.

PURPOSE: The optimal management of primary gastric non-Hodgkin's lymphoma (PGL) remains controversial. The purpose of this paper is to describe the histopathology, clinical behavior and management of 15 patients with PGL.
PATIENTS AND METHODS: All patients were diagnosed of PGL in our Center from January 1985 to September 1995. Resection specimens were reexamined and cases were reclassified according to the concept of MALT-derived lymphoma. Age, sex, symptoms, localization, stage, treatment, complications and response were reported.

RESULTS: 9 patients had low-grade B-cell lymphoma of MALT type, 5 had high-grade B-cell lymphoma (one with evidence of low-grade lymphoma of MALT type) and 1 had centroblastic-centrocytic B-cell lymphoma. Seven cases were diagnosed by endoscopy biopsies, 8 through laparatomy specimens. Stage was I in 3 cases, II1 in 5, II2 in 5, III in 0 and IV in 2. 13 patients had undergone primary gastric resection: 10 received additional chemotherapy and 1 radiotherapy. The two cases with stage IV have died: one died after surgery and one relapsed immediately after chemotherapy. After a median follow-up of 40 months, 13 patients are alive: 11 in complete response and 2 in relapse. Minor surgical complications occurred in 12 patients and major complications in 6 patients.
CONCLUSIONS: Gastric resection still plays an important role in the management of early-stage PGL. Chemotherapy can be an effective therapeutic procedure in unresected or surgical high-risk patients.

PMID: 9045350 [PubMed - indexed for MEDLINE]

249. Leuk Lymphoma. 1995 Nov;19(5-6):461-6.

Primary gastric lymphoma: a clinical and therapeutic evaluation of 82 patients.

Zinzani PL(1), Frezza G, Bendandi M, Barbieri E, Gherlinzoni F, Neri S, Baldissera A, Salvucci M, Babini L, Tura S.

Author information:
(1)Institute of Hematology L. e A. Seràgnoli, University of Bologna, Italy.

Eighty-two patients with primary gastric (IE, II1E, and II2E) non-Hodgkin's lymphoma according to the Musshoff's staging system were treated with combined modality including surgery with/without radiotherapy between January 1985 and December 1991. According to the Updated Kiel classification 54 had high-grade histologic subtypes and 28 low-grade. The strategy throughout the study was to resect primary tumor: all patients underwent gastrectomy, 40 subtotal and 42 total gastrectomy. The resection permitted complete surgical staging utilizing three pathologic features: disease confined within or beyond the serosa, negative/positive regional lymph nodes, and negative/positive surgical margins. If there was no evidence of these pathologic factors, the patients who underwent surgery alone received no further radiotherapy. On the other hand, all patients who presented at least one of three pathologic factors were treated with adjuvant

radiotherapy after the resection. All except 14 patients presented at least one of the pathologic features and 50 (61%) patients had involvement of the whole gastric wall. Radiotherapy included the gastric bed and para-aortic lymph nodes and, for the patients, who had positive regional lymph nodes in combination with the complete involvement of the gastric wall, the irradiation included the whole abdominal approach. The complete response rate was 97% and the 9-year disease-free survival was 93%. All but one of the 5 relapses occurred within 18 months stressing the need for more specific staging. Gastric resection with/without radiotherapy may still represent the primary therapeutic procedure in early stage gastric non-Hodgkin's lymphoma.

PMID: 8590847 [PubMed - indexed for MEDLINE]

250. World J Surg. 1995 Nov-Dec;19(6):836-42.

Angiotensin II (AII) induced hypertension chemotherapy (IHC) for unresectable gastric cancer: with reference to resection after down staging.

Sato H(1), Sugiyama K, Hoshi M, Urushiyama M, Ishizuka K.

Author information:
(1)Institute of Development, Aging, and Cancer, Tohoku University, Sendai, Japan.

Angiotensin II induced hypertension chemotherapy (IHC) is a drug delivery system for augmentation of anti-cancer effects on the experimental basis of the functional difference of microcirculation between tumor and normal tissue. Blood flow in tumor tissue increased selectively when the blood pressure was elevated by the infusion of angiotensin II. Two randomized controlled trials (RCT) for advanced gastric cancer using AFM regimen; a combination of adriamycin (ADM), 5-fluorouracil (5-FU), and mitomycin (MMC), showed increased response rate by IHC (response rate: IHC/non-IHC; 42.9% vs 10.5% in RCT-1, and 31.3% vs 6.7% in RCT-2, respectively). Toxicities were not different statistically between groups. In phase II for stage IVB gastric cancer patients (the criteria according to the General Rules of the Gastric Cancer Study of Japanese Research Society for Gastric Cancer), 5 complete response (CR) and 10 partial response (PR) (58%) were observed out of 26 unresectable cases without prior chemotherapy. Moreover, 5 of 15 responders could received curative gastrectomy and obtained conclusive down staging (19%). Here we discuss the role of enhancement of drug delivery for

cancer chemotherapy on the basis of a series of clinical and experimental evidences.

PMID: 8553675 [PubMed - indexed for MEDLINE]

251. Gan To Kagaku Ryoho. 1995 Nov;22(13):1953-8.

[Induction chemotherapy followed by adjuvant surgery (IC-AS) in patients with stage I-II small cell lung cancer (SCLC)].

[Article in Japanese]

Shibayama T(1), Hiyama J, Ueoka H, Tabata M, Segawa Y, Gemba K, Matsushita A, Ohnoshi T, Harada M, Andoh A, et al.

Author information:
(1)Second Dept. of Medicine, Okayama University Medical School.

Ten patients with stage I-II SCLC received IC-AS between 1984 and 1993. As induction chemotherapy, COMP-VAN alternating chemotherapy and CAV-PVP hybrid chemotherapy were administered. The former consisted of a 4-drug combination of cyclophosphamide (CPA), vincristine (VCR), methotrexate (MTX) and procarbazine alternated with a 3-drug combination of etoposide (ETP), adriamycin (ADM) and nimustine every 4 weeks. In the latter, a 3-drug combination of CPA, ADM and VCR given on day 1, and a 2-drug combination of ETP and cisplatin on day 8, were repeated every 4 weeks. All the patients had an objective response, including one complete response by induction chemotherapy. Post-operative pathology revealed SCLC in 4 patients, adenocarcinoma in 2 and no tumor (pathological CR) in 4. Four patients relapsed, and a intrathoracic relapse was experienced in only 2 patients. Six patients have died: 3 from relapsing SCLC, 2 from stomach cancer, and 1 from squamous lung cancer, who was salvaged from relapsing SCLC. The median survival time was 27.5 months, and the 3-year survival rate 37.5%. These results indicate that IC-AS is highly effective for stage I-II SCLC and warrant additional studies comparing IC-AS with chemo-radiotherapy.

PMID: 7487126 [PubMed - indexed for MEDLINE]

252. Oncology. 1995 Nov-Dec;52(6):474-82.

Prognostic evaluation of curatively resected locally advanced gastric cancer patients with preoperative downstaging chemotherapy assessed by histochemical and pharmacologic means.

Nakano H(1), Namatame K, Suzuki T, Kim J, Sasaki J, Nagasaki H, Makuuchi M, Kumada K.

Author information:
(1)Department of Surgery, Showa University Fujigaoka Hospital, Yokohama, Japan.

The aim of the present study was to investigate whether the rate of thymidylate synthetase inhibition (TSIR) and the rate of proliferating cell nuclear antigen expression (PCNA-R) in gastric cancer tissues, which can be obtained within a short period after surgery, were predictive and quantitative prognostic factors for locally advanced gastric cancer patients with preoperative down-staging chemotherapy. Curatively resected 30 locally advanced gastric cancer patients with preoperative chemotherapies were studied. Three-year survival analysis showed that the higher TSIR and the lower PCNA-R significantly predicted better prognosis ($p < 0.01$ and $p < 0.05$, respectively). Multiple regression test showed that the TSIR was a significantly predictive variable for 1-year survival ($p < 0.05$). The TSIR and PCNA-R could be predictive and quantitative prognostic factors in advanced gastric cancer patients who received preoperative downstaging chemotherapy.

PMID: 7478434 [PubMed - indexed for MEDLINE]

253. Rinsho Ketsueki. 1995 Oct;36(10):1163-9.

[Therapy related leukemia].

[Article in Japanese]

Fujisawa S(1), Maruta A, Sakai R, Ogawa K, Taguchi J, Tomita N, Kodama F, Fukawa H, Noguchi T, Matsuzaki M, et al.

Author information:

(1)Department of Hematology/Chemotherapy, Kanagawa Cancer Center.

Eleven therapy related leukemias (TRL) who were hospitalized in the Department of Hematology and Chemotherapy, Kanagawa Cancer Center between October 1983 and December 1993 were identified. Six of the patients were males and five were females. Their median age was 62 years (range from 14 to 75). Three patients had previously received treatment for breast cancer and two patients for malignant lymphoma. The other patients had received treatment for lung cancer, urinary bladder cancer, gastric cancer, brain tumor, maxillary sinus cancer and macroglobulinemia, respectively. Seven patients had been treated with chemotherapy and four patients had been treated with chemotherapy and irradiation for the primary tumor. The TRL cases consisted of 8 acute non-lymphoid leukemias, two acute lymphoid leukemias and one hypoplastic leukemia, respectively. The status of primary tumors at the development of TRL was complete remission in ten patients and partial remission in one patient. Three of the 10 patients who received anti-leukemic therapy entered complete remission and the median survival time was 36 days (from 7 days to 489 days). One patient expired of pneumonia before he received anti-leukemic therapy. TRL patients showed poor response to chemotherapy and had poor prognosis. These data suggest that the use of reduced doses of carcinogenic drugs for primary tumors might be required to prevent the development of TRL.

PMID: 8531325 [PubMed - indexed for MEDLINE]

254. J Clin Oncol. 1995 Oct;13(10):2524-9.

Efficacy of single-agent chemotherapy in low-grade B-cell mucosa-associated lymphoid tissue lymphoma with prominent gastric expression.

Hammel P(1), Haioun C, Chaumette MT, Gaulard P, Divine M, Reyes F, Delchier JC.

Author information:
(1)Service d'Hépatologie et de Gastroentérologie, Hôpital Henri Mondor, Créteil, France.

PURPOSE: The treatment of low-grade B-cell mucosa-associated lymphoid tissue (MALT) lymphoma with prominent gastric expression is controversial. Total gastrectomy has been proposed, but is associated with significant morbidity. The

aim of this monocentric study was to assess the efficacy of continuous oral chemotherapy with a single alkylating agent.
PATIENTS AND METHODS: Twenty-four consecutive patients, 13 men and 11 women, were studied. Their mean age was 51 years (range, 22 to 79). Low-grade B-cell MALT lymphoma was diagnosed by histologic and immunohistologic examination of endoscopic biopsies. Seventeen patients had stage I disease and seven stage IV disease, with lung and gastric involvement. Two of these seven patients also had bone marrow involvement. The alkylating agent (cyclophosphamide or chlorambucil) was administered orally and daily for periods of 12 to 24 months.
RESULTS: The median follow-up time was 45 months (range, 14 months to 14 years). Complete remission was obtained in 18 patients (75%) after a median treatment duration of 12 months. Five patients relapsed; two of them were successfully re-treated, and one died of MALT lymphoma that had transformed into large-cell lymphoma. Chemotherapy was stopped after 24 months for six patients who only achieved a partial remission; two of them required further treatment for progressive disease (surgery for a small-bowel localization in one case and cyclophosphamide rechallenge in the other). Nine patients had neutropenia that required a reduced chemotherapy dosage.
CONCLUSION: In low-grade MALT lymphoma with prominent gastric expression, continuous monochemotherapy may constitute an efficient alternative to gastrectomy, regardless of disease stage.

PMID: 7595703 [PubMed - indexed for MEDLINE]

255. Gastrointest Endosc Clin N Am. 1995 Jul;5(3):529-36.

Staging concepts for gastrointestinal malignancies: the importance of preoperative locoregional T- and N-staging.

Hölscher AH(1), Siewert JR, Fink U.

Author information:
(1)Department of Surgery, Technische Universität München, Bavaria, Germany.

Preoperative locoregional staging of gastrointestinal tumors is of special significance for evaluation of resectability that means complete tumor removal without residual tumor (R0-resection). This is especially important within a multimodal therapeutic concept including neoadjuvant therapy of nonresectable

tumors. In esophageal cancer above the bifurcation, tumors that are staged T3 or T4 should have neoadjuvant radiochemotherapy in order to achieve a down-staging and increase the chance for a complete tumor resection. Preoperative chemotherapy is further established in esophageal carcinomas below the bifurcation and gastric carcinomas in stage T4. As in pancreatic carcinoma, neoadjuvant treatment has no proven indication. Preoperative locoregional staging mainly concerns the infiltration of retroperitoneal veins, which represents the crucial point for resectability. Concerning carcinomas of the lower GI tract the T- and N-staging is of special relevance for rectal cancer because preoperative radiochemotherapy in T4-stage is an accepted indication. The T- and N-staging of gastrointestinal tumors has important clinical consequences; the demands for accuracy of endosonographic examinations are therefore very high.

PMID: 7582579 [PubMed - indexed for MEDLINE]

256. World J Surg. 1995 Mar-Apr;19(2):210-5.

Preoperative chemotherapy for unresectable gastric cancer.

Wilke H(1), Stahl M, Fink U, Meyer HJ, Siewert JR.

Author information:
(1)Department of Internal Medicine (Cancer Research), Essen University Medical School, West German Cancer Center.

Even with extended surgery, including systematic lymphadenectomy of the lymph node compartment II, only half of the patients with locally advanced gastric cancer (LAGC), which comprises stages IIIA, IIIB, and IV, undergo a macroscopic and microscopic tumor-free resection (i.e., R0 resection, according to UICC 1987/AICC 1988). An improvement of this situation is best accomplished by preoperative treatment modalities to increase the R0 resection rate and by preoperative and postoperative treatment to reduce local recurrences and distant metastases. For LAGC, which includes approximately two-thirds of patients with locoregionally confined tumors, preoperative chemotherapy (CTx) represents a promising approach. Among a group of patients with surgically or clinically staged unresectable LAGC, approximately half underwent R0 resection after down-staging induced by active modern CTx. The long-term survival of these patients seems to be improved. Even in patients who had primarily unresectable

tumors as defined by an explorative laparotomy, the long-term survival was about 20% after preoperative CTx and subsequent surgery. Based on these experiences, randomized trials investigating preoperative CTx versus surgery alone are clearly needed to define whether such an approach has an impact on R0 resection rates and survival of patients with LAGC. Preconditions for such trials are clinical staging procedures, including endoscopic ultrasonography (T category) and surgical laparoscopy plus lavage (excluding peritoneal carcinomatosis), and a standardized surgical procedure.

PMID: 7754625 [PubMed - indexed for MEDLINE]

257. Ann Surg Oncol. 1995 Mar;2(2):101-6.

Intensive multimodality therapy for carcinoma of the esophagus and gastroesophageal junction.

Ferguson MK(1), Reeder LB, Hoffman PC, Haraf DJ, Drinkard LC, Vokes EE.

Author information:
(1)Department of Surgery, University of Chicago, Illinois, USA.

BACKGROUND: We designed a trial of intensive multimodality therapy for carcinoma of the esophagus and gastroesophageal junction to assess tumor response and operability after neoadjuvant chemotherapy and to determine the impact of trimodality therapy on longterm survival.
METHODS: Thirty-two patients with resectable (clinical stage IIa, n = 17; IIb, n = 1; III, n = 14) squamous cell cancer (n = 15) or adenocarcinoma (n = 17) were treated with neoadjuvant chemotherapy (cisplatin, 5-fluorouracil, leukovorin), resection, and postoperative chemoradiotherapy (hydroxyurea, 5-fluorouracil; 50-66 Gy).
RESULTS: Use of neoadjuvant chemotherapy yielded the following results: a measurable clinical response in 22 patients, stable disease in eight patients, disease progression in one patient, and death in one patient. Thirty-one patients underwent resection, with the following results: two operative deaths (6.5%) and nonfatal morbidity in 17 (59%); the median hospital stay was 13 days. Pathologic staging was stage 0, n = 1; I, n = 2; IIa, n = 11; IIb, n = 5; III, n = 7; and IV, n = 5. Postoperative chemoradiotherapy was completed in 23 patients with one death, for an overall treatment-related mortality rate of 12.5% (four of 32). At

a mean follow-up of 22.5 months, median survival is 19.7 months and 14 patients are alive and disease free.
CONCLUSIONS: Neoadjuvant therapy for cancer of the esophagus and cardia results in good tumor response. Esophagectomy in this setting can be accomplished with acceptable morbidity and mortality. Results of an interim analysis of survival are encouraging and suggest that further investigation of this regimen is warranted.

PMID: 7728562 [PubMed - indexed for MEDLINE]

258. Eur J Cancer. 1995;31A(5):665-70.

Feasibility of five courses of pre-operative chemotherapy in patients with resectable adenocarcinoma of the oesophagus or gastrooesophageal junction.

Ajani JA(1), Roth JA, Putnam JB, Walsh G, Lynch PM, Roubein LD, Ryan MB, Natrajan G, Gould P.

Author information:
(1)Department of Gastrointestinal Oncology and Digestive Diseases, University of Texas M.D. Anderson Cancer Center, Houston, USA.

The purpose of this study was to examine the feasibility of administering all chemotherapy pre-operatively to patients with resectable adenocarcinoma of the oesophagus or gastrooesophageal junction. 32 patients with potentially resectable adenocarcinoma of the oesophagus or gastrooesophageal junction were studied in a stepwise fashion in which combination chemotherapy with cisplatin, high-dose arabinoside and 5-fluorouracil was administered. In the first part, 15 patients were to receive three chemotherapy courses pre-operatively and two chemotherapy courses postoperatively. In the second part, the next 15 patients were to receive all five chemotherapy courses pre-operatively, provided there was an objective response after three courses. Endoscopic ultrasonography was also performed, when feasible, prior to chemotherapy and surgery, and in some patients sequentially between chemotherapy courses. All of the 14 assessable patients in the first group tolerated all three courses of pre-operative chemotherapy, and 86% of patients in this group completed all protocol chemotherapy. In the second group, 9 of 18 (50%) assessable patients tolerated all five courses of preoperative chemotherapy, and 100% of patients in this group received all protocol

chemotherapy. The median number of chemotherapy courses for the entire group (32 patients) was five (range one to five). Forty-one per cent (13/32) of patients had a major response to chemotherapy. Sixty-nine per cent (or 76% of 29 patients taken to surgery) had a curative resection. One patient had a pathological complete response. The median survival time of 32 patients was 17 months (range 2-36+ months). 14 patients (37%) remain alive at a median follow-up time of 26+ months. There was a correlation between endoscopic ultrasonographic tumour and nodal stage and pathological tumour and nodal stages in 16 patients. The tumour stage correlation was higher (75%) than the nodal stage correlation (62%). Our data suggest that it is feasible to administer five courses of cisplatin-based chemotherapy to patients with potentially resectable adenocarcinoma of the oesophagus or gastrooesophageal junction. More effective chemotherapy regimens that might result in higher pathological complete response rates and acceptable toxic effects are needed.

PMID: 7640036 [PubMed - indexed for MEDLINE]

259. Ann Thorac Surg. 1994 Dec;58(6):1574-8; discussion 1578-9.

Evolution of treatment strategies for adenocarcinoma of the esophagus and gastroesophageal junction.

Wright CD(1), Mathisen DJ, Wain JC, Grillo HC, Hilgenberg AD, Moncure AC, Carey RW, Choi NC, Daly M, Logan DL.

Author information:
(1)General Thoracic Surgical Unit, Massachusetts General Hospital, Boston 02114.

Between 1980 and 1988, 91 patients with adenocarcinoma of the esophagus were treated by surgical resection and selective postoperative therapy. Operative mortality was 2%. Pathologic stage was I in 4, II in 26, and III in 61. Actuarial 2- and 5-year survival was 24% and 8%. From 1987 to 1989, 16 patients with adenocarcinoma of the esophagus were treated with two cycles of 5-fluorouracil and cisplatin followed by surgical resection. There was 1 complete response (6%), 5 partial responses (31%), and 10 with no response (63%). Twelve patients had resection. Pathologic stage was I in 1, II in 4, and III in 8. There was one chemotherapy-related death and one surgical death. Actuarial 4-year survival is 42%. From 1990 to 1993, 22 patients with adenocarcinoma of the esophagus were

treated with two cycles of etoposide, doxorubicin, and cisplatin followed by surgical resection. There was 1 complete response (5%), 11 partial responses (50%), and 10 with no response (45%). Eighteen patients had resection. Pathologic stage was 0 in 1, II in 8, and III in 9. There were no treatment-related deaths. The actuarial 2-year survival is 58%. Conclusions are necessarily limited because the patients were not treated in a randomized fashion. These preliminary results with preoperative chemotherapy appear improved (p = 0.04 and p = 0.004, respectively) as compared with results from 1980 to 1988 without preoperative chemotherapy.

PMID: 7979718 [PubMed - indexed for MEDLINE]

260. Ann Oncol. 1994 Dec;5(10):909-13.

Extrapulmonary small cell carcinoma: a single-institution experience and review of the literature.

Lo Re G(1), Canzonieri V, Veronesi A, Dal Bo V, Barzan L, Zancanaro C, Trovò M.

Author information:
(1)Division of Medical Oncology, Istituto Nazionale di Ricovero e Cura a Carattere Scientifico, Aviano, Italy.

BACKGROUND: Small cell carcinoma (SCC) is a distinct pathologic entity that may also occur in extrapulmonary sites. In this report the retrospective results of multimodal therapy of primitive extrapulmonary (E) SCC, in a single institution series, are presented.
METHODS: Twenty-four patients (pts) with ESCC were referred to the Centro di Riferimento Oncologico, Aviano, Italy, from 1986 to 1992. Clinico-therapeutic findings were evaluated in 20 pts. Their ages ranged from 20 to 87, with a median of 60.5 years. Primary tumor sites were urinary bladder (5 pts), prostate (4 pts), larynx (3 pts), kidney (2 pts), ovary, skin, oropharynx, trachea, uterine cervix, ethmoid, and stomach (1 pt each); lymph node metastases of unknown origin were observed in 3 pts. More than 50% of pts presented extensive disease.
RESULTS: Histologically, 16 cases were pure ESCCs and 8 cases were combined, 4 of them with adenocarcinoma, 2 with transitional cell carcinoma, and 2 with squamous cell carcinoma. Immunohistochemical studies, performed in 7 cases, demonstrated the epithelial nature of these tumors. The cisplatin-VP16 (PE) regimen was used

in 13 pts, and 9 of them (69%) obtained objective responses after chemotherapy (CT) alone, with 3 complete remissions (CR) and 6 partial remissions (PR). Median CR and PR duration was 13+ and 24 months, respectively. Radiotherapy was performed in 7/13 pts after induction CT and before consolidation CT. The objective response rate was 100%, with 6 CR and 1 PR. No severe toxic side effects and no toxic deaths were reported. A patient treated with surgery alone for a urinary bladder tumor showed continuous long-term survival, while 1 of 2 pts treated with radiotherapy alone obtained PR.
CONCLUSIONS: The PE regimen has an activity similar to the one observed in pulmonary SCC.

PMID: 7696162 [PubMed - indexed for MEDLINE]

261. Clin Ter. 1994 May;144(5):425-30.

[Continuous infusion of doxorubicin for 10 days as third-line chemotherapy in metastasized breast tumors. Initial observations].

[Article in Italian]

Garcia-Giralt E(1), Omodei Zorini C, Ferri L, Jouve M, Dorval T, Beuzeboc P, Palangie T, Livartowski A, Scholl S, Pouillart P.

Author information:
(1)Istituto Curie, Servizio di Medicina Oncologica, Parigi.

The authors undertook a study to test the feasibility and efficacy of doxorubicin (Dox) administered as continuous infusion (c.i.) in the treatment of advanced breast cancer patients. All patients were previously treated as first line chemiotherapy with bolus-administered Dox; then they received a second line treatment without Dox. Patients were eligible if they had advanced measurable breast cancer, resistant to first and second line chemotherapy regimens, and if previous Dox treatment had been performed more than one year before, and after ECG and cardiac echographic controls were performed. A dose of 4.5 mg/m2/day of Dox was planned for 10 consecutive days in c.i. through a central venous catheter, repeated at 28 day intervals, for a maximum of 10 cycles. Among the 71 patients, 56 received 3 or more treatment courses; 10 patients refused further therapy after the first cycle. Objective responses were achieved in 25 patients

(35%), 3 complete and 22 partial remissions. Stabilization was obtained in 11 patients (16%). The median time to progression was 9.3 months. Obviously, haematological toxicity was the major problem; in the 71 patients, 376 courses were administered (mean number of courses per patient: 5.3): grade 4 neutropenia (WHO system) occurred in 2 patients, and grade 3 in 9 patients; while one patient died of gastric haemorrhage. Severe cardiac toxicity occurred in only one patient who died 24 hours after the beginning of therapy, with ECG lateral ischemia. Grade 4 stomatitis was observed in only one patient.(ABSTRACT TRUNCATED AT 250 WORDS)

PMID: 7924181 [PubMed - indexed for MEDLINE]

262. Surg Oncol. 1994 Apr;3(2):115-25.

Primary gastric lymphomas: a clinicopathologic study with literature review.

Roukos DH(1), Hottenrott C, Encke A, Baltogiannis G, Casioumis D.

Author information:
(1)Universitaetsklinik fuer Allgemeinchirurgie, Klinikum der Johann-Wolfgang-Goethe Universitaet, Frankfurt am Main, Germany.

Prognostic factors and treatment results were analysed in 28 consecutive patients with primary gastric lymphoma (PGL) diagnosed and treated, all by surgery and in many cases with additional chemotherapy (CT) and/or radiotherapy (RT), between 1977 and 1988. There were 13 patients in stage IE, 5 in IIE, and 10 in stage IV. The resection rate was 96.4% (27/28). Sixteen patients underwent an extended total and 11 a subtotal gastrectomy. Seventeen out of 25 cases (68%) were diagnosed by endoscopic biopsies. In 10 endoscopically diagnosed PGL cases the clinical staging and separation between stages IE and IIE from stage IV, due to ultrasonographic scan, computed tomography and bone marrow biopsy, was correct and the same with the surgical-pathological staging information. According to the Kiel-classification 18 patients had a low-grade and 9 patients a high-grade lymphoma. One patient could not be classified. All patients were completely followed-up, in an average time of 52 months. The probability of overall 5-year survival was 92% in stage IE, 75% in stage IIE, 88% in stages IE+IIE together, and 35% in stage IV. Extent of surgery (total vs. subtotal gastrectomy), Kiel-classification (low-grade vs. high-grade malignant histologic subtypes) and

adjuvant CT in patients with stage IE (all 11 patients without CT remain in complete remission after an average of 45 months) did not significantly influence survival. The sole prognostic factor with proven impact on survival was the stage of disease (IE+IIE vs. IV: P = 0.001). For the Kiel-classification in particular there was no significant difference between low-grade and high-grade lymphomas with regard to the sex, symptomatic, extent of surgery, and stage at operation. These findings, together with data from the literature, suggest that gastric resection seems to be the optimal primary treatment in clinically assessed stages IE or IIE. In patients with stage IE disease, surgical resection can result in a cure, with no need for further therapy. The CT and/or RT can be effective in unresected and even bulky cases. Because of the difference in primary treatment, a preoperative clinical staging and separation between early stages from stage IV is always indicated.

PMID: 7952391 [PubMed - indexed for MEDLINE]

263. Am J Clin Oncol. 1994 Feb;17(1):14-8.

Adenocarcinoma of the esophagus and gastroesophageal junction. Clinical and pathologic assessment of response to induction chemotherapy.

Adelstein DJ(1), Rice TW, Boyce GA, Sivak MV, Van Kirk MA, Kirby TJ, van Stolk RU, Bukowski RM.

Author information:
(1)Department of Hematology, Cleveland Clinic Foundation, Ohio 44195.

A preoperative induction chemotherapy regimen consisting of two monthly courses of etoposide, doxorubicin, and cisplatin was given to 13 patients with nonmetastatic adenocarcinoma of the distal esophagus or gastroesophageal junction. Esophageal ultrasound examination was performed both before chemotherapy and again before surgery. Induction chemotherapy was poorly tolerated with 10 of the 13 patients experiencing at least one episode of severe neutropenia. Two of the 13 patients refused the second course of treatment. A symptomatic response to chemotherapy, defined as a reduction in the presenting symptom, was noted in 10 of the 13 patients (77%). Endoscopic improvement occurred in 9 of the 13 patients (69%). Esophageal ultrasound evidence of a reduction in either T or N stage was noted in only 2 of the 13 patients (15%),

however, and neither of these responses was confirmed pathologically. Clinical evidence of disease progression was noted in 4 patients during chemotherapy. With a median follow-up of 31 months, the relapse-free and overall survivals are 25% and 31%, respectively. Despite significant toxicity, our chemotherapy regimen would be considered successful if assessed by symptomatic or esophagoscopic improvement. Esophageal ultrasound, careful pathologic staging, and our disappointing survival rates, however, suggest limited, if any, value for this approach.

PMID: 8311001 [PubMed - indexed for MEDLINE]

264. Ann Oncol. 1994;5 Suppl 3:59-68.

Preoperative chemotherapy of locally advanced gastric cancer.

Rougier P(1), Lasser P, Ducreux M, Mahjoubi M, Bognel C, Elias D.

Author information:
(1)Institut Gustave-Roussy, Villejuif, France.

Gastric adenocarcinomas, even in the absence of distant metastases, have a poor prognosis which is particularly dismal when tumors are located in the cardia, in the event of locoregional lymph node involvement and/or bulky tumors. Postoperative adjuvant chemotherapy has never clearly demonstrated its efficacy on survival. Besides ongoing trials using new and more active regimens, preoperative chemotherapy has been used for unresectable cancer due to loco-regional extension and when locally advanced cancer is potentially resectable but with poor prognosis such as bulkiness, when tumors are located in the cardia and when there is tumor in the coeliac area at CAT-scan with suspected metastatic lymph nodes. In case of unresectable tumor at initial surgery five publications have reported the ability of chemotherapy to reduce the tumor volume and to allow subsequent resection of the gastric tumor in 40% to 60% of the cases. In these cases there is a clear survival advantage as the median survival reported in 2 of these studies was 12 and 18 months compared to the 4 to 6 months median survival reported in historical studies in case of unresectable cancer [17, 18]. In case of locally advanced gastric tumors some Japanese case reports have demonstrated the ability of preoperative chemotherapy to concentrate in the tumor tissue and to downstage the tumors. Four North American and European

studies have demonstrated that preoperative chemotherapy is feasible, and will probably increase the resection rate. J. Ajani has reported 2 studies in which tolerance was acceptable: a major response (R) observed in 24% and 31%, the resectability rates were 72% and 77% and the median survival 15 and 16 months, respectively. Our experience is based on 30 patients treated with a combination of continuous i.v. 5-FU and CDDP. Fifteen had a tumor of the cardia, 15/30 had enlarged lymph nodes and 7/30 a linitis plastica (diffuse type). After a mean number of 3 cycles, 27/30 patients were evaluable for response. One patient achieved a CR and 14 a PR (OR rate 56%, 95% CI: 38% to 74%) but only one of those with linitis plastica responded. Twenty-eight patients underwent surgery and 23 had a macroscopically complete resection (82%). Resectability rate was higher after OR (13/15) than in nonresponding patients (4/12). Toxicity was acceptable, however grade 4 leucopenia in 5 patients and one toxicity-related death were observed. There was no increase in postoperative complications. Nine patients received postoperative chemotherapy and 3 patients with positive margins received postoperative external radiotherapy.(ABSTRACT TRUNCATED AT 400 WORDS)

PMID: 8204531 [PubMed - indexed for MEDLINE]

265. Eur J Cancer. 1994;30A(9):1269-75.

Neoadjuvant chemotherapy in locally advanced gastric carcinoma--a phase II trial with combined continuous intravenous 5-fluorouracil and bolus cisplatinum.

Rougier P(1), Mahjoubi M, Lasser P, Ducreux M, Oliveira J, Ychou M, Pignon JP, Elias D, Bellefqih S, Bognel C, et al.

Author information:
(1)Gastro-Intestinal Unit, Institut Gustave Roussy, Villejuif, France.

Locally advanced gastric adenocarcinomas (LAGC) have a poor prognosis, particularly when tumours are bulky, located in the cardia or in the event of locoregional lymph node involvement. Patients bearing these tumours were entered in a phase II trial of neoadjuvant chemotherapy, combining continuous intravenous 5-fluorouracil (5FU) (1000 mg/m2 for 5 days) and cisplatinum (CDDP) (100 mg/m2 on day 2) repeated every 4 weeks, for one to six cycles according to response and tolerance. 30 patients have been entered, 26 after clinical evaluation (CAT scan and upper gastrointestinal endoscopy) and 4 with unresectable tumours at prior

laparotomy. Median age was 60 years, 15/30 patients had a tumour of the cardia, 15/30 had enlarged lymph nodes and 7/30 had linitis plastica (diffuse type). A mean number of three cycles was administered (range 1-6). 27 of the 30 patients were evaluable for response. One patient achieved a complete response (CR) and 14 a partial response (56%; 95% confidence interval 38-74%). No patient had tumour progression, and only 1/6 with linitis plastica responded. 28 patients underwent surgery, and 23 had a macroscopically complete resection (77% of the 30 entered patients); R0 resections were performed in 60% of the cases, mainly after an objective response (13/15 versus 4/12 in nonresponders). No pathological CR were seen. Grade 4 neutropenia was observed in eight cycles (5 patients), with five septic complications and one death due to toxicity. Four postoperative complications were observed: 2 cases of severe pneumonia and 2 subphrenic abscesses. One postoperative death, due to intravascular disseminated coagulation, was observed at day 30. Median survival was 16 months and the 1-, 2- and 3-year survival was 67, 42 and 38%, respectively. Patients with linitis plastica had a significantly shorter survival ($P < 0.002$). We conclude that neodjuvant chemotherapy is feasible in LAGC, although randomised trials are warranted to demonstrate its efficacy on survival and resection rates.

PMID: 7999411 [PubMed - indexed for MEDLINE]

266. Ann Surg Oncol. 1994 Jan;1(1):5-10.

Preoperative high-dose radiation and chemotherapy in adenocarcinoma of the esophagus and esophagogastric junction.

Sauter ER(1), Coia LR, Keller SM.

Author information:
(1)Department of Surgery, Fox Chase Cancer Center, Philadelphia, Pennsylvania 19111.

BACKGROUND: Esophageal adenocarcinoma (EA) incidence is rising. Defining optimal management is essential because median survival after surgery alone is only approximately 12 months. High-dose radiation (> 5000 cGy) and chemotherapy (HDRCT) preoperatively for patients with EA has not been fully investigated. We evaluated tumor response, resectability, and survival following HDRCT in patients with localized EA.

METHODS: Thirty patients with American Joint Committee on Cancer (AJCC) clinical stage I or II EA were prospectively treated with HDRCT. The treatment consisted of 60 Gy radiation at 2 Gy per fraction with concurrent infusional 5-fluorouracil (5-FU) and a bolus of mitomycin C followed by esophagogastrectomy. The range of follow-up was 7 to 69 months, with a median of 31 months.
RESULTS: Twenty of 30 patients (67%) received full-course HDRCT. Severe esophagitis precluded full-dose radiation in 10 patients. Three patients developed neutropenia and fever requiring admission to a hospital. Two patients died preoperatively of treatment-related complications. Nine patients were not explored. Eighteen patients were resected with curative intent; the remaining three had metastatic disease at laparotomy. Seven of 18 resected patients (39%), or 7/30 (23%) of all patients treated, had a pathologic complete response. There was one operative death. Overall local control was seen in 25/30 patients (83%). Median overall survivals for resected and for all patients were 23 and 13 months, respectively.
CONCLUSIONS: Preoperative HDRCT in patients with EA results in encouraging local tumor response and local control. Overall survival, however, may not be improved, and the treatment-related mortality of 10% is higher than reported with surgery alone or with preoperative chemotherapy.

PMID: 7834428 [PubMed - indexed for MEDLINE]

267. J Natl Cancer Inst. 1993 Nov 17;85(22):1839-44.

Preoperative and postoperative combination chemotherapy for potentially resectable gastric carcinoma.

Ajani JA(1), Mayer RJ, Ota DM, Steele GD, Evans D, Roh M, Sugarbaker DJ, Dumas P, Gray C, Vena DA, et al.

Author information:
(1)University of Texas M. D. Anderson Cancer Center, Houston 77030.

BACKGROUND: Median survival of patients with local-regional gastric carcinoma is 10 months. Resection of the primary tumor and regional lymph nodes, with tumor-free margins (curative resection), has been the most effective treatment for local-regional gastric carcinoma. However, median survival of patients with curative resection of gastric carcinoma is 24 months, and the 5-year survival

rate is about 20%. A single institution pilot study has established the feasibility of administering two courses of chemotherapy preoperatively and three courses postoperatively. In another study, a 15% pathologically documented complete response (pathologic complete response) has been reported in unresectable gastric carcinoma treated with etoposide, doxorubicin, and cisplatin.
PURPOSE: Our purpose was to increase the curative resection rate in potentially resectable gastric carcinoma and to delay or eliminate micrometastases and thus improve survival. We also evaluated clinical and pathologic response to chemotherapy.
METHODS: Forty-eight previously untreated patients with potentially resectable gastric carcinoma received a chemotherapy regimen (EAP) consisting of etoposide (120 mg/m2 intravenously over a 2-hour period on days 4, 5, and 6), doxorubicin (20 mg/m2 as a 10-minute intravenous infusion on days 1 and 7), and cisplatin (40 mg/m2 as a 1-hour intravenous infusion on days 2 and 8). Patients received three courses of chemotherapy before resection, and responding patients received two courses postoperatively. Clinical and pathologic response rates, toxicity, patterns of treatment failure, and survival times were assessed.
RESULTS: A median of three courses (range, 1-5) of preoperative therapy was administered; six (12%) of the 48 patients had clinical complete response, and nine (19%) had partial response. Forty-one (85%) underwent surgery; 37 (90%) of these 41 (77% of the 48 patients) had a curative resection. There were no pathologic complete responses. Median survival for all patients is 15.5 months (range, 2-29+ months). Therapy was discontinued because of the toxic effects in one patient before surgery and in six patients after surgery. Doses were reduced in 37 patients (77%), mainly because of hematologic toxicity. Nineteen (40%) were hospitalized because of toxic effects, including 15 patients who developed fever with neutropenia. Grade 3 or 4 nausea and vomiting occurred in 15 patients and grade 3 or 4 diarrhea in seven patients. One death was directly related to chemotherapy.
CONCLUSIONS: These data support that administration of preoperative and postoperative chemotherapy for local-regional gastric carcinoma is feasible in a multi-institutional setting. Our findings demonstrate that this EAP regimen is modestly active but is associated with substantial toxicity.
IMPLICATIONS: Use of preoperative chemotherapy in resectable gastric carcinoma merits further evaluation, but more effective drug regimens will be required before a controlled trial is initiated.

PMID: 8230264 [PubMed - indexed for MEDLINE]

268. Minerva Chir. 1993 Apr 15;48(7):325-30.

[Case histories of extranodal non-Hodgkin's lymphomas with primary gastrointestinal appearance].

[Article in Italian]

Buda F(1), Buda C, La Torre F, Simon G, Binotto F, Bellomo R, Russo F, Croatto T, Duchi M, Mandoliti G.

Author information:
(1)Ospedale Civile S. Maria dei Battuti, Regione Friuli-Venezia Giulia--USL 9, Sanvitese.

Authors refer results obtained in 24 cases of primary NHL of gastrointestinal tract. These cases were observed in the period 1981-1990. All cases can be included as primary extranodal lymphomas satisfying criteria of the literature. Sixty-six per cent (16/24) had a gastric localization half cases were centrocytic-centroblastic lymphomas followed by immunoblastic, centroblastic, follicular centrocytic-centroblastic and lymphocytic-well-differentiated. In intestinal localisation (34% of cases), the most represented was lymphocytic poor-differentiated, followed by mixed centrocytic lymphoma. Therapeutic strategy was: surgery in all patients followed by chemotherapy (CHOP 14/24) or by radiotherapy plus chemotherapy (8/24). Surgery alone was adopted in 2 out 24 patients. Patients who received radiotherapy plus chemotherapy had a sandwich treatment (CVP = 4/8, CHOP = 4/8) consisting of splint course of 3 cycles followed by radiotherapy and completed with other 3 drugs cycles. Sixteen out 24 patients were valuable (4 patients were lost during the follow-up due to problems other than the disease; 4 patients are still under treatment). In the 16 valuable patients we had a complete remission (CR) with a median free-disease survival of 82 months (range: 12-116 months) by means of the primary treatment. Four out 16 patients relapsed. In these patients the free-disease survival was range 8-108 months. All patients reached a second CR by means of chemotherapy (CCNU + VIP16) or radiotherapy. No cases of second tumor insorgence was observed.

PMID: 8327178 [PubMed - indexed for MEDLINE]

269. J Clin Oncol. 1993 Jan;11(1):22-8.

Intensive preoperative chemotherapy with colony-stimulating factor for resectable adenocarcinoma of the esophagus or gastroesophageal junction.

Ajani JA(1), Roth JA, Ryan MB, Putnam JB, Pazdur R, Levin B, Gutterman JU, McMurtrey M.

Author information:
(1)Department of Medical Oncology, University of Texas M.D. Anderson Cancer Center, Houston 77030-4095.

Comment in
J Clin Oncol. 1993 Jan;11(1):1-2.

PURPOSE: The curative resection rate in patients with potentially resectable carcinoma of the esophagus is approximately 55% and their median survival time is 11 months. Preoperative chemotherapy with high doses of chemotherapeutic agents was used to evaluate clinical and pathologic responses, curative resection rate, toxicity, and survival. Colony-stimulating factor (CSF) was added to reduce the severity of myelosuppression.
PATIENTS AND METHODS: Twenty-six consecutive assessable patients with potentially resectable adenocarcinoma of the esophagus or gastroesophageal junction were treated with two preoperative courses of intensive chemotherapy (etoposide, doxorubicin, and cisplatin [EAP]) with granulocyte-macrophage CSF (GM-CSF). Additional three conventional-dose postoperative chemotherapy courses without GM-CSF were given to patients who responded to preoperative chemotherapy.
RESULTS: A median of three courses (range, one to six), were administered. Of 27 patients, 26 were assessable for response to preoperative EAP; 13 (50%) achieved a major response. Among 23 patients who underwent surgery, 15 (65%) had a curative resection (58% of 26 assessable patients); none of the patients had a pathologic complete response, but two patients had only microscopic carcinoma in the resected specimen. Six patients had carcinoma present at the resection margins and received postoperative radiotherapy. Two patients were found to have liver metastases at exploration. At a median follow-up of 22 months, the median survival of 26 patients was 12.5 months (range, 2 to 32 +). Fourteen patients died of their carcinoma; two patients died of treatment-related causes; one died of an unrelated CNS arterial malformation; and the causes of death in two

patients remain unknown. Seven patients are alive with no evidence of relapse. Major toxicities of this regimen included severe myelosuppression, nausea and vomiting, infections, and severe constitutional symptoms related to GM-CSF. However, subcutaneous injection of GM-CSF was well tolerated.
CONCLUSION: High-dose EAP is active against locoregional adenocarcinoma of the esophagus and gastroesophageal junction but can be associated with significant toxicity. Although this strategy remains attractive and needs to be developed further, less toxic and more effective regimens need to be identified.

PMID: 8418237 [PubMed - indexed for MEDLINE]

270. J Clin Oncol. 1992 Dec;10(12):1933-42.

Preoperative systemic chemotherapy followed by adjuvant postoperative intraperitoneal therapy for gastric cancer: a University of Southern California pilot program.

Leichman L(1), Silberman H, Leichman CG, Spears CP, Ray M, Muggia FM, Kiyabu M, Radin R, Laine L, Stain S, et al.

Author information:
(1)University of Southern California School of Medicine and Medical Center, Los Angeles.

PURPOSE: A clinical trial for patients with gastric cancer amenable to curative resection was undertaken to determine feasibility and response to preoperative systemic chemotherapy followed by postoperative intraperitoneal (IP) chemotherapy.
METHODS AND MATERIALS: Thirty-eight patients with resectable gastric tumor received two cycles of protracted intravenous (IV)-infusion fluorouracil (5FU), 200 mg/m2/d, for 3 weeks with weekly IV leucovorin 20 mg/m2 and IV cisplatin 100 mg/m2 days 1 and 29. Resection of the gastric tumor followed within 3 weeks of completion of systemic chemotherapy. Those who had all visible tumor removed with clear margins received two cycles of IP floxuridine 3,000 mg (total dose) per day for 3 days and IP cisplatin 200 mg/m2 with IV sodium thiosulfate on the fourth day of IP therapy.
RESULTS: Thirty-seven of 38 patients (97%) received two cycles of systemic chemotherapy. Thirty-five of 38 patients (92%) underwent laparotomy for gastric

tumor resection. Thirty-three patients (87%) had gastric resections performed; 29 (76%) had all visible tumor removed with microscopically negative margins. No operative mortality was encountered. Twenty-six patients (68%) received IP treatment. IV neoadjuvant treatment was well tolerated and resulted in 68% of the patients reporting improvement in abdominal pain, 45% objective remissions by computed tomography (CT), 38% objective remissions by gastroscopy and biopsy, and 8% had complete surgical pathologic response. Neutropenic sepsis during the IP treatment phase contributed to the only treatment-related death. Four of 29 completely resected patients (14%) have had tumor recurrence. The median follow-up time of patients remaining alive is now 19 months. The median survival for 38 patients entered onto this protocol has not been reached at 17+ months.
CONCLUSION: This novel approach to the treatment of adenocarcinoma of the stomach is feasible. The neoadjuvant systemic therapy results in significant primary tumor regression. The determination of whether systemic or IP components of the program contribute to decreased recurrence or increased survival awaits a prospectively randomized clinical trial.

PMID: 1453207 [PubMed - indexed for MEDLINE]

271. Gan To Kagaku Ryoho. 1992 Nov;19(13):2235-7.

[A five-year-survival case in which complete response was recognized after combined chemotherapy using 5-fluorouracil, adriamycin and mitomycin C (FAM) for unresectable gastric cancer].

[Article in Japanese]

Iwase K(1), Takenaka H, Sumimura J, Ishizaka T, Takagaki M, Bessho T, Ohata T, Inoue M, Oshima S, Tanaka T.

Author information:
(1)Department of Surgery, Social Insurance Kinan General Hospital, Tanabe, Japan.

FAM (5-fluorouracil, adriamycin, mitomycin C) therapy was performed on a 65-year-old man with unresectable gastric cancer. Cancer cells have not been recognized by endoscopic biopsy after the patient's complete response. He is alive without metastasis of recurrence for five years.

PMID: 1444491 [PubMed - indexed for MEDLINE]

272. Cancer. 1992 Nov 1;70(9):2239-45.

Effects of preoperative chemotherapy on gastric adenocarcinomas. A morphologic study of 25 cases.

Kiyabu M(1), Leichman L, Chandrasoma P.

Author information:
(1)Department of Pathology, University of Southern California School of Medicine, Los Angeles.

BACKGROUND: Surgical neoadjuvant therapy for gastric adenocarcinoma affords the opportunity to evaluate critically the histologic effects of preoperative chemotherapy.
METHODS: Morphologic alterations in gastric adenocarcinomas were examined in the surgical-resection specimens from 25 patients after 6 weeks of preoperative chemotherapy. The group included 1 patient with a complete response; 4, with subtotal responses; 4, with partial responses; and 16, with no response to preoperative chemotherapy.
RESULTS: Histologic manifestations of preoperative chemotherapy included mucosal edema, aggregates of histiocytes in the submucosa and muscularis externa, and stromal fibrosis of the submucosa and muscularis externa. Cytologic manifestations were uncommon and included a single case of signet ring cell carcinoma with diminution of the cytoplasmic vacuoles after preoperative chemotherapy. Clinical follow-up was limited, but 3 of the 25 patients died within 5-8 months after the diagnosis of gastric adenocarcinoma. The gastric-resection specimens from these three patients did not show any histologic manifestations of preoperative chemotherapy.
CONCLUSION: As in tumors at other sites, the efficacy of surgical neoadjuvant therapy for gastric adenocarcinoma can be assessed, based on the histologic response of the resected tumor to preoperative chemotherapy.

PMID: 1327491 [PubMed - indexed for MEDLINE]

273. Gan To Kagaku Ryoho. 1992 Feb;19(2):203-9.

[Preoperative role of angiotensin II induced hypertension chemotherapy (IHC) in advanced gastric carcinoma].

[Article in Japanese]

Sugiyama K(1), Sato H, Ishizuka K, Hoshi M, Urushiyama M, Wakui A.

Author information:
(1)Department of Clinical Cancer Chemotherapy, Tohoku University, Sendai, Japan.

To investigate the role of preoperative IHC in advanced gastric carcinoma, clinical, surgical and pathological stagings of 13 patients were analysed retrospectively. These patients were treated with a 3-drug combination of adriamycin, 5-fluorouracil and mitomycin C under angiotensin II induced hypertensive state. The response rate was 69.2% and mean survival time was 850.2 days. "Down staging" in surgical stage was observed in 5 out of 13 cases (38.3%), and in pathological stage 7 cases (53.8%) "down staging" was achieved. Mean survival time of "pathological down-staging" cases was significantly longer than that of "non-down-staging" cases (1039.6 vs 322.1 days, p less than 0.01, generalized Wilcoxon test). IHC brings selective increase of drug delivery to tumor tissue, and will be useful as preoperative chemotherapy in advanced gastric carcinoma in terms of "down staging".

PMID: 1736832 [PubMed - indexed for MEDLINE]

274. Jpn J Surg. 1991 Sep;21(5):556-60.

Advanced gastric cancer associated with acute myelocytic leukemia--report of a case.

Konno H(1), Ida K, Sakaguchi S, Aoki K, Baba S, Ihara M.

Author information:
(1)Second Department of Surgery, Hamamatsu University School of Medicine, Japan.

A case of advanced gastric cancer associated with acute myelocytic leukemia (AML) is reported. Synchronous double malignancies of gastric cancer and AML are very

rare. Combination chemotherapy (BHAC-DMP) was used as the method for induction and consolidation therapy for AML and a complete remission was obtained. However, it failed to show any therapeutic effect on the gastric cancer. A radical subtotal gastrectomy was performed with lymphadenectomy. During the postoperative course, both respiratory failure and severe thrombocytopenia progressed. Fortunately, the patient responded well to mechanical ventilation and the administration of heparin. She was discharged on day 52 after surgery, and no sign of recurrence of either gastric cancer or AML has been observed over the one-year period following the gastrectomy. In principle, in order to achieve a good prognosis, a radical resection should be carried out for gastric cancer associated with AML. However, chemotherapy for AML might make the patient vulnerable to surgical stress, although we could not demonstrate any concrete evidence which could prove the impairment of host immunity in this case. It is, therefore, possible that not only the relapse of AML but also the impairment of host immunity may cause some other difficulties during the post-gastrectomy course.

PMID: 1813693 [PubMed - indexed for MEDLINE]

275. Cancer. 1991 Jul 15;68(2):227-32.

Treatment of neuroendocrine carcinomas with combined etoposide and cisplatin. Evidence of major therapeutic activity in the anaplastic variants of these neoplasms.

Moertel CG(1), Kvols LK, O'Connell MJ, Rubin J.

Author information:
(1)Department of Oncology, Mayo Clinic, Rochester, Minnesota 55905.

Forty-five patients with metastatic neuroendocrine tumors were treated with a regimen of etoposide 130 mg/m2/d for 3 days plus cisplatin 45 mg/m2/d on days 2 and 3. Both drugs were given by continuous intravenous infusion. Among 27 patients with well-differentiated carcinoid tumors or islet cell carcinomas, only two partial objective tumor regressions were observed (7%). Among 18 patients prospectively classified as having anaplastic neuroendocrine carcinomas, however, there were nine partial regressions and three complete regressions, an overall regression rate of 67%. For anaplastic disease, the median duration of regression

was 8 months (range to 21 months). Tumor response was unrelated to primary site, endocrine hyperfunction, or prior therapy experience. The median survival of all patients with anaplastic tumors was 19 months; this seemed favorable when considering the small experiences with these rare tumors reported in the literature. Toxicity, which was severe for most patients, consisted primarily of vomiting, leukopenia, thrombocytopenia, anemia, alopecia, and neuropathy. The anaplastic neuroendocrine tumor is strongly responsive to therapy with combined etoposide and cisplatin. Patients with undifferentiated carcinomas, originating in typical neuroendocrine tumor sites (small and large bowel, pancreas, and stomach) or of unknown origin, who have consistent histologic findings by light microscopy should be evaluated for this possibility with appropriate immune staining or electron microscopy.

PMID: 1712661 [PubMed - indexed for MEDLINE]

276. Tumori. 1991 Apr 30;77(2):160-3.

Mitomycin-C, adriamycin, 5-fluorouracil and leucovorin (L-FAM2) in the treatment of advanced gastric cancer: a phase II study.

Zaniboni A(1), Simoncini E, Marpicati P, Meriggi F, Arcangeli G, Garattini P, Raffaglio E, Ferragni A, Marini G.

Author information:
(1)III Divisione di Medicina Generale, Spedali Civili, Brescia, Italy.

Thirty previously untreated patients with advanced measurable gastric cancer were given a combination chemotherapy consisting of 5-fluorouracil, 400 mg/m2, and leucovorin, 200 mg/m2 iv on days 1 to 3, mitomycin-C, 10 mg/m2 on day 1 (every other cycle) and adriamycin, 40 mg/m2 on day 2, repeated every 21 days. The overall response rate was 46% (14/30; 95% confidence limits: 28%-64%) including 4 patients with a complete remission. Eight patients progressed. Median duration of remission (CR+PR) was 10 months, with a median survival of 13, 8 and 4 months for CR+PR, NC and PD, respectively. Main toxicities were leukopenia (WHO grade III-IV in 36% of the patients) and alopecia. One patient died from myocardial infarction after an adriamycin cumulative dose of 480 mg/m2. No other treatment-related death occurred. L-FAM2 is an effective combination for advanced gastric carcinoma. Further studies based on the association of leucovorin and

5-fluorouracil in combination with other active drugs are warranted.

PMID: 2048229 [PubMed - indexed for MEDLINE]

277. J Clin Oncol. 1990 Jul;8(7):1231-8.

Evaluation of pre- and postoperative chemotherapy for resectable adenocarcinoma of the esophagus or gastroesophageal junction.

Ajani JA(1), Roth JA, Ryan B, McMurtrey M, Rich TA, Jackson DE, Abbruzzese JL, Levin B, DeCaro L, Mountain C.

Author information:
(1)Department of Medical Oncology, University of Texas MD Anderson Cancer Center, Houston TX 77030-4096.

Thirty-five consecutive patients with resectable adenocarcinoma of the esophagus or gastroesophageal junction were treated with two preoperative and three or four postoperative chemotherapy courses consisting of etoposide, fluorouracil, and cisplatin (EFP) to evaluate the rate of curative resection, clinical and pathologic response, toxic effects, and survival. One hundred thirty-seven courses with a median number of five courses (range, one to six) were administered. Preoperative EFP resulted in 17 (49%) major responses, including six patients who did not have carcinoma cells in the repeat endoscopic biopsy specimens and cytologic brushings. Among 32 patients who had surgery, 25 (78%) had curative resection, one patient had a complete pathologic response, and one had microscopic carcinoma in the resected specimen. Six patients had microscopic carcinoma at the resection margins and received postoperative radiotherapy. At a median follow-up of 20 months, the projected survival of 35 patients is 23 months (range, 6 to 33+). Fifteen patients died of their carcinomas, and 15 patients were alive (median follow-up, 20+ months; range, 15+ to 33+ months) with no evidence of relapse. There were no deaths related to chemotherapy, surgery, or radiotherapy. EFP-induced toxic reactions were moderate. Our data suggest that multiple courses of EFP are feasible. Future strategies for this disease should consider prolonged chemotherapy with regimens that result frequently in pathologic complete responses.

PMID: 2358838 [PubMed - indexed for MEDLINE]

278. Yonsei Med J. 1990 Mar;31(1):74-9.

Locally advanced unresectable gastric cancer successfully resected after neoadjuvant chemotherapy with FADE regimen.

Chung HC(1), Roh JK, Park YJ, Lee SI, Min JS, Lee JT, Lee KB, Kim BS.

Author information:
(1)Department of Internal Medicine, Yonsei University College of Medicine, Seoul, Korea.

The prognosis of unresectable advanced gastric cancer is extremely poor. We tried a neoadjuvant chemotherapy in locally advanced unresectable stomach cancer diagnosed by initial explo-laparotomy. After chemotherapy with the FADE regimen (5-fluorouracil + adriamycin + cisplatin + etoposide), the patient was diagnosed clinically as a complete response state on re-staging with radiological gastrointestinal study, fiber-gastroscopy and computerized tomography. During the second-look operation, the advanced cancer was completely resected and the pathological diagnosis was early gastric cancer (EGC) type IIc, stage II (T1N2Mo).

PMID: 2346043 [PubMed - indexed for MEDLINE]

279. Semin Oncol. 1990 Feb;17(1 Suppl 2):61-70.

New developments in the treatment of gastric carcinoma.

Wilke H(1), Preusser P, Fink U, Achterrath W, Meyer HJ, Stahl M, Lenaz L, Meyer J, Siewert JR, Geerlings H, et al.

Author information:
(1)Department of Hematology/Oncology, Hannover University Medical School, FRG.

The recent successes being achieved with combination chemotherapy regimens, such as FAMTX (fluorouracil [5-FU], doxorubicin, methotrexate), EAP (etoposide, doxorubicin, cisplatin), and ELF (etoposide, leucovorin, 5-FU), strongly indicate

that gastric cancer is chemosensitive. With these regimens, objective remission rates of more than 50% were recorded, including approximately 10% complete remissions (CRs). Moreover, some of these CRs were histopathologically confirmed. The finding that locally advanced disease (LAD) and technically unresectable disease could be rendered resectable by preoperative chemotherapy (EAP) was important. Thirty-six patients with LAD had been treated in a phase II trial with preoperative EAP, inducing 24 (70%) overall remissions (two clinical CRs, six pathologic CRs, 16 partial remissions [PRs] in 35 evaluable patients. Twenty-one patients were disease-free after chemotherapy with or without second-look surgery. The median survival time was 18 months for all patients and 24 months for disease-free patients. At 30+ months, 21% of all patients are still living disease-free. The expected survival of patients with unresectable LAD is approximately 4 to 6 months without any treatment and 6 to 9 months with standard chemotherapy. Compared with the latter results, the preoperative use of effective regimens (eg, EAP) seems to improve prognosis of patients with LAD. Moreover, such a multimodal approach may increase the number of long-term survivors among patients with resectable gastric cancer, especially those whose stage indicates a high risk of relapse (stages IIIa or IIIb). However, partly because of the severe toxicities (myelosuppression, nausea/vomiting), a considerable number of patients cannot be treated with these new regimens for the following reasons: Two of three patients with gastrointestinal disease are older than 60 years. Nontumorous diseases of the cardiovascular system, kidney, and others are frequent in this age group and may complicate or even prevent treatment with aggressive regimens. Considering the predominantly palliative treatment intentions in far advanced (metastasized) gastric cancer, regimens with low toxicities and acceptable activity should be preferred. For these reasons, we developed and investigated the combination ELF in a phase II trial in elderly patients (greater than 65 years) and in patients with cardiac risks who could not be treated with anthracyclines. The overall response rate in 51 evaluable patients was 53% (27 of 51) including six clinical CRs (12%). The median remission duration was 9.5 months and the median survival time was 11 months. Tolerability was excellent. Only 16% and 4% of patients, respectively, experienced WHO grades 3 and 4 leukopenia. Nausea/vomiting and mucositis/stomatitis were mild.(ABSTRACT TRUNCATED AT 400 WORDS)

PMID: 2305269 [PubMed - indexed for MEDLINE]

280. J Clin Oncol. 1989 Sep;7(9):1318-26.

Preoperative chemotherapy in locally advanced and nonresectable gastric cancer: a phase II study with etoposide, doxorubicin, and cisplatin.

Wilke H(1), Preusser P, Fink U, Gunzer U, Meyer HJ, Meyer J, Siewert JR, Achterrath W, Lenaz L, Knipp H, et al.

Author information:
(1)Department of Hematology/Oncology, Hannover University, Medical School, West Germany.

Thirty-four patients with locally advanced, nonresectable gastric cancer (staged by laparotomy) received etoposide, Adriamycin (doxorubicin; Adria Laboratories, Columbus, OH), and cisplatin (EAP). Thirty-three patients were evaluable for response and toxicity. Second-look surgery with removal of residual tumor by gastrectomy and lymphadenectomy was performed in case of complete/partial remission (CR/PR) after EAP. After successful resection (R0- and R1-resection), two cycles of EAP were administered for consolidation therapy. Patients refusing reoperation received up to six cycles of EAP. The response rate (CR/PR) after EAP was 70% (23/33), including a 21% (7/33) rate of clinical CRs (CCRs). Two patients had minor remission (MR)/no change and seven had progressive disease. There was one early death. Nineteen of 23 responders (5 CCRs, 14 clinical PRs [CPRs]) and one patient with MR underwent second-look surgery. Five CCRs were pathologically confirmed; 10 patients with CPR were without evidence of disease (NED) after resection. In three patients (CPR), R1-resections (microscopically tumor-cell positive proximal margin) were performed; two patients are disease-free, 22+ and 33+ months after consolidation chemotherapy. In two patients, the tumor was again considered nonresectable. Twenty patients were disease-free after EAP +/- surgery +/- consolidation chemotherapy. Toxicity was primarily hematologic. Leukopenia and thrombocytopenia of World Health Organization (WHO) grade 3 occurred in 30% and 9%, respectively and grade 4 in 18% and 9% of the patients, respectively. There was no increased peri- or postoperative morbidity. After a median follow-up of 20 months for disease-free patients, the relapse rate is 60% (12/20). The median survival time for all patients is 18 months and for disease-free patients 24 months. EAP is highly effective in locally advanced gastric cancer, and offers a chance for surgery with curative intention in patients with an otherwise fatal prognosis.

PMID: 2769330 [PubMed - indexed for MEDLINE]

281. J Clin Oncol. 1989 Sep;7(9):1310-7.

Phase II study with the combination etoposide, doxorubicin, and cisplatin in advanced measurable gastric cancer.

Preusser P(1), Wilke H, Achterrath W, Fink U, Lenaz L, Heinicke A, Meyer J, Meyer HJ, Buente H.

Author information:
(1)Department of Surgery, University Clinics, Muenster, West Germany.

In this phase II multicenter trial, 67 evaluable patients with advanced measurable gastric carcinoma were treated with a combination of etoposide, Adriamycin (doxorubicin; Adria Laboratories, Columbus, OH), and cisplatin (EAP). The overall response rate was 64%, including 21% complete responses (CRs). In 55 patients with metastatic disease, 31 responses (51%) including eight CRs (15%) were achieved. Responses were seen in all metastatic sites, but the response rate was lower in patients with peritoneal carcinomatosis. In 12 patients with locoregional disease, six CRs and six partial responses (PRs) were observed. Eight CRs (three and five in patients with metastatic and locoregional disease, respectively) were pathologically confirmed. The overall median response duration was 7 months; it was 16 months for patients achieving CR (22 months for pathologically confirmed CR [pCR]), and 6 months for PR. The median survival time for all patients was 9 months, for the patients who achieved CR 17 months, for pCR 23 months, and for PR 9.5 months. Median survival time for all patients with metastatic disease was 8 months, and for locoregional disease 12.5 months. Six patients (9%) (four local, two metastatic disease) were alive at 2 years, and four patients are alive and disease free at 35+ to 56+ months. Main toxicities were leukopenia and thrombocytopenia, with 64% of patients developing grade 3 to 4 myelosuppression and 12% severe infections. Nonhematologic toxicities of World Health Organization (WHO) grade 4 were not observed.

PMID: 2671287 [PubMed - indexed for MEDLINE]

282. Gan To Kagaku Ryoho. 1989 Aug;16(8 Pt 2):2936-9.

[Arterial infusion chemotherapy in patients with gastric cancer in liver metastasis and long-term survival after treatment].

[Article in Japanese]

Kitamura M(1), Arai K, Miyashita K, Kosaki G.

Author information:
(1)Dept. of Surgery, Tokyo Metropolitan Komagome Hospital.

Fifty-five gastric cancer patients with liver metastasis received arterial infusion chemotherapy. In 14 patients who had lesions in the liver intra-hepatic artery infusion chemotherapy was performed, while in 41 patients who had lesions in the liver and other sites intra-aortic infusion chemotherapy was performed. Methods for inserting the catheter into the aorta or hepatic artery and treatment schedules were reported previously. Between 1975 to 1981, 33 gastric cancer patients with liver metastasis were treated with 5-FU only (4 cases). MMC.5-FU (18 cases) and ADM.5-FU (11 cases). No response was seen, but minor response was seen in two cases. Between 1982 to 1988, 22 gastric cancer patients with liver metastasis were treated using arterial MMC.5-FU therapy combined with angiotensin II (13 cases), arterial MMC therapy combined with degradable starch microsphere (6 cases) and sequential MTX.5-FU (3 cases). The response rate of MMC.5-FU therapy combined with angiotensin II was 5/13 (38%) including one complete response. The responders of MMC-combined DSM therapy were seen in 3 (50%) out of 6 patients. However, no response was seen in sequential MTX.5-FU therapy. In the present study, a 61-year-old patient treated with MMC.5-FU combined with angiotensin II therapy, survived for 49 months after treatment. In order to improve the prognosis of gastric cancer patients with liver metastasis, it is important to increase the delivery of anticancer drugs to the target tissues by using certain drugs like angiotensin II and DSM. In the future, further studies should be done to prolong the duration of remission by arterial chemotherapy.

PMID: 2506831 [PubMed - indexed for MEDLINE]

283. Wien Med Wochenschr. 1988 Jul 15;138(13):323-30.

[Adjuvant chemotherapy in cancers of the gastrointestinal tract (GIT)].

[Article in German]

Obrecht JP(1).

Author information:
(1)Onkologischen Abteilung, Departements für Innere Medizin, Universität Basel.

A critical review of adjuvant therapies of gastrointestinal tumors shows that this is a field of active clinical investigation. Unfortunately the results that have been achieved until now have not fulfilled the expectations. Neoadjuvant therapies of squamous cell carcinomas of the esophagus and the anus in many cases lead to pathologically documented complete remissions. After a 10 years follow-up, it is evident that for anal carcinoma such pathological complete remissions translate into longterm remissions and eventually cures. The same conclusion, however, can not yet be drawn for esophageal cancer. The role of adjuvant chemotherapy in gastric carcinoma remains unclear. The results of the current FAM-studies are eagerly awaited. In pancreatic cancer the combination of radiotherapy and 5-FU may probably prolong disease-free survival and survival for a limited time period. Many adjuvant studies have been done in colonic cancer, without any conclusive data regarding the role of chemotherapy. Some indications point to biological response modifiers, immuno- and regional therapies to be effective in the adjuvant situation. The results of adjuvant chemotherapy in rectal cancer after radiotherapy or radiochemotherapy are easier to judge. They show that some patients profit from adjuvant therapy. But the most efficient combinations and the role of newer modalities remain to be elucidated (Fig. 8 a-8c).

PMID: 3055691 [PubMed - indexed for MEDLINE]

284. J Thorac Cardiovasc Surg. 1988 Mar;95(3):415-22.

Improved results of surgical treatment for esophageal and gastroesophageal junction carcinomas after preoperative combined chemotherapy and radiation.

MacFarlane SD(1), Hill LD, Jolly PC, Kozarek RA, Anderson RP.

Author information:
(1)Section of General, Thoracic, and Vascular Surgery, Virginia Mason Medical

Center, Seattle, Wash.

Combined treatment with chemotherapy and radiation (chemoradiation) preceding surgical exploration for esophageal or gastroesophageal squamous cell carcinoma or adenocarcinoma was compared with surgical exploration alone to determine if there was an influence on tumor status at exploration, tumor resectability, disease recurrence, and patient survival. Preoperative chemoradiation resulted in significant tumor response as measured by decreased nodal involvement and 36% incidence of no residual tumor at resection (total response) and was reflected by an improvement in resectability. Local tumor recurrence was eliminated by preoperative chemoradiation preceding resection. Distant recurrence was not reduced and remained the major cause of death. The 2-year survival rate after tumor resection alone was 33% versus 66% after preoperative chemoradiation and resection ($p = 0.13$). Patient survival after resection alone was predicted by pathologic extent of local disease as measured by lymph node status. In contrast, survival after chemoradiation and resection was not predicted by pathologic extent of local disease. Surgical resection appears to have been an important component of therapy, primarily because survival was improved in patients after resection of residual local disease.

PMID: 3343850 [PubMed - indexed for MEDLINE]
