# Supplementary material for: Correlation of pathological complete response with survival after neoadjuvant chemotherapy in gastric or gastroesophageal junction cancer treated with radical surgery: A meta-analysis
Source: PLoS One. 2018 Jan 25;13(1):e0189294. doi: 10.1371/journal.pone.0189294 (PMC5784899; doi:10.1371/journal.pone.0189294)
Supplement: S5 File — (PDF) [file pone.0189294.s006.pdf]

Records

1. **The impact of persistent dysphagia (PD) after induction chemotherapy in patients with locoregionally advanced (LRA) adenocarcinoma (ACA) of the esophagus and gastroesophageal junction (E/GEJ) receiving a trimodality treatment regimen**

McNamara M.J., Rybicki L.A., Rodriguez C.P., Videtic G.M.M., Stephans K.L., Greskovich J., Sohal D., Murthy S.C., Raja S., Ives D.I., Bodmann J., Adelstein D.J.

Journal of Clinical Oncology 2015 **33:3 SUPPL. 1**

Embase

Abstract

Background: For patients with LRA ACA of the E/GEJ who receive pre-operative therapy, advanced pathologic stage at surgery is strongly associated with recurrence and death. Identification of such patients prior to resection, however, is problematic. Given the morbidity of esophageal resection, alternative treatment strategies may be desirable in this patient population. Methods: Between 2/08 and 1/12, 60 evaluable patients with LRA ACA of the E/GEJ enrolled in single arm phase II trial of induction chemotherapy, surgery, and post-operative chemoradiotherapy. A clinical stage of T3, N1 or M1a (AJCC 6th) was required for eligibility. Induction chemotherapy with epirubicin 50mg/m2 d1, oxaliplatin 130mg/m2 d1, and fluorouracil 200mg/m2/day continuous infusion for 3 weeks, was given every 21 days for 3 courses and was followed by surgical resection. Adjuvant CRT consisted of 50-55Gy @ 1.8-2.0 Gy/d and 2 courses of cisplatin (20mg/m2/d) and fluorouracil (1000mg/m2/d) given as 96 hour infusions during weeks 1 and 4 of radiotherapy. Dysphagia was assessed at baseline and after induction chemotherapy. Updated results as of March 2014 are presented. Results: Of the 60 evaluable patients enrolled, 54 completed induction therapy and underwent surgery. Of these 54 patients, 44 patients experienced complete resolution of dysphagia, while 10 patients had persistent symptoms. PD was associated with worse distant metastatic control [HR 3.48 (1.43 - 8.43), p=0.006], recurrence free survival [HR 3.04 (1.34 - 6.92), p=0.008], and overall survival [HR 3.31 (1.43 - 7.66), p=0.005]. PD was associated with more advanced pathologic T-descriptor (pT) (p=0.048) and N-descriptor (pN) (p=0.002), a greater median

number of involved lymph nodes (3 v 1, p=0.003), and greater residual tumor viability (p=0.05). No patients with PD had pT0-T2 or pN0 disease. Of the 9 patients with pN3 disease, 5 (56%) had PD. Conclusions: PD after induction chemotherapy is associated with more advanced pathologic stage and inferior outcomes. These patients may require alternative treatment strategies.

Other works by authors of this record

McNamara M.J., Rybicki L.A., Rodriguez C.P., Videtic G.M.M., Stephans K.L., Greskovich J., Sohal D., Murthy S.C., Raja S., Ives D.I., Bodmann J., Adelstein D.J.

Emtree drug index terms

adjuvant, cisplatin, epirubicin, fluorouracil, oxaliplatin

Emtree medical index terms

adenocarcinoma, arm, chemoradiotherapy, continuous infusion, death, **digestive system cancer**, **dysphagia**, **esophagus**, esophagus resection, **human**, **induction chemotherapy**, infusion, **lower esophagus sphincter**, lymph node, morbidity, neoplasm, overall survival, **patient**, phase 2 clinical trial, population, radiotherapy, recurrence free survival, surgery, therapy

Author Address

M.J. McNamara.

Additional Information

Abbreviated Journal Title	J. Clin. Oncol.
ISSN	0732183X
Source Type	Journal
Conference Name	2015 Gastrointestinal Cancers Symposium
Conference Location	San Francisco, CA, United States
Conference Date	2015-01-15 to 2015-01-17
Source Publication Date	2015-01-20
Entry Date	2015-04-10 (Full record)
Publication Type	Conference Abstract
Page Range	
Language of Article	English
Language of Summary	English

Related articles

View related articles on Embase.com

Copyright

Copyright 2015 Elsevier B.V., All rights reserved.

Records

2. **The effect of neoadjuvant chemotherapy (NACT) on lymph node retrieval in gastroesophageal carcinoma: A comparison of two cohorts treated at a single institution**

Felismino T.C., Silva M.J.D.B.E., Fanelli M.F., Da Costa W.L., José F., Coimbra F., Mello C.L.

Journal of Clinical Oncology 2015 **33:3 SUPPL. 1**

Embase

Abstract

Background: Surgery for gastric cancer (GC) has evolved over time. D2-lymphademectomy with a minimum of 16 nodes is the current approach in many countries. In locally advanced GC, surgery alone has high rates of recurrence. Hence, adjuvant therapies are needed. Adjuvant chemoradiotherapy (CRT) and perioperative chemotherapy (PCT) are standard. The effect of PCT causing histological changes and tumor regression is clear. However it is unknown if PCT could change the number of dissected nodes due to tumor response and histological modification. Our aim is to evaluate patients treated with gastrectomy followed by CRT or PCT and surgery and define the impact of PCT in the number of nodes dissected. Methods: We retrospectively analyzed two historical cohorts treated at our institution. Cohort 1 (C1) underwent upfront gastrectomy and CRT. Cohort 2 (C2) underwent PCT and gastrectomy. Results: C1 had 51 patients. Median age was 54y. 60.8% male and 39.2% female. There were 88% primary gastric and 12% esophagastric junction (EGJ). Pathologic stages II or III were described in 92.2%. Median follow-up was 65 m. C2 had 66 patients. Median age was 58y. 70.1% male and 29.9% female. There were 71.6% primary gastric and 28.4% EGJ. cT3 was described in 82% and cN+ in 77.4%. Main chemotherapy regimens were DCF in 35.8%, EOX in 23.9% and CF in 13.4%. At surgery, 67.1% had stages II or III. Six patients had pathologic complete response. Three patients died due to toxicity. Median follow-up time was 34 m. Median number of dissected nodes was 35 in C1 and 31 in C2 (p=0.08). 90.2% in C1 and 91% in C2 had more then 16 nodes dissected. Median number of positive nodes

was four in C1 and one in C2 (p=0.01). Regarding chemotherapy, DCF or a non-taxane regimen had the same median of 31 nodes dissected. 3-year recurrence free survival was 78.3% for C1 and 66% for C2 (p=0.21). 28.3% of patients recurred in C1 and 35% in C2, mainly in distant sites. Only two patients in each group had local failure. Conclusions: Although NACT induces histological changes, it does not reduce the number of retrieved lymph nodes at surgery. Moreover, PCT is associated with a lower median number of positive nodes, related to tumor downstaging.

Other works by authors of this record

Felismino T.C., Silva M.J.D.B.E., Fanelli M.F., Da Costa W.L., José F., Coimbra F., Mello C.L.

Emtree drug index terms

ajulemic acid

Emtree medical index terms

adjuvant chemoradiotherapy, **adjuvant chemotherapy**, adjuvant therapy, **carcinoma**, chemotherapy, **digestive system cancer**, female, follow up, gastrectomy, human, **lymph node**, male, neoplasm, patient, recurrence free survival, stomach cancer, surgery, toxicity, tumor regression

Author Address

T.C. Felismino. Hospital A.C. Camargo.

Additional Information

Abbreviated Journal Title	J. Clin. Oncol.
ISSN	0732183X
Source Type	Journal
Conference Name	2015 Gastrointestinal Cancers Symposium
Conference Location	San Francisco, CA, United States
Conference Date	2015-01-15 to 2015-01-17
Source Publication Date	2015-01-20
Entry Date	2015-04-10 (Full record)
Publication Type	Conference Abstract
Page Range	
Country of Author	Brazil
Language of Article	English
Language of Summary	English

Related articles

View related articles on Embase.com

Copyright

Copyright 2015 Elsevier B.V., All rights reserved.

Records

3. **Retrospective analysis of preoperative chemoradiation therapy for esophageal and gastroesophageal junction (GEJ) cancer: Lessons to be learned from expanding inclusion criteria in this patient population**

Iafolla M.A.J., Spratlin J.L., Ghosh S., Mulder K.E.

Journal of Clinical Oncology 2015 **33:3 SUPPL. 1**

Embase

Abstract

Background: Preoperative chemoradiation therapy utilizing paclitaxel, carboplatin and radiation as per the CROSS Group increased survival rates among patients with potentially curable esophageal or GEJ cancer. A retrospective chart review was performed to determine: 1) the frequency of patients meeting the original trial inclusion/exclusion criteria 2) overall survival (OS), disease free survival (DFS), and pathological response rates (RR) of this patient population. Methods: Data was collected on 89 patients who received preoperative chemoradiation therapy (CROSS protocol) in Edmonton, Alberta between June 1, 2010 and April 11, 2014. Mean and standard deviation were calculated for continuous data and frequency (proportion) for categorical data. Time to event was presented using Kaplan-Meier estimates and log rank tests were used to compare the KM curves. Results: The median age of the entire patient population was 62 years with 77 (86.5%) of the patients being male. Twenty-three (26%) patients met inclusion criteria (MIC), whereas 66 (74%) patients failed to meet inclusion criteria (FMIC). Reasons for FMIC included: clinical stage (39.3%); weight loss > 10% (39.0%); previous cancer history (22.5%); tumor length (9.0%); and age (5.6%). Pathological complete response (CR) occurred in 24% of all patients with a trend to increased CR in MIC vs FMIC (35% vs 20%; p=0.18). Significant improvement in DFS was observed in the MIC group compared to FMIC (p=0.004). Although a trend to improvement was seen for OS in the MIC group, it is not yet significant at this early analysis (p=0.12). Conclusions: A significant proportion

of patients treated with neoadjuvant chemoradiation therapy for esophageal and GEJ cancer at our center did not meet the inclusion/exclusion criteria utilized in the CROSS trial. This had a detrimental impact on FMIC patients questioning its benefit and utility in a broader patient population.(table present).

Other works by authors of this record

Iafolla M.A.J., Spratlin J.L., Ghosh S., Mulder K.E.

Emtree drug index terms

carboplatin, paclitaxel

Emtree medical index terms

Canada, **chemoradiotherapy**, **digestive system cancer**, disease free survival, **human**, Kaplan Meier method, log rank test, **lower esophagus sphincter**, male, medical record review, minimum inhibitory concentration, **neoplasm**, overall survival, **patient**, **population**, radiation, survival rate, **therapy**, weight reduction

Author Address

M.A.J. Iafolla.

Additional Information

Abbreviated Journal Title	J. Clin. Oncol.
ISSN	0732183X
Source Type	Journal
Conference Name	2015 Gastrointestinal Cancers Symposium
Conference Location	San Francisco, CA, United States
Conference Date	2015-01-15 to 2015-01-17
Source Publication Date	2015-01-20
Entry Date	2015-04-10 (Full record)
Publication Type	Conference Abstract
Page Range	
Language of Article	English
Language of Summary	English

Related articles

View related articles on Embase.com

Copyright

Copyright 2015 Elsevier B.V., All rights reserved.

Records

4. **Safety, Efficacy, and Long-Term Follow-Up Evaluation of Perioperative Epirubicin, Cisplatin, and Capecitabine Chemotherapy in Esophageal Resection for Adenocarcinoma**

Van Der Sluis P.C., Ubink I., Van Der Horst S., Boonstra J.J., Voest E.E., Ruurda J.P., Borel Rinkes I.H.M., Wiezer M.J., Schipper M.E.I., Siersema P.D., Los M., Lolkema M.P., Van Hillegersberg R.

[Article in Press] **Annals of Surgical Oncology** 2015

Embase

Go to publisher for the full text

Abstract

Background: Perioperative epirubicin, cisplatin, and capecitabine (ECC) chemotherapy was evaluated in patients who underwent esophageal resection for adenocarcinoma of the esophagus or gastroesophageal junction (GEJ).Methods: A cohort of 93 consecutive patients was analyzed. The median follow-up period was 60 months. Source data verification of adverse events was performed by two independent observers.Results: All three planned preoperative chemotherapy cycles were administered to 65 patients (69.9 %). Only 27 % of the patients completed both pre- and postoperative chemotherapy. The reasons for not receiving postoperative adjuvant chemotherapy could be separated in two main problems: toxicity of the preoperative chemotherapy and postoperative problems involving difficulty in recovery and postoperative complications. Finally, 25 patients (27 %), completed three preoperative and three postoperative cycles. Grades 3 and 4 nonhematologic adverse events of preoperative chemotherapy mainly consisted of thromboembolic events (16.2 %) and cardiac complications (7.5 %). A history of cardiac and vascular disease was independently associated with discontinuation of preoperative chemotherapy and the occurrence of grade 3 or higher adverse events. Surgery was performed for 94 % of all the patients who started with ECC chemotherapy. A radical resection (R0) was achieved in 93 % of the patients. A complete pathologic response was observed in 8 % of the patients. During a median follow-up period of 60 months, the median disease-free survival time was 28 months, and the median overall survival time was 36 months. The 3-year overall survival rate was 50 %, and the 5-year overall

survival rate was 42 %.Conclusion: For patients with adenocarcinoma of the esophagus or GEJ, six cycles of ECC-based perioperative chemotherapy is associated with a relatively high number of adverse events. Although this toxicity did not affect the esophageal resectability rate, this regimen should be used with caution in this patient population.

Other works by authors of this record

Van Der Sluis P.C., Ubink I., Van Der Horst S., Boonstra J.J., Voest E.E., Ruurda J.P., Borel Rinkes I.H.M., Wiezer M.J., Schipper M.E.I., Siersema P.D., Los M., Lolkema M.P., Van Hillegersberg R.

Emtree drug index terms

capecitabine, **cisplatin**, **epirubicin**

Emtree medical index terms

adenocarcinoma, adjuvant chemotherapy, **chemotherapy**, disease free survival, esophagus, **esophagus resection**, **follow up**, human, lower esophagus sphincter, overall survival, patient, population, postoperative complication, radical resection, **safety**, surgery, survival rate, survival time, thromboembolism, toxicity, vascular disease

Author Address

P.C. Van Der Sluis. Department of Surgery, G04.228, University Medical Center Utrecht.

Additional Information

Abbreviated Journal Title	Ann. Surg. Oncol.
ISSN	15344681 (electronic), 10689265
CODEN	ASONF
Source Type	Journal
Source Publication Date	2015-01-07
Entry Date	2015-01-20 (Article in Press)
Publication Type	Article in Press
Page Range	
Country of Author	Netherlands
Country of Source	United States
Language of Article	English
Language of Summary	English

Embase Accession Number 2015665453

Related articles

View related articles on Embase.com

Copyright

Copyright 2015 Elsevier B.V., All rights reserved.

Records

5. **NeoFLOT: Multicenter phase II study of perioperative chemotherapy in resectable adenocarcinoma of the gastroesophageal junction or gastric adenocarcinoma-Very good response predominantly in patients with intestinal type tumors**

Schulz C., Kullmann F., Kunzmann V., Fuchs M., Geissler M., Vehling-Kaiser U., Stauder H., Wein A., Al-Batran S.-E., Kubin T., Schäfer C., Stintzing S., Giessen C., Modest D.P., Ridwelski K., Heinemann V.

[Article in Press] **International Journal of Cancer** 2015
Embase

Go to publisher for the full text

Abstract

Perioperative treatment is a standard of care in locally advanced gastroesophageal cancer (GEC) (gastric adenocarcinoma and gastroesophageal junction (GEJ) adenocarcinoma). While preoperative treatment can be applied to the majority of patients, postoperative chemotherapy can be given only to a fraction. The NeoFLOT-study therefore investigates the application of prolonged neoadjuvant chemotherapy (NACT). Patients with T3, T4, and/or node-positive adenocarcinoma (GEC) were eligible for this multicenter phase II trial. NACT consisted of 6 cycles of oxaliplatin 85 mg/m^2, leucovorin 200 mg/m^2, 5-fluorouracil 2600 mg/m^2 and docetaxel 50 mg/m^2 (FLOT) applied q 2 wks. Application of adjuvant chemotherapy was explicitly not part of the protocol. R0-resection rate was evaluated as a primary endpoint. Of 59 enrolled patients, 50 patients underwent surgery and were assessable for the primary endpoint. R0-resection rate was 86.0% (43/50). Pathologic complete response (pCR) was 20.0% (10/50) and a further 20% (10/50) of patients achieved near complete histological remission (<10% residual tumor). Among these

very good responders, 85% (17/20) had intestinal type tumors, 10% (2/20) had diffuse and 5% (1/20) had mixed type tumors. After 3 cycles of NACT, 6.9% (4/58) of patients developed progressive disease. Median disease-free survival was 32.9 months. The 1-year survival-rate was 79.3%. Grade 3-4 toxicities included neutropenia 29.3%, febrile neutropenia 1.7%, diarrhea 12.1% and mucositis 6.9%. This study indicates that intensified NACT with 6 cycles of FLOT is highly effective and tolerable in resectable GEC. Very good response (pCR and <10% residual tumor) was predominantly observed in patients with intestinal type tumors.

Other works by authors of this record

Schulz C., Kullmann F., Kunzmann V., Fuchs M., Geissler M., Vehling-Kaiser U., Stauder H., Wein A., Al-Batran S.-E., Kubin T., Schäfer C., Stintzing S., Giessen C., Modest D.P., Ridwelski K., Heinemann V.

Emtree drug index terms

docetaxel, fluorouracil, folinic acid, oxaliplatin

Emtree medical index terms

adenocarcinoma, **adjuvant chemotherapy**, **chemotherapy**, diarrhea, disease free survival, febrile neutropenia, health care quality, **human**, **lower esophagus sphincter**, mucosa inflammation, **neoplasm**, neutropenia, **patient**, **phase 2 clinical trial**, preoperative treatment, remission, **stomach adenocarcinoma**, **stomach cancer**, **surgery**, survival rate, toxicity

Author Address

V. Heinemann. Department of Medical OncologyKlinikum Grosshadern and Comprehensive Cancer Center, University of MunichMunich Germany

Additional Information

Abbreviated Journal Title	Int. J. Cancer
ISSN	10970215 (electronic), 00207136
CODEN	IJCNA
Source Type	Journal
Source Publication Date	2015-01-01
Entry Date	2015-03-04 (Article in Press)
Publication Type	Article in Press
Page Range	
Country of Source	United States

Language of Article	English
Language of Summary	English
Embase Accession Number	2015784197

Related articles

View related articles on Embase.com

Copyright

Copyright 2015 Elsevier B.V., All rights reserved.

Records

6. **Preoperative treatment with radiochemotherapy for locally advanced gastroesophageal junction cancer and unresectable locally advanced gastric cancer**

Ratosa I., Oblak I., Anderluh F., Velenik V., But-Hadzic J., Ermenc A.S., Jeromen A.

[In Process] **Radiology and Oncology** 2015 **49:2 (163-172)**

Embase

Go to publisher for the full text

Abstract

Background. To purpose of the study was to analyze the results of preoperative radiochemotherapy in patients with unresectable gastric or locoregionally advanced gastroesophageal junction (GEJ) cancer treated at a single institution. Patients and methods. Between 1/2004 and 6/2012, 90 patients with locoregionally advanced GEJ or unresectable gastric cancer were treated with preoperative radiochemotherapy at the Institute of Oncology Ljubljana. Planned treatment schedule consisted of induction chemotherapy with 5-fluorouracil and cisplatin, followed by concomitant radiochemotherapy four weeks later. Three-dimensional conformal external beam radiotherapy was delivered by dual energy (6 and 15 MV) linear accelerator in 25 daily fractions of 1.8 Gy in 5 weeks with two additional cycles of chemotherapy repeated every 28 days. Surgery was performed 4-6 weeks after completing radiochemotherapy. Following the surgery, multidisciplinary advisory team reassessed patients for the need of adjuvant chemotherapy. The primary endpoints were histopathological R0 resection rate and pathological response rate. The secondary endpoints were toxicity of preoperative radiochemotherapy and survival.

Results. Treatment with preoperative radiochemotherapy was completed according to the protocol in 84 of 90 patients (93.3%). Twenty patients (22.2%) did not undergo the surgery because of the disease progression, serious comorbidity, poor performance status or still unresectable tumour. In 13 patients (14.4%) only exploration was performed because the tumour was assessed as unresectable or diffuse peritoneal carcinomatosis was established. Fifty-seven patients (63.4%) underwent surgery with the aim of complete removal of the tumour. Radical resection was achieved in 50 (55.6%) patients and the remaining seven (7.8%) patients underwent non-radical surgery (R1 in five and R2 in two patients). In this group of patients (n = 57), pathological complete response of tumour was achieved in five patients (5.6% of all treated patients or 8.8% of all operated patients). Down-staging was recorded in 49 patients (86%), in one patient (1.8%) the stage after radiochemotherapy was unchanged while in seven patients (12.3%) the pathological stage was higher than clinical, mainly due to higher pN stage. No death was recorded during preoperative radiochemotherapy. Most grade 3 and 4 toxicities were due to vomiting, nausea and bone marrow suppression (granulocytopenia). Twenty-six (45.6%) patients died due to GEJ or gastric carcinoma, one died because of septic shock following the surgery and a reason for two deaths was unknown. Twenty-eight patients (49.1%) were disease free at the time of analysis, while 29 patients (50.9%) developed the recurrence, mostly as distant metastases. At two years, locoregional control, disease-free survival, disease-specific survival and overall survival were 82.9%, 43.9%, 56.9% and 53.9%, respectively. Conclusions. Preoperative radiochemotherapy was feasible in our group of patients and had acceptable toxicity. Majority of patients achieved down-staging, allowing greater proportion of radical resections (R0), which are essential for patients' cure.

Other works by authors of this record

Ratosa I., Oblak I., Anderluh F., Velenik V., But-Hadzic J., Ermenc A.S., Jeromen A.

Emtree drug index terms

cisplatin, fluorouracil

Emtree medical index terms

adjuvant chemotherapy, bone marrow suppression, carcinomatosis, **chemoradiotherapy**, chemotherapy, comorbidity, death, disease course, disease free survival, disease specific survival, distant metastasis, external beam radiotherapy, granulocytopenia, human, induction chemotherapy, linear accelerator, **lower esophagus sphincter**, nausea, **neoplasm**, oncology, overall survival, patient,

preoperative treatment, radical resection, septic shock, staging, stomach cancer, stomach carcinoma, surgery, survival, toxicity, vomiting

Author Address

I. Oblak. Department of Radiotherapy, Institute of Oncology Ljubljana.

Additional Information

Abbreviated Journal Title	Radiol. Oncol.
ISSN	15813207 (electronic), 13182099
CODEN	RONCE
Source Type	Journal
Source Publication Date	2015-06-01
Entry Date	2015-04-08 (In process)
Publication Type	Article
Page Range	163-172
Country of Author	Slovenia
Country of Source	Germany
Language of Article	English
Language of Summary	English
Embase Accession Number	2015886769
Number of References	46

Related articles

View related articles on Embase.com

Copyright

Copyright 2015 Elsevier B.V., All rights reserved.

Records

7. **Perioperative chemotherapy in locally advanced gastric cancer**

Pereira J., Brito D., Ferreira A., Fernandes C., Ribeiro C., Sousa A., Videira F., Lara Santos L., Pereira D., Abreu De Sousa J.

European Journal of Surgical Oncology 2014 **40:11 (S160)**

Embase

Go to publisher for the full text

Abstract

Background: About two thirds of patients with gastric cancer have locally advanced disease at diagnosis. Recent studies have demonstrated that these patients benefit from perioperative chemotherapy, resulting in an improvement of the T- and N-categories and of the R0 resection rate. The aim of this study is to analyse the results of our institution in this population. Material and methods: Retrospective study, analysing all patients with locally advanced gastric cancer who have been treated with preoperative chemotherapy before surgery in a single institution, between 2008 and 2013. Medical records were reviewed and the data analysis was performed with SPSS. Results: In this period, 50 patients with locally advanced gastric cancer were initially treated with preoperative chemotherapy. The majority of the patients were male - 76% (38) - with a median age of 64 (31-78) years old and an ECOG performance status of 0 (37) or 1 (13). The tumors were cT3 and cT4 (a/b) with clinically positive lymph nodes in 92% (46) of the cases and they were from the gastroesophageal junction (n = 18), gastric fundus (n = 2), gastric body (n = 18), gastric antrum (n = 5), lesser curvature (n = 6) and greater curvature (n = 1). Almost all patients (48) were treated with 5-Fluorouracil and Cisplatin, performed along a median of 3 treatments. A re-evaluation CT-scan was performed in 46 (92%) patients, showing a partial response in 33 (66%) and progression of the disease in 5 patients (10%). A surgery with a curative intent was performed in 74% (37) of the patients, with a R0 resection rate of 92% (34). A D2-lymphadenectomy was performed in all cases, with more than 15 lymph nodes isolated in all patients and more than 25 in 76.3% of them (29). Occult metastases were found in 10 patients (20%). The surgical morbidity rate was 12.5% (6) and one patient have died. All tumors were adenocarcinoma on the histological analysis, 56% (28) of them staged ypN+, 10.4% (5) staged ypT1-2 and one patient have had complete pathologic response. Thirty four patients (74%) received postoperative chemotherapy. The median OS was 30.8 months (M) and the median Disease- free Survival (DFS) was 27 M, with a median time of follow-up of 17.6 M. Conclusions: In patients with resectable locally advanced gastric adenocarcinomas, a perioperative chemotherapy regimen induces a downstaging of the tumor, with excellent rates of R0 resection, OS and DFS, in our case series.

Other works by authors of this record

Pereira J., Brito D., Ferreira A., Fernandes C., Ribeiro C., Sousa A., Videira F., Lara Santos L., Pereira D., Abreu De Sousa J.

Emtree drug index terms

ajulemic acid, cisplatin, fluorouracil

Emtree medical index terms

adenocarcinoma, case study, **chemotherapy**, computer assisted tomography, data analysis, data analysis software, diagnosis, disease free survival, electrocorticography, **European**, follow up, human, lower esophagus sphincter, lymph node, lymph node dissection, male, medical record, metastasis, morbidity, neoplasm, **oncology**, patient, population, retrospective study, **society**, stomach adenocarcinoma, stomach antrum, **stomach cancer**, stomach fundus, surgery

Author Address

J. Pereira. Instituto Português de Oncologia do Porto Francisco Gentil, Oncologia Cirúrgica.

Additional Information

Abbreviated Journal Title	Eur. J. Surg. Oncol.
ISSN	07487983
Source Type	Journal
Conference Name	34th Congress of the European Society of Surgical Oncology in partnership with BASO 2014
Conference Location	Liverpool, United Kingdom
Conference Date	2014-10-29 to 2014-10-31
Source Publication Date	November 2014
Entry Date	2015-03-26 (Full record)
Publication Type	Conference Abstract
Page Range	S160
Country of Author	Portugal
Language of Article	English
Language of Summary	English

Related articles

View related articles on Embase.com

Copyright

Copyright 2015 Elsevier B.V., All rights reserved.

Records

8. **The role of statines in the treatment of esophageal cancer patients**

Anderegg M., Lagarde S., Gisbertz S., Meijer S., Hulshof M., Bergman J., Van Laarhoven H., Van Berge Henegouwen M.

Diseases of the Esophagus 2014 **27 SUPPL. 1 (143A)**

Embase

Go to publisher for the full text

Abstract

Background: Recently, there has been an increasing interest in the potential influence of statins on therapeutic response rates and survival in different types of cancer. However, no studies explored the role of statins in the curative treatment of esophageal cancer. The aim of the present study is to investigate the effect of statins on pathologic complete response (pCR) rates and disease free survival in patients who are treated with neoadjuvant chemo(radio)therapy followed by esophagectomy. Methods: Between March 1994 and September 2013 all consecutive patients with cancer of the esophagus or gastroesophageal junction who underwent esophageal resection after neoadjuvant chemo(radio)therapy were included in the present study. Baseline demographic and clinical characteristics were compared between statin users and nonusers. Results: 463 patients were included, 93 (20.1%) used statines at the time of diagnosis. 88 (19%) underwent preoperative chemotherapy and 375 (81%) underwent neoadjuvant chemoradiation. 85 (18%) patients had a pCR (pyT-0N0M0R0). pCR was not significantly different between statin users and nonstatin users (23% vs 17%, p = 0.239). Median disease free survival was not significantly different between statin users and nonstatin users (44 (95% CI 32.2-55.9) vs 41 (95% CI 30.0-53.7) months, p = 0.509). Discussion: Statin users did not experience different outcomes compared with non-users and statin use did not affect the efficacy of neoadjuvant therapy. These data do not support modification or discontinuation of statin therapy for patients with esophageal cancer.

Other works by authors of this record

Anderegg M., Lagarde S., Gisbertz S., Meijer S., Hulshof M., Bergman J., Van Laarhoven H., Van Berge Henegouwen M.

Emtree drug index terms

hydroxymethylglutaryl coenzyme A reductase inhibitor, statin (protein)

Emtree medical index terms

adjuvant therapy, **cancer patient**, chemoradiotherapy, chemotherapy, diagnosis, disease free survival, **diseases**, **esophagus**, **esophagus cancer**, esophagus resection, **human**, lower esophagus sphincter, neoplasm, patient, **society**, survival, telecommunication, therapy, treatment response

Author Address

M. Anderegg. Academic Medical Center.

Additional Information

Abbreviated Journal Title	Dis. Esophagus
ISSN	11208694
Source Type	Journal
Conference Name	14th World Congress of the International Society for Diseases of the Esophagus, ISDE 2014
Conference Location	Vancouver, BC, Canada
Conference Date	2014-09-22 to 2014-09-24
Source Publication Date	September 2014
Entry Date	2015-02-02 (Full record)
Publication Type	Conference Abstract
Page Range	143A
Country of Author	Netherlands
Language of Article	English
Language of Summary	English

Related articles

View related articles on Embase.com

Copyright

Copyright 2015 Elsevier B.V., All rights reserved.

Records

9. **Induction chemotherapy followed by surgery for advanced esophageal cancer**

Toxopeus E., Talman S., Van Der Gaast A., Spaander M., Van Rij C., Krak N., Biermann K., Tilanus H., Mathijssen R., Van Lanschot J., Wijnhoven B.

Diseases of the Esophagus 2014 **27 SUPPL. 1 (48A)**

Embase

Go to publisher for the full text

Abstract

Background: The treatment of choice for curable esophageal cancer in the Netherlands is chemoradiotherapy followed by surgery. In patients with locoregionally advanced tumors or disputable distant metastases, induction chemotherapy is given with the aim to downstage the tumor before a complete tumor resection is considered. Methods: Patients with esophageal or gastroesophageal junction cancer primarily deemed irresectable but without distant metastases who underwent induction chemotherapy between January 2005 and December 2012 were identified from a prospective institutional database. Response to chemotherapy was assessed by CT. Survival was calculated using the Kaplan Meier method. Uni- and multivariable analyses were performed to identify prognostic factors for survival. Results: In total 124 patients received induction chemotherapy mainly for locoregionally advanced disease (N = 80). Surgery was withheld in 35 patients because of progressive disease (N = 16) and persistent irresectability (N = 19). The median overall survival of this group was 13 months (IQR: 8-19). The remaining 89 patients underwent surgery of which 13 still had an irresectable tumor or distant metastases. Of the 76 patients that underwent an esophagec-tomy, 50 patients had tumor free resection margins (66%) with an estimated 5-year survival of 37%. A positive resection margin (HR 4.148, 95% CI 2.298- 7.488, P < 0.0001) was associated with a worse survival in univariate analysis, but only pathological lymph node status with increasing hazard ratio's (6.283- 10.283, P = 0.001) remained significant after multivariable analysis. Discussion: Induction chemotherapy is able to downstage the tumor and facilitates radical esophagectomy in a substantial number of patients with esophageal cancer deemed irresectable at the time of diagnosis. Pathological lymph node status is an independent prognostic factor for overall survival.

Other works by authors of this record

Toxopeus E., Talman S., Van Der Gaast A., Spaander M., Van Rij C., Krak N., Biermann K., Tilanus H., Mathijssen R., Van Lanschot J., Wijnhoven B.

Emtree drug index terms

Emtree medical index terms

cancer surgery, chemoradiotherapy, chemotherapy, data base, diagnosis, **diseases**, distant metastasis, **esophagus**, **esophagus cancer**, esophagus resection, hazard ratio, human, **induction chemotherapy**, Kaplan Meier method, lower esophagus sphincter, lymph node, neoplasm, Netherlands, overall survival, patient, **society**, **surgery**, survival, univariate analysis

Author Address

E. Toxopeus. Erasmus MC University Medical Center.

Additional Information

Abbreviated Journal Title	Dis. Esophagus
ISSN	11208694
Source Type	Journal
Conference Name	14th World Congress of the International Society for Diseases of the Esophagus, ISDE 2014
Conference Location	Vancouver, BC, Canada
Conference Date	2014-09-22 to 2014-09-24
Source Publication Date	September 2014
Entry Date	2015-02-02 (Full record)
Publication Type	Conference Abstract
Page Range	48A
Country of Author	Netherlands
Language of Article	English
Language of Summary	English

Related articles

View related articles on Embase.com

Copyright

Copyright 2015 Elsevier B.V., All rights reserved.

Records

10. **The relevance of the location of involved nodes in patients with cancer of the esophagus**

Lagarde S., Anderegg M., Gisbertz S., Meijer S., Hulshof M., Bergman J., Van Laarhoven H., Henegouwen M.V.
Diseases of the Esophagus 2014 **27 SUPPL. 1 (73A)**
Embase

Go to publisher for the full text

Abstract

Background: Truncal node metastases as well as lymphatic dissemination in the proximal field (subcarinal, paratracheal and aortopulmonary window lymph nodes) after neoadjuvant chemoradiation therapy does not alter the TNM classification. The incidence and impact of these relatively distant lymph node metastases on long-term survival remains unclear. Therefore the aim of the present study is to identify the incidence and prognostic signifi-cance of the location of lymph node metastasis in patients who underwent neoadjuvant chemoradiation therapy followed by a transthoracic esopha-gectomy (TTE). Methods: Between March 2003 and September 2013 a total of 286 consecutive patients with adenocarcinoma or squamous cell cancer of the mid-to-distal esophagus or gastroesophageal junction (GEJ) who underwent potentially curative esophageal TTE after neoadjuvant chemoradiation therapy were included. Results: The majority of patients was male (219 patients, 76.6%) and had an adenocarcinoma (208 patients, 72.7%). The tumor was located in the mid-esophagus in 53 (18.5%), in the distal esophagus in 210 (73.4%) and at the GEJ/cardia in 23 (8.0%) patients. 279 (97.6%) patients underwent a radical (R0) resection. 112 (39.2%) patients had a complete or near complete pathologic response (tumor regression grade 1 or 2). 110 (38.5%) patients had nodal metastases in the marked resection specimen. 63 (22.0%) patients were classified as N1, 33 (11.5%) patients as N2 and 14 (4.8%) patients as N3. Of the patients with tumorpositive lymph nodes, 40 (36.4%) patients had metas-tases localized in locoregional nodes, 35 (31.8%) patients had localisation of metastases in at least one truncal node, 14 (12.7%) patients had positive nodes in the proximal field and 5 (4.5%) patients had positive truncal nodes as well as positive proximal lymph nodes. Median disease free-survival was months for N0 patients, 65.7 months for patients with nodal metastases limited to locoregional nodes, 18.8 months for patients with truncal nodes, months for patients with lymph node in the proximal field and 10.1 months if nodes were positive in both the truncal and the proximal field (Figure 1: survival functions). In multivariate analysis yN stage as well as location of lymph nodes were independently associated with a worse survival. Discussion: The present study demonstrated that the location of positive nodes after neoadjuvant chemoradiation therapy harbors

important prognostic information.

Other works by authors of this record

Lagarde S., Anderegg M., Gisbertz S., Meijer S., Hulshof M., Bergman J., Van Laarhoven H., Henegouwen M.V.

Emtree drug index terms

Emtree medical index terms

adenocarcinoma, aortopulmonary septal defect, cancer staging, chemoradiotherapy, disease free survival, **diseases**, **esophagus**, **human**, long term survival, lower esophagus sphincter, lymph node, lymph node metastasis, lymph vessel, male, metastasis, multivariate analysis, **neoplasm**, **patient**, radical resection, **society**, squamous cell carcinoma, surgery, survival, therapy, tumor regression

Author Address

S. Lagarde. Academic Medical Center Amsterdam.

Additional Information

Abbreviated Journal Title	Dis. Esophagus
ISSN	11208694
Source Type	Journal
Conference Name	14th World Congress of the International Society for Diseases of the Esophagus, ISDE 2014
Conference Location	Vancouver, BC, Canada
Conference Date	2014-09-22 to 2014-09-24
Source Publication Date	September 2014
Entry Date	2015-02-02 (Full record)
Publication Type	Conference Abstract
Page Range	73A
Country of Author	Netherlands
Language of Article	English
Language of Summary	English

Related articles

View related articles on Embase.com

Copyright

Copyright 2015 Elsevier B.V., All rights reserved.

Records

11. **Survival after pathologic complete response in patients with cancer of the esophagus or gastro-esophageal junction**

Anderegg M., Lagarde S., Borstlap W., Gisbertz S., Meijer S., Hulshof M., Bergman J., Van Laarhoven H., Henegouwen M.V.B.

Diseases of the Esophagus 2014 **27 SUPPL. 1 (76A)**

Embase

Go to publisher for the full text

Abstract

Background: The preferred curative strategy for esophageal cancer patients with locally advanced tumors, but without distant metastases consists of esophagectomy with preceding chemo(radio)therapy (CRT). In 10-40% of patients who are neoadjuvantly treated, there is absence of viable tumor at the time of surgery (pathologic complete response (pCR)). The aim of the present study was to define the outcome of patients with a pCR and identify predictive factors for survival in this group. Methods: Between March 1994 and September 2013 all consecutive patients with cancer of the esophagus or gastroesophageal junction who underwent esophageal resection after neoadjuvant chemo (radio) therapy were included in the present study. Multivariate Cox regression analysis was carried out to identify independent prognostic factors. Results: Of the 463 included patients, 86 (19%) patients had a pCR (pyT-0N0M0R0) (54 men, 32 women, median age: 63 yrs (range 33-82 years)). 48 (56%) patients had an adenocarcinoma. Eight (9%) patients underwent neo-adjuvant chemotherapy and 78 (91%) underwent neoadjuvant chemoradia-tion therapy. During follow-up, 25 (29%) patients developed recurrent disease. Nineteen (76%) patients developed haematogenous metastases, 6 developed lymphatic metastases (of which 3 patients with a distant lymphatic location). 5-year disease free survival was 61%, 5-year overall survival was 58%. Cox regression analysis revealed no prognostic factor for any of the tested variables (sex, age, histologic subtype, tumorlocation, type of neoadjuvant therapy, cTNM stage). Discussion: Patients with a pathologic complete response have a relatively good survival. However, one third of these patients developed recurrent disease. Thus far it is unclear how these patients can be identified.

Other works by authors of this record

Anderegg M., Lagarde S., Borstlap W., Gisbertz S., Meijer S., Hulshof M., Bergman J., Van Laarhoven H., Henegouwen M.V.B.

Emtree drug index terms

Emtree medical index terms

adenocarcinoma, adjuvant chemotherapy, adjuvant therapy, cancer patient, disease free survival, **diseases**, distant metastasis, **esophagus**, esophagus cancer, esophagus resection, female, follow up, **human**, lower esophagus sphincter, lymph node metastasis, lymph vessel, male, metastasis, **neoplasm**, overall survival, **patient**, proportional hazards model, recurrent disease, regression analysis, **society**, surgery, **survival**, telecommunication, therapy

Author Address

M. Anderegg. Academic Medical Center.

Additional Information

Abbreviated Journal Title	Dis. Esophagus
ISSN	11208694
Source Type	Journal
Conference Name	14th World Congress of the International Society for Diseases of the Esophagus, ISDE 2014
Conference Location	Vancouver, BC, Canada
Conference Date	2014-09-22 to 2014-09-24
Source Publication Date	September 2014
Entry Date	2015-02-02 (Full record)
Publication Type	Conference Abstract
Page Range	76A
Country of Author	Netherlands
Language of Article	English
Language of Summary	English

Related articles

View related articles on Embase.com

Copyright

Copyright 2015 Elsevier B.V., All rights reserved.

Records

12. **Survival after pathologic complete response in patients with cancer of the esophagus or gastroesophageal junction**

Lagarde S.M., Anderegg M.C.J., Borstlap W.A., Gisbertz S.S., Meijer S.L., Hulshof M., Bergman J., Van Laarhoven H.W.M., Van Berge Henegouwen M.

Journal of Clinical Oncology 2014 **32:15 SUPPL. 1**

Embase

Abstract

Background: The preferred curative strategy for esophageal cancer patients with locally advanced cancers, but without distant metastases consists of esophagectomy with preceding chemo(radio)therapy (CRT). In 10-40% of patients, In 10-40% of patients who are neoadjuvantly treated, there is absence of viable tumor at the time of surgery (pathologic complete response (pCR)). Therefore the aim of the present study was to define the outcome of patients with a pCR and identify predictive factors for survival in this group. Methods: Between March 1994 and September 2013 all consecutive patients with cancer of the esophagus or gastroesophageal junction who underwent esophageal resection after neoadjuvant chemo (radio) therapy were included in the present study. Multivariate Cox regression analysis was carried out to identify independent prognostic factors. Results: Of the 463 included patients, 86 (19%) patients had a pCR (pyT0N0M0R0) (54 men, 32 women, median age: 63yrs (range 33-82 years)). 48 (56%) patients had an adenocarcinoma. Eight (9%) patients underwent neoadjuvant chemotherapy and 78 (91%) underwent neoadjuvant chemoradiation therapy. During follow-up, 25 (29%) patients developed recurrent disease. Nineteen (76%) patients developed haematogenous metastasis, 6 developed lymphatic metastases (of which 3 patients distant lymphatic location). 5-year disease free survival was 61%, 5 year overall survival was 58%. Cox regression analysis revealed no prognostic factors for any of the variables tested (sex, age, histologic subtype, tumorlocation, type of neoadjuvant therapy, cTNM stage). Conclusions: Patients with a pathologic complete response have a relative good survival. However one third of the patients developed recurrent disease. Thus far it is unclear how these patients can be identified.

Other works by authors of this record

Lagarde S.M., Anderegg M.C.J., Borstlap W.A., Gisbertz S.S., Meijer S.L., Hulshof M., Bergman J., Van Laarhoven H.W.M., Van Berge Henegouwen M.

Emtree drug index terms

Emtree medical index terms

adenocarcinoma, adjuvant chemotherapy, adjuvant therapy, advanced cancer, cancer patient, chemoradiotherapy, disease free survival, distant metastasis, **esophagus**, esophagus cancer, esophagus resection, female, follow up, **human**, **lower esophagus sphincter**, lymph node metastasis, lymph vessel, male, metastasis, **neoplasm**, **oncology**, overall survival, **patient**, proportional hazards model, recurrent disease, regression analysis, **society**, surgery, **survival**, telecommunication, therapy

Author Address

S.M. Lagarde.

Additional Information

Abbreviated Journal Title	J. Clin. Oncol.
ISSN	0732183X
Source Type	Journal
Conference Name	2014 Annual Meeting of the American Society of Clinical Oncology, ASCO
Conference Location	Chicago, IL, United States
Conference Date	2014-05-30 to 2014-06-03
Source Publication Date	2014-05-20
Entry Date	2014-07-16 (Full record)
Publication Type	Conference Abstract
Page Range	
Language of Article	English
Language of Summary	English

Related articles

View related articles on Embase.com

Copyright

Copyright 2014 Elsevier B.V., All rights reserved.

Records

13. **The role of statines in the treatment of esophageal cancer patients**

Lagarde S.M., Anderegg M.C.J., Borstlap W.A., Gisbertz S.S., Meijer S.L., Hulshof M., Bergman J., Van Laarhoven H.W.M., Van Berge Henegouwen M.

Journal of Clinical Oncology 2014 **32:15 SUPPL. 1**

Embase

Abstract

Background: Recently, there has been an increasing interest in the potential role of statins on pCR rates and survival in different types of cancer. However, no studies explored the role of statins in the curative treatment of esophageal cancer. The aim of the present study is to investigate the effect of statins on pathologic CR (pCR) and disease free survival in patients who are treated with after neoadjuvant chemo(radiation) therapy followed by esophagectomy. Methods: Between March 1994 and September 2013 all consecutive patients with cancer of the esophagus or gastroesophageal junction who underwent esophageal resection after neoadjuvant chemo (radio) therapy were included in the present study. Baseline demographic and clinical characteristics were compared between statin users nonusers. Results: 463 patients were included, 93 (20.1%) used statines at the time of diagnosis. 88 (19%) underwent preoperative chemotherapy and 375 (81%) underwent neoadjuvant chemoradiation therapy. 85 (18%) patients had a pCR (pyT0N0M0R0). pCR was not significantly different between statin users and nonstatin users (23% vs 17%, p=0.239). Median disease free survival was not significantly different between statin users and nonstatin users (44 (95% CI 32.2-55.9) vs 41 (95% CI 30.0-53.7) months, p=0.509). Conclusions: Statin users did not experience different outcomes compared with non-users and statin use did not affect the efficacy of neoadjuvant therapy. These data do not support modification or discontinuation of statin therapy for patients with esophageal cancer.

Other works by authors of this record

Lagarde S.M., Anderegg M.C.J., Borstlap W.A., Gisbertz S.S., Meijer S.L., Hulshof M., Bergman J., Van Laarhoven H.W.M., Van Berge Henegouwen M.

Emtree drug index terms

hydroxymethylglutaryl coenzyme A reductase inhibitor, statin (protein)

Emtree medical index terms

adjuvant therapy, **cancer patient**, chemoradiotherapy, chemotherapy, diagnosis, disease free survival, esophagus, **esophagus cancer**, esophagus resection, **human**, lower esophagus sphincter, neoplasm, **oncology**, patient, radiation, **society**, survival, telecommunication, therapy

Author Address

S.M. Lagarde.

Additional Information

Abbreviated Journal Title	J. Clin. Oncol.
ISSN	0732183X
Source Type	Journal
Conference Name	2014 Annual Meeting of the American Society of Clinical Oncology, ASCO
Conference Location	Chicago, IL, United States
Conference Date	2014-05-30 to 2014-06-03
Source Publication Date	2014-05-20
Entry Date	2014-07-16 (Full record)
Publication Type	Conference Abstract
Page Range	
Language of Article	English
Language of Summary	English

Related articles

View related articles on Embase.com

Copyright

Copyright 2014 Elsevier B.V., All rights reserved.

Records

14. **Results of the baseline positron emission tomography can customize therapy of localized esophageal adenocarcinoma patients who achieve a clinical complete response after chemoradiation**

Suzuki A., Xiao L., Taketa T., Sudo K., Wadhwa R., Blum M.A., Skinner H., Komaki

R., Weston B., Lee J.H., Bhutani M.S., Rice D.C., Maru D.M., Erasmus J., Swisher S.G., Hofstetter W.L., Ajani J.A.

Annals of Oncology 2013 **24:11 (2854-2859) Article Number mdt340**

Embase MEDLINE

Go to publisher for the full text

Abstract

Background: Patients with localized esophageal adenocarcinoma (EAC) who achieve a clinical complete response (clinCR) after preoperative chemoradiation (trimodality therapy; TMT) or definitive chemoradiation (bimodality therapy; BMT) live longer than those who achieve a <clinCR (Suzuki A, Xiao LC, Hayashi Y et al. Prognostic significance of baseline positron emission tomography and importance of clinical complete response in patients with esophageal or gastroesophageal junction cancer treated with definitive chemoradiotherapy. Cancer 2011; 117: 4823-4833; Cheedella NK, Suzuki A, Xiao L et al. Association between clinical complete response and pathological complete response after preoperative chemoradiation in patients with gastroesophageal cancer: analysis in a large cohort. Ann Oncol 2013; 24: 1262-1266; Ajani JA, Correa AM, Hofstetter WL et al. Clinical parameters model for predicting pathologic complete response following preoperative chemoradiation in patients with esophageal cancer. Ann Oncol 2012; 23: 2638-2642). We hypothesized that the initial standardized uptake value (iSUV) of positron emission tomography will define novel subsets of clinCR patients. Methods: We analyzed 323 EAC patients, from our prospective database, who achieved a clinCR. Various statistical methods were used to assess the influence of iSUV on patient outcome. Results: The median follow-up of 323 patients was 40.8 months [95% confidence interval (CI) 35.6-47.3 months]. Two hundred six (63.8%) patients had TMT and 117 (36.2%) had BMT. If iSUV was ≥6, TMT patients had a longer median OS (94.8 months; 95% CI 66.07-NA) than BMT patients (31.4 months; 95% CI 21.7-42.1; P ≤0.001). However, if iSUV was <6, the median OS of TMT and BMT patients was similar (P = 0.62). iSVU did not influence the pathologic complete response rate in TMT patients (P = 0.85). Conclusion: clinCR patients with iSUV of <6 are identified as a new subset that fared equally well when treated with TMT or BMT. Future esophageal preservation strategy may be best suited for this newly identified subset of EAC patients. © The Author 2013. Published by Oxford University Press on behalf of the European Society for Medical Oncology All rights reserved.

Other works by authors of this record

Suzuki A., Xiao L., Taketa T., Sudo K., Wadhwa R., Blum M.A., Skinner H., Komaki R., Weston B., Lee J.H., Bhutani M.S., Rice D.C., Maru D.M., Erasmus J., Swisher S.G., Hofstetter W.L., Ajani J.A.

Emtree drug index terms

docetaxel (drug combination), docetaxel (drug therapy), fluorodeoxyglucose f 18 (intravenous drug administration), fluorouracil (drug combination), fluorouracil (drug therapy), fluorouracil (intravenous drug administration), fluorouracil (oral drug administration), oxaliplatin (drug combination), oxaliplatin (drug therapy)

Emtree medical index terms

adult, aged, article, cancer prognosis, **chemoradiotherapy**, continuous infusion, controlled study, esophageal adenocarcinoma (drug therapy), esophageal adenocarcinoma (drug therapy), esophageal adenocarcinoma (radiotherapy), esophageal adenocarcinoma (surgery), esophagus resection, female, follow up, human, initial standardized uptake value, major clinical study, male, multimodality cancer therapy, overall survival, PET scanner, **positron emission tomography**, priority journal, radiological parameters, recurrence free survival

Author Address

J.A. Ajani. Departments of Gastrointestinal Medical Oncology, University of Texas M.D. Anderson Cancer Center, Houston.

Author Email

jajani@mdanderson.org

Additional Information

Abbreviated Journal Title	Ann. Oncol.
ISSN	09237534, 15698041 (electronic)
CODEN	ANONE
Source Type	Journal
Source Publication Date	November 2013
Entry Date	2013-12-06 (Full record), 2013-11-13 (Article in Press/In process)
Publication Type	Article
Page Range	2854-2859
Country of Author	United States
Country of Source	United Kingdom

Language of Article	English
Language of Summary	English
MEDLINE PMID	23994746
Embase Accession Number	2013699645
Article Number	mdt340
Number of References	18
Device Tradenames	Discovery ST-8 (GE Healthcare, United States)
Device Manufacturers	GE Healthcare (United States)
CAS Registry Numbers	docetaxel (114977-28-5)
	fluorodeoxyglucose f 18 (63503-12-8)
	fluorouracil (51-21-8)
	oxaliplatin (61825-94-3)

Related articles

View related articles on Embase.com

Copyright

MEDLINE® is the source for part of the citation data of this record

Records

15. **A phase 2 study of neoadjuvant therapy with cisplatin, docetaxel, panitumumab plus radiation therapy followed by surgery in patients with locally-advanced adenocarcinoma of the distal esophagus: Results of acosog z4051 (alliance)**

Schefter T., Decker P., Meyers B., Ferguson M.K., Oeltjen A., Putnam J.B., Cassivi S., Reed C.E., Lockhart A.C.

International Journal of Radiation Oncology Biology Physics 2013 **87:2 SUPPL. 1 (S85-S86)**
Embase

Go to publisher for the full text

Abstract

Purpose/Objective(s): A number of clinical trials have incorporated preoperative chemoradiation therapy (CRT) in an attempt to improve local tumor control, distant

recurrence and overall survival for patients with locally advanced but resectable adenocarcinoma of the distal esophagus or gastroesophageal junction (GEJ). This multi-centered, cooperative group study combined cisplatin, docetaxel and panitumumab with radiation therapy preoperatively for patients with locoregionally advanced esophageal adenocarcinoma. Pathologic complete response (pCR) of ≥ 35% was the primary endpoint target goal. Materials/Methods: From January 15, 2009 to July 22, 2011, 70 patients with distal esophageal or GEJ adenocarcinomas of clinical stage T3N0M0, T2-3N1M0 or T2-3N0-1M1a (M1a with celiac LN ≤ 2 cm) were accrued. Patients received cisplatin (40 mg/m2), docetaxel (40 mg/m2), and panitumumab (6 mg/kg) on weeks 1, 3, 5, 7 and 9 with RT (5040 cGy, 180 cGy/day x 28d) beginning week 5. Pathologic complete response was defined as no viable residual tumor cells. Secondary objectives included near-pathologic complete response (near-pCR), toxicity, and overall and disease-free survival rates. A near-pCR was defined as ≤ 10% of viable residual cancer cells. Adverse events were graded using the Common Terminology Criteria for Adverse Events Version 3.0. Results: Five of 70 patients were ineligible. One patient had a celiac lymph node > 2 cm, and one patient had two primary tumors. Liver lesions were not investigated in one patient. Two patients had proximal gastric cancers. Of the remaining 65 patients (59 M, 6 F; median age 61), 11 patients did not undergo surgery (4 progressions, 3 patient refusals, 3 other, 1 physician decision), leaving 54 evaluable patients. Median follow-up was 20.1 months. The pCR rate was 33.3% and near-pCR was 20.4%. Total doses of cisplatin, docetaxel and panitumumab were administered in 75%, 80%, and 72%, respectively (n = 65). Ninety-four percent of patients received the total radiation therapy dose. Twenty-eight (43.1%) patients had grade 4 or greater toxicity that was possibly related to treatment. Lymphopenia (15 patients) was most common. Operative mortality was 3.7% (2/54). There was one death attributable to multisystem organ failure and one was due to gastric conduit necrosis. ARDS was encountered in 2 patients (3.7%). Median survival was 18.6 mos; 2-yr disease-free survival Kaplan-Meier estimate was 51.6% (95% CI, 36.7% - 72.6%) and 3-yr overall survival was 46.1% (95% CI 34.3 to 62%). Conclusions: This neoadjuvant regimen of cisplatin, docetaxel and panitumumab and radiation is active (pCR + near-pCR = 53.7%) but did not meet the primary outcome. The toxicity is substantial, but manageable. Further evaluation of this regimen in an unselected population is not recommended.

Other works by authors of this record

Schefter T., Decker P., Meyers B., Ferguson M.K., Oeltjen A., Putnam J.B., Cassivi S., Reed C.E., Lockhart A.C.

Emtree drug index terms

cisplatin, docetaxel, panitumumab

Emtree medical index terms

adenocarcinoma, adjuvant therapy, adult respiratory distress syndrome, cancer cell, cancer control, chemoradiotherapy, clinical trial (topic), death, disease free survival, esophageal adenocarcinoma, esophagus, follow up, human, Kaplan Meier method, liver injury, lower esophagus sphincter, lymph node, lymphocytopenia, multiple organ failure, necrosis, oncology, overall survival, patient, phase 2 clinical trial, physician, population, primary tumor, radiation, radiotherapy, society, stomach cancer, surgery, surgical mortality, survival, survival rate, therapy, toxicity, tumor cell

Author Address

T. Schefter. University of Colorado Denver.

Additional Information

Abbreviated Journal Title	Int. J. Radiat. Oncol. Biol. Phys.
ISSN	03603016
Source Type	Journal
Conference Name	55th Annual Meeting of the American Society for Radiation Oncology, ASTRO 2013
Conference Location	Atlanta, GA, United States
Conference Date	2013-09-22 to 2013-09-25
Source Publication Date	2013-10-01
Entry Date	2013-10-11 (Full record)
Publication Type	Conference Abstract
Page Range	S85-S86
Country of Author	United States
Language of Article	English
Language of Summary	English
Publisher Item Identifier	S0360301613008924

Related articles

View related articles on Embase.com

Copyright

Copyright 2013 Elsevier B.V., All rights reserved.

Records

16. **Frequency of pathological complete response with neo-adjuvant chemoradiotherapy in locally advanced esophageal cancer**

Mehmood T., Ali M., Saeed K., Irfan M., Ghaffar A., Munawar A., Rashid A.

Annals of Oncology 2013 **24 SUPPL. 4 (iv55)**

Embase

Go to publisher for the full text

Abstract

Background: Neoadjuvant chemoradiotherapy followed by esophagectomy has become the standard of care for patients with locally advanced esophageal cancer. The achievement of a pathological complete response is associated with an improved survival. This report analyzes our experience with this treatment approach. Methods: With a median age of 52 years (range 20 - 74), we retrospectively analyzed 61 patients (males 58%, females 42%) between 2003 to 2012 with pathologically confirmed esophageal cancer, treated with neo-adjuvant chemoradiotherapy followed by esophagectomy. Pre-treatment AJCC stage; stage I 6%, stage II 46% and stage III 48% of the patients respectively. Site distribution; upper thoracic 2%, middle thoracic 24%, lower thoracic 62% and gastroesophageal junction tumour in 12% of the patients. Mean tumour length was 5.8 cm (range 2 - 11). Histological sub-types include; squamous cell carcinoma 80% and adenocarcinoma 20% of the patients. 78% of the patients had grade I/II disease while 22% had grade III disease. All patients received neo-adjuvant chemoradiotherapy with cisplatin (100mg/m^2) intravenously on the first day of weeks 1, 4, 7 and 11 and fluorouracil (1000mg/m^2) per day by continuous infusion on the first 4 days of weeks 1, 4, 7 and 11. Radiotherapy 50Gy in 25 fractions 5 days a week over a period of 5 weeks was given starting from day 1 of week 7. Transhiatal esophagectomy was the most common surgical procedure, performed in 67% of the patients. Pathological complete response (pCR) was defined as lack of invasive cancer in both the resected tissue and lymph nodes on final pathological review. Results: The whole course of chemoradiotherapy was very well tolerated by patients with grade III nausea/vomiting, diarrhea and neutropenia seen in only 5%, 3% and 8% of the patients respectively. Operative mortality and morbidity was 6.5% and 20% respectively. Pathologic response was; complete response 44%, partial response 31%, stable disease 17% and progressive

disease in 8% of the patients. Down staging of T stage alone and N stage alone occurred in 77% and 34% of the patients respectively. 18% of the patients failed treatment. Patterns of recurrence includes; local 3%, loco-regional 3% and distant in 13% of the patients. The 5 year overall survival and progression free survival for the whole group was 43% and 33% respectively. Conclusion: Esophagectomy after neo-adjuvant chemoradiotherapy can be performed with low mortality and morbidity. Chemotherapy regimens with high response rates merit evaluation in randomized trials to improve outcome in locally advanced esophageal cancer.

Other works by authors of this record

Mehmood T., Ali M., Saeed K., Irfan M., Ghaffar A., Munawar A., Rashid A.

Emtree drug index terms

cisplatin, fluorouracil

Emtree medical index terms

achievement, adenocarcinoma, **adjuvant chemoradiotherapy**, chemoradiotherapy, chemotherapy, continuous infusion, diarrhea, **digestive system cancer**, **esophagus cancer**, esophagus resection, female, health care quality, human, lower esophagus sphincter, lymph node, male, morbidity, mortality, neoplasm, neutropenia, overall survival, patient, progression free survival, radiotherapy, squamous cell carcinoma, staging, surgical mortality, surgical technique, survival, tissues, tumor invasion

Author Address

T. Mehmood. Shaukat Khanum Memorial Cancer Hospital, Research Centre.

Additional Information

Abbreviated Journal Title	Ann. Oncol.
ISSN	09237534
Source Type	Journal
Conference Name	15th World Congress on Gastrointestinal Cancer, ESMO 2013
Conference Location	Barcelona, Spain
Conference Date	2013-07-03 to 2013-07-06
Source Publication Date	June 2013
Entry Date	2015-02-23 (Full record)
Publication Type	Conference Abstract
Page Range	iv55

Country of Author	Pakistan
Language of Article	English
Language of Summary	English

Related articles

View related articles on Embase.com

Copyright

Copyright 2015 Elsevier B.V., All rights reserved.

Records

17. **A phase II trial of induction epirubicin, oxaliplatin, and flourouracil, surgery and post-operative concurrent cisplatin and fluorouracil chemoradiotherapy (CRT) in patients (pts) with loco-regionally advanced (LRA) adenocarcinoma (ACA) of the esophagus (E) and gastroesophageal junction (GEJ)**

McNamara M.J., Rice T.W., Rybicki L.A., Rodriguez C.P., Videtic G.M.M., Saxton J.P., Stephans K.L., Greskovich J., Sohal D., Mason D.P., Murthy S.C., Ives D.I., Bodmann J., Adelstein D.J.

Journal of Clinical Oncology 2013 **31:15 SUPPL. 1**

Embase

Abstract

Background: In pts with LRA ACA of the E/GEJ, CRT and surgery results in excellent locoregional control. Distant failure remains common, however, suggesting potential benefit from additional chemotherapy. This single arm phase II study investigated the addition of induction chemotherapy to surgery and post-operative CRT. Methods: Pts with an ultrasound-based clinical stage of T3, N1 or M1a (AJCC 6th) ACA of the E/GEJ were eligible. Induction chemotherapy with epirubicin 50mg/m^2 d1, oxaliplatin 130mg/m^2 d1, and flourouracil 200mg/m^2/day continuous infusion for 3 weeks, was given every 21 days for 3 courses and was followed by surgical resection. Adjuvant CRT consisted of 50-55Gy at 1.8-2.0 Gy/d and 2 courses of cisplatin (20mg/m^2/d) and flourouracil (1000mg/m^2/d) given as 96 hour infusions during weeks 1 and 4 of radiotherapy. Results: Between 2/08 and 1/12, 60 evaluable pts enrolled; 95% male, 97% white and 78% with GEJ tumors. Resection was accomplished in 54 pts (90%) and adjuvant CRT in 48 (80%). Toxicity included 1 death during induction (2%), and 2

post-operative deaths (4%). Unplanned hospitalization was required in 18% of pts during induction and 19% during adjuvant CRT. Induction chemotherapy produced a symptomatic response in 79% of pts, a clinical (ultrasound) response in 48% and a pathologic response in 41% (5% complete). With a median follow-up of 31 months, the Kaplan-Meier 3-year projected locoregional control (LRC) is 84%, distant metastatic control (DMC) 44%, relapse-free survival (RFS) 39%, and overall survival (OS) 42%. Symptomatic response to induction and the percentage of remaining viable tumor at surgery proved the strongest predictors of DMC, RFS, and OS. Conclusions: Induction chemotherapy, surgery and adjuvant CRT is feasible and produces outcomes similar to other multimodality treatment schedules in LRA E/GEJ ACA. Despite excellent LRC, projected DMC and OS remain poor. A symptomatic response to induction and less residual viable tumor at surgery are associated with improved outcomes.

Other works by authors of this record

McNamara M.J., Rice T.W., Rybicki L.A., Rodriguez C.P., Videtic G.M.M., Saxton J.P., Stephans K.L., Greskovich J., Sohal D., Mason D.P., Murthy S.C., Ives D.I., Bodmann J., Adelstein D.J.

Emtree drug index terms

adjuvant, **cisplatin**, **epirubicin**, **fluorouracil**, **oxaliplatin**

Emtree medical index terms

adenocarcinoma, arm, **chemoradiotherapy**, chemotherapy, continuous infusion, death, **esophagus**, follow up, hospitalization, **human**, induction chemotherapy, infusion, **lower esophagus sphincter**, male, neoplasm, **oncology**, overall survival, **patient**, **phase 2 clinical trial**, radiotherapy, recurrence free survival, **society**, **surgery**, toxicity, ultrasound

Author Address

M.J. McNamara.

Additional Information

Abbreviated Journal Title	J. Clin. Oncol.
ISSN	0732183X
Source Type	Journal
Conference Name	2013 Annual Meeting of the American Society of Clinical Oncology, ASCO

Conference Location	Chicago, IL, United States
Conference Date	2013-05-31 to 2013-06-04
Source Publication Date	2013-05-20
Entry Date	2013-07-04 (Full record)
Publication Type	Conference Abstract
Page Range	
Language of Article	English
Language of Summary	English

Related articles

View related articles on Embase.com

Copyright

Copyright 2013 Elsevier B.V., All rights reserved.

Records

18. **Preoperative chemotherapy plus bevacizumab with early salvage therapy based on FDG-PET response in locally advanced gastroesophageal junction and gastric adenocarcinoma**

Ku G.Y., Kelsen D.P., Strong V.E., Schöder H., Janjigian Y.Y., Shah M.A., Coit D.G., Brennan M.F., Capanu M., Ilson D.H.

Journal of Clinical Oncology 2013 **31:15 SUPPL. 1**

Embase

Abstract

Background: Response on FDG-PET scan during preoperative chemotherapy has prognostic significance. We performed a phase II trial to examine the effectiveness of FDG-PET directed early switching to salvage chemotherapy measured by 2-year disease free survival (DFS). Methods: Pts with PET avid, endoscopic ultrasound and laparoscopically staged T3 or N+ resectable gastric or GEJ adenocarcinoma received induction epirubicin 50mg/m^2, cisplatin 60mg/m^2 Day 1, capecitabine 625mg/m^2 BID Days 1-21 (ECX) and bevacizumab 15mg/kg Day 1. PET scan was repeated at Week 3. PET responders (35% decline in SUV) continued with ECX for 2 more cycles. PET non-responders were switched to 2 cycles of salvage therapy: docetaxel 30mg/m^2 and irinotecan 50mg/m^{2}Days 1 and 8 q21 days and bevacizumab 15mg/kg Day 1. All pts went to surgery 4 weeks after Cycle 3. Results: Twenty of planned 60 pts were

enrolled before the study closed for poor accrual. Eleven (55%) had a PET response after induction. Ten of 11 underwent R0 resection: 1/10 path complete response, 3/10 path partial response. Nine PET non-responders were switched to the salvage regimen. Seven of 9 non-responders had R0 resection, none achieved a pathological response. The median DFS for PET responders was 27.8 mos (95% CI 10.3-27.8) and DFS in salvage group has not been reached. There was no significant difference in DFS between the two groups (p= 0.4). Follow up for overall survival is ongoing. Conclusions: Response on PET scan during induction chemotherapy can identify early treatment failures. The results for therapy cross-over indicate a potentially improved DFS with salvage chemotherapy. Results from this trial are hypothesis generating and merit evaluation in a larger clinical trial. Updated survival data will be presented.

Other works by authors of this record

Ku G.Y., Kelsen D.P., Strong V.E., Schöder H., Janjigian Y.Y., Shah M.A., Coit D.G., Brennan M.F., Capanu M., Ilson D.H.

Emtree drug index terms

bevacizumab, capecitabine, cisplatin, docetaxel, epirubicin, irinotecan

Emtree medical index terms

adenocarcinoma, **chemotherapy**, clinical trial, disease free survival, endoscopic echography, follow up, human, hypothesis, induction chemotherapy, **lower esophagus sphincter**, **oncology**, overall survival, phase 2 clinical trial, positron emission tomography, **salvage therapy**, **society**, **stomach adenocarcinoma**, surgery, survival, therapy, treatment failure

Author Address

G.Y. Ku.

Additional Information

Abbreviated Journal Title	J. Clin. Oncol.
ISSN	0732183X
Source Type	Journal
Conference Name	2013 Annual Meeting of the American Society of Clinical Oncology, ASCO
Conference Location	Chicago, IL, United States
Conference Date	2013-05-31 to 2013-06-04

Source Publication Date	2013-05-20
Entry Date	2013-07-04 (Full record)
Publication Type	Conference Abstract
Page Range	
Language of Article	English
Language of Summary	English

Related articles

View related articles on Embase.com

Copyright

Copyright 2013 Elsevier B.V., All rights reserved.

Records

19. **Perioperative epirubicin, oxaliplatin, and capectabine (EOX) in locally advanced resectable gastroesophageal junction and gastric adenocarcinoma**

Kalachand R.D., Mongan A.-M., Doherty M., King S., O'Farrell N., Reynolds J.V., O'Byrne K.J.

Journal of Clinical Oncology 2013 **31:15 SUPPL. 1**

Embase

Abstract

Background: Perioperative chemotherapy with epirubicin, cisplatin and fluorouracil (ECF) confers a 13% absolute 5-year survival benefit over surgery alone in resectable gastroesophageal cancer. In the metastatic setting, the EOX regimen is at least as effective as ECF, with a favourable toxicity profile. This retrospective analysis presents the experience of a high-volume tertiary referral center with perioperative EOX in patients with resectable GEJ and gastric adenocarcinoma. Methods: Patients with cTxN+/cT3N0 gastroesophageal adenocarcinoma treated between 2006-2012 with perioperative EOX (epirubicin 50mg/m2 d1 q22d, oxaliplatin 130mg/m2 d1 q22d and capecitabine 625 mg/m2 bd d1-21 q22d), given as three neoadjuvant and three adjuvant cycles, were retrospectively identified from a prospectively recorded upper gastrointestinal cancer database. Efficacy and toxicity data were assessed. Results: 52 patients, including 41 (82%) males, with a median age of 62 (range 35-79) received perioperative EOX with curative intent. GEJ tumours represented 39 (75%)

of cases, and the others were gastric cancers. 47 patients (90%) completed 3 cycles of neoadjuvant EOX. One patient died of non-neutropenic sepsis after cycle 2. 44 (85%) of patients progressed to surgery, 5 of whom were deemed unresectable. 28 (71%) surgeries were D2 resections, and the R0 resection rate was 67%. There was one postoperative death, due to pancreatitis. 28 (54%) of patients commenced adjuvant EOX, and 25 (48%) completed the planned treatment. Grade 3-4 toxicities were observed in 17% of patients, with the commonest being fatigue (8%) and nausea (4%). Pathological response rate, as defined by Mandard Tumour Regression Grade 1-3, was seen in 26%, with 3 (6%) pathological complete response. The median progression-free survival was 7 months, with a median overall survival of 22 months, and a 2-year survival rate of 45%. Conclusions: In our institution, perioperative EOX in gastroesophageal cancer is associated with a reasonable safety profile, and efficacy consistent with that reported in the MAGIC trial.

Other works by authors of this record

Kalachand R.D., Mongan A.-M., Doherty M., King S., O'Farrell N., Reynolds J.V., O'Byrne K.J.

Emtree drug index terms

adjuvant, capecitabine, cisplatin, **epirubicin**, fluorouracil, **oxaliplatin**

Emtree medical index terms

adenocarcinoma, chemotherapy, data base, death, digestive system cancer, fatigue, human, **lower esophagus sphincter**, male, nausea, neoplasm, **oncology**, overall survival, pancreatitis, patient, progression free survival, safety, sepsis, **society**, **stomach adenocarcinoma**, stomach cancer, surgery, survival, survival rate, toxicity

Author Address

R.D. Kalachand.

Additional Information

Abbreviated Journal Title	J. Clin. Oncol.
ISSN	0732183X
Source Type	Journal
Conference Name	2013 Annual Meeting of the American Society of Clinical Oncology, ASCO
Conference Location	Chicago, IL, United States
Conference Date	2013-05-31 to 2013-06-04

Source Publication Date 2013-05-20
Entry Date 2013-07-04 (Full record)
Publication Type Conference Abstract
Page Range
Language of Article English
Language of Summary English

Related articles

View related articles on Embase.com

Copyright

Copyright 2013 Elsevier B.V., All rights reserved.

Records

20. **Experience with perioperative chemotherapy (ECC or ECF) for gastroesophageal cancer in British Columbia: A multicenter experience**

Lim H.J., Al-Barrak J., Cheung W.Y., Peixoto R.D.
Journal of Clinical Oncology 2013 **31:15 SUPPL. 1**
Embase

Abstract

Background: In 2006, the MAGIC trial demonstrated a significant overall survival benefit for perioperative ECF among patients with resectable gastro-esophageal cancer when compared to surgery alone. Since 2008, perioperative chemotherapy has become the standard of care for resectable gastro-esophageal cancer in our institution. We report the results of our experience with this protocol. Methods: The BC Cancer Agency is a multi-centre institution treating the majority of oncology patients for the province. A total of 83 consecutive patients with localized gastric, gastro-esophageal junction (GEJ) or lower esophageal cancer who initiated ECC/ECF perioperative chemotherapy from March 2008 to June 2011 were identified using the pharmacy database. Patient's characteristics were abstracted to an anonymous database and analyzed. Results: In our cohort, 83 patients (66 males and 17 females) with a median age of 62 years (range 37 - 79) began preoperative chemotherapy, of whom 73 (87.9%) completed 3 cycles. The response rate was 49.3%. Among the 83 patients, 78 (93.9%) underwent surgery (2 patients died of chemotherapy toxicities, 1 refused surgery and 2 developed disease progression before surgery), of whom 11

(14.1%) could not have their tumors resected (1 unresectable, 1 with liver metastasis and 9 with peritoneal carcinomatosis). Only one patient died of surgical complications. Six patients (7.69%) achieved pathologic complete response (ypCR) and all of them are alive and recurrence-free. Forty-eight patients (57.8%) subsequently began postoperative chemotherapy, of whom 37 (44.5%) completed 3 cycles. Thirty-nine patients have died as of December 4th, 2012. The observed median survival was 38 months. Conclusions: In our multi-center experience, the results reported in the MAGIC trial were reproduced.

Other works by authors of this record

Lim H.J., Al-Barrak J., Cheung W.Y., Peixoto R.D.

Emtree drug index terms

Emtree medical index terms

Canada, carcinomatosis, **chemotherapy**, data base, disease course, esophagus cancer, female, health care quality, human, liver metastasis, male, **neoplasm**, **oncology**, overall survival, patient, pharmacy, postoperative complication, **society**, surgery, survival, toxicity

Author Address

H.J. Lim.

Additional Information

Abbreviated Journal Title	J. Clin. Oncol.
ISSN	0732183X
Source Type	Journal
Conference Name	2013 Annual Meeting of the American Society of Clinical Oncology, ASCO
Conference Location	Chicago, IL, United States
Conference Date	2013-05-31 to 2013-06-04
Source Publication Date	2013-05-20
Entry Date	2013-07-04 (Full record)
Publication Type	Conference Abstract
Page Range	
Language of Article	English
Language of Summary	English

Related articles

View related articles on Embase.com

Copyright

Copyright 2013 Elsevier B.V., All rights reserved.

Records

21. **A retrospective analysis of the role of gefitinib in the definitive management of esophageal/gastroesophageal junction (E/GEJ) cancer**

Sohal D., Rice T.W., Rybicki L.A., Rodriguez C.P., Videtic G.M.M., Saxton J.P., Murthy S.C., Mason D.P., Phillips B.E., Tubbs R.R., Plesec T., McNamara M.J., Ives D.I., Bodmann J., Adelstein D.J.

Journal of Clinical Oncology 2013 **31:15 SUPPL. 1**

Embase

Abstract

Background: The role of epidermal growth factor receptor (EGFR) inhibition in resectable E/GEJ cancer is uncertain. We update and retrospectively compare results from two Cleveland Clinic trials of concurrent chemoradiotherapy (CCRT) and surgery; the second study differing only by the addition of gefitinib (G) to the treatment regimen. Methods: Eligibility required a diagnosis of E/GEJ squamous cell or adenocarcinoma (ACA), with an endoscopic ultrasound stage of at least T3, N1, or M1a (AJCC 6th). Patients (pts) in both trials received 5-FU (1000mg/m2/d) and cisplatin (20mg/m2/d) as continuous infusions over days 1-4 along with 30 Gy radiation at 1.5 Gy bid. Surgery followed in 4-6 weeks; identical CCRT was given 6-10 weeks later. The second trial added G, 250mg/d, on day 1 for 4 weeks, and again with postoperative CCRT for 2 years. Preliminary results and comparisons have been previously published. Results: Clinical characteristics were similar between the 80 pts on the G trial (2003-2006) and the 93 pts on the no-G trial (1999-2003): median age (58 vs. 59 years), male gender (91% vs. 86%), ACA (94% vs. 83%), and HER2 positivity (28% vs. 18%). Minimum follow-up for all pts was 5 years. Multivariable Cox analyses comparing the G vs. no-G pts and adjusting for statistically significant covariates demonstrated improved overall survival (HR 0.62, 95% CI 0.44-0.88, p=0.008), relapse-free survival (RFS) (HR 0.59, 95% CI 0.41-0.84, p=0.003), and distant metastatic recurrence (HR 0.64, 95% CI 0.43-0.96, p=0.03), but not

locoregional recurrence. Further subgroup analyses demonstrated improved RFS with G in pts not experiencing a pathologic response (HR=0.59, 95% CI 0.38-0.92, p=0.021), with T4 or M1a disease at surgery (HR=0.33, 95% CI 0.13-0.87, p=0.024), or with HER2-negative tumors (HR=0.65, 95% CI 0.42-0.99, p=0.048). Conclusions: Although this retrospective comparison can only be considered exploratory, it suggests that gefitinib may improve clinical outcomes when combined with CCRT and surgery in the definitive treatment of E/GEJ cancer. Clinical benefit may be greatest in pts with a poor response to preoperative chemoradiation, and in those with HER2-negative tumors.

Other works by authors of this record

Sohal D., Rice T.W., Rybicki L.A., Rodriguez C.P., Videtic G.M.M., Saxton J.P., Murthy S.C., Mason D.P., Phillips B.E., Tubbs R.R., Plesec T., McNamara M.J., Ives D.I., Bodmann J., Adelstein D.J.

Emtree drug index terms

cisplatin, epidermal growth factor receptor, fluorouracil, **gefitinib**

Emtree medical index terms

adenocarcinoma, chemoradiotherapy, continuous infusion, diagnosis, endoscopic echography, follow up, gender, hospital, human, male, **neoplasm**, **oncology**, overall survival, patient, radiation, recurrence free survival, **society**, squamous cell, surgery

Author Address

D. Sohal.

Additional Information

Abbreviated Journal Title	J. Clin. Oncol.
ISSN	0732183X
Source Type	Journal
Conference Name	2013 Annual Meeting of the American Society of Clinical Oncology, ASCO
Conference Location	Chicago, IL, United States
Conference Date	2013-05-31 to 2013-06-04
Source Publication Date	2013-05-20
Entry Date	2013-07-04 (Full record)
Publication Type	Conference Abstract
Page Range	

Language of Article English
Language of Summary English

Related articles

View related articles on Embase.com

Copyright

Copyright 2013 Elsevier B.V., All rights reserved.

Records

22. **Various clinical outcomes in patients with esophageal or gastroesophageal junction (E-GEJ) adenocarcinoma undergoing trimodality therapy: Prognostic implications**

Suzuki A., Xiao L., Taketa T., Sudo K., Blum M.A., Komaki R., Lee J.H., Bhutani M.S., Weston B., Hofstetter W.L., Swisher S., Ajani J.A.

Journal of Clinical Oncology 2013 **31:15 SUPPL. 1**
Embase

Abstract

Background: Preoperative chemoradiation (trimodality therapy) has the strongest evidence in trimodality-eligible patients with esophageal or gastroesophageal junction (E-GEJ) adenocarcinoma. Pathological complete response (pathCR) and clinical complete response (clinCR) are independent prognostic factors. We hypothesized that pathCR is associated with best prognosis. Methods: Patients with E-GEJ adenocarcinoma undergoing trimodality therapy were identified from the prospectively maintained database at our institution. The Log rank test, univariate and multivariate Cox proportional hazards regression analysis were applied for the survival analysis. Variables with p value < 0.15 in the univariate analysis were included in the multivariate analysis, the backward selection procedure was used for the model selection. Variables with P value < 0.05 were considered statistically significant. Results: For 314 esophageal cancer patients, the median follow-up time was 44.0 months (95% CI; 34.2-50.9). 107 of 314 patients died at this analysis. 80 patients (25.5%) had a pathCR. 160 patients (51.0%) had a clinCR prior to surgery but did not have pathCR. The remaining 74 (23.6%) had <pathCR and <clinCR. Median OS were: not achieved in pathCR patients, 82.8 months (95% CI; 63.9, NA) in clinCR patients and 27. 6 months (95% CI; 19.4, NA) <pathCR/<clinCR (p<0.001). The

median recurrence-free survival (RFS) were: 79.6 months (95% CI; 37.4, NA) in pathCR patients, 67.4 months (95% CI; 31.8, NA) in clinCR patients and 13.5 months (95% CI; 10.4, 21.4) in <pathCR/<clinCR ($p<0.001$). In multivariate analysis, no lymph node metastasis ($p<0.001$), not poorly differentiated adenocarcinoma ($p=0.002$) and pathCR ($p=0.02$), and cCR ($p<0.001$) were independent prognosticators of OS and RFS. Conclusions: pathCR and clinCR are associated with a better survival ourtcome compared to patients without pathCR/clinCRand may be helpful in devising new therapeutic and surveillance strategies.

Other works by authors of this record

Suzuki A., Xiao L., Taketa T., Sudo K., Blum M.A., Komaki R., Lee J.H., Bhutani M.S., Weston B., Hofstetter W.L., Swisher S., Ajani J.A.

Emtree drug index terms

Emtree medical index terms

adenocarcinoma, cancer patient, chemoradiotherapy, data base, esophagus cancer, follow up, **human**, log rank test, **lower esophagus sphincter**, lymph node metastasis, model, multivariate analysis, **oncology**, **patient**, procedures, prognosis, proportional hazards model, recurrence free survival, **society**, statistical significance, surgery, survival, **therapy**, univariate analysis

Author Address

A. Suzuki.

Additional Information

Abbreviated Journal Title	J. Clin. Oncol.
ISSN	0732183X
Source Type	Journal
Conference Name	2013 Annual Meeting of the American Society of Clinical Oncology, ASCO
Conference Location	Chicago, IL, United States
Conference Date	2013-05-31 to 2013-06-04
Source Publication Date	2013-05-20
Entry Date	2013-07-04 (Full record)
Publication Type	Conference Abstract
Page Range	
Language of Article	English

Language of Summary English

Related articles

View related articles on Embase.com

Copyright

Copyright 2013 Elsevier B.V., All rights reserved.

Records

23. **Various clinical outcomes in patients with esophageal or gastroesophageal junction (E-GEJ) adenocarcinoma undergoing trimodality therapy: Prognostic implications**

Suzuki A., Xiao L., Taketa T., Sudo K., Blum M.A., Komaki R., Liao Z.X., Lee J.H., Bhutani M.S., Weston B., Hofstetter W.L., Swisher S., Ajani J.A.

Journal of Clinical Oncology 2013 **31:4 SUPPL. 1**

Embase

Abstract

Background: Preoperative chemoradiation (trimodality therapy) has the strongest evidence in trimodality-eligible patients with E-GEJ adenocarcinoma. Pathological complete response (pathCR) and clinical complete response (clinCR) are favorable prognostic factors. We hypothesized that pathCR is associated with best prognosis. Methods: Patients with E-GEJ adenocarcinoma undergoing trimodality therapy were identified from the prospectively maintained databases at our institution. Multiple statistical methods were used. Results: For 314 esophageal cancer patients, the median follow-up time was 44.0 months (95% CI; 34.2-50.9). 107 of 314 patients died at this analysis. 80 patients (25.5%) had a pathCR. 160 patients (51.0%) had a clinCR prior to surgery but did not have pathCR. The remaining 74 (23.6%) had <pathCR and <clinCR. Median OS were: not achieved in pathCR patients, 82.8 months (95% CI; 63.9, NA) in clinCR patients and 27. 6 months (95% CI; 19.4, NA) <pathCR/<clinCR (p<0.001). The median recurrence-free survival (RFS) were: 79.6 months (95% CI; 37.4, NA) in pathCR patients, 67.4 months (95% CI; 31.8, NA) in clinCR patients and 13.5 months (95% CI; 10.4, 21.4) in <pathCR/<clinCR (p<0.001). In multivariate analysis, no lymph node metastasis (p<0.001), not poorly differentiated adenocarcinoma (p=0.002) and pathCR (p=0.02), and cCR (p<0.001) were independent prognosticators of OS and RFS. Conclusions: pathCR and clinCR are

independent prognosticators (pathCR producing the best results) and may be helpful in devising new therapeutic and surveillance strategies.

Other works by authors of this record

Suzuki A., Xiao L., Taketa T., Sudo K., Blum M.A., Komaki R., Liao Z.X., Lee J.H., Bhutani M.S., Weston B., Hofstetter W.L., Swisher S., Ajani J.A.

Emtree drug index terms

Emtree medical index terms

adenocarcinoma, cancer patient, chemoradiotherapy, data base, **digestive system cancer**, esophagus cancer, follow up, **human**, **lower esophagus sphincter**, lymph node metastasis, multivariate analysis, **patient**, prognosis, recurrence free survival, statistical analysis, surgery, **therapy**

Author Address

A. Suzuki. University of Texas, MD Anderson Cancer Center.

Additional Information

Abbreviated Journal Title	J. Clin. Oncol.
ISSN	0732183X
Source Type	Journal
Conference Name	2013 Gastrointestinal Cancers Symposium
Conference Location	San Francisco, CA, United States
Conference Date	2013-01-24 to 2013-01-26
Source Publication Date	2013-02-01
Entry Date	2013-06-18 (Full record)
Publication Type	Conference Abstract
Page Range	
Country of Author	United States
Language of Article	English
Language of Summary	English

Related articles

View related articles on Embase.com

Copyright

Copyright 2013 Elsevier B.V., All rights reserved.

Records

24. **Preoperative chemoradiotherapy for locally advanced gastric cancer**

Pepek J.M., Chino J.P., Willett C.G., Palta M., Blazer III D.G., Tyler D.S., Uronis H.E., Czito B.G.

Radiation Oncology 2013 **8:1 Article Number 6**

Embase MEDLINE

Go to publisher for the full text

Abstract

Background: To examine toxicity and outcomes for patients treated with preoperative chemoradiotherapy (CRT) for gastric cancer.Methods: Patients with gastroesophageal (GE) junction (Siewert type II and III) or gastric adenocarcinoma who underwent neoadjuvant CRT followed by planned surgical resection at Duke University between 1987 and 2009 were reviewed. Overall survival (OS), local control (LC) and disease-free survival (DFS) were estimated using the Kaplan-Meier method. Toxicity was graded according to the Common Toxicity Criteria for Adverse Events version 4.0.Results: Forty-eight patients were included. Most (73%) had proximal (GE junction, cardia and fundus) tumors. Median radiation therapy dose was 45 Gy. All patients received concurrent chemotherapy. Thirty-six patients (75%) underwent surgery. Pathologic complete response and R0 resection rates were 19% and 86%, respectively. Thirty-day surgical mortality was 6%. At 42 months median follow-up, 3-year actuarial OS was 40%. For patients undergoing surgery, 3-year OS, LC and DFS were 50%, 73% and 41%, respectively.Conclusions: Preoperative CRT for gastric cancer is well tolerated with acceptable rates of perioperative morbidity and mortality. In this patient cohort with primarily advanced disease, OS, LC and DFS rates in resected patients are comparable to similarly staged, adjuvantly treated patients in randomized trials. Further study comparing neoadjuvant CRT to standard treatment approaches for gastric cancer is indicated. © 2013 Pepek et al.; licensee BioMed Central Ltd.

Other works by authors of this record

Pepek J.M., Chino J.P., Willett C.G., Palta M., Blazer III D.G., Tyler D.S., Uronis H.E., Czito B.G.

Emtree drug index terms

capecitabine (adverse drug reaction), capecitabine (drug therapy), carboplatin (adverse drug reaction), carboplatin (drug therapy), cisplatin (adverse drug reaction), cisplatin (drug therapy), fluorouracil (adverse drug reaction), fluorouracil (drug therapy), methotrexate (adverse drug reaction), methotrexate (drug therapy), mitomycin (adverse drug reaction), mitomycin (drug therapy), paclitaxel (adverse drug reaction), paclitaxel (drug therapy)

Emtree medical index terms

adult, advanced cancer (drug therapy), advanced cancer (drug therapy), advanced cancer (radiotherapy), advanced cancer (surgery), aged, anemia (complication), anemia (side effect), article, cancer adjuvant therapy, cancer chemotherapy, cancer staging, cardia carcinoma, **chemoradiotherapy**, clinical article, cohort analysis, controlled study, dehydration (complication), dehydration (side effect), diarrhea (side effect), disease free survival, dysphagia (complication), dysphagia (side effect), external beam radiotherapy, female, follow up, human, laparotomy, leukopenia (complication), leukopenia (side effect), lower esophagus sphincter, male, medical record review, nausea (complication), nausea (side effect), outcome assessment, overall survival, partial gastrectomy, perforation (complication), perforation (side effect), preoperative care, radiation dose, radiation injury (complication), retrospective study, stomach adenocarcinoma (drug therapy), stomach adenocarcinoma (drug therapy), stomach adenocarcinoma (radiotherapy), stomach adenocarcinoma (surgery), stomach fundus, surgical mortality, thrombocytopenia (complication), thrombocytopenia (side effect), treatment planning, treatment response

Author Address

B.G. Czito. Department of Radiation Oncology, Duke University School of Medicine, Durham, NC, 27710.

Author Email

czito001@mc.duke.edu

Additional Information

Abbreviated Journal Title	Radiat. Oncol.
ISSN	1748717X (electronic)
Source Type	Journal
Source Publication Date	2013-01-04

Entry Date	2013-02-13 (Full record), 2013-01-09 (Article in Press/In process)
Publication Type	Article
Page Range	
Country of Author	United States
Country of Source	United Kingdom
Language of Article	English
Language of Summary	English
MEDLINE PMID	23286735
Embase Accession Number	2013057826
Article Number	6
Number of References	38
CAS Registry Numbers	capecitabine (154361-50-9)<br>carboplatin (41575-94-4)<br>cisplatin (15663-27-1, 26035-31-4, 96081-74-2)<br>fluorouracil (51-21-8)<br>methotrexate (15475-56-6, 59-05-2, 7413-34-5)<br>mitomycin (1404-00-8, 50-07-7, 74349-48-7)<br>paclitaxel (33069-62-4)

Related articles

View related articles on Embase.com

Copyright

MEDLINE® is the source for part of the citation data of this record

Records

25. **Radiotherapy of gastroesophageal junction cancer**

Sterzing F., Grenacher L., Debus J.

Recent Results in Cancer Research 2012 **196 (187-199)**

Embase MEDLINE

Go to publisher for the full text

Abstract

Adenocarcinomas of the gastroesophageal junction (GEJ) require multimodal treatment approaches to accomplish good local control and overall survival. While early T1/2 N0 tumors are treated with surgery alone, they are only found in a small subset of patients due to the lack of symptoms at this stage. Most of the tumors are detected in locally advanced stage where surgery alone results in disappointing outcome. Chemotherapy and/or chemoirradiation in the neoadjuvant setting are used to improve conditions for oncological surgery. They aim to achieve a downsizing with a pathological complete remission in the optimal case, improve R0 rates, and upfront treat microscopic metastatic tumor cells. The optimal neoadjuvant treatment approach - chemotherapy, chemoirradiation, or a multiphase approach of both - is yet unclear. Chemoirradiation can improve local control after incomplete surgery and is an important option for patients unfit for surgery. In addition, it enables symptom relief in a palliative setting, namely dysphagia, pain, or bleeding. While target volumes are very much standardized, new technologies as image-guided intensity-modulated radiotherapy (IG-IMRT) and particle therapy have the potential to improve the therapeutic window by minimizing toxicity. Challenges of the present and the future will be the combination of radiotherapy with other cytostatic drugs and modern targeted therapies. This should ideally be integrated into a multimodal setting that is able to identify risk groups according to predictive markers and tumor response, altogether leading to a personalized oncological approach. © 2012 Springer-Verlag Berlin Heidelberg.

Other works by authors of this record

Sterzing F., Grenacher L., Debus J.

Emtree drug index terms

bevacizumab (clinical trial), bevacizumab (drug combination), bevacizumab (drug therapy), capecitabine (clinical trial), capecitabine (drug combination), capecitabine (drug therapy), carboplatin (drug combination), carboplatin (drug therapy), cetuximab (clinical trial), cetuximab (drug combination), cetuximab (drug therapy), cisplatin (clinical trial), cisplatin (drug combination), cisplatin (drug therapy), docetaxel (clinical trial), docetaxel (drug combination), docetaxel (drug therapy), epirubicin (drug combination), epirubicin (drug therapy), fluorouracil (clinical trial), fluorouracil (drug combination), fluorouracil (drug therapy), irinotecan (clinical trial), irinotecan (drug combination), irinotecan (drug therapy), oxaliplatin (clinical trial), oxaliplatin (drug combination), oxaliplatin (drug therapy), paclitaxel (drug combination), paclitaxel (drug therapy)

Emtree medical index terms

advanced cancer, article, cancer adjuvant therapy, cancer palliative therapy, cancer prognosis, **cancer radiotherapy**, cancer recurrence, cancer therapy, chemoradiotherapy, comorbidity, continuous infusion, disease free survival, early cancer, esophageal adenocarcinoma (drug therapy), esophageal adenocarcinoma (radiotherapy), esophageal squamous cell carcinoma (drug therapy), esophageal squamous cell carcinoma (radiotherapy), esophagus cancer (drug therapy), esophagus cancer (drug therapy), esophagus cancer (radiotherapy), esophagus cancer (surgery), gastroesophageal junction adenocarcinoma (drug therapy), gastroesophageal junction adenocarcinoma (prevention), gastroesophageal junction adenocarcinoma (drug therapy), gastroesophageal junction cancer (drug therapy), gastroesophageal junction cancer (drug therapy), gastroesophageal junction cancer (radiotherapy), gastroesophageal junction cancer (surgery), human, image guided radiotherapy, induction chemotherapy, intensity modulated radiation therapy, **lower esophagus sphincter**, lower esophagus sphincter adenocarcinoma (drug therapy), lower esophagus sphincter adenocarcinoma (radiotherapy), lower esophagus sphincter adenocarcinoma (drug therapy), lymph node metastasis, multimodality cancer therapy, multiple cycle treatment, overall survival, priority journal, response guided therapy, salvage therapy, stomach adenocarcinoma (drug therapy), stomach adenocarcinoma (radiotherapy), treatment response

Author Address

F. Sterzing. Department of Radiation Oncology, 69120 Heidelberg.

Author Email

florian.sterzing@med.uni-heidelberg.de

Additional Information

Abbreviated Journal Title	Recent Results Cancer Res.
ISSN	00800015
ISBN	9783642316289 (volume)
CODEN	RRCRB
Source Type	Book Series
Editor	Otto F., Lutz M.P
Source Publication Date	2012
Entry Date	2012-12-11 (Full record), 2012-12-04 (Article in Press/In process)

Publication Type	Article
Page Range	187-199
Country of Author	Germany
Country of Source	United States
Language of Article	English
Language of Summary	English
MEDLINE PMID	23129375
Embase Accession Number	2012691952
Number of References	39
CAS Registry Numbers	bevacizumab (216974-75-3)<br>capecitabine (154361-50-9)<br>carboplatin (41575-94-4)<br>cetuximab (205923-56-4)<br>cisplatin (15663-27-1, 26035-31-4, 96081-74-2)<br>docetaxel (114977-28-5)<br>epirubicin (56390-09-1, 56420-45-2)<br>fluorouracil (51-21-8)<br>irinotecan (100286-90-6)<br>oxaliplatin (61825-94-3)<br>paclitaxel (33069-62-4)

Related articles

View related articles on Embase.com

Copyright

MEDLINE® is the source for part of the citation data of this record

Records

26. **Definitive chemoradiation for adenocarcinoma of the esophagus and gastroesophageal junction**

Wu A.J., Ilson D.H., Janjigian Y.Y., Gerber N., Remis M., Rizk N.P., Goodman K.A.

International Journal of Radiation Oncology Biology Physics 2012 **84:3 SUPPL. 1 (S311)**

Embase

Go to publisher for the full text

Abstract

Purpose/Objective(s): Definitive chemoradiation (CRT) without surgery is a standard treatment for esophageal cancer, particularly squamous cell carcinoma where studies indicate no survival benefit to surgery after CRT. However, less is known about nonoperative therapy for adenocarcinoma (AC) of the esophagus and gastroesophageal junction (GEJ), raising the question whether those achieving clinical complete response (cCR) after CRT require surgical resection. We reviewed our experience to define survival and local failure after CRT alone for patients with esophageal adenocarcinoma. Materials/Methods: Patients undergoing CRT for nonmetastatic AC of the esophagus and GEJ between 2007 and 2010 were reviewed. Clinical data were available for 61 patients who did not develop distant metastases during CRT and did not go on to planned surgery. Seventy percent of patients were inoperable due to medical comorbidities (n Z 41) or anatomically unresectable disease (n = 2), while 18 (30%) declined surgery after cCR. Median age was 76 (range, 49-93). Most patients (59%) had tumor extending into the GEJ. Fifty-three (87%) had clinical T3+ or node-positive disease. Median radiation dose was 50.4 Gy (36-59.4 Gy). Induction chemotherapy preceded CRT in 46 patients (75%). All were treated with IMRT (67%) or 3D-CRT (33%). Regular surveillance endoscopy with biopsy was performed to assess local failure (LF). Follow-up was calculated from the last day of radiation. Results: Median follow-up was 21 months. Fifty patients (82%) had cCR after CRT: 44 had negative endoscopy and biopsy; 6 had complete PET-CT response but did not undergo endoscopy. Eleven patients (18%) had locally persistent disease (PD). cCR patients had median survival of 35 months with 2 and 3-year OS of 74% and 48%. PD patients had a median survival of 8 months and no survivors at 2 years; this difference was highly significant ($p < 0.001$). Thirty-five cCR patients (57%) developed LF, and 2-year local control was 50%. There was a trend towards improved survival with induction chemotherapy (2-yr OS 65% vs. 48%; $p = 0.07$). At 2 years, 60% of cCR patients were free of distant metastases. Eight patients who developed isolated LF underwent salvage with surgery (n = 5), brachytherapy (2) or laser (1); 6 of these are still alive and 5 are disease-free. Conclusions: Patients with cCR after CRT had relatively good survival, suggesting that omitting surgery is a reasonable strategy for elderly and medically inoperable patients. However, over half developed LF, indicating a need for better predictors of pathologic CR, and perhaps for radiation dose escalation in selected patients. Patients able to undergo salvage therapy for isolated LF appear to do well. Novel approaches are needed for medically

inoperable patients with PD after CRT, given their poor outcome.

Other works by authors of this record

Wu A.J., Ilson D.H., Janjigian Y.Y., Gerber N., Remis M., Rizk N.P., Goodman K.A.

Emtree drug index terms

Emtree medical index terms

adenocarcinoma, aged, biopsy, brachytherapy, **chemoradiotherapy**, clinical study, distant metastasis, endoscopy, esophageal adenocarcinoma, **esophagus**, esophagus cancer, follow up, human, induction chemotherapy, intensity modulated radiation therapy, laser, **lower esophagus sphincter**, neoplasm, **oncology**, patient, radiation, radiation dose, radiation dose escalation, salvage therapy, **society**, squamous cell carcinoma, surgery, survival, survivor, therapy

Author Address

A.J. Wu. Memorial Sloan-Kettering Cancer Center.

Additional Information

Abbreviated Journal Title	Int. J. Radiat. Oncol. Biol. Phys.
ISSN	03603016
Source Type	Journal
Conference Name	54th Annual Meeting of the American Society for Radiation Oncology, ASTRO 2012
Conference Location	Boston, MA, United States
Conference Date	2012-10-28 to 2012-10-31
Source Publication Date	2012-11-01
Entry Date	2012-11-10 (Full record)
Publication Type	Conference Abstract
Page Range	S311
Country of Author	United States
Language of Article	English
Language of Summary	English
Publisher Item Identifier	S0360301612017579

Related articles

View related articles on Embase.com

Copyright

Copyright 2012 Elsevier B.V., All rights reserved.

Records

27. **Perioperative chemotherapy with docetaxel, cisplatin and capecitabine (DCX) in gastro-oesophageal adenocarcinoma: A phase II study of the Arbeitsgemeinschaft Internistische Onkologie (AIO)**

Thuss-Patience P.C., Hofheinz R.D., Arnold D., Florschütz A., Daum S., Kretzschmar A., Mantovani-Löffler L., Bichev D., Breithaupt K., Kneba M., Schumacher G., Glanemann M., Schlattmann P., Reichardt P., Gahn B.

Annals of Oncology 2012 **23:11 (2827-2834) Article Number mds129**

Embase MEDLINE

Go to publisher for the full text

Abstract

Background: This prospective multicentre phase II trial assessed the feasibility and efficacy of perioperative chemotherapy with docetaxel, cisplatin and capecitabine (DCX) in patients with gastro-oesophageal adenocarcinoma. Methods: Patients with curatively resectable adenocarcinoma of the stomach, the gastro-oesophageal junction or the lower third of the oesophagus were enrolled. Patients received docetaxel 75 mg/m. ^{2} plus cisplatin 60 mg/m. ^{2} (day 1), followed by oral capecitabine 1875 mg/m. ^{2} divided into two doses (days 1-14) every 3 weeks. There were three cycles preoperatively and three cycles postoperatively. The primary end point was the R0 resection rate. Results: Fifty-one patients were recruited and assessed for feasibility and efficacy. 94.1% of patients received all three planned cycles preoperatively, and 52.9% received three cycles postoperatively. The R0 resection rate was 90.2%. 13.7% of patients showed complete pathological remission (pCR). Toxicity was acceptably tolerable. Without prophylactic granulocyte colony-stimulating factor administration, neutropenic fever developed in 21.5% of patients preoperatively (grade 3 or 4) and in 11.1% of patients postoperatively. Conclusions: DCX is a safe and feasible perioperative regimen in the treatment of gastro-oesophageal adenocarcinoma with a high percentage of cycles delivered pre- and postoperatively, compared with standard practice. The high efficacy in terms of R0 resection rate and pCR is very promising. © The Author 2012. Published by Oxford University Press on

behalf of the European Society for Medical Oncology. All rights reserved.

Other works by authors of this record

Thuss-Patience P.C., Hofheinz R.D., Arnold D., Florschütz A., Daum S., Kretzschmar A., Mantovani-Löffler L., Bichev D., Breithaupt K., Kneba M., Schumacher G., Glanemann M., Schlattmann P., Reichardt P., Gahn B.

Emtree drug index terms

capecitabine (adverse drug reaction), capecitabine (clinical trial), capecitabine (drug combination), capecitabine (drug therapy), capecitabine (oral drug administration), cisplatin (adverse drug reaction), cisplatin (clinical trial), cisplatin (drug combination), cisplatin (drug therapy), cisplatin (intravenous drug administration), docetaxel (adverse drug reaction), docetaxel (clinical trial), docetaxel (drug combination), docetaxel (drug therapy), docetaxel (intravenous drug administration), granulocyte colony stimulating factor

Emtree medical index terms

adult, aged, alopecia (side effect), anemia (side effect), anorexia (side effect), article, asthenia (side effect), cancer combination chemotherapy, cancer surgery, catheter infection (side effect), chemotherapy induced emesis (side effect), dehydration (side effect), diarrhea (side effect), dizziness (side effect), drug efficacy, drug hypersensitivity (side effect), drug safety, edema (side effect), esophageal adenocarcinoma (drug therapy), esophageal adenocarcinoma (drug therapy), esophageal adenocarcinoma (surgery), event free survival, falling, febrile neutropenia (side effect), female, fever (side effect), gastroesophageal adenocarcinoma (drug therapy), gastroesophageal adenocarcinoma (drug therapy), gastroesophageal adenocarcinoma (surgery), gastrointestinal hemorrhage (side effect), hand foot syndrome (side effect), head injury (side effect), hearing impairment (side effect), human, leukopenia (side effect), **lower esophagus sphincter**, lung embolism (side effect), major clinical study, male, multicenter study, multiple cycle treatment, nail, nausea (side effect), neurotoxicity (side effect), neutropenia (side effect), overall survival, pancreatitis (side effect), paresthesia (side effect), perforation (side effect), **perioperative chemotherapy**, perioperative period, phase 2 clinical trial, priority journal, prospective study, side effect (side effect), stomach adenocarcinoma (drug therapy), stomach adenocarcinoma (drug therapy), stomach adenocarcinoma (surgery), stomatitis (side effect), survival rate, taste, thrombocytopenia (side effect), thrombosis (side effect), tumor regression, visual disorder (side effect)

Author Address

P.C. Thuss-Patience. Department of Haematology, Oncology and Tumorimmunology, Charité -University Medicine Berlin, Campus Virchow-Klinikum, 13353 Berlin.

Author Email

peter.thuss@charite.de

Additional Information

Abbreviated Journal Title	Ann. Oncol.
ISSN	09237534, 15698041 (electronic)
CODEN	ANONE
Source Type	Journal
Source Publication Date	November 2012
Entry Date	2012-11-15 (Full record), 2012-11-07 (Article in Press/In process)
Publication Type	Article
Page Range	2827-2834
Country of Author	Germany
Country of Source	United Kingdom
Language of Article	English
Language of Summary	English
MEDLINE PMID	22734012
Embase Accession Number	2012639284
Article Number	mds129
Number of References	35
CAS Registry Numbers	capecitabine (154361-50-9)<br>cisplatin (15663-27-1, 26035-31-4, 96081-74-2)<br>docetaxel (114977-28-5)
Clinical Trial Numbers	ClinicalTrials.gov (NCT00865982)

Related articles

View related articles on Embase.com

Copyright

MEDLINE® is the source for part of the citation data of this record

Records

28. **Current developments in the management of locally advanced esophageal cancer**

McNamara M.J., Adelstein D.J.

Current Oncology Reports 2012 **14:4 (342-349)**

Embase MEDLINE

Go to publisher for the full text

Abstract

Loco-regionally advanced esophageal cancer is a lethal disease with poor outcomes despite aggressive multimodality therapy. The appropriate management of these patients is contentious and no single standard of care has been defined. Literature suggests that preoperative chemoradiotherapy may be superior to preoperative chemotherapy. Recently, several developments have impacted the care of these patients. The 2010 AJCC TNM staging system now recognizes the biologic heterogeneity of the disease and stages adenocarcinoma and squamous cell carcinoma separately. Studies suggest potentially less toxic chemotherapeutic agents including oxaliplatin may be useful in the management of this disease. FDG-PET imaging appears to have prognostic value and may predict for pathologic response. In addition, several trials have explored inhibition of the ErbB1 (EGFR) and ErbB2 (Her2) receptors. The monoclonal antibody trastuzumab appears to extend survival for patients with metastatic gastric and gastroesophageal junction adenocarcinoma and is under investigation for use in patients with locoregionally advanced disease. © Springer Science+Business Media, LLC 2012.

Other works by authors of this record

McNamara M.J., Adelstein D.J.

Emtree drug index terms

carboplatin (clinical trial), carboplatin (drug therapy), cetuximab (clinical trial), cetuximab (drug combination), cetuximab (drug therapy), cisplatin (adverse drug reaction), cisplatin (drug combination), cisplatin (drug comparison), cisplatin (drug therapy), docetaxel (drug combination), docetaxel (drug therapy), epidermal growth factor receptor (endogenous compound), epidermal growth factor receptor 2 (endogenous compound), erlotinib (drug combination), erlotinib (drug therapy), etoposide (drug therapy), fluorodeoxyglucose, fluorouracil (clinical trial), fluorouracil

(drug combination), fluorouracil (drug therapy), folinic acid (clinical trial), folinic acid (drug combination), folinic acid (drug therapy), gefitinib (adverse drug reaction), gefitinib (drug therapy), oxaliplatin (adverse drug reaction), oxaliplatin (clinical trial), oxaliplatin (drug combination), oxaliplatin (drug comparison), oxaliplatin (drug therapy), paclitaxel (clinical trial), paclitaxel (drug therapy), trastuzumab (drug therapy)

Emtree medical index terms

adenosarcoma, advanced cancer (drug therapy), advanced cancer (diagnosis), advanced cancer (drug therapy), advanced cancer (radiotherapy), advanced cancer (surgery), article, cancer adjuvant therapy, cancer combination chemotherapy, cancer prognosis, cancer radiotherapy, cancer risk, cancer staging, cancer surgery, cancer survival, chemoradiotherapy, continuous infusion, diarrhea (side effect), drug efficacy, esophageal adenocarcinoma (drug therapy), esophageal adenocarcinoma (radiotherapy), esophageal adenocarcinoma (surgery), esophageal squamous cell carcinoma (diagnosis), esophageal squamous cell carcinoma (drug therapy), esophageal squamous cell carcinoma (surgery), esophagus cancer (drug therapy), esophagus cancer (diagnosis), esophagus cancer (drug therapy), esophagus cancer (radiotherapy), esophagus cancer (surgery), esophagus resection, human, induction chemotherapy, low drug dose, metastasis (drug therapy), multiple cycle treatment, mutational analysis, neurotoxicity (side effect), outcome assessment, overall survival, positron emission tomography, preoperative period, progression free survival, protein expression, risk assessment, sensitivity analysis, stomach adenocarcinoma (drug therapy)

Author Address

M.J. McNamara. Cleveland Clinic Foundation, Taussig Cancer Institute, Cleveland, OH 44195.

Author Email

mcnamam@ccf.org

Additional Information

Abbreviated Journal Title	Curr. Oncol. Rep.
ISSN	15233790, 15346269 (electronic)
CODEN	CORUA
Source Type	Journal
Source Publication Date	August 2012

Entry Date	2012-09-17 (Full record), 2012-05-07 (Article in Press/In process)
Publication Type	Article
Page Range	342-349
Country of Author	United States
Country of Source	United Kingdom
Language of Article	English
Language of Summary	English
MEDLINE PMID	22544559
Embase Accession Number	2012513698
Number of References	45
CAS Registry Numbers	carboplatin (41575-94-4)<br>cetuximab (205923-56-4)<br>cisplatin (15663-27-1, 26035-31-4, 96081-74-2)<br>docetaxel (114977-28-5)<br>epidermal growth factor receptor (79079-06-4)<br>epidermal growth factor receptor 2 (137632-09-8)<br>erlotinib (183319-69-9, 183321-74-6)<br>etoposide (33419-42-0)<br>fluorodeoxyglucose (29702-43-0)<br>fluorouracil (51-21-8)<br>folinic acid (58-05-9)<br>gefitinib (184475-35-2, 184475-55-6, 184475-56-7)<br>oxaliplatin (61825-94-3)<br>paclitaxel (33069-62-4)<br>trastuzumab (180288-69-1)

Related articles

View related articles on Embase.com

Copyright

MEDLINE® is the source for part of the citation data of this record

Records

29. **Phase 2 trial of induction and concurrent chemoradiotherapy with weekly**

irinotecan and cisplatin followed by surgery for esophageal cancer

Ilson D.H., Minsky B.D., Ku G.Y., Rusch V., Rizk N., Shah M., Kelsen D.P., Capanu M., Tang L., Campbell J., Bains M.

Cancer 2012 **118:11 (2820-2827)**

Embase MEDLINE

Go to publisher for the full text

Abstract

BACKGROUND: Preoperative chemoradiation improves survival in esophageal and gastroesophageal junction (GEJ) cancer. We evaluated irinotecan and cisplatin as induction chemotherapy followed by concurrent chemoradiation in esophageal cancer. METHODS: Patients with uT1N1M0 or uT2-4NanyM0 resectable squamous cancer or adenocarcinoma of the esophagus or GEJ received irinotecan 65 mg/m ^{2} and cisplatin 30 mg/m^2 for 4 treatments in weeks 1 through 5, followed by 4 treatments in weeks 7 through 11 with 50.4 Gy in daily fractions, followed by surgery. The primary endpoint was pathologic complete response (pCR). Positron emission tomography (PET) scan was performed prior to chemotherapy and as restaging prior to radiotherapy. RESULTS: Fifty-five patients were evaluable, 75% of whom had adenocarcinoma and 65% of whom had uT3N1 disease. Thirty-eight patients underwent R0 resection (69%). The incidence of pCR was 16% (95% confidence interval, 8%-29%). Median overall survival was 31.7 months. An exploratory analysis of PET response to induction chemotherapy indicated a correlation with pCR (32% vs 4%), R0 resection (84% vs 57%), progression-free survival (24.1 vs 7.7 months), and overall survival (40.2 vs 25.5 months). CONCLUSIONS: Weekly treatment with irinotecan, cisplatin, and radiation achieved results no better and potentially inferior to other phase 2 chemoradiotherapy trials with a low rate of pCR. The use of PET scan after induction chemotherapy to direct chemotherapy during subsequent radiotherapy merits further study. Copyright © 2011 American Cancer Society.

Other works by authors of this record

Ilson D.H., Minsky B.D., Ku G.Y., Rusch V., Rizk N., Shah M., Kelsen D.P., Capanu M., Tang L., Campbell J., Bains M.

Emtree drug index terms

cisplatin (adverse drug reaction), cisplatin (clinical trial), cisplatin (drug dose), cisplatin (drug therapy), irinotecan (adverse drug reaction), irinotecan (clinical trial), irinotecan

(drug dose), irinotecan (drug therapy)

Emtree medical index terms

abdominal pain (side effect), adult, aged, alopecia (side effect), anemia (side effect), anorexia (side effect), article, cancer chemotherapy, cancer survival, chemoradiotherapy, chemotherapy induced emesis (side effect), clinical evaluation, constipation (side effect), dehydration (side effect), diarrhea (side effect), drug dose reduction, drug efficacy, drug safety, esophagitis (side effect), esophagus cancer (drug therapy), esophagus cancer (drug therapy), esophagus cancer (radiotherapy), esophagus cancer (surgery), fatigue (side effect), febrile neutropenia (side effect), female, heart arrhythmia (side effect), human, hyperbilirubinemia (side effect), hyponatremia (side effect), major clinical study, male, multiple cycle treatment, nausea (side effect), neuropathy (side effect), neutropenia (side effect), overall survival, phase 2 clinical trial, positron emission tomography, priority journal, progression free survival, salvage therapy, side effect (side effect), thrombocytopenia (side effect), thrombosis (side effect), treatment duration, treatment response, vein thrombosis (side effect)

Author Address

D.H. Ilson. Gastrointestinal Oncology Service, Department of Medicine, Memorial Sloan-Kettering Cancer Center, New York, NY 10065.

Author Email

ilsond@mskcc.org

Additional Information

Abbreviated Journal Title	Cancer
ISSN	0008543X, 10970142 (electronic)
CODEN	CANCA
Source Type	Journal
Source Publication Date	2012-06-01
Entry Date	2012-06-06 (Full record), 2011-10-15 (Article in Press/In process)
Publication Type	Article
Page Range	2820-2827
Country of Author	United States
Country of Source	United States
Language of Article	English

Language of Summary	English
MEDLINE PMID	21990000
Embase Accession Number	2012294975
Number of References	26
CAS Registry Numbers	cisplatin (15663-27-1, 26035-31-4, 96081-74-2)
	irinotecan (100286-90-6)

Related articles

View related articles on Embase.com

Copyright

MEDLINE® is the source for part of the citation data of this record

Records

30. **A phase II study of neoadjuvant therapy with cisplatin, docetaxel, panitumumab plus radiation therapy followed by surgery in patients with locally advanced adenocarcinoma of the distal esophagus (ACOSOG Z4051)**

Reed C.E., Decker P.A., Schefter T.E., Meyers B.F., Ferguson M.K., Oeltjen A.R., Putnam J.B., Cassivi S.D., Lockhart A.C.

Journal of Clinical Oncology 2012 **30:15 SUPPL. 1**

Embase

Abstract

Background: Multiple clinical trials have incorporated preoperative chemotherapy and radiation (RT) in an attempt to improve local tumor control, distant disease failure and overall survival rates for locally advanced but resectable adenocarcinoma of the distal esophagus and gastroesophageal junction (GEJ). This multicenter, cooperative group study combined active chemotherapy agents cisplatin (C), docetaxel (D) and the targeted EGFR agent panitumumab (P) in the induction phase followed by concurrent chemotherapy (CDP) and radiation. Pathologic complete response (pCR), a surrogate for improved survival, was the primary endpoint. Methods: From 01/15/09 to 07/22/11, 70 patients (pts) with Siewert I or II adenocarcinomas and clinical stages T3N0M0, T2-3N1M0 or T2-3N0-1M1a (celiac LN ≤ 2 cm) were accrued. Patients received cisplatin (40 mg/m^2), docetaxel (40 mg/m^2) and panitumumab (6 mg/kg) on weeks 1,

3, 5, 7, 9 with RT (5040 cGy, 180 cGy/day x 28d) beginning week 5. The decision rule had a 90% power with a 0.10 significance level to detect a pCR rate of at least 35%. Secondary objectives included near-pathologic complete response (near-pCR), toxicity, and overall and disease-free survival rates. Results: Five pts were ineligible. Of the remaining 65 pts (59 M, 6 F; median age 61), 12 pts did not undergo surgery (5 progressed, 4 refused, 3 other). Of the 58 evaluable pts, the pCR rate was 32.8% (90% CI: 22.6% -42.9%) and near-pCR 22.4% (90% CI: 13.4%-31.4%). Total doses of C, D, and P were achieved in 76%, 80%, and 73%, respectively (n = 70). 66 pts (94%) received the total RT dose. Sixteen pts (23%) had a grade 4+ non-heme adverse event possibly related to treatment. Venous thrombosis (5 pts) was most common. Conclusions: The CDP regimen in the neoadjuvant setting in patients with esophageal adenocarcinomas is active (pCR + near-pCR = 55.2%) and feasible. The toxicity though tolerable is substantial.

Other works by authors of this record

Reed C.E., Decker P.A., Schefter T.E., Meyers B.F., Ferguson M.K., Oeltjen A.R., Putnam J.B., Cassivi S.D., Lockhart A.C.

Emtree drug index terms

cisplatin, cytidine diphosphate, **docetaxel**, heme, **panitumumab**

Emtree medical index terms

adenocarcinoma, **adjuvant therapy**, cancer control, chemotherapy, clinical trial (topic), disease free survival, esophageal adenocarcinoma, **esophagus**, **human**, lower esophagus sphincter, **oncology**, overall survival, **patient**, **phase 2 clinical trial**, radiation, **radiotherapy**, **society**, **surgery**, survival, survival rate, toxicity, vein thrombosis

Author Address

C.E. Reed.

Additional Information

Abbreviated Journal Title	J. Clin. Oncol.
ISSN	0732183X
Source Type	Journal
Conference Name	2012 Annual Meeting of the American Society of Clinical Oncology, ASCO
Conference Location	Chicago, IL, United States

Conference Date	2012-06-01 to 2012-06-05
Source Publication Date	2012-05-20
Entry Date	2013-03-08 (Full record)
Publication Type	Conference Abstract
Page Range	
Language of Article	English
Language of Summary	English

Related articles

View related articles on Embase.com

Copyright

Copyright 2013 Elsevier B.V., All rights reserved.

Records

31. **NeoFLOT: Multicenter phase II study of perioperative chemotherapy in resectable adenocarcinoma of the gastroesophageal junction or gastric adenocarcinoma**

Schulz C., Kullmann F., Kunzmann V., Schepp W., Geissler M., Kaiser U.V., Stauder H., Wein A., Al-Batran S.-E., Kubin T., Schaefer C., Held S., Stintzing S., Boeck S.H., Haas M., Forstpointner R., Ridwelski K., Heinemann V.

Journal of Clinical Oncology 2012 **30:15 SUPPL. 1**

Embase

Abstract

Background: The rationale of the NeoFLOT-trial was the intensification of neoadjuvant chemotherapy (NACT) by prolongation of preoperative treatment. This strategy is based on the notion that while perioperative treatment is notably beneficial in locally advanced gastroesophageal cancer (GEC), postoperative chemotherapy can only be applied in a fraction of patients (pts). Methods: Pts with T3, T4 and/or N+ adenocarcinoma (GEC) were eligible for this multicenter phase II trial. NACT consisted of 6 cycles of oxaliplatin 85 mg/m^2, leucovorin 200 mg/m^2, fluorouracil 2600 mg/m^2 and docetaxel 50 mg/m^2 (FLOT) applied q 2 wks. Staging was performed after 3 cycles to select pts with progressive disease (PD) for immediate surgery. Primary endpoint was the R0-resection rate. Secondary endpoints included the pathological complete response rate (pCR), histologic tumor regression grade (Becker 2003),

safety, and progression-free survival (PFS). Results: From 10/2009 to 06/2011, 59 pts were enrolled of whom 58 pts were assessable for safety. Median age was 61 years (range: 32-79), 58.6% (34/58) had tumors of the gastroesophageal junction. 50 pts underwent surgery and were assessable for the primary endpoint. R0-resection rate was 86.0% (43/50). pCR was achieved in 20.0% (10/50) of pts. During NACT, 6.9% (4/58) of pts developed progressive disease. Dose reduction was performed in 43.1% (25/58) of pts resulting in a median dose intensity of 89.2%. Grade 3/4 neutropenia was observed in 29.3% (17/58) of pts, febrile neutropenia grade 3/4 in 1.7% (1/58). Common grade 3/4 non-hematologic adverse events were diarrhea (13.8% (8/58)) and mucositis (6.9% (4/58)). Treatment related mortality was 3.4% (2/58) with 2 cases of sepsis. After a median follow-up of 9.1 months, median PFS and OS have not been reached. Conclusions: These data indicate that NACT with 6 cycles FLOT is well-tolerated and highly effective in resectable GEC.

Other works by authors of this record

Schulz C., Kullmann F., Kunzmann V., Schepp W., Geissler M., Kaiser U.V., Stauder H., Wein A., Al-Batran S.-E., Kubin T., Schaefer C., Held S., Stintzing S., Boeck S.H., Haas M., Forstpointner R., Ridwelski K., Heinemann V.

Emtree drug index terms

docetaxel, fluorouracil, folinic acid, oxaliplatin

Emtree medical index terms

adenocarcinoma, adjuvant chemotherapy, **chemotherapy**, diarrhea, drug dose reduction, febrile neutropenia, follow up, **human**, **lower esophagus sphincter**, mortality, mucosa inflammation, neoplasm, neutropenia, **oncology**, patient, **phase 2 clinical trial**, preoperative treatment, progression free survival, safety, sepsis, **society**, staging, **stomach adenocarcinoma**, surgery, tumor regression

Author Address

C. Schulz.

Additional Information

Abbreviated Journal Title	J. Clin. Oncol.
ISSN	0732183X
Source Type	Journal
Conference Name	2012 Annual Meeting of the American Society of Clinical Oncology, ASCO

Conference Location	Chicago, IL, United States
Conference Date	2012-06-01 to 2012-06-05
Source Publication Date	2012-05-20
Entry Date	2013-03-08 (Full record)
Publication Type	Conference Abstract
Page Range	
Language of Article	English
Language of Summary	English

Related articles

View related articles on Embase.com

Copyright

Copyright 2013 Elsevier B.V., All rights reserved.

Records

32. **Targeted therapy in head/neck and gastric cancers**

Hsueh C.-T.

Journal of Hematology and Oncology 2012 **5 SUPPL. 1**

Embase

Abstract

The epidermal growth factor receptor (EGFR/HER1) is a member of the erbB family of receptor tyrosine kinase proteins, which also includes HER2, HER3, and HER4. EGFR is almost universally expressed in squamous cell carcinoma of head and neck (SCCHN), and high levels of expression have been correlated with a poor clinical prognosis [1]. Cetuximab, an IgG1 monoclonal antibody against EGFR, has demonstrated improved survival and disease control when used in combination with radiation therapy for the treatment of locally advanced SCCHN, and in combination with platinum-based chemotherapy in recurrent or metastatic SCCHN [2-4]. Additionally, single-agent cetuximab is active and provides good disease control rate and duration in platinum-refractory SCCHN [5]. Comparison of cetuximab versus cisplatin concurrently with radiotherapy is under investigation in patients with human papillomavirus-associated oropharyngeal cancer, who have better prognosis and may benefit from less toxic treatment [6]. Overexpression of HER2 in gastric cancer results in aggressive clinical course and poor prognosis [7]. Trastuzumab, a monoclonal

antibody against HER2, exhibits antitumor activity in HER2 overexpressed gastric cancer cells, and enhances effects of chemotherapy in gastric cancer xenograft overexpressing HER2 [8]. The ToGA study screened about 3,800 patients with advanced gastric cancer from 24 countries, and HER2 overexpression was detected in 22% [9]. Higher rates of HER2 overexpression occurred in intestinal and proximal or gastroesophageal junction cancers than in diffuse or distal gastric cancers. In TOGA study, 584 patients with HER2 overexpression were randomized to receive fluoropyrimidine and cisplatin treatment with or without trastuzumab. Patients who received trastuzumab plus chemotherapy achieved longer overall survival (13.8 months vs. 11.1 months, P= 0.0046), longer progression-free survival (6.7 months vs. 5.5 months, P=0.0002), and higher response rates (47% vs. 35%, P=0.0017) than those who received chemotherapy alone. Complete response was noted in 5.4% of patients receiving trastuzumab plus chemotherapy vs. 2.4% in chemotherapy alone. There were no significant differences in the toxicities between these two groups. This study has established a new paradigm using trastuzumab in combination with chemotherapy in patients with advanced gastric cancer overexpressing HER2. Neoadjuvant treatment is a standard of care for locally advanced esophageal and gastric cancer. We have previously reported a case of pathological complete response after neoadjuvant chemotherapy with trastuzumab-containing regimen in HER2-overexpressing gastric cancer [10]. Incorporating trastuzumab as a part of neoadjuvant therapy in esophageal and gastric adenocarcinoma overexpressing HER2 is currently under active investigation. Radiation Therapy Oncology Group is conducting a phase III neoadjuvant study in patients with HER2- overexpressing esophageal adenocarcinoma to determine if trastuzumab increases disease-free survival when added to chemoradiotherapy [11]. Other studies conducted in Europe are adding trastuzumab to oxaliplatinbased regimen as perioperative chemotherapy for HER2-overexpressing esophagogastric or gastric adenocarcinoma, and looking for improvement of pathological complete response and disease-free survival [12,13]. Pertuzumab is a monoclonal antibody interfering with HER2 dimerization with other HER receptors such as EGFR, HER3 and HER4. Pertuzumab and trastuzumab bind to HER2 at different sites, and combination of both antibodies leads to stronger inhibition of erbB signaling and greater therapeutic efficacy when combined with docetaxel in breast cancer [14]. Combination of pertuzumab and trastuzumab with platinum-based chemotherapy is currently studied in HER2-overexpressing gastric cancer [15].

Other works by authors of this record

Hsueh C.-T.

Emtree drug index terms

antibody, cetuximab, cisplatin, docetaxel, epidermal growth factor receptor, fluoropyrimidine, monoclonal antibody, pertuzumab, platinum, protein, protein tyrosine kinase, receptor, trastuzumab

Emtree medical index terms

adjuvant chemotherapy, adjuvant therapy, antineoplastic activity, breast cancer, cancer cell, chemoradiotherapy, chemotherapy, dimerization, disease control, disease course, disease free survival, esophageal adenocarcinoma, Europe, health care quality, **hematology**, human, lower esophagus sphincter, neck, neoplasm, **oncology**, oropharynx cancer, overall survival, patient, prognosis, progression free survival, radiotherapy, squamous cell carcinoma, stomach adenocarcinoma, **stomach cancer**, survival, **therapy**, toxicity, tumor xenograft, Wart virus

Author Address

C.-T. Hsueh. Loma Linda University.

Author Email

chsueh@llu.edu

Additional Information

Abbreviated Journal Title	J. Hematol. Oncol.
ISSN	17568722
Source Type	Journal
Conference Name	New Developments in Hematology and Oncology in 2011
Conference Location	Guangzhou, China
Conference Date	2011-12-25 to 2011-12-26
Conference Editor	Liu Q., Li Y., Liu D.
Source Publication Date	2012-04-25
Entry Date	2012-05-30 (Full record)
Publication Type	Conference Abstract
Page Range	
Country of Author	United States
Language of Article	English
Language of Summary	English

Related articles

View related articles on Embase.com

Copyright

Copyright 2012 Elsevier B.V., All rights reserved.

Records

33. **Phase II trial of preoperative chemoradiotherapy with oxaliplatin, cisplatin, and 5-FU in locally advanced esophageal and gastric cancer**

Pera M., Gallego R., Montagut C., Martín-richard M., Iglesias M., Conill C., Reig A., Balagué C., Pétriz L., Momblan D., Bellmunt J., Maurel J.

Annals of Oncology 2012 **23:3 (664-670)**

Embase MEDLINE

Go to publisher for the full text

Abstract

Background: Based on a phase I study showing the feasibility of combining of oxaliplatin, cisplatin, and 5-fluorouracil (5-FU) (OCF) with radiation therapy (RT) in esophageal cancer, the efficacy of this regimen in esophageal, gastroesophageal (GE), and gastric (G) cancer was assessed in this phase II multicenter study. Patients and methods: Patients with resectable tumors were eligible. Treatment included two cycles of oxaliplatin 85 mg/m. ^{2}, cisplatin 55 mg/m. ^{2}, and continuously infused 5-FU 3 g/m. ^{2} in 96 h and concurrent RT (45 Gy), followed by surgery after 6-8 weeks. Primary end point was complete pathologic response (pCR). Results: Forty-one patients were enrolled. Tumor location was esophagus 39% (squamous 10/adenocarcinoma 6), GE junction 32%, and stomach 29%. G3-G4 adverse events included asthenia (27%) and neutropenia (14%). One toxic death occurred. Thirty-one patients (75.6%) underwent surgery (R0 in 94%). Pathologic response was achieved in 58% of patients, with pCR in 50% and 16% of esophageal and GE/G cancer, respectively. pCR was achieved in 67% of squamous cell carcinoma. Survival: median follow-up, 50.4 months; median progression-free survival and overall survival were 23.2 and 28.4 months, respectively. Conclusion: Preoperative OCF plus RT showed an acceptable toxicity and promising activity especially in squamous cell esophageal cancer. © The Author 2011. Published by Oxford University Press on

behalf of the European Society for Medical Oncology. All rights reserved.

Other works by authors of this record

Pera M., Gallego R., Montagut C., Martín-richard M., Iglesias M., Conill C., Reig A., Balagué C., Pétriz L., Momblan D., Bellmunt J., Maurel J.

Emtree drug index terms

cisplatin (adverse drug reaction), cisplatin (clinical trial), cisplatin (drug combination), cisplatin (drug therapy), fluorouracil (adverse drug reaction), fluorouracil (clinical trial), fluorouracil (drug combination), fluorouracil (drug therapy), oxaliplatin (adverse drug reaction), oxaliplatin (clinical trial), oxaliplatin (drug combination), oxaliplatin (drug therapy)

Emtree medical index terms

adult, aged, anemia (side effect), article, asthenia (side effect), **cancer chemotherapy**, cancer localization, **cancer radiotherapy**, chemotherapy induced emesis (side effect), clinical article, continuous infusion, diarrhea (side effect), drug fatality (side effect), esophageal adenocarcinoma (drug therapy), esophageal adenocarcinoma (drug therapy), esophageal adenocarcinoma (radiotherapy), esophageal adenocarcinoma (surgery), esophagus disease (side effect), esophagus resection, fatigue (side effect), female, follow up, gastrectomy, histopathology, human, human tissue, infection (side effect), male, multicenter study, multiple cycle treatment, nausea (side effect), neutropenia (side effect), overall survival, phase 2 clinical trial, preoperative care, priority journal, progression free survival, sensory neuropathy (side effect), side effect (side effect), squamous cell carcinoma (drug therapy), squamous cell carcinoma (radiotherapy), squamous cell carcinoma (surgery), stomach adenocarcinoma (drug therapy), stomach adenocarcinoma (drug therapy), stomach adenocarcinoma (radiotherapy), stomach adenocarcinoma (surgery), stomatitis (side effect), thrombocytopenia (side effect), treatment response, weight reduction

Author Address

M. Pera. Section of Gastrointestinal Surgery, Hospital Universitario del Mar, 08003 Barcelona.

Author Email

pera@parcdesalutmar.cat

Additional Information

Abbreviated Journal Title	Ann. Oncol.
ISSN	09237534, 15698041 (electronic)
CODEN	ANONE
Source Type	Journal
Source Publication Date	March 2012
Entry Date	2012-03-16 (Full record), 2012-03-07 (Article in Press/In process)
Publication Type	Article
Page Range	664-670
Country of Author	Spain
Country of Source	United Kingdom
Language of Article	English
Language of Summary	English
MEDLINE PMID	21652581
Embase Accession Number	2012124105
Number of References	30
CAS Registry Numbers	cisplatin (15663-27-1, 26035-31-4, 96081-74-2)<br>fluorouracil (51-21-8)<br>oxaliplatin (61825-94-3)

Related articles

View related articles on Embase.com

Copyright

MEDLINE® is the source for part of the citation data of this record

Records

34. **Effect of preoperative chemoradiation in clinically resectable gastroesophageal cancer: Single institution experience**

Vosmik M., Sirak I., Dvorak J., Lesko M., Repak R., Kopacova M., Hodek M., Laco J., Melichar B., Petera J.

International Journal of Radiation Oncology Biology Physics 2011 **81:2 SUPPL. 1 (S329)**

Embase

Abstract

Purpose/Objective(s): Based on the results of the MAGIC trial, perioperative chemotherapy (CT) became a standard neoadjuvant treatment in adenocarcinomas of lower esophagus, gastroesophageal junction and stomach. Data on the effect of neoadjuvant chemoradiation (CRT) in these tumors are encouraging, but still limited. Materials/Methods: We retrospectively reviewed the results of preoperative CRT in patients (pts) with locally/regionally advanced, but clinically resectable gastroesophageal adenocarcinomas treated at our institution with curative intent and prescribed dose of radiotherapy (RT) ≥ 40 Gy. The response rate evaluation was based on comparison of pretreatment extent of the disease detected by endosonography and computer tomography and pathological findings in resected specimens. Toxicity was graded according to RTOG scale. Overall survival (OS) and disease-free survival (DFS) probability were calculated using the Kaplan- Meier method. Results: Between January 2001 and August 2010 seventy pts (13 females, 57 males, median age 62 years, range 28-80 years) underwent neoadjuvant concurrent CRT for adenocarcinoma of lower esophagus (19 pts), gastroesophageal junction (19 pts) or stomach (32 pts). Median prescribed dose was 45 Gy (range 40-50.4 Gy). Standard concurrent CT was fluorouracil in continual infusion (70 pts) +/- cisplatin in weekly regimen (48 pts) +/- paclitaxel or docetaxel in weekly regimen in 7 pts. Three patients did not finish RT because of bleeding, unexpected significant skin reaction and hematologic toxicity Grade 4. The concurrent CT administration was discontinued in 25 pts, in most cases due to hematologic toxicity. Severe acute toxicity (Grade 3-4) was hematologic (12 pts), skin (1 patient) and gastrointestinal (2 pts). Two deaths were noted for postoperative complications. Fifty two pts underwent curative surgery (74%), although in 3 cases the resection was R1. In 17 cases the tumor extent or peritoneal dissemination disabled a curative surgery. One patient refused the surgery. Pathologic findings were following: complete remission in 12 cases (17 %), microscopic residuals with regressive changes in 3 cases (4 %), microscopic residuals with vital tumor cells in 14 cases (20 %), partial response in size in 17 cases (23 %) and stable disease in 6 cases (9 %). Overall response rate was 66 %. Twenty pts were treated by subsequent adjuvant CT (mostly pts with primary gastric tumor). For survival evaluation, median follow-up of surviving pts was 38 months, 3-years DFS and OS probability was 37 % and 43 %. Conclusions: Preoperative CRT in gastroesophageal adenocarcinomas is well tolerated treatment with significant percentage of responders. Phase III trial is needed for comparison of neoadjuvant CRT with perioperative CT.

Other works by authors of this record

Vosmik M., Sirak I., Dvorak J., Lesko M., Repak R., Kopacova M., Hodek M., Laco J., Melichar B., Petera J.

Emtree drug index terms

adjuvant, cisplatin, docetaxel, fluorouracil, paclitaxel

Emtree medical index terms

acute toxicity, adenocarcinoma, bleeding, **chemoradiotherapy**, chemotherapy, computer assisted tomography, death, disabled person, disease free survival, endoscopic echography, esophagus, female, follow up, human, infusion, Kaplan Meier method, lower esophagus sphincter, male, **neoplasm**, neoplasm, **oncology**, overall survival, patient, phase 3 clinical trial, postoperative complication, radiotherapy, remission, skin, skin manifestation, **society**, stomach, stomach tumor, surgery, survival, survival rate, toxicity, tumor cell

Author Address

M. Vosmik. Charles University, Medical School, Teaching Hospital.

Additional Information

Abbreviated Journal Title	Int. J. Radiat. Oncol. Biol. Phys.
ISSN	03603016
Source Type	Journal
Conference Name	53rd Annual Meeting of the American Society for Radiation Oncology, ASTRO 2011
Conference Location	Miami Beach, FL, United States
Conference Date	2011-10-02 to 2011-10-06
Source Publication Date	2011-10-01
Entry Date	2012-01-30 (Full record)
Publication Type	Conference Abstract
Page Range	S329
Country of Author	Czech Republic
Language of Article	English
Language of Summary	English

Related articles

View related articles on Embase.com

Copyright

Copyright 2012 Elsevier B.V., All rights reserved.

Records

35. **Toxicity data for preoperative concurrent chemoradiotherapy with oxaliplatin and continuous infusion 5-fluorouracil for locally advanced esophageal cancer**

Thukral A., Metz J., Hwang W.-T., O'Dwyer P., Plastaras J., Both S., Ad V.B.

Diseases of the Esophagus 2011 **24:5 (330-336)**

Embase

Go to publisher for the full text

Abstract

The purpose of this retrospective analysis was to characterize the feasibility and tolerability of oxaliplatin/5-fluorouracil (5-FU) given concurrently with radiotherapy for patients with locally advanced esophageal cancer. Between July 2005 and March 2009, 15 patients with clinical stage T3/T4 and/or N1/M1a lower esophageal or gastroesophageal junction adenocarcinoma were treated with preoperative chemoradiotherapy using oxaliplatin every 2 weeks and continuous infusion 5-FU. The main treatment-related toxicities were oral mucositis and dysphagia. During the first 2 weeks of treatment, 20% of patients presented with grade 1-2 oral mucositis, and one patient developed grade 1 dysphagia. In weeks 3-4, 53% of the patients experienced grade 1-2 mucositis, and 40% experienced grade 1-2 dysphagia. One patient only experienced grade 3 mucositis in week 4. Three patients (20%) had grade 3-4 dysphagia in weeks 3-4 and were continued on intravenous fluids and pain medications. During the last 2 weeks of chemoradiotherapy, 53% of patients reported grade 1-2 oral mucositis, mostly grade 1 and 73% of patients experienced grade 1-2 dysphagia and 26% patients experienced grade 3-4 dysphagia. Other toxicities included fatigue, nausea, neuropathy, and diarrhea. Only one patient experienced > 10% weight loss. The whole group was treated with aggressive supportive care during radiotherapy. Five (33%) patients achieved a pathological complete response. No patients developed locoregional failure. Sixty percent of the patients developed distant metastases and the 2-year disease-free survival was 53%. The median survival was 3.2 years with the 2-year overall survival of 73%. Preoperative

oxaliplatin/5-FU-based chemoradiotherapy for locally advanced esophageal cancer is feasible, but associated with substantial gastrointestinal toxicity. A careful attention to nutrition and hydration throughout the course of therapy is required. © 2010 Copyright the Authors. Journal compilation © 2010, Wiley Periodicals, Inc. and the International Society for Diseases of the Esophagus.

Other works by authors of this record

Thukral A., Metz J., Hwang W.-T., O'Dwyer P., Plastaras J., Both S., Ad V.B.

Emtree drug index terms

antidiarrheal agent (drug therapy), fluorouracil (adverse drug reaction), fluorouracil (drug combination), fluorouracil (drug therapy), infusion fluid, oxaliplatin (adverse drug reaction), oxaliplatin (drug combination), oxaliplatin (drug therapy)

Emtree medical index terms

adult, advanced cancer (drug therapy), advanced cancer (drug therapy), advanced cancer (radiotherapy), advanced cancer (surgery), aged, article, cancer combination chemotherapy, cancer grading, cancer radiotherapy, cancer staging, cancer surgery, cancer survival, **chemoradiotherapy**, continuous infusion, diarrhea (drug therapy), diarrhea (side effect), disease free survival, distant metastasis, drug tolerability, dysphagia (side effect), esophageal adenocarcinoma (drug therapy), esophageal adenocarcinoma (drug therapy), esophageal adenocarcinoma (radiotherapy), esophageal adenocarcinoma (surgery), esophagus resection, fatigue (side effect), feasibility study, female, human, hypoalbuminemia (side effect), lower esophagus sphincter adenocarcinoma (drug therapy), lower esophagus sphincter adenocarcinoma (drug therapy), lower esophagus sphincter adenocarcinoma (radiotherapy), lower esophagus sphincter adenocarcinoma (surgery), major clinical study, malaise (side effect), male, mucosa inflammation (side effect), nausea (side effect), neuropathy (side effect), preoperative period, priority journal, retrospective study, side effect (side effect), thrombocytopenia (side effect), treatment response, weight reduction

Author Address

A. Thukral. Department of Radiation Oncology, University of Pennsylvania, Philadelphia, PA 19104.

Author Email

thukral@uphs.upenn.edu

Additional Information

Abbreviated Journal Title	Dis. Esophagus
ISSN	11208694, 14422050 (electronic)
CODEN	DIESE
Source Type	Journal
Source Publication Date	July 2011
Entry Date	2011-08-02 (Full record), 2011-07-28 (Article in Press/In process)
Publication Type	Article
Page Range	330-336
Country of Author	United States
Country of Source	Australia
Language of Article	English
Language of Summary	English
Embase Accession Number	2011402303
Number of References	21
CAS Registry Numbers	fluorouracil (51-21-8)<br>oxaliplatin (61825-94-3)

Related articles

View related articles on Embase.com

Copyright

Copyright 2011 Elsevier B.V., All rights reserved.

Records

36. **Preoperative taxane-based chemotherapy and celecoxib for carcinoma of the esophagus and gastroesophageal junction: Results of a phase 2 trial**

Altorki N.K., Christos P., Port J.L., Lee P.C., Mirza F., Spinelli C., Keresztes R., Beneck D., Paul S., Stiles B.M., Zhang Y., Schrump D.S.

Journal of Thoracic Oncology 2011 **6:6 (1121-1127)**

Embase MEDLINE

Go to publisher for the full text

Abstract

Purpose: The primary objective of this study was to determine the rate of pathological response after preoperative celecoxib and concurrent taxane-based chemotherapy in patients with cancer of the esophagus and gastroesophageal junction. Methods: Thirty-nine patients were enrolled in this single-arm, phase II clinical trial. Patients were administered daily celecoxib in combination with two to three cycles of carboplatin and paclitaxel with preoperative intent. Levels of cyclooxygenase (COX)-2 expression in resected tumors were analyzed by immunohistochemistry and correlated with clinical outcome measures. Postoperatively, patients were administered daily celecoxib for 1 year or until documented tumor recurrence. Results: All patients received two to three cycles of chemotherapy plus celecoxib 800 mg/d. Toxicities were as expected. A major clinical response (complete response + partial response) was noted in 22 patients (56%); six patients (15%) had a complete clinical response. Thirty-seven patients underwent esophagectomy. Five patients had a major pathological response (12.8%). Four-year overall and disease-free survivals were 40.9% and 30.3%, respectively. Patients with tumors expressing COX-2 demonstrated a higher likelihood of a major clinical response response (62% versus 50%) and an improved overall survival, compared with patients with COX-2-negative tumors. Conclusions: Preoperative celecoxib with concurrent chemotherapy demonstrated sufficient effect on pathologic response to warrant further study. Patients with tumors expressing COX-2 demonstrated trends toward improved response to preoperative therapy and improved overall survival compared with nonexpressors. Copyright © 2011 by the International Association for the Study of Lung Cancer.

Other works by authors of this record

Altorki N.K., Christos P., Port J.L., Lee P.C., Mirza F., Spinelli C., Keresztes R., Beneck D., Paul S., Stiles B.M., Zhang Y., Schrump D.S.

Emtree drug index terms

carboplatin (adverse drug reaction), carboplatin (clinical trial), carboplatin (drug combination), carboplatin (drug therapy), carboplatin (drug toxicity), carboplatin (intravenous drug administration), carboplatin (pharmacology), celecoxib (adverse drug reaction), celecoxib (clinical trial), celecoxib (drug combination), celecoxib (drug therapy), celecoxib (drug toxicity), celecoxib (oral drug administration), celecoxib (pharmacology), cyclooxygenase 2 (endogenous compound), dexamethasone, diphenhydramine, fluorodeoxyglucose f 18, histamine H2 receptor antagonist, paclitaxel (adverse drug reaction), paclitaxel (clinical trial), paclitaxel (drug

combination), paclitaxel (drug therapy), paclitaxel (drug toxicity), paclitaxel (intravenous drug administration), paclitaxel (pharmacology)

Emtree medical index terms

adjuvant therapy, adult, aged, anemia (side effect), article, **cancer adjuvant therapy**, cancer diagnosis, cancer recurrence (diagnosis), cancer surgery, cancer survival, clinical article, computer assisted tomography, death, deep vein thrombosis (side effect), disease free survival, drug efficacy, endoscopic echography, esophagogastroduodenoscopy, esophagus carcinoma (drug therapy), esophagus carcinoma (diagnosis), esophagus carcinoma (drug therapy), esophagus carcinoma (surgery), esophagus reconstruction, esophagus resection, faintness (side effect), fatigue (side effect), febrile neutropenia (side effect), female, heart atrium fibrillation (side effect), histopathology, human, human tissue, hypotension (side effect), immunohistochemistry, **lower esophagus sphincter**, lung embolism (side effect), male, multiple cycle treatment, myalgia (side effect), neutropenia (side effect), outcome assessment, overall survival, phase 2 clinical trial, positron emission tomography, postoperative care, **preoperative treatment**, priority journal, protein expression, side effect (side effect), thrombocytopenia (side effect), treatment duration, treatment response

Author Address

N. K. Altorki. Division of General Thoracic Surgery, New York Presbyterian Hospital, Weill Medical College of Cornell University, New York City, NY 10021.

Author Email

nkaltork@med.cornell.edu

Additional Information

Abbreviated Journal Title	J. Thorac. Oncol.
ISSN	15560864, 15561380 (electronic)
Source Type	Journal
Source Publication Date	June 2011
Entry Date	2011-06-20 (Full record), 2011-05-04 (Article in Press/In process)
Publication Type	Article
Page Range	1121-1127
Country of Author	United States
Country of Source	United States

Language of Article	English
Language of Summary	English
MEDLINE PMID	21532508
Embase Accession Number	2011311967
Number of References	52
CAS Registry Numbers	carboplatin (41575-94-4)
	celecoxib (169590-42-5)
	dexamethasone (50-02-2)
	diphenhydramine (147-24-0, 58-73-1)
	fluorodeoxyglucose f 18 (63503-12-8)
	paclitaxel (33069-62-4)

Related articles

View related articles on Embase.com

Copyright

MEDLINE® is the source for part of the citation data of this record

Records

37. **Association of the VEGF 936C>T polymorphism with FDG uptake, clinical, histopathological, and metabolic response in patients with adenocarcinomas of the esophagogastric junction**

Lorenzen S., Panzram B., Keller G., Lordick F., Herrmann K., Becker K., Langer R., Schwaiger M., Siewert J.R., Ott K.

Molecular Imaging and Biology 2011 **13:1 (178-186)**

Embase MEDLINE

Go to publisher for the full text

Abstract

Purpose: The MUNICON trial confirmed prospectively the usefulness of early response evaluation by 2-deoxy-2-[F-18]fluoro-D-glucose-positron emission tomography (FDG-PET) . Metabolic responders (R) showed initially a higher FDG uptake compared with nonresponders (p = 0.018). An association of the vascular endothelial growth factor (VEGF) 936C>T polymorphism and FDG uptake was

reported for breast cancer. Therefore, we investigated the VEGF 936C>T polymorphism for an association with response and survival. Procedures: The study was based on 110 patients included in the MUNCON trial (103 male, seven female; 75 AEG I, 35 AEG II, event-free survival (EFS) median 21.1 ± 4.6 months). Response was significantly associated with EFS. The VEGF 936C>T polymorphism was determined by PCR and restriction fragment length polymorphism analysis. For analysis, the T-variants were combined. Results: One hundred two patients were evaluable. Seventy-two patients showed the CC, 24 the CT, and six the TT genotype. Median EFS was 29.3 months for CC and 11.7 months for CT/TT ($p = 0.04$). No association of the genotypes (CC or CT/TT) with the SUV or response was found. Multivariate analysis revealed histopathological regression ($p = 0.003$) and genotype ($p = 0.04$) as independent prognostic factors. A combination of genotype and PET response (Gen-PET) defines three prognostic groups early in the course of treatment ($p = 0.002$). Cox regression analysis including clinical and histopathological response and Gen-PET reveals Gen-PET as independent prognostic factor ($p = 0.003$). Conclusion: The VEGF 936C>T polymorphism is a prognostic factor in patients undergoing neoadjuvant chemotherapy, although it is not associated with FDG uptake and response. The combination of metabolic response and VEGF 936C>T polymorphism defines three different prognostic groups. These findings need to be confirmed prospectively. This study has been registered in the European Clinical Trials Database as trial 2007-003356-11. © 2010 Academy of Molecular Imaging and Society for Molecular Imaging.

Other works by authors of this record

Lorenzen S., Panzram B., Keller G., Lordick F., Herrmann K., Becker K., Langer R., Schwaiger M., Siewert J.R., Ott K.

Emtree drug index terms

fluorodeoxyglucose f 18 (adverse drug reaction), fluorodeoxyglucose f 18 (drug analysis), vasculotropin (endogenous compound)

Emtree medical index terms

adenocarcinoma (diagnosis), adult, alopecia (side effect), article, blood toxicity (side effect), cardiotoxicity (side effect), controlled study, drug uptake, event free survival, fatigue (side effect), female, gastroesophageal adenocarcinoma (diagnosis), gastrointestinal toxicity (side effect), **genetic association**, **genetic polymorphism**, genotype, hand foot syndrome (side effect), heterozygosity, histopathology, homozygosity, human, image analysis, image processing, major clinical study, male,

neuropathy (side effect), outcome assessment, polymerase chain reaction, positron emission tomography, priority journal, restriction fragment length polymorphism

Author Address

K. Ott. Department of Surgery, University Hospital of Heidelberg, Heidelberg.

Author Email

Katja.Ott@med.uni-heidelberg.de

Additional Information

Abbreviated Journal Title	Mol. Imaging Biol.
ISSN	15361632, 18602002 (electronic)
CODEN	CPIMF
Source Type	Journal
Source Publication Date	February 2011
Entry Date	2011-02-17 (Full record), 2010-05-17 (Article in Press/In process)
Publication Type	Article
Page Range	178-186
Country of Author	Germany
Country of Source	United States
Language of Article	English
Language of Summary	English
MEDLINE PMID	20449668
Embase Accession Number	2011074054
Number of References	43
CAS Registry Numbers	fluorodeoxyglucose f 18 (63503-12-8)<br>vasculotropin (127464-60-2)

Related articles

View related articles on Embase.com

Copyright

MEDLINE® is the source for part of the citation data of this record

Records

38. **[Patterns of recurrence and survival after complete pathologic response to neoadjuvant treatment for gastric and gastroesophageal junction adenocarcinoma]**

Fields R.C., Strong V.E., Gonen M., Goodman K.A., Rizk N.P., Ilson D.H., Brennan M.F., Coit D.G., Shah M.A.

Annals of Surgical Oncology 2011 **18 SUPPL. 1 (S29)**

Embase

Go to publisher for the full text

Abstract

BACKGROUND: Pathologic complete response (pCR) is rare following neoadjuvant chemotherapy ± radiation therapy (RT) for locally advanced gastric or gastroesophageal junction (GEJ) adenocarcinoma. Timing and patterns of recurrence and survival after pCR are not well characterized. METHODS: A retrospective review of a prospective database identified patients with pCR after neoadjuvant chemotherapy ± RT for gastric or GEJ adenocarcinoma. Timing and location of recurrence, overall survival (OS), recurrence free survival (RFS), and disease specific survival (DSS) were analyzed. RESULTS: From 1985 - 2010, 2,676 patients underwent resection for gastric and GEJ (Siewert II/III) adenocarcinoma. 714 patients (27%) received neoadjuvant treatment: 442 (62%) chemotherapy alone, 272 (38%) neoadjuvant chemoRT. There were 60 patients (8%) with a pCR: 47 (78%) GEJ and 13 (22%) gastric tumors. 51 patients (85%) received neoadjuvant chemoRT. With 46 month median followup, 14 patients (23%; 12 GEJ, 2 gastric) developed a recurrence. Sites of first recurrence were: 8 distant (5 CNS, 2 visceral, 1 skin), 4 local, and 2 regional nodal (table). 13 of 14 recurrences (93%) occurred within 2 years of followup with a median time to recurrence of 13.5 months. 5 patients (42%) recurred in the CNS. 12 of 14 patients (86%) received treatment for their recurrence (8 chemotherapy, 2 surgery, 2 whole brain RT). Median time to death after recurrence was 11.5 months. 5-year OS, RFS, and DSS were 56%, 70%, and 65%, respectively. There were no differences in 5-year OS, RFS, or DSS based on tumor location (gastric v. GEJ: 5-year DSS 66% v. 56%; p=0.94) or receipt of RT (yes v. no: 5-year DSS 62% v. 88%; p=0.51). CONCLUSION: pCR after neoadjuvant therapy for gastric or GEJ adenocarcinoma is associated with improved long-term survival. Despite pCR, there remains significant risk of recurrence and cancer-specific death, especially within the first 2 years. These patients demonstrate an atypical failure pattern with a predominance of CNS recurrences. Whole brain imaging during the first 2 years of

follow-up should be considered. (Table presented).

Other works by authors of this record

Fields R.C., Strong V.E., Gonen M., Goodman K.A., Rizk N.P., Ilson D.H., Brennan M.F., Coit D.G., Shah M.A.

Emtree drug index terms

Emtree medical index terms

adenocarcinoma, adjuvant chemotherapy, adjuvant therapy, brain, central nervous system, chemotherapy, data base, death, follow up, imaging, **lower esophagus sphincter**, **neoplasm**, neoplasm, **oncology**, overall survival, patient, radiotherapy, risk, skin, **society**, stomach tumor, surgery, **survival**, survival time

Author Address

R.C. Fields. Gastric and Mixed Tumor Service, Department of Surgery, Memorial Sloan-Kettering Cancer Center.

Additional Information

Abbreviated Journal Title	Ann. Surg. Oncol.
ISSN	10689265
Source Type	Journal
Conference Name	64th Annual Cancer Symposium of the Society of Surgical Oncology
Conference Location	San Antonio, TX, United States
Conference Date	2011-03-02 to 2011-03-05
Source Publication Date	February 2011
Entry Date	2011-03-11 (Full record)
Publication Type	Conference Abstract
Page Range	S29
Country of Author	United States
Language of Article	English
Language of Summary	English

Related articles

View related articles on Embase.com

Copyright

Copyright 2011 Elsevier B.V., All rights reserved.

Records

39. **Outcomes after complete pathologic response (pCR) to neoadjuvant treatment for gastric and gastroesophageal junction (GEJ) adenocarcinoma.**

Fields R.C., Strong V.E., Gonen M., Brennan M.F., Coit D.G., Shah M.A.

Journal of Clinical Oncology 2011 **29:4 SUPPL. 1**

Embase

Abstract

Background: pCR is rare following neoadjuvant chemotherapy (NAChemo) ± radiation therapy (RT) for locally advanced gastric/GEJ adenocarcinoma. Recurrence and survival after pCR are not well characterized. Methods: A retrospective review of a prospective database identified patients with pCR after NAChemo ± RT for gastric/GEJ adenocarcinoma. Recurrence, overall survival (OS), recurrence free survival (RFS), and disease specific survival (DSS) were analyzed. Results: From 1985 - 2010, 2,676 patients underwent resection for gastric/GEJ adenocarcinoma, and 714 (27%) received NAChemo ± RT. There were 102 (14%) patients with a pCR. 60 patients (8%) had adequate pre-operative staging and follow-up and comprised the study group: 47 (78%) GEJ and 13 (22%) gastric tumors. 51 (85%) received neoadjuvant RT. With 46 month median follow-up, 14 patients (23%; 12 GEJ, 2 gastric) developed a recurrence (Table); 13 of 14 (93%) occurred within 2 years of follow-up with a median time to recurrence of 13.5 months. 5 (42%) recurred in the CNS. Median time to death after recurrence was 11.5 months. 5-year OS, RFS, and DSS were 56%, 70%, and 65%, respectively. Conclusions: pCR occurs in less than 15% of cases after neoadjuvant therapy for gastric and GEJ adenocarcinoma. Despite a pCR, there is a significant risk of recurrence and cancer-specific death during follow-up. As CNS recurrence is more prevalent in this group of patients, whole brain imaging should be considered. (Table presented).

Other works by authors of this record

Fields R.C., Strong V.E., Gonen M., Brennan M.F., Coit D.G., Shah M.A.

Emtree drug index terms

Emtree medical index terms

adenocarcinoma, adjuvant chemotherapy, adjuvant therapy, brain, central nervous system, data base, death, **digestive system cancer**, disease specific survival, follow up, human, imaging, **lower esophagus sphincter**, neoplasm, overall survival, patient, radiotherapy, recurrence free survival, risk, staging, stomach tumor, surgery, survival

Author Address

R.C. Fields. Memorial Sloan-Kettering Cancer Center.

Additional Information

Abbreviated Journal Title	J. Clin. Oncol.
ISSN	0732183X
Source Type	Journal
Conference Name	2011 Gastrointestinal Cancers Symposium
Conference Location	San Francisco, CA, United States
Conference Date	2011-01-20 to 2011-01-22
Source Publication Date	2011-02-01
Entry Date	2012-03-12 (Full record)
Publication Type	Conference Abstract
Page Range	
Country of Author	United States
Language of Article	English
Language of Summary	English

Related articles

View related articles on Embase.com

Copyright

Copyright 2012 Elsevier B.V., All rights reserved.

Records

40. **Single-institution experience of preoperative chemoradiotherapy for locally advanced gastric cancer.**

Pepek J.M., Chino J.P., Willett C.G., Tyler D.S., Uronis H.E., Czito B.G.

Journal of Clinical Oncology 2011 **29:4 SUPPL. 1**
Embase

Abstract

Background: To examine acute toxicity and outcomes for patients treated with preoperative chemoradiotherapy (CRT) for gastric cancer. Methods: Patients with gastroesophageal (GE) junction (Siewert type II and III) or stomach adenocarcinoma who underwent curative intent CRT followed by planned surgical resection at Duke University between 1987 and 2009 were reviewed. Tumors were staged according to AJCC 6th edition. Local recurrence was defined as radiographic or biopsy- proven disease within the radiation treatment field. Overall survival (OS), local control (LC) and disease-free survival (DFS) were estimated using the Kaplan-Meier method. Toxicity was graded according to CTCAE v4.0. Results: Forty-eight patients (60% stage III, 8% stage IV) were included. Most (73%) had proximal (GE junction, cardia and fundus) tumors. Thirty-five percent had signet ring histology, 52% had poorly differentiated tumors and 10% had linitis plastica. Median age was 60 years and median RT dose was 45 Gy. All patients received concurrent chemotherapy (CT) with 40 (83%) receiving 5-FU-based CT. Rates of acute > grade 2 hematologic and non-hematologic toxicity were 38% and 10%, respectively. Six patients (13%) required treatment break and two (4%) were unable to complete the prescribed treatment course. Thirty-six patients (75%) underwent surgery. Patients did not undergo surgery due to distant metastases at laparotomy or restaging (n=9), patient refusal (n=2) or poor performance status (n=1). Pathologic complete response and R0 resection rates were 19% and 86%, respectively. Thirty-day surgical mortality was 6%. At 42 months median follow-up, 3-year actuarial OS for all patients was 40%. For those undergoing surgery, 3-year OS, LC and DFS were 50%, 73% and 41%, respectively. Conclusions: Preoperative CRT for gastric cancer is reasonably well tolerated with acceptable rates of perioperative morbidity and mortality. In this patient cohort with advanced disease, LC, DFS and OS rates in resected patients are comparable to similarly staged, adjuvantly treated historic controls. Further study comparing neoadjuvant CRT to standard treatment approaches for gastric cancer is indicated.

Other works by authors of this record

Pepek J.M., Chino J.P., Willett C.G., Tyler D.S., Uronis H.E., Czito B.G.

Emtree drug index terms

fluorouracil

Emtree medical index terms

acute toxicity, biopsy, cardia, **chemoradiotherapy**, chemotherapy, **digestive system cancer**, disease free survival, distant metastasis, follow up, histology, human, Kaplan Meier method, laparotomy, linitis plastica, lower esophagus sphincter, morbidity, mortality, neoplasm, overall survival, patient, radiotherapy, stomach adenocarcinoma, **stomach cancer**, surgery, surgical mortality, toxicity, university

Author Address

J.M. Pepek. Duke University Medical Center.

Additional Information

Abbreviated Journal Title	J. Clin. Oncol.
ISSN	0732183X
Source Type	Journal
Conference Name	2011 Gastrointestinal Cancers Symposium
Conference Location	San Francisco, CA, United States
Conference Date	2011-01-20 to 2011-01-22
Source Publication Date	2011-02-01
Entry Date	2012-03-12 (Full record)
Publication Type	Conference Abstract
Page Range	
Country of Author	United States
Language of Article	English
Language of Summary	English

Related articles

View related articles on Embase.com

Copyright

Copyright 2012 Elsevier B.V., All rights reserved.

Records

41. **Neoadjuvant chemoradiotherapy for locally advanced gastric adenocarcinoma: A single institution experience**

Pepek J.M., Chino J.P., Willett C.G., Tyler D.S., Uronis H.E., Czito B.G.

International Journal of Radiation Oncology Biology Physics 2010 **78:3 SUPPL. 1 (S73)**
Embase

Go to publisher for the full text

Abstract

Purpose/Objective(s): Randomized trials have demonstrated a survival benefit with either postoperative chemoradiotherapy (CRT) or perioperative chemotherapy (CT) for resectable gastric adenocarcinoma, albeit with high rates of treatment-related toxicity. Neoadjuvant CRT has potential advantages compared to adjuvant approaches and improves outcomes in esophageal and rectalmalignancies. Weexamined acute toxicity and disease-related outcomes for patients treated with neoadjuvantCRTfor gastric cancer at our institution. Materials/Methods: All patients with gastroesophageal (GE) junction (Siewert type II and III) or stomach adenocarcinoma who underwent curative intent CRT followed by planned surgical resection at Duke University between 1987 and 2009 were reviewed. Tumors were clinically staged according to AJCC 6th edition. Local recurrence was defined as radiographic or biopsy-proven disease within the radiation treatment field. Actuarial rates of overall survival (OS), local control (LC) and disease-free survival (DFS) were estimated using the Kaplan-Meier method. Toxicity was graded according to CTCAE v4.0. Results: Forty-eight patients (2% stage IB, 19% stage II, 60% stage III, 8% stage IV, 10% incompletely staged) were included. Most (73%) had proximal (GE junction, cardia and fundus) tumors. Thirty-eight percent had signet ring histology, 52% had poorly differentiated tumors, and 10% had linitis plastica. Median age was 60 years (range, 28-79) and median RT dose was 45 Gy. All patients received concurrent CT with forty (83%) receiving fluoropyrimidine-based CT. Six patients (13%) required treatment break and two (4%) were unable to complete the prescribed treatment course. Rates of acute > grade 2 hematologic and non-hematologic toxicity were 38% and 10%, respectively. Thirty-six patients (75%) underwent surgery. Patients did not receive surgery due to distant metastases noted at laparotomy or restaging (n = 9), patient refusal (n = 2) or poor performance status (n = 1). Pathologic complete response and R0 resection rates were 19% and 86%, respectively. Thirty-day surgical mortality was 6%. At a median follow-up of 42 months, 3-year actuarial OS for the entire cohort was 40%. For patients undergoing surgery, 3-year OS, LC, and DFS were 50%, 73% and 41%, respectively. Conclusions: Neoadjuvant CRT for gastric adenocarcinoma is reasonably well tolerated with acceptable rates of perioperative morbidity and mortality. In this group of patients with advanced disease,

LC, DFS and OS rates in resected patients are comparable to similarly staged, adjuvantly treated historic controls. Further study comparing neoadjuvant CRT to standard treatment approaches for gastric cancer is indicated.

Other works by authors of this record

Pepek J.M., Chino J.P., Willett C.G., Tyler D.S., Uronis H.E., Czito B.G.

Emtree drug index terms

adjuvant, fluoropyrimidine

Emtree medical index terms

acute toxicity, biopsy, cardia, chemotherapy, disease free survival, distant metastasis, follow up, histology, Kaplan Meier method, laparotomy, linitis plastica, lower esophagus sphincter, morbidity, mortality, neoplasm, **oncology**, overall survival, patient, radiotherapy, **society**, **stomach adenocarcinoma**, stomach cancer, surgery, surgical mortality, survival, toxicity, university

Author Address

J.M. Pepek. Duke University Medical Center.

Additional Information

Abbreviated Journal Title	Int. J. Radiat. Oncol. Biol. Phys.
ISSN	03603016
Source Type	Journal
Conference Name	52nd Annual Meeting of the American Society for Radiation Oncology
Conference Location	San Diego, CA, United States
Conference Date	2010-10-31 to 2010-11-04
Source Publication Date	2010-11-01
Entry Date	2010-10-19 (Full record)
Publication Type	Conference Abstract
Page Range	S73
Country of Author	United States
Language of Article	English
Language of Summary	English
Publisher Item Identifier	S0360301610011776

Related articles

View related articles on Embase.com

Copyright

Copyright 2010 Elsevier B.V., All rights reserved.

Records

42. **Multimodality therapy for the curative treatment of cancer of the esophagus and gastroesophageal junction**

Ku G.Y., Ilson D.H.

Expert Review of Anticancer Therapy 2008 **8:12 (1953-1964)**
Embase MEDLINE

Go to publisher for the full text

Abstract

This review examines the role of combined-modality therapy in the treatment of locally advanced esophageal cancer. While surgery remains a cornerstone of treatment, recent studies have demonstrated that pre- or perioperative chemotherapy is associated with improved survival. Primary chemoradiotherapy is the accepted standard of care for medically inoperable patients. Neoadjuvant chemoradiotherapy continues to be investigated and is associated with several advantages over neoadjuvant chemotherapy alone, including an improvement in the pathologic complete response rate and resectability; patients who achieve a pathologic complete response also appear to have improved survival. Adjuvant chemoradiotherapy may be considered for patients who undergo primity resection of lower esophageal/gastroesophageal junction adenocarcinoma. Future directions include the investigation of novel chemotherapy regimens, the addition of targeted therapies and the use of PET to provide an early assessment of response. © 2008 Expert Reviews Ltd.

Other works by authors of this record

Ku G.Y., Ilson D.H.

Emtree drug index terms

5,10 methylenetetrahydrofolate reductase (FADH2) (endogenous compound), antineoplastic agent (clinical trial), antineoplastic agent (drug therapy), bevacizumab

(clinical trial), bevacizumab (drug combination), bevacizumab (drug therapy), bleomycin (clinical trial), bleomycin (drug combination), bleomycin (drug therapy), caldesmon (endogenous compound), capecitabine (clinical trial), capecitabine (drug combination), capecitabine (drug therapy), carboplatin (clinical trial), cisplatin (adverse drug reaction), cisplatin (clinical trial), cisplatin (drug combination), cisplatin (drug therapy), docetaxel (clinical trial), docetaxel (drug combination), epirubicin (clinical trial), epirubicin (drug combination), epirubicin (drug therapy), etoposide (clinical trial), etoposide (drug combination), etoposide (drug therapy), excision repair cross complementing protein 1 (endogenous compound), fluorodeoxyglucose f 18, fluorouracil (adverse drug reaction), fluorouracil (clinical trial), fluorouracil (drug combination), fluorouracil (drug therapy), folinic acid (clinical trial), folinic acid (drug combination), folinic acid (drug therapy), irinotecan (clinical trial), irinotecan (drug combination), multidrug resistance protein 1 (endogenous compound), paclitaxel (adverse drug reaction), paclitaxel (clinical trial), paclitaxel (drug combination), paclitaxel (drug therapy), thymidylate synthase (endogenous compound), vinblastine (clinical trial), vinblastine (drug combination), vinblastine (drug therapy)

Emtree medical index terms

adjuvant chemotherapy, adjuvant therapy, blood toxicity (side effect), bone marrow suppression (side effect), cancer surgery, cancer survival, clinical trial, continuous infusion, disease free survival, drug efficacy, drug targeting, drug tolerability, esophageal adenocarcinoma (drug therapy), esophageal adenocarcinoma (radiotherapy), esophageal adenocarcinoma (surgery), esophagitis (complication), esophagitis (side effect), esophagus cancer (drug therapy), esophagus cancer (drug therapy), esophagus cancer (surgery), esophagus resection, gastrointestinal toxicity (complication), gastrointestinal toxicity (side effect), human, intermethod comparison, **lower esophagus sphincter**, lower esophagus sphincter cancer (drug therapy), lower esophagus sphincter cancer (drug therapy), lower esophagus sphincter cancer (surgery), mucosa inflammation (complication), mucosa inflammation (side effect), **multimodality cancer therapy**, multiple cycle treatment, nausea (complication), nausea (side effect), outcome assessment, overall survival, positron emission tomography, postoperative period, preoperative period, radiation dose, review, squamous cell carcinoma (drug therapy), squamous cell carcinoma (radiotherapy), squamous cell carcinoma (surgery), stomach cancer (drug therapy)

Author Address

D.H. Ilson. Memorial Sloan-Kettering Cancer Center, New York, NY 10065.

Author Email

ilsond@mskcc.org

Additional Information

Abbreviated Journal Title	Expert Rev. Anticancer Ther.
ISSN	14737140, 17448328 (electronic)
CODEN	ERATB
Source Type	Journal
Source Publication Date	2008-01-01
Entry Date	2009-04-24 (Full record)
Publication Type	Review
Page Range	1953-1964
Country of Author	United States
Country of Source	United Kingdom
Language of Article	English
Language of Summary	English
MEDLINE PMID	19046115
Embase Accession Number	2009161257
Number of References	92

CAS Registry Numbers	5,10 methylenetetrahydrofolate reductase (FADH2) (9028-69-7)
	bevacizumab (216974-75-3)
	bleomycin (11056-06-7)
	capecitabine (154361-50-9)
	carboplatin (41575-94-4)
	cisplatin (15663-27-1, 26035-31-4, 96081-74-2)
	docetaxel (114977-28-5)
	epirubicin (56390-09-1, 56420-45-2)
	etoposide (33419-42-0)
	fluorodeoxyglucose f 18 (63503-12-8)
	fluorouracil (51-21-8)
	folinic acid (58-05-9)
	irinotecan (100286-90-6)
	paclitaxel (33069-62-4)
	thymidylate synthase (9031-61-2)
	vinblastine (865-21-4)

Related articles

View related articles on Embase.com

Copyright

MEDLINE® is the source for part of the citation data of this record

Records

43. **Does graded histologic response after neoadjuvant chemotherapy predict survival for completely resected gastric cancer?**

Mansour J.C., Tang L., Shah M., Bentrem D., Klimstra D.S., Gonen M., Kelsen D.P., Brennan M.F., Coit D.G.

Annals of Surgical Oncology 2007 **14:12 (3412-3418)**

Embase MEDLINE

Go to publisher for the full text

Abstract

Background: After publication of the MAGIC trial results, preoperative chemotherapy is increasingly used to treat advanced gastric cancer before resection. Tools for measuring response must be assessed. Methods: We identified all patients with gastric cancer treated with neoadjuvant chemotherapy and R0 resection between 1991 and 2005 from a prospective database. Patients receiving preoperative radiation were excluded. Histologic response to treatment was graded from 0% to 100% by a single pathologist. Kaplan-Meier survival analysis was performed to identify the relationship between response and outcome and to identify factors predictive of disease-specific survival (DSS). Multivariate analysis was performed to identify independent predictors. Results: A total of 168 patients underwent R0 resection after receiving neoadjuvant chemotherapy. Thirty-three percent of tumors were at the gastroesophageal junction. Cisplatin-based therapy was used for 68% of patients. Twenty-two percent of patients had a >50% pathologic response to treatment. Median follow-up after resection for all patients was 25 months. Median DSS for all patients was 33 months. Three-year DSS improved from 44% to 69% with at least a 50% histologic response (P = .01). Factors associated with decreased DSS included positive nodes at resection, pT3 tumor or greater, high grade, perineural or vascular invasion, and <50% response. Multivariate analysis identified nodal status and perineural or vascular invasion as independent predictors of survival. Conclusions:

Posttreatment nodal status and perineural or vascular invasion at resection, but not graded histologic response, independently predict DSS after neoadjuvant chemotherapy and surgical resection of gastric cancer. © 2007 Society of Surgical Oncology.

Other works by authors of this record

Mansour J.C., Tang L., Shah M., Bentrem D., Klimstra D.S., Gonen M., Kelsen D.P., Brennan M.F., Coit D.G.

Emtree drug index terms

antiinfective agent, cisplatin (drug combination), cisplatin (drug therapy), doxorubicin (drug therapy), fluorouracil (drug combination), fluorouracil (drug therapy), irinotecan (drug combination), irinotecan (drug therapy), methotrexate (drug therapy), paclitaxel (drug combination), paclitaxel (drug therapy)

Emtree medical index terms

adjuvant chemotherapy, adjuvant therapy, article, cancer grading, **cancer surgery**, **cancer survival**, combination chemotherapy, controlled study, data base, disease free survival, female, follow up, human, Kaplan Meier method, lower esophagus sphincter, lymph node, major clinical study, male, multivariate analysis, pathology, predictor variable, prospective study, stomach cancer (drug therapy), stomach cancer (surgery), treatment outcome, treatment response, tumor invasion

Author Address

D.G. Coit. Memorial Sloan-Kettering Cancer Center, New York, NY 10021.

Author Email

coitd@mskcc.org

Additional Information

Abbreviated Journal Title	Ann. Surg. Oncol.
ISSN	10689265, 15344681 (electronic)
CODEN	ASONF
Source Type	Journal
Source Publication Date	December 2007
Entry Date	2007-12-04 (Full record)
Publication Type	Article
Page Range	3412-3418

Country of Author	United States
Country of Source	United States
Language of Article	English
Language of Summary	English
MEDLINE PMID	17909917
Embase Accession Number	2007572255
Number of References	11
CAS Registry Numbers	cisplatin (15663-27-1, 26035-31-4, 96081-74-2)<br>doxorubicin (23214-92-8, 25316-40-9)<br>fluorouracil (51-21-8)<br>irinotecan (100286-90-6)<br>methotrexate (15475-56-6, 59-05-2, 7413-34-5)<br>paclitaxel (33069-62-4)

Related articles

View related articles on Embase.com

Copyright

MEDLINE® is the source for part of the citation data of this record

Records

44. **Paclitaxel-Based Chemoradiotherapy in the Treatment of Patients With Operable Esophageal Cancer**

Kelsey C.R., Chino J.P., Willett C.G., Clough R.W., Hurwitz H.I., Morse M.A., Bendell J.C., D'Amico T.A., Czito B.G.

International Journal of Radiation Oncology Biology Physics 2007 **69:3 (770-776)**

Embase MEDLINE

Go to publisher for the full text

Abstract

Purpose: To compare a neoadjuvant regimen of cisplatin/5-fluorouracil (5-FU) and concurrent radiation therapy (RT) with paclitaxel-based regimens and RT in the management of operable esophageal (EC)/gastroesophageal junction (GEJ) cancer.

Methods and Materials: All patients receiving neoadjuvant chemotherapy (CT) and RT for EC/GEJ cancer at Duke University between January 1995 and December 2004 were included. Clinical end points were compared for patients receiving paclitaxel-based regimens (TAX) vs. alternative regimens (non-TAX). Local control (LC), disease-free survival (DFS), and overall survival (OS) were estimated using the Kaplan-Meier method. Chi-square analysis was performed to test the effect of TAX on pathologic complete response (pCR) rates and toxicity. Results: A total of 109 patients received CT-RT followed by esophagectomy (95 M; 14 F). Median RT dose was 45 Gy (range, 36-66 Gy). The TAX and non-TAX groups comprised 47% and 53% of patients, respectively. Most (83%) TAX patients received three drug regimens including platinum and a fluoropyrimidine. In the non-TAX group, 89% of the patients received cisplatin and 5-FU. The remainder received 5-FU or capecitabine alone. Grade 3-4 toxicity occurred in 41% of patients receiving TAX vs. 24% of those receiving non-TAX (p = 0.19). Overall pCR rate was 39% (39% with TAX vs. 40% with non-TAX, p = 0.9). Overall LC, DFS, and OS at 3 years were 80%, 34%, and 37%, respectively. At 3 years, there were no differences in LC (75% vs. 85%, p = 0.33) or OS (37% vs. 37%, p = 0.32) between TAX and non-TAX groups. Conclusions: In this large experience, paclitaxel-containing regimens did not improve pCR rates or clinical end points compared to non-paclitaxel-containing regimens. © 2007 Elsevier Inc. All rights reserved.

Other works by authors of this record

Kelsey C.R., Chino J.P., Willett C.G., Clough R.W., Hurwitz H.I., Morse M.A., Bendell J.C., D'Amico T.A., Czito B.G.

Emtree drug index terms

capecitabine (drug combination), capecitabine (drug therapy), carboplatin (drug combination), carboplatin (drug therapy), fluoropyrimidine (drug therapy), fluorouracil (drug combination), fluorouracil (drug therapy), folinic acid (drug combination), folinic acid (drug therapy), paclitaxel (adverse drug reaction), paclitaxel (drug combination), paclitaxel (drug therapy), platinum (drug therapy), UFT (drug combination), UFT (drug therapy)

Emtree medical index terms

adjuvant therapy, adult, aged, article, cancer chemotherapy, cancer radiotherapy, chi square test, combination chemotherapy, controlled study, dehydration (side effect), diarrhea (side effect), disease free survival, endoscopy, esophagitis (side effect), esophagus cancer (diagnosis), esophagus cancer (drug therapy), esophagus cancer

(radiotherapy), esophagus cancer (surgery), esophagus perforation (side effect), esophagus resection, female, human, Kaplan Meier method, lower esophagus sphincter, major clinical study, male, nausea and vomiting (side effect), neutropenia (side effect), overall survival, positron emission tomography, priority journal, thrombosis (side effect), treatment response, ultrasound

Author Address

C.R. Kelsey. Department of Radiation Oncology, Duke University Medical Center, Durham, NC.

Author Email

kelse003@mc.duke.edu

Additional Information

Abbreviated Journal Title	Int. J. Radiat. Oncol. Biol. Phys.
ISSN	03603016
CODEN	IOBPD
Source Type	Journal
Source Publication Date	2007-11-01
Entry Date	2007-11-01 (Full record)
Publication Type	Article
Page Range	770-776
Country of Author	United States
Country of Source	United States
Language of Article	English
Language of Summary	English
Publisher Item Identifier	S0360301607005573
MEDLINE PMID	17889266
Embase Accession Number	2007454334
Number of References	25
CAS Registry Numbers	UFT (74578-38-4)
	capecitabine (154361-50-9)
	carboplatin (41575-94-4)
	fluoropyrimidine (675-21-8)
	fluorouracil (51-21-8)
	folinic acid (58-05-9)
	paclitaxel (33069-62-4)
	platinum (7440-06-4)

Related articles

View related articles on Embase.com

Copyright

MEDLINE® is the source for part of the citation data of this record

Records

45. **Cancer of the gastroesophageal junction: Current therapy options**

Ilson D.H.

Current Treatment Options in Oncology 2006 **7:5 (410-423)**

Embase MEDLINE

Go to publisher for the full text

Abstract

Active chemotherapy agents in metastatic adenocarcinoma of the esophagus include taxanes (docetaxel or paclitaxel), 5-fluorouracil, irinotecan, platinum drugs (including cisplatin, oxaliplatin, and carboplatin), and anthracyclines. Conventional chemotherapy combines infusional 5-fluorouracil with cisplatin. The addition of a third drug to this backbone results in greater toxicity and only marginal improvements in outcome. Alternative and potentially better-tolerated chemotherapy involves two-drug regimens, combining 5-fluorouracil with a taxane or irinotecan, or combining a platinum drug with irinotecan or a taxane. Although preoperative chemotherapy improves survival compared with surgery alone, the addition of radiation therapy to chemotherapy preoperatively improves rates of curative resection, reduces local tumor recurrence, and achieves a significant rate of pathologic complete response. Combined preoperative chemotherapy and concurrent radiotherapy is the preferred preoperative strategy for locally advanced adenocarcinoma of the esophagus. Survival is improved with postoperative chemotherapy and radiotherapy if none has been delivered preoperatively. Copyright © 2006 by Current Science Inc.

Other works by authors of this record

Ilson D.H.

Emtree drug index terms

acetylsalicylic acid (clinical trial), acetylsalicylic acid (drug combination), acetylsalicylic acid (drug therapy), anthracycline (drug therapy), antineoplastic agent (adverse drug reaction), antineoplastic agent (clinical trial), antineoplastic agent (drug combination), antineoplastic agent (drug comparison), antineoplastic agent (drug therapy), antineoplastic agent (pharmacology), capecitabine (adverse drug reaction), capecitabine (clinical trial), capecitabine (drug combination), capecitabine (drug comparison), capecitabine (drug therapy), carboplatin (adverse drug reaction), carboplatin (clinical trial), carboplatin (drug combination), carboplatin (drug therapy), cetuximab (clinical trial), cetuximab (drug therapy), cetuximab (pharmacology), cisplatin (adverse drug reaction), cisplatin (clinical trial), cisplatin (drug combination), cisplatin (drug comparison), cisplatin (drug therapy), cyclooxygenase 2 inhibitor (clinical trial), cyclooxygenase 2 inhibitor (drug combination), cyclooxygenase 2 inhibitor (drug therapy), cyclooxygenase 2 inhibitor (oral drug administration), cyclooxygenase 2 inhibitor (pharmacology), docetaxel (adverse drug reaction), docetaxel (clinical trial), docetaxel (drug combination), docetaxel (drug comparison), docetaxel (drug therapy), doxorubicin (clinical trial), doxorubicin (drug combination), doxorubicin (drug comparison), doxorubicin (drug therapy), epidermal growth factor receptor antibody (clinical trial), epidermal growth factor receptor antibody (drug combination), epidermal growth factor receptor antibody (drug therapy), epidermal growth factor receptor antibody (pharmacology), epidermal growth factor receptor kinase inhibitor (clinical trial), epidermal growth factor receptor kinase inhibitor (drug therapy), epidermal growth factor receptor kinase inhibitor (pharmacology), epirubicin (clinical trial), epirubicin (drug combination), epirubicin (drug comparison), epirubicin (drug therapy), esomeprazole (clinical trial), esomeprazole (drug combination), esomeprazole (drug therapy), etoposide (clinical trial), etoposide (drug combination), etoposide (drug therapy), fluorouracil (adverse drug reaction), fluorouracil (clinical trial), fluorouracil (drug combination), fluorouracil (drug comparison), fluorouracil (drug therapy), folinic acid (clinical trial), folinic acid (drug combination), folinic acid (drug therapy), irinotecan (adverse drug reaction), irinotecan (clinical trial), irinotecan (drug combination), irinotecan (drug comparison), irinotecan (drug therapy), matuzumab, matuzumab (clinical trial), matuzumab (drug therapy), matuzumab (pharmacology), methotrexate (clinical trial), methotrexate (drug combination), methotrexate (drug comparison), methotrexate (drug therapy), mitomycin (clinical trial), mitomycin (drug combination), mitomycin (drug therapy), monoclonal antibody (clinical trial), monoclonal antibody (drug combination), monoclonal antibody (drug therapy), monoclonal antibody (pharmacology), oxaliplatin (adverse drug reaction), oxaliplatin

(clinical trial), oxaliplatin (drug combination), oxaliplatin (drug comparison), oxaliplatin (drug therapy), paclitaxel (adverse drug reaction), paclitaxel (clinical trial), paclitaxel (drug combination), paclitaxel (drug therapy), panitumumab (clinical trial), panitumumab (drug therapy), panitumumab (pharmacology), platinum derivative (adverse drug reaction), platinum derivative (clinical trial), platinum derivative (drug combination), platinum derivative (drug therapy), taxane derivative (clinical trial), taxane derivative (drug combination), taxane derivative (drug therapy), unindexed drug, vasculotropin antibody (clinical trial), vasculotropin antibody (drug combination), vasculotropin antibody (drug therapy), vasculotropin antibody (pharmacology), vinblastine (clinical trial), vinblastine (drug combination), vinblastine (drug therapy)

Emtree medical index terms

advanced cancer (drug therapy), advanced cancer (radiotherapy), advanced cancer (surgery), bone marrow suppression (side effect), cancer combination chemotherapy, cancer recurrence (drug therapy), cancer recurrence (prevention), cancer recurrence (radiotherapy), cancer recurrence (surgery), cancer survival, clinical trial, disease free survival, drug efficacy, drug safety, drug targeting, drug tolerability, esophageal adenocarcinoma (drug therapy), esophageal adenocarcinoma (prevention), esophageal adenocarcinoma (radiotherapy), esophageal adenocarcinoma (surgery), esophagitis (side effect), febrile neutropenia (side effect), gastrointestinal symptom (side effect), hematologic disease (side effect), human, **lower esophagus sphincter**, metastasis (drug therapy), metastasis (radiotherapy), metastasis (surgery), multimodality cancer therapy, nausea (side effect), neutropenia (side effect), overall survival, postoperative care, preoperative care, review, stomatitis (side effect), treatment response

Author Address

D.H. Ilson. Gastrointestinal Oncology Service, Department of Medicine, Weill-Cornell University Medical College, New York, NY 10021.

Author Email

ilsand@mskcc.org

Additional Information

Abbreviated Journal Title	Curr. Treat. Options Oncol.
ISSN	15272729
CODEN	CTOOB
Source Type	Journal

Source Publication Date	September 2006
Entry Date	2006-10-16 (Full record)
Publication Type	Review
Page Range	410-423
Country of Author	United States
Country of Source	United Kingdom
Language of Article	English
Language of Summary	English
MEDLINE PMID	16904058
Embase Accession Number	2006432852
Number of References	79
Drug Tradenames	aspirin, emd 7200
CAS Registry Numbers	acetylsalicylic acid (493-53-8, 50-78-2, 53663-74-4, 53664-49-6, 63781-77-1)<br>capecitabine (154361-50-9)<br>carboplatin (41575-94-4)<br>cetuximab (205923-56-4)<br>cisplatin (15663-27-1, 26035-31-4, 96081-74-2)<br>docetaxel (114977-28-5)<br>doxorubicin (23214-92-8, 25316-40-9)<br>epirubicin (56390-09-1, 56420-45-2)<br>esomeprazole (119141-88-7, 202742-32-3, 217087-09-7, 217087-10-0)<br>etoposide (33419-42-0)<br>fluorouracil (51-21-8)<br>folinic acid (58-05-9)<br>irinotecan (100286-90-6)<br>matuzumab (339186-68-4)<br>methotrexate (15475-56-6, 59-05-2, 7413-34-5)<br>mitomycin (1404-00-8)<br>oxaliplatin (61825-94-3)<br>paclitaxel (33069-62-4)<br>panitumumab (339177-26-3)<br>vinblastine (865-21-4)

Related articles

View related articles on Embase.com

Copyright

MEDLINE® is the source for part of the citation data of this record

Records

46. **Pathologic nodal status predicts disease-free survival after neoadjuvant chemoradiation for gastroesophageal junction carcinoma**

Gaca J.G., Petersen R.P., Peterson B.L., Harpole Jr. D.H., D'Amico T.A., Pappas T.N., Seigler H.F., Wolfe W.G., Tyler D.S.

Annals of Surgical Oncology 2006 **13:3 (340-346)**

Embase MEDLINE

Go to publisher for the full text

Abstract

Background: The incidence of carcinoma of the gastroesophageal junction (GEJ) is rapidly increasing, and the prognosis remains poor. We examined outcomes in patients who received neoadjuvant chemoradiation for GEJ tumors to identify factors that predict disease-free (DFS) and overall (OS) survival. Methods: A retrospective analysis was performed of 101 consecutive patients who received chemoradiation and surgery for GEJ carcinoma between 1992 and 2001. Results: The median DFS and OS of all patients were 16 and 25 months, respectively. Twenty-eight patients with a complete histological response (T0N0) experienced greater DFS compared with all others (P = .02). Node-negative patients, regardless of T stage, experienced improved median DFS (24 months) compared with N1 patients (9 months; P = .01). Preoperative stage, age, tumor location, or Barrett's esophagus did not independently predict OS by univariate analysis. Multivariate analysis demonstrated that only posttreatment nodal status (P = .03) - not the degree of primary tumor response - predicted DFS. Conclusions: The nodal status of patients with GEJ tumors after neoadjuvant therapy is predictive of DFS after resection. The poor outcome in node-positive patients supports postneoadjuvant therapy nodal staging, because surgical aggressiveness should be tempered by the realization that cure is unlikely and median survival is short. © 2006 The Society of Surgical Oncology, Inc.

Other works by authors of this record

Gaca J.G., Petersen R.P., Peterson B.L., Harpole Jr. D.H., D'Amico T.A., Pappas T.N., Seigler H.F., Wolfe W.G., Tyler D.S.

Emtree drug index terms

antineoplastic agent (drug combination), antineoplastic agent (drug dose), antineoplastic agent (drug therapy), carboplatin (drug combination), carboplatin (drug dose), carboplatin (drug therapy), cisplatin (drug combination), cisplatin (drug dose), cisplatin (drug therapy), doxorubicin (drug combination), doxorubicin (drug dose), doxorubicin (drug therapy), fluorouracil (drug combination), fluorouracil (drug dose), fluorouracil (drug therapy), methotrexate (drug combination), methotrexate (drug dose), methotrexate (drug therapy), paclitaxel (drug combination), paclitaxel (drug dose), paclitaxel (drug therapy)

Emtree medical index terms

adult, age, aged, article, Barrett esophagus, **cancer adjuvant therapy**, cancer chemotherapy, cancer diagnosis, cancer localization, cancer radiotherapy, cancer staging, cancer surgery, cancer survival, controlled study, **disease free survival**, esophagus carcinoma (diagnosis), esophagus carcinoma (drug therapy), esophagus carcinoma (radiotherapy), esophagus carcinoma (surgery), female, histology, human, **lower esophagus sphincter**, major clinical study, male, multivariate analysis, pathology, prediction, treatment outcome

Author Address

D.S. Tyler. Departments of Surgery, Duke University Medical Center, Box 3118, Durham, NC 27710.

Author Email

tyler002@acpub.duke.edu

Additional Information

Abbreviated Journal Title	Ann. Surg. Oncol.
ISSN	10689265, 15344681 (electronic)
CODEN	ASONF
Source Type	Journal
Source Publication Date	March 2006
Entry Date	2006-02-27 (Full record)
Publication Type	Article
Page Range	340-346

Country of Author	United States
Country of Source	United States
Language of Article	English
Language of Summary	English
MEDLINE PMID	16485154
Embase Accession Number	2006086405
Number of References	28
CAS Registry Numbers	carboplatin (41575-94-4)
	cisplatin (15663-27-1, 26035-31-4, 96081-74-2)
	doxorubicin (23214-92-8, 25316-40-9)
	fluorouracil (51-21-8)
	methotrexate (15475-56-6, 59-05-2, 7413-34-5)
	paclitaxel (33069-62-4)

Related articles

View related articles on Embase.com

Copyright

MEDLINE® is the source for part of the citation data of this record

Records

47. **Induction chemotherapy improved outcomes of patients with resectable esophageal cancer who received chemoradiotherapy followed by surgery**

Jin J., Liao Z., Zhang Z., Ajani J., Swisher S., Chang J.Y., Jeter M., Guerrero T., Stevens C.W., Vaporciyan A., Putnam Jr. J., Walsh G., Smythe R., Roth J., Yao J., Allen P., Cox J.D., Komaki R.

International Journal of Radiation Oncology Biology Physics 2004 **60:2 (427-436)**

Embase MEDLINE

Go to publisher for the full text

Abstract

Purpose To investigate the effect of induction chemotherapy (CHT) before trimodality therapy on the outcome of patients with resectable cancer of the esophagus. Methods

and materials This retrospective study included 81 consecutive patients with resectable cancer of the esophagus who received neoadjuvant chemoradiotherapy followed by esophagectomy between January 1990 and December 1998 (inclusive). Thirty-nine patients underwent chemoradiotherapy followed by esophagectomy (CHT/RT+S), 42 received additional induction CHT followed by CHT/RT+S (CHT+CHT/RT+S). Of the 81 patients, 47 were entered in institutional or national prospective trials (6 in the CHT/RT+S and 41 in the CHT+CHT/RT+S group). Induction CHT consisted of three courses of 5-fluorouracil (5-FU), cisplatin, and paclitaxel given in 28-day cycles in 37 patients (88.1%). Concurrent CHT was 5-FU and platinum based. The median radiation dose for patients treated with CHT/RT+S was 30 Gy (range, 30-50.4 Gy) delivered in a median of 10 fractions (range, 10-28 fractions) and 45 Gy (range, 30-45 Gy) in a median of 25 fractions (range, 10-25 fractions) for patients treated with CHT+CHT/RT+S. Esophagectomy was performed 6-8 weeks after completion of concurrent chemoradiotherapy. Most patients underwent transthoracic esophagectomy (n = 66, 82.5%). Results The pretreatment characteristics were well balanced between the two groups except for age. The median follow-up time was 29 months (22 months for the CHT/RT+S group and 38.5 months for the CHT+CHT/RT+S group) for all patients and 49 months for living patients. The actuarial overall survival (OS), disease-free survival (DFS), locoregional control (LRC), and distant metastasis-free survival (DMFS) rate at 5 years for the entire group was 46%, 36.6%, 70.7%, and 53.2%, respectively. Statistically significant differences in the OS, DFS, and LRC rates between the two groups were detected. Specifically, the 5-year OS rate was 22.8% and 71.1% in the CHT/RT+S and CHT+CHT/RT+S group (p = 0.0001), respectively. The 5-year DFS rate was 27.6% and 56.6% in the CHT/RT+S and CHT+CHT/RT+S group (p = 0.003), respectively. The 5-year LRC rate was 64.2% and 85.6% in the CHT/RT+S and CHT+CHT/RT+S group (p = 0.007), respectively. The difference in the DMFS rate between the two groups was statistically significant, with a 2- and 5-year actuarial rate of 63.9% and 51.9%, respectively, in the CHT/RT+S group and 76.9% and 74.1%, respectively, in the CHT+CHT/RT+S group (p = 0.04). The statistically significant differences persisted when patients who received ≥45 Gy in each group were compared. Among those patients, the 5-year OS, DFS, LRC, and DMFS rates were 23.1%, 15.4%, 58.6%, and 39.2%, respectively, for those receiving CHT/RT+S, and 71.4% (p = 0.001), 55.8% (p = 0.0008), 84.6% (p = 0.005), and 77.3% (p = 0.009), respectively, for those receiving CHT+CHT/RT+S. The pathologic complete response (pCR) rate was greater in the CHT+CHT/RT+S group compared with in the CHT/RT+S group (p = 0.008). In univariate analysis, young age, good Karnofsky performance status, Stage II disease, total radiation dose, multiple drug regimen for concurrent CHT, pCR,

R0 resection, distant disease progression, and CHT+CHT/RT+S treatment proved to be prognostic factors for OS. Lower esophageal/gastroesophageal junction tumor location, pCR, R0 resection, and CHT+CHT/RT+S treatment were favorable prognostic factors for LRC. Neither the total radiation dose nor multiple drugs for concurrent CHT were negative prognostic factors for LRC. In multivariate analysis, pCR, R0 resection, and treatment with CHT+CHT/RT+S were independent positive predictive factors for OS, and distant recurrences were negative predictive factors for OS. R0 resection, CHT+CHT/RT+S treatment, and lower esophageal/gastroesophageal junction tumor location were positive predictive factors for LRC. The radiation dose was not identified as an independent prognostic factor for either OS or LRC in the multivariate analysis. Meaningful multivariate analysis could not be performed when the multiple drug variable was included in the model because of the small number of patients. Conclusion Significantly greater LRC, DFS, OS, and DMFS were found in patients treated with CHT+CHT/RT+S compared with those treated with CHT/RT+S. The pCR rate was significantly higher in the CHT+CHT/RT+S group. Induction CHT was an independent favorable prognostic factor for both LRC and OS for the population included in this study. Our data suggest that a randomized trial comparing CHT+CHT/RT+S and CHT/RT+S is warranted to assess further the merits of this treatment in patients with this currently very lethal cancer. © 2004 Elsevier Inc.

Other works by authors of this record

Jin J., Liao Z., Zhang Z., Ajani J., Swisher S., Chang J.Y., Jeter M., Guerrero T., Stevens C.W., Vaporciyan A., Putnam Jr. J., Walsh G., Smythe R., Roth J., Yao J., Allen P., Cox J.D., Komaki R.

Emtree drug index terms

cisplatin (drug combination), cisplatin (drug dose), cisplatin (drug therapy), fluorouracil (drug combination), fluorouracil (drug dose), fluorouracil (drug therapy), irinotecan (drug combination), irinotecan (drug dose), irinotecan (drug therapy), paclitaxel (drug combination), paclitaxel (drug dose), paclitaxel (drug therapy)

Emtree medical index terms

adult, aged, article, cancer chemotherapy, cancer localization, cancer radiotherapy, cancer staging, cancer surgery, cancer survival, clinical article, controlled study, drug dose regimen, esophagus cancer (drug therapy), esophagus cancer (radiotherapy), esophagus cancer (surgery), esophagus resection, female, human, male, priority journal, prognosis, radiation dose, statistical significance, **treatment outcome**

Author Address

Z. Liao. Department of Radiation Oncology, Univ. Texas M. D. Anderson Cancer C., Houston, TX.

Author Email

zliao@mail.mdanderson.org

Additional Information

Abbreviated Journal Title	Int. J. Radiat. Oncol. Biol. Phys.
ISSN	03603016
CODEN	IOBPD
Source Type	Journal
Source Publication Date	2004-10-01
Entry Date	2004-09-27 (Full record)
Publication Type	Article
Page Range	427-436
Country of Author	United States
Country of Source	United States
Language of Article	English
Language of Summary	English
Publisher Item Identifier	S0360301604005140
MEDLINE PMID	15380576
Embase Accession Number	2004398152
Number of References	30
CAS Registry Numbers	cisplatin (15663-27-1, 26035-31-4, 96081-74-2)<br>fluorouracil (51-21-8)<br>irinotecan (100286-90-6)<br>paclitaxel (33069-62-4)

Related articles

View related articles on Embase.com

Copyright

MEDLINE® is the source for part of the citation data of this record

Records

48. **Preoperative induction of CPT-11 and cisplatin chemotherapy followed by chemoradiotherapy in patients with locoregional carcinoma of the esophagus or gastroesophageal junction**

Ajani J.A., Walsh G., Komaki R., Morris J., Swisher S.G., Putnam Jr. J.B., Lynch P.M., Wu T.-T., Smythe R., Vaporciyan A., Faust J., Cohen D.S., Nivers R., Roth J.A.

Cancer 2004 **100:11 (2347-2354)**

Embase MEDLINE

Go to publisher for the full text

Abstract

BACKGROUND. Patients with localized esophageal carcinoma often develop locoregional and distant disease recurrence. The current study investigated the outcome of a new chemotherapy combination as induction therapy before chemoradiotherapy. METHODS. Forty-three patients with resectable carcinoma of the esophagus or gastroesophageal junction were enrolled. Most of the tumors were endoscopic ultrasonography [EUS] $_{EUS}$T3 (84%) and $_{EUS}$N1 (63%). The patients received ≤ 2 6-week cycles of CPT-11 and cisplatin followed by chemoradiotherapy (45 grays with 5-fluorouracil and paclitaxel). Five to six weeks after chemoradiotherapy, the patients underwent staging and surgery. The feasibility, curative resection rates, overall and disease-free survival rates, rate of significant pathologic response, and patterns of disease recurrence were assessed. RESULTS. Of the 43 patients, 39 (91%) underwent an R0 resection. Two patients (5%) died after surgery. A pathologic complete response (pathCR) was observed in 11 (28%) of the 39 patients (or 26% of the 43 patients). In addition, 16 patients (41% of 39 patients or 37% of 43 patients) had < 10% viable tumor in the surgical specimen (pathPR). A comparison of endoscopic ultrasonograpy T and N classifications with surgical T and N classifications demonstrated significant down-staging (P < 0.01). The median survival period of all 43 patients was 22.1 months. Patients who had achieved a pathCR or pathPR had a longer median survival (25.6 months) than those who achieved less than a pathPR (18.5 months; P = 0.52). None of the clinical parameters examined were found to correlate with survival or pathologic response. CONCLUSIONS. CPT-11-based induction chemotherapy resulted in substantial pathCR and pathPR rates, both of which lead to a favorable survival outcome. The three-step strategy needs to be developed further, with the investigation of targeted therapies with chemotherapy and radiotherapy. © 2004 American Cancer Society.

Other works by authors of this record

Ajani J.A., Walsh G., Komaki R., Morris J., Swisher S.G., Putnam Jr. J.B., Lynch P.M., Wu T.-T., Smythe R., Vaporciyan A., Faust J., Cohen D.S., Nivers R., Roth J.A.

Emtree drug index terms

cisplatin (adverse drug reaction), cisplatin (drug combination), cisplatin (drug therapy), cisplatin (intravenous drug administration), fluorouracil (adverse drug reaction), fluorouracil (drug combination), fluorouracil (drug therapy), irinotecan (adverse drug reaction), irinotecan (drug combination), irinotecan (drug therapy), paclitaxel (adverse drug reaction), paclitaxel (drug combination), paclitaxel (drug therapy), paclitaxel (intravenous drug administration)

Emtree medical index terms

adult, aged, article, **cancer combination chemotherapy**, cancer mortality, **cancer radiotherapy**, cancer recurrence, cancer staging, cancer surgery, cancer survival, clinical article, diarrhea (side effect), endoscopic echography, esophagitis (side effect), esophagus carcinoma (drug therapy), esophagus carcinoma (radiotherapy), esophagus carcinoma (surgery), fatigue (side effect), female, fever (side effect), granulocytopenia (side effect), human, **lower esophagus sphincter**, male, myalgia (side effect), nausea (side effect), neuropathy (side effect), **preoperative care**, priority journal, treatment outcome, vomiting (side effect)

Author Address

J.A. Ajani. Department of GI Medical Oncology, Univ. TX M. D. Anderson Cancer Ctr., Unit 426, Houston, TX 77030-4009.

Author Email

jajani@mdanderson.org

Additional Information

Abbreviated Journal Title	Cancer
ISSN	0008543X
CODEN	CANCA
Source Type	Journal
Source Publication Date	2004-06-01
Entry Date	2004-06-16 (Full record)
Publication Type	Article

Page Range	2347-2354
Country of Author	United States
Country of Source	United States
Language of Article	English
Language of Summary	English
MEDLINE PMID	15160337
Embase Accession Number	2004223630
Number of References	18
Drug Tradenames	cpt 11
CAS Registry Numbers	cisplatin (15663-27-1, 26035-31-4, 96081-74-2)<br>fluorouracil (51-21-8)<br>irinotecan (100286-90-6)<br>paclitaxel (33069-62-4)

Related articles

View related articles on Embase.com

Copyright

MEDLINE® is the source for part of the citation data of this record

Records

49. **Complete 5-year follow-up of a prospective phase II trial of preoperative chemoradiotherapy for esophageal cancer**

Posner M.C., Gooding W.E., Lew J.I., Rosenstein M.M., Lembersky B.C.

Surgery 2001 **130:4 (620-628)**

Embase MEDLINE

Go to publisher for the full text

Abstract

Background. Conclusive evidence supporting the routine use of multimodality therapy in esophageal cancer is lacking. However, since long-term survival after esophagectomy alone is unusual, clinical trials designed to identify effective therapeutic regimens are essential. We report here the 5-year results of a phase Ii induction chemoradiotherapy trial. Methods. From August 1991 to January 1995, 44

patients with esophageal or gastroesophageal junction carcinoma were treated with a combination of 5-fluorouracil, cisplatin, and interferon-α with concurrent external beam radiotherapy. Results. Forty-one (93%) patients completed chemoradiotherapy, with most toxic events recorded as grade I or Ii. Curative resection (all gross tumor removed) was achieved in 36 of 37 surgical explorations, with 10 tumors demonstrating complete pathologic response and 23 showing partial pathologic response. Median follow-up for survivors was 75 months (range, 60-100 months). Five-year survival for all patients was 32%, with a median survival of 28 months. Five-year disease-free survival in patients with curative resection was 36% (median, 26 months) and overall survival was 39% (median, 34 months). Five-year survival for patients with curative resection whose disease responded to chemoradiotherapy was 42% (median overall survival, 36 months). Local-regional recurrence alone occurred in 3 patients, distant failure alone in 12 patients, and combined local-regional and distant failure in 2 patients. A Cox proportionatal hazards model identified both pathologic tumor and nodal stage as independent predictors of disease-free survival. Fourteen patients (32%) were 5-year survivors; 1 of these patients later experienced disease recurrence and died. Conclusions. Preoperative chemoradiotherapy can result in a long-term and durable disease-free state. Only large, multi-institutional phase III trials can determine whether combined modality therapy is superior to resection alone.

Other works by authors of this record

Posner M.C., Gooding W.E., Lew J.I., Rosenstein M.M., Lembersky B.C.

Emtree drug index terms

alpha interferon (drug combination), alpha interferon (drug therapy), cisplatin (drug combination), cisplatin (drug therapy), fluorouracil (drug combination), fluorouracil (drug therapy)

Emtree medical index terms

adult, aged, article, cancer chemotherapy, cancer grading, cancer radiotherapy, cancer recurrence, cancer surgery, cancer survival, clinical article, esophagus cancer (drug therapy), esophagus cancer (radiotherapy), esophagus cancer (surgery), female, follow up, human, lower esophagus sphincter, male, multimodality cancer therapy, preoperative treatment, priority journal, prospective study, statistical analysis

Author Address

M.C. Posner. University of Chicago, Section of General Surgery, MC 5031, Chicago,

IL 60637.

Additional Information

Abbreviated Journal Title	Surgery
ISSN	00396060
CODEN	SURGA
Source Type	Journal
Source Publication Date	2001-01-01
Entry Date	2001-10-26 (Full record)
Publication Type	Article
Page Range	620-628
Country of Author	United States
Country of Source	United States
Language of Article	English
Language of Summary	English
MEDLINE PMID	11602892
Embase Accession Number	2001366088
Number of References	20
CAS Registry Numbers	cisplatin (15663-27-1, 26035-31-4, 96081-74-2)
	fluorouracil (51-21-8)

Related articles

View related articles on Embase.com

Copyright

MEDLINE® is the source for part of the citation data of this record

Records

50. **Intensive multimodality therapy for carcinoma of the esophagus and gastroesophageal junction.**

Ferguson M.K., Reeder L.B., Hoffman P.C., Haraf D.J., Drinkard L.C., Vokes E.E.

Annals of surgical oncology : the official journal of the Society of Surgical Oncology 1995 **2:2 (101-106)**

MEDLINE

Abstract

BACKGROUND: We designed a trial of intensive multimodality therapy for carcinoma of the esophagus and gastroesophageal junction to assess tumor response and operability after neoadjuvant chemotherapy and to determine the impact of trimodality therapy on longterm survival. METHODS: Thirty-two patients with resectable (clinical stage IIa, n = 17; IIb, n = 1; III, n = 14) squamous cell cancer (n = 15) or adenocarcinoma (n = 17) were treated with neoadjuvant chemotherapy (cisplatin, 5-fluorouracil, leukovorin), resection, and postoperative chemoradiotherapy (hydroxyurea, 5-fluorouracil; 50-66 Gy). RESULTS: Use of neoadjuvant chemotherapy yielded the following results: a measurable clinical response in 22 patients, stable disease in eight patients, disease progression in one patient, and death in one patient. Thirty-one patients underwent resection, with the following results: two operative deaths (6.5%) and nonfatal morbidity in 17 (59%); the median hospital stay was 13 days. Pathologic staging was stage 0, n = 1; I, n = 2; IIa, n = 11; IIb, n = 5; III, n = 7; and IV, n = 5. Postoperative chemoradiotherapy was completed in 23 patients with one death, for an overall treatment-related mortality rate of 12.5% (four of 32). At a mean follow-up of 22.5 months, median survival is 19.7 months and 14 patients are alive and disease free. CONCLUSIONS: Neoadjuvant therapy for cancer of the esophagus and cardia results in good tumor response. Esophagectomy in this setting can be accomplished with acceptable morbidity and mortality. Results of an interim analysis of survival are encouraging and suggest that further investigation of this regimen is warranted.

Other works by authors of this record

Ferguson M.K., Reeder L.B., Hoffman P.C., Haraf D.J., Drinkard L.C., Vokes E.E.

Emtree drug index terms

antineoplastic agent (drug therapy), cisplatin (drug administration), fluorouracil (drug administration), folinic acid (drug administration), hydroxyurea (drug administration)

Emtree medical index terms

adenocarcinoma (surgery), adjuvant chemotherapy, adjuvant therapy, adult, aged, article, cancer staging, esophagus tumor (surgery), female, follow up, human, lower esophagus sphincter (surgery), male, middle aged, multimodality cancer therapy, pathology, postoperative care, remission, squamous cell carcinoma (surgery), survival rate

Author Address

M.K. Ferguson. Department of Surgery, University of Chicago, Illinois, USA.

Additional Information

Abbreviated Journal Title	Ann. Surg. Oncol.
ISSN	10689265
Source Type	Journal
Source Publication Date	Mar 1995
Entry Date	1995-05-31 (Full record)
Publication Type	Article
Page Range	101-106
Country of Source	United States
Language of Article	English
MEDLINE PMID	7728562
CAS Registry Numbers	cisplatin (15663-27-1, 26035-31-4, 96081-74-2)<br>fluorouracil (51-21-8)<br>folinic acid (58-05-9)<br>hydroxyurea (127-07-1)

Related articles

View related articles on Embase.com

Copyright

MEDLINE® is the source for the citation and abstract of this record

Records

51. **Adenocarcinoma of the esophagus and gastroesophageal junction: Clinical and pathologic assessment of response to induction chemotherapy**

Adelstein D.J., Rice T.W., Boyce G.A., Sivak M.V., Van Kirk M.A., Kirby T.J., Van Stolk R.U., Bukowski R.M.

American Journal of Clinical Oncology: Cancer Clinical Trials 1994 **17:1 (14-18)**

Embase MEDLINE

Abstract

A preoperative induction chemotherapy regimen consisting of two monthly courses of etoposide, doxorubicin, and cisplatin was given to 13 patients with nonmetastatic adenocarcinoma of the distal esophagus or gastroesophageal junction. Esophageal ultrasound examination was performed both before chemotherapy and again before surgery. Induction chemotherapy was poorly tolerated with 10 of the 13 patients experiencing at least one episode of severe neutropenia. Two of the 13 patients refused the second course of treatment. A symptomatic response to chemotherapy, defined as a reduction in the presenting symptom, was noted in 10 of the 13 patients (77%). Endoscopic improvement occurred in 9 of the 13 patients (69%). Esophageal ultrasound evidence of a reduction in either T or N stage was noted in only 2 of the 13 patients (15%), however, and neither of these responses was confirmed pathologically. Clinical evidence of disease progression was noted in 4 patients during chemotherapy. With a median follow-up of 31 months, the relapse-free and overall survivals are 25% and 31%, respectively. Despite significant toxicity, our chemotherapy regimen would be considered successful if assessed by symptomatic or esophagoscopic improvement. Esophageal ultrasound, careful pathologic staging, and our disappointing survival rates, however, suggest limited, if any, value for this approach.

Other works by authors of this record

Adelstein D.J., Rice T.W., Boyce G.A., Sivak M.V., Van Kirk M.A., Kirby T.J., Van Stolk R.U., Bukowski R.M.

Emtree drug index terms

cisplatin (adverse drug reaction), cisplatin (drug combination), cisplatin (drug therapy), doxorubicin (adverse drug reaction), doxorubicin (drug combination), doxorubicin (drug therapy), etoposide (adverse drug reaction), etoposide (drug combination), etoposide (drug therapy)

Emtree medical index terms

adenocarcinoma (drug therapy), adenocarcinoma (radiotherapy), adenocarcinoma (surgery), adult, aged, article, cancer radiotherapy, cancer recurrence, cancer staging, cancer surgery, cancer survival, clinical article, clinical trial, controlled clinical trial, controlled study, esophagoscopy, esophagus carcinoma (drug therapy), esophagus carcinoma (radiotherapy), esophagus carcinoma (surgery), gastrointestinal symptom (side effect), human, kidney disease (side effect), **lower esophagus sphincter**, male, neutropenia (side effect)

Author Address

D.J. Adelstein. Cleveland Clinic Foundation, Cleveland, OH 44195.

Additional Information

Abbreviated Journal Title	AM. J. CLIN. ONCOL. CANCER CLIN. TRIALS
ISSN	02773732
CODEN	AJCOD
Source Type	Journal
Source Publication Date	1994-01-01
Entry Date	1994-03-10 (Full record)
Publication Type	Article
Page Range	14-18
Country of Author	United States
Country of Source	United States
Language of Article	English
Language of Summary	English
MEDLINE PMID	8311001
Embase Accession Number	1994070465
CAS Registry Numbers	cisplatin (15663-27-1, 26035-31-4, 96081-74-2)<br>doxorubicin (23214-92-8, 25316-40-9)<br>etoposide (33419-42-0)

Related articles

View related articles on Embase.com

Copyright

MEDLINE® is the source for part of the citation data of this record

For further information on email alerts please visit the Embase Info site.

Embase provides access to more than 28 million validated biomedical and pharmacological records from Embase and MEDLINE.

This email has been sent to you via Embase, a product of Elsevier B.V., Registered Office: Radarweg 29, 1043 NX Amsterdam, The Netherlands, Tel. +31 (0) 20 485 3911, Registration Number: 33156677, VAT Number NL 005033019B01.

You are receiving this email as a subscriber to Embase alerts. To unsubscribe, please follow the link at the top of this page.

Embase respects your privacy and does not disclose, rent or sell your personal information to any non-affiliated third parties without your consent, except as may be stated in the Embase Privacy Policy.

By using email or alert services, you agree to comply with the Embase Terms and Conditions.

Copyright © 2015 Elsevier B.V.. All rights reserved. Embase is a registered trademark of Elsevier B.V.
