# Supplementary material for: Correlation of pathological complete response with survival after neoadjuvant chemotherapy in gastric or gastroesophageal junction cancer treated with radical surgery: A meta-analysis
Source: PLoS One. 2018 Jan 25;13(1):e0189294. doi: 10.1371/journal.pone.0189294 (PMC5784899; doi:10.1371/journal.pone.0189294)
Supplement: S6 File — (PDF) [file pone.0189294.s007.pdf]

注释: Web of Science™

==

FN Thomson Reuters Web of Science™
VR 1.0

PT J
AU Cho, Haruhiko
   Nakamura, Junichi
   Asaumi, Yoshihide
   Yabusaki, Hiroshi
   Sakon, Masahiro
   Takasu, Naoki
   Kobayashi, Tatsunori
   Aoki, Taro
   Shiraishi, Osamu
   Kishimoto, Hirofumi
   Nunobe, Souya
   Yanagisawa, Shinji
   Suda, Takeshi
   Ueshima, Shigeyuki
   Matono, Satoru
   Maruyama, Hiroshi
   Tatsumi, Mitsutoshi
   Seya, Tomoko
   Tanizawa, Yutaka
   Yoshikawa, Takaki
TI Long-term Survival Outcomes of Advanced Gastric Cancer Patients Who
   Achieved a Pathological Complete Response with Neoadjuvant Chemotherapy:
   A Systematic Review of the Literature.
SO Annals of surgical oncology
VL 22
IS 3
BP 787
EP 92
DI 10.1245/s10434-014-4084-9
PD 2015-Mar
PY 2015

AB BACKGROUND: A pathologic complete response (pCR) can sometimes be induced by intensive or long-term neoadjuvant chemotherapy (NAC). This prognostic research study based on a systematic review of the literature evaluated the impact of a pCR on the long-term survival of gastric cancer (GC) patients.

METHODS: Articles were extracted from PubMed and the Japanese medical search engine "Ichu-shi," using the terms "GC," "NAC," and "pCR." Articles were selected based on the following criteria: (1) full-text case report, (2) R0 resection following NAC for locally advanced GC, and (3) pathological complete response in both the primary stomach and in the lymph nodes. A questionnaire regarding the patients' prognoses was sent to the corresponding authors of the articles selected in July 2013.

RESULTS: Twenty-four articles met the criteria. Twenty authors responded to the questionnaire. Finally, 22 patients from 20 articles were entered into the present study. The median follow-up time (range) of the survivors was 76 (range 13-161) months. Tumors that were stage III/IV (86%: 19/22) and of an undifferentiated histology (61.9%: 13/21) were dominant. An S1-based regimen was frequently selected for the NAC. All patients underwent R0 resection and D2/D3 lymphadenectomy. The overall survival and recurrence-free survival rates at 3 and 5years were 96% and 85% and 91% and 75%, respectively.

CONCLUSIONS: Although a pCR was a relatively rare event, a high pCR rate would be helpful to select the regimen and courses of NAC, especially when the pathological response rates are similar.

TC 0

ZB 0

Z8 0

ZS 0

Z9 0

UT MEDLINE:25223927

PM 25223927

ER

PT J

AU Takishita, Chie
Yajima, Kazuhito
Iwasaki, Yoshiaki
Ohashi, Manabu
Iwanaga, Tomohiro

Oohinata, Ryouki
TI [A case of early gastric cancer with multiple synchronous bone metastases treated complete response with S-1+CDDP].
SO Gan to kagaku ryoho. Cancer & chemotherapy
VL 41
IS 13
BP 2611
EP 4
PD 2014-Dec
PY 2014
AB We report a case of complete response (CR) following induction chemotherapy using S-1 for a patient with early gastric cancer accompanied by multiple synchronous bone metastases. An asymptomatic 70-year-old woman was diagnosed with early gastric cancer by upper gastrointestinal endoscopy during a periodic medical examination. An abdomino-pelvic computed tomography (CT) scan revealed no primary tumor in the stomach and the absence of lymph node or liver metastases. However, osteoplastic changes were detected in the lumbar vertebrae and the ilium. Multiple synchronous bone metastases from early gastric cancer were detected on magnetic resonance imaging, bone scintigraphy, and positron emission tomography- CT. After a regimen consisting of 15 courses of S-1 plus cisplatin (CDDP), and an additional 5 courses of S-1 were administered, clinical CR was confirmed for the bone metastases. Laparoscopic distal gastrectomy with D1 lymphadenectomy was performed for treating the primary gastric cancer 33 months after the initiation of chemotherapy. Pathological CR was also achieved for the primary gastric cancer. Imaging analysis did not show disease progression 48 months after the initiation of chemotherapy. Synchronous bone metastases from early gastric cancer are extremely rare, and a good outcome was achieved in the present case through induction chemotherapy.
TC 0
ZB 0
Z8 0
ZS 0
Z9 0
SN 0385-0684
UT MEDLINE:25596058
PM 25596058
ER

PT J
AU Ishigami, Sumiya
Uenosono, Yoshikazu
Arigami, Takaaki
Yanagita, Shigehiro
Okumura, Hiroshi
Uchikado, Yasuto
Kita, Yoshiaki
Kurahara, Hiroshi
Kijima, Yuko
Nakajo, Akihiro
Maemura, Kosei
Natsugoe, Shoji
TI Clinical utility of perioperative staging laparoscopy for advanced gastric cancer
SO WORLD JOURNAL OF SURGICAL ONCOLOGY
VL 12
AR 350
DI 10.1186/1477-7819-12-350
PD NOV 18 2014
PY 2014
AB Background: Perioperative staging laparoscopy is a useful tool for the detection of occult peritoneal metastases in gastrointestinal cancers. This retrospective study aimed to determine the clinical value of staging laparoscopy for advanced or recurrent gastric cancer.
Methods: A total of 178 patients with advanced or recurred gastric cancer who underwent perioperative staging laparoscopy were enrolled. In the absence of peritoneal deposits (P1) and positive peritoneal cytology (CY1), gastrectomy with lymph node dissection was indicated with curative intent. If P1 or CY1 was detected intraoperatively, patients received intensive chemotherapy and laparoscopic surgical intervention.
Results: Curative gastrectomy was performed in 104 patients after confirmation of P0 and CY0 status. P1 or CY1 was detected for the first time in 23 (15%) patients. A total of 13 patients were converted from gastrectomy to intensive chemotherapy after detection of P1 or CY1. Additional laparoscopic interventions included insertion of intraperitoneal reservoir port in 54 patients, insertion of a metallic stent in five, ileostomy for colon stricture in six, jejunostomy in 19,

and gastrojejunostomy in 16. Of eight patients treated with intensive chemotherapy who underwent R0 gastrectomy after second-look laparoscopy, five are currently free from recurrence of gastric cancer for 25.5 months.
Conclusions: Occult peritoneal dissemination was detected in about 14% in patients with tumors deeper than T2. Moreover, additional laparoscopic interventions can be utilized for P1 or CY1 patients. The excellent surgical outcomes of R0 gastrectomy after chemotherapy and second-look laparoscopy indicate that confirmation of P0 and CY0 status by staging laparoscopy is of value to determine treatment strategy in patients with advanced gastric cancer.

TC 0

ZB 0

Z8 0

ZS 0

Z9 0

SN 1477-7819

UT WOS:000345937700001

PM 25407392

ER

PT J

AU Li, Jian
Shen, Lin

TI [Application of molecular targeted agents in comprehensive treatment of gastrointestinal cancer].

SO Zhonghua wei chang wai ke za zhi = Chinese journal of gastrointestinal surgery

VL 17

IS 11

BP 1062

EP 7

PD 2014-Nov

PY 2014

AB Targeted agents increase response rates and improved overall survival in treatment of metastatic gastrointestinal cancer. Therefore, physicians pay more attention to the role of targeted agents in treatment of local advanced gastrointestinal cancer. The clinical trials are ongoing to evaluate the efficacy of Trastuzumab in neoadjuvant treatment of local

advanced gastric cancer with HER-2 gene over expression. Many studies reported Cetuximab plus chemotherapy as a conversion treatment improve R0 resection rates and prolonged overall survival of the patients with potentially resectable colorectal cancer liver metastasis with wild type KRAS gene status. A phase III(clinical trial is assessing the conversion efficacy of Bevacizumab in unresectable disease with KRAS gene mutation. Current evidence showed that neoadjuvant therapy of targeted agents did not prolong survival of patients with resectable liver metastasis. However, this is controversial. In neoadjuvant therapy of local advanced rectal cancer, Cetuximab did not improve the rates of pathological complete response in most of the phase II(trials. Furthermore, there are no phase III(trials to assess the role of Bevacizumab. Compared to chemotherapy alone for metastatic cancer, it is more important to evaluate the interaction and synergistic action of targeted agents, cytotoxic drugs, surgery and radiation, to make a scientific multidisciplinary model in comprehensive treatment of local advanced cancer.
TC 0
ZB 0
Z8 0
ZS 0
Z9 0
SN 1671-0274
UT MEDLINE:25421761
PM 25421761
ER

PT J
AU Karamitopoulou, Eva
Thies, Svenja
Zlobec, Inti
Ott, Katja
Feith, Marcus
Slotta-Huspenina, Julia
Lordick, Florian
Becker, Karen
Langer, Rupert
TI Assessment of Tumor Regression of Esophageal Adenocarcinomas After Neoadjuvant Chemotherapy Comparison of 2 Commonly Used Scoring

Approaches
SO AMERICAN JOURNAL OF SURGICAL PATHOLOGY
VL 38
IS 11
BP 1551
EP 1556
PD NOV 2014
PY 2014
AB Histopathologic determination of tumor regression provides important prognostic information for locally advanced gastroesophageal carcinomas after neoadjuvant treatment. Regression grading systems mostly refer to the amount of therapyinduced fibrosis in relation to residual tumor or the estimated percentage of residual tumor in relation to the former tumor site. Although these methods are generally accepted, currently there is no common standard for reporting tumor regression in gastroesophageal cancers. We compared the application of these 2 major principles for assessment of tumor regression: hematoxylin and eosin-stained slides from 89 resection specimens of esophageal adenocarcinomas following neoadjuvant chemotherapy were independently reviewed by 3 pathologists from different institutions. Tumor regression was determined by the 5-tiered Mandard system (fibrosis/tumor relation) and the 4-tiered Becker system (residual tumor in %). Interobserver agreement for the Becker system showed better weighted kappa values compared with the Mandard system (0.78 vs. 0.62). Evaluation of the whole embedded tumor site showed improved results (Becker: 0.83; Mandard: 0.73) as compared with only 1 representative slide (Becker: 0.68; Mandard: 0.71). Modification into simplified 3-tiered systems showed comparable interobserver agreement but better prognostic stratification for both systems (log rank Becker: P = 0.015; Mandard P = 0.03), with independent prognostic impact for overall survival (modified Becker: P = 0.011, hazard ratio = 3.07; modified Mandard: P = 0.023, hazard ratio = 2.72). In conclusion, both systems provide substantial to excellent interobserver agreement for estimation of tumor regression after neoadjuvant chemotherapy in esophageal adenocarcinomas. A simple 3-tiered system with the estimation of residual tumor in % (complete regression/1% to 50% residual tumor/ > 50% residual tumor) maintains the highest reproducibility and prognostic value.
TC 0
ZB 0

Z8 0

ZS 0

Z9 0

SN 0147-5185

UT WOS:000343880200013

PM 25140894

ER

PT J

AU Eryilmaz, Melek Karakurt
   Mutlu, Hasan
   Salim, Derya Kivrak
   Musri, Fatma Yalcin
   Tural, Deniz
   Coskun, Hasan Senol

TI The Neutrophil to Lymphocyte Ratio has a High Negative Predictive Value for Pathologic Complete Response in Locally Advanced Breast Cancer Patients Receiving Neoadjuvant Chemotherapy

SO ASIAN PACIFIC JOURNAL OF CANCER PREVENTION

VL 15

IS 18

BP 7737

EP 7740

DI 10.7314/APJCP.2014.15.18.7737

PD OCT 11 2014

PY 2014

AB Background: The neutrophil-to-lymphocyte ratio (NLR) is a strong predictor of mortality in patients with pancreatic, colorectal, lung, gastric cancer and renal cell carcinoma. The aim of this study was to determine the relationship between pathological complete response (pCR) and pretreatment NLR values in locally advanced breast cancer (BC) patients receiving neoadjuvant chemotherapy (NACT). Materials and Methods: Datawere collected retrospectively from the Akdeniz University School of Medicine Database for locally advanced BC patients treated with NACT between January 2000-December 2013. Results: A total of 78 patients were analyzed. Sixteen (20%) patients achieved pCR. Estrogen receptor (ER) positivity was lower in pCR+ than pCR-cases (p=0.011). The median NLR values were similar in both arms. The optimum NLR cut-off point for BC patients with PCR+ was 2.33 (AUC:0.544, 95% CI

[0.401-0.688], p=0.586) with sensitivity, specificity, positive predictive value and negative predictive value (NPV) of 50%, 51,6%, 21,1%, and 80%, respectively. Conclusions: This study showed no relationship between the pCR and pretreatment NLR values. Because of a considerable high NPV, in the patients with higher NLR who had luminal type BC in which pCR is lower after NACT, such treatment may not be recommended.
TC 0
ZB 0
Z8 0
ZS 0
Z9 0
SN 1513-7368
UT WOS:000343833600038
PM 25292055
ER

PT J
AU Xiong, B.
Ma, L.
Cheng, Y.
Zhang, C.
TI Clinical effectiveness of neoadjuvant chemotherapy in advanced gastric cancer: An updated meta-analysis of randomized controlled trials
SO EJSO
VL 40
IS 10
BP 1321
EP 1330
DI 10.1016/j.ejso.2014.01.006
PD OCT 2014
PY 2014
AB Aims: To assess the efficacy and safety of neoadjuvant chemotherapy (NAC) for advanced gastric cancer (AGC).
Methods: By searching electronic databases (PubMed, Embase, Cochrane Library) and ASCO proceedings from 1990 to 2012, all randomized controlled trials (RCTs) which compared the effect of NAC-combined surgery versus surgery alone in AGC were included. All calculations and statistical tests were performed using RevMan 5.0 software.

Results: 12 RCTs with a total of 1820 patients were included. All patients had locally advanced but resectable gastric cancer and received NAC. NAC can slightly improve the survival rate (OR = 1.32, 95% Confidence interval (CI): 1.07-1.64, P = 0.01), with little or no significant benefits in subgroup analyses between either different population or regimens. NAC can significantly improve the 3-year progression-free survival (PFS) (OR: 1.85, 95% CI: 1.39-2.46, p < 0.0001), tumor down-staging rate (OR: 1.71, 95% CI: 1.26, 2.33, p = 0.0006) and R0 resection rate (OR: 1.38, 95% CI: 1.08-1.78, P -= 0.01) of patients with AGC. There was no difference between the two arms, in terms of relapse rates (OR: 1.03, 95% CI: 0.60-1.78, p = 0.92), operative complications (OR: 1.20, 95% CI: 0.90-1.58, p = 0.21), perioperative mortality (OR: 1.14, 95% CI: 0.64-2.05, p = 0.65) and grade 3/4 adverse effects: gastrointestinal problem (OR: 0.57, 95% CI: 0.25-1.30, p = 0.18), leukopenia (OR: 0.88, 95% CI: 0.41-1.91, p = 0.75), thrombocytopenia (OR: 1.27, 95% CI: 0.27-5.93, p = 0.76). Conclusion: NAC is effective and safe. However, further prospective multi-national and multi-center RCTs are still needed in order to investigate the long-term oncological and functional outcomes to define the clinical benefits of NAC and the most effective strategies for AGC.
(C) 2014 Elsevier Ltd. All rights reserved.
TC 0
ZB 0
Z8 0
ZS 0
Z9 0
SN 0748-7983
UT WOS:000343380600022
PM 25239442
ER

PT J
AU Hashemzadeh, Shahriyar
Pourzand, Ali
Somi, Mohammad Hossein
Zarrintan, Sina
Javad-Rashid, Reza
Esfahani, Ali
TI The effects of neoadjuvant chemotherapy on resectability of

locally-advanced gastric adenocarcinoma: A clinical trial
SO INTERNATIONAL JOURNAL OF SURGERY
VL 12
IS 10
BP 1061
EP 1069
DI 10.1016/j.ijsu.2014.08.349
PD OCT 2014
PY 2014
AB Introduction: Surgical resection is the only curative treatment for gastric cancer. However, the overall prognosis of gastric adenocarcinoma is poor and advanced disease may even make surgical treatment impossible. It has been theoretically proposed that administration of chemotherapy before surgical resection may down-stage the disease state and facilitate resectability especially in locally-advanced tumors. Aim: We wanted to assess the effect of administration of neoadjuvant chemotherapy on tumor resectability in patients with locally-advances gastric adenocarcinoma. Materials and methods: During a randomized-controlled trial, we divided 60 patients with locally-advanced gastric adenocarcinoma into two groups of neoadjuvant chemotherapy and surgery (case) versus surgery alone (control). Because of patient dropouts, we analyzed the results for 22 and 29 patients in case and control groups respectively. The study period was March 21, 2011 to March 20, 2014. A non-randomized set of 23 patients were also added to the control group (Multi-center analysis). The analysis was repeated for non-randomized patients (22 case patients versus 52 control patients). Results: The mean age of patients in case and control groups was 58.3 +/- 9.1 and 59.7 +/- 8.7 years of age respectively ($p > 0.05$). Male to female ratio was 15/7 and 41/11 in case and control groups respectively ($p > 0.05$). In Randomized patients, 19 patients (86.4%) were resectable in case group; while 16 patients (55.2%) were resectable in control group ($p < 0.05$). Multicenter analysis also revealed resectability in 19 patients (86.4%) and 31 patients (59.6%) of case and control groups respectively ($p < 0.05$). Conclusion: We conclude that neoadjuvant chemotherapy could increase tumor resectability rate in patients with locally-advanced gastric adenocarcinoma. However, further studies are necessary to confirm the effect of this modality on patients' overall survival. (C) 2014 Surgical Associates Ltd. Published by Elsevier Ltd. All rights reserved.

TC 0
ZB 0
Z8 0
ZS 0
Z9 0
SN 1743-9191
UT WOS:000343588800008
PM 25157992
ER

PT J
AU Davies, Andrew R.
Gossage, James A.
Zylstra, Janine
Mattsson, Fredrik
Lagergren, Jesper
Maisey, Nick
Smyth, Elizabeth C.
Cunningham, David
Allum, William H.
Mason, Robert C.
TI Tumor Stage After Neoadjuvant Chemotherapy Determines Survival After Surgery for Adenocarcinoma of the Esophagus and Esophagogastric Junction
SO JOURNAL OF CLINICAL ONCOLOGY
VL 32
IS 27
BP 2983
EP +
DI 10.1200/JCO.2014.55.9070
PD SEP 20 2014
PY 2014
AB Purpose
Neoadjuvant chemotherapy is established in the management of most resectable esophageal and esophagogastric junction adenocarcinomas. However, assessing the downstaging effects of chemotherapy and predicting response to treatment remain challenging, and the relative importance of tumor stage before and after chemotherapy is debatable.
Methods
We analyzed consecutive resections for esophageal or esophagogastric

junction adenocarcinomas performed at two high-volume cancer centers in London between 2000 and 2010. After standard investigations and multidisciplinary team consensus, all patients were allocated a clinical tumor stage before treatment, which was compared with pathologic stage after surgical resection. Survival analysis was conducted using Kaplan-Meier analysis and Cox regression analysis.
Results
Among 584 included patients, 400 patients (68%) received neoadjuvant chemotherapy. Patients with downstaged tumors after neoadjuvant chemotherapy experienced improved survival compared with patients without response (P < .001), and such downstaging (hazard ratio, 0.43; 95% CI, 0.31 to 0.59) was the strongest independent predictor of survival after adjusting for patient age, tumor grade, clinical tumor stage, lymphovascular invasion, resection margin status, and surgical resection type. Patients downstaged by chemotherapy, compared with patients with no response, experienced lower rates of local recurrence (6% v 13%, respectively; P = .030) and systemic recurrence (19% v 29%, respectively; P = .027) and improved Mandard tumor regression scores (P = .001). Survival was strongly dictated by stage after neoadjuvant chemotherapy, rather than clinical stage at presentation.
Conclusion
The stage of esophageal or esophagogastric junction adenocarcinoma after neoadjuvant chemotherapy determines prognosis rather than the clinical stage before neoadjuvant chemotherapy, indicating the importance of focusing on postchemotherapy staging to more accurately predict outcome and eligibility for surgery. Patients who are downstaged by neoadjuvant chemotherapy benefit from reduced rates of local and systemic recurrence. (C) 2014 by American Society of Clinical Oncology
TC 0
ZB 0
Z8 0
ZS 0
Z9 0
SN 0732-183X
UT WOS:000342062200011
PM 25071104
ER

PT J

AU Noronha, Vanita
Joshi, Amit
Jandyal, Sunny
Jambhekar, Nirmala
Prabhash, Kumar
TI High pathologic complete remission rate from induction docetaxel, platinum and fluorouracil (DCF) combination chemotherapy for locally advanced esophageal and junctional cancer
SO MEDICAL ONCOLOGY
VL 31
IS 9
AR 188
DI 10.1007/s12032-014-0188-0
PD SEP 2014
PY 2014
AB Adding docetaxel to the cisplatin/5-fluorouracil induction regimen for locally advanced esophageal and GEJ cancer may increase the pathologic complete remission (pCR) rate, leading to an improved outcome. Institutional ethics committee approved the protocol of retrospective analysis of patients with locally advanced esophageal and GEJ carcinoma, who received 2-3 cycles of docetaxel, cisplatin and 5-fluorouracil (DCF) induction chemotherapy with primary growth factors and prophylactic antibiotics. Following chemotherapy, a restaging scan was performed. If disease was deemed resectable, surgery was performed. Between February 2010 and October 2013, 31 patients received induction DCF. Ninety-four percent patients had squamous histology. Response rate was 81 %: complete remission (CR)-23 % and partial remission-58 %. Eighty-seven percent patients underwent surgery; R0 resection rate was 67 %. pCR occurred in 26 %. Common grade 3/4 toxicities included anemia-23 %, neutropenia-42 %, febrile neutropenia39 %, diarrhea-39 %, hyponatremia-55 % and hypokalemia- 39 %. There were no toxic deaths. At a median follow-up of 34 months (95 % CI 31.3-36.6), estimated median progression-free survival (PFS) was 27 months (95 % CI 11-39) and the overall survival (OS) at 1 year, 2 years and 3 years was 80, 68 and 55 %, respectively. Patients who attained pCR had a significant longer PFS and OS; median PFS and OS were not reached in patients with pCR and were 15 months (95 % CI 8.4-21.5 months), P = 0.012 and 25 months (95 % CI 10.3-39.7), P = 0.023, respectively, in patients who did not attain a pCR. DCF induction chemotherapy leads to pCR of 26 %, which rivals that

obtained from chemoradiotherapy. Toxicity is substantial but manageable
with adequate supportive care.
TC 0
ZB 0
Z8 0
ZS 0
Z9 0
SN 1357-0560
UT WOS:000341835700054
PM 25148898
ER

PT J
AU Jary, Marine
Ghiringhelli, Francois
Jacquin, Marion
Fein, Francine
Nguyen, Thierry
Cleau, Denis
Nerich, Virginie
El Gani, Maryame
Mathieu, Pierre
Valmary-Degano, Severine
Arnould, Laurent
Lassabe, Catherine
Lamfichekh, Najib
Fratte, Serge
Paget-Bailly, Sophie
Bonnetain, Franck
Borg, Christophe
Kim, Stefano
TI Phase II multicentre study of efficacy and feasibility of
dose-intensified preoperative weekly cisplatin, epirubicin, and
paclitaxel (PET) in resectable gastroesophageal cancer
SO CANCER CHEMOTHERAPY AND PHARMACOLOGY
VL 74
IS 1
BP 141
EP 150

DI 10.1007/s00280-014-2482-0
PD JUL 2014
PY 2014
AB Perioperative chemotherapy improves the overall survival of resectable gastroesophageal adenocarcinoma (GEA) patients. However, more than 40 % of the patients are not healthy enough to complete their post-operative chemotherapy, and the progression-free survival rate is lower than 35 % at 5 years. In order to optimise neoadjuvant chemotherapy regimen, a pilot study of weekly dose-intensified cisplatin, epirubicin, and paclitaxel (PET) was conducted. The primary objective was a complete resection (R0) rate. Then, a R0 rate a parts per thousand currency sign80 % was considered as uninteresting, with an expected R0 rate of 92 %. Secondary objectives were the feasibility, safety, histological response rate (Becker score), and survival (Trial registration: NCT01830270).

Patients with > T1N0M0 GEA were included. Treatment consisted of eight preoperative cycles of weekly PET regimen at 30/50/80 mg/m(2) of cisplatin, epirubicin, and paclitaxel, respectively. Primary prophylaxis by granulocyte colony-stimulating factor was administered. Surgery was performed 4-6 weeks following the last cycle of chemotherapy. Using Fleming two-step design with a unilateral alpha type one error of 5 % and a statistical power of 80 %, it would be required to include 68 patients. At planned interim analysis for futility, it was required to observe at least 25 of 29 patients with R0 resection to pursue inclusion. At the second step, it was required to observe at least 61 of 68 patients with R0 resection to conclude for promising activity of the dose-intensified chemotherapy.

Between May 2011 and January 2013, 29 patients were enrolled. Median age was 62 years (range 39-83 years), and seven (24 %) patients presented signet-ring cell histology. Twenty-seven (93 %) patients underwent surgery. Pathological complete responses (Becker score 1a) were observed in four patients, and nearly complete responses (Becker score 1b) for additional three patients. A R0 rate was achieved for 24 of 29 (82.7 %; 95 % CI 64-94 %) patients. No Becker score 1a/1b response was observed among patients with signet-ring cell GEA. Twenty-one (72 %) patients completed all eight cycles, and 86 % received seven or more cycles. Sixteen (56 %) patients experienced grade 3-4 neutropenia, and five patients had febrile neutropenia. Among non-haematological toxicities, mucositis and fatigue were the most frequent ones. The median-delivered

relative dose intensity (DI) was 80 % for cisplatin, 75 % for epirubicin, and 79 % for paclitaxel. However, only 45 % of the patients received at least 80 % of the planned median DI for all three drugs. Despite high R0 and pathological response rates, neoadjuvant PET chemotherapy did not meet the primary end-point and failed to show an acceptable relative DI. PET chemotherapy is not recommended in resectable GEA patients.
TC 0
ZB 0
Z8 0
ZS 0
Z9 0
SN 0344-5704
UT WOS:000338759700014
PM 24824852
ER

PT J
AU Oki, Eiji
Emi, Yasunori
Kusumoto, Tetsuya
Sakaguchi, Yoshihisa
Yamamoto, Manabu
Sadanaga, Noriaki
Shimokawa, Mototsugu
Yamanaka, Takeharu
Saeki, Hiroshi
Morita, Masaru
Takahashi, Ikuo
Hirabayashi, Naoki
Sakai, Kenji
Orita, Hiroyuki
Aishima, Shinichi
Kakeji, Yoshihiro
Yamaguchi, Kazuya
Yoshida, Kazuhiro
Baba, Hideo
Maehara, Yoshihiko
TI Phase II Study of Docetaxel and S-1 (DS) as Neoadjuvant Chemotherapy for

Clinical Stage III Resectable Gastric Cancer
SO ANNALS OF SURGICAL ONCOLOGY
VL 21
IS 7
BP 2340
EP 2346
DI 10.1245/s10434-014-3594-9
PD JUL 2014
PY 2014
AB We conducted a phase II trial to evaluate the efficacy and safety of preoperative chemotherapy with docetaxel (DTX) plus S-1 for resectable advanced gastric cancer.
A total of 47 patients from 14 centers were centrally registered. Patients received DTX (35 mg/m(2)) on days 1 and 15, and daily oral administration of S-1 (80 mg/m(2)/day) for days 1-14 every 4 weeks for two courses, followed by gastrectomy with D2 lymphadenectomy. The primary endpoint was pathological response rate (pRR). This study was registered in the UMIN clinical trial registry (UMIN000000875).
The primary endpoint pRR was 47 % (90 % confidence interval (CI), 34-60 %; p < 0.0001). The response rate to preoperative chemotherapy using Response Evaluation Criteria in Solid Tumors (RECIST) was 34 %. Forty-six patients (98 %) underwent surgery, and curative resection was performed in 44 patients. Thirty-seven patients completed the protocol treatment. The most common toxicities of neoadjuvant chemotherapy were grade 3/4 neutropenia (42 %), febrile neutropenia (4 %), grade 2 anorexia (21 %), and fatigue (15 %). Treatment-related death and operative mortality was not observed in this study.
The combination of docetaxel and S-1 was well tolerated. This is promising as a preoperative chemotherapy regimen for patients with potentially resectable advanced gastric cancer.
TC 3
ZB 1
Z8 0
ZS 0
Z9 3
SN 1068-9265
UT WOS:000337063400035
PM 24604583
ER

PT J

AU Xiong, Bing-Hong
Cheng, Yong
Ma, Li
Zhang, Cai-Quan

TI An Updated Meta-Analysis of Randomized Controlled Trial Assessing the Effect of Neoadjuvant Chemotherapy in Advanced Gastric Cancer

SO CANCER INVESTIGATION
VL 32
IS 6
BP 272
EP 284
DI 10.3109/07357907.2014.911877
PD JUL 2014
PY 2014

AB Patients with locally advanced gastric cancer (AGC) have a poor outcome. We performed an updatedmeta-analysis to assess the effect of neoadjuvant chemotherapy (NAC). By searching electronic databases (PubMed, Embase, Cochrane Library) and ASCO proceedings from 1990 to 2012, all randomized controlled trials (RCTs) which compared the effect of NAC combined surgery versus surgery alone in advanced gastric and gastroesophageal cancer would be included. All calculations and statistical tests were performed. Twelve RCTs with a total of 1,820 patients were included. All patients had resectable gastric or gastroesophageal cancer and received NAC. NAC can slightly improve the survival rate [OR= 1.32, 95% confidence interval (CI): 1.07-1.64, P= 0.01], little, or no significant benefits were suggested in subgroup analyses between different population and regimens either. It can significantly improved the 3-year progression-free survival (PFS) [OR: 1.85 (1.39, 2.46), p<. 0001], tumor down-staging rate [OR: 1.71 (1.26, 2.33), p=. 0006] and R0 resection rate [OR: 1.38 (1.08, 1.78) p=. 01] of patients with AGC. There were no difference between the two arms, in terms of relapse rates [OR: 1.03 (0.60, 1.78), p= 0.92], operative complications [OR: 1.20 (0.90, 1.58), p= 0.21], perioperativemortality [OR: 1.14 (0.64, 2.05), p= 0.65], and grade 3/4 adverse effects. NAC can significantly down-stage the tumor and improve R0 resection rate of patients with gastric and gastroesophageal cancer. It is safe and feasible, and can be tolerated. NAC can slightly improve the survival rate. It needs further prospective

multinational multicenter RCTs to define the clinical benefits of NAC and the most effective strategies for gastric and gastroesophageal cancer.
TC 0
ZB 0
Z8 0
ZS 0
Z9 0
SN 0735-7907
UT WOS:000337025900009
PM 24800782
ER

PT J
AU Orditura, M.
Galizia, G.
Di Martino, N.
Ancona, E.
Castoro, C.
Pacelli, R.
Morgillo, F.
Rossetti, S.
Gambardella, V.
Farella, A.
Laterza, M. M.
Ruol, A.
Fabozzi, A.
Napolitano, V.
Iovino, F.
Lieto, E.
Fei, L.
Conzo, G.
Ciardiello, F.
De Vita, F.
TI Effect of preoperative chemoradiotherapy on outcome of patients with locally advanced esophagogastric junction adenocarcinoma-a pilot study
SO CURRENT ONCOLOGY
VL 21
IS 3

BP 125
EP 133
DI 10.3747/co.21.1570
PD JUN 2014
PY 2014
AB Background
To date, few studies of preoperative chemotherapy or chemoradiotherapy (CRT) in gastroesophageal junction (GEJ) cancer have been statistically powered; indeed, GEJ tumours have thus far been grouped with esophageal or gastric cancer in phase III trials, thereby generating conflicting results.
Methods
We studied 41 patients affected by locally advanced Siewert type I and II GEJ adenocarcinoma who were treated with a neoadjuvant CRT regimen [FOLFOX4 (leucovorin-5-fluorouracil-oxaliplatin) for 4 cycles, and concurrent computed tomography-based three-dimensional conformal radiotherapy delivered using 5 daily fractions of 1.8 Gy per week for a total dose of 45 Gy], followed by surgery. Completeness of tumour resection (performed approximately 6 weeks after completion of CRT), clinical and pathologic response rates, and safety and outcome of the treatment were the main endpoints of the study.
Results
All 41 patients completed preoperative treatment. Combined therapy was well tolerated, with no treatment-related deaths. Dose reduction was necessary in 8 patients (19.5%). After CRT, 78% of the patients showed a partial clinical response, 17% were stable, examination of surgical specimens demonstrated a 10% complete response rate. The median and mean survival times were 26 and 36 months respectively (95% confidence interval: 14 to 37 months and 30 to 41 months respectively). On multivariate analysis, TNM staging and clinical response were demonstrated to be the only independent variables related to long-term survival.
Conclusions
In our experience, preoperative chemoradiotherapy with FOLFOX4 is feasible in locally advanced GEJ adenocarcinoma, but shows mild efficacy, as suggested by the low rate of pathologic complete response.
RI Napolitano, Victoria/H-4399-2014
OI Napolitano, Victoria/0000-0001-5761-5872
TC 1

ZB 1
Z8 0
ZS 0
Z9 1
SN 1198-0052
UT WOS:000338495000020
PM 24940093
ER

PT J
AU Yoshikawa, Takaki
Tanabe, Kazuaki
Nishikawa, Kazuhiro
Ito, Yuichi
Matsui, Takanori
Kimura, Yutaka
Hasegawa, Shinichi
Aoyama, Toru
Hayashi, Tsutomu
Morita, Satoshi
Miyashita, Yumi
Tsuburaya, Akira
Sakamoto, Junichi
TI Accuracy of CT staging of locally advanced gastric cancer after neoadjuvant chemotherapy: cohort evaluation within a randomized phase II study.
SO Annals of surgical oncology
VL 21 Suppl 3
BP S385
EP 9
DI 10.1245/s10434-014-3615-8
PD 2014-Jun
PY 2014
AB BACKGROUND: Accuracy of the radiologic diagnosis of gastric cancer staging after neoadjuvant chemotherapy remains unclear.
METHODS: Patients enrolled in the COMPASS trial, a randomized phase II study comparing two and four courses of S-1 plus cisplatin and paclitaxel and cisplatin followed by gastrectomy, were examined. The radiologic stage was determined by using thin-slice computed tomography

(CT) or multidetector low CT by following Habermann's method.
RESULTS: A total of 75 patients registered in the COMPASS study who underwent surgical resection were examined in this study. The radiologic T and pathologic T stages were not significantly correlated (p = 0.221). The radiologic accuracy and rates of underdiagnosis and overdiagnosis were 42.7, 10.7, and 46.7%, respectively. When patients were stratified according to the pathologic response of the primary tumor, the correlation was not significant in either the responders (n = 32, p = 0.410) or the nonresponders (n = 43, p = 0.742). The radiologic accuracy was 37.5% in the responders and 42.7% in the nonresponders. The radiologic N and pathologic N stages were significantly correlated (p = 0.000). The radiologic accuracy and rates of underdiagnosis and overdiagnosis were 44, 29.3, and 26.7%, respectively. When stratifying the patients with measurable lymph nodes according only to the radiologic response, the correlation was significant in the nonresponders (n = 23, p = 0.035) but not in the responders (n = 28, p = 0.634). The radiologic accuracy was 39.3% in the responders and 52.1% in the nonresponders.
CONCLUSIONS: Restaging using CT after neoadjuvant chemotherapy for gastric cancer is considered to be inaccurate and unreliable. In particular, the radiologic T-staging determined after neoadjuvant chemotherapy should not be considered in clinical decision-making.
TC 0
ZB 0
Z8 0
ZS 0
Z9 0
UT MEDLINE:24595801
PM 24595801
ER

PT J
AU Nakano, Akira
Endo, Masato
Hirayama, Nobuo
Akai, Takashi
Matsubara, Hisahiro
TI [A case of advanced gastric cancer with para-aortic lymph node metastases responding to S-1/CDDP chemotherapy that leads to a

pathological complete response].
SO Gan to kagaku ryoho. Cancer & chemotherapy
VL 41
IS 5
BP 641
EP 3
PD 2014-May
PY 2014
AB A 66-year-old man complaining of epigastralgia was referred to our hospital. We examined the patient and diagnosed advanced gastric cancer (ML, type 3, por, cT3, cN3, cH0, cP0, cM1[LYM], cStage IV). A poor outcome was predicted, so we attempted induction chemotherapy and expected tumor downstaging. We chose S-1/CDDP therapy. S-1 was administered orally for 21 days, followed by CDDP div on day 8. Total gastrectomy and lymph node dissection (D2+No. 12a, No. 13,16) was performed using Roux-en-Y reconstruction. Histological examination of the resected stomach and lymph nodes revealed no residual cancer cells, suggesting complete histological remission (grade 3) according to the Japanese classification of gastric carcinoma. The patient has been in good health without recurrence for 12 months after surgery.
TC 0
ZB 0
Z8 0
ZS 0
Z9 0
SN 0385-0684
UT MEDLINE:24917013
PM 24917013
ER

PT J
AU Tsuburaya, A.
Mizusawa, J.
Tanaka, Y.
Fukushima, N.
Nashimoto, A.
Sasako, M.
CA Japan Clinical Oncology Grp
TI Neoadjuvant chemotherapy with S-1 and cisplatin followed by D2

gastrectomy with para- aortic lymph node dissection for gastric cancer
with extensive lymph node metastasis
SO BRITISH JOURNAL OF SURGERY
VL 101
IS 6
BP 653
EP 660
PD MAY 2014
PY 2014
CT 8th Annual Gastrointestinal Cancer Symposium of the
American-Society-of-Clinical-Oncology (ASCO)
CY JAN 20-22, 2011
CL San Francisco, CA
SP Amer Soc Clin Oncol
TC 1
ZB 1
Z8 0
ZS 0
Z9 1
SN 0007-1323
UT WOS:000334174200013
PM 24668391
ER

PT J
AU Heger, Ulrike
Blank, Susanne
Wiecha, Christiane
Langer, Rupert
Weichert, Wilko
Lordick, Florian
Bruckner, Thomas
Dobritz, Martin
Burian, Maria
Springfeld, Christoph
Grenacher, Lars
Siewert, Joerg-Ruediger
Buchler, Markus
Ott, Katja

TI Is Preoperative Chemotherapy Followed by Surgery the Appropriate Treatment for Signet Ring Cell Containing Adenocarcinomas of the Esophagogastric Junction and Stomach?

SO ANNALS OF SURGICAL ONCOLOGY

VL 21

IS 5

BP 1739

EP 1748

DI 10.1245/s10434-013-3462-z

PD MAY 2014

PY 2014

AB Background. Recent data suggest primary resection as the preferable approach in patients with signet ring cell gastric cancer (SRC). The aim of our retrospective exploratory study was to evaluate the influence of SRC on prognosis and response in esophagogastric adenocarcinoma treated with neoadjuvant chemotherapy.

Methods. A total of 723 locally advanced esophagogastric adenocarcinomas (cT3/4 N any) documented in a prospective database from two academic centers were classified according to the WHO definition for SRC (more than 50 % SRC) and analyzed for their association with response and prognosis after neoadjuvant treatment.

Results. A total of 235 tumors (32.5 %) contained SRC. Median survival of SRC was 26.3 compared with 46.6 months ($p < 0.001$) for non-SRC. SRC were significantly associated with female gender, gastric localization, advanced ypT and R1/2 categories, and lower risk of surgical complications and anastomotic leakage (each $p < 0.001$). Clinical (21.1 vs. 33.7 %, $p = 0.001$) and histopathological response (less than 10 % residual tumor: 16.3 vs. 28.9 %, $p < 0.001$) were significantly less frequent in SRC. Clinical response ($p = 0.003$) and complete histopathological response (pCR) (3.4 %) ($p = 0.003$) were associated with improved prognosis in SRC. Clinical response, surgical complications, ypTN categories, but not SRC were independent prognostic factors in forward Cox regression analysis in R0 resected patients. Risk of peritoneal carcinomatosis was increased ($p < 0.001$), while local ($p = 0.015$) and distant metastases ($p = 0.02$) were less frequent than in non-SRC.

Conclusions. Prognosis of SRC is unfavorable. Although response to neoadjuvant chemotherapy is rare in SRC, it is associated with improved outcome. Thus, chemotherapy might not generally be abandoned in SRC. A

stratification based on SRC should be included in clinical trials.
TC 1
ZB 0
Z8 0
ZS 0
Z9 1
SN 1068-9265
UT WOS:000334224900054
PM 24419755
ER

PT J
AU Peixoto, R. D.
Cheung, W. Y.
Lim, H. J.
TI Perioperative chemotherapy for gastroesophageal cancer in British Columbia: a multicentre experience
SO CURRENT ONCOLOGY
VL 21
IS 2
BP 77
EP 83
DI 10.3747/co.21.1788
PD APR 2014
PY 2014
AB Background
In 2006, perioperative epirubicin, cisplatin, and 5-fluorouracil (ECF), compared with surgery alone, demonstrated a significant survival benefit in resectable gastroesophageal cancers. We report the results of our experience with that protocol.
Methods
The BC Cancer Agency (BCCA) is a multicentre institution that treats most oncology patients for the province. Characteristics of the 83 BCCA patients with localized gastric, gastroesophageal junction, or lower esophageal cancer who initiated perioperative chemotherapy either ECF or epirubicin, cisplatin, and capecitabine (ECX) from 2008 to 2011 were abstracted to an anonymous database and analyzed.
Results
Of the 83 patients in the cohort [66 men; median age: 62 years (range:

37-79 years)], 87.9% completed 3 cycles of perioperative chemotherapy, and 93.9% (n = 78) underwent an attempt at surgery (2 patients died of chemotherapy toxicities, 1 refused surgery, and 2 developed disease progression before surgery). In 11 of the surgeries (14.1%), tumours could not be resected because of unresectability (n = 1), liver metastasis (n = 1), and peritoneal carcinomatosis (n = 9). One patient died of surgical complications. The 6 patients (7.2%) who achieved a pathologic complete response are all alive and recurrence-free. Of 46 patients (55.4%) who subsequently began postoperative chemotherapy, 44.5% completed 3 cycles. Estimated median survival was 40.3 months. Weight loss was the only significant prognostic factor for worse overall survival.
Conclusions
Our multicentre experience confirmed the feasibility of the magic protocol in a real-world scenario and showed that ECX is also an adequate regimen in the perioperative setting. Weight loss was the only significant prognostic factor for worse overall survival. All patients who achieved a pathologic complete response are recurrence-free after a median follow-up of 40.3 months.
TC 0
ZB 0
Z8 0
ZS 0
Z9 0
SN 1198-0052
UT WOS:000335387300024
PM 24764696
ER

PT J
AU Canbay, Emel
Mizumoto, Akiyoshi
Ichinose, Masumi
Ishibashi, Haruaki
Sako, Shouzou
Hirano, Masamitsu
Takao, Nobuyuki
Yonemura, Yutaka
TI Outcome Data of Patients with Peritoneal Carcinomatosis from Gastric

Origin Treated by a Strategy of Bidirectional Chemotherapy Prior to Cytoreductive Surgery and Hyperthermic Intraperitoneal Chemotherapy in a Single Specialized Center in Japan

SO ANNALS OF SURGICAL ONCOLOGY
VL 21
IS 4
BP 1147
EP 1152
DI 10.1245/s10434-013-3443-2
PD APR 2014
PY 2014

AB Background. Management of peritoneal disseminated gastric cancer (GC) remains a challenging problem. The purpose of our study was to evaluate the outcome of bidirectional induction chemotherapy [bidirectional intraperitoneal and systemic induction chemotherapy (BIPSC)] in patients with peritoneal carcinomatosis (PC) arising from GC who underwent cytoreductive surgery (CRS) and hyperthermic intraperitoneal chemotherapy (HIPEC).

Patients and Methods. Overall, 194 patients with PC arising from GC were treated with BIPSC comprising intraperitoneal docetaxel at a dose of 20 mg/m(2) and cisplatin at a dose of 30 mg/m(2) followed by four cycles of oral S-1 at a dose of 60 mg/m(2). CRS and HIPEC were performed in responders to BIPSC.

Results. Of these 194 patients, 152 (78.3 %) underwent CRS and HIPEC between January 2005 and December 2012. Treatment-related mortality was 3.9 %, and major complications occurred in 23.6 % of patients. The median survival rate was 15.8 months, with 1-, 2-, and 5-year survival rates of 66, 32 and 10.7 %, respectively, in the patients treated with combined treatment. Multivariate analysis identified pathologic response to BIPSC (p = 0.001), low tumor burden [peritoneal cancer index (PCI) <= 6] (p = 0.001), and completeness of CRS (CC-0, CC-1) (p = 0.001) as independent predictors for a better prognosis.

Conclusion. As a viable option, BIPSC with CRS and HIPEC for patients with PC arising from GC may be performed safely, with acceptable morbidity and mortality, in a specialized unit. Response to BIPSC, optimal CRS and limited peritoneal dissemination seem to be essential to achieve the best outcomes in these patients.

TC 2
ZB 1

Z8 0
ZS 0
Z9 2
SN 1068-9265
UT WOS:000334222500016
PM 24356799
ER

PT J
AU Michel, Pierre
   Breysacher, Gilles
   Mornex, Francoise
   Seitz, Jean Francois
   Pere-Verge, Denis
   Martel-Lafay, Isabelle
   Faroux, Roger
   Chapet, Sophie
   Sobhani, Iradj
   Pezet, Denis
   Aparicio, Thomas
   Nguyen, Suzanne
   Dousset, Bertrand
   Jouve, Jean-Louis
   Maillard, Emilie
TI Feasibility of preoperative and postoperative chemoradiotherapy in
   gastric adenocarcinoma. Two phase II studies done in parallel.
   Federation Francophone de Cancerologie Digestive 0308
SO EUROPEAN JOURNAL OF CANCER
VL 50
IS 6
BP 1076
EP 1083
DI 10.1016/j.ejca.2013.12.009
PD APR 2014
PY 2014
AB Background: For resectable gastric cancer, both postoperative
   chemoradiotherapy and perioperative chemotherapy demonstrate high-level
   evidence for improved survival in Western populations. To evaluate the
   feasibility of pre- or postoperative chemoradiotherapy, we proposed two

multicentre phase II studies.
Patients and methods: Patients with localised, histologically confirmed gastric cancer and Eastern Cooperative Oncology Group (ECOG) performance status <2 judged suitable for curative resection were eligible. Eligible patients were assigned to either preoperative chemoradiotherapy followed by surgical resection or surgical resection followed by chemoradiotherapy depending on each centre. Chemoradiotherapy regimen included four courses of FOLF-IRI (5 Fluorouracil, Leucovorin, Irinotecan) regimen then Concurrent fluorouracil at 200 mg/m(2)/d by continuous infusion 5 days each week. A dose of 50 Gy in 25 fractions in the preoperative study, or 45 Gy in 25 fractions in the postoperative study, was delivered. The primary end-point for both studies was the proportion of patients, who completed the therapeutic sequence.
Results: Between September 2007 and January 2010, 63 patients were included in both studies. The postoperative study was stopped for futility at the first step. In the preoperative study, 31 patients (73.8%, confidence interval (CI) 95%: 65.8-90.1%) received complete therapeutic sequence. Serum albumin and dietary restriction evaluated by QLQ-STO22 (Quality of Life-Stomach module) score were significantly linked with chemoradiotherapy feasibility in univariate analysis with respectively Odds-ratio (OR) 1.16 [CI 95%: 1.01-1.33] and 0.17 [0.03-0.89], p = 0.04. Median overall survival time was 26.4 months in the preoperative study.
Conclusion: Feasibility of chemoradiotherapy was not achieved for these studies: 73.8% (CI 95%: 65.8-90.1) and 42.9% (CI 95%: 21.8-66%) in preoperative and postoperative settings respectively. (C) 2014 Published by Elsevier Ltd.
TC 2
ZB 1
Z8 0
ZS 0
Z9 2
SN 0959-8049
UT WOS:000333255200004
PM 24433843
ER

PT J
AU Chu, Dake

Zhao, Zhengwei
Li, Yunming
Li, Jipeng
Zheng, Jianyong
Wang, Weizhong
Zhao, Qingchuan
Ji, Gang
TI Increased MicroRNA-630 Expression in Gastric Cancer Is Associated with Poor Overall Survival
SO PLOS ONE
VL 9
IS 3
AR e90526
DI 10.1371/journal.pone.0090526
PD MAR 12 2014
PY 2014
AB MicroRNAs are noncoding RNAs that regulate multiple cellular processes during cancer progression. Among various microRNAs, MiR-630 has recently been identified to be implicated in many critical processes in human malignancies. We aimed to investigate the significance and prognostic value of miR-630 in human gastric cancer. Gastric cancer and adjacent normal specimens from 236 patients from who had not received neoadjuvant chemotherapy were collected. The expression of miR-630 was investigated by quantitative real-time PCR assay and its association with overall survival of patients was analyzed by statistical analysis. MiR-630 expression level was significantly elevated in gastric cancer in comparison to adjacent normal specimens. It is also proved that miR-630 expression was to be associated with gastric cancer invasion, lymph node metastasis, distant metastasis and TNM stage. In addition, survival analysis proved that elevated miR-630 expression was associated with poor overall survival of patients. Multivariate survival analysis also proved that miR-630 was an independent prognostic marker after adjusted for known prognostic factors. The present study proved the overexpression of miR-630 and its association with tumor progression in human gastric cancer. It also provided the first evidence that miR-630 expression was an independent prognostic factor for patients with gastric cancer, which might be a potential valuable biomarker for gastric cancer.
TC 2

ZB 2
Z8 0
ZS 0
Z9 2
SN 1932-6203
UT WOS:000332845300023
PM 24621930
ER

PT J
AU Schulze, Bjoern
Bergis, Dominik
Balermpas, Panagiotis
Trojan, Joerg
Woeste, Guido
Bechstein, Wolf Otto
Roedel, Claus
Weiss, Christian
TI Neoadjuvant chemoradiation versus perioperative chemotherapy followed by surgery in resectable adenocarcinomas of the esophagogastric junction: A retrospective single center analysis
SO ONCOLOGY LETTERS
VL 7
IS 2
BP 534
EP 540
DI 10.3892/ol.2013.1709
PD FEB 2014
PY 2014
AB The current study presents a retrospective comparison, performed at a single academic center, of preoperative chemoradiation (CRT) and perioperative chemotherapy (CT) in addition to surgery in locally advanced but resectable adenocarcinoma of the esophagogastric junction (AEG). A total of 29 consecutive patients with locally advanced AEGs were retrospectively analyzed. Treatment consisted of preoperative CRT (mean dose, 45.0 Gy) plus two cycles of CT with cisplatin and 5-FU or perioperative CT with epirubicin, cisplatin and capecitabine (three cycles preoperatively and postoperatively). Within four to six weeks following preoperative treatment, surgical therapy was performed. Median

overall survival was 21.0 months in the perioperative CT group versus 41.7 months in the CRT group [P=0.36; hazard ratio (HR), 1.50; 95% confidence interval (CI), 0.58-3.84]. Three-year survival rates were 55 and 38%, respectively, in favor of the CRT group, and progression-free survival was 20.0 months in the CT group compared with 24.1 months in the CRT group (P=0.71; HR, 1.19; 95% CI, 0.46-3.05). The total number of major surgical complications was almost equal in the two groups. Margin-free resections were achieved in all patients of the CRT group, but only 76.9% of the CT group (P=0.05). In addition, significantly higher R0 resection rates and an increased number of pathological complete remissions were demonstrated in the CRT group compared with those of the CT group. These results appear to indicate a trend for improved progression-free and overall survival for the CRT group. As postoperative morbidity and mortality rates were similar in the two groups, the results support the use of CRT for patients with advanced AEG tumors.

TC 0

ZB 0

Z8 0

ZS 0

Z9 0

SN 1792-1074

UT WOS:000332693400047

ER

PT J

AU Nakanoko, Tomonori

Saeki, Hiroshi

Morita, Masaru

Nakashima, Yuichiro

Ando, Koji

Oki, Eiji

Ohga, Takefumi

Kakeji, Yoshihiro

Toh, Yasushi

Maehara, Yoshihiko

TI Rad51 Expression Is a Useful Predictive Factor for the Efficacy of Neoadjuvant Chemoradiotherapy in Squamous Cell Carcinoma of the Esophagus

SO ANNALS OF SURGICAL ONCOLOGY
VL 21
IS 2
BP 597
EP 604
DI 10.1245/s10434-013-3220-2
PD FEB 2014
PY 2014
AB Background. Neoadjuvant chemoradiotherapy (NACRT) for esophageal squamous cell carcinoma (ESCC) is beneficial in the setting of a complete pathological response. Rad51 expression affects both chemo-and radiosensitivity in many cancers; however, its role in ESCC is unclear.
Methods. Rad51 expression was investigated by immunohistochemical staining with resected specimens in 89 ESCC patients who underwent surgery without preoperative therapy. The association with Rad51 and clinicopathological factors was assessed. The expression of Rad51 was also investigated in pretreatment biopsy specimens in 39 ESCC patients who underwent surgery after NACRT and compared with the pathological response to NACRT.
Results. Lymph node metastasis was more frequently observed in Rad51-positive cases than negative cases (58.5 vs. 30.6 %, P = 0.0168) in patients treated with surgery alone. Disease-specific survival was decreased in Rad51-positive cases compared to Rad51-negative cases (5 year survival: 79.6 vs. 59.3 %, P = 0.0324). In NACRT patients, completed pathological responses were more frequently observed in Rad51-negative cases than in Rad51-positive cases (68.8 vs. 46.5 %, P = 0.0171).
Conclusions. Rad51 expression in ESCC was associated with lymph node metastasis and poor survival. Additionally, Rad51 expression in pretreatment biopsy specimens was a predictive factor for the response to NACRT.
TC 2
ZB 3
Z8 1
ZS 0
Z9 5
SN 1068-9265
UT WOS:000332673800037
PM 24065387

ER

PT J
AU Chen, Wenjun
   Shen, Jianguo
   Pan, Tao
   Hu, Wenxian
   Jiang, Zinong
   Yuan, Xiaoming
   Wang, Linbo
TI FOLFOX versus EOX as a neoadjuvant chemotherapy regimen for patients with advanced gastric cancer
SO EXPERIMENTAL AND THERAPEUTIC MEDICINE
VL 7
IS 2
BP 461
EP 467
DI 10.3892/etm.2013.1449
PD FEB 2014
PY 2014
AB Neoadjuvant chemotherapy is the preferred treatment of advanced gastric cancer. However, the choice of an optimal regimen remains controversial. The present study aimed to assess the effectiveness of preoperative chemotherapy with EOX and FOLFOX in Chinese patients with advanced gastric cancer. A total of 87 and 26 patients underwent FOLFOX and EOX regimens, respectively, for advanced gastric cancer between July 2004 and September 2012. Clinicopathological characteristics, pathological T stage, N stage and pathological response to tumour regression were retrospectively compared between the two groups. Following neoadjuvant chemotherapy, a higher number of patients manifested deeper invasive cancer in the FOLFOX group than those in the EOX group (P=0.047). In addition, a higher number of patients also exhibited metastatic lymph nodes in the FOLFOX group (67.8%) than in the EOX group (57.7%) (P=0.000). In the FOLFOX and EOX groups, 4 (4.6%) and 3 (11.5%) cases of complete regression were observed, respectively. A higher number of patients (38.5%) also exhibited tumour regression grades of 3 and 4 in the EOX group than in the FOLFOX group (19.5%) (P=0.047). Results of the present study suggest that the EOX regimen may be more effective than the FOLFOX regimen as preoperative chemotherapy for Chinese patients

with advanced gastric cancer. The EOX regimen may be suitable for younger patients subjected to individual neoadjuvant chemotherapy.
TC 1
ZB 0
Z8 0
ZS 0
Z9 1
SN 1792-0981
UT WOS:000332690900031
ER

PT J
AU Ajani, J. A.
Wang, X.
Song, S.
Suzuki, A.
Taketa, T.
Sudo, K.
Wadhwa, R.
Hofstetter, W. L.
Komaki, R.
Maru, D. M.
Lee, J. H.
Bhutani, M. S.
Weston, B.
Baladandayuthapani, V.
Yao, Y.
Honjo, S.
Scott, A. W.
Skinner, H. D.
Johnson, R. L.
Berry, D.
TI ALDH-1 expression levels predict response or resistance to preoperative chemoradiation in resectable esophageal cancer patients
SO MOLECULAR ONCOLOGY
VL 8
IS 1
BP 142
EP 149

DI 10.1016/j.molonc.2013.10.007
PD FEB 2014
PY 2014
AB Purpose: Operable thoracic esophageal/gastroesophageal junction carcinoma (EC) is often treated with chemoradiation and surgery but tumor responses are unpredictable and heterogeneous. We hypothesized that aldehyde dehydrogenase-1 (ALDH-1) could be associated with response.
Methods: The labeling indices (LIs) of ALDH-1 by immunohistochemistry in untreated tumor specimens were established in EC patients who had chemoradiation and surgery. Univariate logistic regression and 3-fold cross validation were carried out for the training (67% of patients) and validation (33%) sets. Non-clinical experiments in EC cells were performed to generate complimentary data.
Results: Of 167 EC patients analyzed, 40 (24%) had a pathologic complete response (pathCR) and 27 (16%) had an extremely resistant (exCRTR) cancer. The median ALDH-1 LI was 0.2 (range, 0.01-0.85). There was a significant association between pathCR and low ALDH-1 LI (p <= 0.001; odds-ratio [OR] = 0.432). The 3-fold cross validation led to a concordance index (C-index) of 0.798 for the fitted model. There was a significant association between exCRTR and high ALDH-1 LI (p <= 0.001; OR = 3.782). The 3-fold cross validation led to the C-index of 0.960 for the fitted model. In several cell lines, higher ALDH-1 Us correlated with resistant/aggressive phenotype. Cells with induced chemotherapy resistance upregulated ALDH-1 and resistance conferring genes (SOX9 and YAP1). Sorted ALDH-1+ cells were more resistant and had an aggressive phenotype in tumor spheres than ALDH-1- cells.
Conclusions: Our clinical and non-clinical data demonstrate that ALDH-1 Us are predictive of response to therapy and further research could lead to individualized therapeutic strategies and novel therapeutic targets for EC patients. (C) 2013 Federation of European Biochemical Societies. Published by Elsevier B.V. All rights reserved.
TC 1
ZB 1
Z8 0
ZS 0
Z9 1
SN 1574-7891
UT WOS:000331510100014

PM 24210755
ER

PT J
AU Hu, Jian-Bin
Sun, Xiao-Nan
Gu, Ben-Xing
Wang, Qi
Hu, Wen-Xian
TI Effect of Intensity Modulated Radiotherapy Combined with S-1-Based Chemotherapy in Locally Advanced Gastric Cancer Patients
SO ONCOLOGY RESEARCH AND TREATMENT
VL 37
IS 1-2
BP 11
EP 16
DI 10.1159/000358164
PD FEB 2014
PY 2014
AB Background: The optimal radiotherapy technique and combination with systemic therapy in locally advanced gastric cancer patients are far from being resolved despite the fact that radiochemotherapy is becoming more attractive in contemporary clinical practice. Patients and Methods: 40 patients with locally advanced gastric cancer received intensity-modulated radiotherapy (IMRT) at a dosage of 45-50.4 Gy concurrent with chemotherapy using S-1 solely or with a combination of oxaliplatin. Surgery was recommended for those who were evaluated as resectable. Sequential chemotherapy with various regimens was adopted based on the efficacy and tolerance of radiochemotherapy. Results: The overall response rate was 75% according to Response Evaluation Criteria in Solid Tumors and Japanese Gastric Cancer Association criteria. 24 finally underwent surgery, with 22 (91.7%) receiving an R0 resection (resection for cure or complete remission). The overall pathological response rate was 37.5% (9/24). Patients receiving an R0 resection had a higher 2-year overall survival rate (64.7 vs. 16.2%, p = 0.001) and local relapse-free survival rate (90.2 vs. 29.3%, p = 0.000), while there was no difference in distant metastasis-free survival rate (66.1 and 48.1% p = 0.231). Hematological and gastrointestinal toxicities of grade 1 or grade 2 were relatively common. Conclusion: The high rate of

R0 resections and low rate of locoregional recurrence suggest that IMRT combined with S-1-based chemotherapy is an effective treatment for locally advanced gastric cancer patients.
TC 0
ZB 0
Z8 0
ZS 0
Z9 0
SN 2296-5270
UT WOS:000332308100002
PM 24613903
ER

PT J
AU Xu, A-Man
Huang, Lei
Liu, Wei
Gao, Shuang
Han, Wen-Xiu
Wei, Zhi-Jian
TI Neoadjuvant Chemotherapy Followed by Surgery versus Surgery Alone for Gastric Carcinoma: Systematic Review and Meta-Analysis of Randomized Controlled Trials
SO PLOS ONE
VL 9
IS 1
AR e86941
DI 10.1371/journal.pone.0086941
PD JAN 30 2014
PY 2014
AB Background: The effect of neoadjuvant chemotherapy (NAC) on Gastric carcinoma (GC) has been extensively studied, while its survival and surgical benefits remain controversial. This study aims to perform a meta-analysis of high-quality randomized controlled trials (RCTs), comparing efficacy, safety and other outcomes of NAC followed by surgery with surgery alone (SA) for GC.
Methods: We systematically searched databases of MEDLINE, EMBASE, The Cochrane Library and Springer for RCTs comparing NAC with SA when treating GC. Reference lists of relevant articles and reviews,

conference proceedings and ongoing trial databases were also searched. Primary outcomes were 3-year and 5-year survival rates, survival time, and total and perioperative mortalities. Secondary outcomes included down-staging effects, R0 resection rate, and postoperative complications. Meta-analysis was conducted where possible comparing items using relative risks (RRs) and weighted mean differences (WMDs) according to type of data. NAC-related objective response, safety and toxicity were also specifically analyzed.
Results: A total of 9 RCTs comparing NAC (n = 511) with SA (n = 545) published from 1995 to 2010 were identified. SA tended to be accompanied with higher overall mortality rate than NAC (46.03% vs 40.61%, RR: 0.83, 95% CI: 0.65-1.06, P = 0.14). Significantly, higher incidence of cases without regional lymph node metastasis observed upon resection were achieved among patients receiving NAC than those undergoing SA (25.68% vs 16.95%, RR: 1.92, 95% CI: 1.20-3.06, P = 0.006). All other parameters were comparable. Of the evaluable patients, 43.0% demonstrated either complete or partial response. The comprehensive NAC-related side-effect rate was 18.2% among patients available for safety assessment.
Conclusions: NAC contributes to lowering nodal stages, and potentially reduces overall mortality. Response rate may be an important influential factor impacting advantages, with chemotherapy-related adverse effects as a drawback. This level 1a evidence doesn't support NAC to outweigh SA in terms of survival and surgical benefits when dealing with GC.
TC 1
ZB 0
Z8 1
ZS 0
Z9 1
SN 1932-6203
UT WOS:000330617100041
PM 24497999
ER

PT J
AU Jia, Y.
Ye, L.
Ji, K.
Zhang, L.
Hargest, R.

Ji, J.
Jiang, W. G.
TI Death-associated protein-3, DAP-3, correlates with preoperative chemotherapy effectiveness and prognosis of gastric cancer patients following perioperative chemotherapy and radical gastrectomy
SO BRITISH JOURNAL OF CANCER
VL 110
IS 2
BP 421
EP 429
DI 10.1038/bjc.2013.712
PD JAN 21 2014
PY 2014
AB Background: DAP3 is a member of the death-associated protein (DAP) family and is characterised by proapoptotic function. It is involved in both exogenous and endogenous apoptotic pathways. In our previous studies, apoptotic level was found to be correlated with the effectiveness of preoperative chemotherapy. The effectiveness of preoperative chemotherapy was also associated with the overall effectiveness of the combined therapy and prognosis. The present study aimed to investigate the role of DAP3 in the evaluation of preoperative chemotherapy effectiveness and its ability to predict prognosis in gastric cancer.
Methods: Quantitative PCR and immunohistochemistry staining were performed in 87 patients who received combined therapy. Knockdown of DAP3 was conducted in gastric cancer cell lines to investigate its impact on cell growth, migration, adhesion and invasion. Tolerance to chemotherapy agents was determined by assessing apoptosis and caspase-3.
Results: Higher DAP3 expression in gastric tumours was correlated with better prognosis. Knockdown of DAP3 expression promoted cell migration and enhanced resistance to chemotherapy by inhibiting apoptosis.
Conclusion: DAP3 is a potential molecular marker for response to preoperative chemotherapy and for predicting prognosis in gastric cancer patients treated with neoadjuvant chemotherapy and gastrectomy.
TC 0
ZB 0
Z8 0
ZS 0
Z9 0

SN 0007-0920
UT WOS:000330354700019
PM 24300973
ER

PT J
AU Li, Tao
Liang, Meixia
Yuan, Jing
Guo, Xu
Feng, Daofu
Li, Ting
Teng, Da
Peng, Zheng
Wu, Xin
Li, Zexue
Wang, Hua
Chen, Lin
TI [Correlated analysis of 5 fluorouracil metabolic enzymes with tumor response after SOX regimen neoadjuvant chemotherapy in advanced gastric cancer].
SO Zhonghua yi xue za zhi
VL 94
IS 2
BP 127
EP 30
PD 2014-Jan-14
PY 2014
AB OBJECTIVE: To analyze the impact of mRNA expression of oral fluoropyrimidine (S-1) metabolism on treatment outcomes in locally advanced gastric cancer patients on preoperative S-1 oxaliplatin-based chemotherapy.
METHODS: Between June 2012 and March 2013, 32 patients with preoperative AJCC stage II-III gastric cancer patients were enrolled. They received S-1 (80 mg•m⁻ * d⁻, days 1-14) and oxaliplatin (130 mg/m, day 1) every 3 weeks and subsequently underwent gastrectomy with D2 lymphadenectomy. Paired tumor and normal fresh frozen tissues were collected to evaluate the mRNA levels of thymidylate synthase (TS), thymidine phosphorylase (TP), dihydropyrimidine dehydrogenase (DPD) and OPRT with quantitative

reverse transcription(RT) -PCR.
RESULTS: Among them, 21 (65.6%) patients had clinical tumor response and histological response occurred in 10 (31.3%) patients. Quantitative RT-PCR results showed that OPRT mRNA expression was significantly higher in clinical tumor responders than non-responders (3.95 ± 0.81 vs 1.79 ± 0.64, P = 0.005). Diffuse-type gastric cancer patients (n = 22) demonstrated higher OPRT expression levels than intestinal-type(n = 10) ones (2.54 ± 0.75 vs 1.49 ± 0.56, P = 0.014). The mRNA expressions of TS and TP in gastric cancer tissues with lymph node (LN) metastasis (n = 13) were significantly higher than those in gastric cancer tissues without LN metastasis (n = 19, both P < 0.05) .Similar results were not found for comparing dihydropyrimidine dehydrogenase expression levels (all P > 0.05).
CONCLUSION: OPRT, TS and TP may become potential predictive biomarkers in advanced gastric cancer patients on oral fluoropyrimidine (S-1)-based chemotherapy.
TC 0
ZB 0
Z8 0
ZS 0
Z9 0
SN 0376-2491
UT MEDLINE:24721353
PM 24721353
ER

PT J
AU Matsuda, Satoru
Takahashi, Tsunehiro
Fukada, Junichi
Fukuda, Kazumasa
Kawakubo, Hirofumi
Saikawa, Yoshiro
Kawaguchi, Osamu
Takeuchi, Hiroya
Shigematsu, Naoyuki
Kitagawa, Yuko
TI Phase I study of neoadjuvant chemoradiotherapy with S-1 plus biweekly cisplatin for advanced gastric cancer patients with lymph node

metastasis:-KOGC04-
SO RADIATION ONCOLOGY
VL 9
AR 9
DI 10.1186/1748-717X-9-9
PD JAN 8 2014
PY 2014
AB Background: In patients with highly advanced gastric cancer, the recurrence rate remains high and the prognosis disappointing. We previously reported a phase I study of a neoadjuvant chemoradiotherapy of S-1 plus weekly cisplatin. Although adequate safety and efficacy were reported, myelosuppression was frequently observed, leading to treatment delay in several cases. To decrease toxicity and improve efficacy, we planned a phase I study with a modified chemotherapy regimen with biweekly cisplatin.
Methods: Patients with advanced gastric cancer and lymph node metastasis who were treated by our institution between 2011 and 2012 were eligible for inclusion. The initial chemoradiotherapy schedule consisted of 6 weeks of S-1 orally administered on days 1-15 with an escalating dose of cisplatin administered on days 1 and 15. The starting dose (level 1) of cisplatin was 15 mg/m(2), the second dose (level 2) was 20 mg/m(2), and the third dose (level 3) was 25 mg/m(2). Radiation of 40 Gy was administered in 20 fractions. After initial chemoradiotherapy, one cycle of combination chemotherapy with S-1 plus cisplatin was delivered. The second cycle was 42 days in duration and included S-1 administered on days 1-29 plus biweekly cisplatin administered on days 1, 15, and 29. After neoadjuvant treatment, a curative gastrectomy with extended (D2) lymph node dissection was planned.
Results: Nine patients were enrolled. At level 3, one patient had dose-limiting grade 3 diarrhea. Another patient experienced grade 3 nausea and intended to discontinue the treatment. Overall, because 2 of 3 patients experienced dose-limiting toxicity at level 3, we confirmed level 3 (Cisplatin 25 mg/m(2)) as the maximum tolerated dose and level 2 (Cisplatin 20 mg/m(2)) as the recommended dose (RD). The response rate was 78%, and 8 patients underwent curative gastrectomy. Resected specimens showed a histological response in 6 patients (75%), including one with a pathological complete response.
Conclusions: In this phase I trial, RD of cisplatin was identified as 20 mg/m(2). Generally, S-1 plus biweekly cisplatin can be given safely with

concurrent radiation. We have initiated a multicenter phase II trial to further confirm the efficacy and safety of this approach.
TC 2
ZB 0
Z8 0
ZS 0
Z9 2
SN 1748-717X
UT WOS:000331625900001
PM 24398302
ER

PT J
AU Sun, Zhipeng
Zhang, Nengwei
TI Clinical evaluation of CEA, CA19-9, CA72-4 and CA125 in gastric cancer patients with neoadjuvant chemotherapy.
SO World journal of surgical oncology
VL 12
BP 397
EP 397
DI 10.1186/1477-7819-12-397
PD 2014 Dec 29
PY 2014
AB BACKGROUND: In the clinical practice of neoadjuvant chemotherapy, response markers are very important. We aimed o investigate whether tumor markers CEA(carcino-embryonic antigen), CA19-9(carbohydrate antigen 19-9), CA72-4(carbohydrate antigen 72-4), and CA125(carbohydrate antigen 125) can be used to evaluate the response to neoadjuvant chemotherapy, and to evaluate the diagnosis and prognosis value of four tumor markers in the patients of gastric cancer.
METHODS: A retrospective review was performed of 184 gastric cancer patients who underwent a 5-Fu, leucovorin, and oxaliplatin (FOLFOX) neoadjuvant chemotherapy regimen, followed by surgical treatment. Blood samples for CEA, CA19-9, CA72-4, and CA125 levels were taken from patients upon admission to the hospital and after neoadjuvant chemotherapy. Statistical analysis was performed to identify the clinical value of these tumor markers in predicting the survival and the response to neoadjuvant chemotherapy.

RESULTS: Median overall survival times of pretreatment CA19-9-positive and CA72-4-positive patients (14.0 +/-2.8months and 14.8 +/-4.0months, respectively) were significantly less than negative patients (32.5 +/-8.9months and 34.0 +/-10.1months, respectively) (P=0.000 and P=0.002, respectively). Pretreatment status of CA19-9 and CA72-4 were independent prognostic factors in gastric cancer patients (P=0.029 and P=0.008, respectively). Pretreatment CEA >50ng/ml had a positive prediction value for clinical disease progression after neoadjuvant chemotherapy according to the ROC curve (AUC: 0.694, 95% CI: 0.517 to 0.871, P=0.017). The decrease of tumor markers CEA, CA72-4, and CA125 was significant after neoadjuvant chemotherapy (P=0.030, P=0.010, and P=0.009, respectively), especially in patients with disease control (including complete, partial clinical response, and stable disease) (P=0.012, P=0.020, and P=0.025, respectively). A decrease in CA72-4 by more than 70% had a positive prediction value for pathologic response to neoadjuvant chemotherapy according to the ROC curve (AUC: 0.764, 95% CI: 0.584 to 0.945, P=0.020).
CONCLUSIONS: Our results suggest that high preoperative serum levels of CA72-4 and CA19-9 are associated with higher risk of death, high pretreatment CEA levels (>50ng/ml) may predict clinical disease progression after neoadjuvant chemotherapy, and a decrease (>70%) of CA72-4 may predict pathologic response to neoadjuvant chemotherapy.
TC 0
ZB 0
Z8 0
ZS 0
Z9 0
UT MEDLINE:25543664
PM 25543664
ER

PT J
AU Izuishi, Kunihiko
Kobayashi, Mitsuyoshi
Sano, Takanori
Mori, Hirohito
Ebara, Kazuo
TI Pathological complete response and long-term survival in a very elderly patient after neoadjuvant chemotherapy for locally advanced,

unresectable gastric cancer.
SO Case reports in oncological medicine
VL 2014
BP 532924
EP 532924
DI 10.1155/2014/532924
PD 2014
PY 2014
AB We address the pathological complete response and long-term survival of elderly patients after neoadjuvant chemotherapy in locally advanced, unresectable gastric cancer. An 83-year-old man was hospitalized for upper abdominal pain. Gastrointestinal endoscopy showed a large tumor spanning from the gastric angle to the antrum, and extending to the duodenum. Histological analysis of the biopsy specimen revealed a poorly differentiated adenocarcinoma. Computed tomography images showed thickening of the gastric wall and invasion of the body and head of the pancreas, but did not show distant metastases. The patient was diagnosed with unresectable gastric cancer, and was treated with neoadjuvant chemotherapy using S-1 (80mg/m(2)) and paclitaxel (60mg/m(2)). After the third course of chemotherapy, gastrointestinal endoscopy and abdominal computed tomography revealed a remarkable reduction in tumor size. This reduction allowed distal gastrectomy to be conducted. Histological examination of the specimen revealed no cancer cells in the primary lesion or lymph nodes. The patient was treated with adjuvant chemotherapy of oral tegafur-uracil (300mg/day) for one year after surgery. He lived for five years after surgery without recurrence. Neoadjuvant chemotherapy using S-1 and paclitaxel is a potent strategy for improving survival in very elderly patients with unresectable gastric cancer.
TC 0
ZB 0
Z8 0
ZS 0
Z9 0
SN 2090-6706
UT MEDLINE:25298899
PM 25298899
ER

PT J
AU Zhang, Chun
Yao, Cong
Li, Haopeng
Wang, Guoyu
He, Xijing
TI Serum levels of microRNA-133b and microRNA-206 expression predict prognosis in patients with osteosarcoma
SO INTERNATIONAL JOURNAL OF CLINICAL AND EXPERIMENTAL PATHOLOGY
VL 7
IS 7
BP 4194
EP 4203
PD 2014
PY 2014
AB The aim of the present study was to investigate whether the aberrant expression of microRNA (miR)-133b and miR-206 can be used as potential prognostic markers of human osteosarcoma. Quantitative real-time reverse transcriptase-polymerase chain reaction (qRT-PCR) analysis was performed to detect the expression levels of miR-133b and miR-206 in 100 pairs of osteosarcoma tissues and matched noncancerous bone tissues, and serum samples from 100 patients with osteosarcoma as well as in serum samples from 100 healthy controls. As a result, expression levels of miR-133b and miR-206 were both significantly decreased in osteosarcoma tissues and patients' sera (both P<0.001). Then, the downregulation of miR-133b and miR-206 both more frequently occurred in osteosarcoma patients with high tumor grade (both P=0.01), positive metastasis (both P<0.001) and recurrence (both P<0.001). Moreover, the patients with low miR-133b expression and low miR-206 expression both had shorter overall survival (OS, both P<0.001) and disease-free survival (DFS, both P<0.001) than those with high expressions. Of note, the OS and DFS of patients with combined low expression of miR-133b and miR-206 (miR-133b-low/miR-206-low) were the shortest (both P<0.001). Furthermore, low miR-133b expression, low miR-206 expression and conjoined expression of miR-133b/miR-206 were all independent prognostic factors for OS and DFS of osteosarcoma patients. Collectively, the aberrant expression of miR-133b and miR-206 may be implicated in tumorigenesis and tumor progression of osteosarcoma. More interestingly, detection of serum miR-133b and miR-206 expression could be further

developed as novel, non-invasive and efficient markers for prognosis in patients with osteosarcomas.
TC 0
ZB 0
Z8 0
ZS 0
Z9 0
SN 1936-2625
UT WOS:000341264400071
PM 25120799
ER

PT J
AU Yoshikawa, Takaki
Tanabe, Kazuaki
Nishikawa, Kazuhiro
Ito, Yuichi
Matsui, Takanori
Kimura, Yutaka
Hirabayashi, Naoki
Mikata, Shoki
Iwahashi, Makoto
Fukushima, Ryoji
Takiguchi, Nobuhiro
Miyashiro, Isao
Morita, Satoshi
Miyashita, Yumi
Tsuburaya, Aakira
Sakamoto, Junichi
TI Induction of a Pathological Complete Response by Four Courses of Neoadjuvant Chemotherapy for Gastric Cancer: Early Results of the Randomized Phase II COMPASS Trial
SO ANNALS OF SURGICAL ONCOLOGY
VL 21
IS 1
BP 213
EP 219
DI 10.1245/s10434-013-3055-x
PD JAN 2014

PY 2014

AB Background. The prognosis for stage 3 gastric cancer is not satisfactory, even with S-1 adjuvant chemotherapy. A randomized phase II trial was conducted to compare two and four courses of neoadjuvant S-1/cisplatin (SC) and paclitaxel/cisplatin (PC) using a two-by-two factorial design for locally advanced gastric cancer. The primary endpoint was overall survival. We clarified the impact of these regimens on the secondary endpoints, including the clinical and pathological responses, chemotherapy-related toxicities, and surgical results.

Methods. Patients received S-1 (80 mg/m(2) for 21 days with 1 week's rest)/cisplatin (60 mg/m(2) at day 8) or paclitaxel/cisplatin (80 and 25 mg/m(2), respectively, on days 1, 8, and 15 with 1 week's rest) as neoadjuvant chemotherapy.

Results. Eighty-three patients were assigned to arm A (two courses of SC, n = 21), arm B (four courses of SC, n = 20), arm C (two courses of PC, n = 21), and arm D (four courses of PC, n = 21). Pathological response rate was 43 % in arm A, 40 % in arm B, 29 % in arm C, and 38 % in arm D. Pathological complete response was only observed in arms B (10 %) and D (10 %). Most bone marrow toxicities, nausea, vomiting, alopecia, and fatigue were slightly higher but acceptable in arms B and D. Grade 3/4 surgical morbidities were not commonly observed in all four arms.

Conclusions. Pathological complete response could be induced by four courses of neoadjuvant chemotherapy without a marked increase of toxicities, regardless of a SC or PC regimen.

TC 3

ZB 0

Z8 0

ZS 0

Z9 3

SN 1068-9265

UT WOS:000332671700034

PM 23838904

ER

PT J

AU Girotti, P.

Rolinger, J.

Kopp, H-G

Koenigsrainer, A.
Ladurner, R.
TI Gastrointestinal Stromal Tumours Bigger than 20 cm: Experience with Imatinib Chemotherapy in Neoadjuvant Intention
SO ZEITSCHRIFT FUR GASTROENTEROLOGIE
VL 52
IS 1
BP 50
EP 54
DI 10.1055/s-0033-1356371
PD JAN 2014
PY 2014
AB The size of the primary tumour is considered the most important risk factor for the development of metastasis or local recurrence in case of gastrointestinal stromal tumour (GIST). Until now no prospective data are available in the literature about the role of neadjuvant therapy with Imatinib. Between 2009 and 2012 seven patients with a giant GIST >20 cm underwent a neadjuvant treatment with Imatinib, a radical operation, followed by an adjuvant therapy. These patients were controlled with regard to peri-and postoperative morbidity and disease-free survival. Two patients were considered not resectable and one patient showed liver metastasis at the time of diagnosis. RECIST responses to the neoadjuvant Imatinib were: 2/7 patients with stable disease, 3/7 partial response, 2/7 partial response with down-staging (resectable disease). Because of the following tumour localisations (6 gastric and 1 rectal), six gastrectomies (one en-bloc with left pancreas) and one Holm operation were performed. The patient with simultaneous liver metastasis developed a tumour progression during the follow-up but the others are still tumour free after 2 years. We detected a significant tumour volume regression due to the neadjuvant chemotherapy in cases of GIST >20 cm (30%). Our series showed good results for a neadjuvant therapy in cases of giant GIST with the achievement of 100% R0 resection without a high morbidity rate (in the literature a tumor size >10 cm and poor localisation is associated to a high risk of R1 - 2 and high morbidity). Peri-and postoperative morbidity are acceptable and the tumour free survival at 2 years is 85%.
TC 0
ZB 0
Z8 0

ZS 0
Z9 0
SN 0044-2771
UT WOS:000330774200019
PM 24420799
ER

PT J
AU Noble, Fergus
Nolan, Luke
Bateman, Adrian C.
Byrne, James P.
Kelly, Jamie J.
Bailey, Ian S.
Sharland, Donna M.
Rees, Charlotte N.
Iveson, Timothy J.
Underwood, Tim J.
Bateman, Andrew R.
TI Refining pathological evaluation of neoadjuvant therapy for adenocarcinoma of the esophagus
SO WORLD JOURNAL OF GASTROENTEROLOGY
VL 19
IS 48
BP 9282
EP 9293
DI 10.3748/wjg.v19.i48.9282
PD DEC 28 2013
PY 2013
AB AIM: To assess tumour regression grade (TRG) and lymph node downstaging to help define patients who benefit from neoadjuvant chemotherapy.
METHODS: Two hundred and eighteen consecutive patients with adenocarcinoma of the esophagus or gas-tro-esophageal junction treated with surgery alone or neoadjuvant chemotherapy and surgery between 2005 and 2011 at a single institution were reviewed. Triplet neoadjuvant chemotherapy consisting of platinum, fluoropyrimidine and anthracycline was considered for operable patients (World Health Organization performance status <= 2) with clinical stage T2-4 N0-1. Response to neoadjuvant chemotherapy (NAC) was assessed using TRG, as described by

Mandard et al. In addition lymph node downstaging was also assessed. Lymph node downstaging was defined by cN1 at diagnosis: assessed radiologically (computed tomography, positron emission tomography, endoscopic ultrasonography), then pathologically recorded as N0 after surgery; ypN0 if NAC given prior to surgery, or pN0 if surgery alone. Patients were followed up for 5 years post surgery. Recurrence was defined radiologically, with or without pathological confirmation. An association was examined between t TRG and lymph node downstaging with disease free survival (DFS) and a comprehensive range of clinicopathological characteristics.
RESULTS: Two hundred and eighteen patients underwent esophageal resection during the study interval with a mean follow up of 3 years (median follow up: 2.552, 95% CI: 2.022-3.081). There was a 1.8% (n = 4) inpatient mortality rate. One hundred and thirty-six (62.4%) patients received NAC, with 74.3% (n = 101) of patients demonstrating some signs of pathological tumour regression (TRG 1-4) and 5.9% (n = 8) having a complete pathological response. Forty four point one percent (n = 60) had downstaging of their nodal disease (cN1 to ypN0), compared to only 15.9% (n = 13) that underwent surgery alone (pre-operatively overstaged: cN1 to pN0), (P < 0.0001). Response to NAC was associated with significantly increased DFS (mean DFS; TRG 1-2: 5.1 years, 95% CI: 4.6-5.6 vs TRG 3-5: 2.8 years, 95% CI: 2.2-3.3, P < 0.0001). Nodal down-staging conferred a significant DFS advantage for those patients with a poor primary tumour response to NAC (median DFS; TRG 3-5 and nodal down-staging: 5.533 years, 95% CI: 3.558-7.531 vs TRG 3-5 and no nodal down-staging: 1.114 years, 95% CI: 0.961-1.267, P < 0.0001).
CONCLUSION: Response to NAC in the primary tumour and in the lymph nodes are both independently associated with improved DFS. (C) 2013 Baishideng Publishing Group Co., Limited. All rights reserved.

TC 3

ZB 1

Z8 0

ZS 0

Z9 3

SN 1007-9327

UT WOS:000329129700014

PM 24409055

ER

PT J
AU Ulla, Marina
Gentile, Ernestina
Yeyati, Ezequiel Levy
Diez, Maria L
Cavadas, Demetrio
Garcia-Monaco, Ricardo D
Ros, Pablo R
TI Pneumo-CT assessing response to neoadjuvant therapy in esophageal cancer: Imaging-pathological correlation.
SO World journal of gastrointestinal oncology
VL 5
IS 12
BP 222
EP 9
DI 10.4251/wjgo.v5.i12.222
PD 2013-Dec-15
PY 2013
AB Pneumo-computed tomography (PnCT) is a technique primarily developed and used to study stenotic lesions of the esophagus, gastroesophageal junction and stomach for pre-surgical planning. It helps to define both upper and lower borders of neoplasms located in the aforementioned areas. It achieves maximum lumen distension with CO2 highlighting thickened areas of the esophageal wall, thus allowing an accurate quantification of their extents. Although there are other alternatives for distension (oral contrast agents, water and effervescent granules), they may be suboptimal. Patients with locally advanced esophageal cancer have a dismal prognosis despite surgical resection. Therefore, neoadjuvant treatment strategies using radiation therapy and chemotherapy were developed to improve survival. Neoadjuvant therapy improves esophageal tumor prognosis in a substantial proportion of patients, and the use of imaging techniques is mandatory to detect their response. PnCT combined with virtual endoscopy and multiplanar reconstruction enhances morphologic details in esophageal cancer, and thus would allow an improved assessment of response to neoadjuvant treatment. Therefore, more information could be provided to assess the efficacy of pre-surgical treatment. We describe the potential use of PnCT to assess the response to neoadjuvant therapy in esophageal cancer with an imaging pathologic correlation.

TC 1
ZB 1
Z8 0
ZS 0
Z9 1
UT MEDLINE:24363830
PM 24363830
ER

PT J
AU Ozcan, Muhammet Fuat
Dizdar, Omer
Dincer, Nazmiye
Balci, Serdar
Guler, Gulnur
Gok, Bahri
Pektas, Gokhan
Seker, Mehmet Metin
Aksoy, Sercan
Arslan, Cagatay
Yalcin, Suayib
Balbay, Mevlana Derya
TI Low ERCC1 expression is associated with prolonged survival in patients with bladder cancer receiving platinum-based neoadjuvant chemotherapy
SO UROLOGIC ONCOLOGY-SEMINARS AND ORIGINAL INVESTIGATIONS
VL 31
IS 8
BP 1709
EP 1715
DI 10.1016/j.urolonc.2012.06.014
PD NOV 2013
PY 2013
AB Purpose: Excision repair cross-complementation group 1 enzyme (ERCC1) plays a key role in the removal of platinum induced DNA adducts and cisplatin resistance. Prognostic role of ERCC1 expression in the neoadjuvant setting in bladder cancer has not been reported before. We evaluated the prognostic role of ERCC1 expression in bladder cancer receiving platinum-based neoadjuvant chemotherapy.
Materials and methods: Thirty-eight patients with muscle invasive

bladder cancer who received neoadjuvant platinum-based chemotherapy were included. Clinical and histopathologic parameters along with immunohistochemical ERCC1 staining were examined and correlated with response rates and survival.
Results: Pathologic complete response rates were similar between patients with low and high ERCC1 expression. Median disease-free survival (DFS) was 9.3 vs. 20.5 months (P = 0.186) and median overall survival (OS) was 9.3 vs. 26.7 months (P = 0.058) in patients with high ERCC1 expression compared with those with low expression, respectively. In multivariate Cox regression analysis: pathological complete response (pCR) after chemotherapy (hazard ratio (HR) 0.1, 95% CI 0.012-0.842, P = 0.034) and high ERCC1 expression (HR 3.7, 95% CI 1.2-11.2, P = 0.019) were significantly associated with DFS. Patient age (>60 vs. <= 60 years) (HR 3.4, 95% CI 1.2-9.4, P = 0.018), the presence of pCR (HR 0.11, 95% CI 0.014-0.981, P = 0.048) and high ERCC expression (HR 6.1, 95 CI 1.9-19.9, P = 0.002) were significantly associated with OS.
Conclusions: Our results showed that high ERCC1 expression was independently associated with shorter disease-free and overall survival in patients with bladder cancer who received neoadjuvant platinum-based chemotherapy. ERCC1 may represent a potential predictive marker for platinum-based treatment in bladder cancer. (C) 2013 Elsevier Inc. All rights reserved.
RI BALCI, Serdar/B-6401-2011
OI BALCI, Serdar/0000-0002-7852-3851
TC 1
ZB 1
Z8 0
ZS 0
Z9 1
SN 1078-1439
UT WOS:000326556900045
PM 22863869
ER

PT J
AU Isobe, Taro
Hashimoto, Kousuke
Kizaki, Junya
Miyagi, Motoshi

Aoyagi, Keishiro
Koufuji, Kikuo
Shirouzu, Kazuo
TI [Neoadjuvant chemotherapy with combined S-1 plus weekly low-dose cisplatin followed by surgical resection for advanced gastric cancer].
SO Gan to kagaku ryoho. Cancer & chemotherapy
VL 40
IS 10
BP 1331
EP 5
PD 2013-Oct
PY 2013
AB We retrospectively examined patients with advanced gastric cancer who underwent gastrectomy following neoadjuvant chemotherapy (NAC) with S-1 plus weekly low-dose cisplatin (CDDP). Between 2007 and 2009, 27 patients with advanced gastric cancer not amenable to curative surgery were enrolled. One course of NAC comprised S-1 (80 mg/m2/day) for 21 consecutive days and CDDP (20 mg/m2) on days 1, 8, and 15; this was followed by a 2-week rest after the end of S-1 administration. Grade 3 side effects were observed in 5 patients: 3 experienced neutropenia and 2 experienced digestive symptoms. The outpatient completion rate was 81.5% (22/27); there was no incidence of renal dysfunction. During pretherapy diagnosis, depth of invasion was classified as T4 in all cases. Postoperative pathologic results showed that the depth of invasion was T3 or lower in 4 patients. In addition, the number of patients with N0 and M0 classification increased and downstaging was observed in 12 patients (44.4%). A comprehensive assessment revealed that a partial response (PR) was observed in 13 patients and stable disease (SD) was observed in 12 patients, resulting in a response rate of 48.1%. The median survival time was 580 days, and the 1-year survival rate was 72%. NAC with S-1 plus weekly low-dose CDDP can also be administered on an outpatient basis, and it is a potential regimen for the treatment of advanced gastric cancer associated with a poor prognosis.
TC 1
ZB 0
Z8 0
ZS 0
Z9 1

SN 0385-0684
UT MEDLINE:24105055
PM 24105055
ER

PT J
AU Kanazawa, Yoshikazu
   Kato, Shunji
   Fujita, Itsuo
   Onodera, Hiroyuki
   Uchida, Eiji
TI Adjuvant Chemotherapy with S-1 Followed by Docetaxel for Gastric Cancer
   and CY1P0 Peritoneal Metastasis after Relatively Curative Surgery
SO JOURNAL OF NIPPON MEDICAL SCHOOL
VL 80
IS 5
BP 378
EP 383
PD OCT 2013
PY 2013
AB Objective: The aim of this study was to assess the feasibility and
   safety of adjuvant chemotherapy with S-1 followed by docetaxel.
   Patients and Method: Twenty-eight patients with advanced gastric cancer
   underwent gastrectomy without preoperative chemotherapy. These patients
   were divided into 3 groups on the basis of cytologic results of
   peritoneal lavage (CY) and the presence of local peritoneal metastatic
   nodules (P): CY1-P0, CY0-P1, and CY1-P1. Oral S-1 (80 mg/m(2)/day) was
   administered for 3 consecutive weeks, followed by intravenous docetaxel
   (35 mg/m(2)) on days 29 and 43 (1 cycle). This cycle was repeated every
   8 weeks. The primary endpoint was the ability to complete 6 cycles of
   S-1 followed by docetaxel. The secondary endpoints were safety,
   progression-free survival, mean survival time (MST), and overall
   survival (OS).
   Results: The subjects were 18 men and 10 women (39 to 78 years old,
   median age, 64 years). The extent of peritoneal metastasis was CY1-P0 in
   8 patients, CY0-P1 in 14 patients, and CY1-P1 in 6 patients. Both
   hematologic and nonhematologic toxicities were generally mild. The
   completion rate of the planned 6 cycles of the protocol was 71.4% (20 of
   28 patients). Median progression-free survival was 22.9 months, and the

2-year survival rate was 78.6%. The overall MST was 34.3 months, and the MST by group was 34.5 for CY1-P0, 34.3 for CY0-P1, and 19.3 months for CY1-P1. The OS in the CY1-P0 and CY0-P1 groups was significantly longer than that in the CY1-P1 group (P<0.05).
Conclusion: Adjuvant chemotherapy with S-1 followed by docetaxel is safe and well tolerated and has the potential to improve OS in patients with a status of CY1P0 following relatively curative resection.
TC 0
ZB 0
Z8 0
ZS 0
Z9 0
SN 1345-4676
UT WOS:000327005500007
PM 24189356
ER

PT J
AU Bryce, Gavin
Crumley, Andrew
MacKay, Colin
Fullarton, Grant
Craig, Carol
Forshaw, Matthew
TI Complete Pathological Response to Neoadjuvant Chemotherapy in Oesophago-Gastric Cancer: incidence and outcomes
SO BRITISH JOURNAL OF SURGERY
VL 100
BP 41
EP 41
SU 8
PD OCT 2013
PY 2013
CT 17th Annual Scientific Meeting of the Association-of-Upper-Gastrointestinal-Surgeons-of-Great-Britain-and-Ireland (AUGIS)
CY SEP 19-20, 2013
CL Newcastle, ENGLAND
SP Assoc Upper Gastrointestinal Surg Great Britain & Ireland

TC 0
ZB 0
Z8 0
ZS 0
Z9 0
SN 0007-1323
UT WOS:000326234800138
ER

PT J
AU Graziosi, Luigina
   Cantarella, Francesco
   Mingrone, Elvira
   Gunnellini, Marco
   Cavazzoni, Emanuel
   Liberati, Marina
   Donini, Annibale
TI Preliminary results of prophylactic HIPEC in patients with locally
   advanced gastric cancer.
SO Annali italiani di chirurgia
VL 84
IS 5
BP 551
EP 6
PD 2013 Sep-Oct
PY 2013
AB BACKGROUND: The prognosis of locally advanced Gastric Cancer following
   surgical therapy alone is poor. Peritoneum represents a preferential
   site of dissemination in such neoplasm. Hyperthermic intraperitoneal
   chemotherapy (HIPEC) has been used in association with cytoreductive
   surgery (CRS) in the treatment of GC peritoneal carcinomatosis (PC). Aim
   of our preliminary experience is reporting our data on prophylactic
   HIPEC (P-HIPEC) in patients with GC at high risk of developing PC.
   METHODS: Eleven patients underwent P-HIPEC at our General and Emergency
   Surgery Department. All the patients were affected of high risk GC:
   serosa invasive tumors (T4), conventional cytology-positive or
   quantitative PCR detection of CEA mRNA on peritoneal lavage. Seven
   subtotal and four total gastrectomies with D2 or D2+ were performed. All
   the anastomoses were made before HIPEC. The procedure was carried out

for 60 minutes with Mytomicin C and Cisplatin in all patients. Post-operative monitoring in Intensive Care Unit least for 24-48 hours. Oral nutrition was started precociously (day 5) also according with bowel movements and stool/gas passage. Follow-up took place in all patients at 1 month from surgery then every 6 months for 2 years and every 12 months for the following years.
RESULTS: In four patients a neoadjuvant treatment was scheduled due to T or N stage at pre-operative evaluation. Gastric resection was guided on tumor location while the choice of performing a D2 or D2 + lymphadenectomy was up to preoperative imaging and intra-operative nodal status. No intra-operative complications were recorded. Median operation time was 398 minutes. In our series we recorded 20 adverse events. Median number for each patient was 1 adverse effect (range 0-2). Eight patients experienced a surgical adverse effect (G2-G3) that did not require any surgical treatment. Only one patient with duodenal stump dehiscence and intra-abdominal sepsis (G4-G5) underwent re-operation and died for severe hemorrhagic pancreatitis. Another patient died for ARDS. Per-operative mortality was 18%. Both patients were older then 70 years old. Median hospital stay was 14 days. Median follow-up was 15.9 months. Median survival was 29.6 months and median DFS was 20 months. Only one patient developed a peritoneal recurrence at 12 months and died for disease progression. Seven patients are still alive and disease free at last follow-up. One patient affected of variable immunodeficiency died at 9 months for pulmonary sepsis without any sign of local recurrence.
CONCLUSIONS: Peritoneal dissemination appears to be a strong determinant in defining GC patients prognosis. Even after curative resection, peritoneal recurrence develops in about 60% of the patients with T3 and T4 tumors, and up to 40% of resected gastric cancer patients die as a direct result of peritoneal dissemination. Clinical trials showed that surgery plus HIPEC was associated with a significant improvement in survival compared to surgery alone in patients affected of GC with resectable PC. At present day there are not studies evaluating the role of P-HIPEC in patients at high risk of developing PC. The rationale of P-HIPEC is based on the concept that positive peritoneal lavage is considered an M1 (stage IV) similarly to macroscopic PC by the 7th TNM classification. Also analogous is the median survival of this 2 groups of patients. Detection of peritoneal micrometastases with cytologic examination has been considered a major method to predict peritoneal recurrences; the sensitivity of this assay is low. Recently, molecular

approaches using real-time reverse-transcriptase polymerase chain reaction (RT-PCR) technique has made possible the increase in the sensitivity. We can conclude, although the preliminary experience, that prophylactic HIPEC in locally advanced gastric cancer is feasible, increasing median survival compared to surgery alone. For sure this procedure need to be performed in the highly specialized centres strongly respecting the eligibility criteria.
TC 2
ZB 1
Z8 0
ZS 0
Z9 2
UT MEDLINE:24140896
PM 24140896
ER

PT J
AU Lorenzen, S.
Thuss-Patience, P.
Al-Batran, S. E.
Lordick, F.
Haller, B.
Schuster, T.
Pauligk, C.
Luley, K.
Bichev, D.
Schumacher, G.
Homann, N.
TI Impact of pathologic complete response on disease-free survival in patients with esophagogastric adenocarcinoma receiving preoperative docetaxel-based chemotherapy
SO ANNALS OF ONCOLOGY
VL 24
IS 8
BP 2068
EP 2073
DI 10.1093/annonc/mdt141
PD AUG 2013
PY 2013

AB The aim of this study was to evaluate the impact of pathologic complete response (pCR) on outcome in patients with gastric or esophagogastric junction (EGJ) adenocarcinoma after neoadjuvant docetaxel/platin/fluoropyrimidine-based chemotherapy.
Patients received at least one cycle of chemotherapy for potentially operable disease. Pretreatment clinicopathologic factors and pCR were investigated. Disease-free survival (DFS), overall survival (OS) and tumor-related death were correlated with pCR.
One hundred twenty patients were included in this analysis. Eighteen patients (15%) achieved a pCR. Tumor localization in the EGJ was identified as the only significant predictor of pCR (P = 0.019). Median follow-up was 41.1 months. Median DFS and OS for all patients were 24.1 and 48.6 months, respectively. Median DFS for patients with a pCR was not reached versus 22.1 months non-pCR patients (hazard ratio, HR 0.38; 3-year DFS: 71.8% and 37.7%, respectively, P = 0.018). While OS was not significantly different, the risk for tumor-related death was significantly lower for pCR patients compared with non-pCR patients (3-year cumulative incidences of 6.4% and 45.4%, respectively, P = 0.009).
A pCR following preoperative docetaxel/platin/fluoropyrimidine indicates favorable outcome in patients with gastric or EGJ adenocarcinoma. Tumor location in the EGJ is associated with a higher pCR rate.
TC 2
ZB 1
Z8 0
ZS 0
Z9 2
SN 0923-7534
UT WOS:000322339300020
PM 23592699
ER

PT J
AU Molina, R.
Lamarca, A.
Martinez-Amores, B.
Gutierrez, A.
Blazquez, A.
Lopez, A.

Granell, J.
Alvarez-Mon, M.
TI Perioperative chemotherapy for resectable gastroesophageal cancer: A single-center experience
SO EJSO
VL 39
IS 8
BP 814
EP 822
DI 10.1016/j.ejso.2013.05.003
PD AUG 2013
PY 2013
AB Backgrounds: Multimodal treatment for locally advanced gastric cancer has been reported to improve disease-free survival when compared to surgery alone. We aimed to clarify the efficacy and safety of perioperative chemotherapy for locally advanced gastric cancer patients treated in daily clinical practice.

Methods: Patients diagnosed with locally advanced gastric cancer were treated with perioperative chemotherapy and surgery. The primary end point was the complete resection (R0) rate. Secondary end points were disease-free survival (DFS), overall survival (OS), toxicity, radiological response rate, pathological response rate and downstaging rate. We also looked for prognostic and predictive factors for DFS, OS, pathological complete response and the R0 rate.

Results: Forty patients were found eligible for this retrospective analysis. At diagnosis, 52.5% of patients were classified as stage II and 47.5% were stage III. Forty percent of patients completed three preoperative cycles and three postoperative cycles. A tolerable toxicity related to chemotherapy was found. Thirty-nine patients underwent surgery: 80% reached a complete resection (R0), down-staging was detected in 57.5% and 17.5% had a pathologically complete response. The median time of disease-free survival was 34.05 months (95%CI 25.6-42.4), and the median time of overall survival was 39.01 months (95%CI 30.8-47.1). We found that the presence of comorbidities were independent predictive factors for the pathologic response, while the chemotherapy schedule and the clinical response could independently predict a complete resection.

Conclusions: Our results support that perioperative chemotherapy for locally advanced gastric dancer can be safely delivered in daily

clinical practice, obtaining an improvement of the pathologic response and the complete resection of gastric cancer. (C) 2013 Elsevier Ltd. All rights reserved.
TC 3
ZB 0
Z8 1
ZS 0
Z9 4
SN 0748-7983
UT WOS:000322100700003
PM 23755989
ER

PT J
AU Koh, Young Wha
Park, Young Soo
Ryu, Min-Hee
Ryoo, Baek-Yeol
Park, Hye Jin
Yook, Jeong Hwan
Kim, Byung Sik
Kang, Yoon-Koo
TI Postoperative Nodal Status and Diffuse-type Histology Are Independent Prognostic Factors in Resectable Advanced Gastric Carcinomas After Preoperative Chemotherapy
SO AMERICAN JOURNAL OF SURGICAL PATHOLOGY
VL 37
IS 7
BP 1022
EP 1029
PD JUL 2013
PY 2013
AB Surgical resection of primary gastric lesions after neoadjuvant or palliative chemotherapy is performed for curative or palliative purpose in locally advanced (LA) or initially metastatic (IM) gastric cancer. We investigated which histomorphologic features were associated with patient prognosis. We examined 143 patients (57 LA and 86 IM) who underwent gastrectomy after chemotherapy between 2000 and 2009. The tumor regression grade (TRG)-determined by examining the residual

neoplastic cells and background stromal changes-was evaluated. Progression-free (PFS) and overall survival (OS) were evaluated according to pretherapeutic and posttherapeutic clinicopathologic factors using univariate and multivariate analyses. Because both the LA and the IM groups showed similar trends of PFS and OS according to TRG, the 2 groups were analyzed together. Patients with TRG1 (no residual primary tumor) showed a superior PFS and OS than the remaining TRGs. We defined pathologic complete regression (pCR) as TRG1 with negative lymph nodes (LN) and the others as non-pCR. Sixteen patients (11.1%) had pCR with better PFS (P = 0.007) and OS (P = 0.006). Initial disease status (LA or IM) remained as independent prognostic factors for PFS (P = 0.021) but not for OS (P = 0.109). The postoperative negative LN status correlated with good outcome and postoperative diffuse-type histology correlated with poor outcome after multivariate analysis. This study showed that pCR, but not partial regression, provides meaningful prognostic information in gastrectomy after chemotherapy. In addition, postoperative LN positivity and diffuse-type histology were independent poor prognostic factors for PFS and OS.
TC 3
ZB 2
Z8 0
ZS 0
Z9 3
SN 0147-5185
UT WOS:000330377200011
PM 23715160
ER

PT J
AU An, Ji Yeong
Kim, Hyoung-Il
Cheong, Jae-Ho
Hyung, Woo Jin
Kim, Choong Bae
Noh, Sung Noon
TI Pathologic and Oncologic Outcomes in Locally Advanced Gastric Cancer with Neoadjuvant Chemotherapy or Chemoradiotherapy
SO YONSEI MEDICAL JOURNAL
VL 54

IS 4
BP 888
EP 894
DI 10.3349/ymj.2013.54.4.888
PD JUL 1 2013
PY 2013
AB Purpose: Although neoadjuvant therapy has been accepted as a treatment option in locally-advanced gastric cancer, its prognostic value has been difficult to evaluate. Materials and Methods: Seventy-four gastric cancer patients who underwent gastrectomy after neoadjuvant treatment were divided into two groups according to the pathologic response: favorable (ypT0) and others (ypT1-4). The clinicopathologic characteristics, predictive factors for pathologic response, and oncologic outcome were evaluated. Results: Eleven patients (14.8%) demonstrated ypT0 and the remaining 63 patients (85.2%) were ypT1-4. Chemoradiotherapy (CCRTx) rather than chemotherapy (CTx) was the only predictive factor for a favorable pathologic response. Chemotherapeutic factors and tumor marker levels did not predict pathologic response. The 1-, 2-, and 3-year disease-free survivals were 83.4%, 70%, and 52.2%. The 1-, 3-, 5-year overall survivals were 88.5%, 67.5%, and 51.2%, respectively. Although a complete pathologic response (ypT0N0M0) was achieved in 7 patients, 28.6% of them demonstrated recurrence of the tumor within 6 months after curative surgery. Conclusion: CCRTx rather than CTx appears to be more effective for achieving good pathologic response. Although favorable pathologic response has been achieved after neoadjuvant treatment, the survival benefit remains controversial.
TC 3
ZB 1
Z8 1
ZS 0
Z9 4
SN 0513-5796
UT WOS:000321094700012
PM 23709422
ER

PT J
AU Nilsson, Per J.
van Etten, Boudewijn

Hospers, Geke A. P.
Pahlman, Lars
van de Velde, Cornelis J. H.
Beets-Tan, Regina G. H.
Blomqvist, Lennart
Beukema, Jannet C.
Kapiteijn, Ellen
Marijnen, Corrie A. M.
Nagtegaal, Iris D.
Wiggers, Theo
Glimelius, Bengt
TI Short-course radiotherapy followed by neo-adjuvant chemotherapy in locally advanced rectal cancer - the RAPIDO trial
SO BMC CANCER
VL 13
AR 279
DI 10.1186/1471-2407-13-279
PD JUN 7 2013
PY 2013
AB Background: Current standard for most of the locally advanced rectal cancers is preoperative chemoradiotherapy, and, variably per institution, postoperative adjuvant chemotherapy. Short-course preoperative radiation with delayed surgery has been shown to induce tumour down-staging in both randomized and observational studies. The concept of neo-adjuvant chemotherapy has been proven successful in gastric cancer, hepatic metastases from colorectal cancer and is currently tested in primary colon cancer.
Methods and design: Patients with rectal cancer with high risk features for local or systemic failure on magnetic resonance imaging are randomized to either a standard arm or an experimental arm. The standard arm consists of chemoradiation (1.8 Gy x 25 or 2 Gy x 25 with capecitabine) preoperatively, followed by selective postoperative adjuvant chemotherapy. Postoperative chemotherapy is optional and may be omitted by participating institutions. The experimental arm includes short-course radiotherapy (5 Gy x 5) followed by full-dose chemotherapy (capecitabine and oxaliplatin) in 6 cycles before surgery. In the experimental arm, no postoperative chemotherapy is prescribed. Surgery is performed according to TME principles in both study arms. The hypothesis is that short-course radiotherapy with neo-adjuvant

chemotherapy increases disease-free and overall survival without compromising local control. Primary end-point is disease-free survival at 3 years. Secondary endpoints include overall survival, local control, toxicity profile, and treatment completion rate, rate of pathological complete response and microscopically radical resection, and quality of life.
Discussion: Following the advances in rectal cancer management, increased focus on survival rather than only on local control is now justified. In an experimental arm, short-course radiotherapy is combined with full-dose chemotherapy preoperatively, an alternative that offers advantages compared to concomitant chemoradiotherapy with or without postoperative chemotherapy. In a multi-centre setting this regimen is compared to current standard with the aim of improving survival for patients with locally advanced rectal cancer.

RI Nagtegaal, Iris/A-2448-2014

OI Nagtegaal, Iris/0000-0003-0887-4127

TC 6

ZB 1

Z8 0

ZS 0

Z9 6

SN 1471-2407

UT WOS:000320138800001

PM 23742033

ER

PT J

AU Rostom, Y
Zaghloul, H
Khedr, G
El-Shazly, W
Abd-Allah, D

TI Docetaxel-based preoperative chemoradiation in localized gastric cancer: impact of pathological complete response on patient outcome.

SO Journal of gastrointestinal cancer

VL 44

IS 2

BP 162

EP 9

DI 10.1007/s12029-012-9449-3
PD 2013-Jun
PY 2013
AB PURPOSE: This study was conducted to evaluate the feasibility, efficacy, and toxicities of docetaxel-based induction chemotherapy and chemoradiotherapy in patients with localized gastric or gastroesophageal adenocarcinoma.

METHODS: Patients with localized, operable gastric or gastroesophageal adenocarcinoma received two cycles of induction chemotherapy of fluorouracil, docetaxel, and cisplatin (TPF) followed by 45 Gy of radiation and concurrent fluorouracil plus docetaxel then surgery for nonmetastatic patients.

RESULTS: Forty-one patients were included. Pretreatment T3 was encountered in 56 % of patients while 61 % had N1 disease. A pathologic complete response (CR) was noted in 24 % of patients. Pathologic response was significantly associated with baseline T stage (P < 0.001) and N stage (P = 0.002). The 3-year overall survival (OS) and disease-free survival were 47.3 and 42.1 %, respectively. OS was significantly correlated with R0 resection (P = 0.027), pathological response (P = 0.01), dissected pathologically positive lymph node (P = 0.037), and postsurgery (T) stage (P = 0.02). Toxicities were manageable and there were no treatment-related deaths.

CONCLUSION: Docetaxel-based chemoradiotherapy in localized gastric adenocarcinoma patients resulted in 24 % path CR and was not associated with a higher percentage of postoperative complications. A well-designed randomized controlled trial is mandatory to further endorse this evolving approach.
TC 0
ZB 0
Z8 0
ZS 0
Z9 0
UT MEDLINE:23104208
PM 23104208
ER

PT J
AU Zhao, Qun
Li, Yong

Tian, Yuan
Chen, Yan-Ning
Tan, Bi-Bo
Zhao, Xue-Feng
Jiao, Zhi-Kai
Zhang, Zhi-Dong
Chang, Sheng-Li
TI Histological Complete Response after Neoadjuvant XELOX in Advanced Gastric Carcinoma
SO HEPATO-GASTROENTEROLOGY
VL 60
IS 123
BP 638
EP 640
DI 10.5754/hge121131
PD MAY 2013
PY 2013
AB We report on a case of a 65-year-old Chinese male with locally advanced gastric adeno carcinoma achieving pathological complete response after neoadjuvant chemotherapy with capecitabine and oxaliplatin (XELOX) regimen. He underwent esophagogastroduodenoscopy, which revealed a 6cmx5cm gastric ulcer. Biopsy of gastric ulcer revealed adenocarcinoma. Further workups with abdominal enhancement computed tomography (CT) staged his cancer as T4N2M0. He received 2 cycles of neoadjuvant chemotherapy with XELOX without severe toxicity. Afterwards, he underwent curative surgery consisting of total gastrectomy with extended D2 lymph node dissections and a Roux-en-Y esophagojejunostomy. On microscopic examination, no tumor cells were detected in the ulcer scar of the resected stomach and in the regional lymph nodes. The benefit of XELOX regimen as neoadjuvant chemotherapy in gastric cancer is worth further investigation.
TC 0
ZB 0
Z8 0
ZS 0
Z9 0
SN 0172-6390
UT WOS:000320740500049
PM 23340232

ER

PT J
AU Thuss-Patience, P.
   Kutup, A.
   Eble, M.
TI Adenocarcinoma of the stomach and gastroesophageal junction. Neoadjuvant and adjuvant therapy
SO ONKOLOGE
VL 19
IS 5
BP 371
EP +
DI 10.1007/s00761-012-2417-5
PD MAY 2013
PY 2013
AB There is worldwide consensus that curative treatment of gastroesophageal adenocarcinoma can be optimized by a multidisciplinary approach.
   Literature research and analysis of clinical trials.
   In the USA adjuvant chemoradiotherapy is a standard of care for gastric cancer. Asian trials could show an improvement in survival by adjuvant chemotherapy. In Europe the recommendations are based on the British MAGIG trial and the French FNCLCC study. In these trials patients with adenocarcinoma of the stomach and gastroesophageal junction (GEJ) were treated with platinum 5-FU-based perioperative chemotherapy and overall survival could be significantly improved. For the treatment of adenocarcinoma of the GEJ preoperative radiochemotherapy is an equivalent standard. A significant improvement of survival could also be shown for this treatment.
   The German S3 guidelines recommend perioperative chemotherapy for gastric tumors of at least stage uT3 and for GEJ adenocarcinoma either perioperative chemotherapy or preoperative radiochemotherapy.
TC 0
ZB 0
Z8 0
ZS 0
Z9 0
SN 0947-8965
UT WOS:000318513100005

ER

PT J

AU Tsuburaya, Akira
Nagata, Naoki
Cho, Haruhiko
Hirabayashi, Naoki
Kobayashi, Michiya
Kojima, Hiroshi
Munakata, Yasuhiro
Fukushima, Ryoji
Kameda, Yoichi
Shimoda, Tadakazu
Oba, Koji
Sakamoto, Junichi

TI Phase II trial of paclitaxel and cisplatin as neoadjuvant chemotherapy for locally advanced gastric cancer

SO CANCER CHEMOTHERAPY AND PHARMACOLOGY
VL 71
IS 5
BP 1309
EP 1314
DI 10.1007/s00280-013-2130-0
PD MAY 2013
PY 2013

AB Paclitaxel-cisplatin (TC) combination is effective and well tolerated in patients with unresectable gastric cancer. We investigated the efficacy and safety of TC for locally advanced gastric cancers in a neoadjuvant setting.

Patients received 2-4 courses of paclitaxel (80 mg/m(2)) and cisplatin (25 mg/m(2)) on days 1, 8, and 15 in a 4-weekly schedule, followed by radical gastrectomy. Primary endpoint was the pathological response rate: percentage of tumors in which one-third or more parts were affected.

All 52 patients enrolled were eligible. Thirty-six (69.7 %) patients completed two or more courses of chemotherapy. Forty-three patients (82.7 %) underwent surgery, 33 (63.5 %) had R0 resection, and there was no treatment-related death. The pathological response was 34.6 % (95 % CI 22.0-49.1) for all registered patients; the null hypothesis of tumor

response a parts per thousand currency sign10 % was rejected (p <
0.0001). The 3-year overall survival was 41.5 % (95 % CI 27.4-55.0).
The neoadjuvant chemotherapy with TC was safe and effective for patients
with locally advanced gastric cancer, and further study is needed to
confirm the effectiveness of this regimen.
TC 12
ZB 3
Z8 1
ZS 0
Z9 13
SN 0344-5704
UT WOS:000318287600021
PM 23463482
ER

PT J
AU Chen, Liqi
Li, Guoli
Li, Jieshou
Fan, Chaogang
Xu, Jian
Wu, Bo
Liu, Kun
Zhang, Caihua
TI Correlation between expressions of ERCC1/TS mRNA and effects of gastric
cancer to chemotherapy in the short term
SO CANCER CHEMOTHERAPY AND PHARMACOLOGY
VL 71
IS 4
BP 921
EP 928
DI 10.1007/s00280-013-2083-3
PD APR 2013
PY 2013
AB To study the correlation between expression levels of ERCC1/TS mRNA and
the susceptibility of preoperative chemotherapy for patients with
gastric cancer.
A total of forty cases with advanced gastric cancer of T3-4N1-2M0 were
treated with preoperative chemotherapy according to FLEEOX regimen based

on endarterial-intravenous coadministration. Sufficient, fresh gastric tissue specimens were obtained with the help of gastroscope, and the expression levels of ERCC1/TS mRNA were detected by qRT-PCR before chemotherapy. The chemotherapeutic response was evaluated with Choi Criteria after chemotherapy, and pathologic remission extent was observed after surgery. The correlation between the expression levels of ERCC1/TS mRNA before chemotherapy and the chemotherapeutic effect based on imageology and pathology was analyzed.
The response rate of Chemotherapy in this cohort was 80.0 % based on imageology and 51.43 % based on pathology. The expression levels of ERCC1/TS mRNA were significantly associated with imageology remission extent (P = 0.033, P = 0.025) and pathologic remission extent (P = 0.044, P = 0.016), respectively. The chemotherapeutic effect on patients with low-expression levels of ERCC1/TS mRNA was better.
From the perspective of pathology and imageology evaluating the preoperative chemotherapeutic response for patients with gastric cancer, ERCC1 and TS were used as the molecular predictors and provided prognostic information in this study.
TC 3
ZB 2
Z8 0
ZS 0
Z9 3
SN 0344-5704
UT WOS:000316745500010
PM 23355039
ER

PT J
AU Wood, Matthew D.
Zaki, Bassem I.
Gordon, Stuart R.
Sutton, John E., Jr.
Lisovsky, Mikhail
Gui, Jiang
Bubis, Jeffrey A.
Dragnev, Konstantin H.
Rigas, James R.
TI Trimodality Therapy for Stage II-III Carcinoma of the Esophagus: A

Dose-Ranging Study of Concurrent Capecitabine, Docetaxel, and Thoracic Radiotherapy

SO JOURNAL OF THORACIC ONCOLOGY
VL 8
IS 4
BP 487
EP 494
DI 10.1097/JTO.0b013e3182829bf3
PD APR 2013
PY 2013

AB Purpose: This dose-escalation study was performed to determine the recommended phase II dose of oral capecitabine to be delivered concurrently with thoracic radiation therapy and weekly docetaxel in patients with locally advanced esophageal carcinoma.

Methods: Patients with operable stage II or III esophageal carcinoma were staged by endoscopic ultrasonography and computed tomography. Two cycles of docetaxel (80 mg/m(2)) and carboplatin (target area under the concentration-time curve: 6 mg/ml x min) were delivered over 6 weeks. This was followed by concurrent weekly docetaxel (15 mg/m(2)), thoracic radiotherapy (50.4 Gy in 28 fractions), and increasing doses of capecitabine (500-3500 mg) given before each fraction of radiotherapy. After restaging, responding patients continued to esophagectomy within 4 to 8 weeks of completing chemoradiotherapy.

Results: Forty-four patients were enrolled, and 40 were assessable for the dose-ranging component of concurrent chemoradiotherapy. Endoscopic ultrasonography stages at enrollment were T3N1 (29 patients), T3N0 (4 patients), T2N1 (6 patients), and T4N0 (one patient). The maximum tolerated dose of capecitabine was 3500 mg. Thirty-six patients had surgery; 83% had R0 resection, and 17% had pathological complete response. Median overall survival was 23.5 months, with 34 and 27% alive at 3 and 5 years.

Conclusion: The recommended phase II dose of capecitabine is 3500 mg when given concurrently with 50.4 Gy of thoracic radiotherapy in 28 fractions and weekly docetaxel. This trimodality therapy for operable locally advanced esophageal carcinoma was very well tolerated and remarkably active. This regimen holds promise for the treatment of esophageal carcinoma and warrants further investigation.

TC 0
ZB 0

Z8 1
ZS 0
Z9 1
SN 1556-0864
UT WOS:000316206600020
PM 23370365
ER

PT J
AU Wang, Ziyuan
Liang, Xin
Cheng, Zhuoan
Xu, Yufang
Yin, Peihao
Zhu, Huirong
Li, Qi
Qian, Xuhong
Liu, Jianwen
TI Induction of apoptosis and suppression of ERCC1 expression by the potent amonafide analogue 8-c in human colorectal carcinoma cells
SO ANTI-CANCER DRUGS
VL 24
IS 4
BP 355
EP 365
DI 10.1097/CAD.0b013e32835df8b5
PD APR 2013
PY 2013
AB Previous studies have reported that 8-c [6-(2-(2-(dimethylamino) ethylamino) ethylamino)-2-octyl-1H-benzo[de] isoquinoline-1,3(2H)-dione], a novel amonafide analogue, was generated as a new anticancer candidate. However, little is known about its activity in chemoresistant cells. In this study, the antitumor effects of 8-c on the multi-drug-resistant human colorectal carcinoma cancer cell lines HCT-116/L-OHP and HCT-8/VCR have been investigated for the first time. 8-c showed similar concentration-dependent inhibitory activities against multi-drug-resistant cells and corresponding parental cell lines by the MTT assay after 48 h of treatment. 8-c treatment resulted in the induction of apoptosis, as evidenced by fluorescent

staining analysis, comet assay data, and the increase in the number of apoptotic cells as detected by flow cytometry. Western blot, qPCR, and siRNA techniques were used to elucidate the molecular mechanism. Our study suggested that the apoptotic effect of 8-c can be attributed to the upregulation of p53, caspase-3, and cleaved poly(ADP-ribose) polymerase (PARP) and the downregulation of Bcl-2. Furthermore, ERCC1 is essential for nucleotide excision repair. ERCC1 expression was correlated with sensitivity to chemotherapy in various colon cancer cell lines. It is intriguing that decreases in ERCC1 protein and mRNA levels were also observed in the HCT-116/L-OHP and HCT-8/VCR cells after exposure to 8-c. Further transient transfection of multi-drug-resistant cells with ERCC1 siRNA enhanced 8-c-induced cytotoxicity. In contrast, epidermal growth factor-induced increase in ERCC1 protein levels was shown to rescue cell viability upon 8-c treatment. These findings suggest that 8-c has a strong potential to be developed as a new antitumor agent for the treatment of multi-drug-resistant colon cancer cells, and is worthy of further studies. Anti-Cancer Drugs 24: 355-365 (C) 2013 Wolters Kluwer Health vertical bar Lippincott Williams & Wilkins. Anti-Cancer Drugs 2013, 24:355-365
TC 1
ZB 1
Z8 0
ZS 0
Z9 1
SN 0959-4973
UT WOS:000315742800004
PM 23426174
ER

PT J
AU Alcindor, T.
Ferri, L. E.
Marcus, V.
Andalib, A.
Hickeson, M.
Artho, G.
Chasen, M.
Thirlwell, M. P.
Ades, S.

TI Perioperative DCF chemotherapy protocol for patients with gastroesophageal adenocarcinoma: correlation between response to treatment and outcome

SO MEDICAL ONCOLOGY
VL 30
IS 1
AR 377
DI 10.1007/s12032-012-0377-7
PD MAR 2013
PY 2013

AB To determine whether metabolic or pathological response to preoperative chemotherapy can predict the relapse-free survival of gastroesophageal adenocarcinoma patients treated on a perioperative chemotherapy protocol. The prospectively collected data of a recently reported phase II trial of perioperative DCF chemotherapy (docetaxel/cisplatin/5-fluorouracil) were analyzed. Median relapse-free survival (RFS) was compared with the Wilcoxon rank-sum test between responders and non-responders according to defined metabolic (reduction in maximum standard uptake value of at least 35 %) and pathological (greater than 50 % tumor regression or ypN(0) status) criteria. A double-sided p value equal or inferior to 0.05 was considered significant. Patients were followed for a median of 807 days (95 % CI: 607-896). RFS was 576 days in metabolic non-responders versus not reached in metabolic responders (p 0.009) and 562 days in ypN+ versus not reached in ypN(0) patients (p 0.045). No statistically significant RFS difference was seen between low and high pathologic responders classified according to tumor regression criteria, although a trend was observed in favor of high pathologic responders. Simple metabolic and pathologic criteria used for the assessment of response to the preoperative part of perioperative chemotherapy can help to estimate the outcome of gastroesophageal adenocarcinoma patients.

TC 1
ZB 0
Z8 0
ZS 0
Z9 1
SN 1357-0560
UT WOS:000316800800056
PM 23275118

ER

PT J

AU Lee, Michael S.
Mamon, Harvey J.
Hong, Theodore S.
Choi, Noah C.
Fidias, Panagiotis M.
Kwak, Eunice L.
Meyerhardt, Jeffrey A.
Ryan, David P.
Bueno, Raphael
Donahue, Dean M.
Jaklitsch, Michael T.
Lanuti, Michael
Rattner, David W.
Fuchs, Charles S.
Enzinger, Peter C.

TI Preoperative Cetuximab, Irinotecan, Cisplatin, and Radiation Therapy for Patients With Locally Advanced Esophageal Cancer

SO ONCOLOGIST
VL 18
IS 3
BP 281
EP 287
DI 10.1634/theoncologist.2012-0208
PD MAR 2013
PY 2013

AB Purpose. To determine the efficacy and toxicity of weekly neoadjuvant cetuximab combined with irinotecan, cisplatin, and radiation therapy in patients with locally advanced esophageal or gastroesophageal junction cancer.
Methods and Materials. Patients with stage IIA-IVA esophageal or gastroesophageal junction cancer were enrolled in a Simon's two-stage phase II study. Patients received weekly cetuximab on weeks 0-8 and irinotecan and cisplatin on weeks 1, 2, 4, and 5, with concurrent radiotherapy (50.4 Gy on weeks 1-6), followed by surgical resection.
Results. In the first stage, 17 patients were enrolled, 16 of whom had adenocarcinoma. Because of a low pathologic complete response (pCR) rate

in this cohort, the trial was discontinued for patients with
adenocarcinoma but squamous cell carcinoma patients continued to be
enrolled; two additional patients were enrolled before the study was
closed as a result of poor accrual. Of the 19 patients enrolled, 18
patients proceeded to surgery, and 16 patients underwent an R0
resection. Three patients (16%) had a pCR. The median progression-free
survival interval was 10 months, and the median overall survival
duration was 31 months. Severe neutropenia occurred in 47% of patients,
and severe diarrhea occurred in 47% of patients. One patient died
preoperatively from sepsis, and one patient died prior to hospital
discharge following surgical resection.
Conclusions. This schedule of cetuximab in combination with irinotecan,
cisplatin, and radiation therapy was toxic and did not achieve a
sufficient pCR rate in patients with localized esophageal adenocarcinoma
to undergo further evaluation. The Oncologist 2013;18:281-287
TC 8
ZB 6
Z8 4
ZS 0
Z9 12
SN 1083-7159
UT WOS:000316783300009
PM 23429739
ER

PT J
AU Blum, Mariela A.
Takashi, Taketa
Suzuki, Akihiro
Ajani, Jaffer A.
TI Management of localized gastric cancer
SO JOURNAL OF SURGICAL ONCOLOGY
VL 107
IS 3
BP 265
EP 270
DI 10.1002/jso.23183
PD MAR 2013
PY 2013

AB Gastric cancer continues to be a fatal disease with majority of cases presenting in late stages. For patients with advanced disease, we can only recommend palliative therapy. For localized gastric cancer, the approaches vary in various regions of the world. In western countries, preoperative chemotherapy or adjuvant chemo-radiation is preferred; however in Asia, surgery followed by adjuvant chemotherapy is favored. The extent of the lymph node dissection also varies by region. D2 gastrectomy is difficult to implement in most western countries while it is standardized and is a routine in Asia. We recommend multidisciplinary evaluation of each patient before starting any therapy. The prognosis after resection depends of the pathologic stage. Long-term survivors are often <50% in the West and <70% in many Asian countries. Regional and systemic recurrences are common. Improved systemic treatments are needed. Detailed studies of molecular biology might uncover novel therapeutic targets and prognostic subgroups. J. Surg. Oncol. 2013;107:265270. (c) 2013 Wiley Periodicals, Inc.
TC 13
ZB 11
Z8 2
ZS 0
Z9 15
SN 0022-4790
UT WOS:000314869900007
PM 23303654
ER

PT J
AU Ott, Katja
Blank, Susanne
Becker, Karen
Langer, Rupert
Weichert, Wilko
Roth, Wilfried
Sisic, Leila
Stange, Annika
Jaeger, Dirk
Buechler, Markus
Siewert, Joerg-Ruediger
Lordick, Florian

TI Factors predicting prognosis and recurrence in patients with esophago-gastric adenocarcinoma and histopathological response with less than 10 % residual tumor

SO LANGENBECKS ARCHIVES OF SURGERY
VL 398
IS 2
BP 239
EP 249
DI 10.1007/s00423-012-1039-0
PD FEB 2013
PY 2013

AB Neoadjuvant treatment is an accepted standard approach for treating locally advanced esophago-gastric adenocarcinomas. Despite a response of the primary tumor, a significant percentage dies from tumor recurrence. The aim of this retrospective exploratory study from two academic centers was to identify predictors of survival and recurrence in histopathologically responding patients.

Two hundred thirty one patients with adenocarcinomas (esophagus: n = 185, stomach: n = 46, cT3/4, cN0/+, cM0) treated with preoperative chemotherapy (n = 212) or chemoradiotherapy (n = 19) followed by resection achieved a histopathological response (regression 1a: no residual tumor (n = 58), and regression 1b < 10 % residual tumor (n = 173)).

The estimated median overall survival was 92.4 months (5-year survival, 56.6 %) for all patients. For patients with regression 1a, median survival is not reached (5-year survival, 71.6 %) compared to patients with regression 1b with 75.3 months median (5-year survival, 52.2 %) (p = 0.031). Patients with a regression 1a had lymph node metastases in 19.0 versus 33.7 % in regression 1b. The ypT-category (p < 0.001), the M-category (p = 0.005), and the type of treatment (p = 0.04) were found to be independent prognostic factors in R0-resected patients. The recurrence rate was 31.7 % (n = 66) (local, 39.4 %; peritoneal carcinomatosis, 25.7 %; distant metastases, 50 %). Recurrence was predicted by female gender (p = 0.013), ypT-category (p = 0.007), and M-category (p = 0.003) in multivariate analysis.

Response of the primary tumor does not guarantee recurrence-free long-term survival, but histopathological complete responders have better prognosis compared to partial responders. Established prognostic factors strongly influence the outcome, which could, in the future, be

used for stratification of adjuvant treatment approaches. Increasing the rate of histopathological complete responders is a valid endpoint for future clinical trials investigating new drugs.

TC 4

ZB 2

Z8 0

ZS 0

Z9 4

SN 1435-2443

UT WOS:000314900100007

PM 23269519

ER

PT J

AU Wang, Yan
Liu, Tian-shu
Zhuang, Rong-yuan
Cui, Yue-hong
Wang, Zhi-ming
Yu, Yi-yi
Hou, Jun
Sun, Yi-hong
Shen, Kun-tang
Shen, Zhen-bin

TI [Efficacy of neoadjuvant chemotherpy in patients with locally advanced gastric cancer].

SO Zhonghua wei chang wai ke za zhi = Chinese journal of gastrointestinal surgery

VL 16

IS 2

BP 166

EP 9

PD 2013-Feb

PY 2013

AB OBJECTIVE: To evaluate the efficacy and safety of neoadjuvant chemotherapy in patients with locally advanced gastric cancer, and to analyze the relevant factors of recurrent death of gastric cancer after adjuvant chemotherapy.

METHODS: Clinical data of 49 patients who underwent neoadjuvant

chemotherapy for locally advanced gastric cancer between July 2007 and June 2011 were reviewed. Preoperative staging was determined by endoscopic ultrasonography and abdominal computer tomography (CT) or magnetic resonance imaging (MRI). Chemotherapy was administered for regimen of two or three drugs. Prognostic factors were analyzed by univariate and multivariate analysis with Cox proportional hazard model.
RESULTS: The response rate was 33.3% (16/48) and disease control rate was 93.8% (45/48). Forty-four (89.8%, 44/49) patients received curative resection after neoadjuvant chemotherapy, among whom 90.9% (40/44) underwent D2 lymphadenctomy. Thirty-two cases had pathological response and 2 patients had pathological complete response. The average hospital stay was 11.6 days and 2 patients had longer hospitalization because of postoperative pancreatic complications. The toxicities were most in grade 1-2. All the patients were followed up postoperatively and the median follow-up was 21.6 months. Median progression-free survival was 29.6 (95%CI:24.0-35.2) months and median overall survival was 34.6 months (95%CI:29.8-39.4). Imaging response (P=0.038, RR=0.168, 95%CI:0.031-0.904) and pathological response (P=0.007, RR=0.203, 95%CI:0.064-0.642) were identified as independent prognostic factors with COX multivariate analysis.
CONCLUSIONS: Neoadjuvant chemotherapy has quite high disease control rate and R0 resecting rate for patients with locally advanced gastric cancer. Imaging response and pathological response are most important prognostic factors in those patients.
TC 1
ZB 1
Z8 0
ZS 0
Z9 1
SN 1671-0274
UT MEDLINE:23446480
PM 23446480
ER

PT J
AU Pepek, Joseph M.
Chino, Junzo P.
Willett, Christopher G.
Palta, Manisha

Blazer, Dan G., III
Tyler, Douglas S.
Uronis, Hope E.
Czito, Brian G.

TI Preoperative chemoradiotherapy for locally advanced gastric cancer

SO RADIATION ONCOLOGY

VL 8

AR 6

DI 10.1186/1748-717X-8-6

PD JAN 4 2013

PY 2013

AB Background: To examine toxicity and outcomes for patients treated with preoperative chemoradiotherapy (CRT) for gastric cancer.
Methods: Patients with gastroesophageal (GE) junction (Siewert type II and III) or gastric adenocarcinoma who underwent neoadjuvant CRT followed by planned surgical resection at Duke University between 1987 and 2009 were reviewed. Overall survival (OS), local control (LC) and disease-free survival (DFS) were estimated using the Kaplan-Meier method. Toxicity was graded according to the Common Toxicity Criteria for Adverse Events version 4.0.
Results: Forty-eight patients were included. Most (73%) had proximal (GE junction, cardia and fundus) tumors. Median radiation therapy dose was 45 Gy. All patients received concurrent chemotherapy. Thirty-six patients (75%) underwent surgery. Pathologic complete response and R0 resection rates were 19% and 86%, respectively. Thirty-day surgical mortality was 6%. At 42 months median follow-up, 3-year actuarial OS was 40%. For patients undergoing surgery, 3-year OS, LC and DFS were 50%, 73% and 41%, respectively.
Conclusions: Preoperative CRT for gastric cancer is well tolerated with acceptable rates of perioperative morbidity and mortality. In this patient cohort with primarily advanced disease, OS, LC and DFS rates in resected patients are comparable to similarly staged, adjuvantly treated patients in randomized trials. Further study comparing neoadjuvant CRT to standard treatment approaches for gastric cancer is indicated.

TC 2

ZB 0

Z8 0

ZS 0

Z9 2

SN 1748-717X
UT WOS:000313985400001
PM 23286735
ER

PT P
AU ALAVATTAM S
AMLER L C
BENYUNES M C
CLARK E L
DE TOLEDO P C H
KWONG G Z W
MITCHELL L
RATNAYAKE J
ROSS G A
WALKER R
DE TOLEDO PELIZON C H
KWONG GLOVER Z W
WOEKEO R A
TI Extending progression free survival in a human epidermal growth factor receptor 2-positive breast cancer patient population by at least six months comprises administering pertuzumab, trastuzumab and chemotherapy comprising docetaxel
PN US2013095172-A1; WO2013055874-A2; CA2788253-A1; WO2013055874-A3; AU2012322797-A1; KR2014075725-A; AU2012322797-A8; MX2014004021-A1; EP2766040-A2; JP2014530235-W; PH12014500733-A1; VN39918-A
AE GENENTECH INC; HOFFMANN LA ROCHE&CO AG F; ROCHE PROD LTD; ALAVATTAM S; AMLER L C; BENYUNES M C; CLARK E L; DE TOLEDO PELIZON C H; KWONG GLOVER Z W; MITCHELL L; RATNAYAKE J; ROSS G A; WALKER R; DE TOLEDO P C H; KWONG G Z W; HOFFMANN LA ROCHE & CO AG F
AB
NOVELTY - Extending progression free survival in a human epidermal growth factor receptor 2 (HER2)-positive breast cancer patient population by at least 6 months comprises administering pertuzumab, trastuzumab and chemotherapy to the patients in the population.
USE - The method is useful for: extending progression free survival in a HER2-positive breast cancer patient population by at least 6 months, where the breast cancer is metastatic or locally recurrent, unresectable breast cancer, or de novo stage IV disease, or metastatic or locally

advanced; and treating HER2-positive cancer, gastric cancer, HER2-positive non-resectable or metastatic adenocarcinoma of the stomach or gastroesophageal junction, and low HER3 ovarian, primary peritoneal, or Fallopian tube cancer which is epithelial ovarian cancer that is platinum-resistant or platinum-refractory (all claimed).
ADVANTAGE - The method: results in an objective response rate of at least 80% in the patients in the population; improves progression free survival in a human patient with HER2-positive non-resectable or metastatic adenocarcinoma of the stomach or gastroesophageal junction; does not increase cardiac toxicity in a HER2-positive cancer patient population; and reduces the risk of death by at least 34% relative to a patient treated with trastuzumab and the chemotherapy. Test details are described but no results given.
DETAILED DESCRIPTION - INDEPENDENT CLAIMS are also included for:(1) combining two HER2 antibodies to treat HER2-positive cancer without increasing cardiac toxicity in a HER2-positive cancer patient population, comprising administering pertuzumab, trastuzumab, and chemotherapy to the patients in the population;(2) an article of manufacture comprising a vial with pertuzumab in it and a package insert that provides the safety data as in table 3 or 4 of the specification;(3) making the article of manufacture comprising packaging together the vial with pertuzumab and package insert;(4) ensuring safe and effective use of pertuzumab comprising packaging together the vial with pertuzumab and the package insert;(5) treating (A1) early-stage HER2-positive breast cancer comprising administering pertuzumab, trastuzumab and chemotherapy to a patient with the breast cancer, where the chemotherapy comprises anthracycline-based chemotherapy, or carboplatin-based chemotherapy;(6) an intravenous bag containing a stable mixture of pertuzumab and trastuzumab suitable for administration to a cancer patient;(7) treating (A2) HER2-positive gastric cancer in a human subject comprising administering pertuzumab, trastuzumab, and chemotherapy to the subject with HER2-positive gastric cancer;(8) treating (A3) HER2-positive breast cancer in a patient comprising administering pertuzumab, trastuzumab, and aromatase inhibitor to the patient;(9) treating (A4) a cancer patient comprising administering an initial dose of 840 mg of pertuzumab followed every 3 weeks then a dose of 420 mg of pertuzumab, and re-administering an 840 mg dose of pertuzumab to the patient if the time between two sequential 420 mg doses is at least 6 weeks;(10) treating (A5) HER2-positive metastatic or

locally recurrent breast cancer in a patient comprising administering pertuzumab, trastuzumab and taxoid to the patient, where the patient has been previously treated with a trastuzumab and/or lapatinib as adjuvant or neoadjuvant therapy; and(11) treating (A6) low HER3 ovarian, primary peritoneal, or Fallopian tube cancer in a patient comprising administering pertuzumab and chemotherapy to the patient, where the low HER3 cancer expresses HER3 mRNA at less than or equal to 2.81 as assessed by PCR.

Z9 0

UT DIIDW:2013F70946

ER

PT J

AU Teng, Rong Yue
Zhou, Ji Chun
Jiang, Zi Nong
Xu, Chao Yang
Li, Zi Duo
Wang, Qing Chuan
Xu, Chen Pu
Guo, Ju Feng
Shen, Jian Guo
Wang, Lin Bo

TI The relationship between Lin28 and the chemotherapy response of gastric cancer

SO ONCOTARGETS AND THERAPY

VL 6

BP 1341

EP 1345

DI 10.2147/OTT.S45705

PD 2013

PY 2013

AB Objective: The aim of the study reported here was to identify whether a stem cell biomarker, Lin28, may predict the pathologic tumor response to neoadjuvant chemotherapy for patients with locally advanced gastric cancer.

Methods: The study enrolled 47 patients with gastric cancer who underwent neoadjuvant chemotherapy followed by surgery between July 2004 and March 2012. Cancer tissue was biopsied by gastroscopy and Lin28

expression in the tissue was measured by immunohistochemistry.
Statistical analyses were performed to identify the relationship between Lin28 expression and tumor regression grade.
Results: Of the 47 cases, pathologic nonresponse was observed in 29 (61.7%) and pathologic response in 18 (38.3%). Receiver-operating characteristic curve analysis showed that the histo-score of Lin28 expression with 0.325 as a cutoff value could differentiate between pathologic response and nonresponse. Multivariable analysis showed that Lin28 expression was an independent predictive factor for pathologic response to neoadjuvant chemotherapy (P = 0.006).
Conclusion: Lin28 expression was associated with pathologic tumor response in locally advanced gastric cancer patients undergoing neoadjuvant chemotherapy. This may suggest that Lin28 can serve as a predictive biomarker for neoadjuvant chemotherapy in patients with gastric cancer.
TC 4
ZB 3
Z8 1
ZS 0
Z9 5
SN 1178-6930
UT WOS:000324737500001
PM 24098084
ER

PT J
AU Ge, Lei
Wang, Hai-Jiang
Yin, Dong
Lei, Cheng
Zhu, Jin-Feng
Cai, Xiao-Hui
Zhang, Guo-Qing
TI Effectiveness of 5-flurouracil-based neoadjuvant chemotherapy in locally-advanced gastric/gastroesophageal cancer: A meta-analysis
SO WORLD JOURNAL OF GASTROENTEROLOGY
VL 18
IS 48
BP 7384

EP 7393
DI 10.3748/wjg.v18.i48.7384
PD DEC 28 2012
PY 2012
AB AIM: To investigate the effectiveness of 5-flurouracil-based neoadjuvant chemotherapy (NAC) for gastroesophageal and gastric cancer by meta-analysis.
METHODS: MEDLINE and manual searches were performed to identify all published randomized controlled trials (RCTs) investigating the efficacy of the flurouracil-based NAC for gastroesophageal and gastric cancer, and RCTs of NAC for advanced gastroesophageal and gastric cancer vs no therapy before surgery. Studies that included patients with metastases at enrollment were excluded. Primary endpoint was the odds ratio (OR) for improving overall survival rate of patients with gastroesophageal and gastric cancer. Secondary endpoints were the OR of efficiency for down-staging tumor and increasing R0 resection in patients with gastroesophageal and gastric cancer. Safety analyses were also performed. The OR was the principal measurement of effect, which was calculated as the treatment group (NAC plus surgery) vs control group (surgery alone) and was presented as a point estimate with 95% confidence intervals (CI). All calculations and statistical tests were performed using RevMan 5.1 software.
RESULTS: Seven RCTs were included for the analysis. A total of 1249 patients with advanced gastroesophageal and gastric cancer enrolled in the seven trials were divided into treatment group (n = 620) and control group (n = 629). The quality scores of the RCTs were assessed according to the method of Jadad. The RCT quality scores ranged from 2 to 7 (5-point scale), with a mean of 3.75. The median follow-up time in these studies was over 3 years. The meta-analysis showed that NAC improved the overall survival rate (OR 1.40, 95%CI 1.11-1.76; P = 0.005), which was statistically significant. The 3-year progression-free survival rate was significantly higher in treatment group than in control group (37.7% vs 27.3%) (OR 1.62, 95%CI 1.21-2.15; P = 0.001). The tumor down-stage rate was higher in treatment group than in control group (55.76% vs 41.38%) (OR 1.77, 95%CI 1.27-2.49; P = 0.0009) and the R0 resection rate of the gastroesophageal and gastric cancer was higher in treatment group than in control group (75.11% vs 68.56%) (OR 1.38, 95%CI 1.03-1.85; P = 0.03), with significant differences. No obvious safety concerns about mortality and complications were raised in these trials. There were no

statistically significant differences in perioperative mortality (5.08% vs 4.86%) (OR 1.05, 95%CI 0.57-1.94; P = 0.87 fixed-effect model) and in the complication rate between the two groups (13.25% vs 9.66%) (OR 1.40, 95%CI 0.91-2.14; P = 0.12 fixed-effect model). Trials showed that patients from Western countries favored NAC compared with those from Asian countries (OR 1.40, 95%CI 1.07-1.83). Monotherapy was inferior to multiple chemotherapy (OR 1.40, 95%CI 1.07-1.83). Intravenous administration of NAC was more advantageous than oral route (OR 1.41, 95%CI 1.09-1.81).
CONCLUSION: Flurouracil-based NAC can safely improve overall survival rate of patients with gastroesophageal/gastric cancer. Additionally, NAC can down the tumor stage and improve R0 resection. (C) 2012 Baishideng. All rights reserved.
TC 6
ZB 2
Z8 1
ZS 0
Z9 7
SN 1007-9327
UT WOS:000313858300034
PM 23326149
ER

PT J
AU Morton, U. K. Dion
Seymour, Matt
Magill, Laura
Handley, Kelly
Brown, Gina
Ferry, David
West, Nick
Quirke, Philip
Warren, Bryan
Gray, Richard
CA Foxtrot Collaborative Grp
TI Feasibility of preoperative chemotherapy for locally advanced, operable colon cancer: the pilot phase of a randomised controlled trial
SO LANCET ONCOLOGY
VL 13

IS 11
BP 1152
EP 1160
DI 10.1016/S1470-2045(12)70348-0
PD NOV 2012
PY 2012
AB Background Preoperative (neoadjuvant) chemotherapy and radiotherapy are more effective than similar postoperative treatment for oesophageal, gastric, and rectal cancers, perhaps because of more effective micrometastasis eradication and reduced risk of incomplete excision and tumour cell shedding during surgery. The FOxTROT trial aims to investigate the feasibility, safety, and efficacy of preoperative chemotherapy for colon cancer.
Methods In the pilot stage of this randomised controlled trial, 150 patients with radiologically staged locally advanced (T3 with >= 5 mm invasion beyond the muscularis propria or T4) tumours from 35 UK centres were randomly assigned (2:1) to preoperative (three cycles of OxMdG [oxaliplatin 85 mg/m(2), l-folinic acid 175 mg, fluorouracil 400 mg/m(2) bolus, then 2400 mg/m(2) by 46 h infusion] repeated at 2-weekly intervals followed by surgery and a further nine cycles of OxMdG) or standard postoperative chemotherapy (12 cycles of OxMdG). Patients with KRAS wild-type tumours were randomly assigned (1:1) to receive panitumumab (6 mg/kg; every 2 weeks with the first 6 weeks of chemotherapy) or not. Treatment allocation was through a central randomisation service using a minimised randomisation procedure including age, radiological T and N stage, site of tumour, and presence of defunctioning colostomy as stratification variables. Primary outcome measures of the pilot phase were feasibility, safety, and tolerance of preoperative therapy, and accuracy of radiological staging. Analysis was by intention to treat. This trial is registered, number ISRCTN 87163246.
Findings 96% (95 of 99) of patients started and 89% (85 of 95) completed preoperative chemotherapy with grade 3-4 gastrointestinal toxicity in 7% (seven of 94) of patients. All 99 tumours in the preoperative group were resected, with no significant differences in postoperative morbidity between the preoperative and control groups: 14% (14 of 99) versus 12% (six of 51) had complications prolonging hospital stay (p=0.81). 98% (50 of 51) of postoperative chemotherapy patients had T3 or more advanced tumours confirmed at post-resection pathology compared with 91% (90 of 99) of patients following preoperative chemotherapy (p=0.10).

Preoperative therapy resulted in significant downstaging of TNM5
compared with the postoperative group (p=0.04), including two
pathological complete responses, apical node involvement (1% [one of 98]
vs 20% [ten of 50], p<0.0001), resection margin involvement (4% [four of
99] vs 20% [ten of 50], p=0.002), and blinded centrally scored tumour
regression grading: 31% (29 of 94) vs 2% (one of 46) moderate or greater
regression (p=0.0001).
Interpretation Preoperative chemotherapy for radiologically staged,
locally advanced operable primary colon cancer is feasible with
acceptable toxicity and perioperative morbidity. Proceeding to the phase
3 trial, to establish whether the encouraging pathological responses
seen with preoperative therapy translates into improved long-term
oncological outcome, is appropriate.
Funding Cancer Research UK.
TC 32
ZB 13
Z8 0
ZS 0
Z9 33
SN 1470-2045
UT WOS:000310570900046
ER

PT J
AU Chakravarthy, A Bapsi
Catalano, Paul J
Mondschein, Joshua K
Rosenthal, David I
Haller, Daniel G
Whittington, Richard
Spitz, Francis R
Wagner, Henry
Sigurdson, Elin R
Tschetter, Loren K
Bayer, Gerald K
Mulcahy, Mary F
Benson, Al B
TI Phase II Trial of Paclitaxel/Cisplatin Followed by Surgery and Adjuvant
Radiation Therapy and 5-Fluorouracil/Leucovorin for Gastric Cancer (ECOG

E7296).
SO Gastrointestinal cancer research : GCR
VL 5
IS 6
BP 191
EP 7
PD 2012-Nov
PY 2012
AB BACKGROUND: Randomized trials have shown an increase in survival with perioperative chemotherapy as well as with postoperative chemoradiation. It was hypothesized that combining induction chemotherapy with postoperative chemoradiation would be well tolerated and improve pathologic complete response.
METHODS: Patients with resectable cancers of the stomach/gastroesophageal junction were eligible. Neoadjuvant chemotherapy consisted of 3 cycles of paclitaxel and cisplatin. Adjuvant therapy consisted of 1 cycle of 5-fluorouracil (FU) and leucovorin (LV) followed by chemoradiation (45 Gy with concurrent 5-FU/LV). Chemoradiation was followed by 2 additional cycles of 5-FU/LV. Response to neoadjuvant therapy was based on pathology.
RESULTS: From 1999 to 2002, 38 eligible patients were enrolled; 35 completed induction chemotherapy, and 29 went on to surgery. Sixteen patients did not develop metastatic progression, 10 developed metastatic disease, and 12 were unevaluable. There were no pathologic complete responses after induction therapy. Twenty-five of 38 patients suffered grade 3-4 toxicities during induction paclitaxel/cisplatin. Six of the 7 patients who received postoperative therapy suffered grade 3-4 toxicities. Only 3 of 38 (7.9%) eligible patients completed all assigned treatment. The median overall survival was 1.6 years, and the 2-year survival was 40%.
CONCLUSIONS: This regimen of neoadjuvant paclitaxel/cisplatin followed by postoperative 5-FU/LV-based chemoradiation did not have a high enough response rate and proved to be too toxic for further development.
TC 1
ZB 1
Z8 0
ZS 0
Z9 1
SN 1934-7820

UT MEDLINE:23293700
PM 23293700
ER

PT J
AU Foxtrot Collaborative Group
TI Feasibility of preoperative chemotherapy for locally advanced, operable colon cancer: the pilot phase of a randomised controlled trial.
SO The Lancet. Oncology
VL 13
IS 11
BP 1152
EP 60
DI 10.1016/S1470-2045(12)70348-0
PD 2012-Nov
PY 2012
AB BACKGROUND: Preoperative (neoadjuvant) chemotherapy and radiotherapy are more effective than similar postoperative treatment for oesophageal, gastric, and rectal cancers, perhaps because of more effective micrometastasis eradication and reduced risk of incomplete excision and tumour cell shedding during surgery. The FOxTROT trial aims to investigate the feasibility, safety, and efficacy of preoperative chemotherapy for colon cancer.
METHODS: In the pilot stage of this randomised controlled trial, 150 patients with radiologically staged locally advanced (T3 with ≥5 mm invasion beyond the muscularis propria or T4) tumours from 35 UK centres were randomly assigned (2:1) to preoperative (three cycles of OxMdG [oxaliplatin 85 mg/m(2), l-folinic acid 175 mg, fluorouracil 400 mg/m(2) bolus, then 2400 mg/m(2) by 46 h infusion] repeated at 2-weekly intervals followed by surgery and a further nine cycles of OxMdG) or standard postoperative chemotherapy (12 cycles of OxMdG). Patients with KRAS wild-type tumours were randomly assigned (1:1) to receive panitumumab (6 mg/kg; every 2 weeks with the first 6 weeks of chemotherapy) or not. Treatment allocation was through a central randomisation service using a minimised randomisation procedure including age, radiological T and N stage, site of tumour, and presence of defunctioning colostomy as stratification variables. Primary outcome measures of the pilot phase were feasibility, safety, and tolerance of preoperative therapy, and accuracy of radiological staging. Analysis was

by intention to treat. This trial is registered, number ISRCTN 87163246.
FINDINGS: 96% (95 of 99) of patients started and 89% (85 of 95) completed preoperative chemotherapy with grade 3-4 gastrointestinal toxicity in 7% (seven of 94) of patients. All 99 tumours in the preoperative group were resected, with no significant differences in postoperative morbidity between the preoperative and control groups: 14% (14 of 99) versus 12% (six of 51) had complications prolonging hospital stay (p=0•81). 98% (50 of 51) of postoperative chemotherapy patients had T3 or more advanced tumours confirmed at post-resection pathology compared with 91% (90 of 99) of patients following preoperative chemotherapy (p=0•10). Preoperative therapy resulted in significant downstaging of TNM5 compared with the postoperative group (p=0•04), including two pathological complete responses, apical node involvement (1% [one of 98] vs 20% [ten of 50], p<0•0001), resection margin involvement (4% [four of 99] vs 20% [ten of 50], p=0•002), and blinded centrally scored tumour regression grading: 31% (29 of 94) vs 2% (one of 46) moderate or greater regression (p=0•0001).
INTERPRETATION: Preoperative chemotherapy for radiologically staged, locally advanced operable primary colon cancer is feasible with acceptable toxicity and perioperative morbidity. Proceeding to the phase 3 trial, to establish whether the encouraging pathological responses seen with preoperative therapy translates into improved long-term oncological outcome, is appropriate.
FUNDING: Cancer Research UK.
TC 0
ZB 0
Z8 0
ZS 0
Z9 0
UT MEDLINE:23017669
PM 23017669
ER

PT J
AU Liu, Kun
Qian, Tao
Tang, Liming
Wang, Jie
Yang, Haohua

Ren, Jun

TI Decreased expression of microRNA let-7i and its association with chemotherapeutic response in human gastric cancer

SO WORLD JOURNAL OF SURGICAL ONCOLOGY

VL 10

AR 225

DI 10.1186/1477-7819-10-225

PD OCT 29 2012

PY 2012

AB Background: MicroRNA let-7i has been proven to be down-regulated in many human malignancies and correlated with tumor progression and anticancer drug resistance. Our study aims to characterize the contribution of miRNA let-7i to the initiation and malignant progression of locally advanced gastric cancer (LAGC), and evaluate its possible value in neoadjuvant chemotherapeutic efficacy prediction.

Methods: Eighty-six previously untreated LAGC patients who underwent preoperative chemotherapy and radical resection were included in our study. Let-7i expression was examined for pairs of cancer tissues and corresponding normal adjacent tissues (NATs), using quantitative RT-PCR. The relationship of let-7i level to clinicopathological characteristics, pathologic tumor regression grades after chemotherapy, and overall survival (OS) was also investigated.

Results: Let-7i was significantly down-regulated in most tumor tissues (78/86: 91%) compared with paired NATs (P < 0.001), and low levels of let-7i were significantly correlated with local invasion, lymphatic metastasis, and poor pathologic tumor response. Multivariate Cox regression analysis revealed that low let-7i expression was an unfavorable prognostic factor of OS (hazard ratio (HR) = 2.316, P = 0.024) independently of other clinicopathological factors, including tumor node metastasis (TNM) stage (HR = 3.226, P = 0.013), depth of infiltration (HR = 4.167, P < 0.001), and lymph node status (HR = 2.245, P = 0.037).

Conclusions: These findings indicate that let-7i may be a good candidate for use a therapeutic target and a potential tissue marker for the prediction of chemotherapeutic sensitivity and prognosis in LAGC patients.

TC 15

ZB 11

Z8 1

ZS 0
Z9 15
SN 1477-7819
UT WOS:000311546000001
PM 23107361
ER

PT J
AU Wenners, Antonia Sophie
   Mehta, Keyur
   Loibl, Sibylle
   Park, Hyerim
   Mueller, Berit
   Arnold, Norbert
   Hamann, Sigrid
   Weimer, Joerg
   Ataseven, Beyhan
   Darb-Esfahani, Silvia
   Schem, Christian
   Mundhenke, Christoph
   Khandan, Fariba
   Thomssen, Christoph
   Jonat, Walter
   Holzhausen, Hans-Juergen
   von Minckwitz, Gunther
   Denkert, Carsten
   Bauer, Maret
TI Neutrophil Gelatinase-Associated Lipocalin (NGAL) Predicts Response to
   Neoadjuvant Chemotherapy and Clinical Outcome in Primary Human Breast
   Cancer
SO PLOS ONE
VL 7
IS 10
AR e45826
DI 10.1371/journal.pone.0045826
PD OCT 9 2012
PY 2012
AB In our previous work we showed that NGAL, a protein involved in the
   regulation of proliferation and differentiation, is overexpressed in

human breast cancer (BC) and predicts poor prognosis. In neoadjuvant chemotherapy (NACT) pathological complete response (pCR) is a predictor for outcome. The aim of this study was to evaluate NGAL as a predictor of response to NACT and to validate NGAL as a prognostic factor for clinical outcome in patients with primary BC. Immunohistochemistry was performed on tissue microarrays from 652 core biopsies from BC patients, who underwent NACT in the GeparTrio trial. NGAL expression and intensity was evaluated separately. NGAL was detected in 42.2% of the breast carcinomas in the cytoplasm. NGAL expression correlated with negative hormone receptor (HR) status, but not with other baseline parameters. NGAL expression did not correlate with pCR in the full population, however, NGAL expression and staining intensity were significantly associated with higher pCR rates in patients with positive HR status. In addition, strong NGAL expression correlated with higher pCR rates in node negative patients, patients with histological grade 1 or 2 tumors and a tumor size,40 mm. In univariate survival analysis, positive NGAL expression and strong staining intensity correlated with decreased disease-free survival (DFS) in the entire cohort and different subgroups, including HR positive patients. Similar correlations were found for intense staining and decreased overall survival (OS). In multivariate analysis, NGAL expression remained an independent prognostic factor for DFS. The results show that in low-risk subgroups, NGAL was found to be a predictive marker for pCR after NACT. Furthermore, NGAL could be validated as an independent prognostic factor for decreased DFS in primary human BC.
RI Weimer, Jorg/E-3472-2010; Arnold, Norbert/E-3012-2010
OI Weimer, Jorg/0000-0002-4528-8509; Arnold, Norbert/0000-0003-4523-8808
TC 9
ZB 7
Z8 2
ZS 0
Z9 12
SN 1932-6203
UT WOS:000309889400008
PM 23056218
ER

PT J
AU Bauer, Lukas

Langer, Rupert
Becker, Karen
Hapfelmeier, Alexander
Ott, Katja
Novotny, Alexander
Hoefler, Heinz
Keller, Gisela
TI Expression Profiling of Stem Cell-Related Genes in Neoadjuvant-Treated Gastric Cancer: A NOTCH2, GSK3B and beta-catenin Gene Signature Predicts Survival
SO PLOS ONE
VL 7
IS 9
AR e44566
DI 10.1371/journal.pone.0044566
PD SEP 10 2012
PY 2012
AB Cancer stem cell (CSC) based gene expression signatures are associated with prognosis in various tumour types and CSCs are suggested to be particularly drug resistant. The aim of our study was first, to determine the prognostic significance of CSC-related gene expression in residual tumour cells of neoadjuvant-treated gastric cancer (GC) patients. Second, we wished to examine, whether expression alterations between pre- and post-therapeutic tumour samples exist, consistent with an enrichment of drug resistant tumour cells. The expression of 44 genes was analysed in 63 formalin-fixed, paraffin embedded tumour specimens with partial tumour regression (10-50% residual tumour) after neoadjuvant chemotherapy by quantitative real time PCR low-density arrays. A signature of combined GSK3B(high), beta-catenin (CTNNB1)(high) and NOTCH2(low) expression was strongly correlated with better patient survival ($p < 0.001$). A prognostic relevance of these genes was also found analysing publically available gene expression data. The expression of 9 genes was compared between pretherapeutic biopsies and post-therapeutic resected specimens. A significant post-therapeutic increase in NOTCH2, LGR5 and POU5F1 expression was found in tumours with different tumour regression grades. No significant alterations were observed for GSK3B and CTNNB1. Immunohistochemical analysis demonstrated a chemotherapy-associated increase in the intensity of NOTCH2 staining, but not in the percentage of NOTCH2. Taken together, the GSK3B, CTNNB1

and NOTCH2 expression signature is a novel, promising prognostic parameter for GC. The results of the differential expression analysis indicate a prominent role for NOTCH2 and chemotherapy resistance in GC, which seems to be related to an effect of the drugs on NOTCH2 expression rather than to an enrichment of NOTCH2 expressing tumour cells.
TC 1
ZB 1
Z8 1
ZS 0
Z9 2
SN 1932-6203
UT WOS:000308748400023
PM 22970250
ER

PT J
AU Inoue, Tatsushi
Yachida, Shinichi
Usuki, Hisashi
Kimura, Tomoki
Hagiike, Masanobu
Okano, Keiichi
Suzuki, Yasuyuki
TI Pilot Feasibility Study of Neoadjuvant Chemoradiotherapy with S-1 in Patients with Locally Advanced Gastric Cancer Featuring Adjacent Tissue Invasion or JGCA Bulky N2 Lymph Node Metastases
SO ANNALS OF SURGICAL ONCOLOGY
VL 19
IS 9
BP 2937
EP 2945
DI 10.1245/s10434-012-2332-4
PD SEP 2012
PY 2012
AB To improve the prognosis of locally advanced gastric cancer, clinical trials of neoadjuvant chemotherapy (NAC) are being performed. Although neoadjuvant chemoradiotherapy (NACRT) generally achieves superior local tumor control to NAC, its efficacy for locally advanced gastric cancers remains unclear. Therefore, a prospective trial was conducted to explore

the feasibility and safety of NACRT with oral S-1 in a series of cases. Patients who had Japanese Gastric Cancer Association (JGCA) cStage IIIB gastric cancer were enrolled onto this study and received oral S-1 (65 mg/m(2)/day) administration and 50-Gy radiotherapy followed by radical surgery. The primary end points were completion of therapy and safety. Between October 2005 and September 2008, 12 eligible patients were enrolled. Two could not complete the chemotherapy because of grade 3 toxicity. R0 resections were performed in 11 patients (91.7 %) (95 % confidence interval 61.5-99.8). Although operative morbidity was observed in two cases, there were no postoperative deaths. A pathologic response was observed in 10 patients (83.3 %). In five (62.5 %) of eight gastric cancers with invasion to adjacent structures, microscopic tumor deposits were not found in the affected organs. The 3-year survival rate was 58.3 % during a median follow-up period of 36 months.
Although this study is preliminary, the present regimen seems to be feasible and safe as a treatment for locally advanced gastric cancers featuring adjacent tissue invasion or JGCA bulky N2 disease. This treatment approach should now be tested using the new tumor, node, metastasis staging system in a large clinical trial.
TC 5
ZB 2
Z8 1
ZS 0
Z9 6
SN 1068-9265
UT WOS:000308357100022
PM 22466666
ER

PT J
AU Gillies, R. S.
Middleton, M. R.
Blesing, C.
Patel, K.
Warner, N.
Marshall, R. E. K.
Maynard, N. D.
Bradley, K. M.
Gleeson, F. V.

TI Metabolic response at repeat PET/CT predicts pathological response to neoadjuvant chemotherapy in oesophageal cancer
SO EUROPEAN RADIOLOGY
VL 22
IS 9
BP 2035
EP 2043
DI 10.1007/s00330-012-2459-5
PD SEP 2012
PY 2012
AB Reports have suggested that a reduction in tumour 18F-fluorodeoxyglucose (FDG) uptake on positron emission tomography (PET) examination during or after neoadjuvant chemotherapy may predict pathological response in oesophageal cancer. Our aim was to determine whether metabolic response predicts pathological response to a standardised neoadjuvant chemotherapy regimen within a prospective clinical trial.

Consecutive patients staged with potentially curable oesophageal cancer who underwent treatment within a non-randomised clinical trial were included. A standardised chemotherapy regimen (two cycles of oxaliplatin and 5-fluorouracil) was used. PET/CT was performed before chemotherapy and repeated 24-28 days after the start of cycle 2.

Forty-eight subjects were included: mean age 65 years; 37 male. Using the median percentage reduction in SUVmax (42%) to define metabolic response, pathological response was seen in 71% of metabolic responders (17/24) compared with 33% of non-responders (8/24; P = 0.009, sensitivity 68%, specificity 70%). Pathological response was seen in 81% of subjects with a complete metabolic response (13/16) compared with 38% of those with a less than complete response (12/32; P = 0.0042, sensitivity 52%, specificity 87%). There was no significant histology-based effect.

There was a significant association between metabolic response and pathological response; however, accuracy in predicting pathological response was relatively low.

aEuro cent PET/CT may predict tumour response to chemotherapy in oesophageal cancer.

aEuro cent This was a prospective study using a standardised chemotherapy regimen.

aEuro cent A significant association between PET/CT findings and disease response was found.

aEuro cent However accuracy in predicting pathological response was relatively low.

TC 4

ZB 1

Z8 0

ZS 0

Z9 4

SN 0938-7994

UT WOS:000307294500024

PM 22562089

ER

PT J

AU Guo, Kang
Cai, Ling
Zhang, Yu
Zhu, Jian-Fei
Rong, Tie-Hua
Lin, Peng
Hao, Chong-Li
Wang, Wu-Ping
Li, Zhe
Zhang, Lan-Jun

TI The predictive value of histological tumor regression grading (TRG) for therapeutic evaluation in locally advanced esophageal carcinoma treated with neoadjuvant chemotherapy

SO CHINESE JOURNAL OF CANCER

VL 31

IS 8

BP 399

EP 408

DI 10.5732/cjc.011.10406

PD AUG 2012

PY 2012

AB Response criteria remain controversial in therapeutic evaluation for locally advanced esophageal carcinoma treated with neoadjuvant chemotherapy. We aimed to identify the predictive value of tumor regression grading (TRG) in tumor response and prognosis. Fifty-two patients who underwent neoadjuvant chemotherapy followed by

esophagectomy and radical 2-field lymphadenectomy between June 2007 and June 2011 were included in this study. All tissue specimens were reassessed according to the TRG scale. Potential prognostic factors, including clinicopathologic factors, were evaluated. Survival curves were generated by using the Kaplan-Meier method and compared with the log-rank test. Prognostic factors were determined with multivariate analysis by using the Cox regression model. Our results showed that of 52 cases, 43 (83%) were squamous cell carcinoma and 9 (17%) were adenocarcinoma. TRG was correlated with pathologic T (P = 0.006) and N (P < 0.001) categories. Median overall survival for the entire cohort was 33 months. The 1- and 2-year overall survival rates were 71% and 44%, respectively. Univariate survival analysis results showed that favorable prognostic factors were histological subtype (P = 0.003), pathologic T category (P = 0.026), pathologic N category (P < 0.001), and TRG G0 (P = 0.041). Multivariate analyses identified pathologic N category (P < 0.001) as a significant independent prognostic parameter. Our results indicate that histomorphologic TRG can be considered as an alternative option to predict the therapeutic efficacy and prognostic factor for patients with locally advanced esophageal carcinoma treated by neoadjuvant chemotherapy.

TC 1
ZB 0
Z8 0
ZS 0
Z9 1
SN 1000-467X
UT WOS:000209009700006
PM 22572013
ER

PT J
AU McNamara, Michael J.
   Adelstein, David J.
TI Current Developments in the Management of Locally Advanced Esophageal
   Cancer
SO CURRENT ONCOLOGY REPORTS
VL 14
IS 4
BP 342

EP 349
DI 10.1007/s11912-012-0239-7
PD AUG 2012
PY 2012
AB Loco-regionally advanced esophageal cancer is a lethal disease with poor outcomes despite aggressive multimodality therapy. The appropriate management of these patients is contentious and no single standard of care has been defined. Literature suggests that preoperative chemoradiotherapy may be superior to preoperative chemotherapy. Recently, several developments have impacted the care of these patients. The 2010 AJCC TNM staging system now recognizes the biologic heterogeneity of the disease and stages adenocarcinoma and squamous cell carcinoma separately. Studies suggest potentially less toxic chemotherapeutic agents including oxaliplatin may be useful in the management of this disease. FDG-PET imaging appears to have prognostic value and may predict for pathologic response. In addition, several trials have explored inhibition of the ErbB1 (EGFR) and ErbB2 (Her2) receptors. The monoclonal antibody trastuzumab appears to extend survival for patients with metastatic gastric and gastroesophageal junction adenocarcinoma and is under investigation for use in patients with loco-regionally advanced disease.
TC 9
ZB 5
Z8 0
ZS 0
Z9 9
SN 1523-3790
UT WOS:000305953500009
PM 22544559
ER

PT J
AU Lorenzen, Sylvie
Blank, Susanne
Lordick, Florian
Siewert, Joerg-Ruediger
Ott, Katja
TI Prediction of Response and Prognosis by a Score Including Only Pretherapeutic Parameters in 410 Neoadjuvant Treated Gastric Cancer

Patients
SO ANNALS OF SURGICAL ONCOLOGY
VL 19
IS 7
BP 2119
EP 2127
DI 10.1245/s10434-012-2254-1
PD JUL 2012
PY 2012
AB Response to neoadjuvant chemotherapy is an independent prognostic factor in locally advanced gastric cancer. However, no prospectively tested pretherapeutic parameters predicting response and/or survival in gastric cancer are available in clinical routine.

We evaluated the prognostic significance of various clinical pathologic parameters in 410 patients who were treated with neoadjuvant chemotherapy followed by gastrectomy. Clinical and histopathologic response evaluation was performed by using standardized criteria. A prognostic score was created on the basis of the variables identified in the multivariate analysis.

Three pretherapeutic parameters were identified as positive predictive factors for response and prognosis: tumor localization in the middle third of the stomach (P = 0.001), well-differentiated tumors (P = 0.001), and intestinal tumor type according to Laur,n classification (P = 0.03). A prognostic index was constructed, dividing the patients into three risk groups: low (n = 73), intermediate (n = 274), and high (n = 63). The three groups had significantly different clinical (P = 0.007) and histopathologic response rates (P = 0.001) and survival times, with a median survival time that was not reached in the low-risk group, 39.2 months in the intermediate-risk group, and 20.5 months in the high-risk group. The corresponding 5-year survival rates were 65.3, 41.2, and 21.2% (P < 0.001), respectively.

A simple scoring system based on three clinicopathologic parameters accurately predicts response and prognosis in neoadjuvant treated gastric cancer. This system provides additional useful information that could be applied to select gastric cancer patients pretherapeutically for different treatment approaches. Prospective testing of the score in an independent patient cohort is warranted.
TC 12
ZB 6

Z8 0

ZS 0

Z9 12

SN 1068-9265

UT WOS:000305558000009

PM 22395980

ER

PT J

AU Bendell, Johanna C

Meluch, Anthony

Peyton, James

Rubin, Mark

Waterhouse, David

Webb, Charles

Burris, Howard A 3rd

Hainsworth, John D

TI A phase II trial of preoperative concurrent chemotherapy/radiation therapy plus bevacizumab/erlotinib in the treatment of localized esophageal cancer.

SO Clinical advances in hematology & oncology : H&O

VL 10

IS 7

BP 430

EP 7

PD 2012-Jul

PY 2012

AB PURPOSE: To evaluate the efficacy of bevacizumab (Avastin, Genentech) and erlotinib (Tarceva, Genentech/Roche) when added to preoperative chemoradiation therapy with paclitaxel, carboplatin, and infusional 5-fluorouracil (5-FU) in the treatment of localized cancers of the esophagus or gastroesophageal (GE) junction. The primary endpoint was the pathologic complete response (pCR) rate.

METHODS: Eligible patients had previously untreated localized squamous cell, adenocarcinoma, or adenosquamous carcinoma of the esophagus or GE junction, and were considered surgical candidates at enrollment. Daily erlotinib (100 mg orally) was administered on days 1-42 of preoperative treatment. Patients received paclitaxel (200 mg/m2 intravenously [IV]), carboplatin (area under the curve [AUC] 5.0 IV), and bevacizumab (15

mg/kg IV) on days 1 and 22, and 5-FU by continuous infusion (225 mg/m2/day IV) on days 1-35, with radiation therapy in 1.8-Gy single fractions, Monday-Friday (to a total of 45 Gy). Those who were deemed surgical candidates proceeded to resection during weeks 12-14.
RESULTS: Between February 2007 and September 2009, 62 patients (median age, 64 years; 92% male; 94% adenocarcinoma) were enrolled; 44 patients (71%) completed neoadjuvant treatment and proceeded to surgery. Eighteen patients (29%) achieved pCR, with partial pathologic remission in an additional 22 patients (35%). Common grade 3/4 toxicities included leukopenia (64%), neutropenia (44%), mucositis/stomatitis (42%), diarrhea (27%), and esophagitis (27%). There were 40 instances of treatment-related hospitalization, and 2 postoperative deaths.
CONCLUSIONS: The addition of bevacizumab and erlotinib to neoadjuvant chemoradiation did not demonstrate survival benefit or improved pCR rate over similar regimens. While the overall rates of toxicity were not increased, targeted agent-specific toxicity was evident. Further study of this specific regimen is not warranted.
TC 10
ZB 4
Z8 3
ZS 0
Z9 13
SN 1543-0790
UT MEDLINE:22895283
PM 22895283
ER

PT J
AU Zhang Jun
Chen Ren-xiong
Zhang Jing
Cai Jun
Meng Hua
Wu Guo-cong
Zhang Zhong-tao
Wang Yu
Wang Kang-li
TI Efficacy and safety of neoadjuvant chemotherapy with modified FOLFOX7 regimen on the treatment of advanced gastric cancer

SO CHINESE MEDICAL JOURNAL
VL 125
IS 12
BP 2144
EP 2150
DI 10.3760/cma.j.issn.0366-6999.2012.12.012
PD JUN 20 2012
PY 2012
AB Background Gastric cancer is one of the most common types of malignant tumors in China and East Asia and has the highest mortality rate of the malignant gastrointestinal tumors. Neoadjuvant chemotherapy is a systemic or local chemotherapy that is given prior to the local treatment of malignant tumors. Neoadjuvant therapy is currently showing some positive prospects; however, its clinical effects remain controversial. In this study, we used the modified FOLFOX7 (mFOLFOX7) regimen as a neoadjuvant chemotherapy regimen. Perioperative clinical and pathological efficacy, toxicity, effects of surgery, postoperative observation, and prognosis were studied to investigate its clinical efficacy and safety.

Methods Eighty patients with advanced gastric cancer were treated in our surgery department from 2005 to 2009; 38 of these patients received mFOLFOX7 neoadjuvant chemotherapy, the other 42 patients assigned to the control group. The perioperative effects of mFOLFOX7 chemotherapy, including clinical effects and toxicity, were observed in each patient.

Results After mFOLFOX7 chemotherapy, clinical and pathologic stages decreased in 21.1% and 36.8% of the patients, respectively, but the results were not statistically significant (P=0.129). The clinical response rate was 50% (19/38). Toxicity was mild; most adverse events were grade I or II and involved no severe infections or deaths. Compared with the control group, the radical resection rate increased (92.1% vs. 85.7%; P=0.437); surgical effects were completed without an increased incidence of perioperative complications. The 1-, 2-, and 3-year survival rates were 78.70%, 57.40%, and 51.66%, respectively, in the neoadjuvant chemotherapy group and 78.57%, 56.87%, and 43.16%, respectively, in the control group.

Conclusions The mFOLFOX7 regimen was very effective and well-tolerated as a neoadjuvant chemotherapy for advanced gastric cancer. However, the 1-, 2-, and 3-year survival rates in the mFOLFOX7 group were not significantly different from the control group. Chin Med J

2012;125(12):2144-2150
TC 4
ZB 3
Z8 9
ZS 0
Z9 12
SN 0366-6999
UT WOS:000306382400012
PM 22884144
ER

PT J
AU Katayama, Hiroshi
Ito, Seiji
Sano, Takeshi
Takahari, Daisuke
Mizusawa, Junki
Boku, Narikazu
Tsuburaya, Akira
Terashima, Masanori
Sasako, Mitsuru
CA Japan Clinical Oncology Grp
TI A Phase II Study of Systemic Chemotherapy with Docetaxel, Cisplatin, and S-1 (DCS) Followed by Surgery in Gastric Cancer Patients with Extensive Lymph Node Metastasis: Japan Clinical Oncology Group Study JCOG1002
SO JAPANESE JOURNAL OF CLINICAL ONCOLOGY
VL 42
IS 6
BP 556
EP 559
DI 10.1093/jjco/hys054
PD JUN 2012
PY 2012
AB A Phase II trial was initiated in Japan to evaluate the efficacy and safety of preoperative chemotherapy with docetaxel, cisplatin and S-1 for gastric cancer with extensive lymph node metastasis. Patients are eligible to participate in the study if they have para-aortic lymph node metastases (stations no. 16a2/16b1) and/or a bulky lymph node (epsilon 3 cm 1 or epsilon 1.5 cm 2) along the celiac, splenic, common or proper

hepatic arteries or the superior mesenteric vein, while patients with other distant metastases are ineligible. A total of 50 patients will be enrolled over 2.5 years. The primary endpoint is the response rate of the preoperative chemotherapy, which will be assessed based on the Response Evaluation Criteria in Solid Tumors ver. 1.0. The secondary endpoints are 3-year survival, 5-year survival, proportion of patients with R0 resection, proportion of patients who complete the preoperative chemotherapy and surgery, proportion of patients who complete the protocol treatment, pathological response rate and adverse events. This trial was registered at the UMIN Clinical Trials Registry () as UMIN000006069.

TC 3

ZB 1

Z8 1

ZS 0

Z9 4

SN 0368-2811

UT WOS:000304829900015

PM 22525210

ER

PT J

AU Ferri, L. E.
Ades, S.
Alcindor, T.
Chasen, M.
Marcus, V.
Hickeson, M.
Artho, G.
Thirlwell, M. P.

TI Perioperative docetaxel, cisplatin, and 5-fluorouracil (DCF) for locally advanced esophageal and gastric adenocarcinoma: a multicenter phase II trial

SO ANNALS OF ONCOLOGY

VL 23

IS 6

BP 1512

EP 1517

DI 10.1093/annonc/mdr465

PD JUN 2012

PY 2012

AB Background: Although perioperative chemotherapy for esophagogastric adenocarcinoma (ADC) improves survival, the overall poor prognosis suggests that further refinement of treatment is required. Docetaxel, cisplatin, and 5-fluorouracil (5-FU) (DCF) is effective for metastatic ADC of the upper gastrointestinal (GI) tract; we thus sought to investigate the efficacy of this regimen in patients with resectable disease.

Patients and methods: Patients with resectable ADC of the upper GI tract received DCF [docetaxel (Taxotere) 75 mg/m(2) I. V. day 1, cisplatin 75 mg/m(2) I. V. day 1, 5-FU 750 mg/m(2) continuous infusion for 120 h, every 3 weeks] for three cycles before and after resection. Primary end point was complete resection; secondary end points were response, toxicity, surgical morbidity, and overall survival.

Results: Forty-three patients with ADC of the esophagus (11), gastroesophageal junction (25), or stomach (7) started treatment and 86% completed all preoperative cycles with grade 3-4 toxicity arising in 47%. Metabolic response to chemotherapy (reduction in maximal standard uptake value >35%) was achieved in 25/33 (76%) patients. Surgery was carried out in 41/43 and complete resection was achieved in all 41 patients with pathologic complete response in 4/41. Postoperative chemotherapy was started in 29 patients and completed in 24. Three-year overall survival was 60%.

Conclusion: Perioperative DCF is a tolerable and highly effective regimen for the treatment of esophagogastric ADC.

TC 13

ZB 7

Z8 1

ZS 1

Z9 15

SN 0923-7534

UT WOS:000304534000022

PM 22039085

ER

PT J

AU Fujitani, Kazumasa
Mano, Masayuki

Hirao, Motohiro
Kodama, Yoshinori
Tsujinaka, Toshimasa
TI Posttherapy Nodal Status, Not Graded Histologic Response, Predicts Survival after Neoadjuvant Chemotherapy for Advanced Gastric Cancer
SO ANNALS OF SURGICAL ONCOLOGY
VL 19
IS 6
BP 1936
EP 1943
DI 10.1245/s10434-011-2165-6
PD JUN 2012
PY 2012
AB Neoadjuvant chemotherapy (NAC) has been attempted as a means of improving survival of potentially resectable advanced gastric cancer (AGC). In the course of exploring the most promising NAC regimen, a superior surrogate marker reflecting overall survival (OS) is necessary. We investigated prognostic factors in AGC patients who underwent NAC followed by gastric resection and evaluated whether histologic response to NAC was predictive of survival.

Seventy consecutive patients with gastric cancer treated with NAC followed by surgical resection between Jan 1, 2000, and Dec 31, 2009, at Osaka National Hospital were identified from a prospective database. Prognostic factors for OS were investigated by univariate and multivariate analyses.

Median survival time for all patients was 668 days after surgical resection. Age less than 65 years (hazard ratio 0.463, 95% confidence interval 0.244-0.879) and pathologic nodal stage of N0-1 (hazard ratio 0.318, 95% confidence interval 0.160-0.635) were identified as significant independent prognostic factors for longer OS, whereas graded histologic response of primary tumor to NAC was statistically significant on univariate analysis, but not on multivariate analysis, as a prognostic factor.

Posttherapy nodal status, not graded histologic response, predicts survival after NAC for AGC and could serve as a reliable surrogate marker for OS in the course of exploring the most promising regimen for NAC.
TC 2
ZB 1

Z8 0
ZS 0
Z9 2
SN 1068-9265
UT WOS:000304209800030
PM 22187120
ER

PT J
AU Ilson, David H.
Minsky, Bruce D.
Ku, Geoffrey Y.
Rusch, Valerie
Rizk, Nabil
Shah, Manish
Kelsen, David P.
Capanu, Marinela
Tang, Laura
Campbell, Jenny
Bains, Manjit
TI Phase 2 trial of induction and concurrent chemoradiotherapy with weekly irinotecan and cisplatin followed by surgery for esophageal cancer
SO CANCER
VL 118
IS 11
BP 2820
EP 2827
DI 10.1002/cncr.26591
PD JUN 1 2012
PY 2012
AB BACKGROUND: Preoperative chemoradiation improves survival in esophageal and gastroesophageal junction (GEJ) cancer. We evaluated irinotecan and cisplatin as induction chemotherapy followed by concurrent chemoradiation in esophageal cancer. METHODS: Patients with uT1N1M0 or uT2-4NanyM0 resectable squamous cancer or adenocarcinoma of the esophagus or GEJ received irinotecan 65 mg/m2 and cisplatin 30 mg/m2 for 4 treatments in weeks 1 through 5, followed by 4 treatments in weeks 7 through 11 with 50.4 Gy in daily fractions, followed by surgery. The primary endpoint was pathologic complete response (pCR). Positron

emission tomography (PET) scan was performed prior to chemotherapy and as restaging prior to radiotherapy. RESULTS: Fifty-five patients were evaluable, 75% of whom had adenocarcinoma and 65% of whom had uT3N1 disease. Thirty-eight patients underwent R0 resection (69%). The incidence of pCR was 16% (95% confidence interval, 8%-29%). Median overall survival was 31.7 months. An exploratory analysis of PET response to induction chemotherapy indicated a correlation with pCR (32% vs 4%), R0 resection (84% vs 57%), progression-free survival (24.1 vs 7.7 months), and overall survival (40.2 vs 25.5 months). CONCLUSIONS: Weekly treatment with irinotecan, cisplatin, and radiation achieved results no better and potentially inferior to other phase 2 chemoradiotherapy trials with a low rate of pCR. The use of PET scan after induction chemotherapy to direct chemotherapy during subsequent radiotherapy merits further study. Cancer 2011. (c) 2011 American Cancer Society.
TC 8
ZB 0
Z8 0
ZS 0
Z9 8
SN 0008-543X
UT WOS:000304188700007
PM 21990000
ER

PT J
AU Li, Zi-Yu
Koh, Cherry E.
Bu, Zhao-De
Wu, Ai-Wen
Zhang, Lian-Hai
Wu, Xiao-Jiang
Wu, Qi
Zong, Xiang-Long
Ren, Hui
Tang, Lei
Zhang, Xiao-Peng
Li, Ji-You
Hu, Ying

Shen, Lin
Ji, Jia-Fu
TI Neoadjuvant chemotherapy with FOLFOX: Improved outcomes in Chinese patients with locally advanced gastric cancer
SO JOURNAL OF SURGICAL ONCOLOGY
VL 105
IS 8
BP 793
EP 799
DI 10.1002/jso.23009
PD JUN 2012
PY 2012
AB Background Although the role of peri-operative chemotherapy is established in the treatment of locally advanced gastric cancer, the optimal regime remains to be determined. FOLFOX has been used in palliative setting with good response rates but its role in a neoadjuvant setting is not well established. Methods This is a prospective non-randomized study comparing peri-operative FOLFOX versus adjuvant FOLFOX in patients with resectable locally advanced gastric cancer. Response to chemotherapy was assessed according to WHO criteria and pathological changes. KaplanMeier log rank test was used to calculate and compare survival differences. Results There were 73 patients (neoadjuvant?=?36). Complete and partial response was observed in 2 (6%) and 21 (64%) patients, respectively. Four-year overall survival (OS) in the neoadjuvant arm was 78% versus 51% in the adjuvant arm (P?=?0.031). Subgroup analysis found R0 resection (86% vs. 55%, P?=?0.011) and patients with proximal cancers (87% vs. 14%, P?<?0.001) to have improved OS. The most common side effect was grade 12 leukopenia. There were no grade 3 neuropathies, grade 4 cytopaenias, or treatment related deaths. Conclusion Peri-operative treatment with FOLFOX shows promise in patients with resectable locally advanced gastric cancer. It warrants further evaluation and should be considered an alternative to peri-operative ECF. J. Surg. Oncol. 2012; 105:793799. (c) 2011 Wiley Periodicals, Inc.
TC 6
ZB 4
Z8 5
ZS 0
Z9 10

SN 0022-4790
UT WOS:000303916400011
PM 22189752
ER

PT J
AU Zhang, Zhen
TI [Related problems of perioperative radiotherapy for gastric cancer].
SO Zhonghua wei chang wai ke za zhi = Chinese journal of gastrointestinal surgery
VL 15
IS 6
BP 546
EP 8
PD 2012-Jun
PY 2012
AB Radiotherapy has been an important component in the multidisciplinary treatment strategy of gastric cancer. INT0116 has showed the improvement of survival with adjuvant chemoradiation in resected gastric cancer. The benefit of adjuvant chemoradiation has been confirmed further in gastric cancer population. Recent reported ARTIST trial which all patients got D2 dissection has showed the benefit of adjuvant chemoradiation only seen in patients with lymph node positive. As the same observed in other two trail, ACT-GC and CLASSIC, extremely low local recurrence were reported in these trial. We should be very cautious when we interpret these results and treatment into our clinical practice due to the difference of local recurrence between trials and daily practice. Neoadjuvant radiation has been reported its effectiveness of cardiac gastric cancer in earlier randomized trial. Phase II(trials have shown the high pCR rate with neoadjuvant chemoradiation. However, concurrent chemoradiation was with more toxicity and limited it development. Even though, further investigation in neoadjuvant setting is worthy but with reduce of toxicity. New progress in high technique of radiation will help the application of radiotherapy in gastric cancer. The future of radiation in gastric cancer treatment will focus on the selection of patients which are of most benefit, detection of radiosensitivity and how to optimize combination of radiation with surgery and novel chemotherapy and target therapy.
TC 0

ZB 0
Z8 0
ZS 0
Z9 0
SN 1671-0274
UT MEDLINE:22736118
PM 22736118
ER

PT J
AU Chakravarty, Twisha
Crane, Christopher H.
Ajani, Jaffer A.
Mansfield, Paul F.
Briere, Tina M.
Beddar, A. Sam
Mok, Henry
Reed, Valerie K.
Krishnan, Sunil
Delclos, Marc E.
Das, Prajnan
TI Intensity-Modulated Radiation Therapy With Concurrent Chemotherapy as Preoperative Treatment for Localized Gastric Adenocarcinoma
SO INTERNATIONAL JOURNAL OF RADIATION ONCOLOGY BIOLOGY PHYSICS
VL 83
IS 2
BP 581
EP 586
DI 10.1016/j.ijrobp.2011.07.035
PD JUN 1 2012
PY 2012
AB Purpose: The goal of this study was to evaluate dosimetric parameters, acute toxicity, pathologic response, and local control in patients treated with preoperative intensity-modulated radiation therapy (IMRT) and concurrent chemotherapy for localized gastric adenocarcinoma.
Methods: Between November 2007 and April 2010, 25 patients with localized gastric adenocarcinoma were treated with induction chemotherapy, followed by preoperative IMRT and concurrent chemotherapy and, finally, surgical resection. The median radiation therapy dose was

45 Gy. Concurrent chemotherapy was 5-fluorouracil and oxaliplatin in 18 patients, capecitabine in 3, and other regimens in 4. Subsequently, resection was performed with total gastrectomy in 13 patients, subtotal gastrectomy in 7, and other surgeries in 5.
Results: Target coverage, expressed as the ratio of the minimum dose received by 99% of the planning target volume to the prescribed dose, was a median of 0.97 (range, 0.92-1.01). The median V-30 (percentage of volume receiving at least 30 Gy) for the liver was 26%; the median V-20 (percentage of volume receiving at least 20 Gy) for the right and left kidneys was 14% and 24%, respectively; and the median V-40 (percentage of volume receiving at least 40 Gy) for the heart was 18%. Grade 3 acute toxicity developed in 14 patients (56%), including dehydration in 10, nausea in 8, and anorexia in 5. Grade 4 acute toxicity did not develop in any patient. There were no significant differences in the rates of acute toxicity, hospitalization, or feeding tube use in comparison to those in a group of 50 patients treated with preoperative three-dimensional conformal radiation therapy with concurrent chemotherapy. R0 resection was obtained in 20 patients (80%), and pathologic complete response occurred in 5 (20%).
Conclusions: Preoperative IMRT for gastric adenocarcinoma was well tolerated, accomplished excellent target coverage and normal structure sparing, and led to appropriate pathologic outcomes. (C) 2012 Elsevier Inc.
TC 7
ZB 1
Z8 0
ZS 0
Z9 7
SN 0360-3016
UT WOS:000303920800039
PM 22137021
ER

PT J
AU Herskovic, A.
Russell, W.
Liptay, M.
Fidler, M. J.
Al-Sarraf, M.

TI Esophageal carcinoma advances in treatment results for locally advanced disease: review

SO ANNALS OF ONCOLOGY

VL 23

IS 5

BP 1095

EP 1103

DI 10.1093/annonc/mdr433

PD MAY 2012

PY 2012

AB The treatment results of patients with locally advanced esophageal carcinomas have evolved since the publication of the first trial of concurrent mitomycin C and 5-fluorouracil with radiotherapy (RT) in 1983. Subsequent studies refined and improved on the concurrent chemotherapy (chemo) with administration of cisplatin and 5-fluorouracil infusion (PF). Chemo (PF) before surgery improved overall survival (OS) in those patients in most of the randomized trials and in meta-analyses. Two courses of PF concurrent with irradiation followed by additional two courses of PF were superior to RT alone without surgery for both groups. Concurrent chemoradiotherapy followed by surgery was found to have statistically improved OS as compared with surgery only in randomized trials and meta-analyses. In most of these studies, it was found that those patients with pathologic complete response to the initial treatment(s) did better than those who had no improvement at all. Current treatment outcome for these diseases is disappointing; newer strategies including induction chemo with the optimal combination, proper dosage of each drug, and proper number of courses before concurrent chemoradiotherapy; improvement in RT; and immunotherapy with or without subsequent surgery are exciting and definitely need to be investigated in prospective randomized trial(s).

TC 8

ZB 3

Z8 5

ZS 0

Z9 13

SN 0923-7534

UT WOS:000303336400003

PM 22003242

ER

PT J

AU Lee, Duk Joo
Sohn, Tae Sung
Lim, Do Hoon
Ahn, Hee Kyung
Park, Se Hoon
Lee, Jeeyun
Park, Joon Oh
Park, Young Suk
Lim, Ho Yeong
Choi, Dong Il
Kim, Kyoung Mee
Choi, Min Gew
Noh, Jae Hyung
Bae, Jae Moon
Kim, Sung
Min, Byung Hoon
Kang, Won Ki

TI Phase I study of neoadjuvant chemoradiotherapy with S-1 and oxaliplatin in patients with locally advanced gastric cancer

SO CANCER CHEMOTHERAPY AND PHARMACOLOGY
VL 69
IS 5
BP 1333
EP 1338
DI 10.1007/s00280-012-1836-8
PD MAY 2012
PY 2012

AB The aim of this phase I study was to investigate the optimal dose of S-1 and oxaliplatin with concurrent radiotherapy in a preoperative setting for locally advanced gastric cancer.
Twelve patients with histologically confirmed clinical stage T2N+ or T3-T4 gastric adenocarcinoma received dose level -1 (oral S-1 at 60 mg/m(2)/day + oxaliplatin 40 mg/m(2) intravenously on days 1, 8, 15 and 22) or dose level 1 (S-1 80 mg/m(2)/day + oxaliplatin 40 mg/m(2)), with concurrent radiotherapy at daily fractions of 1.8 Gy 5 days per week, to a total dose of 41.4 Gy. Surgical resection, including D2 dissection, was performed within 4 weeks after the last day of chemotherapy.

Chemoradiotherapy was generally well tolerated, with the most common dose-related grade 1 or 2 adverse events being anemia, nausea, vomiting, anorexia and abdominal pain. Two DLTs (prolonged thrombocytopenia and stomach perforation) were observed at dose level 1 (n = 6) and resulted in dose de-escalation to level -1. The recommended dose for future study is dose level -1, at which 1 of 6 patients developed grade 3 vomiting and anorexia. R0 resection was possible in 11 patients. Pathologic down-staging was observed in 6 patients, including one complete response. No clinically relevant postoperative complications occurred. The activity of preoperative concurrent chemoradiotherapy with S-1 (60 mg/m(2)/day for 28 consecutive days) and oxaliplatin (40 mg/m(2) on days 1, 8, 15 and 22) will be explored more extensively in a phase II study in patients with locally advanced GC.
RI Lee, Jee Yun/C-9646-2011; Kim, Sung/G-4114-2014
TC 2
ZB 1
Z8 4
ZS 0
Z9 6
SN 0344-5704
UT WOS:000303418500025
PM 22311161
ER

PT J
AU Mori, Yoshihiro
Ohtani, Hiroshi
Tamamori, Yutaka
Inoue, Takeshi
Azuma, Takashi
TI [Two cases of stage IV gastric cancer undergoing adjuvant surgery after down staging by chemotherapy].
SO Gan to kagaku ryoho. Cancer & chemotherapy
VL 39
IS 4
BP 657
EP 61
PD 2012-Apr
PY 2012

AB CASE 1: A 72-year-old man with epigastralgia was diagnosed with gastric cancer and referred to our hospital. An abdominal CT scan revealed liver metastasis and para-aortic lymph node metastasis. He was treated with S-1+CDDP. After 4 courses of this treatment, the liver metastasis and para-aortic lymph node metastasis disappeared, and adjuvant surgery was performed. There has been no recurrence for 16 months postoperatively. CASE 2: A 66-year-old man with anorexia was diagnosed with gastric cancer and referred to our hospital. An abdominal CT scan revealed para-aortic lymph node metastasis. He was treated with S-1+CDDP. After 9 courses of this treatment, para-aortic lymph node metastasis disappeared, and adjuvant surgery was performed. Eight months after the operation, lymph node metastases were confirmed by abdominal CT scan, and he was treated with chemotherapy as an outpatient as of 13 months after the operation. We experienced two cases of Stage IV gastric cancer undergoing adjuvant surgery after down staging by chemotherapy. It was suggested that adjuvant surgery to highly advanced gastric cancer could improve the prognosis of patients.

TC 0

ZB 0

Z8 0

ZS 0

Z9 0

SN 0385-0684

UT MEDLINE:22504697

PM 22504697

ER

PT J

AU Homann, Nils
Pauligk, Claudia
Luley, Kim
Kraus, Thomas Werner
Bruch, Hans-Peter
Atmaca, Akin
Noack, Frank
Altmannsberger, Hans-Michael
Jaeger, Elke
Al-Batran, Salah-Eddin

TI Pathological complete remission in patients with oesophagogastric cancer

receiving preoperative 5-fluorouracil, oxaliplatin and docetaxel
SO INTERNATIONAL JOURNAL OF CANCER
VL 130
IS 7
BP 1706
EP 1713
DI 10.1002/ijc.26180
PD APR 1 2012
PY 2012
AB The aim of this study was to determine the pathological complete remission (pCR) rate, and its relationship to clinical outcome, in patients with adenocarcinoma of the stomach or oesophagogastric junction receiving preoperative 5-fluorouracil, leucovorin, oxaliplatin and docetaxel (FLOT) every 2 weeks. Data from these patients who received at least one cycle of preoperative FLOT followed by surgery were prospectively collected in three German centres. Outcome analyses were conducted and tumour samples were evaluated for pathological remission by a central pathologist. A total of 46 patients were included in this analysis. All patients had clinical T3- and/or N+-stages and 11 (23.9%) had distant metastases (M1). After a median of 4 (range 28) preoperative cycles, 8 of 46 patients (17.4%) achieved a pCR. The pCR rate was highest in tumours of intestinal type histology (30.8%) and in those located in the oesophagogastric junction (30.4%) and lowest in patients with diffuse/mixed type tumours (0%) or tumours located in the stomach (4.3%; $p < 0.05$ for both comparisons). Patients with pCR had 100% probability of overall and disease-free survival (DFS) during the observation period, which was significantly higher ($p = 0.037$ and $p = 0.009$, respectively) than the survival probability in patients without pCR. In conclusion, treatment intensification using FLOT was associated with significant pCR rates in patients with oesophagogastric cancer. The distribution of pCR appeared to be significantly different according to histological type and location of the tumours.
TC 15
ZB 5
Z8 0
ZS 0
Z9 15
SN 0020-7136
UT WOS:000299633300024

PM 21618509
ER

PT J
AU Li Guo-li
Liu Kun
Bao Yang
Cao Jian-ming
Xu Jian
Wang Xu-ling
Wu Bo
Li Jie-shou
TI Retrospective analysis of 56 patients with advanced gastric cancer treated with combination of intravenous and intra-arterial intensified neoadjuvant chemotherapy
SO CHINESE MEDICAL JOURNAL
VL 125
IS 5
BP 780
EP 785
DI 10.3760/cma.j.issn.0366-6999.2012.05.011
PD MAR 5 2012
PY 2012
AB Background Pre-operative chemotherapy has gained widespread interest while treating advanced gastric cancer in eastern countries. However, there is currently no established standard regimen for gastric cancer. The aim of this research was to explore the value of preoperative chemotherapy with a combination of intravenous and intra-arterial intensified chemotherapy in advanced gastric cancer.

Methods A total of 56 histologically proven gastric cancer patients, who were considered to be stage II or higher with metastatic lymph nodes and with or without distant metastasis (T2-4, N1-3, and M0-1), were treated with a neoadjuvant chemotherapy. Patients received a combination of intravenous 5-Fu (370 mg/m(2)) and leucovorin (200 mg/m(2)) on days 1-5, and intra-arterial etoposide (80 mg/m(2)) and cisplatin (80 mg/m(2)) on days 6 and 20. After two cycles of preoperative chemotherapy, patients with resectable tumors underwent laparotomy.

Results All patients finished two cycles of chemotherapy. The overall response rate was 78.57% (44 cases), of which 7.14% (four cases)

clinical complete response. Forty-six patients underwent resection, including 21 initially unresectable diseases. R0 resection rate for prechemotherapy resectable and unresectable diseases was 96.15% (25/26 cases) and 66.67% (20/30 cases), respectively. Pathological complete response was observed in 8.70% of patients. Toxicity was moderate and there were no chemotherapy-related deaths. With a median follow-up of 31 months (range 6-76 months), the 5-year survival rate for the whole group and patients with initially resectable tumors were 21.8% and 42.3%, respectively. The median survival for initially resectable and unresectable patients were 41 months (95%CI, 31.006-50.994) and 18 months (95`)CI, 13.399-22.601; P<0.01), respectively.
Conclusion Preliminary results proved that the combined intensive chemotherapy was a safe and promising regimen for pre-operative treatment of advanced gastric cancer. Chin Med J 2012;125(5):780-785
TC 2
ZB 1
Z8 4
ZS 0
Z9 5
SN 0366-6999
UT WOS:000301755200012
PM 22490574
ER

PT J
AU Lee, S. D.
Ryu, K. W.
Eom, B. W.
Lee, J. H.
Kook, M. C.
Kim, Y. -W.
TI Prognostic significance of peritoneal washing cytology in patients with gastric cancer
SO BRITISH JOURNAL OF SURGERY
VL 99
IS 3
BP 397
EP 403
DI 10.1002/bjs.7812

PD MAR 2012

PY 2012

AB Background: Positive peritoneal washing cytology is a poor prognostic factor in patients with gastric cancer. The right therapeutic approach for this condition has not been well documented.

Methods: Patients who underwent surgery for gastric cancer with suspected serosal invasion and peritoneal washing cytology at the Korean National Cancer Centre between May 2001 and December 2009 were included in this retrospective study. Clinicopathological factors and overall survival were analysed with respect to the cytological results and presence of peritoneal metastases. Prognostic factors were analysed in patients with positive cytology but without overt peritoneal metastases.

Results: A total of 1072 patients were included in the analysis, of whom 900 had negative cytology (C0 group) and 172 had positive cytology (C1 group). No peritoneal metastases (P0) were found in 830 patients (92.2 per cent) in the C0 group. Peritoneal metastases (P1) were found in 76 patients (44.2 per cent) in the C1 group. Median overall survival times in the P0 C1, P1 C0 and P1 C1 subgroups were 20.0, 14.0 and 10.0 months respectively. Multivariable analysis of the P0 C1 subgroup revealed that clinical N0-2 category and gastric resection were significantly associated with better prognosis (median survival 24.0 versus 13.0 months for N0-2 versus N3, and 21.0 versus 4.0 months for resected versus non-resected).

Conclusion: Positive washing cytology in patients with gastric cancer is a negative prognostic factor for patients with, as well as those without, overt peritoneal metastases. Resection is an option in patients with clinical stage N0-2 disease without peritoneal metastases but with a positive washing cytology finding.

TC 11

ZB 2

Z8 0

ZS 0

Z9 11

SN 0007-1323

UT WOS:000303148800018

PM 22101572

ER

PT J

AU Pera, M.
Gallego, R.
Montagut, C.
Martin-Richard, M.
Iglesias, M.
Conill, C.
Reig, A.
Balague, C.
Petriz, L.
Momblan, D.
Bellmunt, J.
Maurel, J.
TI Phase II trial of preoperative chemoradiotherapy with oxaliplatin, cisplatin, and 5-FU in locally advanced esophageal and gastric cancer
SO ANNALS OF ONCOLOGY
VL 23
IS 3
BP 664
EP +
DI 10.1093/annonc/mdr291
PD MAR 2012
PY 2012
AB Background: Based on a phase I study showing the feasibility of combining of oxaliplatin, cisplatin, and 5-fluorouracil (5-FU) (OCF) with radiation therapy (RT) in esophageal cancer, the efficacy of this regimen in esophageal, gastroesophageal (GE), and gastric (G) cancer was assessed in this phase II multicenter study.
Patients and methods: Patients with resectable tumors were eligible. Treatment included two cycles of oxaliplatin 85 mg/m(2), cisplatin 55 mg/m(2), and continuously infused 5-FU 3 g/m(2) in 96 h and concurrent RT (45 Gy), followed by surgery after 6-8 weeks. Primary end point was complete pathologic response (pCR).
Results: Forty-one patients were enrolled. Tumor location was esophagus 39% (squamous 10/adenocarcinoma 6), GE junction 32%, and stomach 29%. G3-G4 adverse events included asthenia (27%) and neutropenia (14%). One toxic death occurred. Thirty-one patients (75.6%) underwent surgery (R0 in 94%). Pathologic response was achieved in 58% of patients, with pCR in 50% and 16% of esophageal and GE/G cancer, respectively. pCR was achieved in 67% of squamous cell carcinoma. Survival: median follow-up,

50.4 months; median progression-free survival and overall survival were 23.2 and 28.4 months, respectively.
Conclusion: Preoperative OCF plus RT showed an acceptable toxicity and promising activity especially in squamous cell esophageal cancer.
TC 10
ZB 5
Z8 2
ZS 0
Z9 12
SN 0923-7534
UT WOS:000300733300019
PM 21652581
ER

PT J
AU Wang, Lin Bo
Teng, Rong Yue
Jiang, Zi Nong
Hu, Wen Xian
Dong, Min Jun
Yuan, Xiao Ming
Chen, Wen Jun
Jin, Mei
Shen, Jian Guo
TI Clinicopathologic variables predicting tumor response to neoadjuvant chemotherapy in patients with locally advanced gastric cancer
SO JOURNAL OF SURGICAL ONCOLOGY
VL 105
IS 3
BP 293
EP 296
DI 10.1002/jso.22085
PD MAR 2012
PY 2012
AB Background and Objectives To identify clinicopathologic variables that could predict pathologic tumor response to neoadjuvant chemotherapy for patients with locally advanced gastric cancer.
Methods: The study enrolled 108 patients who underwent neoadjuvant chemotherapy followed by surgery between July 2004 and December 2010.

Tumor responses to neoadjuvant chemotherapy were assessed in terms of tumor regression. Statistical analyses were performed to identify factors associated with pathologic tumor response.
Results: Tumor regression was found in 22.2% (24/108) patients, patients with tumor regression observed better overall survival as compared to that of patients without tumor regression. Univariate and multivariate analyses observed that both tumor differentiation and tumor size were independent predictors of tumor regression.
Conclusions: This study suggests that both tumor differentiation and tumor size is the most important clinical predicator of pathologic tumor response, it may be of benefit in the selection of treatment options in locally advanced gastric cancer. J. Surg. Oncol. 2012;105:293-296. (C) 2011 Wiley Periodicals, Inc.
TC 7
ZB 3
Z8 1
ZS 0
Z9 7
SN 0022-4790
UT WOS:000299374300015
PM 21882201
ER

PT J
AU Dipetrillo, Thomas
Suntharalingam, Mohan
Ng, Thomas
Fontaine, Jacques
Horiba, Naomi
Oldenburg, Nicklas
Perez, Kimberly
Birnbaum, Ari
Battafarano, Richard
Burrows, Whitney
Safran, Howard
TI Neoadjuvant Paclitaxel Poliglumex, Cisplatin, and Radiation for Esophageal Cancer A Phase 2 Trial
SO AMERICAN JOURNAL OF CLINICAL ONCOLOGY-CANCER CLINICAL TRIALS
VL 35

IS 1
BP 64
EP 67
DI 10.1097/COC.0b013e318201a126
PD FEB 2012
PY 2012
AB Purpose: To evaluate the pathologic complete response (CR) rate and safety of paclitaxel poliglumex (PPX), cisplatin, and concurrent radiation for patients with esophageal cancer.
Patients and Methods: Patients with adenocarcinoma or squamous cell carcinoma of the esophagus or gastroesophageal junction with no evidence of distant metastasis received PPX (50 mg/m(2)/wk) and cisplatin (25 mg/m(2)/wk) for 6 weeks with 50.4 Gy concurrent radiation. Six to eight weeks after completion of chemoradiotherapy, patients underwent surgical resection.
Results: Forty patients were enrolled, 37 patients with adenocarcinoma and 3 patients with squamous cell cancer. The treatment-related grade 3 nonhematologic toxicities included esophagitis (7%), nausea (7%), and fatigue (5%). Three patients with clinical endoscopic CR (2 with squamous cell cancer) refused surgery. Twelve of the remaining 37 patients (32%) had a pathologic CR. The 12 patients with pathologic CR all had adenocarcinoma.
Conclusion: PPX, cisplatin, and concurrent radiation are well tolerated, easily administered regimen for esophageal cancer with a low incidence of significant esophagitis and a high pathologic CR rate consistent with the preclinical data of PPX and radiation.
TC 5
ZB 6
Z8 2
ZS 0
Z9 7
SN 0277-3732
UT WOS:000299315400011
PM 21297434
ER

PT J
AU Nunobe, Souya
Kiyokawa, Takashi

Hatao, Fumihiko
Wada, Ikuo
Shimizu, Nobuyuki
Nomura, Sachiyo
Mise, Yoshihiro
Sugawara, Yasuhiko
Kokudo, Norihiro
Seto, Yasuyuki
TI Pathological Complete Response of Synchronous Multiple Liver Metastases Associated with Advanced Gastric Cancer to Gastrectomy and Prompt S-1 Treatment in Combination with Fractional Cisplatin: Report of a Case
SO HEPATO-GASTROENTEROLOGY
VL 59
IS 113
BP 307
EP 309
DI 10.5754/hge10238
PD JAN-FEB 2012
PY 2012
AB Systemic chemotherapy is the treatment recommended for prolonged survival in cases of metastatic gastric cancer. There have been a number of clinical reports of surgical resection of liver metastasis in selected patients with gastric cancer. Here, we report on a case of treatment of far advanced gastric cancer with synchronous multiple liver metastases with prompt S-1 in combination with fractional cisplatin sandwiched between two-stage surgery. Metastases including peritoneal dissemination and extensive lymph node involvement were absent so it was feasible to completely remove all of the macroscopic liver metastases. Each step of the chemotherapy progressed satisfactorily and histological examination after the hepatectomy yielded a pathologically complete response of liver metastases from the gastric cancer. This strategy provides a promising treatment for far advanced gastric cancer with a limited number of synchronous liver metastases. The referral to surgical oncology is a crucial step for the documentation of pathological complete response.
TC 0
ZB 0
Z8 0
ZS 0

Z9 0
SN 0172-6390
UT WOS:000301397300072
PM 22251551
ER

PT J
AU Yonemura, Yutaka
Elnemr, Ayman
Endou, Yoshio
Ishibashi, Haruaki
Mizumoto, Akiyoshi
Miura, Masahiro
Li, Yan
TI Effects of neoadjuvant intraperitoneal/systemic chemotherapy (bidirectional chemotherapy) for the treatment of patients with peritoneal metastasis from gastric cancer.
SO International journal of surgical oncology
VL 2012
BP 148420
EP 148420
DI 10.1155/2012/148420
PD 2012
PY 2012
AB Novel multidisciplinary treatment combined with neoadjuvant intraperitoneal-systemic chemotherapy protocol (NIPS) and peritonectomy was developed. Ninety-six patients were enrolled. Peritoneal wash cytology was performed before and after NIPS through a port system. Patients were treated with 60mg/m(2) of oral S-1 for 21 days, followed by a 1-week rest. On days 1, 8, and 15, 30mg/m(2) of Taxotere and 30mg/m(2) of cisplatin with 500mL of saline were introduced through the port. NIPS is done 2 cycles before surgery. Three weeks after NIPS, 82 patients were eligible to intend cytoreductive surgery (CRS) by gastrectomy + D2 dissection + periotnectomy to achieve complete cytoreduction. Sixty-eight patients showed positice cytology before NIPS, and the positive cytology results became negative in 47 (69%) patients after NIPS. Complete pathologic response on PC after NIPS was experienced in 30 (36.8%) patients. Stage migration was experienced in 12 patients (14.6%). Complete cytoreduction was achieved in 58 patients

(70.7%). By the multivariate analysis, complete cytoreduction and pathologic response became a significantly good survival. However the high morbidity and mortality, stringent patient selection is important. The best indications of the therapy are patients with good pathologic response and PCI ≤ 6, which are supposed to be removed completely by peritonectomy.

TC 1

ZB 0

Z8 2

ZS 0

Z9 2

UT MEDLINE:22900159

PM 22900159

ER

PT J

AU Sterzing, Florian
Grenacher, Lars
Debus, Jurgen

TI Radiotherapy of gastroesophageal junction cancer.

SO Recent results in cancer research. Fortschritte der Krebsforschung. Progres dans les recherches sur le cancer

VL 196

BP 187

EP 99

DI 10.1007/978-3-642-31629-6_13

PD 2012

PY 2012

AB Adenocarcinomas of the gastroesophageal junction (GEJ) require multimodal treatment approaches to accomplish good local control and overall survival. While early T1/2 N0 tumors are treated with surgery alone, they are only found in a small subset of patients due to the lack of symptoms at this stage. Most of the tumors are detected in locally advanced stage where surgery alone results in disappointing outcome. Chemotherapy and/or chemoirradiation in the neoadjuvant setting are used to improve conditions for oncological surgery. They aim to achieve a downsizing with a pathological complete remission in the optimal case, improve R0 rates, and upfront treat microscopic metastatic tumor cells. The optimal neoadjuvant treatment approach-chemotherapy,

chemoirradiation, or a multiphase approach of both-is yet unclear. Chemoirradiation can improve local control after incomplete surgery and is an important option for patients unfit for surgery. In addition, it enables symptom relief in a palliative setting, namely dysphagia, pain, or bleeding. While target volumes are very much standardized, new technologies as image-guided intensity-modulated radiotherapy (IG-IMRT) and particle therapy have the potential to improve the therapeutic window by minimizing toxicity. Challenges of the present and the future will be the combination of radiotherapy with other cytostatic drugs and modern targeted therapies. This should ideally be integrated into a multimodal setting that is able to identify risk groups according to predictive markers and tumor response, altogether leading to a personalized oncological approach.
TC 0
ZB 0
Z8 0
ZS 0
Z9 0
SN 0080-0015
UT MEDLINE:23129375
PM 23129375
ER

PT J
AU Choong, Nicholas W.
Mauer, Ann M.
Haraf, Daniel C.
Ferguson, Mark K.
Sandler, Alan B.
Kesler, Kenneth A.
Fishkin, Paul A. S.
Ansari, Rafat H.
Wade, James, III
Krauss, Stuart A.
Sciortino, David F.
Posner, Mitchell C.
Kocherginsky, Masha
Hoffman, Philip C.
Szeto, Livia

Vokes, Everett E.
TI Long-term outcome of a phase II study of docetaxel-based multimodality chemoradiotherapy for locally advanced carcinoma of the esophagus or gastroesophageal junction
SO MEDICAL ONCOLOGY
VL 28
BP S152
EP S161
DI 10.1007/s12032-010-9658-1
SU 1
PD DEC 2011
PY 2011
AB We performed a phase II trial to evaluate a docetaxel-based regimen in locoregionally advanced esophageal cancer. Untreated stage II-IVa esophageal cancer patients with performance status 0-2 were included. Tumor resectability was determined prior to initiation of study. Induction docetaxel (75 mg/m(2)) and cisplatin (75 mg/m(2)) day 1 with prophylactic filgrastim was delivered every 21 days for 3 cycles. Subsequent concomitant chemoradiotherapy (CRT) utilized weekly docetaxel (20 mg/m(2)) and concurrent radiotherapy (2 Gy/day) in resectable/resected patients (50 Gy) and in unresectable patients (66 Gy). A total of 78 patients (15 squamous cell carcinoma, 60 adenocarcinoma, 3 mixed/undifferentiated; 68 men, 10 women; median age 61 years) were accrued. The regimen was administered to 59 (76%) potentially resectable patients and 13 (17%) unresectable patients; 6 patients (8%) received the regimen post-operatively. Response rate in 66 evaluable patients following induction chemotherapy was 30%. Sixty-nine patients underwent CRT. Ten patients had disease progression during CRT. Forty-five out of 59 potentially resectable patients underwent esophagectomy after CRT, and 42 patients had complete tumor resection with negative margins. Eighteen out of 59 patients who were potentially resectable patients had pathologic complete response (pCR-31%). Grade 3/4 toxicity during induction chemotherapy included leucopenia, neutropenia, vomiting, and neuropathy. Esophagitis was the predominant toxicity during CRT. Median overall survival was 11.4 months for unresectable patients, 14.3 months for resectable patients and 10.4 months for patients who received the regimen post-operatively (log-rank P = 0.2492). Docetaxel-based CRT regimen is active and tolerable in esophageal cancer. The observed pCR in the potentially resectable group

indicates good local control.
TC 2
ZB 0
Z8 0
ZS 0
Z9 2
SN 1357-0560
UT WOS:000301047200022
PM 20730572
ER

PT J
AU Hingorani, M.
Crosby, T.
Maraveyas, A.
Dixit, S.
Bateman, A.
Roy, R.
TI Neoadjuvant Chemoradiotherapy for Resectable Oesophageal and
Gastro-oesophageal Junction Cancer-Do We Need Another Randomised Trial?
SO CLINICAL ONCOLOGY
VL 23
IS 10
BP 696
EP 705
DI 10.1016/j.clon.2011.05.005
PD DEC 2011
PY 2011
AB Aims: The optimal neoadjuvant therapy option for locally advanced
oesophageal cancer remains elusive. Neoadjuvant chemoradiotherapy (CRT)
is the preferred modality of choice in the USA. In contrast, neoadjuvant
chemotherapy is commonly used in the UK. We provide a comprehensive
overview of the available evidence for defining the ideal neoadjuvant
treatment algorithm.
Materials and methods: The PubMed database combined with American
Society of Clinical Oncology and American Society for Therapeutic
Radiology and Oncology websites were searched online to identify
randomised studies and published meta-analyses that have compared these
modalities compared with surgery alone. In particular, we searched for

randomised trials that may have directly compared outcomes after
neoadjuvant CRT or chemotherapy.
Results: We identified 17 published randomised studies of neoadjuvant CRT (n = 9) and chemotherapy (n = 8) compared with surgery alone and one prospective series that compared the above modalities against each other. Studies evaluating CRT have reported pathological complete response rates of 15-40% and no increase in postoperative mortality was observed, except in one study that used a hypofractionated radiation schedule. Two randomised studies showed significant survival benefit and the remaining (n = 7) were negative, but showed a trend towards improved survival. Furthermore, at least four meta-analyses have shown improved survival in favour of CRT extending up to an absolute benefit of 13% at 2 years. In comparison, five studies of neoadjuvant chemotherapy showed no survival difference and two of the remaining studies that showed significant benefit included gastric adenocarcinomas and used pen-operative chemotherapy. All the above studies have shown uniformly poor pathological complete response rates of less than 10 percent. Moreover, three meta-analyses were negative, but two showed up to 7% absolute survival benefit at 2 years in favour of chemotherapy. The trial comparing the above modalities showed a trend towards improved survival in favour of CRT, but closed early due to poor recruitment.
Conclusion: Data from the above studies are potentially conflicting and inconclusive for defining the optimal neoadjuvant treatment schedule. In our opinion, the above question can only be answered within the context of a randomised control trial. We have included a proposal for a trial design for direct comparison of these modalities. (C) 2011 The Royal College of Radiologists. Published by Elsevier Ltd. All rights reserved.
TC 7
ZB 3
Z8 4
ZS 0
Z9 9
SN 0936-6555
UT WOS:000297495400008
PM 21684129
ER

PT J
AU Shih, Alan H.

Chung, Stephen S.
Dolezal, Emily K.
Zhang, Su-Jiang
Abdel-Wahab, Omar
Park, Christopher Y.
Nimer, Stephen D.
Levine, Ross L.
Klimek, Virginia M.
TI Mutational Analysis of Therapy-Related MDS/AML
SO BLOOD
VL 118
IS 21
BP 1208
EP 1209
PD NOV 18 2011
PY 2011
CT 53rd Annual Meeting and Exposition of the American-Society-of-Hematology (ASH)/Symposium on the Basic Science of Hemostasis and Thrombosis
CY DEC 10-13, 2011
CL San Diego, CA
SP Amer Soc Hematol (ASH)
TC 0
ZB 0
Z8 0
ZS 0
Z9 0
SN 0006-4971
UT WOS:000299597104153
ER

PT J
AU Sun, X-C
Lin, J.
Ju, A-H
TI Treatment of Borrmann Type IV Gastric Cancer with a Neoadjuvant Chemotherapy Combination of Docetaxel, Cisplatin and 5-Fluorouracil/Leucovorin
SO JOURNAL OF INTERNATIONAL MEDICAL RESEARCH
VL 39

IS 6
BP 2096
EP 2102
PD NOV-DEC 2011
PY 2011
AB This study evaluated the efficacy and safety of docetaxel, cisplatin and 5-fluorouracil/leucovorin as neoadjuvant chemotherapy before surgery (NCT group; n = 29) compared with postoperative chemotherapy alone (non-NCT group; n =26) in the treatment of Borrmann type IV gastric carcinoma. Primary tumour response rate, surgical parameters, incised-edge residue rate, lymphatic metastasis status and side-effects were evaluated. The overall response rate was 58.6% in the NCT group, which included three (10.3%) patients in complete remission and 14 (48.3%) patients in partial remission. The postoperative pathological complete response rate was 6.9% (two patients) in the NCT group. NCT was associated with a significant increase in the radical resection rate and a significant decrease in the rate of incised-edge residues, compared with postoperative chemotherapy alone. Side-effects due to NCT were minimal and resolved with appropriate treatment. There were no chemotherapy-related deaths in either group. In conclusion, docetaxel, cisplatin and 5-fluorouracil/leucovorin was an effective and well-tolerated NCT regimen for Borrmann type IV gastric cancer.
TC 2
ZB 1
Z8 4
ZS 0
Z9 6
SN 0300-0605
UT WOS:000299916500005
PM 22289524
ER

PT J
AU Kobayashi, Kenji
Tanizaki, Keiko
Aoki, Taro
Takachi, Kou
Nishioka, Kiyonori
Matsumoto, Takashi

Komori, Takamichi
Chono, Teruhiro
Kato, Aya
Hyuga, Satoshi
Watanabe, Risa
Uemura, Yoshio
TI [A case of S-1/CDDP chemotherapy for inoperable advanced gastric cancer which led to gastrectomy with histological complete response].
SO Gan to kagaku ryoho. Cancer & chemotherapy
VL 38
IS 12
BP 1951
EP 3
PD 2011-Nov
PY 2011
AB As the treatment for inoperable advanced gastric cancer, S-1/CDDP combination therapy (SP chemotherapy) has become a standard treatment. In our hospital, a second course of chemotherapy was performed on an outpatient basis in order to improve a traditional QOL. In this case, it showed remarkable effects in 15 months after starting chemotherapy. Then gastrectomy was performed. Histological findings of the resected specimens confirmed pCR in all tumors. We report on progress of this case and explain about the ingenuity of SP chemotherapy.
TC 0
ZB 0
Z8 0
ZS 0
Z9 0
SN 0385-0684
UT MEDLINE:22202249
PM 22202249
ER

PT J
AU Sbitti, Yassir
Essaidi I, Ismail
Debbagh, Adil
Kadiri, Habiba
Oukabli, Mohamed

Moussaid, Yassine
Slimani, Khaoula
Fetohi, Mohamed
Elkaoui, Hakim
Albouzidi, Abderrahmane
Mahi, Mohamed
Ali, Abdelmounaim Ait
Ichou, Mohamed
Errihani, Hassan
TI Is there any advantage to combined trastuzumab and chemotherapy in perioperative setting her 2neu positive localized gastric adenocarcinoma?
SO WORLD JOURNAL OF SURGICAL ONCOLOGY
VL 9
AR 112
DI 10.1186/1477-7819-9-112
PD SEP 28 2011
PY 2011
AB We report here a 44-year-old Moroccan man with resectable gastric adenocarcinoma with overexpression of human epidermal growth factor receptor 2 (HER2) by immunohistochemistry who was treated with trastuzumab in combination with chemotherapy in perioperative setting. He received 3 cycles of neoadjuvant chemotherapy consisting of trastuzumab, oxaliplatin, and capecitabine. Afterwards, he received total gastrectomy with extended D2 lymphadenectomy without spleno-pancreatectomy. A pathologic complete response was obtained with a combination of trastuzumab and oxaliplatin and capecitabine. He received 3 more cycles of trastuzumab containing regimen postoperatively.
We conclude that resectable gastric carcinoma with overexpression of the c-erbB-2 protein should ideally be managed with perioperative combination of trastuzumab with chemotherapy. Further research to evaluate trastuzumab in combination with chemotherapy regimens in the perioperative and adjuvant setting is urgently needed.
TC 8
ZB 3
Z8 0
ZS 0
Z9 8

SN 1477-7819
UT WOS:000296744200001
PM 21955806
ER

PT J
AU Higuchi, Mitsunori
Suzuki, Hiroyuki
Shio, Yutaka
Satoh, Yoichi
Kitamura, Masatoshi
Gotoh, Mitsukazu
TI [A case of long-term survival after resection of aortic arch for locally advanced non-small cell lung cancer with induction chemotherapy].
SO Gan to kagaku ryoho. Cancer & chemotherapy
VL 38
IS 8
BP 1349
EP 52
PD 2011-Aug
PY 2011
AB Our patient was a 57-year-old male with a history of esophageal cancer. He was referred to our hospital for squamous cell lung carcinoma(SCC). Chest computed tomography identified a mass in the left lung field, which was suspected to be invading the reconstructed gastric tube, left subclavian artery, common carotid artery, and distal aortic arch. He was diagnosed as primary pulmonary squamous cell carcinoma(SCC)because six years had already passed since a previous surgery for early esophageal cancer. He received three courses of induction chemotherapy including S-1/CDDP. We evaluated the therapy as a partial response. He underwent an extended resection of distal aortic arch and left subclavian artery with left upper lobectomy, and those vessels were reconstructed using prosthetic grafts. Pathological findings showed the tumor as a well differentiated SCC of pT4N0M0 at stage III A, with a residual tumor on the reconstructed gastric tube, even though the effect of induction chemotherapy was Ef2. He received three courses of S-1/CDDP after surgery. The patient has been well without recurrence for 31 months after surgery.
TC 0

ZB 0
Z8 0
ZS 0
Z9 0
SN 0385-0684
UT MEDLINE:21829079
PM 21829079
ER

PT J
AU Diaz-Gonzalez, Juan A.
Rodriguez, Javier
Hernandez-Lizoain, Jose L.
Ciervide, Raquel
Gaztanaga, Miren
San Miguel, Inigo
Arbea, Leire
Javier Aristu, J.
Chopitea, Ana
Martinez-Regueira, Fernando
Valenti, Victor
Garcia-Foncillas, Jesus
Martinez-Monge, Rafael
Sola, Jesus J.
TI PATTERNS OF RESPONSE AFTER PREOPERATIVE TREATMENT IN GASTRIC CANCER
SO INTERNATIONAL JOURNAL OF RADIATION ONCOLOGY BIOLOGY PHYSICS
VL 80
IS 3
BP 698
EP 704
DI 10.1016/j.ijrobp.2010.02.054
PD JUL 1 2011
PY 2011
AB Purpose: To analyze the rate of pathologic response in patients with locally advanced gastric cancer treated with preoperative chemotherapy with and without chemoradiation at our institution.
Methods and Materials: From 2000 to 2007 patients were retrospectively identified who received preoperative treatment for gastric cancer (cT3-4/ N+) with induction chemotherapy (Ch) or with Ch followed by

concurrent chemoradiotherapy (45 Gy in 5 weeks) (ChRT). Surgery was planned 4-6 weeks after the completion of neoadjuvant treatment. Pathologic assessment was used to investigate the patterns of pathologic response after neoadjuvant treatment.
Results: Sixty-one patients were analyzed. Of 61 patients, 58 (95%) underwent surgery. The R0 resection rate was 87%. Pathologic complete response was achieved in 12% of the patients. A major pathologic response (< 10% of residual tumor) was observed in 53% of patients, and T downstaging was observed in 75%. Median follow-up was 38.7 months. Median disease-free survival (DFS) was 36.5 months. The only patient-, tumor-, and treatment-related factor associated with pathologic response was the use of preoperative ChRT. Patients achieving major pathologic response had a 3-year actuarial DFS rate of 63%.
Conclusions: The patterns of pathologic response after preoperative ChRT suggest encouraging intervals of DFS. Such a strategy may be of interest to be explored in gastric cancer. (C) 2011 Elsevier Inc.
TC 3
ZB 0
Z8 0
ZS 0
Z9 3
SN 0360-3016
UT WOS:000291711700009
PM 20656414
ER

PT J
AU Fields, R. C.
Strong, V. E.
Goenen, M.
Goodman, K. A.
Rizk, N. P.
Kelsen, D. P.
Ilson, D. H.
Tang, L. H.
Brennan, M. F.
Coit, D. G.
Shah, M. A.
TI Recurrence and survival after pathologic complete response to

preoperative therapy followed by surgery for gastric or gastrooesophageal adenocarcinoma

SO BRITISH JOURNAL OF CANCER
VL 104
IS 12
BP 1840
EP 1847
DI 10.1038/bjc.2011.175
PD JUN 7 2011
PY 2011

AB BACKGROUND: To characterise recurrence patterns and survival following pathologic complete response (pCR) in patients who received preoperative therapy for localised gastric or gastrooesophageal junction (GEJ) adenocarcinoma.

METHODS: A retrospective review of a prospective database identified patients with pCR after preoperative chemotherapy for gastric or preoperative chemoradiation for GEJ (Siewert II/III) adenocarcinoma. Recurrence patterns, overall survival, recurrence-free survival, and disease-specific survival were analysed.

RESULTS: From 1985 to 2009, 714 patients received preoperative therapy for localised gastric/GEJ adenocarcinoma, and 609 (85%) underwent a subsequent R0 resection. There were 60 patients (8.4%) with a pCR. Median follow-up was 46 months. Recurrence at 5 years was significantly lower for pCR vs non-pCR patients (27% and 51%, respectively, P = 0.01). The probability of recurrence for patients with pCR was similar to non-pCR patients with pathologic stage I or II disease. Although the overall pattern of local/regional (LR) vs distant recurrence was comparable (43% LR vs 57% distant) between pCR and non-pCR groups, there was a significantly higher incidence of central nervous system (CNS) first recurrences in pCR patients (36 vs 4%, P = 0.01).

CONCLUSION: Patients with gastric or GEJ adenocarcinoma who achieve a pCR following preoperative therapy still have a significant risk of recurrence and cancer-specific death following resection. One third of the recurrences in the pCR group were symptomatic CNS recurrences. Increased awareness of the risk of CNS metastases and selective brain imaging in patients who achieve a pCR following preoperative therapy for gastric/GEJ adenocarcinoma is warranted. British Journal of Cancer (2011) 104, 1840-1847. doi:10.1038/bjc.2011.175 www.bjcancer.com
Published online 24 May 2011 (C) 2011 Cancer Research UK

RI Gonen, Mithat/E-4826-2012
TC 15
ZB 7
Z8 1
ZS 0
Z9 16
SN 0007-0920
UT WOS:000291384700007
PM 21610705
ER

PT J
AU Kang, Yoon-Koo
Ryu, Min-Hee
Yoo, Changhoon
Chang, Heung-Moon
Yook, Jeong Hwan
Oh, Sung Tae
Kim, Byung Sik
Kim, Tae Won
TI Phase I/II study of a combination of docetaxel, capecitabine, and cisplatin (DXP) as first-line chemotherapy in patients with advanced gastric cancer
SO CANCER CHEMOTHERAPY AND PHARMACOLOGY
VL 67
IS 6
BP 1435
EP 1443
DI 10.1007/s00280-010-1444-4
PD JUN 2011
PY 2011
AB This study was conducted to determine the optimal dosage of the docetaxel-capecitabine-cisplatin (DXP) regimen and to evaluate its efficacy and safety in patients with advanced gastric cancer.
Patients with advanced gastric or esophagogastric junctional adenocarcinoma received capecitabine (days 1-14) and intravenous docetaxel and cisplatin (day 1) every 3 weeks.
In the phase I study, 15 patients were treated with 4 different dose levels. Asthenia and neutropenic fever were the dose-limiting

toxicities. For the phase II study, 1,125 mg/m(2) of capecitabine was
initially recommended with 60 mg/m(2) docetaxel and 60 mg/m(2)
cisplatin. However, frequent dose modifications at this dose level
resulted in a final optimal dose of 937.5 mg/m(2) capecitabine. Among
the 40 patients enrolled in the phase II study, 4 complete and 23
partial responses were observed, presenting objective response rate of
68%. Ten patients achieving good response with complete disappearance of
distant metastases underwent surgery, and 4 pathologic complete
responses were identified. After the median follow-up of 83.7 months
(range, 20.2-86.5) in surviving patients, the median overall survival
was 14.4 months and median progression-free survival was 7.6 months. The
most frequent grade 3/4 adverse events were neutropenia (62.5%) and
asthenia (37.5%). Ten per cent of the patients experienced neutropenic
fever, with one case of sepsis-induced death.
DXP displays considerable antitumor activity, and may thus present
effective first-line treatment for advanced gastric cancer. Further
investigation of the efficacy and safety of this regimen in both
first-line and neoadjuvant settings is warranted.
TC 9
ZB 5
Z8 2
ZS 0
Z9 11
SN 0344-5704
UT WOS:000291036500024
PM 20811894
ER

PT J
AU Altorki, Nasser K.
Christos, Paul
Port, Jeff L.
Lee, Paul C.
Mirza, Farooq
Spinelli, Cathy
Keresztes, Roger
Beneck, Debra
Paul, Subroto
Stiles, Brendon M.

Zhang, Yuwei
Schrump, David S.
TI Preoperative Taxane-Based Chemotherapy and Celecoxib for Carcinoma of the Esophagus and Gastroesophageal Junction Results of a Phase 2 Trial
SO JOURNAL OF THORACIC ONCOLOGY
VL 6
IS 6
BP 1121
EP 1127
DI 10.1097/JTO.0b013e31821529a9
PD JUN 2011
PY 2011
AB Purpose: The primary objective of this study was to determine the rate of pathological response after preoperative celecoxib and concurrent taxane-based chemotherapy in patients with cancer of the esophagus and gastroesophageal junction.
Methods: Thirty-nine patients were enrolled in this single-arm, phase II clinical trial. Patients were administered daily celecoxib in combination with two to three cycles of carboplatin and paclitaxel with preoperative intent. Levels of cyclooxygenase (COX)-2 expression in resected tumors were analyzed by immunohistochemistry and correlated with clinical outcome measures. Postoperatively, patients were administered daily celecoxib for 1 year or until documented tumor recurrence.
Results: All patients received two to three cycles of chemotherapy plus celecoxib 800 mg/d. Toxicities were as expected. A major clinical response (complete response + partial response) was noted in 22 patients (56%); six patients (15%) had a complete clinical response. Thirty-seven patients underwent esophagectomy. Five patients had a major pathological response (12.8%). Four-year overall and disease-free survivals were 40.9% and 30.3%, respectively. Patients with tumors expressing COX-2 demonstrated a higher likelihood of a major clinical response response (62% versus 50%) and an improved overall survival, compared with patients with COX-2-negative tumors.
Conclusions: Preoperative celecoxib with concurrent chemotherapy demonstrated sufficient effect on pathologic response to warrant further study. Patients with tumors expressing COX-2 demonstrated trends toward improved response to preoperative therapy and improved overall survival compared with nonexpressors.

TC 10
ZB 5
Z8 2
ZS 0
Z9 12
SN 1556-0864
UT WOS:000290766400022
PM 21532508
ER

PT J
AU Janjigian, Yelena Y.
Tang, Laura H.
Coit, Daniel G.
Kelsen, David P.
Francone, Todd D.
Weiser, Martin R.
Jhanwar, Suresh C.
Shah, Manish A.
TI MET Expression and Amplification in Patients with Localized Gastric
Cancer
SO CANCER EPIDEMIOLOGY BIOMARKERS & PREVENTION
VL 20
IS 5
BP 1021
EP 1027
DI 10.1158/1055-9965.EPI-10-1080
PD MAY 2011
PY 2011
AB Background: MET, the receptor for hepatocyte growth factor, has been
proposed as a therapeutic target in gastric cancer. This study assessed
the incidence of MET expression and gene amplification in tumors of
Western patients with gastric cancer.
Methods: Tumor specimens from patients enrolled on a preoperative
chemotherapy study (NCI5700) were examined for the presence of MET gene
amplification by FISH, MET mRNA expression by quantitative PCR, MET
overexpression by immunohistochemistry (IHC), and for evidence of MET
pathway activation by phospho-MET (p-MET) IHC.
Results: Although high levels of MET protein and mRNA were commonly

encountered (in 63% and 50% of resected tumor specimens, respectively), none of these tumors had MET gene amplification by FISH, and only 6.6% had evidence of MET tyrosine kinase activity by p-MET IHC.
Conclusions: In this cohort of patients with localized gastric cancer, the presence of high MET protein and RNA expression does not correlate with MET gene amplification or pathway activation, as evidenced by the absence of amplification by FISH and negative p-MET IHC analysis.
Impact: This article shows a lack of MET amplification and pathway activation in a cohort of 38 patients with localized gastric cancer, suggesting that MET-driven gastric cancers are relatively rare in Western patients. Cancer Epidemiol Biomarkers Prev; 20(5); 1021-27. (C) 2011 AACR.
TC 37
ZB 24
Z8 1
ZS 0
Z9 38
SN 1055-9965
UT WOS:000290251000035
PM 21393565
ER

PT J
AU Becker, Karen
Langer, Rupert
Reim, Daniel
Novotny, Alexander
zum Buschenfelde, Christian Meyer
Engel, Jutta
Friess, Helmut
Hofler, Heinz
TI Significance of Histopathological Tumor Regression After Neoadjuvant Chemotherapy in Gastric Adenocarcinomas A Summary of 480 Cases
SO ANNALS OF SURGERY
VL 253
IS 5
BP 934
EP 939
DI 10.1097/SLA.0b013e318216f449

PD MAY 2011

PY 2011

AB Objective: An increasing number of patients with locally advanced gastric carcinomas (GC) are being treated with preoperative chemotherapy before surgery.

Background: Histopathological tumor regression may have an important prognostic impact in addition to the UICC-TNM classification system.

Methods: We evaluated the histopathological tumor regression in 480 surgical resection specimens of GC after neoadjuvant cisplatin-based chemotherapy, using an established system encompassing three tumor regression grades based on the estimation of the percentage of residual tumor tissue at the primary tumor site in relation to the macroscopically identifiable former tumor bed. Tumor regression was correlated to clinicopathological characteristics and patient survival.

Results: Of the patients in this study, 102 (21.2%) had complete or subtotal tumor regression (< 10% residual tumor), 121 (25.2%) had partial tumor regression (10-50% residual tumor), and 257 (53.5%) had minimal or no regression (> 50% residual tumor). Tumor regression was significantly associated with posttreatment tumor category (pT), lymph node status (pN), lymphatic invasion status (pL), and resection status (P < 0.001). Major histopathological regression was less frequent in tumors of the distal stomach and tumors of nonintestinal type (P = 0.003). Tumor regression (P = 0.009) and postoperative Lymph node status (P < 0.001) were independent prognostic factors for survival in a multivariate analysis of tumor regression, ypT/N/L category, resection status, grading and Lauren's classification.

Conclusions: Assessment of histological tumor regression after preoperative chemotherapy in GC provides objective and highly valuable prognostic information in addition to posttherapeutic lymph node status. A standardized tumor regression grading system should be implemented in pathological reports of these tumors.

RI Reim, Daniel/J-3115-2013

TC 33

ZB 16

Z8 1

ZS 0

Z9 34

SN 0003-4932

UT WOS:000289510600014

PM 21490451
ER

PT J
AU Zhao, Xia
Yang, Weihua
Shi, Changwen
Ma, Wanshan
Liu, Jianing
Wang, Yunshan
Jiang, Guosheng
TI The G1 phase arrest and apoptosis by intrinsic pathway induced by valproic acid inhibit proliferation of BGC-823 gastric carcinoma cells
SO TUMOR BIOLOGY
VL 32
IS 2
BP 335
EP 346
DI 10.1007/s13277-010-0126-5
PD APR 2011
PY 2011
AB Recent studies have demonstrated that the histone deacetylation level was closely related to the genesis and development of tumors. Thus, activating histone acetyltransferases and/or suppressing histone deacetylases (HDACs) can become an approach for tumor chemotherapy. The histone acetylation regulation often results in the inhibition of cell proliferation, induction of cell apoptosis or differentiation, and cell cycle arrest in G1 phase. It has been demonstrated recently that the traditional anticonvulsant valproic acid was an efficient class I HDAC inhibitor (HDACI); however, its antitumor effect and mechanisms on gastric cancers so far has not been elucidated clearly. In the present study, gastric carcinoma cell lines BGC-823, HGC-27, and SGC-7901 were cultured with valproic acid (VPA) in vitro. The cell morphology was observed by invert microscope, the proliferation was detected by MTT assay, the apoptosis and cell cycle were analyzed by flow cytometry assay with Annexin V/PI and PI, the activities and protein expressions of Caspase 3, Caspase 8, Caspase 9 of BGC-823 cells were detected by spectrophotometry and indirect immunofluorescence technique, respectively. The protein expressions of Cyclin A, Cyclin D1, Cyclin E,

P21(Waf/cip1) of BGC-823 cells were analyzed by indirect immunofluorescence assay, and messenger ribonucleic acid (mRNA) expressions were detected by RT-PCR assay. The results showed that the proliferation of three kinds of gastric carcinoma cells could be inhibited obviously by VPA, which was related to the apoptosis induction and cell cycle arrest in G1 phase. The intrinsic pathway (cytochrome C pathway) was chiefly involved in the mechanism of apoptosis, which was indicated by activation of Caspase 9 and Caspase 3. The extrinsic pathway was partially involved, with slight activation of Caspase 8. The mechanism underlying its effect on cell cycle arrest in G1 phase induction was due to the upregulation of P21(Waf/cip1), Mad1 expression and downregulation of Cyclin A, c-Myc expression.
TC 10
ZB 9
Z8 2
ZS 0
Z9 12
SN 1010-4283
UT WOS:000290572600011
PM 21113745
ER

PT J
AU Rao, Sheela
Welsh, Lyndsey
Cunningham, David
Te-Poele, Robert H.
Benson, Martin
Norman, Andrew
Saffery, Claire
Giddings, Ian
Workman, Paul
Clarke, Paul A.
TI Correlation of Overall Survival With Gene Expression Profiles in a Prospective Study of Resectable Esophageal Cancer
SO CLINICAL COLORECTAL CANCER
VL 10
IS 1
BP 48

EP 56
DI 10.3816/CCC.2011.n.007
PD MAR 2011
PY 2011
AB Purpose: Preoperative chemotherapy has demonstrated a survival benefit for patients with potentially resectable esophageal cancer; however, currently it is not possible to predict the benefit of this treatment for an individual patient. This prospective study was designed to correlate gene expression profiles with clinical outcome in this setting. Patients and Methods: Eligible patients were deemed to have resectable disease after staging by computed tomography, endoscopic ultrasound, and leperoscopy as indicated and following discussion at the multidisciplinary team meeting. All patients received neoadjuvant platinum and fluoropyrimidine-based chemotherapy; and clinical data were entered prospectively onto a study-specific database. Total RNA was isolated from pretreatment tumor biopsies obtained at baseline endoscopy and analyzed using a cDNA array consisting of 22,000 cDNA clones. Results: Of the patients with adequate follow-up accrued between 2002 and 2005, 35 satisfied the quality control measures for the microarray profiling. Median follow-up was 938 days. Supervised hierarchical clustering of normalized data revealed 165 significantly differentially expressed genes based on overall survival (OS; P < .01) with 2 distinct clusters: a poor outcome group: N = 17 (1 year OS 46.2%) and a good outcome group: N = 18 (1 year OS 100%). Genes identified included those previously associated with esophageal cancer and, interestingly, a group of genes encoding proteins involved in the regulation of the TOLL receptor-signaling pathway. Conclusion: This initial study has highlighted groups of tumors with distinct gene expression profiles based on survival and warrants further validation in a larger cohort. This approach may further our understanding of individual tumor biology and thus facilitate the development of tailored treatment.
TC 2
ZB 2
Z8 0
ZS 0
Z9 2
SN 1533-0028
UT WOS:000288479500007
PM 21609936

ER

PT J
AU Lorenzen, Sylvie
Panzram, Ben
Keller, Gisela
Lordick, Florian
Herrmann, Ken
Becker, Karin
Langer, Ruppert
Schwaiger, Markus
Siewert, Jorg Rudiger
Ott, Katja
TI Association of the VEGF 936C > T Polymorphism with FDG Uptake, Clinical, Histopathological, and Metabolic Response in Patients with Adenocarcinomas of the Esophagogastric Junction
SO MOLECULAR IMAGING AND BIOLOGY
VL 13
IS 1
BP 178
EP 186
DI 10.1007/s11307-010-0330-0
PD FEB 2011
PY 2011
AB The MUNICON trial confirmed prospectively the usefulness of early response evaluation by 2-deoxy-2-[F-18]fluoro-D-glucose-positron emission tomography (FDG-PET) . Metabolic responders (R) showed initially a higher FDG uptake compared with nonresponders (p = 0.018). An association of the vascular endothelial growth factor (VEGF) 936C > T polymorphism and FDG uptake was reported for breast cancer. Therefore, we investigated the VEGF 936C > T polymorphism for an association with response and survival.
The study was based on 110 patients included in the MUNCON trial (103 male, seven female; 75 AEG I, 35 AEG II, event-free survival (EFS) median 21.1 +/- 4.6 months). Response was significantly associated with EFS. The VEGF 936C > T polymorphism was determined by PCR and restriction fragment length polymorphism analysis. For analysis, the T-variants were combined.
One hundred two patients were evaluable. Seventy-two patients showed the

CC, 24 the CT, and six the TT genotype. Median EFS was 29.3 months for CC and 11.7 months for CT/TT (p = 0.04). No association of the genotypes (CC or CT/TT) with the SUV or response was found. Multivariate analysis revealed histopathological regression (p = 0.003) and genotype (p = 0.04) as independent prognostic factors. A combination of genotype and PET response (Gen-PET) defines three prognostic groups early in the course of treatment (p = 0.002). Cox regression analysis including clinical and histopathological response and Gen-PET reveals Gen-PET as independent prognostic factor (p = 0.003).
The VEGF 936C > T polymorphism is a prognostic factor in patients undergoing neoadjuvant chemotherapy, although it is not associated with FDG uptake and response. The combination of metabolic response and VEGF 936C > T polymorphism defines three different prognostic groups. These findings need to be confirmed prospectively.
This study has been registered in the European Clinical Trials Database as trial 2007-003356-11.
TC 5
ZB 3
Z8 0
ZS 0
Z9 5
SN 1536-1632
UT WOS:000286395600023
PM 20449668
ER

PT J
AU Miyata, Hiroshi
Yamasaki, Makoto
Takiguchi, Shuji
Nakajima, Kiyokazu
Fujiwara, Yoshiyuki
Konishi, Koji
Morii, Eiichi
Mori, Masaki
Doki, Yuichiro
TI Prognostic Value of Endoscopic Biopsy Findings After Induction Chemoradiotherapy With and Without Surgery for Esophageal Cancer
SO ANNALS OF SURGERY

VL 253
IS 2
BP 279
EP 284
DI 10.1097/SLA.0b013e318206824f
PD FEB 2011
PY 2011
AB Objective: To investigate the value of endoscopic biopsy in predicting the clinicopathological response and survival in patients with esophageal cancers who received chemoradiotherapy (CRT) alone or CRT followed by surgery.
Background: Endoscopic biopsy examination after CRT for esophageal cancer has been used to confirm the presence of residual tumor before surgery, but there is little or no information on the clinical significance of the results of endoscopic biopsy in neoadjuvant or definitive CRT.
Methods: We studied 189 patients who underwent endoscopic biopsy after induction CRT (40 Gy) for esophageal cancer, consisting of 123 patients who received neoadjuvant CRT (40 Gy) followed by surgery and 66 patients who underwent definitive CRT (mostly more than 60 Gy). The correlations between the results of endoscopic biopsy and clinicopathological factors, including response to CRT and survival, were examined.
Results: For neoadjuvant CRT, endoscopic biopsy findings correlated significantly with pathological tumor regression and lymph node involvement, although the majority of cases with negative biopsy (64%) displayed residual tumor cells in the surgical specimen. The 5-year survival rate was significantly higher in patients with negative biopsy (48.3%) than in those with positive biopsy (21.8%, P = 0.006). For definitive CRT, patients with negative biopsy at the time of 40 Gy showed clinical complete response to CRT (P = 0.002) and had significantly better 3-year survival (57.0%) than those with positive biopsy (22.5%, P = 0.0008).
Conclusions: The results of endoscopic biopsy examination after induction CRT can predict the response to CRT and prognosis of patients who receive CRT with and without surgery.
TC 7
ZB 0
Z8 0
ZS 0

Z9 7
SN 0003-4932
UT WOS:000286374400011
PM 21169807
ER

PT P
AU CLARK D P
SCHAYOWITZ A
CABRADILLA C
CABRADILLA C D
TI Diagnosing or prognosing diseases e.g. myocarditis and cancer, by determining difference between basal level or state of molecule in sample or molecule after contacting portion of sample with modulator
PN WO2011133477-A2; US2012094853-A1; WO2011133477-A3; AU2011242990-A1; EP2561368-A2; CN102947706-A; JP2013525786-W; KR2013095183-A; SG184507-A1
AE BIOMARKER STRATEGIES LLC
AB

NOVELTY - Method (M1) for diagnosing or prognosing a disease, involves determining difference between basal level or state of a molecule in sample and the level or state of the molecule after contacting a portion of sample with a modulator under ex vivo conditions, where difference is expressed as a value which is an indicative of presence, absence or risk of having disease.

USE - The method (M1) is useful for diagnosing or prognosing disease. The method (M2) is useful for predicting effect of an agent and method (M4) is useful for screening test agents for effect on molecule. The method (M3) is useful for monitoring disease or course of subject's therapy and method (M5) is useful for stratifying patients based on responsiveness to therapeutic agent or regimen. The method (M6) is useful for determining drug sensitivity or resistance and method (M7) is useful for determining functional stratification of live tumor sample. The method (M8) is useful for classifying cancer cell model system and method (M10) is useful for classifying melanoma cells. The method (M9) is useful for predicting outcome of therapeutic regimen in subject and method (M11) is useful for identifying drug resistance mechanisms or oncogene bypass mechanisms of melanoma cells. The disease is chosen from stroke, cardiovascular disease, chronic obstructive pulmonary disorder, myocardial infarction, congestive heart failure, cardiomyopathy,

myocarditis, ischemic heart disease, coronary artery disease, cardiogenic shock, vascular shock, pulmonary hypertension, pulmonary edema, preferably cardiogenic pulmonary edema, cancer, pathogen-mediated disease, pleural effusions, rheumatoid arthritis, diabetic retinopathy, retinitis pigmentosa, and retinopathy (e.g. diabetic retinopathy and retinopathy of prematurity), inflammatory diseases, restenosis, edema (e.g. edema associated with pathologic situations including cancer and edema induced by medical interventions such as chemotherapy), asthma, acute or adult respiratory distress syndrome, lupus, vascular leakage, transplant (e.g. organ transplant, acute transplant, heterograft or homograft rejection, ischemic or reperfusion injury protection, ischemic or reperfusion injury following angioplasty, ischemic or reperfusion injury occurred during organ transplantation and transplantation tolerance induction), arthritis (e.g. rheumatoid arthritis, psoriatic arthritis and osteoarthritis), multiple sclerosis, inflammatory bowel disease (e.g. ulcerative colitis, Crohn's disease and systemic lupus erythematosus), graft versus host diseases, T-cell mediated hypersensitivity diseases (e.g. contact hypersensitivity, delayed-type hypersensitivity, and gluten-sensitive enteropathy), type I diabetes, psoriasis, contact dermatitis, Hashimoto's thyroiditis, Sjogren's syndrome, autoimmune hyperthyroidism (e.g. Grave's disease, Addison's disease, autoimmune disease of adrenal glands, autoimmune polyglandular disease, autoimmune alopecia, pernicious anemia, vitiligo, autoimmune hypopituitarism and Guillain-Barre syndrome), cancers (e.g. colon carcinoma and thymoma), where Src-family kinases are activated or overexpressed or cancers where kinase activity facilitates tumor growth or survival), glomerulonephritis, serum sickness, urticaria, allergic diseases such as respiratory allergies (e.g. asthma, hay fever and allergic rhinitis) or skin allergies, mycosis fungoides, acute inflammatory responses, dermatomyositis, alopecia areata, chronic actinic dermatitis, eczema, Behcet's disease, Pustulosis palmoplantaris, pyoderma gangrenosum, Sezary's syndrome, atopic dermatitis, systemic sclerosis, morphea, peripheral limb ischemia and ischemic limb disease, bone disease (e.g. osteoporosis, osteomalacia, hyperparathyroidism, Paget's disease, and renal osteodystrophy), vascular leak syndromes induced by chemotherapy or immunomodulators, preferably interleukin 2 (IL-2), spinal cord and brain injury or trauma, glaucoma, retinal diseases (e.g. macular degeneration, vitreoretinal disease, pancreatitis, vasculitis, Kawasaki disease, thromboangiitis obliterans

and Wegener's granulomatosis, scleroderma, preeclampsia, thalassemia, Kaposi's sarcoma, and von Hippel Lindau disease. The cancer is chosen from colorectal cancer, esophageal cancer, stomach cancer, lung cancer, prostate cancer, uterine cancer, breast cancer, skin cancer, endocrine cancer, urinary cancer, pancreatic cancer, ovarian cancer, cervical cancer, head and neck cancer, liver cancer, bone cancer, biliary tract cancer, small intestine cancer, hematopoietic cancer, vaginal cancer, testicular cancer, anal cancer, kidney cancer, brain cancer, eye cancer, leukemia, lymphoma, soft tissue cancer, melanoma, and metastases. The pathogen is chosen from bacteria, fungi, viruses, spirochetes and parasites. The virus is chosen from Herpes simplex virus 1 (HSV1), HSV2, respiratory syncytial virus, measles virus, human cytomegalovirus, vaccinia virus, HIV-1, and hepatitis C virus (all claimed).
ADVANTAGE - The method (M1) is rapid, cost-effective, accurate, convenient to perform, and reduces risk of biocontamination. The samples collected can be maintained viably for long period of time.
DETAILED DESCRIPTION - INDEPENDENT CLAIMS are included for the following:(1) predicting (M2) effect of an agent;(2) monitoring (M3) disease or course of subject's therapy;(3) screening (M4) test agents for effect on a molecule;(4) stratifying (M5) patients based on responsiveness to therapeutic agent or therapeutic regimen;(5) determining (M6) drug sensitivity or resistance;(6) determining (M7) functional stratification of live tumor sample of subject;(7) classifying (M8) cancer cell model system;(8) predicting (M9) outcome of therapeutic regimen in subject;(9) classifying (M10) melanoma cells; and(10) identifying (M11) drug resistance mechanisms or oncogene bypass mechanisms of melanoma cells.
Z9 0
UT DIIDW:2011N53369
ER

PT J
AU Twaddell, William S.
Wu, Peter C.
Verhage, Roy J. J.
Feith, Marcus
Ilson, David H.
Schuhmacher, Christoph P.
Luketich, James D.

Bruecher, Bjoern
Vallboehmer, Daniel
Hofstetter, Wayne L.
Krasna, Mark Jonathan
Kandioler, Daniela
Schneider, Paul M.
Wijnhoven, Bas P. L.
Sontag, Stephen J.
BA Giuli, R
TI Barrett's esophagus: treatments of adenocarcinomas II
SO BARRETT'S ESOPHAGUS: THE 10TH OESO WORLD CONGRESS PROCEEDINGS
SE Annals of the New York Academy of Sciences
VL 1232
BP 265
EP 291
DI 10.1111/j.1749-6632.2011.06056.x
PD 2011
PY 2011
AB The following topics are explored in this collection of commentaries on treatments of adenocarcinomas related to Barrett's esophagus: the importance of intraoperative frozen sections of the margins for the detection of high dysplasia; the preferable way for sentinel node dissection; the current role of robotic surgery and of video-endoscopic approach; the value of the Siewert's classification of adenocarcinomas; the indications of two-step esophagectomy; the evaluation of pathological complete response; the role of PET scan in staging and response assessment; the role of p53 in the selection of adenocarcinomas patients; chemotherapy regimens for adenocarcinomas; the use of monoclonal antibodies in the control of cell proliferation; he attempt to define a stagespecific strategy, and the possible indications of selective therapy; and changes in mortality rates from esophageal cancer.
CT 10th World Congress of the World Organization for Specialized Studies on Diseases of the Esophagus (OESO)
CY AUG 28-31, 2010
CL Boston, MA
RI huang, simon/H-3756-2012
TC 1
ZB 1

Z8 0
ZS 0
Z9 1
SN 0077-8923
BN 978-1-57331-829-7
UT WOS:000301188400016
PM 21950818
ER

PT J
AU Okines, Alicia F. C.
Reynolds, Andrew R.
Cunningham, David
TI Targeting Angiogenesis in Esophagogastric Adenocarcinoma
SO ONCOLOGIST
VL 16
IS 6
BP 844
EP 858
DI 10.1634/theoncologist.2010-0387
PD 2011
PY 2011
AB The possibility of targeting tumor angiogenesis was postulated almost 40 years ago. The vascular endothelial growth factor (VEGF) family and its receptors have since been characterized and extensively studied. VEGF overexpression is a common finding in solid tumors, including esophagogastric cancer, and frequently correlates with poor prognosis. Monoclonal antibodies, soluble receptors, and small-molecule tyrosine kinase inhibitors have been developed to inhibit tumor angiogenesis, and antiangiogenic therapy is now a component of standard treatment for advanced renal cell, hepatocellular, colorectal, breast, and non-small cell lung carcinomas. The small-molecule tyrosine kinase inhibitors sunitinib and sorafenib have been evaluated in phase II studies in esophagogastric cancer but appear to have only modest activity. Similarly, despite promising efficacy signals from phase II studies, the addition of the anti-VEGF-A monoclonal antibody bevacizumab to cisplatin plus capecitabine failed to result in a longer overall survival duration than with the chemotherapy doublet plus placebo. The response rate and progression-free survival interval were significantly greater with

bevacizumab, confirming some efficacy in advanced gastric cancer, but
with inadequate benefit to justify the high cost of treatment.
Evaluation of bevacizumab in the neoadjuvant and perioperative settings
continues, hypothesizing that a higher response rate will translate into
longer survival in patients with operable disease. Despite extensive
research, the discovery of a reliable predictive biomarker for
antiangiogenic therapy continues to elude the scientific and oncology
communities, and mechanisms of primary and acquired resistance are
incompletely understood. We are therefore currently unable to
personalize antiangiogenic therapy for established indications, or use
molecular selection for clinical trials evaluating novel indications.
The Oncologist 2011;16:844-858
TC 15
ZB 12
Z8 2
ZS 0
Z9 17
SN 1083-7159
UT WOS:000291928900014
PM 21632459
ER

PT J
AU Jatoi, Aminah
Soori, Gamini
Foster, Nathan R.
Hiatt, Bradley K.
Knost, James A.
Fitch, Tom R.
Callister, Matthew D.
Nichols, Francis C., III
Husted, Tim M.
Alberts, Steven R.
TI Phase II Study of Preoperative Pemetrexed, Carboplatin, and Radiation
Followed by Surgery for Locally Advanced Esophageal Cancer and
Gastroesophageal Junction Tumors
SO JOURNAL OF THORACIC ONCOLOGY
VL 5
IS 12

BP 1994
EP 1998
DI 10.1097/JTO.0b013e3181fb5c3e
PD DEC 2010
PY 2010
AB Introduction: Based on favorable preliminary clinical data and the need to identify effective, well-tolerated neoadjuvant regimens for patients with locally advanced esophageal cancer, this clinical trial was undertaken.
Methods: This phase II study tested 500 mg/m(2) neoadjuvant pemetrexed intravenously and carboplatin with an area under the curve of 6 intravenously on days 1 and 22 in conjunction with concomitant radiation of 5040 centigray, which was given in 28 daily fractions of 180 centigray. The primary endpoint was the rate of pathologic complete response.
Results: This trial closed early because, during an interim analysis, the primary endpoint fell short. However, 26 eligible patients were accrued. Twenty (74%) were men. Performance scores of 0, 1, and 2 were seen in 16 (59%), 9 (33%), and 2 (7%), respectively. Among eligible patients, 6 of 26 (23%; 95% confidence interval 9-44%) demonstrated a pathologic complete response. Twenty-two underwent a complete cancer resection. The median survival was 17.8 months (95% confidence interval: 12.2-30.7 months). In the neoadjuvant setting, 22 patients had at least one grade 3 or worse adverse event, and 8 patients had at least one grade 4 event. Postoperatively (within 30 days of surgery), there were three deaths, one grade 4 event (thrombosis), and three grade 3 events.
Conclusions: The neoadjuvant regimen tested within this phase II trial demonstrated antineoplastic activity but fell short of yielding a complete pathologic response rate that merits further testing.
TC 7
ZB 2
Z8 0
ZS 0
Z9 7
SN 1556-0864
UT WOS:000284579200018
PM 20975604
ER

PT J
AU Li, Wei
Qin, Jing
Sun, Yi-Hong
Liu, Tian-Shu
TI Neoadjuvant chemotherapy for advanced gastric cancer: A meta-analysis
SO WORLD JOURNAL OF GASTROENTEROLOGY
VL 16
IS 44
BP 5621
EP 5628
DI 10.3748/wjg.v16.i44.5621
PD NOV 28 2010
PY 2010
AB AIM: To study the value of neoadjuvant chemotherapy (NAC) for advanced gastric cancer by performing a meta-analysis of the published studies.
METHODS: All published controlled trials of NAC for advanced gastric cancer vs no therapy before surgery were searched. Studies that included patients with metastases at enrollment were excluded. Databases included Cochrane Library of Clinical Comparative Trials, MEDLINE, Embase, and American Society of Clinical Oncology meeting abstracts from 1978 to 2010. The censor date was up to April 2010. Primary outcome was the odds ratio (OR) for improving overall survival rate of patients with advanced gastric cancer. Secondary outcome was the OR for down-staging tumor and increasing R0 resection in patients with advanced gastric cancer. Safety analyses were also performed. All calculations and statistical tests were performed using RevMan 5.0 software.
RESULTS: A total of 2271 patients with advanced gastric cancer enrolled in 14 trials were divided into NAC group (n = 1054) and control group (n = 1217). The patients were followed up for a median time of 54 mo. NAC significantly improved the survival rate [OR = 1.27, 95% confidence interval (CI): 1.04-1.55], tumor stage (OR = 1.71, 95% CI: 1.26-2.33) and R0 resection rate (OR = 1.51, 95% CI: 1.19-1.91) of patients with advanced gastric cancer. No obvious safety concerns were raised in these trials.
CONCLUSION: NAC can improve tumor stage and survival rate of patients with advanced gastric cancer with a rather good safety. (C) 2010 Baishideng. All rights reserved.
TC 33

ZB 14
Z8 4
ZS 1
Z9 36
SN 1007-9327
UT WOS:000284989700013
PM 21105197
ER

PT J
AU Fushida, Sachio
Fujimura, Takashi
Oyama, Katsunobu
Kinoshita, Jun
Fujita, Hideto
Ninomiya, Itasu
Ohta, Tetsuo
TI Neoadjuvant Chemotherapy Combining Docetaxel, Cisplatin and S-1 in Gastric Cancer with Para-aortic Lymph Node Metastases: Report of Five Cases
SO HEPATO-GASTROENTEROLOGY
VL 58
IS 104
BP 1650
EP 1654
PD NOV-DEC 2010
PY 2010
AB We report on five patients with a median age of 56 years (range, 53-68 years) who were diagnosed as advanced gastric cancer with para-aortic lymph node metastases, determined by gastrofiberscope and abdominal spiral computed tomography. These patients received intravenous docetaxel (30mg/m(2)) and cisplatin (30mg/m(2)) on day 1, 15 and oral S-1 (40mg/m(2) bid) on days 1-14 every 4 weeks. After two cycles of neoadjuvant chemotherapy, all patients received total gastrectomy with D2 lymphadenectomy plus para-aortic lymph nodes dissection. No patient revealed hematological or non-hematological toxicities associated with the chemotherapy (more than grade 2). The postoperative courses were uneventful without major surgery-related complications. Pathological complete response was confirmed one patient in primary lesion and three

patients in metastatic lymph nodes including para-aortic lymph nodes. All of five patients are alive without recurrence (median: 36 months, 21-46 months). Thus, neoadjuvant chemotherapy described here may be rather promising regimen for advanced gastric cancer with para-aortic lymph node metastases, considering its low grade toxicities and pathological responses.

TC 1

ZB 1

Z8 0

ZS 0

Z9 1

SN 0172-6390

UT WOS:000287685900058

ER

PT J

AU Stiles, Brendon M.
Mirza, Farooq
Port, Jeffrey L.
Lee, Paul C.
Paul, Subroto
Christos, Paul
Altorki, Nasser K.

TI Predictors of Cervical and Recurrent Laryngeal Lymph Node Metastases From Esophageal Cancer

SO ANNALS OF THORACIC SURGERY

VL 90

IS 6

BP 1805

EP 1811

DI 10.1016/j.athoracsur.2010.06.085

PD NOV 2010

PY 2010

AB Background. Although patients with esophageal cancer (EC) often develop lymph node metastases in the cervical and recurrent laryngeal (CRL) distribution, lymphadenectomy in this field is rarely performed. The purpose of this study was to determine factors associated with CRL node positivity and to determine the appropriate indications to perform a "three field" lymphadenectomy.

Methods. In a retrospective review, EC patients who underwent three-field lymphadenectomy were analyzed. Predictors of positive CRL nodes were examined univariately, then selected for inclusion in a multivariate logistic regression model.
Results. From 1994 to 2009, 185 patients had a three-field lymphadenectomy, of whom 46 patients (24.9%) had positive CRL nodes. Final pathology stages (seventh edition) were I in 24 patients, II in 43, III in 109, and IV in 1 patient. Eight patients had a major pathologic response after induction therapy. On univariate analysis, variables significantly associated with positive CRL nodes included squamous cell histology, proximal location, advanced clinical presentation, the presence of clinical nodal disease, higher pT classification, and higher pN classification. There was no reduction in the rate of positive CRL nodes after induction chemotherapy. On multivariate analysis, higher pN classification (adjusted odds ratio 16.25, 95% confidence interval: 5.40 to 48.87; $p < 0.0001$) and squamous histology (adjusted odds ratio 6.04, 95% confidence interval: 2.21 to 16.56; $p < 0.0001$) predicted positive CRL nodes.
Conclusions. Complete lymphadenectomy is necessary in esophageal cancer to appropriately stage patients. Low rates of positive CRL nodes are present with early clinical stage, with pT0-2 tumors, and with pN0 classification, particularly in patients with adenocarcinoma and gastroesophageal junction tumors. Dissection of the CRL field should be considered with advanced disease for adenocarcinoma and in all patients with squamous cell cancer. (Ann Thorac Surg 2010;90:1805-11) (C) 2010 by The Society of Thoracic Surgeons
CT 46th Annual Meeting of the Society-of-Thoracic-Surgeons
CY JAN 24-27, 2010
CL Ft Lauderdale, FL
SP Soc Thorac Surg
TC 9
ZB 3
Z8 1
ZS 0
Z9 10
SN 0003-4975
UT WOS:000284463200018
PM 21095315
ER

PT J
AU Funaki, Hiroshi
Ohnishi, Toshio
Ohno, Yukako
Tomita, Yasuto
Hosokawa, Kenzo
Yokoi, Miki
Yoshitani, Shinichiro
Kinami, Shinichi
Omote, Kazuhiko
Ueda, Nobuhiko
Nakano, Yasuharu
Kosaka, Takeo
TI [A case of AGC with pCR after preoperative chemotherapy including S-1 plus cisplatin].
SO Gan to kagaku ryoho. Cancer & chemotherapy
VL 37
IS 12
BP 2430
EP 2
PD 2010-Nov
PY 2010
AB A 79-year-old man complaining of epigastralgia was examined and diagnosed with advanced gastric cancer (UML, Type 5, Ant-Less-Gre, cT4a, cN1, cH0, cP1, cStage IV). A poor prognosis was predicted, but we tried preoperative chemotherapy hoping for a down-staging of the tumor. We chose a regimen of S-1 plus cisplatin as follows: S-1 (60 mg/m2) was administered orally for 3 weeks followed 2 weeks of rest, and cisplatin (50 mg/m2) was administered by intravenous drip on day 8. After three cycles of treatment, diagnostic laparoscopic examination revealed a suspected serosal invasion of the main tumor, but peritoneal dissemination was not seen, and abdominal washing cytology was negative. After the fourth cycle of treatment, total gastrectomy with lymph node dissection (D1+No. 7, 8a, 9, R0) was performed. Histological examination of the resected specimens revealed no residual cancer cells in the primary lesion or regional lymph nodes, resulting in a diagnosis of complete response to chemotherapy according to the Japanese Classification of Gastric Carcinoma. The postoperative course was

uneventful, and he has been fine as an outpatient.
TC 0
ZB 0
Z8 0
ZS 0
Z9 0
SN 0385-0684
UT MEDLINE:21224596
PM 21224596
ER

PT J
AU Fushida, Sachio
Fujimura, Takashi
Oyama, Katsunobu
Kinoshita, Jun
Fujita, Hideto
Ninomiya, Itasu
Ohta, Tetsuo
TI Neoadjuvant chemotherapy combining docetaxel, cisplatin and S-1 in gastric cancer with para-aortic lymph node metastases: report of five cases.
SO Hepato-gastroenterology
VL 57
IS 104
BP 1650
EP 4
PD 2010 Nov-Dec
PY 2010
AB We report on five patients with a median age of 56 years (range, 53-68 years) who were diagnosed as advanced gastric cancer with para-aortic lymph node metastases, determined by gastrofiberscope and abdominal spiral computed tomography. These patients received intravenous docetaxel (30 mg/m2) and cisplatin (30 mg/m2) on day 1, 15 and oral S-1 (40 mg/m2 bid) on days 1-14 every 4 weeks. After two cycles of neoadjuvant chemotherapy, all patients received total gastrectomy with D2 lymphadenectomy plus para-aortic lymph nodes dissection. No patient revealed hematological or non-hematological toxicities associated with the chemotherapy (more than grade 2). The postoperative courses were

uneventful without major surgery-related complications. Pathological complete response was confirmed one patient in primary lesion and three patients in metastatic lymph nodes including para-aortic lymph nodes. All of five patients are alive without recurrence (median: 36 months, 21-46 months). Thus, neoadjuvant chemotherapy described here may be rather promising regimen for advanced gastric cancer with para-aortic lymph node metastases, considering its low grade toxicities and pathological responses.
TC 2
ZB 1
Z8 1
ZS 0
Z9 3
SN 0172-6390
UT MEDLINE:21443137
PM 21443137
ER

PT J
AU Staal, E. F. W. Courrech
Aleman, B. M. P.
Boot, H.
van Velthuysen, M. -L. F.
van Tinteren, H.
van Sandick, J. W.
TI Systematic review of the benefits and risks of neoadjuvant chemoradiation for oesophageal cancer
SO BRITISH JOURNAL OF SURGERY
VL 97
IS 10
BP 1482
EP 1496
DI 10.1002/bjs.7175
PD OCT 2010
PY 2010
AB Background: Surgery alone for locally advanced oesophageal cancer is associated with low cure rates. The benefits and risks of neoadjuvant chemoradiation for patients with oesophageal cancer were evaluated. Methods: A systematic review of publications between 2000 and 2008 on

neoadjuvant chemoradiation for oesophageal cancer was undertaken.
Results: Thirty-eight papers comprising 3640 patients met the inclusion criteria. Chemoradiation regimens varied widely with a predominance of 5-fluorouracil/cisplatin chemotherapy. Chemoradiation-related toxicity was reported in only ten studies and consisted mainly of neutropenia. The chemoradiation-related mortality rate was 2.3 per cent. The mean R0 resection rate and pathological complete response (pCR) rate were 88.4 and 25.8 per cent respectively. Postoperative morbidity was not uniformly reported. The in-hospital mortality rate after oesophagectomy following chemoradiation was 5.2 per cent. Five-year survival rates varied from 16 to 59 per cent in all patients and from 34 to 62 per cent in those with a pCR. Chemoradiation had a temporary negative effect on quality of life.
Conclusion: Neoadjuvant chemoradiation regimens for oesophageal cancer vary widely. Besides traditional outcome variables (such as survival), other parameters should be analysed (for example toxicity) to assess whether the risks of chemoradiation are sufficiently compensated for by the benefits.
TC 44
ZB 16
Z8 0
ZS 1
Z9 45
SN 0007-1323
UT WOS:000282011900005
ER

PT J
AU Swisher, Stephen G.
Hofstetter, Wayne
Komaki, Ritsuko
Correa, Arlene M.
Erasmus, Jeremy
Lee, Jeffrey H.
Liao, Zhongxing
Maru, Dipen
Mehran, Reza
Patel, Shital
Rice, David C.

Roth, Jack A.
Vaporciyan, Ara A.
Walsh, Garrett L.
Ajani, Jaffer A.
TI Improved Long-Term Outcome With Chemoradiotherapy Strategies in Esophageal Cancer
SO ANNALS OF THORACIC SURGERY
VL 90
IS 3
BP 892
EP 898
DI 10.1016/j.athoracsur.2010.04.061
PD SEP 2010
PY 2010
AB Background. Controversy currently exists about the optimum preoperative treatment platform for locoregionally advanced esophageal cancer, namely, preoperative chemoradiotherapy (preoperative C/RT) or preoperative chemotherapy alone. We therefore reviewed sequential phase II/III trials performed at a single institution to assess the impact of preoperative chemotherapy versus preoperative C/RT strategies.
Methods. In all, 157 esophageal cancer patients were sequentially enrolled in phase II/III trials at the University of Texas M. D. Anderson Cancer Center from March 27, 1990, to March 8, 2005. The treatment approaches included preoperative chemotherapy, n = 76 (INT 113 and ID90-01); preoperative C/RT, n = 81 (ID96-189 and DM98-349). Analysis was by intention to treat. Factors evaluated included demographics, preoperative staging, type of surgery, pathology, adjuvant therapies, and long-term outcome.
Results. Adenocarcinoma predominated (85%), with cT3 (73%) and cN1 (43%). No significant difference was noted between groups in demographics or perioperative mortality. More patients with preoperative C/RT were staged with endoscopic ultrasound (52% versus 9%, $p < 0.001$). Preoperative C/RT demonstrated increased pathologic complete response (28% versus 4%, $p < 0.001$) and overall survival (3 years, 48% versus 29%, $p = 0.04$). Preoperative C/RT was a significant independent predictor of improved overall survival (hazard ratio 0.58, 95% confidence interval: 0.37 to 0.90, $p = 0.015$) and disease-free survival (hazard ratio 0.55, 95% confidence interval: 0.35 to 0.85, $p = 0.007$) in multivariable regression.

Conclusions. In sequential phase II/III trials involving locoregionally advanced esophageal cancer patients, preoperative C/RT was associated with improved overall and disease-free survival rates (p = 0.046 and p = 0.015, respectively) and increased pathologic complete response (p < 0.001) compared with preoperative chemotherapy. (Ann Thorac Surg 2010;90:892-9) (C) 2010 by The Society of Thoracic Surgeons
CT 56th Annual Meeting of the Southern-Thoracic-Surgical-Association
CY NOV 04-07, 2009
CL Marco Isl, FL
SP So Thorac Surg Assoc
TC 9
ZB 2
Z8 3
ZS 0
Z9 13
SN 0003-4975
UT WOS:000281142600034
PM 20732514
ER

PT J
AU Knox, Jennifer J.
Wong, Rebecca
Visbal, Antonio L.
Horgan, Anne M.
Guindi, Maha
Hornby, Jennifer
Xu, Wei
Ringash, Jolie
Keshavjee, Shaf
Chen, Eric
Haider, Masoom
Darling, Gail
TI Phase 2 Trial of Preoperative Irinotecan Plus Cisplatin and Conformal Radiotherapy, Followed by Surgery for Esophageal Cancer
SO CANCER
VL 116
IS 17
BP 4023

EP 4032
DI 10.1002/cncr.25349
PD SEP 1 2010
PY 2010
AB BACKGROUND: Esophagectomy for locally advanced esophageal cancer (LAEC) is associated with limited survival. Trimodality therapy yields a small survival advantage, with cisplatin and 5-fluorouracil regimens most frequently studied. Newer regimens may impact these poor outcomes. This phase 2 trial assessed the feasibility and efficacy of induction chemoradiotherapy with cisplatin and irinotecan followed by esophagectomy. METHODS: Patients with LAEC of the thoracic esophagus or gastroesophageal junction underwent chemotherapy with preoperative irinotecan (65 mg/m(2)) plus cisplatin (30 mg/m(2)) on Weeks 1, 2, 4, 5, 7, and 8 with concurrent conformal radiotherapy (40 grays [Gy]/20 fractions during Weeks 4-7) and external beam boost (10 Gy/5 fractions at Week 8). Esophagectomy was performed between Weeks 12 and 16. Pathologic response was the primary endpoint with follow-up data on progression, survival, and toxicity as secondary endpoints. RESULTS: Fifty-two patients were enrolled from November 2002 to October 2005. Nineteen patients had American Joint Committee on Cancer stage II, 22 had stage ill, and 11 had stage IVA disease. Grade 3 to 4 toxicity (graded according to the National Cancer Institute Common Toxicity Criteria 2.0) during induction included neutropenia (36%), febrile neutropenia (8%), diarrhea (10%), and esophagitis (4%). Three patients withdrew from treatment due to toxicity. There was 1 treatment-related death. Clinical responses included complete response in 2%, partial response in 30%, stable disease in 62%, and progressive disease in 6% of patients. Dysphagia improved/resolved in 72% of patients during induction. Forty-three patients underwent esophagectomy and 7 (16%) achieved pathologic complete responses. Median and 3-year overall survival for patients receiving trimodality therapy was 36 months and 51%, respectively. CONCLUSIONS: In LAEC, concurrent irinotecan/cisplatin and radiotherapy followed by esophagectomy is reported to be associated with dysphagia improvement in 72% of patients, a significant but manageable toxicity profile, and encouraging survival compared with historic controls. Cancer 2010;116:4023-32. (C) 2010 American Cancer Society
TC 8
ZB 3

Z8 1
ZS 0
Z9 9
SN 0008-543X
UT WOS:000281026000009
PM 20533506
ER

PT J
AU Power, D. G.
Reynolds, J. V.
TI Localized adenocarcinoma of the esophagogastric junction - Is there a standard of care?
SO CANCER TREATMENT REVIEWS
VL 36
IS 5
BP 400
EP 409
DI 10.1016/j.ctrv.2010.01.001
PD AUG 2010
PY 2010
AB Adenocarcinoma of the esophagogastric junction (AEG) is the most rapidly increasing tumour in the Western world. Most patients present with locally advanced resectable disease and treatment can be curative. However, no accepted standard treatment exists. Cancer specialists frequently differ on optimum treatment strategies. Areas of debate include the aetiology of AEG, TNM staging, type and extent of resection, relative benefits of preoperative chemotherapy versus preoperative chemoradiation (CRT) versus postoperative CRT, use of early PET scan, and integration of targeted therapy. Randomized trials are weakened by underpowered numbers for AEG tumours, and by methodologic flaws. RO resection and pathologic complete responses (pCR) predict long-term survival, and most treatment strategies target this as a proxy measure of improved outcome. Some preoperative chemotherapy trials show a benefit but the numbers of true AEG tumours in these studies is unclear. The MAGIC study was powered for gastric cancer only, with just 27% of patients having AEG. Compared with chemotherapy alone, preoperative CRT trials show higher rates of pCR. A large randomized study, with significant toxicity, has shown long-term benefit with adjuvant CRT

after resection of gastric cancer (20% AEG). An international consensus on the true definition and optimum management of AEG is required. Molecular and imaging biomarkers will play a vital role in future trials. Trimodality therapy is likely to be optimum with surgery shifted to later in the treatment pathway. Rectal cancer provides an analogous paradigm in this regard. As systemic disease is the primary cause of mortality chemosensitivity should be determined early. (C) 2010 Elsevier Ltd. All rights reserved.
TC 5
ZB 0
Z8 2
ZS 1
Z9 8
SN 0305-7372
UT WOS:000280981200004
PM 20117883
ER

PT J
AU Holmebakk, T.
Frykholm, G.
Viste, A.
TI Introducing national guidelines on perioperative chemotherapy for gastric cancer in Norway: A retrospective audit
SO EJSO
VL 36
IS 7
BP 610
EP 616
DI 10.1016/j.ejso.2010.05.006
PD JUL 2010
PY 2010
AB Background: In 2006, perioperative chemotherapy with epirubicin, cisplatin/oxaliplatin, and capecitabine was recommended in the National Guidelines for patients with resectable gastric cancer in Norway. We conducted a national audit related to clinical aspects, local organisation and the implementation of this multimodal treatment.
Patients and methods: All Norwegian departments of oncology were asked to submit aggregated data on gastric cancer patients who had started

perioperative chemotherapy for cure; departments of surgery were asked
to report on patients undergoing resection after preoperative
chemotherapy. Data were retrospectively collected.
Results: All 20 departments of oncology and 20 of 21 departments of
surgery responded. Of 336 patients operated on for gastric cancer and
reported by surgeons, 144 (43%) received preoperative chemotherapy. 169
patients were reported by departments of oncology. 152 (90%) completed
the preoperative cycles; 92 (54%) started the postoperative cycles; and
68 (40%) completed all cycles. Toxicity grade >= 3, overall and
haematological, increased during postoperative compared to preoperative
cycles, 50 vs. 34% (P=0.012) and 35 vs. 20% (P=0.012), respectively.
Surgical morbidity and mortality were 26 and <2%, respectively. R0
resection was achieved in 86% of surgically treated patients. Five per
cent had a complete pathological response (ypT0) and 48% were node
negative (ypN0). Within the first year, the National Guidelines were
implemented in 19 of 25 hospitals (76%).
Conclusions: In this population-based series, the tolerability of
perioperative chemotherapy reported in the MAGIC trial was reproduced.
Toxicity grade >= 3 was considerable and significantly increased related
to postoperative cycles. The National Guidelines were rapidly adopted.
(C) 2010 Elsevier Ltd. All rights reserved.
TC 3
ZB 1
Z8 0
ZS 0
Z9 3
SN 0748-7983
UT WOS:000280438700003
PM 20627647
ER

PT J
AU Amin, Anwar Tawfik
Shiraishi, Norio
Ninomiya, Shigeo
Tajima, Masaaki
Inomata, Masafumi
Kitano, Seigo
TI Increased mRNA expression of epidermal growth factor receptor, human

epidermal receptor, and survivin in human gastric cancer after the surgical stress of laparotomy versus carbon dioxide pneumoperitoneum in a murine model
SO SURGICAL ENDOSCOPY AND OTHER INTERVENTIONAL TECHNIQUES
VL 24
IS 6
BP 1427
EP 1433
DI 10.1007/s00464-009-0793-8
PD JUN 2010
PY 2010
AB Surgical impact may be associated with enhanced tumor growth and chemoresistance. This study aimed to evaluate the effect of surgical impact on the mRNA expression of survivin, epidermal growth factor receptor (EGFR), and human epidermal receptor (HER2) in tumors after pneumoperitoneum versus laparotomy.

Nude mice were inoculated intraperitoneally with human gastric cancer cells (MKN45). Then laparotomy, carbon dioxide (CO(2)) pneumoperitoneum, and anesthesia alone were performed randomly, after which EGFR, HER2, and survivin mRNA expression using reverse transcription-polymerase chain reaction (RT-PCR) was evaluated.

The expression of EGFR and HER2 mRNA increased significantly after the experiment. However, it was higher after laparotomy than after CO(2) pneumoperitoneum at almost all examined time points. Survivin mRNA expression increased significantly in the first 48 h, then returned to the control level. It was higher after laparotomy than after CO(2) pneumoperitoneum 48 h after the surgical procedures.

The expression of EGFR, HER2, and survivin increased after each surgical procedure. However it was lower after CO(2) pneumoperitoneum than after laparotomy. This might be associated with changes in the chemosensitivity of the remnant cancer cells after surgery, supporting the use of minimally invasive surgery for cancer.
TC 2
ZB 1
Z8 1
ZS 0
Z9 3
SN 0930-2794
UT WOS:000277713800030

PM 20041268
ER

PT J
AU Spigel, David R.
Greco, F. Anthony
Meluch, Anthony A.
Lane, Cassie M.
Farley, Cynthia
Gray, James R.
Clark, Bobby L.
Burris, Howard A., III
Hainsworth, John D.
TI Phase I/II Trial of Preoperative Oxaliplatin, Docetaxel, and Capecitabine With Concurrent Radiation Therapy in Localized Carcinoma of the Esophagus or Gastroesophageal Junction
SO JOURNAL OF CLINICAL ONCOLOGY
VL 28
IS 13
BP 2213
EP 2219
DI 10.1200/JCO.2009.24.8773
PD MAY 1 2010
PY 2010
AB Purpose
Preoperative chemoradiotherapy is a primary treatment option for patients with resectable esophageal cancer. Combination regimens using newer agents may improve patient outcomes. This multicenter community-based phase I/II trial examined a modern triplet regimen comprised of oxaliplatin, docetaxel, and capecitabine (ODC) combined with radiation therapy (RT).
Patients and Methods
The primary end point was the pathologic complete response (pCR) rate. Eligibility criteria included resectable stage I to III cancer of the mid-/distal-esophagus or gastroesophageal junction, measurable disease, and Eastern Cooperative Oncology Group performance status (ECOG PS) of 0 or 1. Treatment included oxaliplatin 40 mg/m(2), docetaxel 20 mg/m(2) (intravenous, weekly x 5); capecitabine 1,000 mg/m(2) orally twice daily on days 1 to 7, 15 to 21, and 29 to 35; and concurrent RT (45 Gy).

Resection was performed during weeks 9 to 12. ODC and RT safety was determined in a phase I portion (n = 10) preceding phase II.
Results
Fifty-nine patients were enrolled (September 2005 to February 2008; phase I/cohort 1, 10 patients; phase I/cohort 2/phase II, 49 patients). Baseline characteristics included median age of 63 years; 84% male; ECOG PS 0 and 1, 51% and 49%, respectively; adenocarcinoma and squamous cell, 69% and 18%, respectively; stage I, II, and III, 12%, 41%, and 45%, respectively. Phase I revealed no dose-limiting toxicity. Responses: pCR rate, 49%; objective response rate, 61% (24 complete and six partial responses); stable disease, 6%; and progressive disease, 2%. Sixty-nine percent of patients underwent surgery. Survival: median follow-up, 116 weeks; median disease-free survival (DFS) and overall survival (OS) were 16.3 and 24.1 months, respectively. Two-year DFS and OS were 45.1% and 52.2%, respectively. Most common (>= 5%) grade 3 to 4 nonhematologic toxicities were anorexia (20%), dehydration (16%), diarrhea (8%), dysphagia (10%), esophagitis (20%), fatigue (12%), hyperglycemia (6%), nausea (16%), pulmonary symptoms (14%), sepsis (6%), and vomiting (16%). All other grade 3 to 4 hematologic and nonhematologic toxicities were uncommon (< 5%).
Conclusion
Preoperative ODC plus RT is active and relatively safe in patients with locoregional esophageal cancer. Importantly, this therapy can be administered within 8 weeks. This regimen warrants additional study in this setting and in combination with newer biologic agents.
TC 17
ZB 11
Z8 7
ZS 0
Z9 24
SN 0732-183X
UT WOS:000277180300015
PM 20351330
ER

PT J
AU Ajani, Jaffer A.
Correa, Arlene M.
Walsh, Garrett L.

Komaki, Ritsuko
Lee, Jeffrey H.
Vaporciyan, Ara A.
Rice, David C.
Yao, James C.
Maru, Dipen M.
Hofstetter, Wayne L.
Phan, Alexandria T.
Swisher, Stephen G.

TI Trimodality Therapy Without a Platinum Compound for Localized Carcinoma of the Esophagus and Gastroesophageal Junction

SO CANCER

VL 116

IS 7

BP 1656

EP 1663

DI 10.1002/cncr.24935

PD APR 1 2010

PY 2010

AB BACKGROUND: The use of platinum-based chemoradiation for esophageal cancer is routine, but it is unclear which class of cytotoxic are optimum. It was hypothesized that chemoradiotherapy with fluoropyrimidine, taxane, and camptothecin would have preserved or improved efficacy with no compromise in safety. METHODS: Patients with histologically confirmed, resectable esophageal carcinoma were eligible. In addition to other tests, a baseline endoscopic ultrasonography (EUS) was obtained. Patients were medically fit and had near-normal organ functions. Patients received docetaxel and irinotecan, plus 5-fluorouracil as induction therapy and then the same cytotoxics with 50.4 grays of radiotherapy followed by an attempted surgery. Pathologic complete response (pathCR) at a rate of >= 20% was the primary endpoint. The pathCR and R0 resection were correlated with overall survival (OS). Safety was documented. RESULTS: Fifty-five patients were enrolled. Seven were women, and the median age was 56 years. Fifty-three (96%) patients had EUST3, and 41(75%) had EUSN1 disease. Forty-three (78%) patients underwent surgery, 20% achieved a pathCR, and 76.4% underwent an R0 resection. The median survival (n = 55 patients) was 43.3 months (range, 19-75 months). Baseline clinical parameters were not found to be predictive of OS; however, patients with a pathCR (P = .005) and who

underwent RO resection (P <= .0001) had an improved OS. There was 1
treatment-related postsurgical death reported. Grade 3 or 4 toxicity
(graded according to the National Cancer Institute Common Toxicity
Criteria [version 2.0]) was observed in 62% of patients. CONCLUSIONS:
The results of the current study documented that this 3-drug,
noncisplatin-based chemoradiotherapy was feasible, safe, and active but
not better than the published cisplatin-based chemoradiotherapy. A
fluoropyrimidine and another cytotoxic (from any class) may be adequate
to establish a baseline chemoradiotherapy regimen to combine biologics.
Cancer 2010;116:1656-63. (C) 2070 American Cancer Society

TC 8

ZB 2

Z8 0

ZS 0

Z9 8

SN 0008-543X

UT WOS:000275983500007

PM 20143431

ER

PT J

AU Choi, Chel Hun
Song, Sang Yong
Kang, Heeseok
Lee, Yoo-Young
Kim, Chul-Jung
Lee, Jeong-Won
Kim, Tae-Joong
Kim, Byoung-Gie
Lee, Je-Ho
Bae, Duk-Soo

TI Prognostic significance of p-STAT3 in patients with bulky cervical
carcinoma undergoing neoadjuvant chemotherapy

SO JOURNAL OF OBSTETRICS AND GYNAECOLOGY RESEARCH

VL 36

IS 2

BP 304

EP 310

DI 10.1111/j.1447-0756.2009.01131.x

PD APR 2010
PY 2010
AB Aim:
To better predict treatment responses for managing bulky cervical carcinoma with neoadjuvant chemotherapy (NAC).
Methods:
The expression of p-STAT3 was analyzed by immunohistochemistry using paraffin-embedded pretreatment cervical biopsy tissues. The study included 29 patients with bulky IB to IIA cervical squamous cell carcinoma treated with NAC.
Results:
Twenty (69.0%) of 29 patients were scored as p-STAT3-positive. Pathological response to chemotherapy (complete response or residual tumor with less than 3 mm stromal invasion) was observed in eight patients (27.6%). The p-STAT3-positive patients had a longer disease-free survival compared to p-STAT3-negative patients (P = 0.03), though they had more frequent clinical nodal involvement (P = 0.046).
Conclusion:
Pretreatment assessment of p-STAT3 expression may provide additional information for the identification of patients with cervical cancer who have a favorable prognosis.
RI lee, jw/O-6237-2014
TC 5
ZB 4
Z8 1
ZS 0
Z9 6
SN 1341-8076
UT WOS:000276020200013
PM 20492381
ER

PT J
AU Javle, Milind
Hsueh, Chung-Tsen
TI Recent advances in gastrointestinal oncology - updates and insights from the 2009 annual meeting of the American Society of Clinical Oncology
SO JOURNAL OF HEMATOLOGY & ONCOLOGY
VL 3

AR 11
DI 10.1186/1756-8722-3-11
PD MAR 23 2010
PY 2010
AB We have reviewed the pivotal presentations related to gastrointestinal malignancies from 2009 annual meeting of the American Society of Clinical Oncology with the theme of "personalizing cancer care". We have discussed the scientific findings and the impact on practice guidelines and ongoing clinical trials. Adding trastuzumab to chemotherapy improved the survival of patients with advanced gastric cancer overexpressing human epidermal growth factor receptor 2. Gemcitabine plus cisplatin has become a new standard for first-line treatment of advanced biliary cancer. Octreotide LAR significantly lengthened median time to tumor progression compared with placebo in patients with metastatic neuroendocrine tumors of the midgut. Addition of oxaliplatin to fluoropyrimidines for preoperative chemoradiotherapy in patients with stage II or III rectal cancer did not improve local tumor response but increased toxicities. Bevacizumab did not provide additional benefit to chemotherapy in adjuvant chemotherapy for stage II or III colon cancer. In patients with resected stage II colon cancer, recurrence score estimated by multigene RT-PCR assay has been shown to provide additional risk stratification. In stage IV colorectal cancer, data have supported the routine use of prophylactic skin treatment in patients receiving antibody against epidermal growth factor receptor, and the use of upfront chemotherapy as initial management in patients with synchronous metastasis without obstruction or bleeding from the primary site.
TC 18
ZB 6
Z8 1
ZS 0
Z9 19
SN 1756-8722
UT WOS:000276876600001
PM 20331897
ER

PT J
AU Mezhir, James J.
Tang, Laura H.

Coit, Daniel G.
TI Neoadjuvant Therapy of Locally Advanced Gastric Cancer
SO JOURNAL OF SURGICAL ONCOLOGY
VL 101
IS 4
BP 305
EP 314
DI 10.1002/jso.21483
PD MAR 15 2010
PY 2010
AB Treatment of gastric cancer has evolved with the advent of randomized trials demonstrating chemotherapeutic agents with efficacy in advanced disease. Level I evidence Supports delivering chemotherapy in the neoadjuvant settings the data shows improvement in progression-free and overall survival. A clinical response to therapy is associated with improved R0 resection rates, pathologic response. and outcome in patients with locally advanced disease. Early assessment of metabolic response to therapy can potentially be utilized to tailor treatment. J. Surg. Oncol. 2010; 101:305-314. (C) 2010 Wiley-Liss, Inc.
TC 21
ZB 12
Z8 7
ZS 0
Z9 28
SN 0022-4790
UT WOS:000275519900006
PM 20187070
ER

PT J
AU Biffi, Roberto
Fazio, Nicola
Luca, Fabrizio
Chiappa, Antonio
Andreoni, Bruno
Zampino, Maria Giulia
Roth, Arnaud
Schuller, Jan Christian
Fiori, Giancarla

Orsi, Franco
Bonomo, Guido
Crosta, Cristiano
Huber, Olivier
TI Surgical outcome after docetaxel-based neoadjuvant chemotherapy in locally-advanced gastric cancer
SO WORLD JOURNAL OF GASTROENTEROLOGY
VL 16
IS 7
BP 868
EP 874
DI 10.3748/wjg.v16.i7.868
PD FEB 21 2010
PY 2010
AB AIM: To investigate feasibility, morbidity and surgical mortality of a docetaxel-based chemotherapy regimen randomly administered before or after gastrectomy in patients suffering from locally-advanced resectable gastric cancer.
METHODS: Patients suffering from locally-advanced (T3-4 any N MO or any T N1-3 MO) gastric carcinoma, staged with endoscopic ultrasound, bone scan, computed tomography, and laparoscopy, were assigned to receive four 21 d/cycles of TCF (docetaxel 75 mg/m(2) day 1, cisplatin 75 mg/m(2) day 1, and fluorouracil 300 mg/m(2) per day for days 1-14), either before (Arm A) or after (Arm B) gastrectomy. Operative morbidity overall mortality, and severe adverse events were compared by intention-to-treat analysis.
RESULTS: From November 1999 to November 2005, 70 patients were treated. After preoperative TCF (Arm A), thirty-two (94%) resections were performed, 85% of which were R0. Pathological response was complete in 4 patients (11.7%), and partial in 18 (55%). No surgical mortality and 28.5% morbidity rate were observed, similar to those of immediate surgery arm (P = 0.86). Serious chemotherapy adverse events tended to be more frequent in arm B (23% vs 11%, P = 0.07), with a single death per arm.
CONCLUSION: Surgery following docetaxel-based chemotherapy was safe and with similar morbidity to immediate surgery in patients with locally-advanced resectable gastric carcinoma. (C) 2010 Baishideng. All rights reserved.
TC 25

ZB 12
Z8 13
ZS 2
Z9 40
SN 1007-9327
UT WOS:000274846400010
PM 20143466
ER

PT J
AU Yamasaki, Makoto
   Miyata, Hiroshi
   Fujiwara, Yoshiyuki
   Takiguchi, Shuji
   Nakajima, Kiyokazu
   Nishida, Toshirou
   Yasuda, Takushi
   Matsuyama, Jin
   Mori, Masaki
   Doki, Yuichiro
TI p53 Genotype Predicts Response to Chemotherapy in Patients with Squamous
   Cell Carcinoma of the Esophagus
SO ANNALS OF SURGICAL ONCOLOGY
VL 17
IS 2
BP 634
EP 642
DI 10.1245/s10434-009-0851-4
PD FEB 2010
PY 2010
AB Background. Response to chemotherapy and anatomical spread are
   significant prognostic factors in patients with esophageal squamous cell
   carcinoma (ESCC) treated by chemotherapy then surgery. Predicting the
   response to chemotherapy would allow significant optimization of cancer
   treatment.
   Methods. Genomic mutation and protein expression of p53 were
   investigated retrospectively by polymerase chain reaction (PCR)
   single-strand conformation polymorphism (SSCP) and immunohistochemistry
   (IHC) using biopsy specimens from 77 ESCC patients before chemotherapy

with 5-fluorouracil, adriamycin, and cisplatin. p53 status was correlated with various clinicopathological factors. Thereafter, we performed a prospective study of 20 consecutive patients to test our prediction model.
Results. The retrospective study showed mutant p53 genotype and positive p53 IHC staining in 46.8 and 55.8% of patients, respectively, which was not associated with patient's clinicopathological findings including initial tumor stage. Objective response to chemotherapy was observed in 65.9% of patients with wild genotype, but in only 16.7% of patients with mutant genotype. Patients with mutations in p53 therefore showed significantly poorer prognosis than those without mutant p53. In contrast, p53 IHC staining did not correlate with response to chemotherapy, curative resection rate or prognosis. In the prospective study, p53 mutation was seen in 50% (10/20) of patients and was again consistently associated with poorer response to chemotherapy and poorer prognosis.
Conclusions. p53 genotype of pretreatment biopsy is a potentially useful predictor of response to chemotherapy and prognosis in ESCC patients. This information might be valuable to clinicians in deciding on the optimal clinical strategy in patients with ESCC.

RI huang, simon/H-3756-2012

TC 17

ZB 8

Z8 0

ZS 0

Z9 17

SN 1068-9265

UT WOS:000274690900040

PM 19941080

ER

PT S

AU Lurje, Georg
   Lenz, Heinz-Josef

BE Schneider, PM

TI Molecular Response Prediction in Multimodality Treatment for Adenocarcinoma of the Esophagus and Esophagogastric Junction

SO Adenocarcinoma of the Esophagogastric Junction

SE Recent Results in Cancer Research

VL 182
BP 179
EP 191
DI 10.1007/978-3-540-70579-6_15
PD 2010
PY 2010
AB Cancers arising from the esophagus are becoming more common in the United States and Europe. In 2009, an estimate of 14,530 new cases will be diagnosed and more than 90% will die of their disease. Esophageal cancer is currently the most rapidly increasing cancer in the western world and is coinciding with a shift in histological type and primary tumor location. Despite recent improvements M the detection, surgical resection, and (radio-) chemotherapy, the overall survival (OS) of esophageal cancer remains relatively poor. It is becoming increasingly apparent that neoadjuvant chemoradiation followed by surgery may be beneficial in terms of increasing resectability and OS compared to surgery alone. Results from clinical trials are encouraging; however, they also demonstrated that only patients with major histopathological response (pCR) will benefit from neoadjuvant therapy. In addition, these therapies are expensive and the prognoses of patients who do not respond to trimodality treatment strategies appear to be inferior to that of patients who had surgery alone. Accordingly, the development of validated predictive molecular markers may not only be helpful in identifying EA patients who are more likely to respond, but they will also be critical in selecting more efficient treatment strategies with the means of a tailored, targeted, and effective therapy to the molecular profile of both the patient and their disease while minimizing and avoiding life-threatening toxicities.
TC 0
ZB 0
Z8 0
ZS 0
Z9 0
SN 0080-0015(print)
BN 978-3-540-70578-9(H)
UT BIOSIS:PREV201000539875
PM 20676881
ER

PT J

AU Cheong, Teak-Chin
Shin, Ji-Young
Chun, Kyung-Hee

TI Silencing of galectin-3 changes the gene expression and augments the sensitivity of gastric cancer cells to chemotherapeutic agents

SO CANCER SCIENCE

VL 101

IS 1

BP 94

EP 102

DI 10.1111/j.1349-7006.2009.01364.x

PD JAN 2010

PY 2010

AB Galectin-3 is known to modulate cell proliferation and apoptosis and is highly expressed in human cancers, but its function in gastric cancer is still controversial. Here, we examined the role of galectin-3 in gastric cancer cells by silencing it with synthetic double-stranded siRNA. After silencing of galectin-3, cell numbers decreased and cell shape changed. Galectin-3 siRNA treatment also induced G(1) arrest. DNA microarray analysis was used to assess changes in gene expression following galectin-3 silencing. We found that silencing of galectin-3 caused changes in gene expression. RT-PCR and real-time PCR were utilized for validation of the changes found in microarray studies. Western blot analysis confirmed changes in the expression of proteins of interest: cyclin D1, survivin, XIAP, XAF, PUMA, and GADD45 alpha. Generally, it tended to increase the expression of several pro-apoptotic genes, and to decrease the expression of cell cycle progressive genes. We also confirmed that changes in the expression of these genes were caused by galectin-3 overexpression. Finally, we demonstrated that silencing of galectin-3 enhanced apoptosis induction with chemotherapeutic agents by further reducing the expression of anti-apoptotic and/or cell survival molecules such as survivin, cyclin D1, and XIAP, and increasing the expression of pro-apoptotic XAF-1. We conclude that galectin-3 is involved in cancer progression and malignancy by modulating the expression of several relevant genes, and inhibition of galectin-3 may be an approach to improve chemotherapy of gastric cancers. (Cancer Sci 2009).

TC 17

ZB 17
Z8 2
ZS 0
Z9 19
SN 1347-9032
UT WOS:000272631500014
PM 19843071
ER

PT J
AU Schauer, Matthias
   Janssen, Klaus-Peter
   Rimkus, Caroline
   Raggi, Matthias
   Feith, Marcus
   Friess, Helmut
   Theisen, Joerg
TI Microarray-Based Response Prediction in Esophageal Adenocarcinoma
SO CLINICAL CANCER RESEARCH
VL 16
IS 1
BP 330
EP 337
DI 10.1158/1078-0432.CCR-09-1673
PD JAN 1 2010
PY 2010
AB Purpose: In locally advanced (uT(3), N(+)) adenocarcinomas of the
   esophagus, neoadjuvant chemotherapy improves patient outcome. However,
   only a subgroup of patients responds. Therefore, in the present study,
   we evaluated whether the response to neoadjuvant chemotherapy can be
   predicted by a pretreatment tumor biopsy analysis.
   Experimental Design: Biopsies of 47 patients with locally advanced (uT3,
   N+) adenocarcinoma of the esophagus were obtained during primary
   staging. All patients underwent neoadjuvant chemotherapy with cisplatin,
   5-fluorouracil, and leucovorin and subsequent resection of the
   esophagus. Biopsies were used for microarray analysis. The predominance
   of tumor cells within the specimens was >70%. Affymetrix U133 plus 2.0
   gene chips with 54675 probe sets were used. A statistical comparison of
   patients responding to chemotherapy versus nonresponding patients was

done. All patients were examined with immunohistology against Ephrin B3 receptor and Ki-67.
Results: A total of 86 genes were at least 2-fold differentially regulated comparing responding with nonresponding adenocarcinomas of the esophagus. The predominant genes encoded for the regulation of the cell cycle, transduction, translation, cell-cell interaction, cytoskeleton, and the signal transduction. The strongest difference was seen for the Ephrin B3 receptor. This result could be confirmed by immun-histology. A statistical significant correlation between the Ephrin B3 receptor, chemotherapy response, pathologic staging, and grading could be shown.
Conclusions: There were significant differences in the gene profile between patients with adenocarcinoma of the esophagus responding to neoadjuvant chemotherapy compared with nonresponding patients. This suggests that it could be possible to characterize patients responding to chemotherapy even before starting the treatment using customized microarray analysis. Clin Cancer Res; 16(1);330-7. (C) 2010 AACR.
TC 23
ZB 11
Z8 3
ZS 0
Z9 26
SN 1078-0432
UT WOS:000278404500033
PM 20028767
ER

PT J
AU Rivera, Fernando
Galan, Maica
Tabernero, Josep
Cervantes, Andres
Eugenia Vega-Villegas, M.
Gallego, Javier
Laquente, Berta
Rodriguez, Edith
Carrato, Alfredo
Escudero, Pilar
Massuti, Bartomeu
Alonso-Orduna, Vicente

Cardenal, Adelaida
Saenz, Alberto
Giralt, Jordi
Lucia Yuste, Ana
Anton, Antonio
Aranda, Enrique
CA Spanish Cooperative Grp Digestive
TI PHASE II TRIAL OF PREOPERATIVE IRINOTECAN-CISPLATIN FOLLOWED BY CONCURRENT IRINOTECAN-CISPLATIN AND RADIOTHERAPY FOR RESECTABLE LOCALLY ADVANCED GASTRIC AND ESOPHAGOGASTRIC JUNCTION ADENOCARCINOMA
SO INTERNATIONAL JOURNAL OF RADIATION ONCOLOGY BIOLOGY PHYSICS
VL 75
IS 5
BP 1430
EP 1436
DI 10.1016/j.ijrobp.2008.12.087
PD DEC 1 2009
PY 2009
AB Purpose: To determine in a Phase H trial whether preoperative irinotecan-cisplatin (IC) followed by concurrent IC therapy and radiotherapy (IC/RT) improved outcome in patients with resectable, locally advanced gastric adenocarcinoma (GC) or esophagogastric junction cancer (EGJC).
Patients and Methods: Patients with resectable Stage II-IV, M0 GC or EGJC made up the study population. The primary endpoint was pathologic complete response (pCR). Two courses of IC (irinotecan, 65 mg/m(2); cisplatin, 30 mg/m(2) on Days I and 8 every 21 days) were given. Patients without progression then received IC/RT, consisting of daily radiotherapy (45Gy) with concurrent IC (irinotecan, 65 mg/m(2); cisplatin, 30 mg/m(2) on Days 1, 8, 15, and 22). Surgical resection was performed, if feasible, 5-8 weeks after the end of radiotherapy.
Results: Twenty-three patients were included in the study: 10 with EGJC and 13 with GC. Two patients (9 %) achieved pCR. The incidences of Grade 3-4 toxicities were as follows: IC: neutropenia 35 % (febrile 13 %), anemia 22 %, diarrhea 22 %, emesis 8 %; IC/RT: neutropenia 52 % (febrile 5 %), asthenia 19 %, anemia 9 %, emesis 9 %, diarrhea 5 %, cardiotoxicity 5 %. No patients died during IC or IC/RT. R0 resection was achieved in 15 patients (65 %). Median survival was 14.5 months, and the actuarial 2-year survival rate was 35 %.

Conclusions: Preoperative IC followed by IC/RT resulted in moderate response and resection rates with mild toxicity in-patients with GC and EGJC. (C) 2009 Elsevier Inc.
TC 11
ZB 4
Z8 2
ZS 0
Z9 13
SN 0360-3016
UT WOS:000272341800022
PM 19540072
ER

PT J
AU Morimoto, Junya
Oohira, Masaichi
Kubo, Naoshi
Tanaka, Hiroaki
Dan, Nobuhiro
Muguruma, Kazuya
Yashiro, Masakazu
Sawada, Tetsuji
Yamashita, Yoshito
Nishiguchi, Yukio
Hirakawa, Kousei
TI [A case of stage IV advanced esophageal cancer with a long term survival by radiation therapy combined with nedaplatin and 5-FU chemotherapy].
SO Gan to kagaku ryoho. Cancer & chemotherapy
VL 36
IS 12
BP 2436
EP 8
PD 2009-Nov
PY 2009
AB The patient was a 73-year-old man who complained of dysphagea. Various examinations revealed an esophageal cancer with direct invasion to the left main bronchus (cT4, N2 (104R, 106recR), M0, Stage IVa) and gastric cancer (cT2, N0, M0, Stage IB). The patient was given preoperative chemoradiotherapy (40 Gy/20 fr with CDGP 10 mg/body day 1-5, 8- 12,

15-19 and 5-FU 250 mg/body day 1-5, 8-12, 15-19). After the chemoradiotherapy, we estimated that the esophageal cancer was down stage (cT4-->T3), and that a curative operation was possible. Therefore, subtotal esophagectomy and partial gastrectomy were performed without a complication. Pathological therapeutic evaluation of the esophageal cancer was complete response (CR) and the gastric cancer was T2, N0. Adjuvant chemotherapy was undergone with S-1. However, two years after the first operation, we found a recurrence of gastric duct. Therefore a surgical resection for recurrence of gastric duct was performed. The patient is still alive without recurrence 5 years and 2 months after the first treatment. Radiation therapy combined with nedaplatin and 5-FU is a safe and effective method for treating cT4 advanced esophageal cancer.
TC 0
ZB 0
Z8 0
ZS 0
Z9 0
SN 0385-0684
UT MEDLINE:20037448
PM 20037448
ER

PT J
AU Fushida, Sachio
Fujimura, Takashi
Oyama, Katsunobu
Yagi, Yasumichi
Kinoshita, Jun
Ohta, Tetsuo
TI Feasibility and efficacy of preoperative chemotherapy with docetaxel, cisplatin and S-1 in gastric cancer patients with para-aortic lymph node metastases
SO ANTI-CANCER DRUGS
VL 20
IS 8
BP 752
EP 756
DI 10.1097/CAD.0b013e32832ec02b
PD SEP 2009

PY 2009
AB We performed preoperative chemotherapy with combined docetaxel, cisplatin and S-1 (DCS therapy) for treatment of advanced gastric cancer with para-aortic lymph node metastases. The aim of this study was to determine the maximum tolerated dose (MTD) and the dose-limiting toxicities. Furthermore, we evaluated the feasibility of DCS therapy in a preoperative setting, and also examined the pathological response. Fifteen patients received intravenous docetaxel and cisplatin (30, 35 or 40 mg/m(2), each dose escalation was reciprocal) on days 1 and 15 and oral S-1 (40 mg/m(2) twice daily) on days 1-14 every 4 weeks. After one cycle of chemotherapy, toxicities were evaluated and after two cycles of chemotherapy, patients who were judged to be candidates for curative resection underwent gastrectomy with D2 lymphadenectomy plus para-aortic lymph node dissection. The MTD of this combination was presumed to be at dose level 3 (docetaxel 40 mg/m(2) and cisplatin 35 mg/m(2)). The dose-limiting toxicities were grade 4 neutropenia in one patient grade 3 febrile neutropenia in two patients and grade 3 diarrhoea in two patients. Thirteen of the 15 patients received complete resection and there was no operation-related death. Good pathological responses were observed in 12 cases with lesions in the lymph nodes (complete response, n=4; partial response, n=8) and 11 patients with primary stomach lesions (complete response, n=2; partial response, n=9). This preoperative DCS therapy was considered feasible and provided a high pathological response rate in gastric cancer patients with para-aortic lymph node metastases. Anti-Cancer Drugs 20:752-756 (C) 2009 Wolters Kluwer Health | Lippincott Williams & Wilkins.
TC 13
ZB 8
Z8 0
ZS 0
Z9 13
SN 0959-4973
UT WOS:000268732100013
PM 19543076
ER

PT J
AU Pavlovsky, Carolina
Egorin, Merrill J.

Shah, Dhvani D.
Beumer, Jan H.
Rogel, Silvia
Pavlovsky, Santiago
TI Imatinib Mesylate Pharmacokinetics Before and After Sleeve Gastrectomy in a Morbidly Obese Patient with Chronic Myeloid Leukemia
SO PHARMACOTHERAPY
VL 29
IS 9
BP 1152
EP 1156
PD SEP 2009
PY 2009
AB Imatinib is widely used to treat chronic myeloid leukemia and gastrointestinal stromal tumors. The agent, administered orally, has approximately 98% oral bioavailability, achieves maximum plasma concentration approximately 2-4 hours after ingestion, and has a plasma half-life of approximately 18 hours. As maintaining an adequate plasma imatinib concentration is essential to achieving a favorable therapeutic response, it is important to determine whether gastrointestinal surgery, pathologic conditions, or anatomic changes negatively affect imatinib absorption, and thereby result in subtherapeutic plasma imatinib concentrations. We describe a 36-year-old, morbidly obese woman with chronic myeloid leukemia who received treatment with alpha-interferon and cytarabine over 5 years. Her chemotherapy was then switched to imatinib 400 mg/day because she failed to achieve a molecular response with the other two agents. A complete molecular response was achieved with imatinib. Four years later, she underwent a sleeve gastrectomy while receiving imatinib. Imatinib plasma pharmacokinetic values were assessed before and on four occasions during the year after the sleeve gastrectomy. The patient's trough plasma concentration before surgery (1558 ng/ml) was consistent with those found in the literature (>= 1000 ng/ml), whereas her trough concentrations after surgery were 46-60% lower (629-836 ng/ml) than the preoperative value. Despite this, the patient remained in complete molecular remission for I year after surgery. Monitoring plasma imatinib concentrations is recommended in morbidly obese patients with chronic myeloid leukemia or gastrointestinal stromal tumors who undergo gastric procedures. Additional pharmacokinetic studies, however, are needed in these

patients.
TC 15
ZB 11
Z8 0
ZS 0
Z9 15
SN 0277-0008
UT WOS:000269437500013
PM 19698017
ER

PT J
AU Liu, Yin-hua
TI [A prospective study of FOLFOX7 scheme as neoadjuvant chemotherapy for stage III gastric adenocarcinoma].
SO Zhonghua wai ke za zhi [Chinese journal of surgery]
VL 47
IS 17
BP 1305
EP 8
PD 2009-Sep-1
PY 2009
AB OBJECTIVE: To evaluate the efficacy and safety of FOLFOX7 scheme as neoadjuvant chemotherapy in patients with stage III gastric adenocarcinoma.
METHODS: From May 2005 to May 2007, 27 patients with stage III gastric adenocarcinoma were given neoadjuvant chemotherapy with FOLFOX7 scheme. Gastroscopy, endoscopic ultrasonography, abdominal B ultrasonography and abdominal CT was taken before chemotherapy and after 2 - 4 cycles of neoadjuvant chemotherapy to evaluate the objective response rate of the tumor. Then operations were carried out and the pathological responses was evaluated in those cases. The safety, objective response rate and pathological rate of neoadjuvant chemotherapy was assessed according to NCI-CTC v3.0, RECIST 2000, and the criteria established by Japanese Research Society for Gastric Cancer, respectively. R0 resection rate and surgery-related complications was also assessed in this group.
RESULTS: The treatment was well tolerated, no grade 3 - 5 toxicity was observed. Complete response was obtained in 1 case, and partial response in 18 patients, overall response rate was 70.4% (19/27). Twenty-six

patients received operation and R0 resection rate was 88.4% (23/26); no patient died in the perioperative period. The pathological response rate of patients had R0 excision was 60.9% (14/23).
CONCLUSION: FOLFOX7 scheme as neoadjuvant chemotherapy for selected patients with stage III gastric adenocarcinoma can be well tolerated, it could induce tumor down-staging and improve R0 resection rate, although the long term efficacy remains to be evaluated.
TC 0
ZB 0
Z8 0
ZS 0
Z9 0
SN 0529-5815
UT MEDLINE:20092724
PM 20092724
ER

PT J
AU Jhawer, Minaxi
Coit, Daniel
Brennan, Murray
Qin, Li-Xuan
Gonen, Mithat
Klimstra, David
Tang, Laura
Kelsen, David P.
Shah, Manish A.
TI Perineural Invasion After Preoperative Chemotherapy Predicts Poor Survival in Patients With Locally Advanced Gastric Cancer Gene Expression Analysis With Pathologic Validation
SO AMERICAN JOURNAL OF CLINICAL ONCOLOGY-CANCER CLINICAL TRIALS
VL 32
IS 4
BP 356
EP 363
DI 10.1097/COC.0b013e31818c08e8
PD AUG 2009
PY 2009
AB Background: We examined gene expression profiles and clinicopathologic

features (tumor location, stage, graded pathologic response, perineural invasion (PNI), Lauren's classification, and survival) of patients with gastric cancer who received preoperative chemotherapy to identify prognostic markers.
Methods: Thirty-eight patients with locally advanced gastric cancer received preoperative chemotherapy on a phase II trial. Twelve fresh-frozen tumor samples were available for RNA expression analysis. Differential gene expression between tumors with and without PNI was identified and correlated with clinicopathologic features.
Results: Preliminary hierarchical clustering suggested a separation between long- and short-term survivors. The close association between PNI and overall survival was identified and validated immunohistochemically in 31 completely resected gastric tumors. Five-year survival for patients with PNI and without PNI was 5% and 65%, respectively (P < 0.01). PNI added significant prognostic value to posttreatment pathologic stage, (P < 0.01). Differential gene expression profile for PNI and non-PNI tumors identified 111 potentially relevant genes.
Conclusions: Our results demonstrate that the presence of PNI after preoperative chemotherapy is associated with poor survival. These results need to be validated in prospective studies, to help establish whether patients with evidence of. PNI would be candidates for more aggressive therapy or enrollment into clinical trials. The presence of PNI provides additional prognostic importance to posttreatment pathologic stage and may indicate treatment resistance. Understanding the molecular events associated with PNI, may provide insight into new therapeutic agents for this subset of patients with resistant tumors.
RI Gonen, Mithat/E-4826-2012
TC 8
ZB 4
Z8 2
ZS 0
Z9 10
SN 0277-3732
UT WOS:000268761600006
PM 19381079
ER

PT J

AU Mansour, John C.
   Schwarz, Roderich E.
TI Pathologic Response to Preoperative Therapy: Does It Mean What We Think
   It Means?
SO ANNALS OF SURGICAL ONCOLOGY
VL 16
IS 6
BP 1465
EP 1479
DI 10.1245/s10434-009-0374-z
PD JUN 2009
PY 2009
AB Tumors treated with preoperative chemotherapy or radiation often
   demonstrate evidence of response in the resection specimen. Pathologic
   response is often interpreted as a surrogate for recurrence or survival
   outcomes. With a valid surrogate for long-term outcomes, investigators
   can explore treatment strategies with immediate endpoints. The financial
   and time costs of trials measuring recurrence and survival may be
   lessened by using short-term pathologic outcomes as an endpoint. This
   review is intended to examine the current status of pathologic response
   as a surrogate for tumor behavior. We address questions regarding the
   definitions of pathologic response, as well as the current literature
   regarding the meaning of pathologic response for some common tumor
   types. We explore some of the potential confounding effects that may
   explain the frequent discordance between tumor response and patient
   outcomes. In addition, we address some alternative strategies to gauge
   the response of a tumor to preoperative therapy.
TC 3
ZB 3
Z8 0
ZS 0
Z9 3
SN 1068-9265
UT WOS:000265787200007
PM 19326170
ER

PT J
AU Starling, N.

Okines, A.
Cunningham, D.
Allum, W.
Wotherspoon, A.
Benson, M.
Thompson, J.
Thomas, J.
Brown, G.
Riddell, A.
Stavridi, F.
Ashley, S.
Oates, J.
Chau, I.
TI A phase II trial of preoperative chemotherapy with epirubicin, cisplatin and capecitabine for patients with localised gastro-oesophageal junctional adenocarcinoma
SO BRITISH JOURNAL OF CANCER
VL 100
IS 11
BP 1725
EP 1730
DI 10.1038/sj.bjc.6605070
PD MAY 26 2009
PY 2009
AB Preoperative cisplatin/fluorouracil is used for the treatment of localised oesophageal carcinoma. This phase II study aimed to assess the efficacy and safety of administering preoperative epirubicin/cisplatin/capecitabine (ECX). Patients with stage II or III oesophageal/gastro-oesophageal junctional adenocarcinoma from one institution received 4 cycles of ECX (epirubicin 50 mg m(-2) day 1, cisplatin 60 mg m(-2) day 1, capecitabine 625 mg m(-2) b.i.d. daily) followed by surgery. The primary end point was the pathological complete response (pCR) rate based on a Simon two-stage design. Secondary end points included overall and progression-free survival (OS/PFS). Thirty-four patients were recruited: median age 60 years (range 41-81), 91% male, 97% PS 0/1, 80% T3, 68% N1. Thirty-one patients completed four ECX cycles. Grade 3/4 toxicities >= 5% included neutropenia (62%), hand-foot syndrome (15%) and nausea/vomiting (9%). Thirteen out of 28 (46%) evaluable patients responded to chemotherapy by EUS (>= 30%

reduction in maximal tumour thickness). Twenty-six out of 34 (76%) patients underwent resection (R0 = 73%, R1 = 27%). Post-operatively, two patients died within 60 days of surgery. The pCR rate was 5.9% (95% CI 0-14%) in the intent-to-treat population. According to the statistical design, this prompted early study termination. However, with a median follow-up of 34 months the median OS and 1- and 2-year survival rates were 17 months, 67 and 39% respectively. Median PFS was 13 months. Of the 14 relapsed patients, 10 presented with distant metastases. Preoperative ECX is feasible and well tolerated. Although associated with a low pCR rate, survival with ECX was comparable with published studies suggesting that pCR may not correlate with satisfactory outcome from preoperative chemotherapy for localised oesophageal adenocarcinoma. British Journal of Cancer (2009) 100, 1725-1730. doi: 10.1038/sj.bjc.6605070 www.bjcancer.com Published online 12 May 2009 (C) 2009 Cancer Research UK
TC 8
ZB 4
Z8 1
ZS 0
Z9 9
SN 0007-0920
UT WOS:000266517600004
PM 19436301
ER

PT J
AU Sarkaria, Inderpal S.
Rizk, Nabil P.
Bains, Manjit S.
Tang, Laura H.
Ilson, David H.
Minsky, Bruce I.
Rusch, Valerie W.
TI Post-treatment Endoscopic Biopsy Is a Poor-Predictor of Pathologic Response in Patients Undergoing Chemoradiation Therapy for Esophageal Cancer
SO ANNALS OF SURGERY
VL 249
IS 5

BP 764
EP 767
DI 10.1097/SLA.0b013e3181a38e9e
PD MAY 2009
PY 2009
AB Purpose: Endoscopic biopsy after chemoradiation therapy (CRT) for esophageal cancer has been used to determine response to treatment. We wanted to determine if endoscopic biopsy can accurately establish evidence of local pathologic complete response (pCR) in patients undergoing CRT.
Methods: We queried a prospectively maintained database for patients seen at Memorial Sloan-Kettering Cancer Center from 1996 to the present who underwent, (1) CRT for local-regionally advanced esophageal cancer, (2) post-CRT endoscopic biopsy, and (3) esophagectomy. Data points included pathology of post-CRT endoscopy and surgical specimens, tumor histology, and survival. Correlations were analyzed by the chi(2) test and one-way analysis of variance. Survival comparisons were assessed using the Kaplan-Meier method and log-rank analysis.
Results: One hundred fifty-six patients were identified. Over 80% of patients received cisplatin-based chemotherapy and 5040 cGy of radiation. One hundred eighteen patients had no tumor identified on endoscopic biopsy. A negative biopsy at endoscopy was a poor predictor of pCR (negative predictive value: 31%), with 69% having local disease at esophagectomy. A positive biopsy was predictive of residual disease (positive predictive value: 95%). Negative endoscopic biopsy better predicted a pCR for squamous cell carcinomas versus adenocarcinomas (P[r] < 0.001). Nodal status of surgical specimens was not correlated with post-treatment endoscopic findings. Survival was equivalent after surgery in patients with a negative endoscopic biopsy versus patients with positive pathology.
Conclusion: A negative endoscopic biopsy is not a useful predictor of a pCR after CRT, final nodal status, or overall survival.
CT 42nd Annual Meeting of the American-Society-of-Clinical-Oncology
CY JUN 02-06, 2006
CL Atlanta, GA
SP Amer Soc Clin Oncol
TC 26
ZB 6
Z8 0

ZS 0
Z9 26
SN 0003-4932
UT WOS:000265760400014
PM 19387328
ER

PT J
AU Ku, Geoffrey Y.
Ilson, David H.
TI Preoperative Therapy for Esophageal Cancer
SO GASTROENTEROLOGY CLINICS OF NORTH AMERICA
VL 38
IS 1
BP 135
EP +
DI 10.1016/j.gtc.2009.01.012
PD MAR 2009
PY 2009
AB This article examines the role of combined-modality therapy for treating locally advanced esophageal cancer. Although surgery remains a cornerstone of treatment, recent studies have demonstrated that pre- or perioperative chemotherapy is associated with improved survival for patients who have adenocarcinoma histology. Primary chemoradiotherapy is the accepted standard of care for medically inoperable patients. Recent studies also suggest that definitive chemoradiotherapy is acceptable for patients who have squamous histology, while subsequent surgery improves local control without conferring a clear survival benefit. Neoadjuvant chemoradiotherapy continues to be investigated but is associated with several advantages over neoadjuvant chemotherapy alone, including an improvement in the pathologic complete response rate and resectability. Patients who achieve a pathologic complete response also appear to have improved survival. Adjuvant chemoradiotherapy may be considered for patients who undergo primary resection of lower esophageal/gastroesophageal junction adenocarcinoma.
TC 7
ZB 3
Z8 0
ZS 0

Z9 7
SN 0889-8553
UT WOS:000265370100011
PM 19327572
ER

PT J
AU Stahl, Michael
Walz, Martin K.
Stuschke, Martin
Lehmann, Nils
Meyer, Hans-Joachim
Riera-Knorrenschild, Jorge
Langer, Peter
Engenhart-Cabillic, Rita
Bitzer, Michael
Koenigsrainer, Alfred
Budach, Wilfried
Wilke, Hansjochen
TI Phase III Comparison of Preoperative Chemotherapy Compared With Chemoradiotherapy in Patients With Locally Advanced Adenocarcinoma of the Esophagogastric Junction
SO JOURNAL OF CLINICAL ONCOLOGY
VL 27
IS 6
BP 851
EP 856
DI 10.1200/JCO.2008.17.0506
PD FEB 20 2009
PY 2009
AB Purpose
Preoperative chemotherapy is an accepted standard in the treatment of localized esophagogastric adenocarcinoma. Adding radiation therapy to preoperative chemotherapy appears promising, but its definitive value remains unknown.
Patients and Methods
Patients with locally advanced (uT3-4NXM0) adenocarcinoma of the lower esophagus or gastric cardia were randomly allocated to one of two treatment groups: induction chemotherapy (15 weeks) followed by surgery

(arm A); or chemotherapy (12 weeks) followed by chemoradiotherapy (3 weeks) followed by surgery (arm B). Primary outcome was overall survival time. A total of 354 patients were needed to detect a 10% increase in 3-year survival from 25% to 35% by addition of radiation therapy. The study was prematurely closed due to low accrual.
Results
The median observation time was 46 months. A total of 126 patients were randomly assigned and 119 eligible patients were evaluated. The number of patients undergoing complete tumor resection was not different between treatment groups (69.5% v 71.5%). Patients in arm B had a significant higher probability of showing pathologic complete response (15.6% v 2.0%) or tumor-free lymph nodes (64.4% v 37.7%) at resection. Preoperative radiation therapy improved 3-year survival rate from 27.7% to 47.4% (log-rank P = .07, hazard ratio adjusted for randomization strata variables 0.67, 95% CI, 0.41 to 1.07). Postoperative mortality was nonsignificantly increased in the chemoradiotherapy group (10.2% v 3.8%; P = .26).
Conclusion
Although the study was closed early and statistical significance was not achieved, results point to a survival advantage for preoperative chemoradiotherapy compared with preoperative chemotherapy in adenocarcinomas of the esophagogastric junction.

CT 43rd Annual Meeting of the American-Society-of-Clinical-Oncology
CY JUN 01-05, 2007
CL Chicago, IL
SP Amer Soc Clin Oncol
TC 225
ZB 71
Z8 24
ZS 2
Z9 252
SN 0732-183X
UT WOS:000263513000005
PM 19139439
ER

PT J
AU Javeri, Heta
Xiao, Lianchun

Rohren, Eric
Komaki, Ritsuko
Hofstetter, Wayne
Lee, Jeffrey H.
Maru, Dipen
Bhutani, Manoop S.
Swisher, Stephen G.
Wang, Xuemei
Ajani, Jaffer A.
TI Influence of the Baseline 18F-Fluoro-2-deoxy-D-glucose Positron Emission Tomography Results on Survival and Pathologic Response in Patients With Gastroesophageal Cancer Undergoing Chemoradiation
SO CANCER
VL 115
IS 3
BP 624
EP 630
DI 10.1002/cncr.24056
PD FEB 1 2009
PY 2009
AB BACKGROUND: In patients with esophageal cancer who receive chemoradiation, tools to predict/prognosticate outcome before administering therapy are lacking. The authors evaluated initial standardized unit value (iSUV) of 18F-fluoro-2-deoxy-D-glucose positron emission tomography and its association with overall survival and the degree of pathologic response after surgery. METHODS: The authors analyzed 161 patients with esophageal adenocarcinoma who had chemoradiation followed by surgery. The log-rank test, univariate Cox proportional hazards model, Kaplan-Meier survival plot, and Fisher exact test were used to analyze dichotomized iSUV and its association with overall survival and pathologic response. RESULTS: The median age of 161 patients was 61 years (range, 26-80 years) and the majority of patients had lower esophageal or gastroesophageal junction involvement. All patients received fluoropyrimidine and, most commonly, a taxane or platinum compound with concomitant radiation. The median radiation dose was 45 grays (Gy) (range, 45 Gy-50.4 Gy). The median iSUV for all patients was 10.1 (range, 0-58). Using the Fisher exact test, iSUV was not found to be associated with the location of the primary cancer. iSUV higher than the median (10.1) was associated with a better pathologic

response (P = .06). Patients with primary cancer with iSUV > 10.1 had a lower risk for death (hazards ratio of 0.56) compared with those with iSUV < 10.1. Higher iSUV was nonsignificantly associated with improved survival (P = .07). CONCLUSIONS: Data from the current study suggest that lower iSUV is associated with poor survival and lower probability of response to chemoradiation. iSUV needs to be further evaluated because it may be used to complement other imaging or biomarker assessments to individualize therapy. Cancer 2009;115:624-30. (c) 2009 American Cancer Society.
TC 15
ZB 8
Z8 1
ZS 0
Z9 16
SN 0008-543X
UT WOS:000263003400021
PM 19130466
ER

PT J
AU Krasna, Mark J.
TI Stage-Specific Therapy for Cancer of the Oesophagus A New 'Cancer of the Elderly'
SO DRUGS & AGING
VL 26
IS 3
BP 185
EP 194
PD 2009
PY 2009
AB Oesophageal and gastric cancers are amongst the most frequent and lethal of cancers worldwide. In the US alone, some 13 000 individuals are affected each year, and mortality is particularly high in elderly patients with advanced stage disease and multiple co-morbidities. Patients usually do not present until later in the disease when symptoms occur, once the tumour is sufficiently large to cause obstruction or invasion of adjacent structures. Oesophageal cancer can metastasize to almost any organ, and widespread distant metastases are almost always present at the time of death. Overall mortality from this cancer is

around 80-90%.
Curative treatment of oesophageal cancer must achieve local control of the primary lesion as well as control and/or prevention of metastases. These are important contributors to overall results when therapy is undertaken in elderly patients, as are the significant risks of adverse effects such as morbidity from chemoradiation and the morbidity and mortality of oesophagectomy. Surgical resection affords the best chance for local control and the best means of palliation of dysphagia for most patients with localized disease, although both local and systemic recurrence of disease are common when surgery is used alone.
Because of the low cure rates associated with the use of surgery alone, other modalities have been added to the treatment regimen. Elderly patients with significant cardiac and pulmonary co-morbidity are candidates for nonoperative therapy, even at an early disease stage. There are few data to support a survival advantage from adjuvant radiotherapy or chemotherapy following complete resection, in the absence of documented metastatic disease. Chemotherapy and radiotherapy have both been reported to improve survival when administered preoperatively in patients with oesophageal cancer, while current data using trimodal therapy show a trend towards increased treatment-related mortality with only a slight increase in overall survival. There is currently no completely reliable preoperative method for restaging patients following neoadjuvant chemoradiation in order to assess pathological complete response. Novel restaging techniques are therefore required, in addition to further study of the risks and benefits of neoadjuvant chemoradiotherapy for this disease.
TC 0
ZB 0
Z8 0
ZS 0
Z9 0
SN 1170-229X
UT WOS:000265717200001
PM 19358615
ER

PT J
AU Okabe, Toshio
Ohya, Toshihiro

Matsumoto, Hiroshi
Tago, Ken-Ichi
Totsuka, Osamu
Numaga, Yuki
Higuchi, Toru
Iesato, Hiroshi
Yokomori, Tadahiro
Kawate, Susumu
Takeyoshi, Izumi
TI [A case of complete response for advanced gastric cancer with liver metastasis treated with combination chemotherapy of weekly paclitaxel and doxifluridine].
SO Gan to kagaku ryoho. Cancer & chemotherapy
VL 36
IS 1
BP 115
EP 8
PD 2009-Jan
PY 2009
AB A 68-year-old man underwent total gastrectomy for Type 3 gastric cancer with liver metastasis. The final finding was T3(SE), N1, H1, P0, CY0(class IV), Stage IV, Cur C. After surgery, he was treated with combination chemotherapy of weekly paclitaxel(PTX)/doxifluridine(5'-DFUR). Paclitaxel was administered at a dose of 80 mg/m(2) on day 1, 8 and 15, and doxifluridine was orally administered at a dose of 533 mg/m(2) day for five days followed by withdrawal for two days. This regimen was repeated every four weeks. After 2 courses, the tumor marker level normalized, and the size of the liver metastasis was remarkably decreased. After 5 courses, a CT scan revealed the liver metastasis had disappeared, and he has now survived without recurrence after the disappearance of the liver metastasis. No severe adverse reactions were observed, and the man can be treated as an outpatient. This therapy may thus be effective in the treatment of advanced gastric cancer following non-curative operation.
TC 0
ZB 0
Z8 0
ZS 0
Z9 0

SN 0385-0684
UT MEDLINE:19151575
PM 19151575
ER

PT J
AU 김승업
Seong, Jinsil
표주연
Kim, Hoguen
형우진
송시영
TI A case of pathologic complete remission of advanced gastric cancer induced by concurrent chemoradiation with S1 and cisplatin
Z1 수술전 S1 과 cisplatin 병합 동시 항암화학-방사선요법 후 병리학적 완전관해를 확인한 진행성 위암 1 예
SO The Korean Journal of Medicine
S1 대한내과학회지
VL 76
IS 3
BP 343
EP 347
PD 2009
PY 2009
AB Although the required extent of lymph node dissection remains controversial, surgery is the cornerstone of the treatment of advanced gastric cancer. However, only approximately 30% of patients are diagnosed as operable, and an R0 resection will be achieved in only 40~60% of these. Since R0 resection and the treatment response of the primary cancer or resected specimen are significant prognostic factors in locally advanced gastric cancer, various preoperative treatment modalities have been attempted to induce downstaging and improve complete nodal resection. Several recent studies revealed that preoperative chemoradiation therapy can prolong patient survival by

improving the R0 resection rate and treatment response. Here, we present an advanced gastric cancer patient with serosal penetration involving multiple perigastric and celiac lymph nodes who underwent radical surgery and entered complete remission after S1 and cisplatin-based concurrent chemoradiation therapy. Pathology revealed total necrosis of the tumor cells, and fibrous nodules in 2 out of 47 resected lymph nodes indicated dead cancer cells due to chemoradiation therapy. Subsequently, the patient received an additional six rounds of postoperative adjuvant chemotherapy with uracil/tegafur (UFT) and cisplatin. Follow-up imaging showed no evidence of tumor recurrence.
AK 위암의 수술 방법에 대해서는 논란의 여지가 있지만 아직까지 수술이 국소적인

진행성 위암의 근치적인 치료로 첫 번째 방법이다.

하지만 위암을 진단 받은 환자들 중에서 약 30%만이 수술이 가능한 진행성

위암으로 진단을 받으며 그 중 약 40~60%만이 R0

절제술이 가능하다. 저자들은 위 주위 림프절과 복강림프절, 장막을 침범한

진행성 위암을 진단 받은 환자를 5 주간의 동시 항암

화학-방사선 요법으로 치료하였고, 근치적인 목적으로 부분 위 절제술을

시행하였으며 조직병리 검사에서 완전히 괴사된 종양 조직을

관찰할 수 있었고, 수술 후 6 차례의 추가적인 전신 항암화학요법을 시행하였으며

추적 복부전산화단층촬영에서 재발을 시사하는 소견을

보이지 않았다.
TC 0
ZB 0
Z8 0
ZS 0
Z9 0
SN 1226-329X
UT KJD:ART001322506
ER

PT J

AU Ku, Geoffrey Y.
Ilson, David H.
TI Multimodality therapy for the curative treatment of cancer of the esophagus and gastroesophageal junction
SO EXPERT REVIEW OF ANTICANCER THERAPY
VL 8
IS 12
BP 1953
EP 1964
DI 10.1586/14737140.8.12.1953
PD DEC 2008
PY 2008
AB This review examines the role of combined-modality therapy in the treatment of locally advanced esophageal cancer. While surgery remains a cornerstone of treatment, recent studies have demonstrated that pre- or perioperative chemotherapy is associated with improved survival. Primary chemoradiotherapy is the accepted standard of care for medically inoperable patients. Neoadjuvant chemoradiotherapy continues to be investigated and is associated with several advantages over neoadjuvant chemotherapy alone, including an improvement in the pathologic complete response rate and resectability; patients who achieve a pathologic complete response also appear to have improved survival. Adjuvant chemoradiotherapy may be considered for patients who undergo primary resection of lower esophageal/gastroesophageal junction adenocarcinoma. Future directions include the investigation of novel chemotherapy regimens, the addition of targeted therapies and the use of PET to provide an early assessment of response.
TC 1
ZB 0
Z8 0
ZS 0
Z9 1
SN 1473-7140
UT WOS:000262015500017
PM 19046115
ER

PT J
AU Ougolkov, Andrei V.

Bilim, Vladimir N.
Billadeau, Daniel D.
TI Regulation of Pancreatic Tumor Cell Proliferation and Chemoresistance by the Histone Methyltransferase Enhancer of Zeste Homologue 2
SO CLINICAL CANCER RESEARCH
VL 14
IS 21
BP 6790
EP 6796
DI 10.1158/1078-0432.CCR-08-1013
PD NOV 1 2008
PY 2008
AB Purpose: Enhancer of zeste homologue2 (EZH2), a histone methyltransferase, plays a key role in transcriptional repression through chromatin remodeling. Our objectives were to determine the expression pattern of EZH2 and to assess the anticancer effect of EZH2 depletion in pancreatic cancer cells.
Experimental Design: Immunohistochemistry and cytosolic/nuclear fractionation were done to determine the expression pattern of EZH2 in normal pancreas and human pancreatic tumors. We used RNA interference, Western blotting, reverse transcription-PCR, and chromatin immunoprecipitation to study the effect of EZH2 depletion on pancreatic cancer cell proliferation and survival.
Results: We detected nuclear overexpression of EZH2 in pancreatic cancer cell lines and in 71 of 104 (68%) cases of human pancreatic adeno carcinomas. EZH2 nuclear accumulation was more frequent in poorly differentiated pancreatic adenocarcinomas (31 of 34 cases; $P < 0.001$). We found that genetic depletion of EZH2 results in reexpression of p27(Kip1) and decreased pancreatic cancer cell proliferation. Moreover, we showed that EZH2 depletion sensitized pancreatic cancer cells to doxorubicin and gemcitabine, which leads to a significant induction of apoptosis, suggesting that the combination of EZH2 inhibitors and standard chemotherapy could be a superior potential treatment for pancreatic cancer.
Conclusions: Our results show nuclear accumulation of EZH2 as a hallmark of poorly differentiated pancreatic adenocarcinoma; identify the tumor suppressor p27(KiP1) as a new target gene of EZH2; show that EZH2 nuclear overexpression contributes to pancreatic cancer cell proliferation; and suggest EZH2 as a potential therapeutic target for

the treatment of pancreatic cancer.
TC 67
ZB 52
Z8 8
ZS 0
Z9 75
SN 1078-0432
UT WOS:000260732200011
PM 18980972
ER

PT J
AU Sirak, Igor
Petera, Jiri
Hatlova, Jana
Vosmik, Milan
Melichar, Bohuslav
Dvorak, Josef
Zoul, Zdenek
Tycova, Vera
Lesko, Michal
Hajduch, Marian
TI Epidermal Growth Factor Receptor as a Predictor of Tumor Response to Preoperative Chemoradiation in Locally Advanced Gastric Carcinoma
SO STRAHLENTHERAPIE UND ONKOLOGIE
VL 184
IS 11
BP 592
EP 597
DI 10.1007/s00066-008-1880-9
PD NOV 2008
PY 2008
AB The purpose of our study was a retrospective evaluation whether the intensity of epidermal growth factor receptor (EGFR) expression predicts tumor response to preoperative chemoradiotherapy in patients with locally advanced gastric carcinoma.
Thirty-six patients with gastric adenocarcinoma (cT2-4 or N+) were studied. Preoperative treatment consisted of 30-45 Gy of gastric irradiation with continuous 5-fluorouracil and weekly cisplatin.

Surgical resection was performed 4-6 weeks later. EGFR expression in pretreatment tumor biopsies was assessed by immunohistochemistry. Level of EGFR expression was determined from the intensity and extent of staining. Tumor response was defined as a reduction of at least one T-stage level and/or finding of intense tumor regression in histopathologic examination.

Seventeen patients responded to preoperative chemoradiation - 8 patients (22%) had pathologic complete response, 9 patients (25%) were downstaged. Positive EGFR expression was found in 8 tumors (22%), and represented a significant predictive marker of poor tumor response in multivariate logistic regression analysis (p = 0.015). Response to chemoradiotherapy was found in 60% (16/28) of EGFR negative patients and in 13% (1/8) of EGFR positive patients (p = 0.044). None of the eight EGFR positive patients achieved pathologic complete response in comparison with 8/28 (29%) of patients with EGFR negative staining (p = 0.16).

EGFR may represent a molecular marker predictive for poor response to preoperative chemoradiotherapy in locally advanced gastric carcinoma.

RI Hajduch, Marian/J-4015-2014
TC 6
ZB 7
Z8 0
ZS 0
Z9 7
SN 0179-7158
UT WOS:000261037500006
PM 19016018
ER

PT J
AU Masumura, Kyoko
Ninomiya, Motoki
Nishizaki, Masahiko
Harano, Masao
Ohno, Satoshi
Takakura, Norihisa
Takata, Shinichi
TI [A case of long survival in Stage IV gastric carcinoma responding to combination treatment with paclitaxel and 5- fluorouracil followed by

surgical resection].
SO Gan to kagaku ryoho. Cancer & chemotherapy
VL 35
IS 10
BP 1745
EP 8
PD 2008-Oct
PY 2008
AB A 62-year-old female with epigastric pain was diagnosed with Type 4 gastric cancer upon detailed examination. Abdominal computed tomography(CT)revealed metastasis to the paraaortic lymph node and ascites at pelvis, and aspiration cytology of the ascites through vagina was positive(CY1). The clinical stage was determined as T4(panc) N1H0P0CY1M1(LYM), cStageIV. Three courses of neoadjuvant chemotherapy combined with paclitaxel and 5-fluorouracil(FT therapy)were performed. FT therapy showed a substantial reduction of the size of metastatic lymph nodes by sequentialCT examination, which was evaluated as partial response. Surgical resection consisted of total gastrectomy, and D2 lymph node dissection was performed. Operative cytology of ascites proved negative. The pathologic effect on primary lesion and metastatic lymph nodes was diagnosed as Grade 2. Although the prognosis of gastric cancer with carcinomatous peritonitis is poor, we here reported a patient with StageIV gastric cancer who markedly responded to FT therapy, which made surgical resection possible with the anticipation of extended survival. FT therapy may be a useful method for a patient with StageIVgastric cancer.
TC 0
ZB 0
Z8 0
ZS 0
Z9 0
SN 0385-0684
UT MEDLINE:18931580
PM 18931580
ER

PT J
AU Wang, Lin Bo
Shen, Jian Guo

Xu, Chao Yang
Chen, Wen Jun
Song, Xiang Yang
Yuan, Xiao Ming
TI Neoadjuvant Chemotherapy versus Surgery Alone for Locally Advanced Gastric Cancer: A Retrospective Comparative Study
SO HEPATO-GASTROENTEROLOGY
VL 55
IS 86-87
BP 1895
EP 1898
PD SEP-OCT 2008
PY 2008
AB Background/Aims: Preoperative chemotherapy is considered an effective treatment option for patients with gastric cancer. We retrospectively evaluated neoadjuvant chemotherapy with oxaliplatin. leucovorin and 5-flurouracil (OLF) in patients with locally advanced gastric cancer to determine its feasibility, as well as impact on the curative resection rate and patients' survival.

Methodology: A total of 87 patients with locally advanced gastric cancer that underwent preoperative chemotherapy combined with surgery or surgery alone were randomly matched according to the clinical TNM stage. The clinical responses to chemotherapy were assessed. The curative rate, postoperative complications and patients' survival between both groups were compared.

Results: The two groups were well matched. Complete or partial response was observed in 51.7% (15/29) of patients in the OLF group, and three (10.3%) of them had complete pathologic response. The curative resection rates were 89.7% in the OLF group and 77.6% in the surgery alone group. The postoperative complications were equal for both groups. The mean survival is 20.6 months in the OLF group vs. 19.9 months in the surgery alone group (p=0.02).

Conclusions: Neoadjuvant chemotherapy using OLF combination is active in gastric cancer and the toxicity level is acceptable. This treatment improves the curative resection rate and patients' survival in locally advanced gastric cancer.
TC 7
ZB 2
Z8 2

ZS 0
Z9 9
SN 0172-6390
UT WOS:000260868400088
PM 19102417
ER

PT J
AU Persiani, Roberto
   Rausei, Stefano
   Pozzo, Carmelo
   Biondi, Alberto
   Barone, Carlo
   Cananzi, Ferdinando C. M.
   Schinzari, Giovanni
   D'Ugo, Domenico
TI 7-year survival results of perioperative chemotherapy with epidoxorubicin, etoposide, and cisplatin (EEP) in locally advanced resectable gastric cancer: Up-to-date analysis of a phase-II study
SO ANNALS OF SURGICAL ONCOLOGY
VL 15
IS 8
BP 2146
EP 2152
DI 10.1245/s10434-008-9982-2
PD AUG 2008
PY 2008
AB Background: Perioperative chemotherapy is considered an effective treatment option for patients with gastric carcinoma. We report the results after a 7-year follow-up of a study aimed at evaluating a perioperative chemotherapy protocol in a group of patients with locally advanced gastric cancer (LAGC).
   Methods: Between February 1996 and May 2000, 24 patients with LAGC underwent D2-gastrectomy after three preoperative cycles of chemotherapy (Epidoxorubicin, Etoposide, Cisplatinum). Three further cycles were planned after surgery. Differences among groups were evaluated using the chi-square test. Survival rate was calculated after a 7-year follow-up, and differences were assessed using the log-rank test. Multivariate analysis was performed using the Cox proportional hazard model.

Results: A total of 24 patients received preoperative chemotherapy and underwent surgical resection. Of these, 17 (71%) received postoperative treatment. The main toxicity was grade 3-4 neutropenia. Curative resection (R0) was achieved in 83.3% of patients. No pathologic complete responses were documented, but tumor downstaging was obtained in 10 of 24 patients (41.7%). Overall median survival was 40 months, and 7-year survival rate was 46%. At univariate and multivariate analysis, R0 resection and tumor diameter were the most important prognostic factors. Conclusion: Long-term results in our series show a survival benefit for LAGC patients treated by perioperative chemotherapy and D2-gastrectomy when compared with previously studied controls who had surgery with postoperative chemotherapy alone. The high rate and prognostic impact of R0 resection in this study stressed the role of the therapy during the preoperative phase.
TC 5
ZB 2
Z8 5
ZS 1
Z9 10
SN 1068-9265
UT WOS:000257911300011
PM 18543037
ER

PT J
AU Ishigami, Sumiya
Natsugoe, Shoji
Nakajo, Akihiro
Matsumoto, Masataka
Uenosono, Yoshikazu
Arigami, Takaaki
Setoyama, Tetsuro
Arima, Hideo
Uchikado, Yasuto
Kita, Yoshiaki
Sasaki, Ken
Aikou, Takashi
TI Salvage gastrectomy following a combination of biweekly paclitaxel and S-1 for stage IV gastric cancer

SO JOURNAL OF GASTROINTESTINAL SURGERY
VL 12
IS 8
BP 1370
EP 1375
DI 10.1007/s11605-008-0539-2
PD AUG 2008
PY 2008
AB Background and aim We investigated the clinical benefits of salvage gastrectomy for stage IV gastric cancer patients whose distant lesions showed complete response after chemotherapy.
Methods We enrolled 18 stage IV gastric cancer patients whose distant metastases had disappeared or were controlled by a combination of biweekly paclitaxel (PTX) and S-1. After chemotherapy, these patients received gastrectomy with lymph node dissection. The postoperative outcome was analyzed with respect to both the histological effects of chemotherapy and tumor behavior.
Results Of the 18 patients, 8 had distant lymph node metastases, 9 had peritoneal dissemination, and five had multiple liver metastases prior to chemotherapy. Fourteen patients received curative surgery (R0). No severe postoperative complications were encountered. Pathological evaluation revealed grade 3 and grade 2 tumor regression in the primary lesion in one and five patients, respectively, and grade 3 and grade 2 tumor regression in the lymph nodes in one and six patients, respectively. Univariate analysis of the patients' prognosis identified R number, gross tumor type, histological grade of tumor regression, and gender as significant factors. Multivariate analysis showed that only the R number was an independent prognostic factor.
Conclusion R0 salvage gastrectomy following a combination of biweekly PTX and S-1 may have significant clinical efficacy for advanced gastric cancer patients.
TC 5
ZB 2
Z8 0
ZS 1
Z9 6
SN 1091-255X
UT WOS:000258112800013
PM 18516651

ER

PT J
AU Ina, Kenji
   Kataoka, Takae
   Takeuchi, Yuuki
   Fukuoka, Tomoki
   Miwa, Takaya
   Nishio, Tomoko
   Furuta, Ryuichi
   Masaki, Ayako
   Mori, Fumiko
   Kayukawa, Satoshi
   Nagao, Seiji
   Ando, Takafumi
   Goto, Hidemi
TI Pathological complete response induced by the combination therapy of S-1 and 24-h infusion of cisplatin in two cases initially diagnosed as inoperable advanced gastric cancer
SO ONCOLOGY REPORTS
VL 20
IS 2
BP 259
EP 264
DI 10.3892/or_00000001
PD AUG 2008
PY 2008
AB We report on two patients, successfully treated by the combination therapy of S-1 and 24-h infusion of cisplatin (CDDP), who were initially diagnosed with Unresectable stage 4 advanced gastric cancer. Each patient had a very good clinical response and underwent Curative gastrectomy after completion of 14 and 10 courses of S-1/CDDP chemotherapy, respectively. A microscopically detailed examination of surgically obtained specimens showed the complete disappearance of malignant cells in the two cases. S-1/CDDP combination therapy can, therefore, be highly active in incurable advanced gastric carcinoma.
TC 3
ZB 1
Z8 0

ZS 0
Z9 3
SN 1021-335X
UT WOS:000258467800001
PM 18636184
ER

PT J
AU Wang Ting-Ting
Wei Jia
Qian Xiao-ping
Yu Li-xia
Liu Bao-rui
TI Differential gene expression of the inhibitor of apoptosis proteins in docetaxel-resistant gastric cancer cells
SO Zhonghua Zhongliu Zazhi
VL 30
IS 8
BP 573
EP 577
PD AUG 2008
PY 2008
AB Objective Resistance to chemotherapy may indicate an unfavorable outcome for patients with gastric cancer. The purpose of this study was to examine whether docetaxel-resistance could be due in part to the expression of the inhibitor of apoptosis proteins (IAP). Methods Docetaxel-resistant cells, BGC-823/R1, BGC-823/R2 and BGC-823/R3, were established from parent BGC-823 cells by stepwise increasing concentration of docetaxel. To characterize these cells, we examined the effects of docetaxel on cell growth and apoptosis by MTT assay and double staining with both annexin-V-FITC and PI, and analyzed the cross-resistance to various anticancer drugs. Expression of IAP compared with that in parental cells was evaluated by real-time quantitative PCR. Results The BGC-823 resistant cells, BGC-823/R1, R2 and R3 cells, were 10. 2-, 24. 5-, 56. 3-fold more resistant to docetaxel than parental cells, respectively, and this resistance was paralleled with reduced induction of apoptosis. BGC-823/R3 cells showed cross-resistance to paclitaxel, whereas exhibited weak or no cross-resistance against 5-fluorouracil, cisplatin and oxaliplatin. The expressions of survivin

and XIAP were gradually increased with the extent of docetaxel resistance (r 0.909, $P < 0.001$ and r = 0.892, $P < 0.001$, respectively). Conclusion IAP may make an important contribution to the resistance to the apoptotic effect of docetaxel in gastric cancer, and could be used as a potential therapeutic target.

TC 1

ZB 1

Z8 1

ZS 0

Z9 2

SN 0253-3766

UT BIOSIS:PREV200900057946

PM 19102932

ER

PT J

AU Reed, Valerie K.
Krishnan, Sunil
Mansfield, Paul F.
Bhosale, Priya R.
Kim, Michelle
Das, Prajnan
Janjan, Nora A.
Delclos, Marc E.
Lowy, Andrew M.
Feig, Barry W.
Pisters, Peter W. T.
Ajani, Jaffer A.
Crane, Christopher H.

TI Incidence, natural history, and patterns of locoregional recurrence in gastric cancer patients treated with preoperative chemoradiotherapy

SO INTERNATIONAL JOURNAL OF RADIATION ONCOLOGY BIOLOGY PHYSICS

VL 71

IS 3

BP 741

EP 747

DI 10.1016/j.ijrobp.2007.10.030

PD JUL 1 2008

PY 2008

AB Purpose: To retrospectively determine the incidence and patterns (in-field, marginal, or out-of-field) of locoregional gastric cancer recurrence in patients who received preoperative chemoradiotherapy and to determine the outcome in these patients.
Methods and Materials: Between 1994 and 2004, 149 patients with gastric carcinoma were treated according to institutional protocols with preoperative chemoradiotherapy. Ultimately, 105 patients had an R0 resection. Of these 105 patients, 65 received preoperative chemotherapy followed by chemoradiotherapy and 40 received preoperative chemoradiotherapy. Most (96%) of these patients received 5-fluorouracil-based chemotherapy during radiotherapy, and the median radiation dose was 45 Gy. We retrospectively identified and classified the patterns of locoregional recurrence.
Results: The 3-year actuarial incidence of locoregional recurrence was 13%, with locoregional disease recurring as any part of the failure pattern in 14 patients. Most (64%) of the evaluable locoregional recurrences were in-field. Of the 4 patients with a marginal recurrence, 2 had had inadequate coverage of the regional nodal volumes on their oblique fields. The pathologic complete response rate was 23%. A pathologic complete response was the only statistically significant predictor of locoregional control.
Conclusion: Patients with gastric cancer who received preoperative chemoradiotherapy had low rates of locoregional recurrence. This strategy merits prospective multi-institutional and randomized evaluation. (c) 2008 Elsevier Inc.
CT 48th Annual Meeting of the American-Society-for-Therapeutic-Radiology-and-Oncology
CY NOV 05-09, 2006
CL Philadelphia, PA
SP Amer Soc Therapeut Radiol & Oncol
TC 8
ZB 5
Z8 1
ZS 0
Z9 9
SN 0360-3016
UT WOS:000256572400015
PM 18164837
ER

PT J
AU Hsu, Feng-Ming
Lin, Chia-Chi
Lee, Jang-Ming
Chang, Yih-Leong
Hsu, Chih-Hung
Tsai, Yu-Chieh
Lee, Yung-Chie
Cheng, Jason Chia-Hsien
TI Improved local control by surgery and paclitaxel-based chemoradiation for esophageal squamous cell carcinoma: Results of a retrospective non-randomized study
SO JOURNAL OF SURGICAL ONCOLOGY
VL 98
IS 1
BP 34
EP 41
DI 10.1002/jso.21063
PD JUL 1 2008
PY 2008
AB Background and Objectives: To investigate the impact of using paclitaxel in chemoradiation on locally advanced esophageal squamous cell carcinoma (SCC) treated with or without surgery.
Methods: Of 127 patients with AJCC stages II-III esophageal SCC undergoing definitive chemoradiation (DefCRT, n = 44) or ncoadjuvant chemoradiation plus surgery (NeoCRT + S, n = 83), 57 received chemotherapy with paclitaxel and cisplatin (TP), and 70 received 5-fluorouracil and cisplatin (PF). Three-year local progression-free survival (LPFS), distant metastasis-free survival, overall survival, and prognostic factors were retrospectively analyzed.
Results: The median survival was 30 months. Pathological complete response rate was 41 % and 27% with TP and PF, respectively (P = 0. 19). NeoCRT + S achieved significantly higher LPFS than DefCRT (71 % vs. 39%, P < 0.001). Patients receiving TP had significantly higher LPFS than PF (74% vs. 48%, P = 0.04). Local control was similar between DefCRT with TP and NeoCRT + S. Distant metastasis-free survival and overall survival were not different between treatment modalities or chemotherapy regimens. In multivariate analysis, surgery (HR 0.30, P < 0.00 1), TP

regimen (HR 0.38, P = 0.007), and mediastinal lymphadenopathy (HR 2.37, P = 0.008) were independent factors for LPFS.
Conclusions: Both surgery and the use of paclitaxel-based chemoradiation may improve local disease control. Future randomized trials should integrate paclitaxel into definitive chemoradiation.
CT 48th Annual Meeting of the American-Society-for-Therapeutic-Radiology-and-Oncology
CY NOV 05-09, 2006
CL Philadelphia, PA
SP Amer Soc Therapeut Radiol & Oncol
TC 10
ZB 6
Z8 3
ZS 0
Z9 12
SN 0022-4790
UT WOS:000257543400009
PM 18449912
ER

PT J
AU Ku, Geoffrey Y
Ilson, David H
TI Preoperative therapy in esophageal cancer.
SO Clinical advances in hematology & oncology : H&O
VL 6
IS 5
BP 371
EP 9
PD 2008-May
PY 2008
AB Progress has been made in the treatment of locally advanced esophageal cancer. Preoperative and postoperative chemotherapy also appears to improve survival in gastroesophageal junction adenocarcinoma compared to surgery alone. Adding radiotherapy to preoperative chemotherapy enhances rates of curative resection, achieves measurable rates of pathologic complete response, and recent trials indicate a survival benefit for preoperative chemoradiotherapy compared to surgery alone in esophageal cancer. Given the achievement of pathologic complete responses with

combined chemoradiotherapy in esophageal cancer, recent trials have evaluated the contribution of surgery after chemoradiotherapy. With currently available systemic therapy for squamous cancers of the esophagus that respond to combined chemoradiotherapy, there is no clear survival benefit for the addition of surgery after chemoradiotherapy despite improvements in local tumor control with the addition of surgery. Surgery may salvage nonresponding patients with biopsy-positive residual disease. For adenocarcinoma of the esophagus, a histology with consistently lower rates of pathologic complete response than squamous cell cancer, surgery appears to play a greater role. Trials are now evaluating the use of newer chemotherapy agents combined with radiotherapy, including taxanes, irinotecan, and oxaliplatin. Response on postiron emission tomography early on during induction chemotherapy may be a strong prognostic measure of outcome. Targeted agents, including monoclonal antibodies that target the epidermal and vascular endothelial growth factor receptors, are in active development in phase II and III trials.
TC 4
ZB 2
Z8 0
ZS 0
Z9 4
SN 1543-0790
UT MEDLINE:18516027
PM 18516027
ER

PT J
AU Saikawa, Yoshir
Kubota, Tetsuro
Kumagai, Kosm
Nakamura, Rieko
Kumai, Koichiro
Shigematsu, Naoyuki
Kubo, Atsushi
Kitajima, Masaki
Kitagawa, Yuko
TI Phase II study of chemoradiotherapy with S-1 and low-dose cisplatin for inoperable advanced gastric cancer

SO INTERNATIONAL JOURNAL OF RADIATION ONCOLOGY BIOLOGY PHYSICS
VL 71
IS 1
BP 173
EP 179
DI 10.1016/j.ijrobp.2007.09.010
PD MAY 1 2008
PY 2008
AB Purpose: The results of a pilot study using S-1/low-dose cisplatin/radiotherapy led us to hypothesize that the initial chemoradiotherapy regimen would induce a 70% efficacy rate with a 10% pathologic complete response rate.
Patients and Methods: Only patients with unresectable or incurable advanced gastric cancer were eligible. The patients received induction S-1 and cisplatin therapy with radiotherapy followed by chemotherapy alone.
Results: Of the 30 patients recruited and assessed, 29 were eligible for clinical evaluation of measurable lesions. The response rate was 65.5%, with 19 with a partial response, 8 with no change, and 2 with progressive disease of 29 patients. Of the 30 patients recruited, 10 (33.3%) underwent stomach resection and D2 LN dissections. The pathologic complete response rate was 13.3% (4 patients), and the R0 resection rate was 100% (10 patients). The survival analysis showed a median survival time of 25 months. Grade 3 toxicity occurred in 66.7% for leukocytopenia, 33.3% for thrombocytopenia, 23.3% for nausea and appetite loss, and 6.7% for anemia, diarrhea, and renal dysfunction. Although all the patients had been hospitalized with a poor performance status with a giant tumor, 97% (29 of 30) could be discharged after the first cycle, resulting in an improvement in quality of life.
Conclusion: Chemoradiotherapy could be a powerful regimen for controlling tumor progression in advanced gastric cancer, improving patients' quality of life with tolerable toxicity. A complete histologic response rate of >10% would be expected, even for large tumors with metastatic lesions. (c) 2008 Elsevier Inc.
RI Shigematsu, Naoyuki/B-9374-2014
TC 14
ZB 10
Z8 0
ZS 0

Z9 14
SN 0360-3016
UT WOS:000255149000025
PM 17996385
ER

PT J
AU Luu, Theresa D.
   Gaur, Puja
   Force, Seth D.
   Staley, Charles A.
   Mansour, Kamal A.
   Miller, Joseph I., Jr.
   Miller, Daniel L.
TI Neoadjuvant chemoradiation versus chemotherapy for patients undergoing
   esophagectomy for esophageal cancer
SO ANNALS OF THORACIC SURGERY
VL 85
IS 4
BP 1217
EP 1224
DI 10.1016/j.athoracsur.2007.11.070
PD APR 2008
PY 2008
AB Background. Neoadjuvant chemoradiation followed by esophagectomy is
   currently the standard of care for locally advanced esophageal cancer.
   This intense preoperative regimen delays definitive resection and
   increases perioperative risks. With the improvement of chemotherapy
   agents, chemotherapy alone may be better suited for patients awaiting
   esophagectomy because of shorter preoperative treatment time and less
   associated perioperative complications. No recent study has compared
   chemoradiation to chemotherapy alone before esophageal resection with
   respect to operative morbidity and mortality and overall survival.
   Methods. A retrospective review was performed of all patients (281) who
   underwent an esophagectomy for cancer at our institution from July 1995
   through June 2005; 122 patients (43%) had neoadjuvant treatment and form
   the basis of this study.
   Results. Preoperative chemoradiation (CR) was administered in 64
   patients and chemotherapy only (CO) in 58 patients. Operative mortality

was 6% (4 patients) in the CR group and 0% in the CO group (p = 0.12). Overall postoperative complications rate was 48% in CR patients and 33% in CO patients (p = 0.09). Complete pathologic response occurred in 11 CR patients (17%) and in 2 CO patients (4%; p = 0.02). There was no difference in recurrences between the two groups (p = 0.43). Median survival was 17 months in the CR patients and 21 months in the CO patients (p = 0.14). One-, 3-, and 5- year survivals were 76%, 46%, and 41%, respectively, in the CR patients and 70%, 40%, and 31%, respectively, in the CO patients (p = 0.31).
Conclusions. Although neoadjuvant chemoradiation resulted in a significantly better complete pathologic response rate when compared with chemotherapy alone, that did not translate into a long-term survival advantage. Chemotherapy alone may be the preferred neoadjuvant modality to expedite resection, decrease operative mortality and postoperative complications, and improve survival in patients with locally advanced esophageal cancer.
CT 53rd Annual Meeting of the Southern-Thoracic-Surgical-Association
CY NOV 08-11, 2006
CL Tucson, AZ
SP SE Thorac Surg Assoc
TC 33
ZB 6
Z8 1
ZS 0
Z9 34
SN 0003-4975
UT WOS:000254083300014
PM 18355499
ER

PT J
AU Mansour, John C.
Tang, Laura
Shah, Manish
Bentrem, David
Klimstra, David S.
Gonen, Mithat
Kelsen, David P.
Brennan, Murray F.

Coit, Daniel G.
TI Does graded histologic response after neoadjuvant chemotherapy predict survival for completely resected gastric cancer?
SO ANNALS OF SURGICAL ONCOLOGY
VL 14
IS 12
BP 3412
EP 3418
DI 10.1245/s10434-007-9574-6
PD DEC 2007
PY 2007
AB Background: After publication of the MAGIC trial results, preoperative chemotherapy is increasingly used to treat advanced gastric cancer before resection. Tools for measuring response must be assessed.
Methods: We identified all patients with gastric cancer treated with neoadjuvant chemotherapy and R0 resection between 1991 and 2005 from a prospective database. Patients receiving preoperative radiation were excluded. Histologic response to treatment was graded from 0% to 100% by a single pathologist. Kaplan-Meier survival analysis was performed to identify the relationship between response and outcome and to identify factors predictive of disease-specific survival (DSS). Multivariate analysis was performed to identify independent predictors.
Results: A total of 168 patients underwent R0 resection after receiving neoadjuvant chemotherapy. Thirty-three percent of tumors were at the gastroesophageal junction. Cisplatin-based therapy was used for 68% of patients. Twenty-two percent of patients had a > 50% pathologic response to treatment. Median follow-up after resection for all patients was 25 months. Median DSS for all patients was 33 months. Three-year DSS improved from 44% to 69% with at least a 50% histologic response (P = .01). Factors associated with decreased DSS included positive nodes at resection, pT3 tumor or greater, high grade, perineural or vascular invasion, and < 50% response. Multivariate analysis identified nodal status and perineural or vascular invasion as independent predictors of survival.
Conclusions: Posttreatment nodal status and perineural or vascular invasion at resection, but not graded histologic response, independently predict DSS after neoadjuvant chemotherapy and surgical resection of gastric cancer.
RI Gonen, Mithat/E-4826-2012

TC 24
ZB 8
Z8 3
ZS 0
Z9 27
SN 1068-9265
UT WOS:000250976500023
PM 17909917
ER

PT J
AU Wieder, Hinrich A.
Ott, Katja
Lordick, Florian
Becker, Karen
Stahl, Alexander
Herrmann, Ken
Fink, Ulrich
Siewert, Joerg Ruediger
Schwaiger, Markus
Weber, Wolfgang A.
TI Prediction of tumor response by FDG-PET: comparison of the accuracy of single and sequential studies in patients with adenocarcinomas of the esophagogastric junction
SO EUROPEAN JOURNAL OF NUCLEAR MEDICINE AND MOLECULAR IMAGING
VL 34
IS 12
BP 1925
EP 1932
DI 10.1007/s00259-007-0521-3
PD DEC 2007
PY 2007
AB Purpose Positron-emission-tomography with the glucose analog fluorodeoxyglucose (FDG-PET) has shown encouraging results for prediction of tumor response to chemotherapy. However, there is no consensus as to what time after initiation of therapy FDG-PET should be performed. To address this question we studied the time course of changes in tumor FDG-uptake in patients with locally advanced adenocarcinomas of the esophagogastric junction (AEG) treated with

preoperative chemotherapy.
Methods Twenty-four patients with AEG were included and underwent FDG-PET prior to therapy (PET1), 2 weeks after initiation of therapy (PET2), and preoperatively (PET3). Tumor metabolic activity was assessed by standardized uptake values (SUV) and correlated with histopathologic response and patient survival.
Results Baseline tumor SUV was 8.3 +/- 3.5 and decreased to 5.0 +/- 1.8 at PET2 ($p < 0.0001$). At PET3 there was further decrease to 3.5 +/- 1.9 ($p < 0.0001$). The relative decrease of tumor FDG-uptake from PET1 to PET2 and from PET1 to PET3 were both significantly correlated with histopathologic response. Reduction of tumor SUV from PET1 to PET2 was significantly correlated with survival ($p = 0.03$) and there was a similar trend for changes from PET1 to PET3 ($p = 0.09$). In contrast, absolute SUVs did not demonstrate a significant correlation with histopathological response or patient survival at any of the studied time points.
Conclusion In patients with AEG, relative changes in tumor FDG uptake are better predictors for treatment outcome than absolute SUVs. Metabolic changes within the first 2 weeks of therapy are at least as efficient for prediction of histopathologic response and patient survival as later changes.
TC 45
ZB 15
Z8 1
ZS 0
Z9 46
SN 1619-7070
UT WOS:000251370400006
PM 17680242
ER

PT J
AU Kelsey, Chris R.
Chino, Junzo P.
Willett, Christopher G.
Clough, Robert W.
Hurwitz, Herbert I.
Morse, Michael A.
Bendell, Johanna C.

D’Amico, Thomas A.
Czito, Brian G.
TI Paclitaxel-based chemoradiotherapy in the treatment of patients with operable esophageal cancer
SO INTERNATIONAL JOURNAL OF RADIATION ONCOLOGY BIOLOGY PHYSICS
VL 69
IS 3
BP 770
EP 776
DI 10.1016/j.ijrobp.2007.03.035
PD NOV 1 2007
PY 2007
AB Purpose: To compare a neoadjuvant regimen of cisplatin/5-fluorouracil (5-FU) and concurrent radiation therapy (RT) with paclitaxel-based regimens and RT in the management of operable esophageal (EC)/gastroesophageal junction (GEJ) cancer.
Methods and Materials: All patients receiving neoadjuvant chemotherapy (CT) and RT for EC/GEJ cancer at Duke University between January 1995 and December 2004 were included. Clinical end points were compared for patients receiving paclitaxel-based regimens (TAX) vs. alternative regimens (non-TAX). Local control (LC), disease-free survival (DFS), and overall survival (OS) were estimated using the Kaplan-Meier method. Chi-square analysis was performed to test the effect of TAX on pathologic complete response (pCR) rates and toxicity.
Results: A total of 109 patients received CT-RT followed by esophagectomy (95 M; 14 F). Median RT dose was 45 Gy (range, 36-66 Gy). The TAX and non-TAX groups comprised 47% and 53% of patients, respectively. Most (83%) TAX patients received three drug regimens including platinum and a fluoropyrimidine. In the non-TAX group, 89% of the patients received cisplatin and 5-FU. The remainder received 5-FU or capecitabine alone. Grade 3-4 toxicity occurred in 41% of patients receiving TAX vs. 24% of those receiving non-TAX (p =0.19). Overall pCR rate was 39% (39% with TAX vs. 40% with non-TAX, p = 0.9). Overall LC, DFS, and OS at 3 years were 80%, 34%, and 37%, respectively. At 3 years, there were no differences in LC (75% vs. 85%, p = 0.33) or OS (37% vs. 37 %, p = 0.32) between TAX and non-TAX groups.
Conclusions: In this large experience, paclitaxel-containing regimens did not improve pCR rates or clinical end points compared to non-paclitaxel-containing regimens. (C) 2007 Elsevier Inc.

CT 48th Annual Meeting of the
American-Society-for-Therapeutic-Radiology-and-Oncology
CY NOV 05-09, 2006
CL Philadelphia, PA
SP Amer Soc Therapeut Radiol & Oncol
TC 17
ZB 8
Z8 5
ZS 0
Z9 22
SN 0360-3016
UT WOS:000249943000017
PM 17889266
ER

PT J
AU Patel, Pooja R.
Mansfield, Paul F.
Crane, Christopher H.
Wu, Tsung-Teh
Lee, Jeffrey H.
Lynch, Patrick M.
Morris, Jeffrey
Pisters, Peter W.
Feig, Barry
Sunder, Punita K.
Izzo, Julie G.
Ajani, Jaffer A.
TI Clinical stage after preoperative chemoradiation is a better predictor
of patient outcome than the baseline stage for localized gastric cancer
SO CANCER
VL 110
IS 5
BP 989
EP 995
DI 10.1002/cncr.22870
PD SEP 1 2007
PY 2007
AB BACKGROUND. For patients with localized gastric cancer (LGC) who are

receiving 2 preoperative chemoradiation (CTRT), the postsurgical pathologic stage predicts overall survival (OS) better than the baseline stage. The authors hypothesized 4 that presurgical (postCTRT) stage would also correlate better with patient outcome than the baseline stage.
METHODS. The authors analyzed 74 LGC patients treated with preoperative CTRT receiving similar treatment. Patients were staged with baseline endoscopic ultrasonography (EUS) and laparoscopy. Patients received induction chemotherapy, then CTRT (45 Gy), and had an attempted surgery. After CTRT, patients had complete preoperative staging including EUS in 35 patients.
RESULTS. Thirty-five had all 3 sets of staging, baseline, presurgical, and postsurgical. Baseline stage did not associate with OS (P =.16) nor disease-tree survival (DFS; P =.13). However, presurgical stage was associated with OS (P =.01), and DFS (P=.05). OS was also associated with postsurgical stage and was longer for stages 0 and I than for stages III and IV (P =.01 and .04, respectively). Similarly, DFS was longer in postsurgical pathologic stages 0, 1, and 11 than in stage III or IV (P <.001, <.001, and <.01, respectively). Baseline staging did not correlate with the proportion of patients alive at 4 years; however, presurgical staging did. Patients with stage I or 11 survived longer than those with stage III or IV (81% vs 25%; P <.01).
CONCLUSIONS. Heterogeneity in clinical biology of LGC is best reflected after CTRT in presurgical and postsurgical pathologic stages rather than by the baseline stage. Correlation of outcome with presurgical staging may facilitate strategies to individualize therapy for LGC. Cancer 2007; 110:989-95. (c) 2007 American Cancer Society
TC 14
ZB 6
Z8 0
ZS 0
Z9 14
SN 0008-543X
UT WOS:000249191100008
PM 17636525
ER

PT J
AU Ku, Geoffrey Y.

Ilson, David H.
TI Esophageal cancer: Adjuvant therapy
SO CANCER JOURNAL
VL 13
IS 3
BP 162
EP 167
DI 10.1097/PPO.0b013e318074dbe7
PD MAY-JUN 2007
PY 2007
AB In the United States, esophageal cancer is an uncommon but aggressive malignancy. Prior research has focused on the incorporation of chemotherapy and radiotherapy in both the pre- and postoperative setting. Both squamous cell and adenocarcinoma histologies have been treated in trials, with adenocarcinoma now the predominant histology seen in the United States. Although preoperative chemotherapy improves survival compared with surgery alone, the addition of concurrent radiotherapy to preoperative chemotherapy improves rates of curative resection, reduces local tumor recurrence, and achieves a significant rate of pathologic complete response. Combined preoperative chemoradiotherapy is the preferred preoperative strategy for locally advanced esophageal cancers in the United States. Definitive chemoradiotherapy alone appears to be equivalent in terms of overall survival compared with chemoradiotherapy followed by surgery in squamous cancers, although the addition of surgery after chemoradiotherapy may afford superior local control of disease. Postoperatively, survival is improved with postoperative chemotherapy and radiotherapy in adenocarcinoma of the gastroesophageal junction, if none has been delivered preoperatively. Ongoing research involves evaluating regimens with newer chemotherapeutic drugs, such as paclitaxel or irinotecan, as well as the incorporation of targeted molecular therapies.
TC 7
ZB 5
Z8 3
ZS 0
Z9 10
SN 1528-9117
UT WOS:000247717700005
PM 17620765

ER

PT J
AU Muino, C. Bueno
   Vazquez, J. Puente
   Valera, J. Sastre
   Garcia-Saenz, J. A.
   Martin, M.
   Miralles, N. Garcia
   Sanchez-Pernaute, A.
   Diaz-Rubio, E.
TI Pathological complete response following docetaxel-based neoadjuvant
   chemotherapy for locally advanced gastric adenocarcinoma
SO CLINICAL & TRANSLATIONAL ONCOLOGY
VL 9
IS 5
BP 335
EP 338
DI 10.1007/s12094-007-0063-y
PD MAY 2007
PY 2007
AB Locally advanced gastric adenocarcinoma has a poor outcome. Neoadjuvant
   treatment is being tested in locally advanced non-resectable tumours and
   in those resectable tumours with a high risk of recurrence. Efforts to
   identify prognostic factors and more active and less toxic preoperative
   regimens are being searched for. We report the case of a patient
   achieving a complete histopathological complete response following
   docetaxel-based neoadjuvant chemotherapy.
TC 1
ZB 0
Z8 0
ZS 1
Z9 1
SN 1699-048X
UT WOS:000256904500012
ER

PT J
AU Bueno Muino, C

Puente Vazquez, J
Sastre Valera, J
Garcia-Saenz, J A
Martin, M
Garcia Miralles, N
Sanchez-Pernaute, A
Diaz-Rubio, E
TI Pathological complete response following docetaxel-based neoadjuvant chemotherapy for locally advanced gastric adenocarcinoma.
SO Clinical & translational oncology : official publication of the Federation of Spanish Oncology Societies and of the National Cancer Institute of Mexico
VL 9
IS 5
BP 335
EP 8
DI 10.1007/s12094-007-0063-y
PD 2007-May
PY 2007
AB Locally advanced gastric adenocarcinoma has a poor outcome. Neoadjuvant treatment is being tested in locally advanced non-resectable tumours and in those resectable tumours with a high risk of recurrence. Efforts to identify prognostic factors and more active and less toxic preoperative regimens are being searched for. We report the case of a patient achieving a complete histopathological complete response following docetaxel- based neoadjuvant chemotherapy.
TC 1
ZB 0
Z8 0
ZS 1
Z9 1
SN 1699-048X
UT MEDLINE:17525046
PM 17525046
ER

PT J
AU Adelstein, David J.
Rice, Thomas W.

Rybicki, Lisa A.
Saxton, Jerrold P.
Videtic, Gregory M. M.
Murthy, Sudish C.
Zuccaro, Gregory
Vargo, John J.
Dumot, John A.
Carroll, Marjorie A.
TI A phase II trial of accelerated multimodality therapy for locoregionally advanced cancer of the esophagus and gastroesophageal junction - The impact of clinical heterogeneity
SO AMERICAN JOURNAL OF CLINICAL ONCOLOGY-CANCER CLINICAL TRIALS
VL 30
IS 2
BP 172
EP 180
DI 10.1097/01.coc.0000251243.58048.12
PD APR 2007
PY 2007
AB Objectives: This is a report of mature results from a phase II trial of an accelerated multimodality treatment program for locoregionally advanced cancer of the esophagus and gastroesophageal junction with a focus on the impact of clinical heterogeneity on outcomes. A split course of pre- and postoperative hyperfractionated radiation therapy and concurrent chemotherapy was used in an effort to limit perioperative mortality.
Methods: Eligibility required a diagnosis of esophageal or gastroesophageal junction cancer and an esophageal ultrasound stage of at least T3, N1, or M1A. Patients received a 12-day induction course of radiation (1.5 Gy twice a dose to a dose of 30 Gy) concurrent with 4-day continuous intravenous infusions of cisplatin (20 mg/m(2) per day) and 5-fluorouracil (1000 mg/m(2) per day) beginning on day 1. Surgery followed in 4 to 6 weeks followed 6 to 10 weeks later by a second, identical course of chemoradiotherapy.
Results: From October 1999 through March 2003, 93 patients were enrolled; 96% were white, 86% male, and 83% had adenocarcinoma. Resection was possible in 83 patients (89%) with 4 (5%) perioperative deaths. With a median follow up of 50 months (range, 34-72 months), the 3-year projected overall survival rate is 27.9%, freedom from recurrence

30.5%, and distant metastatic control 32.4%. Locoregional control in resected patients is 86%. Freedom from recurrence and distant control were significantly better in patients with 1) earlier pretreatment clinical stage, 2) earlier postinduction pathologic stage, 3) squamous cell cancer, and 4) a pathologic response.
Conclusions: This accelerated multimodality treatment program is feasible and perioperative mortality proved acceptable. Despite excellent locoregional control, freedom from recurrence, and overall
CT 41st Annual Meeting of the American-Society-of-Clinical-Oncology
CY MAY 13-17, 2005
CL Orlando, FL
SP Amer Soc Clin Oncol
TC 9
ZB 0
Z8 0
ZS 0
Z9 9
SN 0277-3732
UT WOS:000245637100011
PM 17414467
ER

PT J
AU Klautke, Gunther
Fietkau, Rainer
TI Significance of radiation therapy for adenocarcinomas of the esophagus, gastroesophageal junction and gastric cancer with special reference to the MAGIC trial
SO STRAHLENTHERAPIE UND ONKOLOGIE
VL 183
IS 4
BP 163
EP 169
DI 10.1007/s00066-007-7702-7
PD APR 2007
PY 2007
TC 15
ZB 10
Z8 0

ZS 0
Z9 15
SN 0179-7158
UT WOS:000245452600001
PM 17406796
ER

PT J
AU Pfau, Patrick R.
Perlman, Scott B.
Stanko, Peter
Frick, Terrence J.
Gopal, Deepak V.
Said, Adnan
Zhang, Zhengjun
Weigel, Tracey
TI The role and clinical value of EUS in a multimodality esophageal carcinoma staging program with CT and positron emission tomography
SO GASTROINTESTINAL ENDOSCOPY
VL 65
IS 3
BP 377
EP 384
DI 10.1016/j.gie.2006.12.015
PD MAR 2007
PY 2007
AB Background: EUS, CT, and positron emission tomography (PET) have all been used in the preoperative staging of esophageal cancer separately or in various combinations.
Objective: Our purpose was to determine the value and role of EUS when used in conjunction with CT and PET imaging in staging cancer of the esophagus and gastroesophageal junction.
Design: Retrospective single-center clinical trial.
Setting: Academic tertiary care center.
Patients: Data were examined for 56 patients who concomitantly underwent examination with EUS, CT, and PET in a multimodality staging program.
Main Outcome Measurements: EUS, CT, and PET were examined for their ability to detect the primary tumor, local tumor stage, locoregional adenopathy, and distant metastases. With use of surgical resection as

baseline therapy, the frequency at which EUS, CT, and PET affected and changed management was examined.
Results: EUS is the only imaging test that identified all primary tumors and provided tumor staging. EUS identified a significantly greater number of patients (58.9%) with locoregional nodes than did CT (26.8%), P = .0006, or PET (37.5%), P = .02. CT identified 14.3% and PET identified 26.8% of patients with distant metastases. With CT alone, 15.2% of patients were not taken to surgery, whereas PET affected management by preventing surgery because of metastatic disease in 28.3% of patients. EUS changed management by guiding the need for neoadjuvant therapy in 34.8% of patients.
Limitations: Retrospective study, nonblinded study, lack of pathologic reference standard.
Conclusion: The primary strength of EUS in a multimodality staging strategy is in identifying patients with locally advanced disease and guiding the need for preoperative neoadjuvant therapy. EUS is not suited to determine resectability of esophageal cancer alone and thus is most effective when used in conjunction with other imaging tests such as CT and PET.
TC 45
ZB 13
Z8 2
ZS 1
Z9 48
SN 0016-5107
UT WOS:000244729700005
PM 17321235
ER

PT J
AU Siewert, J. Ruediger
   Ott, Katja
TI Are squamous and adenocarcinomas of the esophagus the same disease?
SO SEMINARS IN RADIATION ONCOLOGY
VL 17
IS 1
BP 38
EP 44
DI 10.1016/j.semradonc.2006.09.007

PD JAN 2007
PY 2007
TC 75
ZB 39
Z8 3
ZS 2
Z9 79
SN 1053-4296
UT WOS:000245998400006
PM 17185196
ER

PT J
AU Lowy, Andrew M.
   Firdaus, Irfan
   Roychowdhury, Debasish
   Redmond, Kevin
   Howington, John A.
   Sussman, Jeffrey J.
   Safa, Malek
   Ahmad, Syed A.
   Reed, Michael F.
   Rose, Patricia
   James, Laura
   Jazieh, Abdul Rahman
TI A phase II study of sequential neoadjuvant gemcitabine and paclitaxel,
   radiation therapy with cisplatin and 5-fluorouracil and surgery in
   locally advanced esophageal carcinoma
SO AMERICAN JOURNAL OF CLINICAL ONCOLOGY-CANCER CLINICAL TRIALS
VL 29
IS 6
BP 555
EP 561
DI 10.1097/01.coc.0000233997.36073.8e
PD DEC 2006
PY 2006
AB Objective: To evaluate the feasibility and efficacy of sequential
   neoadjuvant chemotherapy, chemoradiation, and surgery in patients with
   locally advanced esophageal cancer.

Patients and Methods: There were 29 patients who received paclitaxel 150 mg/m(2) and gemcitabine 3000 mg/m(2) 2 weeks apart. Two weeks later, patients received cisplatin 75 mg/m(2) and 5-fluorouracil (5-FU) 1000 mg/m(2)/d continuous infusion for 4 days with concurrent radiotherapy in 15 fractions to a total dose of 4000 cGy. After 6 weeks, cisplatin and 5-FU were repeated at the above doses. After 4 to 6 weeks, patients were restaged and underwent surgical resection.
Results: All 29 patients completed the prescribed gemcitabine, paclitaxel, and radiation therapy. Febrile neutropenia occurred in 1 patient and 4 patients received growth factor support. After neoadjuvant treatment, 1 patient refused surgery, 23 underwent R0 resection (82%), while 5 developed progressive disease. Four patients developed anastomotic leaks (17%). Four patients had complete pathologic responses (14%) and 4 (14%) had only residual microscopic disease. Nine patients remain alive at a median follow-up of 48 months. Three-year survival for the entire cohort was 36%.
Conclusion: This regimen was associated with a high rate of compliance and induction therapy had an acceptable toxicity profile. The R0 resection rate and 3-year survival data are similar to recently reported studies. While active, gemcitabine and paclitaxel induction therapy was associated with an increased rate of postoperative complications, but no increase in survival. Patterns of failure continue to demonstrate the need for regimens incorporating greater emphasis on systemic therapy for locally advanced esophageal cancer.
TC 3
ZB 2
Z8 0
ZS 0
Z9 3
SN 0277-3732
UT WOS:000242681700007
PM 17148991
ER

PT J
AU D'Ugo, D.
Persiani, R.
Rausei, S.
Biondi, A.

Vigorita, V.
Boccia, S.
Ricci, R.
TI Response to neoadjuvant chemotherapy and effects of tumor regression in gastric cancer
SO EJSO
VL 32
IS 10
BP 1105
EP 1109
DI 10.1016/j.ejso.2006.07.009
PD DEC 2006
PY 2006
AB Aims: Aim of this study is the evaluation of the effects induced by neoadjuvant chemotherapy (NACT) and its impact oil survival on a series of locally advanced gastric carcinomas.
Methods: Downstaging was assessed comparing pre-treatment clinical and laparoscopic staging with post-operative pathologic staging on 30 consecutive patients who completed a 3-year follow-up. Tumor downstaging and the grade of pathologic response were included in a statistical correlation between tumor regression induced by NACT and 3-year survival.
Results: In this series tumor downstaging was obtained in 13 out of 30 patients. After the completion of 3-year follow-up, overall Survival was > 37.5 months with an overall survival rate of 56.7%; this figure reached up to 70.8% in those cases who benefited from a RO-resection (24/30 patients: RO-resection rate 80.0%).
Conclusions: In this study the 3-year survival for locally advanced gastric cancer treated by NACT prior to "D2" surgical resection, compares favourably with historical series treated by surgery alone. Patients who obtained T-downstaging and subsequently benefited from a RO-resection had a definitely better chance of cure, according to a complete 3-year follow-up. (c) 2006 Elsevier Ltd. Ail rights reserved.
RI Vigorita, Vincenzo/B-1871-2014; Ricci, Riccardo/E-4411-2010
OI Vigorita, Vincenzo/0000-0003-2142-4737; Ricci, Riccardo/0000-0002-9089-5084
TC 19
ZB 4
Z8 1

ZS 0
Z9 20
SN 0748-7983
UT WOS:000243136700008
PM 16930932
ER

PT J
AU Rohatgi, Pooja R.
Mansfield, Paul F.
Crane, Christopher H.
Wu, Tsung-Teh
Sunder, Punita K.
Ross, William A.
Morris, Jeffrey S.
Pisters, Peter W.
Feig, Barry W.
Gunderson, Leonard L.
Ajani, Jaffer A.
TI Surgical pathology stage by American Joint Commission on Cancer criteria predicts patient survival after preoperative chemoradiation for localized gastric carcinoma
SO CANCER
VL 107
IS 7
BP 1475
EP 1482
DI 10.1002/cncr.22180
PD OCT 1 2006
PY 2006
AB BACKGROUND. Preoperative chemoradiation for localized gastric cancer can modify baseline stage, as determined by surgical pathology stage. Therefore, the authors hypothesized that surgical pathology stage would be a better prognosticator of overall survival (OS) than baseline stage. METHODS. Patient populations were combined from 2 prospectively conducted, preoperative chemoradiation trials that used the same therapeutic strategy. Patients must have had localized gastric adenocarcinoma and were staged extensively, including endoscopic ultrasonography and laparoscopy. Patients had to be fit for surgery

medically with a technically resectable cancer. All patients provided written informed consent. Patients first received induction chemotherapy for up to 2 months followed by chemoradiation (45 grays) and an attempted surgery. OS was correlated with pretreatment and posttreatment parameters, including surgical pathology stage according to American Joint Commission on Cancer criteria. R

ESULTS. Of 74 patients who were registered, 69 patients (93%) had undergone surgery. Nineteen patients (26%) had a pathologic complete response (pathCR), and 55 patients (81%) had a curative (R0) resection. None of the pretreatment parameters correlated with OS; however, longer OS correlated with lower pathologic stage ($P < .0001$), R0 resection ($P < .001$), clinical response noted prior to surgery ($P = .002$), pathCR ($P = .004$), lower pathologic lymph node classification ($P = .006$), and lower pathologic tumor classification ($P = .03$). Pathologic stage and R0 resection were independent prognostic factors for OS (multivariate Cox model; both $P = .05$).

CONCLUSIONS. When preoperative chemoradiation strategy was employed for gastric cancer, the surgicaJ pathology stage, a reflection of cancer's biologic heterogeneity, was a better prognosticator of OS than the baseline clinical stage. Surgical pathology stage, in this setting, may serve as an intermediate endpoint for Phase II/III trials.

TC 20

ZB 9

Z8 1

ZS 0

Z9 22

SN 0008-543X

UT WOS:000240909400008

PM 16944539

ER

PT J

AU Lagarde, Sjoerd M.
ten Kate, Fieboj. W.
Reitsma, Johannes B.
Busch, Olivier R. C.
van Lanschot, J. Jan B.

TI Prognostic factors in adenocarcinoma of the esophagus or gastroesophageal junction

SO JOURNAL OF CLINICAL ONCOLOGY

VL 24

IS 26

BP 4347

EP 4355

DI 10.1200/JCO.2005.04.9445

PD SEP 10 2006

PY 2006

AB The incidence of adenocarcinoma of the esophagus is rising rapidly in Western Europe and North America. It is an aggressive disease with early lymphatic and hematogenous dissemination. TNM cancer staging systems predict survival on the basis of the anatomic extent of the tumor. However, the adequacy of the current TNM staging system for adenocarcinoma of the esophagus or gastroesophageal junction (GEJ) is questioned repeatedly. Numerous prognostic factors have been described, but are not included in the TNM system. This review describes clinical parameters, aspects of operative technique, response to preoperative chemoradiotherapy therapy, complications and established pathologic determinants found in the resection specimen that have a prognostic impact. Furthermore, their potential application in the clinical setting in patients with adenocarcinoma of the esophagus or GEJ is discussed. Future directions to improve staging systems are given.

TC 68

ZB 23

Z8 7

ZS 0

Z9 73

SN 0732-183X

UT WOS:000240645300017

PM 16963732

ER

PT J

AU Brenner, B.

Shah, M. A.

Karpeh, M. S.

Gonen, M.

Brennan, M. F.

Coit, D. G.

Klimstra, D. S.
Tang, L. H.
Kelsen, D. P.

TI A phase II trial of neoadjuvant cisplatin-fluorouracil followed by postoperative intraperitoneal floxuridine-leucovorin in patients with locally advanced gastric cancer

SO ANNALS OF ONCOLOGY
VL 17
IS 9
BP 1404
EP 1411
DI 10.1093/annonc/mdl133
PD SEP 2006
PY 2006

AB Background: The aim of the study was to evaluate the efficacy and toxicity of neoadjuvant chemotherapy with intravenous (i.v.) cisplatin and fluorouracil (5-FU), surgery and postoperative intraperitoneal (i.p.) floxuridine (FUdR) and leucovorin (LV) in patients with locally advanced gastric cancer.

Patients and methods: Preoperative staging was confirmed by laparoscopy (LAP). Two cycles of i.v. cisplatin (20 mg/m(2)/day, rapid infusion) and 5-FU (1000 mg/m(2), continuous 24-h infusion), given on days 1-5 and 29-34, were followed by a radical gastrectomy and a D2 lymphadenectomy. Patients having R0 resections were to receive three cycles of i.p. FUdR (1000 mg/m(2)) and LV (240 mg/m(2)), given on days 1-3, 15-17 and 29-31. Intraperitoneal chemotherapy was begun 5-10 days from surgery.

Results: Thirty-eight patients were treated. Both preoperative and postoperative chemotherapy were well tolerated. T stage downstaging (pretreatment LAP versus surgical pathological stage) was seen in 23% of patients. The R0 resection rate was 84%. Neither an increase in postoperative morbidity nor operative mortality was noted. With a median follow-up of 43.0 months, 15 patients (39.5%) are still alive (median survival 30.3 months). Good pathologic response, seen in five patients (15%), was associated with better survival (P = 0.053). Peritoneal and hepatic failures were found in 22% and 9% of patients, respectively. Quality of life seemed to be preserved.

Conclusions: Neoadjuvant cisplatin/5-FU followed by postoperative i.p. FUdR/LV can be safely delivered to patients undergoing radical gastrectomy and D2 lymphadenectomy. The R0 resection and the survival

rates are encouraging. An association between pathologic response and patient outcome was suggested.
RI Gonen, Mithat/E-4826-2012
TC 31
ZB 9
Z8 3
ZS 0
Z9 34
SN 0923-7534
UT WOS:000240587900010
PM 16788003
ER

PT J
AU Malaisrie, S. Chris
Hofsteffer, Wayne L.
Correa, Arlene M.
Ajani, Jaffer A.
Komaki, Ritsuko R.
Rice, David C.
Vaporciyan, Ara A.
Walsh, Garrett L.
Roth, Jack A.
Wu, Tsung T.
Swisher, Stephen G.
TI The addition of induction chemotherapy to preoperative, concurrent chemoradiotherapy improves tumor response in patients with esophageal adenocarcinoma
SO CANCER
VL 107
IS 5
BP 967
EP 974
DI 10.1002/cncr.22077
PD SEP 1 2006
PY 2006
AB BACKGROUND. Tumor viability assessed by pathologic analysis of resected specimens in patients with preoperatively treated esophageal adenocarcinoma (EAC) is a prognostic indicator. The feasibility of

induction chemotherapy followed by concurrent chemoradiotherapy (CCRT) and surgery for patients with locoregionally advanced EAC has been demonstrated. In this study, the authors evaluated the efficacy of CCRT compared with traditional concurrent chemoradiotherapy (CRT).
METHODS. The authors retrospectively reviewed 247 consecutive patients with EAC who presented for planned surgery after treatment with either CCRT or CRT from January 1997 through August 2003. Patient demographics, comorbidities, and tumor characteristics were analyzed. Pathologic tumor response, overall survival, and disease-free survival were assessed according to treatment.
RESULTS. One hundred seventeen patients received CCRT, and 130 patients received CRT before planned surgical resection. CCRT resulted in a 64% tumor response rate compared with a 51% tumor response rate in the CRT group (odds ratio, 1.73; P = .035). In the CCRT group, the median overall survival was 55 months, and the 3-year overall survival rate was 59%; in the CRT group, the median overall survival was 25 months, and the 3-year overall survival rate was 41% (hazard ratio [HR], 0.69; P = .041). In the CCRT group, the median disease-free survival was 43 months, and the 3-year disease-free survival rate was 54%; in the CRT group, the median disease-free survival was 18 months, and the 3-year disease-free survival rate was 36% (HR, 0.72; P =.047). Subset analysis of patients with clinical Stage III/IVA disease showed a median overall survival of 51 months with a 3-year overall survival rate of 58% in the CCRT group and a median overall survival of 20 months with a 3-year overall survival rate of 28% in the CRT group (HR, 0.57; P = .019).
CONCLUSIONS. in patients with EAC, CCRT improved tumor response significantly compared with traditional CRT alone. Overall survival and disease-free survival were increased in patients who received CCRT, especially in the subset of patients who had more advanced disease.
CT 31st Annual Meeting of the Western-Thoracic-Surgical-Association
CY JUN 22-25, 2005
CL Victoria, CANADA
SP Western Thorac Surg Assoc
TC 19
ZB 6
Z8 1
ZS 0
Z9 20
SN 0008-543X

UT WOS:000240054200010
PM 16874819
ER

PT J
AU Ilson, David H
TI Cancer of the gastroesophageal junction: Current therapy options.
SO Current treatment options in oncology
VL 7
IS 5
BP 410
EP 23
DI 10.1007/s11864-006-0009-6
PD 2006-Sep
PY 2006
AB Active chemotherapy agents in metastatic adenocarcinoma of the esophagus include taxanes (docetaxel or paclitaxel), 5-fluorouracil, irinotecan, platinum drugs (including cisplatin, oxaliplatin, and carboplatin), and anthracyclines. Conventional chemotherapy combines infusional 5-fluorouracil with cisplatin. The addition of a third drug to this backbone results in greater toxicity and only marginal improvements in outcome. Alternative and potentially better-tolerated chemotherapy involves two-drug regimens, combining 5-fluorouracil with a taxane or irinotecan, or combining a platinum drug with irinotecan or a taxane. Although preoperative chemotherapy improves survival compared with surgery alone, the addition of radiation therapy to chemotherapy preoperatively improves rates of curative resection, reduces local tumor recurrence, and achieves a significant rate of pathologic complete response. Combined preoperative chemotherapy and concurrent radiotherapy is the preferred preoperative strategy for locally advanced adenocarcinoma of the esophagus. Survival is improved with postoperative chemotherapy and radiotherapy if none has been delivered preoperatively.
TC 6
ZB 3
Z8 0
ZS 0
Z9 6
SN 1527-2729
UT MEDLINE:16904058

PM 16904058
ER

PT J
AU Ajani, Jaffer A.
Winter, Kathryn
Okawara, Gordon S.
Donohue, John H.
Pisters, Peter W. T.
Crane, Christopher H.
Greskovich, John F.
Anne, P. Rani
Bradley, Jeffrey D.
Willett, Christopher
Rich, Tyvin A.
TI Phase II trial of preoperative chemoradiation in patients with localized gastric adenocarcinoma (RTOG 9904): Quality of combined modality therapy and pathologic response
SO JOURNAL OF CLINICAL ONCOLOGY
VL 24
IS 24
BP 3953
EP 3958
DI 10.1200/JCO.2006.06.4840
PD AUG 20 2006
PY 2006
AB Purpose Preoperative therapy for localized gastric cancer has considerable appeal. We hypothesized that, in a cooperative group setting, preoperative chemoradiotherapy would induce a 20% pathologic complete response (pathCR) rate. Combined-modality therapy quality, survival, and safety were secondary end points.
Patients and Methods Patients with localized gastric adenocarcinoma were eligible. A negative laparoscopic evaluation was required. Patients received two cycles of induction fluorouracil, leucovorin, and cisplatin followed by concurrent radiation and chemotherapy (infusional fluorouracil and weekly paclitaxel). Resection was attempted 5 to 6 weeks after chemoradiotherapy was completed. Quality of therapy was assessed with other end points.
Results Twenty institutions participated. Forty-nine patients were

entered and 43 were assessable (12% stage 113; 37% stage II; and 52% stage III). The pathCR and R0 resection rates were 26% and 77%, respectively. At 1 year, more patients with pathCR (82%) are living than those with less than pathCR (69%). Grade 4 toxicity occurred in 21% of patients. Chemotherapy, radiotherapy, and surgery per protocol (including acceptable variations) occurred in 98%, 44%, and 63% of patients, respectively. A D2 dissection was performed in 50% of patients. Of 18 major radiotherapy variations, 17 were due to the lack of inclusion of the L3-4 vertebral interphase as prespecified.
Conclusion For localized gastric cancer, preoperative chemoradiotherapy strategy achieved a pathCR rate of more than 20% in a cooperative group setting. The quality of surgery improved (50% with D2 dissection) possibly because surgery was part of this trial. With some refinements, this preoperative chemoradiotherapy strategy is poised for a randomized comparison with postoperative adjuvant chemoradiotherapy in patients with gastric cancer.
TC 115
ZB 48
Z8 15
ZS 0
Z9 131
SN 0732-183X
UT WOS:000240052300023
PM 16921048
ER

PT J
AU Wu, Xifeng
Gu, Jian
Wu, Tsung-Teh
Swisher, Stephen G.
Liao, Zhongxin
Correa, Arlene Mr.
Liu, Jun
Etzel, Carol J.
Amos, Christopher I.
Huang, Maosheng
Chiang, Silvia S.
Milas, Luke

Hittelman, Walter N.
Ajani, Jaffer A.
TI Genetic variations in radiation and chemotherapy drug action pathways predict clinical outcomes in esophageal cancer
SO JOURNAL OF CLINICAL ONCOLOGY
VL 24
IS 23
BP 3789
EP 3798
DI 10.1200/JCO.2005.03.6640
PD AUG 10 2006
PY 2006
AB Purpose Understanding how specific genetic variants modify drug action pathways may provide informative blueprints for individualized chemotherapy.
Methods We applied a pathway-based approach to examine the impact of a comprehensive panel of genetic polymorphisms on clinical outcomes in 210 esophageal cancer patients.
Results In the Cox proportional hazards model, MTHFR Glu429Ala variant genotypes were associated with significantly improved survival (hazard ratio [HR] = 0.56; 95% CI, 0.35 to 0.89) in patients treated with fluorouracil (FU). The 3-year survival rates for patients with the variant genotypes and the wild genotypes were 65.26% and 46.43%, respectively. Joint analysis of five polymorphisms in three FU pathway genes showed a significant trend for reduced recurrence risk and longer recurrence-free survival as the number of adverse alleles decreased (P = .004). For patients receiving platinum drugs, the MDR1 C3435T variant allele was associated with significantly reduced recurrence risk (HR = 0.25; 95% CI, 0.10 to 0.64) and improved survival (HR = 0.44; 95% CI, 0.23 to 0.85). In nucleotide excision repair genes, there was a significant trend for a decreasing risk of death with a decreasing number of high-risk alleles (P for trend = .0008). In base excision repair genes, the variant alleles of XRCC1 Arg399Gln were significantly associated with the absence of pathologic complete response (odds ratio = 2.75; 95% CI, 1.14 to 6.12) and poor survival (HR = 1.92; 95% CI, 1.00 to 3.72).
Conclusion Several biologically plausible associations between individual single nucleotide polymorphisms and clinical outcomes were found. Our data also strongly suggest that combined pathway-based

analysis may provide valuable prognostic markers of clinical outcomes.
TC 113
ZB 70
Z8 6
ZS 0
Z9 121
SN 0732-183X
UT WOS:000239907500011
PM 16785472
ER

PT J
AU Mukherjee, S
Abraham, J
Brewster, A
Hardwick, R
Havard, T
Lewis, W
Askill, C
Manson, J
Williams, GT
Roberts, SA
Court, J
Crosby, T
TI Pilot study of preoperative combined modality treatment for locally advanced operable oesophageal carcinoma: Toxicities and long-term outcome
SO CLINICAL ONCOLOGY
VL 18
IS 4
BP 338
EP 344
DI 10.1016/j.clon.2005.12.009
PD MAY 2006
PY 2006
AB Aims: Paclitaxel, a radiosensitiser, has significant activity in oesophageal cancer. We aimed to conduct a feasibility study of preoperative chemoradiation using paclitaxel, cisplatin and 5-fluorouracil (5-FU).

Materials and methods: Sixteen eligible patients were enrolled. Infusional 5-FU, paclitaxel and cisplatin were given for 6 weeks before and concurrent with radiation. Conformal radiotherapy was delivered in two phases (45 Gy in 25 fractions).
Results: A total of 62.5% of the patients experienced Grade 3-4 toxicities, 50% required admission; one patient died during the neo-adjuvant phase.
Twelve (75%) patients had oesophagectomy, and two (12.5%) died after surgery. Pathological complete remission (PCR) and minimal residual disease were observed in 25% (95% Cl 0.5-49.5%) and 18% (95% Cl 0-38%) of patients, respectively, who underwent surgery. The median survival was 39.7 months (95% Cl 15, not reached); 1-, 2-, 3-, and 4-year survivals were 75% (95% Cl 56.5-99.5), 56.3% (36.5-86.7), 50% (30.6-81.6), and 50% (30.6-81.6), respectively.
Conclusion: Paclitaxel, cisplatin and 5-FU (TCF)-chemoradiation is an active regimen; the current dose schedule tested is associated with unacceptable toxicity, and cannot be recommended for routine clinical use.
TC 5
ZB 1
Z8 0
ZS 0
Z9 5
SN 0936-6555
UT WOS:000237235200010
PM 16703753
ER

PT J
AU Kodera, Yasuhiro
   Fujiwara, Michitaka
   Koike, Masahiko
   Nakao, Akimasa
TI Chemotherapy as a component of multimodal therapy for gastric carcinoma
SO WORLD JOURNAL OF GASTROENTEROLOGY
VL 12
IS 13
BP 2000
EP 2005

PD APR 7 2006
PY 2006
AB Prognosis of locally advanced gastric cancer remains poor, and several multimodality strategies involving surgery chemotherapy, and radiation have been tested in clinical trials. Phase III trial testing the benefit of postoperative adjuvant chemotherapy over treatment with surgery alone have revealed little impact on survival, with the exception of some small trials in Western nations. A large trial from the United States exploring postoperative chemoradiation was the first major success in this category. Results from Japanese trials suggest that moderate chemotherapy with oral fluoropyrimidines may be effective against less-advanced (T2-stage) cancer, although another confirmative trial is needed to prove this point. Investigators have recently turned to neoadjuvant chemotherapy, and some promising results have been reported from phase II trials using active drug combinations. In 2005, a large phase III trial testing pre- and postoperative chemotherapy has proven its survival benefit for resectable gastric cancer. Since the rate of pathologic complete response is considered to affect treatment results of this strategy, neoadjuvant chemoradiation that further increases the incidence of pathologic complete response could be a breakthrough, and phase III studies testing this strategy may be warranted in the near future. (C) 2006 The WJG Press. All rights reserved.
TC 11
ZB 10
Z8 0
ZS 0
Z9 11
SN 1007-9327
UT WOS:000239996100003
PM 16610047
ER

PT J
AU Gaca, JG
Petersen, RP
Peterson, BL
Harpole, DH
D'Amico, TA
Pappas, TN

Seigler, HF
Wolfe, WG
Tyler, DS
TI Pathologic nodal status predicts disease-free survival after neoadjuvant chemoradiation for gastroesophageal junction carcinoma
SO ANNALS OF SURGICAL ONCOLOGY
VL 13
IS 3
BP 340
EP 346
DI 10.1245/ASO.2006.02.023
PD MAR 2006
PY 2006
AB The incidence of carcinoma of the gastroesophageal junction (GEJ) is rapidly increasing, and the prognosis remains poor. We examined outcomes in patients who received neoadjuvant chemoradiation for GEJ tumors to identify factors that predict disease-free (DFS) and overall (OS) survival.

A retrospective analysis was performed of 101 consecutive patients who received chemoradiation and surgery for GEJ carcinoma between 1992 and 2001.

The median DFS and OS of all patients were 16 and 25 months, respectively. Twenty-eight patients with a complete histological response (T0N0) experienced greater DFS compared with all others (P = .02). Node-negative patients, regardless of T stage, experienced improved median DFS (24 months) compared with N1 patients (9 months; P = .01). Preoperative stage, age, tumor location, or Barrett's esophagus did not independently predict OS by univariate analysis. Multivariate analysis demonstrated that only posttreatment nodal status (P = .03)-not the degree of primary tumor response-predicted DFS.

The nodal status of patients with GEJ tumors after neoadjuvant therapy is predictive of DFS after resection. The poor outcome in node-positive patients supports postneoadjuvant therapy nodal staging, because surgical aggressiveness should be tempered by the realization that cure is unlikely and median survival is short.
CT 43rd Annual Meeting of the Society-for-Surgery-of-the-Alimentary-Tract
CY MAY 19-22, 2002
CL SAN FRANCISCO, CA
SP Soc Surg Alimentary Tract

TC 19
ZB 10
Z8 2
ZS 0
Z9 23
SN 1068-9265
UT WOS:000235449700011
PM 16485154
ER

PT J
AU Santos, AM
Sousa, H
Portela, C
Pereira, D
Pinto, D
Catarino, R
Rodrigues, C
Araujo, AP
Lopes, C
Medeiros, R
TI TP53 and P21 polymorphisms: Response to cisplatinum/paclitaxel-based chemotherapy in ovarian cancer
SO BIOCHEMICAL AND BIOPHYSICAL RESEARCH COMMUNICATIONS
VL 340
IS 1
BP 256
EP 262
DI 10.1016/j.bbrc.2005.11.176
PD FEB 3 2006
PY 2006
AB Ovarian cancer (OC) is the most lethal gynaecologic cancer and its standard treatment consists of platinum-based chemotherapy after cytoreductive surgery. The p53 protein plays a critical role on different cellular processes in response to DNA damage and it is responsible for transcriptional induction of the P21 gene. We have analysed 114 blood samples in order to investigate the effect of the TP53 codon 72 and the P21 3'UTR polymorphisms in response to cisplatinum/paclitaxel chemotherapy for OC treatment. The genotypes of

the TP53 codon 72 and P21 3'UTR polymorphism were identified using
AS-PCR and PCR-RFLP, respectively. Our results indicate that the TP53 P
allele is associated with a worse prognosis (P = 0.011) while P21
polymorphism genotypes did not reveal any statistically significant
result (P > 0.05). Furthermore, simultaneous carriers of the TP53 AA
genotype and the P21 CC genotype demonstrate a longer progression-free
interval (P = 0.020). This study suggests that the characterisation of a
genetic profile can contribute to the definition of a better
chemotherapy treatment. (c) 2005 Elsevier Inc. All rights reserved.
RI Medeiros, Rui/C-7938-2009; Sousa, Hugo/B-7714-2011
OI Medeiros, Rui/0000-0003-3010-8373; Sousa, Hugo/0000-0001-5795-2131
TC 30
ZB 18
Z8 1
ZS 0
Z9 31
SN 0006-291X
UT WOS:000234640600038
PM 16364249
ER

PT J
AU Kobayashi, Michiya
Tsuburaya, Akira
Nagata, Naoki
Miyashita, Yumi
Oba, Koji
Sakamoto, Junichi
TI A feasibility study of sequential paclitaxel and S-1 (PTX/S-1)
chemotherapy as postoperative adjuvant chemotherapy for advanced gastric
cancer.
SO Gastric cancer : official journal of the International Gastric Cancer
Association and the Japanese Gastric Cancer Association
VL 9
IS 2
BP 114
EP 9
DI 10.1007/s10120-006-0364-9
PD 2006

PY 2006

AB BACKGROUND: The most frequent recurrence pattern of advanced gastric cancer is peritoneal dissemination. We investigated the safety of and compliance with sequential chemotherapy consisting of paclitaxel and S-1, both of which are effective in the treatment of peritoneal dissemination.

METHODS: The patients in the study all had histologically proven gastric cancer, classified according to the TNM and the Japanese criteria for gastric cancer as T3-4, N0-2, P0, H0 M0, and CY0-1. In all patients, standard gastrectomy of more than a D2 dissection was performed. A dose of 80 mg/m2 of paclitaxel was administered for three courses. One course comprised weekly administration for 3 weeks, followed by a 1-week rest, except for the first course (following S-1 administration at 80 mg/m2 body surface area), in which paclitaxel was administered for only 2 weeks, followed by a 1-week rest. S-1 was administered from day 78 for four courses, with one course comprising 2 weeks' administration followed by a 1-week rest. Fifty patients received paclitaxel chemotherapy. The median age was 62.5 years overall; among the 34 male patients it was 65.5 years, and among the female patients it was 48.0 years.

RESULTS: The patient compliance rate was 84%. There were no cases of grade 4 hematological toxicity during either paclitaxel or S-1 treatment. With respect to nonhematological toxicities, there was one case of grade 3 neuropathy during the course of paclitaxel treatment and one case of grade 3 diarrhea during the course of S-1 treatment. These patients recovered and completed the scheduled treatment regimen.

CONCLUSION: Sequential chemotherapy of paclitaxel and S-1 as postoperative adjuvant chemotherapy for advanced gastric cancer is feasible.

TC 11

ZB 2

Z8 1

ZS 0

Z9 12

SN 1436-3291

UT MEDLINE:16767367

PM 16767367

ER

PT J
AU Mori, Shusuke
Kishimoto, Hirofumi
Tauchi, Katsunori
Higuchi, Kayoko
TI Histological complete response in advanced gastric cancer after 2 weeks of S-1 administration as neoadjuvant chemotherapy.
SO Gastric cancer : official journal of the International Gastric Cancer Association and the Japanese Gastric Cancer Association
VL 9
IS 2
BP 136
EP 9
DI 10.1007/s10120-005-0358-z
PD 2006
PY 2006
AB Single-agent or combined chemotherapy with the novel oral fluoropyrimidine anticancer drug, S-1 (TS-1), has been reported to be useful for the treatment of advanced gastric cancer. Here, we report a patient with advanced gastric cancer achieving a complete response (CR) after 2 weeks of administration of S-1 as neoadjuvant chemotherapy. A 78-year-old woman with epigastric pain was diagnosed as having advanced gastric cancer. S-1 was administered orally, at a dose of 50 mg twice a day every day for 2 weeks, followed by a 2-week drug-free period. No obvious adverse reactions occurred. Subsequently, the patient underwent distal partial gastrectomy with D2 lymph node dissection. Pathological examination indicated no remnant signet-ring cells in the excised specimen, no lymph node metastasis, and unnatural fibrosis in one of the No. 3 lymph nodes. The neoadjuvant chemotherapy induced a CR according to the Japanese classification of gastric carcinoma.
TC 6
ZB 0
Z8 0
ZS 1
Z9 6
SN 1436-3291
UT MEDLINE:16767370
PM 16767370
ER

PT J
AU Rohatgi, P
Swisher, SG
Correa, AM
Wu, TT
Liao, ZX
Komaki, R
Walsh, GL
Vaporciyan, AA
Rice, DC
Roth, JA
Ajani, JA
TI Characterization of pathologic complete response after preoperative chemoradiotherapy in carcinoma of the esophagus and outcome after pathologic complete response
SO CANCER
VL 104
IS 11
BP 2365
EP 2372
DI 10.1002/cncr.21439
PD DEC 1 2005
PY 2005
AB BACKGROUND. The purpose of the current study was to test the hypothesis that a lower clinical TNM stage is associated with a higher rate of pathologic complete response (pathCR) in patients with esophageal carcinoma receiving preoperative chemoradiotherapy and to determine whether Outcome after pathCR is related to clinical stage or treatment.
METHODS. Clinical parameters and surgical specimens of patients with esophageal carcinoma undergoing preoperative chemoradiotherapy were analyzed to identify predictors of pathCR. In patients with pathCR, predictors of overall survival (OS), disease-free survival (DFS), and distant recurrence were Studied.
RESULTS. Sixty-nine (29%) of 235 patients achieved pathCR. In patients with American joint Committee on Cancer (AJCC) Stage II carcinoma, the proportion achieving pathCR was significantly larger than that achieving <pathCR (65% vs. 35%; P = 0.03). The proportion of patients who received induction chemotherapy was higher in the pathCR group than in the

<pathCR group (54% vs. 46%; P = 0.05). However, neither TNM classification, primary tumor location, histologic type, gender, therapy sequence, or radiation dose (45 grays [Gy] vs. 50.4 Gy) were found to have any influence on OS or DFS. The median OS from pathCR was significantly longer than that from <pathCR (133 mos vs. 34 mos; P = 0.002). Similarly, DFS was longer in the pathCR group than in the <pathCR (P = 0.001).
CONCLUSIONS. Patients ivith clinical AJCC Stage II esophageal carcinoma are more likely to achieve a pathCR after preoperative chemoradiotherapy than are those with Stage III carcinoma. Chemoradiotherapy as primary therapy for patients with Stage I esophageal carcinoma warrants investigation as a means to preserve their esophagus.
TC 60
ZB 24
Z8 3
ZS 0
Z9 62
SN 0008-543X
UT WOS:000233419200009
PM 16245310
ER

PT J
AU Xi, H
Baldus, SE
Warnecke-Eberz, U
Brabender, J
Neiss, S
Metzger, R
Ling, FC
Dienes, HP
Bollschweiler, E
Moenig, S
Mueller, RP
Hoelscher, AH
Schneider, PM
TI High cyclooxygenase-2 expression following neoadjuvant radiochemotherapy is associated with minor histopathologic response and poor prognosis in esophageal cancer

SO CLINICAL CANCER RESEARCH
VL 11
IS 23
BP 8341
EP 8347
DI 10.1158/1078-0432.CCR-04-2373
PD DEC 1 2005
PY 2005
AB Purpose: High expression of cyclooxygenase-2 (COX-2) was shown to inhibit chemotherapy-and radiotherapy- induced apoptosis. We analyzed the association of COX-2 mRNA and protein expression with histomorphologic response to neoadjuvant radiochemotherapy in esophageal cancer.

Experimental Design: Fifty-two patients with resectable esophageal cancers (cT2-4, N-x, and MO) received neoadjuvant radiochemotherapy (cisplatin, 5-5-fluorouracil, 36 Gy) followed by transthoracic en bloc esophagectomy. Histomorphologic regression was defined as major response when resected specimens contained less than 10% of residual vital tumor cells. RNA was isolated from endoscopic biopsies (paired tumor and normal tissue) before neoadjuvant treatment and quantitative real-time reverse transcriptase-PCR (Taqman) assays were done to determine COX-2 mRNA expression levels standardized for beta-actin. COX-2 protein expression in pretreatment biopsies and post-therapeutic resection specimens was analyzed by immunostaining of tumor cells.

Results: Median COX-2 mRNA expression levels were significantly ($P < 0.0001$) different between paired tumor (median, 2.2) and normal tissues (median, 0.159). Comparison of pre-therapeutic and posttherapeutic specimens showed a significant difference ($P < 0.006$) in COX-2 protein expression. Twelve of 52 tumors showed down-regulation and 3 of 52 showed up-regulation of COX-2 protein expression during neoadjuvant radiochemotherapy. High COX-2 protein expression in post-therapeutic resection specimens was significantly associated with minor histopathologic response ($P < 0.04$) and poor prognosis (5-year survival probabilities: 26.3 +/- 8.2% for minor and 58.6% +/- 12.9% for major histopathologic response; $P < 0.01$).

Conclusion: High COX-2 protein expression following neoadjuvant radiochemotherapy in resection specimens is significantly associated with minor histopathologic response to neoadjuvant therapy and very poor prognosis.

TC 48
ZB 27
Z8 1
ZS 0
Z9 49
SN 1078-0432
UT WOS:000233701300018
PM 16322294
ER

PT J
AU Tanemura, Hiromi
Oshita, Hiroo
Kanno, Akihiro
Kusakabe, Mitsuhiko
Hatoh, Tsuneaki
Yamada, Makoto
Adachi, Takahito
Nishio, Kimitoshi
Saito, Shiro
Tomita, Eiichi
Sugiyama, Akihiko
Yamada, Tetsuya
TI [Neoadjuvant chemotherapy using TS-1 and CDDP against large type 3/Type 4/Bulky N 2 advanced gastric cancer].
SO Gan to kagaku ryoho. Cancer & chemotherapy
VL 32
IS 13
BP 2079
EP 85
PD 2005-Dec
PY 2005
AB This study was conducted to assess therapeutic results following neoadjuvant chemotherapy (NAC) for large type 3/type 4/Bulky N 2 advanced gastric cancer having a poor prognosis following resection. The subjects consisted of cases (< or = 75 y.o.) having large type 3 (diameter > or = 8 cm), type 4 or Bulky N 2 gastric cancer curable by resection based on preoperative imaging diagnostics. The NAC regimen consisted of TS-1 at 80-120 mg/body on days 1-21 p. o. and CDDP at 60

mg/m2 on day 8 divided. Upon completion of two courses of 4 weeks per course, gastrectomy with > or = D2 lymph node dissection was carried out on days 21-34. The average age of the subjects was 60.7 years, and the therapy completion rate was 80% (8/10 cases). Five of ten cases were responders diagnosed as grade 2 by histopathological examination of excised specimens (response rate 50%). Two of five responders were histopathologically evaluated as down-staging as a result of NAC (Stage III A--> f Stage I A, Stage IV--> f Stage I A). Three of the five non-responders have relapsed, and the relapse-free interval was an average 238 days. In the five responders,one has relapsed at 331 days,while the other 4 responders have shown no relapse yet. Although NAC consisting of TS-1 and CDDP is considered to be effective against advanced gastric cancer, a phase III study with surgical treatment only will be necessary to confirm its true value.
TC 2
ZB 0
Z8 0
ZS 0
Z9 2
SN 0385-0684
UT MEDLINE:16352932
PM 16352932
ER

PT J
AU Gyorffy, B
Serra, V
Jurchott, K
Abdul-Ghani, R
Garber, M
Stein, U
Petersen, I
Lage, H
Dietel, M
Schafer, R
TI Prediction of doxorubicin sensitivity in breast tumors based on gene expression profiles of drug-resistant cell lines correlates with patient survival
SO ONCOGENE

VL 24
IS 51
BP 7542
EP 7551
DI 10.1038/sj.onc.1208908
PD NOV 17 2005
PY 2005
AB Up to date clinical tests for predicting cancer chemotherapy response are not available and individual markers have shown little predictive value. We hypothesized that gene expression patterns attributable to chemotherapy-resistant cells can predict response and cancer prognosis. We contrasted the expression profiles of 13 different human tumor cell lines of gastric (EPG85-257), pancreatic (EPP85-181), colon (HT29) and breast (MCF7 and MDA-MB-231) origin and their counterparts resistant to the topoisomerase inhibitors daunorubicin, doxorubicin or mitoxantrone. We interrogated cDNA arrays with 43 000 cDNA clones (similar to 30 000 unique genes) to study the expression pattern of these cell lines. We divided gene expression profiles into two sets: we compared the expression patterns of the daunorubicin/doxorubicin- resistant cell lines and the mitoxantrone-resistant cell lines independently to the parental cell lines. For identifying predictive genes, the Prediction Analysis for Mircorarrays algorithm was used. The analysis revealed 79 genes best correlated with doxorubicin resistance and 70 genes with mitoxantrone resistance. In an independent classification experiment, we applied our model of resistance for predicting the sensitivity of 44 previously characterized breast cancer samples. The patient group characterized by the gene expression pro. le similar to those of doxorubicin-sensitive cell lines exhibited longer survival (49.7 +/- 26.1 months, n = 21, P = 0.034) than the resistant group (32.9 +/- 18.7 months, n = 23). The application of gene expression signatures derived from doxorubicin-resistant and -sensitive cell lines allowed to predict effectively clinical survival after doxorubicin monotherapy. Our approach demonstrates the significance of in vitro experiments in the development of new strategies for cancer response prediction.
TC 38
ZB 31
Z8 0
ZS 0
Z9 41

SN 0950-9232
UT WOS:000233333800004
PM 16044152
ER

PT J
AU Fujiwara, Y
Kamikonya, N
Inoue, T
Koishi, K
Yoshikawa, R
Nakao, K
Yagyu, R
Nishiwaki, M
Fujiwara, M
Kojima, S
Nakagawa, K
Yamamura, T
TI Chemoradiotherapy for T3 and T4 squamous cell carcinoma of the esophagus using low-dose FP and radiation: A preliminary report
SO ONCOLOGY REPORTS
VL 14
IS 5
BP 1177
EP 1182
PD NOV 2005
PY 2005
AB We conducted this study to evaluate the clinical significance of preoperative concurrent chemoradiotherapy (CRT) followed by esophagectomy in the management of T3 and T4 esophageal cancer. Thirty patients with squamous cell carcinoma of the esophagus received CRT followed by surgery. Preoperative CRT consisted of 5-fluorouracil (500 mg/m(2) by 24 h infusion for 5 days), cisplatin (15 mg/m(2) on days 1-5), and concurrent radiotherapy (a total dose of 40 Gy delivered in daily fractions of 2 Gy, 5 times per week). Esophagectomy was planned for 4-6 weeks after treatment and restaging. All 30 patients completed preoperative CRT. A clinical response (PR+CR) of the primary tumor was obtained in 82.8 %, and a response of metastatic nodes was seen in 23.1 %. Radical resection was possible in 17 of 29 operated patients (58.6%).

The postoperative mortality rate was 6.9%, and the hospital mortality rate was 10.3%. Ten out of 29 operated patients (34.5%) had no residual cancer in the resected esophagus, corresponding to pathological CR. The 1-year survival rate was 80.6%, the 2-year survival rate was 62.7%, and the 3-year survival rate was 53.8%. The clinical response group and the R0 or R1 group showed better survival than other patients. Preoperative CRT should be given to patients with squamous cell carcinoma, while esophagectomy remains the standard therapy for responders and has a tolerable mortality.
TC 10
ZB 5
Z8 0
ZS 0
Z9 10
SN 1021-335X
UT WOS:000232589400012
PM 16211282
ER

PT J
AU Rohatgi, PR
Swisher, SG
Correa, AM
Wu, TT
Liao, ZX
Komaki, R
Walsh, G
Vaporciyan, A
Lynch, PM
Rice, DC
Roth, JA
Ajani, JA
TI Failure patterns correlate with the proportion of residual carcinoma after preoperative chemoradiotherapy for carcinoma of the esophagus
SO CANCER
VL 104
IS 7
BP 1349
EP 1355

DI 10.1002/cncr.21346
PD OCT 1 2005
PY 2005
AB BACKGROUND. The current study was conducted to test the hypothesis that pat terns of failure are correlated with the degree of residual carcinoma after preop erative chemoradiotherapy (CRT) in patients with esophageal carcinoma.
METHODS. The authors analyzed the clinical characteristics of patients with carci noma of the esophagus who underwent preoperative CRT. The residual carcinoma in the resected specimen was categorized into 3 groups (0%, 1-50%, and > 50%). The initial patterns of failure were analyzed according to these categories.
RESULTS. Of the 235 patients who underwent CRT, 69 (29%) achieved a pathologic 5 complete response (pathCR; Group A), 109 patients (46%) achieved a response but it was less than a pathCR (1-50% residual carcinoma; Group B), and 57 (24%) had 2 no response (> 50% residual carcinoma; Group C). The time to locoregional recurrence was significantly longer for Group A compared with Group C (P = 0.05). The rate of distant metastases was significantly lower in Groups A and B compared with Group C (14% in Group A, 29% in Group B, and 33% in Group C; P = 0.03). The distant metastases-free survival was found to be significantly longer in Groups A and B compared with Group C (Group Avs. Group B, P = 0.01; Group Avs. Group C, P < 0.0001; and Group B vs. Group C, P = 0.03). A significantly higher proportion of patients in the responding groups (Groups A and B) had no disease recurrence compared with Group C (81% in Group A, 67% in Group B, and 61% in Group C; P = 0.04). The overall survival and disease-free survival were found to be significantly longer in Groups A and B compared with Group C.
CONCLUSIONS. Data from the current study demonstrate that the proportion of residual carcinoma after preoperative CRT is significantly correlated with patterns of locoregional and distant failure. Future investigations should focus on reducing the proportion of residual carcinoma and metastatic disease progression in patients with esophageal carcinoma.
TC 64
ZB 25
Z8 0
ZS 0
Z9 64
SN 0008-543X

UT WOS:000232001200003
PM 16130133
ER

PT J
AU Varadhachary, GR
Ajani, JA
TI Preoperative and adjuvant therapies for upper gastrointestinal cancers
SO EXPERT REVIEW OF ANTICANCER THERAPY
VL 5
IS 4
BP 719
EP 725
DI 10.1586/14737140.5.4.719
PD AUG 2005
PY 2005
AB Survival of esophageal, gastrointestinal junction and gastric cancers is poor given that they frequently present with locally advanced or metastatic disease. The incidence of gastrointestinal junction adenocarcinoma is increasing whereas that of squamous cell carcinoma of the esophagus is decreasing. The accuracy of staging has improved with newer diagnostic techniques, including positron emission tomography, endoscopic ultrasound and laparoscopy, and this should be integrated in prospective Phase III clinical trials evaluating neoadjuvant and adjuvant therapies for some esophageal and all gastric carcinomas. For esophageal cancer (except for one trial by Walsh and colleagues), four randomized Phase III trials comparing preoperative chemoradiation followed by surgery versus surgery alone have not shown a survival benefit. Neither have the trials, where preoperative chemoradiation followed by surgery, is compared with definitive chemoradiation. Nevertheless, it is commonly practiced in the USA and has become a preferred combined modality approach. Postoperative chemoradiation is favored in the USA for good performance status patients with resected, high-risk gastric or gastroesophageal junction carcinoma (more than Stage IA). The UK-MAGIC trial results, showing survival benefit with perioperative chemotherapy in operable gastric and lower esophageal cancers, probably has an impact on the treatment practice of these cancers in Europe and Asia. Promising results from trials involving preoperative chemoradiation followed by surgery in gastric cancer

(pathologic complete response of 20-30%) need to be further evaluated in a Phase III setting and compared with postoperative chemoradiation. Active ongoing research will help us clarify the role of preoperative and adjuvant therapies in esophageal and gastric cancers. The role of molecular profiling is evolving and will help us differentiate the responders from the nonresponders.
TC 4
ZB 1
Z8 0
ZS 0
Z9 4
SN 1473-7140
UT WOS:000234106300013
PM 16111471
ER

PT J
AU Macdonald, JS
TI Role of post-operative chemoradiation in resected gastric cancer
SO JOURNAL OF SURGICAL ONCOLOGY
VL 90
IS 3
BP 166
EP 170
DI 10.1002/jso.20223
PD JUN 1 2005
PY 2005
AB The curative management of gastric adenocarcinoma depends upon complete resection of the primary tumor. In patients with lymph node metastases in the resected specimen, the relapse and death rates from recurrent cancer are at least 70%-80%. There is continued debate over whether more extensive lymph node dissection (D2) improves survival when compared to less extensive operations. Until recently, attempts at preventing recurrence have employed adjuvant chemotherapy and have been ineffective. A large U.S. Intergroup study (INT-01 16) demonstrated that combined chemoradiation following complete gastric resection improves median time to relapse (30 vs. 19 months, P<0.0001) and overall survival (35 vs. 28 months, P = 0.01). The improvements in disease-free and overall survival resulting from postoperative chemoradiation have

defined a new standard of care. An update of the results of INT-01 16 analysis performed in 2004 with 7 years median follow-up, not only confirms the benefits from post-operative chemoradiation but also shows that chemoradiation does not produce significant long-term toxicity. The recent publication of the first large adequately powered III neoadjuvant chemotherapy trial suggested this technique might downstage tumors and increase resectability. Future advances in the therapy of resectable gastric cancer may come from studies of pre-operative neoadjuvant chemoradiation and the application of targeted therapies such as growth receptor antagonists and anti angiogenesis agents.
TC 29
ZB 11
Z8 3
ZS 0
Z9 32
SN 0022-4790
UT WOS:000229323800012
PM 15895449
ER

PT J
AU Warnecke-Eberz, U
Hokita, S
Huan, XA
Higashi, H
Baldus, SE
Metzger, R
Brabender, J
Bollschweiler, E
Mueller, RP
Dienes, HP
Hoelscher, AH
Schneider, PM
TI Overexpression of survivin mRNA is associated with a favorable prognosis following neoadjuvant radiochemotherapy in esophageal cancer
SO ONCOLOGY REPORTS
VL 13
IS 6
BP 1241

EP 1246

PD JUN 2005

PY 2005

AB Survivin is a member of the inhibitor of apoptosis (IAP) gene family known to be involved in resistance to chemo- and radiation therapy. We examined the potential of quantitative survivin mRNA expression to predict histopathologic tumor response and prognosis following neoadjuvant radiochemotherapy (cis-platinum, 5-FU, 36 Gy) in patients with locally-advanced esophageal cancer (cT2-4, Nx, MO). Tumor (T) and normal tissue (N) samples from 51 patients were collected by endoscopic biopsy prior to treatment. Survivin mRNA expression was analyzed by quantitative real-time RT-PCR assays. Histomorphologic regression was defined as a major response when resected specimens contained <10% of residual vital tumor cells or if a pathologically complete response was achieved. Some 7/51 patients had progressive disease and 44/51 proceeded to surgical resection. Of 44 resected tumors, 17 (31.4%) showed a major and 27 (61.4%) showed a minor histopathologic response; the survival rates were significantly different (p<0.01). Median absolute survivin expression was 5.1 in the tumor and 2.4 in corresponding normal tissue samples (Wilcoxon, p<0.001). Median relative (T/N ratio) survivin mRNA expression was 1.7. Survivin mRNA expression levels did not show a significant association with histomorphologic regression. Relative survivin mRNA expression of a T/N ratio >1 indicated a favorable prognosis (log-rank, p<0.003). Expression levels of survivin mRNA in pretherapeutic biopsies did not predict the extent of histomorphologic tumor regression following preoperative radiochemotherapy for esophageal cancer. However, overexpression of survivin mRNA in pretreatment biopsies (T/N ratio >1) was associated with superior survival probabilities.

TC 32

ZB 16

Z8 4

ZS 0

Z9 36

SN 1021-335X

UT WOS:000229057300036

PM 15870949

ER

PT J
AU Agarwal, B
Swisher, SG
Ajani, J
Kelly, K
Komaki, RR
Abu-Hamda, E
Correa, AM
Roth, JA
TI Differential response to preoperative chemoradiation and surgery in esophageal adenocarcinomas based on presence of Barrett's esophagus and symptomatic gastroesophageal reflux
SO ANNALS OF THORACIC SURGERY
VL 79
IS 5
BP 1716
EP 1723
DI 10.1016/j.athoracsur.2004.10.026
PD MAY 2005
PY 2005
AB Background. Barrett's esophagus and gastroesophageal reflux disease (GERD) are recognized to predispose to esophageal adenocarcinoma. Abdel-Latif and colleagues recently suggested that esophageal adenocarcinoma patients with GERD might be resistant to multimodality treatment. In this study, we investigated potential differences in clinical outcomes in esophageal adenocarcinoma patients based on the presence of identifiable Barrett's mucosa and/or history of symptomatic GERD.

Methods. Eighty-four patients with resectable esophageal adenocarcinoma, who completed the planned preoperative chemoradiation and underwent a potentially curative esophageal resection were retrospectively evaluated. Postoperative survival was compared between patients with or without underlying Barrett's esophagus and history of symptomatic GERD. Patients with pathologic complete response (path CR) and those with partial or no response (path PR) were compared to determine if presence of Barrett's esophagus and history of symptomatic GERD influence the path CR rates.

Results. We found significantly lower postoperative survival in patients with Barrett's associated adenocarcinoma (vs adenocarcinoma arising de

novo, p = 0.031) and patients with symptomatic GERD (vs patients without symptomatic GERD, p = 0.019). Furthermore, the subset of patients with path PR (vs path CR) after chemoradiation have a significantly higher proportion of patients with Barrett's esophagus (HR = 4.38, confidence interval [CI] = 1.39 to 13.83, p = 0.012) and patients with GERD (HR = 2.71, CI = 1.13 to 6.50, p = 0.026).
Conclusions. Patients with esophageal adenocarcinoma may have differences in response to preoperative chemoradiation based on the presence of Barrett's esophagus and history of symptomatic GERD. © 2005 by The Society of Thoracic Surgeons.
TC 6
ZB 2
Z8 0
ZS 0
Z9 6
SN 0003-4975
UT WOS:000228876300040
PM 15854962
ER

PT J
AU Napieralski, R
Ott, K
Kremer, M
Specht, K
Vogelsang, H
Becker, K
Muller, M
Lordick, F
Fink, U
Siewert, JR
Hofler, H
Keller, G
TI Combined GADD45A and thymidine phosphorylase expression levels predict response and survival of neoadjuvant-treated gastric cancer patients
SO CLINICAL CANCER RESEARCH
VL 11
IS 8
BP 3025

EP 3031

DI 10.1158/1078-0432.CCR-04-1605

PD APR 15 2005

PY 2005

AB Purpose: We evaluated the expression of seven therapy-related genes to predict the clinical outcome of advanced gastric cancer patients treated with a neoadjuvant chemotherapeutic protocol.

Experimental Design: Pretherapeutic, formalin-fixed, and paraffin-embedded biopsies of 61 patients, who received a 5-fluorouracil (5-FU) - and cisplatin-based chemotherapy were studied. The expressions of the 5-FU - related genes TS, DPD, and TP and of the cisplatin-related genes ERCC1, ERCC4, KU80, and GADD45A were analyzed by quantitative real-time PCR. The expression levels of single genes and of various combinations were tested for an association with response and overall survival.

Results: High DPD levels were more frequently found in nonresponding patients and were associated with worse survival. GADD45A and TP levels showed weak associations with response, but GADD45A expression correlated with survival. There was no association with response for TS expression, but tumors with a high TS level were associated with worse survival. The combination of GADD45A and TP revealed the strongest predictive effect. High expression values of TO and/or GADD45A were exclusively found in nonresponding patients (P = 0.002) and were associated with a significantly poorer survival (P = 0.04).

Conclusions: Combined gene expression levels of TP and GADD45A represent a new variable to predict the clinical outcome after neoadjuvant chemotherapy in gastric cancer. The association of DPD expression with response and survival underlines a predominant role of DPD to predict 5-FU sensitivity. The association of TS expression levels with survival but not with response suggests an importance of this gene for tumor progression.

TC 46

ZB 32

Z8 10

ZS 0

Z9 60

SN 1078-0432

UT WOS:000228406300031

PM 15837757

ER

PT J
AU Sadighi, S
Raafat, J
Mohagheghi, Ma
Meemary, F
TI Gastric carcinoma: 5 year experience of a single institute.
SO Asian Pacific journal of cancer prevention : APJCP
VL 6
IS 2
BP 195
EP 6
PD 2005 Apr-Jun
PY 2005
AB PURPOSE: Gastric cancer (GC) is the most common cause of cancer death registered in cancer institute. Background clinical information is important for cancer prevention and therefore we here present characteristics and outcome of GC patients, more than half coming from northern parts of Iran.
MATERIALS AND METHODS: we retrospectively studied records patients with pathologic diagnosis of GC referred to the Medical Oncology Department of the Cancer Institute from 1998 to 2003.
RESULTS: Four hundred and thirteen patients were registered with GC with the average age of 58 and a male to female ratio of 3/1. Tumor stage based on AJCC was stage 2(12.5%), stage 3(22%), stage 4(63%) and 2% unknown. Most common site of involvement was cardia (43%). Median survival time of all patients (with or without treatment) was 10 months overall. Gastrectomy was performed for 214 patients(39% with positive surgical margins), and 175 of the gastrectomised patients received chemotherapy. Median survival with surgery only was 7 months but 20 months with both surgery and chemotherapy. Only 21 patients received neoadjuvant chemotherapy. Median survival of patients who had response to preoperative chemotherapy was 30 months. By multivariate analysis lower extent of disease (p=0.0024), free surgical margin (p=0.0017), and chemotherapy (p=0.001) were associated with better prognosis.
CONCLUSIONS: Only curative resection with free margins was associated with a survival benefit in this study. More than 80% of patients were diagnosed in locally advanced or metastatic stage of disease and even

with neoadjuvant chemotherapy and salvage surgery the outcome was poor. Clearly more efforts need to be given to early detection of lesions to allow a better cure rate.
TC 15
ZB 1
Z8 0
ZS 0
Z9 15
SN 1513-7368
UT MEDLINE:16101332
PM 16101332
ER

PT J
AU Subramaniam, D
Murmu, N
Dieckgraefe, B
Berg, D
Anant, S
TI Helicobacter pylori strain specific difference in gastric epithelial cell response to infection; Differential COX-2 gene expression mediated by DNA and Rna binding proteins
SO GASTROENTEROLOGY
VL 128
IS 4
BP A664
EP A664
SU 2
PD APR 2005
PY 2005
CT Annual Meeting of the American-Gastroenterological-Association/Digestive-Disease-Week
CY MAY 14-19, 2005
CL Chicago, IL
SP Amer Gastroenterolog Assoc
TC 0
ZB 0
Z8 0
ZS 0

Z9 0
SN 0016-5085
UT WOS:000228619305314
ER

PT J
AU Persiani, R
D’Ugo, D
Rausei, S
Sermoneta, D
Barone, C
Pozzo, C
Ricci, R
LA Torre, G
Picciocchi, A
TI Prognostic indicators in locally advanced gastric cancer (LAGC) treated with preoperative chemotherapy and D2-gastrectomy
SO JOURNAL OF SURGICAL ONCOLOGY
VL 89
IS 4
BP 227
EP 236
DI 10.1002/jso.20207
PD MAR 15 2005
PY 2005
AB Background and Objectives: Neoadjuvant chemotherapy is increasingly considered an effective treatment option for patients with gastric carcinoma. Aim of the study is to evaluate the prognostic significance of the pathological response and of known prognostic factors in a group of accurately staged locally advanced gastric cancer (LAGC) patients.
Methods: Thirty-three patients with LAGC, staged by laparoscopy, underwent D2-gastrectomy after preoperative chemotherapy. Survival was calculated by Kaplan-Meier method and differences were assessed by the Log-rank and Breslow test. Multivariate analysis was performed using the Cox proportional hazard model in backward stepwise regression.
Results: Curative resection (R0) was achieved in 81.8% of patients. A complete or subtotal pathological response was documented in 3 and 6%, respectively. Nineteen out of thirty-three (57.6%) patients were alive and 16 of them were free of relapse at last follow-up. Survival rates

were 81, 67, and 59% at 12, 24, and 36 months, respectively. At univariate and multivariate analysis, only R0 resection was found to be an independent prognostic factor.
Conclusions: In the current study, R0 resection is the most important prognostic factor for resectable LAGC; according to our results we feel encouraged to consider neo-adjuvant chemotherapy a promising modality for increasing the R0-percentage of gastric carcinoma patients who could benefit from a curative surgery. (C) 2005 Wiley-Liss, Inc.
RI Ricci, Riccardo/E-4411-2010
OI Ricci, Riccardo/0000-0002-9089-5084
TC 23
ZB 10
Z8 12
ZS 0
Z9 33
SN 0022-4790
UT WOS:000227466000005
PM 15726615
ER

PT J
AU Ajani, JA
Mansfield, PF
Crane, CH
Wu, TT
Lunagomez, S
Lynch, PM
Janjan, N
Feig, B
Faust, J
Yao, JC
Nivers, R
Morris, J
Pisters, PW
TI Paclitaxel-based chemoradiotherapy in localized gastric carcinoma: Degree of pathologic response and not clinical parameters dictated patient outcome
SO JOURNAL OF CLINICAL ONCOLOGY
VL 23

IS 6
BP 1237
EP 1244
DI 10.1200/JCO.2005.01.305
PD FEB 20 2005
PY 2005
AB Purpose Preoperative chemoradiotherapy may increase the R0 (curative) resection rate, overall survival (OS) duration, and disease-free survival (DFS) duration. We evaluated paclitaxel-based induction chemotherapy and chemoradiotherapy in patients with localized gastric or gastroesophageal adenocarcinoma to determine its feasibility, impact on the R0 resection rate, type of pathologic response, OS, and DFS.
Patients and Methods Patients with operable, localized gastric, or gastroesophageal adenocarcinoma were eligible. Staging included endoscopic ultrasonography (EUS) and laparoscopy. Patients received two 28-day cycles of induction chemotherapy of fluorouracil, paclitaxel, and cisplatin followed by 45 Gy of radiation and concurrent fluorouracil plus paclitaxel. The cancer was restaged and surgery was attempted. Postsurgery pathologic findings and R0 resection were correlated with OS and DFS.
Results Forty-one patients were enrolled. Most carcinomas were proximal (83%) and pretreatment stage EUST3 (85%). Forty patients (98%) underwent surgery, and 78% had an R0 resection. We observed a pathologic complete response (pathCR) rate of 20% and a pathologic partial response (pathPR) rate of 15% (< 10% residual cancer cells in the resected specimen). No pretreatment parameter (sex, cancer location, baseline T stage, or baseline N stage) predicted the type of postsurgery pathologic response, OS, or DFS. However, pathCR (P =.02), pathCR + pathPB (P =.006), BO resection (P <.001), and postsurgery T and N stages (P =.01 and P <.001, respectively) were associated with OS. Same parameters were significantly correlated with DFS. Toxicity was manageable.
Conclusion The type of pathologic response but not pretreatment parameters was associated with OS and DFS. Efforts to increase the rate of pathologic response and better systemic cancer control are warranted.
TC 113
ZB 41
Z8 8
ZS 0
Z9 122

SN 0732-183X
UT WOS:000227115000025
PM 15718321
ER

PT J
AU Pedrazzani, Corrado
   Pasini, Felice
   Giacopuzzi, Simone
   Bernini, Marco
   Gabbani, Milena
   Grandinetti, Antonio
   Tomezzoli, Anna
   Ruzzenente, Andrea
   Guglielmi, Alfredo
   de Manzoni, Giovanni
TI [Preliminary results of neoadjuvant treatment of adenocarcinoma of the
   gastro-esophageal junction].
FT Risultati preliminari del trattamento neoadiuvante dell'adenocarcinoma
   del giunto esofago-gastrico.
SO Chirurgia italiana
VL 57
IS 1
BP 9
EP 14
PD 2005 Jan-Feb
PY 2005
AB The prognosis of adenocarcinoma of the gastro-oesophageal junction is
   poor and only surgery yields long-term survival in no more than 30% of
   patients. We tested a new neoadjuvant chemo-radiotherapy regimen based
   on the administration of weekly docetaxel and cisplatin and continuous
   infusion of 5-FU with concurrent radiotherapy in order to evaluate its
   feasibility and efficacy. Thirty-three patients enrolled in a
   dose-finding study and observed at the 1st Division of General Surgery
   of the University of Verona between January 2000 and October 2003
   underwent neoadjuvant chemo-radiotherapy for gastro-oesophageal junction
   adenocarcinoma (Siewert type I and II). The induction treatment was
   completed in 97.0% of cases with no treatment-related mortality. After
   completion of chemo-radiation 30 patients underwent surgery (90.9%)

while three patients did not (progression in 2 cases and chemotherapy toxicity in one). Two operated patients did not undergo resection because of liver metastasis at laparotomy (respectability: 84.8%) and 3 more cases had incomplete tumour resection (R0-resectability: 75.8%). No postoperative in-hospital mortality was observed. A complete response (pT0N0) was achieved in 7 cases (23.3%) while minimal residual disease without evidence of lymph node involvement was found in a further 5 cases (16.7%). Worthy of note is the high rate of positive histopathological responses in the later period (6 out of 8) with 4 cases presenting complete responses. This protocol regimen proved to be feasible and well tolerated. Surgery-related deaths and morbidity were not increased. A high rate of positive pathological responses was obtained particularly in the later period of the study with the increased dosage of the protocol regimen.

TC 1

ZB 1

Z8 0

ZS 0

Z9 1

SN 0009-4773

UT MEDLINE:15832733

PM 15832733

ER

PT J

AU Hamada, Madoka
Tsuji, Akihito
Iwata, Jun
Nishioka, Yutaka
Ozaki, Kazuhide
Shima, Yasuo
Horimi, Tadashi

TI Neoadjuvant chemotherapy with S-1 and surgical resection for a mucinous gastric cancer with peritoneal dissemination.

SO Gastric cancer : official journal of the International Gastric Cancer Association and the Japanese Gastric Cancer Association

VL 8

IS 1

BP 50

EP 4
DI 10.1007/s10120-004-0309-0
PD 2005
PY 2005
AB We herein report the case of a patient with mucinous gastric carcinoma with peritoneal dissemination that disappeared after neoadjuvant chemotherapy with S-1 alone. The patient has survived for over 23 months after surgery, without recurrence. A 60-year old man was referred to our hospital because of an advanced gastric cancer, detected by upper gastrointestinal endoscopy at another hospital. Staging laparoscopy was performed on October 25, 2002, and revealed massive peritoneal dissemination. Two courses of neoadjuvant chemotherapy with S-1 were administered, at 120 mg/day for 28 days, as one course. Total gastrectomy, with D2 lymph node dissection, was performed on January 24, 2003. The peritoneal dissemination had macroscopically disappeared and the cytology of the peritoneal lavage fluid was class III. His final diagnosis was gastric carcinoma, MLU, type 3, T2(SS), P0, H0, M0, N3, CY0, stage IV.
TC 0
ZB 1
Z8 0
ZS 1
Z9 1
SN 1436-3291
UT MEDLINE:15747176
PM 15747176
ER

PT J
AU Lin, FCF
Durkin, AE
Ferguson, MK
TI Induction therapy does not increase surgical morbidity after esophagectomy for cancer
SO ANNALS OF THORACIC SURGERY
VL 78
IS 5
BP 1783
EP 1789

DI 10.1016/j.athorscur.2004.04.081
PD NOV 2004
PY 2004
AB Background. A complete pathological response after induction therapy for esophageal cancer offers survival benefits, but induction therapy may increase the risk of postoperative complications and mortality.
Methods. We performed a retrospective review of consecutive patients who underwent esophagectomy for esophageal cancer to identify preoperative predictors of complications and assess the possible influence of induction therapy on surgical outcomes.
Results. Between 1988 and 2003, 170 esophagectomies were performed on our service; 95 (55.9%) underwent surgery alone and 75 (44.1%) received preoperative chemotherapy, 35 of whom also had preoperative radiation therapy. Based on multivariable regression analyses, independent covariates for complication categories included performance status (pulmonary, cardiovascular, total complications, and death), age (cardiovascular and other complications), and FEV1% (pulmonary complications). Whether patients received induction therapy was unrelated to the incidence of postoperative complications.
Conclusions. We found no evidence that induction therapy adversely influences the incidence of postoperative morbidity or mortality after esophagectomy for cancer. (C) 2004 by The Society of Thoracic Surgeons.
TC 33
ZB 7
Z8 0
ZS 0
Z9 33
SN 0003-4975
UT WOS:000224950400043
PM 15511475
ER

PT J
AU Jin, g
Liao, Zhongxing
Zhang, Zhen
Ajani, Jaffer
Swisher, Stephen
Chang, Joe Y.

Jeter, Melanda
Guerrero, Thomas
Stevens, Craig W.
Vaporciyan, Ara
Putnam, Joe Jr
Walsh, Garret
Smythe, Roy
Roth, Jack
Yao, James
Allen, Pamela
Cox, James D.
Komaki, Ritsuko
TI Induction chemotherapy improved outcomes of patients with resectable esophageal cancer who received chemoradiotherapy followed by surgery
SO International Journal of Radiation Oncology Biology Physics
VL 60
IS 2
BP 427
EP 436
PD October 1, 2004
PY 2004
AB Purpose: To investigate the effect of induction chemotherapy (CHT) before trimodality therapy on the outcome of patients with resectable cancer of the esophagus. Methods and Materials: This retrospective study included 81 consecutive patients with resectable cancer of the esophagus who received neoadjuvant chemoradiotherapy followed by esophagectomy between January 1990 and December 1998 (inclusive). Thirty-nine patients underwent chemoradiotherapy followed by esophagectomy (CHT/RT+S), 42 received additional induction CHT followed by CHT/RT+S (CHT+CHT/RT+S). Of the 81 patients, 47 were entered in institutional or national prospective trials (6 in the CHT/RT+S and 41 in the CHT+CHT/RT+S group). Induction CHT consisted of three courses of 5-fluorouracil (5-FU), cisplatin, and paclitaxel given in 28-day cycles in 37 patients (88.1%). Concurrent CHT was 5-FU and platinum based. The median radiation dose for patients treated with CHT/RT+S was 30 Gy (range, 30-50.4 Gy) delivered in a median of 10 fractions (range, 10-28 fractions) and 45 Gy (range, 30-45 Gy) in a median of 25 fractions (range, 10-25 fractions) for patients treated with CHT+CHT/RT+S. Esophagectomy was performed 6-8 weeks after completion of concurrent chemoradiotherapy. Most patients

underwent transthoracie esophagectomy (n = 66, 82.5%). Results: The pretreatment characteristics were well balanced between the two groups except for age. The median follow-up time was 29 months (22 months for the CHT/RT + S group and 38.5 months for the CHT + CHT/RT + S group) for all patients and 49 months for living patients. The actuarial overall survival (OS), disease-free survival (DFS), locoregional control (LRC), and distant metastasis-free survival (DMFS) rate at 5 years for the entire group was 46%, 36.6%, 70.7%, and 53.2%, respectively. Statistically significant differences in the OS, DFS, and LRC rates between the two groups were detected. Specifically, the 5-year OS rate was 22.8% and 71.1% in the CHT/RT + S and CHT + CHT/RT + S group (p = 0.0001), respectively. The 5-year DFS rate was 27.6% and 56.6% in the CHT/RT+S and CHT+CHT/RT+S group (p = 0.003), respectively. The 5-year LRC rate was 64.2% and 85.6% in the CHT/RT+S and CHT+CHT/RT+S group (p = 0.007), respectively. The difference in the DMFS rate between the two groups was statistically significant, with a 2- and 5-year actuarial rate of 63.9% and 51.9%, respectively, in the CHT/RT + S group and 76.9% and 74.1%, respectively, in the CHT + CHT/RT + S group (p = 0.04). The statistically significant differences persisted when patients who received gtoreq45 Gy in each group were compared. Among those patients, the 5-year OS, DFS, LRC, and DMFS rates were 23.1%, 15.4%, 58.6%, and 39.2%, respectively, for those receiving CHT/RT+S, and 71.4% (p = 0.001), 55.8% (p = 0.0008), 84.6% (p = 0.005), and 77.3% (p = 0.009), respectively, for those receiving CHT+CHT/RT+S. The pathologic complete response (pCR) rate was greater in the CHT+CHT/RT+S group compared with in the CHT/RT+S group (p = 0.008). In univariate analysis, young age, good Karnofsky performance status, Stage II disease, total radiation dose, multiple drug regimen for concurrent CHT, pCR, R0 resection, distant disease progression, and CHT+CHT/RT+S treatment proved to be prognostic factors for OS. Lower esophageallgastroesophageal junction tumor location, pCR, R0 resection, and CHT + CHT/RT + S treatment were favorable prognostic factors for LRC. Neither the total radiation dose nor multiple drugs for concurrent CHT were negative prognostic factors for LRC. In multivariate analysis, pCR, R0 resection, and treatment with CHT+CHT/RT+S were independent positive predictive factors for OS, and distant recurrences were negative predictive factors for OS. R0 resection, CHT+CHT/RT+S treatment, and lower esophageal/gastroesophageal junction tumor location were positive predictive factors for LRC. The radiation dose was not identified as an independent prognostic factor

for either OS or LRC in the multivariate analysis. Meaningful multivariate analysis could not be performed when the multiple drug variable was included in the model because of the small number of patients. Conclusion: Significantly greater LRC, DFS, OS, and DMFS were found in patients treated with CHT+CHT/ RT+S-compared with those treated with CHT/RT + S. The pCR rate was significantly higher in the CHT+CHT/ RT+S group. Induction CHT was an independent favorable prognostic factor for both LRC and OS for the population included in this study. Our data suggest that a randomized trial comparing CHT+CHT/RT+S and CHT/RT+S is warranted to assess further the merits of this treatment in patients with this currently very lethal cancer. Copyright 2004 Elsevier Inc.

TC 0
ZB 0
Z8 0
ZS 0
Z9 0
SN 0360-3016
UT BIOSIS:PREV200500005705
ER

PT J
AU Jin, J
Liao, ZX
Zhang, Z
Ajani, J
Swisher, S
Chang, JY
Jeter, M
Guerrero, T
Stevens, CW
Vaporciyan, A
Putnam, J
Walsh, G
Smythe, R
Roth, J
Yao, J
Allen, P
Cox, JD
Komaki, R

TI Induction chemotherapy improved outcomes of patients with resectable esophageal cancer who received chemoradiotherapy followed by surgery

SO INTERNATIONAL JOURNAL OF RADIATION ONCOLOGY BIOLOGY PHYSICS

VL 60

IS 2

BP 427

EP 436

DI 10.1016/j.ijrobp.2004.03.033

PD OCT 1 2004

PY 2004

AB Purpose: To investigate the effect of induction chemotherapy (CHT) before trimodality therapy on the outcome of patients with resectable cancer of the esophagus.

Methods and Materials: This retrospective study included 81 consecutive patients with resectable cancer of the esophagus who received neoadjuvant chemoradiotherapy followed by esophagectomy between January 1990 and December 1998 (inclusive). Thirty-nine patients underwent chemoradiotherapy followed by esophagectomy (CHT/RT+S), 42 received additional induction CHT followed by CHT/RT+S (CHT+CHT/RT+S). Of the 81 patients, 47 were entered in institutional or national prospective trials (6 in the CHT/RT+S and 41 in the CHT+CHT/RT+S group). Induction CHT consisted of three courses of 5-fluorouracil (5-FU), cisplatin, and paclitaxel given in 28-day cycles in 37 patients (88.1%). Concurrent CHT was 5-FU and platinum based. The median radiation dose for patients treated with CHT/RT+S was 30 Gy (range, 30-50.4 Gy) delivered in a median of 10 fractions (range, 10-28 fractions) and 45 Gy (range, 30-45 Gy) in a median of 25 fractions (range, 10-25 fractions) for patients treated with CHT+CHT/RT+S. Esophagectomy was performed 6-8 weeks after completion of concurrent chemoradiotherapy. Most patients underwent transthoracie esophagectomy (n = 66, 82.5%).

Results: The pretreatment characteristics were well balanced between the two groups except for age. The median follow-up time was 29 months (22 months for the CHT/RT + S group and 38.5 months for the CHT + CHT/RT + S group) for all patients and 49 months for living patients. The actuarial overall survival (OS), disease-free survival (DFS), locoregional control (LRC), and distant metastasis-free survival (DMFS) rate at 5 years for the entire group was 46%, 36.6%, 70.7%, and 53.2%, respectively. Statistically significant differences in the OS, DFS, and LRC rates between the two groups were detected. Specifically, the 5-year OS rate

was 22.8% and 71.1% in the CHT/RT + S and CHT + CHT/RT + S group (p = 0.0001), respectively. The 5-year DFS rate was 27.6% and 56.6% in the CHT/RT+S and CHT+CHT/RT+S group (p = 0.003), respectively. The 5-year LRC rate was 64.2% and 85.6% in the CHT/RT+S and CHT+CHT/RT+S group (p = 0.007), respectively. The difference in the DMFS rate between the two groups was statistically significant, with a 2- and 5-year actuarial rate of 63.9% and 51.9%, respectively, in the CHT/RT + S group and 76.9% and 74.1%, respectively, in the CHT + CHT/RT + S group (p = 0.04). The statistically significant differences persisted when patients who received greater than or equal to45 Gy in each group were compared. Among those patients, the 5-year OS, DFS, LRC, and DMFS rates were 23.1%, 15.4%, 58.6%, and 39.2%, respectively, for those receiving CHT/RT+S, and 71.4% (p = 0.001), 55.8% (p = 0.0008), 84.6% (p = 0.005), and 77.3% (p = 0.009), respectively, for those receiving CHT+CHT/RT+S. The pathologic complete response (pCR) rate was greater in the CHT+CHT/RT+S group compared with in the CHT/RT+S group (p = 0.008). In univariate analysis, young age, good Karnofsky performance status, Stage II disease, total radiation dose, multiple drug regimen for concurrent CHT, pCR, R0 resection, distant disease progression, and CHT+CHT/RT+S treatment proved to be prognostic factors for OS. Lower esophageallgastroesophageal junction tumor location, pCR, R0 resection, and CHT + CHT/RT + S treatment were favorable prognostic factors for LRC. Neither the total radiation dose nor multiple drugs for concurrent CHT were negative prognostic factors for LRC. In multivariate analysis, pCR, R0 resection, and treatment with CHT+CHT/RT+S were independent positive predictive factors for OS, and distant recurrences were negative predictive factors for OS. R0 resection, CHT+CHT/RT+S treatment, and lower esophageal/gastroesophageal junction tumor location were positive predictive factors for LRC. The radiation dose was not identified as an independent prognostic factor for either OS or LRC in the multivariate analysis. Meaningful multivariate analysis could not be performed when the multiple drug variable was included in the model because of the small number of patients.

Conclusion: Significantly greater LRC, DFS, OS, and DMFS were found in patients treated with CHT+CHT/ RT+S-compared with those treated with CHT/RT + S. The pCR rate was significantly higher in the CHT+CHT/ RT+S group. Induction CHT was an independent favorable prognostic factor for both LRC and OS for the population included in this study. Our data suggest that a randomized trial comparing CHT+CHT/RT+S and CHT/RT+S is

warranted to assess further the merits of this treatment in patients
with this currently very lethal cancer. (C) 2004 Elsevier Inc.
CT 44th Annual Meeting of the
American-Society-for-Therapeutic-Radiology-and-Oncology
CY OCT 06-10, 2002
CL NEW ORLEANS, LA
SP Amer Soc Therapeut Radiol & Oncol
TC 25
ZB 9
Z8 3
ZS 0
Z9 28
SN 0360-3016
UT WOS:000223966500012
PM 15380576
ER

PT J
AU Lin, Wan-Long
Li, Ding-Guo
Chen, Qiang
Lu, Han-Ming
TI Clinical and experimental study of oxaliplatin in treating human gastric
carcinoma
SO WORLD JOURNAL OF GASTROENTEROLOGY
VL 10
IS 19
BP 2911
EP 2915
PD OCT 1 2004
PY 2004
AB AIM: To evaluate the therapeutic effectiveness of oxaliplatin on human
gastric carcinoma and to explore its mechanisms.
METHODS: Twenty-two cases of stage IV gastric carcinoma received 4-6
(mean 4.6) cycles of first line combined chemotherapy with oxaliplatin
(oxaliplatin 85 mg/m(2), iv, gtt, 1 h, d 1; leukovorin 200 mg/m(2), iv,
gtt, 1 h, d 1 and d 2; 5-FU 300 mg/m(2), iv, d 1 and d 2, 5-FU,
continuous iv, gtt, 48 h; 1 cycle/2 wk). Response rate, progression-free
survival (PFS), total survival time, toxic side effects were evaluated.

The inhibitory effect of oxaliplatin on human gastric cell line SGC-7901 was detected and IC(50) was calculated by MTT. Transmission electron microscopy, flow cytometry and TUNEL were performed to evaluate the apoptosis of cell line induced by the drug. The expression of Caspase-3 m-RNA was detected by RT-PCR. AC-DEVD-CHO, a Caspase-3 specific inhibitor, was used to elucidate the role of activated Caspase-3 in the process of apoptosis induced by oxaliplatin.
RESULTS: Total response (complete and partial) occurred in 9 (40.9%) patients. Mean PFS was 4.2 mo and mean total survival time was 7.2 mo. Cumulative neurotoxicity (all grade I-II), vomiting and diarrhea, myelosuppression appeared in 93.5%, 20%, 32.9% patients, respectively. IC50 was calculated to be 0.71 mg/L by MTT assay. A maximal inhibitory rate reached 85.3%. Apoptosis index was elevated after incubated with 1 mmol/L oxaliplatin for 30 min, but without statistic significance (P>0.05). However it could be detected at a much higher degree both by flowcytometry and by TUNEL with a statistical significance (68.47 +/- 7.92% and 8.23 +/- 2.67%, respectively, P<0.05) after incubated with 1 mmol/L oxaliplatin for 2 d. By means of RT-PCR, we detected an enhancement of Caspase-3 m-RNA expression induced by oxaliplatin which was also in positive correlation with the apoptotic level. AC-DEVD-CHO, a Caspase-3 specific inhibitor, could significantly inhibit and delay apoptosis induced by oxaliplatin.
CONCLUSION: Oxaliplatin is effective and well-tolerated in patients with advanced gastric carcinoma. Oxaliplatin could significantly inhibit the growth of human gastric cell line SGC-7901. The induction of Caspase-3 m-RNA expression, activation of Caspase-3 and promotion of apoptosis may be some of the therapeutic mechanisms of oxaliplatin on gastric carcinoma. Annexin-V-fluorescein labeling flow cytometry is much more sensitive than TUNEL in detecting early stage apoptosis.
TC 9
ZB 4
Z8 4
ZS 0
Z9 12
SN 1007-9327
UT WOS:000208097300034
PM 15334700
ER

PT J
AU Imdahl, A
Schoffel, U
Ruf, G
Hopf, UT
TI Preoperative chemoradiation in esophageal cancer: Experience of a single center in 102 patients
SO ZENTRALBLATT FUR CHIRURGIE
VL 129
IS 5
BP 350
EP 355
DI 10.1005/s-2004-820389
PD OCT 2004
PY 2004
AB The (dis-)advantages of preoperative chemoradiation in patients with esophageal cancer (EC) are still controversial as data are lacking showing a clear cut benefit. Therefore, data of neoadjuvant therapy of our hospital have been analyzed.
Since 1994 102 patients with an EC (33% adenocarcinoma, 67% squamous cell cancer, scc) were operated after receiving preoperative chemoradiation (36 Gy radiation, 1.8 Gy/day for 4 weeks, 500 mg/m(2) 5-FU for 4 weeks and 20 mg/m(2) Cisplatin, day 1-5, week 1 and 4). Operation was performed usually 8-10 weeks after treatment start.
In 11.7% of patients with an adenocarcinoma a complete pathological response (CR, pT0N0M0) was observed and a pT0 stage in 20.6%. 38.2% of these patients were staged as pN0. Postoperative morbidity was observed in 66% (anastomotic leakage in 20%, recurrent nerve palsy in 23%). In-hospital mortality was 5.9%. 5-year survival was calculated as 30.5%, in patients wit a CR 66%. 26.5% of patients with a scc revealed a CR. However no effect at all was observed in 32% of these patients. 56% were staged as pN0. Postoperative morbidity was observed in 87% (anastomotic leakage in 16%, recurrent nerve palsy in 32%). In-hospital mortality was 11.8%. 5-year survival was calculated as 19.2%, in patients with a CR 45%. The impact of pN stage was significant (p = 0.0052).
These results underline the benefit of neoadjuvant therapy in patients with a CR. Further on, a pN0 stage is an important prognostic indicator. However, it remains open, whether neoadjuvant therapy leads to a downstaging of lymph node involvement, as histological confirmation in

clinically positive lymph node is seldom performed prospectively.
TC 4
ZB 1
Z8 0
ZS 0
Z9 4
SN 0044-409X
UT WOS:000224631700002
PM 15486784
ER

PT J
AU Ajani, JA
Mansfield, PF
Janjan, N
Morris, J
Pisters, PW
Lynch, PM
Feig, B
Myerson, R
Nivers, R
Cohen, DS
Gunderson, LL
TI Multi-institutional trial of preoperative chemoradiotherapy in patients with potentially resectable gastric carcinoma
SO JOURNAL OF CLINICAL ONCOLOGY
VL 22
IS 14
BP 2774
EP 2780
DI 10.1200/JCO.2004.01.015
PD JUL 15 2004
PY 2004
AB Purpose In the West, curative (RO) resection is achieved in approximately 50% of patients with localized gastric carcinoma, and more than 60% die of cancer following an RO resection. A multi-institutional study of preoperative chemoradiotherapy was done to assess the RO resection rate, pathologic complete response (pathCR) rate, safety, and survival in patients with resectable gastric carcinoma.

Patients and Methods Operable patients with localized gastric adenocarcinoma were eligible. Staging also included a laparoscopy and endoscopic ultrasonography (EUS). Patients received up to two 28-day cycles of induction chemotherapy of fluorouracil, leucovorin, and cisplatin, followed by 45 Gy of radiation plus concurrent fluorouracil. Patients were then staged and surgery was attempted.
Results Thirty-four patients were registered at three institutions. One ineligible patient was excluded. Most patients had a promixal cancer and EUST3N1 designation. Twenty-eight (85%) of 33 patients underwent surgery. The R0 resection rate was 70% and pathCR rate was 30%. A pathologic partial response (< 10% residual carcinoma in the primary) occurred in eight patients (24%). EUS T plus N and postsurgery T plus N correlation showed significant downstaging (P = < .01). The median survival time for 33 patients was 33.7 months. Patients achieving a pathCR or pathPR had a significantly longer median survival time (63.9 months) than those achieving less than pathPR (12.6 months; P = .03). There were two treatment-related deaths.
Conclusion Our data suggest that the three-step strategy of preoperative induction chemotherapy followed by chemoradiotherapy resulted in substantial pathologic response that resulted in durable survival time. This strategy is worthy of a direct comparison with postoperative adjuvant chemoradiotherapy. (C) 2004 by American Society of Clinical Oncology.
TC 144
ZB 52
Z8 16
ZS 0
Z9 161
SN 0732-183X
UT WOS:000222729000005
PM 15254045
ER

PT J
AU Ajani, JA
Walsh, G
Komaki, R
Morris, J
Swisher, SG

Putnam, JB
Lynch, PM
Wu, TT
Smythe, R
Vaporciyan, A
Faust, J
Cohen, DS
Nivers, R
Roth, JA

TI Preoperative induction of CPT-11 and cisplatin chemotherapy followed by chemoradiotherapy in patients with locoregional carcinoma of the esophagus or gastroesophageal junction

SO CANCER
VL 100
IS 11
BP 2347
EP 2354
DI 10.1002/cncr.20284
PD JUN 1 2004
PY 2004

AB BACKGROUND. Patients with localized esophageal carcinoma often develop locoregional and distant disease recurrence. The current study investigated the outcome of a new chemotherapy combination as induction therapy before chemoradiotherapy.

METHODS. Forty-three patients with resectable carcinoma of the esophagus or gastroesophageal junction were enrolled. Most of the tumors were endoscopic ultrasonography [EUS] (EUS)T3 (84%) and (EUS)N1 (63%). The patients received less than or equal to 2 6-week cycles of CPT-11 and cisplatin followed by chemoradiotherapy (45 grays with 5-fluorouracil and paclitaxel). Five to six weeks after chemoradiotherapy, the patients underwent staging and surgery. The feasibility, curative resection rates, overall and disease-free survival rates, rate of significant pathologic response, and patterns of disease recurrence were assessed.

RESULTS. Of the 43 patients, 39 (91%) underwent an R0 resection. Two patients (5%) died after surgery. A pathologic complete response (pathCR) was observed in 11 (28%) of the 39 patients (or 26% of the 43 patients). In addition, 16 patients (41% of 39 patients or 37% of 43 patients) had < 10% viable tumor in the surgical specimen (pathPR). A comparison of endoscopic ultrasonograpy T and N classifications with

surgical T and N classifications demonstrated significant down-staging
(P < 0.01). The median survival period of all 43 patients was 22.1
months. Patients who had achieved a pathCR or pathPR had a longer median
survival (25.6 months) than those who achieved less than a pathPR (18.5
months; P = 0.52). None of the clinical parameters examined were found
to correlate with survival or pathologic response.
CONCLUSIONS. CPT-11-based induction chemotherapy resulted in substantial
pathCR and pathPR rates, both of which lead to a favorable survival
outcome. The three-step strategy needs to be developed further, with the
investigation of targeted therapies with chemotherapy and radiotherapy.
(C) 2004 American Cancer Society.
TC 46
ZB 18
Z8 5
ZS 0
Z9 51
SN 0008-543X
UT WOS:000221520400009
PM 15160337
ER

PT J
AU Yamao, T
Ohta, K
Ohyama, S
Ishihara, S
Chin, K
Maruyama, M
Takahashi, T
Nakajima, T
TI A preliminary study of preoperative chemotherapy combining irinotecan
and cisplatin in patients with gastric cancer with unresectable
para-aortic lymph node metastases
SO JAPANESE JOURNAL OF CLINICAL ONCOLOGY
VL 34
IS 5
BP 255
EP 261
DI 10.1093/jjco/hyh046

PD MAY 2004

PY 2004

AB Background: A high response rate has been reported for chemotherapy combining irinotecan (CPT-11) and cisplatin (CDDP) against advanced gastric cancer. The strong anti-tumor activity of this regimen makes it very attractive as a preoperative chemotherapy. We conducted a preliminary study on preoperative chemotherapy with this regimen in patients with unresectable gastric cancer with para-aortic lymph node metastases to evaluate the feasibility of it as a treatment strategy.

Methods: Patients with unresectable para-aortic lymph node metastasis without distant hematogenous metastasis (H0, M0 and M1 LYM) and peritoneal dissemination (P0) were eligible for entry. The preoperative chemotherapy consisted of at least three cycles of CPT-11 (70 mg/m(2)) on days 1 and 15 and CDDP (80 mg/m(2)) on day 15, repeated every 4-6 weeks. Chemotherapy was followed by surgery with extended lymph node dissection in patients who achieved complete or partial responses and whose cancers were judged to be resectable.

Results: Six patients were entered into the study. In total, 18 cycles of chemotherapy were performed and five patients received at least three cycles. Objective partial responses were achieved in four patients. The major toxicities in the chemotherapy were neutropenia and diarrhea, but these were clinically acceptable. Four patients underwent surgery after the chemotherapy, and macroscopically complete resections with extended lymph node dissection were achieved in two patients. There were no therapy-related deaths. We found no pathological complete responses, but observed a definite histopathological effect caused by the chemotherapy in surgical specimens. The median survival time of all patients was 12 months. The longest survival without relapse is >6 years from the start of therapy.

Conclusions: We conclude that preoperative chemotherapy with CPT-11/CDDP therapy is feasible in patients with advanced gastric cancer and that the regimen is safe when followed by surgery. Further clinical studies with larger numbers of patients are warranted to evaluate the efficacy of this strategy.

TC 1

ZB 1

Z8 0

ZS 0

Z9 1

SN 0368-2811
UT WOS:000223607400005
PM 15231860
ER

PT J
AU Cascinu, S
Scartozzi, M
Labianca, R
Catalano, V
Silva, RR
Barni, S
Zaniboni, A
D'Angelo, A
Salvagni, S
Martignoni, G
Beretta, GD
Graziano, F
Berardi, R
Franciosi, V
TI High curative resection rate with weekly cisplatin, 5-fluorouracil, epidoxorubicin, 6S-leucovorin, glutathione, and filgastrim in patients with locally advanced, unresectable gastric cancer: a report from the Italian Group for the Study of Digestive Tract Cancer (GISCAD)
SO BRITISH JOURNAL OF CANCER
VL 90
IS 8
BP 1521
EP 1525
DI 10.1038/sj.bjc.6601752
PD APR 19 2004
PY 2004
AB The aim of the present study was to evaluate the role of a weekly preoperative chemotherapy in locally advanced, unresectable gastric cancer. In all, 82 patients with an Eastern Oncology Cooperative Group PS less than or equal to2 and normal cardiac function were enrolled onto the study. Surgical unresectability was confirmed in 52 patients (63%) at laparotomy, and in 30 (27%) cases by CT scan of the abdomen and endoscopic ultrasonography. Chemotherapy treatment was: cisplatin 40 mg

m(-2); 5- fluorouracil 500 mg m(-2); epidoxorubicin 35 mg m(-2); 6S-leucovorin 250 mg m(-2) and glutathione 1.5 gm(-2) (PELF). One cycle consisted of 8 weekly treatments. Response to chemotherapy was observed in 40 of 82 patients (49%): six (7%) complete and 34 (41%) partial responses, and in four (5%) cases a complete pathological response was confirmed. Of the 40 responding patients, 37 (45%) had potentially curative surgery. Grade 3/4 leucopenia and thrombocytopenia occurred in three and two patients. At a median follow-up of 48 months, 25 of the 37 resected patients (68%) were alive and 24 (65%) were disease free. The median and 4-year survival for the whole group was 17 months and 31%, respectively. The median survival was 12 months for inoperable patients and it was not reached in resected patients.
TC 33
ZB 21
Z8 4
ZS 1
Z9 37
SN 0007-0920
UT WOS:000221129200010
PM 15083179
ER

PT J
AU Mori, T
Fujiwara, Y
Sugita, Y
Azama, T
Ishii, T
Taniguchi, K
Yamazaki, K
Takiguchi, S
Yasuda, T
Yano, M
Monden, M
TI Application of molecular diagnosis for detection of peritoneal micrometastasis and evaluation of preoperative chemotherapy in advanced gastric carcinoma
SO ANNALS OF SURGICAL ONCOLOGY
VL 11

IS 1
BP 14
EP 20
DI 10.1245/ASO.2004.02.016
PD JAN 2004
PY 2004
AB Background: In advanced gastric cancer, peritoneal recurrence is the main cause of death after curative surgical resection. The aim of this report was to describe a novel approach for quantitative genetic diagnosis using peritoneal lavage for the identification of patients at high risk for peritoneal recurrence and for evaluation of the clinical response to intraperitoneal chemotherapy in advanced gastric cancer.
Methods: Nineteen patients with advanced gastric cancer who underwent staging laparoscopy and intraperitoneal chemotherapy before surgical resection or systemic chemotherapy between June 1999 and September 2001 were enrolled in this study. All peritoneal lavage specimens, collected at both staging laparoscopy and gastrectomy, were subjected to real-time quantitative genetic diagnosis.
Results: The reverse transcriptase polymerase chain reaction (RT-PCR) values decreased in 8 cases, stabilized as negative in 5, and increased in 6 during therapy. Patients whose RT-PCR values diminished and were ultimately negative survived except for one, and all but one patient whose values increased during treatment died of recurrence.
Conclusions: Quantitative evaluation of genetic changes can provide accurate, useful information on the effects of preoperative intra-abdominal chemotherapy and overall prognosis for patients with advanced gastric cancer.
TC 20
ZB 5
Z8 2
ZS 0
Z9 22
SN 1068-9265
UT WOS:000187802700006
PM 14699028
ER

PT J
AU Yang, Q

Cleary, KR
Yao, JC
Swisher, SG
Roth, JA
Lynch, PM
Komaki, R
Ajani, JA
Rashid, A
Hamilton, SR
Wu, TT
TI Significance of post-chemoradiation biopsy in predicting residual esophageal carcinoma in the surgical specimen
SO DISEASES OF THE ESOPHAGUS
VL 17
IS 1
BP 38
EP 43
DI 10.1111/j.1442-2050.2004.00355.x
PD 2004
PY 2004
AB Pathologic complete response in the resected esophagus can be achieved in similar to30% of patients with locally advanced esophageal or gastroesophageal junction carcinoma after preoperative chemoradiation therapy. These patients tend to have a longer survival than those who have less than pathologic complete response. Post-chemoradiation esophageal biopsy (PCEB) is used to check for the presence of residual tumor before a definitive resection is performed, but the clinical significance of PCEB findings is not clear due to the possibility of sampling bias and the superficial nature of the specimen obtained. We evaluated the use of PCEB (defined as biopsy taken within 30 days before esophagectomy) in predicting residual cancer in post-treatment esophagectomy specimens. PCEB was performed in 65 of 183 (36%) patients with locally advanced esophageal or gastroesophageal junction carcinoma, who received preoperative chemoradiation therapy. The cancer status in PCEB was correlated with the residual cancer in the esophagectomy specimens. PCEB had no cancer in 80% (52 of 65) of patients (Bx-negative) and cancer in 20% (13 of 65) of patients (Bx-positive). There was no difference in the presence of residual cancer (either in esophagus or lymph node) in esophagectomy specimens between Bx-negative

patients (77%, 40 of 52) or Bx-positive patients (92%, 12 of 13), P = 0.44. The positive predictive value of biopsy was 92% (12 of 13), negative predictive value 23% (12 of 52), sensitivity 23% (12 of 52) and specificity 92% (12 of 13). There was no difference in the residual cancer staging in the esophagectomy specimen between Bx-positive and Bx-negative patients. In contrast, residual metastatic carcinoma in lymph nodes was more frequent in Bx-positive patients (69.2%, 9 of 13) than in Bx-negative patients (28.8%, 15 of 52), P = 0.01. Our data suggest that PCEB is a specific but not a sensitive predictor of residual cancer following esophagectomy. Bx-positive patients tend to have more frequent residual tumor in lymph nodes. The utility of PCEB in predicting residual cancer in the lymph nodes needs to be explored further along with molecular predictors of response to preoperative therapy.

TC 17

ZB 5

Z8 0

ZS 0

Z9 17

SN 1120-8694

UT WOS:000221421000005

PM 15209739

ER

PT J

AU Barone, C
   Cassano, A
   Pozzo, C
   D'Ugo, D
   Schinzari, G
   Persiani, R
   Basso, M
   Brunetti, IM
   Longo, R
   Picciocchi, A

TI Long-term follow-up of a pilot phase II study with neoadjuvant epidoxorubicin, etoposide and cisplatin in gastric cancer

SO ONCOLOGY

VL 67

IS 1
BP 48
EP 53
DI 10.1159/000080285
PD 2004
PY 2004
AB Objective: The prognosis in T3-T4 or N+ gastric cancer is dismal, and the role of adjuvant therapy remains uncertain. Neoadjuvant chemotherapy could improve both resectability and survival. Here, we report the results of the long-term follow-up of a pilot study aimed at evaluating a neoadjuvant treatment in a group of patients carefully staged by computed tomography (CT), endoscopic ultrasound and laparoscopy. Methods: Twenty-five stage II-III patients with histologically proven gastric adenocarcinoma were enrolled in the study. All patients gave informed consent and were thoroughly staged. Patients were treated with epidoxorubicin (40 mg/m(2) i.v.) on days 1 and 4, etoposide (VP-16; 100 mg/m(2)) on days 1, 3 and 4 and cisplatinum (80 mg/m(2)) on day 2, every 21-28 days for 3 pre-operative cycles before CT clinical restaging followed by laparotomy and D2 gastrectomy. Three further cycles of chemotherapy were planned after radical surgery. Results: Twenty-four patients received the planned pre-operative chemotherapy and underwent surgical resection; total (13 patients) or subtotal (7 patients) R0 D2 gastrectomy was possible in 20 patients. One patient died as a result of gastric bleeding. Perioperative complications occurred in 5 patients (failure of anastomosis in 1 patient and wound infection in the other 4). The pathologic response rate included 7 partial responses (29.1%) and 10 patients with stable disease (41.7%). The main toxicity was grade 3/4 neutropenia (68%), which occurred more frequently during the postoperative chemotherapy, and fatigue (68%). Fever or infection, however, were never observed. The median disease-free survival was 37 months, and median survival has not been reached after 40 months of median follow-up. One-, 2- and 3-year survival rates were 80, 64 and 60%, respectively. Conclusion: The notable long-term survival in the present study suggests a comparison between the neoadjuvant approach, including new drug combinations, and adjuvant chemo- or chemoradio-therapy in locally advanced gastric cancer. Copyright (C) 2004 S. Karger AG, Basel.
TC 16
ZB 6

Z8 4
ZS 1
Z9 20
SN 0030-2414
UT WOS:000224208100007
PM 15459495
ER

PT J
AU Ilson, DH
TI Oesophageal cancer: new developments in systemic therapy
SO CANCER TREATMENT REVIEWS
VL 29
IS 6
BP 525
EP 532
DI 10.1016/S0305-7372(03)00104-X
PD DEC 2003
PY 2003
AB Oesophageal cancer is a rare but highly virulent malignancy in the United States and Western countries, and adenocarcinoma of the oesophagus has had the most rapid rate of increase of any solid tumour malignancy. Systemic metastatic disease is present in 50% of patients at diagnosis, and in the remaining 50% of patients presenting initially with loco-regional disease, systemic metastatic disease will develop in the vast majority of these patients. Combined chemotherapy and radiotherapy is the standard of care in the nonsurgical management of oesophageal cancer. Preoperative chemoradiotherapy followed by surgery continues to be actively studied in the surgical management of locally advanced oesophageal cancer. Pathologic complete responses are seen in 20-40% of patients, with five-year survival achieved in 25-35% of patients. The limited efficacy and substantial toxicity of conventional 5-FU-cisplatin-based chemotherapy combined with radiation, or used to treat advanced disease, has prompted the evaluation of newer agents, including the taxanes and irinotecan. These trials have indicated promising antitumour activity and therapy tolerance in both advanced disease and in combined modality therapy trials, depending on the dose and schedule of therapy administered. The advent of newer, targeted therapies, including agents directed against growth factor receptor

pathways, tumour angiogenesis, and tumour invasion and metastasis, is leading to a new generation of clinical trials combining these agents with conventional cytotoxic chemotherapy and radiation. (C) 2003 Elsevier Ltd. All rights reserved.

TC 41

ZB 30

Z8 5

ZS 0

Z9 48

SN 0305-7372

UT WOS:000186748700006

PM 14585262

ER

PT J

AU Koizumi, Yusuke
Hara, Akinori
Tomita, Masayo
Sakamoto, Kenichiro
Obata, Hirozumi
Shiomi, Takehiko
Nakajo, Shinobu
Hashimoto, Kazuaki

TI [A case of advanced gastric cancer attaining histological CR of paraaortic lymph node after TS-1/CDDP neoadjuvant chemotherapy].

SO Gan to kagaku ryoho. Cancer & chemotherapy

VL 30

IS 9

BP 1351

EP 6

PD 2003-Sep

PY 2003

AB A 60-year-old male complaining of anemic symptoms went through examinations and was diagnosed with gastric cancer (cardia, type 3', cT2, cN3, cH0, cP0, cM0, cStage IV). Further inspection showed multiple lymph node metastases, including, No. 1, 3, 7, 11, and 16 (paraaortic LNs). Poor prognosis was predicted, yet we tried neoadjuvant chemotherapy (NAC) expecting down staging of the tumor. With the efficacy and safety previously proven, we chose TS-1 + CDDP as NAC

regimen. TS-1 (tegafur gimestat otastat potassium, = 80 mg/m2) was administered orally for 21 days, followed by CDDP (cisplatin, = 60 mg/m2) i.v. on day 9. One course was completed without any significant adverse effects. The tumor itself showed PR-MR to the chemotherapy, but all the lymph nodes were expected to attain PR from CT findings. Total gastrectomy, lymph node dissection (D3) with Roux-en-Y reconstruction was performed, and histological re-evaluation was made. Macroscopically, the stomach seemed to be penetrated into serosa by the tumor, i.e., se invasion was suggested, yet histologically no cancerous cells were detected within mp and ss layer. Many of the lymph nodes were replaced with fibrosis, some with normal lymph node structure remained. Definitely no malignant cells were detected throughout all the lymph node specimens (Grade 3). Because pathological CR of paraaortic lymph nodes has never been reported previously, this case shows TS-1 + CDDP as a promising NAC regimen for advanced gastric cancer, in a sense that tumors once diagnosed as inoperable would still have the possibility of CR.

TC 0

ZB 0

Z8 0

ZS 0

Z9 0

SN 0385-0684

UT MEDLINE:14518420

PM 14518420

ER

PT J

AU Ilson, David H
   Minsky, Bruce

TI Irinotecan in esophageal cancer.

SO Oncology (Williston Park, N.Y.)

VL 17

IS 9 Suppl 8

BP 32

EP 6

PD 2003-Sep

PY 2003

AB The limited effectiveness of chemotherapy in esophageal cancer used to

palliate metastatic disease or to combine with radiotherapy in locally advanced disease has prompted the evaluation of new systemic agents. Irinotecan (CPT-11, Camptosar) has shown promising activity in a number of gastrointestinal cancers, including esophageal cancer. The phase II evaluation of the combination of weekly irinotecan and cisplatin has shown encouraging response rates exceeding 30% to 50% in esophageal and gastric cancer. Novel regimens include the combination of irinotecan with mitomycin (Mutamycin), the taxanes docetaxel (Taxotere) and paclitaxel, and continuous infusion fluorouracil (5-FU). Irinotecan is an active radiosensitizer, and trials have evaluated the combination of irinotecan with concurrent radiotherapy. We completed a phase I trial combining weekly irinotecan, cisplatin, and concurrent radiotherapy in locally advanced esophageal cancer. Minimal toxicity has been observed, with no grade 3/4 esophagitis or diarrhea, and hematologic toxicity was also surprisingly minimal. Full doses of weekly irinotecan (65 mg/m2) and cisplatin (30 mg/m2) could be combined safely with concurrent radiotherapy, with a significant rate of pathologic complete response. Phase II evaluation of this chemoradiotherapy regimen as preoperative therapy is planned at single institutions and at the cooperative group level in the United States. Further phase I and II investigation of combined irinotecan, cisplatin, and concurrent radiation is ongoing with the addition of targeted agents, including celecoxib (Celebrex), cetuximab (Erbitux), and bevacizumab (Avastin). Alternative combinations of irinotecan with radiotherapy, including the addition of docetaxel and continuous infusion 5-FU, are also undergoing phase I and II evaluation.

TC 3
ZB 1
Z8 2
ZS 0
Z9 5
SN 0890-9091
UT MEDLINE:14569846
PM 14569846
ER

PT J
AU Visser, BC
Venook, AP
Patti, MG

TI Adjuvant and neoadjuvant therapy for esophageal cancer: a critical reappraisal
SO SURGICAL ONCOLOGY-OXFORD
VL 12
IS 1
BP 1
EP 7
DI 10.1016/S0960-7404(02)00072-5
PD JUL 2003
PY 2003
AB Despite important refinements of surgical technique and significant progress in perioperative care, esophageal cancer remains highly lethal. Therefore, hope for improvement in the prognosis of esophageal cancer lies largely in the use of additional therapy. Promising data from numerous Phase II trials and a single Phase III trial led to the widespread adoption of neoadjuvant chemoradiotherapy. However, subsequent randomized trials did not conclusively demonstrate a survival benefit with any of the current neoadjuvant protocols for patients with resectable esophageal cancer. Benefit, if any, exists only for complete pathologic responders. Neoadjuvant chemoradiation should not be used in patients with resectable esophageal cancer outside of the clinical trials. Future investigation must focus on the development of new biologic or chemotherapeutic agents, and the identification of biologic markers that might predict response to chemoradiation. (C) 2003 Elsevier Science Ltd. All rights reserved.
TC 12
ZB 5
Z8 3
ZS 0
Z9 15
SN 0960-7404
UT WOS:000182503800001
PM 12689665
ER

PT J
AU Urschel, JD
Vasan, H
TI A meta-analysis of randomized controlled trials that compared

neoadjuvant chemoradiation and surgery to surgery alone for resectable esophageal cancer
SO AMERICAN JOURNAL OF SURGERY
VL 185
IS 6
BP 538
EP 543
DI 10.1016/S0002-9610(03)00066-7
PD JUN 2003
PY 2003
AB Background: Esophagectomy is a standard treatment for resectable esophageal cancer but relatively few patients are cured. Combining neoadjuvant chemoradiation with surgery may improve survival but treatment morbidity is a concern. We performed a meta-analysis of randomized controlled trials (RCTs) that compared the use of neoadjuvant chemoradiation and surgery with the use of surgery alone for esophageal cancer.
Methods: Medline and manual searches were done to identify all published RCTs that compared neoadjuvant chemoradiation and surgery with surgery alone for esophageal cancer. A random-effects model was used and the odds ratio (OR) was the principal measure of effect. Systematic quantitative review was done for outcomes unique to the neoadjuvant chemoradiation treatment group, such as pathological complete response.
Results: Nine RCTs that included 1,116 patients were selected with quality scores ranging from 1 to 3 (5-point Jadad scale). Odds ratio (95% confidence interval [CI]; P value), expressed as chemoradiation and surgery versus surgery alone (treatment versus control; values <1 favor chemoradiation-surgery arm), was 0.79 (0.59, 1.06; P = 0.12) for 1-year survival, 0.77 (0.56, 1.05; P = 0.10) for 2-year survival, 0.66 (0.47, 0.92; P = 0.016) for 3-year survival, 2.50 (1.05, 5.96; P = 0.038) for rate of resection, 0.53 (0.33, 0.84;,P = 0.007) for rate of complete resection, 1.72 (0.96, 3.07; P = 0.07) for operative mortality, 1.63 (0.99, 2.68; P = 0.053) for all treatment mortality, 0.38 (0.231 0.63; P = 0.0002) for local-regional cancer recurrence, 0.88 (0.55, 1.41; P = 0.60) for distant-cancer recurrence, and 0.47 (0.16, 1.45; P - 0.19) for all cancer recurrence. A complete pathological response to chemoradiation occurred in 21% of patients. The 3-year survival benefit was most pronounced when chemotherapy and radiotherapy were given concurrently (OR 0.45, 95% CI 0.26 to 0.79, P = 0.005) instead of

sequentially (OR 0:82, 95% CI 0.54 to 1.25, P = 0.36).
Conclusions: Compared with surgery alone, neoadjuvant chemoradiation and surgery improved 3-year survival and reduced local-regional cancer recurrence. It was associated with a lower rate of esophageal resection, but a higher rate of complete (R0) resection. There was a nonsignificant trend toward increased treatment mortality with neoadjuvant chemoradiation. Concurrent administration of neoadjuvant chemotherapy and radiotherapy was superior to sequential chemoradiation treatment scheduling. (C) 2003 Excerpta Medica, Inc. All rights reserved.
TC 340
ZB 115
Z8 21
ZS 2
Z9 364
SN 0002-9610
UT WOS:000183188400009
PM 12781882
ER

PT J
AU Roth, AD
Allal, AS
Brundler, MA
de Peyer, R
Mermillod, B
Morel, P
Huber, O
TI Neoadjuvant radiochemotherapy for locally advanced gastric cancer: a phase I-II study
SO ANNALS OF ONCOLOGY
VL 14
IS 1
BP 110
EP 115
DI 10.1093/annonc/mdg023
PD JAN 2003
PY 2003
AB Background: To study in a phase I-II trial the maximum tolerated dose, the toxicity, and the tolerance of adding radiotherapy to systemic

chemotherapy administered preoperatively in patients with locoregionally advanced gastric adenocarcinoma.
Patients and methods: Patients with adenocarcinoma of the stomach (T3-4Nany or TanyN+), performance status less than or equal to1, normal hematological, hepatic and renal functions received two cycles of cisplatin 100 mg/m(2) on day 1, 5-FU 800 mg/m(2) on days I to 4 and leucovorin 60 mg b.i.d. on days 1 to 4 q3w, concomitantly with radiation therapy escalated in three dose tiers (31.2, 38.4 and 45.6 Gy).
Results: Nineteen patients were accrued and 18 completed neoadjuvant therapy. Major toxicity consisted of grade 3/4 leucopenia and mucositis in 89% and 36% of the patients, respectively. Only one episode of febrile neutropenia was recorded. Dose level number 2 (38.4 Gy) with the chemotherapy given q4w is the recommended dose level. All patients were subsequently operated and no fatalities occurred. Pathological assessment showed one complete and eight partial responses. Two- and 3-year relapse-free survival rates were 57% and 50%, respectively. Only one patient relapsed locally. The peritoneum was the most frequent site of relapse.
Conclusions: This neoadjuvant therapeutic program is relatively well tolerated, does not seem to increase the operative risk, and might increase the locoregional control of the disease. The frequency of peritoneal involvement in relapsing patients underscores the need for a more effective systemic treatment.
TC 30
ZB 15
Z8 2
ZS 0
Z9 33
SN 0923-7534
UT WOS:000182604200018
PM 12488302
ER

PT J
AU Terrosu, G
Cedolini, C
Bresadola, V
Baccarani, U
Uzzau, A

Signor, M
Fongione, S
Buffoli, A
Iop, A
Vigevani, E
Sacco, C
Cartei, G
Bresadola, F
TI Preoperative chemoradiotherapy in cancer of the thoracic esophagus
SO DISEASES OF THE ESOPHAGUS
VL 16
IS 1
BP 9
EP 16
DI 10.1046/j.1442-2050.2003.00280.x
PD 2003
PY 2003
AB Surgery with or without adjuvant radiotherapy (RT) is the standard treatment of esophageal cancer. Preoperative radio- and chemotherapy (CT) have been introduced to improve prognosis. We report a phase II prospective non-randomized trial of preoperative RT (42 Gy/25) plus CT (cisplatin 20 mg/mq/day plus 5-fluorouracil 600 mg/mq/day, 1-5 weeks) for the treatment of thoracic esophageal cancer. From 1993, 50 patients were enrolled (40 men and 10 women, mean age 57 years, range 30-75 years). Squamous cell carcinoma accounted for 90% of cases; 10% were adenocarcinoma. Downstaging of the disease was obtained in 77.3% of cases; there were 13 (29.5%) complete responses (CR) and 21 (47.7%) partial responses (PR). Median survival was 28 and 25 months, respectively, for CR and partial response (PR) plus stable disease (SD) and progressive disease (PD) (P = 0.05). Progressive-free median survival was 22 and 17 months, respectively, for CR and PR + SD + PD (P = 0.08). Multimodal treatment of esophageal cancer showed promising results, although not significant, in terms of survival and disease progression for patients achieving a complete pathologic response.
TC 11
ZB 6
Z8 0
ZS 0
Z9 11

SN 1120-8694
UT WOS:000180968000003
PM 12581248
ER

PT J
AU Yoshimizu, Nobunari
Saikawa, Yoshiro
Kubota, Tetsuro
Akiba, Yasutada
Yoshida, Masashi
Otani, Yoshihide
Kumai, Koichiro
Hibi, Toshihumi
Kitajima, Masaki
TI Complete response of a highly advanced gastric carcinoma to preoperative chemoradiotherapy with S-1 and low-dose cisplatin.
SO Gastric cancer : official journal of the International Gastric Cancer Association and the Japanese Gastric Cancer Association
VL 6
IS 3
BP 185
EP 90
DI 10.1007/s10120-003-0239-2
PD 2003
PY 2003
AB S-1 has been developed as a new oral anticancer drug, based on the biological modulation of 5-fluorouracil. We report a patient with highly advanced gastric carcinoma who was treated successfully with a new combination chemoradiotherapy using S-1 and cisplatin (CDDP). The patient was a 37-year-old man who was diagnosed with advanced gastric carcinoma (T4N3M0) that had invaded the diaphragm and the paraaortic tissues. Remarkable tumor reduction was observed in the primary tumor and metastatic lymph nodes around the stomach after three cycles of the therapy. Radiological examination before surgery determined that a partial response (PR) had been achieved by the initial therapy. Adverse effects included only a gastrointestinal disorder that was limited to grade 2 when low-dose CDDP was utilized in the regimen, while an initial high dose of CDDP resulted in grade 3 toxicity, due to myelosuppression.

The patient underwent curative surgery, including total gastrectomy, D2 lymph node dissection, and splenectomy, after completion of the radiochemotherapy regimen. No surgical complication was observed. No tumor cells were detected by pathological evaluation of the resected stomach and all the regional lymph nodes, confirming a pathological complete response (CR; grade 3). This regimen is a potent treatment for advanced gastric carcinoma, especially when used as preoperative chemotherapy to control cancer cells.
TC 3
ZB 1
Z8 0
ZS 0
Z9 3
SN 1436-3291
UT MEDLINE:14520533
PM 14520533
ER

PT J
AU Yano, M
Shiozaki, H
Inoue, M
Tamura, S
Doki, Y
Yasuda, T
Fujiwara, Y
Tsujinaka, T
Monden, M
TI Neoadjuvant chemotherapy followed by salvage surgery: Effect on survival of patients with primary noncurative gastric cancer
SO WORLD JOURNAL OF SURGERY
VL 26
IS 9
BP 1155
EP 1159
DI 10.1007/s00268-002-6362-0
PD SEP 2002
PY 2002
AB The prognosis for gastric cancer patients who undergo noncurative

resection is extremely poor. This study evaluated the effects of neoadjuvant chemotherapy for primary noncurative gastric cancer. Thirty-four patients with biopsy-proven noncurative gastric cancer were treated with either of two neoadjuvant chemotherapies: FEMTXP (5-fluorouracil, epirubicin, methotrexate, cisplatin) or THP-FLPM (pirarubicin, 5-fluorouracil, leucovorin, cisplatin, mitomycin C). Noncurability was determined by conventional staging procedures, staging laparoscopy, and exploratory laparotomy. After chemotherapy the resectability of the tumors was reassessed. Patients who were judged to be candidates for curative resection underwent salvage surgery. Of the final 33 patients, 8 (24.2%) showed a major response [0 complete response (CR), 8 partial response (PR)]. In three patients the second laparoscopy revealed disappearance of the peritoneal metastasis. Of the 33 patients, 14 (42.4%) underwent salvage surgery, including 8 curative resections (2 curability A, 6 curability B). Pathologic examinations revealed a grade 2 response in eight patients but no grade 3 response. Univariate analysis showed the following to be significant prognostic factors: histology type (differentiated type vs. undifferentiated type; p = 0.035), T4 as a noncurative factor (T4 vs. T3 or less; p = 0.025), clinical response (PR + no change vs. progressive disease; p = 0.002), and salvage surgery (resected vs. unresected; p = 0.001). Among these factors, salvage surgery was found to be the only independent prognostic factor by multivariate analysis, with a relative risk of 0.253 and a 95% confidence interval of 0.066 to 0.974. The treatment was well tolerated. Major toxicities of WHO grade 3 or more were leukopenia in 20 (60.6%), gastrointestinal toxicities in 5 (15.2%), renal toxicities in 2 (6.1%), and alopecia in 1 (3.0%). In conclusion, neoadjuvant chemotherapy is effective for primary noncurative gastric cancer when salvage surgery can be performed. A chemotherapy regimen with a higher complete response rate would improve the prognosis of this dismal disease even more.
TC 20
ZB 8
Z8 0
ZS 2
Z9 22
SN 0364-2313
UT WOS:000177946600015
PM 12209246
ER

PT J
AU van Lanschot, JJB
   Aleman, BMP
   Richel, DJ
TI Esophageal carcinoma: surgery, radiotherapy, and chemotherapy
SO CURRENT OPINION IN GASTROENTEROLOGY
VL 18
IS 4
BP 490
EP 495
DI 10.1097/01.MOG.0000014669.50627.34
PD JUL 2002
PY 2002
AB Several new developments in the potentially curative therapy of esophageal cancer have drawn attention over the past year. There is a potential benefit of centralization of esophagectomies in dedicated centers. Early mucosal lesions are increasingly treated by local ablative therapy. Tumors invading the submucosa are preferably treated by surgical resection. There is ongoing controversy about the optimal surgical approach. Positron emission tomography scanning is a promising tool in the preoperative work-up but needs critical evaluation. The question of whether chemoradiation with voice preservation (followed by salvage surgery in case of tumor recurrence) can replace pharyngolaryngectomy in patients with cervical esophageal cancer is still unanswered. A review of eight randomized trials demonstrated that chemoradiation as primary treatment of esophageal cancer provides an absolute reduction of mortality. The addition of new drugs like paclitaxel and irinotecan into induction regimens for the treatment of advanced disease results in higher response rates but also in increased toxicity. Preoperative radiotherapy as single modality treatment does not improve overall survival, whereas the benefit of preoperative chemotherapy and chemoradiation has not been proven unequivocally. Several retrospective studies with a small number of patients suggest that local response parameters like pathologic complete response and downstaging of regional lymph node (N) status are correlated with longer survival. (C) 2002 Lippincott Williams Wilkins. Inc.
TC 2
ZB 2

Z8 0

ZS 0

Z9 3

SN 0267-1379

UT WOS:000176926100015

PM 17033326

ER

PT J

AU Ilson, DH
   Minsky, B
   Kelsen, D

TI Irinotecan, cisplatin, and radiation in esophageal cancer

SO ONCOLOGY-NEW YORK

VL 16

IS 5

BP 11

EP 15

SU 5

PD MAY 2002

PY 2002

AB The limited effectiveness of currently available chemotherapy in the treatment of advanced esophageal cancer, and the poor survival achieved in locally advanced disease with combined chemoradiotherapy with or without surgery, have prompted the evaluation of new agents. Irinotecan (CPT-11, Camptosar) has promising single-agent activity in gastrointestinal cancers. In phase II evaluation of weekly irinotecan plus cisplatin, response rates have exceeded 30% in esophageal and gastric cancers. Irinotecan is an active radiosensitizer in preclinical studies and clinical trials in lung cancer. We performed a phase I trial of weekly irinotecan, cisplatin, and concurrent radiotherapy in locally advanced esophageal cancer. Induction chemotherapy with irinotecan and cisplatin was given prior to radiotherapy, over 6 weeks, cycled on a 2-week-on, 1-week-off schedule to relieve dysphagia. Radiotherapy was given subsequently in 180-cGy daily fractions to a total dose of 5,040 cGy. Doses of chemotherapy, when given with concurrent radiotherapy, were cisplatin at 30 mg/m(2) followed by irinotecan at escalated doses (40, 50, 65, and 80 mg/m(2)), on days 1, 8, 22, and 29. Among 18 patients entered in the trial, minimal toxicity has been observed, with

no grade 314 esophagitis or diarrhea. Hematologic toxicity has been minimal. Dose-limiting toxicity (ie, requiring more than a 2-week delay in radiotherapy) has been seen in one of three patients at the 80-mg/m(2) irinotecan dose level, and accrual continues at this dose level. Among 13 evaluable patients, five complete responses have been seen (38%), including three pathologic complete responses in 10 patients undergoing surgery (30%). Asymptomatic pulmonary emboli were noted on the posttreatment computed tomography scan in 3 of 15 patients, prompting the addition of warfarin sodium (Coumadin) prophylaxis on protocol. Full doses of weekly irinotecan (65 mg/ m(2)) and cisplatin (30 mg/m(2)) can be combined safely with concurrent radiotherapy in patients with locally advanced esophageal cancer.
CT 4th Annual University-of-Texas-M D-Anderson-Cancer-Center Investigators Workshop
CY JUL 25-29, 2001
CL COLORADO SPRINGS, COLORADO
SP Univ Texas MD Anderson Canc Ctr
TC 9
ZB 5
Z8 0
ZS 0
Z9 9
SN 0890-9091
UT WOS:000175823400002
PM 12109799
ER

PT J
AU Posner, MC
Gooding, WE
Lew, JI
Rosenstein, MM
Lembersky, BC
TI Complete 5-year follow-up of a prospective phase II trial of preoperative chemoradiotherapy for esophageal cancer
SO SURGERY
VL 130
IS 4
BP 620

EP 626

DI 10.1067/msy.2001.116673

PD OCT 2001

PY 2001

AB Background. Conclusive evidence supporting the routine use of multimodality therapy in esophageal cancer is lacking. However; since long-term survival after esophagectomy alone is un us tial, clinical trials designed to identify effective therapeutic regimens are essential. M report here the 5-year res tilts of a phase II induction chemoradiotherapy trial.
Methods. Front August 1991 to January 1995, 44 patients with esophageal or gastroesophageal junction carcinoma carcinoma were treated with a combination of 5-fluorouracil, cisplatin, and interferon-alpha with concurrent external beam radiotherapy.
Results. Forty-one (93%) patients completed chemoradiotherapy, with most toxic events recorded is gi-ade I or II. Curative resection (all gross tumor removed) was achieved in 36 of 37 surgical explorations, with 10 tumors demonstrating complete pathologic response and 23 showing partial pathologic response. Median follow-up for survivors was 75 months (range, 60-100 months). Five-year survival for all patients was 32%, with a median survival of 28 months. Five-year disease-free survival in patients with curative resection was 36% (median, 26 months) and overall survival was 39%, (median, 34 months). Five-year survival for patients with curative resection whose disease responded to chemoradiotherapy was 42% (median overall survival, 36 months). Local-regional recurrence alone occurred in 3 Patients, distant failure alone in 12 patients, and combined local-regional and distant failure in 2 patients. A Cox proportionatal hazards model identified both pathologic tumor and nodal stage as independent predictors of disease free survival. Fourteen Patients (32%) were 5-year survivors; 1 of these, patients later experienced disease recurrence and died.
Conclusions. Preoperative chemoradiotherapy can result in a long-term and durable disease-free state. Only large, multi-institutional phase III trials can determine whether combined modality therapy is superior to resection alone.

CT 58th Annual Meeting of the Central-Surgical-Association

CY MAR 07-10, 2001

CL TUCSON, ARIZONA

SP Cent Surgical Assoc

TC 13
ZB 5
Z8 0
ZS 0
Z9 13
SN 0039-6060
UT WOS:000171711100023
PM 11602892
ER

PT J
AU Uchida, K
Hayashi, K
Kuramochi, H
Takasaki, K
TI Changes in intratumoral thymidylate synthase (TS) and dihydropyrimidine dehydrogenase (DPD) mRNA expression in colorectal and gastric cancer during continuous tegafur infusion
SO INTERNATIONAL JOURNAL OF ONCOLOGY
VL 19
IS 2
BP 341
EP 346
PD AUG 2001
PY 2001
AB Thymidylate synthase (TS) is the target enzyme of 5-fluorouracil (5-FU), and dihydropyrimidine dehydrogenase (DPD) is the key enzyme in the 5-FU catabolic pathway. We wanted to determine whether the TS and DPD mRNA expression levels of gastric and colorectal cancer patients would be affected by tegafur (futrafur:FT)-based chemotherapy and whether changes in their expression might be responsible for patient outcome.
Thirty-five patients with resectable advanced primary gastric cancer and 36 patients with resectable advanced primary colorectal cancer were the subjects of this study. They all underwent neoadjuvant chemotherapy with protracted infusion of FT alone or FT plus low doses of cisplatin. The TS and DPD mRNA expression levels of endoscopic biopsy specimens before chemotherapy and surgical specimens after chemotherapy were measured by TaqMan reverse transcription-PCR assay using glyceraldehyde-3-phosphate dehydrogenase (GAPDH) as the internal standard. There was a significant

difference in the DPD mRNA levels during chemotherapy in the colorectal cancers. Although the TS and DPD levels were unrelated to any conventional histopathological grade factors, colorectal cancer patients whose surgical specimens contained lower TS and DPD mRNA levels had longer disease-free intervals. The results of this study suggest that FT may affect DPD mRNA expression in colorectal cancer patients, that TS/DPD expression can be regarded as an independent prognostic factor, and that colorectal cancer patients with low TS and low DPD mRNA are candidates for FT-based adjuvant chemotherapy. In addition, quantitative analysis of the change in TS/DPD mRNA in surgical specimens during FT-based chemotherapy might be a more accurate means of predicting the postoperative disease-free interval of colorectal cancer patients than analysis of endoscopic specimens before chemotherapy. There also seems to be a relation between regulation of TS and DPD during FT chemotherapy. Elucidation of the mechanisms regulating TS and DPD mRNA expression might make it possible to predict sensitivity and/or toxicity to FT.

TC 20
ZB 18
Z8 0
ZS 0
Z9 21
SN 1019-6439
UT WOS:000169872900017
PM 11445849
ER

PT J
AU Ajani, JA
Komaki, R
Putnam, JB
Walsh, G
Nesbitt, J
Pisters, PW
Lynch, PM
Vaporciyan, A
Smythe, R
Lahoti, S
Raijman, I

Swisher, S
Martin, FD
Roth, JA
TI A three-step strategy of induction chemotherapy then chemoradiation followed by surgery in patients with potentially resectable carcinoma of the esophagus or gastroesophageal junction
SO CANCER
VL 92
IS 2
BP 279
EP 286
DI 10.1002/1097-0142(20010715)92:2<279::AID-CNCR1320>3.0.CO;2-2
PD JUL 15 2001
PY 2001
AB BACKGROUND, Patients with locoregional carcinoma of the esophagus or gastroesophageal junction have a poor survival rate after surgery. Preoperative chemotherapy or chemoradiotherapy has not improved the outcome for these patients. Our study was designed to assess the feasibility of preoperative induction combination chemotherapy in addition to chemoradiotherapy to improve the curative resection rate, local control, and survival.
PATIENTS AND METHODS. Patients having histologic proof of localized carcinoma (either squamous cell carcinoma or adenocarcinoma) of the esophagus or gastroesophageal junction underwent full classification including endoscopic ultrasonography (EUS). Patients first received up to two courses of induction chemotherapy consisting of 5-fluorouracil at 750 mg/m(2)/day as continuous infusion on Days 1-5, cisplatin at 15 mg/m(2)/day as an intravenous bolus on Daps 1-5, and paclitaxel at 200 mg/m(2) as a 24-hour intravenous infusion on Day 1. The second course was repeated on Day 29. This was followed by radiotherapy (45 grays in 25 fractions) and concurrent admission of 5-fluorouracil (300 mg/m(2)/day as a continuous infusion 5 days/week) and cisplatin (20 mg/m(2) on Days 1-5 of radiotherapy). After chemoradiotherapy, patients underwent surgery. The feasibility of this approach, curative resection rates, patient survival, and patterns of failure were assessed.
RESULTS. Thirty-seven of 38 patients enrolled were evaluable for toxicity and survival. Adenocarcinoma and distal esophageal location of carcinoma were observed frequently. Thirty-five (95%) of the 37 patients underwent surgery, all of whom had an RO (curative] resection. A

pathologic complete response was noted in 11 (30%) of the 37 total patients. In addition, 5 patients (14%) had only microscopic carcinoma. According to EUS classification, 31 [89%) of the 35 patients who underwent surgery had a T3 carcinoma whereas according to pathologic classification only 3 (9%) had a T3 carcinoma (P less than or equal to 0.01). Similarly, according to EUS classification, 23 patients (66%) had an N1 carcinoma, whereas according to pathologic classification only 7 patients (20%) had an N1 carcinoma (P less than or equal to 0.01). At a median follow-up of 20 months (minimum follow-up, 13+ months; maximum follow-up, 36+ months), the median survival duration for the 37 patients had not pet been reached. In addition, there were two deaths related to surgery.
CONCLUSIONS. These data show that the three-step strategy of preoperative paclitaxel-based induction chemotherapy then chemoradiotherapy followed by surgery is feasible and appears quite active in patients having locoregional carcinoma of the esophagus or gastroesophageal junction. Future investigations should focus on substituting cisplatin with less toxic agents and including more systemic therapy with newer classes of agents. (C) 2001 American Cancer Society.
TC 83
ZB 43
Z8 0
ZS 0
Z9 83
SN 0008-543X
UT WOS:000169943900010
PM 11466680
ER

PT J
AU Lowy, AM
Feig, BW
Janjan, N
Rich, TA
Pisters, PWT
Ajani, JA
Mansfield, PF
TI A pilot study of preoperative chemoradiotherapy for resectable gastric

cancer
SO ANNALS OF SURGICAL ONCOLOGY
VL 8
IS 6
BP 519
EP 524
DI 10.1245/aso.2001.8.6.519
PD JUL 2001
PY 2001
AB Background: The goals of this study were to assess the feasibility and toxicity of a regimen of preoperative chemoradiotherapy, surgery, and intraoperative radiotherapy in the treatment of patients with potentially resectable gastric cancer. A secondary objective was to assess pathologic response to chemoradiotherapy in the treated tumors.

Methods: Twenty-four patients were entered in the protocol. Treatment regimen consisted of 45 Gy of external beam radiotherapy with concurrent 5-FU given as a continuous infusion at a dose of 300 mg/m(2). Patients were restaged 4-6 weeks after chemoradiotherapy and then underwent surgical resection acid intraoperative radiotherapy to a dose of 10 Gy.

Results: Twenty-three patients (96%) completed chemoradiotherapy in accordance with the study protocol. Nineteen (83%) of 23 patients who completed chemoradiotherapy underwent surgical resection with D2 lymphadenectomy. Four patients (17%) had progressive disease and were not resected. The morbidity and mortality rates were 32% and 5%, respectively. Of the resected patients, two (11%) had complete pathologic responses while 12 (63%) had pathologic evidence of significant treatment effect.

Conclusions: Preoperative chemoradiotherapy for gastric cancer can be delivered safely and is well tolerated. The rate of surgical complications is consistent with that of other recently reported prospective trials of gastrectomy alone. Preoperative chemoradiotherapy resulted in significant pathologic responses in the majority of treated tumors, and complete pathologic responses were achieved in some patients.
TC 52
ZB 16
Z8 2
ZS 0
Z9 56

SN 1068-9265
UT WOS:000169879500009
PM 11456051
ER

PT J
AU Yonemura, Y
   Fujimura, T
   Ninomiya, I
   Kim, BS
   Bandou, E
   Sawa, T
   Kinoshita, K
   Endo, Y
   Sugiyama, K
   Sasaki, T
TI Prediction of peritoneal micrometastasis bp peritoneal lavaged cytology
   and reverse transcriptase-polymerase chain reaction for matrix
   metalloproteinase-7 mRNA
SO CLINICAL CANCER RESEARCH
VL 7
IS 6
BP 1647
EP 1653
PD JUN 2001
PY 2001
AB Purpose: Peritoneal dissemination is the most common cause of death
   associated with gastric cancer, In this study, we report the
   significance of molecular diagnosis of peritoneal dissemination by means
   of matrix metalloproteinase-7 (MMP-7) reverse transcriptase-PCR (RT-PCR)
   assay using preoperative peritoneal wash fluid.
   Experimental Design: Preoperative peritoneal lavage by paracentesis was
   performed on 152 patients with gastric cancer, The peritoneal lavaged
   fluid was subjected to RT-PCR analysis with primers specific for MMP-7
   and conventional cytological Papanicolaou examination.
   Results: The MMP-7 RT-PCR assay was able to detect cancer cells at
   densities even lower than 10 cells/sample. There was no signal of MMP-7
   mRNA from mesothelial cells, fibroblasts, peripheral blood, and lavaged
   fluid from patients with benign disease, Cytological examination and

MMP-7 RT-PCR assay results were positive for 27 (18%) and 28 (18%)
samples, respectively. The sensitivity for the prediction of peritoneal
dissemination by cytology and MMP-7 RT-PCR assay were 46% and 33%, but
the combination analysis using both parameters improved the sensitivity
rate with 62%. Logistic regression analysis revealed that the
cytological examination and MMP-7 RT-PCR assay are independent
predictors of peritoneal dissemination,
Conclusion: The combination of cytological examination and RT-PCR assay
of preoperative peritoneal lavaged fluid is a highly efficient and
reliable method for the selection of patients for adjuvant i.p.
chemotherapy.
TC 46
ZB 22
Z8 3
ZS 0
Z9 49
SN 1078-0432
UT WOS:000169310600023
PM 11410502
ER

PT J
AU Zambon, A
Mandruzzato, S
Parenti, A
Macino, B
Dalerba, P
Ruol, A
Merigliano, S
Zaninotto, G
Zanovello, P
TI MAGE, BAGE, and GAGE gene expression in patients with esophageal
squamous cell carcinoma and adenocarcinoma of the gastric cardia
SO CANCER
VL 91
IS 10
BP 1882
EP 1888
DI 10.1002/1097-0142(20010515)91:10<1882::AID-CNCR1210>3.3.CO;2-8

PD MAY 15 2001

PY 2001

AB BACKGROUND. The MAGE, BAGE and GAGE gene families code for distinct, tumor specific antigens that are recognized by cytotoxic T lymphocytes in the context of HLA molecules. The purpose of this study was to analyze MAGE, BAGE, and GAGE gene expression in the two major histologic types of esophageal carcinoma, squamous carcinoma (ESCc) and adenocarcinoma (CAc), and to correlate their expression patterns with the principal prognostic parameters and long term survival.

METHODS. Gene expression was analyzed in surgical samples from 24 patients with ESCc and 24 patients with Che by; reverse transcriptase-polymerase chain reaction amplification (RTF-PC R). None of the patients had received preoperative chemotherapy or radiotherapy, and all were followed until death or for a minimum of 4 years.

RESULTS. Sixteen ESCc samples (67%) and 9 CAc samples (37.5%) expressed at least one of the genes under study. The expression of each MAGE gene in the two histologic types was not significantly different, with the exception of MAGE-4, which was expressed mere in ESCc samples than in CAc samples, BAGE and GAGE expression was rather low and, in every case, was associated with the expression of at least one MAGE gene,

CONCLUSIONS, In the group as a whole, and in bath ESCc and CAc subgroups, no significant correlation emerged between the expression of any gene and prognostic parameters, such as pathologic tumor, lymph node, or disease stage. Nevertheless, BAGE or GAGE expression was related significantly to a poor prognosis, whereas the expression of ht MAGE genes (in the absence of BAGE and GAGE expression) was related significantly to a good prognosis. (C) American Cancer Society.

CT 7th World Congress of the International-Society-for-Diseases-of-the-Esophagus

CY SEP 01-04, 1998

CL MONTREAL, CANADA

SP Int Soc Dis Esophagus

TC 40

ZB 23

Z8 3

ZS 0

Z9 44

SN 0008-543X

UT WOS:000168497900006

PM 11346870
ER

PT J
AU D'Ugo, D
Persiani, R
Pende, V
Picciocchi, A
TI [Neoadjuvant chemotherapy in gastric carcinoma].
FT La chemioterapia neoadiuvante nel cancro gastrico.
SO Annali italiani di chirurgia
VL 72
IS 1
BP 47
EP 53
PD 2001 Jan-Feb
PY 2001
AB A complete surgical resection currently represents the only curative treatment option for gastric carcinoma, but as regards locally advanced cancer the possibility of local or distant recurrence remains extremely high even following a R0 resection. As far as T3-4/N+ tumors are concerned, unsatisfying results of surgery alone have stressed the need for multimodal treatments: in the recent past adjuvant chemotherapy has represented a common complementary treatment for locally advanced gastric cancer, but conclusive results of most randomized trials did not show a significant impact on long term survival. Literature review shows a growing trend throughout the 90's towards the adoption of a preoperative chemotherapy, initially evaluated as a form of "salvage" palliative treatment for unresectable patients. To date a number of phase II study suggests the efficacy of neo-adjuvant treatment administered to resectable patients with the purpose of inducing tumor downstaging, increasing the rate of R0 resections and controlling recurrencies. From March 1996 the Authors have started a controlled study on neo-adjuvant therapy for locally advanced gastric cancer. Accurate staging and patients selection were based upon immediately preoperative laparoscopy. In this ongoing study, patients are administered two preoperative cycles of EEP chemotherapy (Etoposide, Epirubicin, cis-Platin). Preliminary data have been evaluated on the first 15 cases. Grade I myelosuppression has been observed in 12/15

cases and grade II/III in 3/15 cases; 1 patient died by septic complications. Restaging has not shown progression of the disease in 13/14 cases; a macroscopic response was evidenced in 7/14 patients; 14/14 patients could undergo a successful D2 surgical resection following neo-adjuvant therapy. Pathological staging confirmed tumor downstaging in 7 out of 14 cases; 12/14 patients in this group (85.7%) could benefit a R0 resection. These preliminary data encourage us to proceed in our prospective investigation.

TC 0

ZB 0

Z8 0

ZS 0

Z9 0

SN 0003-469X

UT MEDLINE:11464495

PM 11464495

ER

PT J

AU Archer, VR
Mulholland, PJ
Stocken, DD
Darnton, SJ
Ferry, DR

TI Combined results from three phase II trials of neoadjuvant chemotherapy in operable adenocarcinoma of the oesophagus

SO CLINICAL ONCOLOGY

VL 13

IS 3

BP 164

EP 169

DI 10.1007/s001740170068

PD 2001

PY 2001

AB Adenocarcinoma of the oesophagus is a systemic disease at presentation in the majority of patients. This article analyses the impact of preoperative chemotherapy on a cohort of 68 patients. From 1990 to 1996, 68 patients with potentially operable adenocarcinoma of the oesophagus were entered into three sequential Phase II trials of neoadjuvant

chemotherapy with cisplatin/mitomycin C/ifosfamide,
cisplatin/5-fluorouracil (5-FU) and mitomycin C/cisplatin/5-FU.
Twenty-four (35%) patients had a radiological (4 complete; 20 partial) response to chemotherapy, and 52 (76%) went on to have the primary tumour resected. There was only one pathological complete responder. The overall median survival was 13 months (95% confidence interval (CI) 9-16). Survival for the 28 N-0 patients was 34 months (95% CI 14-60). The pattern of failure for resected patients was predominantly systemic (16/17).
These results indicate that neoadjuvant chemotherapy followed by surgery for adenocarcinoma of the oesophagus achieves excellent local control. The dominance, however, of distant recurrence after surgery underlines the fact that, in the majority of patients, the only hope of improving results in the future is to develop better systemic therapies.
TC 8
ZB 0
Z8 0
ZS 0
Z9 8
SN 0936-6555
UT WOS:000169705800005
PM 11527288
ER

PT J
AU Iwahashi, M
Nakamori, M
Tani, M
Yamaue, H
Sakaguchi, S
Nakamura, M
Ueda, K
Ichiro, M
Nishino, E
Tanimura, H
TI Complete response of highly advanced gastric cancer with peritoneal dissemination after new combined chemotherapy of S-1 and low-dose cisplatin: Report of a case
SO ONCOLOGY

VL 61
IS 1
BP 16
EP 22
DI 10.1159/000055347
PD 2001
PY 2001
AB TS-1((R)) (S-1) has been developed as a new oral anticancer drug based on the biological modulation of 5-fluorouracil. We treated a patient with highly advanced gastric carcinoma with a new combination chemotherapy of S-1 and low-dose cisplatin. Remarkable tumor reduction was observed after two cycles of this therapy in the primary tumor and metastatic lymph nodes, and the ascites disappeared. This was concluded to be a partial response. The only adverse effect was skin pigmentation of the fingers (grade 1), leading to early timing of operation after chemotherapy. The gastric tumor showed evident invasion to the serosa. Lymph nodes around the stomach were swollen. Peritoneal dissemination was also recognized in the omentum and mesocolon. Total gastrectomy with regional lymph node dissection was performed. Disseminated tumors were all resected. Histological examination showed that no tumor cells were detected in the gastric primary lesion, metastatic lymph nodes or disseminated peritoneal tumors, suggesting pathological complete remission. It was suggested that this regimen could be a potent combined therapy for the treatment of patients with highly advanced gastric carcinoma, and it could be useful as neoadjuvant chemotherapy. Further studies are necessary to evaluate the efficacy of this therapy.
Copyright (C) 2001 S. Karger AG, Basel.
TC 22
ZB 15
Z8 0
ZS 0
Z9 23
SN 0030-2414
UT WOS:000170289500003
PM 11474243
ER

PT J
AU Matsuda, T

Okihama, Y
Egami, K
Wada, M
Yoshioka, M
Maeda, S
Onda, M
TI Complete cure of malignant lymphoma of the stomach with a huge adrenal lesion achieved by preoperative chemotherapy and surgery: Report of a case
SO SURGERY TODAY-THE JAPANESE JOURNAL OF SURGERY
VL 31
IS 1
BP 62
EP 67
PD 2001
PY 2001
AB We report herein the case of a 53-year-old woman with malignant lymphoma of the stomach who was successfully treated by preoperative chemotherapy and surgery. The patient consulted our hospital with the chief complaint of upper abdominal pain. Endoscopy demonstrated a protruding lesion at the antral posterior wall of the stomach, and a post-biopsy pathological diagnosis of diffuse large cell type B-cell lymphoma was established. Moreover, abdominal ultrasonography. computed tomography, and magnetic resonance imaging demonstrated a tumor measuring approximately 10cm in diameter in the left adrenal gland. A total of three courses of chemotherapy using the CHOP regimen were given preoperatively. The CHOP regimen consisted of 100mg of prednisolone administered for 8 days together with 1.9mg of vincristine, 1000mg of cyclophosphamide, and 60mg of epirubicin administered intravenously on the first day. This resulted in tumor shrinkage, and a distal gastrectomy, lymph node dissection, and left adrenalectomy were subsequently performed. Since the pathological findings of the resected tissue specimen demonstrated complete elimination of the malignant lymphoma, this combination of procedures was defined as having resulted in a complete response. The postoperative course of this patient was uneventful. She is still alive without any sign of tumor recurrence 6 years after her operation, and is being followed up at the outpatient clinic.
TC 2
ZB 1

Z8 0
ZS 0
Z9 2
SN 0941-1291
UT WOS:000166785700013
PM 11213047
ER

PT J
AU Fujii, M
Kochi, M
Mochizuki, F
TI [Significance of neoadjuvant chemotherapy for gastric cancer].
SO Gan to kagaku ryoho. Cancer & chemotherapy
VL 27
IS 13
BP 2028
EP 32
PD 2000-Nov
PY 2000
AB Neoadjuvant chemotherapy for high-risk patients with advanced gastric cancer is important to increase the chance for curative resection and make unresectable gastric cancer tumors resectable by down-staging of the tumor. Tumors with H0, P0, T3, T4, or N3 are the best candidates for this therapy. Randomized controlled phase III studies are needed in conjunction with accurate staging of the disease by laparoscopy. The results of histopathologic evaluation of resected materials following preoperative chemotherapy using oral fluoropyrimidine are thought to be useful as an indicator of chemosensitivity for postoperative adjuvant setting.
TC 0
ZB 0
Z8 3
ZS 0
Z9 3
SN 0385-0684
UT MEDLINE:11103233
PM 11103233
ER

PT J
AU Chak, A
Canto, MI
Cooper, GS
Isenberg, G
Willis, J
Levitan, N
Clayman, J
Forastiere, A
Heath, E
Sivak, MV
TI Endosonographic assessment of multimodality therapy predicts survival of esophageal carcinoma patients
SO CANCER
VL 88
IS 8
BP 1788
EP 1795
PD APR 15 2000
PY 2000
AB BACKGROUND. Standard endosonographic (EUS) staging criteria are unreliable for staging esophageal carcinoma after neoadjuvant therapy; however, measurement of tumor size reduction can identify patients who have achieved a pathologic response. In the current study the authors prospectively compared survival between patients classified as responders and those classified as nonresponders by EUS.
METHODS, The maximal transverse cross-sectional area of the tumor was measured before and after neoadjuvant therapy in patients who were candidates for multimodality treatment. Response was defined as a greater than or equal to 50% reduction in tumor area.
RESULTS. A total of 59 patients at 2 centers were followed for a median of 19 months. EUS assessed response in 34 patients (58%). Overall, responders had a median survival of 17.6 months compared with 14.5 months for nonresponders (P < 0.005). Survival was significantly longer in responders compared with nonresponders in the patient subgroup who underwent surgical resection (19.7 months vs. 14.6 months; P < 0.005), the patient subgroup with adenocarcinoma (21.4 months vs. 10.8 months; P < 0.005), and the patient subgroup initially classified as having T3N1

disease (17.6 months vs. 14.1 months; $P < 0.05$). Survival was not found to differ significantly between responders and nonresponders in the subgroup of patients with squamous cell carcinoma. EUS response nas the only clinical variable that was associated with survival time in a multivariate analysis (relative hazard = 0.27; $P < 0.005$). CONCLUSIONS, Patients with esophageal carcinoma who respond to neoadjuvant treatment as identified by EUS measurement of reduction in tumor size have a significantly better prognosis than nonresponders. Cancer 2000;88:1788-95. (C) 2000 American Cancer Society.
CT Annual Meeting of the American-Society-of-Gastrointestinal-Endoscopy / Digestive Disease Week
CY 1999
CL ORLANDO, FLORIDA
SP Amer Soc Gastrointestinal Endoscopy
TC 59
ZB 18
Z8 1
ZS 0
Z9 61
SN 0008-543X
UT WOS:000086437800005
PM 10760753
ER

PT J
AU Kath, R
Fiehler, J
Schneider, CP
Hoffken, K
TI Gastric cancer in very young adults: apropos four patients and a review of the literature
SO JOURNAL OF CANCER RESEARCH AND CLINICAL ONCOLOGY
VL 126
IS 4
BP 233
EP 237
DI 10.1007/s004320050038
PD APR 2000
PY 2000

AB Whether gastric cancer in young adults differs from gastric cancer in older patients has been a controversial issue. It has long been suspected that young patients with gastric cancer have different biological features with a more aggressive course of disease and a poorer prognosis than older patients. This, however, has not been firmly substantiated. We report on the clinical course of four patients (three female and one male) with locally advanced (n = 1) or metastasized (n = 3) non-resectable gastric cancer diagnosed under the age of 29 years (23, 25, 27, 28 years). Prior to diagnosis, all three women had recently been pregnant (1-22 months). Diagnosis was endoscopically biopsy-proven and staging work-up was performed by primary explorative surgery (n = 1), laparoscopy and explorative surgery (n = 1) or CAT scan and ultrasound (n = 2). The delay between initial symptoms and diagnosis was 8-22 weeks (median, 10 weeks). The histology was signer-ring cell (n = 2) or undifferentiated (n = 2) gastric cancer. All patients had the diffuse type of gastric cancer according to Lauren. Patients were treated with the FLAP polychemotherapy regimen consisting of leucovorin, 5-fluorouracil, doxorubicin and cisplatinum, as previously reported. The best response after chemotherapy was partial in two patients. Two patients showed progressive disease. Secondary surgery was performed in three responding patients (one of them responded only locally). One patient achieved no evidence of disease after complete tumor resection (RO). In two patients surgery was palliative (R2/exploration). Three patients died 6, 4 and 8 months after diagnosis. One patient is still alive. In our series, very young adults with gastric cancer had adverse clinical and pathological features. In accordance with other reports, we observed a predominance of female patients and a possible association with recent pregnancies. Though the delay between the first symptoms and diagnosis in our patients was no different from that reported for older patients, special emphasis should be given to prompt referral and diagnostic investigations, ensuring the diagnosis of gastric cancer early in the course of disease.
TC 22
ZB 11
Z8 2
ZS 0
Z9 27
SN 0171-5216
UT WOS:000086473900008

PM 10782897
ER

PT J
AU Wanebo, HJ
Glicksman, AS
Vezeridis, MP
Clark, J
Tibbetts, L
Koness, RJ
Levy, A
TI Preoperative chemotherapy, radiotherapy, and surgical resection of locally advanced pancreatic cancer
SO ARCHIVES OF SURGERY
VL 135
IS 1
BP 81
EP 87
DI 10.1001/archsurg.135.1.81
PD JAN 2000
PY 2000
AB Hypothesis: Neoadjuvant therapy has the potential to induce regression of high-risk, locally advanced cancers and render them resectable. Preoperative chemoradiotherapy is proposed as a testable treatment concept for locally advanced pancreatic cancer.
Design: Fourteen patients (8 men, 6 women) with locally advanced pancreatic cancer were surgically explored to exclude distant spread of disease, to perform bypass of biliary and/or gastric obstruction, and to provide a jejunostomy feeding tube for long-term nutritional support. A course of chemotherapy with fluorouracil and cisplatin plus radiotherapy was then initiated. Reexploration and resection were planned subsequent to neoadjuvant therapy.
Main Outcome Measures: Tumor regression and survival.
Interventions: Surgically staged patients with locally advanced pancreatic cancer were treated by preoperative chemotherapy with bolus fluorouracil, 400 mg/m(2), on days 1 through 3 and 28 through 30 accompanied by a 3-day infusion of cisplatin, 25 mg m(2), on days 1 through 3 and 28 through 30 and concurrent radiotherapy, 45 Gy. Enteral nutritional support was maintained via jejunostomy tube.

Results: Of 14 patients who enrolled in the protocol and were initially surgically explored, 3 refused the second operation and 11 were reexplored; 2 showed progressive disease and were unresectable and 9 (81%) had definitive resection. Surgical pathologic stages of the resected patients were: Ib (2 patients), II (2 patients), and III (5 patients). Pancreatic resection included standard Whipple resection in 1 patient, resection of body and neck in 1 patient, and extended resection in 6 patients (portal vein resection in 6, arterial resection in 4). One patient who was considered too frail for resection had core biopsies of the pancreatic head, node dissection, and an interstitial implant of the tumorous head. Pathologic response: 2 patients had apparent complete pathologic response; 1 patient had no residual cancer in the pancreatectomy specimen, the other patient who had an iridium 192 interstitial implant had normal core biopsies of the pancreatic head. Five patients had minimal residual cancer in the resected pancreas or microscopic foci only with extensive fibrosis, and 2 patients had fully viable residual cancer. Lymph node downstaging occurred in 2 of 4 patients who had positive peripancreatic nodes at the initial surgical staging. There was 1 postoperative death at 10 days. Sepsis, prolonged ileus, and failure to thrive were major complications. In the definitive surgery group the median survival was 19 months after beginning chemoradiotherapy and 16 months after definitive surgery. The absolute 5-year survival was 11% of 9 patients, 1 is surviving 96 months (with no evidence of disease) after chemoradiotherapy and extended pancreatic resection including resection of the superior mesenteric artery and the portal vein for stage III cancer. In the nonresected group the mean survival was 9 months (survial range, 7-12 months) after initiation of chemoradiotherapy.

Conclusion: A pilot study of preoperative chemoradiotherapy with infusional cisplatin and radiation induced a high rate of clinical pathologic response in patients with locally advanced pancreatic cancer and merits further study in these high-risk patients.

CT 50th Annual Meeting of the Society-of-Surgical-Oncology

CY MAR 20-23, 1997

CL CHICAGO, ILLINOIS

SP Soc Surg Oncol

TC 89

ZB 36

Z8 0

ZS 1
Z9 91
SN 0004-0010
UT WOS:000084555100018
PM 10636353
ER

PT J
AU Geh, JI
Glynne-Jones, R
Kwok, QSK
Banerji, U
Livingstone, JI
Townsend, ER
Harrison, RA
Mitchell, IC
TI Preoperative ECF chemotherapy in gastro-oesophageal adenocarcinoma
SO CLINICAL ONCOLOGY
VL 12
IS 3
BP 182
EP 187
DI 10.1007/s001740070063
PD 2000
PY 2000
AB Epirubicin, cisplatin and continuous 5-fluorouracil (5-FU) infusion (ECF) has been reported to result in high clinical response rates in advanced gastro-oesophageal adenocarcinoma and is currently the 'gold standard' chemotherapy regimen for this tumour site. Despite this, its role as preoperative (neoadjuvant) treatment is unproven and therefore remains under investigation. We report our experience using ECF (intravenous epirubicin 50 mg/m(2) and cisplatin 60 mg/m(2) every 3 weeks, with continuous infusion of 5-FU 200 mg/m(2) per day) as preoperative treatment in locally advanced adenocarcinoma of the lower oesophagus, gastro-oesophageal junction and stomach. Of the 23 patients treated (median age 54 years), 19 had potentially resectable disease, four were unresectable and seven had radiological evidence of lymph node involvement. A median of four cycles of ECF was delivered (range 1-6). Ten of 12 patients (83%) with dysphagia reported improvement of

symptoms. Clinical disease progression occurred in six patients (26%) during chemotherapy. WHO grade 3 or 3 toxicity occurred in six patients (26%): four haematological, one mucositis, one vomiting. Seventeen patients (74%) proceeded to surgery; 14 (61%) were resected and three were unresectable. There were two (12%) postoperative deaths from respiratory failure. Major pathological response was seen in three patients (13%): one pathological complete response, two microscopic residual disease. Two patients had Stage II (T2N0-1) disease and nine were Stage III (T3-4N0-1) None of the patients with initially unresectable disease was rendered resectable. After a median follow-up interval of 33 months (range 26-53), the overall median survival was 12 months and 2-year survival was 30%. All patients who were initially unresectable or had radiological evidence of lymph node involvement have died. Therefore, despite good symptomatic response rates, ECF chemotherapy given in the preoperative setting did not appear to improve the outcome of patients with unresectable or radiologically lymph node-positive gastro-oesophageal adenocarcinoma. The role of ECF chemotherapy in resectable tumours is unclear and is currently under investigation in the randomized MRC Adjuvant Gastric Infusional Chemotherapy (MAGIC) study.
TC 15
ZB 8
Z8 4
ZS 1
Z9 19
SN 0936-6555
UT WOS:000088365500011
PM 10942336
ER

PT J
AU Schuhmacher, C
Fink, U
Siewert, JR
TI Preoperative down-staging in advanced gastric carcinoma: wishful thinking or reality?
SO ZENTRALBLATT FUR CHIRURGIE
VL 125
IS 4

BP 333
EP 340
PD 2000
PY 2000
AB By the time it is diagnosed, gastric carcinoma is usually already advanced and, as a result, has a poor prognosis. Surgery, with complete (RO) resection of the tumor, is the only chance of cure for this disease. However, in locally advanced gastric carcinoma this is only possible in approximately half of all cases. In order to help improve the prognosis of patients with advanced stage carcinomas, the concept of multimodal therapy is presently being evaluated. The results of studies of postoperative adjuvant therapy have been contradictory, with the result that no indication for such treatment outside of study protocols presently exists. Recently, preoperative application of chemotherapy, the so-called "neoadjuvant" therapy concept, has become increasingly important, since it has been demonstrated that, in individual cases, tumors thought to be primarily unresectable have been able to be completely resected after chemotherapy. Based on the available studies, one can assume that, in a subgroup of patients with not yet identified favorable tumor biologic characteristics, a true down staging of the tumor occurs. To what extent a preoperative "overstaging" may be a factor can only be estimated statistically, since the presently available methods for clinical estimation of tumor stage are never as accurate as the final histopathologic evaluation. Since the recently started, randomized multicenter study under the auspices of the EORTC compares surgery alone with a combination of surgery and preoperative chemotherapy in locally advanced gastric carcinoma, information will soon be available which will help clarify the effectiveness of this therapy concept.
TC 0
ZB 0
Z8 4
ZS 0
Z9 4
SN 0044-409X
UT WOS:000087044200006
PM 10829313
ER

PT J
AU Bosing, NM
Heise, JW
Roher, HD
TI The use of adjuvant and neoadjuvant therapies in gastric cancer patients today in Germany
SO ZENTRALBLATT FUR CHIRURGIE
VL 125
IS 4
BP 341
EP 347
PD 2000
PY 2000
AB Introduction: In view of disappointing results after surgery alone multimodal therapeutic regimes are used to improve long-term prognosis in locally advanced gastric carcinomas. In presence of many reports about encouraging results ("down staging", improved RO-resection rates) but simultaneously missing evidence of efficiency of neoadjuvant therapies in respect to long-term survival (large randomized multicenter trials do not exist until today) and the herewith related uncertainties, we started an inquiry among many surgical units with the intention to evaluate the clinical practice of multimodal treatment for gastric cancer patients in Germany today.
Methods: In a questionnaire (3/99) we asked among 97 surgical units (41 university hospitals, 56 big community hospitals) in Germany for the management of gastric cancer patients with special interest to practice and state of adjuvant and neoadjuvant therapeutic strategies. Further we analyzed all resected gastric cancer patients (1986-1995) without neoadjuvant treatment in advanced stage of disease (pT3/4NxMx; stage III/IV (UICC'92) in respect to RO-resection rate and long-term prognosis (Kaplan-Meier).
Results: Overall feedback amounted to 78% (76/97) and was higher in university hospitals (90%) than in big community hospitals (70%). Today, neoadjuvant therapies are of more interest than adjuvant therapeutic regimes. But also neoadjuvant therapy is only used in 32% as a rule tin 16% with, in 16% without study conditions). 25% of all surgical units do not employ any neoadjuvant therapy in locally advanced gastric cancer until today. In all other surgical units neoadjuvant treatment is performed more individually and sporadically (43%) only in some

patients. Neoadjuvant therapies are practiced by haematooncologists in 50%, gastroenterologists in 32% and surgeons in 27%. The predominant neoadjuvant therapeutic strategy is chemotherapy alone (84%). Many surgical units in Germany are interested to participate in a multicenter trial with more interest in neoadjuvant than adjuvant therapy. 185 of 309 resected gastric cancer patients (60%) were classified as stage III a, stage IIIb or stage IV patients. RO-resection rate of these advanced gastric cancer patients amounted to 37%; only 24% of them survived 5 years or more. Conclusions: Considering the missing evidence that multimodal therapies are able to prolong long-term survival in advanced gastric cancer patients, its use without study conditions is questionable.
Conclusions, taken from data of clinical trials regarding carcinomas of the esophagus and esophagealgastric junction, are inconsistent in respect to long-term prognosis and results are not transferable to gastric carcinomas. A prospective randomized multicenter trial in advanced gastric cancer patients is of great importance. Following our data, in Germany a high readiness to participate in the forthcoming EORTC-study is present.
TC 0
ZB 0
Z8 0
ZS 0
Z9 0
SN 0044-409X
UT WOS:000087044200007
PM 10829314
ER

PT J
AU Lerut, T
Coosemans, W
De Leyn, P
Van Raemdonck, D
Deneffe, G
Decker, G
TI Treatment of esophageal carcinoma
SO CHEST
VL 116

IS 6
BP 463S
EP 465S
DI 10.1378/chest.116.suppl_3.463S
SU S
PD DEC 1999
PY 1999
AB Cancel of the esophagus and gastroesophageal junction remains a virulent malignancy with an overall poor prognosis. Especially in the Western hemisphere, the incidence of adenocarcinoma is sharply rising. Over the last two decades, surgery has become the mainstay of treatment. Decreased surgical mortality and standardization of oncologic principles focusing on the completeness of resection are believed to be responsible for the improved 5-year survival rates, which are reaching greater than or equal to 30%. Until now, there has been no proven benefit from combined neoadjuvant treatment modalities using chemotherapy or chemoradiotherapy except for the subset of patients showing a complete response at pathologic examination. Further research should focus on new chemotherapeutic agents and the development of molecular markers that allow better identification of candidates for multimodality regimens.
CT Symposium on Multimodality Therapy of Chest Malignancies - Update 1998
CY MAR 12-14, 1998
CL CAMBRIDGE, MASSACHUSETTS
SP Harvard Med Sch, Dept Oncol; Dana Farber Canc Inst; Div Thorac Surg & Harvard Joint Ctr Radiat Therapy; Brigham & Womens Hosp
TC 21
ZB 8
Z8 1
ZS 0
Z9 22
SN 0012-3692
UT WOS:000084390800007
PM 10619509
ER

PT J
AU Ishikawa, Y
Kubota, T
Otani, Y

Watanabe, M
Teramoto, T
Kumai, K
Takechi, T
Okabe, H
Fukushima, M
Kitajima, M

TI Thymidylate synthetase and dihydropyrimidine dehydrogenase levels in gastric cancer

SO ANTICANCER RESEARCH

VL 19

IS 6C

BP 5635

EP 5640

PD NOV-DEC 1999

PY 1999

AB The measurement of thymidylate synthetase (TS) and dihydropyrimidine dehydrogenase (DPD) enzymatic activities and mRNA levels in tumors may be useful in predicting tumor sensitivity to 5-fluorouracil (5-FU). Ferry-one patients with advanced gastric cancer gave informed consent and were enrolled in this study. Biopsy specimens of gastric cancer were obtained preoperatively through gastrofiberscopy and used to determine TS and DPD mRNA levels. We also measured TS and DPD enzymatic activities and mRNA levels in surgically, resected gastric cancer samples, as well as in adjacent normal gastric mucosa. TS and DPD activities were measured using the TS-binding assay and a radioenzymatic assay, respectively, while mRNA levels were measured by semi-quantitative reverse transcription-PCR (RT-PCR) co-amplified with glyceraldehyde-3-phosphate dehydrogenase (GAPDH) as an internal standard. In resected tumor specimens, TS and DPD activities ranged from 7.1 to 176.6 fmol/mg protein and from 3.6 to 99.8 pmol/min/mg protein, respectively, while TS and DPD mRNA levels ranged from 0.50 to 21.12 and from 0.014 to 7:22, respectively. There were no significant correlations between TS/DPD levels and other clinicopathological factors, except for low DPD mRNA levels in undifferentiated carcinoma. Both TS activity and mRNA levels were significantly higher in tumor tissues compared to normal adjacent mucosa. In contrast, there was no significant difference between tumoral and non-tumoral DPD activity, although tumor tissue showed significantly lower DPD mRNA levels than non-tumoral tissue. High

tumoral TS mRNA levels in preoperative biopsy specimens from patients with stage III/IV was associated with poor survival outcome after surgery compared with patients with low tumoral TS mRNA levels. In contrast, DPD levels had no influence on prognosis. We conclude that high tumoral TS levels and low tumoral DPD mRNA may indicate the selective cytotoxicity of 5-FU on gastric cancer, and that tumoral TS mRNA levels may be a prognostic factor for patients with stage III/IV gastric cancer.
TC 39
ZB 29
Z8 1
ZS 0
Z9 40
SN 0250-7005
UT WOS:000085317400026
PM 10697632
ER

PT J
AU Waters, JS
Norman, A
Cunningham, D
Scarffe, JH
Webb, A
Harper, P
Joffe, JK
Mackean, M
Mansi, J
Leahy, M
Hill, A
Oates, J
Rao, S
Nicolson, M
Hickish, T
TI Long-term survival after epirubicin, cisplatin and fluorouracil for gastric cancer: results of a randomized trial
SO BRITISH JOURNAL OF CANCER
VL 80
IS 1-2

BP 269
EP 272
DI 10.1038/sj.bjc.6690350
PD APR 1999
PY 1999
AB We report the final results of a prospectively randomized study that compared the combination of epirubicin, cisplatin and protracted venous infusion fluorouracil (5-FU) (ECF regimen) with the standard combination of 5-FU, doxorubicin and methotrexate (FAMTX) in previously untreated patients with advanced oesophagogastric cancer. Between 1992 and 1995, 274 patients with adenocarcinoma or undifferentiated carcinoma were randomized from eight oncology centres in the UK and analysed for response and survival. The overall response rate was 46% (95% confidence interval (CI), 37-55%) with EGF, and 21% (95% CI, 13-28%) with FAMTX (P = 0.00003). The median survival was 8.7 months with ECF and 6.1 months with FAMTX (P = 0.0005). The 2-year survival rates were 14% (95% CI, 8-20%) for the ECF arm, and 5% (95% CI, 2-10%) for the FAMTX arm (P = 0.03). Histologically complete surgical resection following chemotherapy was achieved in ten patients in the ECF arm (three pathological complete responses to chemotherapy) and three patients in the FAMTX arm (no pathological complete responses). The ECF regimen resulted in a response and survival advantage compared with FAMTX chemotherapy. The probability of long-term survival following surgical resection of residual disease is increased by this treatment. The high response rates seen with ECF support its use in the neoadjuvant setting.
TC 187
ZB 101
Z8 8
ZS 1
Z9 197
SN 0007-0920
UT WOS:000080028700039
PM 10390007
ER

PT J
AU Lowy, AM
Mansfield, PF
Leach, SD

Pazdur, R
Dumas, P
Ajani, JA
TI Response to neoadjuvant chemotherapy best predicts survival after curative resection of gastric cancer
SO ANNALS OF SURGERY
VL 229
IS 3
BP 303
EP 308
DI 10.1097/00000658-199903000-00001
PD MAR 1999
PY 1999
AB Objective
In Western populations, long-term survival rates after curative resection of gastric cancer remain extremely poor. The lack of effective adjuvant therapy has prompted the evaluation or neoadjuvant approaches. Since 1988, we have conducted three separate phase II trials using neoadjuvant chemotherapy to treat patients with potentially resectable gastric cancer. The present study was conducted to evaluate whether response to neoadjuvant chemotherapy is predictive of survival in patients with resectable gastric cancer.
Methods
Eighty-three patients with pathologically confirmed gastric adenocarcinoma were treated with neoadjuvant chemotherapy before planned surgical resection. Response was assessed by upper gastrointestinal series, endoscopy, computed tomography scan, and pathologic examination.
Results
For the three phase II trials, clinical response rates ranged from 24% to 38%. Three patients (4%) had a complete pathologic response. Sixty-one patients (73%) underwent a curative resection, Median follow-up was 26 months, Univariate analysis revealed T stage, number of positive nodes, and response to chemotherapy to be significant predictors of overall survival, However, on multivariate analysis, response to chemotherapy was found to be the only independent prognostic factor.
Conclusions
Response to neoadjuvant chemotherapy is the single most important predictor of overall survival after neoadjuvant chemotherapy for gastric

cancer. These findings support further evaluation of neoadjuvant approaches in the treatment of this disease.
TC 141
ZB 46
Z8 7
ZS 1
Z9 146
SN 0003-4932
UT WOS:000079021600002
PM 10077040
ER

PT J
AU Torres, C
Turner, JR
Wang, HH
Richards, W
Sugarbaker, D
Shahsafaei, A
Odze, RD
TI Pathologic prognostic factors in Barrett's-associated adenocarcinoma - A follow-up study of 96 patients
SO CANCER
VL 85
IS 3
BP 520
EP 528
DI 10.1002/(SICI)1097-0142(19990201)85:3<520::AID-CNCR2>3.0.CO;2-L
PD FEB 1 1999
PY 1999
AB BACKGROUND. The objective of this study was to evaluate a variety of histologic features, some of which to our knowledge have never been evaluated in Barrett's-associated adenocarcinoma (BAd) (such as Crohn's-like lymphoid reaction and peritumoral lymphoid response) in patients with and without preoperative neoadjuvant chemotherapy combined with radiotherapy (chemrad) to determine their prognostic significance in these two groups of patients.
METHODS. Tumor sections from 96 patients (83 males and 13 females; mean age, 62 years) with resected BAd (61 with chemrad and 35 without

chemrad) were evaluated for numerous histologic features such as pathologic stage according to the American Joint Committee on Cancer TNM staging system, peritumoral lymphoid infiltrate, Crohn’s-like lymphoid reaction, and degree of post chemrad residual tumor and correlated with the preoperative chemrad status and with survival (mean follow-up, 23 months).
RESULTS. By univariate analysis, older patient age (P = 0.02), higher pathologic stage (P = 0.02) (including depth of invasion and lymph node status), infiltrative growth pattern (P = 0.05), perineural invasion (P = 0.05), vascular invasion (P = 0.04), and the absence of a peritumoral lymphoid infiltrate (P = 0.04) were associated with shortened survival in the entire cohort and in patients without chemrad, with the exception of infiltrative growth pattern (P = 0.1 in the nonchemrad group only). Higher stage was the only feature associated with decreased survival in the chemrad group, Subcategorization of lymph nodes according to the number involved with metastases (fewer than four, four to seven, and greater than seven) had no further effect on prognosis. However, subcategorization of T1 tumors into Tla and T1b did influence prognosis in a negative manner. Using multivariate analysis, only older patient age (P = 0.005) and the absence of a peritumoral lymphoid infiltrate (P = 0.05) were statistically associated with poor survival independent of stage. In addition, perineural invasion (P = 0.07) showed a trend toward shortened survival in patients with this feature. Preoperative chemrad had no effect on survival in this retrospective nonrandomized cohort of patients.
CONCLUSIONS. This study confirms the strong prognostic usefulness of the TNM staging system in patients with resected BAd, even in those patients who received preoperative chemrad. In addition, older patient age, the absence of a peritumoral lymphoid infiltrate, and possibly perineural invasion correlate with poor survival independent of pathologic stage in patients with these tumors. Cancer 1999;85: 520-8. (C) 1999 American Cancer Society.
CT 87th Annual Meeting of the United-States-and-Canadian-Academy-of-Pathology
CY FEB 28-MAR 06, 1998
CL BOSTON, MASSACHUSETTS
SP US & Canadian Acad Pathol
RI Turner, Jerrold/A-6895-2009
OI Turner, Jerrold/0000-0003-0627-9455

TC 30
ZB 17
Z8 0
ZS 0
Z9 30
SN 0008-543X
UT WOS:000078390100002
PM 10091725
ER

PT J
AU Zhang, LJ
Chen, KN
Xu, GW
Xing, HP
Shi, XT
TI Congenital expression of mdr-1 gene in tissues of carcinoma and its relation with pathomorphology and prognosis
SO WORLD JOURNAL OF GASTROENTEROLOGY
VL 5
IS 1
BP 53
EP 56
PD FEB 1999
PY 1999
AB AIM To detect the congenital expression patterns of mdr-l gene in commonly encountered malignant tumors in clinic, and the relationship between the expression of mdr-l gene and the prognostic morphology in esophageal carcinomas.
METHODS A total of 151 resected samples of malignant tumors without preoperative treatment were taken from Anyang City Tumor Hospital. The congenital expression of their mdr-1 gene was detected with reverse transcription polymerase chain reaction (RT-PCR) and was compared with each other. The positive incidence of mdr-l gene in 46 samples of esophageal carcinoma was compared with their differentiated grades, TNM stages and macroscopic types, and the precautions and advantages of RT-PCR were evaluated.
RESULTS All the 151 samples were confirmed to be malignant histopathologically, including cancers of stomach and gastric cardia (n

= 51), esophagus (n = 46), colorectum (n = 16), breast (n = 15), thyroid (n = 10), lung (n = 9), uterine cervix (n = 24). The positive expression rate of their mdr-l gene was 33.3%, 37%, 31.3%, 13.2%, 40%, 55%, and 0% respectively. All the 46 samples of esophageal carcinoma were pathologically confirmed to be squamous cell carcinoma. The total expression rate of their mdr-l gene was 37% (17/ 46), 35% (6/ 17), 40% (8/ 20), and 33% (3/ 9) for differentiation grade I, II and III respectively. The expression rate of TNM classification was 33% (6/ 18), 40% (5/ 12) and 37% (6/16) in stage II a, II b and III. The expression rate was 33% (3/ 9) in ulcerous type, 37% (3/ 8) in constrictive one, 33% (5/ 15) in fungoid one, and 40% (6/ 14) in medullary one. No statistically significant difference was found.
CONCLUSION Compared with other methods, RT-PCR is more simple, reliable and accurate in detecting mdr-l gene expression in tissues of tumor. The overexpression of mdr-l gene in these neoplasms suggested that cases should be handled differently for chemotherapy with rational use of drugs. Excision is the chief treatment for carcinoma of esophagus. The expression of mdr-l gene in tissues of esophageal cancer is correlated with the parameters of tumor molecular biology which are independent of histopathological morphology.
TC 25
ZB 11
Z8 0
ZS 0
Z9 25
SN 1007-9327
UT WOS:000078745000018
ER

PT J
AU Keller, SM
Ryan, LM
Coia, LR
Dang, P
Vaught, DJ
Diggs, C
Weiner, LM
Benson, AB
TI High dose chemoradiotherapy followed by esophagectomy for adenocarcinoma

of the esophagus and gastroesophageal junction - Results of a phase II study of the Eastern Cooperative Oncology Group

SO CANCER
VL 83
IS 9
BP 1908
EP 1916
DI 10.1002/(SICI)1097-0142(19981101)83:9<1908::AID-CNCR5>3.0.CO;2-6
PD NOV 1 1998
PY 1998

AB BACKGROUND, To assess the toxicity, local response, and survival associated with multimodality therapy in a cooperative group setting, patients with biopsy-proven clinical Stage I or II adenocarcinoma of the esophagus (staged according to 1983 American Joint Committee on Cancer criteria) or gastroesophageal junction were treated with concomitant radiation and chemotherapy followed by esophagectomy.

METHODS. Radiotherapy was administered in daily 2-gray (Gy) fractions 5 days a week until a total of 60 Gy was reached. 5-fluorouracil (5-FU) was infused continuously at a dose of 1000 mg/m(2)/day for 96 hours on Days 2-5 and 28-31. On Day 2, a 10 mg/m(2) bolus of mitomycin was injected intravenously. Esophagectomy was performed 4-8 weeks following completion of the radiotherapy.

RESULTS. During the 18-month study period (August 1991 through January 1993), 46 eligible patients were accrued from 21 institutions. Eight patients were Stage I and 38 Stage II. Eighty-seven percent of patients (40 of 46) received 6000 centigray (cGy), and all received >5000 cCy. Seventy-eight percent of patients (36 of 46) received >90% of the planned 5-FU dose. Follow-up ranged from 11 to 36 months (median, 22 months). There were eight treatment-related deaths; two were preoperative (from adult respiratory distress syndrome) and six were postoperative. Complete or partial response prior to esophagectomy was observed in 63% of cases, stable disease in 15%, and progression in 20%. Thirty-three patients underwent esophagectomy (transhiatal, n = 14; Ivor Lewis, n = 16; other, n = 3). No tumor was found in the specimens resected from 8 of these 33 patients; this represented a pathologic complete response rate of 17% overall and 24% for those who underwent esophagectomy. Overall median survival was 16.6 months, 1-year survival 57%, and 2-year survival 27%. Survival was significantly worse for patients with circumferential cancers (median, 18.1 months vs, 8.3

months; P < 0.05),
CONCLUSIONS. High dose radiation therapy with concurrent 5-FU and mitomycin may be administered to patients with esophageal adenocarcinoma with acceptable morbidity. However, in a cooperative group setting, esophagogastrectomy following intensive chemoradiotherapy is associated with excessive morbidity and mortality. Circumferential tumor growth is a significant adverse prognostic factor. Cancer 1998;83:1908-16. (C) 1998 American Cancer Society.
CT 31st Annual Meeting of the American-Society-of-Clinical-Oncology
CY MAY 20-23, 1995
CL LOS ANGELES, CALIFORNIA
SP Amer Soc Clin Oncol
TC 53
ZB 12
Z8 1
ZS 0
Z9 55
SN 0008-543X
UT WOS:000076697000005
PM 9806648
ER

PT J
AU Wobst, A
Audisio, RA
Colleoni, M
Geraghty, JG
TI Oesophageal cancer treatment: Studies, strategies and facts
SO ANNALS OF ONCOLOGY
VL 9
IS 9
BP 951
EP 962
DI 10.1023/A:1008273110272
PD SEP 1998
PY 1998
AB Esophageal cancer is among the ten most frequent cancers in the world. Once diagnosis is established prognosis is poor with five-year survival rates below 10%. Over the last few years, the evidence - base for

treatment of oesophageal cancer has changed with the publication of several important articles in this field. This article reviews these and other relevant publications with focus on current evidence which holds potential for an improvement in survival in oesophageal cancer patients. Prevention and early detection represent the mainstay in the ongoing struggle to improve prognosis, which is most stringently linked to tumor stage. Other efforts have been dedicated to optimise surgical treatment, radiotherapy and chemotherapy and to discover the most efficient combinations of these treatment modalities. Strong but not unanimous evidence in favour of a multimodality approach with chemoradiotherapy followed by surgery has accumulated in recent years, and confirmatory trials are presently ongoing. A pathological complete response to chemoradiotherapy has been identified to significantly enhance survival. Among the strategies to achieve higher response rates, variations in the administration of the most commonly used drugs rather than higher drug and radiation dosages seem promising. Occult lymphatic spread has been recognised as a major source of recurrence and has been successfully targeted by three field surgical dissection and extended field radiotherapy.
In search of the optimal treatment for patients with oesophageal cancer, a variety of different tracks are being pursued. This review outlines and analyses current treatment approaches and investigates how recent advances may impact on patient management.
TC 48
ZB 29
Z8 1
ZS 0
Z9 49
SN 0923-7534
UT WOS:000076838200011
PM 9818067
ER

PT J
AU Cascinu, S
Labianca, R
Graziano, F
Pancera, G
Barni, S

Frontini, L
Luporini, G
Cellerino, R
Catalano, G
TI Intensive weekly chemotherapy for locally advanced gastric cancer using 5-fluorouracil, cisplatin, epidoxorubicin, 6S-leucovorin, glutathione and filgrastim: a report from the Italian Group for the Study of Digestive Tract Cancer (GISCAD)
SO BRITISH JOURNAL OF CANCER
VL 78
IS 3
BP 390
EP 393
DI 10.1038/bjc.1998.505
PD AUG 1998
PY 1998
AB Local extension prevents curative resection in more than two-thirds of gastric cancer patients. Unfortunately, resectability is one of the main prognostic factors in these patients, and survival is longer when tumours are completely removed. Preoperative chemotherapy is an attractive concept for obtaining curative resection. Thirty-two locally advanced unresectable gastric cancer patients were enrolled in five Italian Group for the Study of Digestive Tract Cancer (GISCAD) centres. For 16 patients, surgical unresectability was based on computerized tomography scan evaluation of tumour size (four patients) and invasion of adjacent structures (12 patients), whereas in another 16 patients locally advanced disease was confirmed by laparotomy. They received weekly administration of cisplatin 40 mg m(-2), 5-fluorouracil 500 mg m(-2), epidoxorubicin 35 mg m(-2), 6S-stereoisomer of leucovorin 250 mg m(-2) and glutathione 1.5 g m(-2). From the day after to the day before each chemotherapy administration, filgrastim was administered by subcutaneous injection at a dose of 5 mu g kg(-1). One cycle of therapy consisted of eight weekly treatments. Fifteen of 32 patients (47%) responded to chemotherapy, whereas 13 (41%) had stable disease and four (12%) progressed on therapy. Of the 15 responding patients, 13 were completely resected after chemotherapy and two of them had a complete pathological response. Two clinically responding patients were found unresectable at operation because of peritoneal seeding. At a median follow-up from the start of treatment of 24 months (range 11-39 months),

10 of 13 resected patients are alive and eight are relapse free. Three patients died after 11, 12, and 14 months respectively. Toxicity was acceptable: side-effects consisted mainly of grade II National Cancer Institute common toxicity criteria (NCICTC) leucopenia and thrombocytopenia in ten patients. Neither treatment-related death nor surgical complications in patients undergoing surgery were observed. This weekly intensive regimen enabled resection in half of previously inoperable tumours with a moderate toxicity. It can be offered to patients with locally advanced unresectable gastric cancer to obtain curative resection.
TC 32
ZB 22
Z8 0
ZS 0
Z9 32
SN 0007-0920
UT WOS:000075150400021
PM 9703289
ER

PT J
AU Posner, MC
Gooding, WE
Landreneau, RJ
Rosenstein, MM
Clarke, MR
Peterson, MS
Lembersky, BC
TI Preoperative chemoradiotherapy for carcinoma of the esophagus and gastroesophageal junction
SO CANCER JOURNAL FROM SCIENTIFIC AMERICAN
VL 4
IS 4
BP 237
EP 246
PD JUL-AUG 1998
PY 1998
AB PURPOSE
To determine whether combination 5-fluorouracil, cisplatin, and

interferon alfa, an active regimen in advanced esophageal cancer, is efficacious as induction therapy before esophagectomy.

MATERIALS AND METHODS

Forty-four patients with potentially resectable esophageal/gastroesophageal junction adenocarcinoma or squamous cell carcinoma were entered into a phase I/II study of this chemotherapeutic regimen and concurrent external-beam radiotherapy before resection. The initial 16 patients were treated with prolonged-infusion 5-fluorouracil (300 mg/m(2) on days 1 to 28), cisplatin (20 mg/m(2) on days 1 to 5 and 24 to 28), interferon alfa (3 x 10(6) U/m(2) intravenously on days 1 to 5 and 24 to 28; subcutaneous injection every other day on days 6 to 23), and radiation (4000 cGy). The subsequent 28 patients were treated over 21 days with two modifications: dose escalation of 5-fluorouracil (250 to 350 mg/m(2)) and double-fractionated radiotherapy to a total dose of 4500 cGy.

RESULTS

Forty-one patients completed chemoradiotherapy and were evaluable for toxicity. Adverse events were substantial but tolerable, and most toxic episodes were hematologic and gastrointestinal. Three patients died, and one patient had progressive disease before resection. Of the 37 patients eligible for Native resection, 36 had all gross tumor removed. Thirty-three (80%) patients had a major pathologic response: 10 (24%) with no residual tumor and 23 with only microscopic residual tumor. Median survival for all patients was 27 months and for responders was 36 months.

CONCLUSIONS

This combination regimen is active but yields results similar to those of other chemoradiotherapy phase II trials; therefore, the contribution of interferon alfa to treatment efficacy remains uncertain. The true worth of preoperative chemoradiotherapy is unknown pending results of phase III trials.

CT 32nd Annual Meeting of the American-Society-of-Clinical-Oncology
CY MAY 18-21, 1996
CL PHILADELPHIA, PENNSYLVANIA
SP Amer Soc Clin Oncol
TC 23
ZB 6
Z8 0
ZS 0

Z9 23
SN 1081-4442
UT WOS:000075038300006
PM 9689982
ER

PT J
AU Ng, CS
Husband, JES
Macvicar, ADL
Ross, P
Cunningham, DC
TI Correlation of CT with histopathological findings in patients with gastric and gastro-oesophageal carcinomas following neoadjuvant chemotherapy
SO CLINICAL RADIOLOGY
VL 53
IS 6
BP 422
EP 427
DI 10.1016/S0009-9260(98)80270-5
PD JUN 1998
PY 1998
AB Gastric carcinoma is the fourth commonest cause of death from malignant disease in United Kingdom. In the Western hemisphere, it usually presents with advanced disease, which contributes to its very poor prognosis, Pre-operative (neoadjuvant) chemotherapy offers the possibility of down-staging such tumours and the potential to render tumours operable. Computed tomography (CT) plays a central role in the assessment of patients presenting with the disease, and in those who undergo chemotherapy, in evaluating their response.
Objective: This study was undertaken to evaluate the role of CT in predicting locoregional spread of tumour following neoadjuvant chemotherapy in non-metastatic gastric and gastro-oesophageal cancers.
Methods and Materials: We correlated CT evidence of loco-regional spread with pathological findings following surgery in 21 patients who received pre-operative chemotherapy,
Results: Residual masses were seen on CT in 19 patients, and 15 contained active tumour, although in four patients no viable tumour was

demonstrated at histopathology, The overall accuracy of CT in assessing loco-regional disease was disappointing with sensitivities, specificities, positive and negative predictive values of 57%, 43%, 75% and 33%, respectively.
Conclusions: We conclude that CT is not accurate in identifying residual loco-regional spread and therefore should not preclude surgery in those patients who have received neoadjuvant chemotherapy.

TC 21

ZB 7

Z8 0

ZS 0

Z9 21

SN 0009-9260

UT WOS:000074533800005

PM 9651057

ER

PT J

AU Nio, Y
Sato, Y
Nagami, H
Teramoto, M
Inoue, Y
Yano, S
Sumi, S
Tamura, K
Fukumoto, M

TI Neoadjuvant chemotherapy of gastric cancer with oral UFT (a mixture of uracil and fturafur) during the waiting period for surgery

SO ANTICANCER RESEARCH

VL 18

IS 1B

BP 523

EP 530

PD JAN-FEB 1998

PY 1998

AB Our previous experience has demonstrated that growth of gastric cancer during the waiting period for surgely cannot be neglected, and some patients hope to receive prophylactic treatment to inhibit the growth of

tumor until surgery. The present study was designed to assess the clinical benefits of preoperative chemotherapy with Oral UFT for gastric cancer during the waiting period for surgery Fifty patients with gastric cancer (24 early, 25 advanced and 1 recurrent cancers) were treated with oral UFT at 300 similar to 600 mg/day for 7 similar to 36 days before surgery and the objective responses and the postsurgical survival were evaluated. In 42 of 50 patients objective responses of primary lesions were assessed by endoscopy or upper gastrointestinal series examination, and 2 CRs; 15 PRs and 25 NCs were seen (40% response). The histological effect was evaluated in 50 patients and the following classifications were made: grade 3 (complete disappearance or necrosis of tumor cells), 2;grade 2 (necrotic changes > 2/3 area), 4; grade 1b (>1/3 area), 7; grade 1a (<1/3 area), 15; and grade 0 (no histological changes), 22. A longer period of UFT administration was associated with CR or PR. All the patients undenuent gastrectomy (38 curative and 12 palliative gastrectomies): all patients with Stage I similar to III primaly gastric cancer ape alive after surgery, and the 50% survival period of the patients with Stage IV cancer was 20 months. The side effects were not serious including slight myelotoxicity, liver dysfunction and anorexia. it is concluded that preoperative chemotherapy for gastric cancer with oral UFT on outpatient basis may result in down-staging as well as the prevention of tumor growth during the waiting period for surgery without serious side effects.
TC 5
ZB 5
Z8 0
ZS 0
Z9 5
SN 0250-7005
UT WOS:000072955700009
PM 9568172
ER

PT J
AU Ajani, JA
TI Current status of new drugs and multidisciplinary approaches in patients with carcinoma of the esophagus
SO CHEST
VL 113

IS 1
BP 112S
EP 119S
DI 10.1378/chest.113.1_Supplement.112S
SU S
PD JAN 1998
PY 1998
AB The incidence of distal esophageal adenocarcinoma and primary proximal gastric carcinoma has increased substantially in the past 15 years, particularly in North America and in some European countries. Patients with curatively resected cancer consistently have a 10 to 20% 5-year survival rate. Radiation therapy alone should not be recommended. Based on the Radiation Therapy Oncology Group/Eastern Cooperative Oncology Group (ECOG) trial in patients with predominantly squamous cell carcinoma, chemoradiotherapy (fluorouracil [5-FU]/cisplatin + 50 Gy of radiotherapy) has been shown to be superior in this setting. The most active single agents against squamous cell carcinoma are cisplatin, 5-FU, bleomycin, paclitaxel, mitomycin, mitoguazone, vinorelbine, and methotrexate. The most active agents against adenocarcinoma include paclitaxel and probably mitomycin, mitoguazone, and cisplatin. To my knowledge, there is currently no effective postoperative adjuvant therapy (chemotherapy, radiation therapy, or both). Evidence that preoperative therapy can prolong survival of patients with potentially resectable carcinoma of the esophagus is lacking. Preoperative chemoradiotherapy can result in an approximately 25% complete pathologic response of the primary tumor. Preoperative chemoradiotherapy, however, results in substantial morbidity and even mortality. A recent single-institution, randomized study comparing surgery alone with preoperative 5-FU/cisplatin/vinblastine and concurrent radiotherapy demonstrated no difference in median survival (18 months). Nevertheless, combined-modality therapy holds have been formulated and will be investigated in the next few years.
CT International Symposium on Thoracic Malignancies
CY APR 25-26, 1996
CL PITTSBURGH, PENNSYLVANIA
SP Univ Pittsburgh, Sch Med, Ctr Continuing Educ Hlth Sci; Univ Pittsburgh, Canc Inst
TC 14
ZB 6

Z8 0
ZS 0
Z9 14
SN 0012-3692
UT WOS:000071587100020
PM 9438700
ER

PT J
AU Metzger, R
Leichman, CG
Danenberg, KD
Danenberg, PV
Lenz, HJ
Hayashi, K
Groshen, S
Salonga, D
Cohen, H
Laine, L
Crookes, P
Silberman, H
Baranda, J
Konda, K
Leichman, L
TI ERCC1 mRNA levels complement thymidylate synthase mRNA levels in
predicting response and survival for gastric cancer patients receiving
combination cisplatin and fluorouracil chemotherapy
SO JOURNAL OF CLINICAL ONCOLOGY
VL 16
IS 1
BP 309
EP 316
PD JAN 1998
PY 1998
AB Purpose: We have previously shown that relative thymidylate synthase
(TS) mRNA levels in primary gastric adenocarcinomas treated with
fluorouracil (5-FU) and cisplatin are inversely associated with response
and survival. This is a presumed function of TS as a target for 5-FU
activity, We now test the hypotheses that the relative mRNA level of the

excision repair crosscomplementing (ERCC1) gene is inversely associated with response and survival as an independent function of cisplatin efficacy.
Patients and Methods: Patients had intact, untreated, primary gastric adenocarcinoma cancer and were evaluated for eligibility on a preoperative cisplatin infusion-5-FU protocol. cDNA, derived from primary gastric tumors before chemotherapy, was used to determine ERCC1 mRNA levels, expressed as the ratio of polymerase chain reaction (PCR) product of the ERCC1 gene and the beta-actin gene.
Results: The median ERCC1 mRNA level from 38 primary gastric cancers (33 assessable for response) was 5.8 x 10(-3) (range, 1.8 x 10(-3) to 19.5 x 10(-3)). Of 17 responding patients, 13 (76%) were less than or equal to 5.8 x 10(-3) and four were greater than 5.8 x 10(-3) (P = .003). The median survival for patients with ERCC1 mRNA levels less than or equal to 5.8 x 10(-3) has not been reached, whereas for those greater than 5.8 x 10(-3) it was 5.4 months (P = .034). The median TS mRNA level, 3.7 x 10(-3) (range, 0.9 to 18.9) also segregated responsive versus resistant tumors (P = .024). With both ERCC1 and TS mRNA levels below their medians, 11 of 13 patients (85%) responded; with both ERCC1 and TS mRNA levels above their medians, two of 10 patients (20%) responded (P = .003).
Conclusion: Considered separately, either ERCC1 or TS mRNA levels in a primary gastric adenocarcinoma has a statistically significant relationship to response. ERCC I mRNA levels have a statistically significant association with survival; in this cohort TS mRNA levels did not reach statistically significant association with survival as in our previous publication. Whether these molecular parameters are independent of each other as predictors of outcome remains to be determined. (C) 1998 by American Society of Clinical Oncology.
TC 352
ZB 215
Z8 14
ZS 1
Z9 375
SN 0732-183X
UT WOS:000071368500045
PM 9440758
ER

PT J
AU Ross, P J
Rao, S
Cunningham, D
TI Chemotherapy of oesophago-gastric cancer.
SO Pathology oncology research : POR
VL 4
IS 2
BP 87
EP 95
PD 1998
PY 1998
AB Oesophageal and gastric cancers are common tumors that represent a number of challenges for oncologists, gastroenterologists and surgeons. The prognosis remains poor with the majority of patients presenting with advanced disease. Combined chemotherapy and radiotherapy has demonstrated a survival benefit in patients with loco-regional oesophageal cancer compared to radiotherapy alone. In an interim analysis we have observed a 62% response rate using a chemoradiation regimen based on protracted venous infusion of 5-fluorouracil and cisplatin combined with radiotherapy in patients with inoperable oesophageal cancer. Improved outcomes with loco-regional disease has rekindled interest in preoperative therapy. In a trial comparing preoperative chemoradiation to surgery alone in patients with operable oesophageal adenocarcinoma, survival was improved with multimodality treatment. In addition, a study including both adeno- and squamous carcinomas demonstrated a trend towards improved survival. A complete pathological response to chemoradiation was associated with significantly improved survival. Gastric cancer is one of the most chemosensitive solid tumors of the gastrointestinal tract with the majority of patients being suitable for palliative chemotherapy. The ECF (epirubicin, cisplatin, protracted venous infusion 5-fluorouracil) regimen was developed in the Gastrointestinal unit of the Royal Marsden Hospital and first reported in 1991. In a prospective randomised trial including 274 patients ECF has been compared with the standard combination of 5-fluorouracil, adriamycin and methotrexate (FAMTX) in patients with previously untreated gastric cancer. Overall response rate, failure-free and overall survival were significantly improved with ECE, ECF also demonstrated improved quality of life and cost

effectiveness when compared to the FAMTX regimen. ECF should now be regarded as the standard treatment for advanced oesophago-gastric cancer against which new therapies should be compared. In addition the Medical Research Council are conducting a trial randomising patients between surgery alone and perioperative chemotherapy using the ECF regimen in operable gastric cancer.
TC 6
ZB 2
Z8 0
ZS 0
Z9 6
SN 1219-4956
UT MEDLINE:9654592
PM 9654592
ER

PT J
AU Adelstein, DJ
Rice, TW
Becker, M
Larto, MA
Kirby, TJ
Koka, A
Tefft, M
Zuccaro, G
TI Use of concurrent chemotherapy, accelerated fractionation radiation, and surgery for patients with esophageal carcinoma
SO CANCER
VL 80
IS 6
BP 1011
EP 1020
PD SEP 15 1997
PY 1997
AB BACKGROUND. The results of a Phase II study of concurrent chemotherapy and accelerated fractionation radiation therapy followed by surgical resection for patients with both adenocarcinoma and squamous cell carcinoma of the esophagus are presented. Pretreatment and postinduction staging were correlated with pathologic findings at surgery to assess

the role of surgical resection and the predictive value of noninvasive staging techniques.
METHODS. Patients received 2 induction courses with 4-day continuous intravenous infusions of cisplatin (20 mg/m(2)/day) and 5-fluorouracil (1000 mg/m(2)/day) beginning on Day 1 and Day 21, concurrent with a split course of accelerated fractionation radiation (1.5 grays [Gy] twice daily, to a total dose of 45 Gy). All patients were subsequently referred for surgical resection. A single, identical postoperative course of chemotherapy and 24 GS accelerated fractionation radiation was planned for patients with residual tumor at surgery.
RESULTS. Seventy-four patients were entered on this study; 72 patients were considered eligible and evaluable. Induction toxicity included nausea (85%), increased dysphagia (90%), neutropenia (<1000/mm(3)) (43%), thrombocytopenia (<20,000/mm(3)) (10%), and reversible nephrotoxicity (8%). Sixty-seven patients (93%) underwent surgery, and 65 (90%) were found to have resectable tumors. Twelve of these patients (18%) died perioperatively, and 18 (27%) had no residual pathologic evidence of disease. Resolution of symptoms and normalization of radiographic studies, endoscopy, or esophageal ultrasound did not identify pathologic complete responders accurately. No patient completing induction therapy and surgery experienced a locoregional recurrence. The Kaplan-Meier 4-year projected recurrence free and overall survival rates were 49% and 44%, respectively.
CONCLUSIONS, Although this regimen is feasible, there was significant preoperative toxicity and perioperative mortality. Nonetheless, the recurrence free and overall survival rates were encouraging. However, no staging tool can predict a pathologic complete response after induction therapy accurately, suggesting a continued need for surgical resection.
(C) 1997 American Cancer Society.
CT 32nd Annual Meeting of the American-Society-of-Clinical-Oncology
CY MAY 18-21, 1996
CL PHILADELPHIA, PA
SP Amer Soc Clin Oncol
TC 89
ZB 38
Z8 3
ZS 0
Z9 92
SN 0008-543X

UT WOS:A1997XU94200002
PM 9305700
ER

PT J
AU VanRaemdonck, D
VanCutsem, E
Menten, J
Ectors, N
Coosemans, W
DeLeyn, P
Lerut, T
TI Induction therapy for clinical T4 oesophageal carcinoma; A plea for continued surgical exploration
SO EUROPEAN JOURNAL OF CARDIO-THORACIC SURGERY
VL 11
IS 5
BP 828
EP 837
DI 10.1016/S1010-7940(97)01194-9
PD MAY 1997
PY 1997
AB Objective: Complete resection of a locally advanced oesophageal carcinoma is not always feasible when invading mediastinal structures. The use of induction therapy prior to surgical exploration in patients with these clinical T4 tumours is anticipated to improve the resectability rate. Methods: Patients, 18, who presented with a carcinoma of the thoracic oesophagus with clinical invasion into the carina (n = 6), trachea (n = 5), aorta (n = 4), lung (n = 2) and diaphragm (n = 1) were treated with concurrent chemotherapy and radiotherapy followed by surgical exploration. Follow-up was complete (mean of 17 +/- 3 months in all patients and 27 +/- 2 months in surviving patients). Results: All patients completed the induction therapy with acceptable toxicity and no mortality. Subjective improvement in dysphagia was substantial in II patients (in 8/11 patients (73%) however, there was still viable tumour in the resected specimen), it was minimal in six patients and absent in one patient. Objective response on imaging; was complete in one patient, partial in eight patients and minimal in nine patients [in two of these nine

patients (22%) nevertheless, the primary tumour had disappeared completely in the resected specimen (pT0)]. Resection was complete (R0) in 14 patients (78%) and incomplete (R1) in one patient (5%). Resection of the primary tumour was impossible (R2) in three patients (17%) because of macroscopic airway (n = 2) and hilar (n = 1) invasion on exploration. In these three patients the tumour was bypassed using a retrosternal split stomach. One patient was proven at the time of surgery to have a previously unidentified lung metastasis. In three patients (17%), no residual tumour cells were found in the resected oesophagus nor in the lymph nodes (pT0N0M0). There have been no in-hospital deaths. Actuarial 3 year survival was 43% in all patients, 55% in completely resected patients and 100% in sterilized patients (pT0N0M0). Median survival was 18 months in all patients. Conclusions: Chemo/radiotherapy followed by surgery in patients with a clinical T4 oesophageal carcinoma is feasible with acceptable toxicity and no treatment-related mortality. Operability and resectability rate were high (100 and 83%, respectively) compared with historical controls. The primary tumour disappeared completely (pT0N0-1M0-1) in 28%. Tumour sterilization rate was 17%. Survival looks promising compared-with historical controls. Subjective neither objective response following induction therapy clearly correlated with the final pTNM staging. This indicates that, in the absence of tumour progression, neither the patient nor the treating physician should jeopardize the chance for ultimate cure by denying surgical exploration following induction therapy. (C) 1997 Elsevier Science B.V.
CT 9th Annual Meeting of the European-Association-for-Cardio-Thoracic-Surgery
CY SEP 24-27, 1995
CL PARIS, FRANCE
SP European Assoc Cardio Thorac Surg
TC 22
ZB 5
Z8 0
ZS 0
Z9 22
SN 1010-7940
UT WOS:A1997XE01800007
PM 9196296
ER

PT J

AU Thomas, P
Doddoli, C
Giacoia, A
Garbe, L
Perrier, H
Giovannini, M
Seitz, JF
HannounLevi, JM
Giudicelli, R
Fuentes, P

TI Induction therapy for focally advanced oesophageal cancer - Prognostic significance of the histopathological response

SO ANNALES DE CHIRURGIE

VL 51

IS 3

BP 222

EP 231

PD 1997

PY 1997

AB Objective: The purpose of this study was to determine the prognostic significance of the histopathological response to preoperative radio-chemotherapy in patients with locally advanced oesophageal cancer. Methods: Among the 57 patients included in this open prospective study, the disease-free survival of 48 patients (8 females, 40 males; mean age: 56.6 years +/- 8.4) who underwent an oesophagectomy after induction therapy for oesophageal squamous cell (n = 38) or adenocarcinoma (n = 10) was correlated with the histopathological findings. Chemoradiation included 2 cycles associating continuous 5 FU from D1 to 5 and from D22 to 26, cisplatyl on D1 and D22, 15 Gy/5d from D1 to 5 and from D22 to 26. Histopathological response was assessed on the operative specimens by routine examination of serial thin sections each 5 mm along the full oesophageal length, the resection margins and the lymph node dissection. Results: A wide interindividual variability was seen regarding tissue changes related to induction therapy, with a grading in tumor regression and the possibility of dissociated effects on the various treatment targets: tumor, adenopathy and vessel invasion. The 5-year probability of disease-free survival was 22% for the 48 resected patients. The

presence of a complete histopathological response (n = 12) did not preclude metastatic spread in half the: cases. Furthermore, it did not result in improved survival when compared to that of nonresponder patients. Survival of patients who had a complete or major oesophageal response (n = 29, 35% at 5 years) was significantly lower than that of patients who were operated on during the same period for a superficial oesophageal cancer at presentation (n = 29, 57% at 5 years; P = 0.03). After multivariate analysis according to the Cox model, downstaging of the primary tumor was not identified as an independent predictor of disease-free survival. Conclusions: Pathologic assessment of tumor regression on the operative specimen provides little prognostic information.

RI Thomas, Pascal/E-5036-2010; Thomas, Pascal/G-7457-2011

TC 5

ZB 2

Z8 0

ZS 0

Z9 5

SN 0003-3944

UT WOS:A1997WU60300004

PM 9297883

ER

PT J

AU Berdel, WE
Heldmann, T
Germer, C
Wiedenmann, B
Rosewicz, S
BoeseLandgraf, J
Karavias, T
Kreuser, ED
Buhr, HJ
Thiel, E

TI Phase II pilot trial of preoperative high-dose chemotherapy in patients with malignant tumors of the upper gastrointestinal tract

SO INTERNATIONAL JOURNAL OF ONCOLOGY

VL 9

IS 4

BP 613
EP 617
PD OCT 1996
PY 1996
AB The purpose of this trial was to test feasibility and tolerability of a multimodality treatment approach for patients with tumors in the upper gastrointestinal tract (EC, esophageal cancer; JC, cancer of the gastro-esophageal junction; GC, gastric cancer) including preoperative chemotherapy with the EAP-protocol as induction and a consecutive high-dose-chemotherapy for responding patients. Sixteen patients with locally advanced tumors of the esophagus, the gastro-esophageal junction or the stomach were treated with two cycles of EAP-chemotherapy (etoposide, 3x120 mg/m(2); adriamycin, 2x20 mg/m(2); cisplatin, 2x40 mg/m(2)). Responding (cPR, cCR) patients were included into a high-dose MCVB-chemotherapy protocol (mitomycin, 10 mg/m(2); cisplatin, 4x40 mg/m(2); vepeside, 5x200 mg/m(2); BCNU 300 mg/m(2)) and subsequent rescue with peripheral blood stem cells (PBSC). After a second restaging, surgery was performed in patients with no change or further response. Postoperative chemotherapy was given with either two cycles of EAP or FAMTX (methotrexate, 1,500 mg/m(2) + folinic acid rescue; 5-flourouracil, 1,500 mg/m(2); adriamycin, 30 mg/m(2)) according to pathological staging results. A total of 16 patients (EC, 7; JC, 6; GC 3) were treated within the protocol. Six patients achieved a major response upon EAP and 5/6 were included in the high-dose MCVB-protocol with stem cell rescue. All 5 could be yielded R(0) by definitive surgery and 2/5 had a pCR upon surgery. MCVB toxicity was predominantly hematologic (grade 4 in all 5 patients) with non-hematological toxicity not exceeding grade 2 (predominantly mucositis). Median survival time is 12 months for the non-responding patients and has not been reached for the MCVB patients. In conclusion, multimodality therapy including high-dose chemotherapy and stem cell rescue is feasible with tolerable toxicity in patients with locally advanced tumors of the upper gastrointestinal tract and should be further studied in phase II and III trials.
TC 4
ZB 3
Z8 0
ZS 0
Z9 4

SN 1019-6439
UT WOS:A1996VJ79800005
PM 21541559
ER

PT J
AU Fukushima, M
TI Adjuvant therapy of gastric cancer: The Japanese experience
SO SEMINARS IN ONCOLOGY
VL 23
IS 3
BP 369
EP 378
PD JUN 1996
PY 1996
TC 26
ZB 17
Z8 1
ZS 0
Z9 27
SN 0093-7754
UT WOS:A1996UQ28900015
PM 8658221
ER

PT J
AU Shibayama, T
   Hiyama, J
   Ueoka, H
   Tabata, M
   Segawa, Y
   Gemba, K
   Matsushita, A
   Ohnoshi, T
   Harada, M
   Andoh, A
TI [Induction chemotherapy followed by adjuvant surgery (IC-AS) in patients
   with stage I-II small cell lung cancer (SCLC)].
SO Gan to kagaku ryoho. Cancer & chemotherapy

VL 22
IS 13
BP 1953
EP 8
PD 1995-Nov
PY 1995
AB Ten patients with stage I-II SCLC received IC-AS between 1984 and 1993. As induction chemotherapy, COMP-VAN alternating chemotherapy and CAV-PVP hybrid chemotherapy were administered. The former consisted of a 4-drug combination of cyclophosphamide (CPA), vincristine (VCR), methotrexate (MTX) and procarbazine alternated with a 3-drug combination of etoposide (ETP), adriamycin (ADM) and nimustine every 4 weeks. In the latter, a 3-drug combination of CPA, ADM and VCR given on day 1, and a 2-drug combination of ETP and cisplatin on day 8, were repeated every 4 weeks. All the patients had an objective response, including one complete response by induction chemotherapy. Post-operative pathology revealed SCLC in 4 patients, adenocarcinoma in 2 and no tumor (pathological CR) in 4. Four patients relapsed, and a intrathoracic relapse was experienced in only 2 patients. Six patients have died: 3 from relapsing SCLC, 2 from stomach cancer, and 1 from squamous lung cancer, who was salvaged from relapsing SCLC. The median survival time was 27.5 months, and the 3-year survival rate 37.5%. These results indicate that IC-AS is highly effective for stage I-II SCLC and warrant additional studies comparing IC-AS with chemo-radiotherapy.
TC 0
ZB 0
Z8 0
ZS 0
Z9 0
SN 0385-0684
UT MEDLINE:7487126
PM 7487126
ER

PT J
AU ZINZANI, PL
FREZZA, G
BENDANDI, M
BARBIERI, E

GHERLINZONI, F
NERI, S
BALDISSERA, A
SALVUCCI, M
BABINI, L
TURA, S
TI PRIMARY GASTRIC LYMPHOMA - A CLINICAL AND THERAPEUTIC EVALUATION OF 82 PATIENTS
SO LEUKEMIA & LYMPHOMA
VL 19
IS 5-6
BP 461
EP 466
DI 10.3109/10428199509112205
PD NOV 1995
PY 1995
AB Eighty-two patients with primary gastric (I-E, IIIE, and II2E) non-Hodgkin's lymphoma according to the Musshoff's staging system were treated with combined modality including surgery with/without radiotherapy between January 1985 and December 1991. According to the Updated Kiel classification 54 had high-grade histologic subtypes and 28 low-grade. The strategy throughout the study was to resect primary tumor: all patients underwent gastrectomy, 40 subtotal and 42 total gastrectomy. The resection permitted complete surgical staging utilizing three pathologic features: disease confined within or beyond the serosa, negative/positive regional lymph nodes, and negative/positive surgical margins. If there was no evidence of these pathologic factors, the patients who underwent surgery alone received no further radiotherapy. On the other hand, all patients who presented at least one of three pathologic factors were treated with adjuvant radiotherapy after the resection. All except 14 patients presented at least one of the pathologic features and 50 (61%) patients had involvement of the whole gastric wall. Radiotherapy included the gastric bed and para-aortic lymph nodes and, for the patients, who had positive regional lymph nodes in combination with the complete involvement of the gastric wall, the irradiation included the whole abdominal approach. The complete response rate was 97% and the 9-year disease-free survival was 93%. All but one of the 5 relapses occurred within 18 months stressing the need for more specific staging. Gastric resection with/without radiotherapy may still

represent the primary therapeutic procedure in early stage gastric non-Hodgkin's lymphoma.
TC 14
ZB 10
Z8 0
ZS 0
Z9 14
SN 1042-8194
UT WOS:A1995TE70900012
PM 8590847
ER

PT J
AU NAKANO, H
NAMATAME, K
SUZUKI, T
KIM, J
SASAKI, J
NAGASAKI, H
MAKUUCHI, M
KUMADA, K
TI PROGNOSTIC EVALUATION OF CURATIVELY RESECTED LOCALLY ADVANCED GASTRIC-CANCER PATIENTS WITH PREOPERATIVE DOWNSTAGING CHEMOTHERAPY ASSESSED BY HISTOCHEMICAL AND PHARMACOLOGICAL MEANS
SO ONCOLOGY
VL 52
IS 6
BP 474
EP 482
PD NOV-DEC 1995
PY 1995
AB The aim of the present study was to investigate whether the rate of thymidylate synthetase inhibition (TSIR) and the rate of proliferating cell nuclear antigen expression (PCNA-R) in gastric cancer tissues, which can be obtained within a short period after surgery, were predictive and quantitative prognostic factors for locally advanced gastric cancer patients with preoperative down-staging chemotherapy. Curatively resected 30 locally advanced gastric cancer patients with preoperative chemotherapies were studied. Three-year survival analysis

showed that the higher TSIR and the lower PCNA-R significantly predicted better prognosis ($p < 0.01$ and $p < 0.05$, respectively). Multiple regression test showed that the TSIR was a significantly predictive variable for 1-year survival ($p < 0.05$). The TSIR and PCNA-R could be predictive and quantitative prognostic factors in advanced gastric cancer patients who received preoperative downstaging chemotherapy.

RI 幕内, 雅敏/A-2140-2012

TC 1

ZB 2

Z8 1

ZS 0

Z9 3

SN 0030-2414

UT WOS:A1995TF44200009

PM 7478434

ER

PT J

AU VOGEL, SB
MENDENHALL, WM
SOMBECK, MD
MARSH, R
WOODWARD, ER

TI DOWNSTAGING OF ESOPHAGEAL CANCER AFTER PREOPERATIVE RADIATION AND CHEMOTHERAPY

SO ANNALS OF SURGERY

VL 221

IS 6

BP 685

EP 695

DI 10.1097/00000658-199506000-00008

PD JUN 1995

PY 1995

AB Objective
This retrospective, nonrandomized review evaluates 125 patients with esophageal carcinoma (adenocarcinoma and squamous cell) who underwent either surgery only or preoperative chemotherapy and/or radiation therapy followed by surgery. Major end points were survival and postchemoradiation downstaging.

Methods
Forty-four patients underwent radiation therapy of 4500 cGy over 5 weeks. Fluorouracil and cisplatin were administered on the first and fifth week of radiotherapy. Ninety-eight patients underwent ''potentially curative'' resections-transhiatal esophagectomy (70), Lewis esophagogastrectomy (25), and left esophagogastrectomy (3). All patients with preoperative adjuvant therapy underwent endoscopy and biopsy before surgery.
Results
There were no differences in overall mortality (5%) or surgical complications in either group. Fourteen of 44 patients (32%) downstaged to complete pathologic response, with 5-year survival of 57%. Fifteen of 44 patients (34%) downstaged to microscopic residual tumor, with 1- and 3-year survival of 77% and 31%, respectively. Twenty-eight of 29 patients in the two downstaged groups were lymph node negative. Overall, 5-year survival in the adjuvant therapy plus surgery group versus surgery only was 36% and 11% ($p = 0.04$). Five-year survival in lymph node-negative adjuvant therapy and surgery patients was 49% ($p = 0.005$). Positive nodes in the surgery only group was 48% versus 23% in the adjuvant therapy and surgery group ($p = 0.02$).
Conclusion
Although retrospective and nonrandomized, these results suggest that preoperative chemoradiation results in significant clinical and pathologic downstaging, increases survival, and may sterilize local and regional lymph nodes, accounting for both downstaging and survival statistics.
CT 106th Annual Scientific Session of the Southern-Surgical-Association
CY DEC 04-07, 1994
CL PALM BEACH, FL
SP SO Surg Assoc
TC 91
ZB 36
Z8 0
ZS 0
Z9 91
SN 0003-4932
UT WOS:A1995RF53600008
PM 7794073
ER

PT J

AU FERGUSON, MK
REEDER, LB
HOFFMAN, PC
HARAF, DJ
DRINKARD, LC
VOKES, EE

TI INTENSIVE MULTIMODALITY THERAPY FOR CARCINOMA OF THE ESOPHAGUS AND GASTROESOPHAGEAL JUNCTION

SO ANNALS OF SURGICAL ONCOLOGY
VL 2
IS 2
BP 101
EP 106
DI 10.1007/BF02303623
PD MAR 1995
PY 1995

AB Background: We designed a trial of intensive multimodality therapy for carcinoma of the esophagus and gastroesophageal junction to assess tumor response and operability after neoadjuvant chemotherapy and to determine the impact of trimodality therapy on longterm survival.

Methods: Thirty-two patients with resectable (clinical stage IIa, n = 17; IIb, n = 1; III, n = 14) squamous cell cancer (n = 15) or adenocarcinoma(n = 17) were treated with neoadjuvant chemotherapy (cisplatin, 5-fluorouracil, leukovorin), resection, and postoperative chemoradiotherapy (hydroxyurea, 5-fluorouracil; 50-66 Gy).

Results: Use of neoadjuvant chemotherapy yielded the following results: a measurable clinical response in 22 patients, stable disease in eight patients, disease progression in one patient, and death in one patient. Thirty-one patients underwent resection, with the following results: two operative deaths (6.5%) and nonfatal morbidity in 17 (59%); the median hospital stay was 13 days. Pathologic staging was stage 0, n = 1; I, n = 2; IIa, n = 11; IIb, n = 5; III, n = 7; and IV, n = 5. Postoperative chemoradiotherapy was completed in 23 patients with one death, for an overall treatment-related mortality rate of 12.5% (four of 32). At a mean follow-up of 22.5 months, median survival is 19.7 months and 14 patients are alive and disease free.

Conclusions: Neoadjuvant therapy for cancer of the esophagus and cardia

results in good tumor response, Esophagectomy in this setting can be accomplished with acceptable morbidity and mortality. Results of an interim analysis of survival are encouraging and suggest that further investigation of this regimen is warranted.
CT 47th Annual Meeting of the Society-of-Surgical-Oncology
CY MAR 17-20, 1994
CL HOUSTON, TX
SP Soc Surg Oncol
TC 14
ZB 5
Z8 0
ZS 0
Z9 14
SN 1068-9265
UT WOS:A1995QH86800003
PM 7728562
ER

PT J
AU WILKE, H
STAHL, M
FINK, U
MEYER, HJ
SIEWERT, JR
TI PREOPERATIVE CHEMOTHERAPY FOR UNRESECTABLE GASTRIC-CANCER
SO WORLD JOURNAL OF SURGERY
VL 19
IS 2
BP 210
EP 215
PD MAR-APR 1995
PY 1995
AB Even with extended surgery, including systematic lymphadenectomy of the lymph node compartment II, only half of the patients with locally advanced gastric cancer (LAGC), which comprises stages IIIA, IIIB, and IV, undergo a macroscopic and microscopic tumor-free resection (i.e., R0 resection, according to UICC 1987/AICC 1988). An improvement of this situation is best accomplished by preoperative treatment modalities to increase the R0 resection rate and by preoperative and postoperative

treatment to reduce local recurrences and distant metastases. For LAGC, which includes approximately two-thirds of patients with locoregionally confined tumors, preoperative chemotherapy (CTx) represents a promising approach. Among a group of patients with surgically or clinically staged unresectable LAGC, approximately half underwent R0 resection after down-staging induced by active modern CTx. The long-term survival of these patients seems to be improved. Even in patients who had primarily unresectable tumors as defined by an explorative laparotomy, the long-term survival was about 20% after preoperative CTx and subsequent surgery. Based on these experiences, randomized trials investigating preoperative CTx versus surgery alone are clearly needed to define whether such an approach has an impact on R0 resection rates and survival of patients with LAGC. Preconditions for such trials are clinical staging procedures, including endoscopic ultrasonography (T category) and surgical laparoscopy plus lavage (excluding peritoneal carcinomatosis), and a standardized surgical procedure.

TC 22
ZB 9
Z8 1
ZS 0
Z9 23
SN 0364-2313
UT WOS:A1995QU71600008
PM 7754625
ER

PT J
AU AJANI, JA
MANSFIELD, PF
OTA, DM
TI POTENTIALLY RESECTABLE GASTRIC-CARCINOMA - CURRENT APPROACHES TO STAGING AND PREOPERATIVE THERAPY
SO WORLD JOURNAL OF SURGERY
VL 19
IS 2
BP 216
EP 220
PD MAR-APR 1995
PY 1995

AB The incidence of gastric carcinoma has declined worldwide during the past several decades, and yet this cancer remains the most common malignancy in several countries around the world, particularly Japan, Chile, and Costa Rica. Gastric carcinoma, although not as common in the United States as it was in the past, is still the eighth most frequent cause of cancer death. For patients with localized gastric carcinoma, surgery remains the most effective therapy, resulting in a consistent but low rate of cure. Unresectable gastric carcinoma is an incurable disease with the exception of a small fraction of patients who are salvaged with chemoradiotherapy. In Western countries curative resection rates have been dismal because of the lack of early diagnosis. Additionally, postoperative adjuvant strategies in the United States and Europe have been ineffective. Even patients with curative resection frequently develop intraperitoneal and systemic carcinoma in addition to locoregional relapses. Many investigators have therefore embarked on the therapeutic strategies of preoperative chemotherapy and postoperative intraperitoneal chemotherapy. The preoperative chemotherapy strategy has particular appeal because of its potential to reduce the size of the primary tumor, thereby allowing a higher rate of curative resection; early systemic therapy of micrometastates might prove biologically more effective. To date, several studies using preoperative chemotherapy have demonstrated its feasibility. The effectiveness of repeated courses of postoperative intraperitoneal chemotherapy remains unsettled mainly owing to the inadequacy of peritoneal drug distribution and the associated toxic effects. Additional investigations are necessary to improve preoperative staging with the use of endoscopic ultrasonography and laparoscopy (peritoneal staging). More effective preoperative chemotherapy combinations that might lead to 5% to 10% complete pathologic response in the presence of modest toxicity must be established prior to launching large-scale trials. The impact of these novel strategies on resection rates, failure sites, and patients' survival can be determined only by carefully designed, controlled clinical trials.
TC 42
ZB 15
Z8 1
ZS 0
Z9 42
SN 0364-2313

UT WOS:A1995QU71600009
PM 7754626
ER

PT J
AU AJANI, JA
ROTH, JA
PUTNAM, JB
WALSH, G
LYNCH, PM
ROUBEIN, LD
RYAN, MB
NATRAJAN, G
GOULD, P
TI FEASIBILITY OF 5 COURSES OF PREOPERATIVE CHEMOTHERAPY IN PATIENTS WITH RESECTABLE ADENOCARCINOMA OF THE ESOPHAGUS OR GASTROESOPHAGEAL JUNCTION
SO EUROPEAN JOURNAL OF CANCER
VL 31A
IS 5
BP 665
EP 670
DI 10.1016/0959-8049(94)00318-Y
PD 1995
PY 1995
AB The purpose of this study was to examine the feasibility of administering all chemotherapy pre-operatively to patients with resectable adenocarcinoma of the oesophagus or gastrooesophageal junction. 32 patients with potentially resectable adenocarcinoma of the oesophagus or gastrooesophageal junction were studied in a stepwise fashion in which combination chemotherapy with cisplatin, high-dose arabinoside and 5-fluorouracil was administered. In the first part, 15 patients were to receive three chemotherapy courses pre-operatively and two chemotherapy courses postoperatively. In the second part, the next 15 patients were to receive all five chemotherapy courses pre-operatively, provided there was an objective response after three courses. Endoscopic ultrasonography was also performed, when feasible, prior to chemotherapy and surgery, and in some patients sequentially between chemotherapy courses. All of the 14 assessable patients in the first group tolerated all three courses of pre-operative chemotherapy,

and 86% of patients in this group completed all protocol chemotherapy. In the second group, 9 of 18 (50%) assessable patients tolerated all five courses of preoperative chemotherapy, and 100% of patients in this group received all protocol chemotherapy. The median number of chemotherapy courses for the entire group (32 patients) was five (range one to five). Forty-one per cent (13/32) of patients had a major response to chemotherapy. Sixty-nine per cent (or 76% of 29 patients taken to surgery) had a curative resection. One patient had a pathological complete response. The median survival time of 32 patients was 17 months (range 2-36+ months). 14 patients (37%) remain alive at a median follow-up time of 26+ months. There was a correlation between endoscopic ultrasonographic tumour and nodal stage and pathological tumour and nodal stages in 16 patients. The tumour stage correlation was higher (75%) than the nodal stage correlation (62%). Our data suggest that it is feasible to administer five courses of cisplatin-based chemotherapy to patients with potentially resectable adenocarcinoma of the oesophagus or gastrooesophageal junction. More effective chemotherapy regimens that might result in higher pathological complete response rates and acceptable toxic effects are needed.
TC 16
ZB 7
Z8 1
ZS 0
Z9 17
SN 0959-8049
UT WOS:A1995RF15900007
PM 7640036
ER

PT J
AU LORE, G
CANZONIERI, V
VERONESI, A
DALBO, V
BARZAN, L
ZANCANARO, C
TROVO, M
TI EXTRAPULMONARY SMALL-CELL CARCINOMA - A SINGLE-INSTITUTION EXPERIENCE AND REVIEW OF THE LITERATURE

SO ANNALS OF ONCOLOGY
VL 5
IS 10
BP 909
EP 913
PD DEC 1994
PY 1994
AB Background. Small cell carcinoma (SCC) is a distinct pathologic entity that may also occur in extrapulmonary sites. In this report the retrospective results of multimodal therapy of primitive extrapulmonary (E) SCC, in a single institution series, are presented.
Methods. Twenty-four patients (pts) with ESCC were referred to the Centro di Riferimento Oncologico, Aviano, Italy, from 1986 to 1992. Clinico-therapeutic findings were evaluated in 20 pts. Their ages ranged from 20 to 87, with a median of 60.5 years. Primary tumor sites were urinary bladder (5 pts), prostate (4 pts), larynx (3 pts), kidney (2 pts), ovary, skin, oropharynx, trachea, uterine cervix, ethmoid, and stomach (1 pt each); lymph node metastases of unknown origin were observed in 3 pts. More than 50% of pts presented extensive disease.
Results. Histologically, 16 cases were pure ESCCs and 8 cases were combined, 4 of them with adenocarcinoma, 2 with transitional cell carcinoma, and 2 with squamous cell carcinoma. Immunohistochemical studies, performed in 7 cases, demonstrated the epithelial nature of these tumors. The cisplatin-VP16 (PE) regimen was used in 13 pts, and 9 of them (69%) obtained objective responses after chemotherapy (CT) alone, with 3 complete remissions (CR) and 6 partial remissions (PR). Median CR and PR duration was 13+ and 24 months, respectively. Radiotherapy was performed in 7/13 pts after induction CT and before consolidation CT. The objective response rate was 100%, with 6 CR and 1 PR. No severe toxic side effects and no toxic deaths were reported. A patient treated with surgery alone for a urinary bladder tumor showed continuous long-term survival, while 1 of 2 pts treated with radiotherapy alone obtained PR.
Conclusions. The PE regimen has an activity similar to the one observed in pulmonary SCC.
TC 54
ZB 33
Z8 0
ZS 0

Z9 54
SN 0923-7534
UT WOS:A1994QB78900007
PM 7696162
ER

PT J
AU WRIGHT, CD
MATHISEN, DJ
WAIN, JC
GRILLO, HC
HILGENBERG, AD
MONCURE, AC
CAREY, RW
CHOI, NC
DALY, M
LOGAN, DL
TI EVOLUTION OF TREATMENT STRATEGIES FOR ADENOCARCINOMA OF THE ESOPHAGUS AND GASTROESOPHAGEAL JUNCTION
SO ANNALS OF THORACIC SURGERY
VL 58
IS 6
BP 1574
EP 1579
PD DEC 1994
PY 1994
AB Between 1980 and 1988, 91 patients with adenocarcinoma of the esophagus were treated by surgical resection and selective postoperative therapy. Operative mortality was 2%. Pathologic stage was I in 4, II in 26, and III in 61. Actuarial 2- and 5-year survival was 24% and 8%. From 1987 to 1989, 16 patients with adenocarcinoma of the esophagus were treated with two cycles of 5-fluorouracil and cisplatin followed by surgical resection. There was 1 complete response (6%), 5 partial responses (31%), and 10 with no response (63%). Twelve patients had resection. Pathologic stage was I in 1, II in 4, and III in 8. There was one chemotherapy-related death and one surgical death. Actuarial 4-year survival is 42%. From 1990 to 1993, 22 patients with adenocarcinoma of the esophagus were treated with two cycles of etoposide, doxorubicin, and cisplatin followed by surgical resection. There was 1 complete

response (5%), 11 partial responses (50%), and 10 with no response (45%). Eighteen patients had resection. Pathologic stage was 0 in 1, II in 8, and III in 9. There were no treatment-related deaths. The actuarial 2-year survival is 58%. Conclusions are necessarily limited because the patients were not treated in a randomized fashion. These preliminary results with preoperative chemotherapy appear improved (p = 0.04 and p = 0.004, respectively) as compared with results from 1980 to 1988 without preoperative chemotherapy.
CT 30th Annual Meeting of the Society-of-Thoracic-Surgeons
CY JAN 31-FEB 02, 1994
CL NEW ORLEANS, LA
SP SOC THORAC SURGEONS
TC 26
ZB 10
Z8 0
ZS 0
Z9 26
SN 0003-4975
UT WOS:A1994PU98600004
PM 7979718
ER

PT J
AU ADELSTEIN, DJ
RICE, TW
BOYCE, GA
SIVAK, MV
VANKIRK, MA
KIRBY, TJ
VANSTOLK, RU
BUKOWSKI, RM
TI ADENOCARCINOMA OF THE ESOPHAGUS AND GASTROESOPHAGEAL JUNCTION - CLINICAL AND PATHOLOGICAL ASSESSMENT OF RESPONSE TO INDUCTION CHEMOTHERAPY
SO AMERICAN JOURNAL OF CLINICAL ONCOLOGY-CANCER CLINICAL TRIALS
VL 17
IS 1
BP 14
EP 18
DI 10.1097/00000421-199402000-00004

PD FEB 1994
PY 1994
AB A preoperative induction chemotherapy regimen consisting of two monthly courses of etoposide, doxorubicin, and cisplatin was given to 13 patients with nonmetastatic adenocarcinoma of the distal esophagus or gastroesophageal junction. Esophageal ultrasound examination was performed both before chemotherapy and again before surgery. Induction chemotherapy was poorly tolerated with 10 of the 13 patients experiencing at least one episode of severe neutropenia. Two of the 13 patients refused the second course of treatment. A symptomatic response to chemotherapy, defined as a reduction in the presenting symptom, was noted in 10 of the 13 patients (77%). Endoscopic improvement occurred in 9 of the 13 patients (69%). Esophageal ultrasound evidence of a reduction in either T or N stage was noted in only 2 of the 13 patients (15%), however, and neither of these responses was confirmed pathologically. Clinical evidence of disease progression was noted in 4 patients during chemotherapy. With a median follow-up of 31 months, the relapse-free and overall survivals are 25% and 31%, respectively. Despite significant toxicity, our chemotherapy regimen would be considered successful if assessed by symptomatic or esophagoscopic improvement. Esophageal ultrasound, careful pathologic staging, and our disappointing survival rates, however, suggest limited, if any, value for this approach.
TC 16
ZB 11
Z8 0
ZS 0
Z9 16
SN 0277-3732
UT WOS:A1994MX23700004
PM 8311001
ER

PT J
AU SAUTER, ER
COIA, LR
KELLER, SM
TI PREOPERATIVE HIGH-DOSE RADIATION AND CHEMOTHERAPY IN ADENOCARCINOMA OF THE ESOPHAGUS AND ESOPHAGOGASTRIC JUNCTION

SO ANNALS OF SURGICAL ONCOLOGY
VL 1
IS 1
BP 5
EP 10
DI 10.1007/BF02303535
PD JAN 1994
PY 1994
AB Background: Esophageal adenocarcinoma (EA) incidence is rising. Defining optimal management is essential because median survival after surgery alone is only approximately 12 months. High-dose radiation (>5000 cGy) and chemotherapy (HDRCT) preoperatively for patients with EA has not been fully investigated. We evaluated tumor response, resectability, and survival following HDRCT in patients with localized EA.
Methods: Thirty patients with American Joint Committee on Cancer (AJCC) clinical stage I or Il EA were prospectively treated with HDRCT. The treatment consisted of 60 Gy radiation at 2 Gy per fraction with concurrent infusional 5-fluorouracil (5-FU) and a bolus of mitomycin C followed by esophagogastrectomy. The range of follow-up was 7 to 69 months, with a median of 31 months.
Results: Twenty of 30 patients (67%) received full-course HDRCT. Severe esophagitis precluded full-dose radiation in 10 patients. Three patients developed neutropenia and fever requiring admission to a hospital. Two patients died preoperatively of treatment-related complications. Nine patients were not explored. Eighteen patients were resected with curative intent; the remaining three had metastatic disease at laparotomy. Seven of 18 resected patients (39%), or 7/30 (23%) of all patients treated, had a pathologic complete response. There was one operative death. Overall local control was seen in 25/30 patients (83%). Median overall survivals for resected and for all patients were 23 and 13 months, respectively.
Conclusions: Preoperative HDRCT in patients with EA results in encouraging local tumor response and local control. Overall survival, however, may not be improved, and the treatment-related mortality of 10% is higher than reported with surgery alone or with preoperative chemotherapy.
CT 46th Annual Cancer Symposium of the Society-of-Surgical-Oncology
CY MAR 18-21, 1993
CL LOS ANGELES, CA

SP SOC SURG ONCOL

TC 18

ZB 9

Z8 0

ZS 0

Z9 18

SN 1068-9265

UT WOS:A1994NM32800002

PM 7834428

ER

PT J

AU ROUGIER, P

MAHJOUBI, M

LASSER, P

DUCREUX, M

OLIVEIRA, J

YCHOU, M

PIGNON, JP

ELIAS, D

BELLEFQIH, S

BOGNEL, C

LUSINCHI, A

CVITKOVIC, E

DROZ, JP

TI NEOADJUVANT CHEMOTHERAPY IN LOCALLY ADVANCED GASTRIC-CARCINOMA - A PHASE-II TRIAL WITH COMBINED CONTINUOUS INTRAVENOUS 5-FLUOROURACIL AND BOLUS CISPLATINUM

SO EUROPEAN JOURNAL OF CANCER

VL 30A

IS 9

BP 1269

EP 1275

DI 10.1016/0959-8049(94)90171-6

PD 1994

PY 1994

AB Locally advanced gastric adenocarcinomas (LAGC) have a poor prognosis, particularly when tumours are bulky, located in the cardia or in the event of locoregional lymph node involvement. Patients bearing these

tumours were entered in a phase II trial of neoadjuvant chemotherapy, combining continuous intravenous 5-fluorouracil (5FU) (1000 mg/m(2) for 5 days) and cisplatinum (CDDP) (100 mg/m(2) on day 2) repeated every 4 weeks, for one to six cycles according to response and tolerance. 30 patients have been entered, 26 after clinical evaluation (CAT scan and upper gastrointestinal endoscopy) and 4 with unresectable tumours at prior laparotomy. Median age was 60 years, 15/30 patients had a tumour of the cardia, 15/30 had enlarged lymph nodes and 7/30 had linitis plastica (diffuse type). A mean number of three cycles was administered (range 1-6). 27 of the 30 patients were evaluable for response. One patient achieved a complee response (CR) and 14 a partial response (56%; 95% confidence interval 38-74%). No patient had tumour progression, and only 1/6 with linitis plastica responded. 28 patients underwent surgery, and 23 had a macroscopically complete resection (77% of the 30 entered patients); R0 resections were performed in 60% of the cases, mainly after an objective response (13/15 versus 4/12 in nonresponders). No pathological CR were seen. Grade 4 neutropenia was observed in eight cycles (5 patients), with five septic complications and one death due to toxicity. Four postoperative complications were observed: 2 cases of severe pneumonia and 2 subphrenic abscesses. One postoperative death, due to intravascular disseminated coagulation, was observed at day 30. Median survival was 16 months and the 1-, 2- and 3-year survival was 67, 42 and 38%, respectively. Patients with linitis plastica had a significantly shorter survival (P < 0.002). We conclude that neodjuvant chemotherapy is feasible in LAGC, although randomised trials are warranted to demonstrate its efficacy on survival and resection rates.
TC 49
ZB 28
Z8 0
ZS 0
Z9 49
SN 0959-8049
UT WOS:A1994PM85900014
PM 7999411
ER

PT J
AU AJANI, JA
MAYER, RJ

OTA, DM
STEELE, GD
EVANS, D
ROH, M
SUGARBAKER, DJ
DUMAS, P
GRAY, C
VENA, DA
STABLEIN, DM
TI PREOPERATIVE AND POSTOPERATIVE COMBINATION CHEMOTHERAPY FOR POTENTIALLY RESECTABLE GASTRIC-CARCINOMA
SO JOURNAL OF THE NATIONAL CANCER INSTITUTE
VL 85
IS 22
BP 1839
EP 1844
DI 10.1093/jnci/85.22.1839
PD NOV 17 1993
PY 1993
AB Background: Median survival of patients with local-regional gastric carcinoma is 10 months. Resection of the primary tumor and regional lymph nodes, with tumor-free margins (curative resection), has been the most effective treatment for local-regional gastric carcinoma. However, median survival of patients with curative resection of gastric carcinoma is 24 months, and the 5-year survival rate is about 20%. A single institution pilot study has established the feasibility of administering two courses of chemotherapy preoperatively and three courses postoperatively. In another study, a 15% pathologically documented complete response (pathologic complete response) has been reported in unresectable gastric carcinoma treated with etoposide, doxorubicin, and cisplatin. Purpose: Our purpose was to increase the curative resection rate in potentially resectable gastric carcinoma and to delay or eliminate micrometastases and thus improve survival. We also evaluated clinical and pathologic response to chemotherapy. Methods: Forty-eight previously untreated patients with potentially resectable gastric carcinoma received a chemotherapy regimen (EAP) consisting of etoposide (120 mg/m2 intravenously over a 2-hour period on days 4, 5, and 6), doxorubicin (20 mg/m2 as a 10-minute intravenous infusion on days 1 and 7), and cisplatin (40 mg/m2 as a 1-hour intravenous infusion on days 2

and 8). Patients received three courses of chemotherapy before resection, and responding patients received two courses postoperatively. Clinical and pathologic response rates, toxicity, patterns of treatment failure, and survival times were assessed. Results: A median of three courses (range, 1-5) of preoperative therapy was administered; six (12%) of the 48 patients had clinical complete response, and nine (19%) had partial response. Forty-one (85%) underwent surgery; 37 (90%) of these 41 (77% of the 48 patients) had a curative resection. There were no pathologic complete responses. Median survival for all patients is 15.5 months (range, 2-29+ months). Therapy was discontinued because of the toxic effects in one patient before surgery and in six patients after surgery. Doses were reduced in 37 patients (77%), mainly because of hematologic toxicity. Nineteen (40%) were hospitalized because of toxic effects, including 15 patients who developed fever with neutropenia. Grade 3 or 4 nausea and vomiting occurred in 15 patients and grade 3 or 4 diarrhea in seven patients. One death was directly related to chemotherapy. Conclusions: These data support that administration of preoperative and postoperative chemotherapy for local-regional gastric carcinoma is feasible in a multi-institutional setting. Our findings demonstrate that this EAP regimen is modestly active but is associated with substantial toxicity. Implications: Use of preoperative chemotherapy in resectable gastric carcinoma merits further evaluation, but more effective drug regimens will be required before a controlled trial is initiated.
TC 88
ZB 39
Z8 5
ZS 0
Z9 94
SN 0027-8874
UT WOS:A1993MG53000014
PM 8230264
ER

PT J
AU AJANI, JA
ROTH, JA
RYAN, MB
PUTNAM, JB

PAZDUR, R
LEVIN, B
GUTTERMAN, JU
MCMURTREY, M
TI INTENSIVE PREOPERATIVE CHEMOTHERAPY WITH COLONY-STIMULATING FACTOR FOR
RESECTABLE ADENOCARCINOMA OF THE ESOPHAGUS OR GASTROESOPHAGEAL JUNCTION
SO JOURNAL OF CLINICAL ONCOLOGY
VL 11
IS 1
BP 22
EP 28
PD JAN 1993
PY 1993
TC 42
ZB 27
Z8 0
ZS 0
Z9 42
SN 0732-183X
UT WOS:A1993KF53100005
PM 8418237
ER

PT J
AU LEICHMAN, L
SILBERMAN, H
LEICHMAN, CG
SPEARS, CP
RAY, M
MUGGIA, FM
KIYABU, M
RADIN, R
LAINE, L
STAIN, S
FUERST, M
GROSHEN, S
DONOVAN, A
TI PREOPERATIVE SYSTEMIC CHEMOTHERAPY FOLLOWED BY ADJUVANT POSTOPERATIVE
INTRAPERITONEAL THERAPY FOR GASTRIC-CANCER - A

UNIVERSITY-OF-SOUTHERN-CALIFORNIA PILOT PROGRAM
SO JOURNAL OF CLINICAL ONCOLOGY
VL 10
IS 12
BP 1933
EP 1942
PD DEC 1992
PY 1992
TC 88
ZB 42
Z8 5
ZS 0
Z9 94
SN 0732-183X
UT WOS:A1992KA72200016
PM 1453207
ER

PT J
AU Sugiyama, K
Sato, H
Ishizuka, K
Hoshi, M
Urushiyama, M
Wakui, A
TI [Preoperative role of angiotensin II induced hypertension chemotherapy (IHC) in advanced gastric carcinoma].
SO Gan to kagaku ryoho. Cancer & chemotherapy
VL 19
IS 2
BP 203
EP 9
PD 1992-Feb
PY 1992
AB To investigate the role of preoperative IHC in advanced gastric carcinoma, clinical, surgical and pathological stagings of 13 patients were analysed retrospectively. These patients were treated with a 3-drug combination of adriamycin, 5-fluorouracil and mitomycin C under angiotensin II induced hypertensive state. The response rate was 69.2%

and mean survival time was 850.2 days. "Down staging" in surgical stage was observed in 5 out of 13 cases (38.3%), and in pathological stage 7 cases (53.8%) "down staging" was achieved. Mean survival time of "pathological down-staging" cases was significantly longer than that of "non-down-staging" cases (1039.6 vs 322.1 days, p less than 0.01, generalized Wilcoxon test). IHC brings selective increase of drug delivery to tumor tissue, and will be useful as preoperative chemotherapy in advanced gastric carcinoma in terms of "down staging".
TC 2
ZB 1
Z8 0
ZS 0
Z9 2
SN 0385-0684
UT MEDLINE:1736832
PM 1736832
ER

PT J
AU WEESE, JL
NUSSBAUM, ML
TI GASTRIC-CANCER SURGICAL APPROACH
SO HEMATOLOGICAL ONCOLOGY
VL 10
IS 1
BP 31
EP 35
DI 10.1002/hon.2900100107
PD JAN-FEB 1992
PY 1992
AB Although the incidence of carcinoma of the stomach has steadily declined over the last 50 years, approximately 23 000 new cases will be diagnosed in the United States this year and 13 700 patients will die. Despite marked improvement in operative techniques, fewer than 20 per cent of those diagnosed with gastric cancer beyond the most superficial levels of invasion will survive for over five years. Gastric tumours spread by local, lymphatic, and aggressive intra-peritoneal routes as well as hematogenous dissemination. Over 87 per cent of recurrences have local or regional components.

Radiation therapy may decrease local and regional recurrences in those patients with transmural tumours. The neoadjuvant use of etoposide, adriamycin, and platinum may yield complete clinical and pathologic responses in patients found to have 'unresectable' tumours. Other chemotherapy regimens have been shown to have some effect on advanced disease and may have a role in the neoadjuvant setting.
Our current recommendations for the treatment of gastric cancer in a controlled trial setting would be neoadjuvant chemotherapy followed by R2 resection, postoperative +/- intraoperative radiation therapy with the possibility of postoperative chemotherapy. Hopefully, this aggressive multimodality approach will significantly improve the five year survival for this disease.
CT CONF ON CURRENT CANCER THERAPY
CY OCT, 1990
CL TAMPA, FL
SP UNIV S FLORIDA; H LEE MOFFITT CANC CTR & RES INST
TC 4
ZB 3
Z8 0
ZS 0
Z9 4
SN 0278-0232
UT WOS:A1992HP54900006
PM 1563702
ER

PT J
AU AJANI, JA
OTA, DM
JESSUP, JM
AMES, FC
MCBRIDE, C
BODDIE, A
LEVIN, B
JACKSON, DE
ROH, M
HOHN, D
TI RESECTABLE GASTRIC-CARCINOMA - AN EVALUATION OF PREOPERATIVE AND POSTOPERATIVE CHEMOTHERAPY

SO CANCER
VL 68
IS 7
BP 1501
EP 1506
DI 10.1002/1097-0142(19911001)68:7<1501::AID-CNCR2820680706>3.0.CO;2-L
PD OCT 1 1991
PY 1991
AB Patients with locoregional gastric carcinoma often die because of the low rates of curative resection and frequent appearance of distant metastases (mainly peritoneal and hepatic). To evaluate the feasibility of preoperative and postoperative chemotherapy, 25 consecutive previously untreated patients with potentially resectable locoregional gastric carcinoma received two preoperative and three postoperative courses of etoposide, 5-fluorouracil, and cisplatin (EFP). Ninety-eight courses (median, five courses; range, two to five courses) were administered. Six patients had major responses to EFP. Eighteen patients (72%) had curative resections, and three specimens (12%) contained only microscopic carcinoma. At a median follow-up of 25 months, the median survival of 25 patients was 15 months (range, 4 to 32+ months). Peritoneal carcinomatosis was the most common indication of failure. One patient died of postoperative complications, but there were no deaths due to chemotherapy. EFP-induced toxic reactions were moderate. Preoperative and postoperative chemotherapy for locoregional gastric carcinoma is feasible, and additional studies to develop regimens that could result in 5% to 10% complete pathologic responses may be warranted.
TC 129
ZB 61
Z8 5
ZS 0
Z9 133
SN 0008-543X
UT WOS:A1991GF96600005
PM 1893349
ER

PT J
AU AJANI, JA

ROTH, JA
RYAN, B
MCMURTREY, M
RICH, TA
JACKSON, DE
ABBRUZZESE, JL
LEVIN, B
DECARO, L
MOUNTAIN, C
TI EVALUATION OF PREOPERATIVE AND POSTOPERATIVE CHEMOTHERAPY FOR RESECTABLE ADENOCARCINOMA OF THE ESOPHAGUS OR GASTROESOPHAGEAL JUNCTION
SO JOURNAL OF CLINICAL ONCOLOGY
VL 8
IS 7
BP 1231
EP 1238
PD JUL 1990
PY 1990
TC 83
ZB 50
Z8 0
ZS 0
Z9 83
SN 0732-183X
UT WOS:A1990DM20000015
PM 2358838
ER

PT J
AU Chung, H C
Roh, J K
Park, Y J
Lee, S I
Min, J S
Lee, J T
Lee, K B
Kim, B S
TI Locally advanced unresectable gastric cancer successfully resected after neoadjuvant chemotherapy with FADE regimen.

SO Yonsei medical journal
VL 31
IS 1
BP 74
EP 9
PD 1990-Mar
PY 1990
AB The prognosis of unresectable advanced gastric cancer is extremely poor. We tried a neoadjuvant chemotherapy in locally advanced unresectable stomach cancer diagnosed by initial explo-laparotomy. After chemotherapy with the FADE regimen (5-fluorouracil + adriamycin + cisplatin + etoposide), the patient was diagnosed clinically as a complete response state on re-staging with radiological gastrointestinal study, fiber-gastroscopy and computerized tomography. During the second-look operation, the advanced cancer was completely resected and the pathological diagnosis was early gastric cancer (EGC) type IIc, stage II (T1N2Mo).
TC 3
ZB 1
Z8 0
ZS 0
Z9 3
SN 0513-5796
UT MEDLINE:2346043
PM 2346043
ER

PT J
AU WILKE, H
PREUSSER, P
FINK, U
ACHTERRATH, W
MEYER, HJ
STAHL, M
LENAZ, L
MEYER, J
SIEWERT, JR
GEERLINGS, H
KOHNEWOMPNER, CH

HARSTRICK, A
SCHMOLL, HJ
TI NEW DEVELOPMENTS IN THE TREATMENT OF GASTRIC-CARCINOMA
SO SEMINARS IN ONCOLOGY
VL 17
IS 1
BP 61
EP 70
SU 2
PD FEB 1990
PY 1990
CT SYMP AT THE 5TH EUROPEAN CONF ON CLINICAL ONCOLOGY : RECENT ADVANCES IN CANCER CHEMOTHERAPY
CY SEP 03, 1989
CL LONDON, ENGLAND
SP BRISTOL MYERS, DIV ONCOL; BRISTOL MYERS, INT GRP
TC 162
ZB 113
Z8 1
ZS 0
Z9 163
SN 0093-7754
UT WOS:A1990CR80100012
PM 2305269
ER

PT J
AU PREUSSER, P
WILKE, H
ACHTERRATH, W
FINK, U
LENAZ, L
HEINICKE, A
MEYER, J
MEYER, HJ
BUENTE, H
TI PHASE-II STUDY WITH THE COMBINATION ETOPOSIDE, DOXORUBICIN, AND CISPLATIN IN ADVANCED MEASURABLE GASTRIC-CANCER
SO JOURNAL OF CLINICAL ONCOLOGY

VL 7
IS 9
BP 1310
EP 1317
PD SEP 1989
PY 1989
TC 275
ZB 198
Z8 6
ZS 0
Z9 282
SN 0732-183X
UT WOS:A1989AM61500019
PM 2671287
ER

PT J
AU OBRECHT, JP
TI ADJUVANT CHEMOTHERAPY OF GASTROINTESTINAL TUMORS (GIT)
SO WIENER MEDIZINISCHE WOCHENSCHRIFT
VL 138
IS 13
BP 323
EP 330
PD JUL 15 1988
PY 1988
TC 0
ZB 0
Z8 0
ZS 0
Z9 0
SN 0043-5341
UT WOS:A1988P760400008
PM 3055691
ER

PT J
AU MACFARLANE, SD
   HILL, LD

JOLLY, PC
KOZAREK, RA
ANDERSON, RP
TI IMPROVED RESULTS OF SURGICAL-TREATMENT FOR ESOPHAGEAL AND GASTROESOPHAGEAL JUNCTION CARCINOMAS AFTER PREOPERATIVE COMBINED CHEMOTHERAPY AND RADIATION
SO JOURNAL OF THORACIC AND CARDIOVASCULAR SURGERY
VL 95
IS 3
BP 415
EP 422
PD MAR 1988
PY 1988
TC 59
ZB 37
Z8 0
ZS 0
Z9 59
SN 0022-5223
UT WOS:A1988M512600007
PM 3343850
ER

EF
